# The Invasin and Complement-Resistance Protein Rck of Salmonella is More Widely Distributed than Previously Expected

**DOI:** 10.1128/Spectrum.01457-21

**Published:** 2021-10-27

**Authors:** Michael Koczerka, Pierre-Emmanuel Douarre, Florent Kempf, Sébastien Holbert, Michel-Yves Mistou, Olivier Grépinet, Isabelle Virlogeux-Payant

**Affiliations:** a INRAE, Université de Tours, ISP, Nouzilly, France; b Laboratory for Food Safety, Salmonella and Listeria Unit, Anses, Maisons-Alfort, France; Broad Institute

**Keywords:** *Salmonella*, Enterobase, Rck, invasin, virulence, complement resistance

## Abstract

The *rck* open reading frame (ORF) on the *pefI-srgC* operon encodes an outer membrane protein responsible for invasion of nonphagocytic cell lines and resistance to complement-mediated killing. Until now, the *rck* ORF was only detected on the virulence plasmids of three serovars of Salmonella subsp. *enterica* (i.e., Bovismorbificans, Enteritidis, and Typhimurium). The increasing number of Salmonella genome sequences allowed us to use a combination of reference sequences and whole-genome multilocus sequence typing (wgMLST) data analysis to probe the presence of the operon and of *rck* in a wide array of isolates belonging to all Salmonella species and subspecies. We established the presence of partial or complete operons in 61 subsp. *enterica* serovars as well as in 4 other subspecies with various syntenies and frequencies. The *rck* ORF itself was retrieved in 36 subsp. *enterica* serovars and in two subspecies with either chromosomal or plasmid-borne localization. It displays high conservation of its sequence within the genus, and we demonstrated that most of the allelic variations identified did not alter the virulence properties of the protein. However, we demonstrated the importance of the residue at position 38 (at the level of the first extracellular loop of the protein) in the invasin function of Rck. Altogether, our results highlight that *rck* is not restricted to the three formerly identified serovars and could therefore have a more important role in virulence than previously expected. Moreover, this work raises questions about the mechanisms involved in *rck* acquisition and about virulence plasmid distribution and evolution.

**IMPORTANCE** The foodborne pathogen Salmonella is responsible for a wide variety of pathologies depending on the infected host, the infecting serovars, and its set of virulence factors. However, the implication of each of these virulence factors and their role in the specific host-pathogen interplay are not fully understood. The significance of our research is in determining the distribution of one of these factors, the virulence plasmid-encoded invasin and resistance to complement killing protein Rck. In addition to providing elements of reflection concerning the mechanisms of acquisition of specific virulence genes in certain serotypes, this work will help to understand the role of Rck in the pathogenesis of Salmonella.

## INTRODUCTION

Salmonella are Gram-negative bacteria that are responsible for one of the four key global causes of diarrheal diseases [WHO, https://www.who.int/news-room/fact-sheets/detail/salmonella-(non-typhoidal)]. The genus is divided into two species, S. bongori and S. enterica, the latter being itself divided in six subspecies (subsp. *enterica*, *salamae*, *arizonae*, *diarizonae*, *houtenae*, and *indica*) (1). The combination of somatic, flagellar, and capsular antigens allows for the identification of more than 2,600 serovars, which will, depending on the infected host, induce different pathologies, ranging from gastroenteritis to typhoid fever (2). Following contamination, which occurs mainly through the oral route, Salmonella will reach the intestine where it will be able to cross the intestinal barrier. As a facultative intracellular pathogen able to disseminate in its hosts, Salmonella developed along its evolutionary course different virulence mechanisms, including mechanisms involved in cell invasion and resistance to serum complement.

The Rck protein, for *r*esistance to *c*omplement *k*illing, is a 161-amino-acid-long outer membrane protein (OMP) in its mature form, consisting of 8 transmembrane domains and 4 loops exposed to the bacterial surface (3). This invasin belongs to a family of OMPs, including Ail (Yersinia enterocolitica and Yersinia pestis), PagC (Salmonella Typhimurium and Choleraesuis), and OmpX (Enterobacter cloacae), some of which share Rck virulence-associated properties (4). As for Ail and PagC (from S. Choleraesuis), Rck has initially been described for its ability to confer complement resistance to Salmonella (5–7). Rck acts on the three complement pathways: the classical, the alternative, and the lectin pathways. More precisely, the protein is able to block the insertion of the C5b-C9 complex into the bacterial membrane, thus preventing bacterial lysis following the formation of the membrane attack complex. It also has the ability to recruit several regulatory proteins. C4BP, a regulatory protein of the classical and lectin pathways that binds to C4B to alter the process of C3 convertase formation, is recruited through interaction with its α-chain complement control protein domains 6, 7, and 8 by Rck (8). Inhibition of the alternative pathway of the complement is also made possible by Rck through the recruitment of factor H via binding to domains 5, 6, 7, 19, and 20 of this factor. Factor H acts as a cofactor for factor I, which is involved in the reduction of C3 convertase assembly (9). The involvement of the first and third loops of Rck in resistance to complement killing was established through mutagenesis experiments. More precisely, a G118D substitution (third loop) was shown to be responsible for an increase in serum sensitivity of Escherichia coli overexpressing Rck. This phenomenon was heightened when an additional D43K (first loop) substitution was introduced, although this substitution alone did not significantly reduce bacterial survival (3).

The Rck protein has also been characterized for its ability to promote bacterial invasion of mammalian cells *in vitro*. Indeed, Rck has been identified as a factor enabling *in vitro* invasion of diverse cell lines from different cell types and organisms (10, 11). More precisely, a 46-amino-acid-long specific region, termed G114-V159 and comprising the third extracellular loop of the protein, has been shown to promote internalization on its own when expressed on the surface of latex beads. The host cell receptor responsible for Rck-dependent internalization is the epidermal growth factor receptor (EGFR) (12). The interaction between these two actors induces mobilization of actin microfilaments, notably through signaling via the Arp2/3 complex and the GTPases Rac and Cdc42, leading to the internalization of beads or bacteria (13). Moreover, a recent study demonstrated that by inducing internalization-independent DNA damages, the Rck-EGFR interaction activates the DNA damage response, thereby delaying the host cell cycle in the S phase. This alteration of the cell cycle would promote Rck-dependent internalization, as invasion preferentially takes place when the cells are in this phase of the cell cycle (14).

The gene encoding the Rck invasin belongs to the *pefI-srgC* operon, itself carried by few Salmonella virulence plasmids. These large plasmids, characterized by their IncF origin of replication and by the presence of the *spv* locus (*spvRABCD*), display a very restricted distribution and show variation in gene content from one serovar to another (15, 16). Consequently, the complete *pefI-srgC* operon has only been identified on the genomes of three Salmonella enterica subsp. *enterica* serovars, S. Typhimurium, S. Enteritidis, and *S.* Bovismorbificans. While the structure of this operon slightly differs among these three serovars, as well as the regulation mechanisms governing its expression, the sequence of the genes composing this locus remains well conserved (4, 17). In addition to this plasmid localization, a chromosomal location of *pefI* and *srgD* has been suggested by Collighan et al. in some serovars, including S. Enteritidis, *S*. Dublin, and S. Typhi (18). The exact function of the other open reading frames (ORFs) of the operon remains unclear. PefI has been identified as a regulator of the *pef* (*p*lasmid-*e*ncoded *f*imbriae) operon located just upstream of *pefI*. SrgA is a disulfide reductase involved in fimbriae biogenesis and in SpiA (T3SS-2 effector) folding. The protein encoded by *srgB* contains a putative lipoprotein signal sequence and displays a weak homology with the thermostable phytase superfamily. Finally, *srgD* and *srgC* encode putative transcription factors homologous to the LuxR and AraC families, respectively (19, 20). Currently, no functional link has been established between *rck* and any of the other ORFs in the *pefI-srgC* operon.

To this day, the *rck* ORF was identified only in three serovars (i.e., S. Enteritidis, S. Typhimurium, and *S*. Bovismorbificans), with a tendency toward specific associations between alleles and serovars. However, hybridization experiments using a DNA probe targeting the *rck* region and microarray experiments led to the hypothesis that the gene might also be carried on *S*. Blegdam, *S.* Pullorum, *S*. Moscow, S. Typhisuis, and *S*. Sendai genomes (18, 20, 21). Moreover, several studies reported the presence of other genes (e.g., *spvC* and *pefA*) traditionally associated with Salmonella virulence plasmids in serovars, such as *S*. Blegdam, *S.* Onarimon, *S.* Heidelberg, etc. (22–24). Together, these data suggested that the *pefI-srgC* operon, and therefore *rck*, might be more widely distributed than expected. In this study, our aim was to determine the distribution of the *rck* ORF within the Salmonella genus and to study the functionality of the variants of the protein. We took advantage of the large number of Salmonella genome assemblies available in the public database Enterobase to perform a large-scale screening of the *pefI-srgC* ORFs (25). One or more of these ORFs were found in 67,596 genomes associated with 61 serovars of S. enterica subsp. *enterica* but also in subsp. *salamae*, *arizonae*, *diarizonae*, and *houtenae*. The *rck* ORF was retrieved in 36 of them, thus extending greatly the list of serovars encoding this virulence factor. As a polymorphism at the *rck* locus was observed, *in vitro* studies were also carried out to assess the functionality of new Rck variants concerning their ability to confer complement resistance and invasion capacity to the pathogen.

## RESULTS

### Analysis of the *pefI-srgC* operon within Salmonella reference genomes.

In order to find new strains carrying the *pefI-srgC* operon, we first looked for the presence of the operon’s ORFs in the genomes of reference strains. A BLAST search of all these ORFs on 27 genomes retrieved from the ATCC genomic database allowed us to extend the list of *pefI-srgC*^+^ serovars and highlighted variations in the composition and organization of the *pefI-srgC* operon according to the serovar.

**FIG 1:**
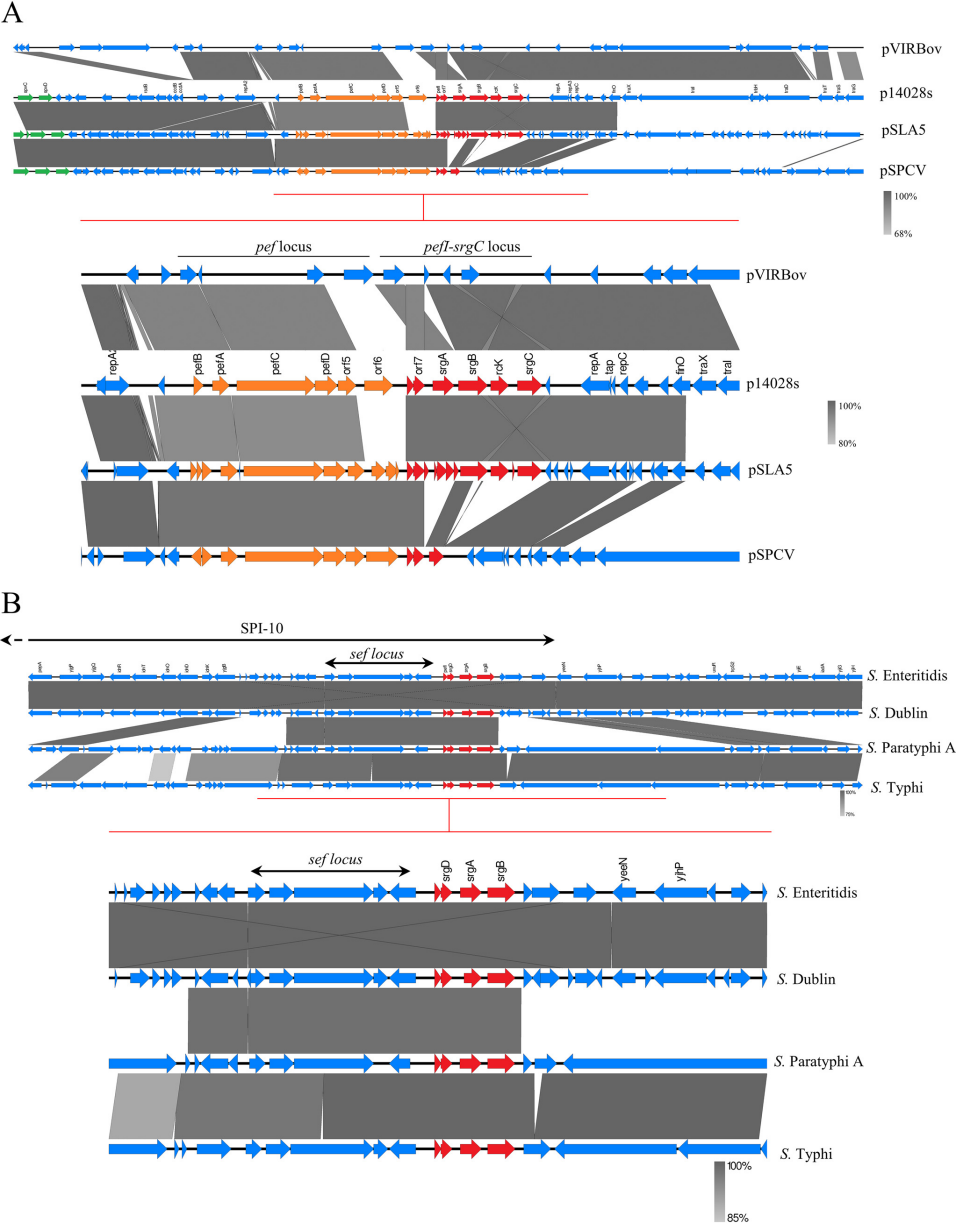
The *pefI-srgC* operon on reference genomes of Salmonella retrieved from ATCC. Alignment of the *pefI-srgC* operon and its surrounding regions on the currently described virulence plasmids of S. Typhimurium ATCC 14028 (p14028s), Bovismorbificans 3114 (pVIRBov), Enteritidis LA5 (pSLA5), and Paratyphi C (pSPCV) (A) or on the chromosomal SPI-10 locus of S. Enteritidis, Dublin, Paratyphi A, and Typhi (B). On the sequences displaying consistent annotations, red, orange, and green arrows represent the ORFs of the *pefI-srgC* operon, the *pef* operon, and the *spv* locus, respectively. Gray areas between the schematic sequences denote nucleotide identity with a gradient specified in the bottom-right corner of the figures.

As stated by Mambu et al. (4), two observations can be made when comparing the three complete operons previously described (retrieved on the virulence plasmids of S. Typhimurium, S. Enteritidis, and S. Bovismorbificans). The first observation is that the structure of the operon varies between these three serovars, as an inversion was observed between the *pefI-srgD* locus and the *srgA* ORF in *S*. Bovismorbificans compared to the sequences of S. Typhimurium and S. Enteritidis (Fig. 1A). The second observation is that the putative promoter sequences of the operon are also completely different between these serovars. While the inversion found in *S.* Bovismorbificans appears to have transposed the intergenic *srgD-srgA* region upstream of the operon, the promoter region found in S. Enteritidis differs completely from the one found in S. Typhimurium due to a completely different nucleotide sequence between *orf5* (in the *pef* operon) and *pefI* in these two serovars. This observation illustrates the results presented by Abed et al. highlighting the differences in the regulatory mechanisms of the operon in these 2 serovars (17). The conditions allowing the transcription of the *pefI-*srgC operon in S. Enteritidis and *S*. Bovismorbificans remain currently unknown.

The complete *pefI-srgC* operon was not found in any other tested genomes retrieved from the ATCC database. However, several genomes harbored one to four ORFs of the operon. The sequence found on the *S*. Paratyphi C virulence plasmid (pSPCV) carries *pefI*, *srgD* (*orf7*), and truncated *srgB* ORFs and is very similar to the corresponding virulence plasmid sequence of S. Enteritidis (pSLA5), especially the upstream region that displays a very similar synteny and high homology (≥97%), suggesting a similar regulatory scheme and a common origin (Fig. 1A). *S*. Dublin, *S.* Paratyphi A, and S. Typhi genomes also carry a part of the operon (i.e., 5′-*pefI*-*srgD*-*srgA*-*srgB-*3′), lacking the two last ORFs *rck* and *srgC*. These partial operons are located on the chromosome of their respective strains near the *sef* (S. Enteritidis fimbriae) operon in the Salmonella pathogenicity island (SPI) SPI-10 (Fig. 1B), thus confirming the chromosomal location of a partial *pefI-srgC* operon in some serovars as suggested by Collighan et al. using Southern hybridizations (18). These data suggest a common evolutionary process of *S.* Paratyphi A, S. Typhi, *S.* Dublin, and S. Enteritidis chromosomes in the *pefI-srgB* region. It is also interesting to note that a *srgB* allele displaying 81% identity with p14028s *srgB* was retrieved alone on 11 other serovar chromosomes, including *S*. Paratyphi B, *S*. Poona, *S.* Sendai, and *S*. Montevideo (data not shown). Even if we did not find any new complete *pefI-srgC* operon sequence in the reference genomes studied, these results highlighted the presence of partial *pefI-srgC* operons in several new serovars and prompted us to perform a wider analysis using Enterobase.

### Distribution of the *pefI-srgC* operon in Salmonella species and subspecies using Enterobase.

To extend this analysis, we retrieved the whole-genome multilocus sequence typing (wgMLST) sequence types (ST) generated by Enterobase from a total of 188,233 assemblies of Salmonella assigned by the Salmonella *in silico* typing resource (SISTR) prediction tool to the two Salmonella species and to all subspecies, including 465 S. enterica subsp. *enterica* different serovars. One should consider a bias in this analysis when interpreting the results: the presence of numerous false-negative hits in the data set. For example, *srgC* was not detected in any *S.* Bovismorbificans strain and usually was lacking in S. Enteritidis by wgMLST, while contig analysis clearly indicated its presence downstream of *rck* (data not shown). Consequently, the absence of an ORF in the wgMLST scheme corresponds either to a real absence of this ORF or to a false negative. As specified on the database documentation, this phenomenon occurs when the sequence of the allele of interest is not trustworthy, being either fragmented or duplicated (https://enterobase.readthedocs.io/en/latest/pipelines/backend-pipeline-nomenclature.html). In contrast, a positive result always signifies the presence of an ORF.

Among the extracted assemblies, 67,596 were associated with at least one ORF of the *pefI-srgC* operon, most of them belonging to S. Enteritidis and S. Typhimurium serovars, whose genomes are the most represented in the database. The profiles of the *pefI-srgC* operon in each serovar are described in Table S2 in the supplemental material. More than 1,500 profiles were identified using this method. Almost all genomes of S. Enteritidis carry the *pefI-srgC* operon (*n* = 35,969/35,996), and only half of the S. Typhimurium genomes harbor this locus (*n* = 17,562/37,533). However, 90% of the assemblies of the S. Typhimurium monophasic variant (antigenic formula: 4;[5];12:i:−) carry the operon, most of the time in its complete form (Table S2). As expected, we recovered numerous *pefI-srgC*^+^ assemblies from *S*. Bovismorbificans (*n* = 157/653), although the total number of genomes associated with this serovar was far less important than that of S. Enteritidis or S. Typhimurium. Most of the assemblies of *S.* Dublin (2,771/2,872), S. Typhi (8,021/8,037), and *S.* Paratyphi A (1,673/1,673) display the *pefI*-*srgD*-*srgA*-*srgB* profile described above on the ATCC reference strains, supporting a wider distribution of a truncated operon on the chromosomes of these serovars (Table S2). Interestingly, the diversity of the allelic profile of these serovars was strongly biased toward a dominant profile displaying very high frequencies (i.e., according to the allelic profiling categories used in Enterobase; *S.* Dublin: profile 5-4-6-64-0-0, *P* = 0.977; *S.* Paratyphi A: profile 20-2-35-55-0-0, *P* = 0.992; S. Typhi: profile 2-2-4-3-0-0, *P* = 0.911). Altogether, these results confirm the data obtained with reference genomes.

**FIG 2:**
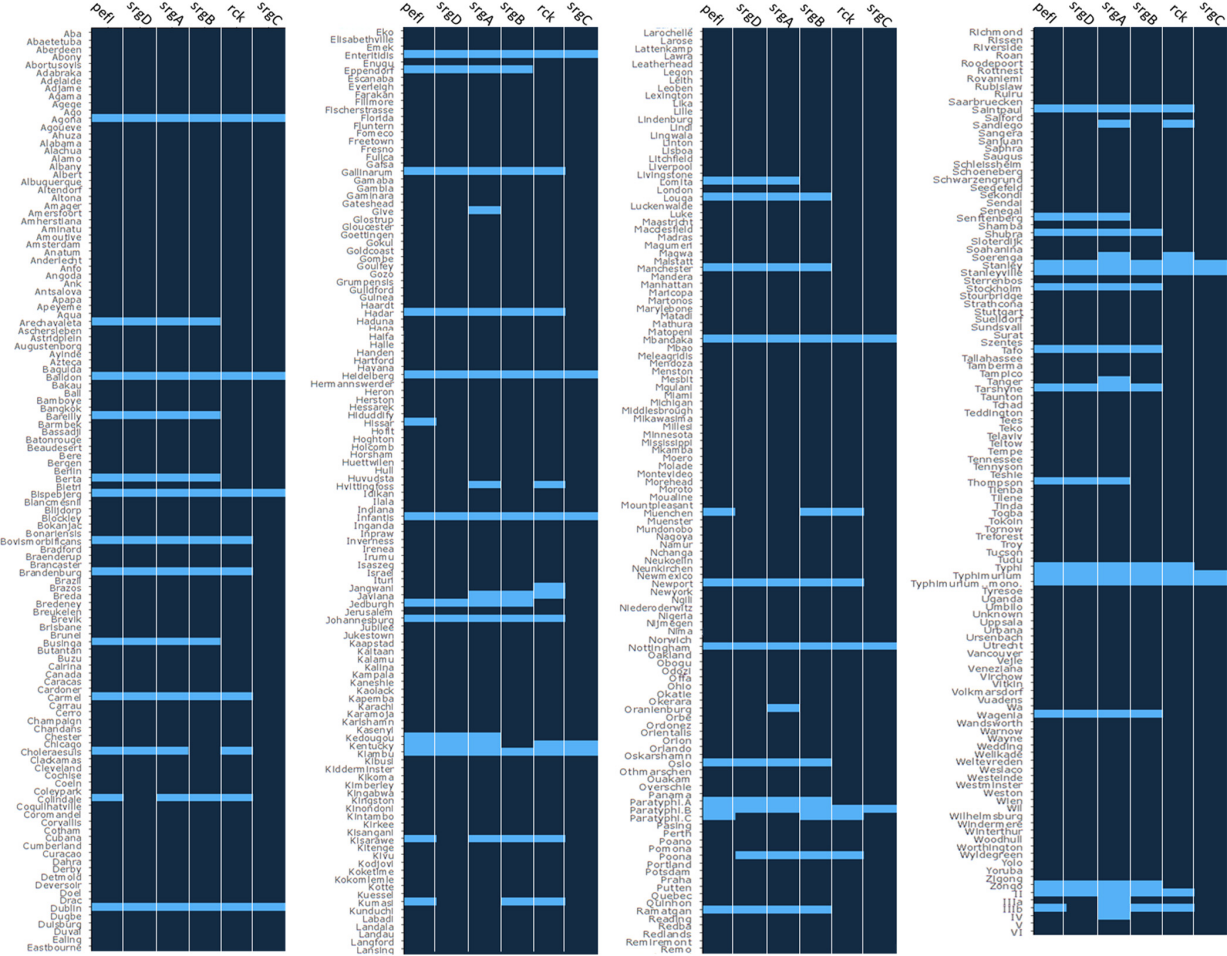
Distribution of the 6 ORFs from the *pefI-srgC* operon in the Enterobase-based data set. wgMLST-based heat map of presence (light blue) and absence (dark blue) of the *pefI-srgC* operon’s ORFs on the different serovars/subspecies/species retrieved in the Enterobase-based data set.

The *pefI-srgC* operon was also identified in new serovars, some of them displaying a very high frequency of the operon. Indeed, all the wgMLST data retrieved from the Berta (680/680) and Manchester (13/13) serovars, as well as most of the Gallinarum serovar (295/297), presented a profile containing at least the first four ORFs of the operon. In a similar manner to what has been previously observed on S. Typhi, *S.* Paratyphi A, and *S.* Dublin chromosomes, these ORFs are located near the *sef* operon in SPI-10 and share high identity (≥90%) with these reference genomes for this region (Fig. S1). Similar to what was described above, *S.* Berta and *S.* Manchester display low allelic profile diversity in this region, with their dominant profile displaying high frequencies (profile 5-4-6-64-0-0, *P* = 0.904, and profile 2-2-42-3-0-0, *P* = 0.846, respectively). By contrast, the allelic profiles appear more diverse on *S.* Gallinarum assemblies (*P*_max_ = 0.313). Highly prevalent serovars *S.* Newport, *S.* Kentucky, and *S.* Infantis also carry some ORFs of the operon but at a very low frequency (9/9,364; 1/5,855; and 2/7,405, respectively) (Fig. 2; Table S2). It is important to note that the complete operon was retrieved in 14 serovars, including *S.* Agona, *S*. Baildon, and *S.* Paratyphi B (Table S2). Finally, ORFs of the operon have also been found in genomes from 51 other serovars of S. enterica subsp. *enterica* but also within genomes of non-*enterica* subspecies. Indeed, four assemblies of our data set associated with S. enterica subsp. *salamae* were found to harbor the first five ORFs of the operon, with very different synteny from one strain to another. While two assemblies associated with S. enterica subsp. *diarizonae* appear to carry multiple ORFs of the operon (profiles 8-0-16-23-7-0 and 9-0-69-0-0-0), *srgA* was retrieved, alone, in numerous S. enterica subsp. *arizonae* (116/419), *diarizonae* (37/769), and *houtenae* (2/383) assemblies (Table S2).

PCR and Southern blots have previously allowed for the identification of genes associated with Salmonella virulence plasmids, such as *pefA*, *spvB*, or *spvC*, in serovars for which such plasmids have not yet been described (e.g., Infantis, Heidelberg, Blegdam, Onarimon, Moscow, Lexington, Panama, Anatum, and Weltevreden) (22, 26–31). Regarding our interesting preliminary results, we intended to investigate further on these serovars except for serovars Blegdam, Onarimon, and Moscow, which were missing in our data set. Only three assemblies were positive for serovars Heidelberg (1/4,528) and Infantis (2/7,405). No assemblies from the other serovars were positive for the *pefI-srgC* operon.

### *rck* ORF distribution within the assembled genomes database Enterobase.

**FIG 3:**
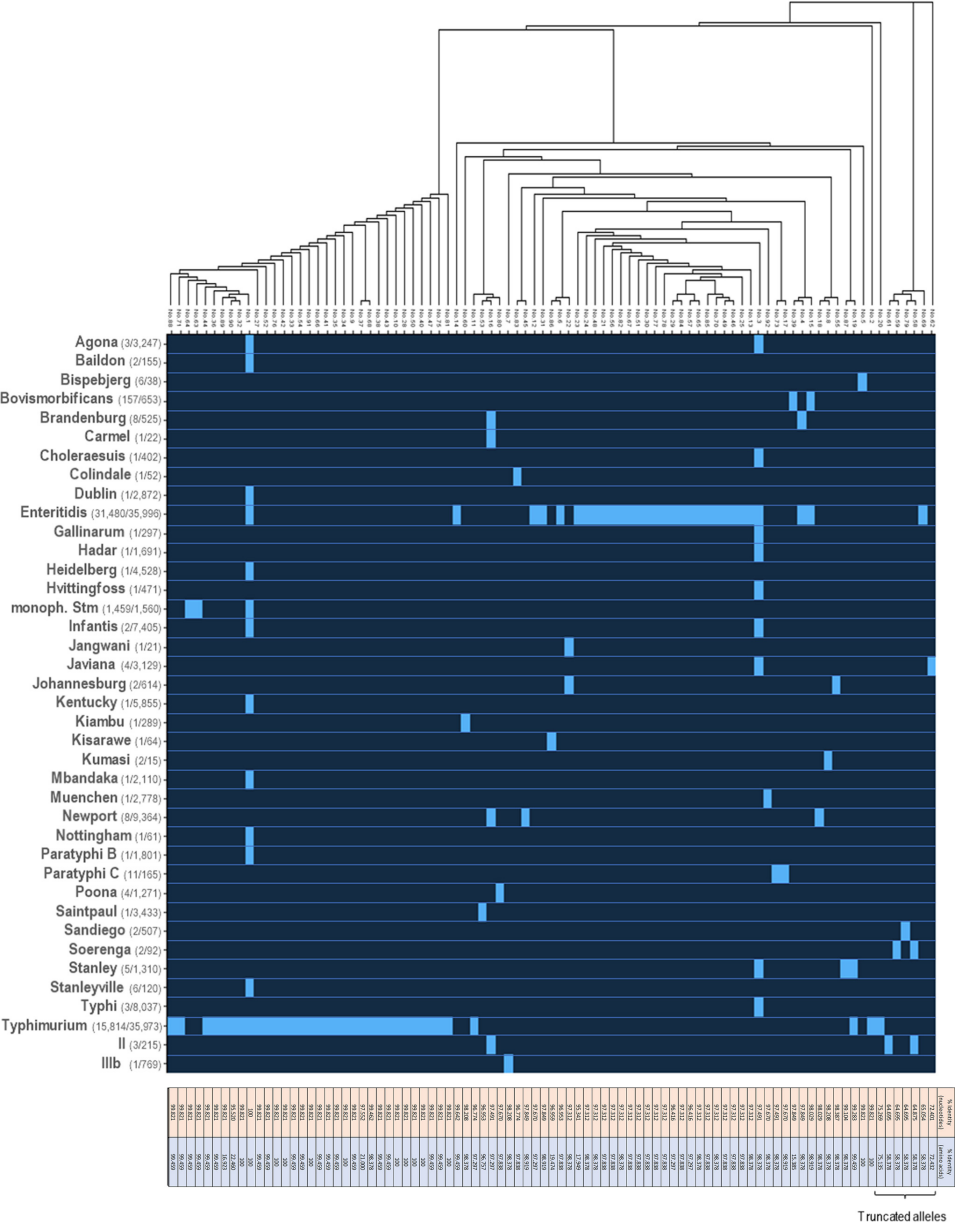
Distribution of the alleles of the *rck* ORF. Top, neighbor-joining tree based on the nucleotide sequences of the 89 *rck* alleles retrieved from the Enterobase data set. Middle, presence (light blue)/absence (dark blue) heat map of the *rck* alleles within the set of each serovar/subspecies assemblies. Bottom, nucleotide (orange) and amino acid (blue) sequence levels of identity with either the nucleotide sequences of allele number 1 of *rck* or its translated counterpart. For each serovar or subspecies listed on the left part, the number of assemblies bearing *rck* out of the total number of assemblies present in Enterobase is indicated in brackets.

The Rck invasin, encoded by *rck*, is the most characterized protein of the *pefI-srgC* operon. It acts as an invasin through interaction with EGFR to mediate a zipper-like internalization within a variety of mammalian cell lines *in vitro*. It also acts as a resistance to complement factor through interaction with multiple inhibitors of this innate defense system. We thus decided to deepen our analysis on the distribution of this virulence protein using the wgMLST data retrieved from Enterobase. We were able to confirm the presence of the ORF in S. Typhimurium (monophasic variant: 1,459/1,560; diphasic variant: 15,814/35,973), S. Enteritidis (31,480/35,996), and *S.* Bovismorbificans (157/653) assemblies. Additionally, 33 new serovars and two subspecies were associated with the gene encoding this invasin (Fig. 3). Among these serovars, some are part (S. Enteritidis and S. Typhimurium) of the most frequently isolated serovars responsible for human salmonellosis in the European Union from 2017 to 2019, such as *S*. Newport (*n* = 8/9,364), *S*. Brandenburg (*n* = 8/525), or *S*. Stanley (*n* = 5/1,310) (32). It is also noteworthy to highlight the detection of the *rck* ORF on 11 assemblies (11/165) associated with *S*. Paratyphi C, for which only a partial operon containing the *pefI*, *srgD*, and *srgB* ORFs was described above.

**FIG 4:**
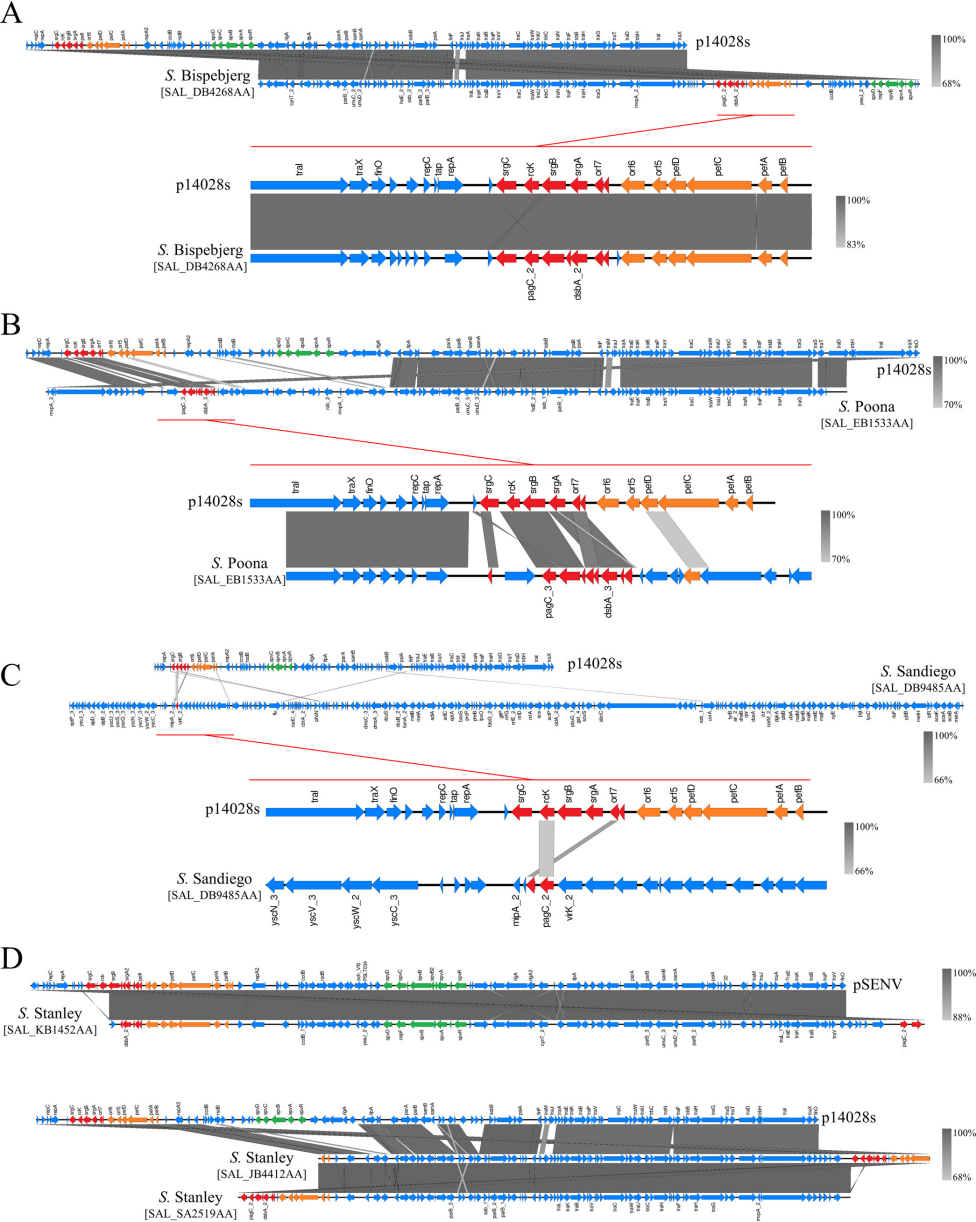
Multiplicity of *rck* molecular supports. Comparison of the genetic environment surrounding the *rck* ORF retrieved from *rck*^+^ representative assemblies, with an example of *rck* ORFs carried by a p14028s-like plasmid (A), a putative *spv*^−^ plasmid (B), directly on the chromosome (C), and on multiple molecular supports within the same serovar (D). On the sequences displaying consistent annotations, red, orange, and green arrows represent the ORFs of the *pefI-srgC* operon, the *pef* operon, and the *spv* locus, respectively. Gray areas between the schematic sequences denote nucleotide identity with a gradient specified in the bottom-right corner of the figures.

To estimate whether *rck* in these strains is plasmid borne or chromosomally encoded, we compared the genetic environment surrounding the *rck* ORF in the contigs where it was detected with S. Typhimurium- or S. Enteritidis-corresponding regions on the large virulence plasmids. Evidence indicated that some strains acquired the virulence plasmid of S. Typhimurium (e.g., *S*. Agona, *S*. Baildon, *S*. Stanleyville, etc.) (Fig. 4A; Fig. S2A) or S. Enteritidis (e.g., *S*. Stanley) (Fig. S2B) or a close derivative of these plasmids (e.g., *S.* Bispebjerg) (data not shown) through horizontal transfer, as contigs displaying from 90% to nearly 100% identity with these references were retrieved in some of these strain assemblies.

Interestingly, the contigs harboring *rck* on some *S*. Paratyphi C assemblies seem to indicate the existence of a novel, undescribed virulence plasmid. In view of the gene content, it appears that this plasmid represents a plasmid distinct from S. Typhimurium and S. Enteritidis virulence plasmids. Indeed, this plasmid, while displaying ≥90% identity on the shared regions with both of these references, lacks the whole *samA* to *traS* region of S. Enteritidis virulence plasmid (pSENV) but kept the *traS* to *finO* region of this plasmid. The loss of *srgA* should also be highlighted in the operon (Fig. S2C).

However, the contigs retrieved in some assemblies of strains associated with serovars *S.* Poona (Fig. 4B), *S.* Johannesburg, *S.* Newport, and *S.* Stanley (Fig. S2D to F) suggested that the operon might be carried on a nonvirulence plasmid, as it colocalized with plasmid-related genes (e.g., *tra*, *par*, etc.) but not with the traditional markers of Salmonella virulence plasmids (i.e., *spv* locus) (16). The absence of *spv* genes was confirmed with wgMLST data, which indicate that *spvRABCD* ORFs were missing from the other contigs of these assemblies.

Finally, colocalization of *rck* with chromosomal genes as observed on *rck*^+^ contigs issued from *S*. Sandiego (e.g., *phoN*, *uvrA*, and *dnaB*) and *S*. Brandenburg (e.g., *bigA*, *damX*, *ybbN*, and *phnT*) assemblies led us to suggest a chromosomal integration (Fig. 4C; Fig. S2G). It is also interesting to note that the genomic localization may vary among strains of the same serovar. This is notably the case for *S*. Stanley, which displays two kinds of supports, some *rck* ORFs associated with pSENV-like large virulence plasmid while others seem to be carried on nonvirulence plasmids (Fig. 4D).

As expected, the consequences of the diversity of *rck* genomic localizations were found at the level of the promoter region of the *pefI-srgC* operon. The strains that probably acquired the ORF by horizontal transfer of an entire virulence plasmid retained the sequence upstream of the operon found in S. Typhimurium and S. Enteritidis and presumably a similar pattern of regulation of its expression. However, this same region on other assemblies presents dissimilarities with those described to date, suggesting different regulatory mechanisms in these cases and different evolutionary routes (Fig. S2A to G).

### Polymorphism of the *rck* ORF.

*rck^+^* sequences were found to harbor a noteworthy diversity of allelic variants. Indeed, 89 different alleles (including those previously described on p14028s [number 1], pSENV [number 3], and pVIRBov [number 15]) harbored by a total of 49,001 strains were retrieved from our data set. Comparison of these sequences revealed that the *rck* ORF is very well conserved, with 81/88 displaying more than 95% of nucleotide identity with allele number 1. However, the phylogenetic tree based on this alignment revealed the existence of two clades, one related to allele number 1 and the other to allele number 3 (Fig. 3). In addition to being the dominant allele retrieved from S. Typhimurium assemblies (monophasic variant: 1,455/1,459; diphasic variant: 15,603/15,814), allele number 1 was also retrieved from 60 S. Enteritidis assemblies as well as from 16 assemblies associated with a total of 10 other serovars, including *S.* Agona, *S.* Baildon, and *S.* Stanleyville. Similarly, allele number 3, the dominant allele of S. Enteritidis (30,748/35,996), was retrieved in 12 assemblies associated with 9 other serovars (e.g., S. Typhi, *S.* Agona, or *S.* Infantis), while allele number 15 (retrieved on pVIRBov) appeared to be specific of *S.* Bovismorbificans. In total, 63 undescribed alleles were retrieved from S. Typhimurium and S. Enteritidis assemblies, while assemblies from other serovars harbored a total of 24 undescribed alleles. These latter alleles showed great identity (ranging from 99.4% to 99.8%) with allele number 1, except seven alleles that displayed less than 76% nucleotide identity with this reference (Fig. 3). These important differences are mainly due to deletion of various portions of the ORF, leading to aberrant Rck proteins. Still, they remain very rare, as they are only found in 13 assemblies (among 49,001 *rck*^+^ assemblies) associated with serovar S. Typhimurium (allele number 20: *n* = 2), *S*. Soerenga (allele number 58: *n* = 1; allele number 59: *n* = 1) *S*. Javiana (allele number 62: *n* = 3), S. Enteritidis (allele number 69: *n* = 2), *S*. Sandiego (allele number 79: *n* = 2), and S. enterica subsp. *salamae* (allele number 58: *n* = 1; allele number 61: *n* = 1).

Altogether, these alleles encode proteins that are greatly similar in their sequence to the Rck protein of S. Typhimurium 14028 strain. They exhibit between 96% and 100% amino acid identity, except for alleles 20, 23, 37, 39, 58, 59, 61, 62, 69, 79, 86, 89, and 90 for which the introduction of a frameshift led to the production of truncated proteins.

### Phenotypic characterization of Rck variants.

**FIG 5:**
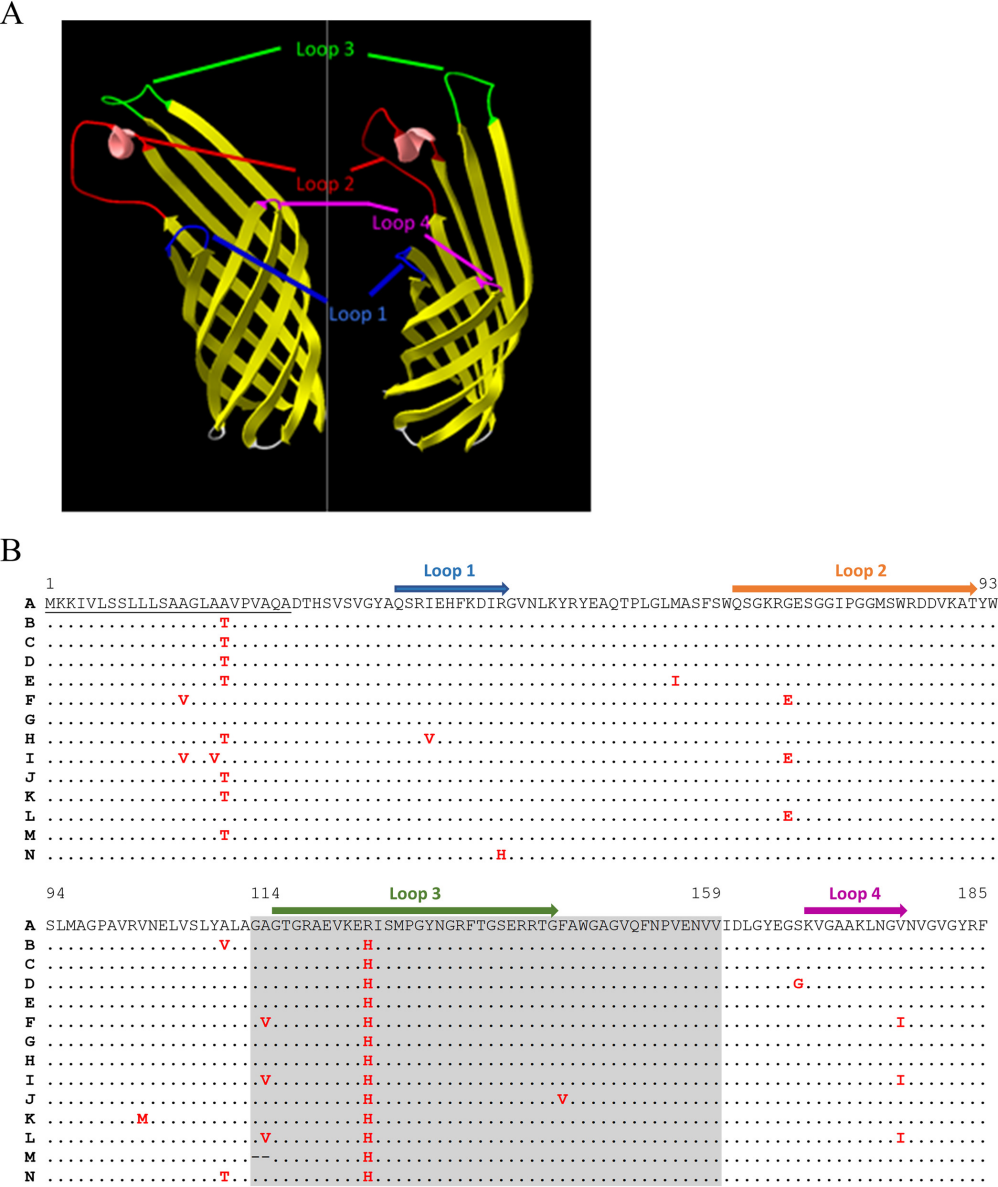
Predicted structure of Rck invasin and polymorphism of the Rck variants. (A) Homology-based structural model of the Rck_A_ invasin from S. Typhimurium 14028 predicted with SWISS-MODEL. (B) Alignment of the translated Rck variants sequences retrieved from Enterobase. The letters A to N on the left of the figure correspond to the 14 variants described in Table 1. The four loops are indicated by colored arrows above the sequence. The gray area represents the 114 to 159 peptide necessary and sufficient to promote invasion of mammalian cells. Underlined amino acids represent the signal peptide. Amino acids indicated in red denote polymorphic residues compared to the sequence of the Rck_A_ variant. Dashes in the Rck_M_ sequence correspond to the deletion of two amino acids.

Several reports demonstrated that even minor variation in the amino acid sequence might generate important variation in protein function (33, 34). We therefore sought to evaluate the impact of the protein polymorphism observed in Rck sequences on its ability to both promote bacterial invasion of host cells and to protect these bacteria from complement attack. To better understand the potential impact of this polymorphism on the three-dimensional (3D) structure of Rck, we generated an *in silico* model of the protein encoded by allele number 1. This model shows great reliance with the prediction made by Guiney’s laboratory and confirms the overall β-barrel transmembrane structure of the protein, exposing four extracellular loops similar to its Ail homolog in Yersinia pestis (Fig. 5A) (3).

The impact of the polymorphism described above was evaluated with a noninvasive E. coli strain sensitive to complement killing to exclude all the other Salmonella factors involved in these phenotypes that could mask the role of Rck. In this heterologous system, 12 uncharacterized variants of the protein, termed Rck_C to N_, specifically recovered from Enterobase *rck*^+^ assemblies were overexpressed and compared to Rck_A_ (of S. Typhimurium 14028, encoded by allele number 1) and Rck_B_ (of S. Enteritidis, encoded by allele number 3), the two Rck proteins phenotypically characterized in the literature (10, 14) (Table 1). The variants were selected based on their distribution within the new *rck*^+^ serovars and their identity level with the most characterized variant of Rck (i.e., Rck_A_) (Fig. 5B). Variants presenting less than 96% of identity with Rck_A_ were not studied as we considered that the accumulation of mutations in these variants will have a great probability to change the overall structure of the protein.

**TABLE 1:** Description of the 14 Rck protein variants

Rck protein variants	Haplotypes	Serovars or subspecies	Amino acid polymorphisms^*a*^^,^^*b*^
A	1; 5; 40	Agona, Baildon, Dublin, Heidelberg, Infantis, Kentucky, Mbandaka, Nottingham, Paratyphi B, Stanleyville, Typhimurium, Bispebjerg	–
B	3; 24	Agona, Choleraesuis, Enteritidis, Gallinarum, Hadar, Hvittingfoss, Infantis, Javiana, Stanley, Typhi, Muenchen	A18T; A111V; R125H
C	15; 17; 45	Bovismorbificans, Paratyphi C, Newport	A18T; R125H
D	7	*S. diarizonae*	A18T; R125H; S167G
E	8; 18; 55	Kumasi, Newport, Johannesburg	A18T; M62I; R125H
F	16	Brandenburg, Carmel, Newport, *S. salamae*	A14V; G73E; A115V; R125H; V177I
G	19	Stanley	R125H
H	22	Jangwani, Johannesburg	A18T; I38V; R125H
I	53	Saintpaul	A14V; A17V; G73E; A115V; R125H; V177I
J	60	Kiambu	A18T; R125H; F144V
K	73	Paratyphi C	A18T; V103M; R125H
L	80	Poona	G73E; A115V; R125H; V177I
M	83	Colindale	A18T; Δ114 to 115; R125H
N	87	Kirasawe	R45H; A111T; R125H

a Amino acid polymorphisms are described taking as reference the sequence of the Rck variant A.

b –, no data.

**FIG 6:**
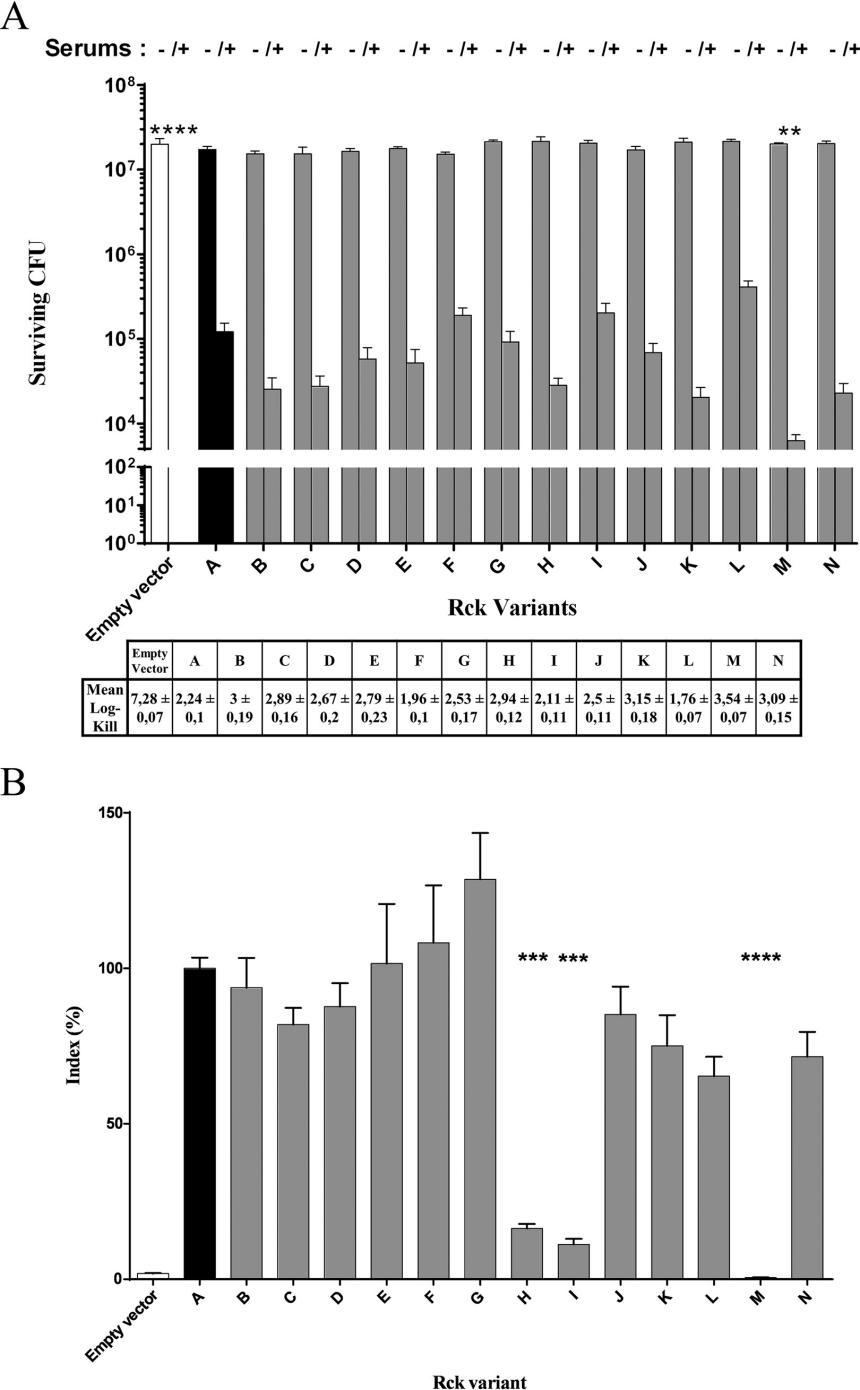
Phenotypic characterization of the Rck variants. (A) Survival rate of E. coli overexpressing the Rck variants following incubation with decomplemented (left bar) or normal (right bar) swine sera. (B) Invasion rate of E. coli overexpressing the Rck variants in JEG-3 cells normalized to the invasion rate of E. coli overexpressing Rck_A_ (of S. Typhimurium ATCC 14028). For both tests, at least two independent experiments were performed in triplicate for each variant. Results were compared using a Kruskal-Wallis test (**, *P *≤ 0.01; ***, *P *≤ 0.001; ****, *P *≤ 0.0001).

Each variant has first been tested for its ability to confer complement resistance by comparing bacterial survival following incubation with normal and decomplemented serum. The MC1061 E. coli strain carried either an empty pSUP202 plasmid for the negative control (absence of serum resistance), the pSUP202 plasmid expressing the Rck protein of S. Typhimurium (Rck_A_) as the positive control of serum resistance, or the pSUP202 plasmid expressing the 13 other variants. The first conclusion that emerged from these tests is that all the evaluated variants confer a certain resistance to the action of complement factors. As expected, E. coli MC1061 harboring the mock vector did not survive this innate immunity defense system, showing more than 7 logs of killing. In contrast, all the strains overexpressing one Rck variant exhibited less than 4 logs of killing compared to results obtained with decomplemented serum (Fig. 6A), thus confirming the conservation of the resistance to complement function of these variants. Nevertheless, a deletion of the amino acids at positions 114 and 115 (Rck_M_) induced a greater sensitivity to complement, because a significant decrease in the survival rate of bacteria overexpressing this variant was observed (3.54 ± 0.07 log kill; *P* = 6.4E−3) compared to strains overexpressing Rck_A_ (2.23 ± 0.23 log kill). These results are in accordance with previous studies showing the importance of the third extracellular loop on the virulence-associated phenotypes granted by Rck and highlight the role of these two amino acids in the function of Rck in serum resistance (3, 10).

Then, the variants were tested for their ability to mediate E. coli invasion into JEG-3 cells. These cells were chosen because this is one of the cell lines for which we observed the greatest difference between the control strains, thus allowing for easier detection of slight effects of amino acid substitutions (10). The first observation that emanated from these experiments was that the polymorphism observed between the three previously sequenced alleles of S. Typhimurium, S. Enteritidis, and *S.* Bovismorbificans generates proteins (Rck_A_, Rck_B_, and Rck_C_, respectively) that share the same ability to promote invasion of JEG-3 cells (Fig. 6B). The second observation was that, unlike what was observed during resistance to complement assays, not all the variants granted the ability to invade this cell line. Indeed, overexpression of Rck_H_, Rck_I_, and Rck_M_ led to a significant decrease of the ability of E. coli to invade JEG-3 cells compared to Rck_A_. The quantity of internalized bacteria counted following treatment with gentamicin indicated a significant decrease of 83.7% (*P* = 4.3E−4), 88.8% (*P* = 1.5E−4), and 99.5% (*P* = 1.6E−11) in the entry rate induced respectively by these variants compared to Rck_A_ (Fig. 6B).

Rck_I_ only differs from its parental variant (Rck_F_), which does not induce altered invasion, by one substitution in the predicted signal peptide in position 17 (A17V substitution) (Fig. 5B). Although the explanation for this decrease in cellular invasion (88.8%) remains unclear, one can hypothesize that the accumulation of two mutations in the signal peptide would lead to a significant, but not a total, decrease in the membrane addressing of this variant. E. coli overexpressing Rck_H_ displayed a significant decrease of invasion rate of 83.7% compared to Rck_A_. This phenotype might appear surprising when comparing the sequence of this variant with the sequence of Rck_C_ given the localization of the substitution observed in the first loop (I38V). Indeed, it was demonstrated that the third loop is sufficient to promote invasion (10). One hypothesis that could explain this result is a cooperation between the loops (3).

Finally, the phenotype observed when comparing the invasion rate of Rck_M_ with our reference protein does not appear surprising given the substantial deletion present in this variant. Indeed, as the amino acids in position 114 and 115 constitute the anchor point of the third extracellular loop in the bacterial membrane, we suggest that the deletion present in this variant greatly alters the structure of the loop, thus hindering a proper interaction with EGFR.

## DISCUSSION

The *rck* ORF, which belongs to the *pefI-srgC* operon itself carried on the large virulence plasmid of Salmonella, encodes an OMP involved in cell invasion and complement resistance. Little was known about its distribution, and while it was assumed that it was restricted to only a few serovars, several reports have established the presence of virulence plasmid-associated genes in less characterized serovars (18, 26, 30). Moreover, other ORFs of the operon (*pefI*, *srgD*, *srgA*, and *srgB*) have been described on the serovar-specific pathogenicity island SPI-10 (35, 36). The constant progress in genomics and the improvement of sequencing techniques has allowed for an increase in the number of sequenced and characterized genomes. We took advantage of the large number of Salmonella genome sequences available to investigate further the distribution of the operon and more specifically of the *rck* ORF using both complete genomes on ATCC and Enterobase databases.

The complete or partial *pefI-srgC* operon was found in 61 S. enterica subsp. *enterica* serovar assemblies, while the *rck* ORF itself was retrieved in 36 of them (i.e., 33 more than previously known). Moreover, the complete or partial operon was identified for the first time in four Salmonella enterica subspecies (*salamae*, *arizonae*, *diarizonae*, and *houtenae*) and *rck* itself in two of them (*salamae* and *diarizonae*). Altogether, these results highlight a greater distribution of this operon and of the *rck* virulence gene, thus suggesting a more important role of this virulence factor than previously expected. This is even more true when considering the fact that a significant number of false-negative hits occurs when processing with the Enterobase-generated wgMLST data. The identification of the operon in subsp. *salamae*, *arizonae*, *diarizonae*, and *houtenae* but not in subsp. *indica* is interesting. Indeed, these subspecies share a lower ancestry with subsp. *enterica* than subsp. *indica*, which is a sister phylogroup of *enterica* (37), suggesting that the operon may have spread among all the *enterica* subspecies.

The complete or partial *pefI-srgC* operon was found either on the chromosome or on related plasmids. Some serovars harbor an incomplete operon, which colocalized with the *sef* fimbrial operon within SPI-10. The integration of this genetic element was previously described for serovars Typhi, Paratyphi A, and Enteritidis, although the reason behind the necessity for the latter to carry both a plasmid-borne and chromosomal copy of these ORFs remains unknown (18, 35). Additionally, another study previously detected the presence of the pathogenicity island (through the presence of the *sef* operon) on genomes of *S.* Washington and S. Typhimurium isolates (38). However, no assemblies of *S.* Washington were retrieved in our data set, hindering us to predict the composition of the island. The same study did not detect the island on *S.* Dublin isolates, while our results demonstrate that the island might be retrieved on a large proportion of this serovar population. The presence of these ORFs on the chromosome of some strains of these serovars implies that they must play a role, although yet undetermined, in these strains.

When the operon was found on plasmids, some strains appeared to carry plasmids very similar, both in size and gene content, to the previously described virulence plasmids of S. Typhimurium (p14028s) or S. Enteritidis (pSENV). While p14028s exhibits the genes necessary for self-conjugation and was previously characterized for its ability to promote it both *in vitro* and *in vivo* in the distal portion of the small intestine (39, 40), pSENV has suffered severe degradation along its evolutionary course, especially deletions of the *tra* operon, making it unable to promote self-conjugation. The detection of the operon with a different architecture on *spv*^−^ plasmids using wgMLST ST and the sequences of *rck*^+^ assemblies of few serovars (*S.* Poona for example) also raised questions concerning the virulence plasmids and their evolution. Combined with previous works on pRST98, a hybrid virulence plasmid of S. Typhi carrying both drug resistance genes and virulence genes, including *rck* (41, 42), our results encourage us to investigate further on the evolutionary mechanisms explaining the emergence of these plasmids.

Whatever the genetic supports, we observed a remarkable low diversity in the sequence of the *rck* ORF. This relative stability of the nucleotide sequence among the serovars has direct repercussions on the amino acid sequence of the protein, which also displays great conservation. The impact of substitutions in virulence determinants on their functional properties has already been documented for some of them. Notably, several studies designed by the Schifferli lab have highlighted a host-specific tropism of strains according to FimH adhesin allelic variants (34, 43). More recently, they characterized the functionality and the affinity of several allelic variants of the PagN invasin of Salmonella with its interactants and highlighted the importance of two amino acids in PagN binding to laminin and mammalian cells as well as in cell invasion (33). Here, the functionality of 14 variants of Rck, engineered in our laboratory to reproduce the proteins presenting polymorphisms in the different domains of the protein (i.e., signal peptide, all four extracellular loops, transmembrane β-barrel regions), was assessed. Only three variants (Rck_H_, Rck_I_, and Rck_M_) displayed altered function (i.e., resistance to complement-mediated killing and/or invasion). The impact of the polymorphism in Rck_M_ (Δ114 to 115) confirmed the essential role of the third loop (G116 to G143) in all the virulence properties of the protein yet described (3, 10). However, the explanation of the decrease in invasion induced by the two other variants remains unclear. One can hypothesize that the I38V substitution harbored by the Rck_H_ variant affects the affinity of the first loop with EGFR in a cooperation between loops during the adhesion/invasion process. A similar suggestion has already been made by Cirillo et al. when they observed that the addition of the D43K substitution to Rck mutants with a G118D substitution induced a more important decrease in serum resistance than that conferred by Rck G118D (3). As both isoleucine and valine are hydrophobic amino acids, these mutations probably did not greatly alter the first loop structure but rather the affinity of the variant with its interactant. The last phenotype observed, induced by the A17V substitution found on Rck_I_, might, however, be the result of the successive mutations of the signal peptide, possibly reducing, but not abrogating, protein addressing to the outer membrane. Indeed, as the variant Rck_I_ was not altered in the complement resistance assay, it means that there is a sufficient quantity of Rck protein correctly expressed in the outer membrane to ensure the recruitment of inhibitors of the different complement pathways involved in the action of Rck. However, this seems not sufficient to properly mediate the interaction with EGFR, resulting in a decrease in the invasion rate. These two hypotheses require deeper investigation to fully understand the phenotypes of these variants. However, these three altered variants are harbored by only 4 strains from 4 different serovars, supporting the idea that the function of the Rck protein is generally very well conserved independently of the variant. As the functionality of the remaining Rck variants is preserved, the other amino acids presenting polymorphisms do not seem to be involved in resistance and invasion phenotypes, suggesting that essential amino acids for both phenotypes remain conserved among the variants.

Finally, it is important to point out that this study does not consider the multiplicity of regulation patterns of expression of the *pefI-srgC* operon. To this day, most of the studies concerning the *pefI-srgC* operon focused on the one retrieved on S. Typhimurium virulence plasmid, and consequently only the regulatory scheme of this operon’s transcription has been described (17, 44, 45). As the promoter region of the *pefI-srgC* operon is completely different between Typhimurium, Enteritidis, and Bovismorbificans serotypes, the results concerning the regulation of *rck* from S. Typhimurium cannot be extended to Enteritidis and Bovismorbificans serotypes. In S. Typhimurium, *pefI-srgC* transcription depends on the presence of acyl-homoserine lactones (AHLs) through the quorum-sensing regulator SdiA and has been postulated to occur within the mammalian gastrointestinal tract in response to other bacterial population/community AHL production (45). However, work performed in our laboratory to determine the kinetics of expression of the operon during infection in murine models of gastroenteritis and typhoid fever using chromosomal transcriptional fusions demonstrated that this signaling pathway is not activated under these physiological conditions. These unpublished results agree with those obtained by B. Ahmer’s lab, which showed that S. Typhimurium does not detect AHLs during its transit through the gastrointestinal tract of several mammal species (i.e., Guinea pigs, rabbits, pigs, chicks, calves, and mice) (46). More recent work identified FliA, a broadly conserved σ factor, as a component of this regulatory scheme, acting both and at different levels on *sdiA* and *rck* transcription (47). The investigations concerning the regulation of *rck* should clearly be deepened to obtain a more comprehensive picture of the role of this invasin in Salmonella pathogenesis.

To conclude, this work extended the distribution of the *pefI-srgC* operon in the Salmonella genus and pointed out the presence of different genetic elements carrying the operon, thus raising questions concerning the mechanisms governing these acquisitions and virulence plasmid evolution. Moreover, the Rck virulence protein was found to be very well conserved, and this study highlighted the importance of the first and third loops on its virulence properties. Altogether, these results suggest a more important role of the Rck protein than previously expected in the virulence of Salmonella.

## MATERIALS AND METHODS

### Bioinformatic analyses.

Reference genomes of 20 Salmonella serovars were downloaded from the ATCC website (https://www.lgcstandards-atcc.org/) and submitted to a BLAST search (identity threshold: 70%; coverage threshold: 80%) (48) for the *pefI*, *srgD*, *srgA*, *srgB*, *rck*, and *srgC* ORFs (Table 2). Whole-genome multilocus sequence typing (wgMLST) profiles of 188,233 Salmonella strains associated with coherent Salmonella *in silico* typing resource (SISTR) serovar prediction were retrieved from the public database Enterobase (25, 49) on 27 March 2019. Allelic data at the *pefI* (SLT-BT0051), *srgD* (SLT-BT0041), *srgA* (SLT-BT0031), *srgB* (SLT-BT0021), *rck* (SLT-BT0011), and *srgC* (SLT-BT0001) loci were also retrieved from the database as well as *rck*^+^ contigs of interest. The distribution of these ORFs was studied in the two Salmonella species and all six S. enterica subspecies. Genome assemblies of *rck*^+^ strains of interest (i.e., all except those belonging to serovars S. Typhimurium, S. Enteritidis, and *S.* Bovismorbificans) were downloaded from Enterobase. For both ATCC reference genomes and Enterobase assemblies, *rck*-carrying contigs were identified using BLAST then extracted and compared to reference sequences (Table 2) using EasyFig v2.2.5 (50) to determine the genetic environment surrounding the *rck* ORF. The allelic data at the *spvR* (SLT-BT0251), *spvA* (SLT-BT0241), *spvB* (SLT-BT0231), *spvC* (SLT-BT0221), and *spvD* (SLT-BT0211) loci were also downloaded from Enterobase and used to predict the presence of a virulence plasmid in the corresponding genomes when the *rck*^+^ contigs were too short to perform a proper comparison. Nucleotide sequences of each *rck* allele as well as their respective translated sequences were aligned and compared to each other using Geneious (v10.2.2) (https://www.geneious.com/). A neighbor-joining tree was calculated using the Jukes-Cantor model in Geneious. Heat maps were generated using the R-Package heatmaply (51).

**TABLE 2:** Reference genomes and sequences used in this study

Serovar	Molecule type	Strain or plasmid name	Accession no.^*a*^
Abaetetuba	Complete genome	ATCC 35640	/
Abony	Complete genome	ATCC_BAA_2162	/
Bovismorbificans	Complete genome	ATCC_BAA_1740	/
Branderup	Complete genome	ATCC_BAA_1739	/
Choleraesuis	Complete genome	ATCC 7001	/
Choleraesuis	Complete genome	ATCC 10708	/
Dublin	Complete genome	ATCC 39184	/
Infantis	Complete genome	ATCC 51741	/
Infantis	Complete genome	ATCC_BAA_1675	/
Montevideo	Complete genome	ATCC 8387	/
Montevideo	Complete genome	ATCC_BAA_1735	/
Muenchen	Complete genome	ATCC_BAA_1674	/
Muenchen	Complete genome	ATCC_BAA_1676	/
Muenster	Complete genome	ATCC_BAA_1575	/
Paratyphi A	Complete genome	ATCC 9150	/
Paratyphi B	Complete genome	ATCC BAA_1250	/
Paratyphi B	Complete genome	ATCC_BAA_1584	/
Paratyphi C	Complete genome	ATCC_BAA_1715	/
Poona	Complete genome	ATCC_BAA_1673	/
Sendai	Complete genome	ATCC BAA_1586	/
Senftenberg	Complete genome	ATCC_BAA_1736	/
Senftenberg	Complete genome	ATCC_43845	/
Stanley	Complete genome	ATCC_BAA_1737	/
Stanley	Complete genome	ATCC 7308	/
Thompson	Complete genome	ATCC_BAA_1738	/
Typhi	Complete genome	ATCC 700931	/
Urbana	Complete genome	ATCC 9261	/
Typhimurium	Virulence plasmid	p14028s (from 14028s)	CP001362
Enteritidis	Virulence plasmid	pSENV (from LA5)	HE663166
Bovismorbificans	Virulence plasmid	pVIRBov	HF969016
Enteritidis	Chromosome	LA5	CAGR00000000
Paratyphi C	Virulence plasmid	pSPCV	CP000858

a /, no data.

### Rck structure modeling.

The 3D structure of Rck was predicted based on the sequence of S. Typhimurium 14028 using the protein structure homology-modeling server SWISS-MODEL (52). Two models presenting GMQE and QMEAN over 0.6 and −2.6, respectively, were generated based on the crystal structures of Ail and OmpX. The second model was chosen to be shown in Fig. 5A, as it exhibits the best overall ERRAT quality factor (88.49). The picture was formatted using the Swiss-Pdbviewer software (http://www.expasy.org/spdbv/) (53).

### Bacterial strains and plasmid construction.

The bacterial strains and plasmids used in this study are listed in Table 3. *rck* was amplified by PCR from the genomes of S. Enteritidis LA5 or *S.* Bovismorbificans 201910217 strains (carrying *rck* alleles number 3 and number 15, respectively) using primers rck-BamHI-fwd and rck-SalI-rev (Table S1 in the supplemental material). The subsequent PCR products were digested by restriction enzymes BamHI and SalI, ligated within BamHI/SalI-digested pSUP202, and used to transform chemically competent E. coli MC1061 bacteria. Recombinant bacteria were selected on tryptic soy agar (TSA) plates containing chloramphenicol (30 μg/ml). Plasmids were checked by PCR and sequencing. The transcription of *rck* in these recombinant pSUP202 plasmids is under the control of the promoter of the Tc^r^ gene, which leads to constitutive expression of Rck.

**TABLE 3:** Strains and plasmids used in this study

Strain or plasmid	Relevant characteristic(s)^*a*^	Source or reference
Strains		
LA5	S. Enteritidis strain harboring *rck* variant no. 3	54
201910217	*S.* Bovismorbificans strain harboring *rck* variant no. 15	55
MC1061	E. coli *hsdR mcrB araD*139 Δ(*araABC*-*leu*)*7679* Δ*lacX74 galU galK rpsL thi*	56
Plasmids		
pSUP202	Cloning vector (Cb^r^, Cm^r^, Tc^r^)	57
pSUP202-rck-A	pSUP202 containing S. Typhimurium 14028 *rck* ORF (variant no. 1) and its RBS (Cm^r^, Cb^r^)	14
pSUP202-rck-B	pSUP202 containing S. Enteritidis LA5 *rck* ORF (variant no. 3) and its RBS (Cm^r^, Cb^r^)	This work
pSUP202-rck-C	pSUP202 containing *S*. Bovismorbificans 201910217 *rck* ORF (variant no. 15) and its RBS (Cm^r^, Cb^r^)	This work
pSUP202-rck-D	pSUP202 containing *rck* gene variant no. 7 and its RBS (Cm^r^, Cb^r^)	This work
pSUP202-rck-E	pSUP202 containing *rck* gene variant no. 8 and its RBS (Cm^r^, Cb^r^)	This work
pSUP202-rck-F	pSUP202 containing *rck* gene variant no. 16 and its RBS (Cm^r^, Cb^r^)	This work
pSUP202-rck-G	pSUP202 containing *rck* gene variant no. 19 and its RBS (Cm^r^, Cb^r^)	This work
pSUP202-rck-H	pSUP202 containing *rck* gene variant no. 22 and its RBS (Cm^r^, Cb^r^)	This work
pSUP202-rck-I	pSUP202 containing *rck* gene variant no. 53 and its RBS (Cm^r^, Cb^r^)	This work
pSUP202-rck-J	pSUP202 containing *rck* gene variant no. 60 and its RBS (Cm^r^, Cb^r^)	This work
pSUP202-rck-K	pSUP202 containing *rck* gene variant no. 73 and its RBS (Cm^r^, Cb^r^)	This work
pSUP202-rck-L	pSUP202 containing *rck* gene variant no. 80 and its RBS (Cm^r^, Cb^r^)	This work
pSUP202-rck-M	pSUP202 containing *rck* gene variant no. 83 and its RBS (Cm^r^, Cb^r^)	This work
pSUP202-rck-N	pSUP202 containing *rck* gene variant no. 87 and its RBS (Cm^r^, Cb^r^)	This work

a Cb^r^, carbenicillin resistance; Cm^r^, chloramphenicol resistance; Tc^r^, tetracycline resistance; RBS, ribosome binding site.

Other pSUP202-rck derivatives were obtained by PCR using primers specifically designed to amplify a whole pSUP202-rck plasmid (inverse PCR) and introduce nucleotide changes in particular sites of the *rck* gene (Table S1). PCR products were then phosphorylated at their 5′ end using T4 polynucleotide kinase as described by the manufacturer (Promega), self-ligated, and used to transform chemically competent E. coli MC1061 bacteria. Recombinant bacteria were selected on TSA plates containing chloramphenicol (30 μg/ml). The resulting plasmids were verified by sequencing.

### Gentamicin protection assay.

Invasion capacities of E. coli MC1061 overexpressing Rck variants were assessed on the human trophoblastic JEG-3 cell line (ATCC HTB-36). Cells were grown in 24-well culture plates in their appropriate growth medium (minimal essential medium [MEM] GlutaMAX supplemented with 10% fetal calf serum, sodium pyruvate [1 mM], and nonessential amino acids [1%]) until a confluent monolayer had formed. E. coli strains were cultured overnight in tryptic soy broth (TSB) at 37°C without agitation. Cells were infected for 60 min at 37°C with 300 μl of bacterial suspension in MEM GlutaMAX at a bacteria-to-cell ratio of 20:1. Invasion capacities were determined by incubating cells with culture medium supplemented with gentamicin (100 μg/ml) for 90 min to eliminate extracellular bacteria. The cells were then washed with their appropriate growth medium then lysed using cold distilled water. Viable bacteria were counted after plating on TSA supplemented with chloramphenicol (30 μg/ml) using an automated spiral plater.

### Resistance to complement assay.

Mid-log-phase bacterial cultures in TSB medium were washed two times in phosphate-buffered saline (PBS), and their concentration was adjusted to 10^8^ bacteria per ml. Bacterial suspensions (100 μl) were then incubated with 100 μl of normal or decomplemented swine serum for 60 min at 37°C. Viable bacteria were counted after plating on TSA supplemented with chloramphenicol (30 μg/ml) using an automated spiral plater. Complement system efficacy was determined as the difference of the number of surviving bacteria between decomplemented and normal serums and expressed in log kill.

### Data availability.

All supporting data and protocols have been provided within the article or through supplementary data files. Data from Enterobase as well as those originating from the ATCC database are publicly available.
