# Supplementary material for: The Invasin and Complement-Resistance Protein Rck of Salmonella is More Widely Distributed than Previously Expected
Source: Microbiol Spectr. 2021 Oct 27;9(2):e01457-21. doi: 10.1128/Spectrum.01457-21 (PMC8549739; doi:10.1128/Spectrum.01457-21)
Supplement: SUPPLEMENTAL FILE 1 — Supplemental material. Download Spectrum.01457-21-s0001.pdf, PDF file, 8.3 MB [file spectrum.01457-21-s0001.pdf]

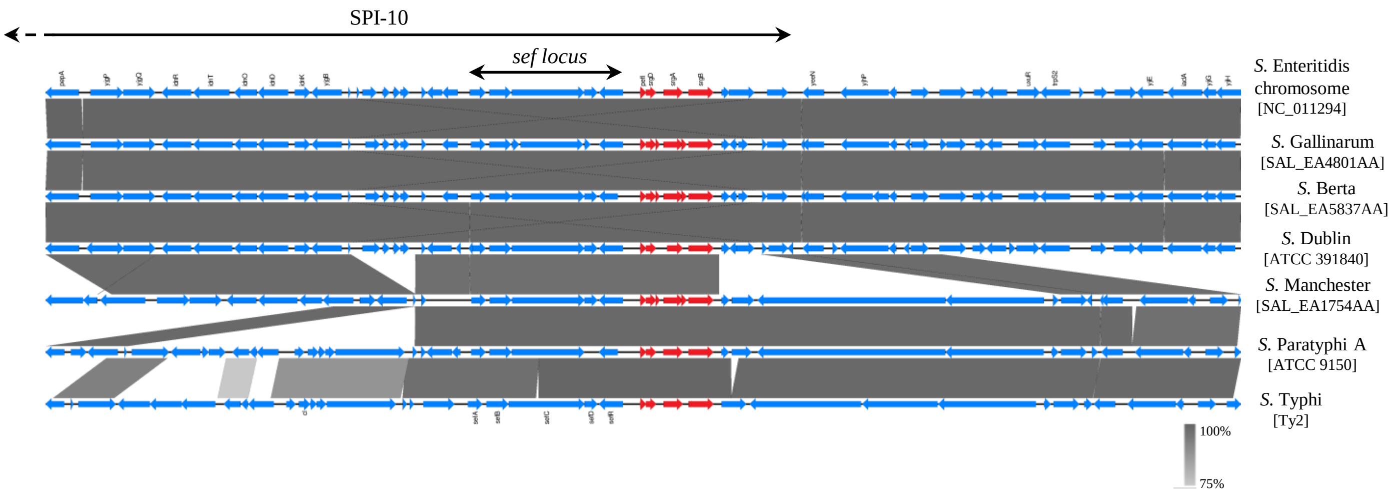

Figure S1. Alignment of the SPI-10 loci found in serovars Gallinarum, Berta and Manchester. SPI-10 loci of representative assemblies of *S*. Gallinarum, Berta and Manchester were compared with the previously described (Fig. 1) SPI-10 loci of *S*. Enteritidis, *S*. Dublin, *S*. Paratyphi A and *S*. Typhi. The ORFs belonging to the *pefI-srgC* operon are indicated by red arrows. Grey areas between the schematic sequences denote nucleotide identity with a gradient specified in the bottom-right corner of the figure.

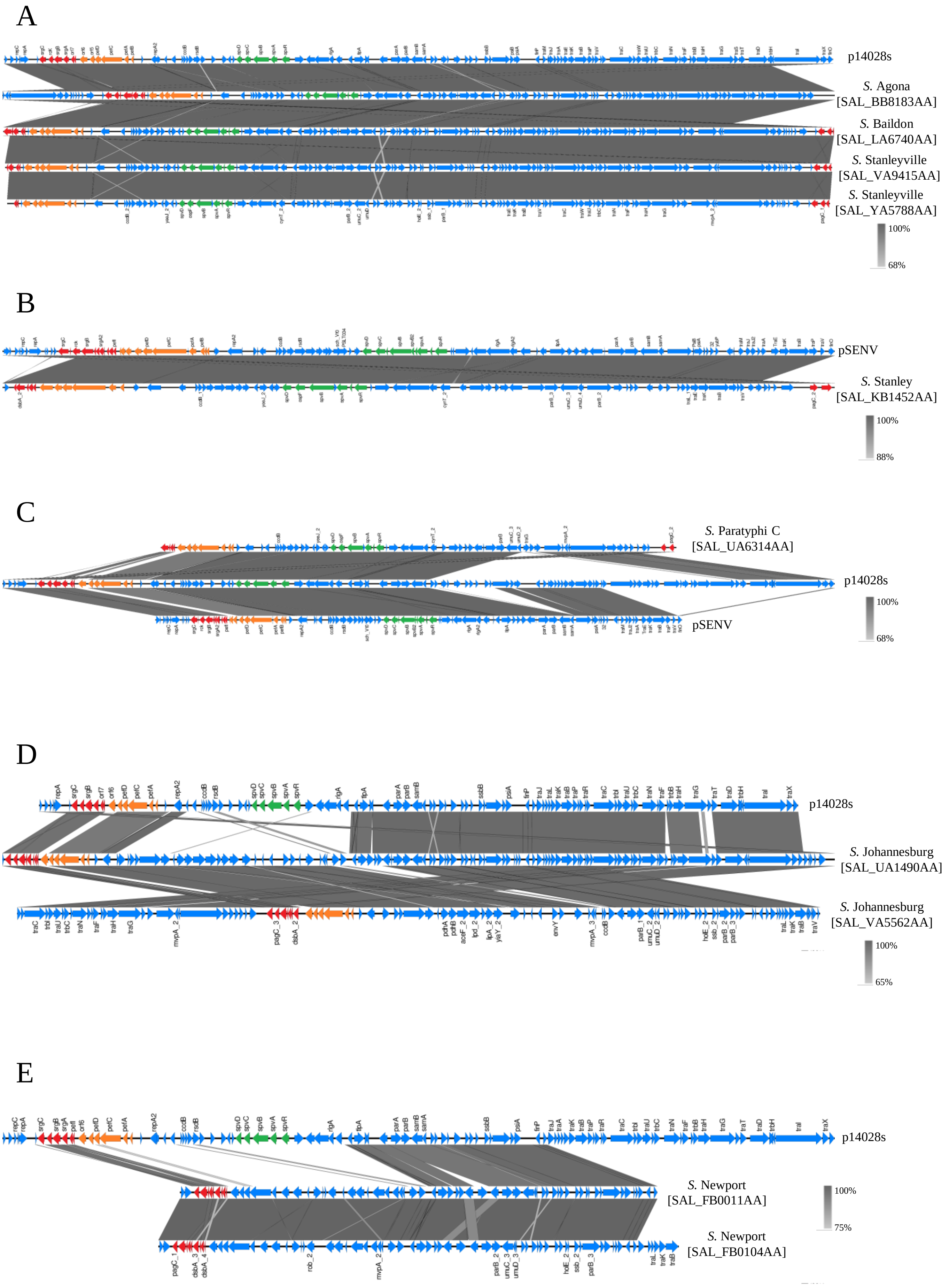
A
p14028s
S. Agona [SAL_BB8183AA]
S. Baildon [SAL_LA6740AA]
S. Stanleyville [SAL_VA9415AA]
S. Stanleyville [SAL_YA5788AA]
100%
68%
B
pSENV
S. Stanley [SAL_KB1452AA]
100%
88%
C
S. Paratyphi C [SAL_UA6314AA]
p14028s
pSENV
100%
68%
D
p14028s
S. Johannesburg [SAL_UA1490AA]
S. Johannesburg [SAL_VA5562AA]
100%
65%
E
p14028s
S. Newport [SAL_FB0011AA]
S. Newport [SAL_FB0104AA]
100%
75%

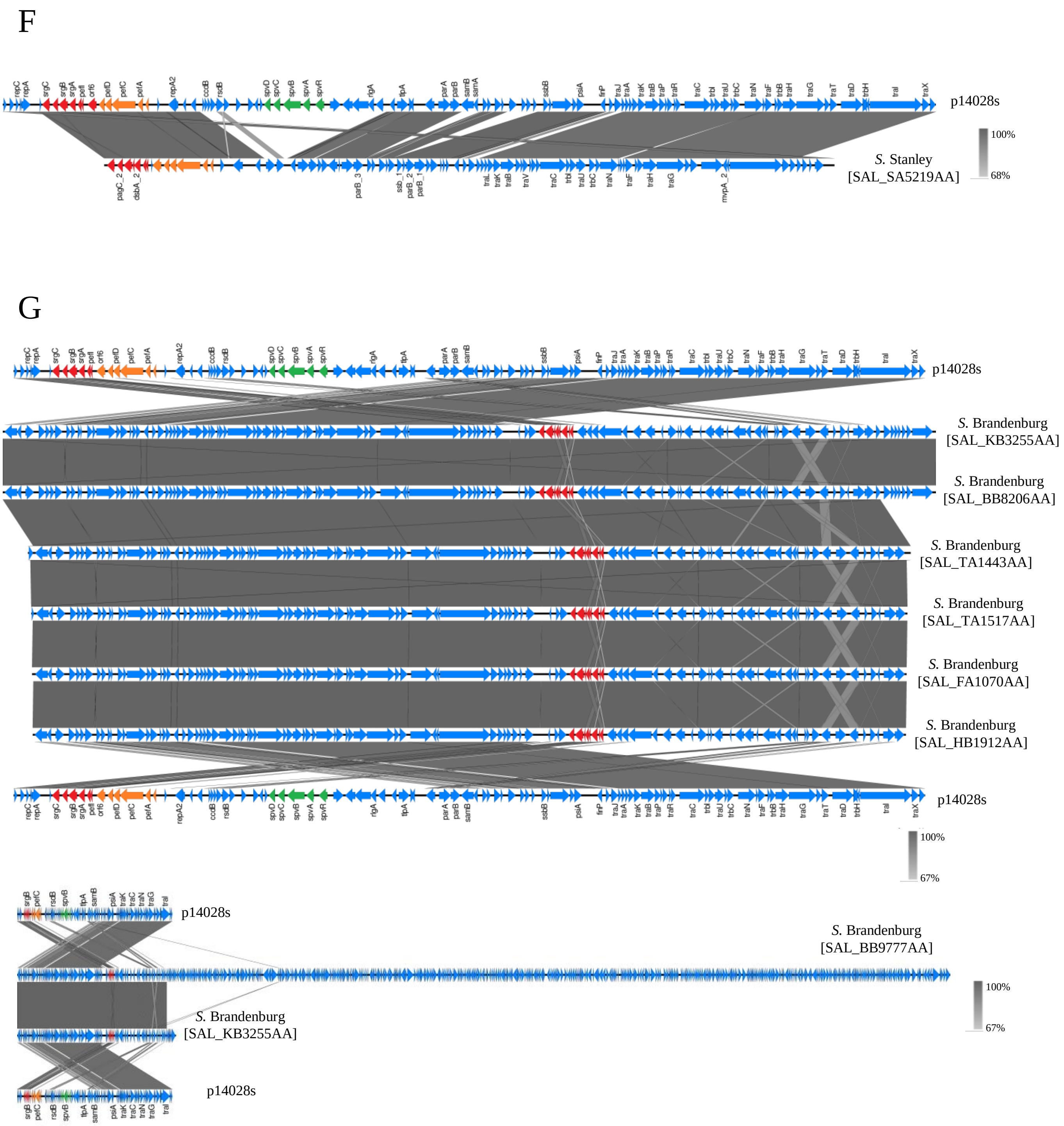

Figure S2. Comparison of *rck*+ regions among serovars.
(A-B) Representative alignment of *rck*+ contigs retrieved (A) from *S*. Agona, *S*. Baildon and *S*. Stanleyville assemblies with p14028s *rck*+ region, or (B) from S. Stanley assembly with pSENV. (C) Representative alignment of *rck*+ contig retrieved from *S*. Paratyphi C assembly with both p14028s and pSENV *rck* loci. (D-G) Alignment of representative *rck+* contigs retrieved from *S*. Johannesburg (D), *S*. Newport (E), *S*. Stanley (F) and *S*. Brandenburg (G) assemblies with p14028s. On the sequences displaying consistent annotations, red, orange and green arrows represent the ORFs of the *pefI-srgC* operon, the *pef* operon and the *spv* locus respectively. Grey areas between the schematic sequences denote nucleotide identity with a gradient specified in the figures.

Table S1. Primers used in this study

Primer name	Sequence (5'-3')[a]	Description
rck-BamHI-fwd	CTCGGATCCCTTAACTGTGTTCAGGGAGTTTTATCATG	Cloning of *rck* from *S*. Enteritidis LA5 and *S*. Bovismorbificans 201910217 in pSUP202
rck-SalI-rev	TCTGTCGACTCCCTTTCCTGCTCTCCGTTATC	Cloning of *rck* from *S*. Enteritidis LA5 and *S*. Bovismorbificans 201910217 in pSUP202
A14V.1	CAGTCGGGCTGGCTGCCGTACCGG	Construction of *rck* variants in pSUP202
A14V.2	CGGACAGCAGCAGTGAGGACAGAACGAT	Construction of *rck* variants in pSUP202
A17V.1	TGGTTGCCGTACCGGTGGCACAGGCT	Construction of *rck* variants in pSUP202
A17V.2	GCCCGGCTGCGGACAGCAGCAG	Construction of *rck* variants in pSUP202
A111T.1	TGTATACACTGGCCGGTGCCGGTAC	Construction of *rck* variants in pSUP202
A111T.2	GAGATACCAGCTCGTTCACACGG	Construction of *rck* variants in pSUP202
A115V.1	GGTGTCGGTACCGGCAGGGCTGAAG	Construction of *rck* variants in pSUP202
A115V.2	GGCCAGTGCATACAGAGATACCAGCTCG	Construction of *rck* variants in pSUP202
G73E.1	GCGAAGAGTCCGGTGGCATTCCTGGCG	Construction of *rck* variants in pSUP202
G73E.2	GCTTACCTGACTGCCAACTGAACGACGCC	Construction of *rck* variants in pSUP202
R45H.1	TCCATGGGGTGAACCTGAAATACCGCT	Construction of *rck* variants in pSUP202
R45H.2	TATCCTTAAAATGCTCTATCCGGCTCTG	Construction of *rck* variants in pSUP202
R125H.1	GAGCATATCAGCATGCCGGGATACAACGG	Construction of *rck* variants in pSUP202
R125H.2	TTTCACTTCAGCCCTGCCGGTA	Construction of *rck* variants in pSUP202
V177I.1	GGCATTAACGTTGGTGTCGGTTACCG	Construction of *rck* variants in pSUP202
V177I.2	GTTCAGTTTCGCTGCGCCAAC	Construction of *rck* variants in pSUP202
del114/115.1	GGTACCGGCAGGGCTGAAGTG	Construction of *rck* variants in pSUP202
del114/115.2	GGCCAGGGCATACAGAGATACCAGC	Construction of *rck* variants in pSUP202
F144V.1	GGGGTTGCCTGGGGAGCCGGCG	Construction of *rck* variants in pSUP202
F144V.2	CGTTCTGCGCTCCGAACCCGTGAACC	Construction of *rck* variants in pSUP202
I38V.1	CGGGTAGAGCATTTTAAGGATATCCGTGGGGTG	Construction of *rck* variants in pSUP202
I38V.2	GCTCTGGGCATATCCCACCGACA	Construction of *rck* variants in pSUP202
M62I.1	TGATAGCGTCGTTCAGCTGGCAGTCAGGT	Construction of *rck* variants in pSUP202
M62I.2	GTCCCAGCGGCGTCTGAGCCTC	Construction of *rck* variants in pSUP202
S167G.1	GGAGGTAAAGTTGGCGCAGCGAAACTGA	Construction of *rck* variants in pSUP202
S167G.2	CTCATAGCCCAGGTCGATGACCAC	Construction of *rck* variants in pSUP202
V103M.1	CGTATGAACGAGCTGGTATCTCTGTATGCCCTGG	Construction of *rck* variants in pSUP202
V103M.2	GACAGCGGGACCCGCCATCAGC	Construction of *rck* variants in pSUP202

Table S2. Allelic profiles of the 188,233 *Salmonella* strains issued from Enterobase at the *pefI-srgC* and *spv* loci

Strains		Serovar (Serotype Prediction (SISTR))	*pefI-srgC* operon						*spv* locus				
Barcode	Name		pefI	srgD	srgA	srgB	rck	srgC	spvR	spvA	spvB	spvC	spvD
SAL_EA7688AA	77346	Aba	0	0	0	0	0	0	0	0	0	0	0
SAL_FA9319AA	CFSAN046216	Aba	0	0	0	0	0	0	0	0	0	0	0
SAL_BA2174AA	FDA00009191	Aba	0	0	0	0	0	0	0	0	0	0	0
SAL_CA5571AA	78760	Aba	0	0	0	0	0	0	0	0	0	0	0
SAL_PA8932AA	186564	Aba	0	0	0	0	0	0	0	0	0	0	0
SAL_QA7445AA	256170	Aba	0	0	0	0	0	0	0	0	0	0	0
SAL_QA9953AA	286808	Aba	0	0	0	0	0	0	0	0	0	0	0
SAL_RA7401AA	387531	Aba	0	0	0	0	0	0	0	0	0	0	0
SAL_SA7526AA	598900	Aba	0	0	0	0	0	0	0	0	0	0	0
SAL_VA9599AA	286807	Aba	0	0	0	0	0	0	0	0	0	0	0
SAL_EA6714AA	FDA00009683	Abaetetuba	0	0	0	0	0	0	0	0	0	0	0
SAL_DA8111AA	FAR0086	Abaetetuba	0	0	0	0	0	0	0	0	0	0	0
SAL_CA7708AA	FDA00002769	Abaetetuba	0	0	0	0	0	0	0	0	0	0	0
SAL_DA9987AA	FAR0032	Abaetetuba	0	0	0	0	0	0	0	0	0	0	0
SAL_FA4007AA	FDA00001328	Abaetetuba	0	0	0	0	0	0	0	0	0	0	0
SAL_EA6854AA	FDA00004116	Abaetetuba	0	0	0	0	0	0	0	0	0	0	0
SAL_EA6774AA	FDA00004118	Abaetetuba	0	0	0	0	0	0	0	0	0	0	0
SAL_EA6713AA	FDA00009682	Abaetetuba	0	0	0	0	0	0	0	0	0	0	0
SAL_EA0236AA	FDN0004	Abaetetuba	0	0	0	0	0	0	0	0	0	0	0
SAL_DA8518AA	FSE0012	Abaetetuba	0	0	0	0	0	0	0	0	0	0	0
SAL_DA8221AA	4498-sc-2010-09-29-985	Abaetetuba	0	0	0	0	0	0	0	0	0	0	0
SAL_DA3815AA	CFSAN023234	Abaetetuba	0	0	0	0	0	0	0	0	0	0	0
SAL_DA2944AA	CFSAN023684	Abaetetuba	0	0	0	0	0	0	0	0	0	0	0
SAL_DA2713AA	FDN0157	Abaetetuba	0	0	0	0	0	0	0	0	0	0	0
SAL_CA7972AA	FDA00000995	Abaetetuba	0	0	0	0	0	0	0	0	0	0	0
SAL_CA7681AA	FDA00003057	Abaetetuba	0	0	0	0	0	0	0	0	0	0	0
SAL_CA2119AA	85695	Abaetetuba	0	0	0	0	0	0	0	0	0	0	0
SAL_CA1436AA	85694	Abaetetuba	0	0	0	0	0	0	0	0	0	0	0
SAL_BA6827AA	FDA00002768	Abaetetuba	0	0	0	0	0	0	0	0	0	0	0
SAL_BA6696AA	FDA00008866	Abaetetuba	0	0	0	0	0	0	0	0	0	0	0
SAL_BA2003AA	FDA00000417	Abaetetuba	0	0	0	0	0	0	0	0	0	0	0
SAL_BA1558AA	FSIS1500987	Abaetetuba	0	0	0	0	0	0	0	0	0	0	0
SAL_GA2067AA	BCW_3073	Abaetetuba	0	0	0	0	0	0	0	0	0	0	0
SAL_GA1717AA	1350/12	Abaetetuba	0	0	0	0	0	0	0	0	0	0	0
SAL_GA5079AA	PNUSAS002648	Abaetetuba	0	0	0	0	0	0	0	0	0	0	0
SAL_GA8757AA	FDA00001447	Abaetetuba	0	0	0	0	0	0	0	0	0	0	0
SAL_HA0837AA	FDA00010720	Abaetetuba	0	0	0	0	0	0	0	0	0	0	0
SAL_HA0838AA	FDA00010721	Abaetetuba	0	0	0	0	0	0	0	0	0	0	0
SAL_HA1150AA	ADRDL-R248	Abaetetuba	0	0	0	0	0	0	0	0	0	0	0
SAL_HA2818AA	NSUOC-0099-W4X	Abaetetuba	0	0	0	0	0	0	0	0	0	0	0
SAL_HA6712AA	NSUOC-0103-W6H	Abaetetuba	0	0	0	0	0	0	0	0	0	0	0
SAL_HA6710AA	NSUOC-0100-W4H	Abaetetuba	0	0	0	0	0	0	0	0	0	0	0
SAL_HA6729AA	NSUOC-0104-W6X	Abaetetuba	0	0	0	0	0	0	0	0	0	0	0
SAL_HA9145AA	NSUOC-0170-W4B	Abaetetuba	0	0	0	0	0	0	0	0	0	0	0
SAL_IA3834AA	PNUSAS008066	Abaetetuba	0	0	0	0	0	0	0	0	0	0	0
SAL_JA0311AA	NSUOC-0272-W4H	Abaetetuba	0	0	0	0	0	0	0	0	0	0	0
SAL_JA1430AA	NSUOC-0289-W4X	Abaetetuba	0	0	0	0	0	0	0	0	0	0	0
SAL_JA1429AA	NSUOC-0288-W4H	Abaetetuba	0	0	0	0	0	0	0	0	0	0	0
SAL_JA4494AA	201702449	Abaetetuba	0	0	0	0	0	0	0	0	0	0	0
SAL_JA4516AA	PNUSAS012907	Abaetetuba	0	0	0	0	0	0	0	0	0	0	0
SAL_JA9562AA	NSUOC-0322-W6X	Abaetetuba	0	0	0	0	0	0	0	0	0	0	0
SAL_JA9108AA	PNUSAS015130	Abaetetuba	0	0	0	0	0	0	0	0	0	0	0
SAL_LA0982AA	FDA00012121	Abaetetuba	0	0	0	0	0	0	0	0	0	0	0
SAL_LA6705AA	PNUSAS023013	Abaetetuba	0	0	0	0	0	0	0	0	0	0	0
SAL_MA1180AA	PNUSAS022466	Abaetetuba	0	0	0	0	0	0	0	0	0	0	0
SAL_DA8508AA	FSE0031	Abaetetuba	0	0	0	0	0	0	0	0	0	0	0
SAL_BA3628AA	CFSAN024497	Abaetetuba	0	0	0	0	0	0	0	0	0	0	0
SAL_BA5441AA	CFSAN024605	Abaetetuba	0	0	0	0	0	0	0	0	0	0	0
SAL_DA6897AA	FAR0146	Abaetetuba	0	0	0	0	0	0	0	0	0	0	0
SAL_CA8924AA	FDA00008602	Abaetetuba	0	0	0	0	0	0	0	0	0	0	0
SAL_CA7233AA	FDA00001055	Abaetetuba	0	0	0	0	0	0	0	0	0	0	0
SAL_BA5161AA	ATCC 35640	Abaetetuba	0	0	0	0	0	0	0	0	0	0	0
SAL_FA2270AA	FDA00001322	Abaetetuba	0	0	0	0	0	0	0	0	0	0	0
SAL_EA6855AA	FDA00004117	Abaetetuba	0	0	0	0	0	0	0	0	0	0	0
SAL_EA6712AA	FDA00009681	Abaetetuba	0	0	0	0	0	0	0	0	0	0	0
SAL_IA0302AA	FDA00002541	Abaetetuba	0	0	0	0	0	0	0	0	0	0	0

Serovar	Profile *pefI-srgC*	Occurrence	Absolute frequency (Occurrence/Occurrence serovar)
Aba	0-0-0-0-0-0	10	/
Abaetetuba	0-0-0-0-0-0	68	/
Aberdeen	0-0-0-0-0-0	101	/
Abony	0-0-0-0-0-0	113	/
Abortusovis	0-0-0-0-0-0	5	/
Adabraka	0-0-0-0-0-0	2	/
Adelaide	0-0-0-0-0-0	423	/
Adjame	0-0-0-0-0-0	41	/
Agama	0-0-0-0-0-0	337	/
Agege	0-0-0-0-0-0	1	/
Ago	0-0-0-0-0-0	23	/
Agona	0-0-0-0-0-0	3243	0.998768094
	1-1-1-78-1-1	1	0.000307977
	0-3-5-83-3-0	1	0.000307977
	0-0-0-411-3-0	1	0.000307977
	16-0-0-0-0-0	1	0.000307977
Agoueve	0-0-0-0-0-0	18	/
Ahuza	0-0-0-0-0-0	3	/
Alabama	0-0-0-0-0-0	3	/
Alachua	0-0-0-0-0-0	132	/
Alamo	0-0-0-0-0-0	3	/
Albany	0-0-0-0-0-0	342	/
Albert	0-0-0-0-0-0	1	/
Albuquerque	0-0-0-0-0-0	2	/
Altendorf	0-0-0-0-0-0	2	/
Altona	0-0-0-0-0-0	261	/
Amager	0-0-0-0-0-0	69	/
Amersfoort	0-0-0-0-0-0	16	/
Amherstiana	0-0-0-0-0-0	1	/
Aminatu	0-0-0-0-0-0	1	/
Amoutive	0-0-0-0-0-0	5	/
Amsterdam	0-0-0-0-0-0	82	/
Anatum	0-0-0-0-0-0	2605	/
Anderlecht	0-0-0-0-0-0	3	/
Anfo	0-0-0-0-0-0	9	/
Angoda	0-0-0-0-0-0	2	/
Ank	0-0-0-0-0-0	4	/
Antsalova	0-0-0-0-0-0	4	/
Apapa	0-0-0-0-0-0	47	/
Apeyeme	0-0-0-0-0-0	24	/
Aqua	0-0-0-0-0-0	4	/
Arechavaleta	0-0-0-0-0-0	57	0.95
	2-2-3-0-0-0	1	0.016666667
	2-2-3-3-0-0	2	0.033333333
Aschersleben	0-0-0-0-0-0	3	/
Astridplein	0-0-0-0-0-0	1	/
Augustenborg	0-0-0-0-0-0	37	/
Ayinde	0-0-0-0-0-0	1	/
Azteca	0-0-0-0-0-0	1	/
Baguida	0-0-0-0-0-0	2	/
Baildon	1-1-1-4-1-1	2	0.012903226
	0-0-0-0-0-0	153	0.987096774
Bakau	0-0-0-0-0-0	1	/
Ball	0-0-0-0-0-0	8	/
Bamboye	0-0-0-0-0-0	1	/
Bangkok	0-0-0-0-0-0	6	/
Bareilly	0-0-0-0-0-0	1643	0.998784195
	5-4-20-21-0-0	1	0.000607903
	1-1-1-4-0-0	1	0.000607903
Barmbek	0-0-0-0-0-0	2	/
Bassadji	0-0-0-0-0-0	2	/
Batonrouge	0-0-0-0-0-0	2	/
Beaudesert	0-0-0-0-0-0	9	/
Bere	0-0-0-0-0-0	38	/
Bergen	0-0-0-0-0-0	10	/
Berlin	0-0-0-0-0-0	1	/
	5-4-6-64-0-0	615	0.904411765
	5-4-6-65-0-0	45	0.066176471

SAL_LA0758AA	FDA00012122	Abaetetuba	0	0	0	0	0	0	0	0	0	0	0		**Berta**	44-4-6-64-0-0	14	0.020588235
SAL_MA6811AA	FDA00001525	Abaetetuba	0	0	0	0	0	0	0	0	0	0	0			5-4-143-64-0-0	4	0.005882353
SAL_NA7627AA	NC_SI06	Abaetetuba	0	0	0	0	0	0	0	0	0	0	0			5-4-6-270-0-0	1	0.001470588
SAL_PA2747AA	PNUSAS039214	Abaetetuba	0	0	0	0	0	0	0	0	0	0	0			5-4-6-328-0-0	1	0.001470588
SAL_PA3505AA	CFSAN079977	Abaetetuba	0	0	0	0	0	0	0	0	0	0	0		**Bietri**	0-0-0-0-0-0	5	/
SAL_SA7303AA	CFSAN085687	Abaetetuba	0	0	0	0	0	0	0	0	0	0	0		**Bispebjerg**	0-0-0-0-0-0	32	0.842105263
SAL_SA7304AA	CFSAN085686	Abaetetuba	0	0	0	0	0	0	0	0	0	0	0			1-6-10-18-5-2	1	0.026315789
SAL_SA7792AA	267004	Abaetetuba	0	0	0	0	0	0	0	0	0	0	0			1-17-10-18-5-2	4	0.105263158
SAL_OA1366AA	PNUSAS034756	Abaetetuba	0	0	0	0	0	0	0	0	0	0	0			1-17-10-404-5-2	1	0.026315789
SAL_VA2544AA	433596	Abaetetuba	0	0	0	0	0	0	0	0	0	0	0		**Blancmesnil**	0-0-0-0-0-0	1	/
SAL_VA9773AA	262774	Abaetetuba	0	0	0	0	0	0	0	0	0	0	0		**Blijdorp**	0-0-0-0-0-0	13	/
SAL_VA9913AA	240176	Abaetetuba	0	0	0	0	0	0	0	0	0	0	0		**Blockley**	0-0-0-0-0-0	230	/
SAL_EA9127AA	CFSAN031743	Aberdeen	0	0	0	0	0	0	0	0	0	0	0		**Bokanjac**	0-0-0-0-0-0	1	/
SAL_CA6731AA	66453	Aberdeen	0	0	0	0	0	0	0	0	0	0	0		**Bonariensis**	0-0-0-0-0-0	38	/
SAL_EA4828AA	FDA00004200	Aberdeen	0	0	0	0	0	0	0	0	0	0	0		**Bovismorbificans**	12-15-40-62-15-0	8	0.012251149
SAL_EA2472AA	FDA00001205	Aberdeen	0	0	0	0	0	0	0	0	0	0	0			12-0-39-62-15-0	107	0.163859112
SAL_EA0763AA	FSW0029	Aberdeen	0	0	0	0	0	0	0	0	0	0	0			0-0-0-0-0-0	496	0.75957121
SAL_FA5956AA	FDA00010112	Aberdeen	0	0	0	0	0	0	0	0	0	0	0			12-0-39-0-15-0	40	0.061255743
SAL_FA5875AA	FDA00009804	Aberdeen	0	0	0	0	0	0	0	0	0	0	0			12-15-40-62-39-0	1	0.001531394
SAL_FA5876AA	FDA00009803	Aberdeen	0	0	0	0	0	0	0	0	0	0	0			12-0-39-0-4-0	1	0.001531394
SAL_FA2276AA	FDA00010006	Aberdeen	0	0	0	0	0	0	0	0	0	0	0		**Bradford**	0-0-0-0-0-0	1	/
SAL_FA0818AA	FDA00009872	Aberdeen	0	0	0	0	0	0	0	0	0	0	0		**Braenderup**	0-0-0-0-0-0	2684	/
SAL_FA0820AA	FDA00009870	Aberdeen	0	0	0	0	0	0	0	0	0	0	0		**Brancaster**	0-0-0-0-0-0	50	/
SAL_FA0806AA	FDA00009898	Aberdeen	0	0	0	0	0	0	0	0	0	0	0		**Brandenburg**	0-0-0-0-0-0	517	0.984761905
SAL_FA0807AA	FDA00009897	Aberdeen	0	0	0	0	0	0	0	0	0	0	0			21-14-36-56-16-0	8	0.015238095
SAL_EA8136AA	FDA00009691	Aberdeen	0	0	0	0	0	0	0	0	0	0	0		**Brazil**	0-0-0-0-0-0	5	/
SAL_EA6517AA	FDA00009692	Aberdeen	0	0	0	0	0	0	0	0	0	0	0		**Brazos**	0-0-0-0-0-0	1	/
SAL_EA5938AA	FDA00001475	Aberdeen	0	0	0	0	0	0	0	0	0	0	0		**Breda**	0-0-0-0-0-0	86	/
SAL_EA5939AA	FDA00001476	Aberdeen	0	0	0	0	0	0	0	0	0	0	0		**Bredeney**	0-0-0-0-0-0	367	/
SAL_EA2476AA	FDA00002679	Aberdeen	0	0	0	0	0	0	0	0	0	0	0		**Breukelen**	0-0-0-0-0-0	1	/
SAL_EA1109AA	M574 / NCTC20262	Aberdeen	0	0	0	0	0	0	0	0	0	0	0		**Brevik**	0-0-0-0-0-0	2	/
SAL_EA0396AA	FCC0055	Aberdeen	0	0	0	0	0	0	0	0	0	0	0		**Brisbane**	0-0-0-0-0-0	1	/
SAL_DA9986AA	FNW19F05	Aberdeen	0	0	0	0	0	0	0	0	0	0	0		**Brunei**	0-0-0-0-0-0	129	/
SAL_DA7521AA	FAR0137	Aberdeen	0	0	0	0	0	0	0	0	0	0	0		**Businga**	5-4-6-8-0-0	1	1
SAL_DA6095AA	FNE0122	Aberdeen	0	0	0	0	0	0	0	0	0	0	0		**Butantan**	0-0-0-0-0-0	4	/
SAL_DA2776AA	FDN0144	Aberdeen	0	0	0	0	0	0	0	0	0	0	0		**Buzu**	0-0-0-0-0-0	6	/
SAL_DA2769AA	FDN0141	Aberdeen	0	0	0	0	0	0	0	0	0	0	0		**Cairina**	0-0-0-0-0-0	1	/
SAL_DA1741AA	FDA00008518	Aberdeen	0	0	0	0	0	0	0	0	0	0	0		**Canada**	0-0-0-0-0-0	9	/
SAL_DA1722AA	FDA00008517	Aberdeen	0	0	0	0	0	0	0	0	0	0	0		**Caracas**	0-0-0-0-0-0	30	/
SAL_DA1715AA	FDA00008516	Aberdeen	0	0	0	0	0	0	0	0	0	0	0		**Cardoner**	0-0-0-0-0-0	1	/
SAL_DA1692AA	FDA00008519	Aberdeen	0	0	0	0	0	0	0	0	0	0	0		**Carmel**	0-0-0-0-0-0	21	0.954545455
SAL_CA8309AA	FDA00001298	Aberdeen	0	0	0	0	0	0	0	0	0	0	0			21-16-36-68-16-0	1	0.045454545
SAL_CA7377AA	FDA00003194	Aberdeen	0	0	0	0	0	0	0	0	0	0	0		**Carrau**	0-0-0-0-0-0	12	/
SAL_CA7325AA	FDA00003193	Aberdeen	0	0	0	0	0	0	0	0	0	0	0		**Cerro**	0-0-0-0-0-0	1120	/
SAL_CA7079AA	FDA00001131	Aberdeen	0	0	0	0	0	0	0	0	0	0	0		**Champaign**	0-0-0-0-0-0	2	/
SAL_CA5841AA	68862	Aberdeen	0	0	0	0	0	0	0	0	0	0	0		**Chandans**	0-0-0-0-0-0	2	/
SAL_CA5079AA	75760	Aberdeen	0	0	0	0	0	0	0	0	0	0	0		**Chester**	0-0-0-0-0-0	589	/
SAL_CA3693AA	41944	Aberdeen	0	0	0	0	0	0	0	0	0	0	0		**Chicago**	0-0-0-0-0-0	12	/
SAL_BA7845AA	4042-sc-2010-09-29-985	Aberdeen	0	0	0	0	0	0	0	0	0	0	0		**Choleraesuis**	0-0-0-0-0-0	400	0.995024876
SAL_BA6349AA	FDA00000871	Aberdeen	0	0	0	0	0	0	0	0	0	0	0			3-0-0-0-0-0	1	0.002487562
SAL_BA5804AA	M74 / NCTC20042	Aberdeen	0	0	0	0	0	0	0	0	0	0	0			18-3-5-0-3-0	1	0.002487562
SAL_BA5792AA	M75 / NCTC20042	Aberdeen	0	0	0	0	0	0	0	0	0	0	0		**Clackamas**	0-0-0-0-0-0	6	/
SAL_BA2672AA	FDA00001227	Aberdeen	0	0	0	0	0	0	0	0	0	0	0		**Cleveland**	0-0-0-0-0-0	3	/
SAL_BA2655AA	FDA00008914	Aberdeen	0	0	0	0	0	0	0	0	0	0	0		**Cochise**	0-0-0-0-0-0	1	/
SAL_BA2635AA	FDA00001357	Aberdeen	0	0	0	0	0	0	0	0	0	0	0		**Coeln**	0-0-0-0-0-0	152	/
SAL_BA2218AA	FDA00000753	Aberdeen	0	0	0	0	0	0	0	0	0	0	0		**Coleypark**	0-0-0-0-0-0	4	/
SAL_BA1539AA	FDA00003390	Aberdeen	0	0	0	0	0	0	0	0	0	0	0		**Colindale**	0-0-0-0-0-0	51	0.980769231
SAL_BA1530AA	FDA00003389	Aberdeen	0	0	0	0	0	0	0	0	0	0	0			92-0-211-425-83-0	1	0.019230769
SAL_BA0963AA	FDA00000401	Aberdeen	0	0	0	0	0	0	0	0	0	0	0		**Coquilhatville**	0-0-0-0-0-0	2	/
SAL_FA6508AA	FDA00010105	Aberdeen	0	0	0	0	0	0	0	0	0	0	0		**Coromandel**	0-0-0-0-0-0	1	/
SAL_FA7845AA	FDA00010104	Aberdeen	0	0	0	0	0	0	0	0	0	0	0		**Corvallis**	0-0-0-0-0-0	334	/
SAL_GA6963AA	FDA00003541	Aberdeen	0	0	0	0	0	0	0	0	0	0	0		**Cotham**	0-0-0-0-0-0	99	/
SAL_GA8766AA	FDA00003494	Aberdeen	0	0	0	0	0	0	0	0	0	0	0		**Cubana**	0-0-0-0-0-0	234	/
SAL_GA8753AA	FDA00003452	Aberdeen	0	0	0	0	0	0	0	0	0	0	0		**Cumberland**	0-0-0-0-0-0	1	/
SAL_GA9245AA	FDA00010590	Aberdeen	0	0	0	0	0	0	0	0	0	0	0		**Curacao**	0-0-0-0-0-0	10	/
SAL_GA9227AA	FDA00010627	Aberdeen	0	0	0	0	0	0	0	0	0	0	0		**Dahra**	0-0-0-0-0-0	1	/
SAL_HA1269AA	PNUSAS004671	Aberdeen	0	0	0	0	0	0	0	0	0	0	0		**Derby**	0-0-0-0-0-0	2154	/
SAL_HA3618AA	56C BS COOKING AREA	Aberdeen	0	0	0	0	0	0	0	0	0	0	0		**Detmold**	0-0-0-0-0-0	1	/
SAL_IA5116AA	FDA00002328	Aberdeen	0	0	0	0	0	0	0	0	0	0	0		**Deversoir**	0-0-0-0-0-0	2	/
SAL_JA4485AA	201610049	Aberdeen	0	0	0	0	0	0	0	0	0	0	0		**Doel**	0-0-0-0-0-0	4	/
SAL_LA2742AA	0809F36481	Aberdeen	0	0	0	0	0	0	0	0	0	0	0		**Drac**	0-0-0-0-0-0	2	/
SAL_LA4240AA	FDA00001470	Aberdeen	0	0	0	0	0	0	0	0	0	0	0			5-4-6-8-0-0	9	0.003133705
SAL_LA7081AA	2012105384	Aberdeen	0	0	0	0	0	0	0	0	0	0	0			5-4-6-27-0-0	9	0.003133705

SAL_BA1356AA	FDA00002008	Aberdeen	0	0	0	0	0	0	0	0	0	0	0			0-0-0-0-0-0	91	0.031685237
SAL_BA1694AA	FDA00001413	Aberdeen	0	0	0	0	0	0	0	0	0	0	0			5-4-6-7-0-0	2718	0.94637883
SAL_FA1630AA	FDA00009946	Aberdeen	0	0	0	0	0	0	0	0	0	0	0			5-4-29-7-0-0	7	0.002437326
SAL_FA8404AA	PNUSAS002192	Aberdeen	0	0	0	0	0	0	0	0	0	0	0			5-4-6-63-0-0	3	0.001044568
SAL_FA0731AA	CFSAN046106	Aberdeen	0	0	0	0	0	0	0	0	0	0	0			5-4-6-126-0-0	5	0.001740947
SAL_EA5940AA	FDA00001474	Aberdeen	0	0	0	0	0	0	0	0	0	0	0			5-0-6-7-0-0	1	0.000348189
SAL_LA7055AA	2007751081	Aberdeen	0	0	0	0	0	0	0	0	0	0	0			0-0-0-7-0-0	2	0.000696379
SAL_KA6898AA	PNUSAS016602	Aberdeen	0	0	0	0	0	0	0	0	0	0	0			5-4-74-7-0-0	1	0.000348189
SAL_MA7096AA	PNUSAS025587	Aberdeen	0	0	0	0	0	0	0	0	0	0	0			5-4-89-7-0-0	4	0.001392758
SAL_MA7037AA	PNUSAS026700	Aberdeen	0	0	0	0	0	0	0	0	0	0	0		**Dublin**	1-1-0-7-1-1	1	0.000348189
SAL_NA0191AA	PNUSAS029319	Aberdeen	0	0	0	0	0	0	0	0	0	0	0			5-4-20-7-0-0	5	0.001740947
SAL_NA2459AA	PNUSAS021395	Aberdeen	0	0	0	0	0	0	0	0	0	0	0			5-48-6-7-0-0	1	0.000348189
SAL_NA4529AA	FDA00012481	Aberdeen	0	0	0	0	0	0	0	0	0	0	0			5-4-0-7-0-0	1	0.000348189
SAL_NA4530AA	FDA00012480	Aberdeen	0	0	0	0	0	0	0	0	0	0	0			5-4-6-231-0-0	1	0.000348189
SAL_NA8052AA	412048	Aberdeen	0	0	0	0	0	0	0	0	0	0	0			5-4-6-334-0-0	3	0.001044568
SAL_OA8092AA	PNUSAS035579	Aberdeen	0	0	0	0	0	0	0	0	0	0	0			5-4-6-335-0-0	2	0.000696379
SAL_OA8084AA	PNUSAS035580	Aberdeen	0	0	0	0	0	0	0	0	0	0	0			78-4-6-7-0-0	1	0.000348189
SAL_OA8035AA	PNUSAS037327	Aberdeen	0	0	0	0	0	0	0	0	0	0	0			0-4-6-7-0-0	4	0.001392758
SAL_OA8252AA	PNUSAS036153	Aberdeen	0	0	0	0	0	0	0	0	0	0	0			0-0-6-7-0-0	1	0.000348189
SAL_PA2253AA	PNUSAS039010	Aberdeen	0	0	0	0	0	0	0	0	0	0	0			89-4-6-7-0-0	2	0.000696379
SAL_PA8534AA	159884	Aberdeen	0	0	0	0	0	0	0	0	0	0	0		**Dugbe**	0-0-0-0-0-0	2	/
SAL_QA2145AA	167027	Aberdeen	0	0	0	0	0	0	0	0	0	0	0		**Duisburg**	0-0-0-0-0-0	20	/
SAL_QA3463AA	159668	Aberdeen	0	0	0	0	0	0	0	0	0	0	0		**Duval**	0-0-0-0-0-0	2	/
SAL_QA5728AA	PNUSAS043661	Aberdeen	0	0	0	0	0	0	0	0	0	0	0		**Ealing**	0-0-0-0-0-0	85	/
SAL_RA0687AA	362105	Aberdeen	0	0	0	0	0	0	0	0	0	0	0		**Eastbourne**	0-0-0-0-0-0	165	/
SAL_RA1454AA	PNUSAS044415	Aberdeen	0	0	0	0	0	0	0	0	0	0	0		**Eko**	0-0-0-0-0-0	29	/
SAL_RA5412AA	267039	Aberdeen	0	0	0	0	0	0	0	0	0	0	0		**Elisabethville**	0-0-0-0-0-0	21	/
SAL_SA2549AA	FDA00013412	Aberdeen	0	0	0	0	0	0	0	0	0	0	0		**Emek**	0-0-0-0-0-0	37	/
SAL_SA3134AA		Aberdeen	0	0	0	0	0	0	0	0	0	0	0			0-3-5-6-3-0	5	0.000138904
SAL_TA6173AA	629327	Aberdeen	0	0	0	0	0	0	0	0	0	0	0			0-3-0-0-3-0	8038	0.223302589
SAL_TA8385AA	633488	Aberdeen	0	0	0	0	0	0	0	0	0	0	0			0-0-0-0-3-0	9404	0.26125125
SAL_PA2602AA	PNUSAS036106	Aberdeen	0	0	0	0	0	0	0	0	0	0	0			0-3-5-12-3-0	8	0.000222247
SAL_GA7198AA	NCTC5791	Aberdeen	0	0	0	0	0	0	0	0	0	0	0			0-0-8-13-3-0	168	0.004667185
SAL_VA7488AA	201704851	Aberdeen	0	0	0	0	0	0	0	0	0	0	0			0-4-8-0-3-0	48	0.001333481
SAL_VA7677AA	201710866	Aberdeen	0	0	0	0	0	0	0	0	0	0	0			0-0-8-14-3-0	76	0.002111346
SAL_VA5880AA	SA20094313	Aberdeen	0	0	0	0	0	0	0	0	0	0	0			6-4-0-17-0-0	5	0.000138904
SAL_VA4458AA	401717	Aberdeen	0	0	0	0	0	0	0	0	0	0	0			0-4-0-0-0-0	2	5.55617E-05
SAL_VA4047AA	CDPH_CSalm376	Aberdeen	0	0	0	0	0	0	0	0	0	0	0			0-4-0-0-12-0	1	2.77809E-05
SAL_WA2386AA	GW_S05299-06	Aberdeen	0	0	0	0	0	0	0	0	0	0	0			1-1-25-39-1-10	1	2.77809E-05
SAL_WA3803AA	FSIS11918992	Aberdeen	0	0	0	0	0	0	0	0	0	0	0			0-0-0-9-3-0	138	0.003833759
SAL_DA1237AA	H124680424	Abony	0	0	0	0	0	0	0	0	0	0	0			0-3-7-0-3-0	2586	0.071841316
SAL_FA5537AA	PNUSAS001884	Abony	0	0	0	0	0	0	0	0	0	0	0			0-0-8-9-3-0	346	0.009612179
SAL_EA8100AA	19655	Abony	0	0	0	0	0	0	0	0	0	0	0			1-1-25-9-1-10	14	0.000388932
SAL_EA7466AA	29956	Abony	0	0	0	0	0	0	0	0	0	0	0			0-0-5-44-3-0	63	0.001750194
SAL_DA8655AA	FNW19H84	Abony	0	0	0	0	0	0	0	0	0	0	0			5-13-30-46-0-0	158	0.004389377
SAL_DA7772AA	FMA0131	Abony	0	0	0	0	0	0	0	0	0	0	0			5-4-5-11-0-0	514	0.014279364
SAL_DA1388AA	H121380303	Abony	0	0	0	0	0	0	0	0	0	0	0			0-0-8-0-3-0	252	0.007000778
SAL_CA6770AA	44720	Abony	0	0	0	0	0	0	0	0	0	0	0			16-3-7-9-0-0	1421	0.039476609
SAL_CA6415AA	49695	Abony	0	0	0	0	0	0	0	0	0	0	0			0-3-5-48-3-0	2	5.55617E-05
SAL_CA6116AA	62536	Abony	0	0	0	0	0	0	0	0	0	0	0			0-0-5-0-3-0	280	0.007778642
SAL_CA5832AA	68880	Abony	0	0	0	0	0	0	0	0	0	0	0			5-4-8-9-0-0	550	0.015279475
SAL_CA4122AA	73191	Abony	0	0	0	0	0	0	0	0	0	0	0			18-3-7-9-0-0	63	0.001750194
SAL_CA3950AA	90495	Abony	0	0	0	0	0	0	0	0	0	0	0			0-0-8-50-3-0	37	0.001027892
SAL_CA3823AA	50861	Abony	0	0	0	0	0	0	0	0	0	0	0			0-3-0-9-3-0	575	0.015973997
SAL_CA2309AA	37071	Abony	0	0	0	0	0	0	0	0	0	0	0			5-4-6-8-0-0	115	0.003194799
SAL_BA8644AA	23084	Abony	0	0	0	0	0	0	0	0	0	0	0			0-3-7-9-3-0	829	0.023030337
SAL_BA7333AA	FDA00002651	Abony	0	0	0	0	0	0	0	0	0	0	0			16-3-33-9-0-0	4	0.000111123
SAL_EA1254AA	)2-sc-2013-01-21T15:41:	Abony	0	0	0	0	0	0	0	0	0	0	0			0-0-8-9-0-0	16	0.000444494
SAL_FA8205AA	FDA00001515	Abony	0	0	0	0	0	0	0	0	0	0	0			24-4-20-67-0-0	25	0.000694522
SAL_GA2071AA	BCW_3069	Abony	0	0	0	0	0	0	0	0	0	0	0			0-0-6-0-3-0	4	0.000111123
SAL_GA6114AA	PNUSAS003053	Abony	0	0	0	0	0	0	0	0	0	0	0			0-0-8-44-3-0	191	0.005306145
SAL_GA6065AA	PNUSAS003020	Abony	0	0	0	0	0	0	0	0	0	0	0			0-3-7-0-0-0	41	0.001139015
SAL_GA8143AA	PNUSAS003450	Abony	0	0	0	0	0	0	0	0	0	0	0			0-3-44-0-3-0	15	0.000416713
SAL_GA8123AA	PNUSAS003429	Abony	0	0	0	0	0	0	0	0	0	0	0			0-3-0-73-3-0	108	0.003000333
SAL_GA8146AA	PNUSAS003091	Abony	0	0	0	0	0	0	0	0	0	0	0			0-3-0-74-3-0	34	0.000944549
SAL_HA6380AA	PNUSAS005889	Abony	0	0	0	0	0	0	0	0	0	0	0			0-3-5-0-3-0	1183	0.032864763
SAL_HA6381AA	PNUSAS005888	Abony	0	0	0	0	0	0	0	0	0	0	0			0-3-5-51-3-0	130	0.003611512
SAL_HA8841AA	NCTC6017	Abony	0	0	0	0	0	0	0	0	0	0	0			0-3-7-72-3-0	6	0.000166685
SAL_IA0827AA	287727	Abony	0	0	0	0	0	0	0	0	0	0	0			0-3-5-9-3-0	25	0.000694522
SAL_IA8244AA	PNUSAS008860	Abony	0	0	0	0	0	0	0	0	0	0	0			5-4-0-0-0-0	27	0.000750083
SAL_JA3277AA	PNUSAS011843	Abony	0	0	0	0	0	0	0	0	0	0	0			5-4-8-9-3-0	12	0.00033337
SAL_JA5868AA	339487	Abony	0	0	0	0	0	0	0	0	0	0	0			0-0-0-0-0-0	27	0.000750083
SAL_JA9474AA	PNUSAS013875	Abony	0	0	0	0	0	0	0	0	0	0	0			5-4-8-44-0-0	65	0.001805756

SAL_KA3520AA	CFSAN065080	Abony	0	0	0	0	0	0	0	0	0	0	0
SAL_LA6947AA	21-25-20170820	Abony	0	0	0	0	0	0	0	0	0	0	0
SAL_LA7403AA	PNUSAS022780	Abony	0	0	0	0	0	0	0	0	0	0	0
SAL_LA9936AA	24-4-20170976	Abony	0	0	0	0	0	0	0	0	0	0	0
SAL_BA1214AA	FDA00000698	Abony	0	0	0	0	0	0	0	0	0	0	0
SAL_CA0474AA	13850	Abony	0	0	0	0	0	0	0	0	0	0	0
SAL_CA2600AA	25351	Abony	0	0	0	0	0	0	0	0	0	0	0
SAL_DA5994AA	FDA00000021	Abony	0	0	0	0	0	0	0	0	0	0	0
SAL_CA1666AA	39462	Abony	0	0	0	0	0	0	0	0	0	0	0
SAL_BA5138AA	14	Abony	0	0	0	0	0	0	0	0	0	0	0
SAL_CA4878AA	FDA00002653	Abony	0	0	0	0	0	0	0	0	0	0	0
SAL_HA9305AA	FDA00010896	Abony	0	0	0	0	0	0	0	0	0	0	0
SAL_FA5535AA	PNUSAS001886	Abony	0	0	0	0	0	0	0	0	0	0	0
SAL_EA7944AA	58580	Abony	0	0	0	0	0	0	0	0	0	0	0
SAL_IA1615AA	140645	Abony	0	0	0	0	0	0	0	0	0	0	0
SAL_JA6539AA	2015K-0327	Abony	0	0	0	0	0	0	0	0	0	0	0
SAL_CA0658AA	13849	Abony	0	0	0	0	0	0	0	0	0	0	0
SAL_MA6627AA	PNUSAS027811	Abony	0	0	0	0	0	0	0	0	0	0	0
SAL_NA0107AA	la_enterica_subs_enteric	Abony	0	0	0	0	0	0	0	0	0	0	0
SAL_NA7644AA	12-121724_S4	Abony	0	0	0	0	0	0	0	0	0	0	0
SAL_OA9916AA	PNUSAS037391	Abony	0	0	0	0	0	0	0	0	0	0	0
SAL_PA3340AA	NC_SA436	Abony	0	0	0	0	0	0	0	0	0	0	0
SAL_PA3338AA	NC_SA281	Abony	0	0	0	0	0	0	0	0	0	0	0
SAL_PA8313AA	NC_SH409	Abony	0	0	0	0	0	0	0	0	0	0	0
SAL_PA8124AA	NC_SH413	Abony	0	0	0	0	0	0	0	0	0	0	0
SAL_PA9140AA	PNUSAS041358	Abony	0	0	0	0	0	0	0	0	0	0	0
SAL_QA0010AA	PNUSAS040163	Abony	0	0	0	0	0	0	0	0	0	0	0
SAL_QA2511AA	122704	Abony	0	0	0	0	0	0	0	0	0	0	0
SAL_QA4976AA	18EP000954	Abony	0	0	0	0	0	0	0	0	0	0	0
SAL_QA3229AA	549549	Abony	0	0	0	0	0	0	0	0	0	0	0
SAL_QA5225AA	294983	Abony	0	0	0	0	0	0	0	0	0	0	0
SAL_QA6263AA	399383	Abony	0	0	0	0	0	0	0	0	0	0	0
SAL_QA6200AA	302891	Abony	0	0	0	0	0	0	0	0	0	0	0
SAL_QA7099AA	PNUSAS042207	Abony	0	0	0	0	0	0	0	0	0	0	0
SAL_QA5822AA	402864	Abony	0	0	0	0	0	0	0	0	0	0	0
SAL_QA7716AA	404318	Abony	0	0	0	0	0	0	0	0	0	0	0
SAL_QA8862AA	311969	Abony	0	0	0	0	0	0	0	0	0	0	0
SAL_RA1045AA	244393	Abony	0	0	0	0	0	0	0	0	0	0	0
SAL_RA2752AA	304608	Abony	0	0	0	0	0	0	0	0	0	0	0
SAL_RA2754AA	155675	Abony	0	0	0	0	0	0	0	0	0	0	0
SAL_RA2844AA	280277	Abony	0	0	0	0	0	0	0	0	0	0	0
SAL_RA3328AA	127537	Abony	0	0	0	0	0	0	0	0	0	0	0
SAL_RA4036AA	542155	Abony	0	0	0	0	0	0	0	0	0	0	0
SAL_RA4020AA	274242	Abony	0	0	0	0	0	0	0	0	0	0	0
SAL_RA4067AA	316210	Abony	0	0	0	0	0	0	0	0	0	0	0
SAL_RA6638AA	127543	Abony	0	0	0	0	0	0	0	0	0	0	0
SAL_RA7850AA	155676	Abony	0	0	0	0	0	0	0	0	0	0	0
SAL_RA8342AA	506860	Abony	0	0	0	0	0	0	0	0	0	0	0
SAL_SA3918AA	SMG-18-1696	Abony	0	0	0	0	0	0	0	0	0	0	0
SAL_SA4847AA	587432	Abony	0	0	0	0	0	0	0	0	0	0	0
SAL_SA5825AA	590856	Abony	0	0	0	0	0	0	0	0	0	0	0
SAL_SA6738AA	127542	Abony	0	0	0	0	0	0	0	0	0	0	0
SAL_SA8815AA	SMG-18-1705	Abony	0	0	0	0	0	0	0	0	0	0	0
SAL_SA7177AA	591847	Abony	0	0	0	0	0	0	0	0	0	0	0
SAL_SA8010AA	301656	Abony	0	0	0	0	0	0	0	0	0	0	0
SAL_SA9539AA	610005	Abony	0	0	0	0	0	0	0	0	0	0	0
SAL_TA0306AA	611066	Abony	0	0	0	0	0	0	0	0	0	0	0
SAL_TA2700AA	617641	Abony	0	0	0	0	0	0	0	0	0	0	0
SAL_TA5221AA	615642	Abony	0	0	0	0	0	0	0	0	0	0	0
SAL_PA3541AA	NC_SA438	Abony	0	0	0	0	0	0	0	0	0	0	0
SAL_PA3346AA	NC_SA330	Abony	0	0	0	0	0	0	0	0	0	0	0
SAL_UA2675AA	SMG-18-2348	Abony	0	0	0	0	0	0	0	0	0	0	0
SAL_UA3021AA	650037	Abony	0	0	0	0	0	0	0	0	0	0	0
SAL_UA4765AA	651048	Abony	0	0	0	0	0	0	0	0	0	0	0
SAL_UA9540AA	617886	Abony	0	0	0	0	0	0	0	0	0	0	0
SAL_VA0008AA	615633	Abony	0	0	0	0	0	0	0	0	0	0	0
SAL_VA0584AA	201803996	Abony	0	0	0	0	0	0	0	0	0	0	0
SAL_VA0635AA	201805993	Abony	0	0	0	0	0	0	0	0	0	0	0
SAL_VA7380AA	201606645	Abony	0	0	0	0	0	0	0	0	0	0	0
SAL_VA3097AA	421589	Abony	0	0	0	0	0	0	0	0	0	0	0
SAL_VA3447AA	401782	Abony	0	0	0	0	0	0	0	0	0	0	0
SAL_VA5927AA	SA20102406	Abony	0	0	0	0	0	0	0	0	0	0	0
SAL_VA5828AA	S4493-08	Abony	0	0	0	0	0	0	0	0	0	0	0

16-3-7-0-0-0	98	0.002722525
0-3-46-0-3-0	24	0.000666741
18-3-0-0-3-0	317	0.008806534
0-3-0-51-3-0	509	0.01414046
0-3-5-49-3-0	9	0.000250028
0-3-0-49-3-0	59	0.001639071
0-3-0-76-3-0	158	0.004389377
0-3-5-76-3-0	59	0.001639071
0-3-7-79-3-0	7	0.000194466
0-0-8-52-3-0	198	0.005500611
18-3-0-76-3-0	6	0.000166685
0-0-8-47-3-0	345	0.009584398
0-0-6-73-3-0	3	8.33426E-05
0-0-8-80-3-0	20	0.000555617
0-0-5-52-3-0	39	0.001083454
0-0-5-80-3-0	13	0.000361151
0-0-8-81-3-0	11	0.00030559
0-3-7-82-3-0	69	0.00191688
18-3-7-0-3-0	191	0.005306145
0-3-0-83-3-0	41	0.001139015
18-3-5-84-3-0	3	8.33426E-05
0-3-0-85-3-0	3	8.33426E-05
0-0-5-47-3-0	80	0.002222469
0-3-7-86-3-0	20	0.000555617
0-0-0-13-3-0	20	0.000555617
0-3-7-88-3-0	19	0.000527836
0-3-44-51-3-0	8	0.000222247
0-0-0-47-3-0	17	0.000472275
0-3-7-89-3-0	40	0.001111235
0-3-7-90-3-0	1	2.77809E-05
0-3-0-6-3-0	14	0.000388932
0-0-0-38-3-0	4	0.000111123
0-0-5-13-3-0	144	0.004000444
0-0-5-52-0-0	2	5.55617E-05
0-3-7-92-3-0	4	0.000111123
0-0-0-73-3-0	5	0.000138904
0-3-48-0-3-0	4	0.000111123
0-3-48-89-3-0	1	2.77809E-05
0-0-49-47-3-0	4	0.000111123
0-3-0-44-3-0	4	0.000111123
0-0-0-80-3-0	6	0.000166685
0-3-0-93-3-0	2	5.55617E-05
0-3-0-75-3-0	515	0.014307145
0-3-0-94-3-0	2	5.55617E-05
0-3-0-95-3-0	18	0.000500056
0-0-50-13-3-0	7	0.000194466
0-3-7-73-3-0	16	0.000444494
0-3-7-96-3-0	1	2.77809E-05
16-3-0-0-0-0	20	0.000555617
0-0-7-9-0-0	2	5.55617E-05
1-1-25-8-1-10	20	0.000555617
0-3-5-97-3-0	52	0.001444605
5-4-51-0-0-0	1	2.77809E-05
18-3-5-0-3-0	322	0.008945438
0-3-0-98-3-0	1	2.77809E-05
0-3-0-12-3-0	22	0.000611179
0-3-7-76-3-0	20	0.000555617
16-3-53-9-3-0	1	2.77809E-05
16-3-53-9-0-0	39	0.001083454
18-3-7-9-3-0	249	0.006917435
18-3-53-9-3-0	1	2.77809E-05
0-3-7-9-0-0	124	0.003444827
16-3-7-9-3-0	14	0.000388932
0-3-5-100-3-0	2	5.55617E-05
16-3-0-9-0-0	17	0.000472275
0-3-7-95-3-0	12	0.00033337
16-3-55-9-0-0	1	2.77809E-05
16-3-56-104-0-0	1	2.77809E-05
16-3-53-105-0-0	1	2.77809E-05
16-3-57-9-0-0	1	2.77809E-05
16-3-58-9-0-0	1	2.77809E-05
27-3-7-9-0-0	1	2.77809E-05
16-3-58-106-0-0	1	2.77809E-05

SAL_VA8758AA	690340	Abony	0	0	0	0	0	0	0	0	0	0	0
SAL_VA9304AA	288781	Abony	0	0	0	0	0	0	0	0	0	0	0
SAL_WA0764AA	183724	Abony	0	0	0	0	0	0	0	0	0	0	0
SAL_WA3264AA	CK_99/00002863-2	Abony	0	0	0	0	0	0	0	0	0	0	0
SAL_WA3155AA	CK_97/00002312	Abony	0	0	0	0	0	0	0	0	0	0	0
SAL_WA2842AA	CK_97/00002355	Abony	0	0	0	0	0	0	0	0	0	0	0
SAL_WA2843AA	CK_97/00002351	Abony	0	0	0	0	0	0	0	0	0	0	0
SAL_BA5810AA	M225 / NCTC20078	Abortusovis	0	0	0	0	0	0	0	0	0	0	0
SAL_KA3752AA	2017_06_20_S41	Abortusovis	0	0	0	0	0	0	15	17	19	24	1
SAL_KA3753AA	2017_06_20_S40	Abortusovis	0	0	0	0	0	0	0	17	19	24	1
SAL_BA4838AA	CFSAN022624	Abortusovis	0	0	0	0	0	0	15	17	19	24	1
SAL_BA5800AA	M226 / NCTC20078	Abortusovis	0	0	0	0	0	0	0	0	0	0	0
SAL_CA0889AA	6450	Adabraka	0	0	0	0	0	0	0	0	0	0	0
SAL_SA4750AA	591905	Adabraka	0	0	0	0	0	0	0	0	0	0	0
SAL_DA1386AA	H123420648	Adelaide	0	0	0	0	0	0	0	0	0	0	0
SAL_BA7386AA	CFSAN006157	Adelaide	0	0	0	0	0	0	0	0	0	0	0
SAL_CA4429AA	38737	Adelaide	0	0	0	0	0	0	0	0	0	0	0
SAL_FA5415AA	FSIS1606194	Adelaide	0	0	0	0	0	0	0	0	0	0	0
SAL_FA4674AA	188349	Adelaide	0	0	0	0	0	0	0	0	0	0	0
SAL_FA4001AA	FSIS1606033	Adelaide	0	0	0	0	0	0	0	0	0	0	0
SAL_FA2819AA	CVM N57494F	Adelaide	0	0	0	0	0	0	0	0	0	0	0
SAL_FA2791AA	CVM N57607F	Adelaide	0	0	0	0	0	0	0	0	0	0	0
SAL_FA1200AA	IEH-NGS-SAL-03551	Adelaide	0	0	0	0	0	0	0	0	0	0	0
SAL_FA0393AA	FSIS1605543	Adelaide	0	0	0	0	0	0	0	0	0	0	0
SAL_EA8652AA	NY-N19886	Adelaide	0	0	0	0	0	0	0	0	0	0	0
SAL_EA8651AA	NY-N19887	Adelaide	0	0	0	0	0	0	0	0	0	0	0
SAL_EA8650AA	NY-N19888	Adelaide	0	0	0	0	0	0	0	0	0	0	0
SAL_EA6471AA	FSIS1504212	Adelaide	0	0	0	0	0	0	0	0	0	0	0
SAL_EA1486AA	FSIS1500654	Adelaide	0	0	0	0	0	0	0	0	0	0	0
SAL_EA1465AA	FSIS1500713	Adelaide	0	0	0	0	0	0	0	0	0	0	0
SAL_EA0296AA	FDN0018	Adelaide	0	0	0	0	0	0	0	0	0	0	0
SAL_DA6827AA	VA-WGS-00255	Adelaide	0	0	0	0	0	0	0	0	0	0	0
SAL_DA2540AA	IEH-NGS-SAL-01433	Adelaide	0	0	0	0	0	0	0	0	0	0	0
SAL_DA1441AA	H130260447	Adelaide	0	0	0	0	0	0	0	0	0	0	0
SAL_DA1416AA	H124960605	Adelaide	0	0	0	0	0	0	0	0	0	0	0
SAL_DA1421AA	H121540985	Adelaide	0	0	0	0	0	0	0	0	0	0	0
SAL_CA9939AA	PNUSAS000065	Adelaide	0	0	0	0	0	0	0	0	0	0	0
SAL_CA9938AA	PNUSAS000066	Adelaide	0	0	0	0	0	0	0	0	0	0	0
SAL_CA9937AA	PNUSAS000067	Adelaide	0	0	0	0	0	0	0	0	0	0	0
SAL_CA9936AA	PNUSAS000068	Adelaide	0	0	0	0	0	0	0	0	0	0	0
SAL_CA9774AA	FNW19i69	Adelaide	0	0	0	0	0	0	0	0	0	0	0
SAL_CA9737AA	2014K-0942	Adelaide	0	0	0	0	0	0	0	0	0	0	0
SAL_CA9736AA	2014K-0943	Adelaide	0	0	0	0	0	0	0	0	0	0	0
SAL_CA9738AA	2014K-0941	Adelaide	0	0	0	0	0	0	0	0	0	0	0
SAL_CA9681AA	FSIS1400366	Adelaide	0	0	0	0	0	0	0	0	0	0	0
SAL_CA9685AA	FSIS1400365	Adelaide	0	0	0	0	0	0	0	0	0	0	0
SAL_CA9674AA	FSIS1400364	Adelaide	0	0	0	0	0	0	0	0	0	0	0
SAL_CA9678AA	FSIS1400367	Adelaide	0	0	0	0	0	0	0	0	0	0	0
SAL_CA9654AA	FSIS1400375	Adelaide	0	0	0	0	0	0	0	0	0	0	0
SAL_CA9395AA	2014K-0973	Adelaide	0	0	0	0	0	0	0	0	0	0	0
SAL_CA9396AA	2014K-0972	Adelaide	0	0	0	0	0	0	0	0	0	0	0
SAL_CA9401AA	2014K-0951	Adelaide	0	0	0	0	0	0	0	0	0	0	0
SAL_CA9394AA	2014K-0974	Adelaide	0	0	0	0	0	0	0	0	0	0	0
SAL_CA6753AA	44671	Adelaide	0	0	0	0	0	0	0	0	0	0	0
SAL_CA5321AA	80298	Adelaide	0	0	0	0	0	0	0	0	0	0	0
SAL_CA5319AA	70654	Adelaide	0	0	0	0	0	0	0	0	0	0	0
SAL_CA3588AA	5918	Adelaide	0	0	0	0	0	0	0	0	0	0	0
SAL_CA2537AA	99746	Adelaide	0	0	0	0	0	0	0	0	0	0	0
SAL_CA2341AA	5371	Adelaide	0	0	0	0	0	0	0	0	0	0	0
SAL_BA8837AA	NY_BAC0500009370	Adelaide	0	0	0	0	0	0	0	0	0	0	0
SAL_BA7597AA	2188960	Adelaide	0	0	0	0	0	0	0	0	0	0	0
SAL_BA1438AA	FDA00000604	Adelaide	0	0	0	0	0	0	0	0	0	0	0
SAL_FA6204AA	FSIS1606335	Adelaide	0	0	0	0	0	0	0	0	0	0	0
SAL_FA7901AA	FSIS1606769	Adelaide	0	0	0	0	0	0	0	0	0	0	0
SAL_FA8054AA	PNUSAS002444	Adelaide	0	0	0	0	0	0	0	0	0	0	0
SAL_FA7293AA	FSIS1606576	Adelaide	0	0	0	0	0	0	0	0	0	0	0
SAL_FA8466AA	FSIS1606835	Adelaide	0	0	0	0	0	0	0	0	0	0	0
SAL_GA2844AA	CRJJGF_00067	Adelaide	0	0	0	0	0	0	0	0	0	0	0
SAL_GA2683AA	06-0804	Adelaide	0	0	0	0	0	0	0	0	0	0	0
SAL_GA2680AA	2009K1429	Adelaide	0	0	0	0	0	0	0	0	0	0	0
SAL_GA2675AA	01-0361	Adelaide	0	0	0	0	0	0	0	0	0	0	0
SAL_GA2670AA	05-0215	Adelaide	0	0	0	0	0	0	0	0	0	0	0
SAL_GA4398AA	PNUSAS002408	Adelaide	0	0	0	0	0	0	0	0	0	0	0

18-3-7-0-0-0	2	5.55617E-05
0-3-5-89-3-0	4	0.000111123
0-0-5-9-3-0	71	0.001972441
16-3-53-0-0-0	4	0.000111123
0-0-0-9-0-0	18	0.000500056
0-0-0-109-3-0	24	0.000666741
5-23-6-9-0-0	2	5.55617E-05
0-3-48-111-3-0	1	2.77809E-05
0-0-5-50-3-0	6	0.000166685
0-3-7-74-3-0	5	0.000138904
0-3-0-112-3-0	13	0.000361151
0-3-0-113-3-0	1	2.77809E-05
0-3-0-114-3-0	11	0.00030559
0-3-5-115-3-0	2	5.55617E-05
0-3-46-111-3-0	1	2.77809E-05
0-3-0-116-3-0	1	2.77809E-05
0-0-5-81-3-0	3	8.33426E-05
0-3-33-117-3-0	1	2.77809E-05
0-3-46-118-3-0	1	2.77809E-05
0-3-5-73-3-0	22	0.000611179
0-3-0-72-3-0	22	0.000611179
0-3-62-0-3-0	2	5.55617E-05
0-4-8-111-3-0	1	2.77809E-05
0-3-5-111-3-0	1	2.77809E-05
0-0-0-119-3-0	13	0.000361151
0-3-0-48-3-0	6	0.000166685
0-3-0-120-3-0	1	2.77809E-05
0-3-62-12-3-0	1	2.77809E-05
0-3-63-121-3-0	1	2.77809E-05
0-3-5-10-3-0	3	8.33426E-05
0-3-0-122-3-0	1	2.77809E-05
0-3-0-92-3-0	12	0.00033337
0-0-8-123-3-0	57	0.001583509
0-3-5-124-3-0	5	0.000138904
18-3-7-121-3-0	1	2.77809E-05
1-0-25-0-1-10	1	2.77809E-05
0-3-7-51-3-0	34	0.000944549
0-3-0-43-3-0	23	0.00063896
0-3-44-73-3-0	3	8.33426E-05
0-3-5-72-3-0	2	5.55617E-05
0-3-7-111-3-0	7	0.000194466
0-3-0-0-21-0	65	0.001805756
0-3-0-125-3-0	1	2.77809E-05
16-3-5-75-0-0	4	0.000111123
0-3-53-0-0-0	2	5.55617E-05
5-4-29-45-0-0	43	0.001194577
0-3-53-0-3-0	19	0.000527836
0-3-0-9-0-0	6	0.000166685
18-3-0-51-3-0	20	0.000555617
16-3-5-76-0-0	3	8.33426E-05
18-3-5-9-0-0	2	5.55617E-05
18-3-53-0-3-0	4	0.000111123
0-3-53-9-3-0	21	0.000583398
0-25-0-9-3-0	1	2.77809E-05
0-3-0-0-0-0	31	0.000861207
0-3-67-0-3-0	200	0.005556173
0-3-5-127-3-0	4	0.000111123
5-4-50-9-0-0	37	0.001027892
16-3-46-75-0-0	1	2.77809E-05
16-3-7-128-0-0	2	5.55617E-05
16-3-5-0-0-0	16	0.000444494
0-0-0-75-3-0	4	0.000111123
0-4-0-0-15-0	16	0.000444494
0-3-5-0-0-0	5	0.000138904
6-4-0-0-15-0	10	0.000277809
0-0-29-9-0-0	1	2.77809E-05
5-4-6-131-0-0	151	0.004194911
16-26-5-75-0-0	3	8.33426E-05
0-3-0-0-23-0	2	5.55617E-05
28-0-0-0-0-0	2	5.55617E-05
5-0-0-0-0-0	6	0.000166685
18-27-0-51-0-0	1	2.77809E-05
16-0-0-9-0-0	1	2.77809E-05

SAL_GA4912AA	FSIS1607127	Adelaide	0	0	0	0	0	0	0	0	0	0	0	16-3-7-75-0-0	16	0.000444494
SAL_GA4744AA	PNUSAS002329	Adelaide	0	0	0	0	0	0	0	0	0	0	0	16-3-70-9-0-0	1	2.77809E-05
SAL_GA5711AA	CVM N58637	Adelaide	0	0	0	0	0	0	0	0	0	0	0	16-3-7-133-0-0	1	2.77809E-05
SAL_FA8422AA	PNUSAS002430	Adelaide	0	0	0	0	0	0	0	0	0	0	0	0-3-71-0-3-0	4	0.000111123
SAL_GA7761AA	FSIS1607344	Adelaide	0	0	0	0	0	0	0	0	0	0	0	0-3-7-134-3-0	1	2.77809E-05
SAL_GA8055AA	FSIS1607439	Adelaide	0	0	0	0	0	0	0	0	0	0	0	16-25-7-0-0-0	1	2.77809E-05
SAL_GA8663AA	PNUSAS003556	Adelaide	0	0	0	0	0	0	0	0	0	0	0	0-3-0-0-25-0	1	2.77809E-05
SAL_GA9057AA	FSIS1607565	Adelaide	0	0	0	0	0	0	0	0	0	0	0	0-3-0-9-21-0	5	0.000138904
SAL_GA9362AA	ADRDL_R194	Adelaide	0	0	0	0	0	0	0	0	0	0	0	0-0-48-44-3-0	6	0.000166685
SAL_GA9380AA	CDPH_C75	Adelaide	0	0	0	0	0	0	0	0	0	0	0	0-3-7-0-21-0	2	5.55617E-05
SAL_HA0856AA	FDA00004101	Adelaide	0	0	0	0	0	0	0	0	0	0	0	5-4-6-9-0-0	17	0.000472275
SAL_HA4646AA	FSIS1608382	Adelaide	0	0	0	0	0	0	0	0	0	0	0	5-4-24-0-3-0	161	0.004472719
SAL_HA4739AA	FSIS1608550	Adelaide	0	0	0	0	0	0	0	0	0	0	0	5-4-24-109-3-0	8	0.000222247
SAL_HA4746AA	FSIS1608389	Adelaide	0	0	0	0	0	0	0	0	0	0	0	5-4-26-40-0-0	4	0.000111123
SAL_HA6131AA	FSIS1608765	Adelaide	0	0	0	0	0	0	0	0	0	0	0	0-30-8-0-3-0	1	2.77809E-05
SAL_HA8820AA	FSIS1609542	Adelaide	0	0	0	0	0	0	0	0	0	0	0	0-4-31-0-14-11	4	0.000111123
SAL_HA8983AA	PNUSAS006558	Adelaide	0	0	0	0	0	0	0	0	0	0	0	0-0-73-109-3-0	1	2.77809E-05
SAL_HA9174AA	PNUSAS006761	Adelaide	0	0	0	0	0	0	0	0	0	0	0	5-4-24-38-0-0	39	0.001083454
SAL_HA9297AA	ADRDL-779	Adelaide	0	0	0	0	0	0	0	0	0	0	0	0-3-0-47-3-0	1	2.77809E-05
SAL_IA0143AA	ADRDL-814	Adelaide	0	0	0	0	0	0	0	0	0	0	0	5-4-24-38-3-0	76	0.002111346
SAL_IA2349AA	FSIS1609758	Adelaide	0	0	0	0	0	0	0	0	0	0	0	0-3-7-138-3-0	11	0.00030559
SAL_IA2612AA	PNUSAS007755	Adelaide	0	0	0	0	0	0	0	0	0	0	0	0-0-0-8-3-0	37	0.001027892
SAL_IA2614AA	PNUSAS007757	Adelaide	0	0	0	0	0	0	0	0	0	0	0	5-4-75-9-0-0	3	8.33426E-05
SAL_IA4671AA	FSIS1710081	Adelaide	0	0	0	0	0	0	0	0	0	0	0	0-0-75-9-3-0	1	2.77809E-05
SAL_IA3704AA	FSIS1710130	Adelaide	0	0	0	0	0	0	0	0	0	0	0	5-4-6-119-0-0	1	2.77809E-05
SAL_IA4890AA	FSIS1710222	Adelaide	0	0	0	0	0	0	0	0	0	0	0	0-0-76-8-3-0	2	5.55617E-05
SAL_IA5177AA	CFSAN057968	Adelaide	0	0	0	0	0	0	0	0	0	0	0	5-31-76-8-0-0	1	2.77809E-05
SAL_IA5358AA	FSIS1710307	Adelaide	0	0	0	0	0	0	0	0	0	0	0	16-3-5-83-0-0	4	0.000111123
SAL_IA6009AA	CFSAN059756	Adelaide	0	0	0	0	0	0	0	0	0	0	0	0-0-6-9-3-0	4	0.000111123
SAL_IA5444AA	PNUSAS007936	Adelaide	0	0	0	0	0	0	0	0	0	0	0	0-3-5-83-3-0	13	0.000361151
SAL_IA6676AA	FSIS1710412	Adelaide	0	0	0	0	0	0	0	0	0	0	0	16-3-67-0-0-0	3	8.33426E-05
SAL_IA7596AA	PNUSAS009118	Adelaide	0	0	0	0	0	0	0	0	0	0	0	0-3-32-0-3-0	8	0.000222247
SAL_IA7704AA	S/20170075	Adelaide	0	0	0	0	0	0	0	0	0	0	0	16-3-0-0-3-0	5	0.000138904
SAL_IA8872AA	PNUSAS008671	Adelaide	0	0	0	0	0	0	0	0	0	0	0	0-3-67-9-3-0	8	0.000222247
SAL_IA9059AA	FSIS1710431	Adelaide	0	0	0	0	0	0	0	0	0	0	0	16-3-7-140-0-0	5	0.000138904
SAL_IA9616AA	PNUSAS009924	Adelaide	0	0	0	0	0	0	0	0	0	0	0	0-0-77-13-3-0	1	2.77809E-05
SAL_IA9737AA	FSIS1710717	Adelaide	0	0	0	0	0	0	0	0	0	0	0	0-0-78-9-0-0	3	8.33426E-05
SAL_JA0338AA	PNUSAS010322	Adelaide	0	0	0	0	0	0	0	0	0	0	0	0-0-5-109-3-0	6	0.000166685
SAL_JA1493AA	PNUSAS010500	Adelaide	0	0	0	0	0	0	0	0	0	0	0	16-33-7-9-0-0	1	2.77809E-05
SAL_JA0363AA	PNUSAS009765	Adelaide	0	0	0	0	0	0	0	0	0	0	0	5-4-79-131-0-0	218	0.006056228
SAL_JA0349AA	PNUSAS010199	Adelaide	0	0	0	0	0	0	0	0	0	0	0	18-3-53-9-0-0	1	2.77809E-05
SAL_JA1761AA	PNUSAS011293	Adelaide	0	0	0	0	0	0	0	0	0	0	0	0-3-7-9-21-0	9	0.000250028
SAL_JA2155AA	FSIS1700017	Adelaide	0	0	0	0	0	0	0	0	0	0	0	0-3-5-79-3-0	2	5.55617E-05
SAL_JA2053AA	FSIS1700191	Adelaide	0	0	0	0	0	0	0	0	0	0	0	0-0-78-9-3-0	3	8.33426E-05
SAL_JA2314AA	PNUSAS011809	Adelaide	0	0	0	0	0	0	0	0	0	0	0	0-3-0-124-3-0	22	0.000611179
SAL_JA3372AA	PNUSAS010579	Adelaide	0	0	0	0	0	0	0	0	0	0	0	0-3-8-52-3-0	1	2.77809E-05
SAL_JA5005AA	PNUSAS012608	Adelaide	0	0	0	0	0	0	0	0	0	0	0	0-3-0-89-3-0	26	0.000722302
SAL_JA6626AA	FSIS1701204	Adelaide	0	0	0	0	0	0	0	0	0	0	0	18-3-46-0-3-0	2	5.55617E-05
SAL_JA7481AA	FSIS1700785	Adelaide	0	0	0	0	0	0	0	0	0	0	0	0-3-0-143-3-0	2	5.55617E-05
SAL_JA7988AA	366314	Adelaide	0	0	0	0	0	0	0	0	0	0	0	0-0-0-0-29-0	2	5.55617E-05
SAL_JA8587AA	FSIS1701266	Adelaide	0	0	0	0	0	0	0	0	0	0	0	0-0-0-144-3-0	2	5.55617E-05
SAL_JA9475AA	PNUSAS014013	Adelaide	0	0	0	0	0	0	0	0	0	0	0	0-3-7-124-3-0	9	0.000250028
SAL_JA8989AA	PNUSAS013845	Adelaide	0	0	0	0	0	0	0	0	0	0	0	0-3-5-146-3-0	4	0.000111123
SAL_JA9288AA	PNUSAS015182	Adelaide	0	0	0	0	0	0	0	0	0	0	0	0-3-7-44-0-0	2	5.55617E-05
SAL_JA9834AA	FSIS1701909	Adelaide	0	0	0	0	0	0	0	0	0	0	0	0-0-5-75-0-0	4	0.000111123
SAL_JA9946AA	PNUSAS015325	Adelaide	0	0	0	0	0	0	0	0	0	0	0	0-3-33-0-3-0	8	0.000222247
SAL_JA8867AA	FSIS1701480	Adelaide	0	0	0	0	0	0	0	0	0	0	0	0-4-0-0-3-0	9	0.000250028
SAL_JA9933AA	PNUSAS015349	Adelaide	0	0	0	0	0	0	0	0	0	0	0	18-3-5-76-3-0	15	0.000416713
SAL_KA0697AA	2015AM-1238	Adelaide	0	0	0	0	0	0	0	0	0	0	0	0-3-0-0-30-0	2	5.55617E-05
SAL_KA0314AA	PNUSAS016112	Adelaide	0	0	0	0	0	0	0	0	0	0	0	16-3-7-134-0-0	1	2.77809E-05
SAL_KA0712AA	2015AM-0453	Adelaide	0	0	0	0	0	0	0	0	0	0	0	33-3-7-9-3-0	1	2.77809E-05
SAL_KA1316AA	PNUSAS016411	Adelaide	0	0	0	0	0	0	0	0	0	0	0	0-3-83-0-3-0	5	0.000138904
SAL_KA0995AA	FSIS1702170	Adelaide	0	0	0	0	0	0	0	0	0	0	0	0-0-5-150-3-0	1	2.77809E-05
SAL_KA1014AA	FSIS1702248	Adelaide	0	0	0	0	0	0	0	0	0	0	0	0-35-48-9-3-0	1	2.77809E-05
SAL_KA2204AA	PNUSAS017024	Adelaide	0	0	0	0	0	0	0	0	0	0	0	0-3-7-151-3-0	6	0.000166685
SAL_KA2893AA	FSIS1701891	Adelaide	0	0	0	0	0	0	0	0	0	0	0	0-0-8-152-3-0	7	0.000194466
SAL_KA3694AA	FSIS1701408	Adelaide	0	0	0	0	0	0	0	0	0	0	0	0-3-63-0-3-0	5	0.000138904
SAL_KA5311AA	PNUSAS016837	Adelaide	0	0	0	0	0	0	0	0	0	0	0	0-0-84-9-3-0	2	5.55617E-05
SAL_KA5301AA	PNUSAS017347	Adelaide	0	0	0	0	0	0	0	0	0	0	0	0-3-0-10-3-0	3	8.33426E-05
SAL_KA5284AA	PNUSAS015254	Adelaide	0	0	0	0	0	0	0	0	0	0	0	0-3-33-153-3-0	1	2.77809E-05
SAL_KA5957AA	PNUSAS018528	Adelaide	0	0	0	0	0	0	0	0	0	0	0	0-0-85-0-3-0	4	0.000111123
SAL_KA5978AA	FSIS1702766	Adelaide	0	0	0	0	0	0	0	0	0	0	0	0-3-5-74-3-0	2	5.55617E-05
SAL_KA6318AA	FSIS1703206	Adelaide	0	0	0	0	0	0	0	0	0	0	0	0-0-0-114-3-0	2	5.55617E-05

SAL_KA6202AA	FSIS1703015	Adelaide	0	0	0	0	0	0	0	0	0	0	0		0-0-0-138-3-0	4	0.000111123
SAL_KA6934AA	PNUSAS019138	Adelaide	0	0	0	0	0	0	0	0	0	0	0		0-3-0-154-3-0	2	5.55617E-05
SAL_KA6676AA	FSIS1703272	Adelaide	0	0	0	0	0	0	0	0	0	0	0		0-3-86-0-3-0	8	0.000222247
SAL_KA8061AA	PNUSAS019527	Adelaide	0	0	0	0	0	0	0	0	0	0	0		0-3-7-155-3-0	1	2.77809E-05
SAL_KA7683AA	FSIS1703468	Adelaide	0	0	0	0	0	0	0	0	0	0	0		5-4-8-14-3-0	5	0.000138904
SAL_KA7917AA	PNUSAS020433	Adelaide	0	0	0	0	0	0	0	0	0	0	0		34-4-8-0-3-0	5	0.000138904
SAL_KA8322AA	FSIS1703375	Adelaide	0	0	0	0	0	0	0	0	0	0	0		0-0-5-119-3-0	2	5.55617E-05
SAL_KA9161AA	ADRDL-912	Adelaide	0	0	0	0	0	0	0	0	0	0	0		0-3-46-95-3-0	1	2.77809E-05
SAL_LA0127AA	19-16-20170640	Adelaide	0	0	0	0	0	0	0	0	0	0	0		0-3-5-118-3-0	5	0.000138904
SAL_KA9517AA	PNUSAS020711	Adelaide	0	0	0	0	0	0	0	0	0	0	0		5-4-87-80-0-0	5	0.000138904
SAL_KA9677AA	PNUSAS020677	Adelaide	0	0	0	0	0	0	0	0	0	0	0		5-37-5-9-0-0	3	8.33426E-05
SAL_LA0430AA	PNUSAS020966	Adelaide	0	0	0	0	0	0	0	0	0	0	0		0-3-7-49-3-0	12	0.00033337
SAL_LA1067AA	PNUSAS021188	Adelaide	0	0	0	0	0	0	0	0	0	0	0		0-0-5-156-0-0	1	2.77809E-05
SAL_LA1843AA	FSIS11703954	Adelaide	0	0	0	0	0	0	0	0	0	0	0		0-3-33-43-3-0	1	2.77809E-05
SAL_LA1840AA	FSIS11704040	Adelaide	0	0	0	0	0	0	0	0	0	0	0		0-0-49-13-3-0	4	0.000111123
SAL_LA4547AA	PNUSAS022548	Adelaide	0	0	0	0	0	0	0	0	0	0	0		0-3-33-89-3-0	1	2.77809E-05
SAL_LA4822AA	PNUSAS023049	Adelaide	0	0	0	0	0	0	0	0	0	0	0		0-3-5-95-3-0	8	0.000222247
SAL_LA6847AA	CFSAN069014	Adelaide	0	0	0	0	0	0	0	0	0	0	0		0-3-33-76-3-0	2	5.55617E-05
SAL_LA7459AA	PNUSAS022914	Adelaide	0	0	0	0	0	0	0	0	0	0	0		0-0-5-47-0-0	8	0.000222247
SAL_LA7329AA	PNUSAS022785	Adelaide	0	0	0	0	0	0	0	0	0	0	0		0-3-7-157-3-0	1	2.77809E-05
SAL_LA9549AA	FSIS11704623	Adelaide	0	0	0	0	0	0	0	0	0	0	0		18-0-0-0-3-0	28	0.000777864
SAL_LA9314AA	PNUSAS021245	Adelaide	0	0	0	0	0	0	0	0	0	0	0		0-3-5-119-3-0	2	5.55617E-05
SAL_LA9348AA	FSIS21720287	Adelaide	0	0	0	0	0	0	0	0	0	0	0		0-0-5-144-3-0	1	2.77809E-05
SAL_MA0213AA	PNUSAS024313	Adelaide	0	0	0	0	0	0	0	0	0	0	0		0-0-84-0-3-0	1	2.77809E-05
SAL_MA3856AA	PNUSAS026140	Adelaide	0	0	0	0	0	0	0	0	0	0	0		0-3-46-159-3-0	1	2.77809E-05
SAL_MA5051AA	FSIS11705367	Adelaide	0	0	0	0	0	0	0	0	0	0	0		0-3-7-160-3-0	2	5.55617E-05
SAL_MA5027AA	FSIS1702650	Adelaide	0	0	0	0	0	0	0	0	0	0	0		0-0-5-73-3-0	1	2.77809E-05
SAL_CA5779AA	78745	Adelaide	0	0	0	0	0	0	0	0	0	0	0		0-0-5-75-3-0	25	0.000694522
SAL_DA1387AA	H124700525	Adelaide	0	0	0	0	0	0	0	0	0	0	0		0-0-84-109-3-0	2	5.55617E-05
SAL_DA0972AA	H123060358	Adelaide	0	0	0	0	0	0	0	0	0	0	0		0-3-7-6-3-0	8	0.000222247
SAL_BA9829AA	:-2012-07-27T11:53:39Z-	Adelaide	0	0	0	0	0	0	0	0	0	0	0		0-0-5-161-3-0	1	2.77809E-05
SAL_FA5002AA	PNUSAS001801	Adelaide	0	0	0	0	0	0	0	0	0	0	0		0-0-43-0-3-0	6	0.000166685
SAL_HA0779AA	PNUSAS004551	Adelaide	0	0	0	0	0	0	0	0	0	0	0		0-3-88-51-3-0	1	2.77809E-05
SAL_HA9328AA	PNUSAS006881	Adelaide	0	0	0	0	0	0	0	0	0	0	0		0-3-5-163-3-0	2	5.55617E-05
SAL_HA6275AA	PNUSAS005583	Adelaide	0	0	0	0	0	0	0	0	0	0	0		0-0-0-164-3-0	1	2.77809E-05
SAL_JA5658AA	PNUSAS012289	Adelaide	0	0	0	0	0	0	0	0	0	0	0		0-3-0-165-3-0	5	0.000138904
SAL_CA2530AA	75924	Adelaide	0	0	0	0	0	0	0	0	0	0	0		0-0-0-50-3-0	1	2.77809E-05
SAL_LA4854AA	PNUSAS018425	Adelaide	0	0	0	0	0	0	0	0	0	0	0		0-38-5-47-3-0	1	2.77809E-05
SAL_MA2614AA	PNUSAS020535	Adelaide	0	0	0	0	0	0	0	0	0	0	0		0-0-8-167-3-0	3	8.33426E-05
SAL_MA6401AA	PNUSAS027753	Adelaide	0	0	0	0	0	0	0	0	0	0	0		0-3-0-168-3-0	1	2.77809E-05
SAL_MA7173AA	PNUSAS025581	Adelaide	0	0	0	0	0	0	0	0	0	0	0		0-3-0-79-3-0	13	0.000361151
SAL_MA7985AA	FSIS21720648	Adelaide	0	0	0	0	0	0	0	0	0	0	0		0-3-7-170-3-0	1	2.77809E-05
SAL_MA8311AA	PNUSAS027353	Adelaide	0	0	0	0	0	0	0	0	0	0	0		0-0-0-0-15-0	147	0.004083787
SAL_MA9050AA	FSIS11705343	Adelaide	0	0	0	0	0	0	0	0	0	0	0		0-0-90-109-3-0	2	5.55617E-05
SAL_MA9788AA	CVM N16S345	Adelaide	0	0	0	0	0	0	0	0	0	0	0		0-3-7-171-3-0	1	2.77809E-05
SAL_NA0810AA	FSIS11705045	Adelaide	0	0	0	0	0	0	0	0	0	0	0		18-3-85-0-3-0	1	2.77809E-05
SAL_NA0570AA	FSIS11706062	Adelaide	0	0	0	0	0	0	0	0	0	0	0		0-3-0-164-3-0	1	2.77809E-05
SAL_NA1329AA	PNUSAS027952	Adelaide	0	0	0	0	0	0	0	0	0	0	0		0-3-46-163-3-0	1	2.77809E-05
SAL_NA1998AA	FSIS11706273	Adelaide	0	0	0	0	0	0	0	0	0	0	0		0-3-46-75-3-0	1	2.77809E-05
SAL_NA2413AA	PNUSAS030262	Adelaide	0	0	0	0	0	0	0	0	0	0	0		5-4-8-75-0-0	25	0.000694522
SAL_NA2223AA	1536_13	Adelaide	0	0	0	0	0	0	0	0	0	0	0		0-3-44-79-3-0	2	5.55617E-05
SAL_NA2204AA	1111_13	Adelaide	0	0	0	0	0	0	0	0	0	0	0		16-3-7-172-0-0	2	5.55617E-05
SAL_NA3903AA	2015AM-2224	Adelaide	0	0	0	0	0	0	0	0	0	0	0		0-3-7-43-3-0	2	5.55617E-05
SAL_NA4627AA	FSIS11706981	Adelaide	0	0	0	0	0	0	0	0	0	0	0		0-3-7-173-3-0	3	8.33426E-05
SAL_NA4810AA	PNUSAS031514	Adelaide	0	0	0	0	0	0	0	0	0	0	0		0-0-85-123-3-0	1	2.77809E-05
SAL_NA4941AA	PNUSAS031652	Adelaide	0	0	0	0	0	0	0	0	0	0	0		0-3-91-95-3-0	1	2.77809E-05
SAL_NA5403AA	PNUSAS030151	Adelaide	0	0	0	0	0	0	0	0	0	0	0		18-3-0-73-3-0	4	0.000111123
SAL_NA6165AA	2015AM-2666	Adelaide	0	0	0	0	0	0	0	0	0	0	0		0-3-0-174-3-0	2	5.55617E-05
SAL_NA5883AA	PNUSAS032054	Adelaide	0	0	0	0	0	0	0	0	0	0	0		0-3-0-80-3-0	1	2.77809E-05
SAL_NA4558AA	FSIS21820903	Adelaide	0	0	0	0	0	0	0	0	0	0	0		0-3-0-175-3-0	1	2.77809E-05
SAL_NA6384AA	FSIS11706959	Adelaide	0	0	0	0	0	0	0	0	0	0	0		0-0-92-0-3-0	1	2.77809E-05
SAL_NA7550AA	CFSAN074822	Adelaide	0	0	0	0	0	0	0	0	0	0	0		0-3-7-143-3-0	1	2.77809E-05
SAL_NA8093AA	2015AM-3605	Adelaide	0	0	0	0	0	0	0	0	0	0	0		1-0-8-176-31-0	1	2.77809E-05
SAL_NA9326AA	PNUSAS026379	Adelaide	0	0	0	0	0	0	0	0	0	0	0		0-0-5-177-3-0	1	2.77809E-05
SAL_OA0148AA	PNUSAS032906	Adelaide	0	0	0	0	0	0	0	0	0	0	0		0-0-0-52-3-0	3	8.33426E-05
SAL_NA9764AA	PNUSAS033733	Adelaide	0	0	0	0	0	0	0	0	0	0	0		0-3-0-138-3-0	9	0.000250028
SAL_OA0995AA	CFSAN075471	Adelaide	0	0	0	0	0	0	0	0	0	0	0		0-0-8-178-3-0	2	5.55617E-05
SAL_OA0999AA	CFSAN075472	Adelaide	0	0	0	0	0	0	0	0	0	0	0		0-3-7-83-3-0	4	0.000111123
SAL_OA7772AA	PNUSAS037244	Adelaide	0	0	0	0	0	0	0	0	0	0	0		0-3-7-179-3-0	1	2.77809E-05
SAL_OA4680AA	PNUSAS025301	Adelaide	0	0	0	0	0	0	0	0	0	0	0		0-3-0-180-3-0	1	2.77809E-05
SAL_OA4658AA	PNUSAS025496	Adelaide	0	0	0	0	0	0	0	0	0	0	0		0-3-7-112-3-0	5	0.000138904
SAL_OA4262AA	PNUSAS035589	Adelaide	0	0	0	0	0	0	0	0	0	0	0		0-3-5-143-3-0	1	2.77809E-05
SAL_OA4252AA	PNUSAS030103	Adelaide	0	0	0	0	0	0	0	0	0	0	0		0-0-0-181-3-0	1	2.77809E-05

SAL_OA4255AA	PNUSAS029293	Adelaide	0	0	0	0	0	0	0	0	0	0	0	0-0-50-80-3-0	2	5.55617E-05
SAL_OA9588AA	PNUSAS037428	Adelaide	0	0	0	0	0	0	0	0	0	0	0	5-4-8-182-0-0	1	2.77809E-05
SAL_PA1407AA	PNUSAS036680	Adelaide	0	0	0	0	0	0	0	0	0	0	0	0-0-8-75-3-0	43	0.001194577
SAL_PA1392AA	PNUSAS036682	Adelaide	0	0	0	0	0	0	0	0	0	0	0	0-0-8-183-3-0	2	5.55617E-05
SAL_PA1303AA	PNUSAS038287	Adelaide	0	0	0	0	0	0	0	0	0	0	0	35-4-8-9-0-0	6	0.000166685
SAL_PA1298AA	PNUSAS038286	Adelaide	0	0	0	0	0	0	0	0	0	0	0	0-3-5-159-3-0	2	5.55617E-05
SAL_PA1973AA	)-e94c-11e6-874d-3c4a9	Adelaide	0	0	0	0	0	0	0	0	0	0	0	0-3-62-95-3-0	1	2.77809E-05
SAL_PA2546AA	FSIS11808494	Adelaide	0	0	0	0	0	0	0	0	0	0	0	0-0-93-9-3-0	1	2.77809E-05
SAL_PA2919AA	PNUSAS038985	Adelaide	0	0	0	0	0	0	0	0	0	0	0	0-0-5-14-0-0	1	2.77809E-05
SAL_PA4064AA	FSIS21821523	Adelaide	0	0	0	0	0	0	0	0	0	0	0	0-0-8-185-3-0	8	0.000222247
SAL_PA4756AA	FSIS21821552	Adelaide	0	0	0	0	0	0	0	0	0	0	0	0-3-33-51-3-0	2	5.55617E-05
SAL_PA5562AA	FSIS11809562	Adelaide	0	0	0	0	0	0	0	0	0	0	0	0-3-5-98-3-0	1	2.77809E-05
SAL_PA6034AA	PNUSAS040087	Adelaide	0	0	0	0	0	0	0	0	0	0	0	0-3-48-83-3-0	1	2.77809E-05
SAL_PA5873AA	PNUSAS040075	Adelaide	0	0	0	0	0	0	0	0	0	0	0	0-3-5-75-3-0	7	0.000194466
SAL_PA6388AA	106522	Adelaide	0	0	0	0	0	0	0	0	0	0	0	0-3-7-186-3-0	2	5.55617E-05
SAL_PA6558AA	211725	Adelaide	0	0	0	0	0	0	0	0	0	0	0	0-3-63-95-3-0	2	5.55617E-05
SAL_PA7442AA	PNUSAS038986	Adelaide	0	0	0	0	0	0	0	0	0	0	0	0-0-8-176-3-0	6	0.000166685
SAL_PA7812AA	18506	Adelaide	0	0	0	0	0	0	0	0	0	0	0	0-3-5-174-3-0	2	5.55617E-05
SAL_PA7635AA	PNUSAS035594	Adelaide	0	0	0	0	0	0	0	0	0	0	0	0-0-5-176-0-0	1	2.77809E-05
SAL_PA8172AA	PNUSAS040088	Adelaide	0	0	0	0	0	0	0	0	0	0	0	0-3-0-189-3-0	2	5.55617E-05
SAL_PA8094AA	FSIS21821663	Adelaide	0	0	0	0	0	0	0	0	0	0	0	0-3-7-167-3-0	1	2.77809E-05
SAL_PA9929AA	21-15-18-8100448-H	Adelaide	0	0	0	0	0	0	0	0	0	0	0	0-3-0-177-3-0	6	0.000166685
SAL_PA9130AA	PNUSAS040951	Adelaide	0	0	0	0	0	0	0	0	0	0	0	0-0-50-0-3-0	4	0.000111123
SAL_PA9680AA	FSIS11810051	Adelaide	0	0	0	0	0	0	0	0	0	0	0	0-3-0-190-3-0	1	2.77809E-05
SAL_QA0186AA	PNUSAS041898	Adelaide	0	0	0	0	0	0	0	0	0	0	0	0-3-0-191-3-0	1	2.77809E-05
SAL_QA0178AA	PNUSAS041900	Adelaide	0	0	0	0	0	0	0	0	0	0	0	0-3-0-192-3-0	33	0.000916769
SAL_QA0132AA	PNUSAS041903	Adelaide	0	0	0	0	0	0	0	0	0	0	0	16-3-7-193-0-0	1	2.77809E-05
SAL_QA0130AA	PNUSAS041905	Adelaide	0	0	0	0	0	0	0	0	0	0	0	5-4-49-75-0-0	3	8.33426E-05
SAL_QA0133AA	PNUSAS041915	Adelaide	0	0	0	0	0	0	0	0	0	0	0	0-3-8-47-3-0	1	2.77809E-05
SAL_QA0134AA	PNUSAS041914	Adelaide	0	0	0	0	0	0	0	0	0	0	0	0-3-44-9-3-0	2	5.55617E-05
SAL_QA0135AA	PNUSAS041906	Adelaide	0	0	0	0	0	0	0	0	0	0	0	0-3-7-192-3-0	2	5.55617E-05
SAL_QA0118AA	PNUSAS041912	Adelaide	0	0	0	0	0	0	0	0	0	0	0	0-0-5-194-3-0	1	2.77809E-05
SAL_QA0121AA	PNUSAS041911	Adelaide	0	0	0	0	0	0	0	0	0	0	0	0-3-7-12-3-0	3	8.33426E-05
SAL_QA0123AA	PNUSAS041902	Adelaide	0	0	0	0	0	0	0	0	0	0	0	5-4-8-0-0-0	3	8.33426E-05
SAL_QA0128AA	PNUSAS041913	Adelaide	0	0	0	0	0	0	0	0	0	0	0	0-3-5-189-3-0	4	0.000111123
SAL_QA0580AA	PNUSAS041862	Adelaide	0	0	0	0	0	0	0	0	0	0	0	0-0-43-195-3-0	2	5.55617E-05
SAL_QA1363AA	421597	Adelaide	0	0	0	0	0	0	0	0	0	0	0	0-3-0-197-3-0	1	2.77809E-05
SAL_QA3758AA	24-11-18-8100513-E	Adelaide	0	0	0	0	0	0	0	0	0	0	0	18-3-0-49-3-0	3	8.33426E-05
SAL_QA1060AA	PNUSAS042536	Adelaide	0	0	0	0	0	0	0	0	0	0	0	0-3-53-9-0-0	1	2.77809E-05
SAL_QA1062AA	PNUSAS042534	Adelaide	0	0	0	0	0	0	0	0	0	0	0	0-40-7-0-3-0	1	2.77809E-05
SAL_QA1028AA	PNUSAS042492	Adelaide	0	0	0	0	0	0	0	0	0	0	0	18-3-0-9-0-0	2	5.55617E-05
SAL_QA1024AA	PNUSAS042489	Adelaide	0	0	0	0	0	0	0	0	0	0	0	0-3-0-198-3-0	1	2.77809E-05
SAL_QA1068AA	PNUSAS042537	Adelaide	0	0	0	0	0	0	0	0	0	0	0	38-3-7-9-0-0	2	5.55617E-05
SAL_QA1019AA	PNUSAS042483	Adelaide	0	0	0	0	0	0	0	0	0	0	0	0-0-48-9-3-0	3	8.33426E-05
SAL_QA1026AA	PNUSAS042493	Adelaide	0	0	0	0	0	0	0	0	0	0	0	5-4-24-199-0-0	2	5.55617E-05
SAL_QA1022AA	PNUSAS041950	Adelaide	0	0	0	0	0	0	0	0	0	0	0	0-4-8-9-3-0	1	2.77809E-05
SAL_QA1021AA	PNUSAS042487	Adelaide	0	0	0	0	0	0	0	0	0	0	0	5-4-28-42-0-0	2	5.55617E-05
SAL_QA1007AA	PNUSAS042381	Adelaide	0	0	0	0	0	0	0	0	0	0	0	0-4-0-200-14-11	1	2.77809E-05
SAL_QA1008AA	PNUSAS042378	Adelaide	0	0	0	0	0	0	0	0	0	0	0	5-4-6-0-0-0	1	2.77809E-05
SAL_QA1004AA	PNUSAS042379	Adelaide	0	0	0	0	0	0	0	0	0	0	0	5-4-24-201-3-0	3	8.33426E-05
SAL_QA0996AA	PNUSAS042382	Adelaide	0	0	0	0	0	0	0	0	0	0	0	0-4-0-0-14-11	3	8.33426E-05
SAL_QA0958AA	PNUSAS042262	Adelaide	0	0	0	0	0	0	0	0	0	0	0	5-4-24-0-0-0	5	0.000138904
SAL_QA1005AA	PNUSAS042380	Adelaide	0	0	0	0	0	0	0	0	0	0	0	0-0-0-0-13-0	2	5.55617E-05
SAL_QA2257AA	187611	Adelaide	0	0	0	0	0	0	0	0	0	0	0	0-0-7-0-3-0	4	0.000111123
SAL_QA2894AA	500295	Adelaide	0	0	0	0	0	0	0	0	0	0	0	0-4-31-200-14-11	1	2.77809E-05
SAL_QA3129AA	PNUSAS042924	Adelaide	0	0	0	0	0	0	0	0	0	0	0	0-0-28-0-12-0	1	2.77809E-05
SAL_QA3125AA	PNUSAS042922	Adelaide	0	0	0	0	0	0	0	0	0	0	0	18-3-7-51-3-0	5	0.000138904
SAL_QA3121AA	PNUSAS042923	Adelaide	0	0	0	0	0	0	0	0	0	0	0	0-3-5-112-3-0	4	0.000111123
SAL_QA3128AA	PNUSAS042494	Adelaide	0	0	0	0	0	0	0	0	0	0	0	0-0-48-0-3-0	1	2.77809E-05
SAL_QA3133AA	PNUSAS042910	Adelaide	0	0	0	0	0	0	0	0	0	0	0	0-3-5-124-0-0	1	2.77809E-05
SAL_QA3130AA	PNUSAS042497	Adelaide	0	0	0	0	0	0	0	0	0	0	0	0-3-5-146-0-0	1	2.77809E-05
SAL_QA3126AA	PNUSAS042916	Adelaide	0	0	0	0	0	0	0	0	0	0	0	16-3-5-9-0-0	2	5.55617E-05
SAL_QA3127AA	PNUSAS042500	Adelaide	0	0	0	0	0	0	0	0	0	0	0	18-3-67-0-3-0	12	0.00033337
SAL_QA3104AA	PNUSAS043211	Adelaide	0	0	0	0	0	0	0	0	0	0	0	0-0-5-9-0-0	4	0.000111123
SAL_QA3120AA	PNUSAS042925	Adelaide	0	0	0	0	0	0	0	0	0	0	0	0-3-0-204-3-0	3	8.33426E-05
SAL_QA3124AA	PNUSAS042495	Adelaide	0	0	0	0	0	0	0	0	0	0	0	0-0-7-9-3-0	23	0.00063896
SAL_QA3647AA	PNUSAS043006	Adelaide	0	0	0	0	0	0	0	0	0	0	0	0-0-78-44-0-0	2	5.55617E-05
SAL_QA4346AA	PNUSAS043385	Adelaide	0	0	0	0	0	0	0	0	0	0	0	16-3-7-44-0-0	11	0.00030559
SAL_QA4384AA	FDA00002726	Adelaide	0	0	0	0	0	0	0	0	0	0	0	16-3-7-114-0-0	1	2.77809E-05
SAL_QA4383AA	FDA00002725	Adelaide	0	0	0	0	0	0	0	0	0	0	0	40-3-48-9-0-0	1	2.77809E-05
SAL_QA4513AA	PNUSAS043109	Adelaide	0	0	0	0	0	0	0	0	0	0	0	1-1-0-0-1-3	1	2.77809E-05
SAL_QA4477AA	PNUSAS039510	Adelaide	0	0	0	0	0	0	0	0	0	0	0	0-0-0-46-3-0	3	8.33426E-05
SAL_QA4505AA	PNUSAS042908	Adelaide	0	0	0	0	0	0	0	0	0	0	0	0-0-30-0-3-0	4	0.000111123

SAL_QA5621AA	PNUSAS043451	Adelaide	0	0	0	0	0	0	0	0	0	0	0	
SAL_QA5622AA	PNUSAS042540	Adelaide	0	0	0	0	0	0	0	0	0	0	0	
SAL_QA5628AA	PNUSAS042541	Adelaide	0	0	0	0	0	0	0	0	0	0	0	
SAL_QA5614AA	PNUSAS043457	Adelaide	0	0	0	0	0	0	0	0	0	0	0	
SAL_QA5935AA	FSIS11810644	Adelaide	0	0	0	0	0	0	0	0	0	0	0	
SAL_QA5925AA	FSIS11810642	Adelaide	0	0	0	0	0	0	0	0	0	0	0	
SAL_QA6031AA	PNUSAS042757	Adelaide	0	0	0	0	0	0	0	0	0	0	0	
SAL_QA0552AA	PNUSAS041949	Adelaide	0	0	0	0	0	0	0	0	0	0	0	
SAL_QA6131AA	FDA00009763	Adelaide	0	0	0	0	0	0	0	0	0	0	0	
SAL_QA6890AA	PNUSAS043823	Adelaide	0	0	0	0	0	0	0	0	0	0	0	
SAL_QA6690AA	CFSAN082348	Adelaide	0	0	0	0	0	0	0	0	0	0	0	
SAL_QA6984AA	PNUSAS043565	Adelaide	0	0	0	0	0	0	0	0	0	0	0	
SAL_QA5999AA	PNUSAS043113	Adelaide	0	0	0	0	0	0	0	0	0	0	0	
SAL_QA6925AA	PNUSAS043816	Adelaide	0	0	0	0	0	0	0	0	0	0	0	
SAL_QA8064AA	FSIS11810909	Adelaide	0	0	0	0	0	0	0	0	0	0	0	
SAL_QA8474AA	PNUSAS043762	Adelaide	0	0	0	0	0	0	0	0	0	0	0	
SAL_QA8475AA	PNUSAS043755	Adelaide	0	0	0	0	0	0	0	0	0	0	0	
SAL_QA9107AA	PNUSAS044619	Adelaide	0	0	0	0	0	0	0	0	0	0	0	
SAL_QA9064AA	PNUSAS044148	Adelaide	0	0	0	0	0	0	0	0	0	0	0	
SAL_QA9072AA	PNUSAS043882	Adelaide	0	0	0	0	0	0	0	0	0	0	0	
SAL_QA9080AA	PNUSAS044532	Adelaide	0	0	0	0	0	0	0	0	0	0	0	
SAL_QA9546AA	5858	Adelaide	0	0	0	0	0	0	0	0	0	0	0	
SAL_QA9603AA	PNUSAS044657	Adelaide	0	0	0	0	0	0	0	0	0	0	0	
SAL_RA0160AA	PNUSAS044553	Adelaide	0	0	0	0	0	0	0	0	0	0	0	
SAL_RA1174AA	FSIS11810848	Adelaide	0	0	0	0	0	0	0	0	0	0	0	
SAL_RA1236AA	PNUSAS043768	Adelaide	0	0	0	0	0	0	0	0	0	0	0	
SAL_RA3120AA	PNUSAS045269	Adelaide	0	0	0	0	0	0	0	0	0	0	0	
SAL_RA3530AA	FSIS11811488	Adelaide	0	0	0	0	0	0	0	0	0	0	0	
SAL_RA4190AA	FSIS11811395	Adelaide	0	0	0	0	0	0	0	0	0	0	0	
SAL_RA6080AA	PNUSAS045354	Adelaide	0	0	0	0	0	0	0	0	0	0	0	
SAL_RA5956AA	PNUSAS044874	Adelaide	0	0	0	0	0	0	0	0	0	0	0	
SAL_RA5887AA	PNUSAS044055	Adelaide	0	0	0	0	0	0	0	0	0	0	0	
SAL_RA6334AA	PNUSAS044971	Adelaide	0	0	0	0	0	0	0	0	0	0	0	
SAL_RA6339AA	PNUSAS045374	Adelaide	0	0	0	0	0	0	0	0	0	0	0	
SAL_RA6712AA	FSIS11811653	Adelaide	0	0	0	0	0	0	0	0	0	0	0	
SAL_RA6845AA	FSIS11811761	Adelaide	0	0	0	0	0	0	0	0	0	0	0	
SAL_RA6948AA	FSIS11811413	Adelaide	0	0	0	0	0	0	0	0	0	0	0	
SAL_RA7586AA	PNUSAS042874	Adelaide	0	0	0	0	0	0	0	0	0	0	0	
SAL_RA7571AA	PNUSAS042875	Adelaide	0	0	0	0	0	0	0	0	0	0	0	
SAL_RA7569AA	PNUSAS042876	Adelaide	0	0	0	0	0	0	0	0	0	0	0	
SAL_RA7560AA	PNUSAS042878	Adelaide	0	0	0	0	0	0	0	0	0	0	0	
SAL_RA7781AA	FSIS11811731	Adelaide	0	0	0	0	0	0	0	0	0	0	0	
SAL_RA8659AA	FSIS11812161	Adelaide	0	0	0	0	0	0	0	0	0	0	0	
SAL_RA9300AA	CFSAN084131	Adelaide	0	0	0	0	0	0	0	0	0	0	0	
SAL_RA9302AA	CFSAN084108	Adelaide	0	0	0	0	0	0	0	0	0	0	0	
SAL_RA9303AA	CFSAN084166	Adelaide	0	0	0	0	0	0	0	0	0	0	0	
SAL_RA9393AA	CFSAN084049	Adelaide	0	0	0	0	0	0	0	0	0	0	0	
SAL_RA9376AA	CFSAN084194	Adelaide	0	0	0	0	0	0	0	0	0	0	0	
SAL_RA9908AA	FSIS11812496	Adelaide	0	0	0	0	0	0	0	0	0	0	0	
SAL_SA0020AA	CFSAN082343	Adelaide	0	0	0	0	0	0	0	0	0	0	0	
SAL_SA0019AA	CFSAN082342	Adelaide	0	0	0	0	0	0	0	0	0	0	0	
SAL_SA0000AA	FSIS11812732	Adelaide	0	0	0	0	0	0	0	0	0	0	0	
SAL_RA9975AA	FSIS11812395	Adelaide	0	0	0	0	0	0	0	0	0	0	0	
SAL_RA7578AA	PNUSAS042877	Adelaide	0	0	0	0	0	0	0	0	0	0	0	
SAL_SA1094AA	FSIS21821912	Adelaide	0	0	0	0	0	0	0	0	0	0	0	
SAL_SA1105AA	FSIS21821913	Adelaide	0	0	0	0	0	0	0	0	0	0	0	
SAL_SA3674AA	FSIS21822040	Adelaide	0	0	0	0	0	0	0	0	0	0	0	
SAL_SA5339AA	CK_99/00007745	Adelaide	0	0	0	0	0	0	0	0	0	0	0	
SAL_SA5337AA	CK_99/00006626	Adelaide	0	0	0	0	0	0	0	0	0	0	0	
SAL_SA5296AA	CK_98/00009070	Adelaide	0	0	0	0	0	0	0	0	0	0	0	
SAL_SA3653AA	FSIS11813681	Adelaide	0	0	0	0	0	0	0	0	0	0	0	
SAL_SA7918AA	376171	Adelaide	0	0	0	0	0	0	0	0	0	0	0	
SAL_SA7880AA	573562	Adelaide	0	0	0	0	0	0	0	0	0	0	0	
SAL_SA8852AA	FSIS11813782	Adelaide	0	0	0	0	0	0	0	0	0	0	0	
SAL_SA9159AA	CVM N17S603	Adelaide	0	0	0	0	0	0	0	0	0	0	0	
SAL_SA9702AA	FSIS21822163	Adelaide	0	0	0	0	0	0	0	0	0	0	0	
SAL_TA0137AA	FSIS11814481	Adelaide	0	0	0	0	0	0	0	0	0	0	0	
SAL_TA1051AA	52-9-18-8101039-P	Adelaide	0	0	0	0	0	0	0	0	0	0	0	
SAL_TA1365AA	FSIS21822305	Adelaide	0	0	0	0	0	0	0	0	0	0	0	
SAL_TA1369AA	FSIS21822298	Adelaide	0	0	0	0	0	0	0	0	0	0	0	
SAL_TA1882AA	FSIS11814866	Adelaide	0	0	0	0	0	0	0	0	0	0	0	
SAL_TA2189AA	FSIS21821990	Adelaide	0	0	0	0	0	0	0	0	0	0	0	
SAL_TA3584AA	FSIS11815058	Adelaide	0	0	0	0	0	0	0	0	0	0	0	

0-0-24-0-3-0	3	8.33426E-05
5-4-27-199-0-0	2	5.55617E-05
0-0-27-0-3-0	3	8.33426E-05
6-4-0-208-0-0	1	2.77809E-05
0-3-33-9-3-0	3	8.33426E-05
0-0-87-47-3-0	1	2.77809E-05
0-0-87-80-3-0	5	0.000138904
0-0-77-123-3-0	1	2.77809E-05
0-0-5-209-3-0	1	2.77809E-05
37-4-6-8-0-0	2	5.55617E-05
0-3-7-127-3-0	4	0.000111123
0-0-48-9-0-0	2	5.55617E-05
18-3-0-75-3-0	3	8.33426E-05
18-3-5-51-3-0	43	0.001194577
18-3-5-9-3-0	9	0.000250028
0-0-5-14-3-0	11	0.00030559
5-4-0-0-3-0	9	0.000250028
5-4-101-8-0-0	3	8.33426E-05
5-4-102-8-0-0	12	0.00033337
0-3-5-212-3-0	1	2.77809E-05
0-0-8-213-3-0	3	8.33426E-05
0-0-0-44-3-0	16	0.000444494
0-3-33-0-0-0	1	2.77809E-05
0-0-0-44-0-0	2	5.55617E-05
0-0-90-0-3-0	1	2.77809E-05
18-3-0-9-3-0	6	0.000166685
5-4-20-8-0-0	3	8.33426E-05
18-3-5-0-0-0	2	5.55617E-05
16-3-7-215-0-0	1	2.77809E-05
0-4-0-40-14-11	1	2.77809E-05
0-0-75-0-3-0	1	2.77809E-05
0-3-0-127-3-0	8	0.000222247
0-3-0-75-21-0	1	2.77809E-05
16-3-5-219-0-0	1	2.77809E-05
0-3-7-75-3-0	18	0.000500056
0-3-0-220-3-0	1	2.77809E-05
0-0-5-121-0-0	1	2.77809E-05
16-3-5-97-0-0	33	0.000916769
1-1-25-0-1-10	12	0.00033337
0-0-109-9-3-0	1	2.77809E-05
5-4-8-190-0-0	1	2.77809E-05
16-3-7-9-24-0	1	2.77809E-05
16-3-7-224-0-0	1	2.77809E-05
0-3-0-0-24-0	233	0.006472941
41-3-7-0-3-0	1	2.77809E-05
0-31-0-0-3-0	25	0.000694522
0-3-0-225-21-0	1	2.77809E-05
0-46-76-8-3-0	1	2.77809E-05
5-0-76-0-0-0	1	2.77809E-05
16-3-7-105-0-0	5	0.000138904
1-0-0-0-3-0	1	2.77809E-05
5-4-20-131-0-0	56	0.001555728
0-0-78-0-3-0	1	2.77809E-05
0-3-7-226-3-0	1	2.77809E-05
18-3-63-0-3-0	1	2.77809E-05
0-3-7-172-3-0	5	0.000138904
0-3-5-84-3-0	21	0.000583398
16-0-0-0-0-0	1	2.77809E-05
16-3-5-97-3-0	2	5.55617E-05
0-3-0-0-46-0	1	2.77809E-05
0-0-78-75-0-0	1	2.77809E-05
16-3-7-0-3-0	6	0.000166685
0-3-0-228-3-0	1	2.77809E-05
18-3-0-229-3-0	1	2.77809E-05
18-3-7-76-3-0	8	0.000222247
18-3-7-75-3-0	2	5.55617E-05
0-3-7-9-24-0	33	0.000916769
0-0-0-49-3-0	2	5.55617E-05
0-3-0-230-3-0	1	2.77809E-05
0-49-7-0-3-0	1	2.77809E-05
18-3-7-73-3-0	5	0.000138904
18-3-114-0-21-0	1	2.77809E-05
5-4-8-44-3-0	1	2.77809E-05

SAL_TA6767AA	FSIS21822615	Adelaide	0	0	0	0	0	0	0	0	0	0	0	
SAL_TA6948AA	627290	Adelaide	0	0	0	0	0	0	0	0	0	0	0	
SAL_PA2557AA	FSIS11808481	Adelaide	0	0	0	0	0	0	0	0	0	0	0	
SAL_OA1396AA	PNUSAS034749	Adelaide	0	0	0	0	0	0	0	0	0	0	0	
SAL_QA7981AA	PNUSAS043758	Adelaide	0	0	0	0	0	0	0	0	0	0	0	
SAL_TA8843AA	FSIS11815951	Adelaide	0	0	0	0	0	0	0	0	0	0	0	
SAL_QA7970AA	PNUSAS043759	Adelaide	0	0	0	0	0	0	0	0	0	0	0	
SAL_QA8006AA	PNUSAS041861	Adelaide	0	0	0	0	0	0	0	0	0	0	0	
SAL_QA8011AA	PNUSAS043735	Adelaide	0	0	0	0	0	0	0	0	0	0	0	
SAL_QA8005AA	PNUSAS043630	Adelaide	0	0	0	0	0	0	0	0	0	0	0	
SAL_QA8022AA	PNUSAS041860	Adelaide	0	0	0	0	0	0	0	0	0	0	0	
SAL_OA1276AA	PNUSAS033908	Adelaide	0	0	0	0	0	0	0	0	0	0	0	
SAL_OA1677AA	PNUSAS034577	Adelaide	0	0	0	0	0	0	0	0	0	0	0	
SAL_OA2389AA	CFSAN075554	Adelaide	0	0	0	0	0	0	0	0	0	0	0	
SAL_OA2377AA	CFSAN075555	Adelaide	0	0	0	0	0	0	0	0	0	0	0	
SAL_OA5637AA	FSIS11808314	Adelaide	0	0	0	0	0	0	0	0	0	0	0	
SAL_OA7444AA	FSIS1701260	Adelaide	0	0	0	0	0	0	0	0	0	0	0	
SAL_PA1411AA	PNUSAS038254	Adelaide	0	0	0	0	0	0	0	0	0	0	0	
SAL_PA2566AA	FSIS11808078	Adelaide	0	0	0	0	0	0	0	0	0	0	0	
SAL_PA2871AA	FSIS21821486	Adelaide	0	0	0	0	0	0	0	0	0	0	0	
SAL_PA2922AA	PNUSAS038984	Adelaide	0	0	0	0	0	0	0	0	0	0	0	
SAL_PA3730AA	PNUSAS038488	Adelaide	0	0	0	0	0	0	0	0	0	0	0	
SAL_TA9805AA	FSIS11816122	Adelaide	0	0	0	0	0	0	0	0	0	0	0	
SAL_TA9867AA	FSIS11816151	Adelaide	0	0	0	0	0	0	0	0	0	0	0	
SAL_TA9932AA	FSIS21822868	Adelaide	0	0	0	0	0	0	0	0	0	0	0	
SAL_TA9988AA	642277	Adelaide	0	0	0	0	0	0	0	0	0	0	0	
SAL_UA4873AA	CFSAN089073	Adelaide	0	0	0	0	0	0	0	0	0	0	0	
SAL_UA5155AA	FSIS11816328	Adelaide	0	0	0	0	0	0	0	0	0	0	0	
SAL_UA5150AA	FSIS11816334	Adelaide	0	0	0	0	0	0	0	0	0	0	0	
SAL_UA5173AA	FSIS11816351	Adelaide	0	0	0	0	0	0	0	0	0	0	0	
SAL_UA5179AA	FSIS11816355	Adelaide	0	0	0	0	0	0	0	0	0	0	0	
SAL_UA5208AA	FSIS21822952	Adelaide	0	0	0	0	0	0	0	0	0	0	0	
SAL_UA5888AA	FSIS11816708	Adelaide	0	0	0	0	0	0	0	0	0	0	0	
SAL_UA6371AA	FSIS31801359	Adelaide	0	0	0	0	0	0	0	0	0	0	0	
SAL_UA7951AA	FSIS21923158	Adelaide	0	0	0	0	0	0	0	0	0	0	0	
SAL_UA7953AA	FSIS31901413	Adelaide	0	0	0	0	0	0	0	0	0	0	0	
SAL_UA7759AA	FSIS11816974	Adelaide	0	0	0	0	0	0	0	0	0	0	0	
SAL_UA7420AA	FSIS31901429	Adelaide	0	0	0	0	0	0	0	0	0	0	0	
SAL_UA7447AA	664947	Adelaide	0	0	0	0	0	0	0	0	0	0	0	
SAL_VA7342AA	201701250	Adelaide	0	0	0	0	0	0	0	0	0	0	0	
SAL_VA2152AA	449722	Adelaide	0	0	0	0	0	0	0	0	0	0	0	
SAL_VA3154AA	FSIS11917339	Adelaide	0	0	0	0	0	0	0	0	0	0	0	
SAL_VA1806AA	PNUSAS055167	Adelaide	0	0	0	0	0	0	0	0	0	0	0	
SAL_VA2606AA	FSIS21923426	Adelaide	0	0	0	0	0	0	0	0	0	0	0	
SAL_VA5734AA	SA20030450	Adelaide	0	0	0	0	0	0	0	0	0	0	0	
SAL_VA4485AA	391254	Adelaide	0	0	0	0	0	0	0	0	0	0	0	
SAL_VA4351AA	391279	Adelaide	0	0	0	0	0	0	0	0	0	0	0	
SAL_VA7700AA	FSIS11917563	Adelaide	0	0	0	0	0	0	0	0	0	0	0	
SAL_VA7743AA	FSIS11917552	Adelaide	0	0	0	0	0	0	0	0	0	0	0	
SAL_VA7859AA	FSIS11917767	Adelaide	0	0	0	0	0	0	0	0	0	0	0	
SAL_VA7927AA	CFSAN091417	Adelaide	0	0	0	0	0	0	0	0	0	0	0	
SAL_VA7967AA	FSIS11917757	Adelaide	0	0	0	0	0	0	0	0	0	0	0	
SAL_VA8349AA	FSIS31901612	Adelaide	0	0	0	0	0	0	0	0	0	0	0	
SAL_VA8536AA	342538	Adelaide	0	0	0	0	0	0	0	0	0	0	0	
SAL_VA8613AA	FSIS11918002	Adelaide	0	0	0	0	0	0	0	0	0	0	0	
SAL_VA8603AA	FSIS11918013	Adelaide	0	0	0	0	0	0	0	0	0	0	0	
SAL_VA8942AA	299449	Adelaide	0	0	0	0	0	0	0	0	0	0	0	
SAL_VA4148AA	CDPH_CSalm157	Adelaide	0	0	0	0	0	0	0	0	0	0	0	
SAL_VA9855AA	FSIS11918034	Adelaide	0	0	0	0	0	0	0	0	0	0	0	
SAL_WA0111AA	FSIS11918297	Adelaide	0	0	0	0	0	0	0	0	0	0	0	
SAL_WA0611AA	188159	Adelaide	0	0	0	0	0	0	0	0	0	0	0	
SAL_WA1062AA	FSIS11918345	Adelaide	0	0	0	0	0	0	0	0	0	0	0	
SAL_VA4078AA	CDPH_CSalm273	Adelaide	0	0	0	0	0	0	0	0	0	0	0	
SAL_VA4070AA	CDPH_CSalm096	Adelaide	0	0	0	0	0	0	0	0	0	0	0	
SAL_WA2875AA	1090800914	Adelaide	0	0	0	0	0	0	0	0	0	0	0	
SAL_WA1470AA	FSIS11918930	Adelaide	0	0	0	0	0	0	0	0	0	0	0	
SAL_WA1584AA	FSIS11918937	Adelaide	0	0	0	0	0	0	0	0	0	0	0	
SAL_WA1885AA	FSIS31901668	Adelaide	0	0	0	0	0	0	0	0	0	0	0	
SAL_WA3495AA	CFSAN087445	Adelaide	0	0	0	0	0	0	0	0	0	0	0	
SAL_WA3651AA	CFSAN087512	Adelaide	0	0	0	0	0	0	0	0	0	0	0	
SAL_WA3996AA	FSIS11919193	Adelaide	0	0	0	0	0	0	0	0	0	0	0	
SAL_WA4015AA	FSIS11919185	Adelaide	0	0	0	0	0	0	0	0	0	0	0	
SAL_JA5441AA	353918	Adjame	0	0	0	0	0	0	0	0	0	0	0	

Enteritidis

45-3-7-9-0-0	4	0.000111123
0-3-116-44-3-0	2	5.55617E-05
0-3-7-153-3-0	2	5.55617E-05
0-3-0-153-3-0	2	5.55617E-05
0-3-7-44-3-0	14	0.000388932
0-3-5-124-48-0	1	2.77809E-05
0-3-0-233-3-0	1	2.77809E-05
18-3-5-83-3-0	5	0.000138904
1-1-1-0-1-3	6	0.000166685
18-3-117-0-3-0	1	2.77809E-05
5-4-6-138-0-0	2	5.55617E-05
18-3-5-13-3-0	2	5.55617E-05
18-3-91-0-3-0	6	0.000166685
46-3-5-0-3-0	3	8.33426E-05
18-3-7-172-3-0	3	8.33426E-05
18-3-0-234-3-0	2	5.55617E-05
18-3-33-0-3-0	24	0.000666741
18-3-5-51-0-0	1	2.77809E-05
18-3-5-235-3-0	1	2.77809E-05
46-3-0-0-3-0	1	2.77809E-05
16-3-7-236-0-0	5	0.000138904
16-3-5-146-0-0	5	0.000138904
5-0-8-75-0-0	1	2.77809E-05
47-4-119-0-3-0	1	2.77809E-05
16-3-120-9-0-0	1	2.77809E-05
18-3-5-180-3-0	1	2.77809E-05
0-3-56-0-21-0	1	2.77809E-05
48-3-0-0-0-0	28	0.000777864
5-0-0-9-0-0	1	2.77809E-05
49-3-0-0-0-0	28	0.000777864
49-3-123-0-0-0	1	2.77809E-05
50-3-124-0-0-0	1	2.77809E-05
48-3-125-0-0-0	1	2.77809E-05
49-3-126-0-0-0	1	2.77809E-05
16-3-127-9-0-0	1	2.77809E-05
48-3-128-0-0-0	1	2.77809E-05
51-3-129-0-0-0	1	2.77809E-05
51-3-0-0-0-0	6	0.000166685
52-3-0-0-0-0	4	0.000111123
53-3-0-0-0-0	5	0.000138904
16-3-130-9-0-0	1	2.77809E-05
50-3-0-0-0-0	7	0.000194466
16-3-131-241-0-0	1	2.77809E-05
54-3-0-0-0-0	3	8.33426E-05
0-3-124-0-0-0	1	2.77809E-05
55-3-0-0-0-0	1	2.77809E-05
16-3-132-9-0-0	1	2.77809E-05
50-3-133-0-0-0	1	2.77809E-05
16-3-124-9-0-0	2	5.55617E-05
54-3-134-83-0-0	1	2.77809E-05
56-3-0-0-0-0	1	2.77809E-05
16-3-135-9-0-0	2	5.55617E-05
57-3-0-0-0-0	1	2.77809E-05
49-3-125-0-0-0	1	2.77809E-05
48-3-135-0-0-0	1	2.77809E-05
49-3-136-0-0-0	1	2.77809E-05
58-3-0-0-0-0	1	2.77809E-05
16-3-137-9-0-0	1	2.77809E-05
0-3-138-0-0-0	1	2.77809E-05
59-3-0-0-0-0	1	2.77809E-05
49-3-139-0-0-0	1	2.77809E-05
60-3-0-0-0-0	1	2.77809E-05
16-3-140-242-0-0	1	2.77809E-05
48-3-141-83-0-0	1	2.77809E-05
49-3-124-0-0-0	1	2.77809E-05
0-3-135-0-0-0	1	2.77809E-05
61-3-0-0-0-0	1	2.77809E-05
0-0-8-75-0-0	6	0.000166685
5-4-77-75-0-0	1	2.77809E-05
0-3-7-0-24-0	21	0.000583398
0-3-7-244-3-0	8	0.000222247
0-0-50-52-3-0	3	8.33426E-05
0-3-5-114-3-0	7	0.000194466

SAL_JA8036AA	355050	Adjame	0	0	0	0	0	0	0	0	0	0	0	0-0-8-245-3-0	1	2.77809E-05
SAL_KA0245AA	SSI-AC209	Adjame	0	0	0	0	0	0	0	0	0	0	0	18-3-7-86-3-0	3	8.33426E-05
SAL_KA8742AA	356310	Adjame	0	0	0	0	0	0	0	0	0	0	0	0-0-0-161-3-0	21	0.000583398
SAL_KA8747AA	385774	Adjame	0	0	0	0	0	0	0	0	0	0	0	0-0-0-246-3-0	1	2.77809E-05
SAL_KA8744AA	374589	Adjame	0	0	0	0	0	0	0	0	0	0	0	18-0-0-247-3-0	1	2.77809E-05
SAL_KA8746AA	381330	Adjame	0	0	0	0	0	0	0	0	0	0	0	0-3-5-109-3-0	1	2.77809E-05
SAL_KA8743AA	357971	Adjame	0	0	0	0	0	0	0	0	0	0	0	0-55-7-0-3-0	1	2.77809E-05
SAL_KA8745AA	367320	Adjame	0	0	0	0	0	0	0	0	0	0	0	0-0-8-248-3-0	3	8.33426E-05
SAL_KA8752AA	388665	Adjame	0	0	0	0	0	0	0	0	0	0	0	0-3-7-225-3-0	1	2.77809E-05
SAL_KA8750AA	387137	Adjame	0	0	0	0	0	0	0	0	0	0	0	0-0-6-249-3-0	3	8.33426E-05
SAL_KA8751AA	387215	Adjame	0	0	0	0	0	0	0	0	0	0	0	0-3-48-12-3-0	1	2.77809E-05
SAL_KA8749AA	387049	Adjame	0	0	0	0	0	0	0	0	0	0	0	0-3-0-212-3-0	3	8.33426E-05
SAL_KA8756AA	389598	Adjame	0	0	0	0	0	0	0	0	0	0	0	0-0-5-250-3-0	1	2.77809E-05
SAL_KA8757AA	388789	Adjame	0	0	0	0	0	0	0	0	0	0	0	0-3-7-251-3-0	1	2.77809E-05
SAL_KA8758AA	399284	Adjame	0	0	0	0	0	0	0	0	0	0	0	0-0-8-44-0-0	10	0.000277809
SAL_KA8755AA	387507	Adjame	0	0	0	0	0	0	0	0	0	0	0	0-0-50-47-3-0	10	0.000277809
SAL_KA8754AA	387511	Adjame	0	0	0	0	0	0	0	0	0	0	0	0-0-0-73-0-0	1	2.77809E-05
SAL_KA8760AA	389724	Adjame	0	0	0	0	0	0	0	0	0	0	0	0-0-6-74-3-0	1	2.77809E-05
SAL_KA8931AA	353868	Adjame	0	0	0	0	0	0	0	0	0	0	0	0-4-0-252-15-0	2	5.55617E-05
SAL_KA8197AA	MS170185	Adjame	0	0	0	0	0	0	0	0	0	0	0	0-3-0-118-3-0	7	0.000194466
SAL_KA8199AA	MS170178	Adjame	0	0	0	0	0	0	0	0	0	0	0	0-3-7-95-0-0	1	2.77809E-05
SAL_LA0975AA	408388	Adjame	0	0	0	0	0	0	0	0	0	0	0	0-3-33-75-3-0	3	8.33426E-05
SAL_LA0974AA	409962	Adjame	0	0	0	0	0	0	0	0	0	0	0	0-0-0-51-3-0	1	2.77809E-05
SAL_LA0973AA	409961	Adjame	0	0	0	0	0	0	0	0	0	0	0	0-3-46-157-3-0	1	2.77809E-05
SAL_LA0976AA	409960	Adjame	0	0	0	0	0	0	0	0	0	0	0	0-3-0-253-3-0	1	2.77809E-05
SAL_LA8146AA	416016	Adjame	0	0	0	0	0	0	0	0	0	0	0	0-3-5-254-3-0	1	2.77809E-05
SAL_LA8149AA	411502	Adjame	0	0	0	0	0	0	0	0	0	0	0	10-4-6-8-0-0	13	0.000361151
SAL_MA5006AA	435414	Adjame	0	0	0	0	0	0	0	0	0	0	0	0-0-76-255-3-0	1	2.77809E-05
SAL_MA4448AA	388695	Adjame	0	0	0	0	0	0	0	0	0	0	0	0-0-5-256-3-0	1	2.77809E-05
SAL_MA4440AA	434304	Adjame	0	0	0	0	0	0	0	0	0	0	0	0-0-5-257-3-0	1	2.77809E-05
SAL_NA4979AA	411501	Adjame	0	0	0	0	0	0	0	0	0	0	0	18-0-8-80-3-0	1	2.77809E-05
SAL_QA5151AA	534836	Adjame	0	0	0	0	0	0	0	0	0	0	0	0-0-0-0-49-0	1	2.77809E-05
SAL_QA8290AA	416020	Adjame	0	0	0	0	0	0	0	0	0	0	0	0-3-7-258-3-0	2	5.55617E-05
SAL_RA6487AA	441469	Adjame	0	0	0	0	0	0	0	0	0	0	0	0-3-5-244-3-0	1	2.77809E-05
SAL_RA8132AA	456193	Adjame	0	0	0	0	0	0	0	0	0	0	0	18-0-8-47-3-0	1	2.77809E-05
SAL_UA8145AA	626680	Adjame	0	0	0	0	0	0	0	0	0	0	0	0-3-144-0-3-0	2	5.55617E-05
SAL_VA0602AA	201804529	Adjame	0	0	0	0	0	0	0	0	0	0	0	0-0-75-138-15-0	1	2.77809E-05
SAL_VA7129AA	683603	Adjame	0	0	0	0	0	0	0	0	0	0	0	18-3-0-124-3-0	2	5.55617E-05
SAL_VA7145AA	683602	Adjame	0	0	0	0	0	0	0	0	0	0	0	27-3-5-76-0-0	2	5.55617E-05
SAL_VA8735AA	683604	Adjame	0	0	0	0	0	0	0	0	0	0	0	18-3-86-0-3-0	1	2.77809E-05
SAL_BA7230AA	4650/1	Agama	0	0	0	0	0	0	0	0	0	0	0	18-3-67-9-3-0	4	0.000111123
SAL_CA6033AA	60032	Agama	0	0	0	0	0	0	0	0	0	0	0	0-0-0-11-3-0	1	2.77809E-05
SAL_CA1228AA	51281	Agama	0	0	0	0	0	0	0	0	0	0	0	0-3-5-259-3-0	2	5.55617E-05
SAL_AA8711AA	PO409	Agama	0	0	0	0	0	0	0	0	0	0	0	0-3-0-260-3-0	1	2.77809E-05
SAL_FA4695AA	112996	Agama	0	0	0	0	0	0	0	0	0	0	0	18-3-7-186-3-0	1	2.77809E-05
SAL_FA3425AA	126186	Agama	0	0	0	0	0	0	0	0	0	0	0	0-0-0-261-15-0	1	2.77809E-05
SAL_EA7511AA	103198	Agama	0	0	0	0	0	0	0	0	0	0	0	18-0-5-52-3-0	1	2.77809E-05
SAL_EA7517AA	43910	Agama	0	0	0	0	0	0	0	0	0	0	0	0-3-63-51-3-0	1	2.77809E-05
SAL_EA7403AA	53031	Agama	0	0	0	0	0	0	0	0	0	0	0	0-3-7-174-3-0	1	2.77809E-05
SAL_EA7125AA	46140	Agama	0	0	0	0	0	0	0	0	0	0	0	0-3-0-262-3-0	1	2.77809E-05
SAL_EA6878AA	82001	Agama	0	0	0	0	0	0	0	0	0	0	0	0-3-0-263-3-0	1	2.77809E-05
SAL_DA4756AA	PO409-2	Agama	0	0	0	0	0	0	0	0	0	0	0	0-0-50-44-3-0	3	8.33426E-05
SAL_DA1434AA	H120300221	Agama	0	0	0	0	0	0	0	0	0	0	0	0-3-0-264-3-0	1	2.77809E-05
SAL_DA1227AA	H124400520	Agama	0	0	0	0	0	0	0	0	0	0	0	0-58-0-212-3-0	1	2.77809E-05
SAL_DA0448AA	H122440453	Agama	0	0	0	0	0	0	0	0	0	0	0	0-3-0-265-3-0	1	2.77809E-05
SAL_CA6577AA	48228	Agama	0	0	0	0	0	0	0	0	0	0	0	0-0-84-73-3-0	1	2.77809E-05
SAL_CA6537AA	66837	Agama	0	0	0	0	0	0	0	0	0	0	0	5-4-8-248-0-0	1	2.77809E-05
SAL_CA6524AA	54063	Agama	0	0	0	0	0	0	0	0	0	0	0	0-0-5-266-3-0	1	2.77809E-05
SAL_CA6500AA	62579	Agama	0	0	0	0	0	0	0	0	0	0	0	0-3-145-0-3-0	1	2.77809E-05
SAL_CA6417AA	60047	Agama	0	0	0	0	0	0	0	0	0	0	0	0-0-6-124-3-0	2	5.55617E-05
SAL_CA6405AA	60409	Agama	0	0	0	0	0	0	0	0	0	0	0	0-3-146-0-3-0	1	2.77809E-05
SAL_CA6291AA	54026	Agama	0	0	0	0	0	0	0	0	0	0	0	0-0-0-247-3-0	1	2.77809E-05
SAL_CA6095AA	66445	Agama	0	0	0	0	0	0	0	0	0	0	0	0-0-0-89-3-0	2	5.55617E-05
SAL_CA6074AA	50143	Agama	0	0	0	0	0	0	0	0	0	0	0	0-0-0-95-3-0	2	5.55617E-05
SAL_CA5825AA	78749	Agama	0	0	0	0	0	0	0	0	0	0	0	0-3-33-267-3-0	1	2.77809E-05
SAL_CA5008AA	80317	Agama	0	0	0	0	0	0	0	0	0	0	0	0-3-5-0-24-0	1	2.77809E-05
SAL_CA4676AA	85752	Agama	0	0	0	0	0	0	0	0	0	0	0	18-3-7-49-3-0	4	0.000111123
SAL_CA4414AA	37790	Agama	0	0	0	0	0	0	0	0	0	0	0	18-3-7-89-3-0	9	0.000250028
SAL_CA4309AA	61903	Agama	0	0	0	0	0	0	0	0	0	0	0	0-0-43-70-3-0	1	2.77809E-05
SAL_CA4262AA	65413	Agama	0	0	0	0	0	0	0	0	0	0	0	0-3-5-85-3-0	1	2.77809E-05
SAL_CA4048AA	65022	Agama	0	0	0	0	0	0	0	0	0	0	0	0-0-90-11-3-0	1	2.77809E-05
SAL_CA3991AA	32513	Agama	0	0	0	0	0	0	0	0	0	0	0	0-3-5-44-0-0	1	2.77809E-05
SAL_CA3718AA	52989	Agama	0	0	0	0	0	0	0	0	0	0	0	0-25-0-0-3-0	2	5.55617E-05

SAL_CA3434AA	96961	Agama	0	0	0	0	0	0	0	0	0	0	0-0-6-103-3-0	2	5.55617E-05
SAL_CA3272AA	99740	Agama	0	0	0	0	0	0	0	0	0	0	0-0-5-123-3-0	9	0.000250028
SAL_CA3102AA	27025	Agama	0	0	0	0	0	0	0	0	0	0	0-0-5-271-3-0	1	2.77809E-05
SAL_CA2865AA	67558	Agama	0	0	0	0	0	0	0	0	0	0	0-0-8-272-3-0	1	2.77809E-05
SAL_CA2816AA	89894	Agama	0	0	0	0	0	0	0	0	0	0	0-0-30-46-3-0	1	2.77809E-05
SAL_CA2721AA	57100	Agama	0	0	0	0	0	0	0	0	0	0	64-4-8-0-3-0	1	2.77809E-05
SAL_CA2527AA	38759	Agama	0	0	0	0	0	0	0	0	0	0	0-3-46-276-3-0	1	2.77809E-05
SAL_CA2226AA	14900	Agama	0	0	0	0	0	0	0	0	0	0	40-3-0-0-3-0	1	2.77809E-05
SAL_CA1600AA	40262	Agama	0	0	0	0	0	0	0	0	0	0	18-3-5-73-3-0	3	8.33426E-05
SAL_CA1147AA	9462	Agama	0	0	0	0	0	0	0	0	0	0	0-3-48-44-3-0	1	2.77809E-05
SAL_CA0566AA	12123	Agama	0	0	0	0	0	0	0	0	0	0	0-0-48-44-0-0	3	8.33426E-05
SAL_BA8074AA	14878	Agama	0	0	0	0	0	0	0	0	0	0	16-3-152-9-0-0	4	0.000111123
SAL_BA7999AA	63724	Agama	0	0	0	0	0	0	0	0	0	0	0-3-0-51-24-0	1	2.77809E-05
SAL_HA5131AA	L04638-15	Agama	0	0	0	0	0	0	0	0	0	0	18-3-7-82-3-0	3	8.33426E-05
SAL_HA5123AA	L00742-14	Agama	0	0	0	0	0	0	0	0	0	0	0-3-0-140-3-0	3	8.33426E-05
SAL_HA5612AA	S00014-15	Agama	0	0	0	0	0	0	0	0	0	0	0-3-0-9-24-0	15	0.000416713
SAL_HA5670AA	L01178-15	Agama	0	0	0	0	0	0	0	0	0	0	18-3-7-0-24-0	10	0.000277809
SAL_HA5671AA	L00756-15	Agama	0	0	0	0	0	0	0	0	0	0	16-3-7-280-0-0	2	5.55617E-05
SAL_IA2942AA	121451	Agama	0	0	0	0	0	0	0	0	0	0	0-3-0-75-24-0	5	0.000138904
SAL_IA4803AA	MS160255	Agama	0	0	0	0	0	0	0	0	0	0	0-3-86-282-3-0	1	2.77809E-05
SAL_IA5061AA	MS160313	Agama	0	0	0	0	0	0	0	0	0	0	0-4-0-283-15-0	1	2.77809E-05
SAL_IA6925AA	MS150254	Agama	0	0	0	0	0	0	0	0	0	0	18-3-5-43-3-0	2	5.55617E-05
SAL_IA7277AA	S/20170029	Agama	0	0	0	0	0	0	0	0	0	0	0-0-8-279-3-0	1	2.77809E-05
SAL_JA6890AA	342654	Agama	0	0	0	0	0	0	0	0	0	0	0-3-0-285-3-0	1	2.77809E-05
SAL_JA8303AA	367228	Agama	0	0	0	0	0	0	0	0	0	0	17-4-6-8-0-0	4	0.000111123
SAL_KA1647AA	agama_HD_60J	Agama	0	0	0	0	0	0	0	0	0	0	18-3-0-9-24-0	1	2.77809E-05
SAL_KA1648AA	agama_HP_22G	Agama	0	0	0	0	0	0	0	0	0	0	0-3-67-75-3-0	1	2.77809E-05
SAL_CA1830AA	65042	Agama	0	0	0	0	0	0	0	0	0	0	0-3-0-0-51-0	1	2.77809E-05
SAL_CA0905AA	69913	Agama	0	0	0	0	0	0	0	0	0	0	0-3-0-225-3-0	1	2.77809E-05
SAL_DA1401AA	H124140823	Agama	0	0	0	0	0	0	0	0	0	0	38-3-7-0-3-0	1	2.77809E-05
SAL_CA5962AA	62537	Agama	0	0	0	0	0	0	0	0	0	0	0-0-158-0-3-0	1	2.77809E-05
SAL_CA0784AA	24049	Agama	0	0	0	0	0	0	0	0	0	0	16-3-5-289-0-0	6	0.000166685
SAL_CA3499AA	31614	Agama	0	0	0	0	0	0	0	0	0	0	18-3-7-225-3-0	2	5.55617E-05
SAL_HA5599AA	S04959-15	Agama	0	0	0	0	0	0	0	0	0	0	0-0-8-293-3-0	4	0.000111123
SAL_HA5665AA	L01836-15	Agama	0	0	0	0	0	0	0	0	0	0	0-3-0-76-24-0	4	0.000111123
SAL_GA1312AA	C1533	Agama	0	0	0	0	0	0	0	0	0	0	0-0-5-176-3-0	1	2.77809E-05
SAL_IA5900AA	MS160413	Agama	0	0	0	0	0	0	0	0	0	0	0-3-7-198-3-0	1	2.77809E-05
SAL_JA1258AA	MS170045	Agama	0	0	0	0	0	0	0	0	0	0	0-25-7-0-3-0	1	2.77809E-05
SAL_JA6227AA	333919	Agama	0	0	0	0	0	0	0	0	0	0	18-3-0-0-24-0	4	0.000111123
SAL_LA3367AA	MS170223	Agama	0	0	0	0	0	0	0	0	0	0	0-3-0-76-21-0	1	2.77809E-05
SAL_NA2592AA	MS170443	Agama	0	0	0	0	0	0	0	0	0	0	18-3-67-51-3-0	2	5.55617E-05
SAL_NA9887AA	MS170458	Agama	0	0	0	0	0	0	0	0	0	0	18-3-114-0-3-0	1	2.77809E-05
SAL_OA3749AA	18-8100156-V	Agama	0	0	0	0	0	0	0	0	0	0	18-3-5-89-24-0	1	2.77809E-05
SAL_PA0431AA	CFSAN064125	Agama	0	0	0	0	0	0	0	0	0	0	18-3-32-0-3-0	1	2.77809E-05
SAL_PA3778AA	18-8100315-F	Agama	0	0	0	0	0	0	0	0	0	0	5-4-5-312-0-0	1	2.77809E-05
SAL_PA4244AA	394001	Agama	0	0	0	0	0	0	0	0	0	0	5-4-8-313-0-0	1	2.77809E-05
SAL_PA4574AA	401615	Agama	0	0	0	0	0	0	0	0	0	0	18-3-0-0-0-0	1	2.77809E-05
SAL_PA4934AA	296156	Agama	0	0	0	0	0	0	0	0	0	0	0-0-0-109-0-0	1	2.77809E-05
SAL_PA6211AA	182086	Agama	0	0	0	0	0	0	0	0	0	0	16-3-67-9-0-0	12	0.00033337
SAL_PA6158AA	318314	Agama	0	0	0	0	0	0	0	0	0	0	24-4-20-314-0-0	5	0.000138904
SAL_PA6372AA	293036	Agama	0	0	0	0	0	0	0	0	0	0	0-0-0-0-56-0	1	2.77809E-05
SAL_PA7757AA	298989	Agama	0	0	0	0	0	0	0	0	0	0	0-67-8-0-3-0	1	2.77809E-05
SAL_PA8059AA	316328	Agama	0	0	0	0	0	0	0	0	0	0	18-0-5-0-3-0	1	2.77809E-05
SAL_PA8348AA	432264	Agama	0	0	0	0	0	0	0	0	0	0	0-0-0-0-57-0	1	2.77809E-05
SAL_PA8368AA	243900	Agama	0	0	0	0	0	0	0	0	0	0	18-0-5-9-3-0	1	2.77809E-05
SAL_PA8178AA	PNUSAS040856	Agama	0	0	0	0	0	0	0	0	0	0	18-3-5-289-0-0	1	2.77809E-05
SAL_PA8665AA	377312	Agama	0	0	0	0	0	0	0	0	0	0	5-4-172-45-0-0	1	2.77809E-05
SAL_PA8916AA	260207	Agama	0	0	0	0	0	0	0	0	0	0	0-3-5-316-3-0	2	5.55617E-05
SAL_PA9503AA	416389	Agama	0	0	0	0	0	0	0	0	0	0	10-4-6-25-0-0	6	0.000166685
SAL_PA9483AA	302557	Agama	0	0	0	0	0	0	0	0	0	0	5-4-28-40-0-0	1	2.77809E-05
SAL_PA9965AA	113031	Agama	0	0	0	0	0	0	0	0	0	0	16-22-59-107-0-0	1	2.77809E-05
SAL_QA0456AA	283179	Agama	0	0	0	0	0	0	0	0	0	0	16-22-7-9-0-0	1	2.77809E-05
SAL_QA0829AA	312095	Agama	0	0	0	0	0	0	0	0	0	0	5-4-27-41-0-0	1	2.77809E-05
SAL_QA2178AA	229043	Agama	0	0	0	0	0	0	0	0	0	0	15-4-6-8-0-0	1	2.77809E-05
SAL_QA2104AA	516305	Agama	0	0	0	0	0	0	0	0	0	0	6-4-34-54-15-0	1	2.77809E-05
SAL_QA2700AA	143641	Agama	0	0	0	0	0	0	0	0	0	0	7-4-0-0-6-0	1	2.77809E-05
SAL_QA2665AA	214732	Agama	0	0	0	0	0	0	0	0	0	0	19-0-0-0-0-0	1	2.77809E-05
SAL_QA3286AA	320940	Agama	0	0	0	0	0	0	0	0	0	0	0-4-0-0-4-0	1	2.77809E-05
SAL_QA4697AA	292945	Agama	0	0	0	0	0	0	0	0	0	0	5-4-20-103-0-0	1	2.77809E-05
SAL_QA5081AA	157233	Agama	0	0	0	0	0	0	0	0	0	0	0-4-50-0-3-0	1	2.77809E-05
SAL_QA5895AA	319683	Agama	0	0	0	0	0	0	0	0	0	0	18-50-5-0-3-0	2	5.55617E-05
SAL_QA6227AA	274272	Agama	0	0	0	0	0	0	0	0	0	0	1-0-0-8-1-10	1	2.77809E-05
SAL_QA6197AA	314933	Agama	0	0	0	0	0	0	0	0	0	0	0-1-25-8-1-10	1	2.77809E-05

SAL_QA7916AA	372677	Agama	0	0	0	0	0	0	0	0	0	0	0	0-31-76-8-3-0	1	2.77809E-05
SAL_QA7844AA	114051	Agama	0	0	0	0	0	0	0	0	0	0	0	0-4-0-214-15-0	1	2.77809E-05
SAL_QA9511AA	134267	Agama	0	0	0	0	0	0	0	0	0	0	0	0-0-103-47-3-0	1	2.77809E-05
SAL_QA9303AA	179630	Agama	0	0	0	0	0	0	0	0	0	0	0	0-3-7-10-3-0	1	2.77809E-05
SAL_RA0332AA	416390	Agama	0	0	0	0	0	0	0	0	0	0	0	0-3-91-12-3-0	1	2.77809E-05
SAL_RA0181AA	224058	Agama	0	0	0	0	0	0	0	0	0	0	0	0-3-91-0-3-0	4	0.000111123
SAL_RA0847AA	427422	Agama	0	0	0	0	0	0	0	0	0	0	0	63-3-7-9-0-0	1	2.77809E-05
SAL_RA0689AA	173555	Agama	0	0	0	0	0	0	0	0	0	0	0	0-0-153-52-3-0	1	2.77809E-05
SAL_RA2891AA	157365	Agama	0	0	0	0	0	0	0	0	0	0	0	18-3-48-44-0-0	1	2.77809E-05
SAL_RA3241AA	261343	Agama	0	0	0	0	0	0	0	0	0	0	0	0-0-8-318-3-0	4	0.000111123
SAL_RA5637AA	155699	Agama	0	0	0	0	0	0	0	0	0	0	0	5-68-173-319-0-0	1	2.77809E-05
SAL_RA5429AA	302577	Agama	0	0	0	0	0	0	0	0	0	0	0	5-4-27-320-0-0	1	2.77809E-05
SAL_RA2615AA	318615	Agama	0	0	0	0	0	0	0	0	0	0	0	18-3-7-9-21-0	1	2.77809E-05
SAL_SA2384AA	UoLiv_IIGH_256_59R	Agama	0	0	0	0	0	0	0	0	0	0	0	0-3-179-9-0-0	1	2.77809E-05
SAL_SA2383AA	UoLiv_IIGH_255_26B	Agama	0	0	0	0	0	0	0	0	0	0	0	0-3-5-229-3-0	3	8.33426E-05
SAL_SA2382AA	UoLiv_IIGH_253_25Y	Agama	0	0	0	0	0	0	0	0	0	0	0	18-3-0-51-24-0	1	2.77809E-05
SAL_SA2381AA	UoLiv_IIGH_251_30Y	Agama	0	0	0	0	0	0	0	0	0	0	0	74-3-7-0-3-0	1	2.77809E-05
SAL_SA2380AA	UoLiv_IIGH_241_W7	Agama	0	0	0	0	0	0	0	0	0	0	0	0-3-62-0-24-0	1	2.77809E-05
SAL_SA2379AA	UoLiv_IIGH_239_8Y	Agama	0	0	0	0	0	0	0	0	0	0	0	18-3-7-236-0-0	1	2.77809E-05
SAL_SA2378AA	UoLiv_IIGH_238_44R	Agama	0	0	0	0	0	0	0	0	0	0	0	16-3-0-9-3-0	1	2.77809E-05
SAL_SA2377AA	UoLiv_IIGH_237_U68	Agama	0	0	0	0	0	0	0	0	0	0	0	0-3-0-323-3-0	1	2.77809E-05
SAL_SA2376AA	UoLiv_IIGH_236_18Y	Agama	0	0	0	0	0	0	0	0	0	0	0	75-3-7-9-0-0	1	2.77809E-05
SAL_SA2375AA	UoLiv_IIGH_228_2Y	Agama	0	0	0	0	0	0	0	0	0	0	0	18-3-67-76-3-0	1	2.77809E-05
SAL_SA2374AA	UoLiv_IIGH_226_82W	Agama	0	0	0	0	0	0	0	0	0	0	0	0-3-7-324-3-0	1	2.77809E-05
SAL_SA2373AA	UoLiv_IIGH_225_99W	Agama	0	0	0	0	0	0	0	0	0	0	0	0-3-0-236-3-0	1	2.77809E-05
SAL_SA2372AA	UoLiv_IIGH_223_53B	Agama	0	0	0	0	0	0	0	0	0	0	0	0-3-5-97-0-0	1	2.77809E-05
SAL_SA2371AA	UoLiv_IIGH_221_49B	Agama	0	0	0	0	0	0	0	0	0	0	0	16-3-7-325-0-0	1	2.77809E-05
SAL_SA2370AA	UoLiv_IIGH_218_45B	Agama	0	0	0	0	0	0	0	0	0	0	0	0-3-0-326-3-0	1	2.77809E-05
SAL_SA2369AA	UoLiv_IIGH_217_42G	Agama	0	0	0	0	0	0	0	0	0	0	0	18-3-7-44-3-0	2	5.55617E-05
SAL_SA2368AA	UoLiv_IIGH_211_17R	Agama	0	0	0	0	0	0	0	0	0	0	0	18-3-5-51-24-0	2	5.55617E-05
SAL_SA2367AA	UoLiv_IIGH_210_9B	Agama	0	0	0	0	0	0	0	0	0	0	0	0-0-78-44-3-0	1	2.77809E-05
SAL_SA2366AA	UoLiv_IIGH_209_12G	Agama	0	0	0	0	0	0	0	0	0	0	0	0-73-0-0-3-0	1	2.77809E-05
SAL_SA2365AA	UoLiv_IIGH_208_4W	Agama	0	0	0	0	0	0	0	0	0	0	0	18-3-5-146-3-0	1	2.77809E-05
SAL_SA2364AA	UoLiv_IIGH_207_8B	Agama	0	0	0	0	0	0	0	0	0	0	0	18-3-67-73-3-0	1	2.77809E-05
SAL_SA2363AA	UoLiv_IIGH_206_5B	Agama	0	0	0	0	0	0	0	0	0	0	0	5-4-0-9-0-0	1	2.77809E-05
SAL_SA2362AA	UoLiv_IIGH_205_32B	Agama	0	0	0	0	0	0	0	0	0	0	0	0-3-5-51-0-0	1	2.77809E-05
SAL_SA2361AA	UoLiv_IIGH_204_26B	Agama	0	0	0	0	0	0	0	0	0	0	0	77-3-7-9-3-0	3	8.33426E-05
SAL_SA2360AA	UoLiv_IIGH_203_33B	Agama	0	0	0	0	0	0	0	0	0	0	0	18-3-0-0-21-0	1	2.77809E-05
SAL_SA2359AA	UoLiv_IIGH_201_19B	Agama	0	0	0	0	0	0	0	0	0	0	0	69-13-30-46-0-0	2	5.55617E-05
SAL_SA2355AA	UoLiv_IIGH_189_2Y	Agama	0	0	0	0	0	0	0	0	0	0	0	0-0-5-336-3-0	1	2.77809E-05
SAL_SA2354AA	UoLiv_IIGH_162_56B	Agama	0	0	0	0	0	0	0	0	0	0	0	18-25-7-0-3-0	1	2.77809E-05
SAL_SA2353AA	UoLiv_IIGH_163_39W	Agama	0	0	0	0	0	0	0	0	0	0	0	0-0-50-343-3-0	1	2.77809E-05
SAL_SA2352AA	UoLiv_IIGH_164_57W	Agama	0	0	0	0	0	0	0	0	0	0	0	0-3-86-51-3-0	5	0.000138904
SAL_SA2351AA	UoLiv_IIGH_160_82W	Agama	0	0	0	0	0	0	0	0	0	0	0	0-3-48-48-3-0	1	2.77809E-05
SAL_SA2350AA	UoLiv_IIGH_136_65B	Agama	0	0	0	0	0	0	0	0	0	0	0	0-0-8-345-3-0	1	2.77809E-05
SAL_SA2349AA	UoLiv_IIGH_137_38B	Agama	0	0	0	0	0	0	0	0	0	0	0	0-3-44-6-3-0	1	2.77809E-05
SAL_SA2348AA	UoLiv_IIGH_134_37B	Agama	0	0	0	0	0	0	0	0	0	0	0	0-3-7-346-3-0	1	2.77809E-05
SAL_SA2347AA	UoLiv_IIGH_141_T81	Agama	0	0	0	0	0	0	0	0	0	0	0	0-0-8-347-3-0	5	0.000138904
SAL_SA2346AA	UoLiv_IIGH_135_63B	Agama	0	0	0	0	0	0	0	0	0	0	0	0-3-62-138-3-0	2	5.55617E-05
SAL_SA2345AA	UoLiv_IIGH_159_52B	Agama	0	0	0	0	0	0	0	0	0	0	0	0-3-46-9-3-0	1	2.77809E-05
SAL_SA2344AA	UoLiv_IIGH_140_45B	Agama	0	0	0	0	0	0	0	0	0	0	0	0-3-7-121-3-0	1	2.77809E-05
SAL_SA2343AA	UoLiv_IIGH_145_82W	Agama	0	0	0	0	0	0	0	0	0	0	0	0-76-0-0-3-0	1	2.77809E-05
SAL_SA2342AA	UoLiv_IIGH_144_7B	Agama	0	0	0	0	0	0	0	0	0	0	0	0-0-8-350-3-0	1	2.77809E-05
SAL_SA2341AA	UoLiv_IIGH_156_8B	Agama	0	0	0	0	0	0	0	0	0	0	0	16-3-7-82-3-0	1	2.77809E-05
SAL_SA2339AA	UoLiv_IIGH_152_42B	Agama	0	0	0	0	0	0	0	0	0	0	0	0-3-46-79-3-0	1	2.77809E-05
SAL_SA2337AA	UoLiv_IIGH_119_96W	Agama	0	0	0	0	0	0	0	0	0	0	0	0-3-0-13-3-0	2	5.55617E-05
SAL_SA2336AA	UoLiv_IIGH_114_59B	Agama	0	0	0	0	0	0	0	0	0	0	0	18-3-48-192-3-0	1	2.77809E-05
SAL_SA2335AA	UoLiv_IIGH_118_32B	Agama	0	0	0	0	0	0	0	0	0	0	0	18-3-0-112-3-0	1	2.77809E-05
SAL_SA2334AA	UoLiv_IIGH_131_60B	Agama	0	0	0	0	0	0	0	0	0	0	0	0-3-5-43-3-0	1	2.77809E-05
SAL_SA2333AA	UoLiv_IIGH_115_69R	Agama	0	0	0	0	0	0	0	0	0	0	0	0-3-48-124-3-0	1	2.77809E-05
SAL_SA2331AA	UoLiv_IIGH_120_79W	Agama	0	0	0	0	0	0	0	0	0	0	0	0-3-33-354-3-0	3	8.33426E-05
SAL_SA2328AA	UoLiv_IIGH_104_19B	Agama	0	0	0	0	0	0	0	0	0	0	0	0-3-44-72-3-0	1	2.77809E-05
SAL_SA2325AA	UoLiv_IIGH_108_25B	Agama	0	0	0	0	0	0	0	0	0	0	0	0-3-7-204-3-0	2	5.55617E-05
SAL_SA2324AA	UoLiv_IIGH_112_103W	Agama	0	0	0	0	0	0	0	0	0	0	0	0-4-0-355-15-0	1	2.77809E-05
SAL_SA2323AA	UoLiv_IIGH_117_I7	Agama	0	0	0	0	0	0	0	0	0	0	0	0-0-8-349-3-0	4	0.000111123
SAL_SA2320AA	UoLiv_IIGH_42a_45B	Agama	0	0	0	0	0	0	0	0	0	0	0	0-4-75-0-15-0	6	0.000166685
SAL_SA2319AA	UoLiv_IIGH_41a_38B	Agama	0	0	0	0	0	0	0	0	0	0	0	0-0-7-89-3-0	1	2.77809E-05
SAL_SA2318AA	UoLiv_IIGH_45a_82W	Agama	0	0	0	0	0	0	0	0	0	0	0	0-3-5-353-0-0	1	2.77809E-05
SAL_SA2316AA	UoLiv_IIGH_48a_46B	Agama	0	0	0	0	0	0	0	0	0	0	0	18-3-5-121-3-0	1	2.77809E-05
SAL_SA2315AA	UoLiv_IIGH_46a_43B	Agama	0	0	0	0	0	0	0	0	0	0	0	0-3-33-357-3-0	1	2.77809E-05
SAL_SA2313AA	UoLiv_IIGH_40_38R	Agama	0	0	0	0	0	0	0	0	0	0	0	0-0-6-89-3-0	1	2.77809E-05
SAL_SA2311AA	UoLiv_IIGH_44_39W	Agama	0	0	0	0	0	0	0	0	0	0	0	0-3-86-358-3-0	1	2.77809E-05
SAL_SA2310AA	UoLiv_IIGH_49_Y69	Agama	0	0	0	0	0	0	0	0	0	0	0	0-3-63-43-3-0	1	2.77809E-05

SAL_SA2357AA	UoLiv_IIGH_196_20B	Agama	0	0	0	0	0	0	0	0	0	0	0
SAL_SA2312AA	UoLiv_IIGH_41_47R	Agama	0	0	0	0	0	0	0	0	0	0	0
SAL_SA2309AA	UoLiv_IIGH_26_3B	Agama	0	0	0	0	0	0	0	0	0	0	0
SAL_SA2308AA	UoLiv_IIGH_46_52W	Agama	0	0	0	0	0	0	0	0	0	0	0
SAL_SA1881AA	SE06/76	Agama	0	0	0	0	0	0	0	0	0	0	0
SAL_SA1780AA	MC_08-0285	Agama	0	0	0	0	0	0	0	0	0	0	0
SAL_SA1779AA	MC_08-0278	Agama	0	0	0	0	0	0	0	0	0	0	0
SAL_SA1766AA	MC_06-0122	Agama	0	0	0	0	0	0	0	0	0	0	0
SAL_RA7471AA	222191	Agama	0	0	0	0	0	0	0	0	0	0	0
SAL_RA7357AA	367501	Agama	0	0	0	0	0	0	0	0	0	0	0
SAL_RA7520AA	245082	Agama	0	0	0	0	0	0	0	0	0	0	0
SAL_RA7812AA	372704	Agama	0	0	0	0	0	0	0	0	0	0	0
SAL_SA3884AA	SMG-18-1659	Agama	0	0	0	0	0	0	0	0	0	0	0
SAL_SA3885AA	SMG-18-1660	Agama	0	0	0	0	0	0	0	0	0	0	0
SAL_SA3887AA	SMG-18-1662	Agama	0	0	0	0	0	0	0	0	0	0	0
SAL_SA3882AA	SMG-18-1658	Agama	0	0	0	0	0	0	0	0	0	0	0
SAL_SA3889AA	SMG-18-1665	Agama	0	0	0	0	0	0	0	0	0	0	0
SAL_SA3891AA	SMG-18-1667	Agama	0	0	0	0	0	0	0	0	0	0	0
SAL_SA3886AA	SMG-18-1663	Agama	0	0	0	0	0	0	0	0	0	0	0
SAL_SA3888AA	SMG-18-1664	Agama	0	0	0	0	0	0	0	0	0	0	0
SAL_SA3941AA	201710297	Agama	0	0	0	0	0	0	0	0	0	0	0
SAL_SA3940AA	201710830	Agama	0	0	0	0	0	0	0	0	0	0	0
SAL_SA3944AA	201802780	Agama	0	0	0	0	0	0	0	0	0	0	0
SAL_SA3945AA	201706979	Agama	0	0	0	0	0	0	0	0	0	0	0
SAL_SA3946AA	201804272	Agama	0	0	0	0	0	0	0	0	0	0	0
SAL_SA3948AA	201805388	Agama	0	0	0	0	0	0	0	0	0	0	0
SAL_SA5244AA	1090600297	Agama	0	0	0	0	0	0	0	0	0	0	0
SAL_SA5977AA	586016	Agama	0	0	0	0	0	0	0	0	0	0	0
SAL_SA5810AA	586015	Agama	0	0	0	0	0	0	0	0	0	0	0
SAL_SA6479AA	586014	Agama	0	0	0	0	0	0	0	0	0	0	0
SAL_SA6335AA	598943	Agama	0	0	0	0	0	0	0	0	0	0	0
SAL_SA6768AA	591932	Agama	0	0	0	0	0	0	0	0	0	0	0
SAL_SA7232AA	155673	Agama	0	0	0	0	0	0	0	0	0	0	0
SAL_SA7411AA	602743	Agama	0	0	0	0	0	0	0	0	0	0	0
SAL_SA7874AA	594727	Agama	0	0	0	0	0	0	0	0	0	0	0
SAL_SA8997AA	385789	Agama	0	0	0	0	0	0	0	0	0	0	0
SAL_SA9522AA	317694	Agama	0	0	0	0	0	0	0	0	0	0	0
SAL_TA0467AA	613680	Agama	0	0	0	0	0	0	0	0	0	0	0
SAL_TA0354AA	612610	Agama	0	0	0	0	0	0	0	0	0	0	0
SAL_TA0364AA	611059	Agama	0	0	0	0	0	0	0	0	0	0	0
SAL_TA0909AA	617856	Agama	0	0	0	0	0	0	0	0	0	0	0
SAL_TA0765AA	203626	Agama	0	0	0	0	0	0	0	0	0	0	0
SAL_TA1440AA	18-SA01936	Agama	0	0	0	0	0	0	0	0	0	0	0
SAL_TA1441AA	07-01639	Agama	0	0	0	0	0	0	0	0	0	0	0
SAL_TA1444AA	99-04095	Agama	0	0	0	0	0	0	0	0	0	0	0
SAL_TA1442AA	15-SA00983	Agama	0	0	0	0	0	0	0	0	0	0	0
SAL_TA1445AA	06-02778	Agama	0	0	0	0	0	0	0	0	0	0	0
SAL_TA1443AA	08-04121	Agama	0	0	0	0	0	0	0	0	0	0	0
SAL_TA2278AA	620504	Agama	0	0	0	0	0	0	0	0	0	0	0
SAL_TA6167AA	607865	Agama	0	0	0	0	0	0	0	0	0	0	0
SAL_TA6560AA	632321	Agama	0	0	0	0	0	0	0	0	0	0	0
SAL_TA7449AA	634653	Agama	0	0	0	0	0	0	0	0	0	0	0
SAL_TA9577AA	640982	Agama	0	0	0	0	0	0	0	0	0	0	0
SAL_TA9904AA	CFSAN083273	Agama	0	0	0	0	0	0	0	0	0	0	0
SAL_TA9909AA	CFSAN083269	Agama	0	0	0	0	0	0	0	0	0	0	0
SAL_TA9908AA	CFSAN083279	Agama	0	0	0	0	0	0	0	0	0	0	0
SAL_TA9906AA	CFSAN083288	Agama	0	0	0	0	0	0	0	0	0	0	0
SAL_TA9900AA	CFSAN083268	Agama	0	0	0	0	0	0	0	0	0	0	0
SAL_TA9907AA	CFSAN083270	Agama	0	0	0	0	0	0	0	0	0	0	0
SAL_TA9962AA	CFSAN083275	Agama	0	0	0	0	0	0	0	0	0	0	0
SAL_UA2000AA	ERS1976889	Agama	0	0	0	0	0	0	0	0	0	0	0
SAL_UA2699AA	648975	Agama	0	0	0	0	0	0	0	0	0	0	0
SAL_UA7266AA	MRS-11/01959	Agama	0	0	0	0	0	0	0	0	0	0	0
SAL_UA7275AA	00/00011101	Agama	0	0	0	0	0	0	0	0	0	0	0
SAL_UA7272AA	07/00001175	Agama	0	0	0	0	0	0	0	0	0	0	0
SAL_UA7268AA	MRS-18/02060	Agama	0	0	0	0	0	0	0	0	0	0	0
SAL_UA7270AA	09/00003560	Agama	0	0	0	0	0	0	0	0	0	0	0
SAL_UA7276AA	MRS-10/04198	Agama	0	0	0	0	0	0	0	0	0	0	0
SAL_UA7271AA	00/00009138	Agama	0	0	0	0	0	0	0	0	0	0	0
SAL_UA7278AA	MRS-18/02234	Agama	0	0	0	0	0	0	0	0	0	0	0
SAL_UA7277AA	MRS-18/00649	Agama	0	0	0	0	0	0	0	0	0	0	0
SAL_UA4849AA	592651	Agama	0	0	0	0	0	0	0	0	0	0	0
SAL_UA6707AA	MS140586	Agama	0	0	0	0	0	0	0	0	0	0	0

6-4-34-208-15-0	1	2.77809E-05
0-3-0-354-3-0	5	0.000138904
0-3-44-124-3-0	2	5.55617E-05
0-3-0-212-0-0	1	2.77809E-05
0-3-0-359-3-0	1	2.77809E-05
18-3-67-177-3-0	1	2.77809E-05
0-3-0-360-3-0	1	2.77809E-05
0-0-5-272-3-0	1	2.77809E-05
0-3-44-225-3-0	1	2.77809E-05
0-3-5-204-3-0	1	2.77809E-05
0-0-187-361-15-0	1	2.77809E-05
0-3-7-118-3-0	3	8.33426E-05
0-3-44-49-3-0	2	5.55617E-05
0-3-5-362-3-0	1	2.77809E-05
81-3-33-354-0-0	1	2.77809E-05
0-0-0-112-3-0	3	8.33426E-05
0-3-0-159-3-0	1	2.77809E-05
0-3-5-121-3-0	2	5.55617E-05
0-3-33-363-3-0	1	2.77809E-05
0-3-0-364-3-0	1	2.77809E-05
0-3-86-9-3-0	1	2.77809E-05
5-77-5-75-0-0	3	8.33426E-05
0-3-63-106-3-0	1	2.77809E-05
0-3-7-215-3-0	1	2.77809E-05
0-3-0-365-3-0	1	2.77809E-05
0-3-62-124-3-0	3	8.33426E-05
5-4-8-152-3-0	1	2.77809E-05
0-3-7-366-3-0	1	2.77809E-05
0-3-7-367-3-0	1	2.77809E-05
0-3-5-369-3-0	1	2.77809E-05
18-3-5-353-3-0	1	2.77809E-05
0-3-7-9-65-0	1	2.77809E-05
0-0-7-9-24-0	1	2.77809E-05
0-3-0-373-3-0	6	0.000166685
0-3-193-229-3-0	1	2.77809E-05
5-4-6-8-69-0	2	5.55617E-05
0-0-0-374-3-0	1	2.77809E-05
0-0-0-375-3-0	2	5.55617E-05
0-0-53-9-0-0	1	2.77809E-05
18-3-63-376-3-0	1	2.77809E-05
18-3-7-282-3-0	1	2.77809E-05
83-3-7-0-3-0	1	2.77809E-05
0-0-50-213-3-0	1	2.77809E-05
18-0-7-9-0-0	1	2.77809E-05
0-0-0-0-70-0	1	2.77809E-05
0-3-0-369-3-0	2	5.55617E-05
0-0-8-380-3-0	1	2.77809E-05
0-3-62-83-3-0	1	2.77809E-05
0-3-0-381-3-0	2	5.55617E-05
0-3-0-382-3-0	1	2.77809E-05
18-3-86-383-3-0	1	2.77809E-05
18-3-5-89-3-0	1	2.77809E-05
0-3-7-352-3-0	1	2.77809E-05
0-3-5-381-3-0	1	2.77809E-05
0-3-46-121-3-0	1	2.77809E-05
0-3-7-341-3-0	1	2.77809E-05
18-3-67-282-3-0	1	2.77809E-05
77-3-7-9-0-0	1	2.77809E-05
0-0-6-118-3-0	1	2.77809E-05
0-3-152-9-3-0	2	5.55617E-05
0-3-67-0-0-0	1	2.77809E-05
0-0-50-9-3-0	5	0.000138904
0-3-7-373-3-0	1	2.77809E-05
0-3-0-385-3-0	1	2.77809E-05
0-3-0-259-3-0	2	5.55617E-05
0-3-7-276-3-0	1	2.77809E-05
0-3-8-13-3-0	1	2.77809E-05
0-0-49-387-3-0	1	2.77809E-05
0-3-5-388-3-0	1	2.77809E-05
0-3-86-75-3-0	1	2.77809E-05
0-3-48-351-3-0	1	2.77809E-05
18-3-5-0-24-0	4	0.000111123
0-0-0-392-3-0	1	2.77809E-05

SAL_UA6708AA	MS130793	Agama	0	0	0	0	0	0	0	0	0	0	0	
SAL_UA6704AA	FXW_01_1215	Agama	0	0	0	0	0	0	0	0	0	0	0	
SAL_UA6709AA	FXW_04_5493	Agama	0	0	0	0	0	0	0	0	0	0	0	
SAL_UA6719AA	MS130768	Agama	0	0	0	0	0	0	0	0	0	0	0	
SAL_UA6725AA	MS130637	Agama	0	0	0	0	0	0	0	0	0	0	0	
SAL_UA6727AA	MS130743	Agama	0	0	0	0	0	0	0	0	0	0	0	
SAL_UA6729AA	MS130255	Agama	0	0	0	0	0	0	0	0	0	0	0	
SAL_UA6742AA	S09-0643	Agama	0	0	0	0	0	0	0	0	0	0	0	
SAL_UA6746AA	S08-0278	Agama	0	0	0	0	0	0	0	0	0	0	0	
SAL_UA6754AA	FXW_201407879	Agama	0	0	0	0	0	0	0	0	0	0	0	
SAL_UA6769AA	FXW_201210562	Agama	0	0	0	0	0	0	0	0	0	0	0	
SAL_UA6768AA	FXW_201208192	Agama	0	0	0	0	0	0	0	0	0	0	0	
SAL_UA6747AA	S08-0285	Agama	0	0	0	0	0	0	0	0	0	0	0	
SAL_UA6774AA	FXW_06_6595	Agama	0	0	0	0	0	0	0	0	0	0	0	
SAL_UA6750AA	FXW_201606062	Agama	0	0	0	0	0	0	0	0	0	0	0	
SAL_UA6751AA	FXW_201604917	Agama	0	0	0	0	0	0	0	0	0	0	0	
SAL_UA6779AA	FXW_201105850	Agama	0	0	0	0	0	0	0	0	0	0	0	
SAL_UA6753AA	FXW_201408360	Agama	0	0	0	0	0	0	0	0	0	0	0	
SAL_UA6755AA	FXW_201601891	Agama	0	0	0	0	0	0	0	0	0	0	0	
SAL_UA6759AA	FXW_201404594	Agama	0	0	0	0	0	0	0	0	0	0	0	
SAL_UA6761AA	FXW_01_3096	Agama	0	0	0	0	0	0	0	0	0	0	0	
SAL_UA6756AA	FXW_201508740	Agama	0	0	0	0	0	0	0	0	0	0	0	
SAL_UA6773AA	FXW_05_0901	Agama	0	0	0	0	0	0	0	0	0	0	0	
SAL_UA6760AA	FXW_201310169	Agama	0	0	0	0	0	0	0	0	0	0	0	
SAL_UA6758AA	FXW_201302816	Agama	0	0	0	0	0	0	0	0	0	0	0	
SAL_UA6775AA	FXW_09_8540	Agama	0	0	0	0	0	0	0	0	0	0	0	
SAL_UA6776AA	FXW_201001012	Agama	0	0	0	0	0	0	0	0	0	0	0	
SAL_UA6778AA	FXW_201005401	Agama	0	0	0	0	0	0	0	0	0	0	0	
SAL_UA6777AA	FXW_201002563	Agama	0	0	0	0	0	0	0	0	0	0	0	
SAL_UA6780AA	FXW_201206447	Agama	0	0	0	0	0	0	0	0	0	0	0	
SAL_UA6745AA	07-1012	Agama	0	0	0	0	0	0	0	0	0	0	0	
SAL_UA6724AA	MS130766	Agama	0	0	0	0	0	0	0	0	0	0	0	
SAL_UA6710AA	FXW_04_5492	Agama	0	0	0	0	0	0	0	0	0	0	0	
SAL_UA6757AA	FXW_201307999	Agama	0	0	0	0	0	0	0	0	0	0	0	
SAL_UA6766AA	07-0147	Agama	0	0	0	0	0	0	0	0	0	0	0	
SAL_UA9880AA	557280	Agama	0	0	0	0	0	0	0	0	0	0	0	
SAL_VA1138AA	508257	Agama	0	0	0	0	0	0	0	0	0	0	0	
SAL_VA2800AA	FXW_00_9826	Agama	0	0	0	0	0	0	0	0	0	0	0	
SAL_VA2798AA	MC_S09-0355	Agama	0	0	0	0	0	0	0	0	0	0	0	
SAL_VA2799AA	MC_MS100596	Agama	0	0	0	0	0	0	0	0	0	0	0	
SAL_SA1528AA	CK_00/00002320-2	Agama	0	0	0	0	0	0	0	0	0	0	0	
SAL_VA7594AA	201707070	Agama	0	0	0	0	0	0	0	0	0	0	0	
SAL_VA1882AA	440913	Agama	0	0	0	0	0	0	0	0	0	0	0	
SAL_VA2764AA	414682	Agama	0	0	0	0	0	0	0	0	0	0	0	
SAL_VA1962AA	438632	Agama	0	0	0	0	0	0	0	0	0	0	0	
SAL_VA5905AA	S00253-09	Agama	0	0	0	0	0	0	0	0	0	0	0	
SAL_WA0837AA	S06044-00	Agama	0	0	0	0	0	0	0	0	0	0	0	
SAL_WA0836AA	L03305-16	Agama	0	0	0	0	0	0	0	0	0	0	0	
SAL_WA0845AA	S06048-00	Agama	0	0	0	0	0	0	0	0	0	0	0	
SAL_WA0835AA	S09925-98	Agama	0	0	0	0	0	0	0	0	0	0	0	
SAL_WA0844AA	S04035-09	Agama	0	0	0	0	0	0	0	0	0	0	0	
SAL_WA0841AA	S06046-00	Agama	0	0	0	0	0	0	0	0	0	0	0	
SAL_WA0843AA	L03308-16	Agama	0	0	0	0	0	0	0	0	0	0	0	
SAL_WA0842AA	S05018-00	Agama	0	0	0	0	0	0	0	0	0	0	0	
SAL_WA0849AA	S06045-00	Agama	0	0	0	0	0	0	0	0	0	0	0	
SAL_WA0846AA	S03008-10	Agama	0	0	0	0	0	0	0	0	0	0	0	
SAL_WA0848AA	S03548-09	Agama	0	0	0	0	0	0	0	0	0	0	0	
SAL_WA0847AA	S06047-00	Agama	0	0	0	0	0	0	0	0	0	0	0	
SAL_WA0850AA	L00108-13	Agama	0	0	0	0	0	0	0	0	0	0	0	
SAL_WA0834AA	S03422-09	Agama	0	0	0	0	0	0	0	0	0	0	0	
SAL_WA0839AA	S03025-09	Agama	0	0	0	0	0	0	0	0	0	0	0	
SAL_WA0840AA	L03309-16	Agama	0	0	0	0	0	0	0	0	0	0	0	
SAL_WA0838AA	S02968-09	Agama	0	0	0	0	0	0	0	0	0	0	0	
SAL_VA4372AA	399372	Agama	0	0	0	0	0	0	0	0	0	0	0	
SAL_VA7177AA	385507	Agama	0	0	0	0	0	0	0	0	0	0	0	
SAL_VA7157AA	685506	Agama	0	0	0	0	0	0	0	0	0	0	0	
SAL_VA8640AA	315181	Agama	0	0	0	0	0	0	0	0	0	0	0	
SAL_VA9070AA	315003	Agama	0	0	0	0	0	0	0	0	0	0	0	
SAL_VA9032AA	301665	Agama	0	0	0	0	0	0	0	0	0	0	0	
SAL_VA8978AA	296803	Agama	0	0	0	0	0	0	0	0	0	0	0	
SAL_VA8903AA	296656	Agama	0	0	0	0	0	0	0	0	0	0	0	
SAL_WA0267AA	216349	Agama	0	0	0	0	0	0	0	0	0	0	0	
SAL_WA0872AA	149203	Agama	0	0	0	0	0	0	0	0	0	0	0	

0-3-0-393-3-0	1	2.77809E-05
18-3-5-79-3-0	1	2.77809E-05
18-3-5-75-3-0	2	5.55617E-05
18-3-7-373-3-0	2	5.55617E-05
0-3-0-212-24-0	1	2.77809E-05
0-3-62-225-3-0	1	2.77809E-05
0-3-7-48-3-0	1	2.77809E-05
0-3-199-0-3-0	1	2.77809E-05
0-3-86-204-3-0	1	2.77809E-05
18-3-199-9-3-0	1	2.77809E-05
0-3-46-76-3-0	1	2.77809E-05
0-0-86-0-3-0	1	2.77809E-05
0-3-5-177-3-0	1	2.77809E-05
18-3-62-6-3-0	1	2.77809E-05
0-0-7-394-3-0	1	2.77809E-05
0-3-86-92-3-0	1	2.77809E-05
0-3-7-212-3-0	1	2.77809E-05
18-3-0-177-3-0	1	2.77809E-05
0-3-44-75-3-0	1	2.77809E-05
0-3-62-73-3-0	1	2.77809E-05
0-3-46-89-3-0	1	2.77809E-05
0-3-0-186-3-0	1	2.77809E-05
0-3-0-177-24-0	1	2.77809E-05
0-0-8-395-3-0	1	2.77809E-05
0-3-5-396-3-0	1	2.77809E-05
5-4-119-44-0-0	1	2.77809E-05
0-0-5-152-3-0	2	5.55617E-05
0-3-44-95-3-0	1	2.77809E-05
85-3-7-9-24-0	1	2.77809E-05
0-3-7-159-3-0	1	2.77809E-05
0-0-0-199-3-0	1	2.77809E-05
86-3-199-0-3-0	1	2.77809E-05
0-3-46-51-3-0	1	2.77809E-05
0-3-48-174-3-0	1	2.77809E-05
0-3-5-399-3-0	1	2.77809E-05
0-3-0-352-3-0	2	5.55617E-05
0-0-8-111-3-0	3	8.33426E-05
18-3-7-114-3-0	1	2.77809E-05
87-3-7-9-0-0	1	2.77809E-05
34-4-8-9-3-0	3	8.33426E-05
0-3-5-401-3-0	1	2.77809E-05
0-0-5-402-3-0	1	2.77809E-05
0-3-0-403-3-0	1	2.77809E-05
5-4-49-13-0-0	2	5.55617E-05
0-0-84-112-3-0	1	2.77809E-05
18-3-7-151-3-0	3	8.33426E-05
0-3-0-244-3-0	1	2.77809E-05
0-79-7-75-3-0	1	2.77809E-05
0-0-0-115-3-0	1	2.77809E-05
6-4-0-208-15-0	1	2.77809E-05
0-80-5-47-3-0	1	2.77809E-05
0-0-0-405-3-0	1	2.77809E-05
0-0-8-313-3-0	1	2.77809E-05
0-0-5-407-3-0	1	2.77809E-05
0-0-0-0-77-0	3	8.33426E-05
0-3-44-83-3-0	2	5.55617E-05
18-3-7-9-24-0	1	2.77809E-05
0-0-0-0-78-0	1	2.77809E-05
0-3-0-282-3-0	2	5.55617E-05
0-3-48-341-3-0	1	2.77809E-05
0-0-50-123-3-0	2	5.55617E-05
0-3-86-259-3-0	1	2.77809E-05
0-0-0-79-3-0	1	2.77809E-05
0-0-50-176-3-0	1	2.77809E-05
0-3-44-259-3-0	1	2.77809E-05
0-3-7-315-3-0	1	2.77809E-05
88-3-7-9-0-0	1	2.77809E-05
0-3-0-410-3-0	2	5.55617E-05
16-3-7-97-0-0	1	2.77809E-05
0-3-63-83-3-0	1	2.77809E-05
0-3-7-236-3-0	1	2.77809E-05
0-3-0-413-3-0	1	2.77809E-05
0-0-5-293-3-0	2	5.55617E-05

SAL_WA0899AA	143991	Agama	0	0	0	0	0	0	0	0	0	0
SAL_WA1251AA	130510	Agama	0	0	0	0	0	0	0	0	0	0
SAL_WA1549AA	77287	Agama	0	0	0	0	0	0	0	0	0	0
SAL_WA1504AA	122283	Agama	0	0	0	0	0	0	0	0	0	0
SAL_WA1695AA	107222	Agama	0	0	0	0	0	0	0	0	0	0
SAL_WA2085AA		Agama	0	0	0	0	0	0	0	0	0	0
SAL_WA2087AA		Agama	0	0	0	0	0	0	0	0	0	0
SAL_WA2088AA		Agama	0	0	0	0	0	0	0	0	0	0
SAL_WA2092AA		Agama	0	0	0	0	0	0	0	0	0	0
SAL_WA2093AA		Agama	0	0	0	0	0	0	0	0	0	0
SAL_WA2089AA		Agama	0	0	0	0	0	0	0	0	0	0
SAL_WA2090AA		Agama	0	0	0	0	0	0	0	0	0	0
SAL_QA9282AA	542092	Agege	0	0	0	0	0	0	0	0	0	0
SAL_EA7790AA	29244	Ago	0	0	0	0	0	0	0	0	0	0
SAL_DA1427AA	H124140699	Ago	0	0	0	0	0	0	0	0	0	0
SAL_EA7285AA	20907	Ago	0	0	0	0	0	0	0	0	0	0
SAL_DA6082AA	MOD1_Sal_834	Ago	0	0	0	0	0	0	0	0	0	0
SAL_DA1429AA	H122180361	Ago	0	0	0	0	0	0	0	0	0	0
SAL_DA1351AA	H123740559	Ago	0	0	0	0	0	0	0	0	0	0
SAL_CA2179AA	98258	Ago	0	0	0	0	0	0	0	0	0	0
SAL_CA2117AA	36510	Ago	0	0	0	0	0	0	0	0	0	0
SAL_HA5582AA	L00592-14	Ago	0	0	0	0	0	0	0	0	0	0
SAL_IA5378AA	13L-4103	Ago	0	0	0	0	0	0	0	0	0	0
SAL_CA4099AA	3766	Ago	0	0	0	0	0	0	0	0	0	0
SAL_CA3654AA	46006	Ago	0	0	0	0	0	0	0	0	0	0
SAL_FA7151AA	PNUSAS002182	Ago	0	0	0	0	0	0	0	0	0	0
SAL_EA7230AA	43900	Ago	0	0	0	0	0	0	0	0	0	0
SAL_KA0841AA	PNUSAS016176	Ago	0	0	0	0	0	0	0	0	0	0
SAL_OA0212AA	PNUSAS028134	Ago	0	0	0	0	0	0	0	0	0	0
SAL_PA6407AA	432262	Ago	0	0	0	0	0	0	0	0	0	0
SAL_QA2107AA	255540	Ago	0	0	0	0	0	0	0	0	0	0
SAL_QA5264AA	130500	Ago	0	0	0	0	0	0	0	0	0	0
SAL_RA3430AA	223595	Ago	0	0	0	0	0	0	0	0	0	0
SAL_UA8686AA	669747	Ago	0	0	0	0	0	0	0	0	0	0
SAL_VA0733AA	513497	Ago	0	0	0	0	0	0	0	0	0	0
SAL_WA1248AA	129375	Ago	0	0	0	0	0	0	0	0	0	0
SAL_EA7903AA	15224	Agona	0	0	0	0	0	0	0	0	0	0
SAL_EA7459AA	21798	Agona	0	0	0	0	0	0	0	0	0	0
SAL_EA7594AA	97249	Agona	0	0	0	0	0	0	0	0	0	0
SAL_BA3435AA	FDA00004332	Agona	0	0	0	0	0	0	0	0	0	0
SAL_FA5822AA	FDA00001231	Agona	0	0	0	0	0	0	0	0	0	0
SAL_FA5754AA	FSIS1606299	Agona	0	0	0	0	0	0	0	0	0	0
SAL_FA5693AA	FDA00002574	Agona	0	0	0	0	0	0	0	0	0	0
SAL_FA5388AA	392869-2	Agona	0	0	0	0	0	0	0	0	0	0
SAL_FA5230AA	IEH-NGS-SAL-04028	Agona	0	0	0	0	0	0	0	0	0	0
SAL_FA5229AA	IEH-NGS-SAL-04029	Agona	0	0	0	0	0	0	0	0	0	0
SAL_FA5221AA	CFSAN047694	Agona	0	0	0	0	0	0	0	0	0	0
SAL_FA4984AA	FDA00003236	Agona	0	0	0	0	0	0	0	0	0	0
SAL_FA4985AA	FDA00003237	Agona	0	0	0	0	0	0	0	0	0	0
SAL_FA4830AA	CVM-N27374	Agona	0	0	0	0	0	0	0	0	0	0
SAL_FA4811AA	CVM-N27376	Agona	0	0	0	0	0	0	0	0	0	0
SAL_FA4787AA	CFSAN032034	Agona	0	0	0	0	0	0	0	0	0	0
SAL_FA4782AA	CFSAN032035	Agona	0	0	0	0	0	0	0	0	0	0
SAL_FA4765AA	FSIS1606070	Agona	0	0	0	0	0	0	0	0	0	0
SAL_FA4660AA	170452	Agona	0	0	0	0	0	0	0	0	0	0
SAL_FA3994AA	178641	Agona	0	0	0	0	0	0	0	0	0	0
SAL_FA3845AA	121468	Agona	0	0	0	0	0	0	0	0	0	0
SAL_FA3722AA	106524	Agona	0	0	0	0	0	0	0	0	0	0
SAL_FA3252AA	FSIS1606026	Agona	0	0	0	0	0	0	0	0	0	0
SAL_FA2970AA	CVM N56981F	Agona	0	0	0	0	0	0	0	0	0	0
SAL_FA2982AA	CVM N56947F	Agona	0	0	0	0	0	0	0	0	0	0
SAL_FA2956AA	CVM N57049F	Agona	0	0	0	0	0	0	0	0	0	0
SAL_FA2949AA	CVM N57063F	Agona	0	0	0	0	0	0	0	0	0	0
SAL_FA2938AA	CVM N57105F	Agona	0	0	0	0	0	0	0	0	0	0
SAL_FA2919AA	CVM N57174F	Agona	0	0	0	0	0	0	0	0	0	0
SAL_FA2903AA	CVM N57217F	Agona	0	0	0	0	0	0	0	0	0	0
SAL_FA2893AA	CVM N57246F	Agona	0	0	0	0	0	0	0	0	0	0
SAL_FA2897AA	CVM N57232F	Agona	0	0	0	0	0	0	0	0	0	0
SAL_FA2882AA	CVM N57287F	Agona	0	0	0	0	0	0	0	0	0	0
SAL_FA2872AA	CVM N57326F	Agona	0	0	0	0	0	0	0	0	0	0
SAL_FA2867AA	CVM N57338F	Agona	0	0	0	0	0	0	0	0	0	0
SAL_FA2864AA	CVM N57346F	Agona	0	0	0	0	0	0	0	0	0	0
SAL_FA2790AA	CVM N57608F	Agona	0	0	0	0	0	0	0	0	0	0

34-4-8-44-3-0	4	0.000111123
34-4-8-52-3-0	2	5.55617E-05
46-3-33-0-3-0	1	2.77809E-05
18-3-7-234-3-0	1	2.77809E-05
0-0-79-0-3-0	1	2.77809E-05
5-4-8-417-0-0	1	2.77809E-05
5-4-205-75-0-0	1	2.77809E-05
1-1-1-418-1-3	1	2.77809E-05
1-1-1-26-1-3	2	5.55617E-05
5-4-75-8-0-0	1	2.77809E-05
18-3-5-12-3-0	1	2.77809E-05
0-0-0-419-15-0	1	2.77809E-05
18-3-7-138-3-0	2	5.55617E-05
0-4-50-9-0-0	2	5.55617E-05
0-0-0-0-82-0	5	0.000138904
0-3-0-117-3-0	2	5.55617E-05
0-3-0-420-3-0	1	2.77809E-05
18-3-5-85-3-0	1	2.77809E-05
0-0-0-49-82-0	1	2.77809E-05
81-3-33-9-0-0	2	5.55617E-05
18-3-7-244-3-0	1	2.77809E-05
18-3-0-421-3-0	1	2.77809E-05
0-0-8-422-3-0	1	2.77809E-05
5-4-84-9-0-0	1	2.77809E-05
5-4-206-9-0-0	3	8.33426E-05
0-3-0-423-3-0	3	8.33426E-05
0-3-0-424-3-0	1	2.77809E-05
0-3-0-276-3-0	2	5.55617E-05
0-0-8-155-3-0	2	5.55617E-05
16-3-152-0-0-0	1	2.77809E-05
0-3-7-342-3-0	1	2.77809E-05
0-3-7-106-3-0	1	2.77809E-05
0-3-0-163-3-0	2	5.55617E-05
5-4-209-119-0-0	1	2.77809E-05
18-3-5-49-3-0	1	2.77809E-05
0-50-0-73-3-0	1	2.77809E-05
0-50-0-0-3-0	1	2.77809E-05
0-0-0-410-3-0	1	2.77809E-05
18-3-0-6-3-0	1	2.77809E-05
38-0-7-9-0-0	1	2.77809E-05
79-3-5-353-3-0	1	2.77809E-05
0-0-53-9-3-0	1	2.77809E-05
0-3-62-159-3-0	1	2.77809E-05
0-3-7-340-3-0	1	2.77809E-05
0-3-0-341-3-0	1	2.77809E-05
0-3-0-351-3-0	1	2.77809E-05
0-0-75-261-15-0	1	2.77809E-05
0-3-185-9-3-0	1	2.77809E-05
0-3-62-259-3-0	1	2.77809E-05
0-3-44-43-3-0	1	2.77809E-05
0-3-62-112-3-0	1	2.77809E-05
0-0-8-348-3-0	1	2.77809E-05
0-0-43-249-3-0	1	2.77809E-05
0-3-86-244-3-0	1	2.77809E-05
0-3-5-352-3-0	1	2.77809E-05
18-3-7-215-3-0	1	2.77809E-05
0-3-62-51-3-0	1	2.77809E-05
0-0-8-368-3-0	1	2.77809E-05
18-3-67-75-3-0	1	2.77809E-05
5-4-29-371-0-0	1	2.77809E-05
0-0-0-0-67-0	1	2.77809E-05
0-3-0-259-24-0	1	2.77809E-05
16-3-188-9-0-0	1	2.77809E-05
0-3-114-0-3-0	1	2.77809E-05
0-3-0-372-3-0	1	2.77809E-05
18-3-33-51-3-0	2	5.55617E-05
0-0-0-0-84-0	1	2.77809E-05
0-3-0-430-3-0	1	2.77809E-05
0-0-8-432-3-0	1	2.77809E-05
0-3-0-433-3-0	1	2.77809E-05
5-4-212-9-0-0	1	2.77809E-05
0-3-0-84-3-0	1	2.77809E-05
0-86-0-0-3-0	1	2.77809E-05

SAL_FA2776AA	CVM N57640F	Agona	0	0	0	0	0	0	0	0	0	0	0			0-3-33-433-3-0	1	2.77809E-05
SAL_FA2767AA	CVM N57663F	Agona	0	0	0	0	0	0	0	0	0	0	0			18-3-7-79-3-0	2	5.55617E-05
SAL_FA2753AA	CVM N57703F	Agona	0	0	0	0	0	0	0	0	0	0	0			0-3-0-434-3-0	1	2.77809E-05
SAL_FA2756AA	CVM N57696F	Agona	0	0	0	0	0	0	0	0	0	0	0			18-3-7-259-3-0	1	2.77809E-05
SAL_FA2735AA	CVM N57754F	Agona	0	0	0	0	0	0	0	0	0	0	0			0-3-0-436-3-0	1	2.77809E-05
SAL_FA2717AA	CVM N57814F	Agona	0	0	0	0	0	0	0	0	0	0	0			0-0-0-74-3-0	1	2.77809E-05
SAL_FA2716AA	CVM N57816F	Agona	0	0	0	0	0	0	0	0	0	0	0			0-0-213-49-3-0	1	2.77809E-05
SAL_FA2709AA	CVM N57835F	Agona	0	0	0	0	0	0	0	0	0	0	0			0-3-62-437-3-0	1	2.77809E-05
SAL_FA2654AA	HIY0151	Agona	0	0	0	0	0	0	0	0	0	0	0			0-3-5-138-3-0	1	2.77809E-05
SAL_FA2641AA	CVM-N26925	Agona	0	0	0	0	0	0	0	0	0	0	0			0-0-214-72-3-0	1	2.77809E-05
SAL_FA2517AA	FDA00002537	Agona	0	0	0	0	0	0	0	0	0	0	0			0-3-46-282-3-0	1	2.77809E-05
SAL_FA2421AA	PNUSAS001733	Agona	0	0	0	0	0	0	0	0	0	0	0			0-3-5-225-3-0	1	2.77809E-05
SAL_FA2411AA	PNUSAS001756	Agona	0	0	0	0	0	0	0	0	0	0	0			0-3-5-97-85-0	1	2.77809E-05
SAL_FA2222AA	CVM-N19767	Agona	0	0	0	0	0	0	0	0	0	0	0			0-3-63-89-3-0	1	2.77809E-05
SAL_FA1602AA	CVM-N26612	Agona	0	0	0	0	0	0	0	0	0	0	0			18-3-48-72-3-0	1	2.77809E-05
SAL_FA1486AA	Derby	Agona	0	0	0	0	0	0	0	0	0	0	0			0-3-199-79-3-0	1	2.77809E-05
SAL_FA1430AA	FDA00004021	Agona	0	0	0	0	0	0	0	0	0	0	0			0-87-7-9-3-0	1	2.77809E-05
SAL_FA1429AA	FDA00004020	Agona	0	0	0	0	0	0	0	0	0	0	0			97-3-5-9-0-0	1	2.77809E-05
SAL_FA1284AA	1601F29331	Agona	0	0	0	0	0	0	0	0	0	0	0			0-0-50-111-3-0	1	2.77809E-05
SAL_FA1129AA	CVM-N26097	Agona	0	0	0	0	0	0	0	0	0	0	0			0-3-44-89-3-0	1	2.77809E-05
SAL_FA1128AA	CVM-N26098	Agona	0	0	0	0	0	0	0	0	0	0	0			0-3-218-51-3-0	1	2.77809E-05
SAL_FA1114AA	CVM-N26610	Agona	0	0	0	0	0	0	0	0	0	0	0			0-3-63-12-3-0	1	2.77809E-05
SAL_FA1082AA	PNUSAS001540	Agona	0	0	0	0	0	0	0	0	0	0	0			74-3-0-0-3-0	1	2.77809E-05
SAL_FA1103AA	PNUSAS001578	Agona	0	0	0	0	0	0	0	0	0	0	0			0-31-0-8-3-0	1	2.77809E-05
SAL_FA1081AA	PNUSAS001541	Agona	0	0	0	0	0	0	0	0	0	0	0			0-31-0-249-3-0	1	2.77809E-05
SAL_FA1083AA	PNUSAS001539	Agona	0	0	0	0	0	0	0	0	0	0	0			0-3-62-115-3-0	1	2.77809E-05
SAL_FA0723AA	HIY0163	Agona	0	0	0	0	0	0	0	0	0	0	0			0-3-44-12-3-0	1	2.77809E-05
SAL_FA0648AA	FDA00001289	Agona	0	0	0	0	0	0	0	0	0	0	0			0-3-5-228-3-0	1	2.77809E-05
SAL_FA0574AA	CFSAN045554	Agona	0	0	0	0	0	0	0	0	0	0	0			0-3-62-9-3-0	1	2.77809E-05
SAL_FA0571AA	CFSAN045557	Agona	0	0	0	0	0	0	0	0	0	0	0			0-0-50-155-3-0	1	2.77809E-05
SAL_FA0390AA	FSIS1605544	Agona	0	0	0	0	0	0	0	0	0	0	0			0-0-0-266-3-0	1	2.77809E-05
SAL_EA9914AA	2011K-0646	Agona	0	0	0	0	0	0	0	0	0	0	0			0-3-44-76-3-0	1	2.77809E-05
SAL_EA9766AA	24249	Agona	0	0	0	0	0	0	0	0	0	0	0			0-80-0-0-3-0	1	2.77809E-05
SAL_EA9564AA	FSIS1605678	Agona	0	0	0	0	0	0	0	0	0	0	0			16-3-53-0-3-0	1	2.77809E-05
SAL_EA9494AA	CFSAN045580	Agona	0	0	0	0	0	0	0	0	0	0	0			0-0-0-445-3-0	1	2.77809E-05
SAL_EA9285AA	NY-N20038	Agona	0	0	0	0	0	0	0	0	0	0	0			18-3-7-111-3-0	1	2.77809E-05
SAL_EA9079AA	NY-N20055	Agona	0	0	0	0	0	0	0	0	0	0	0			0-0-224-119-3-0	1	2.77809E-05
SAL_EA8850AA	CVM-N23746	Agona	0	0	0	0	0	0	0	0	0	0	0			0-3-48-51-3-0	2	5.55617E-05
SAL_EA8730AA	NY-N19987	Agona	0	0	0	0	0	0	0	0	0	0	0			18-3-114-51-21-0	1	2.77809E-05
SAL_EA8704AA	PNUSAS001362	Agona	0	0	0	0	0	0	0	0	0	0	0			0-3-0-111-3-0	1	2.77809E-05
SAL_EA8493AA	CVM-N23769	Agona	0	0	0	0	0	0	0	0	0	0	0			0-0-119-13-3-0	1	2.77809E-05
SAL_EA8476AA	FSIS1504474	Agona	0	0	0	0	0	0	0	0	0	0	0			0-3-7-381-3-0	1	2.77809E-05
SAL_EA8396AA	ADRDL-15-15886	Agona	0	0	0	0	0	0	0	0	0	0	0			0-3-46-73-3-0	1	2.77809E-05
SAL_EA8375AA	FSIS1504600	Agona	0	0	0	0	0	0	0	0	0	0	0			0-3-86-73-3-0	1	2.77809E-05
SAL_EA8400AA	ADRDL-15-15175	Agona	0	0	0	0	0	0	0	0	0	0	0			0-3-7-235-3-0	2	5.55617E-05
SAL_EA8102AA	5972	Agona	0	0	0	0	0	0	0	0	0	0	0			0-0-49-75-3-0	3	8.33426E-05
SAL_EA8098AA	15231	Agona	0	0	0	0	0	0	0	0	0	0	0			0-0-8-175-3-0	1	2.77809E-05
SAL_EA7732AA	95029	Agona	0	0	0	0	0	0	0	0	0	0	0			0-3-0-448-3-0	1	2.77809E-05
SAL_EA7700AA	53030	Agona	0	0	0	0	0	0	0	0	0	0	0			0-3-7-449-3-0	1	2.77809E-05
SAL_EA7559AA	43953	Agona	0	0	0	0	0	0	0	0	0	0	0			0-0-0-236-0-0	1	2.77809E-05
SAL_EA7456AA	45226	Agona	0	0	0	0	0	0	0	0	0	0	0			0-0-0-450-3-0	1	2.77809E-05
SAL_EA7445AA	29270	Agona	0	0	0	0	0	0	0	0	0	0	0			0-0-5-183-3-0	1	2.77809E-05
SAL_EA7435AA	21071	Agona	0	0	0	0	0	0	0	0	0	0	0		**Enugu**	Enugu 0-0-0-0-0-0	3	/
SAL_EA7338AA	58557	Agona	0	0	0	0	0	0	0	0	0	0	0		**Eppendorf**	Eppendorf 2-2-3-3-0-0	1	1
SAL_EA7313AA	29938	Agona	0	0	0	0	0	0	0	0	0	0	0		**Escanaba**	Escanaba 0-0-0-0-0-0	2	/
SAL_EA7179AA	97238	Agona	0	0	0	0	0	0	0	0	0	0	0		**Everleigh**	Everleigh 0-0-0-0-0-0	1	/
SAL_EA7151AA	6494	Agona	0	0	0	0	0	0	0	0	0	0	0		**Farakan**	Farakan 0-0-0-0-0-0	1	/
SAL_EA7149AA	43922	Agona	0	0	0	0	0	0	0	0	0	0	0		**Fillmore**	Fillmore 0-0-0-0-0-0	1	/
SAL_EA6941AA	29239	Agona	0	0	0	0	0	0	0	0	0	0	0		**Fischerstrasse**	Fischerstrasse 0-0-0-0-0-0	3	/
SAL_EA6868AA	CVM-N23744	Agona	0	0	0	0	0	0	0	0	0	0	0		**Florida**	Florida 0-0-0-0-0-0	44	/
SAL_EA6480AA	FSIS1504192	Agona	0	0	0	0	0	0	0	0	0	0	0		**Fluntern**	Fluntern 0-0-0-0-0-0	43	/
SAL_EA6462AA	FDA00002175	Agona	0	0	0	0	0	0	0	0	0	0	0		**Fomeco**	Fomeco 0-0-0-0-0-0	2	/
SAL_EA6379AA	FSIS1503434	Agona	0	0	0	0	0	0	0	0	0	0	0		**Freetown**	Freetown 0-0-0-0-0-0	10	/
SAL_EA6366AA	FSIS1503916	Agona	0	0	0	0	0	0	0	0	0	0	0		**Fresno**	Fresno 0-0-0-0-0-0	89	/
SAL_EA6132AA	FSIS1501287	Agona	0	0	0	0	0	0	0	0	0	0	0		**Fulica**	Fulica 0-0-0-0-0-0	45	/
SAL_EA6138AA	FSIS1501533	Agona	0	0	0	0	0	0	0	0	0	0	0		**Gafsa**	Gafsa 0-0-0-0-0-0	2	/
SAL_EA5961AA	ADRDL-15-8995	Agona	0	0	0	0	0	0	0	0	0	0	0			5-4-9-15-0-0	55	0.185185185
SAL_EA5958AA	ADRDL-15-1091	Agona	0	0	0	0	0	0	0	0	0	0	0			5-4-6-8-0-0	85	0.286195286
SAL_EA5959AA	ADRDL-15-6557	Agona	0	0	0	0	0	0	0	0	0	0	0			5-4-6-53-0-0	37	0.124579125
SAL_EA5952AA	ADRDL-15-1869	Agona	0	0	0	0	0	0	0	0	0	0	0			5-4-96-15-0-0	1	0.003367003
SAL_EA5888AA	CFSAN031478	Agona	0	0	0	0	0	0	0	0	0	0	0			5-4-98-15-0-0	1	0.003367003
SAL_EA5506AA	FSIS1504922	Agona	0	0	0	0	0	0	0	0	0	0	0			5-4-106-8-0-0	1	0.003367003
SAL_EA5495AA	ADRDL-15-5759	Agona	0	0	0	0	0	0	0	0	0	0	0			5-4-6-218-0-0	93	0.313131313

SAL_EA5423AA	CFSAN031500	Agona	0	0	0	0	0	0	0	0	0	0	0	Gallinarum	5-4-29-218-0-0	4	0.013468013
SAL_EA5330AA	FSIS1503818	Agona	0	0	0	0	0	0	0	0	0	0	0		5-4-20-240-0-0	2	0.006734007
SAL_EA4971AA	ADRDL-14-17423	Agona	0	0	0	0	0	0	0	0	0	0	0		5-4-6-243-0-0	8	0.026936027
SAL_EA4065AA	ADRDL-LA-56-2014	Agona	0	0	0	0	0	0	0	0	0	0	0		5-54-20-8-0-0	4	0.013468013
SAL_EA4066AA	ADRDL-LA-55-2014	Agona	0	0	0	0	0	0	0	0	0	0	0		5-4-122-8-0-0	1	0.003367003
SAL_EA4062AA	ADRDL-LA-59-2014	Agona	0	0	0	0	0	0	0	0	0	0	0		5-4-9-102-0-0	2	0.006734007
SAL_EA4056AA	ADRDL-LA-65-2014	Agona	0	0	0	0	0	0	0	0	0	0	0		16-3-7-315-3-0	1	0.003367003
SAL_EA4054AA	ADRDL-LA-67-2014	Agona	0	0	0	0	0	0	0	0	0	0	0		5-4-6-0-0-0	1	0.003367003
SAL_EA4061AA	ADRDL-LA-60-2014	Agona	0	0	0	0	0	0	0	0	0	0	0		5-4-207-53-0-0	1	0.000333667
SAL_EA3969AA	FDA00009577	Agona	0	0	0	0	0	0	0	0	0	0	0	Gamaba	Gamaba 0-0-0-0-0-0	3	/
SAL_EA3856AA	CVM N43827	Agona	0	0	0	0	0	0	0	0	0	0	0	Gambia	Gambia 0-0-0-0-0-0	7	/
SAL_EA3681AA	CVM N48683	Agona	0	0	0	0	0	0	0	0	0	0	0	Gaminara	Gaminara 0-0-0-0-0-0	340	/
SAL_EA3151AA	CVM-N15242	Agona	0	0	0	0	0	0	0	0	0	0	0	Gateshead	Gateshead 0-0-0-0-0-0	1	/
SAL_EA2990AA	VA-WGS-00574	Agona	0	0	0	0	0	0	0	0	0	0	0	Give	G e 0-0-0-0-0-0	804	0.998757764
SAL_EA2982AA	CVM-N16436	Agona	0	0	0	0	0	0	0	0	0	0	0		0-0-82-0-0-0	1	0.001242236
SAL_EA2907AA	FDA00005046	Agona	0	0	0	0	0	0	0	0	0	0	0	Glostrup	Glostrup 0-0-0-0-0-0	59	/
SAL_EA2639AA	WAPHL_SAL-A00853	Agona	0	0	0	0	0	0	0	0	0	0	0	Gloucester	Gloucester 0-0-0-0-0-0	4	/
SAL_EA2545AA	CVM-N15243	Agona	0	0	0	0	0	0	0	0	0	0	0	Goettingen	Goettingen 0-0-0-0-0-0	10	/
SAL_EA2492AA	ADRDL-LA-93-2014	Agona	0	0	0	0	0	0	0	0	0	0	0	Gokul	Gokul 0-0-0-0-0-0	1	/
SAL_EA2495AA	ADRDL-LA-90-2014	Agona	0	0	0	0	0	0	0	0	0	0	0	Goldcoast	Goldcoast 0-0-0-0-0-0	159	/
SAL_EA2487AA	ADRDL-LA-31-2014	Agona	0	0	0	0	0	0	0	0	0	0	0	Gombe	Gombe 0-0-0-0-0-0	2	/
SAL_EA2455AA	CFSAN031420	Agona	0	0	0	0	0	0	0	0	0	0	0	Goulfey	Goulfey 0-0-0-0-0-0	1	/
SAL_EA2459AA	FDA00001951	Agona	0	0	0	0	0	0	0	0	0	0	0	Gozo	Gozo 0-0-0-0-0-0	1	/
SAL_EA2454AA	CFSAN031421	Agona	0	0	0	0	0	0	0	0	0	0	0	Grumpensis	Grumpensis 0-0-0-0-0-0	25	/
SAL_EA2341AA	VA-WGS-00620	Agona	0	0	0	0	0	0	0	0	0	0	0	Guildford	Guildford 0-0-0-0-0-0	8	/
SAL_EA1008AA	1-sc-2013-02-28T11:35:1	Agona	0	0	0	0	0	0	0	0	0	0	0	Guinea	Guinea 0-0-0-0-0-0	19	/
SAL_EA1007AA	1-sc-2013-02-28T11:35:1	Agona	0	0	0	0	0	0	0	0	0	0	0	Haardt	Haardt 0-0-0-0-0-0	6	/
SAL_EA0741AA	CFSAN006264	Agona	0	0	0	0	0	0	0	0	0	0	0	Hadar	Hadar 0-0-0-0-0-0	1690	0.999408634
SAL_EA0747AA	CFSAN006263	Agona	0	0	0	0	0	0	0	0	0	0	0		18-3-114-47-3-0	1	0.000591366
SAL_EA0759AA	CFSAN006265	Agona	0	0	0	0	0	0	0	0	0	0	0	Haduna	Haduna 0-0-0-0-0-0	3	/
SAL_EA0748AA	CFSAN006256	Agona	0	0	0	0	0	0	0	0	0	0	0	Haga	Haga 0-0-0-0-0-0	1	/
SAL_EA0722AA	CFSAN006266	Agona	0	0	0	0	0	0	0	0	0	0	0	Haifa	Haifa 0-0-0-0-0-0	187	/
SAL_EA0733AA	CFSAN006257	Agona	0	0	0	0	0	0	0	0	0	0	0	Halle	Halle 0-0-0-0-0-0	8	/
SAL_EA0720AA	CFSAN006258	Agona	0	0	0	0	0	0	0	0	0	0	0	Handen	Handen 0-0-0-0-0-0	1	/
SAL_EA0702AA	CFSAN006259	Agona	0	0	0	0	0	0	0	0	0	0	0	Hartford	Hartford 0-0-0-0-0-0	465	/
SAL_EA0667AA	CFSAN006262	Agona	0	0	0	0	0	0	0	0	0	0	0	Havana	Havana 0-0-0-0-0-0	362	/
SAL_EA0687AA	CFSAN006255	Agona	0	0	0	0	0	0	0	0	0	0	0	Heidelberg	0-0-0-0-0-0	4527	0.999779152
SAL_EA0510AA	VA_WGS-00010	Agona	0	0	0	0	0	0	0	0	0	0	0		1-1-1-1-1-1	1	0.000220848
SAL_EA0509AA	VA_WGS-00011	Agona	0	0	0	0	0	0	0	0	0	0	0	Hermannswerder	0-0-0-0-0-0	9	/
SAL_EA0505AA	VA_WGS-00012	Agona	0	0	0	0	0	0	0	0	0	0	0	Heron	0-0-0-0-0-0	6	/
SAL_EA0264AA	MDH-2013-00129	Agona	0	0	0	0	0	0	0	0	0	0	0	Herston	0-0-0-0-0-0	20	/
SAL_EA0224AA	FNE0010	Agona	0	0	0	0	0	0	0	0	0	0	0	Hessarek	0-0-0-0-0-0	15	/
SAL_EA0212AA	FSF0010	Agona	0	0	0	0	0	0	0	0	0	0	0	Hiduddify	0-0-0-0-0-0	1	/
SAL_EA0181AA	NY_IDR1000037973	Agona	0	0	0	0	0	0	0	0	0	0	0	Hissar	3-0-0-0-0-0	1	1
SAL_EA0114AA	WAPHL_SAL-A00120	Agona	0	0	0	0	0	0	0	0	0	0	0	Hofit	0-0-0-0-0-0	6	/
SAL_EA0055AA	FL_EPI-005	Agona	0	0	0	0	0	0	0	0	0	0	0	Hoghton	0-0-0-0-0-0	1	/
SAL_EA0005AA	FAR0034	Agona	0	0	0	0	0	0	0	0	0	0	0	Holcomb	0-0-0-0-0-0	40	/
SAL_DA9898AA	FAR0035	Agona	0	0	0	0	0	0	0	0	0	0	0	Horsham	0-0-0-0-0-0	12	/
SAL_DA9837AA	FAR0041	Agona	0	0	0	0	0	0	0	0	0	0	0	Huettwilen	0-0-0-0-0-0	1	/
SAL_DA9729AA	VA_WGS-00054	Agona	0	0	0	0	0	0	0	0	0	0	0	Hull	0-0-0-0-0-0	30	/
SAL_DA9704AA	VA_WGS-00053	Agona	0	0	0	0	0	0	0	0	0	0	0	Huvudsta	0-0-0-0-0-0	1	/
SAL_DA9630AA	FMA0054	Agona	0	0	0	0	0	0	0	0	0	0	0	Hvittingfoss	0-0-0-0-0-0	470	0.997876858
SAL_DA9534AA	NY_IDR1100001463	Agona	0	0	0	0	0	0	0	0	0	0	0		0-0-5-0-3-0	1	0.002123142
SAL_DA9421AA	VA_WGS-00127	Agona	0	0	0	0	0	0	0	0	0	0	0	I 4,[5],12:i:-	1-1-1-4-1-1	1201	0.769871795
SAL_DA9333AA	FNW19H73	Agona	0	0	0	0	0	0	0	0	0	0	0		1-1-1-91-1-1	1	0.000641026
SAL_DA9147AA	WAPHL-SAL-A00251	Agona	0	0	0	0	0	0	0	0	0	0	0		0-0-0-0-0-0	85	0.054487179
SAL_DA9121AA	NY_IDR1100017859	Agona	0	0	0	0	0	0	0	0	0	0	0		1-1-1-4-1-15	1	0.000641026
SAL_DA9075AA	VA_WGS-00016	Agona	0	0	0	0	0	0	0	0	0	0	0		1-1-1-4-1-20	2	0.001282051
SAL_DA9069AA	VA_WGS-00015	Agona	0	0	0	0	0	0	0	0	0	0	0		1-1-1-0-1-1	204	0.130769231
SAL_DA9058AA	VA_WGS-00013	Agona	0	0	0	0	0	0	0	0	0	0	0		0-0-0-0-1-1	1	0.000641026
SAL_DA9006AA	VA_WGS-00014	Agona	0	0	0	0	0	0	0	0	0	0	0		0-0-0-0-0-1	2	0.001282051
SAL_DA8770AA	MDH-2014-00190	Agona	0	0	0	0	0	0	0	0	0	0	0		1-1-80-4-1-1	1	0.000641026
SAL_DA8560AA	VA_WGS-00146	Agona	0	0	0	0	0	0	0	0	0	0	0		1-1-1-0-0-0	2	0.001282051
SAL_DA8555AA	FSE0029	Agona	0	0	0	0	0	0	0	0	0	0	0		39-1-1-4-1-1	1	0.000641026
SAL_DA8519AA	VA_WGS-00156	Agona	0	0	0	0	0	0	0	0	0	0	0		1-1-0-4-1-1	7	0.004487179
SAL_DA8502AA	FSE0023	Agona	0	0	0	0	0	0	0	0	0	0	0		1-1-1-4-1-28	1	0.000641026
SAL_DA8413AA	VA_WGS-00165	Agona	0	0	0	0	0	0	0	0	0	0	0		1-1-1-16-1-1	13	0.008333333
SAL_DA8030AA	FSW0114	Agona	0	0	0	0	0	0	0	0	0	0	0		1-1-110-4-1-1	1	0.000641026
SAL_DA7847AA	FAR0126	Agona	0	0	0	0	0	0	0	0	0	0	0		1-1-118-4-1-1	3	0.001923077
SAL_DA7524AA	MDH-2014-00085	Agona	0	0	0	0	0	0	0	0	0	0	0		1-1-1-4-0-0	10	0.006410256
SAL_DA7407AA	MDH-2014-00073	Agona	0	0	0	0	0	0	0	0	0	0	0		1-1-150-4-0-1	1	0.000641026
SAL_DA7331AA	FSW0184	Agona	0	0	0	0	0	0	0	0	0	0	0		1-1-150-0-1-1	1	0.000641026
SAL_DA7329AA	FSW0161	Agona	0	0	0	0	0	0	0	0	0	0	0		1-1-1-292-1-1	1	0.000641026

SAL_DA7325AA	WAPHL-SAL-A00374	Agona	0	0	0	0	0	0	0	0	0	0	0
SAL_DA7304AA	FLUFL-1101	Agona	0	0	0	0	0	0	0	0	0	0	0
SAL_DA7239AA	FMA0143	Agona	0	0	0	0	0	0	0	0	0	0	0
SAL_DA7225AA	FMA0153	Agona	0	0	0	0	0	0	0	0	0	0	0
SAL_DA7189AA	CFSAN012629	Agona	0	0	0	0	0	0	0	0	0	0	0
SAL_DA7144AA	CFSAN012645	Agona	0	0	0	0	0	0	0	0	0	0	0
SAL_DA7063AA	MDH-2014-00029	Agona	0	0	0	0	0	0	0	0	0	0	0
SAL_DA7040AA	MDH-2014-00020	Agona	0	0	0	0	0	0	0	0	0	0	0
SAL_DA6576AA	FL-NRM096	Agona	0	0	0	0	0	0	0	0	0	0	0
SAL_DA6417AA	MDH-2014-00296	Agona	0	0	0	0	0	0	0	0	0	0	0
SAL_DA6370AA	FLDOH-8	Agona	0	0	0	0	0	0	0	0	0	0	0
SAL_DA6279AA	MDH-2014-00138	Agona	0	0	0	0	0	0	0	0	0	0	0
SAL_DA6199AA	MDH-2014-00319	Agona	0	0	0	0	0	0	0	0	0	0	0
SAL_DA6175AA	MDH-2014-00323	Agona	0	0	0	0	0	0	0	0	0	0	0
SAL_DA6174AA	MDH-2014-00331	Agona	0	0	0	0	0	0	0	0	0	0	0
SAL_DA6142AA	MDH-2014-00336	Agona	0	0	0	0	0	0	0	0	0	0	0
SAL_DA6145AA	MDH-2014-00344	Agona	0	0	0	0	0	0	0	0	0	0	0
SAL_DA6041AA	CFSAN018526	Agona	0	0	0	0	0	0	0	0	0	0	0
SAL_DA5968AA	MDH-2014-00355	Agona	0	0	0	0	0	0	0	0	0	0	0
SAL_DA5773AA	IEH-NGS-SAL-00753	Agona	0	0	0	0	0	0	0	0	0	0	0
SAL_DA5624AA	FDA00004025	Agona	0	0	0	0	0	0	0	0	0	0	0
SAL_DA5623AA	FDA00004026	Agona	0	0	0	0	0	0	0	0	0	0	0
SAL_DA5622AA	FDA00004027	Agona	0	0	0	0	0	0	0	0	0	0	0
SAL_DA5510AA	WAPHL_SAL-A00469	Agona	0	0	0	0	0	0	0	0	0	0	0
SAL_DA5411AA	WAPHL_SAL-A00477	Agona	0	0	0	0	0	0	0	0	0	0	0
SAL_DA5412AA	WAPHL_SAL-A00476	Agona	0	0	0	0	0	0	0	0	0	0	0
SAL_DA5067AA	WAPHL_SAL-A00430	Agona	0	0	0	0	0	0	0	0	0	0	0
SAL_DA5038AA	FNE0005	Agona	0	0	0	0	0	0	0	0	0	0	0
SAL_DA4761AA	I308	Agona	0	0	0	0	0	0	0	0	0	0	0
SAL_DA4319AA	MOD1-Sal_891	Agona	0	0	0	0	0	0	0	0	0	0	0
SAL_DA4313AA	MOD1-Sal_890	Agona	0	0	0	0	0	0	0	0	0	0	0
SAL_DA4312AA	MOD1-Sal_892	Agona	0	0	0	0	0	0	0	0	0	0	0
SAL_DA4269AA	MOD1-Sal_889	Agona	0	0	0	0	0	0	0	0	0	0	0
SAL_DA4091AA	WAPHL_SAL-A00509	Agona	0	0	0	0	0	0	0	0	0	0	0
SAL_DA4071AA	WAPHL_SAL-A00510	Agona	0	0	0	0	0	0	0	0	0	0	0
SAL_DA3968AA	FNE0146	Agona	0	0	0	0	0	0	0	0	0	0	0
SAL_DA3726AA	CFSAN008683	Agona	0	0	0	0	0	0	0	0	0	0	0
SAL_DA3690AA	CFSAN008682	Agona	0	0	0	0	0	0	0	0	0	0	0
SAL_DA3681AA	CFSAN008680	Agona	0	0	0	0	0	0	0	0	0	0	0
SAL_DA3670AA	CFSAN008681	Agona	0	0	0	0	0	0	0	0	0	0	0
SAL_DA3669AA	CFSAN008684	Agona	0	0	0	0	0	0	0	0	0	0	0
SAL_DA3520AA	CFSAN023596	Agona	0	0	0	0	0	0	0	0	0	0	0
SAL_DA3445AA	MDH-2014-00491	Agona	0	0	0	0	0	0	0	0	0	0	0
SAL_DA3307AA	IEH-NGS-SAL-01279	Agona	0	0	0	0	0	0	0	0	0	0	0
SAL_DA3212AA	MDH-2014-00502	Agona	0	0	0	0	0	0	0	0	0	0	0
SAL_DA3143AA	CFSAN023653	Agona	0	0	0	0	0	0	0	0	0	0	0
SAL_DA2935AA	MDH-2014-00516	Agona	0	0	0	0	0	0	0	0	0	0	0
SAL_DA2591AA	FDA00003881	Agona	0	0	0	0	0	0	0	0	0	0	0
SAL_DA2367AA	FDA00008290	Agona	0	0	0	0	0	0	0	0	0	0	0
SAL_DA2383AA	AZ-TG74896	Agona	0	0	0	0	0	0	0	0	0	0	0
SAL_DA2344AA	FNW19a76	Agona	0	0	0	0	0	0	0	0	0	0	0
SAL_DA1984AA	WAPHL_SAL-A00593	Agona	0	0	0	0	0	0	0	0	0	0	0
SAL_DA1579AA	NY-29983	Agona	0	0	0	0	0	0	0	0	0	0	0
SAL_DA1271AA	H131100446	Agona	0	0	0	0	0	0	0	0	0	0	0
SAL_DA1247AA	H131100445	Agona	0	0	0	0	0	0	0	0	0	0	0
SAL_DA1096AA	H131100447	Agona	0	0	0	0	0	0	0	0	0	0	0
SAL_DA1012AA	H131120882	Agona	0	0	0	0	0	0	0	0	0	0	0
SAL_DA0681AA	H131120880	Agona	0	0	0	0	0	0	0	0	0	0	0
SAL_DA0647AA	H131120881	Agona	0	0	0	0	0	0	0	0	0	0	0
SAL_DA0559AA	H131120878	Agona	0	0	0	0	0	0	0	0	0	0	0
SAL_DA0530AA	H123660409	Agona	0	0	0	0	0	0	0	0	0	0	0
SAL_DA0509AA	H131140780	Agona	0	0	0	0	0	0	0	0	0	0	0
SAL_DA0443AA	H131120879	Agona	0	0	0	0	0	0	0	0	0	0	0
SAL_DA0382AA	H131120883	Agona	0	0	0	0	0	0	0	0	0	0	0
SAL_DA0369AA	H130161225	Agona	0	0	0	0	0	0	0	0	0	0	0
SAL_DA0276AA	AZ-TG74320	Agona	0	0	0	0	0	0	0	0	0	0	0
SAL_DA0149AA	FDA00003632	Agona	0	0	0	0	0	0	0	0	0	0	0
SAL_DA0131AA	FDA00003631	Agona	0	0	0	0	0	0	0	0	0	0	0
SAL_CA9983AA	NY-30654	Agona	0	0	0	0	0	0	0	0	0	0	0
SAL_CA9929AA	VA-WGS-00362	Agona	0	0	0	0	0	0	0	0	0	0	0
SAL_CA9799AA	FDA00003696	Agona	0	0	0	0	0	0	0	0	0	0	0
SAL_CA9800AA	FDA00003687	Agona	0	0	0	0	0	0	0	0	0	0	0
SAL_CA9798AA	FDA00003689	Agona	0	0	0	0	0	0	0	0	0	0	0

	1-1-1-294-1-1	1	0.000641026
	1-1-150-4-1-1	6	0.003846154
	1-1-1-268-1-1	1	0.000641026
	1-1-1-4-1-0	4	0.002564103
	1-1-1-329-63-1	1	0.000641026
	1-1-186-4-64-1	2	0.001282051
	1-1-1-378-1-46	1	0.000641026
	0-1-1-4-1-1	1	0.000641026
	1-1-1-338-1-1	1	0.000641026
	1-1-0-4-64-1	1	0.000641026
	1-1-191-4-0-44	1	0.000641026
	1-1-1-435-1-1	1	0.000641026
**Idikan**	0-0-0-0-0-0	97	/
**II**	0-0-0-0-0-0	211	0.981395349
	21-88-36-439-16-0	1	0.004651163
	0-0-45-0-0-0	1	0.004651163
	0-0-11-0-58-0	1	0.004651163
	0-0-0-0-61-0	1	0.004651163
**IIIa**	0-0-2-0-0-0	97	0.23150358
	0-0-0-0-0-0	303	0.723150358
	0-0-12-0-0-0	7	0.016706444
	0-0-82-0-0-0	2	0.00477327
	0-0-108-0-0-0	3	0.007159905
	0-0-18-0-0-0	7	0.016706444
**IIIb**	0-0-0-0-0-0	730	0.949284785
	0-0-19-0-0-0	12	0.015604681
	0-0-45-0-0-0	11	0.014304291
	0-0-54-0-0-0	11	0.014304291
	8-0-16-23-7-0	1	0.00130039
	9-0-69-0-0-0	1	0.00130039
	0-0-108-0-0-0	1	0.00130039
	0-0-151-0-0-0	1	0.00130039
	0-0-104-0-0-0	1	0.00130039
**Ilala**	0-0-0-0-0-0	3	/
**Indiana**	0-0-0-0-0-0	483	/
**Infantis**	0-0-0-0-0-0	7403	0.999729912
	0-0-8-293-3-0	1	0.000135044
	1-1-1-4-1-1	1	0.000135044
**Inganda**	0-0-0-0-0-0	1	/
**Inpraw**	0-0-0-0-0-0	1	/
**Inverness**	0-0-0-0-0-0	445	/
**Irenea**	0-0-0-0-0-0	1	/
**Irumu**	0-0-0-0-0-0	41	/
**Isaszeg**	0-0-0-0-0-0	1	/
**Israel**	0-0-0-0-0-0	1	/
**Ituri**	0-0-0-0-0-0	100	/
**IV**	0-0-0-0-0-0	381	0.994778068
	0-0-112-0-0-0	2	0.005221932
**Jangwani**	0-0-0-0-22-0	1	0.047619048
	0-0-0-0-0-0	20	0.952380952
**Javiana**	0-0-0-0-0-0	3124	0.998402045
	0-0-0-145-0-0	1	0.000319591
	0-0-0-0-62-0	3	0.000958773
	0-0-48-14-3-0	1	0.000319591
**Jedburgh**	0-0-0-0-0-0	19	0.95
	66-4-198-21-0-0	1	0.05
**Jerusalem**	0-0-0-0-0-0	2	/
**Johannesburg**	0-0-0-0-0-0	612	0.996742671
	67-61-161-291-22-0	1	0.001628664
	43-0-167-300-55-0	1	0.001628664
**Jubilee**	0-0-0-0-0-0	1	/
**Jukestown**	0-0-0-0-0-0	4	/
**Kaapstad**	0-0-0-0-0-0	5	/
**Kaitaan**	0-0-0-0-0-0	4	/
**Kalamu**	0-0-0-0-0-0	1	/
**Kalina**	0-0-0-0-0-0	5	/
**Kampala**	0-0-0-0-0-0	1	/
**Kaneshie**	0-0-0-0-0-0	3	/
**Kaolack**	0-0-0-0-0-0	2	/
**Kapemba**	0-0-0-0-0-0	5	/
**Karachi**	0-0-0-0-0-0	1	/
**Karamoja**	0-0-0-0-0-0	3	/
**Karlshamn**	0-0-0-0-0-0	1	/

SAL_CA9797AA	FDA00003690	Agona	0	0	0	0	0	0	0	0	0	0
SAL_CA9796AA	FDA00003693	Agona	0	0	0	0	0	0	0	0	0	0
SAL_CA9795AA	FDA00003694	Agona	0	0	0	0	0	0	0	0	0	0
SAL_CA9794AA	FDA00003699	Agona	0	0	0	0	0	0	0	0	0	0
SAL_CA9790AA	FDA00003698	Agona	0	0	0	0	0	0	0	0	0	0
SAL_CA9788AA	FDA00003688	Agona	0	0	0	0	0	0	0	0	0	0
SAL_CA9787AA	FDA00003692	Agona	0	0	0	0	0	0	0	0	0	0
SAL_CA9375AA	FDA00004812	Agona	0	0	0	0	0	0	0	0	0	0
SAL_CA9193AA	FDA00000853	Agona	0	0	0	0	0	0	0	0	0	0
SAL_CA9180AA	FDA00000832	Agona	0	0	0	0	0	0	0	0	0	0
SAL_CA9131AA	FDA00004817	Agona	0	0	0	0	0	0	0	0	0	0
SAL_CA9129AA	IEH-NGS-SAL-01776	Agona	0	0	0	0	0	0	0	0	0	0
SAL_CA9121AA	FDA00004048	Agona	0	0	0	0	0	0	0	0	0	0
SAL_CA9132AA	FDA00004816	Agona	0	0	0	0	0	0	0	0	0	0
SAL_CA9109AA	FDA00004824	Agona	0	0	0	0	0	0	0	0	0	0
SAL_CA9125AA	CFSAN027172	Agona	0	0	0	0	0	0	0	0	0	0
SAL_CA9019AA	NY-32419	Agona	0	0	0	0	0	0	0	0	0	0
SAL_CA8844AA	FL-NRM106	Agona	0	0	0	0	0	0	0	0	0	0
SAL_CA8835AA	NY-32459	Agona	0	0	0	0	0	0	0	0	0	0
SAL_CA8776AA	NY-32458	Agona	0	0	0	0	0	0	0	0	0	0
SAL_CA8672AA	CFSAN027940	Agona	0	0	0	0	0	0	0	0	0	0
SAL_CA8650AA	FL-NRM142	Agona	0	0	0	0	0	0	0	0	0	0
SAL_CA8639AA	CFSAN027942	Agona	0	0	0	0	0	0	0	0	0	0
SAL_CA8601AA	CFSAN027941	Agona	0	0	0	0	0	0	0	0	0	0
SAL_CA8575AA	94-0312	Agona	0	0	0	0	0	0	0	0	0	0
SAL_CA8465AA	FDA00000869	Agona	0	0	0	0	0	0	0	0	0	0
SAL_CA8402AA	FDA00000976	Agona	0	0	0	0	0	0	0	0	0	0
SAL_CA8348AA	FDA00003436	Agona	0	0	0	0	0	0	0	0	0	0
SAL_CA8340AA	FL-NRM185	Agona	0	0	0	0	0	0	0	0	0	0
SAL_CA8344AA	FDA00003437	Agona	0	0	0	0	0	0	0	0	0	0
SAL_CA8249AA	NY-N16	Agona	0	0	0	0	0	0	0	0	0	0
SAL_CA8235AA	FL-NRM228	Agona	0	0	0	0	0	0	0	0	0	0
SAL_CA8207AA	IEH-NGS-SAL-01897	Agona	0	0	0	0	0	0	0	0	0	0
SAL_CA8058AA	FDA00001270	Agona	0	0	0	0	0	0	0	0	0	0
SAL_CA8054AA	FDA00001282	Agona	0	0	0	0	0	0	0	0	0	0
SAL_CA7939AA	FDA00002930	Agona	0	0	0	0	0	0	0	0	0	0
SAL_CA7729AA	FDA00003030	Agona	0	0	0	0	0	0	0	0	0	0
SAL_CA7710AA	FDA00003031	Agona	0	0	0	0	0	0	0	0	0	0
SAL_CA7644AA	FDA00004746	Agona	0	0	0	0	0	0	0	0	0	0
SAL_CA7642AA	FDA00004744	Agona	0	0	0	0	0	0	0	0	0	0
SAL_CA7637AA	FDA00004743	Agona	0	0	0	0	0	0	0	0	0	0
SAL_CA7509AA	FL-NRM287	Agona	0	0	0	0	0	0	0	0	0	0
SAL_CA7294AA	FDA00001075	Agona	0	0	0	0	0	0	0	0	0	0
SAL_CA7168AA	FLUFL-1112	Agona	0	0	0	0	0	0	0	0	0	0
SAL_CA7155AA	FLUFL-1115	Agona	0	0	0	0	0	0	0	0	0	0
SAL_CA7150AA	NY-N11245	Agona	0	0	0	0	0	0	0	0	0	0
SAL_CA7132AA	NY-N11243	Agona	0	0	0	0	0	0	0	0	0	0
SAL_CA7091AA	NY-N11244	Agona	0	0	0	0	0	0	0	0	0	0
SAL_CA7065AA	NY-N11247	Agona	0	0	0	0	0	0	0	0	0	0
SAL_CA6963AA	NY-N13131	Agona	0	0	0	0	0	0	0	0	0	0
SAL_CA6972AA	NY-N11255	Agona	0	0	0	0	0	0	0	0	0	0
SAL_CA6751AA	62498	Agona	0	0	0	0	0	0	0	0	0	0
SAL_CA6728AA	54031	Agona	0	0	0	0	0	0	0	0	0	0
SAL_CA6714AA	48203	Agona	0	0	0	0	0	0	0	0	0	0
SAL_CA6600AA	66843	Agona	0	0	0	0	0	0	0	0	0	0
SAL_CA6539AA	57612	Agona	0	0	0	0	0	0	0	0	0	0
SAL_CA6242AA	58529	Agona	0	0	0	0	0	0	0	0	0	0
SAL_CA6186AA	46138	Agona	0	0	0	0	0	0	0	0	0	0
SAL_CA6054AA	59993	Agona	0	0	0	0	0	0	0	0	0	0
SAL_CA5972AA	60404	Agona	0	0	0	0	0	0	0	0	0	0
SAL_CA5912AA	57638	Agona	0	0	0	0	0	0	0	0	0	0
SAL_CA5884AA	58461	Agona	0	0	0	0	0	0	0	0	0	0
SAL_CA5826AA	73551	Agona	0	0	0	0	0	0	0	0	0	0
SAL_CA5769AA	80302	Agona	0	0	0	0	0	0	0	0	0	0
SAL_CA5672AA	70686	Agona	0	0	0	0	0	0	0	0	0	0
SAL_CA5658AA	68876	Agona	0	0	0	0	0	0	0	0	0	0
SAL_CA5583AA	78643	Agona	0	0	0	0	0	0	0	0	0	0
SAL_CA5542AA	68879	Agona	0	0	0	0	0	0	0	0	0	0
SAL_CA5484AA	69208	Agona	0	0	0	0	0	0	0	0	0	0
SAL_CA5431AA	43415	Agona	0	0	0	0	0	0	0	0	0	0
SAL_CA5316AA	43412	Agona	0	0	0	0	0	0	0	0	0	0
SAL_CA5286AA	78651	Agona	0	0	0	0	0	0	0	0	0	0
SAL_CA5231AA	43411	Agona	0	0	0	0	0	0	0	0	0	0

**Kasenyi**	0-0-0-0-0-0	2	/
**Kedougou**	0-0-0-0-0-0	195	0.994897959
	1-1-1-0-0-0	1	0.005102041
**Kentucky**	0-0-0-0-0-0	5854	0.999829206
	1-1-1-0-1-1	1	0.000170794
**Kiambu**	0-0-0-0-0-0	288	0.996539792
	73-72-180-322-60-41	1	0.003460208
**Kibusi**	0-0-0-0-0-0	10	/
**Kidderminster**	0-0-0-0-0-0	1	/
**Kikoma**	0-0-0-0-0-0	1	/
**Kimberley**	0-0-0-0-0-0	2	/
**Kingabwa**	0-0-0-0-0-0	19	/
**Kingston**	0-0-0-0-0-0	38	/
**Kinondoni**	0-0-0-0-0-0	4	/
**Kintambo**	0-0-0-0-0-0	33	/
**Kirkee**	0-0-0-0-0-0	17	/
**Kisangani**	0-0-0-0-0-0	29	/
**Kisarawe**	0-0-0-0-0-0	63	0.984375
	Kisarawe 7-0-216-440-86-0	1	0.015625
**Kitenge**	0-0-0-0-0-0	1	/
**Kivu**	0-0-0-0-0-0	3	/
**Kodjovi**	0-0-0-0-0-0	1	/
**Koketime**	0-0-0-0-0-0	23	/
**Kokomlemle**	0-0-0-0-0-0	16	/
**Kotte**	0-0-0-0-0-0	3	/
**Kuessel**	0-0-0-0-0-0	2	/
**Kumasi**	12-0-0-28-8-0	2	0.133333333
	0-0-0-0-0-0	13	0.866666667
**Kunduchi**	0-0-0-0-0-0	1	/
**Labadi**	0-0-0-0-0-0	4	/
**Landala**	0-0-0-0-0-0	3	/
**Landau**	0-0-0-0-0-0	2	/
**Langford**	0-0-0-0-0-0	1	/
**Lansing**	0-0-0-0-0-0	10	/
**Larochelle**	0-0-0-0-0-0	11	/
**Larose**	0-0-0-0-0-0	1	/
**Lattenkamp**	0-0-0-0-0-0	4	/
**Lawra**	0-0-0-0-0-0	1	/
**Leatherhead**	0-0-0-0-0-0	1	/
**Legon**	0-0-0-0-0-0	1	/
**Leith**	0-0-0-0-0-0	1	/
**Leoben**	0-0-0-0-0-0	3	/
**Lexington**	0-0-0-0-0-0	82	/
**Lika**	0-0-0-0-0-0	2	/
**Lille**	0-0-0-0-0-0	72	/
**Lindenburg**	0-0-0-0-0-0	1	/
**Lindi**	0-0-0-0-0-0	1	/
**Lingwala**	0-0-0-0-0-0	3	/
**Linton**	0-0-0-0-0-0	2	/
**Lisboa**	0-0-0-0-0-0	2	/
**Litchfield**	0-0-0-0-0-0	560	/
**Liverpool**	0-0-0-0-0-0	199	/
**Livingstone**	0-0-0-0-0-0	326	/
**Lomita**	0-0-0-0-0-0	1	0.333333333
	0-45-105-0-0-0	1	0.333333333
	0-0-0-0-0-0	1	0.333333333
**London**	0-0-0-0-0-0	435	/
**Louga**	2-2-21-24-0-0	4	1
**Luckenwalde**	0-0-0-0-0-0	1	/
**Luke**	0-0-0-0-0-0	11	/
**Maastricht**	0-0-0-0-0-0	8	/
**Macclesfield**	0-0-0-0-0-0	2	/
**Madras**	0-0-0-0-0-0	4	/
**Magumeri**	0-0-0-0-0-0	4	/
**Magwa**	0-0-0-0-0-0	2	/
**Malstatt**	0-0-0-0-0-0	1	/
**Manchester**	2-2-42-3-0-0	11	0.846153846
	2-2-42-66-0-0	1	0.076923077
	2-2-225-3-0-0	1	0.076923077
**Mandera**	0-0-0-0-0-0	2	/
**Manhattan**	0-0-0-0-0-0	277	/
**Maricopa**	0-0-0-0-0-0	1	/
**Martonos**	0-0-0-0-0-0	1	/

SAL_CA5022AA	71558	Agona	0	0	0	0	0	0	0	0	0	0	0	
SAL_CA4840AA	31912	Agona	0	0	0	0	0	0	0	0	0	0	0	
SAL_CA4836AA	34148	Agona	0	0	0	0	0	0	0	0	0	0	0	
SAL_CA4684AA	22228	Agona	0	0	0	0	0	0	0	0	0	0	0	
SAL_CA4608AA	72300	Agona	0	0	0	0	0	0	0	0	0	0	0	
SAL_CA4586AA	5832	Agona	0	0	0	0	0	0	0	0	0	0	0	
SAL_CA4538AA	84468	Agona	0	0	0	0	0	0	0	0	0	0	0	
SAL_CA4494AA	63931	Agona	0	0	0	0	0	0	0	0	0	0	0	
SAL_CA4480AA	39477	Agona	0	0	0	0	0	0	0	0	0	0	0	
SAL_CA4471AA	45598	Agona	0	0	0	0	0	0	0	0	0	0	0	
SAL_CA4412AA	63878	Agona	0	0	0	0	0	0	0	0	0	0	0	
SAL_CA4388AA	69893	Agona	0	0	0	0	0	0	0	0	0	0	0	
SAL_CA4380AA	96969	Agona	0	0	0	0	0	0	0	0	0	0	0	
SAL_CA4330AA	3738	Agona	0	0	0	0	0	0	0	0	0	0	0	
SAL_CA4327AA	25246	Agona	0	0	0	0	0	0	0	0	0	0	0	
SAL_CA4286AA	9455	Agona	0	0	0	0	0	0	0	0	0	0	0	
SAL_CA4227AA	13381	Agona	0	0	0	0	0	0	0	0	0	0	0	
SAL_CA4104AA	53099	Agona	0	0	0	0	0	0	0	0	0	0	0	
SAL_CA4036AA	31915	Agona	0	0	0	0	0	0	0	0	0	0	0	
SAL_CA3891AA	63772	Agona	0	0	0	0	0	0	0	0	0	0	0	
SAL_CA3802AA	45601	Agona	0	0	0	0	0	0	0	0	0	0	0	
SAL_CA3764AA	57145	Agona	0	0	0	0	0	0	0	0	0	0	0	
SAL_CA3723AA	83307	Agona	0	0	0	0	0	0	0	0	0	0	0	
SAL_CA3721AA	50323	Agona	0	0	0	0	0	0	0	0	0	0	0	
SAL_CA3682AA	31942	Agona	0	0	0	0	0	0	0	0	0	0	0	
SAL_CA3644AA	50311	Agona	0	0	0	0	0	0	0	0	0	0	0	
SAL_CA3583AA	63898	Agona	0	0	0	0	0	0	0	0	0	0	0	
SAL_CA3572AA	90480	Agona	0	0	0	0	0	0	0	0	0	0	0	
SAL_CA3538AA	51246	Agona	0	0	0	0	0	0	0	0	0	0	0	
SAL_CA3521AA	93503	Agona	0	0	0	0	0	0	0	0	0	0	0	
SAL_CA3405AA	3763	Agona	0	0	0	0	0	0	0	0	0	0	0	
SAL_CA3265AA	72276	Agona	0	0	0	0	0	0	0	0	0	0	0	
SAL_CA3013AA	91965	Agona	0	0	0	0	0	0	0	0	0	0	0	
SAL_CA2876AA	31947	Agona	0	0	0	0	0	0	0	0	0	0	0	
SAL_CA2839AA	45555	Agona	0	0	0	0	0	0	0	0	0	0	0	
SAL_CA2751AA	95060	Agona	0	0	0	0	0	0	0	0	0	0	0	
SAL_CA2663AA	40261	Agona	0	0	0	0	0	0	0	0	0	0	0	
SAL_CA2656AA	99072	Agona	0	0	0	0	0	0	0	0	0	0	0	
SAL_CA2657AA	83578	Agona	0	0	0	0	0	0	0	0	0	0	0	
SAL_CA2602AA	73091	Agona	0	0	0	0	0	0	0	0	0	0	0	
SAL_CA2586AA	13824	Agona	0	0	0	0	0	0	0	0	0	0	0	
SAL_CA2579AA	27774	Agona	0	0	0	0	0	0	0	0	0	0	0	
SAL_CA2525AA	67544	Agona	0	0	0	0	0	0	0	0	0	0	0	
SAL_CA2398AA	41954	Agona	0	0	0	0	0	0	0	0	0	0	0	
SAL_CA2369AA	75974	Agona	0	0	0	0	0	0	0	0	0	0	0	
SAL_CA2291AA	91265	Agona	0	0	0	0	0	0	0	0	0	0	0	
SAL_CA2174AA	89991	Agona	0	0	0	0	0	0	0	0	0	0	0	
SAL_CA2118AA	36509	Agona	0	0	0	0	0	0	0	0	0	0	0	
SAL_CA2100AA	37025	Agona	0	0	0	0	0	0	0	0	0	0	0	
SAL_CA1961AA	43419	Agona	0	0	0	0	0	0	0	0	0	0	0	
SAL_CA1921AA	56132	Agona	0	0	0	0	0	0	0	0	0	0	0	
SAL_CA1722AA	56133	Agona	0	0	0	0	0	0	0	0	0	0	0	
SAL_CA1378AA	91234	Agona	0	0	0	0	0	0	0	0	0	0	0	
SAL_CA1308AA	65037	Agona	0	0	0	0	0	0	0	0	0	0	0	
SAL_CA1266AA	40316	Agona	0	0	0	0	0	0	0	0	0	0	0	
SAL_CA1213AA	83309	Agona	0	0	0	0	0	0	0	0	0	0	0	
SAL_CA1183AA	83308	Agona	0	0	0	0	0	0	0	0	0	0	0	
SAL_CA0855AA	93528	Agona	0	0	0	0	0	0	0	0	0	0	0	
SAL_CA0764AA	40.E.08	Agona	0	0	0	0	0	0	0	0	0	0	0	
SAL_CA0755AA	83313	Agona	0	0	0	0	0	0	0	0	0	0	0	
SAL_CA0546AA	6441	Agona	0	0	0	0	0	0	0	0	0	0	0	
SAL_CA0382AA	24998	Agona	0	0	0	0	0	0	0	0	0	0	0	
SAL_CA0052AA	25010	Agona	0	0	0	0	0	0	0	0	0	0	0	
SAL_CA0055AA	40256	Agona	0	0	0	0	0	0	0	0	0	0	0	
SAL_BA9949AA	9165	Agona	0	0	0	0	0	0	0	0	0	0	0	
SAL_BA9869AA	94073	Agona	0	0	0	0	0	0	0	0	0	0	0	
SAL_BA9806AA	98279	Agona	0	0	0	0	0	0	0	0	0	0	0	
SAL_BA9802AA	:-2012-07-27T11:53:52Z-	Agona	0	0	0	0	0	0	0	0	0	0	0	
SAL_BA9800AA	:-2012-07-27T11:53:53Z-	Agona	0	0	0	0	0	0	0	0	0	0	0	
SAL_BA9568AA	41953	Agona	0	0	0	0	0	0	0	0	0	0	0	
SAL_BA9442AA	96933	Agona	0	0	0	0	0	0	0	0	0	0	0	
SAL_BA9375AA	43422	Agona	0	0	0	0	0	0	0	0	0	0	0	
SAL_BA9314AA	14882	Agona	0	0	0	0	0	0	0	0	0	0	0	

**Marylebone**	0-0-0-0-0-0	8	/
**Matadi**	0-0-0-0-0-0	18	/
**Mathura**	0-0-0-0-0-0	1	/
**Matopeni**	0-0-0-0-0-0	27	/
**Mbandaka**	0-0-0-0-0-0	2109	0.999526066
	1-1-1-4-1-1	1	0.000473934
**Mbao**	0-0-0-0-0-0	1	/
**Meleagridis**	0-0-0-0-0-0	370	/
**Mendoza**	0-0-0-0-0-0	1	/
**Menston**	0-0-0-0-0-0	5	/
**Mesbit**	0-0-0-0-0-0	1	/
**Mgulani**	0-0-0-0-0-0	30	/
**Miami**	0-0-0-0-0-0	98	/
**Michigan**	0-0-0-0-0-0	64	/
**Middlesbrough**	0-0-0-0-0-0	1	/
**Mikawasima**	0-0-0-0-0-0	342	/
**Millesi**	0-0-0-0-0-0	6	/
**Minnesota**	0-0-0-0-0-0	294	/
**Mississippi**	0-0-0-0-0-0	430	/
**Mkamba**	0-0-0-0-0-0	1	/
**Moero**	0-0-0-0-0-0	8	/
**Molade**	0-0-0-0-0-0	10	/
**Montevideo**	0-0-0-0-0-0	3508	/
**Morehead**	0-0-0-0-0-0	3	/
**Moroto**	0-0-0-0-0-0	2	/
**Moualine**	0-0-0-0-0-0	1	/
**Mountpleasant**	0-0-0-0-0-0	10	/
**Muenchen**	0-0-0-0-0-0	2777	0.999640029
	99-0-0-447-92-0	1	0.000359971
**Muenster**	0-0-0-0-0-0	718	/
**Mundonobo**	0-0-0-0-0-0	1	/
**Nagoya**	0-0-0-0-0-0	2	/
**Namur**	0-0-0-0-0-0	1	/
**Nchanga**	0-0-0-0-0-0	36	/
**Neukoelln**	0-0-0-0-0-0	10	/
**Neunkirchen**	0-0-0-0-0-0	3	/
**Newmexico**	0-0-0-0-0-0	7	/
**Newport**	0-0-0-0-0-0	9355	0.999038872
	23-0-41-0-18-0	1	0.000106792
	43-0-0-227-45-0	1	0.000106792
	18-3-62-384-0-0	1	0.000106792
	90-82-36-412-16-0	5	0.00053396
	21-85-36-56-16-0	1	0.000106792
**Newyork**	0-0-0-0-0-0	105	/
**Ngili**	0-0-0-0-0-0	6	/
**Niederoderwitz**	0-0-0-0-0-0	1	/
**Nigeria**	0-0-0-0-0-0	13	/
**Nijmegen**	0-0-0-0-0-0	6	/
**Nima**	0-0-0-0-0-0	55	/
**Norwich**	0-0-0-0-0-0	426	/
**Nottingham**	0-0-0-0-0-0	59	0.967213115
	11-1-22-26-1-4	1	0.016393443
	5-4-6-370-0-0	1	0.016393443
**Oakland**	0-0-0-0-0-0	9	/
**Obogu**	0-0-0-0-0-0	3	/
**Odozi**	0-0-0-0-0-0	3	/
**Offa**	0-0-0-0-0-0	22	/
**Ohio**	0-0-0-0-0-0	521	/
**Okatie**	0-0-0-0-0-0	1	/
**Okerara**	0-0-0-0-0-0	26	/
**Oranienburg**	0-0-0-0-0-0	1859	0.999462366
	0-0-108-0-0-0	1	0.000537634
**Orbe**	0-0-0-0-0-0	13	/
**Ordonez**	0-0-0-0-0-0	6	/
**Orientalis**	0-0-0-0-0-0	38	/
**Orion**	0-0-0-0-0-0	158	/
**Orlando**	0-0-0-0-0-0	1	/
**Oskarshamn**	0-0-0-0-0-0	1	/
**Oslo**	0-0-0-0-0-0	259	0.996153846
	5-4-6-21-0-0	1	0.003846154
**Othmarschen**	0-0-0-0-0-0	9	/
**Ouakam**	0-0-0-0-0-0	92	/
**Overschie**	0-0-0-0-0-0	5	/

SAL_BA9254AA	38378	Agona	0	0	0	0	0	0	0	0	0	0	0
SAL_BA8783AA	65416	Agona	0	0	0	0	0	0	0	0	0	0	0
SAL_BA8760AA	40789	Agona	0	0	0	0	0	0	0	0	0	0	0
SAL_BA8683AA	13825	Agona	0	0	0	0	0	0	0	0	0	0	0
SAL_BA8664AA	NY_BAC0800001777	Agona	0	0	0	0	0	0	0	0	0	0	0
SAL_BA8468AA	80580	Agona	0	0	0	0	0	0	0	0	0	0	0
SAL_BA8419AA	98275	Agona	0	0	0	0	0	0	0	0	0	0	0
SAL_BA8223AA	69269	Agona	0	0	0	0	0	0	0	0	0	0	0
SAL_BA8214AA	CFSAN005731	Agona	0	0	0	0	0	0	0	0	0	0	0
SAL_BA8196AA	CFSAN005740	Agona	0	0	0	0	0	0	0	0	0	0	0
SAL_BA7858AA	86882	Agona	0	0	0	0	0	0	0	0	0	0	0
SAL_BA7522AA	93562	Agona	0	0	0	0	0	0	0	0	0	0	0
SAL_BA7535AA	21759	Agona	0	0	0	0	0	0	0	0	0	0	0
SAL_BA7367AA	CFSAN030301	Agona	0	0	0	0	0	0	0	0	0	0	0
SAL_BA6664AA	08:MF0010405	Agona	0	0	0	0	0	0	0	0	0	0	0
SAL_BA6519AA	2015K-0227	Agona	0	0	0	0	0	0	0	0	0	0	0
SAL_BA6145AA	FLUFL-04E01556	Agona	0	0	0	0	0	0	0	0	0	0	0
SAL_BA5506AA	460004 2-1	Agona	0	0	0	0	0	0	0	0	0	0	0
SAL_BA5388AA	CFSAN024614	Agona	0	0	0	0	0	0	0	0	0	0	0
SAL_BA5295AA	-2012-11-09T12:23:29Z-	Agona	0	0	0	0	0	0	0	0	0	0	0
SAL_BA5278AA	c-2012-11-09T12:23:37Z	Agona	0	0	0	0	0	0	0	0	0	0	0
SAL_BA4729AA	FDA00002500	Agona	0	0	0	0	0	0	0	0	0	0	0
SAL_BA4691AA	FDA00002505	Agona	0	0	0	0	0	0	0	0	0	0	0
SAL_BA4649AA	CFSAN006254	Agona	0	0	0	0	0	0	0	0	0	0	0
SAL_BA3667AA	CFSAN006260	Agona	0	0	0	0	0	0	0	0	0	0	0
SAL_BA3642AA	CFSAN006261	Agona	0	0	0	0	0	0	0	0	0	0	0
SAL_BA3577AA	CFSAN024550	Agona	0	0	0	0	0	0	0	0	0	0	0
SAL_BA3576AA	CFSAN024576	Agona	0	0	0	0	0	0	0	0	0	0	0
SAL_BA3526AA	CFSAN024542	Agona	0	0	0	0	0	0	0	0	0	0	0
SAL_BA2990AA	NY_FSL S10-1750	Agona	0	0	0	0	0	0	0	0	0	0	0
SAL_BA2778AA	CFSAN030636	Agona	0	0	0	0	0	0	0	0	0	0	0
SAL_BA2595AA	ADRDL-14-15340	Agona	0	0	0	0	0	0	0	0	0	0	0
SAL_BA2054AA	ADRDL-14-18916	Agona	0	0	0	0	0	0	0	0	0	0	0
SAL_BA2006AA	NY-N14646	Agona	0	0	0	0	0	0	0	0	0	0	0
SAL_BA1423AA	ADRDL-LA-1-2014	Agona	0	0	0	0	0	0	0	0	0	0	0
SAL_BA1421AA	ADRDL-LA-6-2014	Agona	0	0	0	0	0	0	0	0	0	0	0
SAL_BA1390AA	ADRDL-LA-13-2014	Agona	0	0	0	0	0	0	0	0	0	0	0
SAL_BA1391AA	ADRDL-LA-9-2014	Agona	0	0	0	0	0	0	0	0	0	0	0
SAL_BA1330AA	FDA00002242	Agona	0	0	0	0	0	0	0	0	0	0	0
SAL_BA1332AA	ADRDL-LA-36-2014	Agona	0	0	0	0	0	0	0	0	0	0	0
SAL_BA1329AA	ADRDL-LA-35-2014	Agona	0	0	0	0	0	0	0	0	0	0	0
SAL_BA1294AA	ADRDL-LA-38-2014	Agona	0	0	0	0	0	0	0	0	0	0	0
SAL_BA1291AA	ADRDL-LA-37-2014	Agona	0	0	0	0	0	0	0	0	0	0	0
SAL_BA1281AA	FDA00002243	Agona	0	0	0	0	0	0	0	0	0	0	0
SAL_BA1275AA	ADRDL-LA-47-2014	Agona	0	0	0	0	0	0	0	0	0	0	0
SAL_BA1272AA	ADRDL-LA-48-2014	Agona	0	0	0	0	0	0	0	0	0	0	0
SAL_BA1268AA	ADRDL-LA-50-2014	Agona	0	0	0	0	0	0	0	0	0	0	0
SAL_BA1262AA	ADRDL-LA-49-2014	Agona	0	0	0	0	0	0	0	0	0	0	0
SAL_BA1269AA	ADRDL-LA-39-2014	Agona	0	0	0	0	0	0	0	0	0	0	0
SAL_BA1206AA	CVM-N15232	Agona	0	0	0	0	0	0	0	0	0	0	0
SAL_BA1053AA	CFSAN031422	Agona	0	0	0	0	0	0	0	0	0	0	0
SAL_BA0785AA	FDA00001885	Agona	0	0	0	0	0	0	0	0	0	0	0
SAL_AA9846AA	c-2013-10-08T14:25:38Z	Agona	0	0	0	0	0	0	0	0	0	0	0
SAL_AA9845AA	c-2013-10-08T14:25:39Z	Agona	0	0	0	0	0	0	0	0	0	0	0
SAL_FA6186AA	FSIS1606393	Agona	0	0	0	0	0	0	0	0	0	0	0
SAL_FA6185AA	FSIS1606340	Agona	0	0	0	0	0	0	0	0	0	0	0
SAL_FA6175AA	FSIS1606410	Agona	0	0	0	0	0	0	0	0	0	0	0
SAL_FA6403AA	FDA00001513	Agona	0	0	0	0	0	0	0	0	0	0	0
SAL_FA6431AA	PNUSAS001701	Agona	0	0	0	0	0	0	0	0	0	0	0
SAL_FA6300AA	FSIS1606513	Agona	0	0	0	0	0	0	0	0	0	0	0
SAL_FA6302AA	FSIS1606522	Agona	0	0	0	0	0	0	0	0	0	0	0
SAL_FA6295AA	FSIS1606520	Agona	0	0	0	0	0	0	0	0	0	0	0
SAL_FA6488AA	FDA00003234	Agona	0	0	0	0	0	0	0	0	0	0	0
SAL_FA6487AA	FDA00003235	Agona	0	0	0	0	0	0	0	0	0	0	0
SAL_FA6666AA	CVM-N27410	Agona	0	0	0	0	0	0	0	0	0	0	0
SAL_FA6840AA	CVM-N27413	Agona	0	0	0	0	0	0	0	0	0	0	0
SAL_FA7276AA	FDA00010148	Agona	0	0	0	0	0	0	0	0	0	0	0
SAL_FA7285AA	FDA00001153	Agona	0	0	0	0	0	0	0	0	0	0	0
SAL_FA7511AA	CVM-N28614	Agona	0	0	0	0	0	0	0	0	0	0	0
SAL_FA3184AA	PE018	Agona	0	0	0	0	0	0	0	0	0	0	0
SAL_EA2602AA	CVM-N15800	Agona	0	0	0	0	0	0	0	0	0	0	0
SAL_FA8185AA	FDA00003621	Agona	0	0	0	0	0	0	0	0	0	0	0

**Panama**	0-0-0-0-0-0	606	/
**Paratyphi A**	20-2-35-55-0-0	1660	0.992229528
	20-2-35-137-0-0	2	0.001195457
	20-2-35-158-0-0	8	0.004781829
	20-60-35-55-0-0	2	0.001195457
	20-2-35-400-0-0	1	0.000597729
**Paratyphi B**	0-0-0-0-0-0	1800	0.999444753
	1-1-1-4-1-1	1	0.000555247
**Paratyphi C**	3-0-0-0-0-0	141	0.854545455
	0-0-0-0-0-0	10	0.060606061
	22-0-0-61-17-0	6	0.036363636
	22-0-0-0-17-0	4	0.024242424
	3-0-0-0-73-0	1	0.006060606
	94-0-0-0-0-0	2	0.012121212
	95-0-0-0-0-0	1	0.006060606
**Pasing**	0-0-0-0-0-0	2	/
**Perth**	0-0-0-0-0-0	4	/
**Poano**	0-0-0-0-0-0	12	/
**Pomona**	0-0-0-0-0-0	238	/
**Poona**	0-0-0-0-0-0	1267	0.996852872
	0-12-23-409-80-0	3	0.002360346
	0-0-23-409-80-0	1	0.000786782
**Portland**	0-0-0-0-0-0	2	/
**Potsdam**	0-0-0-0-0-0	110	/
**Praha**	0-0-0-0-0-0	7	/
**Putten**	0-0-0-0-0-0	54	/
**Quebec**	0-0-0-0-0-0	1	/
**Quinhon**	0-0-0-0-0-0	1	/
**Ramatgan**	0-0-0-0-0-0	5	0.833333333
	5-4-6-21-0-0	1	0.166666667
**Reading**	0-0-0-0-0-0	1126	/
**Redba**	0-0-0-0-0-0	1	/
**Redlands**	0-0-0-0-0-0	78	/
**Remiremont**	0-0-0-0-0-0	1	/
**Remo**	0-0-0-0-0-0	2	/
**Richmond**	0-0-0-0-0-0	95	/
**Rissen**	0-0-0-0-0-0	394	/
**Riverside**	0-0-0-0-0-0	11	/
**Roan**	0-0-0-0-0-0	1	/
**Roodepoort**	0-0-0-0-0-0	14	/
**Rottnest**	0-0-0-0-0-0	1	/
**Rovaniemi**	0-0-0-0-0-0	3	/
**Rubislaw**	0-0-0-0-0-0	889	/
**Ruiru**	0-0-0-0-0-0	20	/
**Saarbruecken**	0-0-0-0-0-0	2	/
**Saintpaul**	0-0-0-0-0-0	3429	0.998834838
	5-4-6-21-0-0	2	0.000582581
	0-0-18-0-0-0	1	0.00029129
	0-0-23-296-53-0	1	0.00029129
**Salford**	0-0-0-0-0-0	6	/
**Sandiego**	0-0-0-0-0-0	505	0.996055227
	0-0-11-0-79-0	2	0.003944773
**Sangera**	0-0-0-0-0-0	1	/
**Sanjuan**	0-0-0-0-0-0	9	/
**Saphra**	0-0-0-0-0-0	22	/
**Saugus**	0-0-0-0-0-0	5	/
**Schleissheim**	0-0-0-0-0-0	783	/
**Schoeneberg**	0-0-0-0-0-0	1	/
**Schwarzengrund**	0-0-0-0-0-0	1633	/
**Seegefeld**	0-0-0-0-0-0	2	/
**Sekondi**	0-0-0-0-0-0	1	/
**Sendai**	0-0-0-0-0-0	18	/
**Senegal**	0-0-0-0-0-0	4	/
**Senftenberg**	0-0-0-0-0-0	1975	0.999493927
	5-4-5-0-0-0	1	0.000506073
**Shamba**	0-0-0-0-0-0	2	/
**Shubra**	0-0-0-0-0-0	11	0.916666667
	5-59-17-277-0-0	1	0.083333333
**Sloterdijk**	0-0-0-0-0-0	1	/
**Soahanina**	0-0-0-0-0-0	50	/
	0-0-0-0-0-0	90	0.97826087
**Soerenga**	0-0-11-0-58-0	1	0.010869565

SAL_GA2755AA	CRJJGF_00158	Agona	0	0	0	0	0	0	0	0	0	0	0
SAL_GA3993AA	SOP_12	Agona	0	0	0	0	0	0	0	0	0	0	0
SAL_GA3802AA	DMS 203/74	Agona	0	0	0	0	0	0	0	0	0	0	0
SAL_GA3724AA	BCW_2028	Agona	0	0	0	0	0	0	0	0	0	0	0
SAL_GA3704AA	BCW_2168	Agona	0	0	0	0	0	0	0	0	0	0	0
SAL_GA3527AA	BCW_2832	Agona	0	0	0	0	0	0	0	0	0	0	0
SAL_GA3422AA	BCW_2079	Agona	0	0	0	0	0	0	0	0	0	0	0
SAL_GA3405AA	BCW_2134	Agona	0	0	0	0	0	0	0	0	0	0	0
SAL_GA3337AA	BCW_1598	Agona	0	0	0	0	0	0	0	0	0	0	0
SAL_GA3206AA	N31394	Agona	0	0	0	0	0	0	0	0	0	0	0
SAL_GA3208AA	N30652	Agona	0	0	0	0	0	0	0	0	0	0	0
SAL_GA3080AA	BCW_4358	Agona	0	0	0	0	0	0	0	0	0	0	0
SAL_GA3078AA	BCW_4362	Agona	0	0	0	0	0	0	0	0	0	0	0
SAL_GA2892AA	CRJJGF_00019	Agona	0	0	0	0	0	0	0	0	0	0	0
SAL_GA2891AA	CRJJGF_00020	Agona	0	0	0	0	0	0	0	0	0	0	0
SAL_GA2395AA	BCW_2226	Agona	0	0	0	0	0	0	0	0	0	0	0
SAL_GA2390AA	BCW_2436	Agona	0	0	0	0	0	0	0	0	0	0	0
SAL_GA2341AA	BCW_2632	Agona	0	0	0	0	0	0	0	0	0	0	0
SAL_GA2246AA	BCW_5909	Agona	0	0	0	0	0	0	0	0	0	0	0
SAL_GA2145AA	BCW_6292	Agona	0	0	0	0	0	0	0	0	0	0	0
SAL_GA2113AA	BCW_6614	Agona	0	0	0	0	0	0	0	0	0	0	0
SAL_GA2109AA	BCW_6639	Agona	0	0	0	0	0	0	0	0	0	0	0
SAL_GA2104AA	BCW_6660	Agona	0	0	0	0	0	0	0	0	0	0	0
SAL_GA2103AA	BCW_6661	Agona	0	0	0	0	0	0	0	0	0	0	0
SAL_GA1944AA	BCW_4895	Agona	0	0	0	0	0	0	0	0	0	0	0
SAL_GA1949AA	BCW_4890	Agona	0	0	0	0	0	0	0	0	0	0	0
SAL_GA1939AA	BCW_4900	Agona	0	0	0	0	0	0	0	0	0	0	0
SAL_GA1928AA	BCW_4911	Agona	0	0	0	0	0	0	0	0	0	0	0
SAL_GA1922AA	BCW_4917	Agona	0	0	0	0	0	0	0	0	0	0	0
SAL_GA1925AA	BCW_4914	Agona	0	0	0	0	0	0	0	0	0	0	0
SAL_GA1909AA	BCW_4930	Agona	0	0	0	0	0	0	0	0	0	0	0
SAL_GA1908AA	BCW_4931	Agona	0	0	0	0	0	0	0	0	0	0	0
SAL_GA1907AA	BCW_4932	Agona	0	0	0	0	0	0	0	0	0	0	0
SAL_GA1816AA	BCW_4345	Agona	0	0	0	0	0	0	0	0	0	0	0
SAL_GA1766AA	N29318	Agona	0	0	0	0	0	0	0	0	0	0	0
SAL_GA1764AA	N29334	Agona	0	0	0	0	0	0	0	0	0	0	0
SAL_GA1646AA	BCW_4357	Agona	0	0	0	0	0	0	0	0	0	0	0
SAL_GA1644AA	BCW_4363	Agona	0	0	0	0	0	0	0	0	0	0	0
SAL_GA1641AA	BCW_4367	Agona	0	0	0	0	0	0	0	0	0	0	0
SAL_GA1633AA	BCW_4375	Agona	0	0	0	0	0	0	0	0	0	0	0
SAL_GA1631AA	BCW_4377	Agona	0	0	0	0	0	0	0	0	0	0	0
SAL_GA1626AA	BCW_4383	Agona	0	0	0	0	0	0	0	0	0	0	0
SAL_GA4386AA	CVM-N23623	Agona	0	0	0	0	0	0	0	0	0	0	0
SAL_GA0720AA	pw3498_6-sc-2235487	Agona	0	0	0	0	0	0	0	0	0	0	0
SAL_FA9556AA	CVM-N23721	Agona	0	0	0	0	0	0	0	0	0	0	0
SAL_FA9553AA	CVM-N23720	Agona	0	0	0	0	0	0	0	0	0	0	0
SAL_GA4914AA	FSIS1607130	Agona	0	0	0	0	0	0	0	0	0	0	0
SAL_GA4745AA	PNUSAS002328	Agona	0	0	0	0	0	0	0	0	0	0	0
SAL_FA9470AA	FDA00002808	Agona	0	0	0	0	0	0	0	0	0	0	0
SAL_FA9413AA	CVM-N19735	Agona	0	0	0	0	0	0	0	0	0	0	0
SAL_FA9177AA	CVM N38874	Agona	0	0	0	0	0	0	0	0	0	0	0
SAL_FA9157AA	CVM N38894	Agona	0	0	0	0	0	0	0	0	0	0	0
SAL_FA9152AA	CVM N38904	Agona	0	0	0	0	0	0	0	0	0	0	0
SAL_GA5830AA	FDA00001403	Agona	0	0	0	0	0	0	0	0	0	0	0
SAL_GA5745AA	CVM N56243	Agona	0	0	0	0	0	0	0	0	0	0	0
SAL_GA5623AA	CVM N62476	Agona	0	0	0	0	0	0	0	0	0	0	0
SAL_GA5615AA	CVM N62487	Agona	0	0	0	0	0	0	0	0	0	0	0
SAL_GA5612AA	CVM N62490	Agona	0	0	0	0	0	0	0	0	0	0	0
SAL_GA5604AA	CVM N62499	Agona	0	0	0	0	0	0	0	0	0	0	0
SAL_GA5546AA	PNUSAS002975	Agona	0	0	0	0	0	0	0	0	0	0	0
SAL_FA9046AA	CVM N40410	Agona	0	0	0	0	0	0	0	0	0	0	0
SAL_FA8948AA	CVM N42238	Agona	0	0	0	0	0	0	0	0	0	0	0
SAL_FA8933AA	CVM N42338	Agona	0	0	0	0	0	0	0	0	0	0	0
SAL_FA8924AA	CVM N42457	Agona	0	0	0	0	0	0	0	0	0	0	0
SAL_FA8862AA	CVM N42520	Agona	0	0	0	0	0	0	0	0	0	0	0
SAL_FA8843AA	CVM N29309	Agona	0	0	0	0	0	0	0	0	0	0	0
SAL_FA8834AA	CVM N29318	Agona	0	0	0	0	0	0	0	0	0	0	0
SAL_FA8830AA	CVM N29322	Agona	0	0	0	0	0	0	0	0	0	0	0
SAL_FA8818AA	CVM N29334	Agona	0	0	0	0	0	0	0	0	0	0	0
SAL_FA8753AA	CVM N30651	Agona	0	0	0	0	0	0	0	0	0	0	0
SAL_FA8752AA	CVM N30652	Agona	0	0	0	0	0	0	0	0	0	0	0
SAL_FA8748AA	CVM N30656	Agona	0	0	0	0	0	0	0	0	0	0	0
SAL_FA8684AA	CVM N31394	Agona	0	0	0	0	0	0	0	0	0	0	0

	0-0-0-0-59-0	1	0.010869565
Stanley	0-0-0-0-0-0	1305	0.996183206
	65-17-47-77-19-3	1	0.000763359
	65-17-47-0-19-3	1	0.000763359
	96-72-217-0-87-54	1	0.000763359
	1-17-47-77-19-3	1	0.000763359
	18-3-5-0-3-0	1	0.000763359
Stanleyville	0-0-0-0-0-0	111	0.925
	5-4-6-8-0-0	3	0.025
	1-1-1-0-1-1	2	0.016666667
	1-1-0-0-1-1	2	0.016666667
	1-1-203-386-1-3	1	0.008333333
	1-1-0-444-1-1	1	0.008333333
Sterrenbos	0-0-0-0-0-0	3	/
Stockholm	0-0-0-0-0-0	2	0.5
	5-4-195-8-0-0	2	0.5
Stourbridge	0-0-0-0-0-0	15	/
Strathcona	0-0-0-0-0-0	5	/
Stuttgart	0-0-0-0-0-0	1	/
Suelldorf	0-0-0-0-0-0	7	/
Sundsvall	0-0-0-0-0-0	11	/
Surat	0-0-0-0-0-0	1	/
Szentes	0-0-0-0-0-0	15	/
Tafo	5-4-169-8-0-0	1	0.333333333
	5-4-194-8-0-0	1	0.333333333
	0-0-0-0-0-0	1	0.333333333
Tallahassee	0-0-0-0-0-0	25	/
Tamberma	0-0-0-0-0-0	11	/
Tampico	0-0-0-0-0-0	1	/
Tanger	0-0-196-0-0-0	2	/
Tarshyne	2-2-3-0-0-0	2	0.333333333
	2-2-3-3-0-0	3	0.5
	0-0-0-0-0-0	1	0.166666667
Taunton	0-0-0-0-0-0	1	/
Tchad	0-0-0-0-0-0	3	/
Teddington	0-0-0-0-0-0	5	/
Tees	0-0-0-0-0-0	5	/
Teko	0-0-0-0-0-0	22	/
Telaviv	0-0-0-0-0-0	16	/
Teltow	0-0-0-0-0-0	1	/
Tempe	0-0-0-0-0-0	1	/
Tennessee	0-0-0-0-0-0	507	/
Tennyson	0-0-0-0-0-0	4	/
Teshie	0-0-0-0-0-0	18	/
Thompson	0-0-0-0-0-0	2349	0.999149298
	0-0-164-0-0-0	1	0.000425351
	18-3-5-0-0-0	1	0.000425351
Tienba	0-0-0-0-0-0	1	/
Tilene	0-0-0-0-0-0	5	/
Tinda	0-0-0-0-0-0	10	/
Togba	0-0-0-0-0-0	1	/
Tokoin	0-0-0-0-0-0	2	/
Tornow	0-0-0-0-0-0	1	/
Treforest	0-0-0-0-0-0	15	/
Troy	0-0-0-0-0-0	1	/
Tucson	0-0-0-0-0-0	46	/
Tudu	0-0-0-0-0-0	10	/
	2-2-4-3-0-0	7318	0.910538758
	2-2-4-20-0-0	130	0.01617519
	2-2-37-3-0-0	75	0.00933184
	2-2-4-58-0-0	298	0.037078512
	2-2-38-3-0-0	27	0.003359462
	2-2-4-108-0-0	2	0.000248849
	2-2-60-3-0-0	30	0.003732736
	2-2-61-3-0-0	11	0.00136867
	0-0-0-0-0-0	8	0.000995396
	2-2-4-57-0-0	9	0.001119821
	2-2-4-0-3-0	1	0.000124425
	2-2-68-3-0-0	1	0.000124425
	2-2-4-60-0-0	6	0.000746547
	2-2-4-129-0-0	13	0.001617519
	32-2-4-3-0-0	1	0.000124425
	2-2-4-149-0-0	7	0.000870972

SAL_FA8583AA	CVM N32776	Agona	0	0	0	0	0	0	0	0	0	0	0			2-2-4-55-0-0	29	0.003608312
SAL_FA8703AA	CVM N30701	Agona	0	0	0	0	0	0	0	0	0	0	0			2-2-4-187-3-0	1	0.000124425
SAL_GA5973AA	FSIS1607174	Agona	0	0	0	0	0	0	0	0	0	0	0			2-2-95-3-0-0	3	0.000373274
SAL_GA5938AA	FSIS1607159	Agona	0	0	0	0	0	0	0	0	0	0	0			2-41-4-3-0-0	7	0.000870972
SAL_GA5933AA	FSIS1607113	Agona	0	0	0	0	0	0	0	0	0	0	0			2-2-4-202-0-0	1	0.000124425
SAL_GA6044AA	FSIS1607168	Agona	0	0	0	0	0	0	0	0	0	0	0			2-2-4-206-0-0	1	0.000124425
SAL_GA6659AA	FSIS1607287	Agona	0	0	0	0	0	0	0	0	0	0	0			2-2-4-207-0-0	6	0.000746547
SAL_GA6891AA	A03R	Agona	0	0	0	0	0	0	0	0	0	0	0			2-42-4-3-0-0	1	0.000124425
SAL_GA7179AA	CFSAN025360	Agona	0	0	0	0	0	0	0	0	0	0	0			2-2-97-3-0-0	1	0.000124425
SAL_GA6960AA	FDA00004690	Agona	0	0	0	0	0	0	0	0	0	0	0			2-2-37-58-0-0	1	0.000124425
SAL_GA7385AA	FDA00001428	Agona	0	0	0	0	0	0	0	0	0	0	0			2-43-4-3-0-0	6	0.000746547
SAL_GA7371AA	FDA00004751	Agona	0	0	0	0	0	0	0	0	0	0	0			2-2-4-210-0-0	1	0.000124425
SAL_GA7547AA	CFSAN025368	Agona	0	0	0	0	0	0	0	0	0	0	0			2-2-99-3-0-0	1	0.000124425
SAL_GA7551AA	CFSAN025365	Agona	0	0	0	0	0	0	0	0	0	0	0		**Typhi**	0-2-4-3-0-0	1	0.000124425
SAL_GA7548AA	CFSAN025366	Agona	0	0	0	0	0	0	0	0	0	0	0			2-44-4-3-0-0	1	0.000124425
SAL_GA7959AA	2014AM-2743	Agona	0	0	0	0	0	0	0	0	0	0	0			2-2-100-3-0-0	1	0.000124425
SAL_GA7819AA	2014AM-1098	Agona	0	0	0	0	0	0	0	0	0	0	0			2-2-4-221-0-0	2	0.000248849
SAL_GA7813AA	2014AM-2796	Agona	0	0	0	0	0	0	0	0	0	0	0			2-51-4-3-0-0	1	0.000124425
SAL_GA8037AA	FSIS1607384	Agona	0	0	0	0	0	0	0	0	0	0	0			2-2-38-55-0-0	2	0.000248849
SAL_GA8447AA	FSIS1607446	Agona	0	0	0	0	0	0	0	0	0	0	0			2-2-4-237-0-0	1	0.000124425
SAL_GA8748AA	CFSAN025373	Agona	0	0	0	0	0	0	0	0	0	0	0			2-52-4-3-0-0	1	0.000124425
SAL_GA8747AA	CFSAN025374	Agona	0	0	0	0	0	0	0	0	0	0	0			2-53-4-3-0-0	1	0.000124425
SAL_GA9063AA	FSIS1607563	Agona	0	0	0	0	0	0	0	0	0	0	0			2-2-4-238-0-0	1	0.000124425
SAL_GA9087AA	OH-1212730	Agona	0	0	0	0	0	0	0	0	0	0	0			2-2-4-239-0-0	1	0.000124425
SAL_GA9269AA	PNUSAS003849	Agona	0	0	0	0	0	0	0	0	0	0	0			2-2-121-3-0-0	1	0.000124425
SAL_GA9239AA	FDA00010596	Agona	0	0	0	0	0	0	0	0	0	0	0			2-2-4-0-0-0	3	0.000373274
SAL_GA9223AA	FDA00010638	Agona	0	0	0	0	0	0	0	0	0	0	0			2-2-148-3-0-0	4	0.000497698
SAL_GA9459AA	FSIS1607651	Agona	0	0	0	0	0	0	0	0	0	0	0			2-2-4-273-0-0	1	0.000124425
SAL_GA9843AA	PNUSAS003954	Agona	0	0	0	0	0	0	0	0	0	0	0			2-2-37-55-0-0	1	0.000124425
SAL_HA0234AA	ADRDL-JS676	Agona	0	0	0	0	0	0	0	0	0	0	0			2-2-4-59-0-0	1	0.000124425
SAL_HA0417AA	CFSAN025423	Agona	0	0	0	0	0	0	0	0	0	0	0			0-0-170-123-3-0	1	0.000124425
SAL_HA0933AA	PNUSAS003810	Agona	0	0	0	0	0	0	0	0	0	0	0			2-2-176-3-0-0	1	0.000124425
SAL_HA0864AA	PNUSAS004123	Agona	0	0	0	0	0	0	0	0	0	0	0			2-2-4-321-0-0	1	0.000124425
SAL_HA0725AA	PNUSAS004492	Agona	0	0	0	0	0	0	0	0	0	0	0			2-2-4-377-0-0	1	0.000124425
SAL_HA0517AA	PNUSAS004379	Agona	0	0	0	0	0	0	0	0	0	0	0			2-2-4-397-0-0	2	0.000248849
SAL_HA0625AA	2014AM-0173	Agona	0	0	0	0	0	0	0	0	0	0	0			2-2-4-408-0-0	1	0.000124425
SAL_HA1144AA	TX-ACF1601964	Agona	0	0	0	0	0	0	0	0	0	0	0			2-2-4-414-0-0	3	0.000373274
SAL_HA1630AA	2014AM-3343	Agona	0	0	0	0	0	0	0	0	0	0	0			2-2-204-3-0-0	1	0.000124425
SAL_HA1542AA	PNUSAS004814	Agona	0	0	0	0	0	0	0	0	0	0	0			2-2-4-415-0-0	1	0.000124425
SAL_GA9739AA	FDA00004800	Agona	0	0	0	0	0	0	0	0	0	0	0			2-2-208-3-0-0	2	0.000248849
SAL_GA9816AA	PNUSAS003969	Agona	0	0	0	0	0	0	0	0	0	0	0			2-2-210-3-0-0	1	0.000124425
SAL_HA2793AA	ADRDL-16-6327	Agona	0	0	0	0	0	0	0	0	0	0	0			0-0-4-3-0-0	1	0.000124425
SAL_HA2874AA	PNUSAS005131	Agona	0	0	0	0	0	0	0	0	0	0	0			2-2-61-57-0-0	1	0.000124425
SAL_HA3074AA	PNUSAS004846	Agona	0	0	0	0	0	0	0	0	0	0	0			1-1-1-4-1-1	8217	0.228421316
SAL_HA3083AA	PNUSAS004790	Agona	0	0	0	0	0	0	0	0	0	0	0			0-0-0-0-0-0	19886	0.552803492
SAL_HA3132AA	PNUSAS005047	Agona	0	0	0	0	0	0	0	0	0	0	0			4-1-1-5-1-1	1	2.77986E-05
SAL_HA2879AA	PNUSAS005130	Agona	0	0	0	0	0	0	0	0	0	0	0			1-9-1-4-1-1	77	0.002140494
SAL_HA2877AA	PNUSAS005135	Agona	0	0	0	0	0	0	0	0	0	0	0			1-1-1-1-1-1	1288	0.035804631
SAL_HA2872AA	PNUSAS005136	Agona	0	0	0	0	0	0	0	0	0	0	0			1-1-1-29-0-0	2	5.55973E-05
SAL_HA2779AA	ADRDL-16-8871	Agona	0	0	0	0	0	0	0	0	0	0	0			1-1-1-29-1-1	56	0.001556723
SAL_HA2356AA	L03781-15	Agona	0	0	0	0	0	0	0	0	0	0	0			1-10-1-4-1-5	68	0.001890307
SAL_HA2220AA	S05105-15	Agona	0	0	0	0	0	0	0	0	0	0	0			1-1-1-31-1-1	25	0.000694966
SAL_HA2224AA	S05085-15	Agona	0	0	0	0	0	0	0	0	0	0	0			1-1-1-16-1-1	348	0.009673922
SAL_HA2227AA	S04970-15	Agona	0	0	0	0	0	0	0	0	0	0	0			1-1-1-32-1-1	91	0.002529675
SAL_HA2229AA	S04832-15	Agona	0	0	0	0	0	0	0	0	0	0	0			1-1-1-1-9-1	59	0.001640119
SAL_HA2245AA	S04766-15	Agona	0	0	0	0	0	0	0	0	0	0	0			1-1-1-34-1-7	5	0.000138993
SAL_HA2170AA	L02123-15	Agona	0	0	0	0	0	0	0	0	0	0	0			1-1-1-4-10-1	2	5.55973E-05
SAL_HA2156AA	S06132-15	Agona	0	0	0	0	0	0	0	0	0	0	0			1-1-1-31-1-8	6	0.000166792
SAL_HA2158AA	S06127-15	Agona	0	0	0	0	0	0	0	0	0	0	0			1-1-1-36-1-1	57	0.001584522
SAL_HA1979AA	S02704-13	Agona	0	0	0	0	0	0	0	0	0	0	0			1-1-1-69-1-1	1	2.77986E-05
SAL_HA2381AA	L00276-15	Agona	0	0	0	0	0	0	0	0	0	0	0			1-1-1-71-1-1	1	2.77986E-05
SAL_HA3531AA	IFSH00036	Agona	0	0	0	0	0	0	0	0	0	0	0			1-1-1-4-1-12	52	0.001445529
SAL_HA4085AA	PNUSAS005211	Agona	0	0	0	0	0	0	0	0	0	0	0			1-1-1-0-1-1	3729	0.103661079
SAL_HA4054AA	PNUSAS005053	Agona	0	0	0	0	0	0	0	0	0	0	0			0-0-0-0-0-1	16	0.000444778
SAL_HA4035AA	PNUSAS005210	Agona	0	0	0	0	0	0	0	0	0	0	0			0-0-0-0-1-1	169	0.004697968
SAL_HA4245AA	PNUSAS005379	Agona	0	0	0	0	0	0	0	0	0	0	0			1-1-1-0-0-0	10	0.000277986
SAL_HA4360AA	FDA00000484	Agona	0	0	0	0	0	0	0	0	0	0	0			1-17-47-77-19-3	3	8.33959E-05
SAL_HA4741AA	FSIS1608547	Agona	0	0	0	0	0	0	0	0	0	0	0			1-1-1-78-1-1	129	0.003586023
SAL_HA4752AA	FSIS1608384	Agona	0	0	0	0	0	0	0	0	0	0	0			1-1-0-4-1-1	207	0.005754316
SAL_HA4320AA	CFSAN048477	Agona	0	0	0	0	0	0	0	0	0	0	0			1-1-1-30-1-1	25	0.000694966
SAL_HA4796AA	900203	Agona	0	0	0	0	0	0	0	0	0	0	0			1-1-1-4-0-0	158	0.004392183
SAL_HA4801AA	557605	Agona	0	0	0	0	0	0	0	0	0	0	0			1-18-1-4-1-1	1	2.77986E-05
SAL_HA4755AA	10-0327	Agona	0	0	0	0	0	0	0	0	0	0	0			1-1-0-0-0-0	21	0.000583771

SAL_HA4893AA	45231	Agona	0	0	0	0	0	0	0	0	0	0	0	
SAL_HA4923AA	1951766	Agona	0	0	0	0	0	0	0	0	0	0	0	
SAL_HA4921AA	1950671	Agona	0	0	0	0	0	0	0	0	0	0	0	
SAL_HA5215AA	217291	Agona	0	0	0	0	0	0	0	0	0	0	0	
SAL_HA5213AA	1001801	Agona	0	0	0	0	0	0	0	0	0	0	0	
SAL_HA5135AA	12-0859	Agona	0	0	0	0	0	0	0	0	0	0	0	
SAL_HA5174AA	1347321	Agona	0	0	0	0	0	0	0	0	0	0	0	
SAL_HA5073AA	L00430-15	Agona	0	0	0	0	0	0	0	0	0	0	0	
SAL_HA5042AA	2403267	Agona	0	0	0	0	0	0	0	0	0	0	0	
SAL_HA5052AA	444105	Agona	0	0	0	0	0	0	0	0	0	0	0	
SAL_HA5070AA	13-0431	Agona	0	0	0	0	0	0	0	0	0	0	0	
SAL_HA5030AA	471864	Agona	0	0	0	0	0	0	0	0	0	0	0	
SAL_HA5445AA	13-4758	Agona	0	0	0	0	0	0	0	0	0	0	0	
SAL_HA5425AA	13-2460	Agona	0	0	0	0	0	0	0	0	0	0	0	
SAL_HA5442AA	13-3157	Agona	0	0	0	0	0	0	0	0	0	0	0	
SAL_HA5424AA	14-0656	Agona	0	0	0	0	0	0	0	0	0	0	0	
SAL_HA5435AA	13-5657	Agona	0	0	0	0	0	0	0	0	0	0	0	
SAL_HA5047AA	505833	Agona	0	0	0	0	0	0	0	0	0	0	0	
SAL_HA5725AA	PNUSAS005307	Agona	0	0	0	0	0	0	0	0	0	0	0	
SAL_HA3398AA	wt14	Agona	0	0	0	0	0	0	0	0	0	0	0	
SAL_HA4086AA	PNUSAS005165	Agona	0	0	0	0	0	0	0	0	0	0	0	
SAL_HA2342AA	S01316-15	Agona	0	0	0	0	0	0	0	0	0	0	0	
SAL_HA2353AA	L04283-15	Agona	0	0	0	0	0	0	0	0	0	0	0	
SAL_HA2357AA	L03779-15	Agona	0	0	0	0	0	0	0	0	0	0	0	
SAL_HA2367AA	L00984-15	Agona	0	0	0	0	0	0	0	0	0	0	0	
SAL_HA2369AA	L00866-15	Agona	0	0	0	0	0	0	0	0	0	0	0	
SAL_HA2371AA	L00805-15	Agona	0	0	0	0	0	0	0	0	0	0	0	
SAL_HA2377AA	L00405-15	Agona	0	0	0	0	0	0	0	0	0	0	0	
SAL_HA2378AA	L00380-15	Agona	0	0	0	0	0	0	0	0	0	0	0	
SAL_HA2383AA	L00184-15	Agona	0	0	0	0	0	0	0	0	0	0	0	
SAL_HA2382AA	L00188-15	Agona	0	0	0	0	0	0	0	0	0	0	0	
SAL_HA2384AA	L00183-15	Agona	0	0	0	0	0	0	0	0	0	0	0	
SAL_HA2385AA	L00182-15	Agona	0	0	0	0	0	0	0	0	0	0	0	
SAL_HA2388AA	L00009-15	Agona	0	0	0	0	0	0	0	0	0	0	0	
SAL_HA2442AA	FDA00010869	Agona	0	0	0	0	0	0	0	0	0	0	0	
SAL_HA2436AA	FDA00010867	Agona	0	0	0	0	0	0	0	0	0	0	0	
SAL_HA6471AA	FSIS1609104	Agona	0	0	0	0	0	0	0	0	0	0	0	
SAL_HA2633AA	FDA00010805	Agona	0	0	0	0	0	0	0	0	0	0	0	
SAL_HA4149AA	OH-1301245	Agona	0	0	0	0	0	0	0	0	0	0	0	
SAL_HA4043AA	PNUSAS005214	Agona	0	0	0	0	0	0	0	0	0	0	0	
SAL_HA6676AA	CFSAN056763	Agona	0	0	0	0	0	0	0	0	0	0	0	
SAL_HA6672AA	CFSAN056761	Agona	0	0	0	0	0	0	0	0	0	0	0	
SAL_HA6673AA	CFSAN056760	Agona	0	0	0	0	0	0	0	0	0	0	0	
SAL_HA6666AA	CFSAN056759	Agona	0	0	0	0	0	0	0	0	0	0	0	
SAL_HA6665AA	CFSAN056758	Agona	0	0	0	0	0	0	0	0	0	0	0	
SAL_HA6942AA	FSIS1608902	Agona	0	0	0	0	0	0	0	0	0	0	0	
SAL_HA6924AA	PNUSAS006096	Agona	0	0	0	0	0	0	0	0	0	0	0	
SAL_HA7197AA	CFSAN056764	Agona	0	0	0	0	0	0	0	0	0	0	0	
SAL_HA6177AA	PNUSAS005344	Agona	0	0	0	0	0	0	0	0	0	0	0	
SAL_HA7630AA	CFSAN056822	Agona	0	0	0	0	0	0	0	0	0	0	0	
SAL_HA7647AA	FSIS1609220	Agona	0	0	0	0	0	0	0	0	0	0	0	
SAL_HA8714AA	OH-16-20695	Agona	0	0	0	0	0	0	0	0	0	0	0	
SAL_HA8814AA	FSIS1609543	Agona	0	0	0	0	0	0	0	0	0	0	0	
SAL_HA9192AA	PNUSAS006674	Agona	0	0	0	0	0	0	0	0	0	0	0	
SAL_HA9181AA	PNUSAS006675	Agona	0	0	0	0	0	0	0	0	0	0	0	
SAL_HA9441AA	CFSAN057822	Agona	0	0	0	0	0	0	0	0	0	0	0	
SAL_HA9708AA	CFSAN058811	Agona	0	0	0	0	0	0	0	0	0	0	0	
SAL_HA9714AA	CFSAN058812	Agona	0	0	0	0	0	0	0	0	0	0	0	
SAL_IA0536AA	PNUSAS007423	Agona	0	0	0	0	0	0	0	0	0	0	0	
SAL_IA0849AA	168511	Agona	0	0	0	0	0	0	0	0	0	0	0	
SAL_IA2064AA	FSIS1609519	Agona	0	0	0	0	0	0	0	0	0	0	0	
SAL_IA2106AA	PNUSAS005772	Agona	0	0	0	0	0	0	0	0	0	0	0	
SAL_IA1091AA	285861	Agona	0	0	0	0	0	0	0	0	0	0	0	
SAL_IA1503AA	287789	Agona	0	0	0	0	0	0	0	0	0	0	0	
SAL_IA2329AA	FSIS1609762	Agona	0	0	0	0	0	0	0	0	0	0	0	
SAL_IA2372AA	FSIS1609388	Agona	0	0	0	0	0	0	0	0	0	0	0	
SAL_IA2509AA	FDA00011194	Agona	0	0	0	0	0	0	0	0	0	0	0	
SAL_IA2508AA	FDA00011192	Agona	0	0	0	0	0	0	0	0	0	0	0	
SAL_IA2511AA	FDA00011191	Agona	0	0	0	0	0	0	0	0	0	0	0	
SAL_IA3403AA	285669	Agona	0	0	0	0	0	0	0	0	0	0	0	
SAL_IA3298AA	285709	Agona	0	0	0	0	0	0	0	0	0	0	0	
SAL_IA2749AA	CFSAN059251	Agona	0	0	0	0	0	0	0	0	0	0	0	
SAL_IA2750AA	CFSAN059250	Agona	0	0	0	0	0	0	0	0	0	0	0	

1-19-1-4-1-1	17	0.000472577
25-1-1-16-1-1	44	0.00122314
1-1-1-87-1-1	55	0.001528924
1-11-1-4-1-1	81	0.002251689
1-1-1-4-1-13	9	0.000250188
1-1-1-4-1-0	12	0.000333584
1-1-1-4-1-14	52	0.001445529
1-20-1-4-1-1	1	2.77986E-05
1-1-52-4-1-1	1	2.77986E-05
1-1-1-99-1-1	1	2.77986E-05
1-1-1-0-20-1	2	5.55973E-05
0-0-1-4-1-1	32	0.000889556
1-1-1-101-1-1	1	2.77986E-05
1-1-1-4-1-16	1	2.77986E-05
1-10-1-0-1-5	33	0.000917355
1-21-1-0-1-1	2	5.55973E-05
0-0-0-4-1-1	23	0.000639368
1-1-0-0-1-1	14	0.000389181
1-1-0-0-1-17	2	5.55973E-05
1-1-0-4-1-17	10	0.000277986
1-19-1-4-0-0	1	2.77986E-05
1-1-1-0-1-0	5	0.000138993
1-1-1-30-1-0	2	5.55973E-05
1-1-1-16-0-0	7	0.00019459
1-1-1-110-1-1	1	2.77986E-05
1-1-1-0-9-1	49	0.001362133
1-1-1-0-1-18	1	2.77986E-05
25-1-1-0-1-1	10	0.000277986
1-1-1-4-1-19	14	0.000389181
1-1-64-4-1-1	1	2.77986E-05
1-24-1-4-1-1	1	2.77986E-05
1-1-65-16-1-1	1	2.77986E-05
1-1-66-4-1-1	3	8.33959E-05
1-1-1-4-1-21	3	8.33959E-05
1-0-1-4-1-1	11	0.000305785
1-10-1-130-1-5	5	0.000138993
1-1-1-4-1-22	10	0.000277986
1-1-1-4-0-1	4	0.000111195
1-10-1-4-1-0	2	5.55973E-05
1-1-1-0-0-1	5	0.000138993
1-1-1-132-1-1	3	8.33959E-05
1-28-1-4-1-1	1	2.77986E-05
1-10-1-135-1-5	1	2.77986E-05
1-1-1-136-1-1	22	0.00061157
1-1-1-4-1-23	1	2.77986E-05
29-1-1-0-1-1	1	2.77986E-05
1-29-0-0-0-0	4	0.000111195
1-1-1-1-1-0	10	0.000277986
1-1-1-4-27-1	1	2.77986E-05
1-11-1-139-1-1	6	0.000166792
1-1-1-4-1-24	1	2.77986E-05
29-1-1-4-1-1	5	0.000138993
1-32-1-16-1-1	1	2.77986E-05
30-1-1-78-1-1	1	2.77986E-05
31-1-1-1-1-1	2	5.55973E-05
1-1-1-4-28-1	2	5.55973E-05
1-1-1-141-1-1	1	2.77986E-05
1-11-1-0-1-1	9	0.000250188
1-34-1-4-1-25	1	2.77986E-05
1-1-1-142-1-1	1	2.77986E-05
1-1-1-139-1-1	8	0.000222389
1-9-1-4-0-0	1	2.77986E-05
13-1-1-33-1-1	7	0.00019459
1-1-81-147-1-1	1	2.77986E-05
1-1-1-148-1-1	1	2.77986E-05
1-9-1-0-1-1	34	0.000945153
1-36-1-4-1-1	1	2.77986E-05
1-1-1-35-1-1	5	0.000138993
0-0-1-0-1-1	1	2.77986E-05
1-1-1-162-0-0	1	2.77986E-05
1-1-1-166-1-1	1	2.77986E-05
1-1-1-169-1-1	3	8.33959E-05
1-1-1-4-1-18	1	2.77986E-05

SAL_IA2755AA	CFSAN059254	Agona	0	0	0	0	0	0	0	0	0	0	0			1-1-1-36-1-0	1	2.77986E-05
SAL_IA2726AA	CFSAN059190	Agona	0	0	0	0	0	0	0	0	0	0	0			0-0-0-4-1-17	3	8.33959E-05
SAL_IA2694AA	CFSAN059255	Agona	0	0	0	0	0	0	0	0	0	0	0			1-1-1-4-32-1	4	0.000111195
SAL_IA2624AA	CFSAN059239	Agona	0	0	0	0	0	0	0	0	0	0	0			1-39-1-1-9-1	2	5.55973E-05
SAL_IA2607AA	PNUSAS007395	Agona	0	0	0	0	0	0	0	0	0	0	0			1-1-0-1-1-1	3	8.33959E-05
SAL_IA2625AA	CFSAN059238	Agona	0	0	0	0	0	0	0	0	0	0	0			1-1-94-4-1-1	1	2.77986E-05
SAL_IA2630AA	CFSAN059240	Agona	0	0	0	0	0	0	0	0	0	0	0			36-1-1-4-1-1	2	5.55973E-05
SAL_IA2703AA	CFSAN059256	Agona	0	0	0	0	0	0	0	0	0	0	0			25-1-1-16-0-0	3	8.33959E-05
SAL_IA3169AA	285923	Agona	0	0	0	0	0	0	0	0	0	0	0			1-1-1-30-0-1	14	0.000389181
SAL_IA3786AA	PNUSAS007343	Agona	0	0	0	0	0	0	0	0	0	0	0			1-29-1-4-1-1	2	5.55973E-05
SAL_IA3703AA	FSIS1710131	Agona	0	0	0	0	0	0	0	0	0	0	0			1-1-1-188-1-1	1	2.77986E-05
SAL_IA4706AA	FSIS1710000	Agona	0	0	0	0	0	0	0	0	0	0	0			1-10-1-4-33-5	2	5.55973E-05
SAL_IA4959AA	CFSAN059279	Agona	0	0	0	0	0	0	0	0	0	0	0			1-1-1-196-1-1	1	2.77986E-05
SAL_IA4964AA	CFSAN059285	Agona	0	0	0	0	0	0	0	0	0	0	0			1-1-1-4-1-26	1	2.77986E-05
SAL_IA5155AA	CFSAN058124	Agona	0	0	0	0	0	0	0	0	0	0	0			1-1-1-33-1-1	100	0.002779863
SAL_IA5060AA	MS160315	Agona	0	0	0	0	0	0	0	0	0	0	0			1-1-1-4-1-3	7	0.00019459
SAL_IA5068AA	MS160297	Agona	0	0	0	0	0	0	0	0	0	0	0			1-1-1-203-34-1	7	0.00019459
SAL_IA4948AA	OH-16-24375	Agona	0	0	0	0	0	0	0	0	0	0	0			1-1-0-16-1-1	2	5.55973E-05
SAL_IA5915AA	MS160391	Agona	0	0	0	0	0	0	0	0	0	0	0			1-0-1-136-1-1	1	2.77986E-05
SAL_IA5911AA	MS160396	Agona	0	0	0	0	0	0	0	0	0	0	0			1-1-1-139-0-0	1	2.77986E-05
SAL_IA5868AA	PNUSAS007923	Agona	0	0	0	0	0	0	0	0	0	0	0			1-1-1-4-1-27	1	2.77986E-05
SAL_IA5886AA	MS160181_P	Agona	0	0	0	0	0	0	0	0	0	0	0			1-1-1-32-0-0	2	5.55973E-05
SAL_IA5887AA	MS160181_N	Agona	0	0	0	0	0	0	0	0	0	0	0			1-1-1-1-35-1	1	2.77986E-05
SAL_IA6334AA	FSIS1709967	Agona	0	0	0	0	0	0	0	0	0	0	0			1-10-1-4-0-0	2	5.55973E-05
SAL_IA6228AA	PNUSAS007911	Agona	0	0	0	0	0	0	0	0	0	0	0			1-1-1-4-36-1	1	2.77986E-05
SAL_IA5559AA	FSIS1710238	Agona	0	0	0	0	0	0	0	0	0	0	0			1-21-1-4-1-1	3	8.33959E-05
SAL_IA5607AA	PNUSAS008086	Agona	0	0	0	0	0	0	0	0	0	0	0			26-1-1-4-1-1	1	2.77986E-05
SAL_IA5422AA	PNUSAS008459	Agona	0	0	0	0	0	0	0	0	0	0	0			1-1-1-4-37-1	1	2.77986E-05
SAL_IA6405AA	FSIS1710507	Agona	0	0	0	0	0	0	0	0	0	0	0			1-1-1-217-1-1	1	2.77986E-05
SAL_IA7194AA	PNUSAS007346	Agona	0	0	0	0	0	0	0	0	0	0	0			1-1-1-4-38-1	7	0.00019459
SAL_IA7076AA	CFSAN059787	Agona	0	0	0	0	0	0	0	0	0	0	0			1-1-1-78-0-0	1	2.77986E-05
SAL_IA7350AA	S/20170055	Agona	0	0	0	0	0	0	0	0	0	0	0			30-1-1-78-40-1	11	0.000305785
SAL_IA7457AA	PNUSAS008811	Agona	0	0	0	0	0	0	0	0	0	0	0			1-1-1-4-0-29	3	8.33959E-05
SAL_IA7404AA	OH-17-1272B	Agona	0	0	0	0	0	0	0	0	0	0	0			1-1-1-0-0-29	3	8.33959E-05
SAL_IA7573AA	PNUSAS009140	Agona	0	0	0	0	0	0	0	0	0	0	0			1-10-1-4-41-5	3	8.33959E-05
SAL_IA7568AA	PNUSAS009134	Agona	0	0	0	0	0	0	0	0	0	0	0			1-1-1-4-42-1	1	2.77986E-05
SAL_IA8411AA	PNUSAS008766	Agona	0	0	0	0	0	0	0	0	0	0	0			1-1-1-4-43-1	1	2.77986E-05
SAL_IA8393AA	PNUSAS008629	Agona	0	0	0	0	0	0	0	0	0	0	0			1-1-1-223-1-1	1	2.77986E-05
SAL_IA8887AA	FDA00004598	Agona	0	0	0	0	0	0	0	0	0	0	0			1-47-1-4-1-1	2	5.55973E-05
SAL_IA8964AA	ADRDL-901	Agona	0	0	0	0	0	0	0	0	0	0	0			1-1-1-4-1-30	1	2.77986E-05
SAL_IA8957AA	ADRDL-893	Agona	0	0	0	0	0	0	0	0	0	0	0			1-1-1-0-1-12	21	0.000583771
SAL_IA8967AA	ADRDL-905	Agona	0	0	0	0	0	0	0	0	0	0	0			1-1-1-4-1-31	20	0.000555973
SAL_IA9078AA	S/20170093	Agona	0	0	0	0	0	0	0	0	0	0	0			1-1-1-0-1-32	1	2.77986E-05
SAL_IA9412AA	PNUSAS008234	Agona	0	0	0	0	0	0	0	0	0	0	0			1-1-1-1-1-32	2	5.55973E-05
SAL_IA9318AA	PNUSAS006969	Agona	0	0	0	0	0	0	0	0	0	0	0			1-1-1-4-47-1	1	2.77986E-05
SAL_IA9272AA	PNUSAS009545	Agona	0	0	0	0	0	0	0	0	0	0	0			1-1-1-232-1-1	1	2.77986E-05
SAL_IA9355AA	PNUSAS009694	Agona	0	0	0	0	0	0	0	0	0	0	0			1-1-115-4-1-1	15	0.000416979
SAL_IA9793AA	FSIS1711037	Agona	0	0	0	0	0	0	0	0	0	0	0			1-1-1-4-1-33	1	2.77986E-05
SAL_JA0042AA	FSIS1711057	Agona	0	0	0	0	0	0	0	0	0	0	0			62-1-1-4-1-1	1	2.77986E-05
SAL_JA0217AA	A3-R	Agona	0	0	0	0	0	0	0	0	0	0	0			1-0-142-0-1-1	1	2.77986E-05
SAL_GA6889AA	A01	Agona	0	0	0	0	0	0	0	0	0	0	0			1-56-1-4-1-1	1	2.77986E-05
SAL_GA6887AA	A02	Agona	0	0	0	0	0	0	0	0	0	0	0			1-1-1-33-1-12	1	2.77986E-05
SAL_GA6890AA	A05	Agona	0	0	0	0	0	0	0	0	0	0	0			1-57-1-4-1-1	7	0.00019459
SAL_JA0218AA	A8-R	Agona	0	0	0	0	0	0	0	0	0	0	0			1-1-1-0-1-14	7	0.00019459
SAL_GA6893AA	A08	Agona	0	0	0	0	0	0	0	0	0	0	0			1-1-1-269-0-0	2	5.55973E-05
SAL_GA7999AA	A06	Agona	0	0	0	0	0	0	0	0	0	0	0			1-1-149-4-1-1	2	5.55973E-05
SAL_GA6888AA	A03	Agona	0	0	0	0	0	0	0	0	0	0	0			0-1-1-16-1-1	1	2.77986E-05
SAL_GA6894AA	A07	Agona	0	0	0	0	0	0	0	0	0	0	0		**Typhimurium**	1-1-1-16-0-1	2	5.55973E-05
SAL_GA6898AA	A13	Agona	0	0	0	0	0	0	0	0	0	0	0			1-0-1-16-1-1	17	0.000472577
SAL_GA6895AA	A11	Agona	0	0	0	0	0	0	0	0	0	0	0			1-0-1-0-1-1	8	0.000222389
SAL_GA6892AA	A09	Agona	0	0	0	0	0	0	0	0	0	0	0			0-1-1-0-1-1	1	2.77986E-05
SAL_JA0219AA	A10-R	Agona	0	0	0	0	0	0	0	0	0	0	0			1-1-1-16-1-0	1	2.77986E-05
SAL_GA6899AA	A12	Agona	0	0	0	0	0	0	0	0	0	0	0			1-1-1-4-50-1	9	0.000250188
SAL_GA6906AA	A23	Agona	0	0	0	0	0	0	0	0	0	0	0			1-1-1-274-1-1	1	2.77986E-05
SAL_GA6905AA	A21	Agona	0	0	0	0	0	0	0	0	0	0	0			0-0-0-0-1-0	1	2.77986E-05
SAL_GA6902AA	A18	Agona	0	0	0	0	0	0	0	0	0	0	0			1-1-1-1-0-0	2	5.55973E-05
SAL_GA6901AA	A15	Agona	0	0	0	0	0	0	0	0	0	0	0			1-1-1-275-1-1	1	2.77986E-05
SAL_GA6904AA	A20	Agona	0	0	0	0	0	0	0	0	0	0	0			1-1-107-4-1-1	4	0.000111195
SAL_GA6903AA	A19	Agona	0	0	0	0	0	0	0	0	0	0	0			1-1-1-196-1-22	2	5.55973E-05
SAL_GA6907AA	A22	Agona	0	0	0	0	0	0	0	0	0	0	0			1-1-1-31-0-0	1	2.77986E-05
SAL_GA6909AA	A24	Agona	0	0	0	0	0	0	0	0	0	0	0			1-1-1-281-1-1	1	2.77986E-05
SAL_GA6912AA	A26	Agona	0	0	0	0	0	0	0	0	0	0	0			1-1-1-4-9-1	1	2.77986E-05
SAL_GA6908AA	A25	Agona	0	0	0	0	0	0	0	0	0	0	0			1-1-1-284-1-1	4	0.000111195

SAL_GA6910AA	A27	Agona	0	0	0	0	0	0	0	0	0	0	0
SAL_GA6916AA	A33	Agona	0	0	0	0	0	0	0	0	0	0	0
SAL_GA6913AA	A30	Agona	0	0	0	0	0	0	0	0	0	0	0
SAL_GA6911AA	A28	Agona	0	0	0	0	0	0	0	0	0	0	0
SAL_GA6919AA	A34	Agona	0	0	0	0	0	0	0	0	0	0	0
SAL_GA6914AA	A31	Agona	0	0	0	0	0	0	0	0	0	0	0
SAL_GA6917AA	A35	Agona	0	0	0	0	0	0	0	0	0	0	0
SAL_GA6922AA	A39	Agona	0	0	0	0	0	0	0	0	0	0	0
SAL_GA6915AA	A32	Agona	0	0	0	0	0	0	0	0	0	0	0
SAL_GA6920AA	A37	Agona	0	0	0	0	0	0	0	0	0	0	0
SAL_GA6925AA	A42	Agona	0	0	0	0	0	0	0	0	0	0	0
SAL_GA6921AA	A38	Agona	0	0	0	0	0	0	0	0	0	0	0
SAL_GA6918AA	A36	Agona	0	0	0	0	0	0	0	0	0	0	0
SAL_GA6923AA	A40	Agona	0	0	0	0	0	0	0	0	0	0	0
SAL_JA0223AA	Sa-A41	Agona	0	0	0	0	0	0	0	0	0	0	0
SAL_JA0222AA	Sa-A17	Agona	0	0	0	0	0	0	0	0	0	0	0
SAL_JA0221AA	Sa-A29	Agona	0	0	0	0	0	0	0	0	0	0	0
SAL_JA0220AA	Sa-A04	Agona	0	0	0	0	0	0	0	0	0	0	0
SAL_JA1134AA	10B08061A-1	Agona	0	0	0	0	0	0	0	0	0	0	0
SAL_JA1077AA	PNUSAS007063	Agona	0	0	0	0	0	0	0	0	0	0	0
SAL_JA1591AA	PNUSAS010781	Agona	0	0	0	0	0	0	0	0	0	0	0
SAL_JA1528AA	PNUSAS010814	Agona	0	0	0	0	0	0	0	0	0	0	0
SAL_JA1513AA	PNUSAS010819	Agona	0	0	0	0	0	0	0	0	0	0	0
SAL_JA1829AA	8-25-20170165	Agona	0	0	0	0	0	0	0	0	0	0	0
SAL_JA1016AA	PNUSAS011222	Agona	0	0	0	0	0	0	0	0	0	0	0
SAL_JA0932AA	PNUSAS009349	Agona	0	0	0	0	0	0	0	0	0	0	0
SAL_JA0477AA	ADRDL-1039	Agona	0	0	0	0	0	0	0	0	0	0	0
SAL_JA0271AA	PNUSAS010196	Agona	0	0	0	0	0	0	0	0	0	0	0
SAL_JA1665AA	PNUSAS011306	Agona	0	0	0	0	0	0	0	0	0	0	0
SAL_JA1654AA	PNUSAS011304	Agona	0	0	0	0	0	0	0	0	0	0	0
SAL_JA0496AA	PNUSAS010746	Agona	0	0	0	0	0	0	0	0	0	0	0
SAL_JA1711AA	PNUSAS011680	Agona	0	0	0	0	0	0	0	0	0	0	0
SAL_JA1989AA	PNUSAS011364	Agona	0	0	0	0	0	0	0	0	0	0	0
SAL_JA0418AA	PNUSAS010654	Agona	0	0	0	0	0	0	0	0	0	0	0
SAL_JA2620AA	PNUSAS011748	Agona	0	0	0	0	0	0	0	0	0	0	0
SAL_JA2797AA	FSIS1711019	Agona	0	0	0	0	0	0	0	0	0	0	0
SAL_GA6934AA	A53	Agona	0	0	0	0	0	0	0	0	0	0	0
SAL_GA6939AA	A55	Agona	0	0	0	0	0	0	0	0	0	0	0
SAL_GA6941AA	A60	Agona	0	0	0	0	0	0	0	0	0	0	0
SAL_GA6947AA	A66	Agona	0	0	0	0	0	0	0	0	0	0	0
SAL_GA6952AA	A69	Agona	0	0	0	0	0	0	0	0	0	0	0
SAL_GA6926AA	A44	Agona	0	0	0	0	0	0	0	0	0	0	0
SAL_GA6924AA	A43	Agona	0	0	0	0	0	0	0	0	0	0	0
SAL_GA6930AA	A49	Agona	0	0	0	0	0	0	0	0	0	0	0
SAL_GA6955AA	A73	Agona	0	0	0	0	0	0	0	0	0	0	0
SAL_GA6943AA	A61	Agona	0	0	0	0	0	0	0	0	0	0	0
SAL_GA6950AA	A68	Agona	0	0	0	0	0	0	0	0	0	0	0
SAL_GA6942AA	A62	Agona	0	0	0	0	0	0	0	0	0	0	0
SAL_GA6948AA	A67	Agona	0	0	0	0	0	0	0	0	0	0	0
SAL_GA6949AA	A65	Agona	0	0	0	0	0	0	0	0	0	0	0
SAL_GA6929AA	A47	Agona	0	0	0	0	0	0	0	0	0	0	0
SAL_GA6932AA	A50	Agona	0	0	0	0	0	0	0	0	0	0	0
SAL_GA6937AA	A57	Agona	0	0	0	0	0	0	0	0	0	0	0
SAL_GA6935AA	A52	Agona	0	0	0	0	0	0	0	0	0	0	0
SAL_GA6927AA	A45	Agona	0	0	0	0	0	0	0	0	0	0	0
SAL_GA6940AA	A59	Agona	0	0	0	0	0	0	0	0	0	0	0
SAL_GA6928AA	A46	Agona	0	0	0	0	0	0	0	0	0	0	0
SAL_GA6945AA	A64	Agona	0	0	0	0	0	0	0	0	0	0	0
SAL_GA6946AA	A63	Agona	0	0	0	0	0	0	0	0	0	0	0
SAL_GA6951AA	A70	Agona	0	0	0	0	0	0	0	0	0	0	0
SAL_GA6953AA	A71	Agona	0	0	0	0	0	0	0	0	0	0	0
SAL_GA6936AA	A54	Agona	0	0	0	0	0	0	0	0	0	0	0
SAL_GA6938AA	A56	Agona	0	0	0	0	0	0	0	0	0	0	0
SAL_GA6944AA	A58	Agona	0	0	0	0	0	0	0	0	0	0	0
SAL_GA6954AA	A72	Agona	0	0	0	0	0	0	0	0	0	0	0
SAL_GA6933AA	A48	Agona	0	0	0	0	0	0	0	0	0	0	0
SAL_JA3342AA	PNUSAS012254	Agona	0	0	0	0	0	0	0	0	0	0	0
SAL_JA3636AA	FDA00004022	Agona	0	0	0	0	0	0	0	0	0	0	0
SAL_JA3686AA	FSIS1700614	Agona	0	0	0	0	0	0	0	0	0	0	0
SAL_JA3600AA	FSIS1700444	Agona	0	0	0	0	0	0	0	0	0	0	0
SAL_JA3971AA	PNUSAS012797	Agona	0	0	0	0	0	0	0	0	0	0	0
SAL_JA3962AA	PNUSAS012801	Agona	0	0	0	0	0	0	0	0	0	0	0
SAL_JA4224AA	FSIS1700563	Agona	0	0	0	0	0	0	0	0	0	0	0

1-1-155-4-1-1	5	0.000138993
1-1-156-4-1-1	1	2.77986E-05
1-1-1-288-1-1	2	5.55973E-05
1-1-160-4-1-1	1	2.77986E-05
1-1-1-4-52-1	2	5.55973E-05
1-62-1-4-1-1	1	2.77986E-05
1-1-162-4-1-1	1	2.77986E-05
1-1-1-295-1-1	1	2.77986E-05
1-1-1-4-1-34	1	2.77986E-05
1-1-163-4-1-1	2	5.55973E-05
1-1-1-298-1-1	2	5.55973E-05
1-1-1-4-1-17	6	0.000166792
1-1-1-0-38-1	7	0.00019459
1-63-1-4-1-1	1	2.77986E-05
1-1-1-299-1-1	3	8.33959E-05
1-1-1-78-54-1	1	2.77986E-05
1-1-1-4-1-36	1	2.77986E-05
1-1-1-4-1-9	2	5.55973E-05
1-1-1-4-1-37	2	5.55973E-05
1-1-165-4-1-1	2	5.55973E-05
1-10-1-0-33-5	3	8.33959E-05
1-1-166-1-1-1	2	5.55973E-05
68-1-1-4-1-1	1	2.77986E-05
1-1-1-301-0-0	1	2.77986E-05
1-65-1-4-1-1	1	2.77986E-05
1-1-1-269-1-1	3	8.33959E-05
1-1-1-310-1-1	1	2.77986E-05
1-66-1-1-1-1	1	2.77986E-05
1-1-1-311-1-1	1	2.77986E-05
1-1-171-4-1-1	3	8.33959E-05
72-1-1-4-1-1	1	2.77986E-05
0-1-0-4-0-0	1	2.77986E-05
1-1-1-4-1-6	1	2.77986E-05
1-1-1-0-1-7	2	5.55973E-05
14-12-23-37-11-0	1	2.77986E-05
42-1-1-4-1-1	1	2.77986E-05
1-1-1-2-2-1	1	2.77986E-05
1-1-1-4-44-1	1	2.77986E-05
1-1-1-4-1-38	1	2.77986E-05
1-1-1-317-1-1	1	2.77986E-05
1-1-1-4-1-40	1	2.77986E-05
1-11-1-33-1-1	3	8.33959E-05
1-9-1-16-1-1	2	5.55973E-05
1-1-177-4-1-1	1	2.77986E-05
1-71-1-4-1-1	1	2.77986E-05
1-11-1-16-1-1	1	2.77986E-05
1-1-1-327-1-1	1	2.77986E-05
1-19-1-0-1-1	7	0.00019459
1-11-1-31-1-1	1	2.77986E-05
1-1-182-4-1-1	1	2.77986E-05
1-1-0-0-0-1	1	2.77986E-05
1-74-1-4-1-1	2	5.55973E-05
1-1-1-330-1-1	1	2.77986E-05
1-1-1-331-1-1	1	2.77986E-05
1-10-1-332-1-5	1	2.77986E-05
1-1-1-333-0-0	1	2.77986E-05
1-1-1-302-1-1	1	2.77986E-05
1-1-183-4-1-1	1	2.77986E-05
1-1-1-297-1-1	2	5.55973E-05
1-1-1-337-1-1	1	2.77986E-05
13-1-1-0-1-1	6	0.000166792
1-1-1-344-1-1	2	5.55973E-05
80-1-1-4-1-1	1	2.77986E-05
1-1-1-0-32-1	3	8.33959E-05
1-1-1-356-1-1	1	2.77986E-05
1-1-1-0-66-1	1	2.77986E-05
1-11-1-269-1-1	2	5.55973E-05
1-75-1-4-1-1	4	0.000111195
1-1-189-0-1-1	1	2.77986E-05
1-1-1-0-1-19	1	2.77986E-05
1-1-1-0-1-3	3	8.33959E-05
1-1-1-379-1-1	1	2.77986E-05
1-1-1-0-1-47	15	0.000416979

SAL_JA4237AA	FDA00002570	Agona	0	0	0	0	0	0	0	0	0	0	0			1-1-1-0-71-1	1	2.77986E-05
SAL_JA4278AA	FDA00011733	Agona	0	0	0	0	0	0	0	0	0	0	0			1-75-1-0-1-1	3	8.33959E-05
SAL_JA4823AA	FDA00011667	Agona	0	0	0	0	0	0	0	0	0	0	0			1-1-1-4-1-47	1	2.77986E-05
SAL_JA5283AA	9-7-20170208	Agona	0	0	0	0	0	0	0	0	0	0	0			13-78-1-33-1-1	2	5.55973E-05
SAL_JA5292AA	9-19-20170283	Agona	0	0	0	0	0	0	0	0	0	0	0			1-17-47-0-19-3	3	8.33959E-05
SAL_JA4682AA	CVM 43758	Agona	0	0	0	0	0	0	0	0	0	0	0			1-1-1-0-1-22	1	2.77986E-05
SAL_JA4596AA	CVM 43844	Agona	0	0	0	0	0	0	0	0	0	0	0			1-1-166-0-1-1	1	2.77986E-05
SAL_JA4664AA	CVM 43776	Agona	0	0	0	0	0	0	0	0	0	0	0			84-1-1-4-1-1	1	2.77986E-05
SAL_JA4515AA	PNUSAS012912	Agona	0	0	0	0	0	0	0	0	0	0	0			1-1-1-389-1-1	1	2.77986E-05
SAL_JA4892AA	PNUSAS005209	Agona	0	0	0	0	0	0	0	0	0	0	0			1-1-1-390-1-48	1	2.77986E-05
SAL_JA4626AA	CVM 43814	Agona	0	0	0	0	0	0	0	0	0	0	0			1-74-1-0-1-1	1	2.77986E-05
SAL_JA4644AA	CVM 43796	Agona	0	0	0	0	0	0	0	0	0	0	0			84-1-1-0-1-1	3	8.33959E-05
SAL_JA5256AA	PNUSAS013638	Agona	0	0	0	0	0	0	0	0	0	0	0			1-1-1-0-1-26	1	2.77986E-05
SAL_JA6524AA	361810	Agona	0	0	0	0	0	0	0	0	0	0	0			1-1-149-0-1-1	1	2.77986E-05
SAL_JA6470AA	356298	Agona	0	0	0	0	0	0	0	0	0	0	0			1-57-1-0-1-1	1	2.77986E-05
SAL_JA6451AA	344039	Agona	0	0	0	0	0	0	0	0	0	0	0			1-1-201-4-1-1	1	2.77986E-05
SAL_JA6367AA	366307	Agona	0	0	0	0	0	0	0	0	0	0	0			1-1-202-0-1-1	1	2.77986E-05
SAL_JA6326AA	352114	Agona	0	0	0	0	0	0	0	0	0	0	0			1-1-1-0-75-1	2	5.55973E-05
SAL_JA6337AA	329807	Agona	0	0	0	0	0	0	0	0	0	0	0			1-1-1-0-1-9	3	8.33959E-05
SAL_JA6323AA	363595	Agona	0	0	0	0	0	0	0	0	0	0	0			1-1-1-0-76-1	2	5.55973E-05
SAL_JA6288AA	352160	Agona	0	0	0	0	0	0	0	0	0	0	0			1-1-1-0-43-1	2	5.55973E-05
SAL_JA6295AA	329793	Agona	0	0	0	0	0	0	0	0	0	0	0			1-1-0-4-1-0	5	0.000138993
SAL_JA6257AA	352112	Agona	0	0	0	0	0	0	0	0	0	0	0			1-1-1-0-1-50	1	2.77986E-05
SAL_JA6186AA	350650	Agona	0	0	0	0	0	0	0	0	0	0	0			1-1-1-0-34-1	1	2.77986E-05
SAL_JA6113AA	PNUSAS013584	Agona	0	0	0	0	0	0	0	0	0	0	0			1-1-1-406-1-1	1	2.77986E-05
SAL_JA6018AA	363498	Agona	0	0	0	0	0	0	0	0	0	0	0			1-1-1-0-81-1	1	2.77986E-05
SAL_JA5986AA	360331	Agona	0	0	0	0	0	0	0	0	0	0	0			1-81-1-0-1-1	4	0.000111195
SAL_JA5939AA	PNUSAS013596	Agona	0	0	0	0	0	0	0	0	0	0	0			1-81-1-4-1-1	4	0.000111195
SAL_JA5904AA	352147	Agona	0	0	0	0	0	0	0	0	0	0	0			1-1-1-0-1-51	1	2.77986E-05
SAL_JA5881AA	369915	Agona	0	0	0	0	0	0	0	0	0	0	0			1-1-1-139-1-51	3	8.33959E-05
SAL_JA5738AA	359269	Agona	0	0	0	0	0	0	0	0	0	0	0			0-0-108-0-0-0	2	5.55973E-05
SAL_JA5536AA	348777	Agona	0	0	0	0	0	0	0	0	0	0	0			1-1-1-416-1-1	1	2.77986E-05
SAL_JA5534AA	367197	Agona	0	0	0	0	0	0	0	0	0	0	0			1-11-192-4-1-1	1	2.77986E-05
SAL_JA5609AA	364190	Agona	0	0	0	0	0	0	0	0	0	0	0			1-0-0-0-0-0	1	2.77986E-05
SAL_JA5622AA	362072	Agona	0	0	0	0	0	0	0	0	0	0	0			1-1-171-0-1-1	2	5.55973E-05
SAL_JA5558AA	363576	Agona	0	0	0	0	0	0	0	0	0	0	0			1-1-1-4-1-52	1	2.77986E-05
SAL_JA5817AA	PNUSAS013319	Agona	0	0	0	0	0	0	0	0	0	0	0			1-1-1-0-1-36	1	2.77986E-05
SAL_JA5465AA	367256	Agona	0	0	0	0	0	0	0	0	0	0	0			1-1-1-0-1-43	1	2.77986E-05
SAL_JA5418AA	PNUSAS013357	Agona	0	0	0	0	0	0	0	0	0	0	0			1-1-107-0-1-1	1	2.77986E-05
SAL_JA5404AA	365806	Agona	0	0	0	0	0	0	0	0	0	0	0			1-1-1-0-1-13	1	2.77986E-05
SAL_JA5390AA	369873	Agona	0	0	0	0	0	0	0	0	0	0	0			1-1-1-0-54-1	1	2.77986E-05
SAL_JA5403AA	348030	Agona	0	0	0	0	0	0	0	0	0	0	0			1-1-1-0-1-21	1	2.77986E-05
SAL_JA6887AA	352089	Agona	0	0	0	0	0	0	0	0	0	0	0			1-1-1-0-1-31	3	8.33959E-05
SAL_JA6903AA	333425	Agona	0	0	0	0	0	0	0	0	0	0	0			1-1-1-0-68-1	1	2.77986E-05
SAL_JA5571AA	343781	Agona	0	0	0	0	0	0	0	0	0	0	0			1-1-190-0-1-1	1	2.77986E-05
SAL_JA5319AA	PNUSAS013598	Agona	0	0	0	0	0	0	0	0	0	0	0			1-1-1-0-1-45	1	2.77986E-05
SAL_JA5329AA	PNUSAS013181	Agona	0	0	0	0	0	0	0	0	0	0	0			91-1-1-4-1-1	2	5.55973E-05
SAL_JA5346AA	PNUSAS013011	Agona	0	0	0	0	0	0	0	0	0	0	0			91-1-1-0-1-1	1	2.77986E-05
SAL_JA6873AA	368279	Agona	0	0	0	0	0	0	0	0	0	0	0			1-1-1-426-1-1	2	5.55973E-05
SAL_JA6796AA	PNUSAS008534	Agona	0	0	0	0	0	0	0	0	0	0	0			1-83-1-0-1-1	1	2.77986E-05
SAL_JA5567AA	348783	Agona	0	0	0	0	0	0	0	0	0	0	0			1-84-1-0-1-1	1	2.77986E-05
SAL_JA7198AA	10-17-20170351	Agona	0	0	0	0	0	0	0	0	0	0	0			1-1-1-429-1-1	2	5.55973E-05
SAL_JA6974AA	CFSAN001064	Agona	0	0	0	0	0	0	0	0	0	0	0			93-1-1-0-1-1	1	2.77986E-05
SAL_JA7374AA	PNUSAS014329	Agona	0	0	0	0	0	0	0	0	0	0	0			1-1-215-438-1-1	1	2.77986E-05
SAL_JA7357AA	PNUSAS013764	Agona	0	0	0	0	0	0	0	0	0	0	0			13-1-1-0-88-1	3	8.33959E-05
SAL_JA8426AA	364145	Agona	0	0	0	0	0	0	0	0	0	0	0			1-1-219-4-1-1	1	2.77986E-05
SAL_JA8387AA	352105	Agona	0	0	0	0	0	0	0	0	0	0	0			1-1-1-4-1-42	1	2.77986E-05
SAL_JA8386AA	352129	Agona	0	0	0	0	0	0	0	0	0	0	0			1-1-1-4-1-55	1	2.77986E-05
SAL_JA8382AA	362757	Agona	0	0	0	0	0	0	0	0	0	0	0			1-1-1-0-89-1	1	2.77986E-05
SAL_JA8344AA	363629	Agona	0	0	0	0	0	0	0	0	0	0	0			1-89-1-4-1-1	1	2.77986E-05
SAL_JA8293AA	366341	Agona	0	0	0	0	0	0	0	0	0	0	0			1-1-220-78-27-1	1	2.77986E-05
SAL_JA8343AA	365302	Agona	0	0	0	0	0	0	0	0	0	0	0			1-1-1-4-90-1	1	2.77986E-05
SAL_JA8269AA	361778	Agona	0	0	0	0	0	0	0	0	0	0	0			1-1-221-0-1-1	1	2.77986E-05
SAL_JA8262AA	335466	Agona	0	0	0	0	0	0	0	0	0	0	0			1-1-1-4-1-56	1	2.77986E-05
SAL_JA8230AA	351311	Agona	0	0	0	0	0	0	0	0	0	0	0			1-1-222-441-1-1	1	2.77986E-05
SAL_JA8165AA	356077	Agona	0	0	0	0	0	0	0	0	0	0	0			1-90-1-442-1-1	1	2.77986E-05
SAL_JA8164AA	360492	Agona	0	0	0	0	0	0	0	0	0	0	0			98-1-1-0-1-1	1	2.77986E-05
SAL_JA8110AA	352118	Agona	0	0	0	0	0	0	0	0	0	0	0			1-1-1-443-1-1	1	2.77986E-05
SAL_JA8067AA	358042	Agona	0	0	0	0	0	0	0	0	0	0	0			1-1-223-0-1-1	1	2.77986E-05
SAL_JA8078AA	348785	Agona	0	0	0	0	0	0	0	0	0	0	0			1-1-1-0-91-1	1	2.77986E-05
SAL_JA8041AA	333910	Agona	0	0	0	0	0	0	0	0	0	0	0			1-91-1-0-1-1	1	2.77986E-05
SAL_JA8033AA	352143	Agona	0	0	0	0	0	0	0	0	0	0	0			0-1-1-4-1-1	1	2.77986E-05
SAL_JA7995AA	352163	Agona	0	0	0	0	0	0	0	0	0	0	0		**Tyresoe**	0-0-0-0-0-0	11	/

SAL_JA7986AA	362092	Agona	0	0	0	0	0	0	0	0	0	0	0
SAL_JA7968AA	369924	Agona	0	0	0	0	0	0	0	0	0	0	0
SAL_JA7917AA	362756	Agona	0	0	0	0	0	0	0	0	0	0	0
SAL_JA7911AA	352097	Agona	0	0	0	0	0	0	0	0	0	0	0
SAL_JA8242AA	367493	Agona	0	0	0	0	0	0	0	0	0	0	0
SAL_JA7799AA	356535	Agona	0	0	0	0	0	0	0	0	0	0	0
SAL_JA7826AA	363610	Agona	0	0	0	0	0	0	0	0	0	0	0
SAL_JA7851AA	364142	Agona	0	0	0	0	0	0	0	0	0	0	0
SAL_JA8391AA	352149	Agona	0	0	0	0	0	0	0	0	0	0	0
SAL_JA7777AA	352107	Agona	0	0	0	0	0	0	0	0	0	0	0
SAL_JA8395AA	367489	Agona	0	0	0	0	0	0	0	0	0	0	0
SAL_JA8451AA	11-31-20170410	Agona	0	0	0	0	0	0	0	0	0	0	0
SAL_JA8392AA	352088	Agona	0	0	0	0	0	0	0	0	0	0	0
SAL_JA8071AA	352133	Agona	0	0	0	0	0	0	0	0	0	0	0
SAL_JA8742AA	PNUSAS014623	Agona	0	0	0	0	0	0	0	0	0	0	0
SAL_JA8685AA	PNUSAS012447	Agona	0	0	0	0	0	0	0	0	0	0	0
SAL_JA8583AA	FSIS1701085	Agona	0	0	0	0	0	0	0	0	0	0	0
SAL_JA8556AA	FDA00000727	Agona	0	0	0	0	0	0	0	0	0	0	0
SAL_JA7888AA	352091	Agona	0	0	0	0	0	0	0	0	0	0	0
SAL_JA9575AA	FDA00000248	Agona	0	0	0	0	0	0	0	0	0	0	0
SAL_JA9449AA	PNUSAS010964	Agona	0	0	0	0	0	0	0	0	0	0	0
SAL_JA8864AA	FSIS1701473	Agona	0	0	0	0	0	0	0	0	0	0	0
SAL_JA9704AA	FSIS1701820	Agona	0	0	0	0	0	0	0	0	0	0	0
SAL_JA9032AA	MS170063	Agona	0	0	0	0	0	0	0	0	0	0	0
SAL_JA9017AA	MS170119	Agona	0	0	0	0	0	0	0	0	0	0	0
SAL_JA9015AA	CFSAN064111	Agona	0	0	0	0	0	0	0	0	0	0	0
SAL_JA8996AA	PNUSAS013832	Agona	0	0	0	0	0	0	0	0	0	0	0
SAL_JA9831AA	FSIS1701907	Agona	0	0	0	0	0	0	0	0	0	0	0
SAL_JA8960AA	HIY0071	Agona	0	0	0	0	0	0	0	0	0	0	0
SAL_KA0194AA	E2016008482	Agona	0	0	0	0	0	0	0	0	0	0	0
SAL_KA0020AA	PNUSAS015901	Agona	0	0	0	0	0	0	0	0	0	0	0
SAL_JA9957AA	PNUSAS014895	Agona	0	0	0	0	0	0	0	0	0	0	0
SAL_KA0116AA	PNUSAS015317	Agona	0	0	0	0	0	0	0	0	0	0	0
SAL_KA0439AA	PNUSAS014021	Agona	0	0	0	0	0	0	0	0	0	0	0
SAL_KA0410AA	PNUSAS014514	Agona	0	0	0	0	0	0	0	0	0	0	0
SAL_KA0411AA	PNUSAS014513	Agona	0	0	0	0	0	0	0	0	0	0	0
SAL_KA0835AA	PNUSAS016187	Agona	0	0	0	0	0	0	0	0	0	0	0
SAL_KA1311AA	PNUSAS016175	Agona	0	0	0	0	0	0	0	0	0	0	0
SAL_KA1121AA	2015AM-0662	Agona	0	0	0	0	0	0	0	0	0	0	0
SAL_KA1077AA	HIY0082	Agona	0	0	0	0	0	0	0	0	0	0	0
SAL_KA0967AA	FSIS1701742	Agona	0	0	0	0	0	0	0	0	0	0	0
SAL_KA1251AA	PNUSAS015230	Agona	0	0	0	0	0	0	0	0	0	0	0
SAL_KA1112AA	2015AM-1688	Agona	0	0	0	0	0	0	0	0	0	0	0
SAL_KA2064AA	12-6-20170424	Agona	0	0	0	0	0	0	0	0	0	0	0
SAL_KA1049AA	PNUSAS016248	Agona	0	0	0	0	0	0	0	0	0	0	0
SAL_KA1858AA	PNUSAS014663	Agona	0	0	0	0	0	0	0	0	0	0	0
SAL_KA2963AA	FSIS1701897	Agona	0	0	0	0	0	0	0	0	0	0	0
SAL_KA2884AA	FSIS1702546	Agona	0	0	0	0	0	0	0	0	0	0	0
SAL_KA2642AA	PNUSAS015880	Agona	0	0	0	0	0	0	0	0	0	0	0
SAL_KA2413AA	FSIS1702507	Agona	0	0	0	0	0	0	0	0	0	0	0
SAL_KA3544AA	FSIS1702436	Agona	0	0	0	0	0	0	0	0	0	0	0
SAL_KA3481AA	FSIS1702603	Agona	0	0	0	0	0	0	0	0	0	0	0
SAL_KA3307AA	PNUSAS016241	Agona	0	0	0	0	0	0	0	0	0	0	0
SAL_KA3188AA	PNUSAS017782	Agona	0	0	0	0	0	0	0	0	0	0	0
SAL_KA3695AA	SARB1	Agona	0	0	0	0	0	0	0	0	0	0	0
SAL_KA4529AA	31.H.09	Agona	0	0	0	0	0	0	0	0	0	0	0
SAL_KA4530AA	22.H.04	Agona	0	0	0	0	0	0	0	0	0	0	0
SAL_KA4532AA	69.H.06	Agona	0	0	0	0	0	0	0	0	0	0	0
SAL_KA4533AA	16.H.08	Agona	0	0	0	0	0	0	0	0	0	0	0
SAL_KA4531AA	20052893	Agona	0	0	0	0	0	0	0	0	0	0	0
SAL_KA4827AA	FSIS1702771	Agona	0	0	0	0	0	0	0	0	0	0	0
SAL_KA4690AA	PNUSAS017180	Agona	0	0	0	0	0	0	0	0	0	0	0
SAL_KA4303AA	CK_95/00003387	Agona	0	0	0	0	0	0	0	0	0	0	0
SAL_KA4304AA	08.A.05	Agona	0	0	0	0	0	0	0	0	0	0	0
SAL_KA4311AA	GA4973TB	Agona	0	0	0	0	0	0	0	0	0	0	0
SAL_KA4312AA	SF_R0571/09	Agona	0	0	0	0	0	0	0	0	0	0	0
SAL_KA4313AA	S08-0771	Agona	0	0	0	0	0	0	0	0	0	0	0
SAL_KA4273AA	10.A.05	Agona	0	0	0	0	0	0	0	0	0	0	0
SAL_KA4274AA	SF_R0040/09	Agona	0	0	0	0	0	0	0	0	0	0	0
SAL_KA4280AA	SF_R0362/09	Agona	0	0	0	0	0	0	0	0	0	0	0
SAL_KA4281AA	53.F.08	Agona	0	0	0	0	0	0	0	0	0	0	0
SAL_KA4288AA	11.A.05	Agona	0	0	0	0	0	0	0	0	0	0	0
SAL_KA4297AA	09.F.08	Agona	0	0	0	0	0	0	0	0	0	0	0

**Uganda**	0-0-0-0-0-0	673	/
**Umbilo**	0-0-0-0-0-0	33	/
**Uppsala**	0-0-0-0-0-0	1	/
**Urbana**	0-0-0-0-0-0	138	/
**Ursenbach**	0-0-0-0-0-0	1	/
**Utrecht**	0-0-0-0-0-0	1	/
**V**	0-0-0-0-0-0	111	/
**Vancouver**	0-0-0-0-0-0	2	/
**Vejle**	0-0-0-0-0-0	5	/
**Veneziana**	0-0-0-0-0-0	59	/
**VI**	0-0-0-0-0-0	25	/
**Virchow**	0-0-0-0-0-0	1110	/
**Vitkin**	0-0-0-0-0-0	31	/
**Volkmarsdorf**	0-0-0-0-0-0	1	/
**Vuadens**	0-0-0-0-0-0	3	/
**Wa**	0-0-0-0-0-0	3	/
**Wagenia**	0-0-0-0-0-0	5	0.833333333
	5-4-17-391-0-0	1	0.166666667
**Wandsworth**	0-0-0-0-0-0	71	/
**Warnow**	0-0-0-0-0-0	3	/
**Wayne**	0-0-0-0-0-0	3	/
**Wedding**	0-0-0-0-0-0	4	/
**Welikade**	0-0-0-0-0-0	8	/
**Weltevreden**	0-0-0-0-0-0	1471	/
**Weslaco**	0-0-0-0-0-0	19	/
**Westeinde**	0-0-0-0-0-0	1	/
**Westminster**	0-0-0-0-0-0	8	/
**Weston**	0-0-0-0-0-0	2	/
**Wien**	0-0-0-0-0-0	60	/
**Wil**	0-0-0-0-0-0	2	/
**Wilhelmsburg**	0-0-0-0-0-0	8	/
**Windermere**	0-0-0-0-0-0	3	/
**Winterthur**	0-0-0-0-0-0	2	/
**Woodhull**	0-0-0-0-0-0	2	/
**Worthington**	0-0-0-0-0-0	334	/
**Wyldegreen**	0-0-0-0-0-0	1	/
**Yolo**	0-0-0-0-0-0	1	/
**Yoruba**	0-0-0-0-0-0	11	/
**Zigong**	0-0-0-0-0-0	3	/
**Zongo**	5-4-169-8-0-0	1	1

SAL_KA4242AA	SF_R0038/09	Agona	0	0	0	0	0	0	0	0	0	0	0
SAL_KA4249AA	SF_R0041/09	Agona	0	0	0	0	0	0	0	0	0	0	0
SAL_KA4250AA	57.A.08	Agona	0	0	0	0	0	0	0	0	0	0	0
SAL_KA4252AA	S09-0426	Agona	0	0	0	0	0	0	0	0	0	0	0
SAL_KA4255AA	1086/05	Agona	0	0	0	0	0	0	0	0	0	0	0
SAL_KA4257AA	71.E.05	Agona	0	0	0	0	0	0	0	0	0	0	0
SAL_KA4259AA	05.O.06	Agona	0	0	0	0	0	0	0	0	0	0	0
SAL_KA4265AA	43.E.09	Agona	0	0	0	0	0	0	0	0	0	0	0
SAL_KA4266AA	54.O.08	Agona	0	0	0	0	0	0	0	0	0	0	0
SAL_KA4271AA	912/05	Agona	0	0	0	0	0	0	0	0	0	0	0
SAL_KA4289AA	SF_R0474/09	Agona	0	0	0	0	0	0	0	0	0	0	0
SAL_KA4290AA	52.F.08	Agona	0	0	0	0	0	0	0	0	0	0	0
SAL_KA4305AA	SF_R0517/09	Agona	0	0	0	0	0	0	0	0	0	0	0
SAL_KA4306AA	50.E.08	Agona	0	0	0	0	0	0	0	0	0	0	0
SAL_KA4231AA	H102900575	Agona	0	0	0	0	0	0	0	0	0	0	0
SAL_KA4233AA	55.U.08	Agona	0	0	0	0	0	0	0	0	0	0	0
SAL_KA4234AA	59.F.08	Agona	0	0	0	0	0	0	0	0	0	0	0
SAL_KA4239AA	JM_08/10	Agona	0	0	0	0	0	0	0	0	0	0	0
SAL_KA4241AA	MC_04-0873	Agona	0	0	0	0	0	0	0	0	0	0	0
SAL_KA4226AA	SF_R0037/09	Agona	0	0	0	0	0	0	0	0	0	0	0
SAL_KA4227AA	70.E.05	Agona	0	0	0	0	0	0	0	0	0	0	0
SAL_KA4236AA	56.O.08	Agona	0	0	0	0	0	0	0	0	0	0	0
SAL_KA4243AA	14.E.05	Agona	0	0	0	0	0	0	0	0	0	0	0
SAL_KA4017AA	DBS_20081012	Agona	0	0	0	0	0	0	0	0	0	0	0
SAL_KA4025AA	S08-0770	Agona	0	0	0	0	0	0	0	0	0	0	0
SAL_KA3967AA	P5494570	Agona	0	0	0	0	0	0	0	0	0	0	0
SAL_KA4958AA	PNUSAS018227	Agona	0	0	0	0	0	0	0	0	0	0	0
SAL_KA4937AA	PNUSAS017911	Agona	0	0	0	0	0	0	0	0	0	0	0
SAL_KA5002AA	2015AM-0910	Agona	0	0	0	0	0	0	0	0	0	0	0
SAL_KA5535AA	PNUSAS018266	Agona	0	0	0	0	0	0	0	0	0	0	0
SAL_KA5155AA	PNUSAS018734	Agona	0	0	0	0	0	0	0	0	0	0	0
SAL_KA6156AA	2015AM-0061	Agona	0	0	0	0	0	0	0	0	0	0	0
SAL_KA5985AA	FSIS1702764	Agona	0	0	0	0	0	0	0	0	0	0	0
SAL_KA5921AA	PNUSAS017012	Agona	0	0	0	0	0	0	0	0	0	0	0
SAL_KA5866AA	PNUSAS016564	Agona	0	0	0	0	0	0	0	0	0	0	0
SAL_KA5828AA	PNUSAS018450	Agona	0	0	0	0	0	0	0	0	0	0	0
SAL_KA5820AA	PNUSAS019294	Agona	0	0	0	0	0	0	0	0	0	0	0
SAL_KA5862AA	PNUSAS016552	Agona	0	0	0	0	0	0	0	0	0	0	0
SAL_KA6281AA	FSIS1703129	Agona	0	0	0	0	0	0	0	0	0	0	0
SAL_KA6266AA	FDA00003704	Agona	0	0	0	0	0	0	0	0	0	0	0
SAL_KA6890AA	PNUSAS017135	Agona	0	0	0	0	0	0	0	0	0	0	0
SAL_KA6729AA	PNUSAS017915	Agona	0	0	0	0	0	0	0	0	0	0	0
SAL_KA6713AA	PNUSAS019177	Agona	0	0	0	0	0	0	0	0	0	0	0
SAL_KA6709AA	PNUSAS019174	Agona	0	0	0	0	0	0	0	0	0	0	0
SAL_KA6587AA	PNUSAS007779	Agona	0	0	0	0	0	0	0	0	0	0	0
SAL_KA7098AA	FSIS1703329	Agona	0	0	0	0	0	0	0	0	0	0	0
SAL_KA7118AA	FSIS1703353	Agona	0	0	0	0	0	0	0	0	0	0	0
SAL_KA7152AA	FSIS1703191	Agona	0	0	0	0	0	0	0	0	0	0	0
SAL_KA7610AA	PNUSAS016923	Agona	0	0	0	0	0	0	0	0	0	0	0
SAL_KA7661AA	FDA00012034	Agona	0	0	0	0	0	0	0	0	0	0	0
SAL_KA7533AA	PNUSAS019820	Agona	0	0	0	0	0	0	0	0	0	0	0
SAL_KA8854AA	17018052	Agona	0	0	0	0	0	0	0	0	0	0	0
SAL_KA7260AA	ADRDL-765	Agona	0	0	0	0	0	0	0	0	0	0	0
SAL_KA8055AA	PNUSAS019333	Agona	0	0	0	0	0	0	0	0	0	0	0
SAL_KA8057AA	PNUSAS019791	Agona	0	0	0	0	0	0	0	0	0	0	0
SAL_KA7963AA	PNUSAS016374	Agona	0	0	0	0	0	0	0	0	0	0	0
SAL_KA8303AA	PNUSAS020018	Agona	0	0	0	0	0	0	0	0	0	0	0
SAL_KA8222AA	PNUSAS019537	Agona	0	0	0	0	0	0	0	0	0	0	0
SAL_KA8548AA	PNUSAS020146	Agona	0	0	0	0	0	0	0	0	0	0	0
SAL_KA8913AA	PNUSAS019386	Agona	0	0	0	0	0	0	0	0	0	0	0
SAL_KA9029AA	PNUSAS020625	Agona	0	0	0	0	0	0	0	0	0	0	0
SAL_KA9185AA	FDA00012049	Agona	0	0	0	0	0	0	0	0	0	0	0
SAL_KA9013AA	PNUSAS020496	Agona	0	0	0	0	0	0	0	0	0	0	0
SAL_KA9173AA	FDA00012046	Agona	0	0	0	0	0	0	0	0	0	0	0
SAL_KA9187AA	FDA00012043	Agona	0	0	0	0	0	0	0	0	0	0	0
SAL_KA9188AA	FDA00012040	Agona	0	0	0	0	0	0	0	0	0	0	0
SAL_KA8959AA	2015AM-0340	Agona	0	0	0	0	0	0	0	0	0	0	0
SAL_KA9776AA	PNUSAS020999	Agona	0	0	0	0	0	0	0	0	0	0	0
SAL_KA9539AA	PNUSAS019853	Agona	0	0	0	0	0	0	0	0	0	0	0
SAL_KA7609AA	PNUSAS017429	Agona	0	0	0	0	0	0	0	0	0	0	0
SAL_KA7662AA	FDA00012033	Agona	0	0	0	0	0	0	0	0	0	0	0
SAL_KA9236AA	PNUSAS020309	Agona	0	0	0	0	0	0	0	0	0	0	0
SAL_KA9982AA	PNUSAS021022	Agona	0	0	0	0	0	0	0	0	0	0	0

SAL_KA9822AA	FSIS1703750	Agona	0	0	0	0	0	0	0	0	0	0	0
SAL_LA0386AA	VA-WGS-17092	Agona	0	0	0	0	0	0	0	0	0	0	0
SAL_LA0357AA	FDA00012061	Agona	0	0	0	0	0	0	0	0	0	0	0
SAL_LA0375AA	VA-WGS-17088	Agona	0	0	0	0	0	0	0	0	0	0	0
SAL_LA0350AA	FDA00012058	Agona	0	0	0	0	0	0	0	0	0	0	0
SAL_LA0354AA	FDA00012063	Agona	0	0	0	0	0	0	0	0	0	0	0
SAL_LA0355AA	FDA00012060	Agona	0	0	0	0	0	0	0	0	0	0	0
SAL_LA0334AA	FDA00012069	Agona	0	0	0	0	0	0	0	0	0	0	0
SAL_LA0342AA	FDA00012074	Agona	0	0	0	0	0	0	0	0	0	0	0
SAL_LA0343AA	FDA00012076	Agona	0	0	0	0	0	0	0	0	0	0	0
SAL_LA0349AA	FDA00012066	Agona	0	0	0	0	0	0	0	0	0	0	0
SAL_LA0351AA	FDA00012057	Agona	0	0	0	0	0	0	0	0	0	0	0
SAL_LA0447AA	PNUSAS020928	Agona	0	0	0	0	0	0	0	0	0	0	0
SAL_LA0408AA	FSIS11703942	Agona	0	0	0	0	0	0	0	0	0	0	0
SAL_LA0595AA	FSIS11703963	Agona	0	0	0	0	0	0	0	0	0	0	0
SAL_LA0931AA	PNUSAS019567	Agona	0	0	0	0	0	0	0	0	0	0	0
SAL_LA1064AA	PNUSAS021740	Agona	0	0	0	0	0	0	0	0	0	0	0
SAL_LA0825AA	30584	Agona	0	0	0	0	0	0	0	0	0	0	0
SAL_LA0818AA	30601	Agona	0	0	0	0	0	0	0	0	0	0	0
SAL_LA0790AA	30376	Agona	0	0	0	0	0	0	0	0	0	0	0
SAL_LA0809AA	31353	Agona	0	0	0	0	0	0	0	0	0	0	0
SAL_LA1819AA	FSIS11704057	Agona	0	0	0	0	0	0	0	0	0	0	0
SAL_LA3132AA	20051622	Agona	0	0	0	0	0	0	0	0	0	0	0
SAL_LA3134AA	20080978	Agona	0	0	0	0	0	0	0	0	0	0	0
SAL_LA3136AA	20070385	Agona	0	0	0	0	0	0	0	0	0	0	0
SAL_LA3137AA	20050085	Agona	0	0	0	0	0	0	0	0	0	0	0
SAL_LA3130AA	20061763	Agona	0	0	0	0	0	0	0	0	0	0	0
SAL_LA3128AA	20052501	Agona	0	0	0	0	0	0	0	0	0	0	0
SAL_LA3095AA	20081243	Agona	0	0	0	0	0	0	0	0	0	0	0
SAL_LA3064AA	20081579	Agona	0	0	0	0	0	0	0	0	0	0	0
SAL_LA3126AA	20081181	Agona	0	0	0	0	0	0	0	0	0	0	0
SAL_LA3127AA	20080940	Agona	0	0	0	0	0	0	0	0	0	0	0
SAL_LA3074AA	20081917	Agona	0	0	0	0	0	0	0	0	0	0	0
SAL_LA3123AA	20080965	Agona	0	0	0	0	0	0	0	0	0	0	0
SAL_LA3118AA	20080970	Agona	0	0	0	0	0	0	0	0	0	0	0
SAL_LA2789AA	20051274	Agona	0	0	0	0	0	0	0	0	0	0	0
SAL_LA2799AA	20081008	Agona	0	0	0	0	0	0	0	0	0	0	0
SAL_LA2812AA	20070383	Agona	0	0	0	0	0	0	0	0	0	0	0
SAL_LA2704AA	20030252	Agona	0	0	0	0	0	0	0	0	0	0	0
SAL_LA2769AA	20080944	Agona	0	0	0	0	0	0	0	0	0	0	0
SAL_LA2775AA	20080984	Agona	0	0	0	0	0	0	0	0	0	0	0
SAL_LA2777AA	20071841	Agona	0	0	0	0	0	0	0	0	0	0	0
SAL_LA3133AA	20090312	Agona	0	0	0	0	0	0	0	0	0	0	0
SAL_LA3141AA	20050459	Agona	0	0	0	0	0	0	0	0	0	0	0
SAL_LA3086AA	20081195	Agona	0	0	0	0	0	0	0	0	0	0	0
SAL_LA3121AA	20100169	Agona	0	0	0	0	0	0	0	0	0	0	0
SAL_LA2784AA	20080934	Agona	0	0	0	0	0	0	0	0	0	0	0
SAL_LA2794AA	20080872	Agona	0	0	0	0	0	0	0	0	0	0	0
SAL_LA2810AA	20080858	Agona	0	0	0	0	0	0	0	0	0	0	0
SAL_LA2833AA	20080613	Agona	0	0	0	0	0	0	0	0	0	0	0
SAL_LA2770AA	20081313	Agona	0	0	0	0	0	0	0	0	0	0	0
SAL_LA2778AA	20080873	Agona	0	0	0	0	0	0	0	0	0	0	0
SAL_LA2783AA	20081009	Agona	0	0	0	0	0	0	0	0	0	0	0
SAL_LA2792AA	20051846	Agona	0	0	0	0	0	0	0	0	0	0	0
SAL_LA2803AA	20061075	Agona	0	0	0	0	0	0	0	0	0	0	0
SAL_LA2805AA	20051537	Agona	0	0	0	0	0	0	0	0	0	0	0
SAL_LA2808AA	20080919	Agona	0	0	0	0	0	0	0	0	0	0	0
SAL_LA2828AA	20060239	Agona	0	0	0	0	0	0	0	0	0	0	0
SAL_LA3119AA	20081030	Agona	0	0	0	0	0	0	0	0	0	0	0
SAL_LA3125AA	20050750	Agona	0	0	0	0	0	0	0	0	0	0	0
SAL_LA3120AA	20070384	Agona	0	0	0	0	0	0	0	0	0	0	0
SAL_LA2717AA	13.E.05	Agona	0	0	0	0	0	0	0	0	0	0	0
SAL_LA2771AA	20061000	Agona	0	0	0	0	0	0	0	0	0	0	0
SAL_LA2785AA	20091690	Agona	0	0	0	0	0	0	0	0	0	0	0
SAL_LA2787AA	20060901	Agona	0	0	0	0	0	0	0	0	0	0	0
SAL_LA2788AA	SA07.149	Agona	0	0	0	0	0	0	0	0	0	0	0
SAL_LA2800AA	20052335	Agona	0	0	0	0	0	0	0	0	0	0	0
SAL_LA2814AA	20081028	Agona	0	0	0	0	0	0	0	0	0	0	0
SAL_LA2790AA	20100362	Agona	0	0	0	0	0	0	0	0	0	0	0
SAL_LA2811AA	0808F22680	Agona	0	0	0	0	0	0	0	0	0	0	0
SAL_LA2807AA	20080917	Agona	0	0	0	0	0	0	0	0	0	0	0
SAL_LA2780AA	20063027	Agona	0	0	0	0	0	0	0	0	0	0	0
SAL_LA2801AA	20091513	Agona	0	0	0	0	0	0	0	0	0	0	0

SAL_LA2809AA	20062097	Agona	0	0	0	0	0	0	0	0	0	0	0
SAL_LA2818AA	20080660	Agona	0	0	0	0	0	0	0	0	0	0	0
SAL_LA2826AA	0808T17032	Agona	0	0	0	0	0	0	0	0	0	0	0
SAL_LA2819AA	20061833	Agona	0	0	0	0	0	0	0	0	0	0	0
SAL_LA2772AA	SA06.018	Agona	0	0	0	0	0	0	0	0	0	0	0
SAL_LA2774AA	20100875	Agona	0	0	0	0	0	0	0	0	0	0	0
SAL_LA2776AA	20081080	Agona	0	0	0	0	0	0	0	0	0	0	0
SAL_LA3135AA	20081138	Agona	0	0	0	0	0	0	0	0	0	0	0
SAL_LA3139AA	60.O.08	Agona	0	0	0	0	0	0	0	0	0	0	0
SAL_LA3116AA	20081315	Agona	0	0	0	0	0	0	0	0	0	0	0
SAL_LA2815AA	20052714	Agona	0	0	0	0	0	0	0	0	0	0	0
SAL_LA2796AA	20070382	Agona	0	0	0	0	0	0	0	0	0	0	0
SAL_LA2816AA	20100223	Agona	0	0	0	0	0	0	0	0	0	0	0
SAL_LA2791AA	20080969	Agona	0	0	0	0	0	0	0	0	0	0	0
SAL_LA2798AA	20080701	Agona	0	0	0	0	0	0	0	0	0	0	0
SAL_LA2793AA	20080351	Agona	0	0	0	0	0	0	0	0	0	0	0
SAL_LA2806AA	20081159	Agona	0	0	0	0	0	0	0	0	0	0	0
SAL_LA2830AA	20070199	Agona	0	0	0	0	0	0	0	0	0	0	0
SAL_LA2336AA	FSIS21720103	Agona	0	0	0	0	0	0	0	0	0	0	0
SAL_LA2782AA	20080712	Agona	0	0	0	0	0	0	0	0	0	0	0
SAL_LA2773AA	20051674	Agona	0	0	0	0	0	0	0	0	0	0	0
SAL_LA3117AA	20090027	Agona	0	0	0	0	0	0	0	0	0	0	0
SAL_LA2768AA	20080988	Agona	0	0	0	0	0	0	0	0	0	0	0
SAL_LA2132AA	ZJ-943	Agona	0	0	0	0	0	0	0	0	0	0	0
SAL_LA2104AA	ZJ-A	Agona	0	0	0	0	0	0	0	0	0	0	0
SAL_LA2110AA	ZJ-936	Agona	0	0	0	0	0	0	0	0	0	0	0
SAL_LA2115AA	ZJ-1000	Agona	0	0	0	0	0	0	0	0	0	0	0
SAL_LA2121AA	938	Agona	0	0	0	0	0	0	0	0	0	0	0
SAL_LA3250AA	PNUSAS022030	Agona	0	0	0	0	0	0	0	0	0	0	0
SAL_LA1996AA	20001590	Agona	0	0	0	0	0	0	0	0	0	0	0
SAL_LA1998AA	20030055	Agona	0	0	0	0	0	0	0	0	0	0	0
SAL_LA2000AA	20030216	Agona	0	0	0	0	0	0	0	0	0	0	0
SAL_LA2004AA	20021732	Agona	0	0	0	0	0	0	0	0	0	0	0
SAL_LA2006AA	20030180	Agona	0	0	0	0	0	0	0	0	0	0	0
SAL_LA1964AA	20030948	Agona	0	0	0	0	0	0	0	0	0	0	0
SAL_LA1965AA	61.O.08	Agona	0	0	0	0	0	0	0	0	0	0	0
SAL_LA1974AA	20031675	Agona	0	0	0	0	0	0	0	0	0	0	0
SAL_LA1978AA	20041744	Agona	0	0	0	0	0	0	0	0	0	0	0
SAL_LA1981AA	20042846	Agona	0	0	0	0	0	0	0	0	0	0	0
SAL_LA1983AA	20003526	Agona	0	0	0	0	0	0	0	0	0	0	0
SAL_LA1986AA	CK_03/00001461	Agona	0	0	0	0	0	0	0	0	0	0	0
SAL_LA1985AA	20004492	Agona	0	0	0	0	0	0	0	0	0	0	0
SAL_LA1989AA	20003294	Agona	0	0	0	0	0	0	0	0	0	0	0
SAL_LA1991AA	20003707	Agona	0	0	0	0	0	0	0	0	0	0	0
SAL_LA1994AA	20091706	Agona	0	0	0	0	0	0	0	0	0	0	0
SAL_LA2001AA	CG04.173	Agona	0	0	0	0	0	0	0	0	0	0	0
SAL_LA1935AA	20031284	Agona	0	0	0	0	0	0	0	0	0	0	0
SAL_LA1937AA	20040595	Agona	0	0	0	0	0	0	0	0	0	0	0
SAL_LA1939AA	RK_08:MF0017271R	Agona	0	0	0	0	0	0	0	0	0	0	0
SAL_LA1943AA	20003376	Agona	0	0	0	0	0	0	0	0	0	0	0
SAL_LA1945AA	20003907	Agona	0	0	0	0	0	0	0	0	0	0	0
SAL_LA1948AA	20002228	Agona	0	0	0	0	0	0	0	0	0	0	0
SAL_LA1952AA	20004759	Agona	0	0	0	0	0	0	0	0	0	0	0
SAL_LA1953AA	RK_08:MF0010405R	Agona	0	0	0	0	0	0	0	0	0	0	0
SAL_LA1955AA	20010024	Agona	0	0	0	0	0	0	0	0	0	0	0
SAL_LA1957AA	20021733	Agona	0	0	0	0	0	0	0	0	0	0	0
SAL_LA1959AA	20030198	Agona	0	0	0	0	0	0	0	0	0	0	0
SAL_LA1961AA	20021634	Agona	0	0	0	0	0	0	0	0	0	0	0
SAL_LA1962AA	20030065	Agona	0	0	0	0	0	0	0	0	0	0	0
SAL_LA1967AA	20030968	Agona	0	0	0	0	0	0	0	0	0	0	0
SAL_LA1969AA	20032560	Agona	0	0	0	0	0	0	0	0	0	0	0
SAL_LA1971AA	20041315	Agona	0	0	0	0	0	0	0	0	0	0	0
SAL_LA1976AA	20040760	Agona	0	0	0	0	0	0	0	0	0	0	0
SAL_LA1979AA	1090801410	Agona	0	0	0	0	0	0	0	0	0	0	0
SAL_LA1928AA	20032023	Agona	0	0	0	0	0	0	0	0	0	0	0
SAL_LA1930AA	20041090	Agona	0	0	0	0	0	0	0	0	0	0	0
SAL_LA1931AA	73.H.09	Agona	0	0	0	0	0	0	0	0	0	0	0
SAL_LA1933AA	1090602167	Agona	0	0	0	0	0	0	0	0	0	0	0
SAL_LA1950AA	20003663	Agona	0	0	0	0	0	0	0	0	0	0	0
SAL_LA3615AA	64.H.00	Agona	0	0	0	0	0	0	0	0	0	0	0
SAL_LA2820AA	20050551	Agona	0	0	0	0	0	0	0	0	0	0	0
SAL_LA2823AA	20081238	Agona	0	0	0	0	0	0	0	0	0	0	0
SAL_LA2822AA	20080915	Agona	0	0	0	0	0	0	0	0	0	0	0

SAL_LA2824AA	20061936	Agona	0	0	0	0	0	0	0	0	0	0	0
SAL_LA2827AA	20071186	Agona	0	0	0	0	0	0	0	0	0	0	0
SAL_LA2825AA	20080828	Agona	0	0	0	0	0	0	0	0	0	0	0
SAL_LA2829AA	20081055	Agona	0	0	0	0	0	0	0	0	0	0	0
SAL_LA2168AA	SF_R0318/09	Agona	0	0	0	0	0	0	0	0	0	0	0
SAL_LA2174AA	ZJ-967	Agona	0	0	0	0	0	0	0	0	0	0	0
SAL_LA2161AA	ZJ-93	Agona	0	0	0	0	0	0	0	0	0	0	0
SAL_LA2166AA	ZJ-G	Agona	0	0	0	0	0	0	0	0	0	0	0
SAL_LA2147AA	ZJ-946	Agona	0	0	0	0	0	0	0	0	0	0	0
SAL_LA2153AA	ZJ-C	Agona	0	0	0	0	0	0	0	0	0	0	0
SAL_LA4035AA	SARA61_FB_1	Agona	0	0	0	0	0	0	0	0	0	0	0
SAL_LA3657AA	PNUSAS022374	Agona	0	0	0	0	0	0	0	0	0	0	0
SAL_LA4274AA	PNUSAS022776	Agona	0	0	0	0	0	0	0	0	0	0	0
SAL_LA4365AA	FSIS11704244	Agona	0	0	0	0	0	0	0	0	0	0	0
SAL_LA4377AA	FSIS11704285	Agona	0	0	0	0	0	0	0	0	0	0	0
SAL_LA4478AA	PNUSAS023008	Agona	0	0	0	0	0	0	0	0	0	0	0
SAL_LA4936AA	PNUSAS023104	Agona	0	0	0	0	0	0	0	0	0	0	0
SAL_LA4790AA	FSIS11704365	Agona	0	0	0	0	0	0	0	0	0	0	0
SAL_LA4731AA	PNUSAS010736	Agona	0	0	0	0	0	0	0	0	0	0	0
SAL_LA5140AA	17041677	Agona	0	0	0	0	0	0	0	0	0	0	0
SAL_LA2821AA	20081145	Agona	0	0	0	0	0	0	0	0	0	0	0
SAL_LA3106AA	20081265	Agona	0	0	0	0	0	0	0	0	0	0	0
SAL_LA3114AA	20081975	Agona	0	0	0	0	0	0	0	0	0	0	0
SAL_LA6943AA	21-30-20170825	Agona	0	0	0	0	0	0	0	0	0	0	0
SAL_LA6945AA	21-23-20170818	Agona	0	0	0	0	0	0	0	0	0	0	0
SAL_LA5593AA	FDA00000743	Agona	0	0	0	0	0	0	0	0	0	0	0
SAL_LA6861AA	PNUSAS023705	Agona	0	0	0	0	0	0	0	0	0	0	0
SAL_LA6851AA	CFSAN068054	Agona	0	0	0	0	0	0	0	0	0	0	0
SAL_LA6855AA	CFSAN068053	Agona	0	0	0	0	0	0	0	0	0	0	0
SAL_LA6752AA	CFSAN068051	Agona	0	0	0	0	0	0	0	0	0	0	0
SAL_LA6717AA	PNUSAS021639	Agona	0	0	0	0	0	0	0	0	0	0	0
SAL_LA6719AA	MT20171062007	Agona	0	0	0	0	0	0	0	0	0	0	0
SAL_LA6697AA	PNUSAS023174	Agona	0	0	0	0	0	0	0	0	0	0	0
SAL_LA6432AA	PNUSAS022656	Agona	0	0	0	0	0	0	0	0	0	0	0
SAL_LA6910AA	CFSAN068056	Agona	0	0	0	0	0	0	0	0	0	0	0
SAL_LA6886AA	CFSAN068055	Agona	0	0	0	0	0	0	0	0	0	0	0
SAL_LA6914AA	CFSAN067905	Agona	0	0	0	0	0	0	0	0	0	0	0
SAL_LA6284AA	HIY0100	Agona	0	0	0	0	0	0	0	0	0	0	0
SAL_LA6287AA	34908	Agona	0	0	0	0	0	0	0	0	0	0	0
SAL_LA6288AA	31554	Agona	0	0	0	0	0	0	0	0	0	0	0
SAL_LA6272AA	CFSAN069024	Agona	0	0	0	0	0	0	0	0	0	0	0
SAL_LA6909AA	CFSAN068052	Agona	0	0	0	0	0	0	0	0	0	0	0
SAL_LA6243AA	CFSAN069029	Agona	0	0	0	0	0	0	0	0	0	0	0
SAL_LA6185AA	CFSAN069202	Agona	0	0	0	0	0	0	0	0	0	0	0
SAL_LA6144AA	CFSAN069181	Agona	0	0	0	0	0	0	0	0	0	0	0
SAL_LA6184AA	CFSAN069180	Agona	0	0	0	0	0	0	0	0	0	0	0
SAL_LA6100AA	CFSAN069189	Agona	0	0	0	0	0	0	0	0	0	0	0
SAL_LA6112AA	CFSAN069178	Agona	0	0	0	0	0	0	0	0	0	0	0
SAL_LA6083AA	CFSAN069174	Agona	0	0	0	0	0	0	0	0	0	0	0
SAL_LA6088AA	CFSAN069177	Agona	0	0	0	0	0	0	0	0	0	0	0
SAL_LA6125AA	CFSAN069139	Agona	0	0	0	0	0	0	0	0	0	0	0
SAL_LA7346AA	PNUSAS022025	Agona	0	0	0	0	0	0	0	0	0	0	0
SAL_LA7392AA	PNUSAS013124	Agona	0	0	0	0	0	0	0	0	0	0	0
SAL_LA9974AA	24-28-20171018	Agona	0	0	0	0	0	0	0	0	0	0	0
SAL_LA8150AA	34912	Agona	0	0	0	0	0	0	0	0	0	0	0
SAL_LA9815AA	PNUSAS024255	Agona	0	0	0	0	0	0	0	0	0	0	0
SAL_MA0261AA	PNUSAS024141	Agona	0	0	0	0	0	0	0	0	0	0	0
SAL_MA0276AA	PNUSAS024837	Agona	0	0	0	0	0	0	0	0	0	0	0
SAL_MA0168AA	PNUSAS024220	Agona	0	0	0	0	0	0	0	0	0	0	0
SAL_MA0142AA	2017K-0778	Agona	0	0	0	0	0	0	0	0	0	0	0
SAL_MA0087AA	PNUSAS023480	Agona	0	0	0	0	0	0	0	0	0	0	0
SAL_MA0016AA	34914	Agona	0	0	0	0	0	0	0	0	0	0	0
SAL_LA9985AA	34921	Agona	0	0	0	0	0	0	0	0	0	0	0
SAL_LA9984AA	34913	Agona	0	0	0	0	0	0	0	0	0	0	0
SAL_LA9987AA	34918	Agona	0	0	0	0	0	0	0	0	0	0	0
SAL_MA0463AA	PNUSAS025029	Agona	0	0	0	0	0	0	0	0	0	0	0
SAL_MA0341AA	FSIS11704820	Agona	0	0	0	0	0	0	0	0	0	0	0
SAL_MA1027AA	PNUSAS025898	Agona	0	0	0	0	0	0	0	0	0	0	0
SAL_MA0965AA	PNUSAS025332	Agona	0	0	0	0	0	0	0	0	0	0	0
SAL_MA1155AA	MS170276	Agona	0	0	0	0	0	0	0	0	0	0	0
SAL_MA1131AA	MS170304	Agona	0	0	0	0	0	0	0	0	0	0	0
SAL_MA1146AA	MS170287	Agona	0	0	0	0	0	0	0	0	0	0	0
SAL_LA2831AA	20100198	Agona	0	0	0	0	0	0	0	0	0	0	0

SAL_LA3131AA	RK_08:MF0017270R	Agona	0	0	0	0	0	0	0	0	0	0	0
SAL_LA3388AA	MS170266	Agona	0	0	0	0	0	0	0	0	0	0	0
SAL_MA1434AA	PNUSAS025508	Agona	0	0	0	0	0	0	0	0	0	0	0
SAL_MA1633AA	PNUSAS025741	Agona	0	0	0	0	0	0	0	0	0	0	0
SAL_MA1565AA	PNUSAS025661	Agona	0	0	0	0	0	0	0	0	0	0	0
SAL_MA1711AA	PNUSAS020914	Agona	0	0	0	0	0	0	0	0	0	0	0
SAL_MA2320AA	FSIS21720342	Agona	0	0	0	0	0	0	0	0	0	0	0
SAL_MA2297AA	FSIS21720362	Agona	0	0	0	0	0	0	0	0	0	0	0
SAL_MA3001AA	D15-005943	Agona	0	0	0	0	0	0	0	0	0	0	0
SAL_MA3000AA	D15-005561	Agona	0	0	0	0	0	0	0	0	0	0	0
SAL_MA2983AA	D15-022799	Agona	0	0	0	0	0	0	0	0	0	0	0
SAL_MA2982AA	D15-025026	Agona	0	0	0	0	0	0	0	0	0	0	0
SAL_MA2943AA	PNUSAS026198	Agona	0	0	0	0	0	0	0	0	0	0	0
SAL_MA2998AA	D15-003906	Agona	0	0	0	0	0	0	0	0	0	0	0
SAL_MA2997AA	D15-001073	Agona	0	0	0	0	0	0	0	0	0	0	0
SAL_MA2885AA	PNUSAS025283	Agona	0	0	0	0	0	0	0	0	0	0	0
SAL_MA2892AA	PNUSAS022537	Agona	0	0	0	0	0	0	0	0	0	0	0
SAL_MA3357AA	PNUSAS024562	Agona	0	0	0	0	0	0	0	0	0	0	0
SAL_MA3354AA	PNUSAS020849	Agona	0	0	0	0	0	0	0	0	0	0	0
SAL_MA3335AA	PNUSAS026418	Agona	0	0	0	0	0	0	0	0	0	0	0
SAL_MA3138AA	D14-056229	Agona	0	0	0	0	0	0	0	0	0	0	0
SAL_MA3141AA	D14-059606	Agona	0	0	0	0	0	0	0	0	0	0	0
SAL_MA3140AA	D14-057411	Agona	0	0	0	0	0	0	0	0	0	0	0
SAL_MA3222AA	PNUSAS024716	Agona	0	0	0	0	0	0	0	0	0	0	0
SAL_MA3514AA	PNUSAS026494	Agona	0	0	0	0	0	0	0	0	0	0	0
SAL_MA3159AA	FSIS11704838	Agona	0	0	0	0	0	0	0	0	0	0	0
SAL_MA3927AA	CFSAN070519	Agona	0	0	0	0	0	0	0	0	0	0	0
SAL_MA3890AA	FSIS11705114	Agona	0	0	0	0	0	0	0	0	0	0	0
SAL_MA3930AA	CFSAN070522	Agona	0	0	0	0	0	0	0	0	0	0	0
SAL_MA4566AA	PNUSAS026824	Agona	0	0	0	0	0	0	0	0	0	0	0
SAL_MA4815AA	PNUSAS027072	Agona	0	0	0	0	0	0	0	0	0	0	0
SAL_MA4753AA	FSIS21720509	Agona	0	0	0	0	0	0	0	0	0	0	0
SAL_MA5036AA	FSIS1702657	Agona	0	0	0	0	0	0	0	0	0	0	0
SAL_MA5232AA	27-22-8101175	Agona	0	0	0	0	0	0	0	0	0	0	0
SAL_MA5248AA	27-13-8101125	Agona	0	0	0	0	0	0	0	0	0	0	0
SAL_MA5285AA	FDA00001150	Agona	0	0	0	0	0	0	0	0	0	0	0
SAL_MA5340AA	FSIS21720516	Agona	0	0	0	0	0	0	0	0	0	0	0
SAL_MA5445AA	28-24-8101211	Agona	0	0	0	0	0	0	0	0	0	0	0
SAL_EA1441AA	FSIS1500761	Agona	0	0	0	0	0	0	0	0	0	0	0
SAL_BA5584AA	WAPHL_SAL-A00768	Agona	0	0	0	0	0	0	0	0	0	0	0
SAL_CA2666AA	14115	Agona	0	0	0	0	0	0	0	0	0	0	0
SAL_BA6094AA	FDA00001159	Agona	0	0	0	0	0	0	0	0	0	0	0
SAL_DA8688AA	FNW19I06	Agona	0	0	0	0	0	0	0	0	0	0	0
SAL_BA2139AA	NY_FSL S10-1759	Agona	0	0	0	0	0	0	0	0	0	0	0
SAL_CA7154AA	FDA00002409	Agona	0	0	0	0	0	0	0	0	0	0	0
SAL_CA5995AA	46610	Agona	0	0	0	0	0	0	0	0	0	0	0
SAL_DA4370AA	WAPHL_SAL-A00419	Agona	0	0	0	0	0	0	0	0	0	0	0
SAL_CA5479AA	69709	Agona	0	0	0	0	0	0	0	0	0	0	0
SAL_CA8752AA	CFSAN027487	Agona	0	0	0	0	0	0	0	0	0	0	0
SAL_CA7843AA	CFSAN028626	Agona	0	0	0	0	0	0	0	0	0	0	0
SAL_DA9717AA	VA_WGS-00052	Agona	0	0	0	0	0	0	0	0	0	0	0
SAL_EA3695AA	CVM N47726	Agona	0	0	0	0	0	0	0	0	0	0	0
SAL_DA1540AA	FDA00004008	Agona	0	0	0	0	0	0	0	0	0	0	0
SAL_HA2165AA	L02899-15	Agona	0	0	0	0	0	0	0	0	0	0	0
SAL_HA1596AA	PNUSAS004707	Agona	0	0	0	0	0	0	0	0	0	0	0
SAL_HA3054AA	FSIS1608202	Agona	0	0	0	0	0	0	0	0	0	0	0
SAL_HA0011AA	PNUSAS004107	Agona	0	0	0	0	0	0	0	0	0	0	0
SAL_FA6442AA	PNUSAS001704	Agona	0	0	0	0	0	0	0	0	0	0	0
SAL_HA5031AA	13-0392	Agona	0	0	0	0	0	0	0	0	0	0	0
SAL_HA3972AA	FSIS1608216	Agona	0	0	0	0	0	0	0	0	0	0	0
SAL_EA9774AA	SL483	Agona	0	0	0	0	0	0	0	0	0	0	0
SAL_FA8912AA	CVM N42469	Agona	0	0	0	0	0	0	0	0	0	0	0
SAL_FA2760AA	CVM N57686F	Agona	0	0	0	0	0	0	0	0	0	0	0
SAL_FA2875AA	CVM N57313F	Agona	0	0	0	0	0	0	0	0	0	0	0
SAL_EA7328AA	6492	Agona	0	0	0	0	0	0	0	0	0	0	0
SAL_EA6179AA	ADRDL-15-5300	Agona	0	0	0	0	0	0	0	0	0	0	0
SAL_IA4794AA	MS160270	Agona	0	0	0	0	0	0	0	0	0	0	0
SAL_IA5347AA	FSIS1710230	Agona	0	0	0	0	0	0	0	0	0	0	0
SAL_JA0213AA	FDA00002524	Agona	0	0	0	0	0	0	0	0	0	0	0
SAL_JA0911AA	PNUSAS010641	Agona	0	0	0	0	0	0	0	0	0	0	0
SAL_JA2563AA	PNUSAS011663	Agona	0	0	0	0	0	0	0	0	0	0	0
SAL_JA4548AA	PNUSAS013082	Agona	0	0	0	0	0	0	0	0	0	0	0
SAL_JA6053AA	FSIS1701114	Agona	0	0	0	0	0	0	0	0	0	0	0

SAL_JA8311AA	351309	Agona	0	0	0	0	0	0	0	0	0	0	0
SAL_JA8779AA	FSIS1700770	Agona	0	0	0	0	0	0	0	0	0	0	0
SAL_BA8880AA	83310	Agona	0	0	0	0	0	0	0	0	0	0	0
SAL_JA9734AA	FSIS1700783	Agona	0	0	0	0	0	0	0	0	0	0	0
SAL_KA6359AA	PNUSAS019133	Agona	0	0	0	0	0	0	0	0	0	0	0
SAL_KA8494AA	PNUSAS017939	Agona	0	0	0	0	0	0	0	0	0	0	0
SAL_KA9734AA	PNUSAS019926	Agona	0	0	0	0	0	0	0	0	0	0	0
SAL_LA3625AA	FSIS21720119	Agona	0	0	0	0	0	0	0	0	0	0	0
SAL_LA4987AA	PNUSAS022631	Agona	0	0	0	0	0	0	0	0	0	0	0
SAL_LA9913AA	2015AM-1541	Agona	0	0	0	0	0	0	0	0	0	0	0
SAL_MA3874AA	FSIS11705132	Agona	0	0	0	0	0	0	0	0	0	0	0
SAL_MA5696AA	ERS2000936	Agona	0	0	0	0	0	0	0	0	0	0	0
SAL_MA5701AA	ERS2000931	Agona	0	0	0	0	0	0	0	0	0	0	0
SAL_MA6069AA	SA20041063	Agona	0	0	0	0	0	0	0	0	0	0	0
SAL_MA6404AA	PNUSAS027750	Agona	0	0	0	0	0	0	0	0	0	0	0
SAL_MA6324AA	PNUSAS025713	Agona	0	0	0	0	0	0	0	0	0	0	0
SAL_MA6101AA	FSIS11704884	Agona	0	0	0	0	0	0	0	0	0	0	0
SAL_MA6636AA	PNUSAS026819	Agona	0	0	0	0	0	0	0	0	0	0	0
SAL_MA6750AA	348008	Agona	0	0	0	0	0	0	0	0	0	0	0
SAL_MA7392AA	CFSAN071804	Agona	0	0	0	0	0	0	0	0	0	0	0
SAL_MA7394AA	ADRDL-1554	Agona	0	0	0	0	0	0	0	0	0	0	0
SAL_MA7310AA	PNUSAS027905	Agona	0	0	0	0	0	0	0	0	0	0	0
SAL_MA7231AA	PNUSAS028181	Agona	0	0	0	0	0	0	0	0	0	0	0
SAL_MA7116AA	CFSAN071477	Agona	0	0	0	0	0	0	0	0	0	0	0
SAL_MA7024AA	CFSAN071845	Agona	0	0	0	0	0	0	0	0	0	0	0
SAL_MA6938AA	FSL R8-8615	Agona	0	0	0	0	0	0	0	0	0	0	0
SAL_MA6923AA	ADRDL-1270	Agona	0	0	0	0	0	0	0	0	0	0	0
SAL_MA6855AA	CFSAN071459	Agona	0	0	0	0	0	0	0	0	0	0	0
SAL_MA6876AA	FSL S10-1761	Agona	0	0	0	0	0	0	0	0	0	0	0
SAL_MA6877AA	FSL S10-1760	Agona	0	0	0	0	0	0	0	0	0	0	0
SAL_MA6866AA	FSL R8-8619	Agona	0	0	0	0	0	0	0	0	0	0	0
SAL_MA6818AA	FSL M8-0485	Agona	0	0	0	0	0	0	0	0	0	0	0
SAL_MA6829AA	SAL-17-VL-SD-IA-0024	Agona	0	0	0	0	0	0	0	0	0	0	0
SAL_MA7298AA	ADRDL-1568	Agona	0	0	0	0	0	0	0	0	0	0	0
SAL_MA7599AA	PNUSAS027978	Agona	0	0	0	0	0	0	0	0	0	0	0
SAL_MA7513AA	ADRDL-1257	Agona	0	0	0	0	0	0	0	0	0	0	0
SAL_MA7853AA	PNUSAS027555	Agona	0	0	0	0	0	0	0	0	0	0	0
SAL_MA7697AA	PNUSAS028118	Agona	0	0	0	0	0	0	0	0	0	0	0
SAL_MA7740AA	PNUSAS027598	Agona	0	0	0	0	0	0	0	0	0	0	0
SAL_MA8093AA	104266	Agona	0	0	0	0	0	0	0	0	0	0	0
SAL_MA8398AA	PNUSAS028778	Agona	0	0	0	0	0	0	0	0	0	0	0
SAL_MA8356AA	PNUSAS028794	Agona	0	0	0	0	0	0	0	0	0	0	0
SAL_MA8252AA	PNUSAS029164	Agona	0	0	0	0	0	0	0	0	0	0	0
SAL_MA8995AA	PNUSAS028751	Agona	0	0	0	0	0	0	0	0	0	0	0
SAL_MA8898AA	FSIS11705999	Agona	0	0	0	0	0	0	0	0	0	0	0
SAL_NA0401AA	CFSAN070571	Agona	0	0	0	0	0	0	0	0	0	0	0
SAL_NA0281AA	PNUSAS029137	Agona	0	0	0	0	0	0	0	0	0	0	0
SAL_NA0225AA	PNUSAS029216	Agona	0	0	0	0	0	0	0	0	0	0	0
SAL_MA9837AA	CVM N16S389	Agona	0	0	0	0	0	0	0	0	0	0	0
SAL_MA9775AA	CVM N16S244	Agona	0	0	0	0	0	0	0	0	0	0	0
SAL_MA9838AA	CVM N16S390	Agona	0	0	0	0	0	0	0	0	0	0	0
SAL_MA9749AA	CVM N16S354	Agona	0	0	0	0	0	0	0	0	0	0	0
SAL_MA9627AA	CVM N16S281	Agona	0	0	0	0	0	0	0	0	0	0	0
SAL_MA9651AA	CVM N16S196	Agona	0	0	0	0	0	0	0	0	0	0	0
SAL_MA9566AA	CVM N16S305	Agona	0	0	0	0	0	0	0	0	0	0	0
SAL_NA0739AA	PNUSAS029247	Agona	0	0	0	0	0	0	0	0	0	0	0
SAL_NA0697AA	PNUSAS029427	Agona	0	0	0	0	0	0	0	0	0	0	0
SAL_NA0661AA	PNUSAS027087	Agona	0	0	0	0	0	0	0	0	0	0	0
SAL_NA0821AA	PNUSAS029474	Agona	0	0	0	0	0	0	0	0	0	0	0
SAL_NA0426AA	FSIS11706044	Agona	0	0	0	0	0	0	0	0	0	0	0
SAL_NA0454AA	FSIS11706168	Agona	0	0	0	0	0	0	0	0	0	0	0
SAL_NA0544AA	ADRDL-1253	Agona	0	0	0	0	0	0	0	0	0	0	0
SAL_NA0605AA	PNUSAS024734	Agona	0	0	0	0	0	0	0	0	0	0	0
SAL_NA0663AA	2017K-1105	Agona	0	0	0	0	0	0	0	0	0	0	0
SAL_NA1128AA	PNUSAS027929	Agona	0	0	0	0	0	0	0	0	0	0	0
SAL_NA1287AA	PNUSAS026042	Agona	0	0	0	0	0	0	0	0	0	0	0
SAL_NA1649AA	FSIS11706315	Agona	0	0	0	0	0	0	0	0	0	0	0
SAL_NA1658AA	FSIS11705512	Agona	0	0	0	0	0	0	0	0	0	0	0
SAL_NA1410AA	CFSAN072249	Agona	0	0	0	0	0	0	0	0	0	0	0
SAL_NA1423AA	CFSAN071458	Agona	0	0	0	0	0	0	0	0	0	0	0
SAL_NA2020AA	CFSAN072618	Agona	0	0	0	0	0	0	0	0	0	0	0
SAL_NA1874AA	PNUSAS029566	Agona	0	0	0	0	0	0	0	0	0	0	0
SAL_NA2067AA	PNUSAS029973	Agona	0	0	0	0	0	0	0	0	0	0	0

SAL_NA2484AA	PNUSAS029917	Agona	0	0	0	0	0	0	0	0	0	0	0
SAL_NA2383AA	CFSAN073495	Agona	0	0	0	0	0	0	0	0	0	0	0
SAL_NA2999AA	PNUSAS030168	Agona	0	0	0	0	0	0	0	0	0	0	0
SAL_NA2798AA	CFSAN031905	Agona	0	0	0	0	0	0	0	0	0	0	0
SAL_NA2753AA	CFSAN031904	Agona	0	0	0	0	0	0	0	0	0	0	0
SAL_NA2758AA	CFSAN031868	Agona	0	0	0	0	0	0	0	0	0	0	0
SAL_NA3038AA	PNUSAS030087	Agona	0	0	0	0	0	0	0	0	0	0	0
SAL_CA0765AA	39.O.03	Agona	0	0	0	0	0	0	0	0	0	0	0
SAL_NA3287AA	FSIS11706649	Agona	0	0	0	0	0	0	0	0	0	0	0
SAL_NA3271AA	CFSAN073565	Agona	0	0	0	0	0	0	0	0	0	0	0
SAL_NA3219AA	CFSAN073567	Agona	0	0	0	0	0	0	0	0	0	0	0
SAL_NA3226AA	CFSAN073571	Agona	0	0	0	0	0	0	0	0	0	0	0
SAL_NA3251AA	CFSAN073592	Agona	0	0	0	0	0	0	0	0	0	0	0
SAL_NA3252AA	CFSAN073591	Agona	0	0	0	0	0	0	0	0	0	0	0
SAL_NA3217AA	CFSAN073568	Agona	0	0	0	0	0	0	0	0	0	0	0
SAL_NA3231AA	FSIS11706301	Agona	0	0	0	0	0	0	0	0	0	0	0
SAL_NA3166AA	CFSAN073593	Agona	0	0	0	0	0	0	0	0	0	0	0
SAL_NA3169AA	CFSAN073566	Agona	0	0	0	0	0	0	0	0	0	0	0
SAL_NA3168AA	CFSAN073594	Agona	0	0	0	0	0	0	0	0	0	0	0
SAL_NA3409AA	FSIS21720864	Agona	0	0	0	0	0	0	0	0	0	0	0
SAL_NA3414AA	FSIS21720867	Agona	0	0	0	0	0	0	0	0	0	0	0
SAL_NA3445AA	PNUSAS027884	Agona	0	0	0	0	0	0	0	0	0	0	0
SAL_NA3478AA	201200491	Agona	0	0	0	0	0	0	0	0	0	0	0
SAL_NA3479AA	201200593	Agona	0	0	0	0	0	0	0	0	0	0	0
SAL_NA3481AA	201200661	Agona	0	0	0	0	0	0	0	0	0	0	0
SAL_NA3650AA	PNUSAS030698	Agona	0	0	0	0	0	0	0	0	0	0	0
SAL_NA3514AA	PNUSAS030831	Agona	0	0	0	0	0	0	0	0	0	0	0
SAL_NA4161AA	PNUSAS030986	Agona	0	0	0	0	0	0	0	0	0	0	0
SAL_NA4118AA	PNUSAS031099	Agona	0	0	0	0	0	0	0	0	0	0	0
SAL_NA4147AA	FSIS11706711	Agona	0	0	0	0	0	0	0	0	0	0	0
SAL_NA4325AA	PNUSAS031340	Agona	0	0	0	0	0	0	0	0	0	0	0
SAL_NA4328AA	PNUSAS031258	Agona	0	0	0	0	0	0	0	0	0	0	0
SAL_NA4424AA	36-1-8100231	Agona	0	0	0	0	0	0	0	0	0	0	0
SAL_NA4425AA	36-2-8100283	Agona	0	0	0	0	0	0	0	0	0	0	0
SAL_NA4449AA	36-20-8101390	Agona	0	0	0	0	0	0	0	0	0	0	0
SAL_NA4531AA	FSIS11706796	Agona	0	0	0	0	0	0	0	0	0	0	0
SAL_NA4518AA	CFSAN074246	Agona	0	0	0	0	0	0	0	0	0	0	0
SAL_NA4653AA	FSIS11706693	Agona	0	0	0	0	0	0	0	0	0	0	0
SAL_NA4634AA	FSIS11706974	Agona	0	0	0	0	0	0	0	0	0	0	0
SAL_NA4878AA	PNUSAS031602	Agona	0	0	0	0	0	0	0	0	0	0	0
SAL_NA4933AA	PNUSAS031650	Agona	0	0	0	0	0	0	0	0	0	0	0
SAL_NA5139AA	PNUSAS031797	Agona	0	0	0	0	0	0	0	0	0	0	0
SAL_NA5134AA	PNUSAS031625	Agona	0	0	0	0	0	0	0	0	0	0	0
SAL_NA5435AA	FDA00012488	Agona	0	0	0	0	0	0	0	0	0	0	0
SAL_NA5359AA	PNUSAS030994	Agona	0	0	0	0	0	0	0	0	0	0	0
SAL_NA5252AA	2015AM-2321	Agona	0	0	0	0	0	0	0	0	0	0	0
SAL_NA5536AA	PNUSAS029422	Agona	0	0	0	0	0	0	0	0	0	0	0
SAL_NA5547AA	PNUSAS031977	Agona	0	0	0	0	0	0	0	0	0	0	0
SAL_NA5458AA	PNUSAS031793	Agona	0	0	0	0	0	0	0	0	0	0	0
SAL_NA5652AA	200206185	Agona	0	0	0	0	0	0	0	0	0	0	0
SAL_NA5653AA	201208107	Agona	0	0	0	0	0	0	0	0	0	0	0
SAL_NA5654AA	201109617	Agona	0	0	0	0	0	0	0	0	0	0	0
SAL_NA5655AA	200607575	Agona	0	0	0	0	0	0	0	0	0	0	0
SAL_NA6197AA	CFSAN074228	Agona	0	0	0	0	0	0	0	0	0	0	0
SAL_NA6194AA	CFSAN074229	Agona	0	0	0	0	0	0	0	0	0	0	0
SAL_NA6059AA	CFSAN074222	Agona	0	0	0	0	0	0	0	0	0	0	0
SAL_NA6061AA	2015AM-2565	Agona	0	0	0	0	0	0	0	0	0	0	0
SAL_NA6646AA	02-13-18.8100032	Agona	0	0	0	0	0	0	0	0	0	0	0
SAL_NA6662AA	02-22-18.8100041	Agona	0	0	0	0	0	0	0	0	0	0	0
SAL_NA5839AA	CFSAN074244	Agona	0	0	0	0	0	0	0	0	0	0	0
SAL_NA5924AA	PNUSAS031723	Agona	0	0	0	0	0	0	0	0	0	0	0
SAL_NA5857AA	CFSAN074240	Agona	0	0	0	0	0	0	0	0	0	0	0
SAL_NA5862AA	CFSAN074245	Agona	0	0	0	0	0	0	0	0	0	0	0
SAL_NA5844AA	CFSAN074241	Agona	0	0	0	0	0	0	0	0	0	0	0
SAL_NA5841AA	CFSAN074242	Agona	0	0	0	0	0	0	0	0	0	0	0
SAL_NA5842AA	CFSAN074243	Agona	0	0	0	0	0	0	0	0	0	0	0
SAL_NA5672AA	CFSAN074790	Agona	0	0	0	0	0	0	0	0	0	0	0
SAL_NA5674AA	CFSAN074789	Agona	0	0	0	0	0	0	0	0	0	0	0
SAL_NA6560AA	PNUSAS031494	Agona	0	0	0	0	0	0	0	0	0	0	0
SAL_NA6436AA	FSIS11706284	Agona	0	0	0	0	0	0	0	0	0	0	0
SAL_NA6448AA	2015AM-2937	Agona	0	0	0	0	0	0	0	0	0	0	0
SAL_NA6399AA	FSIS11706958	Agona	0	0	0	0	0	0	0	0	0	0	0
SAL_NA6296AA	FSIS21820953	Agona	0	0	0	0	0	0	0	0	0	0	0

SAL_NA7037AA	PNUSAS028956	Agona	0	0	0	0	0	0	0	0	0	0	0
SAL_NA6903AA	PNUSAS032167	Agona	0	0	0	0	0	0	0	0	0	0	0
SAL_NA6836AA	PNUSAS031813	Agona	0	0	0	0	0	0	0	0	0	0	0
SAL_NA6775AA	PNUSAS032514	Agona	0	0	0	0	0	0	0	0	0	0	0
SAL_NA6786AA	PNUSAS032515	Agona	0	0	0	0	0	0	0	0	0	0	0
SAL_NA7182AA	PNUSAS032469	Agona	0	0	0	0	0	0	0	0	0	0	0
SAL_NA6873AA	PNUSAS031896	Agona	0	0	0	0	0	0	0	0	0	0	0
SAL_NA7258AA	3-17-8100862	Agona	0	0	0	0	0	0	0	0	0	0	0
SAL_CA0746AA	49.E.09	Agona	0	0	0	0	0	0	0	0	0	0	0
SAL_CA0707AA	68.U.05	Agona	0	0	0	0	0	0	0	0	0	0	0
SAL_CA0709AA	67.H.09	Agona	0	0	0	0	0	0	0	0	0	0	0
SAL_CA0748AA	48.E.08	Agona	0	0	0	0	0	0	0	0	0	0	0
SAL_CA0711AA	66.F.99	Agona	0	0	0	0	0	0	0	0	0	0	0
SAL_CA0727AA	58.E.08	Agona	0	0	0	0	0	0	0	0	0	0	0
SAL_CA0742AA	51.E.09	Agona	0	0	0	0	0	0	0	0	0	0	0
SAL_CA0756AA	44.E.09	Agona	0	0	0	0	0	0	0	0	0	0	0
SAL_CA0750AA	47.E.09	Agona	0	0	0	0	0	0	0	0	0	0	0
SAL_CA0753AA	46.E.09	Agona	0	0	0	0	0	0	0	0	0	0	0
SAL_CA0754AA	45.E.09	Agona	0	0	0	0	0	0	0	0	0	0	0
SAL_CA0698AA	72.A.52	Agona	0	0	0	0	0	0	0	0	0	0	0
SAL_CA0762AA	41.E.09	Agona	0	0	0	0	0	0	0	0	0	0	0
SAL_CA0830AA	04.O.05	Agona	0	0	0	0	0	0	0	0	0	0	0
SAL_CA0767AA	38.O.03	Agona	0	0	0	0	0	0	0	0	0	0	0
SAL_CA0836AA	01.O.05	Agona	0	0	0	0	0	0	0	0	0	0	0
SAL_CA0713AA	65.H.72	Agona	0	0	0	0	0	0	0	0	0	0	0
SAL_CA0832AA	03.O.05	Agona	0	0	0	0	0	0	0	0	0	0	0
SAL_CA0719AA	62.H.72	Agona	0	0	0	0	0	0	0	0	0	0	0
SAL_CA0717AA	63.H.87	Agona	0	0	0	0	0	0	0	0	0	0	0
SAL_CA0771AA	36.H.00	Agona	0	0	0	0	0	0	0	0	0	0	0
SAL_CA0769AA	37.F.02	Agona	0	0	0	0	0	0	0	0	0	0	0
SAL_CA0785AA	29.O.08	Agona	0	0	0	0	0	0	0	0	0	0	0
SAL_CA0783AA	30.H.04	Agona	0	0	0	0	0	0	0	0	0	0	0
SAL_CA0775AA	34.H.09	Agona	0	0	0	0	0	0	0	0	0	0	0
SAL_CA0779AA	32.A.00	Agona	0	0	0	0	0	0	0	0	0	0	0
SAL_CA0777AA	33.A.05	Agona	0	0	0	0	0	0	0	0	0	0	0
SAL_CA0793AA	24.H.04	Agona	0	0	0	0	0	0	0	0	0	0	0
SAL_CA0786AA	28.O.08	Agona	0	0	0	0	0	0	0	0	0	0	0
SAL_CA0788AA	27.O.98	Agona	0	0	0	0	0	0	0	0	0	0	0
SAL_CA0794AA	23.F.01	Agona	0	0	0	0	0	0	0	0	0	0	0
SAL_CA0802AA	19.F.03	Agona	0	0	0	0	0	0	0	0	0	0	0
SAL_CA0798AA	21.H.10	Agona	0	0	0	0	0	0	0	0	0	0	0
SAL_CA0814AA	12.A.06	Agona	0	0	0	0	0	0	0	0	0	0	0
SAL_CA0810AA	15.H.03	Agona	0	0	0	0	0	0	0	0	0	0	0
SAL_CA0826AA	06.O.05	Agona	0	0	0	0	0	0	0	0	0	0	0
SAL_CA0824AA	07.O.05	Agona	0	0	0	0	0	0	0	0	0	0	0
SAL_CA0760AA	42.E.09	Agona	0	0	0	0	0	0	0	0	0	0	0
SAL_CA0806AA	17.H.06	Agona	0	0	0	0	0	0	0	0	0	0	0
SAL_CA0804AA	18.H.07	Agona	0	0	0	0	0	0	0	0	0	0	0
SAL_NA7736AA	17-94873_S29	Agona	0	0	0	0	0	0	0	0	0	0	0
SAL_NA7270AA	CFSAN071986	Agona	0	0	0	0	0	0	0	0	0	0	0
SAL_NA7265AA	CFSAN071985	Agona	0	0	0	0	0	0	0	0	0	0	0
SAL_NA7268AA	CFSAN071980	Agona	0	0	0	0	0	0	0	0	0	0	0
SAL_NA7269AA	CFSAN071984	Agona	0	0	0	0	0	0	0	0	0	0	0
SAL_NA7271AA	CFSAN071983	Agona	0	0	0	0	0	0	0	0	0	0	0
SAL_NA7266AA	CFSAN071982	Agona	0	0	0	0	0	0	0	0	0	0	0
SAL_NA7267AA	CFSAN071981	Agona	0	0	0	0	0	0	0	0	0	0	0
SAL_NA8207AA	CFSAN071973	Agona	0	0	0	0	0	0	0	0	0	0	0
SAL_NA8182AA	CFSAN071979	Agona	0	0	0	0	0	0	0	0	0	0	0
SAL_NA8179AA	CFSAN071976	Agona	0	0	0	0	0	0	0	0	0	0	0
SAL_NA8183AA	CFSAN071975	Agona	0	0	0	0	0	0	0	0	0	0	0
SAL_NA8184AA	CFSAN071974	Agona	0	0	0	0	0	0	0	0	0	0	0
SAL_NA8180AA	CFSAN071977	Agona	0	0	0	0	0	0	0	0	0	0	0
SAL_NA8181AA	CFSAN071978	Agona	0	0	0	0	0	0	0	0	0	0	0
SAL_NA8096AA	2015AM-3606	Agona	0	0	0	0	0	0	0	0	0	0	0
SAL_NA8564AA	PNUSAS032995	Agona	0	0	0	0	0	0	0	0	0	0	0
SAL_NA8496AA	PNUSAS032850	Agona	0	0	0	0	0	0	0	0	0	0	0
SAL_NA8497AA	PNUSAS032234	Agona	0	0	0	0	0	0	0	0	0	0	0
SAL_NA8475AA	PNUSAS032236	Agona	0	0	0	0	0	0	0	0	0	0	0
SAL_NA8474AA	PNUSAS032235	Agona	0	0	0	0	0	0	0	0	0	0	0
SAL_NA8464AA	PNUSAS032237	Agona	0	0	0	0	0	0	0	0	0	0	0
SAL_NA8386AA	PNUSAS028450	Agona	0	0	0	0	0	0	0	0	0	0	0
SAL_NA8381AA	PNUSAS028449	Agona	0	0	0	0	0	0	0	0	0	0	0
SAL_NA8330AA	PNUSAS032419	Agona	0	0	0	0	0	0	0	0	0	0	0

SAL_NA8358AA	PNUSAS029379	Agona	0	0	0	0	0	0	0	0	0	0	0
SAL_NA8829AA	PNUSAS033144	Agona	0	0	0	0	0	0	0	0	0	0	0
SAL_NA8738AA	FSIS21821040	Agona	0	0	0	0	0	0	0	0	0	0	0
SAL_NA8760AA	CFSAN075237	Agona	0	0	0	0	0	0	0	0	0	0	0
SAL_NA8657AA	PNUSAS033255	Agona	0	0	0	0	0	0	0	0	0	0	0
SAL_NA8708AA	PNUSAS033099	Agona	0	0	0	0	0	0	0	0	0	0	0
SAL_NA9509AA	PNUSAS033643	Agona	0	0	0	0	0	0	0	0	0	0	0
SAL_NA9452AA	PNUSAS033338	Agona	0	0	0	0	0	0	0	0	0	0	0
SAL_NA9424AA	PNUSAS032518	Agona	0	0	0	0	0	0	0	0	0	0	0
SAL_NA9353AA	PNUSAS033567	Agona	0	0	0	0	0	0	0	0	0	0	0
SAL_NA9257AA	PNUSAS029878	Agona	0	0	0	0	0	0	0	0	0	0	0
SAL_OA0095AA	PNUSAS028875	Agona	0	0	0	0	0	0	0	0	0	0	0
SAL_OA0011AA	PNUSAS032199	Agona	0	0	0	0	0	0	0	0	0	0	0
SAL_NA9974AA	FSIS11807559	Agona	0	0	0	0	0	0	0	0	0	0	0
SAL_OA0592AA	PNUSAS033627	Agona	0	0	0	0	0	0	0	0	0	0	0
SAL_OA0569AA	FSIS11807539	Agona	0	0	0	0	0	0	0	0	0	0	0
SAL_OA1120AA	PNUSAS034057	Agona	0	0	0	0	0	0	0	0	0	0	0
SAL_OA1045AA	PNUSAS034924	Agona	0	0	0	0	0	0	0	0	0	0	0
SAL_OA0994AA	CFSAN075483	Agona	0	0	0	0	0	0	0	0	0	0	0
SAL_OA5912AA	FSIS31800252	Agona	0	0	0	0	0	0	0	0	0	0	0
SAL_OA5750AA	PNUSAS033211	Agona	0	0	0	0	0	0	0	0	0	0	0
SAL_OA5692AA	ADRDL-1874	Agona	0	0	0	0	0	0	0	0	0	0	0
SAL_OA7316AA	FSIS31800281	Agona	0	0	0	0	0	0	0	0	0	0	0
SAL_OA5433AA	PNUSAS035643	Agona	0	0	0	0	0	0	0	0	0	0	0
SAL_OA5185AA	PNUSAS035865	Agona	0	0	0	0	0	0	0	0	0	0	0
SAL_PA0024AA	PNUSAS036328	Agona	0	0	0	0	0	0	0	0	0	0	0
SAL_PA0034AA	PNUSAS037930	Agona	0	0	0	0	0	0	0	0	0	0	0
SAL_OA7837AA	PNUSAS035709	Agona	0	0	0	0	0	0	0	0	0	0	0
SAL_OA4762AA	PNUSAS036869	Agona	0	0	0	0	0	0	0	0	0	0	0
SAL_OA4721AA	PNUSAS036668	Agona	0	0	0	0	0	0	0	0	0	0	0
SAL_OA4681AA	PNUSAS025407	Agona	0	0	0	0	0	0	0	0	0	0	0
SAL_OA4539AA	PNUSAS036130	Agona	0	0	0	0	0	0	0	0	0	0	0
SAL_OA4493AA	PNUSAS036571	Agona	0	0	0	0	0	0	0	0	0	0	0
SAL_OA4264AA	PNUSAS033325	Agona	0	0	0	0	0	0	0	0	0	0	0
SAL_OA4452AA	PNUSAS036176	Agona	0	0	0	0	0	0	0	0	0	0	0
SAL_OA3775AA	FSIS31800218	Agona	0	0	0	0	0	0	0	0	0	0	0
SAL_PA2116AA	14-25-18-8100274-T	Agona	0	0	0	0	0	0	0	0	0	0	0
SAL_OA9719AA	sg_wb19	Agona	0	0	0	0	0	0	0	0	0	0	0
SAL_PA0914AA	PNUSAS035845	Agona	0	0	0	0	0	0	0	0	0	0	0
SAL_PA1147AA	FSIS31800308	Agona	0	0	0	0	0	0	0	0	0	0	0
SAL_PA1011AA	PNUSAS036890	Agona	0	0	0	0	0	0	0	0	0	0	0
SAL_PA1313AA	PNUSAS038121	Agona	0	0	0	0	0	0	0	0	0	0	0
SAL_PA2850AA	15-5-18-8100270-E	Agona	0	0	0	0	0	0	0	0	0	0	0
SAL_PA2856AA	15-15-18-8100287-W	Agona	0	0	0	0	0	0	0	0	0	0	0
SAL_PA1931AA	PNUSAS038734	Agona	0	0	0	0	0	0	0	0	0	0	0
SAL_PA2103AA	CFSAN078665	Agona	0	0	0	0	0	0	0	0	0	0	0
SAL_PA2618AA	PNUSAS035385	Agona	0	0	0	0	0	0	0	0	0	0	0
SAL_PA3942AA	FDA00004340	Agona	0	0	0	0	0	0	0	0	0	0	0
SAL_PA3945AA	FDA00004341	Agona	0	0	0	0	0	0	0	0	0	0	0
SAL_PA4162AA	CFSAN078630	Agona	0	0	0	0	0	0	0	0	0	0	0
SAL_PA4314AA	495813	Agona	0	0	0	0	0	0	0	0	0	0	0
SAL_PA4318AA	371812	Agona	0	0	0	0	0	0	0	0	0	0	0
SAL_PA4278AA	122686	Agona	0	0	0	0	0	0	0	0	0	0	0
SAL_PA4286AA	159901	Agona	0	0	0	0	0	0	0	0	0	0	0
SAL_PA4257AA	312801	Agona	0	0	0	0	0	0	0	0	0	0	0
SAL_PA4446AA	PNUSAS039501	Agona	0	0	0	0	0	0	0	0	0	0	0
SAL_PA4628AA	363489	Agona	0	0	0	0	0	0	0	0	0	0	0
SAL_PA4629AA	493445	Agona	0	0	0	0	0	0	0	0	0	0	0
SAL_PA4630AA	500290	Agona	0	0	0	0	0	0	0	0	0	0	0
SAL_PA4632AA	373913	Agona	0	0	0	0	0	0	0	0	0	0	0
SAL_PA4345AA	PNUSAS039817	Agona	0	0	0	0	0	0	0	0	0	0	0
SAL_PA4608AA	528056	Agona	0	0	0	0	0	0	0	0	0	0	0
SAL_PA4609AA	373186	Agona	0	0	0	0	0	0	0	0	0	0	0
SAL_PA4610AA	516906	Agona	0	0	0	0	0	0	0	0	0	0	0
SAL_PA4611AA	524395	Agona	0	0	0	0	0	0	0	0	0	0	0
SAL_PA4612AA	104258	Agona	0	0	0	0	0	0	0	0	0	0	0
SAL_PA4613AA	363613	Agona	0	0	0	0	0	0	0	0	0	0	0
SAL_PA4614AA	363528	Agona	0	0	0	0	0	0	0	0	0	0	0
SAL_PA4615AA	526781	Agona	0	0	0	0	0	0	0	0	0	0	0
SAL_PA4616AA	527494	Agona	0	0	0	0	0	0	0	0	0	0	0
SAL_PA4617AA	361786	Agona	0	0	0	0	0	0	0	0	0	0	0
SAL_PA4618AA	527499	Agona	0	0	0	0	0	0	0	0	0	0	0
SAL_PA4619AA	259448	Agona	0	0	0	0	0	0	0	0	0	0	0

SAL_PA4620AA	508284	Agona	0	0	0	0	0	0	0	0	0	0	0
SAL_PA4621AA	524381	Agona	0	0	0	0	0	0	0	0	0	0	0
SAL_PA4622AA	511621	Agona	0	0	0	0	0	0	0	0	0	0	0
SAL_PA4623AA	516928	Agona	0	0	0	0	0	0	0	0	0	0	0
SAL_PA4624AA	283143	Agona	0	0	0	0	0	0	0	0	0	0	0
SAL_PA4625AA	245628	Agona	0	0	0	0	0	0	0	0	0	0	0
SAL_PA4626AA	506069	Agona	0	0	0	0	0	0	0	0	0	0	0
SAL_PA4627AA	360997	Agona	0	0	0	0	0	0	0	0	0	0	0
SAL_PA4587AA	376335	Agona	0	0	0	0	0	0	0	0	0	0	0
SAL_PA4588AA	524394	Agona	0	0	0	0	0	0	0	0	0	0	0
SAL_PA4590AA	173714	Agona	0	0	0	0	0	0	0	0	0	0	0
SAL_PA4591AA	527512	Agona	0	0	0	0	0	0	0	0	0	0	0
SAL_PA4592AA	241778	Agona	0	0	0	0	0	0	0	0	0	0	0
SAL_PA4593AA	363533	Agona	0	0	0	0	0	0	0	0	0	0	0
SAL_PA4594AA	520149	Agona	0	0	0	0	0	0	0	0	0	0	0
SAL_PA4595AA	399954	Agona	0	0	0	0	0	0	0	0	0	0	0
SAL_PA4596AA	360510	Agona	0	0	0	0	0	0	0	0	0	0	0
SAL_PA4597AA	524787	Agona	0	0	0	0	0	0	0	0	0	0	0
SAL_PA4598AA	399908	Agona	0	0	0	0	0	0	0	0	0	0	0
SAL_PA4599AA	199853	Agona	0	0	0	0	0	0	0	0	0	0	0
SAL_PA4600AA	207582	Agona	0	0	0	0	0	0	0	0	0	0	0
SAL_PA4601AA	348695	Agona	0	0	0	0	0	0	0	0	0	0	0
SAL_PA4602AA	215121	Agona	0	0	0	0	0	0	0	0	0	0	0
SAL_PA4603AA	236263	Agona	0	0	0	0	0	0	0	0	0	0	0
SAL_PA4604AA	203992	Agona	0	0	0	0	0	0	0	0	0	0	0
SAL_PA4605AA	358424	Agona	0	0	0	0	0	0	0	0	0	0	0
SAL_PA4606AA	345587	Agona	0	0	0	0	0	0	0	0	0	0	0
SAL_PA4607AA	408501	Agona	0	0	0	0	0	0	0	0	0	0	0
SAL_PA4576AA	101327	Agona	0	0	0	0	0	0	0	0	0	0	0
SAL_PA4575AA	240168	Agona	0	0	0	0	0	0	0	0	0	0	0
SAL_PA4577AA	363511	Agona	0	0	0	0	0	0	0	0	0	0	0
SAL_PA4578AA	511650	Agona	0	0	0	0	0	0	0	0	0	0	0
SAL_PA4581AA	527516	Agona	0	0	0	0	0	0	0	0	0	0	0
SAL_PA4580AA	365844	Agona	0	0	0	0	0	0	0	0	0	0	0
SAL_PA4584AA	142520	Agona	0	0	0	0	0	0	0	0	0	0	0
SAL_PA4583AA	142270	Agona	0	0	0	0	0	0	0	0	0	0	0
SAL_PA4582AA	526784	Agona	0	0	0	0	0	0	0	0	0	0	0
SAL_PA4585AA	376304	Agona	0	0	0	0	0	0	0	0	0	0	0
SAL_PA4586AA	509961	Agona	0	0	0	0	0	0	0	0	0	0	0
SAL_PA4579AA	374052	Agona	0	0	0	0	0	0	0	0	0	0	0
SAL_PA4589AA	365801	Agona	0	0	0	0	0	0	0	0	0	0	0
SAL_PA4651AA	PNUSAS037268	Agona	0	0	0	0	0	0	0	0	0	0	0
SAL_PA4805AA	401774	Agona	0	0	0	0	0	0	0	0	0	0	0
SAL_PA4831AA	373891	Agona	0	0	0	0	0	0	0	0	0	0	0
SAL_PA4639AA	PNUSAS039795	Agona	0	0	0	0	0	0	0	0	0	0	0
SAL_PA5150AA	507291	Agona	0	0	0	0	0	0	0	0	0	0	0
SAL_PA5151AA	236237	Agona	0	0	0	0	0	0	0	0	0	0	0
SAL_PA5152AA	348739	Agona	0	0	0	0	0	0	0	0	0	0	0
SAL_PA5153AA	500382	Agona	0	0	0	0	0	0	0	0	0	0	0
SAL_PA5090AA	PNUSAS039858	Agona	0	0	0	0	0	0	0	0	0	0	0
SAL_PA5015AA	CFSAN080436	Agona	0	0	0	0	0	0	0	0	0	0	0
SAL_PA4886AA	316268	Agona	0	0	0	0	0	0	0	0	0	0	0
SAL_PA5818AA	18-19-18-8100288-A	Agona	0	0	0	0	0	0	0	0	0	0	0
SAL_PA5573AA	FSIS11809712	Agona	0	0	0	0	0	0	0	0	0	0	0
SAL_PA5716AA	PNUSAS040032	Agona	0	0	0	0	0	0	0	0	0	0	0
SAL_PA5665AA	PNUSAS039763	Agona	0	0	0	0	0	0	0	0	0	0	0
SAL_PA5620AA	285595	Agona	0	0	0	0	0	0	0	0	0	0	0
SAL_PA5622AA	316346	Agona	0	0	0	0	0	0	0	0	0	0	0
SAL_PA5627AA	249339	Agona	0	0	0	0	0	0	0	0	0	0	0
SAL_PA5607AA	520177	Agona	0	0	0	0	0	0	0	0	0	0	0
SAL_PA5616AA	528032	Agona	0	0	0	0	0	0	0	0	0	0	0
SAL_PA6006AA	PNUSAS038471	Agona	0	0	0	0	0	0	0	0	0	0	0
SAL_PA6242AA	387135	Agona	0	0	0	0	0	0	0	0	0	0	0
SAL_PA6224AA	187506	Agona	0	0	0	0	0	0	0	0	0	0	0
SAL_PA6197AA	265752	Agona	0	0	0	0	0	0	0	0	0	0	0
SAL_PA6180AA	352830	Agona	0	0	0	0	0	0	0	0	0	0	0
SAL_PA6185AA	293056	Agona	0	0	0	0	0	0	0	0	0	0	0
SAL_PA6193AA	107279	Agona	0	0	0	0	0	0	0	0	0	0	0
SAL_PA6139AA	113494	Agona	0	0	0	0	0	0	0	0	0	0	0
SAL_PA6346AA	PNUSAS040025	Agona	0	0	0	0	0	0	0	0	0	0	0
SAL_PA6352AA	PNUSAS037754	Agona	0	0	0	0	0	0	0	0	0	0	0
SAL_PA6330AA	PNUSAS040581	Agona	0	0	0	0	0	0	0	0	0	0	0
SAL_PA6269AA	PNUSAS040216	Agona	0	0	0	0	0	0	0	0	0	0	0

SAL_PA6449AA	528493	Agona	0	0	0	0	0	0	0	0	0	0	0
SAL_PA6500AA	PNUSAS040462	Agona	0	0	0	0	0	0	0	0	0	0	0
SAL_PA7469AA	19-30-18-8100405.D	Agona	0	0	0	0	0	0	0	0	0	0	0
SAL_PA6550AA	370616	Agona	0	0	0	0	0	0	0	0	0	0	0
SAL_PA7143AA	FSIS11809878	Agona	0	0	0	0	0	0	0	0	0	0	0
SAL_PA7293AA	273626	Agona	0	0	0	0	0	0	0	0	0	0	0
SAL_PA7249AA	CFSAN080911	Agona	0	0	0	0	0	0	0	0	0	0	0
SAL_PA7259AA	381659	Agona	0	0	0	0	0	0	0	0	0	0	0
SAL_PA7237AA	FSIS11809383	Agona	0	0	0	0	0	0	0	0	0	0	0
SAL_PA7393AA	PNUSAS039806	Agona	0	0	0	0	0	0	0	0	0	0	0
SAL_PA7513AA	306539	Agona	0	0	0	0	0	0	0	0	0	0	0
SAL_PA7628AA	PNUSAS041081	Agona	0	0	0	0	0	0	0	0	0	0	0
SAL_PA7784AA	208936	Agona	0	0	0	0	0	0	0	0	0	0	0
SAL_PA7749AA	321061	Agona	0	0	0	0	0	0	0	0	0	0	0
SAL_PA7750AA	529857	Agona	0	0	0	0	0	0	0	0	0	0	0
SAL_PA7772AA	207562	Agona	0	0	0	0	0	0	0	0	0	0	0
SAL_PA7793AA	176411	Agona	0	0	0	0	0	0	0	0	0	0	0
SAL_PA7744AA	536082	Agona	0	0	0	0	0	0	0	0	0	0	0
SAL_PA7726AA	279533	Agona	0	0	0	0	0	0	0	0	0	0	0
SAL_PA8003AA	279392	Agona	0	0	0	0	0	0	0	0	0	0	0
SAL_PA7950AA	288889	Agona	0	0	0	0	0	0	0	0	0	0	0
SAL_PA7876AA	478521	Agona	0	0	0	0	0	0	0	0	0	0	0
SAL_PA8263AA	PNUSAS041353	Agona	0	0	0	0	0	0	0	0	0	0	0
SAL_PA8264AA	PNUSAS041351	Agona	0	0	0	0	0	0	0	0	0	0	0
SAL_PA8362AA	174778	Agona	0	0	0	0	0	0	0	0	0	0	0
SAL_PA8371AA	529244	Agona	0	0	0	0	0	0	0	0	0	0	0
SAL_PA8402AA	116040	Agona	0	0	0	0	0	0	0	0	0	0	0
SAL_PA8206AA	PNUSAS041063	Agona	0	0	0	0	0	0	0	0	0	0	0
SAL_PA8792AA	PNUSAS041190	Agona	0	0	0	0	0	0	0	0	0	0	0
SAL_PA8581AA	363499	Agona	0	0	0	0	0	0	0	0	0	0	0
SAL_PA8583AA	432746	Agona	0	0	0	0	0	0	0	0	0	0	0
SAL_PA8830AA	20-9-18-8100412-Q	Agona	0	0	0	0	0	0	0	0	0	0	0
SAL_PA8484AA	290789	Agona	0	0	0	0	0	0	0	0	0	0	0
SAL_PA8612AA	211145	Agona	0	0	0	0	0	0	0	0	0	0	0
SAL_PA8592AA	301612	Agona	0	0	0	0	0	0	0	0	0	0	0
SAL_PA8845AA	20-15-18-8100418-J	Agona	0	0	0	0	0	0	0	0	0	0	0
SAL_PA8847AA	20-10-18-8100413-H	Agona	0	0	0	0	0	0	0	0	0	0	0
SAL_PA9927AA	21-10-18-8100443-R	Agona	0	0	0	0	0	0	0	0	0	0	0
SAL_PA9932AA	21-18-18-8100451-Q	Agona	0	0	0	0	0	0	0	0	0	0	0
SAL_PA9174AA	PNUSAS041445	Agona	0	0	0	0	0	0	0	0	0	0	0
SAL_PA9187AA	PNUSAS041594	Agona	0	0	0	0	0	0	0	0	0	0	0
SAL_PA9043AA	267822	Agona	0	0	0	0	0	0	0	0	0	0	0
SAL_PA9050AA	427429	Agona	0	0	0	0	0	0	0	0	0	0	0
SAL_PA9373AA	FSIS11809866	Agona	0	0	0	0	0	0	0	0	0	0	0
SAL_PA9038AA	135143	Agona	0	0	0	0	0	0	0	0	0	0	0
SAL_PA8893AA	432175	Agona	0	0	0	0	0	0	0	0	0	0	0
SAL_PA9528AA	516868	Agona	0	0	0	0	0	0	0	0	0	0	0
SAL_PA9474AA	372670	Agona	0	0	0	0	0	0	0	0	0	0	0
SAL_PA9460AA	421164	Agona	0	0	0	0	0	0	0	0	0	0	0
SAL_PA9485AA	534004	Agona	0	0	0	0	0	0	0	0	0	0	0
SAL_PA9433AA	CFSAN081691	Agona	0	0	0	0	0	0	0	0	0	0	0
SAL_PA9560AA	PNUSAS041303	Agona	0	0	0	0	0	0	0	0	0	0	0
SAL_PA9552AA	PNUSAS041299	Agona	0	0	0	0	0	0	0	0	0	0	0
SAL_PA9633AA	397392	Agona	0	0	0	0	0	0	0	0	0	0	0
SAL_PA9643AA	155605	Agona	0	0	0	0	0	0	0	0	0	0	0
SAL_PA9615AA	FSIS11808801	Agona	0	0	0	0	0	0	0	0	0	0	0
SAL_PA9618AA	FSIS11809634	Agona	0	0	0	0	0	0	0	0	0	0	0
SAL_PA9986AA	368292	Agona	0	0	0	0	0	0	0	0	0	0	0
SAL_PA9955AA	168471	Agona	0	0	0	0	0	0	0	0	0	0	0
SAL_QA0169AA	PNUSAS041934	Agona	0	0	0	0	0	0	0	0	0	0	0
SAL_QA0077AA	PNUSAS041552	Agona	0	0	0	0	0	0	0	0	0	0	0
SAL_QA0509AA	539051	Agona	0	0	0	0	0	0	0	0	0	0	0
SAL_QA0491AA	402760	Agona	0	0	0	0	0	0	0	0	0	0	0
SAL_QA0468AA	435371	Agona	0	0	0	0	0	0	0	0	0	0	0
SAL_QA0450AA	182894	Agona	0	0	0	0	0	0	0	0	0	0	0
SAL_QA0452AA	200649	Agona	0	0	0	0	0	0	0	0	0	0	0
SAL_QA0389AA	421689	Agona	0	0	0	0	0	0	0	0	0	0	0
SAL_QA0728AA	371908	Agona	0	0	0	0	0	0	0	0	0	0	0
SAL_QA1958AA	23-17-18-8100492-W	Agona	0	0	0	0	0	0	0	0	0	0	0
SAL_QA0461AA	274307	Agona	0	0	0	0	0	0	0	0	0	0	0
SAL_QA1366AA	539045	Agona	0	0	0	0	0	0	0	0	0	0	0
SAL_QA1343AA	259258	Agona	0	0	0	0	0	0	0	0	0	0	0
SAL_QA1338AA	248554	Agona	0	0	0	0	0	0	0	0	0	0	0

SAL_QA1311AA	352094	Agona	0	0	0	0	0	0	0	0	0	0
SAL_QA1322AA	467173	Agona	0	0	0	0	0	0	0	0	0	0
SAL_QA1166AA	FSIS11810196	Agona	0	0	0	0	0	0	0	0	0	0
SAL_QA3753AA	24-12-18-8100514-Y	Agona	0	0	0	0	0	0	0	0	0	0
SAL_QA1050AA	PNUSAS040197	Agona	0	0	0	0	0	0	0	0	0	0
SAL_QA1054AA	PNUSAS041759	Agona	0	0	0	0	0	0	0	0	0	0
SAL_QA0872AA	399487	Agona	0	0	0	0	0	0	0	0	0	0
SAL_QA0836AA	186584	Agona	0	0	0	0	0	0	0	0	0	0
SAL_QA0849AA	539878	Agona	0	0	0	0	0	0	0	0	0	0
SAL_QA1542AA	373846	Agona	0	0	0	0	0	0	0	0	0	0
SAL_QA1545AA	506864	Agona	0	0	0	0	0	0	0	0	0	0
SAL_QA1473AA	193351	Agona	0	0	0	0	0	0	0	0	0	0
SAL_QA1448AA	193304	Agona	0	0	0	0	0	0	0	0	0	0
SAL_QA1450AA	362119	Agona	0	0	0	0	0	0	0	0	0	0
SAL_QA1457AA	529850	Agona	0	0	0	0	0	0	0	0	0	0
SAL_QA1446AA	265294	Agona	0	0	0	0	0	0	0	0	0	0
SAL_QA1426AA	425578	Agona	0	0	0	0	0	0	0	0	0	0
SAL_QA1893AA	509908	Agona	0	0	0	0	0	0	0	0	0	0
SAL_QA1904AA	412227	Agona	0	0	0	0	0	0	0	0	0	0
SAL_QA1833AA	528494	Agona	0	0	0	0	0	0	0	0	0	0
SAL_QA1743AA	428140	Agona	0	0	0	0	0	0	0	0	0	0
SAL_QA2507AA	142205	Agona	0	0	0	0	0	0	0	0	0	0
SAL_QA2444AA	399364	Agona	0	0	0	0	0	0	0	0	0	0
SAL_QA2393AA	109274	Agona	0	0	0	0	0	0	0	0	0	0
SAL_QA2398AA	414751	Agona	0	0	0	0	0	0	0	0	0	0
SAL_QA2401AA	440013	Agona	0	0	0	0	0	0	0	0	0	0
SAL_QA2364AA	546583	Agona	0	0	0	0	0	0	0	0	0	0
SAL_QA2351AA	264167	Agona	0	0	0	0	0	0	0	0	0	0
SAL_QA2177AA	359267	Agona	0	0	0	0	0	0	0	0	0	0
SAL_QA2148AA	265753	Agona	0	0	0	0	0	0	0	0	0	0
SAL_QA2033AA	378602	Agona	0	0	0	0	0	0	0	0	0	0
SAL_QA2042AA	449462	Agona	0	0	0	0	0	0	0	0	0	0
SAL_QA2622AA	FSIS31800542	Agona	0	0	0	0	0	0	0	0	0	0
SAL_QA1990AA	535271	Agona	0	0	0	0	0	0	0	0	0	0
SAL_QA2596AA	FSIS31800544	Agona	0	0	0	0	0	0	0	0	0	0
SAL_QA2928AA	FSIS11810458	Agona	0	0	0	0	0	0	0	0	0	0
SAL_QA2892AA	432288	Agona	0	0	0	0	0	0	0	0	0	0
SAL_QA2848AA	288804	Agona	0	0	0	0	0	0	0	0	0	0
SAL_QA2793AA	532483	Agona	0	0	0	0	0	0	0	0	0	0
SAL_QA2806AA	FSIS11810474	Agona	0	0	0	0	0	0	0	0	0	0
SAL_QA2769AA	180556	Agona	0	0	0	0	0	0	0	0	0	0
SAL_QA2679AA	178939	Agona	0	0	0	0	0	0	0	0	0	0
SAL_QA2653AA	513310	Agona	0	0	0	0	0	0	0	0	0	0
SAL_QA2662AA	467351	Agona	0	0	0	0	0	0	0	0	0	0
SAL_QA2986AA	PNUSAS043027	Agona	0	0	0	0	0	0	0	0	0	0
SAL_QA3052AA	PNUSAS042059	Agona	0	0	0	0	0	0	0	0	0	0
SAL_QA1441AA	424575	Agona	0	0	0	0	0	0	0	0	0	0
SAL_QA3041AA	PNUSAS042744	Agona	0	0	0	0	0	0	0	0	0	0
SAL_QA3500AA	543875	Agona	0	0	0	0	0	0	0	0	0	0
SAL_QA3423AA	175579	Agona	0	0	0	0	0	0	0	0	0	0
SAL_QA3405AA	136573	Agona	0	0	0	0	0	0	0	0	0	0
SAL_QA3353AA	ADRDL-1936	Agona	0	0	0	0	0	0	0	0	0	0
SAL_QA3356AA	416220	Agona	0	0	0	0	0	0	0	0	0	0
SAL_QA3337AA	516314	Agona	0	0	0	0	0	0	0	0	0	0
SAL_QA3344AA	253905	Agona	0	0	0	0	0	0	0	0	0	0
SAL_QA3411AA	304603	Agona	0	0	0	0	0	0	0	0	0	0
SAL_QA3291AA	412093	Agona	0	0	0	0	0	0	0	0	0	0
SAL_QA3224AA	484133	Agona	0	0	0	0	0	0	0	0	0	0
SAL_QA3212AA	414666	Agona	0	0	0	0	0	0	0	0	0	0
SAL_QA3539AA	PNUSAS039053	Agona	0	0	0	0	0	0	0	0	0	0
SAL_QA3537AA	PNUSAS039068	Agona	0	0	0	0	0	0	0	0	0	0
SAL_QA3934AA	171287	Agona	0	0	0	0	0	0	0	0	0	0
SAL_QA3903AA	435375	Agona	0	0	0	0	0	0	0	0	0	0
SAL_QA3884AA	320949	Agona	0	0	0	0	0	0	0	0	0	0
SAL_QA3858AA	421173	Agona	0	0	0	0	0	0	0	0	0	0
SAL_QA3860AA	445180	Agona	0	0	0	0	0	0	0	0	0	0
SAL_QA3897AA	377307	Agona	0	0	0	0	0	0	0	0	0	0
SAL_QA2400AA	545276	Agona	0	0	0	0	0	0	0	0	0	0
SAL_QA4076AA	416215	Agona	0	0	0	0	0	0	0	0	0	0
SAL_QA4000AA	118894	Agona	0	0	0	0	0	0	0	0	0	0
SAL_QA4320AA	PNUSAS037585	Agona	0	0	0	0	0	0	0	0	0	0
SAL_QA4285AA	PNUSAS039020	Agona	0	0	0	0	0	0	0	0	0	0
SAL_QA4572AA	PNUSAS043440	Agona	0	0	0	0	0	0	0	0	0	0

SAL_QA4960AA	351329	Agona	0	0	0	0	0	0	0	0	0	0	0
SAL_QA4950AA	539067	Agona	0	0	0	0	0	0	0	0	0	0	0
SAL_QA4954AA	533432	Agona	0	0	0	0	0	0	0	0	0	0	0
SAL_QA4937AA	185451	Agona	0	0	0	0	0	0	0	0	0	0	0
SAL_QA4940AA	134272	Agona	0	0	0	0	0	0	0	0	0	0	0
SAL_QA4936AA	238442	Agona	0	0	0	0	0	0	0	0	0	0	0
SAL_QA4882AA	170370	Agona	0	0	0	0	0	0	0	0	0	0	0
SAL_QA4888AA	428154	Agona	0	0	0	0	0	0	0	0	0	0	0
SAL_QA4886AA	418896	Agona	0	0	0	0	0	0	0	0	0	0	0
SAL_QA4873AA	352101	Agona	0	0	0	0	0	0	0	0	0	0	0
SAL_QA4856AA	157337	Agona	0	0	0	0	0	0	0	0	0	0	0
SAL_QA4815AA	164910	Agona	0	0	0	0	0	0	0	0	0	0	0
SAL_QA4768AA	127414	Agona	0	0	0	0	0	0	0	0	0	0	0
SAL_QA4766AA	552409	Agona	0	0	0	0	0	0	0	0	0	0	0
SAL_QA4692AA	382852	Agona	0	0	0	0	0	0	0	0	0	0	0
SAL_QA4719AA	553254	Agona	0	0	0	0	0	0	0	0	0	0	0
SAL_QA4740AA	109256	Agona	0	0	0	0	0	0	0	0	0	0	0
SAL_QA5560AA	248566	Agona	0	0	0	0	0	0	0	0	0	0	0
SAL_QA5569AA	388699	Agona	0	0	0	0	0	0	0	0	0	0	0
SAL_QA5539AA	146105	Agona	0	0	0	0	0	0	0	0	0	0	0
SAL_QA5519AA	157297	Agona	0	0	0	0	0	0	0	0	0	0	0
SAL_QA5531AA	206693	Agona	0	0	0	0	0	0	0	0	0	0	0
SAL_QA5411AA	352126	Agona	0	0	0	0	0	0	0	0	0	0	0
SAL_QA5254AA	409974	Agona	0	0	0	0	0	0	0	0	0	0	0
SAL_QA5244AA	169636	Agona	0	0	0	0	0	0	0	0	0	0	0
SAL_QA5199AA	428187	Agona	0	0	0	0	0	0	0	0	0	0	0
SAL_QA5645AA	PNUSAS043647	Agona	0	0	0	0	0	0	0	0	0	0	0
SAL_QA5155AA	135018	Agona	0	0	0	0	0	0	0	0	0	0	0
SAL_QA5607AA	PNUSAS043164	Agona	0	0	0	0	0	0	0	0	0	0	0
SAL_QA5125AA	296704	Agona	0	0	0	0	0	0	0	0	0	0	0
SAL_QA5075AA	316308	Agona	0	0	0	0	0	0	0	0	0	0	0
SAL_QA5086AA	299011	Agona	0	0	0	0	0	0	0	0	0	0	0
SAL_QA5959AA	505456	Agona	0	0	0	0	0	0	0	0	0	0	0
SAL_QA4053AA	376217	Agona	0	0	0	0	0	0	0	0	0	0	0
SAL_QA4058AA	140201	Agona	0	0	0	0	0	0	0	0	0	0	0
SAL_QA5910AA	539154	Agona	0	0	0	0	0	0	0	0	0	0	0
SAL_QA5856AA	352146	Agona	0	0	0	0	0	0	0	0	0	0	0
SAL_QA5901AA	427519	Agona	0	0	0	0	0	0	0	0	0	0	0
SAL_QA5774AA	454886	Agona	0	0	0	0	0	0	0	0	0	0	0
SAL_QA5807AA	299014	Agona	0	0	0	0	0	0	0	0	0	0	0
SAL_QA5838AA	534027	Agona	0	0	0	0	0	0	0	0	0	0	0
SAL_QA6015AA	PNUSAS041872	Agona	0	0	0	0	0	0	0	0	0	0	0
SAL_QA6010AA	PNUSAS042750	Agona	0	0	0	0	0	0	0	0	0	0	0
SAL_QA6296AA	535297	Agona	0	0	0	0	0	0	0	0	0	0	0
SAL_QA6283AA	420618	Agona	0	0	0	0	0	0	0	0	0	0	0
SAL_QA6167AA	548593	Agona	0	0	0	0	0	0	0	0	0	0	0
SAL_QA6597AA	140594	Agona	0	0	0	0	0	0	0	0	0	0	0
SAL_QA6596AA	433582	Agona	0	0	0	0	0	0	0	0	0	0	0
SAL_QA6537AA	536048	Agona	0	0	0	0	0	0	0	0	0	0	0
SAL_QA6542AA	539161	Agona	0	0	0	0	0	0	0	0	0	0	0
SAL_QA6545AA	435433	Agona	0	0	0	0	0	0	0	0	0	0	0
SAL_QA6549AA	211155	Agona	1	1	1	78	1	1	1	1	4	1	1
SAL_QA6554AA	529819	Agona	0	0	0	0	0	0	0	0	0	0	0
SAL_QA6501AA	492544	Agona	0	0	0	0	0	0	0	0	0	0	0
SAL_QA6512AA	448256	Agona	0	0	0	0	0	0	0	0	0	0	0
SAL_QA6518AA	201509	Agona	0	0	0	0	0	0	0	0	0	0	0
SAL_QA6525AA	531243	Agona	0	0	0	0	0	0	0	0	0	0	0
SAL_QA6912AA	PNUSAS043828	Agona	0	0	0	0	0	0	0	0	0	0	0
SAL_QA5325AA	369875	Agona	0	0	0	0	0	0	0	0	0	0	0
SAL_QA6769AA	404893	Agona	0	0	0	0	0	0	0	0	0	0	0
SAL_QA6730AA	251830	Agona	0	0	0	0	0	0	0	0	0	0	0
SAL_QA6731AA	248567	Agona	0	0	0	0	0	0	0	0	0	0	0
SAL_QA6735AA	272429	Agona	0	0	0	0	0	0	0	0	0	0	0
SAL_QA6710AA	199802	Agona	0	0	0	0	0	0	0	0	0	0	0
SAL_QA7225AA	FDA00011275	Agona	0	0	0	0	0	0	0	0	0	0	0
SAL_QA7173AA	PNUSAS043491	Agona	0	0	0	0	0	0	0	0	0	0	0
SAL_RA0482AA	SMG-18-843	Agona	0	0	0	0	0	0	0	0	0	0	0
SAL_QA7013AA	PNUSAS043317	Agona	0	0	0	0	0	0	0	0	0	0	0
SAL_QA7402AA	533425	Agona	0	0	0	0	0	0	0	0	0	0	0
SAL_QA7406AA	492623	Agona	0	0	0	0	0	0	0	0	0	0	0
SAL_QA7419AA	100484	Agona	0	0	0	0	0	0	0	0	0	0	0
SAL_QA7337AA	391774	Agona	0	0	0	0	0	0	0	0	0	0	0
SAL_QA7293AA	110968	Agona	0	0	0	0	0	0	0	0	0	0	0

SAL_QA7259AA	425681	Agona	0	0	0	0	0	0	0	0	0	0	0
SAL_QA7925AA	387069	Agona	0	0	0	0	0	0	0	0	0	0	0
SAL_QA7903AA	374585	Agona	0	0	0	0	0	0	0	0	0	0	0
SAL_QA6253AA	480708	Agona	0	0	0	0	0	0	0	0	0	0	0
SAL_QA7820AA	432772	Agona	0	0	0	0	0	0	0	0	0	0	0
SAL_QA6403AA	256145	Agona	0	0	0	0	0	0	0	0	0	0	0
SAL_QA7689AA	186534	Agona	0	0	0	0	0	0	0	0	0	0	0
SAL_QA7705AA	205726	Agona	0	0	0	0	0	0	0	0	0	0	0
SAL_QA7684AA	409992	Agona	0	0	0	0	0	0	0	0	0	0	0
SAL_QA8133AA	FSIS11810951	Agona	0	0	0	0	0	0	0	0	0	0	0
SAL_QA7595AA	118963	Agona	0	0	0	0	0	0	0	0	0	0	0
SAL_QA8078AA	FSIS11810943	Agona	0	0	0	0	0	0	0	0	0	0	0
SAL_QA7566AA	296802	Agona	0	0	0	0	0	0	0	0	0	0	0
SAL_QA7568AA	373304	Agona	0	0	0	0	0	0	0	0	0	0	0
SAL_QA7513AA	486487	Agona	0	0	0	0	0	0	0	0	0	0	0
SAL_QA7524AA	399968	Agona	0	0	0	0	0	0	0	0	0	0	0
SAL_QA7507AA	449721	Agona	0	0	0	0	0	0	0	0	0	0	0
SAL_QA8434AA	391105	Agona	0	0	0	0	0	0	0	0	0	0	0
SAL_QA8354AA	434316	Agona	0	0	0	0	0	0	0	0	0	0	0
SAL_QA8289AA	238366	Agona	0	0	0	0	0	0	0	0	0	0	0
SAL_QA8295AA	533383	Agona	0	0	0	0	0	0	0	0	0	0	0
SAL_QA8254AA	529242	Agona	0	0	0	0	0	0	0	0	0	0	0
SAL_QA8264AA	418242	Agona	0	0	0	0	0	0	0	0	0	0	0
SAL_QA8229AA	296299	Agona	0	0	0	0	0	0	0	0	0	0	0
SAL_QA8195AA	412021	Agona	0	0	0	0	0	0	0	0	0	0	0
SAL_QA8204AA	553884	Agona	0	0	0	0	0	0	0	0	0	0	0
SAL_RA4611AA	452473	Agona	0	0	0	0	0	0	0	0	0	0	0
SAL_RA4610AA	452472	Agona	0	0	0	0	0	0	0	0	0	0	0
SAL_RA4612AA	452474	Agona	0	0	0	0	0	0	0	0	0	0	0
SAL_RA4614AA	452475	Agona	0	0	0	0	0	0	0	0	0	0	0
SAL_RA4682AA	376355	Agona	0	0	0	0	0	0	0	0	0	0	0
SAL_RA4685AA	376358	Agona	0	0	0	0	0	0	0	0	0	0	0
SAL_QA8806AA	439508	Agona	0	0	0	0	0	0	0	0	0	0	0
SAL_QA8764AA	166381	Agona	0	0	0	0	0	0	0	0	0	0	0
SAL_QA8670AA	529853	Agona	0	3	5	83	3	0	4	4	5	5	1
SAL_QA8686AA	548927	Agona	0	0	0	0	0	0	0	0	0	0	0
SAL_QA8648AA	211142	Agona	0	0	0	0	0	0	0	0	0	0	0
SAL_QA8980AA	PNUSAS044765	Agona	0	0	0	0	0	0	0	0	0	0	0
SAL_QA8936AA	PNUSAS044434	Agona	0	0	0	0	0	0	0	0	0	0	0
SAL_QA8914AA	FSIS11811129	Agona	0	0	0	0	0	0	0	0	0	0	0
SAL_QA9535AA	399969	Agona	0	0	0	0	0	0	0	0	0	0	0
SAL_QA9440AA	250758	Agona	0	0	0	0	0	0	0	0	0	0	0
SAL_QA9394AA	120591	Agona	0	0	0	0	0	0	0	0	0	0	0
SAL_QA9337AA	373153	Agona	0	0	0	0	0	0	0	0	0	0	0
SAL_QA9296AA	305265	Agona	0	0	0	0	0	0	0	0	0	0	0
SAL_QA9310AA	535355	Agona	0	0	0	0	0	0	0	0	0	0	0
SAL_QA9277AA	321070	Agona	0	0	0	0	0	0	0	0	0	0	0
SAL_QA9700AA	FSIS11811112	Agona	0	0	0	0	0	0	0	0	0	0	0
SAL_QA9255AA	CE06.035	Agona	0	0	0	0	0	0	0	0	0	0	0
SAL_RA6997AA	SMG-18-937	Agona	0	0	0	0	0	0	0	0	0	0	0
SAL_RA0004AA	557667	Agona	0	0	0	0	0	0	0	0	0	0	0
SAL_QA9818AA	302548	Agona	0	0	0	0	0	0	0	0	0	0	0
SAL_QA9819AA	418219	Agona	0	0	0	0	0	0	0	0	0	0	0
SAL_RA0168AA	PNUSAS044035	Agona	0	0	0	0	0	0	0	0	0	0	0
SAL_RA0259AA	376175	Agona	0	0	0	0	0	0	0	0	0	0	0
SAL_RA0229AA	430253	Agona	0	0	0	0	0	0	0	0	0	0	0
SAL_RA0209AA	120638	Agona	0	0	0	0	0	0	0	0	0	0	0
SAL_RA1122AA	399307	Agona	0	0	0	0	0	0	0	0	0	0	0
SAL_RA0179AA	457943	Agona	0	0	0	0	0	0	0	0	0	0	0
SAL_RA1106AA	373277	Agona	0	0	0	0	0	0	0	0	0	0	0
SAL_RA1050AA	443524	Agona	0	0	0	0	0	0	0	0	0	0	0
SAL_RA1025AA	182857	Agona	0	0	0	0	0	0	0	0	0	0	0
SAL_RA1005AA	272430	Agona	0	0	0	0	0	0	0	0	0	0	0
SAL_RA0957AA	434341	Agona	0	0	0	0	0	0	0	0	0	0	0
SAL_RA0963AA	316232	Agona	0	0	0	0	0	0	0	0	0	0	0
SAL_RA0894AA	178840	Agona	0	0	0	0	0	0	0	0	0	0	0
SAL_RA0910AA	465603	Agona	0	0	0	0	0	0	0	0	0	0	0
SAL_RA0832AA	436073	Agona	0	0	0	0	0	0	0	0	0	0	0
SAL_RA0842AA	436058	Agona	0	0	0	0	0	0	0	0	0	0	0
SAL_RA0761AA	524309	Agona	0	0	0	0	0	0	0	0	0	0	0
SAL_RA0735AA	323033	Agona	0	0	0	0	0	0	0	0	0	0	0
SAL_RA0757AA	116571	Agona	0	0	0	0	0	0	0	0	0	0	0
SAL_RA0768AA	409977	Agona	0	0	0	0	0	0	0	0	0	0	0

SAL_RA0604AA	224118	Agona	0	0	0	0	0	0	0	0	0	0	0
SAL_RA0545AA	528550	Agona	0	0	0	0	0	0	0	0	0	0	0
SAL_RA1136AA	FDA00013161	Agona	0	0	0	0	0	0	0	0	0	0	0
SAL_RA1354AA	339508	Agona	0	0	0	0	0	0	0	0	0	0	0
SAL_QA8790AA	550765	Agona	0	0	0	0	0	0	0	0	0	0	0
SAL_QA8791AA	545310	Agona	0	0	0	0	0	0	0	0	0	0	0
SAL_RA2518AA	PNUSAS045023	Agona	0	0	0	0	0	0	0	0	0	0	0
SAL_RA2444AA	1305F39594	Agona	0	0	0	0	0	0	0	0	0	0	0
SAL_RA2445AA	1708T62231	Agona	0	0	0	0	0	0	0	0	0	0	0
SAL_RA2446AA	1310H13608	Agona	0	0	0	0	0	0	0	0	0	0	0
SAL_RA2447AA	1708F23406	Agona	0	0	0	0	0	0	0	0	0	0	0
SAL_RA2448AA	1710F30084	Agona	0	0	0	0	0	0	0	0	0	0	0
SAL_RA2449AA	1706H44906	Agona	0	0	0	0	0	0	0	0	0	0	0
SAL_RA2450AA	1705S48857	Agona	0	0	0	0	0	0	0	0	0	0	0
SAL_RA2451AA	1706F17922	Agona	0	0	0	0	0	0	0	0	0	0	0
SAL_RA2452AA	1706H44746	Agona	0	0	0	0	0	0	0	0	0	0	0
SAL_RA2453AA	1706H45773	Agona	0	0	0	0	0	0	0	0	0	0	0
SAL_RA2454AA	1706T55154	Agona	0	0	0	0	0	0	0	0	0	0	0
SAL_RA2455AA	1706F19609	Agona	0	0	0	0	0	0	0	0	0	0	0
SAL_RA2421AA	1306H57878	Agona	0	0	0	0	0	0	0	0	0	0	0
SAL_RA2426AA	1308F51880	Agona	0	0	0	0	0	0	0	0	0	0	0
SAL_RA2427AA	1308T7328	Agona	0	0	0	0	0	0	0	0	0	0	0
SAL_RA2428AA	1309T11277	Agona	0	0	0	0	0	0	0	0	0	0	0
SAL_RA2431AA	1310S47870	Agona	0	0	0	0	0	0	0	0	0	0	0
SAL_RA2432AA	1310T13555	Agona	0	0	0	0	0	0	0	0	0	0	0
SAL_RA2433AA	1311M34750	Agona	0	0	0	0	0	0	0	0	0	0	0
SAL_RA2434AA	1311S48182	Agona	0	0	0	0	0	0	0	0	0	0	0
SAL_RA2435AA	1312T22371	Agona	0	0	0	0	0	0	0	0	0	0	0
SAL_RA2436AA	1402F14974	Agona	0	0	0	0	0	0	0	0	0	0	0
SAL_RA2437AA	1312T23841	Agona	0	0	0	0	0	0	0	0	0	0	0
SAL_RA2438AA	1401H28157	Agona	0	0	0	0	0	0	0	0	0	0	0
SAL_RA2439AA	1401M40562	Agona	0	0	0	0	0	0	0	0	0	0	0
SAL_RA2442AA	1410F41142	Agona	0	0	0	0	0	0	0	0	0	0	0
SAL_RA2443AA	1411S5999	Agona	0	0	0	0	0	0	0	0	0	0	0
SAL_RA2456AA	1706M52807	Agona	0	0	0	0	0	0	0	0	0	0	0
SAL_RA2422AA	1311T17267	Agona	0	0	0	0	0	0	0	0	0	0	0
SAL_RA2423AA	1311W33515	Agona	0	0	0	0	0	0	0	0	0	0	0
SAL_RA2424AA	1403T33190	Agona	0	0	0	0	0	0	0	0	0	0	0
SAL_RA2425AA	1508S18910	Agona	0	0	0	0	0	0	0	0	0	0	0
SAL_RA2429AA	1309T11455	Agona	0	0	0	0	0	0	0	0	0	0	0
SAL_RA2430AA	1310F408	Agona	0	0	0	0	0	0	0	0	0	0	0
SAL_RA2440AA	1401M40570	Agona	0	0	0	0	0	0	0	0	0	0	0
SAL_RA2441AA	1402W46457	Agona	0	0	0	0	0	0	0	0	0	0	0
SAL_RA2761AA	348043	Agona	0	0	0	0	0	0	0	0	0	0	0
SAL_RA2784AA	201515	Agona	0	0	0	0	0	0	0	0	0	0	0
SAL_RA2786AA	430179	Agona	0	0	0	0	0	0	0	0	0	0	0
SAL_RA2731AA	101477	Agona	0	0	0	0	0	0	0	0	0	0	0
SAL_RA2739AA	301762	Agona	0	0	0	0	0	0	0	0	0	0	0
SAL_RA2762AA	209664	Agona	0	0	0	0	0	0	0	0	0	0	0
SAL_RA8630AA	SMG-18-1077	Agona	0	0	0	0	0	0	0	0	0	0	0
SAL_RA2923AA	531314	Agona	0	0	0	0	0	0	0	0	0	0	0
SAL_RA2903AA	543885	Agona	0	0	0	0	0	0	0	0	0	0	0
SAL_RA2813AA	FSIS11811253	Agona	0	0	0	0	0	0	0	0	0	0	0
SAL_RA3432AA	106546	Agona	0	0	0	0	0	0	0	0	0	0	0
SAL_RA3379AA	545279	Agona	0	0	0	0	0	0	0	0	0	0	0
SAL_RA3396AA	531308	Agona	0	0	0	0	0	0	0	0	0	0	0
SAL_RA3372AA	550768	Agona	0	0	0	0	0	0	0	0	0	0	0
SAL_RA3337AA	550775	Agona	0	0	0	0	0	0	0	0	0	0	0
SAL_RA3301AA	152492	Agona	0	0	0	0	0	0	0	0	0	0	0
SAL_RA3268AA	387533	Agona	0	0	0	0	0	0	0	0	0	0	0
SAL_RA3274AA	294776	Agona	0	0	0	0	0	0	0	0	0	0	0
SAL_RA3286AA	287741	Agona	0	0	0	0	0	0	0	0	0	0	0
SAL_RA3258AA	527517	Agona	0	0	0	0	0	0	0	0	0	0	0
SAL_RA3260AA	203625	Agona	0	0	0	0	0	0	0	0	0	0	0
SAL_RA3692AA	474898	Agona	0	0	0	0	0	0	0	0	0	0	0
SAL_RA3669AA	391074	Agona	0	0	0	0	0	0	0	0	0	0	0
SAL_RA3695AA	541999	Agona	0	0	0	0	0	0	0	0	0	0	0
SAL_RA3627AA	224146	Agona	0	0	0	0	0	0	0	0	0	0	0
SAL_RA3658AA	456195	Agona	0	0	0	0	0	0	0	0	0	0	0
SAL_RA3601AA	172418	Agona	0	0	0	0	0	0	0	0	0	0	0
SAL_RA3603AA	519518	Agona	0	0	0	0	0	0	0	0	0	0	0
SAL_RA3551AA	363556	Agona	0	0	0	0	0	0	0	0	0	0	0
SAL_RA3536AA	FSIS11811385	Agona	0	0	0	0	0	0	0	0	0	0	0

SAL_RA3531AA	FSIS11811485	Agona	0	0	0	0	0	0	0	0	0	0	0
SAL_RA4187AA	FSIS11811391	Agona	0	0	0	0	0	0	0	0	0	0	0
SAL_RA4177AA	500410	Agona	0	0	0	0	0	0	0	0	0	0	0
SAL_RA4105AA	174656	Agona	0	0	0	0	0	0	0	0	0	0	0
SAL_RA4025AA	459905	Agona	0	0	0	0	0	0	0	0	0	0	0
SAL_RA4047AA	500352	Agona	0	0	0	0	0	0	0	0	0	0	0
SAL_RA4021AA	373945	Agona	0	0	0	0	0	0	0	0	0	0	0
SAL_RA4234AA	FDA00013162	Agona	0	0	0	0	0	0	0	0	0	0	0
SAL_RA3897AA	224120	Agona	0	0	0	0	0	0	0	0	0	0	0
SAL_QA9893AA	209979	Agona	0	0	0	0	0	0	0	0	0	0	0
SAL_RA4566AA	458079	Agona	0	0	0	0	0	0	0	0	0	0	0
SAL_QA9824AA	531209	Agona	0	0	0	0	0	0	0	0	0	0	0
SAL_RA4533AA	423877	Agona	0	0	0	0	0	0	0	0	0	0	0
SAL_RA4494AA	417396	Agona	0	0	0	0	0	0	0	0	0	0	0
SAL_RA4505AA	492495	Agona	0	0	0	0	0	0	0	0	0	0	0
SAL_RA4470AA	435385	Agona	0	0	0	0	0	0	0	0	0	0	0
SAL_RA4475AA	435387	Agona	0	0	0	0	0	0	0	0	0	0	0
SAL_RA4382AA	229104	Agona	0	0	0	0	0	0	0	0	0	0	0
SAL_RA4329AA	217083	Agona	0	0	0	0	0	0	0	0	0	0	0
SAL_RA4310AA	178666	Agona	0	0	0	0	0	0	0	0	0	0	0
SAL_RA4316AA	449713	Agona	0	0	0	0	0	0	0	0	0	0	0
SAL_RA4317AA	376354	Agona	0	0	0	0	0	0	0	0	0	0	0
SAL_RA4325AA	451016	Agona	0	0	0	0	0	0	0	0	0	0	0
SAL_RA4287AA	323018	Agona	0	0	0	0	0	0	0	0	0	0	0
SAL_RA4280AA	317691	Agona	0	0	0	0	0	0	0	0	0	0	0
SAL_RA6003AA	PNUSAS045388	Agona	0	0	0	0	0	0	0	0	0	0	0
SAL_RA5811AA	178635	Agona	0	0	0	0	0	0	0	0	0	0	0
SAL_RA5787AA	235053	Agona	0	0	0	0	0	0	0	0	0	0	0
SAL_RA5696AA	452471	Agona	0	0	0	0	0	0	0	0	0	0	0
SAL_RA5700AA	458078	Agona	0	0	0	0	0	0	0	0	0	0	0
SAL_RA5709AA	423883	Agona	0	0	0	0	0	0	0	0	0	0	0
SAL_RA5714AA	534030	Agona	0	0	0	0	0	0	0	0	0	0	0
SAL_RA5660AA	423873	Agona	0	0	0	0	0	0	0	0	0	0	0
SAL_RA5576AA	318414	Agona	0	0	0	0	0	0	0	0	0	0	0
SAL_RA5586AA	435384	Agona	0	0	0	0	0	0	0	0	0	0	0
SAL_RA5589AA	435381	Agona	0	0	0	0	0	0	0	0	0	0	0
SAL_RA5592AA	449640	Agona	0	0	0	0	0	0	0	0	0	0	0
SAL_RA5617AA	199942	Agona	0	0	0	0	0	0	0	0	0	0	0
SAL_RA5544AA	435383	Agona	0	0	0	0	0	0	0	0	0	0	0
SAL_RA5554AA	323021	Agona	0	0	0	0	0	0	0	0	0	0	0
SAL_RA5600AA	417392	Agona	0	0	0	0	0	0	0	0	0	0	0
SAL_RA5546AA	542005	Agona	0	0	0	0	0	0	0	0	0	0	0
SAL_RA5580AA	113043	Agona	0	0	0	0	0	0	0	0	0	0	0
SAL_RA5548AA	404308	Agona	0	0	0	0	0	0	0	0	0	0	0
SAL_RA5432AA	417402	Agona	0	0	0	0	0	0	0	0	0	0	0
SAL_RA5453AA	435382	Agona	0	0	0	0	0	0	0	0	0	0	0
SAL_SA0883AA	SMG-18-1160	Agona	0	0	0	0	0	0	0	0	0	0	0
SAL_RA2614AA	387046	Agona	0	0	0	0	0	0	0	0	0	0	0
SAL_RA2416AA	1703H35660	Agona	0	0	0	0	0	0	0	0	0	0	0
SAL_RA2420AA	1510T44974	Agona	0	0	0	0	0	0	0	0	0	0	0
SAL_RA2415AA	1509T42140	Agona	0	0	0	0	0	0	0	0	0	0	0
SAL_RA2419AA	0305F12290	Agona	0	0	0	0	0	0	0	0	0	0	0
SAL_RA2417AA	1508T37294	Agona	0	0	0	0	0	0	0	0	0	0	0
SAL_RA2418AA	1510H38748	Agona	0	0	0	0	0	0	0	0	0	0	0
SAL_RA6450AA	PNUSAS046038	Agona	0	0	0	0	0	0	0	0	0	0	0
SAL_SA1009AA	SMG-18-1192	Agona	0	0	0	0	0	0	0	0	0	0	0
SAL_RA6409AA	PNUSAS046022	Agona	0	0	0	0	0	0	0	0	0	0	0
SAL_RA6417AA	PNUSAS045821	Agona	0	0	0	0	0	0	0	0	0	0	0
SAL_RA6701AA	FSIS11811660	Agona	0	0	0	0	0	0	0	0	0	0	0
SAL_RA6651AA	510169	Agona	0	0	0	0	0	0	0	0	0	0	0
SAL_RA6636AA	520589	Agona	0	0	0	0	0	0	0	0	0	0	0
SAL_RA6639AA	112984	Agona	0	0	0	0	0	0	0	0	0	0	0
SAL_RA6642AA	291790	Agona	0	0	0	0	0	0	0	0	0	0	0
SAL_SA1423AA	DB_18	Agona	0	0	0	0	0	0	0	0	0	0	0
SAL_SA1427AA	NJ08.122	Agona	0	0	0	0	0	0	0	0	0	0	0
SAL_SA2144AA	0611W34312	Agona	0	0	0	0	0	0	0	0	0	0	0
SAL_SA1535AA	M218 Contaminant	Agona	0	0	0	0	0	0	0	0	0	0	0
SAL_SA1978AA	ZJ-959	Agona	0	0	0	0	0	0	0	0	0	0	0
SAL_SA1977AA	ZJ-923	Agona	0	0	0	0	0	0	0	0	0	0	0
SAL_SA1975AA	ZJ-102	Agona	0	0	0	0	0	0	0	0	0	0	0
SAL_SA1874AA	05-0529	Agona	0	0	0	0	0	0	0	0	0	0	0
SAL_SA1870AA	SF_R0483/09	Agona	0	0	0	0	0	0	0	0	0	0	0
SAL_SA1869AA	SF_R0453/09	Agona	0	0	0	0	0	0	0	0	0	0	0

SAL_SA1868AA	SF_R0450/09	Agona	0	0	0	0	0	0	0	0	0	0	0
SAL_SA1867AA	SF_R0417/09	Agona	0	0	0	0	0	0	0	0	0	0	0
SAL_SA1866AA	SF_R0414/09	Agona	0	0	0	0	0	0	0	0	0	0	0
SAL_SA1862AA	SF_R0372/09	Agona	0	0	0	0	0	0	0	0	0	0	0
SAL_SA1860AA	SF_R0364/09	Agona	0	0	0	0	0	0	0	0	0	0	0
SAL_SA1859AA	SF_R0363/09	Agona	0	0	0	0	0	0	0	0	0	0	0
SAL_SA1858AA	SF_R0361/09	Agona	0	0	0	0	0	0	0	0	0	0	0
SAL_SA1857AA	SF_R0360/09	Agona	0	0	0	0	0	0	0	0	0	0	0
SAL_SA1856AA	SF_R0359/09	Agona	0	0	0	0	0	0	0	0	0	0	0
SAL_SA1855AA	SF_R0358/09	Agona	0	0	0	0	0	0	0	0	0	0	0
SAL_SA1854AA	SF_R0355/09	Agona	0	0	0	0	0	0	0	0	0	0	0
SAL_SA1853AA	SF_R0347/09	Agona	0	0	0	0	0	0	0	0	0	0	0
SAL_SA1852AA	SF_R0346/09	Agona	0	0	0	0	0	0	0	0	0	0	0
SAL_SA1851AA	SF_R0321/09	Agona	0	0	0	0	0	0	0	0	0	0	0
SAL_SA1850AA	SF_R0319/09	Agona	0	0	0	0	0	0	0	0	0	0	0
SAL_SA1848AA	SF_R0314/09	Agona	0	0	0	0	0	0	0	0	0	0	0
SAL_SA1847AA	SF_R0313/09	Agona	0	0	0	0	0	0	0	0	0	0	0
SAL_SA1846AA	SF_R0312/09	Agona	0	0	0	0	0	0	0	0	0	0	0
SAL_SA1845AA	SF_R0311/09	Agona	0	0	0	0	0	0	0	0	0	0	0
SAL_SA1844AA	SF_R0310/09	Agona	0	0	0	0	0	0	0	0	0	0	0
SAL_SA1843AA	SF_R0308/09	Agona	0	0	0	0	0	0	0	0	0	0	0
SAL_SA1842AA	SF_R0307/09	Agona	0	0	0	0	0	0	0	0	0	0	0
SAL_SA1841AA	SF_R0298/09	Agona	0	0	0	0	0	0	0	0	0	0	0
SAL_SA1840AA	SF_R0276/09	Agona	0	0	0	0	0	0	0	0	0	0	0
SAL_SA1839AA	SF_R0275/09	Agona	0	0	0	0	0	0	0	0	0	0	0
SAL_SA1838AA	SF_R0267/09	Agona	0	0	0	0	0	0	0	0	0	0	0
SAL_SA1836AA	20042835	Agona	0	0	0	0	0	0	0	0	0	0	0
SAL_SA1835AA	20091569	Agona	0	0	0	0	0	0	0	0	0	0	0
SAL_SA1834AA	20080932	Agona	0	0	0	0	0	0	0	0	0	0	0
SAL_SA1833AA	20080909	Agona	0	0	0	0	0	0	0	0	0	0	0
SAL_SA1832AA	20080905	Agona	0	0	0	0	0	0	0	0	0	0	0
SAL_SA1831AA	20080886	Agona	0	0	0	0	0	0	0	0	0	0	0
SAL_SA1830AA	20080754	Agona	0	0	0	0	0	0	0	0	0	0	0
SAL_SA1829AA	20061425	Agona	0	0	0	0	0	0	0	0	0	0	0
SAL_SA1828AA	20041390	Agona	0	0	0	0	0	0	0	0	0	0	0
SAL_SA2480AA	SMG-18-1275	Agona	0	0	0	0	0	0	0	0	0	0	0
SAL_RA6600AA	317663	Agona	0	0	0	0	0	0	0	0	0	0	0
SAL_RA6611AA	372671	Agona	0	0	0	0	0	0	0	0	0	0	0
SAL_RA6580AA	424701	Agona	0	0	0	0	0	0	0	0	0	0	0
SAL_RA6534AA	178637	Agona	0	0	0	0	0	0	0	0	0	0	0
SAL_RA6552AA	492497	Agona	0	0	0	0	0	0	0	0	0	0	0
SAL_SA2807AA	SMG-18-1384	Agona	0	0	0	0	0	0	0	0	0	0	0
SAL_RA6464AA	424694	Agona	0	0	0	0	0	0	0	0	0	0	0
SAL_RA6466AA	376357	Agona	0	0	0	0	0	0	0	0	0	0	0
SAL_SA2819AA	SMG-18-1398	Agona	0	0	0	0	0	0	0	0	0	0	0
SAL_RA6835AA	PNUSAS043596	Agona	0	0	0	0	0	0	0	0	0	0	0
SAL_RA1983AA	e45-1645-47c8-bb34-03a	Agona	0	0	0	0	0	0	0	0	0	0	0
SAL_RA1986AA	185-6731-4f40-abe7-826	Agona	0	0	0	0	0	0	0	0	0	0	0
SAL_RA1987AA	0b8-5b7f-4013-8016-22d	Agona	0	0	0	0	0	0	0	0	0	0	0
SAL_RA1988AA	e98-28ae-496a-bdd7-14b	Agona	0	0	0	0	0	0	0	0	0	0	0
SAL_RA1984AA	b79-2e89-45d5-a9a1-809	Agona	0	0	0	0	0	0	0	0	0	0	0
SAL_RA1985AA	ebd-755b-4147-8429-467	Agona	0	0	0	0	0	0	0	0	0	0	0
SAL_RA6754AA	PNUSAS044311	Agona	0	0	0	0	0	0	0	0	0	0	0
SAL_RA6177AA	PNUSAS042947	Agona	0	0	0	0	0	0	0	0	0	0	0
SAL_RA6969AA	FSIS11811369	Agona	0	0	0	0	0	0	0	0	0	0	0
SAL_RA6921AA	526795	Agona	0	0	0	0	0	0	0	0	0	0	0
SAL_RA6920AA	533386	Agona	0	0	0	0	0	0	0	0	0	0	0
SAL_RA6925AA	526791	Agona	0	0	0	0	0	0	0	0	0	0	0
SAL_RA6926AA	524803	Agona	0	0	0	0	0	0	0	0	0	0	0
SAL_RA6927AA	524814	Agona	0	0	0	0	0	0	0	0	0	0	0
SAL_RA6928AA	524804	Agona	0	0	0	0	0	0	0	0	0	0	0
SAL_RA6931AA	534803	Agona	0	0	0	0	0	0	0	0	0	0	0
SAL_RA6924AA	526816	Agona	0	0	0	0	0	0	0	0	0	0	0
SAL_RA6906AA	520588	Agona	0	0	0	0	0	0	0	0	0	0	0
SAL_RA6909AA	528560	Agona	0	0	0	0	0	0	0	0	0	0	0
SAL_RA6913AA	516942	Agona	0	0	0	0	0	0	0	0	0	0	0
SAL_RA6912AA	528013	Agona	0	0	0	0	0	0	0	0	0	0	0
SAL_RA6916AA	534015	Agona	0	0	0	0	0	0	0	0	0	0	0
SAL_RA6917AA	538340	Agona	0	0	0	0	0	0	0	0	0	0	0
SAL_RA6915AA	526794	Agona	0	0	0	0	0	0	0	0	0	0	0
SAL_RA6918AA	528556	Agona	0	0	0	0	0	0	0	0	0	0	0
SAL_RA6923AA	524813	Agona	0	0	0	0	0	0	0	0	0	0	0
SAL_RA6929AA	533389	Agona	0	0	0	0	0	0	0	0	0	0	0

SAL_RA6922AA	545252	Agona	0	0	0	0	0	0	0	0	0	0	0
SAL_RA6919AA	549518	Agona	0	0	0	0	0	0	0	0	0	0	0
SAL_RA6930AA	536054	Agona	0	0	0	0	0	0	0	0	0	0	0
SAL_RA6905AA	535353	Agona	0	0	0	0	0	0	0	0	0	0	0
SAL_RA6907AA	524801	Agona	0	0	0	0	0	0	0	0	0	0	0
SAL_RA6904AA	526792	Agona	0	0	0	0	0	0	0	0	0	0	0
SAL_RA6910AA	526796	Agona	0	0	0	0	0	0	0	0	0	0	0
SAL_RA6911AA	526790	Agona	0	0	0	0	0	0	0	0	0	0	0
SAL_RA6895AA	526793	Agona	0	0	0	0	0	0	0	0	0	0	0
SAL_RA6896AA	526797	Agona	0	0	0	0	0	0	0	0	0	0	0
SAL_RA6897AA	199941	Agona	0	0	0	0	0	0	0	0	0	0	0
SAL_RA6899AA	529858	Agona	0	0	0	0	0	0	0	0	0	0	0
SAL_RA6902AA	526789	Agona	0	0	0	0	0	0	0	0	0	0	0
SAL_RA6903AA	543846	Agona	0	0	0	0	0	0	0	0	0	0	0
SAL_RA6900AA	529257	Agona	0	0	0	0	0	0	0	0	0	0	0
SAL_RA6901AA	526815	Agona	0	0	0	0	0	0	0	0	0	0	0
SAL_RA6898AA	541375	Agona	0	0	0	0	0	0	0	0	0	0	0
SAL_RA6894AA	529234	Agona	0	0	0	0	0	0	0	0	0	0	0
SAL_RA6893AA	550101	Agona	0	0	0	0	0	0	0	0	0	0	0
SAL_RA7511AA	492496	Agona	0	0	0	0	0	0	0	0	0	0	0
SAL_RA7475AA	449710	Agona	0	0	0	0	0	0	0	0	0	0	0
SAL_RA7468AA	298831	Agona	0	0	0	0	0	0	0	0	0	0	0
SAL_RA7450AA	457299	Agona	0	0	0	0	0	0	0	0	0	0	0
SAL_RA7375AA	152447	Agona	0	0	0	0	0	0	0	0	0	0	0
SAL_RA7367AA	186541	Agona	0	0	0	0	0	0	0	0	0	0	0
SAL_RA7320AA	147833	Agona	0	0	0	0	0	0	0	0	0	0	0
SAL_RA7326AA	568282	Agona	0	0	0	0	0	0	0	0	0	0	0
SAL_RA7288AA	172441	Agona	0	0	0	0	0	0	0	0	0	0	0
SAL_RA7336AA	524812	Agona	0	0	0	0	0	0	0	0	0	0	0
SAL_RA7279AA	376356	Agona	0	0	0	0	0	0	0	0	0	0	0
SAL_RA7280AA	308757	Agona	0	0	0	0	0	0	0	0	0	0	0
SAL_RA7261AA	475836	Agona	0	0	0	0	0	0	0	0	0	0	0
SAL_RA7602AA	PNUSAS045384	Agona	0	0	0	0	0	0	0	0	0	0	0
SAL_RA7208AA	552420	Agona	0	0	0	0	0	0	0	0	0	0	0
SAL_RA7158AA	323019	Agona	0	0	0	0	0	0	0	0	0	0	0
SAL_RA7117AA	243826	Agona	0	0	0	0	0	0	0	0	0	0	0
SAL_RA7081AA	302550	Agona	0	0	0	0	0	0	0	0	0	0	0
SAL_RA7009AA	308530	Agona	0	0	0	0	0	0	0	0	0	0	0
SAL_RA7885AA	495801	Agona	0	0	0	0	0	0	0	0	0	0	0
SAL_RA7855AA	311438	Agona	0	0	0	0	0	0	0	0	0	0	0
SAL_RA7839AA	445970	Agona	0	0	0	0	0	0	0	0	0	0	0
SAL_RA7827AA	417400	Agona	0	0	0	0	0	0	0	0	0	0	0
SAL_RA7806AA	231110	Agona	0	0	0	0	0	0	0	0	0	0	0
SAL_RA7815AA	555442	Agona	0	0	0	0	0	0	0	0	0	0	0
SAL_RA7768AA	FSIS11811623	Agona	0	0	0	0	0	0	0	0	0	0	0
SAL_RA7704AA	423879	Agona	0	0	0	0	0	0	0	0	0	0	0
SAL_RA7695AA	123991	Agona	0	0	0	0	0	0	0	0	0	0	0
SAL_RA7682AA	488730	Agona	0	0	0	0	0	0	0	0	0	0	0
SAL_RA7696AA	359285	Agona	0	0	0	0	0	0	0	0	0	0	0
SAL_RA7675AA	117306	Agona	0	0	0	0	0	0	0	0	0	0	0
SAL_RA7649AA	FSIS11811947	Agona	0	0	0	0	0	0	0	0	0	0	0
SAL_RA8032AA	190776	Agona	0	0	0	0	0	0	0	0	0	0	0
SAL_RA8037AA	266970	Agona	0	0	0	0	0	0	0	0	0	0	0
SAL_RA8008AA	479463	Agona	0	0	0	0	0	0	0	0	0	0	0
SAL_RA7949AA	435485	Agona	0	0	0	0	0	0	0	0	0	0	0
SAL_RA7946AA	570837	Agona	0	0	0	0	0	0	0	0	0	0	0
SAL_RA8359AA	416221	Agona	0	0	0	0	0	0	0	0	0	0	0
SAL_RA8309AA	316654	Agona	0	0	0	0	0	0	0	0	0	0	0
SAL_RA8289AA	178636	Agona	0	0	0	0	0	0	0	0	0	0	0
SAL_RA8273AA	166317	Agona	0	0	0	0	0	0	0	0	0	0	0
SAL_RA8241AA	296684	Agona	0	0	0	0	0	0	0	0	0	0	0
SAL_RA8178AA	SAL-18-VL-LA-KS-0001	Agona	0	0	0	0	0	0	0	0	0	0	0
SAL_RA8564AA	294766	Agona	0	0	0	0	0	0	0	0	0	0	0
SAL_RA8741AA	FSIS11812306	Agona	0	0	0	0	0	0	0	0	0	0	0
SAL_RA5438AA	417398	Agona	0	0	0	0	0	0	0	0	0	0	0
SAL_RA9344AA	CFSAN084088	Agona	0	0	0	0	0	0	0	0	0	0	0
SAL_RA9350AA	CFSAN084148	Agona	0	0	0	0	0	0	0	0	0	0	0
SAL_RA9293AA	CFSAN084082	Agona	0	0	0	0	0	0	0	0	0	0	0
SAL_RA9296AA	CFSAN083996	Agona	0	0	0	0	0	0	0	0	0	0	0
SAL_RA9266AA	CFSAN084081	Agona	0	0	0	0	0	0	0	0	0	0	0
SAL_RA9222AA	CFSAN084083	Agona	0	0	0	0	0	0	0	0	0	0	0
SAL_RA9409AA	CFSAN084026	Agona	0	0	0	0	0	0	0	0	0	0	0
SAL_RA9388AA	CFSAN084047	Agona	0	0	0	0	0	0	0	0	0	0	0

SAL_RA9384AA	CFSAN084054	Agona	0	0	0	0	0	0	0	0	0	0	0
SAL_RA9386AA	CFSAN084048	Agona	0	0	0	0	0	0	0	0	0	0	0
SAL_RA9382AA	CFSAN084189	Agona	0	0	0	0	0	0	0	0	0	0	0
SAL_RA9369AA	CFSAN084038	Agona	0	0	0	0	0	0	0	0	0	0	0
SAL_RA9373AA	CFSAN084183	Agona	0	0	0	0	0	0	0	0	0	0	0
SAL_RA9356AA	CFSAN084087	Agona	0	0	0	0	0	0	0	0	0	0	0
SAL_RA9370AA	CFSAN084184	Agona	0	0	0	0	0	0	0	0	0	0	0
SAL_RA9785AA	CFSAN084073	Agona	0	0	0	0	0	0	0	0	0	0	0
SAL_RA9765AA	CFSAN084072	Agona	0	0	0	0	0	0	0	0	0	0	0
SAL_RA9773AA	CFSAN084074	Agona	0	0	0	0	0	0	0	0	0	0	0
SAL_RA9892AA	CFSAN084236	Agona	0	0	0	0	0	0	0	0	0	0	0
SAL_RA9882AA	CFSAN084237	Agona	0	0	0	0	0	0	0	0	0	0	0
SAL_RA9883AA	CFSAN084223	Agona	0	0	0	0	0	0	0	0	0	0	0
SAL_RA9872AA	CFSAN084222	Agona	0	0	0	0	0	0	0	0	0	0	0
SAL_SA0217AA	CVM N17S877	Agona	0	0	0	0	0	0	0	0	0	0	0
SAL_SA3458AA	SMG-18-1421	Agona	0	0	0	0	0	0	0	0	0	0	0
SAL_SA0178AA	CVM N17S722	Agona	0	0	0	0	0	0	0	0	0	0	0
SAL_SA0128AA	CVM N17S1483	Agona	0	0	0	0	0	0	0	0	0	0	0
SAL_SA0117AA	CVM N17S283	Agona	0	0	0	0	0	0	0	0	0	0	0
SAL_SA0118AA	CVM N17S1428	Agona	0	0	0	0	0	0	0	0	0	0	0
SAL_SA0093AA	CVM N17S1446	Agona	0	0	0	0	0	0	0	0	0	0	0
SAL_SA0026AA	FDA00013264	Agona	0	0	0	0	0	0	0	0	0	0	0
SAL_SA0424AA		Agona	0	0	0	0	0	0	0	0	0	0	0
SAL_SA0414AA		Agona	0	0	0	0	0	0	0	0	0	0	0
SAL_SA0402AA		Agona	0	0	0	0	0	0	0	0	0	0	0
SAL_SA0393AA		Agona	0	0	0	0	0	0	0	0	0	0	0
SAL_SA0382AA		Agona	0	0	0	0	0	0	0	0	0	0	0
SAL_SA0367AA		Agona	0	0	0	0	0	0	0	0	0	0	0
SAL_SA0368AA		Agona	0	0	0	0	0	0	0	0	0	0	0
SAL_SA0369AA		Agona	0	0	0	0	0	0	0	0	0	0	0
SAL_SA0370AA		Agona	0	0	0	0	0	0	0	0	0	0	0
SAL_SA0371AA		Agona	0	0	0	0	0	0	0	0	0	0	0
SAL_SA0372AA		Agona	0	0	0	0	0	0	0	0	0	0	0
SAL_SA0374AA		Agona	0	0	0	0	0	0	0	0	0	0	0
SAL_SA0361AA		Agona	0	0	0	0	0	0	0	0	0	0	0
SAL_SA0360AA		Agona	0	0	0	0	0	0	0	0	0	0	0
SAL_SA0365AA		Agona	0	0	0	0	0	0	0	0	0	0	0
SAL_SA0362AA		Agona	0	0	0	0	0	0	0	0	0	0	0
SAL_SA0363AA		Agona	0	0	0	0	0	0	0	0	0	0	0
SAL_RA9967AA	FSIS11812446	Agona	0	0	0	0	0	0	0	0	0	0	0
SAL_SA0358AA		Agona	0	0	0	0	0	0	0	0	0	0	0
SAL_RA6908AA	524802	Agona	0	0	0	0	0	0	0	0	0	0	0
SAL_RA6914AA	480713	Agona	0	0	0	0	0	0	0	0	0	0	0
SAL_RA7458AA	568479	Agona	0	0	0	0	0	0	0	0	0	0	0
SAL_SA0590AA	FSIS11812421	Agona	0	0	0	0	0	0	0	0	0	0	0
SAL_RA7439AA	475815	Agona	0	0	0	0	0	0	0	0	0	0	0
SAL_RA7830AA	178639	Agona	0	0	0	0	0	0	0	0	0	0	0
SAL_RA8075AA	568454	Agona	0	0	0	0	0	0	0	0	0	0	0
SAL_SA0866AA	FSIS11812701	Agona	0	0	0	0	0	0	0	0	0	0	0
SAL_SA0853AA	FSIS11812711	Agona	0	0	0	0	0	0	0	0	0	0	0
SAL_SA0858AA	FSIS11812743	Agona	0	0	0	0	0	0	0	0	0	0	0
SAL_SA0995AA	FSIS31800793	Agona	0	0	0	0	0	0	0	0	0	0	0
SAL_RA9380AA	CFSAN084055	Agona	0	0	0	0	0	0	0	0	0	0	0
SAL_RA9378AA	CFSAN084182	Agona	0	0	0	0	0	0	0	0	0	0	0
SAL_SA2409AA	TTU_232	Agona	0	0	0	0	0	0	0	0	0	0	0
SAL_SA3924AA	SMG-18-1698	Agona	0	0	0	0	0	0	0	0	0	0	0
SAL_SA2864AA	FSIS11813215	Agona	0	0	0	0	0	0	0	0	0	0	0
SAL_SA2859AA	FSIS11813214	Agona	0	0	0	0	0	0	0	0	0	0	0
SAL_RA9218AA	FSIS11812289	Agona	0	0	0	0	0	0	0	0	0	0	0
SAL_SA3193AA		Agona	0	0	0	0	0	0	0	0	0	0	0
SAL_SA5613AA	20060793	Agona	0	0	0	0	0	0	0	0	0	0	0
SAL_SA3189AA		Agona	0	0	0	0	0	0	0	0	0	0	0
SAL_SA1116AA	FDA00004914	Agona	0	0	0	0	0	0	0	0	0	0	0
SAL_SA1115AA	FDA00004913	Agona	0	0	0	0	0	0	0	0	0	0	0
SAL_SA3321AA	OH-18-18132-13	Agona	0	0	0	0	0	0	0	0	0	0	0
SAL_SA3108AA		Agona	0	0	0	0	0	0	0	0	0	0	0
SAL_SA3107AA		Agona	0	0	0	0	0	0	0	0	0	0	0
SAL_SA3080AA		Agona	0	0	0	0	0	0	0	0	0	0	0
SAL_SA3082AA		Agona	0	0	0	0	0	0	0	0	0	0	0
SAL_SA3084AA		Agona	0	0	0	0	0	0	0	0	0	0	0
SAL_SA3091AA		Agona	0	0	0	0	0	0	0	0	0	0	0
SAL_SA3092AA		Agona	0	0	0	0	0	0	0	0	0	0	0
SAL_SA3086AA		Agona	0	0	0	0	0	0	0	0	0	0	0

SAL_SA3085AA		Agona	0	0	0	0	0	0	0	0	0	0	0
SAL_SA3088AA		Agona	0	0	0	0	0	0	0	0	0	0	0
SAL_SA3094AA		Agona	0	0	0	0	0	0	0	0	0	0	0
SAL_SA3087AA		Agona	0	0	0	0	0	0	0	0	0	0	0
SAL_SA3093AA		Agona	0	0	0	0	0	0	0	0	0	0	0
SAL_SA3096AA		Agona	0	0	0	0	0	0	0	0	0	0	0
SAL_SA3098AA		Agona	0	0	0	0	0	0	0	0	0	0	0
SAL_SA3100AA		Agona	0	0	0	0	0	0	0	0	0	0	0
SAL_SA3099AA		Agona	0	0	0	0	0	0	0	0	0	0	0
SAL_SA3101AA		Agona	0	0	0	0	0	0	0	0	0	0	0
SAL_SA3064AA		Agona	0	0	0	0	0	0	0	0	0	0	0
SAL_SA3061AA		Agona	0	0	0	0	0	0	0	0	0	0	0
SAL_SA3057AA		Agona	0	0	0	0	0	0	0	0	0	0	0
SAL_SA3062AA		Agona	0	0	0	0	0	0	0	0	0	0	0
SAL_SA3070AA		Agona	0	0	0	0	0	0	0	0	0	0	0
SAL_SA3071AA		Agona	0	0	0	0	0	0	0	0	0	0	0
SAL_SA3075AA		Agona	0	0	0	0	0	0	0	0	0	0	0
SAL_SA3073AA		Agona	0	0	0	0	0	0	0	0	0	0	0
SAL_SA3076AA		Agona	0	0	0	0	0	0	0	0	0	0	0
SAL_SA3077AA		Agona	0	0	0	0	0	0	0	0	0	0	0
SAL_SA3078AA		Agona	0	0	0	0	0	0	0	0	0	0	0
SAL_SA3050AA		Agona	0	0	0	0	0	0	0	0	0	0	0
SAL_SA3049AA		Agona	0	0	0	0	0	0	0	0	0	0	0
SAL_SA3052AA		Agona	0	0	0	0	0	0	0	0	0	0	0
SAL_SA3065AA		Agona	0	0	0	0	0	0	0	0	0	0	0
SAL_SA3066AA		Agona	0	0	0	0	0	0	0	0	0	0	0
SAL_SA3051AA		Agona	0	0	0	0	0	0	0	0	0	0	0
SAL_SA3047AA		Agona	0	0	0	0	0	0	0	0	0	0	0
SAL_SA3069AA		Agona	0	0	0	0	0	0	0	0	0	0	0
SAL_SA3068AA		Agona	0	0	0	0	0	0	0	0	0	0	0
SAL_SA3048AA		Agona	0	0	0	0	0	0	0	0	0	0	0
SAL_SA3053AA		Agona	0	0	0	0	0	0	0	0	0	0	0
SAL_SA3055AA		Agona	0	0	0	0	0	0	0	0	0	0	0
SAL_SA3056AA		Agona	0	0	0	0	0	0	0	0	0	0	0
SAL_SA3043AA		Agona	0	0	0	0	0	0	0	0	0	0	0
SAL_SA3083AA		Agona	0	0	0	0	0	0	0	0	0	0	0
SAL_SA3089AA		Agona	0	0	0	0	0	0	0	0	0	0	0
SAL_SA3045AA		Agona	0	0	0	0	0	0	0	0	0	0	0
SAL_SA3046AA		Agona	0	0	0	0	0	0	0	0	0	0	0
SAL_SA3058AA		Agona	0	0	0	0	0	0	0	0	0	0	0
SAL_SA3624AA	FSIS31800958	Agona	0	0	0	0	0	0	0	0	0	0	0
SAL_SA3604AA	FSIS31800950	Agona	0	0	0	0	0	0	0	0	0	0	0
SAL_SA3755AA	FSIS11813433	Agona	0	0	0	0	0	0	0	0	0	0	0
SAL_SA3684AA	FSIS11813704	Agona	0	0	0	0	0	0	0	0	0	0	0
SAL_SA5159AA	DB_41	Agona	0	0	0	0	0	0	0	0	0	0	0
SAL_SA5160AA	DB_42	Agona	0	0	0	0	0	0	0	0	0	0	0
SAL_SA5161AA	DB_43	Agona	0	0	0	0	0	0	0	0	0	0	0
SAL_SA5158AA	DB_40	Agona	0	0	0	0	0	0	0	0	0	0	0
SAL_SA5157AA	DB_38	Agona	0	0	0	0	0	0	0	0	0	0	0
SAL_SA5156AA	DB_37	Agona	0	0	0	0	0	0	0	0	0	0	0
SAL_SA5155AA	DB_36	Agona	0	0	0	0	0	0	0	0	0	0	0
SAL_SA5154AA	DB_34	Agona	0	0	0	0	0	0	0	0	0	0	0
SAL_SA5153AA	DB_33	Agona	0	0	0	0	0	0	0	0	0	0	0
SAL_SA5152AA	DB_32	Agona	0	0	0	0	0	0	0	0	0	0	0
SAL_SA5151AA	DB_31	Agona	0	0	0	0	0	0	0	0	0	0	0
SAL_SA5149AA	DB_27	Agona	0	0	0	0	0	0	0	0	0	0	0
SAL_SA5144AA	DB_12	Agona	0	0	0	0	0	0	0	0	0	0	0
SAL_SA5148AA	DB_26	Agona	0	0	0	0	0	0	0	0	0	0	0
SAL_SA5147AA	DB_25	Agona	0	0	0	0	0	0	0	0	0	0	0
SAL_SA5145AA	DB_13	Agona	0	0	0	0	0	0	0	0	0	0	0
SAL_SA5146AA	DB_14	Agona	0	0	0	0	0	0	0	0	0	0	0
SAL_SA5143AA	DB_11	Agona	0	0	0	0	0	0	0	0	0	0	0
SAL_SA3067AA		Agona	0	0	0	0	0	0	0	0	0	0	0
SAL_SA3060AA		Agona	0	0	0	0	0	0	0	0	0	0	0
SAL_SA3054AA		Agona	0	0	0	0	0	0	0	0	0	0	0
SAL_SA4358AA	FSIS31800997	Agona	0	0	0	0	0	0	0	0	0	0	0
SAL_SA4049AA	FSIS21822074	Agona	0	0	0	0	0	0	0	0	0	0	0
SAL_SA4392AA	FDA00013427	Agona	0	0	0	0	0	0	0	0	0	0	0
SAL_SA4901AA	585215	Agona	0	0	0	0	0	0	0	0	0	0	0
SAL_SA4845AA	575115	Agona	0	0	0	0	0	0	0	0	0	0	0
SAL_SA4758AA	575640	Agona	0	0	0	0	0	0	0	0	0	0	0
SAL_SA4777AA	598059	Agona	0	0	0	0	0	0	0	0	0	0	0
SAL_SA4688AA	587760	Agona	0	0	0	0	0	0	0	0	0	0	0

SAL_SA6017AA	595564	Agona	0	0	0	0	0	0	0	0	0	0	0
SAL_SA5996AA	590500	Agona	0	0	0	0	0	0	0	0	0	0	0
SAL_SA6007AA	573630	Agona	0	0	0	0	0	0	0	0	0	0	0
SAL_SA5908AA	378623	Agona	0	0	0	0	0	0	0	0	0	0	0
SAL_SA5890AA	572540	Agona	0	0	0	0	0	0	0	0	0	0	0
SAL_SA5829AA	577902	Agona	0	0	0	0	0	0	0	0	0	0	0
SAL_SA6426AA	600749	Agona	0	0	0	0	0	0	0	0	0	0	0
SAL_SA6359AA	602635	Agona	0	0	0	0	0	0	0	0	0	0	0
SAL_SA6383AA	542153	Agona	0	0	0	0	0	0	0	0	0	0	0
SAL_SA6508AA	OH-18-18132-7	Agona	0	0	0	0	0	0	0	0	0	0	0
SAL_SA6229AA	579075	Agona	0	0	0	0	0	0	0	0	0	0	0
SAL_SA6239AA	576325	Agona	0	0	0	0	0	0	0	0	0	0	0
SAL_SA6211AA	598115	Agona	0	0	0	0	0	0	0	0	0	0	0
SAL_SA6220AA	587374	Agona	0	0	0	0	0	0	0	0	0	0	0
SAL_SA6082AA	598020	Agona	0	0	0	0	0	0	0	0	0	0	0
SAL_SA6119AA	598067	Agona	0	0	0	0	0	0	0	0	0	0	0
SAL_SA6076AA	599540	Agona	0	0	0	0	0	0	0	0	0	0	0
SAL_SA6792AA	369911	Agona	0	0	0	0	0	0	0	0	0	0	0
SAL_SA6728AA	298902	Agona	0	0	0	0	0	0	0	0	0	0	0
SAL_SA6740AA	591948	Agona	0	0	0	0	0	0	0	0	0	0	0
SAL_SA6732AA	601697	Agona	0	0	0	0	0	0	0	0	0	0	0
SAL_SA6560AA	596270	Agona	0	0	0	0	0	0	0	0	0	0	0
SAL_SA6559AA	579088	Agona	0	0	0	0	0	0	0	0	0	0	0
SAL_SA7614AA	FSIS11814082	Agona	0	0	0	0	0	0	0	0	0	0	0
SAL_SA7290AA	FSIS21822143	Agona	0	0	0	0	0	0	0	0	0	0	0
SAL_SA7571AA	588906	Agona	0	0	0	0	0	0	0	0	0	0	0
SAL_SA7087AA	578039	Agona	0	0	0	0	0	0	0	0	0	0	0
SAL_SA7815AA	106519	Agona	0	0	0	0	0	0	0	0	0	0	0
SAL_SA7559AA	178634	Agona	0	0	0	0	0	0	0	0	0	0	0
SAL_SA6055AA	603287	Agona	0	0	0	0	0	0	0	0	0	0	0
SAL_SA6065AA	590879	Agona	0	0	0	0	0	0	0	0	0	0	0
SAL_SA7408AA	601604	Agona	0	0	0	0	0	0	0	0	0	0	0
SAL_SA7737AA	446000	Agona	0	0	0	0	0	0	0	0	0	0	0
SAL_SA7688AA	408544	Agona	0	0	0	0	0	0	0	0	0	0	0
SAL_SA7693AA	392942	Agona	0	0	0	0	0	0	0	0	0	0	0
SAL_SA7698AA	159673	Agona	0	0	0	0	0	0	0	0	0	0	0
SAL_SA7706AA	529205	Agona	0	0	0	0	0	0	0	0	0	0	0
SAL_SA7669AA	454894	Agona	0	0	0	0	0	0	0	0	0	0	0
SAL_SA7638AA	605811	Agona	0	0	0	0	0	0	0	0	0	0	0
SAL_SA7589AA	423875	Agona	0	0	0	0	0	0	0	0	0	0	0
SAL_SA7963AA	325710	Agona	0	0	0	0	0	0	0	0	0	0	0
SAL_SA7983AA	299807	Agona	0	0	0	0	0	0	0	0	0	0	0
SAL_SA7921AA	438005	Agona	0	0	0	0	0	0	0	0	0	0	0
SAL_SA7951AA	593511	Agona	0	0	0	0	0	0	0	0	0	0	0
SAL_SA7881AA	292971	Agona	0	0	0	0	0	0	0	0	0	0	0
SAL_SA7838AA	397659	Agona	0	0	0	0	0	0	0	0	0	0	0
SAL_SA8728AA	143989	Agona	0	0	0	0	0	0	0	0	0	0	0
SAL_SA8756AA	458077	Agona	0	0	0	0	0	0	0	0	0	0	0
SAL_SA8671AA	178633	Agona	0	0	0	0	0	0	0	0	0	0	0
SAL_SA8676AA	538374	Agona	0	0	0	0	0	0	0	0	0	0	0
SAL_SA8760AA	FSIS11814210	Agona	0	0	0	0	0	0	0	0	0	0	0
SAL_SA8956AA	413008	Agona	0	0	0	0	0	0	0	0	0	0	0
SAL_SA8929AA	273529	Agona	0	0	0	0	0	0	0	0	0	0	0
SAL_SA8876AA	416387	Agona	0	0	0	0	0	0	0	0	0	0	0
SAL_SA8902AA	576316	Agona	0	0	0	0	0	0	0	0	0	0	0
SAL_SA8842AA	FSIS31801082	Agona	0	0	0	0	0	0	0	0	0	0	0
SAL_SA9285AA	CVM N17S1281	Agona	0	0	0	0	0	0	0	0	0	0	0
SAL_SA9252AA	CVM N17S309	Agona	0	0	0	0	0	0	0	0	0	0	0
SAL_SA9211AA	CVM N17S1565	Agona	0	0	0	0	0	0	0	0	0	0	0
SAL_SA9121AA	CVM N17S587	Agona	0	0	0	0	0	0	0	0	0	0	0
SAL_SA9528AA	440167	Agona	0	0	0	0	0	0	0	0	0	0	0
SAL_SA9414AA	264173	Agona	0	0	0	0	0	0	0	0	0	0	0
SAL_SA9420AA	410894	Agona	0	0	0	0	0	0	0	0	0	0	0
SAL_SA9400AA	238468	Agona	0	0	0	0	0	0	0	0	0	0	0
SAL_SA9650AA	FSIS11814282	Agona	0	0	0	0	0	0	0	0	0	0	0
SAL_SA8918AA	109907	Agona	0	0	0	0	0	0	0	0	0	0	0
SAL_SA8943AA	412023	Agona	0	0	0	0	0	0	0	0	0	0	0
SAL_SA9667AA	TXACF1801677	Agona	0	0	0	0	0	0	0	0	0	0	0
SAL_TA0027AA	FSIS21822239	Agona	0	0	0	0	0	0	0	0	0	0	0
SAL_TA0023AA	FSIS11813728	Agona	0	0	0	0	0	0	0	0	0	0	0
SAL_TA0096AA	FSIS11814460	Agona	0	0	0	0	0	0	0	0	0	0	0
SAL_TA0242AA	49-19-18.8100956.P	Agona	0	0	0	0	0	0	0	0	0	0	0
SAL_TA0476AA	612988	Agona	0	0	0	0	0	0	0	0	0	0	0

SAL_TA0300AA	613784	Agona	0	0	0	0	0	0	0	0	0	0	0
SAL_TA0360AA	611064	Agona	0	0	0	0	0	0	0	0	0	0	0
SAL_TA0568AA	18029	Agona	0	0	0	411	3	0	4	4	5	5	1
SAL_TA0874AA	616188	Agona	0	0	0	0	0	0	0	0	0	0	0
SAL_TA0876AA	510168	Agona	0	0	0	0	0	0	0	0	0	0	0
SAL_TA0821AA	615532	Agona	0	0	0	0	0	0	0	0	0	0	0
SAL_TA0811AA	613816	Agona	0	0	0	0	0	0	0	0	0	0	0
SAL_TA0830AA	158956	Agona	0	0	0	0	0	0	0	0	0	0	0
SAL_TA0802AA	608453	Agona	0	0	0	0	0	0	0	0	0	0	0
SAL_TA1471AA	FSIS11814666	Agona	0	0	0	0	0	0	0	0	0	0	0
SAL_TA1479AA	FSIS11814659	Agona	0	0	0	0	0	0	0	0	0	0	0
SAL_TA1527AA	53-31-18-8101094-Z	Agona	0	0	0	0	0	0	0	0	0	0	0
SAL_TA1533AA	53-5-18-8101061-H	Agona	0	0	0	0	0	0	0	0	0	0	0
SAL_TA1849AA	80608.14	Agona	0	0	0	0	0	0	0	0	0	0	0
SAL_TA1843AA	51408.1	Agona	0	0	0	0	0	0	0	0	0	0	0
SAL_TA1846AA	61208.04	Agona	0	0	0	0	0	0	0	0	0	0	0
SAL_TA1933AA	DV01	Agona	0	0	0	0	0	0	0	0	0	0	0
SAL_TA1937AA	DV06	Agona	0	0	0	0	0	0	0	0	0	0	0
SAL_TA2030AA	CFSAN043221	Agona	0	0	0	0	0	0	0	0	0	0	0
SAL_TA2397AA	624403	Agona	0	0	0	0	0	0	0	0	0	0	0
SAL_TA2365AA	620454	Agona	0	0	0	0	0	0	0	0	0	0	0
SAL_TA2304AA	620437	Agona	0	0	0	0	0	0	0	0	0	0	0
SAL_TA2309AA	621473	Agona	0	0	0	0	0	0	0	0	0	0	0
SAL_TA2251AA	620430	Agona	0	0	0	0	0	0	0	0	0	0	0
SAL_TA2726AA	619882	Agona	0	0	0	0	0	0	0	0	0	0	0
SAL_TA3642AA	201808808	Agona	0	0	0	0	0	0	0	0	0	0	0
SAL_TA2681AA	FSIS11814821	Agona	0	0	0	0	0	0	0	0	0	0	0
SAL_TA3859AA	200403162	Agona	0	0	0	0	0	0	0	0	0	0	0
SAL_TA3863AA	200409457	Agona	0	0	0	0	0	0	0	0	0	0	0
SAL_TA3861AA	200309357	Agona	0	0	0	0	0	0	0	0	0	0	0
SAL_TA3860AA	201711159	Agona	0	0	0	0	0	0	0	0	0	0	0
SAL_TA3864AA	200404722	Agona	0	0	0	0	0	0	0	0	0	0	0
SAL_TA3865AA	200500509	Agona	0	0	0	0	0	0	0	0	0	0	0
SAL_TA3862AA	201711272	Agona	0	0	0	0	0	0	0	0	0	0	0
SAL_TA3866AA	200500829	Agona	0	0	0	0	0	0	0	0	0	0	0
SAL_TA3869AA	200500834	Agona	0	0	0	0	0	0	0	0	0	0	0
SAL_TA3867AA	201009061	Agona	0	0	0	0	0	0	0	0	0	0	0
SAL_TA3870AA	201009108	Agona	0	0	0	0	0	0	0	0	0	0	0
SAL_TA3872AA	201008634	Agona	0	0	0	0	0	0	0	0	0	0	0
SAL_TA3874AA	201101441	Agona	0	0	0	0	0	0	0	0	0	0	0
SAL_TA3873AA	201100741	Agona	0	0	0	0	0	0	0	0	0	0	0
SAL_TA3871AA	201100455	Agona	0	0	0	0	0	0	0	0	0	0	0
SAL_TA3875AA	201204277	Agona	0	0	0	0	0	0	0	0	0	0	0
SAL_TA3876AA	201100900	Agona	0	0	0	0	0	0	0	0	0	0	0
SAL_TA3877AA	201100462	Agona	0	0	0	0	0	0	0	0	0	0	0
SAL_TA3879AA	201502110	Agona	0	0	0	0	0	0	0	0	0	0	0
SAL_TA3878AA	201206582	Agona	0	0	0	0	0	0	0	0	0	0	0
SAL_NA3480AA	201303570	Agona	0	0	0	0	0	0	0	0	0	0	0
SAL_TA3880AA	201504506	Agona	0	0	0	0	0	0	0	0	0	0	0
SAL_TA3883AA	201511618	Agona	0	0	0	0	0	0	0	0	0	0	0
SAL_TA3884AA	201512023	Agona	0	0	0	0	0	0	0	0	0	0	0
SAL_TA3887AA	201611331	Agona	0	0	0	0	0	0	0	0	0	0	0
SAL_TA3881AA	201504737	Agona	0	0	0	0	0	0	0	0	0	0	0
SAL_TA3882AA	201510055	Agona	0	0	0	0	0	0	0	0	0	0	0
SAL_TA3886AA	201512248	Agona	0	0	0	0	0	0	0	0	0	0	0
SAL_TA3885AA	201602111	Agona	0	0	0	0	0	0	0	0	0	0	0
SAL_TA3888AA	201603042	Agona	0	0	0	0	0	0	0	0	0	0	0
SAL_TA3892AA	201703400	Agona	0	0	0	0	0	0	0	0	0	0	0
SAL_TA3893AA	201603659	Agona	0	0	0	0	0	0	0	0	0	0	0
SAL_TA3891AA	201703401	Agona	0	0	0	0	0	0	0	0	0	0	0
SAL_TA3889AA	201701978	Agona	0	0	0	0	0	0	0	0	0	0	0
SAL_TA3890AA	201702247	Agona	0	0	0	0	0	0	0	0	0	0	0
SAL_TA3894AA	201704902	Agona	0	0	0	0	0	0	0	0	0	0	0
SAL_TA3895AA	201703582	Agona	0	0	0	0	0	0	0	0	0	0	0
SAL_TA3898AA	201710954	Agona	0	0	0	0	0	0	0	0	0	0	0
SAL_TA3896AA	201707565	Agona	0	0	0	0	0	0	0	0	0	0	0
SAL_TA3899AA	201711805	Agona	0	0	0	0	0	0	0	0	0	0	0
SAL_TA3897AA	201707710	Agona	0	0	0	0	0	0	0	0	0	0	0
SAL_TA3901AA	201711906	Agona	0	0	0	0	0	0	0	0	0	0	0
SAL_TA3900AA	201711904	Agona	0	0	0	0	0	0	0	0	0	0	0
SAL_TA3902AA	201711889	Agona	0	0	0	0	0	0	0	0	0	0	0
SAL_TA3903AA	201711799	Agona	0	0	0	0	0	0	0	0	0	0	0
SAL_TA3905AA	201700287	Agona	0	0	0	0	0	0	0	0	0	0	0

SAL_TA3904AA	201800464	Agona	0	0	0	0	0	0	0	0	0	0	0
SAL_TA3906AA	201711915	Agona	0	0	0	0	0	0	0	0	0	0	0
SAL_TA3908AA	201702004	Agona	0	0	0	0	0	0	0	0	0	0	0
SAL_TA3909AA	201610634	Agona	0	0	0	0	0	0	0	0	0	0	0
SAL_TA3910AA	201701611	Agona	0	0	0	0	0	0	0	0	0	0	0
SAL_TA3911AA	201701788	Agona	0	0	0	0	0	0	0	0	0	0	0
SAL_TA3912AA	201702152	Agona	0	0	0	0	0	0	0	0	0	0	0
SAL_TA3913AA	201702244	Agona	0	0	0	0	0	0	0	0	0	0	0
SAL_TA3914AA	201702831	Agona	0	0	0	0	0	0	0	0	0	0	0
SAL_TA3915AA	201703684	Agona	0	0	0	0	0	0	0	0	0	0	0
SAL_TA3916AA	201703631	Agona	0	0	0	0	0	0	0	0	0	0	0
SAL_TA3917AA	201702931	Agona	0	0	0	0	0	0	0	0	0	0	0
SAL_TA3922AA	201707179	Agona	0	0	0	0	0	0	0	0	0	0	0
SAL_TA3919AA	201704147	Agona	0	0	0	0	0	0	0	0	0	0	0
SAL_TA3918AA	201703836	Agona	0	0	0	0	0	0	0	0	0	0	0
SAL_TA3920AA	201703962	Agona	0	0	0	0	0	0	0	0	0	0	0
SAL_TA3923AA	201707823	Agona	0	0	0	0	0	0	0	0	0	0	0
SAL_TA3924AA	201708353	Agona	0	0	0	0	0	0	0	0	0	0	0
SAL_TA3921AA	201705442	Agona	0	0	0	0	0	0	0	0	0	0	0
SAL_TA3926AA	201709230	Agona	0	0	0	0	0	0	0	0	0	0	0
SAL_TA3928AA	201710895	Agona	0	0	0	0	0	0	0	0	0	0	0
SAL_TA3927AA	201709452	Agona	0	0	0	0	0	0	0	0	0	0	0
SAL_TA3925AA	201708540	Agona	0	0	0	0	0	0	0	0	0	0	0
SAL_TA3929AA	201708736	Agona	0	0	0	0	0	0	0	0	0	0	0
SAL_NA1229AA	201708953	Agona	0	0	0	0	0	0	0	0	0	0	0
SAL_TA3930AA	201710779	Agona	0	0	0	0	0	0	0	0	0	0	0
SAL_TA3931AA	201711113	Agona	0	0	0	0	0	0	0	0	0	0	0
SAL_TA4200AA	56-4-18-8101152-B	Agona	0	0	0	0	0	0	0	0	0	0	0
SAL_TA4618AA	201809107	Agona	0	0	0	0	0	0	0	0	0	0	0
SAL_TA4619AA	201809108	Agona	0	0	0	0	0	0	0	0	0	0	0
SAL_TA5335AA	620452	Agona	0	0	0	0	0	0	0	0	0	0	0
SAL_TA5258AA	627318	Agona	0	0	0	0	0	0	0	0	0	0	0
SAL_TA5198AA	621504	Agona	0	0	0	0	0	0	0	0	0	0	0
SAL_TA5993AA	621514	Agona	0	0	0	0	0	0	0	0	0	0	0
SAL_TA6140AA	OH-18-20225-2-S	Agona	0	0	0	0	0	0	0	0	0	0	0
SAL_TA6214AA	629335	Agona	0	0	0	0	0	0	0	0	0	0	0
SAL_TA6217AA	629323	Agona	0	0	0	0	0	0	0	0	0	0	0
SAL_TA6381AA	67	Agona	0	0	0	0	0	0	0	0	0	0	0
SAL_TA6383AA	69	Agona	0	0	0	0	0	0	0	0	0	0	0
SAL_TA6382AA	68	Agona	0	0	0	0	0	0	0	0	0	0	0
SAL_TA6338AA	24	Agona	0	0	0	0	0	0	0	0	0	0	0
SAL_TA6353AA	36	Agona	0	0	0	0	0	0	0	0	0	0	0
SAL_TA6377AA	64	Agona	0	0	0	0	0	0	0	0	0	0	0
SAL_TA6660AA	628833	Agona	0	0	0	0	0	0	0	0	0	0	0
SAL_TA6624AA	629315	Agona	0	0	0	0	0	0	0	0	0	0	0
SAL_TA6582AA	FSIS31801189	Agona	0	0	0	0	0	0	0	0	0	0	0
SAL_TA6539AA	621754	Agona	0	0	0	0	0	0	0	0	0	0	0
SAL_TA6445AA	FSIS11815297	Agona	0	0	0	0	0	0	0	0	0	0	0
SAL_TA6456AA	FSIS11815168	Agona	0	0	0	0	0	0	0	0	0	0	0
SAL_TA6777AA	FSIS11815515	Agona	0	0	0	0	0	0	0	0	0	0	0
SAL_TA6845AA	FSIS11815534	Agona	0	0	0	0	0	0	0	0	0	0	0
SAL_TA6949AA	633445	Agona	0	0	0	0	0	0	0	0	0	0	0
SAL_TA7020AA	CFSAN087703	Agona	0	0	0	0	0	0	0	0	0	0	0
SAL_TA7019AA	CFSAN087646	Agona	0	0	0	0	0	0	0	0	0	0	0
SAL_TA7038AA	CFSAN087708	Agona	0	0	0	0	0	0	0	0	0	0	0
SAL_TA6983AA	CFSAN087701	Agona	0	0	0	0	0	0	0	0	0	0	0
SAL_TA6991AA	CFSAN087718	Agona	0	0	0	0	0	0	0	0	0	0	0
SAL_TA6994AA	CFSAN087688	Agona	0	0	0	0	0	0	0	0	0	0	0
SAL_TA6998AA	CFSAN087681	Agona	0	0	0	0	0	0	0	0	0	0	0
SAL_TA7141AA	CFSAN087633	Agona	0	0	0	0	0	0	0	0	0	0	0
SAL_TA7153AA	CFSAN087690	Agona	0	0	0	0	0	0	0	0	0	0	0
SAL_TA7156AA	CFSAN087685	Agona	0	0	0	0	0	0	0	0	0	0	0
SAL_TA7162AA	CFSAN087700	Agona	0	0	0	0	0	0	0	0	0	0	0
SAL_TA7163AA	CFSAN087702	Agona	0	0	0	0	0	0	0	0	0	0	0
SAL_TA7165AA	CFSAN087686	Agona	0	0	0	0	0	0	0	0	0	0	0
SAL_TA7170AA	CFSAN087644	Agona	0	0	0	0	0	0	0	0	0	0	0
SAL_TA7172AA	CFSAN087682	Agona	0	0	0	0	0	0	0	0	0	0	0
SAL_TA7180AA	CFSAN087687	Agona	0	0	0	0	0	0	0	0	0	0	0
SAL_TA7303AA	201809433	Agona	0	0	0	0	0	0	0	0	0	0	0
SAL_SA3106AA		Agona	0	0	0	0	0	0	0	0	0	0	0
SAL_SA3104AA		Agona	0	0	0	0	0	0	0	0	0	0	0
SAL_SA3103AA		Agona	0	0	0	0	0	0	0	0	0	0	0
SAL_SA3102AA		Agona	0	0	0	0	0	0	0	0	0	0	0

SAL_SA3105AA		Agona	0	0	0	0	0	0	0	0	0	0	0
SAL_OA1698AA	PNUSAS034198	Agona	0	0	0	0	0	0	0	0	0	0	0
SAL_QA5329AA	262735	Agona	0	0	0	0	0	0	0	0	0	0	0
SAL_OA1946AA	PNUSAS033114	Agona	0	0	0	0	0	0	0	0	0	0	0
SAL_QA3730AA	PNUSAS041430	Agona	0	0	0	0	0	0	0	0	0	0	0
SAL_TA7889AA	SMG-18-2209	Agona	0	0	0	0	0	0	0	0	0	0	0
SAL_CA0834AA	02.O.05	Agona	0	0	0	0	0	0	0	0	0	0	0
SAL_CA0791AA	26.F.98	Agona	0	0	0	0	0	0	0	0	0	0	0
SAL_TA7810AA	l samples of swine - sero	Agona	0	0	0	0	0	0	0	0	0	0	0
SAL_TA7820AA	al samples of swine - ser	Agona	0	0	0	0	0	0	0	0	0	0	0
SAL_JA8775AA	FSIS1700930	Agona	0	0	0	0	0	0	0	0	0	0	0
SAL_NA5188AA	CFSAN074292	Agona	0	0	0	0	0	0	0	0	0	0	0
SAL_NA7579AA	PNUSAS031624	Agona	0	0	0	0	0	0	0	0	0	0	0
SAL_OA1426AA	FSIS11807631	Agona	0	0	0	0	0	0	0	0	0	0	0
SAL_TA8501AA	637850	Agona	0	0	0	0	0	0	0	0	0	0	0
SAL_TA8383AA	637728	Agona	0	0	0	0	0	0	0	0	0	0	0
SAL_TA8357AA	608560	Agona	0	0	0	0	0	0	0	0	0	0	0
SAL_TA8325AA	633411	Agona	0	0	0	0	0	0	0	0	0	0	0
SAL_TA8491AA	637880	Agona	0	0	0	0	0	0	0	0	0	0	0
SAL_TA8224AA	ADRDL-2265	Agona	0	0	0	0	0	0	0	0	0	0	0
SAL_TA8198AA	CFSAN087802	Agona	0	0	0	0	0	0	0	0	0	0	0
SAL_TA8692AA	201809896	Agona	0	0	0	0	0	0	0	0	0	0	0
SAL_RA9664AA		Agona	0	0	0	0	0	0	0	0	0	0	0
SAL_OA1382AA	PNUSAS034770	Agona	0	0	0	0	0	0	0	0	0	0	0
SAL_RA9629AA		Agona	0	0	0	0	0	0	0	0	0	0	0
SAL_RA9600AA		Agona	0	0	0	0	0	0	0	0	0	0	0
SAL_RA9596AA		Agona	0	0	0	0	0	0	0	0	0	0	0
SAL_SA6304AA	587815	Agona	0	0	0	0	0	0	0	0	0	0	0
SAL_RA9656AA		Agona	0	0	0	0	0	0	0	0	0	0	0
SAL_RA9662AA		Agona	0	0	0	0	0	0	0	0	0	0	0
SAL_RA9665AA		Agona	0	0	0	0	0	0	0	0	0	0	0
SAL_RA9660AA		Agona	0	0	0	0	0	0	0	0	0	0	0
SAL_RA9650AA		Agona	0	0	0	0	0	0	0	0	0	0	0
SAL_RA9648AA		Agona	0	0	0	0	0	0	0	0	0	0	0
SAL_RA9652AA		Agona	0	0	0	0	0	0	0	0	0	0	0
SAL_RA9646AA		Agona	0	0	0	0	0	0	0	0	0	0	0
SAL_RA9613AA		Agona	0	0	0	0	0	0	0	0	0	0	0
SAL_RA9490AA		Agona	0	0	0	0	0	0	0	0	0	0	0
SAL_RA9609AA		Agona	0	0	0	0	0	0	0	0	0	0	0
SAL_NA8832AA	PNUSAS033148	Agona	0	0	0	0	0	0	0	0	0	0	0
SAL_RA9658AA		Agona	0	0	0	0	0	0	0	0	0	0	0
SAL_RA9462AA		Agona	0	0	0	0	0	0	0	0	0	0	0
SAL_KA2150AA	FDA00004663	Agona	0	0	0	0	0	0	0	0	0	0	0
SAL_RA9615AA		Agona	0	0	0	0	0	0	0	0	0	0	0
SAL_RA9608AA		Agona	0	0	0	0	0	0	0	0	0	0	0
SAL_RA9611AA		Agona	0	0	0	0	0	0	0	0	0	0	0
SAL_RA9426AA		Agona	0	0	0	0	0	0	0	0	0	0	0
SAL_RA9433AA		Agona	0	0	0	0	0	0	0	0	0	0	0
SAL_RA9428AA		Agona	0	0	0	0	0	0	0	0	0	0	0
SAL_OA1365AA	PNUSAS034764	Agona	0	0	0	0	0	0	0	0	0	0	0
SAL_RA9644AA		Agona	0	0	0	0	0	0	0	0	0	0	0
SAL_RA9642AA		Agona	0	0	0	0	0	0	0	0	0	0	0
SAL_RA9590AA		Agona	0	0	0	0	0	0	0	0	0	0	0
SAL_RA9605AA		Agona	0	0	0	0	0	0	0	0	0	0	0
SAL_RA9625AA		Agona	0	0	0	0	0	0	0	0	0	0	0
SAL_RA9633AA		Agona	0	0	0	0	0	0	0	0	0	0	0
SAL_RA9623AA		Agona	0	0	0	0	0	0	0	0	0	0	0
SAL_RA9479AA		Agona	0	0	0	0	0	0	0	0	0	0	0
SAL_RA9484AA		Agona	0	0	0	0	0	0	0	0	0	0	0
SAL_RA9441AA		Agona	0	0	0	0	0	0	0	0	0	0	0
SAL_RA9632AA		Agona	0	0	0	0	0	0	0	0	0	0	0
SAL_RA9598AA		Agona	0	0	0	0	0	0	0	0	0	0	0
SAL_RA9627AA		Agona	0	0	0	0	0	0	0	0	0	0	0
SAL_RA9630AA		Agona	0	0	0	0	0	0	0	0	0	0	0
SAL_RA9594AA		Agona	0	0	0	0	0	0	0	0	0	0	0
SAL_RA9593AA		Agona	0	0	0	0	0	0	0	0	0	0	0
SAL_RA9447AA		Agona	0	0	0	0	0	0	0	0	0	0	0
SAL_QA9891AA	226995	Agona	0	0	0	0	0	0	0	0	0	0	0
SAL_OA3751AA	FSIS11807625	Agona	0	0	0	0	0	0	0	0	0	0	0
SAL_KA2127AA	FSIS1702020	Agona	0	0	0	0	0	0	0	0	0	0	0
SAL_RA9588AA		Agona	0	0	0	0	0	0	0	0	0	0	0
SAL_RA9654AA		Agona	0	0	0	0	0	0	0	0	0	0	0
SAL_RA9618AA		Agona	0	0	0	0	0	0	0	0	0	0	0

SAL_RA9620AA		Agona	0	0	0	0	0	0	0	0	0	0	0
SAL_RA9641AA		Agona	0	0	0	0	0	0	0	0	0	0	0
SAL_RA9639AA		Agona	0	0	0	0	0	0	0	0	0	0	0
SAL_RA9637AA		Agona	0	0	0	0	0	0	0	0	0	0	0
SAL_RA9635AA		Agona	0	0	0	0	0	0	0	0	0	0	0
SAL_RA9667AA		Agona	0	0	0	0	0	0	0	0	0	0	0
SAL_OA1345AA	PNUSAS020552	Agona	0	0	0	0	0	0	0	0	0	0	0
SAL_RA9616AA		Agona	0	0	0	0	0	0	0	0	0	0	0
SAL_RA9420AA		Agona	0	0	0	0	0	0	0	0	0	0	0
SAL_RA9603AA		Agona	0	0	0	0	0	0	0	0	0	0	0
SAL_RA9473AA		Agona	0	0	0	0	0	0	0	0	0	0	0
SAL_QA2450AA	199843	Agona	0	0	0	0	0	0	0	0	0	0	0
SAL_QA4276AA	PNUSAS040486	Agona	0	0	0	0	0	0	0	0	0	0	0
SAL_QA5309AA	449477	Agona	0	0	0	0	0	0	0	0	0	0	0
SAL_QA5321AA	304570	Agona	0	0	0	0	0	0	0	0	0	0	0
SAL_QA5393AA	404337	Agona	0	0	0	0	0	0	0	0	0	0	0
SAL_QA5406AA	287562	Agona	0	0	0	0	0	0	0	0	0	0	0
SAL_OA1638AA	PNUSAS034238	Agona	0	0	0	0	0	0	0	0	0	0	0
SAL_OA1298AA	PNUSAS033191	Agona	0	0	0	0	0	0	0	0	0	0	0
SAL_OA1882AA	PNUSAS035177	Agona	0	0	0	0	0	0	0	0	0	0	0
SAL_OA2076AA	FSIS21821150	Agona	0	0	0	0	0	0	0	0	0	0	0
SAL_OA1906AA	PNUSAS035161	Agona	0	0	0	0	0	0	0	0	0	0	0
SAL_OA2388AA	FDA00012613	Agona	0	0	0	0	0	0	0	0	0	0	0
SAL_OA2254AA	PNUSAS033969	Agona	0	0	0	0	0	0	0	0	0	0	0
SAL_OA4383AA	PNUSAS036576	Agona	0	0	0	0	0	0	0	0	0	0	0
SAL_OA5182AA	PNUSAS036186	Agona	0	0	0	0	0	0	0	0	0	0	0
SAL_OA5542AA	PNUSAS034455	Agona	0	0	0	0	0	0	0	0	0	0	0
SAL_OA5486AA	PNUSAS036218	Agona	0	0	0	0	0	0	0	0	0	0	0
SAL_OA6658AA	CFSAN078055	Agona	0	0	0	0	0	0	0	0	0	0	0
SAL_OA5162AA	PNUSAS035863	Agona	0	0	0	0	0	0	0	0	0	0	0
SAL_PA1195AA	CFSAN077980	Agona	0	0	0	0	0	0	0	0	0	0	0
SAL_PA1149AA	FSIS21821404	Agona	0	0	0	0	0	0	0	0	0	0	0
SAL_PA1510AA	PNUSAS038390	Agona	0	0	0	0	0	0	0	0	0	0	0
SAL_PA1515AA	PNUSAS038388	Agona	0	0	0	0	0	0	0	0	0	0	0
SAL_PA1613AA	FSIS21821434	Agona	0	0	0	0	0	0	0	0	0	0	0
SAL_PA1711AA	FSIS11809063	Agona	0	0	0	0	0	0	0	0	0	0	0
SAL_PA1901AA	PNUSAS038130	Agona	0	0	0	0	0	0	0	0	0	0	0
SAL_PA2156AA	FSIS11808917	Agona	0	0	0	0	0	0	0	0	0	0	0
SAL_PA2211AA	PNUSAS036224	Agona	0	0	0	0	0	0	0	0	0	0	0
SAL_PA2451AA	PNUSAS036940	Agona	0	0	0	0	0	0	0	0	0	0	0
SAL_PA2824AA	PNUSAS038998	Agona	0	0	0	0	0	0	0	0	0	0	0
SAL_PA3664AA	PNUSAS037952	Agona	0	0	0	0	0	0	0	0	0	0	0
SAL_PA3769AA	16-6-18-8100301-K	Agona	0	0	0	0	0	0	0	0	0	0	0
SAL_PA8714AA	412248	Agona	0	0	0	0	0	0	0	0	0	0	0
SAL_TA9615AA	FSIS11809122	Agona	0	0	0	0	0	0	0	0	0	0	0
SAL_TA9547AA	641631	Agona	0	0	0	0	0	0	0	0	0	0	0
SAL_TA9751AA	641630	Agona	0	0	0	0	0	0	0	0	0	0	0
SAL_TA9891AA	FSIS11816140	Agona	0	0	0	0	0	0	0	0	0	0	0
SAL_SA1827AA	20021473	Agona	0	0	0	0	0	0	0	0	0	0	0
SAL_SA1861AA	SF_R0370/09	Agona	0	0	0	0	0	0	0	0	0	0	0
SAL_UA0055AA	201810321	Agona	0	0	0	0	0	0	0	0	0	0	0
SAL_UA0057AA	201810315	Agona	0	0	0	0	0	0	0	0	0	0	0
SAL_UA0092AA	201810375	Agona	0	0	0	0	0	0	0	0	0	0	0
SAL_UA0093AA	201810352	Agona	0	0	0	0	0	0	0	0	0	0	0
SAL_UA2301AA	201810489	Agona	0	0	0	0	0	0	0	0	0	0	0
SAL_UA1702AA	647802	Agona	0	0	0	0	0	0	0	0	0	0	0
SAL_UA1650AA	FSIS31801314	Agona	0	0	0	0	0	0	0	0	0	0	0
SAL_UA2696AA	648590	Agona	0	0	0	0	0	0	0	0	0	0	0
SAL_UA2781AA	648596	Agona	0	0	0	0	0	0	0	0	0	0	0
SAL_UA2758AA	647431	Agona	0	0	0	0	0	0	0	0	0	0	0
SAL_UA2830AA	645997	Agona	0	0	0	0	0	0	0	0	0	0	0
SAL_UA2853AA	649902	Agona	0	0	0	0	0	0	0	0	0	0	0
SAL_UA2930AA	602630	Agona	0	0	0	0	0	0	0	0	0	0	0
SAL_UA2917AA	639808	Agona	0	0	0	0	0	0	0	0	0	0	0
SAL_UA2902AA	650062	Agona	0	0	0	0	0	0	0	0	0	0	0
SAL_UA6785AA	661069	Agona	0	0	0	0	0	0	0	0	0	0	0
SAL_UA7159AA	201811253	Agona	0	0	0	0	0	0	0	0	0	0	0
SAL_UA7160AA	201811203	Agona	0	0	0	0	0	0	0	0	0	0	0
SAL_UA7238AA	FSIS11816711	Agona	0	0	0	0	0	0	0	0	0	0	0
SAL_UA7232AA	FSIS11816965	Agona	0	0	0	0	0	0	0	0	0	0	0
SAL_UA2935AA	601644	Agona	0	0	0	0	0	0	0	0	0	0	0
SAL_UA3002AA	604959	Agona	0	0	0	0	0	0	0	0	0	0	0
SAL_UA3031AA	649015	Agona	0	0	0	0	0	0	0	0	0	0	0

SAL_UA2991AA	647856	Agona	0	0	0	0	0	0	0	0	0	0	0
SAL_UA3095AA	31_Agona_cp	Agona	0	0	0	0	0	0	0	0	0	0	0
SAL_UA4748AA	651069	Agona	0	0	0	0	0	0	0	0	0	0	0
SAL_UA4762AA	651074	Agona	0	0	0	0	0	0	0	0	0	0	0
SAL_UA4851AA	592669	Agona	0	0	0	0	0	0	0	0	0	0	0
SAL_UA4874AA	CFSAN089072	Agona	0	0	0	0	0	0	0	0	0	0	0
SAL_UA4863AA	CFSAN089102	Agona	0	0	0	0	0	0	0	0	0	0	0
SAL_UA5064AA	FSIS11816382	Agona	0	0	0	0	0	0	0	0	0	0	0
SAL_UA5123AA	FSIS21822988	Agona	0	0	0	0	0	0	0	0	0	0	0
SAL_UA5276AA	201810914	Agona	0	0	0	0	0	0	0	0	0	0	0
SAL_UA5275AA	201810913	Agona	0	0	0	0	0	0	0	0	0	0	0
SAL_UA5337AA	651052	Agona	0	0	0	0	0	0	0	0	0	0	0
SAL_UA5331AA	653641	Agona	0	0	0	0	0	0	0	0	0	0	0
SAL_UA5368AA	620449	Agona	0	0	0	0	0	0	0	0	0	0	0
SAL_UA5603AA	591241	Agona	0	0	0	0	0	0	0	0	0	0	0
SAL_UA5690AA	201808653	Agona	0	0	0	0	0	0	0	0	0	0	0
SAL_UA5650AA	201810785	Agona	0	0	0	0	0	0	0	0	0	0	0
SAL_UA5689AA	201808477	Agona	0	0	0	0	0	0	0	0	0	0	0
SAL_UA5691AA	201808287	Agona	0	0	0	0	0	0	0	0	0	0	0
SAL_UA5692AA	201808715	Agona	0	0	0	0	0	0	0	0	0	0	0
SAL_UA5939AA	FSIS11816545	Agona	0	0	0	0	0	0	0	0	0	0	0
SAL_UA6064AA	656768	Agona	0	0	0	0	0	0	0	0	0	0	0
SAL_UA6077AA	621744	Agona	0	0	0	0	0	0	0	0	0	0	0
SAL_UA6187AA	582940	Agona	0	0	0	0	0	0	0	0	0	0	0
SAL_UA6147AA	585941	Agona	0	0	0	0	0	0	0	0	0	0	0
SAL_UA6164AA	579233	Agona	0	0	0	0	0	0	0	0	0	0	0
SAL_UA6210AA	637848	Agona	0	0	0	0	0	0	0	0	0	0	0
SAL_UA6475AA	FSIS11816339	Agona	0	0	0	0	0	0	0	0	0	0	0
SAL_UA6522AA	576383	Agona	0	0	0	0	0	0	0	0	0	0	0
SAL_CA0800AA	20.H.06	Agona	0	0	0	0	0	0	0	0	0	0	0
SAL_UA6701AA	JE_S09-000989	Agona	0	0	0	0	0	0	0	0	0	0	0
SAL_CA0773AA	35.H.08	Agona	0	0	0	0	0	0	0	0	0	0	0
SAL_UA7086AA	AG18-0057	Agona	0	0	0	0	0	0	0	0	0	0	0
SAL_UA7089AA	AG18-0040	Agona	0	0	0	0	0	0	0	0	0	0	0
SAL_GA6931AA	A51	Agona	0	0	0	0	0	0	0	0	0	0	0
SAL_GA6896AA	A10	Agona	0	0	0	0	0	0	0	0	0	0	0
SAL_UA7950AA	FSIS21923161	Agona	0	0	0	0	0	0	0	0	0	0	0
SAL_UA7793AA	662761	Agona	0	0	0	0	0	0	0	0	0	0	0
SAL_UA7520AA	AHD44	Agona	0	0	0	0	0	0	0	0	0	0	0
SAL_UA7392AA	664946	Agona	0	0	0	0	0	0	0	0	0	0	0
SAL_UA8154AA	668717	Agona	0	0	0	0	0	0	0	0	0	0	0
SAL_UA8315AA	568428	Agona	0	0	0	0	0	0	0	0	0	0	0
SAL_UA9496AA	201900214	Agona	0	0	0	0	0	0	0	0	0	0	0
SAL_UA9649AA	Sal0522	Agona	0	0	0	0	0	0	0	0	0	0	0
SAL_UA8381AA	NC_810120176	Agona	0	0	0	0	0	0	0	0	0	0	0
SAL_UA8740AA	668712	Agona	0	0	0	0	0	0	0	0	0	0	0
SAL_UA8883AA	AG19-0004	Agona	0	0	0	0	0	0	0	0	0	0	0
SAL_UA9635AA	668755	Agona	0	0	0	0	0	0	0	0	0	0	0
SAL_UA9640AA	634723	Agona	0	0	0	0	0	0	0	0	0	0	0
SAL_UA9562AA	628866	Agona	0	0	0	0	0	0	0	0	0	0	0
SAL_UA9556AA	615614	Agona	0	0	0	0	0	0	0	0	0	0	0
SAL_UA8697AA	670190	Agona	0	0	0	0	0	0	0	0	0	0	0
SAL_UA9703AA	672556	Agona	0	0	0	0	0	0	0	0	0	0	0
SAL_UA9691AA	672555	Agona	0	0	0	0	0	0	0	0	0	0	0
SAL_UA9926AA	553896	Agona	0	0	0	0	0	0	0	0	0	0	0
SAL_UA9782AA	550834	Agona	0	0	0	0	0	0	0	0	0	0	0
SAL_VA1233AA	201900226	Agona	0	0	0	0	0	0	0	0	0	0	0
SAL_VA1648AA	SMG-19-159	Agona	0	0	0	0	0	0	0	0	0	0	0
SAL_VA0729AA	526183	Agona	16	0	0	0	0	0	0	0	0	0	0
SAL_VA0541AA	SAL-18-VL-SD-MN-0022	Agona	0	0	0	0	0	0	0	0	0	0	0
SAL_VA0533AA	FSLS5-0647	Agona	0	0	0	0	0	0	0	0	0	0	0
SAL_VA0532AA	FSLS5-0547	Agona	0	0	0	0	0	0	0	0	0	0	0
SAL_VA0848AA	539159	Agona	0	0	0	0	0	0	0	0	0	0	0
SAL_VA0822AA	529840	Agona	0	0	0	0	0	0	0	0	0	0	0
SAL_VA0798AA	545263	Agona	0	0	0	0	0	0	0	0	0	0	0
SAL_VA0791AA	550783	Agona	0	0	0	0	0	0	0	0	0	0	0
SAL_VA0749AA	520525	Agona	0	0	0	0	0	0	0	0	0	0	0
SAL_VA0345AA	534797	Agona	0	0	0	0	0	0	0	0	0	0	0
SAL_VA0325AA	546618	Agona	0	0	0	0	0	0	0	0	0	0	0
SAL_VA0311AA	553787	Agona	0	0	0	0	0	0	0	0	0	0	0
SAL_VA0319AA	545296	Agona	0	0	0	0	0	0	0	0	0	0	0
SAL_VA0297AA	553748	Agona	0	0	0	0	0	0	0	0	0	0	0
SAL_VA0890AA	ADRDL-2401	Agona	0	0	0	0	0	0	0	0	0	0	0

SAL_UA9828AA	561122	Agona	0	0	0	0	0	0	0	0	0	0	0
SAL_VA1151AA	527508	Agona	0	0	0	0	0	0	0	0	0	0	0
SAL_VA1175AA	506057	Agona	0	0	0	0	0	0	0	0	0	0	0
SAL_VA1177AA	506428	Agona	0	0	0	0	0	0	0	0	0	0	0
SAL_VA1081AA	511837	Agona	0	0	0	0	0	0	0	0	0	0	0
SAL_VA1091AA	511842	Agona	0	0	0	0	0	0	0	0	0	0	0
SAL_VA7051AA	SMG-19-218	Agona	0	0	0	0	0	0	0	0	0	0	0
SAL_VA1429AA	482164	Agona	0	0	0	0	0	0	0	0	0	0	0
SAL_VA1386AA	673561	Agona	0	0	0	0	0	0	0	0	0	0	0
SAL_VA1339AA	486535	Agona	0	0	0	0	0	0	0	0	0	0	0
SAL_VA1348AA	676364	Agona	0	0	0	0	0	0	0	0	0	0	0
SAL_VA1347AA	673585	Agona	0	0	0	0	0	0	0	0	0	0	0
SAL_VA1278AA	676392	Agona	0	0	0	0	0	0	0	0	0	0	0
SAL_VA7490AA	201704897	Agona	0	0	0	0	0	0	0	0	0	0	0
SAL_VA7661AA	201710128	Agona	0	0	0	0	0	0	0	0	0	0	0
SAL_VA2153AA	452474	Agona	0	0	0	0	0	0	0	0	0	0	0
SAL_VA2087AA	461169	Agona	0	0	0	0	0	0	0	0	0	0	0
SAL_VA2275AA	467175	Agona	0	0	0	0	0	0	0	0	0	0	0
SAL_VA2060AA	458080	Agona	0	0	0	0	0	0	0	0	0	0	0
SAL_VA1946AA	435386	Agona	0	0	0	0	0	0	0	0	0	0	0
SAL_VA3280AA	412032	Agona	0	0	0	0	0	0	0	0	0	0	0
SAL_VA3283AA	417394	Agona	0	0	0	0	0	0	0	0	0	0	0
SAL_VA3267AA	FSIS31901510	Agona	0	0	0	0	0	0	0	0	0	0	0
SAL_VA3171AA	417386	Agona	0	0	0	0	0	0	0	0	0	0	0
SAL_VA3122AA	412151	Agona	0	0	0	0	0	0	0	0	0	0	0
SAL_VA3106AA	414680	Agona	0	0	0	0	0	0	0	0	0	0	0
SAL_VA3076AA	412111	Agona	0	0	0	0	0	0	0	0	0	0	0
SAL_VA3176AA	424600	Agona	0	0	0	0	0	0	0	0	0	0	0
SAL_VA3224AA	408547	Agona	0	0	0	0	0	0	0	0	0	0	0
SAL_VA3174AA	408390	Agona	0	0	0	0	0	0	0	0	0	0	0
SAL_VA3462AA	399303	Agona	0	0	0	0	0	0	0	0	0	0	0
SAL_VA3401AA	417388	Agona	0	0	0	0	0	0	0	0	0	0	0
SAL_VA3433AA	399300	Agona	0	0	0	0	0	0	0	0	0	0	0
SAL_VA2379AA	481512	Agona	0	0	0	0	0	0	0	0	0	0	0
SAL_VA2355AA	473198	Agona	0	0	0	0	0	0	0	0	0	0	0
SAL_VA2255AA	493489	Agona	0	0	0	0	0	0	0	0	0	0	0
SAL_VA2324AA	493381	Agona	0	0	0	0	0	0	0	0	0	0	0
SAL_VA3424AA	404881	Agona	0	0	0	0	0	0	0	0	0	0	0
SAL_VA2219AA	470206	Agona	0	0	0	0	0	0	0	0	0	0	0
SAL_VA2165AA	452475	Agona	0	0	0	0	0	0	0	0	0	0	0
SAL_VA2035AA	449709	Agona	0	0	0	0	0	0	0	0	0	0	0
SAL_VA1978AA	435481	Agona	0	0	0	0	0	0	0	0	0	0	0
SAL_VA1999AA	442155	Agona	0	0	0	0	0	0	0	0	0	0	0
SAL_VA1928AA	445937	Agona	0	0	0	0	0	0	0	0	0	0	0
SAL_VA1888AA	442167	Agona	0	0	0	0	0	0	0	0	0	0	0
SAL_VA1895AA	440916	Agona	0	0	0	0	0	0	0	0	0	0	0
SAL_VA1857AA	448162	Agona	0	0	0	0	0	0	0	0	0	0	0
SAL_VA2531AA	429735	Agona	0	0	0	0	0	0	0	0	0	0	0
SAL_VA2483AA	422476	Agona	0	0	0	0	0	0	0	0	0	0	0
SAL_VA2444AA	452473	Agona	0	0	0	0	0	0	0	0	0	0	0
SAL_VA2469AA	432773	Agona	0	0	0	0	0	0	0	0	0	0	0
SAL_VA2769AA	678041	Agona	0	0	0	0	0	0	0	0	0	0	0
SAL_VA2717AA	454887	Agona	0	0	0	0	0	0	0	0	0	0	0
SAL_VA2697AA	423859	Agona	0	0	0	0	0	0	0	0	0	0	0
SAL_VA2699AA	416222	Agona	0	0	0	0	0	0	0	0	0	0	0
SAL_VA2700AA	678555	Agona	0	0	0	0	0	0	0	0	0	0	0
SAL_VA3319AA	AG19-0037	Agona	0	0	0	0	0	0	0	0	0	0	0
SAL_VA8313AA	201900832	Agona	0	0	0	0	0	0	0	0	0	0	0
SAL_VA5938AA	MZ0695	Agona	0	0	0	0	0	0	0	0	0	0	0
SAL_VA5931AA	SA20062417	Agona	0	0	0	0	0	0	0	0	0	0	0
SAL_VA5934AA	SA20093321	Agona	0	0	0	0	0	0	0	0	0	0	0
SAL_VA5685AA	SA20094946	Agona	0	0	0	0	0	0	0	0	0	0	0
SAL_VA5550AA	CFS0032	Agona	0	0	0	0	0	0	0	0	0	0	0
SAL_VA4824AA	Agona ATCC-BAA-707	Agona	0	0	0	0	0	0	0	0	0	0	0
SAL_VA4841AA	Agona ATCC-BAA-707	Agona	0	0	0	0	0	0	0	0	0	0	0
SAL_VA4773AA	Agona ATCC-BAA-707	Agona	0	0	0	0	0	0	0	0	0	0	0
SAL_VA4774AA	Agona ATCC-BAA-707	Agona	0	0	0	0	0	0	0	0	0	0	0
SAL_VA4775AA	Agona ATCC-BAA-707	Agona	0	0	0	0	0	0	0	0	0	0	0
SAL_VA4776AA	Agona ATCC-BAA-707	Agona	0	0	0	0	0	0	0	0	0	0	0
SAL_VA4777AA	Agona ATCC-BAA-707	Agona	0	0	0	0	0	0	0	0	0	0	0
SAL_VA4778AA	Agona ATCC-BAA-707	Agona	0	0	0	0	0	0	0	0	0	0	0
SAL_VA4781AA	Agona ATCC-BAA-707	Agona	0	0	0	0	0	0	0	0	0	0	0
SAL_VA4782AA	Agona ATCC-BAA-707	Agona	0	0	0	0	0	0	0	0	0	0	0

SAL_VA4762AA	Agona ATCC-BAA-707	Agona	0	0	0	0	0	0	0	0	0	0	0
SAL_VA4779AA	Agona ATCC-BAA-707	Agona	0	0	0	0	0	0	0	0	0	0	0
SAL_VA4780AA	Agona ATCC-BAA-707	Agona	0	0	0	0	0	0	0	0	0	0	0
SAL_VA4681AA	Agona ATCC-BAA-707	Agona	0	0	0	0	0	0	0	0	0	0	0
SAL_VA4712AA	Agona ATCC-BAA-707	Agona	0	0	0	0	0	0	0	0	0	0	0
SAL_VA4713AA	Agona ATCC-BAA-707	Agona	0	0	0	0	0	0	0	0	0	0	0
SAL_VA4714AA	Agona ATCC-BAA-707	Agona	0	0	0	0	0	0	0	0	0	0	0
SAL_VA4716AA	Agona ATCC-BAA-707	Agona	0	0	0	0	0	0	0	0	0	0	0
SAL_VA4717AA	Agona ATCC-BAA-707	Agona	0	0	0	0	0	0	0	0	0	0	0
SAL_VA4719AA	Agona ATCC-BAA-707	Agona	0	0	0	0	0	0	0	0	0	0	0
SAL_VA4720AA	Agona ATCC-BAA-707	Agona	0	0	0	0	0	0	0	0	0	0	0
SAL_VA4721AA	Agona ATCC-BAA-707	Agona	0	0	0	0	0	0	0	0	0	0	0
SAL_VA4724AA	Agona ATCC-BAA-707	Agona	0	0	0	0	0	0	0	0	0	0	0
SAL_VA4730AA	Agona ATCC-BAA-707	Agona	0	0	0	0	0	0	0	0	0	0	0
SAL_VA4671AA	Agona ATCC-BAA-707	Agona	0	0	0	0	0	0	0	0	0	0	0
SAL_VA4672AA	Agona ATCC-BAA-707	Agona	0	0	0	0	0	0	0	0	0	0	0
SAL_VA4673AA	Agona ATCC-BAA-707	Agona	0	0	0	0	0	0	0	0	0	0	0
SAL_VA4674AA	Agona ATCC-BAA-707	Agona	0	0	0	0	0	0	0	0	0	0	0
SAL_VA4675AA	Agona ATCC-BAA-707	Agona	0	0	0	0	0	0	0	0	0	0	0
SAL_VA4676AA	Agona ATCC-BAA-707	Agona	0	0	0	0	0	0	0	0	0	0	0
SAL_VA4677AA	Agona ATCC-BAA-707	Agona	0	0	0	0	0	0	0	0	0	0	0
SAL_VA4678AA	Agona ATCC-BAA-707	Agona	0	0	0	0	0	0	0	0	0	0	0
SAL_VA4679AA	Agona ATCC-BAA-707	Agona	0	0	0	0	0	0	0	0	0	0	0
SAL_VA4680AA	Agona ATCC-BAA-707	Agona	0	0	0	0	0	0	0	0	0	0	0
SAL_VA4634AA	Agona ATCC-BAA-707	Agona	0	0	0	0	0	0	0	0	0	0	0
SAL_VA4643AA	Agona ATCC-BAA-707	Agona	0	0	0	0	0	0	0	0	0	0	0
SAL_VA4644AA	Agona ATCC-BAA-707	Agona	0	0	0	0	0	0	0	0	0	0	0
SAL_VA4645AA	Agona ATCC-BAA-707	Agona	0	0	0	0	0	0	0	0	0	0	0
SAL_VA4646AA	Agona ATCC-BAA-707	Agona	0	0	0	0	0	0	0	0	0	0	0
SAL_VA4647AA	Agona ATCC-BAA-707	Agona	0	0	0	0	0	0	0	0	0	0	0
SAL_VA4648AA	Agona ATCC-BAA-707	Agona	0	0	0	0	0	0	0	0	0	0	0
SAL_VA4715AA	Agona ATCC-BAA-707	Agona	0	0	0	0	0	0	0	0	0	0	0
SAL_VA4484AA	402795	Agona	0	0	0	0	0	0	0	0	0	0	0
SAL_VA4406AA	388740	Agona	0	0	0	0	0	0	0	0	0	0	0
SAL_VA4414AA	423885	Agona	0	0	0	0	0	0	0	0	0	0	0
SAL_VA4436AA	681320	Agona	0	0	0	0	0	0	0	0	0	0	0
SAL_WA1131AA	SMG-19-327	Agona	0	0	0	0	0	0	0	0	0	0	0
SAL_WA1144AA	SMG-19-343	Agona	0	0	0	0	0	0	0	0	0	0	0
SAL_VA4341AA	385918	Agona	0	0	0	0	0	0	0	0	0	0	0
SAL_VA4376AA	387074	Agona	0	0	0	0	0	0	0	0	0	0	0
SAL_VA3643AA	not applicale	Agona	0	0	0	0	0	0	0	0	0	0	0
SAL_VA3644AA	not applicale	Agona	0	0	0	0	0	0	0	0	0	0	0
SAL_VA3645AA	not applicale	Agona	0	0	0	0	0	0	0	0	0	0	0
SAL_VA3646AA	not applicale	Agona	0	0	0	0	0	0	0	0	0	0	0
SAL_VA3647AA	not applicale	Agona	0	0	0	0	0	0	0	0	0	0	0
SAL_VA3648AA	not applicale	Agona	0	0	0	0	0	0	0	0	0	0	0
SAL_VA3652AA	not applicale	Agona	0	0	0	0	0	0	0	0	0	0	0
SAL_VA3653AA	not applicale	Agona	0	0	0	0	0	0	0	0	0	0	0
SAL_VA3654AA	not applicale	Agona	0	0	0	0	0	0	0	0	0	0	0
SAL_VA3655AA	not applicale	Agona	0	0	0	0	0	0	0	0	0	0	0
SAL_VA3656AA	not applicale	Agona	0	0	0	0	0	0	0	0	0	0	0
SAL_VA3657AA	not applicale	Agona	0	0	0	0	0	0	0	0	0	0	0
SAL_VA3658AA	not applicale	Agona	0	0	0	0	0	0	0	0	0	0	0
SAL_VA3660AA	not applicale	Agona	0	0	0	0	0	0	0	0	0	0	0
SAL_VA3661AA	not applicale	Agona	0	0	0	0	0	0	0	0	0	0	0
SAL_VA3662AA	not applicale	Agona	0	0	0	0	0	0	0	0	0	0	0
SAL_VA3663AA	not applicale	Agona	0	0	0	0	0	0	0	0	0	0	0
SAL_VA3664AA	not applicale	Agona	0	0	0	0	0	0	0	0	0	0	0
SAL_VA3665AA	not applicale	Agona	0	0	0	0	0	0	0	0	0	0	0
SAL_VA3666AA	not applicale	Agona	0	0	0	0	0	0	0	0	0	0	0
SAL_VA3667AA	not applicale	Agona	0	0	0	0	0	0	0	0	0	0	0
SAL_VA3668AA	not applicale	Agona	0	0	0	0	0	0	0	0	0	0	0
SAL_VA3669AA	not applicale	Agona	0	0	0	0	0	0	0	0	0	0	0
SAL_VA3670AA	not applicale	Agona	0	0	0	0	0	0	0	0	0	0	0
SAL_VA3671AA	not applicale	Agona	0	0	0	0	0	0	0	0	0	0	0
SAL_VA3672AA	not applicale	Agona	0	0	0	0	0	0	0	0	0	0	0
SAL_VA3673AA	not applicale	Agona	0	0	0	0	0	0	0	0	0	0	0
SAL_VA3674AA	not applicale	Agona	0	0	0	0	0	0	0	0	0	0	0
SAL_VA3675AA	not applicale	Agona	0	0	0	0	0	0	0	0	0	0	0
SAL_VA3676AA	not applicale	Agona	0	0	0	0	0	0	0	0	0	0	0
SAL_VA3678AA	not applicale	Agona	0	0	0	0	0	0	0	0	0	0	0
SAL_VA3679AA	not applicale	Agona	0	0	0	0	0	0	0	0	0	0	0
SAL_VA3681AA	not applicale	Agona	0	0	0	0	0	0	0	0	0	0	0

SAL_VA3682AA	not applicale	Agona	0	0	0	0	0	0	0	0	0	0	0
SAL_VA3683AA	not applicale	Agona	0	0	0	0	0	0	0	0	0	0	0
SAL_VA3684AA	not applicale	Agona	0	0	0	0	0	0	0	0	0	0	0
SAL_VA3685AA	not applicale	Agona	0	0	0	0	0	0	0	0	0	0	0
SAL_VA3686AA	not applicale	Agona	0	0	0	0	0	0	0	0	0	0	0
SAL_VA6940AA	376030	Agona	0	0	0	0	0	0	0	0	0	0	0
SAL_VA6899AA	683046	Agona	0	0	0	0	0	0	0	0	0	0	0
SAL_VA6919AA	402785	Agona	0	0	0	0	0	0	0	0	0	0	0
SAL_VA6861AA	373830	Agona	0	0	0	0	0	0	0	0	0	0	0
SAL_VA7034AA	AMC 270	Agona	0	0	0	0	0	0	0	0	0	0	0
SAL_VA7213AA	373145	Agona	0	0	0	0	0	0	0	0	0	0	0
SAL_VA7223AA	370014	Agona	0	0	0	0	0	0	0	0	0	0	0
SAL_VA7091AA	370631	Agona	0	0	0	0	0	0	0	0	0	0	0
SAL_VA7104AA	682237	Agona	0	0	0	0	0	0	0	0	0	0	0
SAL_VA7726AA	FSIS11917536	Agona	0	0	0	0	0	0	0	0	0	0	0
SAL_VA7827AA	FSIS21923539	Agona	0	0	0	0	0	0	0	0	0	0	0
SAL_VA8294AA	378707	Agona	0	0	0	0	0	0	0	0	0	0	0
SAL_VA8261AA	690395	Agona	0	0	0	0	0	0	0	0	0	0	0
SAL_VA8207AA	686642	Agona	0	0	0	0	0	0	0	0	0	0	0
SAL_VA8222AA	366356	Agona	0	0	0	0	0	0	0	0	0	0	0
SAL_VA8179AA	373890	Agona	0	0	0	0	0	0	0	0	0	0	0
SAL_VA8151AA	376289	Agona	0	0	0	0	0	0	0	0	0	0	0
SAL_VA8152AA	685514	Agona	0	0	0	0	0	0	0	0	0	0	0
SAL_VA8154AA	417390	Agona	0	0	0	0	0	0	0	0	0	0	0
SAL_VA8165AA	689000	Agona	0	0	0	0	0	0	0	0	0	0	0
SAL_VA8588AA	341281	Agona	0	0	0	0	0	0	0	0	0	0	0
SAL_VA8387AA	360354	Agona	0	0	0	0	0	0	0	0	0	0	0
SAL_VA8341AA	FSIS31901602	Agona	0	0	0	0	0	0	0	0	0	0	0
SAL_VA8513AA	383775	Agona	0	0	0	0	0	0	0	0	0	0	0
SAL_VA8514AA	349300	Agona	0	0	0	0	0	0	0	0	0	0	0
SAL_VA8497AA	350686	Agona	0	0	0	0	0	0	0	0	0	0	0
SAL_VA8494AA	376023	Agona	0	0	0	0	0	0	0	0	0	0	0
SAL_VA8500AA	355111	Agona	0	0	0	0	0	0	0	0	0	0	0
SAL_VA8464AA	325711	Agona	0	0	0	0	0	0	0	0	0	0	0
SAL_VA8458AA	690371	Agona	0	0	0	0	0	0	0	0	0	0	0
SAL_VA8612AA	FSIS11917999	Agona	0	0	0	0	0	0	0	0	0	0	0
SAL_VA8427AA	327984	Agona	0	0	0	0	0	0	0	0	0	0	0
SAL_VA8620AA	330440	Agona	0	0	0	0	0	0	0	0	0	0	0
SAL_VA8792AA	320197	Agona	0	0	0	0	0	0	0	0	0	0	0
SAL_VA8764AA	360482	Agona	0	0	0	0	0	0	0	0	0	0	0
SAL_VA8771AA	323020	Agona	0	0	0	0	0	0	0	0	0	0	0
SAL_VA8671AA	315620	Agona	0	0	0	0	0	0	0	0	0	0	0
SAL_VA8679AA	323017	Agona	0	0	0	0	0	0	0	0	0	0	0
SAL_VA8672AA	320297	Agona	0	0	0	0	0	0	0	0	0	0	0
SAL_VA8680AA	365830	Agona	0	0	0	0	0	0	0	0	0	0	0
SAL_VA9184AA	306475	Agona	0	0	0	0	0	0	0	0	0	0	0
SAL_VA9189AA	311198	Agona	0	0	0	0	0	0	0	0	0	0	0
SAL_VA9137AA	305198	Agona	0	0	0	0	0	0	0	0	0	0	0
SAL_VA9139AA	346908	Agona	0	0	0	0	0	0	0	0	0	0	0
SAL_VA9113AA	306719	Agona	0	0	0	0	0	0	0	0	0	0	0
SAL_VA9069AA	317699	Agona	0	0	0	0	0	0	0	0	0	0	0
SAL_VA9022AA	292949	Agona	0	0	0	0	0	0	0	0	0	0	0
SAL_VA8929AA	302902	Agona	0	0	0	0	0	0	0	0	0	0	0
SAL_VA8967AA	302541	Agona	0	0	0	0	0	0	0	0	0	0	0
SAL_VA8962AA	311167	Agona	0	0	0	0	0	0	0	0	0	0	0
SAL_VA9370AA	286817	Agona	0	0	0	0	0	0	0	0	0	0	0
SAL_VA9335AA	279941	Agona	0	0	0	0	0	0	0	0	0	0	0
SAL_VA4149AA	CDPH_CSalm158	Agona	0	0	0	0	0	0	0	0	0	0	0
SAL_VA9587AA	268669	Agona	0	0	0	0	0	0	0	0	0	0	0
SAL_VA4175AA	CDPH_CSalm005	Agona	0	0	0	0	0	0	0	0	0	0	0
SAL_VA9561AA	267821	Agona	0	0	0	0	0	0	0	0	0	0	0
SAL_VA9491AA	278635	Agona	0	0	0	0	0	0	0	0	0	0	0
SAL_VA9509AA	296157	Agona	0	0	0	0	0	0	0	0	0	0	0
SAL_VA9796AA	249325	Agona	0	0	0	0	0	0	0	0	0	0	0
SAL_VA9806AA	695964	Agona	0	0	0	0	0	0	0	0	0	0	0
SAL_VA9760AA	254646	Agona	0	0	0	0	0	0	0	0	0	0	0
SAL_VA9708AA	274299	Agona	0	0	0	0	0	0	0	0	0	0	0
SAL_VA9721AA	692640	Agona	0	0	0	0	0	0	0	0	0	0	0
SAL_VA9723AA	692576	Agona	0	0	0	0	0	0	0	0	0	0	0
SAL_VA9750AA	308520	Agona	0	0	0	0	0	0	0	0	0	0	0
SAL_VA9683AA	262765	Agona	0	0	0	0	0	0	0	0	0	0	0
SAL_VA9694AA	253892	Agona	0	0	0	0	0	0	0	0	0	0	0
SAL_VA9695AA	695972	Agona	0	0	0	0	0	0	0	0	0	0	0

SAL_VA9968AA	224134	Agona	0	0	0	0	0	0	0	0	0	0
SAL_WA0276AA	205601	Agona	0	0	0	0	0	0	0	0	0	0
SAL_WA0242AA	693353	Agona	0	0	0	0	0	0	0	0	0	0
SAL_WA0248AA	206739	Agona	0	0	0	0	0	0	0	0	0	0
SAL_WA0214AA	231104	Agona	0	0	0	0	0	0	0	0	0	0
SAL_WA0226AA	246199	Agona	0	0	0	0	0	0	0	0	0	0
SAL_WA0219AA	203646	Agona	0	0	0	0	0	0	0	0	0	0
SAL_WA0151AA	212749	Agona	0	0	0	0	0	0	0	0	0	0
SAL_WA0161AA	200656	Agona	0	0	0	0	0	0	0	0	0	0
SAL_WA0607AA	190789	Agona	0	0	0	0	0	0	0	0	0	0
SAL_WA0126AA	232084	Agona	0	0	0	0	0	0	0	0	0	0
SAL_WA0597AA	183645	Agona	0	0	0	0	0	0	0	0	0	0
SAL_WA0506AA	191873	Agona	0	0	0	0	0	0	0	0	0	0
SAL_WA0483AA	184408	Agona	0	0	0	0	0	0	0	0	0	0
SAL_WA0343AA	178640	Agona	0	0	0	0	0	0	0	0	0	0
SAL_WA0761AA	160841	Agona	0	0	0	0	0	0	0	0	0	0
SAL_WA0917AA	178638	Agona	0	0	0	0	0	0	0	0	0	0
SAL_WA0945AA	163161	Agona	0	0	0	0	0	0	0	0	0	0
SAL_WA0972AA	152419	Agona	0	0	0	0	0	0	0	0	0	0
SAL_WA0892AA	166289	Agona	0	0	0	0	0	0	0	0	0	0
SAL_VA4076AA	CDPH_CSalm251	Agona	0	0	0	0	0	0	0	0	0	0
SAL_WA3297AA	MC_02-0142	Agona	0	0	0	0	0	0	0	0	0	0
SAL_WA3301AA	MC_02-0154	Agona	0	0	0	0	0	0	0	0	0	0
SAL_WA3295AA	MC_01-1165	Agona	0	0	0	0	0	0	0	0	0	0
SAL_WA3277AA	MC_01-0992	Agona	0	0	0	0	0	0	0	0	0	0
SAL_WA3262AA	MC_02-0642	Agona	0	0	0	0	0	0	0	0	0	0
SAL_WA3179AA	MC_01-0136	Agona	0	0	0	0	0	0	0	0	0	0
SAL_WA3156AA	MC_01-0221	Agona	0	0	0	0	0	0	0	0	0	0
SAL_WA3153AA	MC_01-0314	Agona	0	0	0	0	0	0	0	0	0	0
SAL_WA3074AA	SE09/42	Agona	0	0	0	0	0	0	0	0	0	0
SAL_WA3020AA	MC_03-0059	Agona	0	0	0	0	0	0	0	0	0	0
SAL_WA2970AA	MC_02-0097	Agona	0	0	0	0	0	0	0	0	0	0
SAL_WA2897AA	MC_02-1006	Agona	0	0	0	0	0	0	0	0	0	0
SAL_WA2855AA	MC_01-0764	Agona	0	0	0	0	0	0	0	0	0	0
SAL_WA2777AA	JE_S09-000988	Agona	0	0	0	0	0	0	0	0	0	0
SAL_WA2772AA	MC_01-0398	Agona	0	0	0	0	0	0	0	0	0	0
SAL_WA2771AA	MC_01-0364	Agona	0	0	0	0	0	0	0	0	0	0
SAL_WA2769AA	MC_01-0315	Agona	0	0	0	0	0	0	0	0	0	0
SAL_WA2765AA	MC_01-0399	Agona	0	0	0	0	0	0	0	0	0	0
SAL_WA2718AA	1085/05	Agona	0	0	0	0	0	0	0	0	0	0
SAL_WA2631AA	MC_03-0201	Agona	0	0	0	0	0	0	0	0	0	0
SAL_WA2596AA	MC_01-0313	Agona	0	0	0	0	0	0	0	0	0	0
SAL_WA2556AA	SM-DU-2007-09-003363	Agona	0	0	0	0	0	0	0	0	0	0
SAL_WA2557AA	SM-DU-2007-09-000456	Agona	0	0	0	0	0	0	0	0	0	0
SAL_WA2555AA	SM-DU-2007-08-003266	Agona	0	0	0	0	0	0	0	0	0	0
SAL_WA2554AA	SM-DU-2007-08-005186	Agona	0	0	0	0	0	0	0	0	0	0
SAL_WA2542AA	MC_00-3799	Agona	0	0	0	0	0	0	0	0	0	0
SAL_WA2566AA	MC_00-3798	Agona	0	0	0	0	0	0	0	0	0	0
SAL_WA2472AA	MC_01-0727	Agona	0	0	0	0	0	0	0	0	0	0
SAL_WA2466AA	MC_01-0421	Agona	0	0	0	0	0	0	0	0	0	0
SAL_WA2463AA	MC_01-0694	Agona	0	0	0	0	0	0	0	0	0	0
SAL_WA2467AA	MC_01-0521	Agona	0	0	0	0	0	0	0	0	0	0
SAL_WA2461AA	MC_02-0875	Agona	0	0	0	0	0	0	0	0	0	0
SAL_WA2453AA	MC_03-0139	Agona	0	0	0	0	0	0	0	0	0	0
SAL_WA2465AA	MC_01-0520	Agona	0	0	0	0	0	0	0	0	0	0
SAL_WA2412AA	MC_01-0312	Agona	0	0	0	0	0	0	0	0	0	0
SAL_WA2393AA	JM_06/52	Agona	0	0	0	0	0	0	0	0	0	0
SAL_WA2350AA	MC_01-0209	Agona	0	0	0	0	0	0	0	0	0	0
SAL_WA2344AA	MC_01-0137	Agona	0	0	0	0	0	0	0	0	0	0
SAL_WA2348AA	MC_01-0208	Agona	0	0	0	0	0	0	0	0	0	0
SAL_WA2349AA	MC_01-0153	Agona	0	0	0	0	0	0	0	0	0	0
SAL_WA2333AA	MC_01-0103	Agona	0	0	0	0	0	0	0	0	0	0
SAL_WA2319AA	MC_01-0087	Agona	0	0	0	0	0	0	0	0	0	0
SAL_WA2317AA	MC_03-0522	Agona	0	0	0	0	0	0	0	0	0	0
SAL_WA2316AA	MC_03-0559	Agona	0	0	0	0	0	0	0	0	0	0
SAL_WA2312AA	MC_03-0561	Agona	0	0	0	0	0	0	0	0	0	0
SAL_WA2301AA	MC_00-3728	Agona	0	0	0	0	0	0	0	0	0	0
SAL_WA2300AA	MC_00-3777	Agona	0	0	0	0	0	0	0	0	0	0
SAL_WA2298AA	MC_00-3780	Agona	0	0	0	0	0	0	0	0	0	0
SAL_WA2299AA	MC_00-3778	Agona	0	0	0	0	0	0	0	0	0	0
SAL_WA2302AA	1110/05	Agona	0	0	0	0	0	0	0	0	0	0
SAL_WA2296AA	MC_00-3781	Agona	0	0	0	0	0	0	0	0	0	0
SAL_WA2293AA	MC_02-0159	Agona	0	0	0	0	0	0	0	0	0	0

SAL_WA2279AA	JE_S09-001003	Agona	0	0	0	0	0	0	0	0	0	0	0
SAL_WA2282AA	MC_03-1099	Agona	0	0	0	0	0	0	0	0	0	0	0
SAL_WA2272AA	1051/05	Agona	0	0	0	0	0	0	0	0	0	0	0
SAL_WA2271AA	986/05	Agona	0	0	0	0	0	0	0	0	0	0	0
SAL_WA2314AA	MC_03-0560	Agona	0	0	0	0	0	0	0	0	0	0	0
SAL_WA2268AA	911/05	Agona	0	0	0	0	0	0	0	0	0	0	0
SAL_WA2265AA	910/05	Agona	0	0	0	0	0	0	0	0	0	0	0
SAL_WA1309AA	699797	Agona	0	0	0	0	0	0	0	0	0	0	0
SAL_WA1249AA	130363	Agona	0	0	0	0	0	0	0	0	0	0	0
SAL_WA1185AA	130399	Agona	0	0	0	0	0	0	0	0	0	0	0
SAL_VA3638AA	not applicale	Agona	0	0	0	0	0	0	0	0	0	0	0
SAL_VA3639AA	not applicale	Agona	0	0	0	0	0	0	0	0	0	0	0
SAL_VA3637AA	not applicale	Agona	0	0	0	0	0	0	0	0	0	0	0
SAL_VA3641AA	not applicale	Agona	0	0	0	0	0	0	0	0	0	0	0
SAL_VA3650AA	not applicale	Agona	0	0	0	0	0	0	0	0	0	0	0
SAL_VA3651AA	not applicale	Agona	0	0	0	0	0	0	0	0	0	0	0
SAL_VA3642AA	not applicale	Agona	0	0	0	0	0	0	0	0	0	0	0
SAL_VA3640AA	not applicale	Agona	0	0	0	0	0	0	0	0	0	0	0
SAL_WA2178AA	MC_02-0644	Agona	0	0	0	0	0	0	0	0	0	0	0
SAL_WA1836AA	HIY0194	Agona	0	0	0	0	0	0	0	0	0	0	0
SAL_WA1837AA	HIY0193	Agona	0	0	0	0	0	0	0	0	0	0	0
SAL_WA1833AA	HIY0192	Agona	0	0	0	0	0	0	0	0	0	0	0
SAL_WA1832AA	HIY0191	Agona	0	0	0	0	0	0	0	0	0	0	0
SAL_WA1865AA	FSIS11918531	Agona	0	0	0	0	0	0	0	0	0	0	0
SAL_WA1850AA	HIY0195	Agona	0	0	0	0	0	0	0	0	0	0	0
SAL_WA1879AA	FSIS21923745	Agona	0	0	0	0	0	0	0	0	0	0	0
SAL_WA1946AA	FSIS11918975	Agona	0	0	0	0	0	0	0	0	0	0	0
SAL_WA1919AA	FSIS11919079	Agona	0	0	0	0	0	0	0	0	0	0	0
SAL_WA2149AA	FSIS21923694	Agona	0	0	0	0	0	0	0	0	0	0	0
SAL_WA3382AA	SMG-19-400	Agona	0	0	0	0	0	0	0	0	0	0	0
SAL_WA3378AA	SMG-19-395	Agona	0	0	0	0	0	0	0	0	0	0	0
SAL_WA1381AA	SAL-18-VL-SD-MN-0033	Agona	0	0	0	0	0	0	0	0	0	0	0
SAL_VA5696AA	SA20112117	Agona	0	0	0	0	0	0	0	0	0	0	0
SAL_VA4718AA	Agona ATCC-BAA-707	Agona	0	0	0	0	0	0	0	0	0	0	0
SAL_VA8089AA	FSIS31901600	Agona	0	0	0	0	0	0	0	0	0	0	0
SAL_VA3649AA	not applicale	Agona	0	0	0	0	0	0	0	0	0	0	0
SAL_WA3342AA	705251	Agona	0	0	0	0	0	0	0	0	0	0	0
SAL_WA3499AA	CFSAN087441	Agona	0	0	0	0	0	0	0	0	0	0	0
SAL_WA3512AA	FSIS21923742	Agona	0	0	0	0	0	0	0	0	0	0	0
SAL_WA3485AA	CFSAN084248	Agona	0	0	0	0	0	0	0	0	0	0	0
SAL_WA3487AA	CFSAN087465	Agona	0	0	0	0	0	0	0	0	0	0	0
SAL_WA3484AA	CFSAN084247	Agona	0	0	0	0	0	0	0	0	0	0	0
SAL_WA3709AA	HIY0196	Agona	0	0	0	0	0	0	0	0	0	0	0
SAL_WA3670AA	HIY0197	Agona	0	0	0	0	0	0	0	0	0	0	0
SAL_WA3681AA	CFSAN084254	Agona	0	0	0	0	0	0	0	0	0	0	0
SAL_WA3682AA	CFSAN084255	Agona	0	0	0	0	0	0	0	0	0	0	0
SAL_WA3689AA	CFSAN087458	Agona	0	0	0	0	0	0	0	0	0	0	0
SAL_WA3649AA	CFSAN087488	Agona	0	0	0	0	0	0	0	0	0	0	0
SAL_WA3595AA	CFSAN087470	Agona	0	0	0	0	0	0	0	0	0	0	0
SAL_WA3571AA	CFSAN087575	Agona	0	0	0	0	0	0	0	0	0	0	0
SAL_WA3578AA	CFSAN087602	Agona	0	0	0	0	0	0	0	0	0	0	0
SAL_WA3584AA	CFSAN087576	Agona	0	0	0	0	0	0	0	0	0	0	0
SAL_WA3590AA	CFSAN087471	Agona	0	0	0	0	0	0	0	0	0	0	0
SAL_WA3588AA	CFSAN087560	Agona	0	0	0	0	0	0	0	0	0	0	0
SAL_WA3743AA	CFSAN087577	Agona	0	0	0	0	0	0	0	0	0	0	0
SAL_WA3733AA	AG19-0083	Agona	0	0	0	0	0	0	0	0	0	0	0
SAL_WA3725AA	CFSAN087594	Agona	0	0	0	0	0	0	0	0	0	0	0
SAL_WA3726AA	CFSAN087593	Agona	0	0	0	0	0	0	0	0	0	0	0
SAL_WA3694AA	HIY0198	Agona	0	0	0	0	0	0	0	0	0	0	0
SAL_WA3525AA	CFSAN087585	Agona	0	0	0	0	0	0	0	0	0	0	0
SAL_WA3529AA	CFSAN087466	Agona	0	0	0	0	0	0	0	0	0	0	0
SAL_WA3530AA	CFSAN087504	Agona	0	0	0	0	0	0	0	0	0	0	0
SAL_WA3535AA	CFSAN087521	Agona	0	0	0	0	0	0	0	0	0	0	0
SAL_WA3559AA	CFSAN087505	Agona	0	0	0	0	0	0	0	0	0	0	0
SAL_WA3783AA	FSIS31901679	Agona	0	0	0	0	0	0	0	0	0	0	0
SAL_WA3941AA	702943	Agona	0	0	0	0	0	0	0	0	0	0	0
SAL_WA4029AA	FSIS11918535	Agona	0	0	0	0	0	0	0	0	0	0	0
SAL_WA3692AA	CFSAN087459	Agona	0	0	0	0	0	0	0	0	0	0	0
SAL_WA4004AA	FSIS11919210	Agona	0	0	0	0	0	0	0	0	0	0	0
SAL_WA4227AA	201901573	Agona	0	0	0	0	0	0	0	0	0	0	0
SAL_WA4229AA	201901608	Agona	0	0	0	0	0	0	0	0	0	0	0
SAL_WA4225AA	201901658	Agona	0	0	0	0	0	0	0	0	0	0	0
SAL_EA0600AA	FSW0049	Agoueve	0	0	0	0	0	0	0	0	0	0	0

SAL_EA0568AA	FSW0056	Agoueve	0	0	0	0	0	0	0	0	0	0	0
SAL_DA5566AA	IEH-NGS-SAL-00856	Agoueve	0	0	0	0	0	0	0	0	0	0	0
SAL_LA7471AA	PNUSAS022011	Agoueve	0	0	0	0	0	0	0	0	0	0	0
SAL_MA5029AA	FSIS1702652	Agoueve	0	0	0	0	0	0	0	0	0	0	0
SAL_MA5021AA	FSIS1702643	Agoueve	0	0	0	0	0	0	0	0	0	0	0
SAL_PA1715AA	FSIS11809059	Agoueve	0	0	0	0	0	0	0	0	0	0	0
SAL_PA6964AA	PNUSAS040618	Agoueve	0	0	0	0	0	0	0	0	0	0	0
SAL_QA4415AA	PNUSAS042759	Agoueve	0	0	0	0	0	0	0	0	0	0	0
SAL_VA7402AA	201701191	Agoueve	0	0	0	0	0	0	0	0	0	0	0
SAL_VA7401AA	201701190	Agoueve	0	0	0	0	0	0	0	0	0	0	0
SAL_VA7564AA	201706513	Agoueve	0	0	0	0	0	0	0	0	0	0	0
SAL_VA7629AA	201707828	Agoueve	0	0	0	0	0	0	0	0	0	0	0
SAL_VA5855AA	SA20104097	Agoueve	0	0	0	0	0	0	0	0	0	0	0
SAL_VA5724AA	SA20081223	Agoueve	0	0	0	0	0	0	0	0	0	0	0
SAL_VA5704AA	SA20102405	Agoueve	0	0	0	0	0	0	0	0	0	0	0
SAL_VA6937AA	381323	Agoueve	0	0	0	0	0	0	0	0	0	0	0
SAL_VA7180AA	683605	Agoueve	0	0	0	0	0	0	0	0	0	0	0
SAL_BA5280AA	sc-2012-11-09T12:23:34Z	Ahuza	0	0	0	0	0	0	0	0	0	0	0
SAL_WA0312AA	177432	Ahuza	0	0	0	0	0	0	0	0	0	0	0
SAL_WA3183AA	GW_S01608-07	Ahuza	0	0	0	0	0	0	0	0	0	0	0
SAL_MA0394AA	PNUSAS024166	Alabama	0	0	0	0	0	0	0	0	0	0	0
SAL_HA8880AA	NCTC9868	Alabama	0	0	0	0	0	0	0	0	0	0	0
SAL_OA3735AA	MOD1-7992-RedHawk	Alabama	0	0	0	0	0	0	0	0	0	0	0
SAL_EA4887AA	CFSAN034754	Alachua	0	0	0	0	0	0	0	0	0	0	0
SAL_FA5307AA	FDA00010082	Alachua	0	0	0	0	0	0	0	0	0	0	0
SAL_FA5195AA	FDA00001638	Alachua	0	0	0	0	0	0	0	0	0	0	0
SAL_FA5143AA	CFSAN032033	Alachua	0	0	0	0	0	0	0	0	0	0	0
SAL_FA5106AA	CFSAN032029	Alachua	0	0	0	0	0	0	0	0	0	0	0
SAL_FA5111AA	CFSAN032026	Alachua	0	0	0	0	0	0	0	0	0	0	0
SAL_FA3026AA	CVM N58002	Alachua	0	0	0	0	0	0	0	0	0	0	0
SAL_FA2558AA	CVM-N19786	Alachua	0	0	0	0	0	0	0	0	0	0	0
SAL_FA2559AA	CVM-N19785	Alachua	0	0	0	0	0	0	0	0	0	0	0
SAL_EA8998AA	WAPHL-SAL-A01035	Alachua	0	0	0	0	0	0	0	0	0	0	0
SAL_EA5565AA	VA-WGS-00448	Alachua	0	0	0	0	0	0	0	0	0	0	0
SAL_EA1476AA	FSIS1500650	Alachua	0	0	0	0	0	0	0	0	0	0	0
SAL_EA1347AA	AZ_TG68284	Alachua	0	0	0	0	0	0	0	0	0	0	0
SAL_EA0476AA	FCC0042	Alachua	0	0	0	0	0	0	0	0	0	0	0
SAL_DA5165AA	FSF0156	Alachua	0	0	0	0	0	0	0	0	0	0	0
SAL_DA2618AA	CFSAN024036	Alachua	0	0	0	0	0	0	0	0	0	0	0
SAL_DA0325AA	AZ-TG74272	Alachua	0	0	0	0	0	0	0	0	0	0	0
SAL_DA0298AA	AZ-TG74268	Alachua	0	0	0	0	0	0	0	0	0	0	0
SAL_CA8497AA	CFSAN028526	Alachua	0	0	0	0	0	0	0	0	0	0	0
SAL_CA2791AA	3905	Alachua	0	0	0	0	0	0	0	0	0	0	0
SAL_BA9861AA	65408	Alachua	0	0	0	0	0	0	0	0	0	0	0
SAL_BA7836AA	5184-sc-2010-09-29-985	Alachua	0	0	0	0	0	0	0	0	0	0	0
SAL_BA7821AA	0564-sc-2010-09-29-985	Alachua	0	0	0	0	0	0	0	0	0	0	0
SAL_BA5479AA	FDA00002556	Alachua	0	0	0	0	0	0	0	0	0	0	0
SAL_BA5455AA	FDA00002555	Alachua	0	0	0	0	0	0	0	0	0	0	0
SAL_BA3114AA	FSIS1501010	Alachua	0	0	0	0	0	0	0	0	0	0	0
SAL_FA7670AA	FDA00003718	Alachua	0	0	0	0	0	0	0	0	0	0	0
SAL_FA7669AA	FDA00003717	Alachua	0	0	0	0	0	0	0	0	0	0	0
SAL_FA7668AA	FDA00003715	Alachua	0	0	0	0	0	0	0	0	0	0	0
SAL_FA7667AA	FDA00003714	Alachua	0	0	0	0	0	0	0	0	0	0	0
SAL_FA7666AA	FDA00003713	Alachua	0	0	0	0	0	0	0	0	0	0	0
SAL_FA7777AA	FDA00003716	Alachua	0	0	0	0	0	0	0	0	0	0	0
SAL_FA7775AA	FDA00003708	Alachua	0	0	0	0	0	0	0	0	0	0	0
SAL_FA8175AA	PNUSAS002227	Alachua	0	0	0	0	0	0	0	0	0	0	0
SAL_FA8240AA	FSIS1606920	Alachua	0	0	0	0	0	0	0	0	0	0	0
SAL_GA2821AA	CRJJGF_00090	Alachua	0	0	0	0	0	0	0	0	0	0	0
SAL_GA2820AA	CRJJGF_00091	Alachua	0	0	0	0	0	0	0	0	0	0	0
SAL_GA3695AA	BCW_2186	Alachua	0	0	0	0	0	0	0	0	0	0	0
SAL_GA3267AA	BCW_2043	Alachua	0	0	0	0	0	0	0	0	0	0	0
SAL_GA2563AA	08-1229	Alachua	0	0	0	0	0	0	0	0	0	0	0
SAL_GA2558AA	2010K-0732	Alachua	0	0	0	0	0	0	0	0	0	0	0
SAL_GA2542AA	04-0301	Alachua	0	0	0	0	0	0	0	0	0	0	0
SAL_GA2536AA	85-0191	Alachua	0	0	0	0	0	0	0	0	0	0	0
SAL_GA4489AA	CFSAN051822	Alachua	0	0	0	0	0	0	0	0	0	0	0
SAL_GA2222AA	BCW_6089	Alachua	0	0	0	0	0	0	0	0	0	0	0
SAL_GA4498AA	FDA00001394	Alachua	0	0	0	0	0	0	0	0	0	0	0
SAL_GA4490AA	CFSAN051821	Alachua	0	0	0	0	0	0	0	0	0	0	0
SAL_GA3835AA	BCW_1608	Alachua	0	0	0	0	0	0	0	0	0	0	0
SAL_GA3722AA	BCW_2034	Alachua	0	0	0	0	0	0	0	0	0	0	0
SAL_FA9442AA	CVM-N26635	Alachua	0	0	0	0	0	0	0	0	0	0	0

SAL_FA9443AA	CVM-N26634	Alachua	0	0	0	0	0	0	0	0	0	0
SAL_FA8690AA	CVM N31388	Alachua	0	0	0	0	0	0	0	0	0	0
SAL_GA5953AA	FSIS1607103	Alachua	0	0	0	0	0	0	0	0	0	0
SAL_HA1279AA	FSIS1607971	Alachua	0	0	0	0	0	0	0	0	0	0
SAL_HA3954AA	FSIS1608369	Alachua	0	0	0	0	0	0	0	0	0	0
SAL_HA4721AA	FSIS1608445	Alachua	0	0	0	0	0	0	0	0	0	0
SAL_IA2011AA	FSIS1609673	Alachua	0	0	0	0	0	0	0	0	0	0
SAL_IA3689AA	FSIS1609403	Alachua	0	0	0	0	0	0	0	0	0	0
SAL_IA5345AA	FSIS1710225	Alachua	0	0	0	0	0	0	0	0	0	0
SAL_IA6288AA	PNUSAS004342	Alachua	0	0	0	0	0	0	0	0	0	0
SAL_IA6996AA	PNUSAS008657	Alachua	0	0	0	0	0	0	0	0	0	0
SAL_IA9745AA	FSIS1710922	Alachua	0	0	0	0	0	0	0	0	0	0
SAL_JA3908AA	PNUSAS011885	Alachua	0	0	0	0	0	0	0	0	0	0
SAL_JA8223AA	365877	Alachua	0	0	0	0	0	0	0	0	0	0
SAL_JA9102AA	PNUSAS014734	Alachua	0	0	0	0	0	0	0	0	0	0
SAL_KA0164AA	OH-16-21366-2	Alachua	0	0	0	0	0	0	0	0	0	0
SAL_KA1275AA	PNUSAS012042	Alachua	0	0	0	0	0	0	0	0	0	0
SAL_KA2403AA	FSIS1702220	Alachua	0	0	0	0	0	0	0	0	0	0
SAL_KA3435AA	PNUSAS017497	Alachua	0	0	0	0	0	0	0	0	0	0
SAL_KA7144AA	FSIS1703411	Alachua	0	0	0	0	0	0	0	0	0	0
SAL_KA7158AA	FSIS1703192	Alachua	0	0	0	0	0	0	0	0	0	0
SAL_KA7405AA	PNUSAS015952	Alachua	0	0	0	0	0	0	0	0	0	0
SAL_KA7996AA	FSIS1703486	Alachua	0	0	0	0	0	0	0	0	0	0
SAL_LA9551AA	FSIS11704567	Alachua	0	0	0	0	0	0	0	0	0	0
SAL_LA9564AA	FDA00000888	Alachua	0	0	0	0	0	0	0	0	0	0
SAL_MA1240AA	CFSAN068434	Alachua	0	0	0	0	0	0	0	0	0	0
SAL_MA1216AA	CFSAN068433	Alachua	0	0	0	0	0	0	0	0	0	0
SAL_MA1257AA	34950	Alachua	0	0	0	0	0	0	0	0	0	0
SAL_MA3008AA	34940	Alachua	0	0	0	0	0	0	0	0	0	0
SAL_MA2994AA	34946	Alachua	0	0	0	0	0	0	0	0	0	0
SAL_MA2969AA	34922	Alachua	0	0	0	0	0	0	0	0	0	0
SAL_MA2869AA	PNUSAS024152	Alachua	0	0	0	0	0	0	0	0	0	0
SAL_MA2838AA	PNUSAS026348	Alachua	0	0	0	0	0	0	0	0	0	0
SAL_MA3134AA	D14-056775	Alachua	0	0	0	0	0	0	0	0	0	0
SAL_MA3139AA	D14-056774	Alachua	0	0	0	0	0	0	0	0	0	0
SAL_MA4808AA	FSIS11704577	Alachua	0	0	0	0	0	0	0	0	0	0
SAL_BA4369AA	FDA00004639	Alachua	0	0	0	0	0	0	0	0	0	0
SAL_DA3970AA	CFSAN023183	Alachua	0	0	0	0	0	0	0	0	0	0
SAL_HA8874AA	NCTC8261	Alachua	0	0	0	0	0	0	0	0	0	0
SAL_FA5311AA	FDA00010078	Alachua	0	0	0	0	0	0	0	0	0	0
SAL_JA6818AA	PNUSAS013278	Alachua	0	0	0	0	0	0	0	0	0	0
SAL_MA6459AA	FSIS11704886	Alachua	0	0	0	0	0	0	0	0	0	0
SAL_NA0275AA	PNUSAS028696	Alachua	0	0	0	0	0	0	0	0	0	0
SAL_MA9716AA	CVM N16S319	Alachua	0	0	0	0	0	0	0	0	0	0
SAL_NA0987AA	FDA00012400	Alachua	0	0	0	0	0	0	0	0	0	0
SAL_NA2602AA	CFSAN068435	Alachua	0	0	0	0	0	0	0	0	0	0
SAL_NA2607AA	CFSAN068432	Alachua	0	0	0	0	0	0	0	0	0	0
SAL_NA2823AA	PNUSAS030067	Alachua	0	0	0	0	0	0	0	0	0	0
SAL_NA6972AA	PNUSAS029605	Alachua	0	0	0	0	0	0	0	0	0	0
SAL_OA7415AA	PNUSAS036851	Alachua	0	0	0	0	0	0	0	0	0	0
SAL_OA8096AA	FSIS11808629	Alachua	0	0	0	0	0	0	0	0	0	0
SAL_QA3627AA	PNUSAS043183	Alachua	0	0	0	0	0	0	0	0	0	0
SAL_QA5163AA	490269	Alachua	0	0	0	0	0	0	0	0	0	0
SAL_QA5826AA	200660	Alachua	0	0	0	0	0	0	0	0	0	0
SAL_QA6068AA	PNUSAS042929	Alachua	0	0	0	0	0	0	0	0	0	0
SAL_QA6420AA	383965	Alachua	0	0	0	0	0	0	0	0	0	0
SAL_QA7607AA	404313	Alachua	0	0	0	0	0	0	0	0	0	0
SAL_RA1247AA	FSIS11811259	Alachua	0	0	0	0	0	0	0	0	0	0
SAL_RA3219AA	393913	Alachua	0	0	0	0	0	0	0	0	0	0
SAL_RA6406AA	PNUSAS046023	Alachua	0	0	0	0	0	0	0	0	0	0
SAL_RA9985AA	FSIS11812699	Alachua	0	0	0	0	0	0	0	0	0	0
SAL_SA1125AA	FSIS11812423	Alachua	0	0	0	0	0	0	0	0	0	0
SAL_SA3650AA	FSIS11813669	Alachua	0	0	0	0	0	0	0	0	0	0
SAL_SA4058AA	FSIS21822068	Alachua	0	0	0	0	0	0	0	0	0	0
SAL_SA7295AA	VA-WGS-18085	Alachua	0	0	0	0	0	0	0	0	0	0
SAL_TA0944AA	617845	Alachua	0	0	0	0	0	0	0	0	0	0
SAL_TA1407AA	FSIS21822250	Alachua	0	0	0	0	0	0	0	0	0	0
SAL_TA7837AA	samples of swine - serot	Alachua	0	0	0	0	0	0	0	0	0	0
SAL_TA8787AA	FSIS11815747	Alachua	0	0	0	0	0	0	0	0	0	0
SAL_QA7969AA	PNUSAS043970	Alachua	0	0	0	0	0	0	0	0	0	0
SAL_PA2148AA	FSIS11808937	Alachua	0	0	0	0	0	0	0	0	0	0
SAL_UA6870AA	CFSAN089904	Alachua	0	0	0	0	0	0	0	0	0	0
SAL_VA0302AA	FSIS31901499	Alachua	0	0	0	0	0	0	0	0	0	0

SAL_VA1324AA	488726	Alachua	0	0	0	0	0	0	0	0	0	0
SAL_VA3100AA	409971	Alachua	0	0	0	0	0	0	0	0	0	0
SAL_VA5800AA	SA20112589	Alachua	0	0	0	0	0	0	0	0	0	0
SAL_VA8351AA	FSIS31901618	Alachua	0	0	0	0	0	0	0	0	0	0
SAL_VA9029AA	299424	Alachua	0	0	0	0	0	0	0	0	0	0
SAL_WA0869AA	697902	Alachua	0	0	0	0	0	0	0	0	0	0
SAL_WA1667AA	128225	Alachua	0	0	0	0	0	0	0	0	0	0
SAL_WA2020AA	FDA00014062	Alachua	0	0	0	0	0	0	0	0	0	0
SAL_WA1944AA	FSIS11918969	Alachua	0	0	0	0	0	0	0	0	0	0
SAL_DA4250AA	FDN0193	Alamo	0	0	0	0	0	0	0	0	0	0
SAL_FA1873AA	FDA00000044	Alamo	0	0	0	0	0	0	0	0	0	0
SAL_NA5889AA	PNUSAS031953	Alamo	0	0	0	0	0	0	0	0	0	0
SAL_EA1884AA	CVM N55395	Albany	0	0	0	0	0	0	0	0	0	0
SAL_FA4802AA	CVM-N27365	Albany	0	0	0	0	0	0	0	0	0	0
SAL_FA3055AA	CVM N57539	Albany	0	0	0	0	0	0	0	0	0	0
SAL_FA2768AA	CVM N57660F	Albany	0	0	0	0	0	0	0	0	0	0
SAL_FA2740AA	CVM N57733F	Albany	0	0	0	0	0	0	0	0	0	0
SAL_FA2487AA	IEH-NGS-SAL-03789	Albany	0	0	0	0	0	0	0	0	0	0
SAL_FA2384AA	CFSAN047060	Albany	0	0	0	0	0	0	0	0	0	0
SAL_FA1320AA	WAPHL-SAL-A01042	Albany	0	0	0	0	0	0	0	0	0	0
SAL_FA1105AA	PNUSAS001521	Albany	0	0	0	0	0	0	0	0	0	0
SAL_FA0864AA	CVM-N26076	Albany	0	0	0	0	0	0	0	0	0	0
SAL_FA0629AA	NY-N20104	Albany	0	0	0	0	0	0	0	0	0	0
SAL_FA0611AA	CVM-N23668	Albany	0	0	0	0	0	0	0	0	0	0
SAL_EA9273AA	NY-N20024	Albany	0	0	0	0	0	0	0	0	0	0
SAL_EA9228AA	FDA00009332	Albany	0	0	0	0	0	0	0	0	0	0
SAL_EA9227AA	FDA00009333	Albany	0	0	0	0	0	0	0	0	0	0
SAL_EA8842AA	CVM-N23793	Albany	0	0	0	0	0	0	0	0	0	0
SAL_EA8755AA	CVM-N23832	Albany	0	0	0	0	0	0	0	0	0	0
SAL_EA8746AA	WAPHL-SAL-A01004	Albany	0	0	0	0	0	0	0	0	0	0
SAL_EA8172AA	CVM-N23742	Albany	0	0	0	0	0	0	0	0	0	0
SAL_EA8124AA	NY-N19899	Albany	0	0	0	0	0	0	0	0	0	0
SAL_EA7997AA	15205	Albany	0	0	0	0	0	0	0	0	0	0
SAL_EA7221AA	53048	Albany	0	0	0	0	0	0	0	0	0	0
SAL_EA6967AA	81994	Albany	0	0	0	0	0	0	0	0	0	0
SAL_EA6748AA	VA-WGS-00488	Albany	0	0	0	0	0	0	0	0	0	0
SAL_EA5802AA	VA-WGS-00460	Albany	0	0	0	0	0	0	0	0	0	0
SAL_EA5801AA	VA-WGS-00461	Albany	0	0	0	0	0	0	0	0	0	0
SAL_EA5800AA	VA-WGS-00462	Albany	0	0	0	0	0	0	0	0	0	0
SAL_EA5744AA	FSIS1502868	Albany	0	0	0	0	0	0	0	0	0	0
SAL_EA4847AA	FDA00000511	Albany	0	0	0	0	0	0	0	0	0	0
SAL_EA3808AA	CVM N45398	Albany	0	0	0	0	0	0	0	0	0	0
SAL_EA3745AA	CVM N46827	Albany	0	0	0	0	0	0	0	0	0	0
SAL_EA3672AA	CVM N48692	Albany	0	0	0	0	0	0	0	0	0	0
SAL_EA3582AA	CVM N51278	Albany	0	0	0	0	0	0	0	0	0	0
SAL_EA2761AA	CVM-N15753	Albany	0	0	0	0	0	0	0	0	0	0
SAL_EA2707AA	FDA00000534	Albany	0	0	0	0	0	0	0	0	0	0
SAL_EA2556AA	CVM-N15237	Albany	0	0	0	0	0	0	0	0	0	0
SAL_EA2557AA	CVM-N15236	Albany	0	0	0	0	0	0	0	0	0	0
SAL_EA2470AA	FDA00009418	Albany	0	0	0	0	0	0	0	0	0	0
SAL_EA2136AA	CVM N52022	Albany	0	0	0	0	0	0	0	0	0	0
SAL_EA2058AA	CVM N53045	Albany	0	0	0	0	0	0	0	0	0	0
SAL_EA1972AA	CVM N54281	Albany	0	0	0	0	0	0	0	0	0	0
SAL_EA1960AA	CVM N54702	Albany	0	0	0	0	0	0	0	0	0	0
SAL_EA1892AA	CVM N55387	Albany	0	0	0	0	0	0	0	0	0	0
SAL_EA0765AA	MDH-2013-00196	Albany	0	0	0	0	0	0	0	0	0	0
SAL_EA0439AA	FSF0061	Albany	0	0	0	0	0	0	0	0	0	0
SAL_EA0417AA	FSF0053	Albany	0	0	0	0	0	0	0	0	0	0
SAL_DA9892AA	FNW19G20	Albany	0	0	0	0	0	0	0	0	0	0
SAL_DA9842AA	MDH-2013-00085	Albany	0	0	0	0	0	0	0	0	0	0
SAL_DA9807AA	FNW19G19	Albany	0	0	0	0	0	0	0	0	0	0
SAL_DA8226AA	2106-sc-2010-09-29-985	Albany	0	0	0	0	0	0	0	0	0	0
SAL_DA7417AA	VA_WGS-00145	Albany	0	0	0	0	0	0	0	0	0	0
SAL_DA7365AA	VA_WGS-00144	Albany	0	0	0	0	0	0	0	0	0	0
SAL_DA7358AA	FSW0189	Albany	0	0	0	0	0	0	0	0	0	0
SAL_DA7347AA	VA_WGS-00143	Albany	0	0	0	0	0	0	0	0	0	0
SAL_DA6227AA	FSE0157	Albany	0	0	0	0	0	0	0	0	0	0
SAL_FA6010AA	FSIS1606497	Albany	0	0	0	0	0	0	0	0	0	0
SAL_FA6009AA	FSIS1606498	Albany	0	0	0	0	0	0	0	0	0	0
SAL_FA6012AA	FSIS1606499	Albany	0	0	0	0	0	0	0	0	0	0
SAL_FA6011AA	FSIS1606495	Albany	0	0	0	0	0	0	0	0	0	0
SAL_DA5763AA	FCC0156	Albany	0	0	0	0	0	0	0	0	0	0
SAL_DA5254AA	FDA00000211	Albany	0	0	0	0	0	0	0	0	0	0

SAL_DA5057AA	FNE0035	Albany	0	0	0	0	0	0	0	0	0	0	0
SAL_DA4407AA	FNE0094	Albany	0	0	0	0	0	0	0	0	0	0	0
SAL_DA4323AA	MOD1-Sal_895	Albany	0	0	0	0	0	0	0	0	0	0	0
SAL_DA4309AA	MOD1-Sal_893	Albany	0	0	0	0	0	0	0	0	0	0	0
SAL_DA4308AA	MOD1-Sal_896	Albany	0	0	0	0	0	0	0	0	0	0	0
SAL_DA4292AA	MOD1-Sal_894	Albany	0	0	0	0	0	0	0	0	0	0	0
SAL_DA3946AA	FCC0211	Albany	0	0	0	0	0	0	0	0	0	0	0
SAL_DA1126AA	H124200190	Albany	0	0	0	0	0	0	0	0	0	0	0
SAL_DA0861AA	H132960589	Albany	0	0	0	0	0	0	0	0	0	0	0
SAL_DA0623AA	H122200419	Albany	0	0	0	0	0	0	0	0	0	0	0
SAL_CA8612AA	FDA00000922	Albany	0	0	0	0	0	0	0	0	0	0	0
SAL_CA8484AA	FL-NRM171	Albany	0	0	0	0	0	0	0	0	0	0	0
SAL_CA5748AA	78693	Albany	0	0	0	0	0	0	0	0	0	0	0
SAL_CA5521AA	79026	Albany	0	0	0	0	0	0	0	0	0	0	0
SAL_CA5487AA	71541	Albany	0	0	0	0	0	0	0	0	0	0	0
SAL_CA5062AA	69695	Albany	0	0	0	0	0	0	0	0	0	0	0
SAL_CA4190AA	40331	Albany	0	0	0	0	0	0	0	0	0	0	0
SAL_CA3879AA	38380	Albany	0	0	0	0	0	0	0	0	0	0	0
SAL_CA3769AA	13843	Albany	0	0	0	0	0	0	0	0	0	0	0
SAL_CA3590AA	38396	Albany	0	0	0	0	0	0	0	0	0	0	0
SAL_CA3229AA	31908	Albany	0	0	0	0	0	0	0	0	0	0	0
SAL_CA2667AA	9303	Albany	0	0	0	0	0	0	0	0	0	0	0
SAL_CA2559AA	25228	Albany	0	0	0	0	0	0	0	0	0	0	0
SAL_BA9202AA	69271	Albany	0	0	0	0	0	0	0	0	0	0	0
SAL_BA8929AA	37001	Albany	0	0	0	0	0	0	0	0	0	0	0
SAL_BA8805AA	NY_BAC0600009422	Albany	0	0	0	0	0	0	0	0	0	0	0
SAL_BA8407AA	26993	Albany	0	0	0	0	0	0	0	0	0	0	0
SAL_BA8352AA	38379	Albany	0	0	0	0	0	0	0	0	0	0	0
SAL_BA7760AA	13818	Albany	0	0	0	0	0	0	0	0	0	0	0
SAL_BA3737AA	FDA00004301	Albany	0	0	0	0	0	0	0	0	0	0	0
SAL_BA2983AA	AZ_TG68152	Albany	0	0	0	0	0	0	0	0	0	0	0
SAL_BA2430AA	WAPHL_SAL-A00826	Albany	0	0	0	0	0	0	0	0	0	0	0
SAL_AA7612AA	CG59/3	Albany	0	0	0	0	0	0	0	0	0	0	0
SAL_FA6158AA	FSIS1606496	Albany	0	0	0	0	0	0	0	0	0	0	0
SAL_FA6016AA	FSIS1501723	Albany	0	0	0	0	0	0	0	0	0	0	0
SAL_FA2367AA	IEH-NGS-SAL-03820	Albany	0	0	0	0	0	0	0	0	0	0	0
SAL_FA6793AA	FSIS1501921	Albany	0	0	0	0	0	0	0	0	0	0	0
SAL_FA7510AA	CVM-N28616	Albany	0	0	0	0	0	0	0	0	0	0	0
SAL_FA7430AA	ADRDL15-26750	Albany	0	0	0	0	0	0	0	0	0	0	0
SAL_FA7735AA	FDA00000120	Albany	0	0	0	0	0	0	0	0	0	0	0
SAL_FA7746AA	PNUSAS002236	Albany	0	0	0	0	0	0	0	0	0	0	0
SAL_GA2771AA	CRJJGF_00142	Albany	0	0	0	0	0	0	0	0	0	0	0
SAL_GA2603AA	06-0796	Albany	0	0	0	0	0	0	0	0	0	0	0
SAL_GA2600AA	08-0005	Albany	0	0	0	0	0	0	0	0	0	0	0
SAL_GA2592AA	95-0155	Albany	0	0	0	0	0	0	0	0	0	0	0
SAL_GA2571AA	74-2811	Albany	0	0	0	0	0	0	0	0	0	0	0
SAL_GA2572AA	70-0076	Albany	0	0	0	0	0	0	0	0	0	0	0
SAL_GA3894AA	BCW_2888	Albany	0	0	0	0	0	0	0	0	0	0	0
SAL_GA3709AA	BCW_2111	Albany	0	0	0	0	0	0	0	0	0	0	0
SAL_GA3404AA	BCW_2135	Albany	0	0	0	0	0	0	0	0	0	0	0
SAL_GA3362AA	BCW_2634	Albany	0	0	0	0	0	0	0	0	0	0	0
SAL_GA3274AA	BCW_2027	Albany	0	0	0	0	0	0	0	0	0	0	0
SAL_GA3224AA	BCW_2132	Albany	0	0	0	0	0	0	0	0	0	0	0
SAL_GA0367AA	VNS10317-sc-2280573	Albany	0	0	0	0	0	0	0	0	0	0	0
SAL_GA0366AA	VNS10338-sc-2280575	Albany	0	0	0	0	0	0	0	0	0	0	0
SAL_GA0353AA	VNSC2301-sc-2280606	Albany	0	0	0	0	0	0	0	0	0	0	0
SAL_GA0352AA	VNSC2303-sc-2280608	Albany	0	0	0	0	0	0	0	0	0	0	0
SAL_GA0342AA	'NS116_MTKT-sc-228062	Albany	0	0	0	0	0	0	0	0	0	0	0
SAL_GA0312AA	71_H_227-sc-2280693	Albany	0	0	0	0	0	0	0	0	0	0	0
SAL_GA0313AA	71_H_226-sc-2280690	Albany	0	0	0	0	0	0	0	0	0	0	0
SAL_GA0267AA	74_V_140-sc-2280753	Albany	0	0	0	0	0	0	0	0	0	0	0
SAL_GA0265AA	74_V_405-sc-2280756	Albany	0	0	0	0	0	0	0	0	0	0	0
SAL_GA0255AA	CG02C_1_1-sc-2280766	Albany	0	0	0	0	0	0	0	0	0	0	0
SAL_GA0229AA	MT11C_1_1-sc-2280803	Albany	0	0	0	0	0	0	0	0	0	0	0
SAL_GA0228AA	MT11C_2_1-sc-2280804	Albany	0	0	0	0	0	0	0	0	0	0	0
SAL_GA0227AA	MT11C_3_1-sc-2280806	Albany	0	0	0	0	0	0	0	0	0	0	0
SAL_GA0203AA	VNS30002-sc-2280594	Albany	0	0	0	0	0	0	0	0	0	0	0
SAL_GA0143AA	VNB842-sc-2280637	Albany	0	0	0	0	0	0	0	0	0	0	0
SAL_GA5151AA	FDA00010420	Albany	0	0	0	0	0	0	0	0	0	0	0
SAL_FA9264AA	CFSAN047012	Albany	0	0	0	0	0	0	0	0	0	0	0
SAL_FA9190AA	CVM N38860	Albany	0	0	0	0	0	0	0	0	0	0	0
SAL_GA5161AA	V-CLASP-D-59	Albany	0	0	0	0	0	0	0	0	0	0	0
SAL_GA5708AA	CVM N58640	Albany	0	0	0	0	0	0	0	0	0	0	0

SAL_GA5663AA	CVM N59323	Albany	0	0	0	0	0	0	0	0	0	0	0
SAL_GA5652AA	CVM N62445	Albany	0	0	0	0	0	0	0	0	0	0	0
SAL_FA8941AA	CVM N42330	Albany	0	0	0	0	0	0	0	0	0	0	0
SAL_FA8646AA	CVM N31839	Albany	0	0	0	0	0	0	0	0	0	0	0
SAL_FA8645AA	CVM N31840	Albany	0	0	0	0	0	0	0	0	0	0	0
SAL_GA8051AA	FDA00010524	Albany	0	0	0	0	0	0	0	0	0	0	0
SAL_GA9488AA	CVM N62953	Albany	0	0	0	0	0	0	0	0	0	0	0
SAL_GA9536AA	CVM N58684	Albany	0	0	0	0	0	0	0	0	0	0	0
SAL_HA2763AA	CFSAN048263	Albany	0	0	0	0	0	0	0	0	0	0	0
SAL_HA4322AA	CFSAN048478	Albany	0	0	0	0	0	0	0	0	0	0	0
SAL_HA4327AA	CFSAN048484	Albany	0	0	0	0	0	0	0	0	0	0	0
SAL_HA3402AA	wt5	Albany	0	0	0	0	0	0	0	0	0	0	0
SAL_HA3385AA	wb25	Albany	0	0	0	0	0	0	0	0	0	0	0
SAL_HA3383AA	wb23	Albany	0	0	0	0	0	0	0	0	0	0	0
SAL_HA3382AA	wb22	Albany	0	0	0	0	0	0	0	0	0	0	0
SAL_HA3384AA	wb24	Albany	0	0	0	0	0	0	0	0	0	0	0
SAL_HA5624AA	S05930-15	Albany	0	0	0	0	0	0	0	0	0	0	0
SAL_HA5626AA	S05596-15	Albany	0	0	0	0	0	0	0	0	0	0	0
SAL_GA7422AA	CVM-N23667	Albany	0	0	0	0	0	0	0	0	0	0	0
SAL_HA9727AA	CFSAN058825	Albany	0	0	0	0	0	0	0	0	0	0	0
SAL_IA0586AA	PNUSAS006236	Albany	0	0	0	0	0	0	0	0	0	0	0
SAL_IA1919AA	PNUSAS006542	Albany	0	0	0	0	0	0	0	0	0	0	0
SAL_IA1933AA	PNUSAS007288	Albany	0	0	0	0	0	0	0	0	0	0	0
SAL_IA2874AA	138404	Albany	0	0	0	0	0	0	0	0	0	0	0
SAL_IA2700AA	CFSAN059204	Albany	0	0	0	0	0	0	0	0	0	0	0
SAL_IA2701AA	CFSAN059206	Albany	0	0	0	0	0	0	0	0	0	0	0
SAL_IA5188AA	FSIS1709980	Albany	0	0	0	0	0	0	0	0	0	0	0
SAL_IA5517AA	CFSAN059780	Albany	0	0	0	0	0	0	0	0	0	0	0
SAL_IA6570AA	CFSAN058777	Albany	0	0	0	0	0	0	0	0	0	0	0
SAL_IA6578AA	CFSAN058778	Albany	0	0	0	0	0	0	0	0	0	0	0
SAL_IA7117AA	ADRDL-953	Albany	0	0	0	0	0	0	0	0	0	0	0
SAL_JA0172AA	PNUSAS009052	Albany	0	0	0	0	0	0	0	0	0	0	0
SAL_JA2035AA	PNUSAS008903	Albany	0	0	0	0	0	0	0	0	0	0	0
SAL_JA3294AA	PNUSAS011842	Albany	0	0	0	0	0	0	0	0	0	0	0
SAL_JA3230AA	PNUSAS011417	Albany	0	0	0	0	0	0	0	0	0	0	0
SAL_JA3032AA	OH-16-14814-4	Albany	0	0	0	0	0	0	0	0	0	0	0
SAL_JA3709AA	PNUSAS012986	Albany	0	0	0	0	0	0	0	0	0	0	0
SAL_JA4465AA	OH-16-15767-1	Albany	0	0	0	0	0	0	0	0	0	0	0
SAL_JA6562AA	351257	Albany	0	0	0	0	0	0	0	0	0	0	0
SAL_JA7500AA	FSIS1700585	Albany	0	0	0	0	0	0	0	0	0	0	0
SAL_KA0170AA	OH-16-23179-3	Albany	0	0	0	0	0	0	0	0	0	0	0
SAL_KA0161AA	OH-16-22632-1	Albany	0	0	0	0	0	0	0	0	0	0	0
SAL_KA0484AA	PNUSAS012970	Albany	0	0	0	0	0	0	0	0	0	0	0
SAL_KA2288AA	14-24-20170543	Albany	0	0	0	0	0	0	0	0	0	0	0
SAL_KA4315AA	ZJ-56	Albany	0	0	0	0	0	0	0	0	0	0	0
SAL_KA5046AA	FDA00003578	Albany	0	0	0	0	0	0	0	0	0	0	0
SAL_KA5052AA	FDA00003577	Albany	0	0	0	0	0	0	0	0	0	0	0
SAL_KA5099AA	16-15-20170576	Albany	0	0	0	0	0	0	0	0	0	0	0
SAL_KA7104AA	FSIS1703332	Albany	0	0	0	0	0	0	0	0	0	0	0
SAL_LA1145AA	CFSAN067838	Albany	0	0	0	0	0	0	0	0	0	0	0
SAL_LA3100AA	SQ08.010	Albany	0	0	0	0	0	0	0	0	0	0	0
SAL_LA3115AA	SA08.301	Albany	0	0	0	0	0	0	0	0	0	0	0
SAL_LA2341AA	FSIS21720098	Albany	0	0	0	0	0	0	0	0	0	0	0
SAL_LA4385AA	FSIS11704275	Albany	0	0	0	0	0	0	0	0	0	0	0
SAL_LA6147AA	CFSAN069145	Albany	0	0	0	0	0	0	0	0	0	0	0
SAL_LA6098AA	CFSAN069230	Albany	0	0	0	0	0	0	0	0	0	0	0
SAL_LA7325AA	CFSAN069278	Albany	0	0	0	0	0	0	0	0	0	0	0
SAL_LA9788AA	PNUSAS022871	Albany	0	0	0	0	0	0	0	0	0	0	0
SAL_LA9575AA	FSIS11704694	Albany	0	0	0	0	0	0	0	0	0	0	0
SAL_MA2678AA	PNUSAS026163	Albany	0	0	0	0	0	0	0	0	0	0	0
SAL_MA2988AA	CFSAN070484	Albany	0	0	0	0	0	0	0	0	0	0	0
SAL_MA3925AA	CFSAN070490	Albany	0	0	0	0	0	0	0	0	0	0	0
SAL_MA3929AA	CFSAN070489	Albany	0	0	0	0	0	0	0	0	0	0	0
SAL_DA8592AA	FSE0086	Albany	0	0	0	0	0	0	0	0	0	0	0
SAL_EA0235AA	FSW0085	Albany	0	0	0	0	0	0	0	0	0	0	0
SAL_EA9239AA	FDA00009331	Albany	0	0	0	0	0	0	0	0	0	0	0
SAL_HA1056AA	CFSAN048212	Albany	0	0	0	0	0	0	0	0	0	0	0
SAL_GA3203AA	N31839	Albany	0	0	0	0	0	0	0	0	0	0	0
SAL_GA0337AA	VNS188_NKY-sc-2280638	Albany	0	0	0	0	0	0	0	0	0	0	0
SAL_JA7985AA	336988	Albany	0	0	0	0	0	0	0	0	0	0	0
SAL_JA9209AA	FSIS1701316	Albany	0	0	0	0	0	0	0	0	0	0	0
SAL_KA0167AA	OH-16-21117-KS	Albany	0	0	0	0	0	0	0	0	0	0	0
SAL_KA5844AA	PNUSAS017675	Albany	0	0	0	0	0	0	0	0	0	0	0

SAL_LA0632AA	FSIS1703739	Albany	0	0	0	0	0	0	0	0	0	0	0
SAL_MA5727AA	CFSAN071200	Albany	0	0	0	0	0	0	0	0	0	0	0
SAL_BA5166AA	ATCC 51960	Albany	0	0	0	0	0	0	0	0	0	0	0
SAL_MA5745AA	CFSAN071224	Albany	0	0	0	0	0	0	0	0	0	0	0
SAL_MA5747AA	CFSAN071203	Albany	0	0	0	0	0	0	0	0	0	0	0
SAL_MA5752AA	CFSAN071208	Albany	0	0	0	0	0	0	0	0	0	0	0
SAL_MA5764AA	CFSAN071202	Albany	0	0	0	0	0	0	0	0	0	0	0
SAL_MA5765AA	CFSAN071206	Albany	0	0	0	0	0	0	0	0	0	0	0
SAL_MA7437AA	FSIS11705872	Albany	0	0	0	0	0	0	0	0	0	0	0
SAL_MA7843AA	FDA00012363	Albany	0	0	0	0	0	0	0	0	0	0	0
SAL_MA7953AA	CFSAN071822	Albany	0	0	0	0	0	0	0	0	0	0	0
SAL_MA7952AA	CFSAN071823	Albany	0	0	0	0	0	0	0	0	0	0	0
SAL_MA9099AA	CFSAN070548	Albany	0	0	0	0	0	0	0	0	0	0	0
SAL_MA8773AA	FSIS11705991	Albany	0	0	0	0	0	0	0	0	0	0	0
SAL_MA9089AA	CFSAN071838	Albany	0	0	0	0	0	0	0	0	0	0	0
SAL_MA9022AA	FSIS11705330	Albany	0	0	0	0	0	0	0	0	0	0	0
SAL_NA0404AA	CFSAN070558	Albany	0	0	0	0	0	0	0	0	0	0	0
SAL_NA0773AA	32-20-8101291	Albany	0	0	0	0	0	0	0	0	0	0	0
SAL_NA0172AA	CFSAN072127	Albany	0	0	0	0	0	0	0	0	0	0	0
SAL_MA9962AA	CVM N16S222	Albany	0	0	0	0	0	0	0	0	0	0	0
SAL_MA9847AA	CVM N16S121	Albany	0	0	0	0	0	0	0	0	0	0	0
SAL_NA0934AA	CFSAN071824	Albany	0	0	0	0	0	0	0	0	0	0	0
SAL_NA2364AA	CFSAN073469	Albany	0	0	0	0	0	0	0	0	0	0	0
SAL_NA2355AA	CFSAN073482	Albany	0	0	0	0	0	0	0	0	0	0	0
SAL_NA2356AA	CFSAN073558	Albany	0	0	0	0	0	0	0	0	0	0	0
SAL_NA3275AA	CFSAN073560	Albany	0	0	0	0	0	0	0	0	0	0	0
SAL_NA3297AA	FSIS11706650	Albany	0	0	0	0	0	0	0	0	0	0	0
SAL_NA3735AA	PNUSAS030419	Albany	0	0	0	0	0	0	0	0	0	0	0
SAL_NA6154AA	CFSAN074271	Albany	0	0	0	0	0	0	0	0	0	0	0
SAL_NA7524AA	CFSAN074819	Albany	0	0	0	0	0	0	0	0	0	0	0
SAL_NA7536AA	CFSAN074815	Albany	0	0	0	0	0	0	0	0	0	0	0
SAL_NA8456AA	PNUSAS032940	Albany	0	0	0	0	0	0	0	0	0	0	0
SAL_NA8959AA	CFSAN075270	Albany	0	0	0	0	0	0	0	0	0	0	0
SAL_NA9673AA	FSIS21821057	Albany	0	0	0	0	0	0	0	0	0	0	0
SAL_OA0531AA	FDA00012542	Albany	0	0	0	0	0	0	0	0	0	0	0
SAL_OA0745AA	UGBAR434	Albany	0	0	0	0	0	0	0	0	0	0	0
SAL_OA5365AA	PNUSAS028149	Albany	0	0	0	0	0	0	0	0	0	0	0
SAL_OA3805AA	CFSAN075635	Albany	0	0	0	0	0	0	0	0	0	0	0
SAL_OA3848AA	CFSAN076776	Albany	0	0	0	0	0	0	0	0	0	0	0
SAL_OA8019AA	CFSAN078038	Albany	0	0	0	0	0	0	0	0	0	0	0
SAL_OA8175AA	PNUSAS034465	Albany	0	0	0	0	0	0	0	0	0	0	0
SAL_PA2135AA	18EP000518	Albany	0	0	0	0	0	0	0	0	0	0	0
SAL_PA0507AA	CFSAN076782	Albany	0	0	0	0	0	0	0	0	0	0	0
SAL_PA0548AA	CFSAN076757	Albany	0	0	0	0	0	0	0	0	0	0	0
SAL_PA0637AA	CFSAN076765	Albany	0	0	0	0	0	0	0	0	0	0	0
SAL_PA0613AA	CFSAN076762	Albany	0	0	0	0	0	0	0	0	0	0	0
SAL_PA0600AA	CFSAN076766	Albany	0	0	0	0	0	0	0	0	0	0	0
SAL_PA1849AA	CFSAN078653	Albany	0	0	0	0	0	0	0	0	0	0	0
SAL_PA3796AA	CFSAN080021	Albany	0	0	0	0	0	0	0	0	0	0	0
SAL_PA3795AA	CFSAN080009	Albany	0	0	0	0	0	0	0	0	0	0	0
SAL_PA6551AA	159686	Albany	0	0	0	0	0	0	0	0	0	0	0
SAL_PA7241AA	FSIS11808501	Albany	0	0	0	0	0	0	0	0	0	0	0
SAL_PA7432AA	CFSAN080922	Albany	0	0	0	0	0	0	0	0	0	0	0
SAL_PA8024AA	238697	Albany	0	0	0	0	0	0	0	0	0	0	0
SAL_QA0658AA	201546	Albany	0	0	0	0	0	0	0	0	0	0	0
SAL_QA0637AA	CFSAN081786	Albany	0	0	0	0	0	0	0	0	0	0	0
SAL_QA0625AA	CFSAN081783	Albany	0	0	0	0	0	0	0	0	0	0	0
SAL_QA0624AA	CFSAN081777	Albany	0	0	0	0	0	0	0	0	0	0	0
SAL_QA0627AA	CFSAN081785	Albany	0	0	0	0	0	0	0	0	0	0	0
SAL_QA0622AA	CFSAN081778	Albany	0	0	0	0	0	0	0	0	0	0	0
SAL_QA0628AA	CFSAN081784	Albany	0	0	0	0	0	0	0	0	0	0	0
SAL_QA2495AA	401792	Albany	0	0	0	0	0	0	0	0	0	0	0
SAL_QA2394AA	172440	Albany	0	0	0	0	0	0	0	0	0	0	0
SAL_QA5176AA	238461	Albany	0	0	0	0	0	0	0	0	0	0	0
SAL_QA5624AA	PNUSAS043156	Albany	0	0	0	0	0	0	0	0	0	0	0
SAL_RA4744AA	172449	Albany	0	0	0	0	0	0	0	0	0	0	0
SAL_RA2915AA	500293	Albany	0	0	0	0	0	0	0	0	0	0	0
SAL_RA3390AA	449730	Albany	0	0	0	0	0	0	0	0	0	0	0
SAL_RA4452AA	FSIS11811514	Albany	0	0	0	0	0	0	0	0	0	0	0
SAL_RA4291AA	390074	Albany	0	0	0	0	0	0	0	0	0	0	0
SAL_RA5486AA	271090	Albany	0	0	0	0	0	0	0	0	0	0	0
SAL_RA6560AA	103730	Albany	0	0	0	0	0	0	0	0	0	0	0
SAL_RA6818AA	PNUSAS045899	Albany	0	0	0	0	0	0	0	0	0	0	0

SAL_RA7147AA	238462	Albany	0	0	0	0	0	0	0	0	0	0	0
SAL_RA8159AA	FSIS31800757	Albany	0	0	0	0	0	0	0	0	0	0	0
SAL_RA9275AA	CFSAN084092	Albany	0	0	0	0	0	0	0	0	0	0	0
SAL_RA9259AA	CFSAN084013	Albany	0	0	0	0	0	0	0	0	0	0	0
SAL_RA9224AA	CFSAN084106	Albany	0	0	0	0	0	0	0	0	0	0	0
SAL_RA9414AA	CFSAN084058	Albany	0	0	0	0	0	0	0	0	0	0	0
SAL_RA9751AA	CFSAN084173	Albany	0	0	0	0	0	0	0	0	0	0	0
SAL_SA0388AA		Albany	0	0	0	0	0	0	0	0	0	0	0
SAL_SA0373AA		Albany	0	0	0	0	0	0	0	0	0	0	0
SAL_RA9316AA	CFSAN084093	Albany	0	0	0	0	0	0	0	0	0	0	0
SAL_SA3601AA	FSIS21822039	Albany	0	0	0	0	0	0	0	0	0	0	0
SAL_SA5276AA	CK_94/00007681-1	Albany	0	0	0	0	0	0	0	0	0	0	0
SAL_SA4961AA	CIT_D2.22	Albany	0	0	0	0	0	0	0	0	0	0	0
SAL_SA4054AA	FSIS31800975	Albany	0	0	0	0	0	0	0	0	0	0	0
SAL_SA7435AA	577908	Albany	0	0	0	0	0	0	0	0	0	0	0
SAL_SA9272AA	CVM N17S808	Albany	0	0	0	0	0	0	0	0	0	0	0
SAL_SA9273AA	CVM N17S812	Albany	0	0	0	0	0	0	0	0	0	0	0
SAL_TA0480AA	608522	Albany	0	0	0	0	0	0	0	0	0	0	0
SAL_TA1962AA	AG03	Albany	0	0	0	0	0	0	0	0	0	0	0
SAL_TA6695AA	FSIS31801210	Albany	0	0	0	0	0	0	0	0	0	0	0
SAL_TA7731AA	R17.2828	Albany	0	0	0	0	0	0	0	0	0	0	0
SAL_TA7729AA	R17.2117	Albany	0	0	0	0	0	0	0	0	0	0	0
SAL_TA7712AA	R16.4194	Albany	0	0	0	0	0	0	0	0	0	0	0
SAL_TA7699AA	R17.5973	Albany	0	0	0	0	0	0	0	0	0	0	0
SAL_TA7682AA	R17.1799	Albany	0	0	0	0	0	0	0	0	0	0	0
SAL_TA7614AA	R16.3208	Albany	0	0	0	0	0	0	0	0	0	0	0
SAL_TA7583AA	R17.4301	Albany	0	0	0	0	0	0	0	0	0	0	0
SAL_TA7571AA	R16.0257	Albany	0	0	0	0	0	0	0	0	0	0	0
SAL_TA7542AA	R15.2725	Albany	0	0	0	0	0	0	0	0	0	0	0
SAL_TA7532AA	R15.2267	Albany	0	0	0	0	0	0	0	0	0	0	0
SAL_TA7531AA	R15.2199	Albany	0	0	0	0	0	0	0	0	0	0	0
SAL_OA1421AA	FSIS11807690	Albany	0	0	0	0	0	0	0	0	0	0	0
SAL_TA8601AA	OH-17-18870-2	Albany	0	0	0	0	0	0	0	0	0	0	0
SAL_RA6163AA	Avian	Albany	0	0	0	0	0	0	0	0	0	0	0
SAL_OA1833AA	ADRDL-1852	Albany	0	0	0	0	0	0	0	0	0	0	0
SAL_PA2420AA	CFSAN078649	Albany	0	0	0	0	0	0	0	0	0	0	0
SAL_PA3518AA	CFSAN078716	Albany	0	0	0	0	0	0	0	0	0	0	0
SAL_PA3530AA	CFSAN078713	Albany	0	0	0	0	0	0	0	0	0	0	0
SAL_PA3591AA	CFSAN075578	Albany	0	0	0	0	0	0	0	0	0	0	0
SAL_UA6903AA	CFSAN089926	Albany	0	0	0	0	0	0	0	0	0	0	0
SAL_UA6911AA	CFSAN089925	Albany	0	0	0	0	0	0	0	0	0	0	0
SAL_UA6322AA	FSIS11814505	Albany	0	0	0	0	0	0	0	0	0	0	0
SAL_UA7934AA	CFSAN090604	Albany	0	0	0	0	0	0	0	0	0	0	0
SAL_UA8355AA	NC_1605120165	Albany	0	0	0	0	0	0	0	0	0	0	0
SAL_VA0540AA	ADRDL-2242	Albany	0	0	0	0	0	0	0	0	0	0	0
SAL_VA0572AA	FSIS11917252	Albany	0	0	0	0	0	0	0	0	0	0	0
SAL_VA7611AA	201701987	Albany	0	0	0	0	0	0	0	0	0	0	0
SAL_VA3315AA	FSIS11917334	Albany	0	0	0	0	0	0	0	0	0	0	0
SAL_VA6745AA	FSIS21923484	Albany	0	0	0	0	0	0	0	0	0	0	0
SAL_VA2738AA	429709	Albany	0	0	0	0	0	0	0	0	0	0	0
SAL_OA9716AA	sg_wt5	Albany	0	0	0	0	0	0	0	0	0	0	0
SAL_VA5849AA	SA20032029	Albany	0	0	0	0	0	0	0	0	0	0	0
SAL_VA5752AA	SA20032098	Albany	0	0	0	0	0	0	0	0	0	0	0
SAL_VA5736AA	SA20031616	Albany	0	0	0	0	0	0	0	0	0	0	0
SAL_VA6964AA	FSIS31901547	Albany	0	0	0	0	0	0	0	0	0	0	0
SAL_VA7759AA	FSIS11917612	Albany	0	0	0	0	0	0	0	0	0	0	0
SAL_VA8076AA	NYVetLIRN-131	Albany	0	0	0	0	0	0	0	0	0	0	0
SAL_VA8753AA	314984	Albany	0	0	0	0	0	0	0	0	0	0	0
SAL_VA8730AA	346574	Albany	0	0	0	0	0	0	0	0	0	0	0
SAL_VA9245AA	CFSAN089909	Albany	0	0	0	0	0	0	0	0	0	0	0
SAL_WA0185AA	205734	Albany	0	0	0	0	0	0	0	0	0	0	0
SAL_WA3624AA	CFSAN087473	Albany	0	0	0	0	0	0	0	0	0	0	0
SAL_RA8142AA	283126	Albert	0	0	0	0	0	0	0	0	0	0	0
SAL_EA5413AA	POR1-0076	Albuquerque	0	0	0	0	0	0	0	0	0	0	0
SAL_JA9660AA	FDA00001232	Albuquerque	0	0	0	0	0	0	0	0	0	0	0
SAL_JA7473AA	CFSAN059897	Altendorf	0	0	0	0	0	0	0	0	0	0	0
SAL_VA7507AA	201705230	Altendorf	0	0	0	0	0	0	0	0	0	0	0
SAL_EA3023AA	FSIS1502794	Altona	0	0	0	0	0	0	0	0	0	0	0
SAL_EA7434AA	21862	Altona	0	0	0	0	0	0	0	0	0	0	0
SAL_FA5930AA	CFSAN048159	Altona	0	0	0	0	0	0	0	0	0	0	0
SAL_FA5924AA	IEH-NGS-SAL-04089	Altona	0	0	0	0	0	0	0	0	0	0	0
SAL_FA5052AA	PNUSAS001843	Altona	0	0	0	0	0	0	0	0	0	0	0
SAL_FA4284AA	140239	Altona	0	0	0	0	0	0	0	0	0	0	0

SAL_FA2936AA	CVM N57113F	Altona	0	0	0	0	0	0	0	0	0	0	0
SAL_FA2442AA	CFSAN042390	Altona	0	0	0	0	0	0	0	0	0	0	0
SAL_FA2440AA	CFSAN042392	Altona	0	0	0	0	0	0	0	0	0	0	0
SAL_FA2439AA	CFSAN042393	Altona	0	0	0	0	0	0	0	0	0	0	0
SAL_FA2429AA	CFSAN042405	Altona	0	0	0	0	0	0	0	0	0	0	0
SAL_FA1463AA	CFSAN042407	Altona	0	0	0	0	0	0	0	0	0	0	0
SAL_FA1459AA	CFSAN042411	Altona	0	0	0	0	0	0	0	0	0	0	0
SAL_FA1446AA	CFSAN042424	Altona	0	0	0	0	0	0	0	0	0	0	0
SAL_FA1448AA	CFSAN042422	Altona	0	0	0	0	0	0	0	0	0	0	0
SAL_FA1453AA	CFSAN042417	Altona	0	0	0	0	0	0	0	0	0	0	0
SAL_FA1444AA	CFSAN042426	Altona	0	0	0	0	0	0	0	0	0	0	0
SAL_FA1450AA	CFSAN042420	Altona	0	0	0	0	0	0	0	0	0	0	0
SAL_FA1443AA	CFSAN042427	Altona	0	0	0	0	0	0	0	0	0	0	0
SAL_EA7630AA	21070	Altona	0	0	0	0	0	0	0	0	0	0	0
SAL_EA6460AA	FDA00002277	Altona	0	0	0	0	0	0	0	0	0	0	0
SAL_EA6459AA	FDA00002276	Altona	0	0	0	0	0	0	0	0	0	0	0
SAL_EA5239AA	FSIS1502824	Altona	0	0	0	0	0	0	0	0	0	0	0
SAL_EA5229AA	FSIS1502822	Altona	0	0	0	0	0	0	0	0	0	0	0
SAL_EA4973AA	ADRDL-14-17772	Altona	0	0	0	0	0	0	0	0	0	0	0
SAL_EA0203AA	FL-UFL-47	Altona	0	0	0	0	0	0	0	0	0	0	0
SAL_EA0079AA	NY_IDR1100018135TX2	Altona	0	0	0	0	0	0	0	0	0	0	0
SAL_DA9716AA	FL_EPI-010	Altona	0	0	0	0	0	0	0	0	0	0	0
SAL_DA9694AA	FL_EPI-013	Altona	0	0	0	0	0	0	0	0	0	0	0
SAL_DA9668AA	MDH-2013-00200	Altona	0	0	0	0	0	0	0	0	0	0	0
SAL_DA8685AA	FDN0038	Altona	0	0	0	0	0	0	0	0	0	0	0
SAL_DA8628AA	FDN0052	Altona	0	0	0	0	0	0	0	0	0	0	0
SAL_DA8612AA	FDN0056	Altona	0	0	0	0	0	0	0	0	0	0	0
SAL_DA8193AA	VA_WGS-00169	Altona	0	0	0	0	0	0	0	0	0	0	0
SAL_DA8104AA	FAR0081	Altona	0	0	0	0	0	0	0	0	0	0	0
SAL_DA6828AA	VA-WGS-00249	Altona	0	0	0	0	0	0	0	0	0	0	0
SAL_DA6825AA	VA-WGS-00252	Altona	0	0	0	0	0	0	0	0	0	0	0
SAL_DA6795AA	VA-WGS-00253	Altona	0	0	0	0	0	0	0	0	0	0	0
SAL_DA2270AA	06-0764	Altona	0	0	0	0	0	0	0	0	0	0	0
SAL_DA0143AA	FDA00003640	Altona	0	0	0	0	0	0	0	0	0	0	0
SAL_DA0083AA	FDA00003638	Altona	0	0	0	0	0	0	0	0	0	0	0
SAL_DA0078AA	FDA00003639	Altona	0	0	0	0	0	0	0	0	0	0	0
SAL_DA0039AA	2011K-1691	Altona	0	0	0	0	0	0	0	0	0	0	0
SAL_CA6034AA	60365	Altona	0	0	0	0	0	0	0	0	0	0	0
SAL_CA3262AA	73197	Altona	0	0	0	0	0	0	0	0	0	0	0
SAL_BA8504AA	FL_EPI-014	Altona	0	0	0	0	0	0	0	0	0	0	0
SAL_BA8502AA	FL_EPI-015	Altona	0	0	0	0	0	0	0	0	0	0	0
SAL_BA7371AA	FDA00002546	Altona	0	0	0	0	0	0	0	0	0	0	0
SAL_BA6573AA	FDA00002545	Altona	0	0	0	0	0	0	0	0	0	0	0
SAL_BA6429AA	FDA00002630	Altona	0	0	0	0	0	0	0	0	0	0	0
SAL_BA1480AA	FSIS1503201	Altona	0	0	0	0	0	0	0	0	0	0	0
SAL_FA6124AA	PNUSAS002054	Altona	0	0	0	0	0	0	0	0	0	0	0
SAL_FA7906AA	FSIS1606763	Altona	0	0	0	0	0	0	0	0	0	0	0
SAL_FA8168AA	PNUSAS002313	Altona	0	0	0	0	0	0	0	0	0	0	0
SAL_FA8241AA	FSIS1606928	Altona	0	0	0	0	0	0	0	0	0	0	0
SAL_GA5695AA	CVM N58653	Altona	0	0	0	0	0	0	0	0	0	0	0
SAL_GA5692AA	CVM N58656	Altona	0	0	0	0	0	0	0	0	0	0	0
SAL_HA3045AA	FSIS1608039	Altona	0	0	0	0	0	0	0	0	0	0	0
SAL_HA2948AA	FSIS1608098	Altona	0	0	0	0	0	0	0	0	0	0	0
SAL_HA2924AA	FSIS1608107	Altona	0	0	0	0	0	0	0	0	0	0	0
SAL_HA3970AA	FSIS1608224	Altona	0	0	0	0	0	0	0	0	0	0	0
SAL_HA4188AA	CFSAN048325	Altona	0	0	0	0	0	0	0	0	0	0	0
SAL_HA4235AA	PNUSAS005388	Altona	0	0	0	0	0	0	0	0	0	0	0
SAL_HA6022AA	CFSAN056621	Altona	0	0	0	0	0	0	0	0	0	0	0
SAL_HA6024AA	CFSAN056622	Altona	0	0	0	0	0	0	0	0	0	0	0
SAL_HA7179AA	CFSAN056778	Altona	0	0	0	0	0	0	0	0	0	0	0
SAL_HA8704AA	OH-16-12495	Altona	0	0	0	0	0	0	0	0	0	0	0
SAL_HA9003AA	PNUSAS006090	Altona	0	0	0	0	0	0	0	0	0	0	0
SAL_HA9242AA	PNUSAS006179	Altona	0	0	0	0	0	0	0	0	0	0	0
SAL_HA9707AA	CFSAN058808	Altona	0	0	0	0	0	0	0	0	0	0	0
SAL_HA9706AA	CFSAN058809	Altona	0	0	0	0	0	0	0	0	0	0	0
SAL_HA9686AA	CFSAN058846	Altona	0	0	0	0	0	0	0	0	0	0	0
SAL_HA9709AA	CFSAN058810	Altona	0	0	0	0	0	0	0	0	0	0	0
SAL_HA9677AA	CFSAN058839	Altona	0	0	0	0	0	0	0	0	0	0	0
SAL_HA9679AA	CFSAN058841	Altona	0	0	0	0	0	0	0	0	0	0	0
SAL_HA9684AA	CFSAN058840	Altona	0	0	0	0	0	0	0	0	0	0	0
SAL_HA9682AA	CFSAN058838	Altona	0	0	0	0	0	0	0	0	0	0	0
SAL_HA9687AA	CFSAN058842	Altona	0	0	0	0	0	0	0	0	0	0	0
SAL_IA0423AA	PNUSAS007246	Altona	0	0	0	0	0	0	0	0	0	0	0

SAL_IA2291AA	CFSAN059161	Altona	0	0	0	0	0	0	0	0	0	0	0
SAL_IA2290AA	CFSAN059162	Altona	0	0	0	0	0	0	0	0	0	0	0
SAL_IA2294AA	CFSAN059176	Altona	0	0	0	0	0	0	0	0	0	0	0
SAL_IA2719AA	CFSAN059268	Altona	0	0	0	0	0	0	0	0	0	0	0
SAL_IA2715AA	CFSAN059269	Altona	0	0	0	0	0	0	0	0	0	0	0
SAL_IA2717AA	CFSAN059270	Altona	0	0	0	0	0	0	0	0	0	0	0
SAL_IA4974AA	CFSAN059296	Altona	0	0	0	0	0	0	0	0	0	0	0
SAL_IA6181AA	CFSAN059124	Altona	0	0	0	0	0	0	0	0	0	0	0
SAL_IA5533AA	CFSAN059813	Altona	0	0	0	0	0	0	0	0	0	0	0
SAL_IA6496AA	PNUSAS008108	Altona	0	0	0	0	0	0	0	0	0	0	0
SAL_IA7105AA	CFSAN059832	Altona	0	0	0	0	0	0	0	0	0	0	0
SAL_IA9410AA	PNUSAS007049	Altona	0	0	0	0	0	0	0	0	0	0	0
SAL_JA1129AA	PNUSAS007056	Altona	0	0	0	0	0	0	0	0	0	0	0
SAL_JA5261AA	PNUSAS013602	Altona	0	0	0	0	0	0	0	0	0	0	0
SAL_JA6058AA	FSIS1701107	Altona	0	0	0	0	0	0	0	0	0	0	0
SAL_JA5962AA	PNUSAS011892	Altona	0	0	0	0	0	0	0	0	0	0	0
SAL_JA9023AA	MS170112	Altona	0	0	0	0	0	0	0	0	0	0	0
SAL_KA1715AA	PNUSAS016684	Altona	0	0	0	0	0	0	0	0	0	0	0
SAL_KA3586AA	FSIS1702516	Altona	0	0	0	0	0	0	0	0	0	0	0
SAL_KA5562AA	FSIS1703063	Altona	0	0	0	0	0	0	0	0	0	0	0
SAL_KA6190AA	2017-01-1266-1	Altona	0	0	0	0	0	0	0	0	0	0	0
SAL_KA6188AA	2017-01-1265-1	Altona	0	0	0	0	0	0	0	0	0	0	0
SAL_KA9190AA	17035838	Altona	0	0	0	0	0	0	0	0	0	0	0
SAL_KA9396AA	CFSAN067257	Altona	0	0	0	0	0	0	0	0	0	0	0
SAL_LA0671AA	FSIS1703463	Altona	0	0	0	0	0	0	0	0	0	0	0
SAL_LA1079AA	PNUSAS021181	Altona	0	0	0	0	0	0	0	0	0	0	0
SAL_LA4410AA	PNUSAS022500	Altona	0	0	0	0	0	0	0	0	0	0	0
SAL_LA4795AA	FSIS1703879	Altona	0	0	0	0	0	0	0	0	0	0	0
SAL_LA5147AA	17041750	Altona	0	0	0	0	0	0	0	0	0	0	0
SAL_LA6201AA	CFSAN069207	Altona	0	0	0	0	0	0	0	0	0	0	0
SAL_LA6210AA	CCK-T-027	Altona	0	0	0	0	0	0	0	0	0	0	0
SAL_LA6193AA	CFSAN069264	Altona	0	0	0	0	0	0	0	0	0	0	0
SAL_LA6093AA	CFSAN069258	Altona	0	0	0	0	0	0	0	0	0	0	0
SAL_LA6105AA	CFSAN069256	Altona	0	0	0	0	0	0	0	0	0	0	0
SAL_LA6136AA	CFSAN069265	Altona	0	0	0	0	0	0	0	0	0	0	0
SAL_LA6179AA	CFSAN069257	Altona	0	0	0	0	0	0	0	0	0	0	0
SAL_MA1215AA	CFSAN068477	Altona	0	0	0	0	0	0	0	0	0	0	0
SAL_MA1220AA	CFSAN068475	Altona	0	0	0	0	0	0	0	0	0	0	0
SAL_MA1218AA	CFSAN068481	Altona	0	0	0	0	0	0	0	0	0	0	0
SAL_MA1219AA	CFSAN068478	Altona	0	0	0	0	0	0	0	0	0	0	0
SAL_MA1487AA	CFSAN070477	Altona	0	0	0	0	0	0	0	0	0	0	0
SAL_MA1500AA	CFSAN070478	Altona	0	0	0	0	0	0	0	0	0	0	0
SAL_MA1279AA	CFSAN068482	Altona	0	0	0	0	0	0	0	0	0	0	0
SAL_MA1288AA	CFSAN070270	Altona	0	0	0	0	0	0	0	0	0	0	0
SAL_MA1294AA	CFSAN070268	Altona	0	0	0	0	0	0	0	0	0	0	0
SAL_MA1306AA	CFSAN068474	Altona	0	0	0	0	0	0	0	0	0	0	0
SAL_MA1307AA	CFSAN068473	Altona	0	0	0	0	0	0	0	0	0	0	0
SAL_MA1310AA	CFSAN068471	Altona	0	0	0	0	0	0	0	0	0	0	0
SAL_MA1308AA	CFSAN068472	Altona	0	0	0	0	0	0	0	0	0	0	0
SAL_MA3018AA	CFSAN070492	Altona	0	0	0	0	0	0	0	0	0	0	0
SAL_MA3006AA	CFSAN070499	Altona	0	0	0	0	0	0	0	0	0	0	0
SAL_MA3016AA	CFSAN070493	Altona	0	0	0	0	0	0	0	0	0	0	0
SAL_MA3917AA	CFSAN070501	Altona	0	0	0	0	0	0	0	0	0	0	0
SAL_MA3913AA	CFSAN070500	Altona	0	0	0	0	0	0	0	0	0	0	0
SAL_MA3922AA	CFSAN070491	Altona	0	0	0	0	0	0	0	0	0	0	0
SAL_MA4900AA	PNUSAS025794	Altona	0	0	0	0	0	0	0	0	0	0	0
SAL_MA5328AA	OH-17-21246	Altona	0	0	0	0	0	0	0	0	0	0	0
SAL_MA5330AA	OH-17-21303	Altona	0	0	0	0	0	0	0	0	0	0	0
SAL_DA1425AA	H123540677	Altona	0	0	0	0	0	0	0	0	0	0	0
SAL_DA1399AA	H122360425	Altona	0	0	0	0	0	0	0	0	0	0	0
SAL_HA9731AA	CFSAN058834	Altona	0	0	0	0	0	0	0	0	0	0	0
SAL_HA8363AA	FSIS1608962	Altona	0	0	0	0	0	0	0	0	0	0	0
SAL_GA8053AA	FDA00010525	Altona	0	0	0	0	0	0	0	0	0	0	0
SAL_JA9795AA	FSIS1701475	Altona	0	0	0	0	0	0	0	0	0	0	0
SAL_MA6434AA	CFSAN071425	Altona	0	0	0	0	0	0	0	0	0	0	0
SAL_MA7323AA	CFSAN071800	Altona	0	0	0	0	0	0	0	0	0	0	0
SAL_MA6947AA	CFSAN071472	Altona	0	0	0	0	0	0	0	0	0	0	0
SAL_MA6952AA	CFSAN071481	Altona	0	0	0	0	0	0	0	0	0	0	0
SAL_MA6915AA	CFSAN071476	Altona	0	0	0	0	0	0	0	0	0	0	0
SAL_MA6937AA	CFSAN071470	Altona	0	0	0	0	0	0	0	0	0	0	0
SAL_MA6934AA	CFSAN071467	Altona	0	0	0	0	0	0	0	0	0	0	0
SAL_MA6860AA	CFSAN071453	Altona	0	0	0	0	0	0	0	0	0	0	0
SAL_MA6861AA	CFSAN071452	Altona	0	0	0	0	0	0	0	0	0	0	0

SAL_MA6846AA	CFSAN071451	Altona	0	0	0	0	0	0	0	0	0	0	0
SAL_MA6858AA	CFSAN071454	Altona	0	0	0	0	0	0	0	0	0	0	0
SAL_MA7947AA	CFSAN071827	Altona	0	0	0	0	0	0	0	0	0	0	0
SAL_MA9088AA	CFSAN071840	Altona	0	0	0	0	0	0	0	0	0	0	0
SAL_MA9096AA	CFSAN070483	Altona	0	0	0	0	0	0	0	0	0	0	0
SAL_MA9075AA	CFSAN070553	Altona	0	0	0	0	0	0	0	0	0	0	0
SAL_NA0405AA	CFSAN070555	Altona	0	0	0	0	0	0	0	0	0	0	0
SAL_NA0410AA	CFSAN070556	Altona	0	0	0	0	0	0	0	0	0	0	0
SAL_NA0402AA	CFSAN070554	Altona	0	0	0	0	0	0	0	0	0	0	0
SAL_NA0923AA	CFSAN072590	Altona	0	0	0	0	0	0	0	0	0	0	0
SAL_NA1150AA	PNUSAS026795	Altona	0	0	0	0	0	0	0	0	0	0	0
SAL_NA1663AA	FSIS11705830	Altona	0	0	0	0	0	0	0	0	0	0	0
SAL_NA1639AA	FSIS11705061	Altona	0	0	0	0	0	0	0	0	0	0	0
SAL_NA1428AA	CFSAN071482	Altona	0	0	0	0	0	0	0	0	0	0	0
SAL_NA2609AA	CFSAN068480	Altona	0	0	0	0	0	0	0	0	0	0	0
SAL_NA2611AA	CFSAN068476	Altona	0	0	0	0	0	0	0	0	0	0	0
SAL_NA2610AA	CFSAN068479	Altona	0	0	0	0	0	0	0	0	0	0	0
SAL_NA3815AA	2015AM-2789	Altona	0	0	0	0	0	0	0	0	0	0	0
SAL_NA4490AA	CFSAN074258	Altona	0	0	0	0	0	0	0	0	0	0	0
SAL_NA4474AA	CFSAN074257	Altona	0	0	0	0	0	0	0	0	0	0	0
SAL_NA4478AA	CFSAN074259	Altona	0	0	0	0	0	0	0	0	0	0	0
SAL_NA8279AA	FSIS11706479	Altona	0	0	0	0	0	0	0	0	0	0	0
SAL_NA8567AA	PNUSAS033001	Altona	0	0	0	0	0	0	0	0	0	0	0
SAL_NA8929AA	CFSAN075283	Altona	0	0	0	0	0	0	0	0	0	0	0
SAL_NA9210AA	CFSAN075257	Altona	0	0	0	0	0	0	0	0	0	0	0
SAL_NA9880AA	PNUSAS033933	Altona	0	0	0	0	0	0	0	0	0	0	0
SAL_OA0975AA	CFSAN075611	Altona	0	0	0	0	0	0	0	0	0	0	0
SAL_OA0992AA	CFSAN075475	Altona	0	0	0	0	0	0	0	0	0	0	0
SAL_OA4358AA	FDA00012762	Altona	0	0	0	0	0	0	0	0	0	0	0
SAL_PA1842AA	CFSAN078683	Altona	0	0	0	0	0	0	0	0	0	0	0
SAL_PA3342AA	CFSAN078699	Altona	0	0	0	0	0	0	0	0	0	0	0
SAL_PA9346AA	CFSAN081762	Altona	0	0	0	0	0	0	0	0	0	0	0
SAL_PA9348AA	CFSAN081763	Altona	0	0	0	0	0	0	0	0	0	0	0
SAL_PA9338AA	CFSAN081761	Altona	0	0	0	0	0	0	0	0	0	0	0
SAL_PA9435AA	CFSAN081759	Altona	0	0	0	0	0	0	0	0	0	0	0
SAL_QA2352AA	296289	Altona	0	0	0	0	0	0	0	0	0	0	0
SAL_QA2255AA	489206	Altona	0	0	0	0	0	0	0	0	0	0	0
SAL_QA4302AA	PNUSAS038547	Altona	0	0	0	0	0	0	0	0	0	0	0
SAL_QA5489AA	130384	Altona	0	0	0	0	0	0	0	0	0	0	0
SAL_QA6510AA	247203	Altona	0	0	0	0	0	0	0	0	0	0	0
SAL_QA6486AA	488734	Altona	0	0	0	0	0	0	0	0	0	0	0
SAL_QA6487AA	209692	Altona	0	0	0	0	0	0	0	0	0	0	0
SAL_QA7543AA	390051	Altona	0	0	0	0	0	0	0	0	0	0	0
SAL_QA8157AA	CFSAN082371	Altona	0	0	0	0	0	0	0	0	0	0	0
SAL_RA0114AA	PNUSAS043948	Altona	0	0	0	0	0	0	0	0	0	0	0
SAL_RA4216AA	TTU_224	Altona	0	0	0	0	0	0	0	0	0	0	0
SAL_RA4201AA	TTU_225	Altona	0	0	0	0	0	0	0	0	0	0	0
SAL_RA1790AA	95f-558b-465d-bf36-8ee	Altona	0	0	0	0	0	0	0	0	0	0	0
SAL_RA7552AA	PNUSAS044305	Altona	0	0	0	0	0	0	0	0	0	0	0
SAL_RA8530AA	TTU_236	Altona	0	0	0	0	0	0	0	0	0	0	0
SAL_RA9986AA	FSIS11812703	Altona	0	0	0	0	0	0	0	0	0	0	0
SAL_SA2539AA	FSIS11812673	Altona	0	0	0	0	0	0	0	0	0	0	0
SAL_SA4302AA	FSIS11813891	Altona	0	0	0	0	0	0	0	0	0	0	0
SAL_TA6604AA	OH-18-22026	Altona	0	0	0	0	0	0	0	0	0	0	0
SAL_TA6793AA	FSIS11815524	Altona	0	0	0	0	0	0	0	0	0	0	0
SAL_OA2378AA	CFSAN075544	Altona	0	0	0	0	0	0	0	0	0	0	0
SAL_OA0825AA	PNUSAS034322	Altona	0	0	0	0	0	0	0	0	0	0	0
SAL_QA7952AA	PNUSAS043973	Altona	0	0	0	0	0	0	0	0	0	0	0
SAL_OA1589AA	CFSAN075622	Altona	0	0	0	0	0	0	0	0	0	0	0
SAL_OA1832AA	PNUSAS035108	Altona	0	0	0	0	0	0	0	0	0	0	0
SAL_OA2178AA	FDA00012568	Altona	0	0	0	0	0	0	0	0	0	0	0
SAL_PA1536AA	PNUSAS037709	Altona	0	0	0	0	0	0	0	0	0	0	0
SAL_PA1567AA	OH-17-27229	Altona	0	0	0	0	0	0	0	0	0	0	0
SAL_PA3589AA	CFSAN074844	Altona	0	0	0	0	0	0	0	0	0	0	0
SAL_UA3691AA	599568	Altona	0	0	0	0	0	0	0	0	0	0	0
SAL_UA6803AA	660973	Altona	0	0	0	0	0	0	0	0	0	0	0
SAL_UA5749AA	SAL-18-VL-NY-GA-0007	Altona	0	0	0	0	0	0	0	0	0	0	0
SAL_UA6430AA	579250	Altona	0	0	0	0	0	0	0	0	0	0	0
SAL_UA6321AA	FSIS11814299	Altona	0	0	0	0	0	0	0	0	0	0	0
SAL_UA8900AA	ADRDL-2269	Altona	0	0	0	0	0	0	0	0	0	0	0
SAL_VA0899AA	ADRDL-2391	Altona	0	0	0	0	0	0	0	0	0	0	0
SAL_VA7646AA	201708398	Altona	0	0	0	0	0	0	0	0	0	0	0
SAL_VA3145AA	FSIS11917350	Altona	0	0	0	0	0	0	0	0	0	0	0

SAL_VA2706AA	421181	Altona	0	0	0	0	0	0	0	0	0	0	0
SAL_VA2831AA	SAL-18-VL-OH-GA-0007	Altona	0	0	0	0	0	0	0	0	0	0	0
SAL_VA2819AA	AG19-0068	Altona	0	0	0	0	0	0	0	0	0	0	0
SAL_VA3387AA	NC_WHO_S072	Altona	0	0	0	0	0	0	0	0	0	0	0
SAL_VA5926AA	SA20102782	Altona	0	0	0	0	0	0	0	0	0	0	0
SAL_VA5563AA	CFSAN058796	Altona	0	0	0	0	0	0	0	0	0	0	0
SAL_VA5565AA	CFSAN058807	Altona	0	0	0	0	0	0	0	0	0	0	0
SAL_VA5566AA	CFSAN058806	Altona	0	0	0	0	0	0	0	0	0	0	0
SAL_VA5567AA	CFSAN058805	Altona	0	0	0	0	0	0	0	0	0	0	0
SAL_VA5568AA	CFSAN058803	Altona	0	0	0	0	0	0	0	0	0	0	0
SAL_VA5569AA	CFSAN058804	Altona	0	0	0	0	0	0	0	0	0	0	0
SAL_VA5557AA	CFSAN058797	Altona	0	0	0	0	0	0	0	0	0	0	0
SAL_VA5558AA	CFSAN058801	Altona	0	0	0	0	0	0	0	0	0	0	0
SAL_VA5559AA	CFSAN058798	Altona	0	0	0	0	0	0	0	0	0	0	0
SAL_VA5560AA	CFSAN058800	Altona	0	0	0	0	0	0	0	0	0	0	0
SAL_VA5561AA	CFSAN058799	Altona	0	0	0	0	0	0	0	0	0	0	0
SAL_VA3834AA	OH-17-27674	Altona	0	0	0	0	0	0	0	0	0	0	0
SAL_VA4308AA	CFSAN071783	Altona	0	0	0	0	0	0	0	0	0	0	0
SAL_VA9250AA	DAC29	Altona	0	0	0	0	0	0	0	0	0	0	0
SAL_VA9257AA	DAC54	Altona	0	0	0	0	0	0	0	0	0	0	0
SAL_WA3024AA	NL_R43	Altona	0	0	0	0	0	0	0	0	0	0	0
SAL_WA3022AA	NL_R46	Altona	0	0	0	0	0	0	0	0	0	0	0
SAL_WA3021AA	NL_R47	Altona	0	0	0	0	0	0	0	0	0	0	0
SAL_WA2797AA	NL_R44	Altona	0	0	0	0	0	0	0	0	0	0	0
SAL_WA2763AA	NL_R45	Altona	0	0	0	0	0	0	0	0	0	0	0
SAL_WA2767AA	NL_R42	Altona	0	0	0	0	0	0	0	0	0	0	0
SAL_WA2439AA	NL_R51	Altona	0	0	0	0	0	0	0	0	0	0	0
SAL_WA2338AA	1090900215	Altona	0	0	0	0	0	0	0	0	0	0	0
SAL_WA2234AA	NL_R49	Altona	0	0	0	0	0	0	0	0	0	0	0
SAL_WA2233AA	NL_R48	Altona	0	0	0	0	0	0	0	0	0	0	0
SAL_WA2231AA	NL_R50	Altona	0	0	0	0	0	0	0	0	0	0	0
SAL_WA2230AA	NL_R53	Altona	0	0	0	0	0	0	0	0	0	0	0
SAL_WA2229AA	NL_R52	Altona	0	0	0	0	0	0	0	0	0	0	0
SAL_WA2227AA	NL_R54	Altona	0	0	0	0	0	0	0	0	0	0	0
SAL_WA3568AA	CFSAN087472	Altona	0	0	0	0	0	0	0	0	0	0	0
SAL_WA3729AA	CFSAN087586	Altona	0	0	0	0	0	0	0	0	0	0	0
SAL_DA2687AA	FDA00004213	Amager	0	0	0	0	0	0	0	0	0	0	0
SAL_FA4532AA	130420	Amager	0	0	0	0	0	0	0	0	0	0	0
SAL_FA4343AA	130417	Amager	0	0	0	0	0	0	0	0	0	0	0
SAL_EA8976AA	22828722	Amager	0	0	0	0	0	0	0	0	0	0	0
SAL_EA8886AA	1509H35917	Amager	0	0	0	0	0	0	0	0	0	0	0
SAL_EA6982AA	21853	Amager	0	0	0	0	0	0	0	0	0	0	0
SAL_EA2422AA	FDA00003455	Amager	0	0	0	0	0	0	0	0	0	0	0
SAL_EA0398AA	FSF0041	Amager	0	0	0	0	0	0	0	0	0	0	0
SAL_DA6433AA	FAR0186	Amager	0	0	0	0	0	0	0	0	0	0	0
SAL_DA1368AA	H124040536	Amager	0	0	0	0	0	0	0	0	0	0	0
SAL_CA6474AA	63556	Amager	0	0	0	0	0	0	0	0	0	0	0
SAL_CA3419AA	53012	Amager	0	0	0	0	0	0	0	0	0	0	0
SAL_CA0182AA	69908	Amager	0	0	0	0	0	0	0	0	0	0	0
SAL_BA9294AA	CFSAN002720	Amager	0	0	0	0	0	0	0	0	0	0	0
SAL_BA9300AA	CFSAN002717	Amager	0	0	0	0	0	0	0	0	0	0	0
SAL_BA5615AA	FSF0161	Amager	0	0	0	0	0	0	0	0	0	0	0
SAL_BA4169AA	FDA00009132	Amager	0	0	0	0	0	0	0	0	0	0	0
SAL_BA4167AA	FDA00009131	Amager	0	0	0	0	0	0	0	0	0	0	0
SAL_BA2474AA	FDA00009157	Amager	0	0	0	0	0	0	0	0	0	0	0
SAL_GA3361AA	BCW_2635	Amager	0	0	0	0	0	0	0	0	0	0	0
SAL_GA3360AA	BCW_2636	Amager	0	0	0	0	0	0	0	0	0	123	0
SAL_GA5389AA	CFSAN042363	Amager	0	0	0	0	0	0	0	0	0	0	0
SAL_GA5388AA	CFSAN042364	Amager	0	0	0	0	0	0	0	0	0	0	0
SAL_GA8741AA	CFSAN031397	Amager	0	0	0	0	0	0	0	0	0	0	0
SAL_HA3629AA	56C BS BEDROOM	Amager	0	0	0	0	0	0	0	0	0	0	0
SAL_HA3619AA	56C BS PERIMETER	Amager	0	0	0	0	0	0	0	0	0	0	0
SAL_HA4715AA	FSIS1608669	Amager	0	0	0	0	0	0	0	0	0	0	0
SAL_HA2684AA	ICOPHAI16755	Amager	0	0	0	0	0	0	0	0	0	0	0
SAL_HA9126AA	CFSAN058084	Amager	0	0	0	0	0	0	0	0	0	0	0
SAL_IA0697AA	223571	Amager	0	0	0	0	0	0	0	0	0	0	0
SAL_IA5275AA	CFSAN058010	Amager	0	0	0	0	0	0	0	0	0	0	0
SAL_IA5254AA	CFSAN057990	Amager	0	0	0	0	0	0	0	0	0	0	0
SAL_IA5266AA	CFSAN058000	Amager	0	0	0	0	0	0	0	0	0	0	0
SAL_IA5273AA	CFSAN058013	Amager	0	0	0	0	0	0	0	0	0	0	0
SAL_IA5260AA	CFSAN057998	Amager	0	0	0	0	0	0	0	0	0	0	0
SAL_IA5265AA	CFSAN058001	Amager	0	0	0	0	0	0	0	0	0	0	0
SAL_IA5280AA	CFSAN058011	Amager	0	0	0	0	0	0	0	0	0	0	0

SAL_IA5268AA	CFSAN057999	Amager	0	0	0	0	0	0	0	0	0	0	0
SAL_JA2916AA	PNUSAS010828	Amager	0	0	0	0	0	0	0	0	0	0	0
SAL_JA3910AA	PNUSAS011881	Amager	0	0	0	0	0	0	0	0	0	0	0
SAL_KA5037AA	CFSAN065048	Amager	0	0	0	0	0	0	0	0	0	0	0
SAL_KA5509AA	PNUSAS017657	Amager	0	0	0	0	0	0	0	0	0	0	0
SAL_KA7155AA	FSIS1703193	Amager	0	0	0	0	0	0	0	0	0	0	0
SAL_LA4619AA	PNUSAS022592	Amager	0	0	0	0	0	0	0	0	0	0	0
SAL_EA2421AA	FDA00003454	Amager	0	0	0	0	0	0	0	0	0	0	0
SAL_GA8810AA	PNUSAS003680	Amager	0	0	0	0	0	0	0	0	0	0	0
SAL_GA5387AA	CFSAN042365	Amager	0	0	0	0	0	0	0	0	0	0	0
SAL_MA8317AA	PNUSAS026566	Amager	0	0	0	0	0	0	0	0	0	0	0
SAL_NA1136AA	PNUSAS027392	Amager	0	0	0	0	0	0	0	0	0	0	0
SAL_NA9328AA	S04338-88	Amager	0	0	0	0	0	0	0	0	0	0	0
SAL_OA4919AA	PNUSAS035592	Amager	0	0	0	0	0	0	0	0	0	0	0
SAL_PA4799AA	451977	Amager	0	0	0	0	0	0	0	0	0	0	0
SAL_PA4770AA	314914	Amager	0	0	0	0	0	0	0	0	0	0	0
SAL_PA8624AA	FSIS31800452	Amager	0	0	0	0	0	0	0	0	0	0	0
SAL_PA9968AA	155747	Amager	0	0	0	0	0	0	0	0	0	0	0
SAL_QA4793AA	155746	Amager	0	0	0	0	0	0	0	0	0	0	0
SAL_QA5850AA	159693	Amager	0	0	0	0	0	0	0	0	0	0	0
SAL_RA3303AA	101463	Amager	0	0	0	0	0	0	0	0	0	0	0
SAL_RA4102AA	308524	Amager	0	0	0	0	0	0	0	0	0	0	0
SAL_QA9826AA	204432	Amager	0	0	0	0	0	0	0	0	0	0	0
SAL_RA5702AA	130422	Amager	0	0	0	0	0	0	0	0	0	0	0
SAL_RA6976AA	FSIS11811370	Amager	0	0	0	0	0	0	0	0	0	0	0
SAL_RA8675AA	FSIS11812172	Amager	0	0	0	0	0	0	0	0	0	0	0
SAL_SA2901AA	FSIS31800918	Amager	0	0	0	0	0	0	0	0	0	0	0
SAL_UA7008AA	FDA00013776	Amager	0	0	0	0	0	0	0	0	0	0	0
SAL_VA3038AA	DAC31	Amager	0	0	0	0	0	0	0	0	0	0	0
SAL_VA2603AA	FDA00013846	Amager	0	0	0	0	0	0	0	0	0	0	0
SAL_WA1588AA	130419	Amager	0	0	0	0	0	0	0	0	0	0	0
SAL_WA1528AA	101461	Amager	0	0	0	0	0	0	0	0	0	0	0
SAL_DA6110AA	MOD1_Sal_848	Amersfoort	0	0	0	0	0	0	0	0	0	0	0
SAL_DA3198AA	CFSAN023674	Amersfoort	0	0	0	0	0	0	0	0	0	0	0
SAL_CA2002AA	50885	Amersfoort	0	0	0	0	0	0	0	0	0	0	0
SAL_GA4500AA	FDA00001390	Amersfoort	0	0	0	0	0	0	0	0	0	0	0
SAL_GA6242AA	VUPRC	Amersfoort	0	0	0	0	0	0	0	0	0	0	0
SAL_JA3788AA	PNUSAS012411	Amersfoort	0	0	0	0	0	0	0	0	0	0	0
SAL_KA4207AA	4 PAR-79	Amersfoort	0	0	0	0	0	0	0	0	0	0	0
SAL_MA0964AA	PNUSAS025528	Amersfoort	0	0	0	0	0	0	0	0	0	0	0
SAL_CA1566AA	37761	Amersfoort	0	0	0	0	0	0	0	0	0	0	0
SAL_GA5829AA	FDA00001412	Amersfoort	0	0	0	0	0	0	0	0	0	0	0
SAL_GA3346AA	BCW_2656	Amersfoort	0	0	0	0	0	0	0	0	0	0	0
SAL_JA8199AA	339503	Amersfoort	0	0	0	0	0	0	0	0	0	0	0
SAL_QA6589AA	181030	Amersfoort	0	0	0	0	0	0	0	0	0	0	0
SAL_RA2743AA	247225	Amersfoort	0	0	0	0	0	0	0	0	0	0	0
SAL_TA5218AA	620444	Amersfoort	0	0	0	0	0	0	0	0	0	0	0
SAL_WA3201AA	CK_02/00000290	Amersfoort	0	0	0	0	0	0	0	0	0	0	0
SAL_HA8892AA	NCTC6385	Amherstiana	0	0	0	0	0	0	0	0	0	0	0
SAL_BA1431AA	FDA00000633	Aminatu	0	0	0	0	0	0	0	0	0	0	0
SAL_DA6119AA	MOD1_Sal_835	Amoutive	0	0	0	0	0	0	0	0	0	0	0
SAL_JA5943AA	350678	Amoutive	0	0	0	0	0	0	0	0	0	0	0
SAL_WA2957AA	AU123 KOB-5	Amoutive	0	0	0	0	0	0	0	0	0	0	0
SAL_WA2719AA	AU5 KAK-SR	Amoutive	0	0	0	0	0	0	0	0	0	0	0
SAL_WA2198AA	129 TIR-8	Amoutive	0	0	0	0	0	0	0	0	0	0	0
SAL_CA9118AA	CFSAN027175	Amsterdam	0	0	0	0	0	0	0	0	0	0	0
SAL_DA9345AA	FNW19H30	Amsterdam	0	0	0	0	0	0	0	0	0	0	0
SAL_FA5869AA	FDA00002559	Amsterdam	0	0	0	0	0	0	0	0	0	0	0
SAL_FA5863AA	FDA00002565	Amsterdam	0	0	0	0	0	0	0	0	0	0	0
SAL_FA5862AA	FDA00002566	Amsterdam	0	0	0	0	0	0	0	0	0	0	0
SAL_FA5700AA	FDA00001343	Amsterdam	0	0	0	0	0	0	0	0	0	0	0
SAL_FA4784AA	CFSAN032037	Amsterdam	0	0	0	0	0	0	0	0	0	0	0
SAL_EA2830AA	FDA00001866	Amsterdam	0	0	0	0	0	0	0	0	0	0	0
SAL_DA9349AA	FNW19H60	Amsterdam	0	0	0	0	0	0	0	0	0	0	0
SAL_DA6786AA	FDN0131	Amsterdam	0	0	0	0	0	0	0	0	0	0	0
SAL_DA6241AA	FSE0130	Amsterdam	0	0	0	0	0	0	0	0	0	0	0
SAL_DA6179AA	FSE0115	Amsterdam	0	0	0	0	0	0	0	0	0	0	0
SAL_DA5463AA	FDA00004350	Amsterdam	0	0	0	0	0	0	0	0	0	0	0
SAL_DA5141AA	FSF0178	Amsterdam	0	0	0	0	0	0	0	0	0	0	0
SAL_DA3838AA	FDA00004100	Amsterdam	0	0	0	0	0	0	0	0	0	0	0
SAL_DA2723AA	FDA00004223	Amsterdam	0	0	0	0	0	0	0	0	0	0	0
SAL_DA2033AA	FDA00000085	Amsterdam	0	0	0	0	0	0	0	0	0	0	0
SAL_CA9124AA	CFSAN027173	Amsterdam	0	0	0	0	0	0	0	0	0	0	0

SAL_CA4137AA	36994	Amsterdam	0	0	0	0	0	0	0	0	0	0	0
SAL_CA4025AA	13829	Amsterdam	0	0	0	0	0	0	0	0	0	0	0
SAL_BA6143AA	FDA00001163	Amsterdam	0	0	0	0	0	0	0	0	0	0	0
SAL_BA1745AA	FDA00001455	Amsterdam	0	0	0	0	0	0	0	0	0	0	0
SAL_BA1722AA	FDA00003294	Amsterdam	0	0	0	0	0	0	0	0	0	0	0
SAL_BA1668AA	FDA00003292	Amsterdam	0	0	0	0	0	0	0	0	0	0	0
SAL_BA1653AA	FDA00003293	Amsterdam	0	0	0	0	0	0	0	0	0	0	0
SAL_FA6566AA	PNUSAS002015	Amsterdam	0	0	0	0	0	0	0	0	0	0	0
SAL_FA7613AA	CFSAN032671	Amsterdam	0	0	0	0	0	0	0	0	0	0	0
SAL_GA3486AA	BCW_2891	Amsterdam	0	0	0	0	0	0	0	0	0	0	0
SAL_FA8446AA	FDA00000146	Amsterdam	0	0	0	0	0	0	0	0	0	0	0
SAL_GA8593AA	FDA00010557	Amsterdam	0	0	0	0	0	0	0	0	0	0	0
SAL_GA9742AA	FDA00003970	Amsterdam	0	0	0	0	0	0	0	0	0	0	0
SAL_IA7684AA	FSIS1710344	Amsterdam	0	0	0	0	0	0	0	0	0	0	0
SAL_JA3526AA	PNUSAS012267	Amsterdam	0	0	0	0	0	0	0	0	0	0	0
SAL_JA3955AA	PNUSAS011883	Amsterdam	0	0	0	0	0	0	0	0	0	0	0
SAL_KA6101AA	CFSAN065561	Amsterdam	0	0	0	0	0	0	0	0	0	0	0
SAL_KA7048AA	PNUSAS017312	Amsterdam	0	0	0	0	0	0	0	0	0	0	0
SAL_KA7480AA	PNUSAS019552	Amsterdam	0	0	0	0	0	0	0	0	0	0	0
SAL_LA4212AA	PNUSAS022055	Amsterdam	0	0	0	0	0	0	0	0	0	0	0
SAL_MA1339AA	FSIS11704710	Amsterdam	0	0	0	0	0	0	0	0	0	0	0
SAL_DA9396AA	FNE0057	Amsterdam	0	0	0	0	0	0	0	0	0	0	0
SAL_FA7966AA	PNUSAS002174	Amsterdam	0	0	0	0	0	0	0	0	0	0	0
SAL_MA9032AA	FSIS11705083	Amsterdam	0	0	0	0	0	0	0	0	0	0	0
SAL_NA0565AA	FSIS11706101	Amsterdam	0	0	0	0	0	0	0	0	0	0	0
SAL_NA6985AA	PNUSAS032440	Amsterdam	0	0	0	0	0	0	0	0	0	0	0
SAL_PA0016AA	PNUSAS037809	Amsterdam	0	0	0	0	0	0	0	0	0	0	0
SAL_OA4597AA	PNUSAS036927	Amsterdam	0	0	0	0	0	0	0	0	0	0	0
SAL_PA6208AA	191961	Amsterdam	0	0	0	0	0	0	0	0	0	0	0
SAL_QA2922AA	343784	Amsterdam	0	0	0	0	0	0	0	0	0	0	0
SAL_QA5538AA	424561	Amsterdam	0	0	0	0	0	0	0	0	0	0	0
SAL_QA5939AA	FSIS11810637	Amsterdam	0	0	0	0	0	0	0	0	0	0	0
SAL_QA6484AA	147839	Amsterdam	0	0	0	0	0	0	0	0	0	0	0
SAL_QA6935AA	FSIS11810656	Amsterdam	0	0	0	0	0	0	0	0	0	0	0
SAL_QA7614AA	328685	Amsterdam	0	0	0	0	0	0	0	0	0	0	0
SAL_QA8503AA	PNUSAS044198	Amsterdam	0	0	0	0	0	0	0	0	0	0	0
SAL_QA8799AA	480759	Amsterdam	0	0	0	0	0	0	0	0	0	0	0
SAL_RA3832AA	FDA00001570	Amsterdam	0	0	0	0	0	0	0	0	0	0	0
SAL_TA0396AA	602760	Amsterdam	0	0	0	0	0	0	0	0	0	0	0
SAL_TA5935AA	FSIS31801187	Amsterdam	0	0	0	0	0	0	0	0	0	0	0
SAL_TA6092AA	HC-285	Amsterdam	0	0	0	0	0	0	0	0	0	0	0
SAL_TA7272AA	HC-220	Amsterdam	0	0	0	0	0	0	0	0	0	0	0
SAL_TA7270AA	HC-304	Amsterdam	0	0	0	0	0	0	0	0	0	0	0
SAL_FA7808AA	CFSAN032661	Amsterdam	0	0	0	0	0	0	0	0	0	0	0
SAL_FA7807AA	CFSAN032660	Amsterdam	0	0	0	0	0	0	0	0	0	0	0
SAL_FA7805AA	CFSAN032658	Amsterdam	0	0	0	0	0	0	0	0	0	0	0
SAL_PA3304AA	FSIS31800339	Amsterdam	0	0	0	0	0	0	0	0	0	0	0
SAL_UA0001AA	607328	Amsterdam	0	0	0	0	0	0	0	0	0	0	0
SAL_UA2242AA	O-ZG246	Amsterdam	0	0	0	0	0	0	0	0	0	0	0
SAL_UA3580AA	HC-087	Amsterdam	0	0	0	0	0	0	0	0	0	0	0
SAL_UA3576AA	HC-130	Amsterdam	0	0	0	0	0	0	0	0	0	0	0
SAL_UA3559AA	HS-126	Amsterdam	0	0	0	0	0	0	0	0	0	0	0
SAL_UA3566AA	HC-232	Amsterdam	0	0	0	0	0	0	0	0	0	0	0
SAL_UA3572AA	HC-086	Amsterdam	0	0	0	0	0	0	0	0	0	0	0
SAL_UA3561AA	HC-246	Amsterdam	0	0	0	0	0	0	0	0	0	0	0
SAL_UA5951AA	654703	Amsterdam	0	0	0	0	0	0	0	0	0	0	0
SAL_UA8119AA	666283	Amsterdam	0	0	0	0	0	0	0	0	0	0	0
SAL_UA9648AA	669743	Amsterdam	0	0	0	0	0	0	0	0	0	0	0
SAL_VA1646AA	SMG-19-157	Amsterdam	0	0	0	0	0	0	0	0	0	0	0
SAL_VA0416AA	FSIS31901472	Amsterdam	0	0	0	0	0	0	0	0	0	0	0
SAL_VA5863AA	SA20110986	Amsterdam	0	0	0	0	0	0	0	0	0	0	0
SAL_VA5812AA	SA20093585	Amsterdam	0	0	0	0	0	0	0	0	0	0	0
SAL_VA5764AA	SA20090680	Amsterdam	0	0	0	0	0	0	0	0	0	0	0
SAL_WA2625AA	QA05/14	Amsterdam	0	0	0	0	0	0	0	0	0	0	0
SAL_EA8380AA	FSIS1504599	Anatum	0	0	0	0	0	0	0	0	0	0	0
SAL_DA8666AA	FDN0033	Anatum	0	0	0	0	0	0	0	0	0	0	0
SAL_BA3756AA	CFSAN003960	Anatum	0	0	0	0	0	0	0	0	0	0	0
SAL_DA6864AA	CFSAN003978	Anatum	0	0	0	0	0	0	0	0	0	0	0
SAL_BA0775AA	CFSAN031385	Anatum	0	0	0	0	0	0	0	0	0	0	0
SAL_EA0515AA	AZ_TG68507	Anatum	0	0	0	0	0	0	0	0	0	0	0
SAL_EA8793AA	FSIS1504947	Anatum	0	0	0	0	0	0	0	0	0	0	0
SAL_EA9890AA	FSIS1605555	Anatum	0	0	0	0	0	0	0	0	0	0	0
SAL_BA3944AA	CFSAN004015	Anatum	0	0	0	0	0	0	0	0	0	0	0

SAL_EA5595AA	FLUFL-1944	Anatum	0	0	0	0	0	0	0	0	0	0	0
SAL_FA6000AA	CFSAN045325	Anatum	0	0	0	0	0	0	0	0	0	0	0
SAL_FA5980AA	CFSAN045304	Anatum	0	0	0	0	0	0	0	0	0	0	0
SAL_FA5972AA	CFSAN045312	Anatum	0	0	0	0	0	0	0	0	0	0	0
SAL_FA5981AA	CFSAN045303	Anatum	0	0	0	0	0	0	0	0	0	0	0
SAL_FA5977AA	CFSAN045307	Anatum	0	0	0	0	0	0	0	0	0	0	0
SAL_FA5982AA	CFSAN045301	Anatum	0	0	0	0	0	0	0	0	0	0	0
SAL_FA5975AA	CFSAN045309	Anatum	0	0	0	0	0	0	0	0	0	0	0
SAL_FA5964AA	CFSAN045320	Anatum	0	0	0	0	0	0	0	0	0	0	0
SAL_FA5932AA	CFSAN048157	Anatum	0	0	0	0	0	0	0	0	0	0	0
SAL_FA5931AA	CFSAN048158	Anatum	0	0	0	0	0	0	0	0	0	0	0
SAL_FA5705AA	FDA00004514	Anatum	0	0	0	0	0	0	0	0	0	0	0
SAL_FA5697AA	FDA00007888	Anatum	0	0	0	0	0	0	0	0	0	0	0
SAL_FA5685AA	FDA00003896	Anatum	0	0	0	0	0	0	0	0	0	0	0
SAL_FA5613AA	CFSAN032082	Anatum	0	0	0	0	0	0	0	0	0	0	0
SAL_FA5616AA	CFSAN032089	Anatum	0	0	0	0	0	0	0	0	0	0	0
SAL_FA5446AA	FSIS1606274	Anatum	0	0	0	0	0	0	0	0	0	0	0
SAL_FA5444AA	FSIS1606276	Anatum	0	0	0	0	0	0	0	0	0	0	0
SAL_FA5441AA	FSIS1606281	Anatum	0	0	0	0	0	0	0	0	0	0	0
SAL_FA5431AA	FSIS1606181	Anatum	0	0	0	0	0	0	0	0	0	0	0
SAL_FA5424AA	FSIS1606289	Anatum	0	0	0	0	0	0	0	0	0	0	0
SAL_FA5426AA	FSIS1606183	Anatum	0	0	0	0	0	0	0	0	0	0	0
SAL_FA5417AA	FSIS1606191	Anatum	0	0	0	0	0	0	0	0	0	0	0
SAL_FA5421AA	FSIS1606288	Anatum	0	0	0	0	0	0	0	0	0	0	0
SAL_FA5419AA	FSIS1606197	Anatum	0	0	0	0	0	0	0	0	0	0	0
SAL_FA5385AA	JSDA-ARS-USMARC-1765	Anatum	0	0	0	0	0	0	0	0	0	0	0
SAL_FA5386AA	JSDA-ARS-USMARC-1736	Anatum	0	0	0	0	0	0	0	0	0	0	0
SAL_FA5384AA	JSDA-ARS-USMARC-1783	Anatum	0	0	0	0	0	0	0	0	0	0	0
SAL_FA5152AA	FSIS1606260	Anatum	0	0	0	0	0	0	0	0	0	0	0
SAL_FA4821AA	CVM-N19806	Anatum	0	0	0	0	0	0	0	0	0	0	0
SAL_FA4781AA	CFSAN032043	Anatum	0	0	0	0	0	0	0	0	0	0	0
SAL_FA4780AA	CFSAN032048	Anatum	0	0	0	0	0	0	0	0	0	0	0
SAL_FA4769AA	FSIS1606119	Anatum	0	0	0	0	0	0	0	0	0	0	0
SAL_FA4763AA	FSIS1606080	Anatum	0	0	0	0	0	0	0	0	0	0	0
SAL_FA4755AA	FSIS1606122	Anatum	0	0	0	0	0	0	0	0	0	0	0
SAL_FA4766AA	FSIS1606074	Anatum	0	0	0	0	0	0	0	0	0	0	0
SAL_FA4753AA	FSIS1606114	Anatum	0	0	0	0	0	0	0	0	0	0	0
SAL_FA4714AA	164932	Anatum	0	0	0	0	0	0	0	0	0	0	0
SAL_FA4347AA	140635	Anatum	0	0	0	0	0	0	0	0	0	0	0
SAL_FA4299AA	172420	Anatum	0	0	0	0	0	0	0	0	0	0	0
SAL_FA4218AA	176273	Anatum	0	0	0	0	0	0	0	0	0	0	0
SAL_FA4100AA	161066	Anatum	0	0	0	0	0	0	0	0	0	0	0
SAL_FA4056AA	FSIS1606130	Anatum	0	0	0	0	0	0	0	0	0	0	0
SAL_FA4051AA	FSIS1606129	Anatum	0	0	0	0	0	0	0	0	0	0	0
SAL_FA4050AA	FSIS1606131	Anatum	0	0	0	0	0	0	0	0	0	0	0
SAL_FA4008AA	FDA00002924	Anatum	0	0	0	0	0	0	0	0	0	0	0
SAL_FA3253AA	FSIS1606030	Anatum	0	0	0	0	0	0	0	0	0	0	0
SAL_FA3260AA	FSIS1606027	Anatum	0	0	0	0	0	0	0	0	0	0	0
SAL_FA3261AA	FSIS1606024	Anatum	0	0	0	0	0	0	0	0	0	0	0
SAL_FA3254AA	FSIS1606028	Anatum	0	0	0	0	0	0	0	0	0	0	0
SAL_FA2989AA	CVM N56921F	Anatum	0	0	0	0	0	0	0	0	0	0	0
SAL_FA2975AA	CVM N56967F	Anatum	0	0	0	0	0	0	0	0	0	0	0
SAL_FA2981AA	CVM N56949F	Anatum	0	0	0	0	0	0	0	0	0	0	0
SAL_FA2962AA	CVM N57020F	Anatum	0	0	0	0	0	0	0	0	0	0	0
SAL_FA2967AA	CVM N57003F	Anatum	0	0	0	0	0	0	0	0	0	0	0
SAL_FA2973AA	CVM N56974F	Anatum	0	0	0	0	0	0	0	0	0	0	0
SAL_FA2966AA	CVM N57007F	Anatum	0	0	0	0	0	0	0	0	0	0	0
SAL_FA2958AA	CVM N57043F	Anatum	0	0	0	0	0	0	0	0	0	0	0
SAL_FA2968AA	CVM N56996F	Anatum	0	0	0	0	0	0	0	0	0	0	0
SAL_FA2972AA	CVM N56978F	Anatum	0	0	0	0	0	0	0	0	0	0	0
SAL_FA2964AA	CVM N57010F	Anatum	0	0	0	0	0	0	0	0	0	0	0
SAL_FA2963AA	CVM N57017F	Anatum	0	0	0	0	0	0	0	0	0	0	0
SAL_FA2946AA	CVM N57072F	Anatum	0	0	0	0	0	0	0	0	0	0	0
SAL_FA2943AA	CVM N57080F	Anatum	0	0	0	0	0	0	0	0	0	0	0
SAL_FA2934AA	CVM N57118F	Anatum	0	0	0	0	0	0	0	0	0	0	0
SAL_FA2932AA	CVM N57127F	Anatum	0	0	0	0	0	0	0	0	0	0	0
SAL_FA2915AA	CVM N57189F	Anatum	0	0	0	0	0	0	0	0	0	0	0
SAL_FA2904AA	CVM N57215F	Anatum	0	0	0	0	0	0	0	0	0	0	0
SAL_FA2907AA	CVM N57208F	Anatum	0	0	0	0	0	0	0	0	0	0	0
SAL_FA2896AA	CVM N57238F	Anatum	0	0	0	0	0	0	0	0	0	0	0
SAL_FA2885AA	CVM N57277F	Anatum	0	0	0	0	0	0	0	0	0	0	0
SAL_FA2886AA	CVM N57274F	Anatum	0	0	0	0	0	0	0	0	0	0	0
SAL_FA2889AA	CVM N57263F	Anatum	0	0	0	0	0	0	0	0	0	0	0

SAL_FA2881AA	CVM N57291F	Anatum	0	0	0	0	0	0	0	0	0	0	0
SAL_FA2868AA	CVM N57335F	Anatum	0	0	0	0	0	0	0	0	0	0	0
SAL_FA2865AA	CVM N57343F	Anatum	0	0	0	0	0	0	0	0	0	0	0
SAL_FA2862AA	CVM N57350F	Anatum	0	0	0	0	0	0	0	0	0	0	0
SAL_FA2857AA	CVM N57361F	Anatum	0	0	0	0	0	0	0	0	0	0	0
SAL_FA2845AA	CVM N57396F	Anatum	0	0	0	0	0	0	0	0	0	0	0
SAL_FA2835AA	CVM N57441F	Anatum	0	0	0	0	0	0	0	0	0	0	0
SAL_FA2852AA	CVM N57384F	Anatum	0	0	0	0	0	0	0	0	0	0	0
SAL_FA2842AA	CVM N57405F	Anatum	0	0	0	0	0	0	0	0	0	0	0
SAL_FA2821AA	CVM N57490F	Anatum	0	0	0	0	0	0	0	0	0	0	0
SAL_FA2830AA	CVM N57458F	Anatum	0	0	0	0	0	0	0	0	0	0	0
SAL_FA2825AA	CVM N57480F	Anatum	0	0	0	0	0	0	0	0	0	0	0
SAL_FA2801AA	CVM N57583F	Anatum	0	0	0	0	0	0	0	0	0	0	0
SAL_FA2805AA	CVM N57563F	Anatum	0	0	0	0	0	0	0	0	0	0	0
SAL_FA2789AA	CVM N57613F	Anatum	0	0	0	0	0	0	0	0	0	0	0
SAL_FA2782AA	CVM N57627F	Anatum	0	0	0	0	0	0	0	0	0	0	0
SAL_FA2766AA	CVM N57665F	Anatum	0	0	0	0	0	0	0	0	0	0	0
SAL_FA2754AA	CVM N57701F	Anatum	0	0	0	0	0	0	0	0	0	0	0
SAL_FA2751AA	CVM N57705F	Anatum	0	0	0	0	0	0	0	0	0	0	0
SAL_FA2738AA	CVM N57740F	Anatum	0	0	0	0	0	0	0	0	0	0	0
SAL_FA2711AA	CVM N57831F	Anatum	0	0	0	0	0	0	0	0	0	0	0
SAL_FA2714AA	CVM N57824F	Anatum	0	0	0	0	0	0	0	0	0	0	0
SAL_FA2707AA	CVM N57840F	Anatum	0	0	0	0	0	0	0	0	0	0	0
SAL_FA2700AA	CVM N57858F	Anatum	0	0	0	0	0	0	0	0	0	0	0
SAL_FA2693AA	CVM N57890F	Anatum	0	0	0	0	0	0	0	0	0	0	0
SAL_FA2681AA	CVM N57934F	Anatum	0	0	0	0	0	0	0	0	0	0	0
SAL_FA2675AA	CVM N57952F	Anatum	0	0	0	0	0	0	0	0	0	0	0
SAL_FA2676AA	CVM N57946F	Anatum	0	0	0	0	0	0	0	0	0	0	0
SAL_FA2659AA	CVM N58623F	Anatum	0	0	0	0	0	0	0	0	0	0	0
SAL_FA2663AA	CVM N58617F	Anatum	0	0	0	0	0	0	0	0	0	0	0
SAL_FA2614AA	CFSAN047726	Anatum	0	0	0	0	0	0	0	0	0	0	0
SAL_FA2615AA	CFSAN047725	Anatum	0	0	0	0	0	0	0	0	0	0	0
SAL_FA2611AA	IEH-NGS-SAL-03875	Anatum	0	0	0	0	0	0	0	0	0	0	0
SAL_FA2535AA	PNUSAS001766	Anatum	0	0	0	0	0	0	0	0	0	0	0
SAL_FA2466AA	FSIS1605974	Anatum	0	0	0	0	0	0	0	0	0	0	0
SAL_FA2465AA	FSIS1605976	Anatum	0	0	0	0	0	0	0	0	0	0	0
SAL_FA2267AA	FDA00003007	Anatum	0	0	0	0	0	0	0	0	0	0	0
SAL_FA2252AA	DPHFDLB-F1602021-013	Anatum	0	0	0	0	0	0	0	0	0	0	0
SAL_FA2253AA	DPHFDLB-F1602021-013	Anatum	0	0	0	0	0	0	0	0	0	0	0
SAL_FA2200AA	FSIS1605903	Anatum	0	0	0	0	0	0	0	0	0	0	0
SAL_FA2196AA	FSIS1605908	Anatum	0	0	0	0	0	0	0	0	0	0	0
SAL_FA2163AA	FSIS1605831	Anatum	0	0	0	0	0	0	0	0	0	0	0
SAL_FA2158AA	FSIS1606017	Anatum	0	0	0	0	0	0	0	0	0	0	0
SAL_FA2156AA	FSIS1606020	Anatum	0	0	0	0	0	0	0	0	0	0	0
SAL_FA1886AA	FSIS1605896	Anatum	0	0	0	0	0	0	0	0	0	0	0
SAL_FA1887AA	FSIS1605899	Anatum	0	0	0	0	0	0	0	0	0	0	0
SAL_FA1885AA	FSIS1605744	Anatum	0	0	0	0	0	0	0	0	0	0	0
SAL_FA1879AA	FSIS1605822	Anatum	0	0	0	0	0	0	0	0	0	0	0
SAL_FA1878AA	FSIS1605828	Anatum	0	0	0	0	0	0	0	0	0	0	0
SAL_FA1634AA	FSIS1605909	Anatum	0	0	0	0	0	0	0	0	0	0	0
SAL_FA1598AA	CFSAN045740	Anatum	0	0	0	0	0	0	0	0	0	0	0
SAL_FA1410AA	FSIS1502170	Anatum	0	0	0	0	0	0	0	0	0	0	0
SAL_FA1409AA	FSIS1605458	Anatum	0	0	0	0	0	0	0	0	0	0	0
SAL_FA1403AA	FSIS1605739	Anatum	0	0	0	0	0	0	0	0	0	0	0
SAL_FA1405AA	FSIS1605737	Anatum	0	0	0	0	0	0	0	0	0	0	0
SAL_FA1201AA	IEH-NGS-SAL-03550	Anatum	0	0	0	0	0	0	0	0	0	0	0
SAL_FA1197AA	IEH-NGS-SAL-03554	Anatum	0	0	0	0	0	0	0	0	0	0	0
SAL_FA1198AA	IEH-NGS-SAL-03553	Anatum	0	0	0	0	0	0	0	0	0	0	0
SAL_FA1147AA	NY-N20112	Anatum	0	0	0	0	0	0	0	0	0	0	0
SAL_FA1138AA	FSIS1605746	Anatum	0	0	0	0	0	0	0	0	0	0	0
SAL_FA1135AA	FSIS1605750	Anatum	0	0	0	0	0	0	0	0	0	0	0
SAL_FA1080AA	PNUSAS001542	Anatum	0	0	0	0	0	0	0	0	0	0	0
SAL_FA1079AA	PNUSAS001543	Anatum	0	0	0	0	0	0	0	0	0	0	0
SAL_FA0911AA	FDA00007886	Anatum	0	0	0	0	0	0	0	0	0	0	0
SAL_FA0888AA	FSIS1605675	Anatum	0	0	0	0	0	0	0	0	0	0	0
SAL_FA0894AA	FSIS1605674	Anatum	0	0	0	0	0	0	0	0	0	0	0
SAL_FA0905AA	FSIS1605573	Anatum	0	0	0	0	0	0	0	0	0	0	0
SAL_FA0852AA	FSIS1605659	Anatum	0	0	0	0	0	0	0	0	0	0	0
SAL_FA0842AA	FSIS1605668	Anatum	0	0	0	0	0	0	0	0	0	0	0
SAL_FA0835AA	FSIS1605662	Anatum	0	0	0	0	0	0	0	0	0	0	0
SAL_FA0815AA	FDA00009875	Anatum	0	0	0	0	0	0	0	0	0	0	0
SAL_FA0817AA	FDA00009873	Anatum	0	0	0	0	0	0	0	0	0	0	0
SAL_FA0816AA	FDA00009874	Anatum	0	0	0	0	0	0	0	0	0	0	0

SAL_FA0814AA	FDA00009876	Anatum	0	0	0	0	0	0	0	0	0	0	0
SAL_FA0492AA	FSIS1505205	Anatum	0	0	0	0	0	0	0	0	0	0	0
SAL_FA0493AA	FSIS1505228	Anatum	0	0	0	0	0	0	0	0	0	0	0
SAL_FA0501AA	FSIS1605446	Anatum	0	0	0	0	0	0	0	0	0	0	0
SAL_FA0396AA	FSIS1605552	Anatum	0	0	0	0	0	0	0	0	0	0	0
SAL_EA9896AA	FSIS1605554	Anatum	0	0	0	0	0	0	0	0	0	0	0
SAL_EA9888AA	FSIS1605556	Anatum	0	0	0	0	0	0	0	0	0	0	0
SAL_EA9887AA	FSIS1605560	Anatum	0	0	0	0	0	0	0	0	0	0	0
SAL_EA9879AA	FSIS1605441	Anatum	0	0	0	0	0	0	0	0	0	0	0
SAL_EA9874AA	FSIS1605428	Anatum	0	0	0	0	0	0	0	0	0	0	0
SAL_EA9742AA	JSDA-ARS-USMARC-1764	Anatum	0	0	0	0	0	0	0	0	0	0	0
SAL_EA9737AA	JSDA-ARS-USMARC-1175	Anatum	0	0	0	0	0	0	0	0	0	0	0
SAL_EA9733AA	JSDA-ARS-USMARC-1735	Anatum	0	0	0	0	0	0	0	0	0	0	0
SAL_EA9562AA	FSIS1605679	Anatum	0	0	0	0	0	0	0	0	0	0	0
SAL_EA9486AA	MOD1-GC-12	Anatum	0	0	0	0	0	0	0	0	0	0	0
SAL_EA9485AA	MOD1-GC-11	Anatum	0	0	0	0	0	0	0	0	0	0	0
SAL_EA9265AA	FSIS1504813	Anatum	0	0	0	0	0	0	0	0	0	0	0
SAL_EA9089AA	FSIS1501186	Anatum	0	0	0	0	0	0	0	0	0	0	0
SAL_EA9088AA	FSIS1501192	Anatum	0	0	0	0	0	0	0	0	0	0	0
SAL_EA9087AA	FSIS1501188	Anatum	0	0	0	0	0	0	0	0	0	0	0
SAL_EA8820AA	FDA00001968	Anatum	0	0	0	0	0	0	0	0	0	0	0
SAL_EA8802AA	FSIS1504938	Anatum	0	0	0	0	0	0	0	0	0	0	0
SAL_EA8799AA	FSIS1504932	Anatum	0	0	0	0	0	0	0	0	0	0	0
SAL_EA8796AA	FSIS1504965	Anatum	0	0	0	0	0	0	0	0	0	0	0
SAL_EA8794AA	FSIS1504956	Anatum	0	0	0	0	0	0	0	0	0	0	0
SAL_EA8790AA	FSIS1504954	Anatum	0	0	0	0	0	0	0	0	0	0	0
SAL_EA8788AA	FSIS1505006	Anatum	0	0	0	0	0	0	0	0	0	0	0
SAL_EA8787AA	FSIS1505008	Anatum	0	0	0	0	0	0	0	0	0	0	0
SAL_EA8783AA	FSIS1504768	Anatum	0	0	0	0	0	0	0	0	0	0	0
SAL_EA8779AA	FSIS1504810	Anatum	0	0	0	0	0	0	0	0	0	0	0
SAL_EA8762AA	TX-ACF1503392	Anatum	0	0	0	0	0	0	0	0	0	0	0
SAL_EA8656AA	NY-N19875	Anatum	0	0	0	0	0	0	0	0	0	0	0
SAL_EA8600AA	POR1-0160	Anatum	0	0	0	0	0	0	0	0	0	0	0
SAL_EA8592AA	POR1-0186	Anatum	0	0	0	0	0	0	0	0	0	0	0
SAL_EA8474AA	FSIS1504483	Anatum	0	0	0	0	0	0	0	0	0	0	0
SAL_EA8480AA	FSIS1504484	Anatum	0	0	0	0	0	0	0	0	0	0	0
SAL_EA8470AA	FSIS1505026	Anatum	0	0	0	0	0	0	0	0	0	0	0
SAL_EA8469AA	FSIS1505112	Anatum	0	0	0	0	0	0	0	0	0	0	0
SAL_EA8382AA	FSIS1504673	Anatum	0	0	0	0	0	0	0	0	0	0	0
SAL_EA8037AA	56963	Anatum	0	0	0	0	0	0	0	0	0	0	0
SAL_EA7995AA	43908	Anatum	0	0	0	0	0	0	0	0	0	0	0
SAL_EA7934AA	43889	Anatum	0	0	0	0	0	0	0	0	0	0	0
SAL_EA7770AA	63681	Anatum	0	0	0	0	0	0	0	0	0	0	0
SAL_EA7758AA	53024	Anatum	0	0	0	0	0	0	0	0	0	0	0
SAL_EA7670AA	82537	Anatum	0	0	0	0	0	0	0	0	0	0	0
SAL_EA7298AA	99739	Anatum	0	0	0	0	0	0	0	0	0	0	0
SAL_EA7138AA	3776	Anatum	0	0	0	0	0	0	0	0	0	0	0
SAL_EA7099AA	37085	Anatum	0	0	0	0	0	0	0	0	0	0	0
SAL_EA6957AA	20912	Anatum	0	0	0	0	0	0	0	0	0	0	0
SAL_EA6752AA	VA-WGS-00484	Anatum	0	0	0	0	0	0	0	0	0	0	0
SAL_EA6749AA	VA-WGS-00487	Anatum	0	0	0	0	0	0	0	0	0	0	0
SAL_EA6603AA	FSIS1504188	Anatum	0	0	0	0	0	0	0	0	0	0	0
SAL_EA6602AA	FSIS1503719	Anatum	0	0	0	0	0	0	0	0	0	0	0
SAL_EA6577AA	FSIS1505175	Anatum	0	0	0	0	0	0	0	0	0	0	0
SAL_EA6532AA	FDA00000306	Anatum	0	0	0	0	0	0	0	0	0	0	0
SAL_EA6513AA	FDA00007880	Anatum	0	0	0	0	0	0	0	0	0	0	0
SAL_EA6473AA	FSIS1504213	Anatum	0	0	0	0	0	0	0	0	0	0	0
SAL_EA6385AA	FSIS1503563	Anatum	0	0	0	0	0	0	0	0	0	0	0
SAL_EA6365AA	FSIS1503949	Anatum	0	0	0	0	0	0	0	0	0	0	0
SAL_EA6371AA	FSIS1504578	Anatum	0	0	0	0	0	0	0	0	0	0	0
SAL_EA6368AA	FSIS1503941	Anatum	0	0	0	0	0	0	0	0	0	0	0
SAL_EA6360AA	FSIS1503950	Anatum	0	0	0	0	0	0	0	0	0	0	0
SAL_EA6331AA	FSIS1505157	Anatum	0	0	0	0	0	0	0	0	0	0	0
SAL_EA6332AA	FSIS1505163	Anatum	0	0	0	0	0	0	0	0	0	0	0
SAL_EA6283AA	MOD1-Lipp-492	Anatum	0	0	0	0	0	0	0	0	0	0	0
SAL_EA6174AA	ADRDL-15-10605	Anatum	0	0	0	0	0	0	0	0	0	0	0
SAL_EA6143AA	FSIS1501524	Anatum	0	0	0	0	0	0	0	0	0	0	0
SAL_EA6140AA	FSIS1501527	Anatum	0	0	0	0	0	0	0	0	0	0	0
SAL_EA6148AA	FSIS1501060	Anatum	0	0	0	0	0	0	0	0	0	0	0
SAL_EA6149AA	FSIS1501062	Anatum	0	0	0	0	0	0	0	0	0	0	0
SAL_EA6136AA	FSIS1501293	Anatum	0	0	0	0	0	0	0	0	0	0	0
SAL_EA6133AA	FSIS1501532	Anatum	0	0	0	0	0	0	0	0	0	0	0
SAL_EA6105AA	FSIS1501541	Anatum	0	0	0	0	0	0	0	0	0	0	0

SAL_EA6102AA	FSIS1501868	Anatum	0	0	0	0	0	0	0	0	0	0	0
SAL_EA6101AA	FSIS1501931	Anatum	0	0	0	0	0	0	0	0	0	0	0
SAL_EA5986AA	FLUFL-1950	Anatum	0	0	0	0	0	0	0	0	0	0	0
SAL_EA5960AA	ADRDL-15-11061	Anatum	0	0	0	0	0	0	0	0	0	0	0
SAL_EA5947AA	VA-WGS-00630	Anatum	0	0	0	0	0	0	0	0	0	0	0
SAL_EA5942AA	VA-WGS-00635	Anatum	0	0	0	0	0	0	0	0	0	0	0
SAL_EA5896AA	FSIS1504462	Anatum	0	0	0	0	0	0	0	0	0	0	0
SAL_EA5895AA	FSIS1504464	Anatum	0	0	0	0	0	0	0	0	0	0	0
SAL_EA5901AA	FSIS1504458	Anatum	0	0	0	0	0	0	0	0	0	0	0
SAL_EA5739AA	FSIS1503041	Anatum	0	0	0	0	0	0	0	0	0	0	0
SAL_EA5733AA	FSIS1503032	Anatum	0	0	0	0	0	0	0	0	0	0	0
SAL_EA5731AA	FSIS1503046	Anatum	0	0	0	0	0	0	0	0	0	0	0
SAL_EA5726AA	FSIS1503271	Anatum	0	0	0	0	0	0	0	0	0	0	0
SAL_EA5720AA	FSIS1502610	Anatum	0	0	0	0	0	0	0	0	0	0	0
SAL_EA5725AA	FSIS1503279	Anatum	0	0	0	0	0	0	0	0	0	0	0
SAL_EA5612AA	FSIS1503488	Anatum	0	0	0	0	0	0	0	0	0	0	0
SAL_EA5597AA	FLUFL-1942	Anatum	0	0	0	0	0	0	0	0	0	0	0
SAL_EA5594AA	FLUFL-1945	Anatum	0	0	0	0	0	0	0	0	0	0	0
SAL_EA5591AA	FLUFL-1948	Anatum	0	0	0	0	0	0	0	0	0	0	0
SAL_EA5589AA	VA-WGS-00525	Anatum	0	0	0	0	0	0	0	0	0	0	0
SAL_EA5517AA	MOD1-lc-26	Anatum	0	0	0	0	0	0	0	0	0	0	0
SAL_EA5514AA	FSIS1504924	Anatum	0	0	0	0	0	0	0	0	0	0	0
SAL_EA5511AA	FSIS1504927	Anatum	0	0	0	0	0	0	0	0	0	0	0
SAL_EA5510AA	FSIS1504919	Anatum	0	0	0	0	0	0	0	0	0	0	0
SAL_EA5399AA	POR1-0145	Anatum	0	0	0	0	0	0	0	0	0	0	0
SAL_EA5326AA	FSIS1503458	Anatum	0	0	0	0	0	0	0	0	0	0	0
SAL_EA5211AA	FLUFL-1930	Anatum	0	0	0	0	0	0	0	0	0	0	0
SAL_EA5209AA	FLUFL-1932	Anatum	0	0	0	0	0	0	0	0	0	0	0
SAL_EA5204AA	FLUFL-1937	Anatum	0	0	0	0	0	0	0	0	0	0	0
SAL_EA5203AA	FLUFL-1938	Anatum	0	0	0	0	0	0	0	0	0	0	0
SAL_EA5205AA	FLUFL-1936	Anatum	0	0	0	0	0	0	0	0	0	0	0
SAL_EA5168AA	VA-WGS-00506	Anatum	0	0	0	0	0	0	0	0	0	0	0
SAL_EA4711AA	CFSAN033311	Anatum	0	0	0	0	0	0	0	0	0	0	0
SAL_EA4710AA	CFSAN033312	Anatum	0	0	0	0	0	0	0	0	0	0	0
SAL_EA4712AA	CFSAN033310	Anatum	0	0	0	0	0	0	0	0	0	0	0
SAL_EA4709AA	CFSAN033313	Anatum	0	0	0	0	0	0	0	0	0	0	0
SAL_EA4707AA	IEH-NGS-SAL-03126	Anatum	0	0	0	0	0	0	0	0	0	0	0
SAL_EA4708AA	CFSAN033314	Anatum	0	0	0	0	0	0	0	0	0	0	0
SAL_EA4669AA	CFSAN033306	Anatum	0	0	0	0	0	0	0	0	0	0	0
SAL_EA4667AA	CFSAN033308	Anatum	0	0	0	0	0	0	0	0	0	0	0
SAL_EA3956AA	FSIS1504696	Anatum	0	0	0	0	0	0	0	0	0	0	0
SAL_EA3553AA	CVM N51307	Anatum	0	0	0	0	0	0	0	0	0	0	0
SAL_EA3208AA	FDA00001523	Anatum	0	0	0	0	0	0	0	0	0	0	0
SAL_EA3125AA	FLUFL-1913	Anatum	0	0	0	0	0	0	0	0	0	0	0
SAL_EA3115AA	FLUFL-1923	Anatum	0	0	0	0	0	0	0	0	0	0	0
SAL_EA3128AA	FLUFL-1910	Anatum	0	0	0	0	0	0	0	0	0	0	0
SAL_EA3120AA	FLUFL-1918	Anatum	0	0	0	0	0	0	0	0	0	0	0
SAL_EA3113AA	FLUFL-1925	Anatum	0	0	0	0	0	0	0	0	0	0	0
SAL_EA3056AA	TX-ACF1502134	Anatum	0	0	0	0	0	0	0	0	0	0	0
SAL_EA3035AA	FSIS1502802	Anatum	0	0	0	0	0	0	0	0	0	0	0
SAL_EA2457AA	FDA00009339	Anatum	0	0	0	0	0	0	0	0	0	0	0
SAL_EA2382AA	FLUFL-1895	Anatum	0	0	0	0	0	0	0	0	0	0	0
SAL_EA2381AA	FLUFL-1896	Anatum	0	0	0	0	0	0	0	0	0	0	0
SAL_EA2383AA	FLUFL-1894	Anatum	0	0	0	0	0	0	0	0	0	0	0
SAL_EA2376AA	FLUFL-1901	Anatum	0	0	0	0	0	0	0	0	0	0	0
SAL_EA2375AA	FLUFL-1902	Anatum	0	0	0	0	0	0	0	0	0	0	0
SAL_EA2370AA	FLUFL-1907	Anatum	0	0	0	0	0	0	0	0	0	0	0
SAL_EA2378AA	FLUFL-1899	Anatum	0	0	0	0	0	0	0	0	0	0	0
SAL_EA2377AA	FLUFL-1900	Anatum	0	0	0	0	0	0	0	0	0	0	0
SAL_EA2371AA	FLUFL-1906	Anatum	0	0	0	0	0	0	0	0	0	0	0
SAL_EA2346AA	VA-WGS-00618	Anatum	0	0	0	0	0	0	0	0	0	0	0
SAL_EA2124AA	CVM N52034	Anatum	0	0	0	0	0	0	0	0	0	0	0
SAL_EA1942AA	CVM N54721	Anatum	0	0	0	0	0	0	0	0	0	0	0
SAL_EA1877AA	CVM N55824	Anatum	0	0	0	0	0	0	0	0	0	0	0
SAL_EA1783AA	FSIS1503888	Anatum	0	0	0	0	0	0	0	0	0	0	0
SAL_EA1781AA	FSIS1503889	Anatum	0	0	0	0	0	0	0	0	0	0	0
SAL_EA1662AA	FSIS1500751	Anatum	0	0	0	0	0	0	0	0	0	0	0
SAL_EA1661AA	FSIS1500744	Anatum	0	0	0	0	0	0	0	0	0	0	0
SAL_EA1475AA	FSIS1500652	Anatum	0	0	0	0	0	0	0	0	0	0	0
SAL_EA1471AA	FSIS1500715	Anatum	0	0	0	0	0	0	0	0	0	0	0
SAL_EA1442AA	FSIS1500759	Anatum	0	0	0	0	0	0	0	0	0	0	0
SAL_EA1437AA	FSIS1500758	Anatum	0	0	0	0	0	0	0	0	0	0	0
SAL_EA1440AA	FSIS1500763	Anatum	0	0	0	0	0	0	0	0	0	0	0

SAL_EA1346AA	AZ_TG68288	Anatum	0	0	0	0	0	0	0	0	0	0	0
SAL_EA1324AA	FL_FLDACS-13137	Anatum	0	0	0	0	0	0	0	0	0	0	0
SAL_EA1326AA	FL_FLDACS-13061	Anatum	0	0	0	0	0	0	0	0	0	0	0
SAL_EA1321AA	FL_FLDACS-13136	Anatum	0	0	0	0	0	0	0	0	0	0	0
SAL_EA1316AA	FL_FLDACS-13063	Anatum	0	0	0	0	0	0	0	0	0	0	0
SAL_EA1110AA	M575 / NCTC20263	Anatum	0	0	0	0	0	0	0	0	0	0	0
SAL_EA0787AA	FL_FLDACS-48396	Anatum	0	0	0	0	0	0	0	0	0	0	0
SAL_EA0775AA	FL_FLDACS-01561-Sub-A	Anatum	0	0	0	0	0	0	0	0	0	0	0
SAL_EA0744AA	FCC0007	Anatum	0	0	0	0	0	0	0	0	0	0	0
SAL_EA0599AA	FL_EPI-023	Anatum	0	0	0	0	0	0	0	0	0	0	0
SAL_EA0581AA	FL_FLDACS-17821	Anatum	0	0	0	0	0	0	0	0	0	0	0
SAL_EA0565AA	FCC0039	Anatum	0	0	0	0	0	0	0	0	0	0	0
SAL_EA0492AA	FSF0032	Anatum	0	0	0	0	0	0	0	0	0	0	0
SAL_EA0485AA	FCC0043	Anatum	0	0	0	0	0	0	0	0	0	0	0
SAL_EA0434AA	FCC0089	Anatum	0	0	0	0	0	0	0	0	0	0	0
SAL_EA0421AA	AZ_TG68499	Anatum	0	0	0	0	0	0	0	0	0	0	0
SAL_EA0413AA	AZ_TG68503	Anatum	0	0	0	0	0	0	0	0	0	0	0
SAL_EA0319AA	FSW0061	Anatum	0	0	0	0	0	0	0	0	0	0	0
SAL_EA0267AA	FDN0025	Anatum	0	0	0	0	0	0	0	0	0	0	0
SAL_EA0234AA	FSW0094	Anatum	0	0	0	0	0	0	0	0	0	0	0
SAL_EA0213AA	FL-UFL-183	Anatum	0	0	0	0	0	0	0	0	0	0	0
SAL_EA0185AA	FSF0007	Anatum	0	0	0	0	0	0	0	0	0	0	0
SAL_EA0117AA	NY_IDR1000034294	Anatum	0	0	0	0	0	0	0	0	0	0	0
SAL_EA0125AA	NY_IDR1100009450X1	Anatum	0	0	0	0	0	0	0	0	0	0	0
SAL_DA9917AA	FMA0021	Anatum	0	0	0	0	0	0	0	0	0	0	0
SAL_DA9838AA	NY_BAC0800005752	Anatum	0	0	0	0	0	0	0	0	0	0	0
SAL_DA9689AA	FL_EPI-025	Anatum	0	0	0	0	0	0	0	0	0	0	0
SAL_DA9676AA	FNW19G28	Anatum	0	0	0	0	0	0	0	0	0	0	0
SAL_DA9622AA	FMA0043	Anatum	0	0	0	0	0	0	0	0	0	0	0
SAL_DA9614AA	FAR0063	Anatum	0	0	0	0	0	0	0	0	0	0	0
SAL_DA9605AA	FMA0038	Anatum	0	0	0	0	0	0	0	0	0	0	0
SAL_DA9600AA	FNW19G79	Anatum	0	0	0	0	0	0	0	0	0	0	0
SAL_DA9578AA	FMA0010	Anatum	0	0	0	0	0	0	0	0	0	0	0
SAL_DA9542AA	VA_WGS-00102	Anatum	0	0	0	0	0	0	0	0	0	0	0
SAL_DA9517AA	VA_WGS-00089	Anatum	0	0	0	0	0	0	0	0	0	0	0
SAL_DA9504AA	VA_WGS-00073	Anatum	0	0	0	0	0	0	0	0	0	0	0
SAL_DA9501AA	VA_WGS-00095	Anatum	0	0	0	0	0	0	0	0	0	0	0
SAL_DA9412AA	VA_WGS-00090	Anatum	0	0	0	0	0	0	0	0	0	0	0
SAL_DA9404AA	VA_WGS-00068	Anatum	0	0	0	0	0	0	0	0	0	0	0
SAL_DA9393AA	FL_EPI-027	Anatum	0	0	0	0	0	0	0	0	0	0	0
SAL_DA8887AA	WAPHL-SAL-A00264	Anatum	0	0	0	0	0	0	0	0	0	0	0
SAL_DA8872AA	WAPHL-SAL-A00263	Anatum	0	0	0	0	0	0	0	0	0	0	0
SAL_DA8763AA	FMA0063	Anatum	0	0	0	0	0	0	0	0	0	0	0
SAL_DA8764AA	FMA0026	Anatum	0	0	0	0	0	0	0	0	0	0	0
SAL_DA8661AA	FDN0036	Anatum	0	0	0	0	0	0	0	0	0	0	0
SAL_DA8604AA	FL-MDD-372	Anatum	0	0	0	0	0	0	0	0	0	0	0
SAL_DA8613AA	FDN0053	Anatum	0	0	0	0	0	0	0	0	0	0	0
SAL_DA8610AA	FDN0049	Anatum	0	0	0	0	0	0	0	0	0	0	0
SAL_DA8607AA	FDN0041	Anatum	0	0	0	0	0	0	0	0	0	0	0
SAL_DA8591AA	VA_WGS-00155	Anatum	0	0	0	0	0	0	0	0	0	0	0
SAL_DA8562AA	FSE0091	Anatum	0	0	0	0	0	0	0	0	0	0	0
SAL_DA8543AA	FSE0090	Anatum	0	0	0	0	0	0	0	0	0	0	0
SAL_DA8544AA	VA_WGS-00149	Anatum	0	0	0	0	0	0	0	0	0	0	0
SAL_DA8514AA	FSE0040	Anatum	0	0	0	0	0	0	0	0	0	0	0
SAL_DA8499AA	NY-FSL-R8-0842	Anatum	0	0	0	0	0	0	0	0	0	0	0
SAL_DA8494AA	NY-FSL-R8-0847	Anatum	0	0	0	0	0	0	0	0	0	0	0
SAL_DA8406AA	FNW18t55	Anatum	0	0	0	0	0	0	0	0	0	0	0
SAL_DA8197AA	NY-FSL-R8-0969	Anatum	0	0	0	0	0	0	0	0	0	0	0
SAL_DA8155AA	NY-FSL-R8-0968	Anatum	0	0	0	0	0	0	0	0	0	0	0
SAL_DA8119AA	FAR0077	Anatum	0	0	0	0	0	0	0	0	0	0	0
SAL_DA8083AA	WAPHL-SAL-A00372	Anatum	0	0	0	0	0	0	0	0	0	0	0
SAL_DA8090AA	FAR0109	Anatum	0	0	0	0	0	0	0	0	0	0	0
SAL_DA8061AA	NY-FSL-R6-0928	Anatum	0	0	0	0	0	0	0	0	0	0	0
SAL_DA8035AA	VA_WGS-00185	Anatum	0	0	0	0	0	0	0	0	0	0	0
SAL_DA8026AA	VA_WGS-00181	Anatum	0	0	0	0	0	0	0	0	0	0	0
SAL_DA7976AA	VA_WGS-00193	Anatum	0	0	0	0	0	0	0	0	0	0	0
SAL_DA7862AA	FAR0122	Anatum	0	0	0	0	0	0	0	0	0	0	0
SAL_DA7518AA	FLUFL-182	Anatum	0	0	0	0	0	0	0	0	0	0	0
SAL_DA7471AA	MDH-2014-00082	Anatum	0	0	0	0	0	0	0	0	0	0	0
SAL_DA7404AA	NY-FSL-R8-0348	Anatum	0	0	0	0	0	0	0	0	0	0	0
SAL_DA7366AA	NY-FSL-R8-0187	Anatum	0	0	0	0	0	0	0	0	0	0	0
SAL_DA7321AA	NY-FSL-R8-0350	Anatum	0	0	0	0	0	0	0	0	0	0	0
SAL_DA7308AA	MDH-2014-00055	Anatum	0	0	0	0	0	0	0	0	0	0	0

SAL_DA7276AA	MDH-2014-00059	Anatum	0	0	0	0	0	0	0	0	0	0	0
SAL_DA7215AA	FNW18t80	Anatum	0	0	0	0	0	0	0	0	0	0	0
SAL_DA7200AA	FNW18t90	Anatum	0	0	0	0	0	0	0	0	0	0	0
SAL_DA6990AA	NY-FSL-R8-0905	Anatum	0	0	0	0	0	0	0	0	0	0	0
SAL_DA6949AA	NY-FSL-R8-0907	Anatum	0	0	0	0	0	0	0	0	0	0	0
SAL_DA6869AA	CFSAN003983	Anatum	0	0	0	0	0	0	0	0	0	0	0
SAL_DA6872AA	CFSAN003982	Anatum	0	0	0	0	0	0	0	0	0	0	0
SAL_DA6871AA	CFSAN003949	Anatum	0	0	0	0	0	0	0	0	0	0	0
SAL_DA6865AA	CFSAN003980	Anatum	0	0	0	0	0	0	0	0	0	0	0
SAL_DA6853AA	CFSAN003977	Anatum	0	0	0	0	0	0	0	0	0	0	0
SAL_DA6854AA	CFSAN003976	Anatum	0	0	0	0	0	0	0	0	0	0	0
SAL_DA6870AA	CFSAN003959	Anatum	0	0	0	0	0	0	0	0	0	0	0
SAL_DA6858AA	CFSAN003984	Anatum	0	0	0	0	0	0	0	0	0	0	0
SAL_DA6843AA	CFSAN003985	Anatum	0	0	0	0	0	0	0	0	0	0	0
SAL_DA6851AA	CFSAN003947	Anatum	0	0	0	0	0	0	0	0	0	0	0
SAL_DA6847AA	CFSAN003968	Anatum	0	0	0	0	0	0	0	0	0	0	0
SAL_DA6846AA	CFSAN003981	Anatum	0	0	0	0	0	0	0	0	0	0	0
SAL_DA6849AA	CFSAN003979	Anatum	0	0	0	0	0	0	0	0	0	0	0
SAL_DA6695AA	NY-FSL-R8-1402	Anatum	0	0	0	0	0	0	0	0	0	0	0
SAL_DA6436AA	NY-FSL-R8-3653	Anatum	0	0	0	0	0	0	0	0	0	0	0
SAL_DA6412AA	NY-FSL-R8-3652	Anatum	0	0	0	0	0	0	0	0	0	0	0
SAL_DA6381AA	CFSAN013369	Anatum	0	0	0	0	0	0	0	0	0	0	0
SAL_DA6389AA	IEH-NGS-SAL-00558	Anatum	0	0	0	0	0	0	0	0	0	0	0
SAL_DA6378AA	FAR0090	Anatum	0	0	0	0	0	0	0	0	0	0	0
SAL_DA6380AA	CFSAN013371	Anatum	0	0	0	0	0	0	0	0	0	0	0
SAL_DA6269AA	MDH-2014-00317	Anatum	0	0	0	0	0	0	0	0	0	0	0
SAL_DA6088AA	FCC0155	Anatum	0	0	0	0	0	0	0	0	0	0	0
SAL_DA6048AA	CFSAN018507	Anatum	0	0	0	0	0	0	0	0	0	0	0
SAL_DA5891AA	FSE0194	Anatum	0	0	0	0	0	0	0	0	0	0	0
SAL_DA5650AA	NY-FSL-R8-2860	Anatum	0	0	0	0	0	0	0	0	0	0	0
SAL_DA5470AA	CFSAN018728	Anatum	0	0	0	0	0	0	0	0	0	0	0
SAL_DA5393AA	AZ-TG74412	Anatum	0	0	0	0	0	0	0	0	0	0	0
SAL_DA5118AA	MDH-2014-00395	Anatum	0	0	0	0	0	0	0	0	0	0	0
SAL_DA5107AA	MDH-2014-00403	Anatum	0	0	0	0	0	0	0	0	0	0	0
SAL_DA4432AA	FCC0130	Anatum	0	0	0	0	0	0	0	0	0	0	0
SAL_DA4302AA	MOD1-Sal_897	Anatum	0	0	0	0	0	0	0	0	0	0	0
SAL_DA4279AA	MOD1-Sal_898	Anatum	0	0	0	0	0	0	0	0	0	0	0
SAL_DA4262AA	FLUFL-1327	Anatum	0	0	0	0	0	0	0	0	0	0	0
SAL_DA4259AA	FLUFL-1326	Anatum	0	0	0	0	0	0	0	0	0	0	0
SAL_DA4187AA	CFSAN012625	Anatum	0	0	0	0	0	0	0	0	0	0	0
SAL_DA4152AA	MDH-2014-00424	Anatum	0	0	0	0	0	0	0	0	0	0	0
SAL_DA4112AA	CFSAN023135	Anatum	0	0	0	0	0	0	0	0	0	0	0
SAL_DA4086AA	FLUFL-1328	Anatum	0	0	0	0	0	0	0	0	0	0	0
SAL_DA3924AA	MDH-2014-00462	Anatum	0	0	0	0	0	0	0	0	0	0	0
SAL_DA3853AA	FDA00004261	Anatum	0	0	0	0	0	0	0	0	0	0	0
SAL_DA3852AA	MDH-2014-00473	Anatum	0	0	0	0	0	0	0	0	0	0	0
SAL_DA3422AA	MDH-2014-00488	Anatum	0	0	0	0	0	0	0	0	0	0	0
SAL_DA3423AA	MDH-2014-00487	Anatum	0	0	0	0	0	0	0	0	0	0	0
SAL_DA3003AA	IEH-NGS-SAL-01053	Anatum	0	0	0	0	0	0	0	0	0	0	0
SAL_DA2554AA	MDH-2014-00536	Anatum	0	0	0	0	0	0	0	0	0	0	0
SAL_DA2395AA	FLUFL-1361	Anatum	0	0	0	0	0	0	0	0	0	0	0
SAL_DA2364AA	FDN0073	Anatum	0	0	0	0	0	0	0	0	0	0	0
SAL_DA2180AA	CFSAN025207	Anatum	0	0	0	0	0	0	0	0	0	0	0
SAL_DA2181AA	CFSAN025206	Anatum	0	0	0	0	0	0	0	0	0	0	0
SAL_DA1944AA	FLUFL-1362	Anatum	0	0	0	0	0	0	0	0	0	0	0
SAL_DA1946AA	POR1-0020	Anatum	0	0	0	0	0	0	0	0	0	0	0
SAL_DA1750AA	POR1-0021	Anatum	0	0	0	0	0	0	0	0	0	0	0
SAL_DA1743AA	POR1-0035	Anatum	0	0	0	0	0	0	0	0	0	0	0
SAL_DA1289AA	H130260445	Anatum	0	0	0	0	0	0	0	0	0	0	0
SAL_DA1220AA	H121260567	Anatum	0	0	0	0	0	0	0	0	0	0	0
SAL_DA1122AA	H131080555	Anatum	0	0	0	0	0	0	0	0	0	0	0
SAL_DA0781AA	H123420698	Anatum	0	0	0	0	0	0	0	0	0	0	0
SAL_DA0491AA	H123820538	Anatum	0	0	0	0	0	0	0	0	0	0	0
SAL_DA0150AA	FDA00003629	Anatum	0	0	0	0	0	0	0	0	0	0	0
SAL_DA0000AA	FDA00003554	Anatum	0	0	0	0	0	0	0	0	0	0	0
SAL_CA9772AA	FNW19i75	Anatum	0	0	0	0	0	0	0	0	0	0	0
SAL_CA9668AA	FDA00003727	Anatum	0	0	0	0	0	0	0	0	0	0	0
SAL_CA9633AA	VA-WGS-00379	Anatum	0	0	0	0	0	0	0	0	0	0	0
SAL_CA9621AA	FNW19i81	Anatum	0	0	0	0	0	0	0	0	0	0	0
SAL_CA9595AA	CFSAN027098	Anatum	0	0	0	0	0	0	0	0	0	0	0
SAL_CA9554AA	CFSAN027109	Anatum	0	0	0	0	0	0	0	0	0	0	0
SAL_CA9344AA	IEH-NGS-SAL-01662	Anatum	0	0	0	0	0	0	0	0	0	0	0
SAL_CA9264AA	FDA00008044	Anatum	0	0	0	0	0	0	0	0	0	0	0

SAL_CA9225AA	CFSAN027142	Anatum	0	0	0	0	0	0	0	0	0	0	0
SAL_CA9204AA	PNUSAS000124	Anatum	0	0	0	0	0	0	0	0	0	0	0
SAL_CA9084AA	IEH-NGS-SAL-01771	Anatum	0	0	0	0	0	0	0	0	0	0	0
SAL_CA9076AA	CFSAN027533	Anatum	0	0	0	0	0	0	0	0	0	0	0
SAL_CA9071AA	CFSAN027539	Anatum	0	0	0	0	0	0	0	0	0	0	0
SAL_CA9055AA	CFSAN027528	Anatum	0	0	0	0	0	0	0	0	0	0	0
SAL_CA9051AA	CFSAN027529	Anatum	0	0	0	0	0	0	0	0	0	0	0
SAL_CA9035AA	CFSAN027515	Anatum	0	0	0	0	0	0	0	0	0	0	0
SAL_CA9034AA	CFSAN027521	Anatum	0	0	0	0	0	0	0	0	0	0	0
SAL_CA9037AA	CFSAN027513	Anatum	0	0	0	0	0	0	0	0	0	0	0
SAL_CA9033AA	CFSAN027522	Anatum	0	0	0	0	0	0	0	0	0	0	0
SAL_CA9027AA	IEH-NGS-SAL-01743	Anatum	0	0	0	0	0	0	0	0	0	0	0
SAL_CA9022AA	IEH-NGS-SAL-01758	Anatum	0	0	0	0	0	0	0	0	0	0	0
SAL_CA8929AA	CFSAN004220	Anatum	0	0	0	0	0	0	0	0	0	0	0
SAL_CA8898AA	CFSAN004219	Anatum	0	0	0	0	0	0	0	0	0	0	0
SAL_CA8897AA	CFSAN004221	Anatum	0	0	0	0	0	0	0	0	0	0	0
SAL_CA8893AA	CFSAN024833	Anatum	0	0	0	0	0	0	0	0	0	0	0
SAL_CA8889AA	CFSAN004218	Anatum	0	0	0	0	0	0	0	0	0	0	0
SAL_CA8832AA	AZ-TG74784	Anatum	0	0	0	0	0	0	0	0	0	0	0
SAL_CA8679AA	CFSAN027929	Anatum	0	0	0	0	0	0	0	0	0	0	0
SAL_CA8654AA	IEH-NGS-SAL-01831	Anatum	0	0	0	0	0	0	0	0	0	0	0
SAL_CA8644AA	IEH-NGS-SAL-01835	Anatum	0	0	0	0	0	0	0	0	0	0	0
SAL_CA8633AA	CFSAN027949	Anatum	0	0	0	0	0	0	0	0	0	0	0
SAL_CA8535AA	FL-NRM154	Anatum	0	0	0	0	0	0	0	0	0	0	0
SAL_CA8396AA	FDA00001304	Anatum	0	0	0	0	0	0	0	0	0	0	0
SAL_CA8378AA	FDA00002059	Anatum	0	0	0	0	0	0	0	0	0	0	0
SAL_CA8373AA	FDA00002060	Anatum	0	0	0	0	0	0	0	0	0	0	0
SAL_CA8330AA	CFSAN027965	Anatum	0	0	0	0	0	0	0	0	0	0	0
SAL_CA8267AA	CFSAN028005	Anatum	0	0	0	0	0	0	0	0	0	0	0
SAL_CA8239AA	FDA00002774	Anatum	0	0	0	0	0	0	0	0	0	0	0
SAL_CA8238AA	FDA00002775	Anatum	0	0	0	0	0	0	0	0	0	0	0
SAL_CA8204AA	IEH-NGS-SAL-01908	Anatum	0	0	0	0	0	0	0	0	0	0	0
SAL_CA8203AA	IEH-NGS-SAL-01894	Anatum	0	0	0	0	0	0	0	0	0	0	0
SAL_CA8019AA	CFSAN028591	Anatum	0	0	0	0	0	0	0	0	0	0	0
SAL_CA7986AA	CFSAN028596	Anatum	0	0	0	0	0	0	0	0	0	0	0
SAL_CA7906AA	CFSAN004234	Anatum	0	0	0	0	0	0	0	0	0	0	0
SAL_CA7889AA	CFSAN004238	Anatum	0	0	0	0	0	0	0	0	0	0	0
SAL_CA7881AA	CFSAN004236	Anatum	0	0	0	0	0	0	0	0	0	0	0
SAL_CA7882AA	CFSAN004235	Anatum	0	0	0	0	0	0	0	0	0	0	0
SAL_CA7862AA	CFSAN004259	Anatum	0	0	0	0	0	0	0	0	0	0	0
SAL_CA7850AA	CFSAN004237	Anatum	0	0	0	0	0	0	0	0	0	0	0
SAL_CA7835AA	CFSAN004260	Anatum	0	0	0	0	0	0	0	0	0	0	0
SAL_CA7559AA	FDA00008841	Anatum	0	0	0	0	0	0	0	0	0	0	0
SAL_CA7379AA	IEH-NGS-SAL-02230	Anatum	0	0	0	0	0	0	0	0	0	0	0
SAL_CA7343AA	CFSAN004217	Anatum	0	0	0	0	0	0	0	0	0	0	0
SAL_CA7262AA	FDA00004485	Anatum	0	0	0	0	0	0	0	0	0	0	0
SAL_CA7198AA	FDA00008856	Anatum	0	0	0	0	0	0	0	0	0	0	0
SAL_CA7170AA	NY-N11249	Anatum	0	0	0	0	0	0	0	0	0	0	0
SAL_CA7141AA	NY-N11230	Anatum	0	0	0	0	0	0	0	0	0	0	0
SAL_CA7128AA	NY-N11228	Anatum	0	0	0	0	0	0	0	0	0	0	0
SAL_CA6872AA	2015K-0177	Anatum	0	0	0	0	0	0	0	0	0	0	0
SAL_CA6720AA	54042	Anatum	0	0	0	0	0	0	0	0	0	0	0
SAL_CA5915AA	60020	Anatum	0	0	0	0	0	0	0	0	0	0	0
SAL_CA5879AA	CFSAN002015	Anatum	0	0	0	0	0	0	0	0	0	0	0
SAL_CA5875AA	CFSAN002010	Anatum	0	0	0	0	0	0	0	0	0	0	0
SAL_CA5869AA	CFSAN002008	Anatum	0	0	0	0	0	0	0	0	0	0	0
SAL_CA5864AA	CFSAN002001	Anatum	0	0	0	0	0	0	0	0	0	0	0
SAL_CA5866AA	CFSAN002009	Anatum	0	0	0	0	0	0	0	0	0	0	0
SAL_CA5863AA	CFSAN002007	Anatum	0	0	0	0	0	0	0	0	0	0	0
SAL_CA5870AA	CFSAN002004	Anatum	0	0	0	0	0	0	0	0	0	0	0
SAL_CA5088AA	78632	Anatum	0	0	0	0	0	0	0	0	0	0	0
SAL_CA4932AA	69754	Anatum	0	0	0	0	0	0	0	0	0	0	0
SAL_CA4348AA	96226	Anatum	0	0	0	0	0	0	0	0	0	0	0
SAL_CA4196AA	23450	Anatum	0	0	0	0	0	0	0	0	0	0	0
SAL_CA4191AA	41964	Anatum	0	0	0	0	0	0	0	0	0	0	0
SAL_CA3968AA	23066	Anatum	0	0	0	0	0	0	0	0	0	0	0
SAL_CA3066AA	83333	Anatum	0	0	0	0	0	0	0	0	0	0	0
SAL_CA2741AA	57116	Anatum	0	0	0	0	0	0	0	0	0	0	0
SAL_CA2646AA	25322	Anatum	0	0	0	0	0	0	0	0	0	0	0
SAL_CA1959AA	95006	Anatum	0	0	0	0	0	0	0	0	0	0	0
SAL_CA1665AA	CFSAN000207	Anatum	0	0	0	0	0	0	0	0	0	0	0
SAL_CA1289AA	37053	Anatum	0	0	0	0	0	0	0	0	0	0	0
SAL_CA0908AA	52986	Anatum	0	0	0	0	0	0	0	0	0	0	0

SAL_CA0874AA	65350	Anatum	0	0	0	0	0	0	0	0	0	0	0
SAL_CA0251AA	65316	Anatum	0	0	0	0	0	0	0	0	0	0	0
SAL_BA9865AA	84396	Anatum	0	0	0	0	0	0	0	0	0	0	0
SAL_BA8948AA	CFSAN005702	Anatum	0	0	0	0	0	0	0	0	0	0	0
SAL_BA8954AA	CFSAN005699	Anatum	0	0	0	0	0	0	0	0	0	0	0
SAL_BA8934AA	CFSAN005709	Anatum	0	0	0	0	0	0	0	0	0	0	0
SAL_BA8921AA	CFSAN005716	Anatum	0	0	0	0	0	0	0	0	0	0	0
SAL_BA8916AA	52975	Anatum	0	0	0	0	0	0	0	0	0	0	0
SAL_BA8866AA	NY_BAC0400012517	Anatum	0	0	0	0	0	0	0	0	0	0	0
SAL_BA8795AA	NY_BAC0700000573	Anatum	0	0	0	0	0	0	0	0	0	0	0
SAL_BA8643AA	CFSAN005680	Anatum	0	0	0	0	0	0	0	0	0	0	0
SAL_BA8633AA	61875	Anatum	0	0	0	0	0	0	0	0	0	0	0
SAL_BA8579AA	CFSAN005695	Anatum	0	0	0	0	0	0	0	0	0	0	0
SAL_BA8577AA	IEH_NGS_SAL_00236	Anatum	0	0	0	0	0	0	0	0	0	0	0
SAL_BA8259AA	FL_FLDACS-12507	Anatum	0	0	0	0	0	0	0	0	0	0	0
SAL_BA8231AA	CFSAN005718	Anatum	0	0	0	0	0	0	0	0	0	0	0
SAL_BA8194AA	CFSAN005741	Anatum	0	0	0	0	0	0	0	0	0	0	0
SAL_BA7550AA	50334	Anatum	0	0	0	0	0	0	0	0	0	0	0
SAL_BA7556AA	65404	Anatum	0	0	0	0	0	0	0	0	0	0	0
SAL_BA7388AA	CFSAN006183	Anatum	0	0	0	0	0	0	0	0	0	0	0
SAL_BA7009AA	CFSAN006216	Anatum	0	0	0	0	0	0	0	0	0	0	0
SAL_BA6985AA	CFSAN006168	Anatum	0	0	0	0	0	0	0	0	0	0	0
SAL_BA6939AA	CFSAN006178	Anatum	0	0	0	0	0	0	0	0	0	0	0
SAL_BA6880AA	CFSAN006177	Anatum	0	0	0	0	0	0	0	0	0	0	0
SAL_BA6806AA	CFSAN006209	Anatum	0	0	0	0	0	0	0	0	0	0	0
SAL_BA6080AA	FDA00001192	Anatum	0	0	0	0	0	0	0	0	0	0	0
SAL_BA5862AA	M131 / NCTC20057	Anatum	0	0	0	0	0	0	0	0	0	0	0
SAL_BA5819AA	FDA00004456	Anatum	0	0	0	0	0	0	0	0	0	0	0
SAL_BA5625AA	FSF0125	Anatum	0	0	0	0	0	0	0	0	0	0	0
SAL_BA5106AA	CFSAN005652	Anatum	0	0	0	0	0	0	0	0	0	0	0
SAL_BA5071AA	CFSAN024548	Anatum	0	0	0	0	0	0	0	0	0	0	0
SAL_BA5014AA	CFSAN024488	Anatum	0	0	0	0	0	0	0	0	0	0	0
SAL_BA4883AA	CFSAN006179	Anatum	0	0	0	0	0	0	0	0	0	0	0
SAL_BA4874AA	CFSAN006192	Anatum	0	0	0	0	0	0	0	0	0	0	0
SAL_BA4894AA	FDA00002539	Anatum	0	0	0	0	0	0	0	0	0	0	0
SAL_BA4878AA	CFSAN006191	Anatum	0	0	0	0	0	0	0	0	0	0	0
SAL_BA4811AA	NY-N13660	Anatum	0	0	0	0	0	0	0	0	0	0	0
SAL_BA4699AA	FDA00002501	Anatum	0	0	0	0	0	0	0	0	0	0	0
SAL_BA4679AA	CFSAN006234	Anatum	0	0	0	0	0	0	0	0	0	0	0
SAL_BA3940AA	CFSAN004013	Anatum	0	0	0	0	0	0	0	0	0	0	0
SAL_BA3942AA	CFSAN004014	Anatum	0	0	0	0	0	0	0	0	0	0	0
SAL_BA3934AA	CFSAN004010	Anatum	0	0	0	0	0	0	0	0	0	0	0
SAL_BA3928AA	CFSAN004002	Anatum	0	0	0	0	0	0	0	0	0	0	0
SAL_BA3932AA	CFSAN004005	Anatum	0	0	0	0	0	0	0	0	0	0	0
SAL_BA3930AA	CFSAN004004	Anatum	0	0	0	0	0	0	0	0	0	0	0
SAL_BA3924AA	CFSAN004000	Anatum	0	0	0	0	0	0	0	0	0	0	0
SAL_BA3927AA	CFSAN004001	Anatum	0	0	0	0	0	0	0	0	0	0	0
SAL_BA3918AA	CFSAN003964	Anatum	0	0	0	0	0	0	0	0	0	0	0
SAL_BA3947AA	CFSAN004017	Anatum	0	0	0	0	0	0	0	0	0	0	0
SAL_BA3920AA	CFSAN003963	Anatum	0	0	0	0	0	0	0	0	0	0	0
SAL_BA3779AA	CFSAN003934	Anatum	0	0	0	0	0	0	0	0	0	0	0
SAL_BA3777AA	CFSAN003935	Anatum	0	0	0	0	0	0	0	0	0	0	0
SAL_BA3775AA	CFSAN003936	Anatum	0	0	0	0	0	0	0	0	0	0	0
SAL_BA3773AA	CFSAN003937	Anatum	0	0	0	0	0	0	0	0	0	0	0
SAL_BA3771AA	CFSAN003938	Anatum	0	0	0	0	0	0	0	0	0	0	0
SAL_BA3765AA	CFSAN003944	Anatum	0	0	0	0	0	0	0	0	0	0	0
SAL_BA3767AA	CFSAN003943	Anatum	0	0	0	0	0	0	0	0	0	0	0
SAL_BA3759AA	CFSAN003950	Anatum	0	0	0	0	0	0	0	0	0	0	0
SAL_BA3763AA	CFSAN003945	Anatum	0	0	0	0	0	0	0	0	0	0	0
SAL_BA3754AA	CFSAN003961	Anatum	0	0	0	0	0	0	0	0	0	0	0
SAL_BA3752AA	CFSAN003962	Anatum	0	0	0	0	0	0	0	0	0	0	0
SAL_BA3750AA	CFSAN003965	Anatum	0	0	0	0	0	0	0	0	0	0	0
SAL_BA3746AA	CFSAN003967	Anatum	0	0	0	0	0	0	0	0	0	0	0
SAL_BA3744AA	CFSAN003969	Anatum	0	0	0	0	0	0	0	0	0	0	0
SAL_BA3734AA	CFSAN003974	Anatum	0	0	0	0	0	0	0	0	0	0	0
SAL_BA3740AA	CFSAN003971	Anatum	0	0	0	0	0	0	0	0	0	0	0
SAL_BA3742AA	CFSAN003970	Anatum	0	0	0	0	0	0	0	0	0	0	0
SAL_BA3736AA	CFSAN003973	Anatum	0	0	0	0	0	0	0	0	0	0	0
SAL_BA3732AA	CFSAN003975	Anatum	0	0	0	0	0	0	0	0	0	0	0
SAL_BA3720AA	2015K-0220	Anatum	0	0	0	0	0	0	0	0	0	0	0
SAL_BA3739AA	CFSAN003972	Anatum	0	0	0	0	0	0	0	0	0	0	0
SAL_BA3718AA	2015K-0194	Anatum	0	0	0	0	0	0	0	0	0	0	0
SAL_BA3612AA	TX-ACF1401611	Anatum	0	0	0	0	0	0	0	0	0	0	0

SAL_BA3596AA	CFSAN024491	Anatum	0	0	0	0	0	0	0	0	0	0	0
SAL_BA3492AA	CFSAN029982	Anatum	0	0	0	0	0	0	0	0	0	0	0
SAL_BA2469AA	FDA00009156	Anatum	0	0	0	0	0	0	0	0	0	0	0
SAL_BA2072AA	FL_FLDACS-16438	Anatum	0	0	0	0	0	0	0	0	0	0	0
SAL_BA2042AA	FL_MJ402	Anatum	0	0	0	0	0	0	0	0	0	0	0
SAL_BA1933AA	TX-ACF1402790	Anatum	0	0	0	0	0	0	0	0	0	0	0
SAL_BA1579AA	FSIS1501057	Anatum	0	0	0	0	0	0	0	0	0	0	0
SAL_BA1573AA	FSIS1501058	Anatum	0	0	0	0	0	0	0	0	0	0	0
SAL_BA1529AA	FDA00003403	Anatum	0	0	0	0	0	0	0	0	0	0	0
SAL_BA1476AA	FSIS1503206	Anatum	0	0	0	0	0	0	0	0	0	0	0
SAL_BA1483AA	FSIS1503205	Anatum	0	0	0	0	0	0	0	0	0	0	0
SAL_BA1461AA	FSIS1503192	Anatum	0	0	0	0	0	0	0	0	0	0	0
SAL_BA1464AA	FSIS1503194	Anatum	0	0	0	0	0	0	0	0	0	0	0
SAL_BA1407AA	TX-ACF1501824	Anatum	0	0	0	0	0	0	0	0	0	0	0
SAL_BA1309AA	CVM-N14794	Anatum	0	0	0	0	0	0	0	0	0	0	0
SAL_BA1305AA	ADRDL-LA-29-2014	Anatum	0	0	0	0	0	0	0	0	0	0	0
SAL_AA9835AA	:-2013-10-08T14:24:19Z-	Anatum	0	0	0	0	0	0	0	0	0	0	0
SAL_AA8397AA	908397	Anatum	0	0	0	0	0	0	0	0	0	0	0
SAL_FA6097AA	FDA00010122	Anatum	0	0	0	0	0	0	0	0	0	0	0
SAL_FA6098AA	FDA00010121	Anatum	0	0	0	0	0	0	0	0	0	0	0
SAL_FA6210AA	FSIS1606331	Anatum	0	0	0	0	0	0	0	0	0	0	0
SAL_FA6203AA	FSIS1606329	Anatum	0	0	0	0	0	0	0	0	0	0	0
SAL_FA6208AA	FSIS1606336	Anatum	0	0	0	0	0	0	0	0	0	0	0
SAL_FA6188AA	FSIS1606341	Anatum	0	0	0	0	0	0	0	0	0	0	0
SAL_FA6182AA	FSIS1606398	Anatum	0	0	0	0	0	0	0	0	0	0	0
SAL_FA6181AA	FSIS1606339	Anatum	0	0	0	0	0	0	0	0	0	0	0
SAL_FA6178AA	FSIS1606337	Anatum	0	0	0	0	0	0	0	0	0	0	0
SAL_FA6167AA	FSIS1606407	Anatum	0	0	0	0	0	0	0	0	0	0	0
SAL_FA6260AA	CFSAN032102	Anatum	0	0	0	0	0	0	0	0	0	0	0
SAL_FA6259AA	CFSAN032105	Anatum	0	0	0	0	0	0	0	0	0	0	0
SAL_FA6419AA	PNUSAS002112	Anatum	0	0	0	0	0	0	0	0	0	0	0
SAL_FA6331AA	FSIS1606508	Anatum	0	0	0	0	0	0	0	0	0	0	0
SAL_FA6337AA	FSIS1606502	Anatum	0	0	0	0	0	0	0	0	0	0	0
SAL_FA6333AA	FSIS1606506	Anatum	0	0	0	0	0	0	0	0	0	0	0
SAL_FA6328AA	FSIS1606511	Anatum	0	0	0	0	0	0	0	0	0	0	0
SAL_FA6329AA	FSIS1606510	Anatum	0	0	0	0	0	0	0	0	0	0	0
SAL_FA6296AA	FSIS1606516	Anatum	0	0	0	0	0	0	0	0	0	0	0
SAL_FA6289AA	2016K-0244	Anatum	0	0	0	0	0	0	0	0	0	0	0
SAL_FA7269AA	FDA00000077	Anatum	0	0	0	0	0	0	0	0	0	0	0
SAL_FA7267AA	FDA00010194	Anatum	0	0	0	0	0	0	0	0	0	0	0
SAL_FA7287AA	FDA00007887	Anatum	0	0	0	0	0	0	0	0	0	0	0
SAL_FA7904AA	FSIS1606765	Anatum	0	0	0	0	0	0	0	0	0	0	0
SAL_FA7891AA	FSIS1606779	Anatum	0	0	0	0	0	0	0	0	0	0	0
SAL_FA7888AA	FSIS1606785	Anatum	0	0	0	0	0	0	0	0	0	0	0
SAL_FA7889AA	FSIS1606776	Anatum	0	0	0	0	0	0	0	0	0	0	0
SAL_FA8052AA	FSIS1502314	Anatum	0	0	0	0	0	0	0	0	0	0	0
SAL_FA8050AA	FDA00009735	Anatum	0	0	0	0	0	0	0	0	0	0	0
SAL_FA8145AA	FSIS1606879	Anatum	0	0	0	0	0	0	0	0	0	0	0
SAL_FA8132AA	FSIS1606886	Anatum	0	0	0	0	0	0	0	0	0	0	0
SAL_FA2381AA	CFSAN047063	Anatum	0	0	0	0	0	0	0	0	0	0	0
SAL_FA8198AA	FDA00010290	Anatum	0	0	0	0	0	0	0	0	0	0	0
SAL_FA8257AA	FSIS1606921	Anatum	0	0	0	0	0	0	0	0	0	0	0
SAL_FA8254AA	FSIS1606922	Anatum	0	0	0	0	0	0	0	0	0	0	0
SAL_FA8232AA	FSIS1606958	Anatum	0	0	0	0	0	0	0	0	0	0	0
SAL_FA8230AA	FSIS1606956	Anatum	0	0	0	0	0	0	0	0	0	0	0
SAL_FA7590AA	FSIS1606692	Anatum	0	0	0	0	0	0	0	0	0	0	0
SAL_FA7587AA	FSIS1606698	Anatum	0	0	0	0	0	0	0	0	0	0	0
SAL_FA7583AA	FSIS1606694	Anatum	0	0	0	0	0	0	0	0	0	0	0
SAL_FA7582AA	FSIS1606695	Anatum	0	0	0	0	0	0	0	0	0	0	0
SAL_FA7290AA	FSIS1606568	Anatum	0	0	0	0	0	0	0	0	0	0	0
SAL_FA7173AA	FSIS1606686	Anatum	0	0	0	0	0	0	0	0	0	0	0
SAL_FA6936AA	FSIS1606628	Anatum	0	0	0	0	0	0	0	0	0	0	0
SAL_FA6927AA	FSIS1606633	Anatum	0	0	0	0	0	0	0	0	0	0	0
SAL_FA8467AA	FSIS1606827	Anatum	0	0	0	0	0	0	0	0	0	0	0
SAL_FA9782AA	FSIS1504581	Anatum	0	0	0	0	0	0	0	0	0	0	0
SAL_FA9779AA	FSIS1504264	Anatum	0	0	0	0	0	0	0	0	0	0	0
SAL_FA9774AA	FSIS1502422	Anatum	0	0	0	0	0	0	0	0	0	0	0
SAL_FA9771AA	FSIS1502178	Anatum	0	0	0	0	0	0	0	0	0	0	0
SAL_FA9769AA	FSIS1502175	Anatum	0	0	0	0	0	0	0	0	0	0	0
SAL_FA9767AA	FSIS1502176	Anatum	0	0	0	0	0	0	0	0	0	0	0
SAL_FA9766AA	FSIS1502431	Anatum	0	0	0	0	0	0	0	0	0	0	0
SAL_FA9759AA	FSIS1503577	Anatum	0	0	0	0	0	0	0	0	0	0	0
SAL_FA9757AA	FSIS1502304	Anatum	0	0	0	0	0	0	0	0	0	0	0

SAL_GA2806AA	CRJJGF_00105	Anatum	0	0	0	0	0	0	0	0	0	0	0
SAL_GA2791AA	CRJJGF_00121	Anatum	0	0	0	0	0	0	0	0	0	0	0
SAL_GA3474AA	BCW_3953	Anatum	0	0	0	0	0	0	0	0	0	0	0
SAL_GA3473AA	BCW_3954	Anatum	0	0	0	0	0	0	0	0	0	0	0
SAL_GA3462AA	BCW_3970	Anatum	0	0	0	0	0	0	0	0	0	0	0
SAL_GA3438AA	BCW_4006	Anatum	0	0	0	0	0	0	0	0	0	0	0
SAL_GA3435AA	BCW_4010	Anatum	0	0	0	0	0	0	0	0	0	0	0
SAL_GA3415AA	BCW_2104	Anatum	0	0	0	0	0	0	0	0	0	0	0
SAL_GA3359AA	BCW_2637	Anatum	0	0	0	0	0	0	0	0	0	0	0
SAL_GA3358AA	BCW_2638	Anatum	0	0	0	0	0	0	0	0	0	0	0
SAL_GA3317AA	CDC B1487	Anatum	0	0	0	0	0	0	0	0	0	0	0
SAL_GA3243AA	N26608	Anatum	0	0	0	0	0	0	0	0	0	0	0
SAL_GA3140AA	NCTR-SF121	Anatum	0	0	0	0	0	0	0	0	0	0	0
SAL_GA2885AA	CRJJGF_00026	Anatum	0	0	0	0	0	0	0	0	0	0	0
SAL_GA2434AA	BCW_1981	Anatum	0	0	0	0	0	0	0	0	0	0	0
SAL_GA4539AA	FDA00001709	Anatum	0	0	0	0	0	0	0	0	0	0	0
SAL_GA2121AA	BCW_6532	Anatum	0	0	0	0	0	0	0	0	0	0	0
SAL_GA2073AA	BCW_3067	Anatum	0	0	0	0	0	0	0	0	0	0	0
SAL_GA4504AA	FDA00000160	Anatum	0	0	0	0	0	0	0	0	0	0	0
SAL_GA1841AA	BCW_5109	Anatum	0	0	0	0	0	0	0	0	0	0	0
SAL_GA1775AA	N26636	Anatum	0	0	0	0	0	0	0	0	0	0	0
SAL_GA1659AA	BCW_3955	Anatum	0	0	0	0	0	0	0	0	0	0	0
SAL_GA1652AA	BCW_4009	Anatum	0	0	0	0	0	0	0	0	0	0	0
SAL_GA0324AA	71_G_354-sc-2280665	Anatum	0	0	0	0	0	0	0	0	0	0	0
SAL_GA0309AA	71_H_300-sc-2280698	Anatum	0	0	0	0	0	0	0	0	0	0	0
SAL_GA0288AA	72_H_059-sc-2280731	Anatum	0	0	0	0	0	0	0	0	0	0	0
SAL_GA0286AA	72_H_222-sc-2280733	Anatum	0	0	0	0	0	0	0	0	0	0	0
SAL_GA0238AA	CT14C_2_1-sc-2280784	Anatum	0	0	0	0	0	0	0	0	0	0	0
SAL_GA0202AA	VNS30078-sc-2280598	Anatum	0	0	0	0	0	0	0	0	0	0	0
SAL_FA9650AA	WAPHL-SAL-A00956	Anatum	0	0	0	0	0	0	0	0	0	0	0
SAL_GA4757AA	PNUSAS002773	Anatum	0	0	0	0	0	0	0	0	0	0	0
SAL_GA4755AA	PNUSAS002774	Anatum	0	0	0	0	0	0	0	0	0	0	0
SAL_GA4756AA	PNUSAS002843	Anatum	0	0	0	0	0	0	0	0	0	0	0
SAL_GA4747AA	PNUSAS002326	Anatum	0	0	0	0	0	0	0	0	0	0	0
SAL_GA4718AA	FSIS1607042	Anatum	0	0	0	0	0	0	0	0	0	0	0
SAL_GA4712AA	FSIS1606972	Anatum	0	0	0	0	0	0	0	0	0	0	0
SAL_GA4717AA	FSIS1606964	Anatum	0	0	0	0	0	0	0	0	0	0	0
SAL_GA4700AA	FSIS1606967	Anatum	0	0	0	0	0	0	0	0	0	0	0
SAL_GA4699AA	FSIS1606965	Anatum	0	0	0	0	0	0	0	0	0	0	0
SAL_GA4698AA	FSIS1607045	Anatum	0	0	0	0	0	0	0	0	0	0	0
SAL_GA4690AA	FSIS1501872	Anatum	0	0	0	0	0	0	0	0	0	0	0
SAL_GA4696AA	FSIS1607040	Anatum	0	0	0	0	0	0	0	0	0	0	0
SAL_FA9441AA	CVM-N26636	Anatum	0	0	0	0	0	0	0	0	0	0	0
SAL_GA5109AA	PNUSAS002881	Anatum	0	0	0	0	0	0	0	0	0	0	0
SAL_GA5063AA	PNUSAS002765	Anatum	0	0	0	0	0	0	0	0	0	0	0
SAL_GA5062AA	PNUSAS002766	Anatum	0	0	0	0	0	0	0	0	0	0	0
SAL_GA5061AA	PNUSAS002767	Anatum	0	0	0	0	0	0	0	0	0	0	0
SAL_GA5059AA	PNUSAS002769	Anatum	0	0	0	0	0	0	0	0	0	0	0
SAL_GA5060AA	PNUSAS002768	Anatum	0	0	0	0	0	0	0	0	0	0	0
SAL_GA5058AA	PNUSAS002770	Anatum	0	0	0	0	0	0	0	0	0	0	0
SAL_GA5056AA	PNUSAS002842	Anatum	0	0	0	0	0	0	0	0	0	0	0
SAL_GA5057AA	PNUSAS002771	Anatum	0	0	0	0	0	0	0	0	0	0	0
SAL_GA5055AA	PNUSAS002772	Anatum	0	0	0	0	0	0	0	0	0	0	0
SAL_GA5045AA	PNUSAS002775	Anatum	0	0	0	0	0	0	0	0	0	0	0
SAL_FA9180AA	CVM N38870	Anatum	0	0	0	0	0	0	0	0	0	0	0
SAL_FA9175AA	CVM N38876	Anatum	0	0	0	0	0	0	0	0	0	0	0
SAL_FA9176AA	CVM N38875	Anatum	0	0	0	0	0	0	0	0	0	0	0
SAL_GA5174AA	V-CLASP-D-46	Anatum	0	0	0	0	0	0	0	0	0	0	0
SAL_GA5175AA	V-CLASP-D-45	Anatum	0	0	0	0	0	0	0	0	0	0	0
SAL_GA5385AA	CFSAN042370	Anatum	0	0	0	0	0	0	0	0	0	0	0
SAL_GA5384AA	CFSAN042371	Anatum	0	0	0	0	0	0	0	0	0	0	0
SAL_GA5323AA	CFSAN042886	Anatum	0	0	0	0	0	0	0	0	0	0	0
SAL_GA5322AA	CFSAN042887	Anatum	0	0	0	0	0	0	0	0	0	0	0
SAL_GA5318AA	CFSAN042891	Anatum	0	0	0	0	0	0	0	0	0	0	0
SAL_GA5319AA	CFSAN042890	Anatum	0	0	0	0	0	0	0	0	0	0	0
SAL_GA5317AA	CFSAN042892	Anatum	0	0	0	0	0	0	0	0	0	0	0
SAL_GA5316AA	CFSAN042893	Anatum	0	0	0	0	0	0	0	0	0	0	0
SAL_GA5266AA	FSIS1607053	Anatum	0	0	0	0	0	0	0	0	0	0	0
SAL_GA5261AA	FDA00007885	Anatum	0	0	0	0	0	0	0	0	0	0	0
SAL_FA8908AA	CVM N42473	Anatum	0	0	0	0	0	0	0	0	0	0	0
SAL_GA5386AA	CFSAN042366	Anatum	0	0	0	0	0	0	0	0	0	0	0
SAL_FA8668AA	CVM N31410	Anatum	0	0	0	0	0	0	0	0	0	0	0
SAL_FA8455AA	FSIS1606701	Anatum	0	0	0	0	0	0	0	0	0	0	0

SAL_GA6016AA	FLUFL-1982	Anatum	0	0	0	0	0	0	0	0	0	0	0
SAL_GA6010AA	FLUFL-1988	Anatum	0	0	0	0	0	0	0	0	0	0	0
SAL_GA5978AA	FDA00007882	Anatum	0	0	0	0	0	0	0	0	0	0	0
SAL_GA5955AA	FSIS1607054	Anatum	0	0	0	0	0	0	0	0	0	0	0
SAL_GA5954AA	FSIS1607055	Anatum	0	0	0	0	0	0	0	0	0	0	0
SAL_GA5944AA	FSIS1607161	Anatum	0	0	0	0	0	0	0	0	0	0	0
SAL_GA5943AA	FSIS1607108	Anatum	0	0	0	0	0	0	0	0	0	0	0
SAL_GA5940AA	FSIS1607160	Anatum	0	0	0	0	0	0	0	0	0	0	0
SAL_GA5936AA	FSIS1607105	Anatum	0	0	0	0	0	0	0	0	0	0	0
SAL_GA6048AA	FSIS1607201	Anatum	0	0	0	0	0	0	0	0	0	0	0
SAL_GA6043AA	FSIS1607172	Anatum	0	0	0	0	0	0	0	0	0	0	0
SAL_GA6041AA	FSIS1607200	Anatum	0	0	0	0	0	0	0	0	0	0	0
SAL_GA6373AA	2016K-0571	Anatum	0	0	0	0	0	0	0	0	0	0	0
SAL_GA6309AA	FDA00001742	Anatum	0	0	0	0	0	0	0	0	0	0	0
SAL_GA6302AA	FDA00002502	Anatum	0	0	0	0	0	0	0	0	0	0	0
SAL_GA6608AA	PNUSAS003010	Anatum	0	0	0	0	0	0	0	0	0	0	0
SAL_GA6602AA	2016K-0548	Anatum	0	0	0	0	0	0	0	0	0	0	0
SAL_GA6556AA	PNUSAS003180	Anatum	0	0	0	0	0	0	0	0	0	0	0
SAL_GA6545AA	2016K-0547	Anatum	0	0	0	0	0	0	0	0	0	0	0
SAL_GA6669AA	FSIS1607291	Anatum	0	0	0	0	0	0	0	0	0	0	0
SAL_GA6658AA	FSIS1607292	Anatum	0	0	0	0	0	0	0	0	0	0	0
SAL_GA7023AA	CFSAN045021	Anatum	0	0	0	0	0	0	0	0	0	0	0
SAL_GA6967AA	FDA00003276	Anatum	0	0	0	0	0	0	0	0	0	0	0
SAL_GA7484AA	PNUSAS003278	Anatum	0	0	0	0	0	0	0	0	0	0	0
SAL_GA7756AA	FSIS1607388	Anatum	0	0	0	0	0	0	0	0	0	0	0
SAL_GA7751AA	FSIS1607393	Anatum	0	0	0	0	0	0	0	0	0	0	0
SAL_GA8158AA	ADRDL15-24023	Anatum	0	0	0	0	0	0	0	0	0	0	0
SAL_GA8218AA	ADRDL16-1176	Anatum	0	0	0	0	0	0	0	0	0	0	0
SAL_GA8033AA	FSIS1607399	Anatum	0	0	0	0	0	0	0	0	0	0	0
SAL_GA8032AA	FSIS1607401	Anatum	0	0	0	0	0	0	0	0	0	0	0
SAL_GA8048AA	FDA00010332	Anatum	0	0	0	0	0	0	0	0	0	0	0
SAL_GA8205AA	PNUSAS003485	Anatum	0	0	0	0	0	0	0	0	0	0	0
SAL_GA8438AA	FSIS1607456	Anatum	0	0	0	0	0	0	0	0	0	0	0
SAL_GA8451AA	FSIS1607440	Anatum	0	0	0	0	0	0	0	0	0	0	0
SAL_GA8439AA	FSIS1607458	Anatum	0	0	0	0	0	0	0	0	0	0	0
SAL_GA8465AA	ADRDL_R135	Anatum	0	0	0	0	0	0	0	0	0	0	0
SAL_GA8658AA	PNUSAS002776	Anatum	0	0	0	0	0	0	0	0	0	0	0
SAL_GA8722AA	FSIS1607407	Anatum	0	0	0	0	0	0	0	0	0	0	0
SAL_GA8700AA	FSIS1607483	Anatum	0	0	0	0	0	0	0	0	0	0	0
SAL_GA8709AA	FSIS1607492	Anatum	0	0	0	0	0	0	0	0	0	0	0
SAL_GA8702AA	FSIS1607491	Anatum	0	0	0	0	0	0	0	0	0	0	0
SAL_GA8705AA	FSIS1607487	Anatum	0	0	0	0	0	0	0	0	0	0	0
SAL_GA8826AA	OH-1103399	Anatum	0	0	0	0	0	0	0	0	0	0	0
SAL_GA9070AA	TX-ACF1601758	Anatum	0	0	0	0	0	0	0	0	0	0	0
SAL_GA9196AA	CFSAN031517	Anatum	0	0	0	0	0	0	0	0	0	0	0
SAL_GA9199AA	CFSAN031514	Anatum	0	0	0	0	0	0	0	0	0	0	0
SAL_GA9198AA	CFSAN031515	Anatum	0	0	0	0	0	0	0	0	0	0	0
SAL_GA9197AA	CFSAN031516	Anatum	0	0	0	0	0	0	0	0	0	0	0
SAL_GA9297AA	FSIS1607558	Anatum	0	0	0	0	0	0	0	0	0	0	0
SAL_GA9306AA	FSIS1607534	Anatum	0	0	0	0	0	0	0	0	0	0	0
SAL_GA9316AA	FSIS1607543	Anatum	0	0	0	0	0	0	0	0	0	0	0
SAL_GA9353AA	ADRDL_R125	Anatum	0	0	0	0	0	0	0	0	0	0	0
SAL_GA9358AA	ADRDL_R163	Anatum	0	0	0	0	0	0	0	0	0	0	0
SAL_GA9563AA	FDA00003898	Anatum	0	0	0	0	0	0	0	0	0	0	0
SAL_GA9564AA	FDA00003897	Anatum	0	0	0	0	0	0	0	0	0	0	0
SAL_GA9463AA	FSIS1607644	Anatum	0	0	0	0	0	0	0	0	0	0	0
SAL_GA9451AA	FSIS1607661	Anatum	0	0	0	0	0	0	0	0	0	0	0
SAL_GA9738AA	FDA00004801	Anatum	0	0	0	0	0	0	0	0	0	0	0
SAL_GA9710AA	CFSAN042367	Anatum	0	0	0	0	0	0	0	0	0	0	0
SAL_HA0005AA	PNUSAS003558	Anatum	0	0	0	0	0	0	0	0	0	0	0
SAL_HA0102AA	NYVetLIRN-2	Anatum	0	0	0	0	0	0	0	0	0	0	0
SAL_HA0112AA	FDA00004943	Anatum	0	0	0	0	0	0	0	0	0	0	0
SAL_HA0130AA	FSIS1607662	Anatum	0	0	0	0	0	0	0	0	0	0	0
SAL_HA0312AA	PNUSAS003464	Anatum	0	0	0	0	0	0	0	0	0	0	0
SAL_HA0466AA	PNUSAS004317	Anatum	0	0	0	0	0	0	0	0	0	0	0
SAL_HA0465AA	PNUSAS004318	Anatum	0	0	0	0	0	0	0	0	0	0	0
SAL_HA0855AA	FDA00004238	Anatum	0	0	0	0	0	0	0	0	0	0	0
SAL_HA0830AA	FSIS1607803	Anatum	0	0	0	0	0	0	0	0	0	0	0
SAL_HA0854AA	FDA00004247	Anatum	0	0	0	0	0	0	0	0	0	0	0
SAL_HA0850AA	FDA00004297	Anatum	0	0	0	0	0	0	0	0	0	0	0
SAL_HA0833AA	FSIS1607797	Anatum	0	0	0	0	0	0	0	0	0	0	0
SAL_HA0674AA	NSUOC-0013-W4X	Anatum	0	0	0	0	0	0	0	0	0	0	0
SAL_HA0623AA	2014AM-0662	Anatum	0	0	0	0	0	0	0	0	0	0	0

SAL_HA0675AA	NSUOC-0014-W4X2	Anatum	0	0	0	0	0	0	0	0	0	0	0
SAL_HA0947AA	NSUOC-0049-S1.1B2	Anatum	0	0	0	0	0	0	0	0	0	0	0
SAL_HA0954AA	NSUOC-0050-S1.1B3	Anatum	0	0	0	0	0	0	0	0	0	0	0
SAL_HA1148AA	ADRDL-R200	Anatum	0	0	0	0	0	0	0	0	0	0	0
SAL_HA1282AA	FSIS1607965	Anatum	0	0	0	0	0	0	0	0	0	0	0
SAL_HA1285AA	FSIS1607865	Anatum	0	0	0	0	0	0	0	0	0	0	0
SAL_HA1289AA	FSIS1607875	Anatum	0	0	0	0	0	0	0	0	0	0	0
SAL_HA1280AA	FSIS1607974	Anatum	0	0	0	0	0	0	0	0	0	0	0
SAL_HA1283AA	FSIS1607869	Anatum	0	0	0	0	0	0	0	0	0	0	0
SAL_HA1291AA	FSIS1607883	Anatum	0	0	0	0	0	0	0	0	0	0	0
SAL_HA1293AA	FSIS1607873	Anatum	0	0	0	0	0	0	0	0	0	0	0
SAL_HA1320AA	FSIS1607885	Anatum	0	0	0	0	0	0	0	0	0	0	0
SAL_HA1311AA	FSIS1607960	Anatum	0	0	0	0	0	0	0	0	0	0	0
SAL_HA1068AA	ARIM-SSCHW350-08	Anatum	0	0	0	0	0	0	0	0	0	0	0
SAL_HA1755AA	FSIS1608057	Anatum	0	0	0	0	0	0	0	0	0	0	0
SAL_HA1761AA	PNUSAS002725	Anatum	0	0	0	0	0	0	0	0	0	0	0
SAL_HA2770AA	ADRDL-16-10280	Anatum	0	0	0	0	0	0	0	0	0	0	0
SAL_HA2755AA	CFSAN048256	Anatum	0	0	0	0	0	0	0	0	0	0	0
SAL_HA3048AA	FSIS1607791	Anatum	0	0	0	0	0	0	0	0	0	0	0
SAL_HA3051AA	FSIS1608204	Anatum	0	0	0	0	0	0	0	0	0	0	0
SAL_HA2918AA	FSIS1608111	Anatum	0	0	0	0	0	0	0	0	0	0	0
SAL_HA2934AA	FSIS1608145	Anatum	0	0	0	0	0	0	0	0	0	0	0
SAL_HA2942AA	FSIS1608103	Anatum	0	0	0	0	0	0	0	0	0	0	0
SAL_HA2951AA	FSIS1608050	Anatum	0	0	0	0	0	0	0	0	0	0	0
SAL_HA2905AA	FSIS1608197	Anatum	0	0	0	0	0	0	0	0	0	0	0
SAL_HA3243AA	FSIS1608261	Anatum	0	0	0	0	0	0	0	0	0	0	0
SAL_HA3250AA	FSIS1608263	Anatum	0	0	0	0	0	0	0	0	0	0	0
SAL_HA2856AA	FSIS1608089	Anatum	0	0	0	0	0	0	0	0	0	0	0
SAL_HA2790AA	NSUOC-0080-S1.1H	Anatum	0	0	0	0	0	0	0	0	0	0	0
SAL_HA2752AA	CFSAN048257	Anatum	0	0	0	0	0	0	0	0	0	0	0
SAL_HA2716AA	FSIS1608315	Anatum	0	0	0	0	0	0	0	0	0	0	0
SAL_HA2758AA	CFSAN048258	Anatum	0	0	0	0	0	0	0	0	0	0	0
SAL_HA2759AA	CFSAN048260	Anatum	0	0	0	0	0	0	0	0	0	0	0
SAL_HA2091AA	S08502-07	Anatum	0	0	0	0	0	0	0	0	0	0	0
SAL_HA2101AA	S05107-06	Anatum	0	0	0	0	0	0	0	0	0	0	0
SAL_HA2051AA	S06187-07	Anatum	0	0	0	0	0	0	0	0	0	0	0
SAL_HA2096AA	S00430-07	Anatum	0	0	0	0	0	0	0	0	0	0	0
SAL_HA3988AA	FSIS1608355	Anatum	0	0	0	0	0	0	0	0	0	0	0
SAL_HA3973AA	FSIS1608222	Anatum	0	0	0	0	0	0	0	0	0	0	0
SAL_HA3527AA	IFSH00031	Anatum	0	0	0	0	0	0	0	0	0	0	0
SAL_HA4369AA	FDA00002050	Anatum	0	0	0	0	0	0	0	0	0	0	0
SAL_HA4372AA	FDA00002022	Anatum	0	0	0	0	0	0	0	0	0	0	0
SAL_HA4638AA	FSIS1608629	Anatum	0	0	0	0	0	0	0	0	0	0	0
SAL_HA4647AA	FSIS1608380	Anatum	0	0	0	0	0	0	0	0	0	0	0
SAL_HA4743AA	FSIS1608546	Anatum	0	0	0	0	0	0	0	0	0	0	0
SAL_HA4753AA	FSIS1608390	Anatum	0	0	0	0	0	0	0	0	0	0	0
SAL_HA4557AA	FSIS1608502	Anatum	0	0	0	0	0	0	0	0	0	0	0
SAL_HA4702AA	FSIS1608645	Anatum	0	0	0	0	0	0	0	0	0	0	0
SAL_HA4718AA	FSIS1608670	Anatum	0	0	0	0	0	0	0	0	0	0	0
SAL_HA4735AA	FSIS1608552	Anatum	0	0	0	0	0	0	0	0	0	0	0
SAL_HA4710AA	FSIS1608644	Anatum	0	0	0	0	0	0	0	0	0	0	0
SAL_HA5116AA	L01032-14	Anatum	0	0	0	0	0	0	0	0	0	0	0
SAL_HA4212AA	FSIS1608337	Anatum	0	0	0	0	0	0	0	0	0	0	0
SAL_HA4976AA	978821	Anatum	0	0	0	0	0	0	0	0	0	0	0
SAL_HA5485AA	CFSAN056378	Anatum	0	0	0	0	0	0	0	0	0	0	0
SAL_HA5472AA	CFSAN056368	Anatum	0	0	0	0	0	0	0	0	0	0	0
SAL_HA5510AA	CFSAN056571	Anatum	0	0	0	0	0	0	0	0	0	0	0
SAL_HA5791AA	PNUSAS005736	Anatum	0	0	0	0	0	0	0	0	0	0	0
SAL_HA2720AA	FSIS1608314	Anatum	0	0	0	0	0	0	0	0	0	0	0
SAL_HA0946AA	NSUOC-0040-S1.1X	Anatum	0	0	0	0	0	0	0	0	0	0	0
SAL_HA5645AA	S01754-15	Anatum	0	0	0	0	0	0	0	0	0	0	0
SAL_GA8603AA	FSIS1607400	Anatum	0	0	0	0	0	0	0	0	0	0	0
SAL_HA6466AA	FSIS1609098	Anatum	0	0	0	0	0	0	0	0	0	0	0
SAL_HA6460AA	FSIS1608975	Anatum	0	0	0	0	0	0	0	0	0	0	0
SAL_HA6455AA	FSIS1608976	Anatum	0	0	0	0	0	0	0	0	0	0	0
SAL_HA6467AA	FSIS1609097	Anatum	0	0	0	0	0	0	0	0	0	0	0
SAL_HA6470AA	FSIS1609103	Anatum	0	0	0	0	0	0	0	0	0	0	0
SAL_HA2645AA	FDA00010802	Anatum	0	0	0	0	0	0	0	0	0	0	0
SAL_HA2644AA	FDA00010803	Anatum	0	0	0	0	0	0	0	0	0	0	0
SAL_HA3999AA	FSIS1608135	Anatum	0	0	0	0	0	0	0	0	0	0	0
SAL_HA3991AA	FSIS1608362	Anatum	0	0	0	0	0	0	0	0	0	0	0
SAL_HA3993AA	FSIS1608365	Anatum	0	0	0	0	0	0	0	0	0	0	0
SAL_HA3983AA	FSIS1608360	Anatum	0	0	0	0	0	0	0	0	0	0	0

SAL_HA3945AA	FSIS1608407	Anatum	0	0	0	0	0	0	0	0	0	0	0
SAL_HA3951AA	FSIS1608367	Anatum	0	0	0	0	0	0	0	0	0	0	0
SAL_HA4270AA	FSIS1608156	Anatum	0	0	0	0	0	0	0	0	0	0	0
SAL_HA3944AA	FSIS1608408	Anatum	0	0	0	0	0	0	0	0	0	0	0
SAL_HA5692AA	PNUSAS005665	Anatum	0	0	0	0	0	0	0	0	0	0	0
SAL_HA6643AA	FSIS1608991	Anatum	0	0	0	0	0	0	0	0	0	0	0
SAL_HA6630AA	FSIS1609001	Anatum	0	0	0	0	0	0	0	0	0	0	0
SAL_HA6628AA	FDA00011063	Anatum	0	0	0	0	0	0	0	0	0	0	0
SAL_HA5848AA	PNUSAS005795	Anatum	0	0	0	0	0	0	0	0	0	0	0
SAL_HA6098AA	FSIS1608807	Anatum	0	0	0	0	0	0	0	0	0	0	0
SAL_HA6099AA	FSIS1608809	Anatum	0	0	0	0	0	0	0	0	0	0	0
SAL_HA6946AA	FSIS1608904	Anatum	0	0	0	0	0	0	0	0	0	0	0
SAL_HA6945AA	FSIS1608905	Anatum	0	0	0	0	0	0	0	0	0	0	0
SAL_HA6948AA	FSIS1608899	Anatum	0	0	0	0	0	0	0	0	0	0	0
SAL_HA6125AA	FSIS1608764	Anatum	0	0	0	0	0	0	0	0	0	0	0
SAL_HA6865AA	PNUSAS006131	Anatum	0	0	0	0	0	0	0	0	0	0	0
SAL_HA6853AA	PNUSAS006017	Anatum	0	0	0	0	0	0	0	0	0	0	0
SAL_HA7209AA	FDA00011079	Anatum	0	0	0	0	0	0	0	0	0	0	0
SAL_HA7213AA	FDA00011078	Anatum	0	0	0	0	0	0	0	0	0	0	0
SAL_HA7219AA	FDA00011077	Anatum	0	0	0	0	0	0	0	0	0	0	0
SAL_HA6429AA	FSIS1608775	Anatum	0	0	0	0	0	0	0	0	0	0	0
SAL_HA6354AA	PNUSAS005532	Anatum	0	0	0	0	0	0	0	0	0	0	0
SAL_HA6281AA	FDA00011009	Anatum	0	0	0	0	0	0	0	0	0	0	0
SAL_HA6185AA	FSIS1608789	Anatum	0	0	0	0	0	0	0	0	0	0	0
SAL_HA6127AA	FSIS1608768	Anatum	0	0	0	0	0	0	0	0	0	0	0
SAL_HA6129AA	FSIS1608771	Anatum	0	0	0	0	0	0	0	0	0	0	0
SAL_HA6130AA	FSIS1608766	Anatum	0	0	0	0	0	0	0	0	0	0	0
SAL_HA7604AA	CFSAN056836	Anatum	0	0	0	0	0	0	0	0	0	0	0
SAL_HA7594AA	CFSAN056850	Anatum	0	0	0	0	0	0	0	0	0	0	0
SAL_HA7602AA	CFSAN056835	Anatum	0	0	0	0	0	0	0	0	0	0	0
SAL_HA7704AA	FSIS1609208	Anatum	0	0	0	0	0	0	0	0	0	0	0
SAL_HA7716AA	FSIS1609252	Anatum	0	0	0	0	0	0	0	0	0	0	0
SAL_HA7718AA	FSIS1609209	Anatum	0	0	0	0	0	0	0	0	0	0	0
SAL_HA7646AA	FSIS1609214	Anatum	0	0	0	0	0	0	0	0	0	0	0
SAL_HA7682AA	FSIS1608777	Anatum	0	0	0	0	0	0	0	0	0	0	0
SAL_HA7687AA	FSIS1609117	Anatum	0	0	0	0	0	0	0	0	0	0	0
SAL_HA7694AA	FSIS1609233	Anatum	0	0	0	0	0	0	0	0	0	0	0
SAL_HA7715AA	FSIS1609251	Anatum	0	0	0	0	0	0	0	0	0	0	0
SAL_HA7640AA	FSIS1609216	Anatum	0	0	0	0	0	0	0	0	0	0	0
SAL_HA7774AA	FSIS1609089	Anatum	0	0	0	0	0	0	0	0	0	0	0
SAL_HA7756AA	FSIS1608896	Anatum	0	0	0	0	0	0	0	0	0	0	0
SAL_HA7729AA	PNUSAS006291	Anatum	0	0	0	0	0	0	0	0	0	0	0
SAL_HA7763AA	FSIS1608891	Anatum	0	0	0	0	0	0	0	0	0	0	0
SAL_HA8301AA	FSIS1609249	Anatum	0	0	0	0	0	0	0	0	0	0	0
SAL_HA8304AA	FSIS1609244	Anatum	0	0	0	0	0	0	0	0	0	0	0
SAL_HA8362AA	FSIS1608840	Anatum	0	0	0	0	0	0	0	0	0	0	0
SAL_HA8231AA	FSIS1609495	Anatum	0	0	0	0	0	0	0	0	0	0	0
SAL_HA8695AA	FSIS1609443	Anatum	0	0	0	0	0	0	0	0	0	0	0
SAL_HA8703AA	FSIS1609444	Anatum	0	0	0	0	0	0	0	0	0	0	0
SAL_HA8702AA	FSIS1609445	Anatum	0	0	0	0	0	0	0	0	0	0	0
SAL_HA8297AA	FSIS1609316	Anatum	0	0	0	0	0	0	0	0	0	0	0
SAL_HA8715AA	OH-16-25847	Anatum	0	0	0	0	0	0	0	0	0	0	0
SAL_HA8873AA	NCTC8258	Anatum	0	0	0	0	0	0	0	0	0	0	0
SAL_HA8803AA	NSUOC-0151-S6.1H	Anatum	0	0	0	0	0	0	0	0	0	0	0
SAL_HA8816AA	FSIS1609544	Anatum	0	0	0	0	0	0	0	0	0	0	0
SAL_HA8824AA	FSIS1609553	Anatum	0	0	0	0	0	0	0	0	0	0	0
SAL_HA8945AA	JSDA-ARS-USMARC-1766	Anatum	0	0	0	0	0	0	0	0	0	0	0
SAL_HA8947AA	JSDA-ARS-USMARC-1677	Anatum	0	0	0	0	0	0	0	0	0	0	0
SAL_HA8946AA	JSDA-ARS-USMARC-1728	Anatum	0	0	0	0	0	0	0	0	0	0	0
SAL_HA8948AA	JSDA-ARS-USMARC-1676	Anatum	0	0	0	0	0	0	0	0	0	0	0
SAL_HA9150AA	FSIS1609115	Anatum	0	0	0	0	0	0	0	0	0	0	0
SAL_HA9132AA	CFSAN058089	Anatum	0	0	0	0	0	0	0	0	0	0	0
SAL_HA9108AA	CFSAN058100	Anatum	0	0	0	0	0	0	0	0	0	0	0
SAL_HA9290AA	ADRDL-771	Anatum	0	0	0	0	0	0	0	0	0	0	0
SAL_HA9294AA	ADRDL-778	Anatum	0	0	0	0	0	0	0	0	0	0	0
SAL_HA9554AA	PNUSAS006661	Anatum	0	0	0	0	0	0	0	0	0	0	0
SAL_HA9308AA	FDA00011170	Anatum	0	0	0	0	0	0	0	0	0	0	0
SAL_HA9620AA	FSIS1609340	Anatum	0	0	0	0	0	0	0	0	0	0	0
SAL_HA9729AA	CFSAN058830	Anatum	0	0	0	0	0	0	0	0	0	0	0
SAL_HA9863AA	FSIS1609587	Anatum	0	0	0	0	0	0	0	0	0	0	0
SAL_IA0138AA	ADRDL-810	Anatum	0	0	0	0	0	0	0	0	0	0	0
SAL_IA0151AA	ADRDL-821	Anatum	0	0	0	0	0	0	0	0	0	0	0
SAL_IA0352AA	CFSAN058879	Anatum	0	0	0	0	0	0	0	0	0	0	0

SAL_IA0306AA	FSIS1609550	Anatum	0	0	0	0	0	0	0	0	0	0	0
SAL_IA0308AA	FSIS1609560	Anatum	0	0	0	0	0	0	0	0	0	0	0
SAL_IA0188AA	PNUSAS006210	Anatum	0	0	0	0	0	0	0	0	0	0	0
SAL_IA0619AA	FDA00011135	Anatum	0	0	0	0	0	0	0	0	0	0	0
SAL_IA0574AA	PNUSAS007502	Anatum	0	0	0	0	0	0	0	0	0	0	0
SAL_IA1400AA	323107	Anatum	0	0	0	0	0	0	0	0	0	0	0
SAL_IA0934AA	287776	Anatum	0	0	0	0	0	0	0	0	0	0	0
SAL_IA0756AA	232767	Anatum	0	0	0	0	0	0	0	0	0	0	0
SAL_IA2013AA	FSIS1609733	Anatum	0	0	0	0	0	0	0	0	0	0	0
SAL_IA2015AA	FSIS1609725	Anatum	0	0	0	0	0	0	0	0	0	0	0
SAL_IA2016AA	FSIS1609731	Anatum	0	0	0	0	0	0	0	0	0	0	0
SAL_IA1989AA	FSIS1609671	Anatum	0	0	0	0	0	0	0	0	0	0	0
SAL_IA2207AA	FSIS1609714	Anatum	0	0	0	0	0	0	0	0	0	0	0
SAL_IA2111AA	PNUSAS007633	Anatum	0	0	0	0	0	0	0	0	0	0	0
SAL_IA2087AA	FSIS1609574	Anatum	0	0	0	0	0	0	0	0	0	0	0
SAL_IA2074AA	FSIS1609511	Anatum	0	0	0	0	0	0	0	0	0	0	0
SAL_IA2093AA	FSIS1609619	Anatum	0	0	0	0	0	0	0	0	0	0	0
SAL_IA2200AA	FSIS1609713	Anatum	0	0	0	0	0	0	0	0	0	0	0
SAL_IA2361AA	FSIS1609402	Anatum	0	0	0	0	0	0	0	0	0	0	0
SAL_IA2347AA	FSIS1709839	Anatum	0	0	0	0	0	0	0	0	0	0	0
SAL_IA2383AA	FSIS1609384	Anatum	0	0	0	0	0	0	0	0	0	0	0
SAL_IA2363AA	FSIS1609404	Anatum	0	0	0	0	0	0	0	0	0	0	0
SAL_IA2374AA	FSIS1609390	Anatum	0	0	0	0	0	0	0	0	0	0	0
SAL_IA2752AA	CFSAN059248	Anatum	0	0	0	0	0	0	0	0	0	0	0
SAL_IA2763AA	MS160132	Anatum	0	0	0	0	0	0	0	0	0	0	0
SAL_IA2699AA	CFSAN059257	Anatum	0	0	0	0	0	0	0	0	0	0	0
SAL_IA3710AA	FSIS1710101	Anatum	0	0	0	0	0	0	0	0	0	0	0
SAL_IA4711AA	FSIS1709983	Anatum	0	0	0	0	0	0	0	0	0	0	0
SAL_IA4712AA	FSIS1709862	Anatum	0	0	0	0	0	0	0	0	0	0	0
SAL_IA4659AA	FSIS1710123	Anatum	0	0	0	0	0	0	0	0	0	0	0
SAL_IA4653AA	FSIS1709881	Anatum	0	0	0	0	0	0	0	0	0	0	0
SAL_IA4739AA	MS160207	Anatum	0	0	0	0	0	0	0	0	0	0	0
SAL_IA4860AA	FSIS1709857	Anatum	0	0	0	0	0	0	0	0	0	0	0
SAL_IA4885AA	FSIS1710158	Anatum	0	0	0	0	0	0	0	0	0	0	0
SAL_IA4904AA	FSIS1710221	Anatum	0	0	0	0	0	0	0	0	0	0	0
SAL_IA4929AA	CFSAN059303	Anatum	0	0	0	0	0	0	0	0	0	0	0
SAL_IA5151AA	CFSAN058122	Anatum	0	0	0	0	0	0	0	0	0	0	0
SAL_IA5072AA	MS160293	Anatum	0	0	0	0	0	0	0	0	0	0	0
SAL_IA5360AA	FSIS1710310	Anatum	0	0	0	0	0	0	0	0	0	0	0
SAL_IA5354AA	FSIS1710001	Anatum	0	0	0	0	0	0	0	0	0	0	0
SAL_IA5930AA	MS160372	Anatum	0	0	0	0	0	0	0	0	0	0	0
SAL_IA6263AA	PNUSAS007937	Anatum	0	0	0	0	0	0	0	0	0	0	0
SAL_IA6270AA	CFSAN059142	Anatum	0	0	0	0	0	0	0	0	0	0	0
SAL_IA5578AA	FSIS1710237	Anatum	0	0	0	0	0	0	0	0	0	0	0
SAL_IA5565AA	FSIS1709860	Anatum	0	0	0	0	0	0	0	0	0	0	0
SAL_IA5549AA	FSIS1710384	Anatum	0	0	0	0	0	0	0	0	0	0	0
SAL_IA5560AA	FSIS1710383	Anatum	0	0	0	0	0	0	0	0	0	0	0
SAL_IA5558AA	FSIS1710180	Anatum	0	0	0	0	0	0	0	0	0	0	0
SAL_IA5706AA	FDA00011250	Anatum	0	0	0	0	0	0	0	0	0	0	0
SAL_IA5689AA	PNUSAS008140	Anatum	0	0	0	0	0	0	0	0	0	0	0
SAL_IA5488AA	FSIS1710352	Anatum	0	0	0	0	0	0	0	0	0	0	0
SAL_IA5465AA	FSIS1710347	Anatum	0	0	0	0	0	0	0	0	0	0	0
SAL_IA5468AA	FSIS1710239	Anatum	0	0	0	0	0	0	0	0	0	0	0
SAL_IA5705AA	FDA00011249	Anatum	0	0	0	0	0	0	0	0	0	0	0
SAL_IA5628AA	PNUSAS007808	Anatum	0	0	0	0	0	0	0	0	0	0	0
SAL_IA6407AA	FSIS1710514	Anatum	0	0	0	0	0	0	0	0	0	0	0
SAL_IA6510AA	PNUSAS008282	Anatum	0	0	0	0	0	0	0	0	0	0	0
SAL_IA6555AA	CFSAN058765	Anatum	0	0	0	0	0	0	0	0	0	0	0
SAL_IA6591AA	FSIS1710360	Anatum	0	0	0	0	0	0	0	0	0	0	0
SAL_IA6592AA	FSIS1710355	Anatum	0	0	0	0	0	0	0	0	0	0	0
SAL_IA6624AA	FSIS1710369	Anatum	0	0	0	0	0	0	0	0	0	0	0
SAL_IA6613AA	FSIS1710359	Anatum	0	0	0	0	0	0	0	0	0	0	0
SAL_IA6668AA	FSIS1710072	Anatum	0	0	0	0	0	0	0	0	0	0	0
SAL_IA6667AA	FSIS1710294	Anatum	0	0	0	0	0	0	0	0	0	0	0
SAL_IA6653AA	PNUSAS008329	Anatum	0	0	0	0	0	0	0	0	0	0	0
SAL_IA6682AA	FSIS1710258	Anatum	0	0	0	0	0	0	0	0	0	0	0
SAL_IA6638AA	FDA00011278	Anatum	0	0	0	0	0	0	0	0	0	0	0
SAL_IA7166AA	ADRDL-941	Anatum	0	0	0	0	0	0	0	0	0	0	0
SAL_IA7082AA	CFSAN059785	Anatum	0	0	0	0	0	0	0	0	0	0	0
SAL_IA7419AA	CFSAN060231	Anatum	0	0	0	0	0	0	0	0	0	0	0
SAL_IA7421AA	CFSAN060230	Anatum	0	0	0	0	0	0	0	0	0	0	0
SAL_IA7563AA	FSIS1710372	Anatum	0	0	0	0	0	0	0	0	0	0	0
SAL_IA7556AA	FSIS1710374	Anatum	0	0	0	0	0	0	0	0	0	0	0

SAL_IA7527AA	FSIS1710446	Anatum	0	0	0	0	0	0	0	0	0	0	0
SAL_IA7531AA	FSIS1710521	Anatum	0	0	0	0	0	0	0	0	0	0	0
SAL_IA7639AA	FDA00011293	Anatum	0	0	0	0	0	0	0	0	0	0	0
SAL_IA7672AA	FSIS1709975	Anatum	0	0	0	0	0	0	0	0	0	0	0
SAL_IA7824AA	MDP105	Anatum	0	0	0	0	0	0	0	0	0	0	0
SAL_IA8199AA	ADRDL-818	Anatum	0	0	0	0	0	0	0	0	0	0	0
SAL_IA8402AA	PNUSAS007493	Anatum	0	0	0	0	0	0	0	0	0	0	0
SAL_IA8748AA	CFSAN057172	Anatum	0	0	0	0	0	0	0	0	0	0	0
SAL_IA8729AA	CFSAN057191	Anatum	0	0	0	0	0	0	0	0	0	0	0
SAL_IA8730AA	CFSAN057190	Anatum	0	0	0	0	0	0	0	0	0	0	0
SAL_IA8736AA	CFSAN057189	Anatum	0	0	0	0	0	0	0	0	0	0	0
SAL_IA8724AA	CFSAN057193	Anatum	0	0	0	0	0	0	0	0	0	0	0
SAL_IA8620AA	CFSAN057295	Anatum	0	0	0	0	0	0	0	0	0	0	0
SAL_IA8624AA	CFSAN057296	Anatum	0	0	0	0	0	0	0	0	0	0	0
SAL_IA8625AA	CFSAN057298	Anatum	0	0	0	0	0	0	0	0	0	0	0
SAL_IA9031AA	FSIS1710395	Anatum	0	0	0	0	0	0	0	0	0	0	0
SAL_IA9000AA	FSIS1710848	Anatum	0	0	0	0	0	0	0	0	0	0	0
SAL_IA9003AA	FSIS1710858	Anatum	0	0	0	0	0	0	0	0	0	0	0
SAL_IA9020AA	FSIS1710578	Anatum	0	0	0	0	0	0	0	0	0	0	0
SAL_IA9034AA	FSIS1710577	Anatum	0	0	0	0	0	0	0	0	0	0	0
SAL_IA8623AA	CFSAN057297	Anatum	0	0	0	0	0	0	0	0	0	0	0
SAL_IA8985AA	FSIS1710851	Anatum	0	0	0	0	0	0	0	0	0	0	0
SAL_IA9442AA	PNUSAS006944	Anatum	0	0	0	0	0	0	0	0	0	0	0
SAL_IA9370AA	PNUSAS009549	Anatum	0	0	0	0	0	0	0	0	0	0	0
SAL_IA9187AA	PNUSAS010016	Anatum	0	0	0	0	0	0	0	0	0	0	0
SAL_IA9152AA	PNUSAS010092	Anatum	0	0	0	0	0	0	0	0	0	0	0
SAL_IA9627AA	PNUSAS009996	Anatum	0	0	0	0	0	0	0	0	0	0	0
SAL_IA9629AA	PNUSAS009998	Anatum	0	0	0	0	0	0	0	0	0	0	0
SAL_IA9628AA	PNUSAS009999	Anatum	0	0	0	0	0	0	0	0	0	0	0
SAL_IA9613AA	PNUSAS009595	Anatum	0	0	0	0	0	0	0	0	0	0	0
SAL_IA9626AA	PNUSAS010000	Anatum	0	0	0	0	0	0	0	0	0	0	0
SAL_IA9630AA	PNUSAS009997	Anatum	0	0	0	0	0	0	0	0	0	0	0
SAL_IA9777AA	FSIS1711027	Anatum	0	0	0	0	0	0	0	0	0	0	0
SAL_IA9753AA	FSIS1710941	Anatum	0	0	0	0	0	0	0	0	0	0	0
SAL_IA9776AA	FSIS1711032	Anatum	0	0	0	0	0	0	0	0	0	0	0
SAL_IA9784AA	FSIS1711028	Anatum	0	0	0	0	0	0	0	0	0	0	0
SAL_IA9787AA	FSIS1711030	Anatum	0	0	0	0	0	0	0	0	0	0	0
SAL_IA9792AA	FSIS1711038	Anatum	0	0	0	0	0	0	0	0	0	0	0
SAL_IA9739AA	FSIS1710559	Anatum	0	0	0	0	0	0	0	0	0	0	0
SAL_IA9765AA	FSIS1710936	Anatum	0	0	0	0	0	0	0	0	0	0	0
SAL_IA9712AA	FSIS1710947	Anatum	0	0	0	0	0	0	0	0	0	0	0
SAL_IA9683AA	PNUSAS010003	Anatum	0	0	0	0	0	0	0	0	0	0	0
SAL_IA9684AA	PNUSAS010002	Anatum	0	0	0	0	0	0	0	0	0	0	0
SAL_IA9915AA	PNUSAS010001	Anatum	0	0	0	0	0	0	0	0	0	0	0
SAL_IA9917AA	PNUSAS009577	Anatum	0	0	0	0	0	0	0	0	0	0	0
SAL_JA0041AA	FSIS1710871	Anatum	0	0	0	0	0	0	0	0	0	0	0
SAL_JA0048AA	FSIS1710721	Anatum	0	0	0	0	0	0	0	0	0	0	0
SAL_JA0028AA	FSIS1710884	Anatum	0	0	0	0	0	0	0	0	0	0	0
SAL_JA0192AA	PNUSAS009928	Anatum	0	0	0	0	0	0	0	0	0	0	0
SAL_JA0155AA	PNUSAS010401	Anatum	0	0	0	0	0	0	0	0	0	0	0
SAL_JA0094AA	PNUSAS010402	Anatum	0	0	0	0	0	0	0	0	0	0	0
SAL_JA0508AA	PNUSAS010554	Anatum	0	0	0	0	0	0	0	0	0	0	0
SAL_JA0448AA	PNUSAS010325	Anatum	0	0	0	0	0	0	0	0	0	0	0
SAL_JA0842AA	ADRDL-1017	Anatum	0	0	0	0	0	0	0	0	0	0	0
SAL_JA0966AA	PNUSAS009927	Anatum	0	0	0	0	0	0	0	0	0	0	0
SAL_JA1209AA	PNUSAS011064	Anatum	0	0	0	0	0	0	0	0	0	0	0
SAL_JA1570AA	FDA00011408	Anatum	0	0	0	0	0	0	0	0	0	0	0
SAL_JA1416AA	NSUOC-0175-W1H	Anatum	0	0	0	0	0	0	0	0	0	0	0
SAL_JA1159AA	ADRDL-889	Anatum	0	0	0	0	0	0	0	0	0	0	0
SAL_JA1008AA	PNUSAS010991	Anatum	0	0	0	0	0	0	0	0	0	0	0
SAL_JA0950AA	PNUSAS009929	Anatum	0	0	0	0	0	0	0	0	0	0	0
SAL_JA0947AA	PNUSAS010372	Anatum	0	0	0	0	0	0	0	0	0	0	0
SAL_JA0948AA	PNUSAS009930	Anatum	0	0	0	0	0	0	0	0	0	0	0
SAL_JA0913AA	PNUSAS009610	Anatum	0	0	0	0	0	0	0	0	0	0	0
SAL_JA0483AA	ADRDL-1005	Anatum	0	0	0	0	0	0	0	0	0	0	0
SAL_JA0476AA	ADRDL-1037	Anatum	0	0	0	0	0	0	0	0	0	0	0
SAL_IA7696AA	MS160193	Anatum	0	0	0	0	0	0	0	0	0	0	0
SAL_JA0227AA	2017K-0188	Anatum	0	0	0	0	0	0	0	0	0	0	0
SAL_JA1692AA	TX-ACF1700702	Anatum	0	0	0	0	0	0	0	0	0	0	0
SAL_JA1616AA	PNUSAS011340	Anatum	0	0	0	0	0	0	0	0	0	0	0
SAL_JA0431AA	PNUSAS010682	Anatum	0	0	0	0	0	0	0	0	0	0	0
SAL_JA1866AA	FSIS1710966	Anatum	0	0	0	0	0	0	0	0	0	0	0
SAL_JA1868AA	FSIS1710967	Anatum	0	0	0	0	0	0	0	0	0	0	0

SAL_JA2192AA	FSIS1711062	Anatum	0	0	0	0	0	0	0	0	0	0	0
SAL_JA2140AA	FSIS1700108	Anatum	0	0	0	0	0	0	0	0	0	0	0
SAL_JA2107AA	FSIS1710679	Anatum	0	0	0	0	0	0	0	0	0	0	0
SAL_JA2054AA	FSIS1700074	Anatum	0	0	0	0	0	0	0	0	0	0	0
SAL_JA2336AA	HIY0051	Anatum	0	0	0	0	0	0	0	0	0	0	0
SAL_JA2333AA	HIY0059	Anatum	0	0	0	0	0	0	0	0	0	0	0
SAL_JA2332AA	HIY0055	Anatum	0	0	0	0	0	0	0	0	0	0	0
SAL_JA2305AA	FSIS1700271	Anatum	0	0	0	0	0	0	0	0	0	0	0
SAL_JA2360AA	FSIS1710671	Anatum	0	0	0	0	0	0	0	0	0	0	0
SAL_JA2690AA	HIY0060	Anatum	0	0	0	0	0	0	0	0	0	0	0
SAL_JA2684AA	PNUSAS010880	Anatum	0	0	0	0	0	0	0	0	0	0	0
SAL_JA2614AA	PNUSAS011403	Anatum	0	0	0	0	0	0	0	0	0	0	0
SAL_JA2605AA	PNUSAS011751	Anatum	0	0	0	0	0	0	0	0	0	0	0
SAL_JA2430AA	PNUSAS011579	Anatum	0	0	0	0	0	0	0	0	0	0	0
SAL_JA2806AA	FSIS1700216	Anatum	0	0	0	0	0	0	0	0	0	0	0
SAL_JA2817AA	VA-WGS-17055	Anatum	0	0	0	0	0	0	0	0	0	0	0
SAL_JA2802AA	FSIS1711017	Anatum	0	0	0	0	0	0	0	0	0	0	0
SAL_JA2843AA	FSIS1700221	Anatum	0	0	0	0	0	0	0	0	0	0	0
SAL_JA2835AA	FSIS1700218	Anatum	0	0	0	0	0	0	0	0	0	0	0
SAL_JA2960AA	FSIS1700064	Anatum	0	0	0	0	0	0	0	0	0	0	0
SAL_JA2949AA	2017K-0297	Anatum	0	0	0	0	0	0	0	0	0	0	0
SAL_JA2952AA	FSIS1700434	Anatum	0	0	0	0	0	0	0	0	0	0	0
SAL_JA2963AA	FSIS1700067	Anatum	0	0	0	0	0	0	0	0	0	0	0
SAL_JA2929AA	PNUSAS010336	Anatum	0	0	0	0	0	0	0	0	0	0	0
SAL_JA2912AA	PNUSAS011718	Anatum	0	0	0	0	0	0	0	0	0	0	0
SAL_JA2958AA	FSIS1700435	Anatum	0	0	0	0	0	0	0	0	0	0	0
SAL_JA2948AA	2017K-0295	Anatum	0	0	0	0	0	0	0	0	0	0	0
SAL_JA2950AA	2017K-0296	Anatum	0	0	0	0	0	0	0	0	0	0	0
SAL_JA3433AA	FSIS1700738	Anatum	0	0	0	0	0	0	0	0	0	0	0
SAL_JA3391AA	PNUSAS011371	Anatum	0	0	0	0	0	0	0	0	0	0	0
SAL_JA3428AA	FSIS1700420	Anatum	0	0	0	0	0	0	0	0	0	0	0
SAL_JA3452AA	FSIS1700287	Anatum	0	0	0	0	0	0	0	0	0	0	0
SAL_JA3401AA	PNUSAS011199	Anatum	0	0	0	0	0	0	0	0	0	0	0
SAL_JA3398AA	PNUSAS012246	Anatum	0	0	0	0	0	0	0	0	0	0	0
SAL_JA3149AA	PNUSAS011492	Anatum	0	0	0	0	0	0	0	0	0	0	0
SAL_JA3060AA	CNSV-T2-MD04-10-TT-D	Anatum	0	0	0	0	0	0	0	0	0	0	0
SAL_JA3082AA	CNSV-T2-MD04-10-RV-E	Anatum	0	0	0	0	0	0	0	0	0	0	0
SAL_JA3087AA	CNSV-T2-MD04-10-RV-D	Anatum	0	0	0	0	0	0	0	0	0	0	0
SAL_JA3057AA	CNSV-T2-MD04-10-TT-F	Anatum	0	0	0	0	0	0	0	0	0	0	0
SAL_JA3058AA	CNSV-T2-MD04-10-TT-E	Anatum	0	0	0	0	0	0	0	0	0	0	0
SAL_JA3599AA	FSIS1700438	Anatum	0	0	0	0	0	0	0	0	0	0	0
SAL_JA3539AA	PNUSAS012390	Anatum	0	0	0	0	0	0	0	0	0	0	0
SAL_JA3488AA	PNUSAS011200	Anatum	0	0	0	0	0	0	0	0	0	0	0
SAL_JA3976AA	PNUSAS011978	Anatum	0	0	0	0	0	0	0	0	0	0	0
SAL_JA3921AA	PNUSAS012249	Anatum	0	0	0	0	0	0	0	0	0	0	0
SAL_JA4207AA	FSIS1700449	Anatum	0	0	0	0	0	0	0	0	0	0	0
SAL_JA3751AA	PNUSAS012817	Anatum	0	0	0	0	0	0	0	0	0	0	0
SAL_JA4475AA	OH-16-17139-1	Anatum	0	0	0	0	0	0	0	0	0	0	0
SAL_JA4476AA	OH-16-16468-1	Anatum	0	0	0	0	0	0	0	0	0	0	0
SAL_JA4470AA	OH-16-17139-3	Anatum	0	0	0	0	0	0	0	0	0	0	0
SAL_JA5039AA	FSIS1701150	Anatum	0	0	0	0	0	0	0	0	0	0	0
SAL_JA5047AA	FSIS1700731	Anatum	0	0	0	0	0	0	0	0	0	0	0
SAL_JA5046AA	FSIS1701139	Anatum	0	0	0	0	0	0	0	0	0	0	0
SAL_JA4929AA	PNUSAS006237	Anatum	0	0	0	0	0	0	0	0	0	0	0
SAL_JA6281AA	PNUSAS013682	Anatum	0	0	0	0	0	0	0	0	0	0	0
SAL_JA6166AA	PNUSAS011150	Anatum	0	0	0	0	0	0	0	0	0	0	0
SAL_JA6057AA	FSIS1701108	Anatum	0	0	0	0	0	0	0	0	0	0	0
SAL_JA6050AA	FSIS1700934	Anatum	0	0	0	0	0	0	0	0	0	0	0
SAL_JA6054AA	FSIS1701112	Anatum	0	0	0	0	0	0	0	0	0	0	0
SAL_JA6028AA	FSIS1701081	Anatum	0	0	0	0	0	0	0	0	0	0	0
SAL_JA6036AA	FSIS1701076	Anatum	0	0	0	0	0	0	0	0	0	0	0
SAL_JA6035AA	FSIS1701079	Anatum	0	0	0	0	0	0	0	0	0	0	0
SAL_JA5506AA	PNUSAS004733	Anatum	0	0	0	0	0	0	0	0	0	0	0
SAL_JA6037AA	FSIS1700779	Anatum	0	0	0	0	0	0	0	0	0	0	0
SAL_JA6742AA	FSIS1701038	Anatum	0	0	0	0	0	0	0	0	0	0	0
SAL_JA6760AA	FSIS1700899	Anatum	0	0	0	0	0	0	0	0	0	0	0
SAL_JA6934AA	FSIS1701297	Anatum	0	0	0	0	0	0	0	0	0	0	0
SAL_JA6936AA	FSIS1701296	Anatum	0	0	0	0	0	0	0	0	0	0	0
SAL_JA6937AA	FSIS1701298	Anatum	0	0	0	0	0	0	0	0	0	0	0
SAL_JA6029AA	FSIS1701080	Anatum	0	0	0	0	0	0	0	0	0	0	0
SAL_JA7370AA	PNUSAS014323	Anatum	0	0	0	0	0	0	0	0	0	0	0
SAL_JA7246AA	FSIS1700722	Anatum	0	0	0	0	0	0	0	0	0	0	0
SAL_JA7238AA	FSIS1701448	Anatum	0	0	0	0	0	0	0	0	0	0	0

SAL_JA8366AA	364187	Anatum	0	0	0	0	0	0	0	0	0	0	0
SAL_JA8060AA	355042	Anatum	0	0	0	0	0	0	0	0	0	0	0
SAL_JA7993AA	355112	Anatum	0	0	0	0	0	0	0	0	0	0	0
SAL_JA7875AA	358030	Anatum	0	0	0	0	0	0	0	0	0	0	0
SAL_JA7743AA	PNUSAS013413	Anatum	0	0	0	0	0	0	0	0	0	0	0
SAL_JA7766AA	PNUSAS014340	Anatum	0	0	0	0	0	0	0	0	0	0	0
SAL_JA7706AA	PNUSAS014235	Anatum	0	0	0	0	0	0	0	0	0	0	0
SAL_JA8005AA	343788	Anatum	0	0	0	0	0	0	0	0	0	0	0
SAL_JA7407AA	PNUSAS012118	Anatum	0	0	0	0	0	0	0	0	0	0	0
SAL_JA7408AA	PNUSAS014269	Anatum	0	0	0	0	0	0	0	0	0	0	0
SAL_JA8740AA	PNUSAS014619	Anatum	0	0	0	0	0	0	0	0	0	0	0
SAL_JA8783AA	FSIS1700929	Anatum	0	0	0	0	0	0	0	0	0	0	0
SAL_JA8776AA	FSIS1700769	Anatum	0	0	0	0	0	0	0	0	0	0	0
SAL_JA8777AA	FSIS1700771	Anatum	0	0	0	0	0	0	0	0	0	0	0
SAL_JA8778AA	FSIS1700935	Anatum	0	0	0	0	0	0	0	0	0	0	0
SAL_JA8786AA	FSIS1700587	Anatum	0	0	0	0	0	0	0	0	0	0	0
SAL_JA8573AA	FSIS1701379	Anatum	0	0	0	0	0	0	0	0	0	0	0
SAL_JA8580AA	FSIS1701090	Anatum	0	0	0	0	0	0	0	0	0	0	0
SAL_JA8597AA	FSIS1700873	Anatum	0	0	0	0	0	0	0	0	0	0	0
SAL_JA8566AA	FSIS1701547	Anatum	0	0	0	0	0	0	0	0	0	0	0
SAL_JA8584AA	FSIS1701086	Anatum	0	0	0	0	0	0	0	0	0	0	0
SAL_JA9648AA	FDA00002780	Anatum	0	0	0	0	0	0	0	0	0	0	0
SAL_JA9600AA	NSUOC-0350-S2-1X	Anatum	0	0	0	0	0	0	0	0	0	0	0
SAL_JA9619AA	NSUOC-0355-S5-1X	Anatum	0	0	0	0	0	0	0	0	0	0	0
SAL_JA9589AA	NSUOC-0366-W3B	Anatum	0	0	0	0	0	0	0	0	0	0	0
SAL_JA9524AA	NSUOC-0367-W1X	Anatum	0	0	0	0	0	0	0	0	0	0	0
SAL_JA9551AA	NSUOC-0354-W5H	Anatum	0	0	0	0	0	0	0	0	0	0	0
SAL_JA9555AA	NSUOC-0357-S5-2X	Anatum	0	0	0	0	0	0	0	0	0	0	0
SAL_JA8849AA	FSIS1701279	Anatum	0	0	0	0	0	0	0	0	0	0	0
SAL_JA8905AA	FSIS1701845	Anatum	0	0	0	0	0	0	0	0	0	0	0
SAL_JA9728AA	FSIS1701470	Anatum	0	0	0	0	0	0	0	0	0	0	0
SAL_JA9742AA	FSIS1701633	Anatum	0	0	0	0	0	0	0	0	0	0	0
SAL_JA9720AA	FSIS1701833	Anatum	0	0	0	0	0	0	0	0	0	0	0
SAL_JA8931AA	FSIS1701803	Anatum	0	0	0	0	0	0	0	0	0	0	0
SAL_JA8862AA	FSIS1701477	Anatum	0	0	0	0	0	0	0	0	0	0	0
SAL_JA9203AA	FSIS1700997	Anatum	0	0	0	0	0	0	0	0	0	0	0
SAL_JA9616AA	NSUOC-0349-W2B	Anatum	0	0	0	0	0	0	0	0	0	0	0
SAL_JA9157AA	PNUSAS014752	Anatum	0	0	0	0	0	0	0	0	0	0	0
SAL_JA9060AA	PNUSAS013948	Anatum	0	0	0	0	0	0	0	0	0	0	0
SAL_JA9093AA	PNUSAS015114	Anatum	0	0	0	0	0	0	0	0	0	0	0
SAL_JA8858AA	FSIS1701285	Anatum	0	0	0	0	0	0	0	0	0	0	0
SAL_JA8938AA	FSIS1701808	Anatum	0	0	0	0	0	0	0	0	0	0	0
SAL_JA8846AA	FSIS1701282	Anatum	0	0	0	0	0	0	0	0	0	0	0
SAL_JA9133AA	PNUSAS014607	Anatum	0	0	0	0	0	0	0	0	0	0	0
SAL_JA8954AA	FSIS1701806	Anatum	0	0	0	0	0	0	0	0	0	0	0
SAL_JA9160AA	PNUSAS014744	Anatum	0	0	0	0	0	0	0	0	0	0	0
SAL_JA9833AA	FSIS1701910	Anatum	0	0	0	0	0	0	0	0	0	0	0
SAL_JA9849AA	FSIS1701920	Anatum	0	0	0	0	0	0	0	0	0	0	0
SAL_JA9804AA	FSIS1701593	Anatum	0	0	0	0	0	0	0	0	0	0	0
SAL_JA9807AA	FSIS1701734	Anatum	0	0	0	0	0	0	0	0	0	0	0
SAL_KA0657AA	FSIS1702120	Anatum	0	0	0	0	0	0	0	0	0	0	0
SAL_KA0656AA	FSIS1702109	Anatum	0	0	0	0	0	0	0	0	0	0	0
SAL_KA0468AA	PNUSAS012975	Anatum	0	0	0	0	0	0	0	0	0	0	0
SAL_KA1029AA	FSIS1702052	Anatum	0	0	0	0	0	0	0	0	0	0	0
SAL_KA1031AA	FSIS1702043	Anatum	0	0	0	0	0	0	0	0	0	0	0
SAL_KA1079AA	HIY0079	Anatum	0	0	0	0	0	0	0	0	0	0	0
SAL_KA1017AA	FSIS1702245	Anatum	0	0	0	0	0	0	0	0	0	0	0
SAL_KA1037AA	FSIS1702055	Anatum	0	0	0	0	0	0	0	0	0	0	0
SAL_KA0987AA	2015AM-0115	Anatum	0	0	0	0	0	0	0	0	0	0	0
SAL_KA1068AA	PNUSAS016252	Anatum	0	0	0	0	0	0	0	0	0	0	0
SAL_KA1035AA	FSIS1702034	Anatum	0	0	0	0	0	0	0	0	0	0	0
SAL_KA1254AA	PNUSAS014874	Anatum	0	0	0	0	0	0	0	0	0	0	0
SAL_KA1034AA	FSIS1702039	Anatum	0	0	0	0	0	0	0	0	0	0	0
SAL_KA1600AA	FSIS1701731	Anatum	0	0	0	0	0	0	0	0	0	0	0
SAL_KA1605AA	FSIS1701721	Anatum	0	0	0	0	0	0	0	0	0	0	0
SAL_KA1113AA	2015AM-2096	Anatum	0	0	0	0	0	0	0	0	0	0	0
SAL_KA1793AA	PNUSAS016203	Anatum	0	0	0	0	0	0	0	0	0	0	0
SAL_KA1682AA	FSIS1702289	Anatum	0	0	0	0	0	0	0	0	0	0	0
SAL_KA1078AA	HIY0078	Anatum	0	0	0	0	0	0	0	0	0	0	0
SAL_KA2035AA	FSIS1702202	Anatum	0	0	0	0	0	0	0	0	0	0	0
SAL_KA2141AA	FDA00000519	Anatum	0	0	0	0	0	0	0	0	0	0	0
SAL_KA2140AA	FDA00000503	Anatum	0	0	0	0	0	0	0	0	0	0	0
SAL_KA2123AA	FDA00011990	Anatum	0	0	0	0	0	0	0	0	0	0	0

SAL_KA2126AA	FDA00011989	Anatum	0	0	0	0	0	0	0	0	0	0	0
SAL_KA2116AA	FDA00011987	Anatum	0	0	0	0	0	0	0	0	0	0	0
SAL_KA2120AA	FDA00011986	Anatum	0	0	0	0	0	0	0	0	0	0	0
SAL_KA2117AA	FDA00011985	Anatum	0	0	0	0	0	0	0	0	0	0	0
SAL_KA2278AA	14-12-20170513	Anatum	0	0	0	0	0	0	0	0	0	0	0
SAL_KA2965AA	FSIS1701893	Anatum	0	0	0	0	0	0	0	0	0	0	0
SAL_KA2880AA	FSIS1702539	Anatum	0	0	0	0	0	0	0	0	0	0	0
SAL_KA2774AA	PNUSAS015218	Anatum	0	0	0	0	0	0	0	0	0	0	0
SAL_KA2393AA	FSIS1702227	Anatum	0	0	0	0	0	0	0	0	0	0	0
SAL_KA2406AA	FSIS1702505	Anatum	0	0	0	0	0	0	0	0	0	0	0
SAL_KA2394AA	FSIS1702221	Anatum	0	0	0	0	0	0	0	0	0	0	0
SAL_KA2351AA	2015AM-1521	Anatum	0	0	0	0	0	0	0	0	0	0	0
SAL_KA3088AA	CNSV-T5-MD04-10-TT-B	Anatum	0	0	0	0	0	0	0	0	0	0	0
SAL_KA3113AA	CNSV-T5-MD04-10-RV-A	Anatum	0	0	0	0	0	0	0	0	0	0	0
SAL_KA2380AA	FSIS1702588	Anatum	0	0	0	0	0	0	0	0	0	0	0
SAL_KA3141AA	PNUSAS016444	Anatum	0	0	0	0	0	0	0	0	0	0	0
SAL_KA3006AA	FSIS1702352	Anatum	0	0	0	0	0	0	0	0	0	0	0
SAL_KA3014AA	FSIS1702339	Anatum	0	0	0	0	0	0	0	0	0	0	0
SAL_KA2414AA	FSIS1702222	Anatum	0	0	0	0	0	0	0	0	0	0	0
SAL_KA2371AA	FSIS1702589	Anatum	0	0	0	0	0	0	0	0	0	0	0
SAL_KA3008AA	FSIS1702468	Anatum	0	0	0	0	0	0	0	0	0	0	0
SAL_KA2398AA	FSIS1702502	Anatum	0	0	0	0	0	0	0	0	0	0	0
SAL_KA3090AA	CNSV-T5-MD04-10-TT-C	Anatum	0	0	0	0	0	0	0	0	0	0	0
SAL_KA3488AA	FSIS1702499	Anatum	0	0	0	0	0	0	0	0	0	0	0
SAL_KA3244AA	PNUSAS017586	Anatum	0	0	0	0	0	0	0	0	0	0	0
SAL_KA3590AA	FSIS1702513	Anatum	0	0	0	0	0	0	0	0	0	0	0
SAL_KA3581AA	FSIS1702519	Anatum	0	0	0	0	0	0	0	0	0	0	0
SAL_KA3713AA	FSIS1702804	Anatum	0	0	0	0	0	0	0	0	0	0	0
SAL_KA3719AA	FSIS1702806	Anatum	0	0	0	0	0	0	0	0	0	0	0
SAL_KA3689AA	FSIS1701270	Anatum	0	0	0	0	0	0	0	0	0	0	0
SAL_KA3707AA	FSIS1702809	Anatum	0	0	0	0	0	0	0	0	0	0	0
SAL_KA4841AA	PNUSAS015457	Anatum	0	0	0	0	0	0	0	0	0	0	0
SAL_KA4652AA	PNUSAS018268	Anatum	0	0	0	0	0	0	0	0	0	0	0
SAL_KA4588AA	FSIS1702364	Anatum	0	0	0	0	0	0	0	0	0	0	0
SAL_KA8724AA	SARB2 (Fidelma Boyd)	Anatum	0	0	0	0	0	0	0	0	0	0	0
SAL_KA4122AA	H103540827	Anatum	0	0	0	0	0	0	0	0	0	0	0
SAL_KA4061AA	RM_392	Anatum	0	0	0	0	0	0	0	0	0	0	0
SAL_KA4003AA	RM_473	Anatum	0	0	0	0	0	0	0	0	0	0	0
SAL_KA3975AA	RM_226	Anatum	0	0	0	0	0	0	0	0	0	0	0
SAL_KA4979AA	FSIS1701374	Anatum	0	0	0	0	0	0	0	0	0	0	0
SAL_KA4943AA	PNUSAS017895	Anatum	0	0	0	0	0	0	0	0	0	0	0
SAL_KA5038AA	CFSAN065053	Anatum	0	0	0	0	0	0	0	0	0	0	0
SAL_KA5547AA	FSIS1703077	Anatum	0	0	0	0	0	0	0	0	0	0	0
SAL_KA5554AA	FSIS1703069	Anatum	0	0	0	0	0	0	0	0	0	0	0
SAL_KA5553AA	FSIS1703071	Anatum	0	0	0	0	0	0	0	0	0	0	0
SAL_KA5598AA	FSIS1702924	Anatum	0	0	0	0	0	0	0	0	0	0	0
SAL_KA5136AA	FSIS1702975	Anatum	0	0	0	0	0	0	0	0	0	0	0
SAL_KA5128AA	FSIS1702928	Anatum	0	0	0	0	0	0	0	0	0	0	0
SAL_KA5129AA	FSIS1702989	Anatum	0	0	0	0	0	0	0	0	0	0	0
SAL_KA5727AA	FSIS1702795	Anatum	0	0	0	0	0	0	0	0	0	0	0
SAL_KA5729AA	FSIS1703087	Anatum	0	0	0	0	0	0	0	0	0	0	0
SAL_KA5732AA	FSIS1702990	Anatum	0	0	0	0	0	0	0	0	0	0	0
SAL_KA5741AA	FSIS1703090	Anatum	0	0	0	0	0	0	0	0	0	0	0
SAL_KA5688AA	2015AM-1722	Anatum	0	0	0	0	0	0	0	0	0	0	0
SAL_KA5718AA	FSIS1703082	Anatum	0	0	0	0	0	0	0	0	0	0	0
SAL_KA6097AA	CFSAN065566	Anatum	0	0	0	0	0	0	0	0	0	0	0
SAL_KA6110AA	CFSAN065544	Anatum	0	0	0	0	0	0	0	0	0	0	0
SAL_KA6107AA	CFSAN065550	Anatum	0	0	0	0	0	0	0	0	0	0	0
SAL_KA6091AA	CFSAN065541	Anatum	0	0	0	0	0	0	0	0	0	0	0
SAL_KA6177AA	FSIS1703124	Anatum	0	0	0	0	0	0	0	0	0	0	0
SAL_KA6068AA	PNUSAS018247	Anatum	0	0	0	0	0	0	0	0	0	0	0
SAL_KA6274AA	FSIS1703141	Anatum	0	0	0	0	0	0	0	0	0	0	0
SAL_KA6282AA	FSIS1703128	Anatum	0	0	0	0	0	0	0	0	0	0	0
SAL_KA6310AA	FSIS1703205	Anatum	0	0	0	0	0	0	0	0	0	0	0
SAL_KA6216AA	CFSAN064276	Anatum	0	0	0	0	0	0	0	0	0	0	0
SAL_KA7030AA	PNUSAS017674	Anatum	0	0	0	0	0	0	0	0	0	0	0
SAL_KA6992AA	PNUSAS017664	Anatum	0	0	0	0	0	0	0	0	0	0	0
SAL_KA6704AA	FSIS1703222	Anatum	0	0	0	0	0	0	0	0	0	0	0
SAL_KA6639AA	FSIS1703217	Anatum	0	0	0	0	0	0	0	0	0	0	0
SAL_KA6653AA	FSIS1703223	Anatum	0	0	0	0	0	0	0	0	0	0	0
SAL_KA7114AA	FSIS1703315	Anatum	0	0	0	0	0	0	0	0	0	0	0
SAL_KA7138AA	FSIS1703186	Anatum	0	0	0	0	0	0	0	0	0	0	0
SAL_KA7540AA	PNUSAS019612	Anatum	0	0	0	0	0	0	0	0	0	0	0

SAL_KA7335AA	PNUSAS018962	Anatum	0	0	0	0	0	0	0	0	0	0	0
SAL_KA7687AA	FSIS1703467	Anatum	0	0	0	0	0	0	0	0	0	0	0
SAL_KA7684AA	FSIS1703465	Anatum	0	0	0	0	0	0	0	0	0	0	0
SAL_KA7686AA	FSIS1703464	Anatum	0	0	0	0	0	0	0	0	0	0	0
SAL_KA8305AA	PNUSAS018284	Anatum	0	0	0	0	0	0	0	0	0	0	0
SAL_KA8344AA	FSIS1703384	Anatum	0	0	0	0	0	0	0	0	0	0	0
SAL_KA8317AA	FSIS1703364	Anatum	0	0	0	0	0	0	0	0	0	0	0
SAL_KA8319AA	FSIS1703370	Anatum	0	0	0	0	0	0	0	0	0	0	0
SAL_KA9120AA	PNUSAS020693	Anatum	0	0	0	0	0	0	0	0	0	0	0
SAL_KA9155AA	TX-ACF1701817	Anatum	0	0	0	0	0	0	0	0	0	0	0
SAL_KA9795AA	PNUSAS020487	Anatum	0	0	0	0	0	0	0	0	0	0	0
SAL_LA0124AA	19-21-20170708	Anatum	0	0	0	0	0	0	0	0	0	0	0
SAL_KA9648AA	PNUSAS020799	Anatum	0	0	0	0	0	0	0	0	0	0	0
SAL_LA0575AA	17ER2396	Anatum	0	0	0	0	0	0	0	0	0	0	0
SAL_KA9379AA	PNUSAS020416	Anatum	0	0	0	0	0	0	0	0	0	0	0
SAL_KA9389AA	CFSAN067309	Anatum	0	0	0	0	0	0	0	0	0	0	0
SAL_KA9392AA	CFSAN067299	Anatum	0	0	0	0	0	0	0	0	0	0	0
SAL_KA9394AA	CFSAN067295	Anatum	0	0	0	0	0	0	0	0	0	0	0
SAL_KA9398AA	CFSAN067296	Anatum	0	0	0	0	0	0	0	0	0	0	0
SAL_KA9399AA	CFSAN067301	Anatum	0	0	0	0	0	0	0	0	0	0	0
SAL_KA9251AA	CFSAN067308	Anatum	0	0	0	0	0	0	0	0	0	0	0
SAL_KA9346AA	PNUSAS021235	Anatum	0	0	0	0	0	0	0	0	0	0	0
SAL_LA0038AA	FSIS21720007	Anatum	0	0	0	0	0	0	0	0	0	0	0
SAL_LA0037AA	FSIS21720008	Anatum	0	0	0	0	0	0	0	0	0	0	0
SAL_LA0027AA	FSIS21720023	Anatum	0	0	0	0	0	0	0	0	0	0	0
SAL_LA0019AA	FSIS1703564	Anatum	0	0	0	0	0	0	0	0	0	0	0
SAL_LA0006AA	FSIS1703546	Anatum	0	0	0	0	0	0	0	0	0	0	0
SAL_LA0015AA	FSIS1703569	Anatum	0	0	0	0	0	0	0	0	0	0	0
SAL_KA9998AA	FSIS1703658	Anatum	0	0	0	0	0	0	0	0	0	0	0
SAL_KA9340AA	CFSAN067300	Anatum	0	0	0	0	0	0	0	0	0	0	0
SAL_KA9916AA	FSIS1703647	Anatum	0	0	0	0	0	0	0	0	0	0	0
SAL_KA9853AA	FSIS1703575	Anatum	0	0	0	0	0	0	0	0	0	0	0
SAL_KA9829AA	FSIS1703731	Anatum	0	0	0	0	0	0	0	0	0	0	0
SAL_KA9832AA	FSIS1703864	Anatum	0	0	0	0	0	0	0	0	0	0	0
SAL_KA9838AA	FSIS1703553	Anatum	0	0	0	0	0	0	0	0	0	0	0
SAL_KA9865AA	PNUSAS020540	Anatum	0	0	0	0	0	0	0	0	0	0	0
SAL_KA9835AA	FSIS1703472	Anatum	0	0	0	0	0	0	0	0	0	0	0
SAL_KA9852AA	FSIS1703555	Anatum	0	0	0	0	0	0	0	0	0	0	0
SAL_LA0221AA	CFSAN067618	Anatum	0	0	0	0	0	0	0	0	0	0	0
SAL_LA0188AA	2015AM-0541	Anatum	0	0	0	0	0	0	0	0	0	0	0
SAL_LA0399AA	FSIS11703943	Anatum	0	0	0	0	0	0	0	0	0	0	0
SAL_LA0662AA	FSIS1703536	Anatum	0	0	0	0	0	0	0	0	0	0	0
SAL_LA0634AA	FSIS1703747	Anatum	0	0	0	0	0	0	0	0	0	0	0
SAL_LA0641AA	FSIS11703933	Anatum	0	0	0	0	0	0	0	0	0	0	0
SAL_LA0642AA	FSIS11703927	Anatum	0	0	0	0	0	0	0	0	0	0	0
SAL_LA0635AA	FSIS11703930	Anatum	0	0	0	0	0	0	0	0	0	0	0
SAL_LA0606AA	FSIS11703968	Anatum	0	0	0	0	0	0	0	0	0	0	0
SAL_LA0679AA	FSIS1703711	Anatum	0	0	0	0	0	0	0	0	0	0	0
SAL_LA0938AA	FDA00004408	Anatum	0	0	0	0	0	0	0	0	0	0	0
SAL_LA0942AA	FDA00012130	Anatum	0	0	0	0	0	0	0	0	0	0	0
SAL_LA0843AA	CFSAN067826	Anatum	0	0	0	0	0	0	0	0	0	0	0
SAL_LA0816AA	CFSAN067840	Anatum	0	0	0	0	0	0	0	0	0	0	0
SAL_LA0835AA	CFSAN067839	Anatum	0	0	0	0	0	0	0	0	0	0	0
SAL_LA0804AA	CFSAN067855	Anatum	0	0	0	0	0	0	0	0	0	0	0
SAL_LA0767AA	CFSAN067825	Anatum	0	0	0	0	0	0	0	0	0	0	0
SAL_LA0762AA	FDA00012127	Anatum	0	0	0	0	0	0	0	0	0	0	0
SAL_LA0769AA	CFSAN067827	Anatum	0	0	0	0	0	0	0	0	0	0	0
SAL_LA0768AA	CFSAN067828	Anatum	0	0	0	0	0	0	0	0	0	0	0
SAL_LA0765AA	FDA00012128	Anatum	0	0	0	0	0	0	0	0	0	0	0
SAL_LA0775AA	FDA00012129	Anatum	0	0	0	0	0	0	0	0	0	0	0
SAL_LA1129AA	CFSAN067882	Anatum	0	0	0	0	0	0	0	0	0	0	0
SAL_LA1141AA	CFSAN067274	Anatum	0	0	0	0	0	0	0	0	0	0	0
SAL_LA1139AA	CFSAN067273	Anatum	0	0	0	0	0	0	0	0	0	0	0
SAL_LA1399AA	CFSAN067885	Anatum	0	0	0	0	0	0	0	0	0	0	0
SAL_LA1328AA	CFSAN067886	Anatum	0	0	0	0	0	0	0	0	0	0	0
SAL_LA1318AA	CFSAN067824	Anatum	0	0	0	0	0	0	0	0	0	0	0
SAL_LA1851AA	FSIS1703543	Anatum	0	0	0	0	0	0	0	0	0	0	0
SAL_LA1806AA	FSIS11704095	Anatum	0	0	0	0	0	0	0	0	0	0	0
SAL_LA1834AA	FSIS1703734	Anatum	0	0	0	0	0	0	0	0	0	0	0
SAL_LA2712AA	H103540743	Anatum	0	0	0	0	0	0	0	0	0	0	0
SAL_LA2358AA	FSIS11704069	Anatum	0	0	0	0	0	0	0	0	0	0	0
SAL_LA3379AA	FSIS21720083	Anatum	0	0	0	0	0	0	0	0	0	0	0
SAL_LA3622AA	FSIS21720116	Anatum	0	0	0	0	0	0	0	0	0	0	0

SAL_LA2299AA	PNUSAS021426	Anatum	0	0	0	0	0	0	0	0	0	0	0
SAL_LA0672AA	FSIS1703537	Anatum	0	0	0	0	0	0	0	0	0	0	0
SAL_LA3917AA	PNUSAS021055	Anatum	0	0	0	0	0	0	0	0	0	0	0
SAL_LA4335AA	PNUSAS022429	Anatum	0	0	0	0	0	0	0	0	0	0	0
SAL_LA4288AA	PNUSAS022430	Anatum	0	0	0	0	0	0	0	0	0	0	0
SAL_LA4003AA	FSIS21720152	Anatum	0	0	0	0	0	0	0	0	0	0	0
SAL_LA4354AA	FSIS11704266	Anatum	0	0	0	0	0	0	0	0	0	0	0
SAL_LA4386AA	FSIS11704248	Anatum	0	0	0	0	0	0	0	0	0	0	0
SAL_LA4383AA	FSIS11704234	Anatum	0	0	0	0	0	0	0	0	0	0	0
SAL_LA4384AA	FSIS11704245	Anatum	0	0	0	0	0	0	0	0	0	0	0
SAL_LA4435AA	PNUSAS023029	Anatum	0	0	0	0	0	0	0	0	0	0	0
SAL_LA5053AA	FSIS11704387	Anatum	0	0	0	0	0	0	0	0	0	0	0
SAL_LA5054AA	FSIS11704393	Anatum	0	0	0	0	0	0	0	0	0	0	0
SAL_LA5060AA	FSIS11704392	Anatum	0	0	0	0	0	0	0	0	0	0	0
SAL_LA4372AA	FSIS11704230	Anatum	0	0	0	0	0	0	0	0	0	0	0
SAL_LA4916AA	PNUSAS022827	Anatum	0	0	0	0	0	0	0	0	0	0	0
SAL_LA5596AA	FDA00004763	Anatum	0	0	0	0	0	0	0	0	0	0	0
SAL_LA5649AA	FSIS21720223	Anatum	0	0	0	0	0	0	0	0	0	0	0
SAL_LA5646AA	FSIS21720222	Anatum	0	0	0	0	0	0	0	0	0	0	0
SAL_LA6786AA	PNUSAS023353	Anatum	0	0	0	0	0	0	0	0	0	0	0
SAL_LA6768AA	PNUSAS023531	Anatum	0	0	0	0	0	0	0	0	0	0	0
SAL_LA6695AA	CFSAN068321	Anatum	0	0	0	0	0	0	0	0	0	0	0
SAL_LA6737AA	CFSAN068066	Anatum	0	0	0	0	0	0	0	0	0	0	0
SAL_LA6795AA	PNUSAS022845	Anatum	0	0	0	0	0	0	0	0	0	0	0
SAL_LA6690AA	CFSAN068067	Anatum	0	0	0	0	0	0	0	0	0	0	0
SAL_LA6644AA	CFSAN068323	Anatum	0	0	0	0	0	0	0	0	0	0	0
SAL_LA6652AA	CFSAN068322	Anatum	0	0	0	0	0	0	0	0	0	0	0
SAL_LA6629AA	PNUSAS020294	Anatum	0	0	0	0	0	0	0	0	0	0	0
SAL_LA6433AA	PNUSAS022001	Anatum	0	0	0	0	0	0	0	0	0	0	0
SAL_LA6230AA	CFSAN069046	Anatum	0	0	0	0	0	0	0	0	0	0	0
SAL_LA6190AA	CFSAN069260	Anatum	0	0	0	0	0	0	0	0	0	0	0
SAL_LA6096AA	CFSAN069133	Anatum	0	0	0	0	0	0	0	0	0	0	0
SAL_LA6140AA	CFSAN069259	Anatum	0	0	0	0	0	0	0	0	0	0	0
SAL_LA6082AA	CFSAN069175	Anatum	0	0	0	0	0	0	0	0	0	0	0
SAL_LA7461AA	PNUSAS022916	Anatum	0	0	0	0	0	0	0	0	0	0	0
SAL_LA7421AA	PNUSAS022717	Anatum	0	0	0	0	0	0	0	0	0	0	0
SAL_LA7882AA	FSIS21720229	Anatum	0	0	0	0	0	0	0	0	0	0	0
SAL_LA8211AA	FSIS21720239	Anatum	0	0	0	0	0	0	0	0	0	0	0
SAL_LA8216AA	FSIS21720220	Anatum	0	0	0	0	0	0	0	0	0	0	0
SAL_LA9894AA	2015AM-1369	Anatum	0	0	0	0	0	0	0	0	0	0	0
SAL_LA9558AA	FSIS11704385	Anatum	0	0	0	0	0	0	0	0	0	0	0
SAL_LA9580AA	FSIS11704686	Anatum	0	0	0	0	0	0	0	0	0	0	0
SAL_LA9363AA	FSIS21720238	Anatum	0	0	0	0	0	0	0	0	0	0	0
SAL_LA9341AA	PNUSAS018242	Anatum	0	0	0	0	0	0	0	0	0	0	0
SAL_LA9284AA	PNUSAS022674	Anatum	0	0	0	0	0	0	0	0	0	0	0
SAL_LA9296AA	PNUSAS023721	Anatum	0	0	0	0	0	0	0	0	0	0	0
SAL_LA9239AA	PNUSAS024190	Anatum	0	0	0	0	0	0	0	0	0	0	0
SAL_LA9207AA	PNUSAS023962	Anatum	0	0	0	0	0	0	0	0	0	0	0
SAL_MA0244AA	PNUSAS023613	Anatum	0	0	0	0	0	0	0	0	0	0	0
SAL_MA0164AA	PNUSAS022432	Anatum	0	0	0	0	0	0	0	0	0	0	0
SAL_MA0165AA	PNUSAS022434	Anatum	0	0	0	0	0	0	0	0	0	0	0
SAL_MA0157AA	PNUSAS022433	Anatum	0	0	0	0	0	0	0	0	0	0	0
SAL_MA0511AA	PNUSAS025150	Anatum	0	0	0	0	0	0	0	0	0	0	0
SAL_MA0363AA	FSIS11704761	Anatum	0	0	0	0	0	0	0	0	0	0	0
SAL_MA0390AA	PNUSAS024178	Anatum	0	0	0	0	0	0	0	0	0	0	0
SAL_MA1025AA	PNUSAS025897	Anatum	0	0	0	0	0	0	0	0	0	0	0
SAL_MA0889AA	FDA00012174	Anatum	0	0	0	0	0	0	0	0	0	0	0
SAL_MA0881AA	FDA00012172	Anatum	0	0	0	0	0	0	0	0	0	0	0
SAL_MA1235AA	CFSAN068468	Anatum	0	0	0	0	0	0	0	0	0	0	0
SAL_MA1462AA	PNUSAS023654	Anatum	0	0	0	0	0	0	0	0	0	0	0
SAL_MA1477AA	CFSAN068464	Anatum	0	0	0	0	0	0	0	0	0	0	0
SAL_MA1478AA	CFSAN068463	Anatum	0	0	0	0	0	0	0	0	0	0	0
SAL_MA1480AA	CFSAN068461	Anatum	0	0	0	0	0	0	0	0	0	0	0
SAL_MA1484AA	CFSAN068459	Anatum	0	0	0	0	0	0	0	0	0	0	0
SAL_MA1486AA	CFSAN068460	Anatum	0	0	0	0	0	0	0	0	0	0	0
SAL_MA1479AA	CFSAN068462	Anatum	0	0	0	0	0	0	0	0	0	0	0
SAL_MA1374AA	PNUSAS025690	Anatum	0	0	0	0	0	0	0	0	0	0	0
SAL_MA1362AA	PNUSAS025691	Anatum	0	0	0	0	0	0	0	0	0	0	0
SAL_MA1371AA	PNUSAS025686	Anatum	0	0	0	0	0	0	0	0	0	0	0
SAL_MA1303AA	CFSAN068469	Anatum	0	0	0	0	0	0	0	0	0	0	0
SAL_MA1302AA	CFSAN068470	Anatum	0	0	0	0	0	0	0	0	0	0	0
SAL_MA1254AA	CFSAN068458	Anatum	0	0	0	0	0	0	0	0	0	0	0
SAL_MA1524AA	FSIS11704776	Anatum	0	0	0	0	0	0	0	0	0	0	0

SAL_MA1526AA	FSIS11704801	Anatum	0	0	0	0	0	0	0	0	0	0	0
SAL_MA1642AA	PNUSAS025357	Anatum	0	0	0	0	0	0	0	0	0	0	0
SAL_MA1719AA	PNUSAS025080	Anatum	0	0	0	0	0	0	0	0	0	0	0
SAL_MA2311AA	FSIS21720371	Anatum	0	0	0	0	0	0	0	0	0	0	0
SAL_MA2313AA	FSIS21720372	Anatum	0	0	0	0	0	0	0	0	0	0	0
SAL_MA2247AA	FSIS21720400	Anatum	0	0	0	0	0	0	0	0	0	0	0
SAL_MA2272AA	FSIS21720338	Anatum	0	0	0	0	0	0	0	0	0	0	0
SAL_MA2281AA	FSIS21720390	Anatum	0	0	0	0	0	0	0	0	0	0	0
SAL_MA2289AA	FSIS21720361	Anatum	0	0	0	0	0	0	0	0	0	0	0
SAL_MA2252AA	FSIS21720360	Anatum	0	0	0	0	0	0	0	0	0	0	0
SAL_MA2591AA	FSIS21720438	Anatum	0	0	0	0	0	0	0	0	0	0	0
SAL_MA3244AA	PNUSAS024705	Anatum	0	0	0	0	0	0	0	0	0	0	0
SAL_MA3151AA	FSIS11704236	Anatum	0	0	0	0	0	0	0	0	0	0	0
SAL_MA3155AA	FSIS11704898	Anatum	0	0	0	0	0	0	0	0	0	0	0
SAL_MA3588AA	FDA00004779	Anatum	0	0	0	0	0	0	0	0	0	0	0
SAL_MA3591AA	PNUSAS026677	Anatum	0	0	0	0	0	0	0	0	0	0	0
SAL_MA3892AA	FSIS11705099	Anatum	0	0	0	0	0	0	0	0	0	0	0
SAL_MA3863AA	FSIS11705142	Anatum	0	0	0	0	0	0	0	0	0	0	0
SAL_MA3855AA	FSIS11705128	Anatum	0	0	0	0	0	0	0	0	0	0	0
SAL_MA4739AA	PNUSAS027059	Anatum	0	0	0	0	0	0	0	0	0	0	0
SAL_MA4682AA	PNUSAS027060	Anatum	0	0	0	0	0	0	0	0	0	0	0
SAL_MA4650AA	PNUSAS026437	Anatum	0	0	0	0	0	0	0	0	0	0	0
SAL_MA4459AA	FDA00000273	Anatum	0	0	0	0	0	0	0	0	0	0	0
SAL_MA4869AA	FSIS11704665	Anatum	0	0	0	0	0	0	0	0	0	0	0
SAL_MA4793AA	FSIS11704602	Anatum	0	0	0	0	0	0	0	0	0	0	0
SAL_MA4804AA	FSIS11704584	Anatum	0	0	0	0	0	0	0	0	0	0	0
SAL_MA4783AA	FSIS11704654	Anatum	0	0	0	0	0	0	0	0	0	0	0
SAL_MA4784AA	FSIS11704580	Anatum	0	0	0	0	0	0	0	0	0	0	0
SAL_MA4773AA	FSIS11704579	Anatum	0	0	0	0	0	0	0	0	0	0	0
SAL_MA4778AA	FSIS11704592	Anatum	0	0	0	0	0	0	0	0	0	0	0
SAL_MA5180AA	PNUSAS026979	Anatum	0	0	0	0	0	0	0	0	0	0	0
SAL_MA5202AA	FSIS11704907	Anatum	0	0	0	0	0	0	0	0	0	0	0
SAL_MA5111AA	PNUSAS025804	Anatum	0	0	0	0	0	0	0	0	0	0	0
SAL_MA5195AA	FSIS11704916	Anatum	0	0	0	0	0	0	0	0	0	0	0
SAL_MA5209AA	FSIS11705089	Anatum	0	0	0	0	0	0	0	0	0	0	0
SAL_MA5083AA	PNUSAS025814	Anatum	0	0	0	0	0	0	0	0	0	0	0
SAL_MA5043AA	FSIS1702785	Anatum	0	0	0	0	0	0	0	0	0	0	0
SAL_MA5020AA	FSIS1702638	Anatum	0	0	0	0	0	0	0	0	0	0	0
SAL_MA5061AA	FSIS1702783	Anatum	0	0	0	0	0	0	0	0	0	0	0
SAL_MA5282AA	FDA00012290	Anatum	0	0	0	0	0	0	0	0	0	0	0
SAL_MA5309AA	FSIS11705151	Anatum	0	0	0	0	0	0	0	0	0	0	0
SAL_MA5374AA	OH-17-19031	Anatum	0	0	0	0	0	0	0	0	0	0	0
SAL_BA5085AA	FDA00002327	Anatum	0	0	0	0	0	0	0	0	0	0	0
SAL_BA8743AA	NY_BAC0800002252	Anatum	0	0	0	0	0	0	0	0	0	0	0
SAL_EA1488AA	FSIS1500658	Anatum	0	0	0	0	0	0	0	0	0	0	0
SAL_EA1478AA	FSIS1500657	Anatum	0	0	0	0	0	0	0	0	0	0	0
SAL_DA0057AA	POR1-0058	Anatum	0	0	0	0	0	0	0	0	0	0	0
SAL_BA6596AA	S-1806	Anatum	0	0	0	0	0	0	0	0	0	0	0
SAL_DA1941AA	AZ-TG74568	Anatum	0	0	0	0	0	0	0	0	0	0	0
SAL_EA1372AA	FL_MJ422	Anatum	0	0	0	0	0	0	0	0	0	0	0
SAL_DA9426AA	FNW19H17	Anatum	0	0	0	0	0	0	0	0	0	0	0
SAL_BA6522AA	2015K-0192	Anatum	0	0	0	0	0	0	0	0	0	0	0
SAL_CA2683AA	89992	Anatum	0	0	0	0	0	0	0	0	0	0	0
SAL_CA2665AA	25203	Anatum	0	0	0	0	0	0	0	0	0	0	0
SAL_DA8066AA	NY-FSL-R6-0929	Anatum	0	0	0	0	0	0	0	0	0	0	0
SAL_DA2493AA	FDA00003918	Anatum	0	0	0	0	0	0	0	0	0	0	0
SAL_DA3981AA	IEH-NGS-SAL-01153	Anatum	0	0	0	0	0	0	0	0	0	0	0
SAL_EA0640AA	FSW0040	Anatum	0	0	0	0	0	0	0	0	0	0	0
SAL_GA9209AA	CFSAN031373	Anatum	0	0	0	0	0	0	0	0	0	0	0
SAL_CA4179AA	36366	Anatum	0	0	0	0	0	0	0	0	0	0	0
SAL_BA3938AA	CFSAN004011	Anatum	0	0	0	0	0	0	0	0	0	0	0
SAL_HA8943AA	JSDA-ARS-USMARC-1781	Anatum	0	0	0	0	0	0	0	0	0	0	0
SAL_EA9707AA	GT-01	Anatum	0	0	0	0	0	0	0	0	0	0	0
SAL_BA8067AA	ATCC BAA-1592	Anatum	0	0	0	0	0	0	0	0	0	0	0
SAL_HA6270AA	PNUSAS005581	Anatum	0	0	0	0	0	0	0	0	0	0	0
SAL_HA8292AA	FSIS1609243	Anatum	0	0	0	0	0	0	0	0	0	0	0
SAL_HA4661AA	FDA00010934	Anatum	0	0	0	0	0	0	0	0	0	0	0
SAL_GA5320AA	CFSAN042889	Anatum	0	0	0	0	0	0	0	0	0	0	0
SAL_GA2340AA	BCW_2639	Anatum	0	0	0	0	0	0	0	0	0	0	0
SAL_HA8949AA	JSDA-ARS-USMARC-1727	Anatum	0	0	0	0	0	0	0	0	0	0	0
SAL_GA0322AA	71_H_023-sc-2280669	Anatum	0	0	0	0	0	0	0	0	0	0	0
SAL_HA9138AA	CFSAN058097	Anatum	0	0	0	0	0	0	0	0	0	0	0
SAL_GA5168AA	V-CLASP-D-52	Anatum	0	0	0	0	0	0	0	0	0	0	0

SAL_GA0358AA	VNS30014-sc-2280596	Anatum	0	0	0	0	0	0	0	0	0	0	0
SAL_GA2842AA	CRJJGF_00069	Anatum	0	0	0	0	0	0	0	0	0	0	0
SAL_FA8458AA	FSIS1606826	Anatum	0	0	0	0	0	0	0	0	0	0	0
SAL_GA2410AA	BCW_2008	Anatum	0	0	0	0	0	0	0	0	0	0	0
SAL_BA7593AA	1382201	Anatum	0	0	0	0	0	0	0	0	0	0	0
SAL_GA7375AA	FDA00003762	Anatum	0	0	0	0	0	0	0	0	0	0	0
SAL_GA6320AA	FSIS1607206	Anatum	0	0	0	0	0	0	0	0	0	0	0
SAL_GA6012AA	FLUFL-1986	Anatum	0	0	0	0	0	0	0	0	0	0	0
SAL_FA7466AA	CFSAN032580	Anatum	0	0	0	0	0	0	0	0	0	0	0
SAL_FA4942AA	CFSAN045257	Anatum	0	0	0	0	0	0	0	0	0	0	0
SAL_FA4751AA	FSIS1606125	Anatum	0	0	0	0	0	0	0	0	0	0	0
SAL_FA2747AA	CVM N57717F	Anatum	0	0	0	0	0	0	0	0	0	0	0
SAL_FA3255AA	FSIS1606021	Anatum	0	0	0	0	0	0	0	0	0	0	0
SAL_FA2515AA	FDA00007881	Anatum	0	0	0	0	0	0	0	0	0	0	0
SAL_FA2167AA	FSIS1605972	Anatum	0	0	0	0	0	0	0	0	0	0	0
SAL_FA1442AA	CFSAN042428	Anatum	0	0	0	0	0	0	0	0	0	0	0
SAL_EA6486AA	FSIS1504202	Anatum	0	0	0	0	0	0	0	0	0	0	0
SAL_IA0152AA	ADRDL-822	Anatum	0	0	0	0	0	0	0	0	0	0	0
SAL_IA2071AA	FSIS1609513	Anatum	0	0	0	0	0	0	0	0	0	0	0
SAL_IA4952AA	CFSAN059273	Anatum	0	0	0	0	0	0	0	0	0	0	0
SAL_IA6590AA	FSIS1710362	Anatum	0	0	0	0	0	0	0	0	0	0	0
SAL_IA7172AA	ADRDL-940	Anatum	0	0	0	0	0	0	0	0	0	0	0
SAL_IA7680AA	FSIS1710585	Anatum	0	0	0	0	0	0	0	0	0	0	0
SAL_IA7551AA	FSIS1710538	Anatum	0	0	0	0	0	0	0	0	0	0	0
SAL_IA8227AA	PNUSAS009202	Anatum	0	0	0	0	0	0	0	0	0	0	0
SAL_IA9163AA	PNUSAS010087	Anatum	0	0	0	0	0	0	0	0	0	0	0
SAL_IA9701AA	PNUSAS009585	Anatum	0	0	0	0	0	0	0	0	0	0	0
SAL_JA1562AA	PNUSAS010879	Anatum	0	0	0	0	0	0	0	0	0	0	0
SAL_JA2057AA	FSIS1700036	Anatum	0	0	0	0	0	0	0	0	0	0	0
SAL_JA7488AA	FSIS1700574	Anatum	0	0	0	0	0	0	0	0	0	0	0
SAL_KA3318AA	PNUSAS015584	Anatum	0	0	0	0	0	0	0	0	0	0	0
SAL_KA9820AA	FSIS1703733	Anatum	0	0	0	0	0	0	0	0	0	0	0
SAL_LA0751AA	PNUSAS020558	Anatum	0	0	0	0	0	0	0	0	0	0	0
SAL_KA9997AA	FSIS1703551	Anatum	0	0	0	0	0	0	0	0	0	0	0
SAL_LA1313AA	CFSAN067823	Anatum	0	0	0	0	0	0	0	0	0	0	0
SAL_LA1342AA	CFSAN067869	Anatum	0	0	0	0	0	0	0	0	0	0	0
SAL_LA2366AA	FSIS11704065	Anatum	0	0	0	0	0	0	0	0	0	0	0
SAL_LA3829AA	PNUSAS022431	Anatum	0	0	0	0	0	0	0	0	0	0	0
SAL_LA6151AA	CFSAN069195	Anatum	0	0	0	0	0	0	0	0	0	0	0
SAL_MA3144AA	FSIS11704401	Anatum	0	0	0	0	0	0	0	0	0	0	0
SAL_MA5674AA	ERS2000985	Anatum	0	0	0	0	0	0	0	0	0	0	0
SAL_MA5506AA	CFSAN068038	Anatum	0	0	0	0	0	0	0	0	0	0	0
SAL_MA5655AA	FSIS21720603	Anatum	0	0	0	0	0	0	0	0	0	0	0
SAL_MA5654AA	FSIS21720604	Anatum	0	0	0	0	0	0	0	0	0	0	0
SAL_MA6095AA	FSIS11704873	Anatum	0	0	0	0	0	0	0	0	0	0	0
SAL_MA6460AA	FSIS11704888	Anatum	0	0	0	0	0	0	0	0	0	0	0
SAL_MA6443AA	FSIS11704868	Anatum	0	0	0	0	0	0	0	0	0	0	0
SAL_MA6453AA	FSIS11704891	Anatum	0	0	0	0	0	0	0	0	0	0	0
SAL_MA6696AA	FSIS1701758	Anatum	0	0	0	0	0	0	0	0	0	0	0
SAL_MA7370AA	FSIS21720641	Anatum	0	0	0	0	0	0	0	0	0	0	0
SAL_MA6964AA	ADRDL-1298	Anatum	0	0	0	0	0	0	0	0	0	0	0
SAL_MA6920AA	CFSAN071442	Anatum	0	0	0	0	0	0	0	0	0	0	0
SAL_MA6914AA	ADRDL-1597	Anatum	0	0	0	0	0	0	0	0	0	0	0
SAL_MA6886AA	ADRDL-1290	Anatum	0	0	0	0	0	0	0	0	0	0	0
SAL_MA6894AA	ADRDL-1299	Anatum	0	0	0	0	0	0	0	0	0	0	0
SAL_MA6916AA	CFSAN071441	Anatum	0	0	0	0	0	0	0	0	0	0	0
SAL_MA6896AA	FDA00012296	Anatum	0	0	0	0	0	0	0	0	0	0	0
SAL_MA6870AA	ADRDL-1124	Anatum	0	0	0	0	0	0	0	0	0	0	0
SAL_MA6822AA	ADRDL-1116	Anatum	0	0	0	0	0	0	0	0	0	0	0
SAL_MA6797AA	FDA00004936	Anatum	0	0	0	0	0	0	0	0	0	0	0
SAL_MA6816AA	ADRDL-1279	Anatum	0	0	0	0	0	0	0	0	0	0	0
SAL_MA6817AA	ADRDL-1121	Anatum	0	0	0	0	0	0	0	0	0	0	0
SAL_MA7665AA	CFSAN071179	Anatum	0	0	0	0	0	0	0	0	0	0	0
SAL_MA7604AA	PNUSAS027977	Anatum	0	0	0	0	0	0	0	0	0	0	0
SAL_MA7521AA	PNUSAS028238	Anatum	0	0	0	0	0	0	0	0	0	0	0
SAL_MA7490AA	FSIS11705575	Anatum	0	0	0	0	0	0	0	0	0	0	0
SAL_MA7496AA	FSIS11705464	Anatum	0	0	0	0	0	0	0	0	0	0	0
SAL_MA7455AA	NSUOC-0358-S5-2H	Anatum	0	0	0	0	0	0	0	0	0	0	0
SAL_MA7487AA	FSIS11705867	Anatum	0	0	0	0	0	0	0	0	0	0	0
SAL_MA7501AA	FSIS11705465	Anatum	0	0	0	0	0	0	0	0	0	0	0
SAL_MA7837AA	CFSAN071807	Anatum	0	0	0	0	0	0	0	0	0	0	0
SAL_MA8064AA	ARIMSCHES656-17	Anatum	0	0	0	0	0	0	0	0	0	0	0
SAL_MA8079AA	104263	Anatum	0	0	0	0	0	0	0	0	0	0	0

SAL_MA7999AA	ARIMSCHES655-17	Anatum	0	0	0	0	0	0	0	0	0	0	0
SAL_MA8273AA	FSIS21720668	Anatum	0	0	0	0	0	0	0	0	0	0	0
SAL_MA8192AA	PNUSAS028788	Anatum	0	0	0	0	0	0	0	0	0	0	0
SAL_MA8228AA	PNUSAS028787	Anatum	0	0	0	0	0	0	0	0	0	0	0
SAL_MA8974AA	PNUSAS029095	Anatum	0	0	0	0	0	0	0	0	0	0	0
SAL_MA8980AA	PNUSAS028978	Anatum	0	0	0	0	0	0	0	0	0	0	0
SAL_MA8899AA	FSIS11706004	Anatum	0	0	0	0	0	0	0	0	0	0	0
SAL_MA8907AA	FSIS11705976	Anatum	0	0	0	0	0	0	0	0	0	0	0
SAL_MA8849AA	FSIS11705526	Anatum	0	0	0	0	0	0	0	0	0	0	0
SAL_MA8839AA	FSIS11705507	Anatum	0	0	0	0	0	0	0	0	0	0	0
SAL_MA8840AA	FSIS11705347	Anatum	0	0	0	0	0	0	0	0	0	0	0
SAL_MA8837AA	FSIS11705527	Anatum	0	0	0	0	0	0	0	0	0	0	0
SAL_MA8881AA	FSIS11706030	Anatum	0	0	0	0	0	0	0	0	0	0	0
SAL_MA8879AA	FSIS11705998	Anatum	0	0	0	0	0	0	0	0	0	0	0
SAL_MA8819AA	FSIS11705656	Anatum	0	0	0	0	0	0	0	0	0	0	0
SAL_MA8820AA	FSIS11705659	Anatum	0	0	0	0	0	0	0	0	0	0	0
SAL_MA8827AA	FSIS11705647	Anatum	0	0	0	0	0	0	0	0	0	0	0
SAL_MA8829AA	FSIS11705640	Anatum	0	0	0	0	0	0	0	0	0	0	0
SAL_MA8780AA	FSIS11705048	Anatum	0	0	0	0	0	0	0	0	0	0	0
SAL_MA8786AA	FSIS11705054	Anatum	0	0	0	0	0	0	0	0	0	0	0
SAL_MA8858AA	FSIS11705351	Anatum	0	0	0	0	0	0	0	0	0	0	0
SAL_MA8771AA	FSIS11705985	Anatum	0	0	0	0	0	0	0	0	0	0	0
SAL_MA9055AA	FSIS11705077	Anatum	0	0	0	0	0	0	0	0	0	0	0
SAL_MA9056AA	FSIS11705331	Anatum	0	0	0	0	0	0	0	0	0	0	0
SAL_MA9021AA	FSIS11705860	Anatum	0	0	0	0	0	0	0	0	0	0	0
SAL_MA6458AA	FSIS11704889	Anatum	0	0	0	0	0	0	0	0	0	0	0
SAL_NA0409AA	FSIS11705506	Anatum	0	0	0	0	0	0	0	0	0	0	0
SAL_NA0407AA	FSIS11705837	Anatum	0	0	0	0	0	0	0	0	0	0	0
SAL_NA0408AA	FSIS11705836	Anatum	0	0	0	0	0	0	0	0	0	0	0
SAL_NA0333AA	PNUSAS029149	Anatum	0	0	0	0	0	0	0	0	0	0	0
SAL_NA0350AA	FSIS21720689	Anatum	0	0	0	0	0	0	0	0	0	0	0
SAL_NA0243AA	PNUSAS029204	Anatum	0	0	0	0	0	0	0	0	0	0	0
SAL_NA0254AA	PNUSAS029185	Anatum	0	0	0	0	0	0	0	0	0	0	0
SAL_NA0257AA	PNUSAS029211	Anatum	0	0	0	0	0	0	0	0	0	0	0
SAL_MA9966AA	CVM N16S226	Anatum	0	0	0	0	0	0	0	0	0	0	0
SAL_MA9970AA	CVM N16S228	Anatum	0	0	0	0	0	0	0	0	0	0	0
SAL_MA9874AA	CVM N16S405	Anatum	0	0	0	0	0	0	0	0	0	0	0
SAL_NA0809AA	FSIS11706124	Anatum	0	0	0	0	0	0	0	0	0	0	0
SAL_NA0851AA	PNUSAS028786	Anatum	0	0	0	0	0	0	0	0	0	0	0
SAL_NA0860AA	PNUSAS028785	Anatum	0	0	0	0	0	0	0	0	0	0	0
SAL_NA0462AA	FSIS11705847	Anatum	0	0	0	0	0	0	0	0	0	0	0
SAL_NA0466AA	FSIS11705842	Anatum	0	0	0	0	0	0	0	0	0	0	0
SAL_NA0468AA	FSIS11705849	Anatum	0	0	0	0	0	0	0	0	0	0	0
SAL_NA0567AA	FSIS11706158	Anatum	0	0	0	0	0	0	0	0	0	0	0
SAL_NA0807AA	FSIS11706122	Anatum	0	0	0	0	0	0	0	0	0	0	0
SAL_NA0936AA	CFSAN071809	Anatum	0	0	0	0	0	0	0	0	0	0	0
SAL_NA1112AA	PNUSAS027407	Anatum	0	0	0	0	0	0	0	0	0	0	0
SAL_NA1356AA	FSIS21720729	Anatum	0	0	0	0	0	0	0	0	0	0	0
SAL_OA0189AA	HJ29-VA-WGS-17107	Anatum	0	0	0	0	0	0	0	0	0	0	0
SAL_NA1247AA	VA-WGS-17107	Anatum	0	0	0	0	0	0	0	0	0	0	0
SAL_NA1719AA	FSIS11706197	Anatum	0	0	0	0	0	0	0	0	0	0	0
SAL_NA1700AA	PNUSAS030057	Anatum	0	0	0	0	0	0	0	0	0	0	0
SAL_NA1652AA	FSIS11706316	Anatum	0	0	0	0	0	0	0	0	0	0	0
SAL_NA1656AA	FSIS11706318	Anatum	0	0	0	0	0	0	0	0	0	0	0
SAL_NA1664AA	FSIS11705644	Anatum	0	0	0	0	0	0	0	0	0	0	0
SAL_NA1673AA	PNUSAS029450	Anatum	0	0	0	0	0	0	0	0	0	0	0
SAL_NA1632AA	FSIS11705502	Anatum	0	0	0	0	0	0	0	0	0	0	0
SAL_NA1629AA	FSIS11706323	Anatum	0	0	0	0	0	0	0	0	0	0	0
SAL_NA1625AA	FSIS11706327	Anatum	0	0	0	0	0	0	0	0	0	0	0
SAL_NA1636AA	FSIS11705505	Anatum	0	0	0	0	0	0	0	0	0	0	0
SAL_NA1615AA	FSIS11706334	Anatum	0	0	0	0	0	0	0	0	0	0	0
SAL_NA1375AA	FSIS11706497	Anatum	0	0	0	0	0	0	0	0	0	0	0
SAL_NA2006AA	FSIS11706272	Anatum	0	0	0	0	0	0	0	0	0	0	0
SAL_NA1995AA	FSIS11706003	Anatum	0	0	0	0	0	0	0	0	0	0	0
SAL_NA1876AA	PNUSAS029733	Anatum	0	0	0	0	0	0	0	0	0	0	0
SAL_NA2104AA	CFSAN073512	Anatum	0	0	0	0	0	0	0	0	0	0	0
SAL_NA2103AA	CFSAN073513	Anatum	0	0	0	0	0	0	0	0	0	0	0
SAL_NA2483AA	PNUSAS029924	Anatum	0	0	0	0	0	0	0	0	0	0	0
SAL_NA2395AA	PNUSAS030029	Anatum	0	0	0	0	0	0	0	0	0	0	0
SAL_NA2362AA	CFSAN072637	Anatum	0	0	0	0	0	0	0	0	0	0	0
SAL_NA2342AA	FSIS21720777	Anatum	0	0	0	0	0	0	0	0	0	0	0
SAL_NA2613AA	CFSAN068466	Anatum	0	0	0	0	0	0	0	0	0	0	0
SAL_NA2612AA	CFSAN068467	Anatum	0	0	0	0	0	0	0	0	0	0	0

SAL_NA2614AA	CFSAN068465	Anatum	0	0	0	0	0	0	0	0	0	0	0
SAL_NA2548AA	PNUSAS025408	Anatum	0	0	0	0	0	0	0	0	0	0	0
SAL_NA2629AA	FSIS21720800	Anatum	0	0	0	0	0	0	0	0	0	0	0
SAL_NA2814AA	CFSAN031887	Anatum	0	0	0	0	0	0	0	0	0	0	0
SAL_NA2752AA	CFSAN031882	Anatum	0	0	0	0	0	0	0	0	0	0	0
SAL_NA2684AA	FSIS11706645	Anatum	0	0	0	0	0	0	0	0	0	0	0
SAL_NA2686AA	FSIS11706646	Anatum	0	0	0	0	0	0	0	0	0	0	0
SAL_NA2716AA	CFSAN031933	Anatum	0	0	0	0	0	0	0	0	0	0	0
SAL_NA2668AA	PNUSAS027066	Anatum	0	0	0	0	0	0	0	0	0	0	0
SAL_NA3325AA	FSIS21720834	Anatum	0	0	0	0	0	0	0	0	0	0	0
SAL_NA3326AA	FSIS21720833	Anatum	0	0	0	0	0	0	0	0	0	0	0
SAL_NA3308AA	FSIS21720831	Anatum	0	0	0	0	0	0	0	0	0	0	0
SAL_NA3243AA	CFSAN073578	Anatum	0	0	0	0	0	0	0	0	0	0	0
SAL_NA3398AA	FSIS11706474	Anatum	0	0	0	0	0	0	0	0	0	0	0
SAL_NA3400AA	FSIS11705670	Anatum	0	0	0	0	0	0	0	0	0	0	0
SAL_NA3396AA	FSIS11705666	Anatum	0	0	0	0	0	0	0	0	0	0	0
SAL_NA3696AA	PNUSAS030819	Anatum	0	0	0	0	0	0	0	0	0	0	0
SAL_NA4116AA	PNUSAS031104	Anatum	0	0	0	0	0	0	0	0	0	0	0
SAL_NA4148AA	FSIS11706619	Anatum	0	0	0	0	0	0	0	0	0	0	0
SAL_NA4103AA	PNUSAS031111	Anatum	0	0	0	0	0	0	0	0	0	0	0
SAL_NA4013AA	2017K-1219	Anatum	0	0	0	0	0	0	0	0	0	0	0
SAL_NA4015AA	2017K-1220	Anatum	0	0	0	0	0	0	0	0	0	0	0
SAL_NA4020AA	2017K-1218	Anatum	0	0	0	0	0	0	0	0	0	0	0
SAL_NA4050AA	FSIS11706630	Anatum	0	0	0	0	0	0	0	0	0	0	0
SAL_NA4011AA	2017K-1221	Anatum	0	0	0	0	0	0	0	0	0	0	0
SAL_NA4336AA	PNUSAS031333	Anatum	0	0	0	0	0	0	0	0	0	0	0
SAL_NA4347AA	PNUSAS031257	Anatum	0	0	0	0	0	0	0	0	0	0	0
SAL_NA4321AA	PNUSAS031334	Anatum	0	0	0	0	0	0	0	0	0	0	0
SAL_NA4546AA	FSIS11706797	Anatum	0	0	0	0	0	0	0	0	0	0	0
SAL_NA4522AA	FSIS11706493	Anatum	0	0	0	0	0	0	0	0	0	0	0
SAL_NA4515AA	CFSAN074266	Anatum	0	0	0	0	0	0	0	0	0	0	0
SAL_NA4532AA	FSIS11706491	Anatum	0	0	0	0	0	0	0	0	0	0	0
SAL_NA4528AA	FSIS11706485	Anatum	0	0	0	0	0	0	0	0	0	0	0
SAL_NA4536AA	FSIS11706946	Anatum	0	0	0	0	0	0	0	0	0	0	0
SAL_NA4534AA	FSIS11706943	Anatum	0	0	0	0	0	0	0	0	0	0	0
SAL_NA4666AA	PNUSAS031588	Anatum	0	0	0	0	0	0	0	0	0	0	0
SAL_NA4649AA	FSIS11706701	Anatum	0	0	0	0	0	0	0	0	0	0	0
SAL_NA4623AA	FSIS11706976	Anatum	0	0	0	0	0	0	0	0	0	0	0
SAL_NA4897AA	PNUSAS031356	Anatum	0	0	0	0	0	0	0	0	0	0	0
SAL_NA4795AA	PNUSAS031534	Anatum	0	0	0	0	0	0	0	0	0	0	0
SAL_NA4922AA	PNUSAS031669	Anatum	0	0	0	0	0	0	0	0	0	0	0
SAL_NA4913AA	PNUSAS031676	Anatum	0	0	0	0	0	0	0	0	0	0	0
SAL_NA5185AA	CFSAN074275	Anatum	0	0	0	0	0	0	0	0	0	0	0
SAL_NA5428AA	2015AM-0897	Anatum	0	0	0	0	0	0	0	0	0	0	0
SAL_NA5395AA	PNUSAS030158	Anatum	0	0	0	0	0	0	0	0	0	0	0
SAL_NA5339AA	2015AM-0593	Anatum	0	0	0	0	0	0	0	0	0	0	0
SAL_NA5986AA	PNUSAS031677	Anatum	0	0	0	0	0	0	0	0	0	0	0
SAL_NA5907AA	PNUSAS032041	Anatum	0	0	0	0	0	0	0	0	0	0	0
SAL_NA5840AA	CFSAN074235	Anatum	0	0	0	0	0	0	0	0	0	0	0
SAL_NA5754AA	PNUSAS031678	Anatum	0	0	0	0	0	0	0	0	0	0	0
SAL_NA6521AA	2015AM-2969	Anatum	0	0	0	0	0	0	0	0	0	0	0
SAL_NA6427AA	FSIS11706289	Anatum	0	0	0	0	0	0	0	0	0	0	0
SAL_NA6395AA	FSIS11706971	Anatum	0	0	0	0	0	0	0	0	0	0	0
SAL_NA6410AA	FSIS11706607	Anatum	0	0	0	0	0	0	0	0	0	0	0
SAL_NA6390AA	FSIS11706961	Anatum	0	0	0	0	0	0	0	0	0	0	0
SAL_NA6412AA	FSIS11706609	Anatum	0	0	0	0	0	0	0	0	0	0	0
SAL_NA6345AA	FSIS31800046	Anatum	0	0	0	0	0	0	0	0	0	0	0
SAL_NA6337AA	FSIS21820937	Anatum	0	0	0	0	0	0	0	0	0	0	0
SAL_NA6354AA	FSIS21820927	Anatum	0	0	0	0	0	0	0	0	0	0	0
SAL_NA6280AA	FSIS21820976	Anatum	0	0	0	0	0	0	0	0	0	0	0
SAL_NA6319AA	FSIS11706985	Anatum	0	0	0	0	0	0	0	0	0	0	0
SAL_NA6213AA	FSIS11807159	Anatum	0	0	0	0	0	0	0	0	0	0	0
SAL_NA7048AA	PNUSAS028949	Anatum	0	0	0	0	0	0	0	0	0	0	0
SAL_NA6787AA	PNUSAS031951	Anatum	0	0	0	0	0	0	0	0	0	0	0
SAL_NA7084AA	2018K-0007	Anatum	0	0	0	0	0	0	0	0	0	0	0
SAL_NA7083AA	2018K-0006	Anatum	0	0	0	0	0	0	0	0	0	0	0
SAL_NA7212AA	FSIS11807134	Anatum	0	0	0	0	0	0	0	0	0	0	0
SAL_NA7227AA	FSIS11807113	Anatum	0	0	0	0	0	0	0	0	0	0	0
SAL_FA7268AA	CFSAN050752	Anatum	0	0	0	0	0	0	0	0	0	0	0
SAL_NA7585AA	PNUSAS032075	Anatum	0	0	0	0	0	0	0	0	0	0	0
SAL_NA7589AA	PNUSAS032211	Anatum	0	0	0	0	0	0	0	0	0	0	0
SAL_NA7514AA	FSIS31800067	Anatum	0	0	0	0	0	0	0	0	0	0	0
SAL_NA7330AA	PNUSAS032749	Anatum	0	0	0	0	0	0	0	0	0	0	0

SAL_NA8225AA	PNUSAS032933	Anatum	0	0	0	0	0	0	0	0	0	0	0
SAL_NA8192AA	FSIS11807287	Anatum	0	0	0	0	0	0	0	0	0	0	0
SAL_NA8264AA	FSIS11706476	Anatum	0	0	0	0	0	0	0	0	0	0	0
SAL_NA8275AA	FSIS11706482	Anatum	0	0	0	0	0	0	0	0	0	0	0
SAL_NA8301AA	4-3-8100057	Anatum	0	0	0	0	0	0	0	0	0	0	0
SAL_NA8500AA	PNUSAS032848	Anatum	0	0	0	0	0	0	0	0	0	0	0
SAL_NA8503AA	PNUSAS032847	Anatum	0	0	0	0	0	0	0	0	0	0	0
SAL_NA8550AA	PNUSAS033023	Anatum	0	0	0	0	0	0	0	0	0	0	0
SAL_NA8582AA	PNUSAS032986	Anatum	0	0	0	0	0	0	0	0	0	0	0
SAL_NA8365AA	CFSAN075297	Anatum	0	0	0	0	0	0	0	0	0	0	0
SAL_NA8368AA	PNUSAS031950	Anatum	0	0	0	0	0	0	0	0	0	0	0
SAL_NA8320AA	CFSAN075293	Anatum	0	0	0	0	0	0	0	0	0	0	0
SAL_NA9159AA	CFSAN075461	Anatum	0	0	0	0	0	0	0	0	0	0	0
SAL_NA8936AA	FSIS11807274	Anatum	0	0	0	0	0	0	0	0	0	0	0
SAL_NA8841AA	PNUSAS032983	Anatum	0	0	0	0	0	0	0	0	0	0	0
SAL_NA8805AA	FSIS11807211	Anatum	0	0	0	0	0	0	0	0	0	0	0
SAL_NA8816AA	PNUSAS033170	Anatum	0	0	0	0	0	0	0	0	0	0	0
SAL_NA8828AA	PNUSAS033165	Anatum	0	0	0	0	0	0	0	0	0	0	0
SAL_NA8773AA	FSIS11807260	Anatum	0	0	0	0	0	0	0	0	0	0	0
SAL_NA8700AA	PNUSAS032618	Anatum	0	0	0	0	0	0	0	0	0	0	0
SAL_NA8679AA	FDA00004859	Anatum	0	0	0	0	0	0	0	0	0	0	0
SAL_NA8692AA	PNUSAS032776	Anatum	0	0	0	0	0	0	0	0	0	0	0
SAL_NA8651AA	2017K-0883	Anatum	0	0	0	0	0	0	0	0	0	0	0
SAL_NA9298AA	PNUSAS032621	Anatum	0	0	0	0	0	0	0	0	0	0	0
SAL_NA9264AA	PNUSAS032620	Anatum	0	0	0	0	0	0	0	0	0	0	0
SAL_NA9234AA	PNUSAS022925	Anatum	0	0	0	0	0	0	0	0	0	0	0
SAL_NA9211AA	CFSAN075245	Anatum	0	0	0	0	0	0	0	0	0	0	0
SAL_NA9639AA	FSIS11807399	Anatum	0	0	0	0	0	0	0	0	0	0	0
SAL_NA9642AA	FSIS11807401	Anatum	0	0	0	0	0	0	0	0	0	0	0
SAL_NA9648AA	FSIS11807379	Anatum	0	0	0	0	0	0	0	0	0	0	0
SAL_OA0167AA	FSIS11807365	Anatum	0	0	0	0	0	0	0	0	0	0	0
SAL_OA0160AA	FSIS11807550	Anatum	0	0	0	0	0	0	0	0	0	0	0
SAL_OA0153AA	FSIS11807364	Anatum	0	0	0	0	0	0	0	0	0	0	0
SAL_OA0154AA	FSIS11807555	Anatum	0	0	0	0	0	0	0	0	0	0	0
SAL_NA9953AA	FSIS31800091	Anatum	0	0	0	0	0	0	0	0	0	0	0
SAL_NA9961AA	FSIS11807572	Anatum	0	0	0	0	0	0	0	0	0	0	0
SAL_OA0419AA	PNUSAS033508	Anatum	0	0	0	0	0	0	0	0	0	0	0
SAL_OA0395AA	PNUSAS032259	Anatum	0	0	0	0	0	0	0	0	0	0	0
SAL_OA0565AA	FSIS11807538	Anatum	0	0	0	0	0	0	0	0	0	0	0
SAL_OA0573AA	FSIS11807544	Anatum	0	0	0	0	0	0	0	0	0	0	0
SAL_OA0538AA	FSIS31800149	Anatum	0	0	0	0	0	0	0	0	0	0	0
SAL_OA0540AA	FSIS31800144	Anatum	0	0	0	0	0	0	0	0	0	0	0
SAL_OA0782AA	FSIS11807723	Anatum	0	0	0	0	0	0	0	0	0	0	0
SAL_OA0769AA	FSIS11807717	Anatum	0	0	0	0	0	0	0	0	0	0	0
SAL_OA0772AA	FSIS11807737	Anatum	0	0	0	0	0	0	0	0	0	0	0
SAL_OA1123AA	PNUSAS033252	Anatum	0	0	0	0	0	0	0	0	0	0	0
SAL_OA1110AA	PNUSAS033606	Anatum	0	0	0	0	0	0	0	0	0	0	0
SAL_OA7259AA	PNUSAS037166	Anatum	0	0	0	0	0	0	0	0	0	0	0
SAL_OA7389AA	PNUSAS035947	Anatum	0	0	0	0	0	0	0	0	0	0	0
SAL_OA7344AA	PNUSAS037264	Anatum	0	0	0	0	0	0	0	0	0	0	0
SAL_OA7445AA	FSIS1701263	Anatum	0	0	0	0	0	0	0	0	0	0	0
SAL_OA7452AA	FSIS1701265	Anatum	0	0	0	0	0	0	0	0	0	0	0
SAL_PA0063AA	PNUSAS037924	Anatum	0	0	0	0	0	0	0	0	0	0	0
SAL_PA0008AA	PNUSAS036635	Anatum	0	0	0	0	0	0	0	0	0	0	0
SAL_OA9980AA	FSIS11808294	Anatum	0	0	0	0	0	0	0	0	0	0	0
SAL_OA9985AA	FSIS11808292	Anatum	0	0	0	0	0	0	0	0	0	0	0
SAL_OA7486AA	FSIS11808087	Anatum	0	0	0	0	0	0	0	0	0	0	0
SAL_OA7491AA	FSIS11808288	Anatum	0	0	0	0	0	0	0	0	0	0	0
SAL_OA4989AA	PNUSAS036789	Anatum	0	0	0	0	0	0	0	0	0	0	0
SAL_OA5003AA	PNUSAS036272	Anatum	0	0	0	0	0	0	0	0	0	0	0
SAL_OA4971AA	PNUSAS036804	Anatum	0	0	0	0	0	0	0	0	0	0	0
SAL_OA4978AA	PNUSAS036275	Anatum	0	0	0	0	0	0	0	0	0	0	0
SAL_OA4990AA	PNUSAS036797	Anatum	0	0	0	0	0	0	0	0	0	0	0
SAL_OA4341AA	FSIS21821337	Anatum	0	0	0	0	0	0	0	0	0	0	0
SAL_OA4388AA	PNUSAS035674	Anatum	0	0	0	0	0	0	0	0	0	0	0
SAL_OA3793AA	CFSAN075639	Anatum	0	0	0	0	0	0	0	0	0	0	0
SAL_OA3827AA	FSIS21821228	Anatum	0	0	0	0	0	0	0	0	0	0	0
SAL_OA3772AA	FSIS31800195	Anatum	0	0	0	0	0	0	0	0	0	0	0
SAL_OA7999AA	CFSAN078033	Anatum	0	0	0	0	0	0	0	0	0	0	0
SAL_OA8102AA	FSIS11808626	Anatum	0	0	0	0	0	0	0	0	0	0	0
SAL_OA8263AA	PNUSAS036423	Anatum	0	0	0	0	0	0	0	0	0	0	0
SAL_OA9599AA	CFSAN078074	Anatum	0	0	0	0	0	0	0	0	0	0	0
SAL_OA9649AA	PNUSAS037599	Anatum	0	0	0	0	0	0	0	0	0	0	0

SAL_PA0469AA	CFSAN076756	Anatum	0	0	0	0	0	0	0	0	0	0	0
SAL_PA0640AA	CFSAN076754	Anatum	0	0	0	0	0	0	0	0	0	0	0
SAL_PA0584AA	PNUSAS037960	Anatum	0	0	0	0	0	0	0	0	0	0	0
SAL_PA0585AA	PNUSAS037967	Anatum	0	0	0	0	0	0	0	0	0	0	0
SAL_PA0644AA	FSIS11808935	Anatum	0	0	0	0	0	0	0	0	0	0	0
SAL_PA0904AA	PNUSAS036315	Anatum	0	0	0	0	0	0	0	0	0	0	0
SAL_PA1154AA	FSIS31800309	Anatum	0	0	0	0	0	0	0	0	0	0	0
SAL_PA1127AA	FSIS21821389	Anatum	0	0	0	0	0	0	0	0	0	0	0
SAL_PA0996AA	PNUSAS038189	Anatum	0	0	0	0	0	0	0	0	0	0	0
SAL_PA1406AA	PNUSAS028782	Anatum	0	0	0	0	0	0	0	0	0	0	0
SAL_PA1416AA	PNUSAS038261	Anatum	0	0	0	0	0	0	0	0	0	0	0
SAL_PA1239AA	TXAML1800277	Anatum	0	0	0	0	0	0	0	0	0	0	0
SAL_PA0571AA	CFSAN076753	Anatum	0	0	0	0	0	0	0	0	0	0	0
SAL_PA2147AA	FSIS11808942	Anatum	0	0	0	0	0	0	0	0	0	0	0
SAL_PA2473AA	CFSAN078646	Anatum	0	0	0	0	0	0	0	0	0	0	0
SAL_PA2779AA	PNUSAS032533	Anatum	0	0	0	0	0	0	0	0	0	0	0
SAL_PA2567AA	FSIS11808488	Anatum	0	0	0	0	0	0	0	0	0	0	0
SAL_PA2936AA	PNUSAS039183	Anatum	0	0	0	0	0	0	0	0	0	0	0
SAL_PA2558AA	FSIS11808086	Anatum	0	0	0	0	0	0	0	0	0	0	0
SAL_PA3328AA	CFSAN079987	Anatum	0	0	0	0	0	0	0	0	0	0	0
SAL_PA3330AA	CFSAN079988	Anatum	0	0	0	0	0	0	0	0	0	0	0
SAL_PA3318AA	CFSAN079980	Anatum	0	0	0	0	0	0	0	0	0	0	0
SAL_PA3319AA	CFSAN079989	Anatum	0	0	0	0	0	0	0	0	0	0	0
SAL_PA3320AA	CFSAN079984	Anatum	0	0	0	0	0	0	0	0	0	0	0
SAL_PA3236AA	Anatum	Anatum	0	0	0	0	0	0	0	0	0	0	0
SAL_PA4075AA	FSIS21821524	Anatum	0	0	0	0	0	0	0	0	0	0	0
SAL_PA4178AA	FSIS31800366	Anatum	0	0	0	0	0	0	0	0	0	0	0
SAL_PA4563AA	251873	Anatum	0	0	0	0	0	0	0	0	0	0	0
SAL_PA4562AA	FSIS11809555	Anatum	0	0	0	0	0	0	0	0	0	0	0
SAL_PA4402AA	PNUSAS039767	Anatum	0	0	0	0	0	0	0	0	0	0	0
SAL_PA4749AA	FSIS21821506	Anatum	0	0	0	0	0	0	0	0	0	0	0
SAL_PA5120AA	PNUSAS037838	Anatum	0	0	0	0	0	0	0	0	0	0	0
SAL_PA5072AA	PNUSAS039238	Anatum	0	0	0	0	0	0	0	0	0	0	0
SAL_PA5031AA	CFSAN080450	Anatum	0	0	0	0	0	0	0	0	0	0	0
SAL_PA5016AA	CFSAN080433	Anatum	0	0	0	0	0	0	0	0	0	0	0
SAL_PA4919AA	262688	Anatum	0	0	0	0	0	0	0	0	0	0	0
SAL_PA5247AA	CFSAN080587	Anatum	0	0	0	0	0	0	0	0	0	0	0
SAL_PA5477AA	FSIS11809628	Anatum	0	0	0	0	0	0	0	0	0	0	0
SAL_PA5544AA	FSIS11809575	Anatum	0	0	0	0	0	0	0	0	0	0	0
SAL_PA5915AA	PNUSAS038406	Anatum	0	0	0	0	0	0	0	0	0	0	0
SAL_PA6059AA	FSIS11809723	Anatum	0	0	0	0	0	0	0	0	0	0	0
SAL_PA6286AA	PNUSAS039080	Anatum	0	0	0	0	0	0	0	0	0	0	0
SAL_PA7101AA	PNUSAS040827	Anatum	0	0	0	0	0	0	0	0	0	0	0
SAL_PA7067AA	PNUSAS041002	Anatum	0	0	0	0	0	0	0	0	0	0	0
SAL_PA7117AA	FSIS11809881	Anatum	0	0	0	0	0	0	0	0	0	0	0
SAL_PA7126AA	FSIS11809887	Anatum	0	0	0	0	0	0	0	0	0	0	0
SAL_PA7268AA	399384	Anatum	0	0	0	0	0	0	0	0	0	0	0
SAL_PA7240AA	FSIS11809377	Anatum	0	0	0	0	0	0	0	0	0	0	0
SAL_PA7231AA	FSIS11809868	Anatum	0	0	0	0	0	0	0	0	0	0	0
SAL_PA7216AA	FSIS11809802	Anatum	0	0	0	0	0	0	0	0	0	0	0
SAL_PA7350AA	FSIS11809391	Anatum	0	0	0	0	0	0	0	0	0	0	0
SAL_PA7330AA	FSIS11809711	Anatum	0	0	0	0	0	0	0	0	0	0	0
SAL_PA7416AA	PNUSAS040800	Anatum	0	0	0	0	0	0	0	0	0	0	0
SAL_PA7497AA	CFSAN080910	Anatum	0	0	0	0	0	0	0	0	0	0	0
SAL_PA7596AA	PNUSAS039793	Anatum	0	0	0	0	0	0	0	0	0	0	0
SAL_PA7774AA	130400	Anatum	0	0	0	0	0	0	0	0	0	0	0
SAL_PA7725AA	185923	Anatum	0	0	0	0	0	0	0	0	0	0	0
SAL_PA7968AA	257017	Anatum	0	0	0	0	0	0	0	0	0	0	0
SAL_PA7877AA	425575	Anatum	0	0	0	0	0	0	0	0	0	0	0
SAL_PA8283AA	PNUSAS040904	Anatum	0	0	0	0	0	0	0	0	0	0	0
SAL_PA8305AA	FSIS11809400	Anatum	0	0	0	0	0	0	0	0	0	0	0
SAL_PA8309AA	FSIS11809955	Anatum	0	0	0	0	0	0	0	0	0	0	0
SAL_PA8342AA	408238	Anatum	0	0	0	0	0	0	0	0	0	0	0
SAL_PA8363AA	123915	Anatum	0	0	0	0	0	0	0	0	0	0	0
SAL_PA8106AA	PNUSAS040170	Anatum	0	0	0	0	0	0	0	0	0	0	0
SAL_PA8529AA	155620	Anatum	0	0	0	0	0	0	0	0	0	0	0
SAL_PA9119AA	FSIS31800427	Anatum	0	0	0	0	0	0	0	0	0	0	0
SAL_PA9097AA	FSIS21821683	Anatum	0	0	0	0	0	0	0	0	0	0	0
SAL_PA9066AA	402771	Anatum	0	0	0	0	0	0	0	0	0	0	0
SAL_PA9067AA	392859	Anatum	0	0	0	0	0	0	0	0	0	0	0
SAL_PA9372AA	FSIS11808794	Anatum	0	0	0	0	0	0	0	0	0	0	0
SAL_PA8983AA	FSIS11810001	Anatum	0	0	0	0	0	0	0	0	0	0	0
SAL_PA8974AA	FSIS11810007	Anatum	0	0	0	0	0	0	0	0	0	0	0

SAL_PA8981AA	FSIS11810003	Anatum	0	0	0	0	0	0	0	0	0	0	0
SAL_PA9397AA	CFSAN081724	Anatum	0	0	0	0	0	0	0	0	0	0	0
SAL_PA9402AA	CFSAN081726	Anatum	0	0	0	0	0	0	0	0	0	0	0
SAL_PA9403AA	CFSAN081725	Anatum	0	0	0	0	0	0	0	0	0	0	0
SAL_PA9404AA	CFSAN081770	Anatum	0	0	0	0	0	0	0	0	0	0	0
SAL_PA9408AA	CFSAN081729	Anatum	0	0	0	0	0	0	0	0	0	0	0
SAL_PA9410AA	CFSAN081735	Anatum	0	0	0	0	0	0	0	0	0	0	0
SAL_PA9409AA	CFSAN081728	Anatum	0	0	0	0	0	0	0	0	0	0	0
SAL_PA9412AA	CFSAN081731	Anatum	0	0	0	0	0	0	0	0	0	0	0
SAL_PA9411AA	CFSAN081727	Anatum	0	0	0	0	0	0	0	0	0	0	0
SAL_PA9390AA	CFSAN081733	Anatum	0	0	0	0	0	0	0	0	0	0	0
SAL_PA9391AA	CFSAN081732	Anatum	0	0	0	0	0	0	0	0	0	0	0
SAL_PA9398AA	CFSAN081723	Anatum	0	0	0	0	0	0	0	0	0	0	0
SAL_PA9532AA	CFSAN081738	Anatum	0	0	0	0	0	0	0	0	0	0	0
SAL_PA9547AA	PNUSAS041292	Anatum	0	0	0	0	0	0	0	0	0	0	0
SAL_PA9825AA	PNUSAS041687	Anatum	0	0	0	0	0	0	0	0	0	0	0
SAL_PA9716AA	397704	Anatum	0	0	0	0	0	0	0	0	0	0	0
SAL_PA9613AA	FSIS11808800	Anatum	0	0	0	0	0	0	0	0	0	0	0
SAL_QA0222AA	PNUSAS041727	Anatum	0	0	0	0	0	0	0	0	0	0	0
SAL_QA0170AA	PNUSAS041937	Anatum	0	0	0	0	0	0	0	0	0	0	0
SAL_PA9413AA	CFSAN081730	Anatum	0	0	0	0	0	0	0	0	0	0	0
SAL_PA9415AA	CFSAN081736	Anatum	0	0	0	0	0	0	0	0	0	0	0
SAL_PA9414AA	CFSAN081737	Anatum	0	0	0	0	0	0	0	0	0	0	0
SAL_PA9417AA	CFSAN081722	Anatum	0	0	0	0	0	0	0	0	0	0	0
SAL_QA0480AA	261358	Anatum	0	0	0	0	0	0	0	0	0	0	0
SAL_QA0568AA	PNUSAS041943	Anatum	0	0	0	0	0	0	0	0	0	0	0
SAL_QA0635AA	CFSAN081787	Anatum	0	0	0	0	0	0	0	0	0	0	0
SAL_QA0616AA	CFSAN081780	Anatum	0	0	0	0	0	0	0	0	0	0	0
SAL_QA0459AA	428169	Anatum	0	0	0	0	0	0	0	0	0	0	0
SAL_QA0814AA	PNUSAS041398	Anatum	0	0	0	0	0	0	0	0	0	0	0
SAL_QA0793AA	PNUSAS019348	Anatum	0	0	0	0	0	0	0	0	0	0	0
SAL_QA1229AA	FSIS11810053	Anatum	0	0	0	0	0	0	0	0	0	0	0
SAL_QA1200AA	FSIS21821771	Anatum	0	0	0	0	0	0	0	0	0	0	0
SAL_QA1151AA	FSIS11810193	Anatum	0	0	0	0	0	0	0	0	0	0	0
SAL_QA1153AA	FSIS11808940	Anatum	0	0	0	0	0	0	0	0	0	0	0
SAL_QA1156AA	FSIS11809397	Anatum	0	0	0	0	0	0	0	0	0	0	0
SAL_QA1163AA	FSIS11810189	Anatum	0	0	0	0	0	0	0	0	0	0	0
SAL_QA1114AA	PNUSAS042591	Anatum	0	0	0	0	0	0	0	0	0	0	0
SAL_QA0841AA	327974	Anatum	0	0	0	0	0	0	0	0	0	0	0
SAL_QA1479AA	267746	Anatum	0	0	0	0	0	0	0	0	0	0	0
SAL_QA1424AA	288968	Anatum	0	0	0	0	0	0	0	0	0	0	0
SAL_QA2499AA	433565	Anatum	0	0	0	0	0	0	0	0	0	0	0
SAL_QA2466AA	186566	Anatum	0	0	0	0	0	0	0	0	0	0	0
SAL_QA2335AA	174777	Anatum	0	0	0	0	0	0	0	0	0	0	0
SAL_QA2292AA	140703	Anatum	0	0	0	0	0	0	0	0	0	0	0
SAL_QA2188AA	136559	Anatum	0	0	0	0	0	0	0	0	0	0	0
SAL_QA2219AA	202254	Anatum	0	0	0	0	0	0	0	0	0	0	0
SAL_QA2100AA	342660	Anatum	0	0	0	0	0	0	0	0	0	0	0
SAL_QA2599AA	FSIS31800510	Anatum	0	0	0	0	0	0	0	0	0	0	0
SAL_QA2571AA	PNUSAS042725	Anatum	0	0	0	0	0	0	0	0	0	0	0
SAL_QA3214AA	127424	Anatum	0	0	0	0	0	0	0	0	0	0	0
SAL_QA3707AA	PNUSAS041409	Anatum	0	0	0	0	0	0	0	0	0	0	0
SAL_QA3670AA	PNUSAS020317	Anatum	0	0	0	0	0	0	0	0	0	0	0
SAL_QA3672AA	PNUSAS019321	Anatum	0	0	0	0	0	0	0	0	0	0	0
SAL_QA3662AA	PNUSAS019323	Anatum	0	0	0	0	0	0	0	0	0	0	0
SAL_QA3747AA	FSIS21821889	Anatum	0	0	0	0	0	0	0	0	0	0	0
SAL_QA4336AA	PNUSAS036495	Anatum	0	0	0	0	0	0	0	0	0	0	0
SAL_QA4222AA	PNUSAS043142	Anatum	0	0	0	0	0	0	0	0	0	0	0
SAL_QA4225AA	PNUSAS043138	Anatum	0	0	0	0	0	0	0	0	0	0	0
SAL_QA4487AA	PNUSAS039509	Anatum	0	0	0	0	0	0	0	0	0	0	0
SAL_QA4615AA	FSIS11810430	Anatum	0	0	0	0	0	0	0	0	0	0	0
SAL_QA4605AA	FSIS11810418	Anatum	0	0	0	0	0	0	0	0	0	0	0
SAL_QA4584AA	FSIS11810448	Anatum	0	0	0	0	0	0	0	0	0	0	0
SAL_QA4609AA	FSIS11810429	Anatum	0	0	0	0	0	0	0	0	0	0	0
SAL_QA2812AA	FSIS11810466	Anatum	0	0	0	0	0	0	0	0	0	0	0
SAL_QA4805AA	216327	Anatum	0	0	0	0	0	0	0	0	0	0	0
SAL_QA5476AA	245609	Anatum	0	0	0	0	0	0	0	0	0	0	0
SAL_QA5219AA	370625	Anatum	0	0	0	0	0	0	0	0	0	0	0
SAL_QA5662AA	PNUSAS040632	Anatum	0	0	0	0	0	0	0	0	0	0	0
SAL_QA5919AA	FSIS11810727	Anatum	0	0	0	0	0	0	0	0	0	0	0
SAL_QA5934AA	FSIS11810646	Anatum	0	0	0	0	0	0	0	0	0	0	0
SAL_QA5887AA	474903	Anatum	0	0	0	0	0	0	0	0	0	0	0
SAL_QA5743AA	338389	Anatum	0	0	0	0	0	0	0	0	0	0	0

SAL_QA6260AA	393977	Anatum	0	0	0	0	0	0	0	0	0	0	0
SAL_QA6201AA	302556	Anatum	0	0	0	0	0	0	0	0	0	0	0
SAL_QA6145AA	FDA00005055	Anatum	0	0	0	0	0	0	0	0	0	0	0
SAL_QA6508AA	298800	Anatum	0	0	0	0	0	0	0	0	0	0	0
SAL_QA6647AA	TTU_071	Anatum	0	0	0	0	0	0	0	0	0	0	0
SAL_QA6888AA	FSIS11810659	Anatum	0	0	0	0	0	0	0	0	0	0	0
SAL_QA6831AA	TTU_070	Anatum	0	0	0	0	0	0	0	0	0	0	0
SAL_QA6673AA	TTU_075	Anatum	0	0	0	0	0	0	0	0	0	0	0
SAL_QA6680AA	TTU_077	Anatum	0	0	0	0	0	0	0	0	0	0	0
SAL_QA7218AA	TTU_074	Anatum	0	0	0	0	0	0	0	0	0	0	0
SAL_QA7219AA	TTU_069	Anatum	0	0	0	0	0	0	0	0	0	0	0
SAL_QA7432AA	404934	Anatum	0	0	0	0	0	0	0	0	0	0	0
SAL_QA7378AA	399787	Anatum	0	0	0	0	0	0	0	0	0	0	0
SAL_QA7279AA	360308	Anatum	0	0	0	0	0	0	0	0	0	0	0
SAL_QA6002AA	PNUSAS043110	Anatum	0	0	0	0	0	0	0	0	0	0	0
SAL_QA7559AA	127420	Anatum	0	0	0	0	0	0	0	0	0	0	0
SAL_QA7486AA	302864	Anatum	0	0	0	0	0	0	0	0	0	0	0
SAL_QA8186AA	503562	Anatum	0	0	0	0	0	0	0	0	0	0	0
SAL_QA8500AA	PNUSAS042301	Anatum	0	0	0	0	0	0	0	0	0	0	0
SAL_QA8655AA	342657	Anatum	0	0	0	0	0	0	0	0	0	0	0
SAL_QA8949AA	PNUSAS044771	Anatum	0	0	0	0	0	0	0	0	0	0	0
SAL_QA8955AA	PNUSAS044572	Anatum	0	0	0	0	0	0	0	0	0	0	0
SAL_QA9244AA	FSIS31800634	Anatum	0	0	0	0	0	0	0	0	0	0	0
SAL_QA8897AA	PNUSAS042766	Anatum	0	0	0	0	0	0	0	0	0	0	0
SAL_QA9495AA	436913	Anatum	0	0	0	0	0	0	0	0	0	0	0
SAL_QA9667AA	PNUSAS044474	Anatum	0	0	0	0	0	0	0	0	0	0	0
SAL_QA9265AA	FSIS11811110	Anatum	0	0	0	0	0	0	0	0	0	0	0
SAL_QA9695AA	FSIS11811113	Anatum	0	0	0	0	0	0	0	0	0	0	0
SAL_QA9697AA	FSIS11811164	Anatum	0	0	0	0	0	0	0	0	0	0	0
SAL_RA0418AA	178745	Anatum	0	0	0	0	0	0	0	0	0	0	0
SAL_RA1037AA	420579	Anatum	0	0	0	0	0	0	0	0	0	0	0
SAL_RA1038AA	185962	Anatum	0	0	0	0	0	0	0	0	0	0	0
SAL_RA0990AA	404938	Anatum	0	0	0	0	0	0	0	0	0	0	0
SAL_RA0950AA	FSIS11811244	Anatum	0	0	0	0	0	0	0	0	0	0	0
SAL_RA0927AA	FSIS11810240	Anatum	0	0	0	0	0	0	0	0	0	0	0
SAL_RA0928AA	FSIS11811245	Anatum	0	0	0	0	0	0	0	0	0	0	0
SAL_RA0841AA	275964	Anatum	0	0	0	0	0	0	0	0	0	0	0
SAL_RA0786AA	453218	Anatum	0	0	0	0	0	0	0	0	0	0	0
SAL_RA0731AA	397700	Anatum	0	0	0	0	0	0	0	0	0	0	0
SAL_RA1177AA	FSIS11811099	Anatum	0	0	0	0	0	0	0	0	0	0	0
SAL_RA1179AA	FSIS11811102	Anatum	0	0	0	0	0	0	0	0	0	0	0
SAL_RA1278AA	FSIS11811096	Anatum	0	0	0	0	0	0	0	0	0	0	0
SAL_RA1244AA	FSIS11811255	Anatum	0	0	0	0	0	0	0	0	0	0	0
SAL_RA1266AA	FSIS11811178	Anatum	0	0	0	0	0	0	0	0	0	0	0
SAL_RA1200AA	PNUSAS044830	Anatum	0	0	0	0	0	0	0	0	0	0	0
SAL_RA2521AA	PNUSAS044601	Anatum	0	0	0	0	0	0	0	0	0	0	0
SAL_RA2528AA	PNUSAS045105	Anatum	0	0	0	0	0	0	0	0	0	0	0
SAL_RA2749AA	373208	Anatum	0	0	0	0	0	0	0	0	0	0	0
SAL_RA3398AA	337087	Anatum	0	0	0	0	0	0	0	0	0	0	0
SAL_RA3738AA	311423	Anatum	0	0	0	0	0	0	0	0	0	0	0
SAL_RA3543AA	FSIS11811384	Anatum	0	0	0	0	0	0	0	0	0	0	0
SAL_RA3547AA	FSIS11811378	Anatum	0	0	0	0	0	0	0	0	0	0	0
SAL_RA4189AA	FSIS11811478	Anatum	0	0	0	0	0	0	0	0	0	0	0
SAL_RA4111AA	360502	Anatum	0	0	0	0	0	0	0	0	0	0	0
SAL_RA4249AA	FDA00005056	Anatum	0	0	0	0	0	0	0	0	0	0	0
SAL_RA3977AA	416028	Anatum	0	0	0	0	0	0	0	0	0	0	0
SAL_RA4226AA	TTU_193	Anatum	0	0	0	0	0	0	0	0	0	0	0
SAL_RA3879AA	CFSAN082369	Anatum	0	0	0	0	0	0	0	0	0	0	0
SAL_RA3845AA	FDA00001571	Anatum	0	0	0	0	0	0	0	0	0	0	0
SAL_RA3865AA	TTU_068	Anatum	0	0	0	0	0	0	0	0	0	0	0
SAL_RA3838AA	TTU_076	Anatum	0	0	0	0	0	0	0	0	0	0	0
SAL_RA3827AA	HIY0161	Anatum	0	0	0	0	0	0	0	0	0	0	0
SAL_RA4264AA	CFSAN082368	Anatum	0	0	0	0	0	0	0	0	0	0	0
SAL_RA4520AA	261434	Anatum	0	0	0	0	0	0	0	0	0	0	0
SAL_RA4327AA	203633	Anatum	0	0	0	0	0	0	0	0	0	0	0
SAL_RA4301AA	272436	Anatum	0	0	0	0	0	0	0	0	0	0	0
SAL_RA5832AA	PNUSAS043178	Anatum	0	0	0	0	0	0	0	0	0	0	0
SAL_RA5743AA	CFSAN082948	Anatum	0	0	0	0	0	0	0	0	0	0	0
SAL_RA5745AA	CFSAN082947	Anatum	0	0	0	0	0	0	0	0	0	0	0
SAL_RA5644AA	267020	Anatum	0	0	0	0	0	0	0	0	0	0	0
SAL_RA5686AA	397664	Anatum	0	0	0	0	0	0	0	0	0	0	0
SAL_RA5482AA	231236	Anatum	0	0	0	0	0	0	0	0	0	0	0
SAL_SA0882AA	SMG-18-1161	Anatum	0	0	0	0	0	0	0	0	0	0	0

SAL_RA6381AA	PNUSAS044931	Anatum	0	0	0	0	0	0	0	0	0	0	0
SAL_RA6643AA	338407	Anatum	0	0	0	0	0	0	0	0	0	0	0
SAL_RA6646AA	434323	Anatum	0	0	0	0	0	0	0	0	0	0	0
SAL_SA1774AA	MC_07-0751	Anatum	0	0	0	0	0	0	0	0	0	0	0
SAL_RA6785AA	PNUSAS046233	Anatum	0	0	0	0	0	0	0	0	0	0	0
SAL_RA6964AA	FSIS11811375	Anatum	0	0	0	0	0	0	0	0	0	0	0
SAL_RA6944AA	FSIS11811372	Anatum	0	0	0	0	0	0	0	0	0	0	0
SAL_RA7610AA	PNUSAS043205	Anatum	0	0	0	0	0	0	0	0	0	0	0
SAL_RA7187AA	363502	Anatum	0	0	0	0	0	0	0	0	0	0	0
SAL_RA7155AA	227024	Anatum	0	0	0	0	0	0	0	0	0	0	0
SAL_RA7100AA	300680	Anatum	0	0	0	0	0	0	0	0	0	0	0
SAL_RA7022AA	265288	Anatum	0	0	0	0	0	0	0	0	0	0	0
SAL_RA7835AA	178846	Anatum	0	0	0	0	0	0	0	0	0	0	0
SAL_RA7772AA	FSIS11811639	Anatum	0	0	0	0	0	0	0	0	0	0	0
SAL_RA7787AA	FSIS11811617	Anatum	0	0	0	0	0	0	0	0	0	0	0
SAL_RA7767AA	FSIS11811628	Anatum	0	0	0	0	0	0	0	0	0	0	0
SAL_RA7646AA	FSIS11811949	Anatum	0	0	0	0	0	0	0	0	0	0	0
SAL_RA7643AA	FSIS11811943	Anatum	0	0	0	0	0	0	0	0	0	0	0
SAL_RA7638AA	FSIS11811953	Anatum	0	0	0	0	0	0	0	0	0	0	0
SAL_RA7640AA	FSIS11811940	Anatum	0	0	0	0	0	0	0	0	0	0	0
SAL_RA7653AA	FSIS11811962	Anatum	0	0	0	0	0	0	0	0	0	0	0
SAL_RA7633AA	FSIS11811950	Anatum	0	0	0	0	0	0	0	0	0	0	0
SAL_RA8335AA	221713	Anatum	0	0	0	0	0	0	0	0	0	0	0
SAL_RA8270AA	312108	Anatum	0	0	0	0	0	0	0	0	0	0	0
SAL_RA8420AA	FSIS11811967	Anatum	0	0	0	0	0	0	0	0	0	0	0
SAL_RA8412AA	FSIS11811108	Anatum	0	0	0	0	0	0	0	0	0	0	0
SAL_RA8180AA	SAL-18-VL-LA-TX-0001	Anatum	0	0	0	0	0	0	0	0	0	0	0
SAL_RA8528AA	TTU_243	Anatum	0	0	0	0	0	0	0	0	0	0	0
SAL_RA8527AA	TTU_250	Anatum	0	0	0	0	0	0	0	0	0	0	0
SAL_RA8687AA	FSIS11812187	Anatum	0	0	0	0	0	0	0	0	0	0	0
SAL_RA8692AA	FSIS11812191	Anatum	0	0	0	0	0	0	0	0	0	0	0
SAL_RA8660AA	FSIS11812163	Anatum	0	0	0	0	0	0	0	0	0	0	0
SAL_RA8645AA	FSIS11812221	Anatum	0	0	0	0	0	0	0	0	0	0	0
SAL_RA8707AA	FSIS11812196	Anatum	0	0	0	0	0	0	0	0	0	0	0
SAL_RA8729AA	FSIS11812039	Anatum	0	0	0	0	0	0	0	0	0	0	0
SAL_RA9243AA	CFSAN083985	Anatum	0	0	0	0	0	0	0	0	0	0	0
SAL_RA9754AA	CFSAN084027	Anatum	0	0	0	0	0	0	0	0	0	0	0
SAL_RA9770AA	TTU_247	Anatum	0	0	0	0	0	0	0	0	0	0	0
SAL_RA9889AA	SAL-18-VL-LA-TX-0004	Anatum	0	0	0	0	0	0	0	0	0	0	0
SAL_SA0111AA	CVM N17S263	Anatum	0	0	0	0	0	0	0	0	0	0	0
SAL_RA9997AA	FSIS31800843	Anatum	0	0	0	0	0	0	0	0	0	0	0
SAL_RA9972AA	FSIS11812397	Anatum	0	0	0	0	0	0	0	0	0	0	0
SAL_RA9965AA	FSIS11812451	Anatum	0	0	0	0	0	0	0	0	0	0	0
SAL_RA9960AA	FSIS11812450	Anatum	0	0	0	0	0	0	0	0	0	0	0
SAL_SA0597AA	FSIS11812440	Anatum	0	0	0	0	0	0	0	0	0	0	0
SAL_SA0585AA	FSIS11812688	Anatum	0	0	0	0	0	0	0	0	0	0	0
SAL_RA7579AA	PNUSAS045154	Anatum	0	0	0	0	0	0	0	0	0	0	0
SAL_RA7061AA	431279	Anatum	0	0	0	0	0	0	0	0	0	0	0
SAL_SA0851AA	FSIS11812736	Anatum	0	0	0	0	0	0	0	0	0	0	0
SAL_SA0963AA	CC06.031	Anatum	0	0	0	0	0	0	0	0	0	0	0
SAL_SA0965AA	CA08.145	Anatum	0	0	0	0	0	0	0	0	0	0	0
SAL_SA0966AA	CS182	Anatum	0	0	0	0	0	0	0	0	0	0	0
SAL_SA0957AA	R16.3927	Anatum	0	0	0	0	0	0	0	0	0	0	0
SAL_SA0958AA	R16.4304	Anatum	0	0	0	0	0	0	0	0	0	0	0
SAL_SA0960AA	D013	Anatum	0	0	0	0	0	0	0	0	0	0	0
SAL_SA0959AA	D020	Anatum	0	0	0	0	0	0	0	0	0	0	0
SAL_SA0961AA	R17.3211	Anatum	0	0	0	0	0	0	0	0	0	0	0
SAL_SA0962AA	CF09.078	Anatum	0	0	0	0	0	0	0	0	0	0	0
SAL_SA0964AA	CC04.028	Anatum	0	0	0	0	0	0	0	0	0	0	0
SAL_SA0942AA	R13.1215	Anatum	0	0	0	0	0	0	0	0	0	0	0
SAL_SA0948AA	R16.2821	Anatum	0	0	0	0	0	0	0	0	0	0	0
SAL_SA0949AA	R16.1231	Anatum	0	0	0	0	0	0	0	0	0	0	0
SAL_SA0950AA	R16.1486	Anatum	0	0	0	0	0	0	0	0	0	0	0
SAL_SA0952AA	R17.4426	Anatum	0	0	0	0	0	0	0	0	0	0	0
SAL_SA0953AA	R17.4643	Anatum	0	0	0	0	0	0	0	0	0	0	0
SAL_SA0954AA	R17.5171	Anatum	0	0	0	0	0	0	0	0	0	0	0
SAL_SA0955AA	SA11.164	Anatum	0	0	0	0	0	0	0	0	0	0	0
SAL_SA0956AA	SG06.139	Anatum	0	0	0	0	0	0	0	0	0	0	0
SAL_SA0967AA	CI07.001	Anatum	0	0	0	0	0	0	0	0	0	0	0
SAL_RA8529AA	TTU_249	Anatum	0	0	0	0	0	0	0	0	0	0	0
SAL_SA0943AA	R16.3355	Anatum	0	0	0	0	0	0	0	0	0	0	0
SAL_SA0946AA	R16.3115	Anatum	0	0	0	0	0	0	0	0	0	0	0
SAL_SA0951AA	R17.3086	Anatum	0	0	0	0	0	0	0	0	0	0	0

SAL_SA0947AA	R16.2802	Anatum	0	0	0	0	0	0	0	0	0	0	0
SAL_SA0968AA	CH07.062	Anatum	0	0	0	0	0	0	0	0	0	0	0
SAL_SA0969AA	CH05.023	Anatum	0	0	0	0	0	0	0	0	0	0	0
SAL_SA0932AA	R15.0695	Anatum	0	0	0	0	0	0	0	0	0	0	0
SAL_SA0933AA	R15.0913	Anatum	0	0	0	0	0	0	0	0	0	0	0
SAL_SA0935AA	R13.1671	Anatum	0	0	0	0	0	0	0	0	0	0	0
SAL_SA0936AA	R13.2266	Anatum	0	0	0	0	0	0	0	0	0	0	0
SAL_SA0937AA	R14.1408	Anatum	0	0	0	0	0	0	0	0	0	0	0
SAL_SA0934AA	R17.3154	Anatum	0	0	0	0	0	0	0	0	0	0	0
SAL_SA0939AA	P165	Anatum	0	0	0	0	0	0	0	0	0	0	0
SAL_SA0940AA	PS23	Anatum	0	0	0	0	0	0	0	0	0	0	0
SAL_SA0941AA	R13.0957	Anatum	0	0	0	0	0	0	0	0	0	0	0
SAL_SA0944AA	R16.3623	Anatum	0	0	0	0	0	0	0	0	0	0	0
SAL_SA0945AA	R16.2885	Anatum	0	0	0	0	0	0	0	0	0	0	0
SAL_SA0938AA	R15.0600	Anatum	0	0	0	0	0	0	0	0	0	0	0
SAL_SA0930AA	R16.4391	Anatum	0	0	0	0	0	0	0	0	0	0	0
SAL_SA0929AA	R17.3110	Anatum	0	0	0	0	0	0	0	0	0	0	0
SAL_SA0931AA	R17.3160	Anatum	0	0	0	0	0	0	0	0	0	0	0
SAL_SA0928AA	SN08.005	Anatum	0	0	0	0	0	0	0	0	0	0	0
SAL_SA0917AA	R15.1365	Anatum	0	0	0	0	0	0	0	0	0	0	0
SAL_SA0918AA	R15.1294	Anatum	0	0	0	0	0	0	0	0	0	0	0
SAL_SA0920AA	R16.0460	Anatum	0	0	0	0	0	0	0	0	0	0	0
SAL_SA0919AA	R16.0569	Anatum	0	0	0	0	0	0	0	0	0	0	0
SAL_SA0908AA	EA04.039	Anatum	0	0	0	0	0	0	0	0	0	0	0
SAL_SA0909AA	MS32915	Anatum	0	0	0	0	0	0	0	0	0	0	0
SAL_SA0911AA	NJ08.181	Anatum	0	0	0	0	0	0	0	0	0	0	0
SAL_SA0910AA	MS32850	Anatum	0	0	0	0	0	0	0	0	0	0	0
SAL_SA0912AA	NC04.178	Anatum	0	0	0	0	0	0	0	0	0	0	0
SAL_SA0914AA	NK04.008	Anatum	0	0	0	0	0	0	0	0	0	0	0
SAL_SA0925AA	R17.3203	Anatum	0	0	0	0	0	0	0	0	0	0	0
SAL_SA0926AA	R16.4880	Anatum	0	0	0	0	0	0	0	0	0	0	0
SAL_SA0927AA	R17.3140	Anatum	0	0	0	0	0	0	0	0	0	0	0
SAL_SA0903AA	R17.0132	Anatum	0	0	0	0	0	0	0	0	0	0	0
SAL_SA0913AA	NL05.024	Anatum	0	0	0	0	0	0	0	0	0	0	0
SAL_SA0904AA	P164	Anatum	0	0	0	0	0	0	0	0	0	0	0
SAL_SA0923AA	R16.1070	Anatum	0	0	0	0	0	0	0	0	0	0	0
SAL_SA0922AA	R16.0274	Anatum	0	0	0	0	0	0	0	0	0	0	0
SAL_SA0905AA	P049	Anatum	0	0	0	0	0	0	0	0	0	0	0
SAL_SA1145AA	FSIS11813008	Anatum	0	0	0	0	0	0	0	0	0	0	0
SAL_SA1132AA	FSIS11812420	Anatum	0	0	0	0	0	0	0	0	0	0	0
SAL_SA1134AA	FSIS11813006	Anatum	0	0	0	0	0	0	0	0	0	0	0
SAL_SA1135AA	FSIS11812999	Anatum	0	0	0	0	0	0	0	0	0	0	0
SAL_SA2497AA	AG18-0021	Anatum	0	0	0	0	0	0	0	0	0	0	0
SAL_SA2542AA	FSIS11812681	Anatum	0	0	0	0	0	0	0	0	0	0	0
SAL_SA2574AA	FSIS11812983	Anatum	0	0	0	0	0	0	0	0	0	0	0
SAL_SA2580AA	FSIS11812984	Anatum	0	0	0	0	0	0	0	0	0	0	0
SAL_SA2639AA	FSIS11812973	Anatum	0	0	0	0	0	0	0	0	0	0	0
SAL_SA2622AA	FSIS11812965	Anatum	0	0	0	0	0	0	0	0	0	0	0
SAL_SA2677AA	FSIS11813222	Anatum	0	0	0	0	0	0	0	0	0	0	0
SAL_SA2853AA	FSIS11813205	Anatum	0	0	0	0	0	0	0	0	0	0	0
SAL_SA2855AA	FSIS11813192	Anatum	0	0	0	0	0	0	0	0	0	0	0
SAL_SA2844AA	R18.1458	Anatum	0	0	0	0	0	0	0	0	0	0	0
SAL_SA2843AA	R18.1457	Anatum	0	0	0	0	0	0	0	0	0	0	0
SAL_SA2846AA	FSIS11813202	Anatum	0	0	0	0	0	0	0	0	0	0	0
SAL_SA2927AA	FSIS21821978	Anatum	0	0	0	0	0	0	0	0	0	0	0
SAL_SA0916AA	R15.1977	Anatum	0	0	0	0	0	0	0	0	0	0	0
SAL_SA0915AA	R15.2697	Anatum	0	0	0	0	0	0	0	0	0	0	0
SAL_SA0924AA	R16.0696	Anatum	0	0	0	0	0	0	0	0	0	0	0
SAL_SA0906AA	R17.3161	Anatum	0	0	0	0	0	0	0	0	0	0	0
SAL_SA0907AA	EA04.047	Anatum	0	0	0	0	0	0	0	0	0	0	0
SAL_SA3315AA	OH-18-18132-1	Anatum	0	0	0	0	0	0	0	0	0	0	0
SAL_SA3438AA	FDA00013420	Anatum	0	0	0	0	0	0	0	0	0	0	0
SAL_SA3431AA	FDA00004915	Anatum	0	0	0	0	0	0	0	0	0	0	0
SAL_SA3625AA	FSIS31800953	Anatum	0	0	0	0	0	0	0	0	0	0	0
SAL_SA3613AA	FSIS31800957	Anatum	0	0	0	0	0	0	0	0	0	0	0
SAL_SA3649AA	FSIS11813686	Anatum	0	0	0	0	0	0	0	0	0	0	0
SAL_SA3637AA	FSIS11813665	Anatum	0	0	0	0	0	0	0	0	0	0	0
SAL_SA3790AA	SAL-18-VL-NY-FL-0004	Anatum	0	0	0	0	0	0	0	0	0	0	0
SAL_SA3769AA	FSIS11813409	Anatum	0	0	0	0	0	0	0	0	0	0	0
SAL_SA3778AA	FSIS11813413	Anatum	0	0	0	0	0	0	0	0	0	0	0
SAL_SA3759AA	FSIS11813232	Anatum	0	0	0	0	0	0	0	0	0	0	0
SAL_SA3724AA	FSIS11813427	Anatum	0	0	0	0	0	0	0	0	0	0	0
SAL_SA3713AA	FSIS11813426	Anatum	0	0	0	0	0	0	0	0	0	0	0

SAL_SA3787AA	SAL-18-VL-NY-GA-T-0001	Anatum	0	0	0	0	0	0	0	0	0	0	0
SAL_SA3690AA	FSIS11813703	Anatum	0	0	0	0	0	0	0	0	0	0	0
SAL_SA3841AA	SAL-18-VL-SD-IA-0004	Anatum	0	0	0	0	0	0	0	0	0	0	0
SAL_SA3850AA	SAL-18-VL-SD-OK-0004	Anatum	0	0	0	0	0	0	0	0	0	0	0
SAL_SA0921AA	R16.0348	Anatum	0	0	0	0	0	0	0	0	0	0	0
SAL_SA3745AA	FSIS11813429	Anatum	0	0	0	0	0	0	0	0	0	0	0
SAL_SA3706AA	FSIS11813706	Anatum	0	0	0	0	0	0	0	0	0	0	0
SAL_SA4061AA	FSIS31800983	Anatum	0	0	0	0	0	0	0	0	0	0	0
SAL_SA4032AA	FSIS21822075	Anatum	0	0	0	0	0	0	0	0	0	0	0
SAL_SA4317AA	FSIS21822096	Anatum	0	0	0	0	0	0	0	0	0	0	0
SAL_SA4326AA	FSIS21822095	Anatum	0	0	0	0	0	0	0	0	0	0	0
SAL_SA4288AA	FSIS11813882	Anatum	0	0	0	0	0	0	0	0	0	0	0
SAL_SA4274AA	FSIS11813445	Anatum	0	0	0	0	0	0	0	0	0	0	0
SAL_SA4285AA	FSIS11813446	Anatum	0	0	0	0	0	0	0	0	0	0	0
SAL_SA5758AA	FSIS31800981	Anatum	0	0	0	0	0	0	0	0	0	0	0
SAL_SA5771AA	FSIS11813894	Anatum	0	0	0	0	0	0	0	0	0	0	0
SAL_SA4796AA	587454	Anatum	0	0	0	0	0	0	0	0	0	0	0
SAL_SA6030AA	FSIS11813893	Anatum	0	0	0	0	0	0	0	0	0	0	0
SAL_SA5828AA	578011	Anatum	0	0	0	0	0	0	0	0	0	0	0
SAL_SA6398AA	565958	Anatum	0	0	0	0	0	0	0	0	0	0	0
SAL_SA6368AA	602615	Anatum	0	0	0	0	0	0	0	0	0	0	0
SAL_SA5765AA	FSIS31801043	Anatum	0	0	0	0	0	0	0	0	0	0	0
SAL_SA7249AA	575097	Anatum	0	0	0	0	0	0	0	0	0	0	0
SAL_SA7203AA	578023	Anatum	0	0	0	0	0	0	0	0	0	0	0
SAL_SA6893AA		Anatum	0	0	0	0	0	0	0	0	0	0	0
SAL_SA6894AA		Anatum	0	0	0	0	0	0	0	0	0	0	0
SAL_SA7376AA	FSIS11813694	Anatum	0	0	0	0	0	0	0	0	0	0	0
SAL_SA7625AA	FSIS11814166	Anatum	0	0	0	0	0	0	0	0	0	0	0
SAL_SA7927AA	373956	Anatum	0	0	0	0	0	0	0	0	0	0	0
SAL_SA8709AA	453226	Anatum	0	0	0	0	0	0	0	0	0	0	0
SAL_SA8566AA	438775	Anatum	0	0	0	0	0	0	0	0	0	0	0
SAL_SA8559AA	594707	Anatum	0	0	0	0	0	0	0	0	0	0	0
SAL_SA8778AA	FSIS11814105	Anatum	0	0	0	0	0	0	0	0	0	0	0
SAL_SA8784AA	FSIS11814104	Anatum	0	0	0	0	0	0	0	0	0	0	0
SAL_SA9008AA	FDA00007869	Anatum	0	0	0	0	0	0	0	0	0	0	0
SAL_SA9011AA	FDA00004886	Anatum	0	0	0	0	0	0	0	0	0	0	0
SAL_SA9164AA	CVM N17S621	Anatum	0	0	0	0	0	0	0	0	0	0	0
SAL_SA9532AA	424594	Anatum	0	0	0	0	0	0	0	0	0	0	0
SAL_SA9538AA	492499	Anatum	0	0	0	0	0	0	0	0	0	0	0
SAL_SA9655AA	FSIS11814297	Anatum	0	0	0	0	0	0	0	0	0	0	0
SAL_SA9657AA	FSIS11814306	Anatum	0	0	0	0	0	0	0	0	0	0	0
SAL_SA9664AA	FSIS31801089	Anatum	0	0	0	0	0	0	0	0	0	0	0
SAL_SA9609AA	FSIS21822188	Anatum	0	0	0	0	0	0	0	0	0	0	0
SAL_SA9634AA	FSIS11814301	Anatum	0	0	0	0	0	0	0	0	0	0	0
SAL_SA9638AA	FSIS11814303	Anatum	0	0	0	0	0	0	0	0	0	0	0
SAL_SA9645AA	FSIS11814290	Anatum	0	0	0	0	0	0	0	0	0	0	0
SAL_SA9636AA	FSIS11814314	Anatum	0	0	0	0	0	0	0	0	0	0	0
SAL_SA9710AA	FSIS31801046	Anatum	0	0	0	0	0	0	0	0	0	0	0
SAL_SA9686AA	TTU_251	Anatum	0	0	0	0	0	0	0	0	0	0	0
SAL_SA9695AA	FSIS31801047	Anatum	0	0	0	0	0	0	0	0	0	0	0
SAL_SA9683AA	TXACF1801691	Anatum	0	0	0	0	0	0	0	0	0	0	0
SAL_TA0031AA	FSIS21822240	Anatum	0	0	0	0	0	0	0	0	0	0	0
SAL_TA0160AA	FSIS11814486	Anatum	0	0	0	0	0	0	0	0	0	0	0
SAL_TA0134AA	FSIS31801109	Anatum	0	0	0	0	0	0	0	0	0	0	0
SAL_TA0122AA	FSIS31801116	Anatum	0	0	0	0	0	0	0	0	0	0	0
SAL_TA0128AA	FSIS31801120	Anatum	0	0	0	0	0	0	0	0	0	0	0
SAL_TA0095AA	FSIS11814463	Anatum	0	0	0	0	0	0	0	0	0	0	0
SAL_TA0515AA	FSIS31801125	Anatum	0	0	0	0	0	0	0	0	0	0	0
SAL_TA0622AA	FSIS21822272	Anatum	0	0	0	0	0	0	0	0	0	0	0
SAL_TA1385AA	FSIS11814633	Anatum	0	0	0	0	0	0	0	0	0	0	0
SAL_TA1372AA	FSIS11814635	Anatum	0	0	0	0	0	0	0	0	0	0	0
SAL_TA1373AA	FSIS11814655	Anatum	0	0	0	0	0	0	0	0	0	0	0
SAL_TA1363AA	FSIS21822249	Anatum	0	0	0	0	0	0	0	0	0	0	0
SAL_TA1257AA	FDA00007863	Anatum	0	0	0	0	0	0	0	0	0	0	0
SAL_TA1258AA	FDA00007879	Anatum	0	0	0	0	0	0	0	0	0	0	0
SAL_TA1255AA	FDA00007862	Anatum	0	0	0	0	0	0	0	0	0	0	0
SAL_TA1416AA	FSIS21822259	Anatum	0	0	0	0	0	0	0	0	0	0	0
SAL_TA1402AA	FSIS11814628	Anatum	0	0	0	0	0	0	0	0	0	0	0
SAL_TA1446AA	FSIS11814890	Anatum	0	0	0	0	0	0	0	0	0	0	0
SAL_TA1447AA	FSIS11814889	Anatum	0	0	0	0	0	0	0	0	0	0	0
SAL_TA1565AA	FSIS21822331	Anatum	0	0	0	0	0	0	0	0	0	0	0
SAL_TA1906AA	FSIS11814869	Anatum	0	0	0	0	0	0	0	0	0	0	0
SAL_TA1908AA	FSIS11814868	Anatum	0	0	0	0	0	0	0	0	0	0	0

SAL_TA1883AA	FSIS11814734	Anatum	0	0	0	0	0	0	0	0	0	0	0
SAL_TA2018AA	FSIS11814888	Anatum	0	0	0	0	0	0	0	0	0	0	0
SAL_TA2007AA	FSIS11814843	Anatum	0	0	0	0	0	0	0	0	0	0	0
SAL_TA1980AA	FC01	Anatum	0	0	0	0	0	0	0	0	0	0	0
SAL_TA1968AA	AC06	Anatum	0	0	0	0	0	0	0	0	0	0	0
SAL_TA1973AA	FC02	Anatum	0	0	0	0	0	0	0	0	0	0	0
SAL_TA2006AA	FSIS11814844	Anatum	0	0	0	0	0	0	0	0	0	0	0
SAL_TA1966AA	AC12	Anatum	0	0	0	0	0	0	0	0	0	0	0
SAL_TA2032AA	CFSAN043217	Anatum	0	0	0	0	0	0	0	0	0	0	0
SAL_TA2102AA	FDA00013354	Anatum	0	0	0	0	0	0	0	0	0	0	0
SAL_TA2103AA	FDA00013355	Anatum	0	0	0	0	0	0	0	0	0	0	0
SAL_TA2181AA	TTU_359	Anatum	0	0	0	0	0	0	0	0	0	0	0
SAL_TA2198AA	FSIS31801164	Anatum	0	0	0	0	0	0	0	0	0	0	0
SAL_TA2318AA	621462	Anatum	0	0	0	0	0	0	0	0	0	0	0
SAL_TA2683AA	FSIS11814825	Anatum	0	0	0	0	0	0	0	0	0	0	0
SAL_TA2611AA	24	Anatum	0	0	0	0	0	0	0	0	0	0	0
SAL_TA2678AA	FSIS11814827	Anatum	0	0	0	0	0	0	0	0	0	0	0
SAL_TA2674AA	FSIS11814823	Anatum	0	0	0	0	0	0	0	0	0	0	0
SAL_TA2580AA	10	Anatum	0	0	0	0	0	0	0	0	0	0	0
SAL_TA2501AA	295	Anatum	0	0	0	0	0	0	0	0	0	0	0
SAL_TA2503AA	297	Anatum	0	0	0	0	0	0	0	0	0	0	0
SAL_TA2502AA	294	Anatum	0	0	0	0	0	0	0	0	0	0	0
SAL_TA2499AA	302	Anatum	0	0	0	0	0	0	0	0	0	0	0
SAL_TA2500AA	301	Anatum	0	0	0	0	0	0	0	0	0	0	0
SAL_TA2497AA	300	Anatum	0	0	0	0	0	0	0	0	0	0	0
SAL_TA2482AA	279	Anatum	0	0	0	0	0	0	0	0	0	0	0
SAL_TA2539AA	272	Anatum	0	0	0	0	0	0	0	0	0	0	0
SAL_TA2540AA	271	Anatum	0	0	0	0	0	0	0	0	0	0	0
SAL_TA2548AA	54	Anatum	0	0	0	0	0	0	0	0	0	0	0
SAL_TA2549AA	55	Anatum	0	0	0	0	0	0	0	0	0	0	0
SAL_TA3590AA	FSIS11815070	Anatum	0	0	0	0	0	0	0	0	0	0	0
SAL_TA3033AA	FSIS11815057	Anatum	0	0	0	0	0	0	0	0	0	0	0
SAL_TA3595AA	FSIS11815081	Anatum	0	0	0	0	0	0	0	0	0	0	0
SAL_TA4187AA	615602	Anatum	0	0	0	0	0	0	0	0	0	0	0
SAL_TA6041AA	FSIS11815271	Anatum	0	0	0	0	0	0	0	0	0	0	0
SAL_TA6094AA	OH-18-20895	Anatum	0	0	0	0	0	0	0	0	0	0	0
SAL_TA6147AA	FSIS11815154	Anatum	0	0	0	0	0	0	0	0	0	0	0
SAL_TA6098AA	OH-18-21102	Anatum	0	0	0	0	0	0	0	0	0	0	0
SAL_TA6070AA	FSIS11815171	Anatum	0	0	0	0	0	0	0	0	0	0	0
SAL_TA6077AA	FSIS11815286	Anatum	0	0	0	0	0	0	0	0	0	0	0
SAL_TA6066AA	FSIS11815291	Anatum	0	0	0	0	0	0	0	0	0	0	0
SAL_TA6419AA	FSIS11815097	Anatum	0	0	0	0	0	0	0	0	0	0	0
SAL_TA6735AA	632967	Anatum	0	0	0	0	0	0	0	0	0	0	0
SAL_TA6801AA	FSIS11815502	Anatum	0	0	0	0	0	0	0	0	0	0	0
SAL_TA6791AA	FSIS11815511	Anatum	0	0	0	0	0	0	0	0	0	0	0
SAL_TA6783AA	FSIS11815513	Anatum	0	0	0	0	0	0	0	0	0	0	0
SAL_TA6787AA	FSIS11815495	Anatum	0	0	0	0	0	0	0	0	0	0	0
SAL_TA6818AA	FDA00013613	Anatum	0	0	0	0	0	0	0	0	0	0	0
SAL_TA6821AA	FDA00013611	Anatum	0	0	0	0	0	0	0	0	0	0	0
SAL_TA6817AA	FDA00013619	Anatum	0	0	0	0	0	0	0	0	0	0	0
SAL_TA6824AA	FDA00013612	Anatum	0	0	0	0	0	0	0	0	0	0	0
SAL_TA6825AA	FDA00013620	Anatum	0	0	0	0	0	0	0	0	0	0	0
SAL_TA7143AA	NC_S775	Anatum	0	0	0	0	0	0	0	0	0	0	0
SAL_TA7145AA	NC_S773	Anatum	0	0	0	0	0	0	0	0	0	0	0
SAL_TA7146AA	CFSAN087634	Anatum	0	0	0	0	0	0	0	0	0	0	0
SAL_TA7240AA	FSIS21822669	Anatum	0	0	0	0	0	0	0	0	0	0	0
SAL_OA2054AA	FSIS11807886	Anatum	0	0	0	0	0	0	0	0	0	0	0
SAL_OA2456AA	MOD1-Lipp-640	Anatum	0	0	0	0	0	0	0	0	0	0	0
SAL_PA2495AA	FSIS21821454	Anatum	0	0	0	0	0	0	0	0	0	0	0
SAL_OA5638AA	FSIS11808305	Anatum	0	0	0	0	0	0	0	0	0	0	0
SAL_PA1661AA	PNUSAS038699	Anatum	0	0	0	0	0	0	0	0	0	0	0
SAL_FA0398AA	FSIS1605547	Anatum	0	0	0	0	0	0	0	0	0	0	0
SAL_FA7788AA	FDA00002695	Anatum	0	0	0	0	0	0	0	0	0	0	0
SAL_FA7785AA	FDA00002703	Anatum	0	0	0	0	0	0	0	0	0	0	0
SAL_FA7791AA	FDA00002697	Anatum	0	0	0	0	0	0	0	0	0	0	0
SAL_FA7787AA	FDA00007884	Anatum	0	0	0	0	0	0	0	0	0	0	0
SAL_TA8083AA	AP-1-VA-WGS-18096	Anatum	0	0	0	0	0	0	0	0	0	0	0
SAL_FA7786AA	FDA00002702	Anatum	0	0	0	0	0	0	0	0	0	0	0
SAL_FA7789AA	FDA00002694	Anatum	0	0	0	0	0	0	0	0	0	0	0
SAL_FA7790AA	FDA00002693	Anatum	0	0	0	0	0	0	0	0	0	0	0
SAL_JA9062AA	PNUSAS013949	Anatum	0	0	0	0	0	0	0	0	0	0	0
SAL_OA1472AA	FSIS11807735	Anatum	0	0	0	0	0	0	0	0	0	0	0
SAL_FA7793AA	FDA00002692	Anatum	0	0	0	0	0	0	0	0	0	0	0

SAL_KA2389AA	FSIS1702334	Anatum	0	0	0	0	0	0	0	0	0	0	0
SAL_KA5557AA	FSIS1703067	Anatum	0	0	0	0	0	0	0	0	0	0	0
SAL_OA1404AA	FSIS11807705	Anatum	0	0	0	0	0	0	0	0	0	0	0
SAL_TA8457AA	636349	Anatum	0	0	0	0	0	0	0	0	0	0	0
SAL_TA8401AA	638747	Anatum	0	0	0	0	0	0	0	0	0	0	0
SAL_TA8268AA	FSIS21822660	Anatum	0	0	0	0	0	0	0	0	0	0	0
SAL_TA8232AA	CFSAN087803	Anatum	0	0	0	0	0	0	0	0	0	0	0
SAL_TA8208AA	ADRDL-2281	Anatum	0	0	0	0	0	0	0	0	0	0	0
SAL_TA8786AA	FSIS11815730	Anatum	0	0	0	0	0	0	0	0	0	0	0
SAL_TA8802AA	FSIS11815723	Anatum	0	0	0	0	0	0	0	0	0	0	0
SAL_TA8836AA	FSIS11815703	Anatum	0	0	0	0	0	0	0	0	0	0	0
SAL_TA8815AA	FSIS11815712	Anatum	0	0	0	0	0	0	0	0	0	0	0
SAL_KA7424AA	PNUSAS018069	Anatum	0	0	0	0	0	0	0	0	0	0	0
SAL_OA3703AA	FSIS21821244	Anatum	0	0	0	0	0	0	0	0	0	0	0
SAL_KA5151AA	FSIS1702976	Anatum	0	0	0	0	0	0	0	0	0	0	0
SAL_RA0022AA	FSIS31800657	Anatum	0	0	0	0	0	0	0	0	0	0	0
SAL_QA5369AA	390046	Anatum	0	0	0	0	0	0	0	0	0	0	0
SAL_QA5590AA	FSIS31800558	Anatum	0	0	0	0	0	0	0	0	0	0	0
SAL_OA1898AA	PNUSAS035180	Anatum	0	0	0	0	0	0	0	0	0	0	0
SAL_OA2462AA	MOD1-31777-Emu	Anatum	0	0	0	0	0	0	0	0	0	0	0
SAL_OA2468AA	MOD1-Lipp-181	Anatum	0	0	0	0	0	0	0	0	0	0	0
SAL_OA5632AA	FSIS11808308	Anatum	0	0	0	0	0	0	0	0	0	0	0
SAL_OA5560AA	PNUSAS036150	Anatum	0	0	0	0	0	0	0	0	0	0	0
SAL_OA5219AA	PNUSAS036040	Anatum	0	0	0	0	0	0	0	0	0	0	0
SAL_OA9822AA	FSIS11808551	Anatum	0	0	0	0	0	0	0	0	0	0	0
SAL_PA0422AA	FSIS11808785	Anatum	0	0	0	0	0	0	0	0	0	0	0
SAL_PA1242AA	TXAML1800312	Anatum	0	0	0	0	0	0	0	0	0	0	0
SAL_PA1400AA	PNUSAS028783	Anatum	0	0	0	0	0	0	0	0	0	0	0
SAL_PA1601AA	FSIS21821421	Anatum	0	0	0	0	0	0	0	0	0	0	0
SAL_PA1699AA	PNUSAS038685	Anatum	0	0	0	0	0	0	0	0	0	0	0
SAL_PA1789AA	FSIS11809071	Anatum	0	0	0	0	0	0	0	0	0	0	0
SAL_PA2346AA	PNUSAS038809	Anatum	0	0	0	0	0	0	0	0	0	0	0
SAL_PA2417AA	PNUSAS038936	Anatum	0	0	0	0	0	0	0	0	0	0	0
SAL_PA2526AA	FSIS11808458	Anatum	0	0	0	0	0	0	0	0	0	0	0
SAL_PA2563AA	FSIS11808490	Anatum	0	0	0	0	0	0	0	0	0	0	0
SAL_PA2562AA	FSIS11807900	Anatum	0	0	0	0	0	0	0	0	0	0	0
SAL_PA2750AA	PNUSAS039211	Anatum	0	0	0	0	0	0	0	0	0	0	0
SAL_PA2780AA	PNUSAS039199	Anatum	0	0	0	0	0	0	0	0	0	0	0
SAL_PA2801AA	PNUSAS039044	Anatum	0	0	0	0	0	0	0	0	0	0	0
SAL_PA3048AA	FSIS11809232	Anatum	0	0	0	0	0	0	0	0	0	0	0
SAL_PA3061AA	PNUSAS035686	Anatum	0	0	0	0	0	0	0	0	0	0	0
SAL_PA3295AA	2015AM-0896	Anatum	0	0	0	0	0	0	0	0	0	0	0
SAL_PA3561AA	CFSAN079983	Anatum	0	0	0	0	0	0	0	0	0	0	0
SAL_PA4101AA	FSIS11809239	Anatum	0	0	0	0	0	0	0	0	0	0	0
SAL_TA9642AA	FDA00013719	Anatum	0	0	0	0	0	0	0	0	0	0	0
SAL_TA9646AA	FSIS21822836	Anatum	0	0	0	0	0	0	0	0	0	0	0
SAL_TA9536AA	FSIS11815918	Anatum	0	0	0	0	0	0	0	0	0	0	0
SAL_TA9781AA	FSIS31801281	Anatum	0	0	0	0	0	0	0	0	0	0	0
SAL_TA9671AA	FDA00013727	Anatum	0	0	0	0	0	0	0	0	0	0	0
SAL_TA9883AA	FSIS11816167	Anatum	0	0	0	0	0	0	0	0	0	0	0
SAL_TA9877AA	FSIS11816143	Anatum	0	0	0	0	0	0	0	0	0	0	0
SAL_TA9884AA	FSIS11816166	Anatum	0	0	0	0	0	0	0	0	0	0	0
SAL_TA9880AA	FSIS11816145	Anatum	0	0	0	0	0	0	0	0	0	0	0
SAL_TA9936AA	FSIS21822847	Anatum	0	0	0	0	0	0	0	0	0	0	0
SAL_TA9985AA	642304	Anatum	0	0	0	0	0	0	0	0	0	0	0
SAL_SA0894AA	R16.0676	Anatum	0	0	0	0	0	0	0	0	0	0	0
SAL_TA8765AA	CFSAN076215	Anatum	0	0	0	0	0	0	0	0	0	0	0
SAL_UA1784AA	CFSAN088513	Anatum	0	0	0	0	0	0	0	0	0	0	0
SAL_UA1766AA	CFSAN088288	Anatum	0	0	0	0	0	0	0	0	0	0	0
SAL_UA2184AA	FSIS11816161	Anatum	0	0	0	0	0	0	0	0	0	0	0
SAL_UA2194AA	FSIS11816163	Anatum	0	0	0	0	0	0	0	0	0	0	0
SAL_UA2192AA	FSIS11816164	Anatum	0	0	0	0	0	0	0	0	0	0	0
SAL_UA2189AA	FSIS11816162	Anatum	0	0	0	0	0	0	0	0	0	0	0
SAL_UA1674AA	TXACF1801914	Anatum	0	0	0	0	0	0	0	0	0	0	0
SAL_UA2778AA	648616	Anatum	0	0	0	0	0	0	0	0	0	0	0
SAL_UA2733AA	647811	Anatum	0	0	0	0	0	0	0	0	0	0	0
SAL_UA2866AA	648973	Anatum	0	0	0	0	0	0	0	0	0	0	0
SAL_UA6310AA	SMG-18-2464	Anatum	0	0	0	0	0	0	0	0	0	0	0
SAL_UA6842AA	CFSAN089921	Anatum	0	0	0	0	0	0	0	0	0	0	0
SAL_UA7234AA	FSIS11816571	Anatum	0	0	0	0	0	0	0	0	0	0	0
SAL_UA3115AA	10_Anatum_cp	Anatum	0	0	0	0	0	0	0	0	0	0	0
SAL_UA3550AA	SAL-18-VL-LA-TX-0011	Anatum	0	0	0	0	0	0	0	0	0	0	0
SAL_UA3742AA	598127	Anatum	0	0	0	0	0	0	0	0	0	0	0

SAL_UA4868AA	CFSAN089123	Anatum	0	0	0	0	0	0	0	0	0	0	0
SAL_UA5115AA	FSIS21822984	Anatum	0	0	0	0	0	0	0	0	0	0	0
SAL_UA5146AA	FSIS11807378	Anatum	0	0	0	0	0	0	0	0	0	0	0
SAL_UA5157AA	FSIS11816327	Anatum	0	0	0	0	0	0	0	0	0	0	0
SAL_UA5174AA	FSIS11816354	Anatum	0	0	0	0	0	0	0	0	0	0	0
SAL_UA5168AA	FSIS11816356	Anatum	0	0	0	0	0	0	0	0	0	0	0
SAL_UA5165AA	FSIS31801330	Anatum	0	0	0	0	0	0	0	0	0	0	0
SAL_UA5240AA	FDA00013750	Anatum	0	0	0	0	0	0	0	0	0	0	0
SAL_UA5238AA	FDA00013749	Anatum	0	0	0	0	0	0	0	0	0	0	0
SAL_UA5405AA	FSIS21823002	Anatum	0	0	0	0	0	0	0	0	0	0	0
SAL_UA5902AA	FSIS11816707	Anatum	0	0	0	0	0	0	0	0	0	0	0
SAL_UA5894AA	FSIS11816705	Anatum	0	0	0	0	0	0	0	0	0	0	0
SAL_UA5937AA	FSIS11816503	Anatum	0	0	0	0	0	0	0	0	0	0	0
SAL_UA5890AA	FSIS11816694	Anatum	0	0	0	0	0	0	0	0	0	0	0
SAL_UA5914AA	FSIS11816691	Anatum	0	0	0	0	0	0	0	0	0	0	0
SAL_UA5910AA	FSIS11816569	Anatum	0	0	0	0	0	0	0	0	0	0	0
SAL_UA6031AA	586091	Anatum	0	0	0	0	0	0	0	0	0	0	0
SAL_UA6180AA	FSIS21823024	Anatum	0	0	0	0	0	0	0	0	0	0	0
SAL_UA6324AA	FSIS11814305	Anatum	0	0	0	0	0	0	0	0	0	0	0
SAL_UA6323AA	FSIS11814309	Anatum	0	0	0	0	0	0	0	0	0	0	0
SAL_UA6425AA	579117	Anatum	0	0	0	0	0	0	0	0	0	0	0
SAL_UA6478AA	FSIS11816923	Anatum	0	0	0	0	0	0	0	0	0	0	0
SAL_UA6964AA	FSIS21823118	Anatum	0	0	0	0	0	0	0	0	0	0	0
SAL_UA7743AA	FSIS11816980	Anatum	0	0	0	0	0	0	0	0	0	0	0
SAL_UA7470AA	664923	Anatum	0	0	0	0	0	0	0	0	0	0	0
SAL_UA7414AA	FSIS31901435	Anatum	0	0	0	0	0	0	0	0	0	0	0
SAL_UA7424AA	FSIS21923222	Anatum	0	0	0	0	0	0	0	0	0	0	0
SAL_UA7805AA	OH-16-4996	Anatum	0	0	0	0	0	0	0	0	0	0	0
SAL_UA8362AA	NC_810120194	Anatum	0	0	0	0	0	0	0	0	0	0	0
SAL_UA8722AA	FSIS11917071	Anatum	0	0	0	0	0	0	0	0	0	0	0
SAL_UA8901AA	AG19-0002	Anatum	0	0	0	0	0	0	0	0	0	0	0
SAL_UA8720AA	FSIS11917075	Anatum	0	0	0	0	0	0	0	0	0	0	0
SAL_UA9644AA	671922	Anatum	0	0	0	0	0	0	0	0	0	0	0
SAL_UA9584AA	623198	Anatum	0	0	0	0	0	0	0	0	0	0	0
SAL_UA8703AA	FSIS11917077	Anatum	0	0	0	0	0	0	0	0	0	0	0
SAL_UA8710AA	FSIS11917078	Anatum	0	0	0	0	0	0	0	0	0	0	0
SAL_UA8714AA	FSIS11917258	Anatum	0	0	0	0	0	0	0	0	0	0	0
SAL_UA9982AA	643088	Anatum	0	0	0	0	0	0	0	0	0	0	0
SAL_VA0228AA	FSIS21923290	Anatum	0	0	0	0	0	0	0	0	0	0	0
SAL_VA0224AA	FSIS21923293	Anatum	0	0	0	0	0	0	0	0	0	0	0
SAL_UA9757AA	637864	Anatum	0	0	0	0	0	0	0	0	0	0	0
SAL_VA0543AA	ADRDL-2241	Anatum	0	0	0	0	0	0	0	0	0	0	0
SAL_VA0888AA	ADRDL-2397	Anatum	0	0	0	0	0	0	0	0	0	0	0
SAL_VA0551AA	FSIS21923318	Anatum	0	0	0	0	0	0	0	0	0	0	0
SAL_VA0880AA	ADRDL-2384	Anatum	0	0	0	0	0	0	0	0	0	0	0
SAL_VA0891AA	ADRDL-2383	Anatum	0	0	0	0	0	0	0	0	0	0	0
SAL_VA1669AA	PNUSAS052484	Anatum	0	0	0	0	0	0	0	0	0	0	0
SAL_VA1592AA	FSIS21923389	Anatum	0	0	0	0	0	0	0	0	0	0	0
SAL_VA3286AA	411459	Anatum	0	0	0	0	0	0	0	0	0	0	0
SAL_VA1286AA	492498	Anatum	0	0	0	0	0	0	0	0	0	0	0
SAL_VA3155AA	FSIS11917338	Anatum	0	0	0	0	0	0	0	0	0	0	0
SAL_VA2940AA	FSIS31901454	Anatum	0	0	0	0	0	0	0	0	0	0	0
SAL_VA3510AA	404898	Anatum	0	0	0	0	0	0	0	0	0	0	0
SAL_VA1777AA	PNUSAS056340	Anatum	0	0	0	0	0	0	0	0	0	0	0
SAL_VA6694AA	BCW_4989	Anatum	0	0	0	0	0	0	0	0	0	0	0
SAL_VA2394AA	HIY0181	Anatum	0	0	0	0	0	0	0	0	0	0	0
SAL_VA2602AA	FDA00013836	Anatum	0	0	0	0	0	0	0	0	0	0	0
SAL_VA2608AA	FSIS21923415	Anatum	0	0	0	0	0	0	0	0	0	0	0
SAL_VA2620AA	FSIS21923421	Anatum	0	0	0	0	0	0	0	0	0	0	0
SAL_VA2652AA	FSIS11917383	Anatum	0	0	0	0	0	0	0	0	0	0	0
SAL_VA2643AA	FSIS11917381	Anatum	0	0	0	0	0	0	0	0	0	0	0
SAL_VA1687AA	PNUSAS051002	Anatum	0	0	0	0	0	0	0	0	0	0	0
SAL_VA3343AA	AG19-0041	Anatum	0	0	0	0	0	0	0	0	0	0	0
SAL_VA3346AA	AG19-0036	Anatum	0	0	0	0	0	0	0	0	0	0	0
SAL_VA3323AA	AG19-0055	Anatum	0	0	0	0	0	0	0	0	0	0	0
SAL_VA6462AA	CFSAN031886	Anatum	0	0	0	0	0	0	0	0	0	0	0
SAL_VA5873AA	SA20030572	Anatum	0	0	0	0	0	0	0	0	0	0	0
SAL_VA5727AA	SA20102810	Anatum	0	0	0	0	0	0	0	0	0	0	0
SAL_VA5706AA	SA20100278	Anatum	0	0	0	0	0	0	0	0	0	0	0
SAL_VA5336AA	LJH#1217	Anatum	0	0	0	0	0	0	0	0	0	0	0
SAL_VA5337AA	LJH#720	Anatum	0	0	0	0	0	0	0	0	0	0	0
SAL_VA5365AA	345	Anatum	0	0	0	0	0	0	0	0	0	0	0
SAL_VA6790AA	FSIS11917530	Anatum	0	0	0	0	0	0	0	0	0	0	0

SAL_VA6961AA	FSIS31901559	Anatum	0	0	0	0	0	0	0	0	0	0	0
SAL_VA6839AA	HIY0183	Anatum	0	0	0	0	0	0	0	0	0	0	0
SAL_VA6844AA	HIY0187	Anatum	0	0	0	0	0	0	0	0	0	0	0
SAL_VA4495AA	402781	Anatum	0	0	0	0	0	0	0	0	0	0	0
SAL_VA4501AA	681317	Anatum	0	0	0	0	0	0	0	0	0	0	0
SAL_VA4427AA	681316	Anatum	0	0	0	0	0	0	0	0	0	0	0
SAL_VA4346AA	391290	Anatum	0	0	0	0	0	0	0	0	0	0	0
SAL_VA7017AA	FSIS21923533	Anatum	0	0	0	0	0	0	0	0	0	0	0
SAL_VA7016AA	FSIS21923505	Anatum	0	0	0	0	0	0	0	0	0	0	0
SAL_VA7219AA	406060	Anatum	0	0	0	0	0	0	0	0	0	0	0
SAL_VA7713AA	FSIS11917566	Anatum	0	0	0	0	0	0	0	0	0	0	0
SAL_VA7709AA	FSIS11917577	Anatum	0	0	0	0	0	0	0	0	0	0	0
SAL_VA7706AA	FSIS11917586	Anatum	0	0	0	0	0	0	0	0	0	0	0
SAL_VA7252AA	FSIS31901541	Anatum	0	0	0	0	0	0	0	0	0	0	0
SAL_VA7742AA	FSIS11917522	Anatum	0	0	0	0	0	0	0	0	0	0	0
SAL_VA7755AA	FSIS11917601	Anatum	0	0	0	0	0	0	0	0	0	0	0
SAL_VA7727AA	FSIS11917554	Anatum	0	0	0	0	0	0	0	0	0	0	0
SAL_VA7744AA	FSIS11917540	Anatum	0	0	0	0	0	0	0	0	0	0	0
SAL_VA7835AA	FSIS11917768	Anatum	0	0	0	0	0	0	0	0	0	0	0
SAL_VA7857AA	FSIS11917800	Anatum	0	0	0	0	0	0	0	0	0	0	0
SAL_VA7966AA	FSIS11917759	Anatum	0	0	0	0	0	0	0	0	0	0	0
SAL_VA8000AA	FSIS11917788	Anatum	0	0	0	0	0	0	0	0	0	0	0
SAL_VA8009AA	FSIS11917581	Anatum	0	0	0	0	0	0	0	0	0	0	0
SAL_VA7790AA	S765	Anatum	0	0	0	0	0	0	0	0	0	0	0
SAL_VA8058AA	FSIS21923502	Anatum	0	0	0	0	0	0	0	0	0	0	0
SAL_VA8011AA	FSIS11917783	Anatum	0	0	0	0	0	0	0	0	0	0	0
SAL_VA7858AA	FSIS11917775	Anatum	0	0	0	0	0	0	0	0	0	0	0
SAL_VA8300AA	392944	Anatum	0	0	0	0	0	0	0	0	0	0	0
SAL_VA8277AA	686618	Anatum	0	0	0	0	0	0	0	0	0	0	0
SAL_VA6020AA	CFS0056	Anatum	0	0	0	0	0	0	0	0	0	0	0
SAL_VA8092AA	FSIS31901593	Anatum	0	0	0	0	0	0	0	0	0	0	0
SAL_VA8615AA	FSIS11918006	Anatum	0	0	0	0	0	0	0	0	0	0	0
SAL_VA4267AA	CDPH_CSalm230	Anatum	0	0	0	0	0	0	0	0	0	0	0
SAL_VA8673AA	320942	Anatum	0	0	0	0	0	0	0	0	0	0	0
SAL_VA8625AA	FSIS21923621	Anatum	0	0	0	0	0	0	0	0	0	0	0
SAL_VA9006AA	299448	Anatum	0	0	0	0	0	0	0	0	0	0	0
SAL_VA9275AA	P8S734	Anatum	0	0	0	0	0	0	0	0	0	0	0
SAL_VA9249AA	DAC30	Anatum	0	0	0	0	0	0	0	0	0	0	0
SAL_VA4164AA	CDPH_CSalm028	Anatum	0	0	0	0	0	0	0	0	0	0	0
SAL_VA4163AA	CDPH_CSalm027	Anatum	0	0	0	0	0	0	0	0	0	0	0
SAL_VA9521AA	280340	Anatum	0	0	0	0	0	0	0	0	0	0	0
SAL_VA9539AA	273594	Anatum	0	0	0	0	0	0	0	0	0	0	0
SAL_VA9499AA	267017	Anatum	0	0	0	0	0	0	0	0	0	0	0
SAL_VA9658AA	249362	Anatum	0	0	0	0	0	0	0	0	0	0	0
SAL_VA9853AA	FSIS11918036	Anatum	0	0	0	0	0	0	0	0	0	0	0
SAL_VA9866AA	FSIS31901647	Anatum	0	0	0	0	0	0	0	0	0	0	0
SAL_WA0079AA	FSIS11918146	Anatum	0	0	0	0	0	0	0	0	0	0	0
SAL_WA0084AA	FSIS11918138	Anatum	0	0	0	0	0	0	0	0	0	0	0
SAL_WA0098AA	FSIS11918140	Anatum	0	0	0	0	0	0	0	0	0	0	0
SAL_WA0108AA	FSIS11918149	Anatum	0	0	0	0	0	0	0	0	0	0	0
SAL_WA0105AA	FSIS11918144	Anatum	0	0	0	0	0	0	0	0	0	0	0
SAL_VA9863AA	FSIS31901648	Anatum	0	0	0	0	0	0	0	0	0	0	0
SAL_WA0067AA	FSIS11918145	Anatum	0	0	0	0	0	0	0	0	0	0	0
SAL_WA0078AA	FSIS11918135	Anatum	0	0	0	0	0	0	0	0	0	0	0
SAL_WA0074AA	FSIS11918150	Anatum	0	0	0	0	0	0	0	0	0	0	0
SAL_WA0089AA	FSIS11918152	Anatum	0	0	0	0	0	0	0	0	0	0	0
SAL_WA0073AA	FSIS11918139	Anatum	0	0	0	0	0	0	0	0	0	0	0
SAL_WA0081AA	FSIS11918339	Anatum	0	0	0	0	0	0	0	0	0	0	0
SAL_WA0101AA	FSIS11918142	Anatum	0	0	0	0	0	0	0	0	0	0	0
SAL_WA0237AA	208944	Anatum	0	0	0	0	0	0	0	0	0	0	0
SAL_WA0050AA	FDA00013952	Anatum	0	0	0	0	0	0	0	0	0	0	0
SAL_WA0048AA	FDA00013953	Anatum	0	0	0	0	0	0	0	0	0	0	0
SAL_WA0140AA	207531	Anatum	0	0	0	0	0	0	0	0	0	0	0
SAL_WA0495AA	183664	Anatum	0	0	0	0	0	0	0	0	0	0	0
SAL_WA0466AA	194252	Anatum	0	0	0	0	0	0	0	0	0	0	0
SAL_WA0492AA	192911	Anatum	0	0	0	0	0	0	0	0	0	0	0
SAL_WA0617AA	FSIS11918579	Anatum	0	0	0	0	0	0	0	0	0	0	0
SAL_WA0668AA	FSIS11918667	Anatum	0	0	0	0	0	0	0	0	0	0	0
SAL_WA0746AA	182149	Anatum	0	0	0	0	0	0	0	0	0	0	0
SAL_WA0809AA	171299	Anatum	0	0	0	0	0	0	0	0	0	0	0
SAL_VA8129AA	363503	Anatum	0	0	0	0	0	0	0	0	0	0	0
SAL_WA1100AA	FSIS11918685	Anatum	0	0	0	0	0	0	0	0	0	0	0
SAL_VA4112AA	CDPH_CSalm320	Anatum	0	0	0	0	0	0	0	0	0	0	0

SAL_WA0769AA	160798	Anatum	0	0	0	0	0	0	0	0	0	0	0
SAL_WA3215AA	CIT_D2.18	Anatum	0	0	0	0	0	0	0	0	0	0	0
SAL_WA3126AA	CK_93/00001031	Anatum	0	0	0	0	0	0	0	0	0	0	0
SAL_WA3065AA	NL_H13	Anatum	0	0	0	0	0	0	0	0	0	0	0
SAL_WA2824AA	CK_93/00004822	Anatum	0	0	0	0	0	0	0	0	0	0	0
SAL_WA2645AA	CIT_D76	Anatum	0	0	0	0	0	0	0	0	0	0	0
SAL_WA2640AA	NL_P16	Anatum	0	0	0	0	0	0	0	0	0	0	0
SAL_WA2641AA	NL_P15	Anatum	0	0	0	0	0	0	0	0	0	0	0
SAL_WA2560AA	NL_P13	Anatum	0	0	0	0	0	0	0	0	0	0	0
SAL_WA2536AA	CK_07/00001464-2	Anatum	0	0	0	0	0	0	0	0	0	0	0
SAL_WA2481AA	NL_L20	Anatum	0	0	0	0	0	0	0	0	0	0	0
SAL_WA2423AA	04ba 6789-3	Anatum	0	0	0	0	0	0	0	0	0	0	0
SAL_WA1230AA	143956	Anatum	0	0	0	0	0	0	0	0	0	0	0
SAL_WA1478AA	FSIS11918936	Anatum	0	0	0	0	0	0	0	0	0	0	0
SAL_WA1416AA	120632	Anatum	0	0	0	0	0	0	0	0	0	0	0
SAL_WA1580AA	FSIS11918939	Anatum	0	0	0	0	0	0	0	0	0	0	0
SAL_WA1582AA	FSIS11918932	Anatum	0	0	0	0	0	0	0	0	0	0	0
SAL_WA1503AA	119443	Anatum	0	0	0	0	0	0	0	0	0	0	0
SAL_WA1796AA	FSIS21923709	Anatum	0	0	0	0	0	0	0	0	0	0	0
SAL_WA1849AA	FSIS11918528	Anatum	0	0	0	0	0	0	0	0	0	0	0
SAL_WA1949AA	FSIS11918968	Anatum	0	0	0	0	0	0	0	0	0	0	0
SAL_WA2025AA	FDA00014059	Anatum	0	0	0	0	0	0	0	0	0	0	0
SAL_WA2098AA	AG19-0118	Anatum	0	0	0	0	0	0	0	0	0	0	0
SAL_WA2125AA	P7S733	Anatum	0	0	0	0	0	0	0	0	0	0	0
SAL_WA2160AA	FSIS21923697	Anatum	0	0	0	0	0	0	0	0	0	0	0
SAL_WA2134AA	FSIS11919170	Anatum	0	0	0	0	0	0	0	0	0	0	0
SAL_WA2021AA	FDA00014063	Anatum	0	0	0	0	0	0	0	0	0	0	0
SAL_WA2019AA	FDA00014061	Anatum	0	0	0	0	0	0	0	0	0	0	0
SAL_WA3482AA	CFSAN092332	Anatum	0	0	0	0	0	0	0	0	0	0	0
SAL_WA3699AA	CFSAN084246	Anatum	0	0	0	0	0	0	0	0	0	0	0
SAL_WA3661AA	CFSAN087491	Anatum	0	0	0	0	0	0	0	0	0	0	0
SAL_WA3638AA	CFSAN087476	Anatum	0	0	0	0	0	0	0	0	0	0	0
SAL_WA3806AA	FSIS11918521	Anatum	0	0	0	0	0	0	0	0	0	0	0
SAL_WA3809AA	FSIS11918683	Anatum	0	0	0	0	0	0	0	0	0	0	0
SAL_WA4055AA	FSIS11919420	Anatum	0	0	0	0	0	0	0	0	0	0	0
SAL_WA4050AA	FSIS11919298	Anatum	0	0	0	0	0	0	0	0	0	0	0
SAL_WA4047AA	FSIS11919424	Anatum	0	0	0	0	0	0	0	0	0	0	0
SAL_WA3988AA	FSIS11919214	Anatum	0	0	0	0	0	0	0	0	0	0	0
SAL_VA8084AA	NYVetLIRN-128	Anatum	0	0	0	0	0	0	0	0	0	0	0
SAL_VA9059AA	FDA00013908	Anatum	0	0	0	0	0	0	0	0	0	0	0
SAL_WA4249AA	712475	Anatum	0	0	0	0	0	0	0	0	0	0	0
SAL_PA1166AA	031618-09_3934-6	Anderlecht	0	0	0	0	0	0	0	0	0	0	0
SAL_PA1184AA	031618-09_3934-7	Anderlecht	0	0	0	0	0	0	0	0	0	0	0
SAL_PA1192AA	031618-09_3934-3	Anderlecht	0	0	0	0	0	0	0	0	0	0	0
SAL_BA1304AA	FDA00002254	Anfo	0	0	0	0	0	0	0	0	0	0	0
SAL_BA1767AA	FDA00002255	Anfo	0	0	0	0	0	0	0	0	0	0	0
SAL_VA2596AA	SAL1857	Anfo	0	0	0	0	0	0	0	0	0	0	0
SAL_VA2597AA	SAL1464	Anfo	0	0	0	0	0	0	0	0	0	0	0
SAL_VA2595AA	SAL1340	Anfo	0	0	0	0	0	0	0	0	0	0	0
SAL_VA2594AA	SAL1461	Anfo	0	0	0	0	0	0	0	0	0	0	0
SAL_VA2593AA	SAL1462	Anfo	0	0	0	0	0	0	0	0	0	0	0
SAL_VA2592AA	SAL1463	Anfo	0	0	0	0	0	0	0	0	0	0	0
SAL_VA4221AA	CDPH_CSalm354	Anfo	0	0	0	0	0	0	0	0	0	0	0
SAL_QA0331AA	353922	Angoda	0	0	0	0	0	0	0	0	0	0	0
SAL_VA0288AA	539071	Angoda	0	0	0	0	0	0	0	0	0	0	0
SAL_HA5598AA	S05898-15	Ank	0	0	0	0	0	0	0	0	0	0	0
SAL_GA3357AA	BCW_2640	Ank	0	0	0	0	0	0	0	0	0	0	0
SAL_OA7765AA	PNUSAS037243	Ank	0	0	0	0	0	0	0	0	0	0	0
SAL_WA3434AA	702860	Ank	0	0	0	0	0	0	0	0	0	0	0
SAL_EA3486AA	BS_03452-sc-2322327	Antsalova	0	0	0	0	0	0	0	0	0	0	0
SAL_JA9305AA	PNUSAS014382	Antsalova	0	0	0	0	0	0	0	0	0	0	0
SAL_MA6076AA	S01-0511	Antsalova	0	0	0	0	0	0	0	0	0	0	0
SAL_TA2913AA	616198	Antsalova	0	0	0	0	0	0	0	0	0	0	0
SAL_EA3954AA	FSIS1504701	Apapa	0	0	0	0	0	0	0	0	0	0	0
SAL_FA1476AA	PNUSAS001616	Apapa	0	0	0	0	0	0	0	0	0	0	0
SAL_EA0701AA	FAR0004	Apapa	0	0	0	0	0	0	0	0	0	0	0
SAL_DA6134AA	MOD1_Sal_836	Apapa	0	0	0	0	0	0	0	0	0	0	0
SAL_DA0986AA	H121720311	Apapa	0	0	0	0	0	0	0	0	0	0	0
SAL_CA4558AA	89881	Apapa	0	0	0	0	0	0	0	0	0	0	0
SAL_CA2376AA	27055	Apapa	0	0	0	0	0	0	0	0	0	0	0
SAL_BA9217AA	WAPHL_SAL-A00023	Apapa	0	0	0	0	0	0	0	0	0	0	0
SAL_BA9223AA	39464	Apapa	0	0	0	0	0	0	0	0	0	0	0
SAL_BA3729AA	FDA00009149	Apapa	0	0	0	0	0	0	0	0	0	0	0

SAL_FA6443AA	PNUSAS001703	Apapa	0	0	0	0	0	0	0	0	0	0	0
SAL_HA5284AA	PNUSAS003710	Apapa	0	0	0	0	0	0	0	0	0	0	0
SAL_IA7379AA	PNUSAS008448	Apapa	0	0	0	0	0	0	0	0	0	0	0
SAL_JA0565AA	PNUSAS009740	Apapa	0	0	0	0	0	0	0	0	0	0	0
SAL_JA4971AA	PNUSAS013533	Apapa	0	0	0	0	0	0	0	0	0	0	0
SAL_JA6520AA	361819	Apapa	0	0	0	0	0	0	0	0	0	0	0
SAL_JA7131AA	PNUSAS013424	Apapa	0	0	0	0	0	0	0	0	0	0	0
SAL_KA6735AA	PNUSAS019194	Apapa	0	0	0	0	0	0	0	0	0	0	0
SAL_KA8260AA	PNUSAS019616	Apapa	0	0	0	0	0	0	0	0	0	0	0
SAL_LA2202AA	PNUSAS020729	Apapa	0	0	0	0	0	0	0	0	0	0	0
SAL_LA3843AA	PNUSAS022302	Apapa	0	0	0	0	0	0	0	0	0	0	0
SAL_MA5239AA	27-9-8101121	Apapa	0	0	0	0	0	0	0	0	0	0	0
SAL_DA1149AA	H124120638	Apapa	0	0	0	0	0	0	0	0	0	0	0
SAL_GA2339AA	BCW_2641	Apapa	0	0	0	0	0	0	0	0	0	0	0
SAL_IA5917AA	MS160389	Apapa	0	0	0	0	0	0	0	0	0	0	0
SAL_MA6072AA	SA20060561	Apapa	0	0	0	0	0	0	0	0	0	0	0
SAL_MA6804AA	FDA00004892	Apapa	0	0	0	0	0	0	0	0	0	0	0
SAL_MA8918AA	PNUSAS028989	Apapa	0	0	0	0	0	0	0	0	0	0	0
SAL_MA8835AA	FSIS11705509	Apapa	0	0	0	0	0	0	0	0	0	0	0
SAL_NA1787AA	PNUSAS029297	Apapa	0	0	0	0	0	0	0	0	0	0	0
SAL_NA4590AA	2014AM-1997	Apapa	0	0	0	0	0	0	0	0	0	0	0
SAL_NA9423AA	PNUSAS032522	Apapa	0	0	0	0	0	0	0	0	0	0	0
SAL_PA5414AA	PNUSAS040264	Apapa	0	0	0	0	0	0	0	0	0	0	0
SAL_PA6461AA	PNUSAS040535	Apapa	0	0	0	0	0	0	0	0	0	0	0
SAL_QA0763AA	PNUSAS042112	Apapa	0	0	0	0	0	0	0	0	0	0	0
SAL_QA5198AA	155631	Apapa	0	0	0	0	0	0	0	0	0	0	0
SAL_QA8458AA	PNUSAS043927	Apapa	0	0	0	0	0	0	0	0	0	0	0
SAL_SA5340AA	CK_99/00008108	Apapa	0	0	0	0	0	0	0	0	0	0	0
SAL_SA5336AA	CK_99/00005530	Apapa	0	0	0	0	0	0	0	0	0	0	0
SAL_SA5335AA	CK_99/00004332	Apapa	0	0	0	0	0	0	0	0	0	0	0
SAL_SA5351AA	CK_00/00000531	Apapa	0	0	0	0	0	0	0	0	0	0	0
SAL_SA5292AA	CK_98/00004117	Apapa	0	0	0	0	0	0	0	0	0	0	0
SAL_OA0861AA	I2018001940-1	Apapa	0	0	0	0	0	0	0	0	0	0	0
SAL_PA2577AA	PNUSAS039005	Apapa	0	0	0	0	0	0	0	0	0	0	0
SAL_PA2594AA	PNUSAS036492	Apapa	0	0	0	0	0	0	0	0	0	0	0
SAL_UA4481AA	OH-15-14896	Apapa	0	0	0	0	0	0	0	0	0	0	0
SAL_VA3209AA	495789	Apapa	0	0	0	0	0	0	0	0	0	0	0
SAL_DA7809AA	FMA0128	Apeyeme	0	0	0	0	0	0	0	0	0	0	0
SAL_IA1058AA	287581	Apeyeme	0	0	0	0	0	0	0	0	0	0	0
SAL_IA5620AA	PNUSAS007811	Apeyeme	0	0	0	0	0	0	0	0	0	0	0
SAL_IA8824AA	PNUSAS008847	Apeyeme	0	0	0	0	0	0	0	0	0	0	0
SAL_JA1607AA	17010013	Apeyeme	0	0	0	0	0	0	0	0	0	0	0
SAL_JA6846AA	340173	Apeyeme	0	0	0	0	0	0	0	0	0	0	0
SAL_JA8432AA	346613	Apeyeme	0	0	0	0	0	0	0	0	0	0	0
SAL_KA0448AA	PNUSAS014020	Apeyeme	0	0	0	0	0	0	0	0	0	0	0
SAL_KA8838AA	17019963	Apeyeme	0	0	0	0	0	0	0	0	0	0	0
SAL_MA2565AA	SSI-AC393	Apeyeme	0	0	0	0	0	0	0	0	0	0	0
SAL_DA9653AA	FSF0065	Apeyeme	0	0	0	0	0	0	0	0	0	0	0
SAL_IA2659AA	CFSAN059211	Apeyeme	0	0	0	0	0	0	0	0	0	0	0
SAL_NA2368AA	CFSAN073485	Apeyeme	0	0	0	0	0	0	0	0	0	0	0
SAL_OA5377AA	PNUSAS035392	Apeyeme	0	0	0	0	0	0	0	0	0	0	0
SAL_QA7720AA	318573	Apeyeme	0	0	0	0	0	0	0	0	0	0	0
SAL_QA9797AA	475837	Apeyeme	0	0	0	0	0	0	0	0	0	0	0
SAL_RA3757AA	248523	Apeyeme	0	0	0	0	0	0	0	0	0	0	0
SAL_VA2280AA	467149	Apeyeme	0	0	0	0	0	0	0	0	0	0	0
SAL_VA6918AA	379337	Apeyeme	0	0	0	0	0	0	0	0	0	0	0
SAL_VA9744AA	264020	Apeyeme	0	0	0	0	0	0	0	0	0	0	0
SAL_WA0794AA	161072	Apeyeme	0	0	0	0	0	0	0	0	0	0	0
SAL_VA4063AA	CDPH_CSalm372	Apeyeme	0	0	0	0	0	0	0	0	0	0	0
SAL_WA3466AA	708225	Apeyeme	0	0	0	0	0	0	0	0	0	0	0
SAL_WA4268AA	712243	Apeyeme	0	0	0	0	0	0	0	0	0	0	0
SAL_GA6250AA	VUPR	Aqua	0	0	0	0	0	0	0	0	0	0	0
SAL_GA2819AA	CRJJGF_00092	Aqua	0	0	0	0	0	0	0	0	0	0	0
SAL_UA5338AA	650060	Aqua	0	0	0	0	0	0	0	0	0	0	0
SAL_WA2535AA	CK_07/00001464-1	Aqua	0	0	0	0	0	0	0	0	0	0	0
SAL_DA8257AA	0036-sc-2010-09-29-985	Arechavaleta	0	0	0	0	0	0	0	0	0	0	0
SAL_BA7847AA	08_3567	Arechavaleta	0	0	0	0	0	0	0	0	0	0	0
SAL_FA4222AA	117305	Arechavaleta	0	0	0	0	0	0	0	0	0	0	0
SAL_DA8265AA	669426-sc-2010-09-29-9	Arechavaleta	0	0	0	0	0	0	0	0	0	0	0
SAL_DA8266AA	060423-sc-2010-09-29-9	Arechavaleta	0	0	0	0	0	0	0	0	0	0	0
SAL_DA6098AA	MOD1_Sal_837	Arechavaleta	0	0	0	0	0	0	0	0	0	0	0
SAL_DA0753AA	H123640762	Arechavaleta	0	0	0	0	0	0	0	0	0	0	0
SAL_DA0463AA	H124440676	Arechavaleta	0	0	0	0	0	0	0	0	0	0	0

SAL_CA5483AA	73618	Arechavaleta	0	0	0	0	0	0	0	0	0	0	0
SAL_CA4660AA	75932	Arechavaleta	0	0	0	0	0	0	0	0	0	0	0
SAL_BA1190AA	CFSAN035077	Arechavaleta	0	0	0	0	0	0	0	0	0	0	0
SAL_FA6417AA	PNUSAS002111	Arechavaleta	0	0	0	0	0	0	0	0	0	0	0
SAL_FA6426AA	PNUSAS001914	Arechavaleta	0	0	0	0	0	0	0	0	0	0	0
SAL_FA7969AA	PNUSAS002172	Arechavaleta	0	0	0	0	0	0	0	0	0	0	0
SAL_GA4725AA	FDA00010381	Arechavaleta	2	2	3	0	0	0	0	0	0	0	0
SAL_FA8489AA	PNUSAS002184	Arechavaleta	0	0	0	0	0	0	0	0	0	0	0
SAL_GA9385AA	PNUSAS003042	Arechavaleta	0	0	0	0	0	0	0	0	0	0	0
SAL_IA1300AA	285974	Arechavaleta	0	0	0	0	0	0	0	0	0	0	0
SAL_IA3568AA	121465	Arechavaleta	0	0	0	0	0	0	0	0	0	0	0
SAL_IA6502AA	PNUSAS008109	Arechavaleta	0	0	0	0	0	0	0	0	0	0	0
SAL_IA7162AA	ADRDL-930	Arechavaleta	0	0	0	0	0	0	0	0	0	0	0
SAL_JA0389AA	PNUSAS010386	Arechavaleta	0	0	0	0	0	0	0	0	0	0	0
SAL_JA2096AA	ADRDL-987	Arechavaleta	0	0	0	0	0	0	0	0	0	0	0
SAL_LA0095AA	18-16-20170667	Arechavaleta	0	0	0	0	0	0	0	0	0	0	0
SAL_LA2619AA	131K	Arechavaleta	0	0	0	0	0	0	0	0	0	0	0
SAL_LA4421AA	PNUSAS022492	Arechavaleta	0	0	0	0	0	0	0	0	0	0	0
SAL_MA0100AA	PNUSAS023371	Arechavaleta	0	0	0	0	0	0	0	0	0	0	0
SAL_MA0101AA	PNUSAS023369	Arechavaleta	0	0	0	0	0	0	0	0	0	0	0
SAL_BA0053AA	KeN140	Arechavaleta	2	2	3	3	0	0	0	0	0	0	0
SAL_FA7220AA	PNUSAS001975	Arechavaleta	0	0	0	0	0	0	0	0	0	0	0
SAL_IA2617AA	PNUSAS007752	Arechavaleta	0	0	0	0	0	0	0	0	0	0	0
SAL_JA2505AA	PNUSAS010404	Arechavaleta	0	0	0	0	0	0	0	0	0	0	0
SAL_JA9303AA	PNUSAS014726	Arechavaleta	0	0	0	0	0	0	0	0	0	0	0
SAL_KA1133AA	2015AM-1537	Arechavaleta	0	0	0	0	0	0	0	0	0	0	0
SAL_MA6898AA	ADRDL-1588	Arechavaleta	0	0	0	0	0	0	0	0	0	0	0
SAL_MA6899AA	ADRDL-1599	Arechavaleta	0	0	0	0	0	0	0	0	0	0	0
SAL_NA5955AA	PNUSAS029417	Arechavaleta	0	0	0	0	0	0	0	0	0	0	0
SAL_NA7420AA	2015AM-3479	Arechavaleta	0	0	0	0	0	0	0	0	0	0	0
SAL_NA8689AA	PNUSAS032778	Arechavaleta	0	0	0	0	0	0	0	0	0	0	0
SAL_NA9745AA	PNUSAS033948	Arechavaleta	0	0	0	0	0	0	0	0	0	0	0
SAL_QA2131AA	166201	Arechavaleta	0	0	0	0	0	0	0	0	0	0	0
SAL_QA4506AA	PNUSAS037973	Arechavaleta	0	0	0	0	0	0	0	0	0	0	0
SAL_QA6292AA	538432	Arechavaleta	2	2	3	3	0	0	0	0	0	0	0
SAL_QA7679AA	296651	Arechavaleta	0	0	0	0	0	0	0	0	0	0	0
SAL_QA8334AA	133988	Arechavaleta	0	0	0	0	0	0	0	0	0	0	0
SAL_RA0375AA	420622	Arechavaleta	0	0	0	0	0	0	0	0	0	0	0
SAL_RA1099AA	128217	Arechavaleta	0	0	0	0	0	0	0	0	0	0	0
SAL_RA0772AA	333922	Arechavaleta	0	0	0	0	0	0	0	0	0	0	0
SAL_SA1439AA	S/19981349	Arechavaleta	0	0	0	0	0	0	0	0	0	0	0
SAL_SA1440AA	S/19990610	Arechavaleta	0	0	0	0	0	0	0	0	0	0	0
SAL_RA6562AA	180798	Arechavaleta	0	0	0	0	0	0	0	0	0	0	0
SAL_RA8079AA	353860	Arechavaleta	0	0	0	0	0	0	0	0	0	0	0
SAL_SA3214AA		Arechavaleta	0	0	0	0	0	0	0	0	0	0	0
SAL_SA7729AA	576286	Arechavaleta	0	0	0	0	0	0	0	0	0	0	0
SAL_TA6530AA	631575	Arechavaleta	0	0	0	0	0	0	0	0	0	0	0
SAL_OA2439AA	MOD1-Tw-229b	Arechavaleta	0	0	0	0	0	0	0	0	0	0	0
SAL_OA1945AA	PNUSAS035013	Arechavaleta	0	0	0	0	0	0	0	0	0	0	0
SAL_WA0587AA	187592	Arechavaleta	0	0	0	0	0	0	0	0	0	0	0
SAL_WA1229AA	130508	Arechavaleta	0	0	0	0	0	0	0	0	0	0	0
SAL_WA1435AA	126210	Arechavaleta	0	0	0	0	0	0	0	0	0	0	0
SAL_DA2432AA	FDA00004073	Aschersleben	0	0	0	0	0	0	0	0	0	0	0
SAL_EA0714AA	FSW0021	Aschersleben	0	0	0	0	0	0	0	0	0	0	0
SAL_VA0368AA	548519	Aschersleben	0	0	0	0	0	0	0	0	0	0	0
SAL_DA0096AA	FDA00003669	Astridplein	0	0	0	0	0	0	0	0	0	0	0
SAL_BA2680AA	FDA00001341	Augustenborg	0	0	0	0	0	0	0	0	0	0	0
SAL_CA7220AA	FDA00001219	Augustenborg	0	0	0	0	0	0	0	0	0	0	0
SAL_FA4374AA	136765	Augustenborg	0	0	0	0	0	0	0	0	0	0	0
SAL_FA1344AA	FDA00004308	Augustenborg	0	0	0	0	0	0	0	0	0	0	0
SAL_DA9131AA	FNE0077	Augustenborg	0	0	0	0	0	0	0	0	0	0	0
SAL_DA8559AA	FSE0079	Augustenborg	0	0	0	0	0	0	0	0	0	0	0
SAL_DA6607AA	FMA0185	Augustenborg	0	0	0	0	0	0	0	0	0	0	0
SAL_DA6081AA	MOD1_Sal_838	Augustenborg	0	0	0	0	0	0	0	0	0	0	0
SAL_DA3962AA	FNE0150	Augustenborg	0	0	0	0	0	0	0	0	0	0	0
SAL_DA1067AA	H132720101	Augustenborg	0	0	0	0	0	0	0	0	0	0	0
SAL_DA0891AA	H122400363	Augustenborg	0	0	0	0	0	0	0	0	0	0	0
SAL_CA8434AA	FDA00000910	Augustenborg	0	0	0	0	0	0	0	0	0	0	0
SAL_CA7891AA	FDA00003005	Augustenborg	0	0	0	0	0	0	0	0	0	0	0
SAL_CA7355AA	FDA00003324	Augustenborg	0	0	0	0	0	0	0	0	0	0	0
SAL_CA7250AA	FDA00001030	Augustenborg	0	0	0	0	0	0	0	0	0	0	0
SAL_CA2352AA	25220	Augustenborg	0	0	0	0	0	0	0	0	0	0	0
SAL_GA3585AA	BCW_2732	Augustenborg	0	0	0	0	0	0	0	0	0	0	0

SAL_GA3356AA	BCW_2642	Augustenborg	0	0	0	0	0	0	0	0	0	0	0
SAL_IA2680AA	FDA00011056	Augustenborg	0	0	0	0	0	0	0	0	0	0	0
SAL_IA7827AA	FDA00011287	Augustenborg	0	0	0	0	0	0	0	0	0	0	0
SAL_BA5613AA	FSF0179	Augustenborg	0	0	0	0	0	0	0	0	0	0	0
SAL_FA4197AA	170459	Augustenborg	0	0	0	0	0	0	0	0	0	0	0
SAL_OA1536AA	FDA00012555	Augustenborg	0	0	0	0	0	0	0	0	0	0	0
SAL_OA4673AA	PNUSAS025487	Augustenborg	0	0	0	0	0	0	0	0	0	0	0
SAL_PA5768AA	136762	Augustenborg	0	0	0	0	0	0	0	0	0	0	0
SAL_QA2129AA	310198	Augustenborg	0	0	0	0	0	0	0	0	0	0	0
SAL_QA5277AA	158974	Augustenborg	0	0	0	0	0	0	0	0	0	0	0
SAL_QA7843AA	302513	Augustenborg	0	0	0	0	0	0	0	0	0	0	0
SAL_RA1437AA	FDA00013261	Augustenborg	0	0	0	0	0	0	0	0	0	0	0
SAL_RA1994AA	FDA00013260	Augustenborg	0	0	0	0	0	0	0	0	0	0	0
SAL_RA7842AA	376212	Augustenborg	0	0	0	0	0	0	0	0	0	0	0
SAL_SA4609AA	FDA00013428	Augustenborg	0	0	0	0	0	0	0	0	0	0	0
SAL_SA8649AA	158972	Augustenborg	0	0	0	0	0	0	0	0	0	0	0
SAL_VA0849AA	553271	Augustenborg	0	0	0	0	0	0	0	0	0	0	0
SAL_WA0172AA	247198	Augustenborg	0	0	0	0	0	0	0	0	0	0	0
SAL_WA0901AA	159906	Augustenborg	0	0	0	0	0	0	0	0	0	0	0
SAL_WA3700AA	FDA00014075	Augustenborg	0	0	0	0	0	0	0	0	0	0	0
SAL_VA7532AA	201705659	Ayinde	0	0	0	0	0	0	0	0	0	0	0
SAL_JA8726AA	PNUSAS014613	Azteca	0	0	0	0	0	0	0	0	0	0	0
SAL_DA9041AA	FNE0006	Baguida	0	0	0	0	0	0	0	0	0	0	0
SAL_DA5056AA	FNE0095	Baguida	0	0	0	0	0	0	0	0	0	0	0
SAL_CA5520AA	79110	Baildon	1	1	1	4	1	1	1	1	1	1	1
SAL_BA3360AA	PNUSAS000422	Baildon	0	0	0	0	0	0	0	0	0	0	0
SAL_FA5218AA	CFSAN047699	Baildon	0	0	0	0	0	0	0	0	0	0	0
SAL_FA5217AA	CFSAN047700	Baildon	0	0	0	0	0	0	0	0	0	0	0
SAL_FA5216AA	CFSAN047701	Baildon	0	0	0	0	0	0	0	0	0	0	0
SAL_FA5209AA	CFSAN047698	Baildon	0	0	0	0	0	0	0	0	0	0	0
SAL_FA5065AA	PNUSA001867	Baildon	0	0	0	0	0	0	0	0	0	0	0
SAL_FA1098AA	PNUSAS001525	Baildon	0	0	0	0	0	0	0	0	0	0	0
SAL_EA0138AA	NY_IDR1100011916	Baildon	0	0	0	0	0	0	0	0	0	0	0
SAL_DA8697AA	FL-MDD-75	Baildon	0	0	0	0	0	0	0	0	0	0	0
SAL_DA6100AA	FLDOH-38	Baildon	0	0	0	0	0	0	0	0	0	0	0
SAL_DA6084AA	MOD1_Sal_839	Baildon	0	0	0	0	0	0	0	0	0	0	0
SAL_DA5488AA	FLUFL-872	Baildon	0	0	0	0	0	0	0	0	0	0	0
SAL_CA9201AA	FLUFL-874	Baildon	0	0	0	0	0	0	0	0	0	0	0
SAL_CA9189AA	FLUFL-868	Baildon	0	0	0	0	0	0	0	0	0	0	0
SAL_CA8116AA	PNUSAS000161	Baildon	0	0	0	0	0	0	0	0	0	0	0
SAL_CA8119AA	PNUSAS000158	Baildon	0	0	0	0	0	0	0	0	0	0	0
SAL_CA8117AA	PNUSAS000160	Baildon	0	0	0	0	0	0	0	0	0	0	0
SAL_CA8120AA	PNUSAS000157	Baildon	0	0	0	0	0	0	0	0	0	0	0
SAL_CA8118AA	PNUSAS000159	Baildon	0	0	0	0	0	0	0	0	0	0	0
SAL_CA7578AA	2015K-0085	Baildon	0	0	0	0	0	0	0	0	0	0	0
SAL_CA7579AA	2015K-0086	Baildon	0	0	0	0	0	0	0	0	0	0	0
SAL_CA7576AA	2015K-0088	Baildon	0	0	0	0	0	0	0	0	0	0	0
SAL_CA7575AA	2015K-0089	Baildon	0	0	0	0	0	0	0	0	0	0	0
SAL_CA7395AA	PNUSAS000164	Baildon	0	0	0	0	0	0	0	0	0	0	0
SAL_CA7396AA	PNUSAS000163	Baildon	0	0	0	0	0	0	0	0	0	0	0
SAL_BA8717AA	NY_FSL S10-975	Baildon	0	0	0	0	0	0	0	0	0	0	0
SAL_BA8271AA	FL_FLDACS-09260	Baildon	0	0	0	0	0	0	0	0	0	0	0
SAL_BA3724AA	2015K-0129	Baildon	0	0	0	0	0	0	0	0	0	0	0
SAL_BA3092AA	PNUSAS000423	Baildon	0	0	0	0	0	0	0	0	0	0	0
SAL_BA3091AA	PNUSAS000424	Baildon	0	0	0	0	0	0	0	0	0	0	0
SAL_BA2329AA	2015K-0438	Baildon	0	0	0	0	0	0	0	0	0	0	0
SAL_BA2327AA	2015K-0440	Baildon	0	0	0	0	0	0	0	0	0	0	0
SAL_BA2128AA	2015K-0439	Baildon	0	0	0	0	0	0	0	0	0	0	0
SAL_BA2121AA	2015K-0467	Baildon	0	0	0	0	0	0	0	0	0	0	0
SAL_BA2122AA	2015K-0466	Baildon	0	0	0	0	0	0	0	0	0	0	0
SAL_FA7954AA	PNUSAS002447	Baildon	0	0	0	0	0	0	0	0	0	0	0
SAL_FA8161AA	PNUSAS002320	Baildon	0	0	0	0	0	0	0	0	0	0	0
SAL_GA2562AA	08-1674	Baildon	0	0	0	0	0	0	0	0	0	0	0
SAL_GA2557AA	2010K-1384	Baildon	0	0	0	0	0	0	0	0	0	0	0
SAL_GA2551AA	96-0126	Baildon	0	0	0	0	0	0	0	0	0	0	0
SAL_GA2525AA	88-0456	Baildon	0	0	0	0	0	0	0	0	0	0	0
SAL_GA2524AA	91-0202	Baildon	0	0	0	0	0	0	0	0	0	0	0
SAL_GA4763AA	PNUSAS001665	Baildon	0	0	0	0	0	0	0	0	0	0	0
SAL_GA5078AA	PNUSAS002652	Baildon	0	0	0	0	0	0	0	0	0	0	0
SAL_GA7516AA	PNUSAS003335	Baildon	0	0	0	0	0	0	0	0	0	0	0
SAL_GA9410AA	PNUSAS003707	Baildon	0	0	0	0	0	0	0	0	0	0	0
SAL_HA0965AA	PNUSAS004575	Baildon	0	0	0	0	0	0	0	0	0	0	0
SAL_HA8263AA	PNUSAS006483	Baildon	0	0	0	0	0	0	0	0	0	0	0

SAL_IA0437AA	PNUSAS007238	Baildon	0	0	0	0	0	0	0	0	0	0	0
SAL_IA4643AA	PNUSAS008063	Baildon	0	0	0	0	0	0	0	0	0	0	0
SAL_IA4960AA	CFSAN059280	Baildon	0	0	0	0	0	0	0	0	0	0	0
SAL_IA5306AA	NSUOC-0205-W5H	Baildon	0	0	0	0	0	0	0	0	0	0	0
SAL_IA5339AA	FSIS1710096	Baildon	0	0	0	0	0	0	0	0	0	0	0
SAL_IA6321AA	PNUSAS004338	Baildon	0	0	0	0	0	0	0	0	0	0	0
SAL_IA6532AA	PNUSAS008740	Baildon	0	0	0	0	0	0	0	0	0	0	0
SAL_IA8802AA	NSUOC-0247-W3H	Baildon	0	0	0	0	0	0	0	0	0	0	0
SAL_IA8803AA	NSUOC-0246-W3	Baildon	0	0	0	0	0	0	0	0	0	0	0
SAL_IA9415AA	PNUSAS009691	Baildon	0	0	0	0	0	0	0	0	0	0	0
SAL_IA9122AA	PNUSAS009514	Baildon	0	0	0	0	0	0	0	0	0	0	0
SAL_IA9585AA	PNUSAS009363	Baildon	0	0	0	0	0	0	0	0	0	0	0
SAL_JA0207AA	PNUSAS010596	Baildon	0	0	0	0	0	0	0	0	0	0	0
SAL_JA0310AA	267	Baildon	0	0	0	0	0	0	0	0	0	0	0
SAL_JA0313AA	263	Baildon	0	0	0	0	0	0	0	0	0	0	0
SAL_JA0305AA	262	Baildon	0	0	0	0	0	0	0	0	0	0	0
SAL_JA0304AA	NSUOC-0266-S2-2H	Baildon	0	0	0	0	0	0	0	0	0	0	0
SAL_JA1809AA	PNUSAS011237	Baildon	0	0	0	0	0	0	0	0	0	0	0
SAL_JA2005AA	PNUSAS011530	Baildon	0	0	0	0	0	0	0	0	0	0	0
SAL_JA0416AA	PNUSAS010648	Baildon	0	0	0	0	0	0	0	0	0	0	0
SAL_JA2623AA	PNUSAS012059	Baildon	0	0	0	0	0	0	0	0	0	0	0
SAL_JA2451AA	PNUSAS011354	Baildon	0	0	0	0	0	0	0	0	0	0	0
SAL_JA3551AA	PNUSAS012587	Baildon	0	0	0	0	0	0	0	0	0	0	0
SAL_JA3549AA	PNUSAS012578	Baildon	0	0	0	0	0	0	0	0	0	0	0
SAL_JA3927AA	PNUSAS011971	Baildon	0	0	0	0	0	0	0	0	0	0	0
SAL_JA3924AA	PNUSAS011902	Baildon	0	0	0	0	0	0	0	0	0	0	0
SAL_JA6509AA	PNUSAS013685	Baildon	0	0	0	0	0	0	0	0	0	0	0
SAL_JA6011AA	PNUSAS013329	Baildon	0	0	0	0	0	0	0	0	0	0	0
SAL_JA5330AA	PNUSAS013135	Baildon	0	0	0	0	0	0	0	0	0	0	0
SAL_JA6822AA	PNUSAS008776	Baildon	0	0	0	0	0	0	0	0	0	0	0
SAL_JA7139AA	PNUSAS012915	Baildon	0	0	0	0	0	0	0	0	0	0	0
SAL_JA7137AA	PNUSAS013897	Baildon	0	0	0	0	0	0	0	0	0	0	0
SAL_JA9526AA	NSUOC-0327-S3-2X	Baildon	0	0	0	0	0	0	0	0	0	0	0
SAL_JA9535AA	NSUOC-0192-W1X	Baildon	0	0	0	0	0	0	0	0	0	0	0
SAL_JA9552AA	NSUOC-0315-W2B	Baildon	0	0	0	0	0	0	0	0	0	0	0
SAL_JA9558AA	NSUOC-0317-W3X	Baildon	0	0	0	0	0	0	0	0	0	0	0
SAL_JA9563AA	NSUOC-0329-S3-1B	Baildon	0	0	0	0	0	0	0	0	0	0	0
SAL_JA9591AA	NSUOC-0371-S5-2X	Baildon	0	0	0	0	0	0	0	0	0	0	0
SAL_JA9324AA	PNUSAS012584	Baildon	0	0	0	0	0	0	0	0	0	0	0
SAL_JA9325AA	PNUSAS010174	Baildon	0	0	0	0	0	0	0	0	0	0	0
SAL_KA0639AA	2015AM-0005	Baildon	0	0	0	0	0	0	0	0	0	0	0
SAL_KA0401AA	PNUSAS015703	Baildon	0	0	0	0	0	0	0	0	0	0	0
SAL_KA0337AA	PNUSAS016151	Baildon	0	0	0	0	0	0	0	0	0	0	0
SAL_KA0339AA	PNUSAS016150	Baildon	0	0	0	0	0	0	0	0	0	0	0
SAL_KA0597AA	2015AM-1417	Baildon	0	0	0	0	0	0	0	0	0	0	0
SAL_KA1150AA	2015AM-0768	Baildon	0	0	0	0	0	0	0	0	0	0	0
SAL_KA2930AA	PNUSAS016908	Baildon	0	0	0	0	0	0	0	0	0	0	0
SAL_KA3263AA	PNUSAS017820	Baildon	0	0	0	0	0	0	0	0	0	0	0
SAL_KA3628AA	PNUSAS017246	Baildon	0	0	0	0	0	0	0	0	0	0	0
SAL_KA4837AA	PNUSAS017036	Baildon	0	0	0	0	0	0	0	0	0	0	0
SAL_KA4763AA	PNUSAS017697	Baildon	0	0	0	0	0	0	0	0	0	0	0
SAL_KA5498AA	PNUSAS018867	Baildon	0	0	0	0	0	0	0	0	0	0	0
SAL_KA5494AA	PNUSAS017643	Baildon	0	0	0	0	0	0	0	0	0	0	0
SAL_KA5310AA	PNUSAS016546	Baildon	0	0	0	0	0	0	0	0	0	0	0
SAL_KA5888AA	PNUSAS018932	Baildon	0	0	0	0	0	0	0	0	0	0	0
SAL_KA7584AA	PNUSAS018309	Baildon	0	0	0	0	0	0	0	0	0	0	0
SAL_LA0196AA	2015AM-0275	Baildon	0	0	0	0	0	0	0	0	0	0	0
SAL_LA1325AA	NSUOC-0193-W1H	Baildon	0	0	0	0	0	0	0	0	0	0	0
SAL_LA6409AA	PNUSAS023666	Baildon	0	0	0	0	0	0	0	0	0	0	0
SAL_MA1023AA	PNUSAS025904	Baildon	0	0	0	0	0	0	0	0	0	0	0
SAL_MA4689AA	PNUSAS027260	Baildon	0	0	0	0	0	0	0	0	0	0	0
SAL_EA5427AA	CFSAN041501	Baildon	0	0	0	0	0	0	0	0	0	0	0
SAL_CA7577AA	2015K-0087	Baildon	0	0	0	0	0	0	0	0	0	0	0
SAL_CA7394AA	PNUSAS000165	Baildon	0	0	0	0	0	0	0	0	0	0	0
SAL_GA2543AA	03-0685	Baildon	1	1	1	4	1	1	1	1	1	1	1
SAL_JA7031AA	PNUSAS014464	Baildon	0	0	0	0	0	0	0	0	0	0	0
SAL_MA6199AA	PNUSAS027836	Baildon	0	0	0	0	0	0	0	0	0	0	0
SAL_MA7457AA	NSUOC-0200-W3X	Baildon	0	0	0	0	0	0	0	0	0	0	0
SAL_MA8950AA	PNUSAS028993	Baildon	0	0	0	0	0	0	0	0	0	0	0
SAL_NA1972AA	PNUSAS030020	Baildon	0	0	0	0	0	0	0	0	0	0	0
SAL_NA2524AA	PNUSAS030019	Baildon	0	0	0	0	0	0	0	0	0	0	0
SAL_NA2833AA	PNUSAS030076	Baildon	0	0	0	0	0	0	0	0	0	0	0
SAL_NA2666AA	PNUSAS027067	Baildon	0	0	0	0	0	0	0	0	0	0	0

SAL_NA3540AA	PNUSAS031004	Baildon	0	0	0	0	0	0	0	0	0	0	0
SAL_NA4952AA	PNUSAS031487	Baildon	0	0	0	0	0	0	0	0	0	0	0
SAL_OA5871AA	PNUSAS034911	Baildon	0	0	0	0	0	0	0	0	0	0	0
SAL_OA5753AA	PNUSAS033887	Baildon	0	0	0	0	0	0	0	0	0	0	0
SAL_OA7432AA	PNUSAS036846	Baildon	0	0	0	0	0	0	0	0	0	0	0
SAL_OA5211AA	PNUSAS036287	Baildon	0	0	0	0	0	0	0	0	0	0	0
SAL_OA8149AA	PNUSAS036911	Baildon	0	0	0	0	0	0	0	0	0	0	0
SAL_OA8298AA	PNUSAS036856	Baildon	0	0	0	0	0	0	0	0	0	0	0
SAL_PA1653AA	PNUSAS038333	Baildon	0	0	0	0	0	0	0	0	0	0	0
SAL_PA1684AA	PNUSAS038682	Baildon	0	0	0	0	0	0	0	0	0	0	0
SAL_PA6035AA	PNUSAS040569	Baildon	0	0	0	0	0	0	0	0	0	0	0
SAL_PA6325AA	PNUSAS040574	Baildon	0	0	0	0	0	0	0	0	0	0	0
SAL_PA9170AA	PNUSAS041443	Baildon	0	0	0	0	0	0	0	0	0	0	0
SAL_PA9752AA	PNUSAS041644	Baildon	0	0	0	0	0	0	0	0	0	0	0
SAL_QA3009AA	PNUSAS043088	Baildon	0	0	0	0	0	0	0	0	0	0	0
SAL_QA4104AA	443564	Baildon	0	0	0	0	0	0	0	0	0	0	0
SAL_QA6397AA	TTU_063	Baildon	0	0	0	0	0	0	0	0	0	0	0
SAL_QA6378AA	TTU_057	Baildon	0	0	0	0	0	0	0	0	0	0	0
SAL_QA6643AA	TTU_064	Baildon	0	0	0	0	0	0	0	0	0	0	0
SAL_QA6678AA	TTU_056	Baildon	0	0	0	0	0	0	0	0	0	0	0
SAL_RA0196AA	130361	Baildon	0	0	0	0	0	0	0	0	0	0	0
SAL_RA3846AA	TTU_065	Baildon	0	0	0	0	0	0	0	0	0	0	0
SAL_RA3843AA	TTU_055	Baildon	0	0	0	0	0	0	0	0	0	0	0
SAL_RA6357AA	PNUSAS044930	Baildon	0	0	0	0	0	0	0	0	0	0	0
SAL_OA2330AA	PNUSAS033478	Baildon	0	0	0	0	0	0	0	0	0	0	0
SAL_JA8751AA	PNUSAS015005	Baildon	0	0	0	0	0	0	0	0	0	0	0
SAL_OA3721AA	MOD1-Dr-1	Baildon	0	0	0	0	0	0	0	0	0	0	0
SAL_OA1377AA	PNUSAS031698	Baildon	0	0	0	0	0	0	0	0	0	0	0
SAL_OA2016AA	PNUSAS035448	Baildon	0	0	0	0	0	0	0	0	0	0	0
SAL_PA3927AA	PNUSAS038941	Baildon	0	0	0	0	0	0	0	0	0	0	0
SAL_VA1691AA	PNUSAS050995	Baildon	0	0	0	0	0	0	0	0	0	0	0
SAL_VA6023AA	227	Baildon	0	0	0	0	0	0	0	0	0	0	0
SAL_WA0863AA	152417	Baildon	0	0	0	0	0	0	0	0	0	0	0
SAL_FA4577AA	199917	Bakau	0	0	0	0	0	0	0	0	0	0	0
SAL_EA0219AA	FNE0011	Ball	0	0	0	0	0	0	0	0	0	0	0
SAL_DA5818AA	MOD1_Sal_840	Ball	0	0	0	0	0	0	0	0	0	0	0
SAL_DA3454AA	FNE0177	Ball	0	0	0	0	0	0	0	0	0	0	0
SAL_EA7830AA	19659	Ball	0	0	0	0	0	0	0	0	0	0	0
SAL_FA8476AA	PNUSAS002191	Ball	0	0	0	0	0	0	0	0	0	0	0
SAL_IA0933AA	168333	Ball	0	0	0	0	0	0	0	0	0	0	0
SAL_KA8588AA	PNUSAS018678	Ball	0	0	0	0	0	0	0	0	0	0	0
SAL_RA5850AA	PNUSAS044629	Ball	0	0	0	0	0	0	0	0	0	0	0
SAL_KA4197AA	100 TIR-9	Bamboye	0	0	0	0	0	0	0	0	0	0	0
SAL_DA4364AA	FSF0134	Bangkok	0	0	0	0	0	0	0	0	0	0	0
SAL_GA5981AA	FDA00002990	Bangkok	0	0	0	0	0	0	0	0	0	0	0
SAL_DA2348AA	FNW19a83	Bangkok	0	0	0	0	0	0	0	0	0	0	0
SAL_DA4237AA	FDN0204	Bangkok	0	0	0	0	0	0	0	0	0	0	0
SAL_DA0132AA	FDA00000781	Bangkok	0	0	0	0	0	0	0	0	0	0	0
SAL_EA2864AA	FDA00001845	Bangkok	0	0	0	0	0	0	0	0	0	0	0
SAL_EA8672AA	VA-WGS-00652	Bareilly	0	0	0	0	0	0	0	0	0	0	0
SAL_DA4558AA	9505/04	Bareilly	0	0	0	0	0	0	0	0	0	16	0
SAL_DA0032AA	2014K-0523	Bareilly	0	0	0	0	0	0	0	0	0	0	0
SAL_EA5708AA	MOD1-Dem-033	Bareilly	0	0	0	0	0	0	0	0	0	0	0
SAL_DA8598AA	FSE0069	Bareilly	0	0	0	0	0	0	0	0	0	0	0
SAL_DA7808AA	FSW0132	Bareilly	0	0	0	0	0	0	0	0	0	0	0
SAL_BA7754AA	31910	Bareilly	0	0	0	0	0	0	0	0	0	0	0
SAL_BA7418AA	2015K-0187	Bareilly	0	0	0	0	0	0	0	0	0	0	0
SAL_DA8711AA	FL-MDD-76	Bareilly	0	0	0	0	0	0	0	0	0	0	0
SAL_EA0347AA	FL-UFL-458	Bareilly	0	0	0	0	0	0	0	0	0	0	0
SAL_EA8154AA	CFSAN034510	Bareilly	0	0	0	0	0	0	0	0	0	0	0
SAL_FA5186AA	FDA00003946	Bareilly	0	0	0	0	0	0	0	0	0	0	0
SAL_FA5174AA	CFSAN000224	Bareilly	0	0	0	0	0	0	0	0	0	0	0
SAL_FA5185AA	FDA00003945	Bareilly	0	0	0	0	0	0	0	0	0	0	0
SAL_FA5173AA	CFSAN001089	Bareilly	0	0	0	0	0	0	0	0	0	0	0
SAL_FA5175AA	CFSAN000201	Bareilly	0	0	0	0	0	0	0	0	0	0	0
SAL_FA4866AA	FDA00007814	Bareilly	0	0	0	0	0	0	0	0	0	0	0
SAL_FA4865AA	FDA00007816	Bareilly	0	0	0	0	0	0	0	0	0	0	0
SAL_FA4711AA	107181	Bareilly	0	0	0	0	0	0	0	0	0	0	0
SAL_FA4693AA	187505	Bareilly	0	0	0	0	0	0	0	0	0	0	0
SAL_FA4582AA	112991	Bareilly	0	0	0	0	0	0	0	0	0	0	0
SAL_FA4535AA	211722	Bareilly	0	0	0	0	0	0	0	0	0	0	0
SAL_FA4466AA	175565	Bareilly	0	0	0	0	0	0	0	0	0	0	0
SAL_FA4433AA	211708	Bareilly	0	0	0	0	0	0	0	0	0	0	0

SAL_FA4252AA	170317	Bareilly	0	0	0	0	0	0	0	0	0	0	0
SAL_FA4138AA	122206	Bareilly	0	0	0	0	0	0	0	0	0	0	0
SAL_FA4099AA	181996	Bareilly	0	0	0	0	0	0	0	0	0	0	0
SAL_FA4070AA	179826	Bareilly	0	0	0	0	0	0	0	0	0	0	0
SAL_FA2592AA	PNUSAS001780	Bareilly	0	0	0	0	0	0	0	0	0	0	0
SAL_FA2591AA	PNUSAS001781	Bareilly	0	0	0	0	0	0	0	0	0	0	0
SAL_FA1930AA	PNUSAS001661	Bareilly	0	0	0	0	0	0	0	0	0	0	0
SAL_FA1915AA	2016K-0111	Bareilly	0	0	0	0	0	0	0	0	0	0	0
SAL_FA1693AA	CVM-N18596	Bareilly	0	0	0	0	0	0	0	0	0	0	0
SAL_FA1680AA	2016K-0116	Bareilly	0	0	0	0	0	0	0	0	0	0	0
SAL_FA1535AA	CVM-N18601	Bareilly	0	0	0	0	0	0	0	0	0	0	0
SAL_FA1531AA	CVM-N18605	Bareilly	0	0	0	0	0	0	0	0	0	0	0
SAL_FA1525AA	CVM-N18611	Bareilly	0	0	0	0	0	0	0	0	0	0	0
SAL_FA1368AA	CVM-N18580	Bareilly	0	0	0	0	0	0	0	0	0	0	0
SAL_FA1369AA	CVM-N18581	Bareilly	0	0	0	0	0	0	0	0	0	0	0
SAL_EA9605AA	PNUSAS001342	Bareilly	0	0	0	0	0	0	0	0	0	0	0
SAL_EA8678AA	VA-WGS-00646	Bareilly	0	0	0	0	0	0	0	0	0	0	0
SAL_EA8149AA	CFSAN034506	Bareilly	0	0	0	0	0	0	0	0	0	0	0
SAL_EA8027AA	63747	Bareilly	0	0	0	0	0	0	0	0	0	0	0
SAL_EA7974AA	23479	Bareilly	0	0	0	0	0	0	0	0	0	0	0
SAL_EA7841AA	21874	Bareilly	0	0	0	0	0	0	0	0	0	0	0
SAL_EA7835AA	1840	Bareilly	0	0	0	0	0	0	0	0	0	0	0
SAL_EA7691AA	81995	Bareilly	0	0	0	0	0	0	0	0	0	0	0
SAL_EA7598AA	23478	Bareilly	0	0	0	0	0	0	0	0	0	0	0
SAL_EA7494AA	99076	Bareilly	0	0	0	0	0	0	0	0	0	0	0
SAL_EA7241AA	1865	Bareilly	0	0	0	0	0	0	0	0	0	0	0
SAL_EA6999AA	9126	Bareilly	0	0	0	0	0	0	0	0	0	0	0
SAL_EA6955AA	1857	Bareilly	0	0	0	0	0	0	0	0	0	0	0
SAL_EA6721AA	FDA00005050	Bareilly	0	0	0	0	0	0	0	0	0	0	0
SAL_EA6706AA	FDA00009675	Bareilly	0	0	0	0	0	0	0	0	0	0	0
SAL_EA6617AA	2015K-1428	Bareilly	0	0	0	0	0	0	0	0	0	0	0
SAL_EA6284AA	MOD1-Lipp-605	Bareilly	0	0	0	0	0	0	0	0	0	0	0
SAL_EA5866AA	CFSAN034377	Bareilly	0	0	0	0	0	0	0	0	0	0	0
SAL_EA5559AA	MDH-2014-00728	Bareilly	0	0	0	0	0	0	0	0	0	0	0
SAL_EA5536AA	MOD1-Lipp-011	Bareilly	0	0	0	0	0	0	0	0	0	0	0
SAL_EA5538AA	MOD1-Jwm-25	Bareilly	0	0	0	0	0	0	0	0	0	0	0
SAL_EA4630AA	IEH-NGS-SAL-03062	Bareilly	0	0	0	0	0	0	0	0	0	0	0
SAL_EA4631AA	CFSAN033250	Bareilly	0	0	0	0	0	0	0	0	0	0	0
SAL_EA4624AA	CFSAN033257	Bareilly	0	0	0	0	0	0	0	0	0	0	0
SAL_EA4273AA	FDA00009607	Bareilly	0	0	0	0	0	0	0	0	0	0	0
SAL_EA3970AA	FDA00004279	Bareilly	0	0	0	0	0	0	0	0	0	0	0
SAL_EA3234AA	CFSAN000199	Bareilly	0	0	0	0	0	0	0	0	0	0	0
SAL_EA3219AA	CFSAN000230	Bareilly	0	0	0	0	0	0	0	0	0	0	0
SAL_EA3140AA	MD12-715530	Bareilly	0	0	0	0	0	0	0	0	0	0	0
SAL_EA3138AA	MD12-715532	Bareilly	0	0	0	0	0	0	0	0	0	0	0
SAL_EA3136AA	MD12-715535	Bareilly	0	0	0	0	0	0	0	0	0	0	0
SAL_EA3133AA	MD12-715570	Bareilly	0	0	0	0	0	0	0	0	0	0	0
SAL_EA3135AA	MD12-715534	Bareilly	0	0	0	0	0	0	0	0	0	0	0
SAL_EA3137AA	MD12-715533	Bareilly	0	0	0	0	0	0	0	0	0	0	0
SAL_EA3139AA	MD12-715531	Bareilly	0	0	0	0	0	0	0	0	0	0	0
SAL_EA3134AA	MD12-715569	Bareilly	0	0	0	0	0	0	0	0	0	0	0
SAL_EA2886AA	FDA00001862	Bareilly	0	0	0	0	0	0	0	0	0	0	0
SAL_EA2474AA	CFSAN000198	Bareilly	0	0	0	0	0	0	0	0	0	0	0
SAL_EA2456AA	FDA00009340	Bareilly	0	0	0	0	0	0	0	0	0	0	0
SAL_EA1367AA	FL_MJ469	Bareilly	0	0	0	0	0	0	0	0	0	0	0
SAL_EA0583AA	FL_FLDACS-22982	Bareilly	0	0	0	0	0	0	0	0	0	0	0
SAL_EA0539AA	MOD1_SALC_127	Bareilly	0	0	0	0	0	0	0	0	0	0	0
SAL_EA0382AA	NY_198076001-10	Bareilly	0	0	0	0	0	0	0	0	0	0	0
SAL_EA0200AA	FL-UFL-101	Bareilly	0	0	0	0	0	0	0	0	0	0	0
SAL_EA0052AA	FL-MJ684	Bareilly	0	0	0	0	0	0	0	0	0	0	0
SAL_DA9690AA	VA_WGS-00062	Bareilly	0	0	0	0	0	0	0	0	0	0	0
SAL_DA9344AA	FNW19H79	Bareilly	0	0	0	0	0	0	0	0	0	0	0
SAL_DA8893AA	UFL-647	Bareilly	0	0	0	0	0	0	0	0	0	0	0
SAL_DA8639AA	CFSAN003834	Bareilly	0	0	0	0	0	0	0	0	0	0	0
SAL_DA8423AA	VA_WGS-00151	Bareilly	0	0	0	0	0	0	0	0	0	0	0
SAL_DA8432AA	VA_WGS-00135	Bareilly	0	0	0	0	0	0	0	0	0	0	0
SAL_DA8067AA	FSW0106	Bareilly	0	0	0	0	0	0	0	0	0	0	0
SAL_DA8043AA	CFSAN001108	Bareilly	0	0	0	0	0	0	0	0	0	0	0
SAL_DA8040AA	CFSAN001140	Bareilly	0	0	0	0	0	0	0	0	0	0	0
SAL_DA8038AA	CFSAN001115	Bareilly	0	0	0	0	0	0	0	0	0	0	0
SAL_DA8009AA	FMA0104	Bareilly	0	0	0	0	0	0	0	0	0	0	0
SAL_DA7860AA	FAR0125	Bareilly	0	0	0	0	0	0	0	0	0	0	0
SAL_DA7820AA	FSW0129	Bareilly	0	0	0	0	0	0	0	0	0	0	0

SAL_DA7785AA	FMA0125	Bareilly	0	0	0	0	0	0	0	0	0	0	0
SAL_DA7517AA	FLUFL-829	Bareilly	0	0	0	0	0	0	0	0	0	0	0
SAL_DA7472AA	FLUFL-836	Bareilly	0	0	0	0	0	0	0	0	0	0	0
SAL_DA7280AA	FLUFL-1059	Bareilly	0	0	0	0	0	0	0	0	0	0	0
SAL_DA6789AA	CFSAN000968	Bareilly	0	0	0	0	0	0	0	0	0	0	0
SAL_DA6511AA	CFSAN017960	Bareilly	0	0	0	0	0	0	0	0	0	0	0
SAL_DA6399AA	CFSAN017962	Bareilly	0	0	0	0	0	0	0	0	0	0	0
SAL_DA6308AA	FCC0129	Bareilly	0	0	0	0	0	0	0	0	0	0	0
SAL_DA6290AA	FNE0108	Bareilly	0	0	0	0	0	0	0	0	0	0	0
SAL_DA5876AA	CFSAN008844	Bareilly	0	0	0	0	0	0	0	0	0	0	0
SAL_DA5877AA	CFSAN008855	Bareilly	0	0	0	0	0	0	0	0	0	0	0
SAL_DA5867AA	CFSAN008854	Bareilly	0	0	0	0	0	0	0	0	0	0	0
SAL_DA5879AA	CFSAN008853	Bareilly	0	0	0	0	0	0	0	0	0	0	0
SAL_DA5866AA	CFSAN008852	Bareilly	0	0	0	0	0	0	0	0	0	0	0
SAL_DA5598AA	FDA00004210	Bareilly	0	0	0	0	0	0	0	0	0	0	0
SAL_DA5273AA	FSF0129	Bareilly	0	0	0	0	0	0	0	0	0	0	0
SAL_DA5264AA	FSF0106	Bareilly	0	0	0	0	0	0	0	0	0	0	0
SAL_DA4532AA	E698	Bareilly	0	0	0	0	0	0	0	0	0	0	0
SAL_DA3741AA	CFSAN008882	Bareilly	0	0	0	0	0	0	0	0	0	0	0
SAL_DA3739AA	CFSAN008920	Bareilly	0	0	0	0	0	0	0	0	0	0	0
SAL_DA3720AA	CFSAN008884	Bareilly	0	0	0	0	0	0	0	0	0	0	0
SAL_DA3685AA	CFSAN008859	Bareilly	0	0	0	0	0	0	0	0	0	0	0
SAL_DA3676AA	CFSAN008858	Bareilly	0	0	0	0	0	0	0	0	0	0	0
SAL_DA3653AA	CFSAN008885	Bareilly	0	0	0	0	0	0	0	0	0	0	0
SAL_DA3635AA	CFSAN008887	Bareilly	0	0	0	0	0	0	0	0	0	0	0
SAL_DA3648AA	CFSAN008918	Bareilly	0	0	0	0	0	0	0	0	0	0	0
SAL_DA3605AA	CFSAN008881	Bareilly	0	0	0	0	0	0	0	0	0	0	0
SAL_DA3600AA	CFSAN008883	Bareilly	0	0	0	0	0	0	0	0	0	0	0
SAL_DA3604AA	CFSAN008919	Bareilly	0	0	0	0	0	0	0	0	0	0	0
SAL_DA3595AA	CFSAN008886	Bareilly	0	0	0	0	0	0	0	0	0	0	0
SAL_DA3524AA	FDA00008182	Bareilly	0	0	0	0	0	0	0	0	0	0	0
SAL_DA3461AA	FNE0161	Bareilly	0	0	0	0	0	0	0	0	0	0	0
SAL_DA3124AA	CFSAN024162	Bareilly	0	0	0	0	0	0	0	0	0	0	0
SAL_DA3119AA	CFSAN024159	Bareilly	0	0	0	0	0	0	0	0	0	0	0
SAL_DA3116AA	CFSAN024219	Bareilly	0	0	0	0	0	0	0	0	0	0	0
SAL_DA3099AA	CFSAN024158	Bareilly	0	0	0	0	0	0	0	0	0	0	0
SAL_DA3101AA	CFSAN024232	Bareilly	0	0	0	0	0	0	0	0	0	0	0
SAL_DA3110AA	CFSAN024207	Bareilly	0	0	0	0	0	0	0	0	0	0	0
SAL_DA3082AA	CFSAN024140	Bareilly	0	0	0	0	0	0	0	0	0	0	0
SAL_DA3083AA	CFSAN024218	Bareilly	0	0	0	0	0	0	0	0	0	0	0
SAL_DA3085AA	CFSAN024141	Bareilly	0	0	0	0	0	0	0	0	0	0	0
SAL_DA3070AA	CFSAN024233	Bareilly	0	0	0	0	0	0	0	0	0	0	0
SAL_DA3059AA	CFSAN024142	Bareilly	0	0	0	0	0	0	0	0	0	0	0
SAL_DA3054AA	CFSAN024157	Bareilly	0	0	0	0	0	0	0	0	0	0	0
SAL_DA3033AA	CFSAN024161	Bareilly	0	0	0	0	0	0	0	0	0	0	0
SAL_DA2992AA	CFSAN024160	Bareilly	0	0	0	0	0	0	0	0	0	0	0
SAL_DA2039AA	2014K-0532	Bareilly	0	0	0	0	0	0	0	0	0	0	0
SAL_DA1543AA	FDA00007805	Bareilly	0	0	0	0	0	0	0	0	0	0	0
SAL_DA1538AA	FDA00007812	Bareilly	0	0	0	0	0	0	0	0	0	0	0
SAL_DA1545AA	FDA00007811	Bareilly	0	0	0	0	0	0	0	0	0	0	0
SAL_DA1512AA	H133120696	Bareilly	0	0	0	0	0	0	0	0	0	0	0
SAL_DA0974AA	H131260331	Bareilly	0	0	0	0	0	0	0	0	0	0	0
SAL_DA0675AA	H131100439	Bareilly	0	0	0	0	0	0	0	0	0	0	0
SAL_DA0593AA	H130220270	Bareilly	0	0	0	0	0	0	0	0	0	0	0
SAL_DA0133AA	FDA00008579	Bareilly	0	0	0	0	0	0	0	0	0	0	0
SAL_CA9337AA	FLUFL-828	Bareilly	0	0	0	0	0	0	0	0	0	0	0
SAL_CA9325AA	FLUFL-837	Bareilly	0	0	0	0	0	0	0	0	0	0	0
SAL_CA8539AA	WCU-PED021	Bareilly	0	0	0	0	0	0	0	0	0	0	0
SAL_CA8545AA	WCU-PED003	Bareilly	0	0	0	0	0	0	0	0	0	0	0
SAL_CA8163AA	IEH-NGS-SAL-01910	Bareilly	0	0	0	0	0	0	0	0	0	0	0
SAL_CA8053AA	FDA00001598	Bareilly	0	0	0	0	0	0	0	0	0	0	0
SAL_CA6845AA	44721	Bareilly	0	0	0	0	0	0	0	0	0	0	0
SAL_CA6823AA	44710	Bareilly	0	0	0	0	0	0	0	0	0	0	0
SAL_CA6522AA	66456	Bareilly	0	0	0	0	0	0	0	0	0	0	0
SAL_CA6465AA	48221	Bareilly	0	0	0	0	0	0	0	0	0	0	0
SAL_CA6193AA	54012	Bareilly	0	0	0	0	0	0	0	0	0	0	0
SAL_CA6130AA	58525	Bareilly	0	0	0	0	0	0	0	0	0	0	0
SAL_CA6103AA	58524	Bareilly	0	0	0	0	0	0	0	0	0	0	0
SAL_CA5809AA	78681	Bareilly	0	0	0	0	0	0	0	0	0	0	0
SAL_CA5772AA	70684	Bareilly	0	0	0	0	0	0	0	0	0	0	0
SAL_CA5671AA	79095	Bareilly	0	0	0	0	0	0	0	0	0	0	0
SAL_CA5567AA	81645	Bareilly	0	0	0	0	0	0	0	0	0	0	0
SAL_CA5170AA	81166	Bareilly	0	0	0	0	0	0	0	0	0	0	0

SAL_CA5171AA	75753	Bareilly	0	0	0	0	0	0	0	0	0	0	0
SAL_CA4879AA	WCU-PED002	Bareilly	0	0	0	0	0	0	0	0	0	0	0
SAL_CA4678AA	94226	Bareilly	0	0	0	0	0	0	0	0	0	0	0
SAL_CA4569AA	9276	Bareilly	0	0	0	0	0	0	0	0	0	0	0
SAL_CA4434AA	31610	Bareilly	0	0	0	0	0	0	0	0	0	0	0
SAL_CA4396AA	5904	Bareilly	0	0	0	0	0	0	0	0	0	0	0
SAL_CA4335AA	5377	Bareilly	0	0	0	0	0	0	0	0	0	0	0
SAL_CA4289AA	5829	Bareilly	0	0	0	0	0	0	0	0	0	0	0
SAL_CA4066AA	95064	Bareilly	0	0	0	0	0	0	0	0	0	0	0
SAL_CA3963AA	37070	Bareilly	0	0	0	0	0	0	0	0	0	0	0
SAL_CA3827AA	6491	Bareilly	0	0	0	0	0	0	0	0	0	0	0
SAL_CA3776AA	46548	Bareilly	0	0	0	0	0	0	0	0	0	0	0
SAL_CA3686AA	46564	Bareilly	0	0	0	0	0	0	0	0	0	0	0
SAL_CA3581AA	23464	Bareilly	0	0	0	0	0	0	0	0	0	0	0
SAL_CA3530AA	68705	Bareilly	0	0	0	0	0	0	0	0	0	0	0
SAL_CA3397AA	69927	Bareilly	0	0	0	0	0	0	0	0	0	0	0
SAL_CA3370AA	41951	Bareilly	0	0	0	0	0	0	0	0	0	0	0
SAL_CA3323AA	65130	Bareilly	0	0	0	0	0	0	0	0	0	0	0
SAL_CA3189AA	73209	Bareilly	0	0	0	0	0	0	0	0	0	0	0
SAL_CA3177AA	12158	Bareilly	0	0	0	0	0	0	0	0	0	0	0
SAL_CA3085AA	55548	Bareilly	0	0	0	0	0	0	0	0	0	0	0
SAL_CA2901AA	45979	Bareilly	0	0	0	0	0	0	0	0	0	0	0
SAL_CA2844AA	38732	Bareilly	0	0	0	0	0	0	0	0	0	0	0
SAL_CA2627AA	99768	Bareilly	0	0	0	0	0	0	0	0	0	0	0
SAL_CA2609AA	94092	Bareilly	0	0	0	0	0	0	0	0	0	0	0
SAL_CA2469AA	63016	Bareilly	0	0	0	0	0	0	0	0	0	0	0
SAL_CA2351AA	75983	Bareilly	0	0	0	0	0	0	0	0	0	0	0
SAL_CA2345AA	63575	Bareilly	0	0	0	0	0	0	0	0	0	0	0
SAL_CA2331AA	5906	Bareilly	0	0	0	0	0	0	0	0	0	0	0
SAL_CA2293AA	38744	Bareilly	0	0	0	0	0	0	0	0	0	0	0
SAL_CA2108AA	24980	Bareilly	0	0	0	0	0	0	0	0	0	0	0
SAL_CA2092AA	53003	Bareilly	0	0	0	0	0	0	0	0	0	0	0
SAL_CA1699AA	CFSAN000188	Bareilly	0	0	0	0	0	0	0	0	0	0	0
SAL_CA1686AA	CFSAN000192	Bareilly	0	0	0	0	0	0	0	0	0	0	0
SAL_CA1694AA	CFSAN000193	Bareilly	0	0	0	0	0	0	0	0	0	0	0
SAL_CA1688AA	CFSAN000198	Bareilly	0	0	0	0	0	0	0	0	0	0	0
SAL_CA1692AA	CFSAN000196	Bareilly	0	0	0	0	0	0	0	0	0	0	0
SAL_CA1683AA	CFSAN000194	Bareilly	0	0	0	0	0	0	0	0	0	0	0
SAL_CA1681AA	CFSAN000197	Bareilly	0	0	0	0	0	0	0	0	0	0	0
SAL_CA1679AA	CFSAN000200	Bareilly	0	0	0	0	0	0	0	0	0	0	0
SAL_CA1675AA	CFSAN000187	Bareilly	0	0	0	0	0	0	0	0	0	0	0
SAL_CA1677AA	CFSAN000184	Bareilly	0	0	0	0	0	0	0	0	0	0	0
SAL_CA1667AA	CFSAN000205	Bareilly	0	0	0	0	0	0	0	0	0	0	0
SAL_CA1657AA	CFSAN000206	Bareilly	0	0	0	0	0	0	0	0	0	0	0
SAL_CA1661AA	CFSAN000210	Bareilly	0	0	0	0	0	0	0	0	0	0	0
SAL_CA1663AA	CFSAN000209	Bareilly	0	0	0	0	0	0	0	0	0	0	0
SAL_CA1655AA	CFSAN000182	Bareilly	0	0	0	0	0	0	0	0	0	0	0
SAL_CA1659AA	CFSAN000208	Bareilly	0	0	0	0	0	0	0	0	0	0	0
SAL_CA1653AA	CFSAN000183	Bareilly	0	0	0	0	0	0	0	0	0	0	0
SAL_CA1647AA	CFSAN000180	Bareilly	0	0	0	0	0	0	0	0	0	0	0
SAL_CA1649AA	CFSAN000179	Bareilly	0	0	0	0	0	0	0	0	0	0	0
SAL_CA1639AA	CFSAN000213	Bareilly	0	0	0	0	0	0	0	0	0	0	0
SAL_CA1643AA	CFSAN000236	Bareilly	0	0	0	0	0	0	0	0	0	0	0
SAL_CA1641AA	CFSAN000202	Bareilly	0	0	0	0	0	0	0	0	0	0	0
SAL_CA1637AA	CFSAN000214	Bareilly	0	0	0	0	0	0	0	0	0	0	0
SAL_CA1633AA	CFSAN000217	Bareilly	0	0	0	0	0	0	0	0	0	0	0
SAL_CA1626AA	CFSAN000222	Bareilly	0	0	0	0	0	0	0	0	0	0	0
SAL_CA1645AA	CFSAN000235	Bareilly	0	0	0	0	0	0	0	0	0	0	0
SAL_CA1635AA	CFSAN000215	Bareilly	0	0	0	0	0	0	0	0	0	0	0
SAL_CA1630AA	CFSAN000212	Bareilly	0	0	0	0	0	0	0	0	0	0	0
SAL_CA1622AA	CFSAN000220	Bareilly	0	0	0	0	0	0	0	0	0	106	0
SAL_CA1620AA	CFSAN000223	Bareilly	0	0	0	0	0	0	0	0	0	0	0
SAL_CA1628AA	CFSAN000216	Bareilly	0	0	0	0	0	0	0	0	0	0	0
SAL_CA1624AA	CFSAN000219	Bareilly	0	0	0	0	0	0	0	0	0	0	0
SAL_CA1616AA	CFSAN000218	Bareilly	0	0	0	0	0	0	0	0	0	0	0
SAL_CA1632AA	CFSAN000228	Bareilly	0	0	0	0	0	0	0	0	0	0	0
SAL_CA1614AA	CFSAN000211	Bareilly	0	0	0	0	0	0	0	0	0	0	0
SAL_CA1605AA	84433	Bareilly	0	0	0	0	0	0	0	0	0	0	0
SAL_CA1567AA	CFSAN000662	Bareilly	0	0	0	0	0	0	0	0	0	0	0
SAL_CA1556AA	91215	Bareilly	0	0	0	0	0	0	0	0	0	0	0
SAL_CA1555AA	CFSAN000754	Bareilly	0	0	0	0	0	0	0	0	0	0	0
SAL_CA1551AA	CFSAN000952	Bareilly	0	0	0	0	0	0	0	0	0	0	0
SAL_CA1557AA	CFSAN000753	Bareilly	0	0	0	0	0	0	0	0	0	0	0

SAL_CA1559AA	CFSAN000752	Bareilly	0	0	0	0	0	0	0	0	0	0	0
SAL_CA1561AA	CFSAN000700	Bareilly	0	0	0	0	0	0	0	0	0	0	0
SAL_CA1553AA	CFSAN000951	Bareilly	0	0	0	0	0	0	0	0	0	0	0
SAL_CA1565AA	CFSAN000669	Bareilly	0	0	0	0	0	0	0	0	0	0	0
SAL_CA1569AA	CFSAN000661	Bareilly	0	0	0	0	0	0	0	0	0	0	0
SAL_CA1563AA	CFSAN000680	Bareilly	0	0	0	0	0	0	0	0	0	0	0
SAL_CA1541AA	CFSAN000957	Bareilly	0	0	0	0	0	0	0	0	0	0	0
SAL_CA1539AA	CFSAN000958	Bareilly	0	0	0	0	0	0	0	0	0	0	0
SAL_CA1538AA	90523	Bareilly	0	0	0	0	0	0	0	0	0	0	0
SAL_CA1537AA	CFSAN000959	Bareilly	0	0	0	0	0	0	0	0	0	0	0
SAL_CA1536AA	CFSAN000960	Bareilly	0	0	0	0	0	0	0	0	0	0	0
SAL_CA1534AA	CFSAN000961	Bareilly	0	0	0	0	0	0	0	0	0	0	0
SAL_CA1529AA	CFSAN000963	Bareilly	0	0	0	0	0	0	0	0	0	0	0
SAL_CA1525AA	CFSAN000964	Bareilly	0	0	0	0	0	0	0	0	0	0	0
SAL_CA1515AA	CFSAN000970	Bareilly	0	0	0	0	0	0	0	0	0	0	0
SAL_CA1518AA	CFSAN000969	Bareilly	0	0	0	0	0	0	0	0	0	0	0
SAL_CA1523AA	CFSAN000965	Bareilly	0	0	0	0	0	0	0	0	0	0	0
SAL_CA1130AA	45961	Bareilly	0	0	0	0	0	0	0	0	0	0	0
SAL_CA0757AA	86876	Bareilly	0	0	0	0	0	0	0	0	0	0	0
SAL_CA0622AA	41948	Bareilly	0	0	0	0	0	0	0	0	0	0	0
SAL_CA0598AA	23465	Bareilly	0	0	0	0	0	0	0	0	0	0	0
SAL_BA9476AA	CFSAN000954	Bareilly	0	0	0	0	0	0	0	0	0	0	0
SAL_BA9380AA	31937	Bareilly	0	0	0	0	0	0	0	0	0	0	0
SAL_BA8459AA	86890	Bareilly	0	0	0	0	0	0	0	0	0	0	0
SAL_BA8403AA	7377	Bareilly	0	0	0	0	0	0	0	0	0	0	0
SAL_BA8396AA	86863	Bareilly	0	0	0	0	0	0	0	0	0	0	0
SAL_BA8301AA	14894	Bareilly	0	0	0	0	0	0	0	0	0	0	0
SAL_BA7513AA	83698	Bareilly	0	0	0	0	0	0	0	0	0	0	0
SAL_BA7466AA	31627	Bareilly	0	0	0	0	0	0	0	0	0	0	0
SAL_BA7451AA	5827	Bareilly	0	0	0	0	0	0	0	0	0	0	0
SAL_BA7440AA	63651	Bareilly	0	0	0	0	0	0	0	0	0	0	0
SAL_BA7019AA	FDA00002723	Bareilly	0	0	0	0	0	0	0	0	0	0	0
SAL_BA6521AA	2015K-0193	Bareilly	0	0	0	0	0	0	0	0	0	0	0
SAL_BA5846AA	M154	Bareilly	0	0	0	0	0	0	0	0	0	0	0
SAL_BA5630AA	FSF0110	Bareilly	0	0	0	0	0	0	0	0	0	0	0
SAL_BA3761AA	CFSAN003948	Bareilly	0	0	0	0	0	0	0	0	0	0	0
SAL_BA3545AA	CFSAN029943	Bareilly	0	0	0	0	0	0	0	0	0	0	0
SAL_BA2868AA	CFSAN030656	Bareilly	0	0	0	0	0	0	0	0	0	0	0
SAL_BA2833AA	CFSAN030648	Bareilly	0	0	0	0	0	0	0	0	0	0	0
SAL_BA2707AA	CFSAN030654	Bareilly	0	0	0	0	0	0	0	0	0	0	0
SAL_BA2532AA	CFSAN029942	Bareilly	0	0	0	0	0	0	0	0	0	0	0
SAL_BA2216AA	FDA00001210	Bareilly	0	0	0	0	0	0	0	0	0	0	0
SAL_BA1880AA	TX-ACF1402530	Bareilly	0	0	0	0	0	0	0	0	0	16	0
SAL_BA1677AA	FDA00009235	Bareilly	0	0	0	0	0	0	0	0	0	0	0
SAL_BA1373AA	FDA00007868	Bareilly	0	0	0	0	0	0	0	0	0	0	0
SAL_BA1358AA	CFSAN003832	Bareilly	0	0	0	0	0	0	0	0	0	0	0
SAL_BA1318AA	FDA00009272	Bareilly	0	0	0	0	0	0	0	0	0	0	0
SAL_FA6096AA	FDA00010125	Bareilly	0	0	0	0	0	0	0	0	0	0	0
SAL_FA6095AA	FDA00010126	Bareilly	0	0	0	0	0	0	0	0	0	0	0
SAL_FA6055AA	PNUSAS001806	Bareilly	0	0	0	0	0	0	0	0	0	0	0
SAL_FA6494AA	FDA00010124	Bareilly	0	0	0	0	0	0	0	0	0	0	0
SAL_FA6489AA	FDA00010144	Bareilly	0	0	0	0	0	0	0	0	0	0	0
SAL_FA6493AA	FDA00010127	Bareilly	0	0	0	0	0	0	0	0	0	0	0
SAL_FA7515AA	FSE0153	Bareilly	0	0	0	0	0	0	0	0	0	0	0
SAL_FA7989AA	2016K-0428	Bareilly	0	0	0	0	0	0	0	0	0	0	0
SAL_FA7987AA	2016K-0425	Bareilly	0	0	0	0	0	0	0	0	0	0	0
SAL_FA7986AA	2016K-0424	Bareilly	0	0	0	0	0	0	0	0	0	0	0
SAL_FA7985AA	2016K-0439	Bareilly	0	0	0	0	0	0	0	0	0	0	0
SAL_FA7978AA	2016K-0438	Bareilly	0	0	0	0	0	0	0	0	0	0	0
SAL_FA7981AA	2016K-0433	Bareilly	0	0	0	0	0	0	0	0	0	0	0
SAL_FA7977AA	2016K-0437	Bareilly	0	0	0	0	0	0	0	0	0	0	0
SAL_FA7975AA	2016K-0434	Bareilly	0	0	0	0	0	0	0	0	0	0	0
SAL_FA7963AA	PNUSAS002201	Bareilly	0	0	0	0	0	0	0	0	0	0	0
SAL_FA8044AA	FDA00010266	Bareilly	0	0	0	0	0	0	0	0	0	0	0
SAL_FA8115AA	2016K-0468	Bareilly	0	0	0	0	0	0	0	0	0	0	0
SAL_FA8199AA	FDA00010289	Bareilly	0	0	0	0	0	0	0	0	0	0	0
SAL_GA4346AA	PNUSAS002654	Bareilly	0	0	0	0	0	0	0	0	0	0	0
SAL_GA4347AA	PNUSAS002382	Bareilly	0	0	0	0	0	0	0	0	0	0	0
SAL_GA4332AA	PNUSAS002383	Bareilly	0	0	0	0	0	0	0	0	0	0	0
SAL_GA3466AA	BCW_3964	Bareilly	0	0	0	0	0	0	0	0	0	0	0
SAL_GA3354AA	BCW_2644	Bareilly	0	0	0	0	0	0	0	0	0	0	0
SAL_GA3353AA	BCW_2645	Bareilly	0	0	0	0	0	0	0	0	0	0	0
SAL_GA4448AA	PNUSAS002379	Bareilly	0	0	0	0	0	0	0	0	0	0	0

SAL_GA4447AA	PNUSAS002380	Bareilly	0	0	0	0	0	0	0	0	0	0	0
SAL_GA4395AA	2016K-0491	Bareilly	0	0	0	0	0	0	0	0	0	0	0
SAL_GA1675AA	BCW_2890	Bareilly	0	0	0	0	0	0	0	0	0	0	0
SAL_GA4685AA	NSW SB1	Bareilly	0	0	0	0	0	0	0	0	0	0	0
SAL_GA0319AA	71_H_070-sc-2280676	Bareilly	0	0	0	0	0	0	0	0	0	0	0
SAL_GA0285AA	72_H_296-sc-2280734	Bareilly	0	0	0	0	0	0	0	0	0	0	0
SAL_GA4885AA	22	Bareilly	0	0	0	0	0	0	0	0	0	0	0
SAL_GA4961AA	NSW SB17	Bareilly	0	0	0	0	0	0	0	0	0	0	0
SAL_GA4962AA	NSW SB9	Bareilly	0	0	0	0	0	0	0	0	0	0	0
SAL_GA4963AA	NSW SB10	Bareilly	0	0	0	0	0	0	0	0	0	0	0
SAL_GA4964AA	NSW SB26	Bareilly	0	0	0	0	0	0	0	0	0	0	0
SAL_GA4965AA	NSW SB18	Bareilly	0	0	0	0	0	0	0	0	0	0	0
SAL_GA4966AA	NSW SB3	Bareilly	0	0	0	0	0	0	0	0	0	0	0
SAL_GA4967AA	NSW SB11	Bareilly	0	0	0	0	0	0	0	0	0	0	0
SAL_GA4968AA	NSW SB27	Bareilly	0	0	0	0	0	0	0	0	0	0	0
SAL_GA4969AA	NSW SB19	Bareilly	0	0	0	0	0	0	0	0	0	0	0
SAL_FA9635AA	PNUSAS001365	Bareilly	0	0	0	0	0	0	0	0	0	0	0
SAL_GA4970AA	NSW SB4	Bareilly	0	0	0	0	0	0	0	0	0	0	0
SAL_GA4971AA	NSW SB20	Bareilly	0	0	0	0	0	0	0	0	0	0	0
SAL_GA4972AA	NSW SB12	Bareilly	0	0	0	0	0	0	0	0	0	0	0
SAL_GA4973AA	NSW SB28	Bareilly	0	0	0	0	0	0	0	0	0	0	0
SAL_GA4974AA	NSW SB5	Bareilly	0	0	0	0	0	0	0	0	0	0	0
SAL_GA4975AA	NSW SB13	Bareilly	0	0	0	0	0	0	0	0	0	0	0
SAL_GA4976AA	NSW SB21	Bareilly	0	0	0	0	0	0	0	0	0	0	0
SAL_GA4977AA	NSW SB6	Bareilly	0	0	0	0	0	0	0	0	0	0	0
SAL_FA9578AA	2015K-1490	Bareilly	0	0	0	0	0	0	0	0	0	0	0
SAL_GA4978AA	NSW SB29	Bareilly	0	0	0	0	0	0	0	0	0	0	0
SAL_GA4980AA	NSW SB14	Bareilly	0	0	0	0	0	0	0	0	0	0	0
SAL_GA5870AA	NSW SB23	Bareilly	0	0	0	0	0	0	0	0	0	0	0
SAL_GA4984AA	NSW SB25	Bareilly	0	0	0	0	0	0	0	0	0	0	0
SAL_GA4985AA	21	Bareilly	0	0	0	0	0	0	0	0	0	0	0
SAL_GA4987AA	NSW SB41	Bareilly	0	0	0	0	0	0	0	0	0	0	0
SAL_GA4794AA	PNUSAS002381	Bareilly	0	0	0	0	0	0	0	0	0	0	0
SAL_GA4772AA	PNUSAS002833	Bareilly	0	0	0	0	0	0	0	0	0	0	0
SAL_GA4768AA	PNUSAS002837	Bareilly	0	0	0	0	0	0	0	0	0	0	0
SAL_FA9471AA	2016K-0115	Bareilly	0	0	0	0	0	0	0	0	0	0	0
SAL_FA9381AA	FDA00007810	Bareilly	0	0	0	0	0	0	0	0	0	0	0
SAL_GA5050AA	2016K-0515	Bareilly	0	0	0	0	0	0	0	0	0	0	0
SAL_GA5774AA	NSW SB34	Bareilly	0	0	0	0	0	0	0	0	0	0	0
SAL_GA5771AA	NSW SB43	Bareilly	0	0	0	0	0	0	0	0	0	0	0
SAL_GA5777AA	NSW SB38	Bareilly	0	0	0	0	0	0	0	0	0	0	0
SAL_GA5778AA	NSW SB44	Bareilly	0	0	0	0	0	0	0	0	0	0	0
SAL_GA5768AA	NSW SB47	Bareilly	0	0	0	0	0	0	0	0	0	0	0
SAL_GA5769AA	NSW SB2	Bareilly	0	0	0	0	0	0	0	0	0	0	0
SAL_GA5770AA	NSW SB31	Bareilly	0	0	0	0	0	0	0	0	0	0	0
SAL_GA5772AA	NSW SB33	Bareilly	0	0	0	0	0	0	0	0	0	0	0
SAL_GA5773AA	NSW SB39	Bareilly	0	0	0	0	0	0	0	0	0	0	0
SAL_GA5775AA	NSW SB37	Bareilly	0	0	0	0	0	0	0	0	0	0	0
SAL_GA5776AA	NSW SB42	Bareilly	0	0	0	0	0	0	0	0	0	0	0
SAL_GA5779AA	NSW SB40	Bareilly	0	0	0	0	0	0	0	0	0	0	0
SAL_GA5780AA	NSW SB46	Bareilly	0	0	0	0	0	0	0	0	0	0	0
SAL_GA5781AA	NSW SB45	Bareilly	0	0	0	0	0	0	0	0	0	0	0
SAL_GA5783AA	NSW SB8	Bareilly	0	0	0	0	0	0	0	0	0	0	0
SAL_GA5782AA	NSW SB24	Bareilly	0	0	0	0	0	0	0	0	0	0	0
SAL_GA5786AA	NSW SB32	Bareilly	0	0	0	0	0	0	0	0	0	0	0
SAL_GA5785AA	NSW SB16	Bareilly	0	0	0	0	0	0	0	0	0	0	0
SAL_GA5787AA	NSW SB36	Bareilly	0	0	0	0	0	0	0	0	0	0	0
SAL_FA8482AA	PNUSAS002384	Bareilly	0	0	0	0	0	0	0	0	0	0	0
SAL_FA8407AA	PNUSAS002616	Bareilly	0	0	0	0	0	0	0	0	0	0	0
SAL_GA7080AA	CFSAN044963	Bareilly	0	0	0	0	0	0	0	0	0	0	0
SAL_GA7084AA	CFSAN044959	Bareilly	0	0	0	0	0	0	0	0	0	0	0
SAL_GA7083AA	CFSAN044960	Bareilly	0	0	0	0	0	0	0	0	0	0	0
SAL_GA7043AA	CFSAN045001	Bareilly	0	0	0	0	0	0	0	0	0	0	0
SAL_GA7041AA	CFSAN045003	Bareilly	0	0	0	0	0	0	0	0	0	0	0
SAL_GA7028AA	CFSAN045016	Bareilly	0	0	0	0	0	0	0	0	0	0	0
SAL_GA7018AA	CFSAN045026	Bareilly	0	0	0	0	0	0	0	0	0	0	0
SAL_GA7010AA	CFSAN045034	Bareilly	0	0	0	0	0	0	0	0	0	0	0
SAL_GA8229AA	SB25-1	Bareilly	0	0	0	0	0	0	0	0	0	0	0
SAL_GA8228AA	SB23-1	Bareilly	0	0	0	0	0	0	0	0	0	0	0
SAL_GA8574AA	PNUSAS003491	Bareilly	0	0	0	0	0	0	0	0	0	0	0
SAL_GA8590AA	FDA00010560	Bareilly	0	0	0	0	0	0	0	0	0	0	0
SAL_GA8649AA	PNUSAS003509	Bareilly	0	0	0	0	0	0	0	0	0	0	0
SAL_GA8984AA	CFSAN047952	Bareilly	0	0	0	0	0	0	0	0	0	0	0

SAL_GA9011AA	CFSAN047940	Bareilly	0	0	0	0	0	0	0	0	0	0	0
SAL_GA8987AA	CFSAN047944	Bareilly	0	0	0	0	0	0	0	0	0	0	0
SAL_GA8986AA	CFSAN047945	Bareilly	0	0	0	0	0	0	0	0	0	0	0
SAL_GA8979AA	CFSAN047966	Bareilly	0	0	0	0	0	0	0	0	0	0	0
SAL_GA8985AA	CFSAN047951	Bareilly	0	0	0	0	0	0	0	0	0	0	0
SAL_GA9007AA	CFSAN047926	Bareilly	0	0	0	0	0	0	0	0	0	0	0
SAL_GA8991AA	CFSAN047964	Bareilly	0	0	0	0	0	0	0	0	0	0	0
SAL_GA8992AA	CFSAN047963	Bareilly	0	0	0	0	0	0	0	0	0	0	0
SAL_GA8988AA	CFSAN047942	Bareilly	0	0	0	0	0	0	0	0	0	0	0
SAL_GA9021AA	CFSAN047941	Bareilly	0	0	0	0	0	0	0	0	0	0	0
SAL_GA9012AA	CFSAN047939	Bareilly	0	0	0	0	0	0	0	0	0	0	0
SAL_GA9016AA	CFSAN047950	Bareilly	0	0	0	0	0	0	0	0	0	0	0
SAL_GA9211AA	CFSAN000953	Bareilly	0	0	0	0	0	0	0	0	0	0	0
SAL_GA9266AA	PNUSAS003762	Bareilly	0	0	0	0	0	0	0	0	0	0	0
SAL_GA9234AA	FDA00010591	Bareilly	0	0	0	0	0	0	0	0	0	0	0
SAL_GA9454AA	FSIS1607664	Bareilly	0	0	0	0	0	0	0	0	0	0	0
SAL_HA0155AA	HIY0304	Bareilly	0	0	0	0	0	0	0	0	0	0	0
SAL_HA0332AA	PNUSAS004328	Bareilly	0	0	0	0	0	0	0	0	0	0	0
SAL_HA0313AA	PNUSAS003463	Bareilly	0	0	0	0	0	0	0	0	0	0	0
SAL_HA0311AA	PNUSAS003465	Bareilly	0	0	0	0	0	0	0	0	0	0	0
SAL_HA0304AA	PNUSAS003719	Bareilly	0	0	0	0	0	0	0	0	0	0	0
SAL_HA0421AA	HIY0286	Bareilly	0	0	0	0	0	0	0	0	0	0	0
SAL_HA0420AA	HIY0301	Bareilly	0	0	0	0	0	0	0	0	0	0	0
SAL_HA0419AA	HIY0302	Bareilly	0	0	0	0	0	0	0	0	0	0	0
SAL_HA0422AA	HIY0285	Bareilly	0	0	0	0	0	0	0	0	0	0	0
SAL_HA0754AA	PNUSAS004440	Bareilly	0	0	0	0	0	0	0	0	0	0	0
SAL_HA1445AA	CFSAN035962	Bareilly	0	0	0	0	0	0	0	0	0	0	0
SAL_HA1443AA	CFSAN035969	Bareilly	0	0	0	0	0	0	0	0	0	0	0
SAL_HA1367AA	PNUSAS004517	Bareilly	0	0	0	0	0	0	0	0	0	0	0
SAL_HA3065AA	PNUSAS004705	Bareilly	0	0	0	0	0	0	0	0	0	0	0
SAL_HA4056AA	PNUSAS005005	Bareilly	0	0	0	0	0	0	0	0	0	0	0
SAL_HA4367AA	FDA00010918	Bareilly	0	0	0	0	0	0	0	0	0	0	0
SAL_HA4514AA	CFSAN035960	Bareilly	0	0	0	0	0	0	0	0	0	0	0
SAL_HA4499AA	CFSAN035970	Bareilly	0	0	0	0	0	0	0	0	0	0	0
SAL_HA4507AA	CFSAN035967	Bareilly	0	0	0	0	0	0	0	0	0	0	0
SAL_HA4502AA	CFSAN035966	Bareilly	0	0	0	0	0	0	0	0	0	0	0
SAL_HA4491AA	CFSAN035973	Bareilly	0	0	0	0	0	0	0	0	0	0	0
SAL_HA4501AA	CFSAN035968	Bareilly	0	0	0	0	0	0	0	0	0	0	0
SAL_HA4483AA	CFSAN035971	Bareilly	0	0	0	0	0	0	0	0	0	0	0
SAL_HA4485AA	CFSAN035972	Bareilly	0	0	0	0	0	0	0	0	0	0	0
SAL_HA4528AA	CFSAN035956	Bareilly	0	0	0	0	0	0	0	0	0	0	0
SAL_HA4479AA	CFSAN035983	Bareilly	0	0	0	0	0	0	0	0	0	0	0
SAL_HA4521AA	CFSAN035957	Bareilly	0	0	0	0	0	0	0	0	0	0	0
SAL_HA4505AA	CFSAN035965	Bareilly	0	0	0	0	0	0	0	0	0	0	0
SAL_HA5499AA	CFSAN056564	Bareilly	0	0	0	0	0	0	0	0	0	0	0
SAL_HA0014AA	PNUSAS004104	Bareilly	0	0	0	0	0	0	0	0	0	0	0
SAL_HA2529AA	CFSAN035764	Bareilly	0	0	0	0	0	0	0	0	0	0	0
SAL_HA2536AA	CFSAN035763	Bareilly	0	0	0	0	0	0	0	0	0	0	0
SAL_HA2558AA	CFSAN035736	Bareilly	0	0	0	0	0	0	0	0	0	0	0
SAL_HA2584AA	CFSAN035707	Bareilly	0	0	0	0	0	0	0	0	0	0	0
SAL_HA2582AA	CFSAN035708	Bareilly	0	0	0	0	0	0	0	0	0	0	0
SAL_HA3893AA	PNUSAS005224	Bareilly	0	0	0	0	0	0	0	0	0	0	0
SAL_HA4464AA	CFSAN035984	Bareilly	0	0	0	0	0	0	0	0	0	0	0
SAL_HA6838AA	PNUSAS005721	Bareilly	0	0	0	0	0	0	0	0	0	0	0
SAL_HA6820AA	PNUSAS006108	Bareilly	0	0	0	0	0	0	0	0	0	0	0
SAL_HA6777AA	PNUSAS005632	Bareilly	0	0	0	0	0	0	0	0	0	0	0
SAL_HA6970AA	CFSAN056796	Bareilly	0	0	0	0	0	0	0	0	0	0	0
SAL_HA6964AA	CFSAN056797	Bareilly	0	0	0	0	0	0	0	0	0	0	0
SAL_HA6383AA	PNUSAS005890	Bareilly	0	0	0	0	0	0	0	0	0	0	0
SAL_HA6176AA	PNUSAS005343	Bareilly	0	0	0	0	0	0	0	0	0	0	0
SAL_HA7343AA	PNUSAS005777	Bareilly	0	0	0	0	0	0	0	0	0	0	0
SAL_HA7349AA	PNUSAS005775	Bareilly	0	0	0	0	0	0	0	0	0	0	0
SAL_HA7707AA	CFSAN036041	Bareilly	0	0	0	0	0	0	0	0	0	0	0
SAL_HA7711AA	CFSAN036042	Bareilly	0	0	0	0	0	0	0	0	0	0	0
SAL_HA7706AA	CFSAN036038	Bareilly	0	0	0	0	0	0	0	0	0	0	0
SAL_HA7709AA	CFSAN036037	Bareilly	0	0	0	0	0	0	0	0	0	0	0
SAL_HA7670AA	CFSAN036057	Bareilly	0	0	0	0	0	0	0	0	0	0	0
SAL_HA7671AA	CFSAN036055	Bareilly	0	0	0	0	0	0	0	0	0	0	0
SAL_HA7669AA	CFSAN036056	Bareilly	0	0	0	0	0	0	0	0	0	0	0
SAL_HA7668AA	CFSAN036058	Bareilly	0	0	0	0	0	0	0	0	0	0	0
SAL_HA8608AA	PNUSAS006345	Bareilly	0	0	0	0	0	0	0	0	0	0	0
SAL_HA8357AA	PNUSAS006402	Bareilly	0	0	0	0	0	0	0	0	0	0	0
SAL_HA8321AA	PNUSAS005778	Bareilly	0	0	0	0	0	0	0	0	0	0	0

SAL_HA9179AA	PNUSAS006755	Bareilly	0	0	0	0	0	0	0	0	0	16	0
SAL_IA0101AA	0-3918-11e6-bcc0-3c4a9	Bareilly	0	0	0	0	0	0	0	0	0	0	0
SAL_IA0267AA	PNUSAS006815	Bareilly	0	0	0	0	0	0	0	0	0	0	0
SAL_IA0220AA	PNUSAS006114	Bareilly	0	0	0	0	0	0	0	0	0	0	0
SAL_IA0225AA	PNUSAS006115	Bareilly	0	0	0	0	0	0	0	0	0	0	0
SAL_IA0458AA	PNUSAS007076	Bareilly	0	0	0	0	0	0	0	0	0	0	0
SAL_IA0411AA	PNUSAS007364	Bareilly	0	0	0	0	0	0	0	0	0	0	0
SAL_IA0965AA	287738	Bareilly	0	0	0	0	0	0	0	0	0	0	0
SAL_IA1906AA	PNUSAS007315	Bareilly	0	0	0	0	0	0	0	0	0	0	0
SAL_IA2252AA	MS160040	Bareilly	0	0	0	0	0	0	0	0	0	0	0
SAL_IA3580AA	189194	Bareilly	0	0	0	0	0	0	0	0	0	0	0
SAL_IA3180AA	188186	Bareilly	0	0	0	0	0	0	0	0	0	0	0
SAL_IA3867AA	PNUSAS007955	Bareilly	0	0	0	0	0	0	0	0	0	0	0
SAL_IA3754AA	PNUSAS008051	Bareilly	0	0	0	0	0	0	0	0	0	0	0
SAL_IA4817AA	MS160238	Bareilly	0	0	0	0	0	0	0	0	0	0	0
SAL_IA5032AA	MS160344	Bareilly	0	0	0	0	0	0	0	0	0	0	0
SAL_IA6326AA	PNUSAS005587	Bareilly	0	0	0	0	0	0	0	0	0	0	0
SAL_IA6168AA	PNUSAS007785	Bareilly	0	0	0	0	0	0	0	0	0	0	0
SAL_IA5526AA	CFSAN059810	Bareilly	0	0	0	0	0	0	0	0	0	0	0
SAL_IA5427AA	PNUSAS008464	Bareilly	0	0	0	0	0	0	0	0	0	0	0
SAL_IA6421AA	PNUSAS008519	Bareilly	0	0	0	0	0	0	0	0	0	0	0
SAL_IA6521AA	PNUSAS008642	Bareilly	0	0	0	0	0	0	0	0	0	0	0
SAL_IA6702AA	MS150326	Bareilly	0	0	0	0	0	0	0	0	0	0	0
SAL_IA7081AA	CFSAN059809	Bareilly	0	0	0	0	0	0	0	0	0	0	0
SAL_IA7075AA	CFSAN059792	Bareilly	0	0	0	0	0	0	0	0	0	0	0
SAL_IA7340AA	S/20170057	Bareilly	0	0	0	0	0	0	0	0	0	0	0
SAL_IA7579AA	PNUSAS009131	Bareilly	0	0	0	0	0	0	0	0	0	16	0
SAL_IA7589AA	PNUSAS009117	Bareilly	0	0	0	0	0	0	0	0	0	0	0
SAL_IA8178AA	FDA00011346	Bareilly	0	0	0	0	0	0	0	0	0	0	0
SAL_IA8174AA	FDA00003944	Bareilly	0	0	0	0	0	0	0	0	0	0	0
SAL_IA8432AA	PNUSAS009207	Bareilly	0	0	0	0	0	0	0	0	0	0	0
SAL_IA8774AA	CFSAN057146	Bareilly	0	0	0	0	0	0	0	0	0	0	0
SAL_IA8771AA	CFSAN057148	Bareilly	0	0	0	0	0	0	0	0	0	0	0
SAL_IA8773AA	CFSAN057147	Bareilly	0	0	0	0	0	0	0	0	0	0	0
SAL_IA8777AA	CFSAN057144	Bareilly	0	0	0	0	0	0	0	0	0	0	0
SAL_IA8775AA	CFSAN057145	Bareilly	0	0	0	0	0	0	0	0	0	0	0
SAL_IA8763AA	CFSAN057164	Bareilly	0	0	0	0	0	0	0	0	0	0	0
SAL_IA8753AA	CFSAN057170	Bareilly	0	0	0	0	0	0	0	0	0	0	0
SAL_IA8754AA	CFSAN057166	Bareilly	0	0	0	0	0	0	0	0	0	0	0
SAL_IA8755AA	CFSAN057165	Bareilly	0	0	0	0	0	0	0	0	0	0	0
SAL_IA8756AA	CFSAN057161	Bareilly	0	0	0	0	0	0	0	0	0	0	0
SAL_IA8757AA	CFSAN057162	Bareilly	0	0	0	0	0	0	0	0	0	0	0
SAL_IA8760AA	CFSAN057163	Bareilly	0	0	0	0	0	0	0	0	0	0	0
SAL_IA8749AA	CFSAN057168	Bareilly	0	0	0	0	0	0	0	0	0	0	0
SAL_IA8750AA	CFSAN057167	Bareilly	0	0	0	0	0	0	0	0	0	0	0
SAL_IA8751AA	CFSAN057171	Bareilly	0	0	0	0	0	0	0	0	0	0	0
SAL_IA8752AA	CFSAN057169	Bareilly	0	0	0	0	0	0	0	0	0	0	0
SAL_IA8707AA	CFSAN057212	Bareilly	0	0	0	0	0	0	0	0	0	0	0
SAL_IA8708AA	CFSAN057213	Bareilly	0	0	0	0	0	0	0	0	0	0	0
SAL_IA8671AA	CFSAN057244	Bareilly	0	0	0	0	0	0	0	0	0	0	0
SAL_IA8675AA	CFSAN057243	Bareilly	0	0	0	0	0	0	0	0	0	0	0
SAL_IA8677AA	CFSAN057242	Bareilly	0	0	0	0	0	0	0	0	0	0	0
SAL_IA8711AA	CFSAN057211	Bareilly	0	0	0	0	0	0	0	0	0	0	0
SAL_IA8732AA	CFSAN057184	Bareilly	0	0	0	0	0	0	0	0	0	0	0
SAL_IA8640AA	CFSAN057285	Bareilly	0	0	0	0	0	0	0	0	0	0	0
SAL_IA8679AA	CFSAN057241	Bareilly	0	0	0	0	0	0	0	0	0	0	0
SAL_IA8643AA	CFSAN057284	Bareilly	0	0	0	0	0	0	0	0	0	0	0
SAL_IA9385AA	PNUSAS008944	Bareilly	0	0	0	0	0	0	0	0	0	0	0
SAL_IA9805AA	PNUSAS010031	Bareilly	0	0	0	0	0	0	0	0	0	0	0
SAL_IA9846AA	PNUSAS009812	Bareilly	0	0	0	0	0	0	0	0	0	0	0
SAL_IA9666AA	PNUSAS009406	Bareilly	5	4	20	21	0	0	0	0	0	0	0
SAL_JA0181AA	PNUSAS010110	Bareilly	0	0	0	0	0	0	0	0	0	0	0
SAL_JA0189AA	PNUSAS010604	Bareilly	0	0	0	0	0	0	0	0	0	0	0
SAL_JA0118AA	PNUSAS010613	Bareilly	0	0	0	0	0	0	0	0	0	0	0
SAL_JA0154AA	PNUSAS010610	Bareilly	0	0	0	0	0	0	0	0	0	0	0
SAL_JA0085AA	PNUSAS010043	Bareilly	0	0	0	0	0	0	0	0	0	0	0
SAL_JA0109AA	PNUSAS010606	Bareilly	0	0	0	0	0	0	0	0	0	0	0
SAL_JA1802AA	PNUSAS010367	Bareilly	0	0	0	0	0	0	0	0	0	0	0
SAL_JA2013AA	PNUSAS011460	Bareilly	0	0	0	0	0	0	0	0	0	0	0
SAL_JA2006AA	PNUSAS011529	Bareilly	0	0	0	0	0	0	0	0	0	0	0
SAL_JA0423AA	PNUSAS010651	Bareilly	0	0	0	0	0	0	0	0	0	0	0
SAL_JA2325AA	VA-WGS-17045	Bareilly	0	0	0	0	0	0	0	0	0	0	0
SAL_JA2555AA	PNUSAS011514	Bareilly	0	0	0	0	0	0	0	0	0	0	0

SAL_JA2663AA	PNUSAS011967	Bareilly	0	0	0	0	0	0	0	0	0	0	0
SAL_JA2380AA	PNUSAS010327	Bareilly	0	0	0	0	0	0	0	0	0	0	0
SAL_JA2879AA	PNUSAS011991	Bareilly	0	0	0	0	0	0	0	0	0	0	0
SAL_JA3165AA	PNUSAS012224	Bareilly	0	0	0	0	0	0	0	0	0	0	0
SAL_JA3141AA	PNUSAS011990	Bareilly	0	0	0	0	0	0	0	0	0	0	0
SAL_JA3052AA	CNSV-T2-MD10-10-TT-B	Bareilly	0	0	0	0	0	0	0	0	0	0	0
SAL_JA3051AA	CNSV-T3-MD11-10-TT-D	Bareilly	0	0	0	0	0	0	0	0	0	0	0
SAL_JA3053AA	CNSV-T3-MD11-10-TT-E	Bareilly	0	0	0	0	0	0	0	0	0	0	0
SAL_JA3063AA	CNSV-T2-MD10-10-RV-A	Bareilly	0	0	0	0	0	0	0	0	0	0	0
SAL_JA3049AA	CNSV-T3-MD11-10-RV-B	Bareilly	0	0	0	0	0	0	0	0	0	0	0
SAL_JA3042AA	CNSV-T3-MD11-1-TT-D	Bareilly	0	0	0	0	0	0	0	0	0	0	0
SAL_JA3048AA	CNSV-T3-MD11-10-TT-C	Bareilly	0	0	0	0	0	0	0	0	0	0	0
SAL_JA3055AA	CNSV-T2-MD10-10-TT-C	Bareilly	0	0	0	0	0	0	0	0	0	0	0
SAL_JA3054AA	CNSV-T2-MD10-10-TT-A	Bareilly	0	0	0	0	0	0	0	0	0	0	0
SAL_JA3047AA	CNSV-T3-MD11-10-RV-A	Bareilly	0	0	0	0	0	0	0	0	0	0	0
SAL_JA3037AA	CNSV-T3-MD11-1-TT-B	Bareilly	0	0	0	0	0	0	0	0	0	0	0
SAL_JA3064AA	CNSV-T2-MD10-10-RV-C	Bareilly	0	0	0	0	0	0	0	0	0	0	0
SAL_JA3056AA	CNSV-T2-MD10-10-TT-D	Bareilly	0	0	0	0	0	0	0	0	0	0	0
SAL_JA3050AA	CNSV-T3-MD11-10-TT-F	Bareilly	0	0	0	0	0	0	0	0	0	0	0
SAL_JA3065AA	CNSV-T2-MD10-10-RV-D	Bareilly	0	0	0	0	0	0	0	0	0	0	0
SAL_JA3066AA	CNSV-T2-MD10-10-RV-E	Bareilly	0	0	0	0	0	0	0	0	0	0	0
SAL_JA3067AA	CNSV-T2-MD10-10-RV-F	Bareilly	0	0	0	0	0	0	0	0	0	0	0
SAL_JA3038AA	CNSV-T2-MD10-10-TT-F	Bareilly	0	0	0	0	0	0	0	0	0	0	0
SAL_JA3039AA	CNSV-T2-MD10-10-TT-E	Bareilly	0	0	0	0	0	0	0	0	0	0	0
SAL_JA3044AA	CNSV-T3-MD11-1-TT-F	Bareilly	0	0	0	0	0	0	0	0	0	0	0
SAL_JA3043AA	CNSV-T3-MD11-1-TT-E	Bareilly	0	0	0	0	0	0	0	0	0	0	0
SAL_JA3062AA	CNSV-T2-MD10-10-RV-B	Bareilly	0	0	0	0	0	0	0	0	0	0	0
SAL_JA3041AA	CNSV-T3-MD11-1-TT-C	Bareilly	0	0	0	0	0	0	0	0	0	0	0
SAL_JA3040AA	CNSV-T3-MD11-1-TT-A	Bareilly	0	0	0	0	0	0	0	0	0	0	0
SAL_JA3046AA	CNSV-T3-MD11-10-TT-A	Bareilly	0	0	0	0	0	0	0	0	0	0	0
SAL_JA3045AA	CNSV-T3-MD11-10-TT-B	Bareilly	0	0	0	0	0	0	0	0	0	0	0
SAL_JA3936AA	PNUSAS012642	Bareilly	0	0	0	0	0	0	0	0	0	0	0
SAL_JA3782AA	PNUSAS012496	Bareilly	0	0	0	0	0	0	0	0	0	0	0
SAL_JA4254AA	CNSV-T1-MD09-1-RV-A	Bareilly	0	0	0	0	0	0	0	0	0	0	0
SAL_JA4280AA	CNSV-T1-MD04-10-RV-B	Bareilly	0	0	0	0	0	0	0	0	0	0	0
SAL_JA4246AA	CNSV-T1-MD04-10-RV-F	Bareilly	0	0	0	0	0	0	0	0	0	0	0
SAL_JA4257AA	CNSV-T1-MD04-10-RV-E	Bareilly	0	0	0	0	0	0	0	0	0	0	0
SAL_JA4240AA	CNSV-T1-MD09-1-RV-B	Bareilly	0	0	0	0	0	0	0	0	0	0	0
SAL_JA4261AA	CNSV-T1-MD04-10-RV-C	Bareilly	0	0	0	0	0	0	0	0	0	0	0
SAL_JA4258AA	CNSV-T1-MD09-1-RV-C	Bareilly	0	0	0	0	0	0	0	0	0	0	0
SAL_JA4256AA	FDA00002497	Bareilly	0	0	0	0	0	0	0	0	0	0	0
SAL_JA4259AA	CNSV-T1-MD04-10-RV-A	Bareilly	0	0	0	0	0	0	0	0	0	0	0
SAL_JA4271AA	CNSV-T1-MD04-10-RV-D	Bareilly	0	0	0	0	0	0	0	0	0	0	0
SAL_JA4461AA	CNSV-T3-MD11-1-RV-A	Bareilly	0	0	0	0	0	0	0	0	0	0	0
SAL_JA4859AA	CNSV-T3-MD11-10-RV-C	Bareilly	0	0	0	0	0	0	0	0	0	0	0
SAL_JA4857AA	CNSV-T3-MD11-1-RV-D	Bareilly	0	0	0	0	0	0	0	0	0	0	0
SAL_JA4858AA	CNSV-T3-MD11-1-RV-C	Bareilly	0	0	0	0	0	0	0	0	0	0	0
SAL_JA4861AA	CNSV-T3-MD11-10-RV-F	Bareilly	0	0	0	0	0	0	0	0	0	0	0
SAL_JA4862AA	CNSV-T3-MD11-10-RV-E	Bareilly	0	0	0	0	0	0	0	0	0	0	0
SAL_JA4860AA	CNSV-T3-MD11-10-RV-D	Bareilly	0	0	0	0	0	0	0	0	0	0	0
SAL_JA4985AA	PNUSAS013419	Bareilly	0	0	0	0	0	0	0	0	0	0	0
SAL_JA4921AA	CNSV-T1-MD09-10-TT-F	Bareilly	0	0	0	0	0	0	0	0	0	0	0
SAL_JA4910AA	CNSV-T1-MD09-1-TT-D	Bareilly	0	0	0	0	0	0	0	0	0	0	0
SAL_JA4916AA	CNSV-T1-MD09-10-TT-C	Bareilly	0	0	0	0	0	0	0	0	0	0	0
SAL_JA4925AA	PNUSAS012934	Bareilly	0	0	0	0	0	0	0	0	0	0	0
SAL_JA4911AA	CNSV-T1-MD09-1-TT-F	Bareilly	0	0	0	0	0	0	0	0	0	0	0
SAL_JA4912AA	CNSV-T1-MD09-1-TT-C	Bareilly	0	0	0	0	0	0	0	0	0	0	0
SAL_JA4882AA	PNUSAS013904	Bareilly	0	0	0	0	0	0	0	0	0	0	0
SAL_JA4907AA	CNSV-T1-MD09-1-TT-A	Bareilly	0	0	0	0	0	0	0	0	0	0	0
SAL_JA4909AA	CNSV-T1-MD09-1-TT-E	Bareilly	0	0	0	0	0	0	0	0	0	0	0
SAL_JA4917AA	CNSV-T1-MD09-10-TT-A	Bareilly	0	0	0	0	0	0	0	0	0	0	0
SAL_JA4908AA	CNSV-T1-MD09-1-TT-B	Bareilly	0	0	0	0	0	0	0	0	0	0	0
SAL_JA4878AA	PNUSAS013903	Bareilly	0	0	0	0	0	0	0	0	0	0	0
SAL_JA5111AA	PNUSAS013905	Bareilly	0	0	0	0	0	0	0	0	0	0	0
SAL_JA6563AA	363593	Bareilly	0	0	0	0	0	0	0	0	0	0	0
SAL_JA6317AA	PNUSAS012131	Bareilly	0	0	0	0	0	0	0	0	0	0	0
SAL_JA6081AA	PNUSAS012856	Bareilly	0	0	0	0	0	0	0	0	0	0	0
SAL_JA5979AA	PNUSAS011891	Bareilly	0	0	0	0	0	0	0	0	0	0	0
SAL_JA5906AA	348708	Bareilly	0	0	0	0	0	0	0	0	0	0	0
SAL_JA5409AA	348784	Bareilly	0	0	0	0	0	0	0	0	0	0	0
SAL_JA5324AA	PNUSAS013187	Bareilly	0	0	0	0	0	0	0	0	0	0	0
SAL_JA5695AA	336983	Bareilly	0	0	0	0	0	0	0	0	0	0	0
SAL_JA6905AA	369003	Bareilly	0	0	0	0	0	0	0	0	0	0	0

SAL_JA6884AA	352132	Bareilly	0	0	0	0	0	0	0	0	0	0	0
SAL_JA6817AA	PNUSAS013510	Bareilly	0	0	0	0	0	0	0	0	0	0	0
SAL_JA6802AA	PNUSAS010129	Bareilly	0	0	0	0	0	0	0	0	0	0	0
SAL_JA7390AA	PNUSAS012174	Bareilly	0	0	0	0	0	0	0	0	0	0	0
SAL_JA7366AA	PNUSAS013039	Bareilly	0	0	0	0	0	0	0	0	0	0	0
SAL_JA8251AA	343790	Bareilly	0	0	0	0	0	0	0	0	0	0	0
SAL_JA8241AA	356631	Bareilly	0	0	0	0	0	0	0	0	0	0	0
SAL_JA8206AA	347991	Bareilly	0	0	0	0	0	0	0	0	0	0	0
SAL_JA8149AA	360993	Bareilly	0	0	0	0	0	0	0	0	0	0	0
SAL_JA8059AA	356514	Bareilly	0	0	0	0	0	0	0	0	0	0	0
SAL_JA8040AA	352151	Bareilly	0	0	0	0	0	0	0	0	0	0	0
SAL_JA7945AA	369874	Bareilly	0	0	0	0	0	0	0	0	0	0	0
SAL_JA7904AA	356279	Bareilly	0	0	0	0	0	0	0	0	0	0	0
SAL_JA7894AA	345741	Bareilly	0	0	0	0	0	0	0	0	0	0	0
SAL_JA7586AA	PNUSAS010955	Bareilly	0	0	0	0	0	0	0	0	0	0	0
SAL_JA7580AA	PNUSAS010954	Bareilly	0	0	0	0	0	0	0	0	0	0	0
SAL_JA8008AA	355118	Bareilly	0	0	0	0	0	0	0	0	0	0	0
SAL_JA7789AA	348711	Bareilly	0	0	0	0	0	0	0	0	0	0	0
SAL_JA8768AA	PNUSAS015060	Bareilly	0	0	0	0	0	0	0	0	0	0	0
SAL_JA8763AA	PNUSAS014728	Bareilly	0	0	0	0	0	0	0	0	0	0	0
SAL_JA8772AA	PNUSAS014963	Bareilly	0	0	0	0	0	0	0	0	0	0	0
SAL_JA8496AA	PNUSAS015002	Bareilly	0	0	0	0	0	0	0	0	0	0	0
SAL_JA9653AA	CNSV-T3-MD10-10-RV-D	Bareilly	0	0	0	0	0	0	0	0	0	0	0
SAL_JA9651AA	CNSV-T3-MD10-10-RV-F	Bareilly	0	0	0	0	0	0	0	0	0	0	0
SAL_JA9557AA	CNSV-T3-MD10-10-RV-C	Bareilly	0	0	0	0	0	0	0	0	0	0	0
SAL_JA9341AA	PNUSAS014534	Bareilly	0	0	0	0	0	0	0	0	0	0	0
SAL_JA9077AA	PNUSAS015094	Bareilly	0	0	0	0	0	0	0	0	0	0	0
SAL_JA9039AA	PNUSAS013554	Bareilly	0	0	0	0	0	0	0	0	0	0	0
SAL_JA8923AA	PNUSAS015623	Bareilly	0	0	0	0	0	0	0	0	0	0	0
SAL_JA9854AA	PNUSAS014160	Bareilly	0	0	0	0	0	0	0	0	0	0	0
SAL_JA9851AA	PNUSAS014150	Bareilly	0	0	0	0	0	0	0	0	0	0	0
SAL_KA0083AA	PNUSAS015308	Bareilly	0	0	0	0	0	0	0	0	0	0	0
SAL_KA0089AA	PNUSAS014964	Bareilly	0	0	0	0	0	0	0	0	0	0	0
SAL_KA0773AA	PNUSAS015611	Bareilly	0	0	0	0	0	0	0	0	0	0	0
SAL_KA0433AA	PNUSAS014494	Bareilly	0	0	0	0	0	0	0	0	0	0	0
SAL_KA0406AA	PNUSAS015698	Bareilly	0	0	0	0	0	0	0	0	0	0	0
SAL_KA0347AA	PNUSAS016133	Bareilly	0	0	0	0	0	0	0	0	0	0	0
SAL_KA0346AA	PNUSAS016145	Bareilly	0	0	0	0	0	0	0	0	0	0	0
SAL_KA1155AA	2015AM-0769	Bareilly	0	0	0	0	0	0	0	0	0	0	0
SAL_KA2076AA	12-18-20170450	Bareilly	0	0	0	0	0	0	0	0	0	0	0
SAL_KA1745AA	PNUSAS015854	Bareilly	0	0	0	0	0	0	0	0	0	0	0
SAL_KA1700AA	PNUSAS016503	Bareilly	0	0	0	0	0	0	0	0	0	0	0
SAL_KA1805AA	PNUSAS015597	Bareilly	0	0	0	0	0	0	0	0	0	0	0
SAL_KA1809AA	PNUSAS016332	Bareilly	0	0	0	0	0	0	0	0	0	0	0
SAL_KA2142AA	FDA00011981	Bareilly	0	0	0	0	0	0	0	0	0	0	0
SAL_KA2143AA	FDA00011980	Bareilly	0	0	0	0	0	0	0	0	0	0	0
SAL_KA2118AA	FDA00011982	Bareilly	0	0	0	0	0	0	0	0	0	0	0
SAL_KA2908AA	PNUSAS016921	Bareilly	0	0	0	0	0	0	0	0	0	0	0
SAL_KA2698AA	PNUSAS015013	Bareilly	0	0	0	0	0	0	0	0	0	0	0
SAL_KA2688AA	PNUSAS016120	Bareilly	0	0	0	0	0	0	0	0	0	0	0
SAL_KA2618AA	PNUSAS016077	Bareilly	0	0	0	0	0	0	0	0	0	0	0
SAL_KA2432AA	PNUSAS015010	Bareilly	0	0	0	0	0	0	0	0	0	0	0
SAL_KA3437AA	PNUSAS017501	Bareilly	0	0	0	0	0	0	0	0	0	0	0
SAL_KA3349AA	PNUSAS016583	Bareilly	0	0	0	0	0	0	0	0	0	0	0
SAL_KA3336AA	PNUSAS016651	Bareilly	0	0	0	0	0	0	0	0	0	0	0
SAL_KA3199AA	PNUSAS016657	Bareilly	0	0	0	0	0	0	0	0	0	0	0
SAL_KA3201AA	PNUSAS016661	Bareilly	0	0	0	0	0	0	0	0	0	0	0
SAL_KA3231AA	PNUSAS016951	Bareilly	0	0	0	0	0	0	0	0	0	0	0
SAL_KA3649AA	PNUSAS016458	Bareilly	0	0	0	0	0	0	0	0	0	0	0
SAL_KA4843AA	PNUSAS015459	Bareilly	0	0	0	0	0	0	0	0	0	0	0
SAL_KA4850AA	PNUSAS017549	Bareilly	0	0	0	0	0	0	0	0	0	0	0
SAL_KA4854AA	PNUSAS017541	Bareilly	0	0	0	0	0	0	0	0	0	0	0
SAL_KA4857AA	PNUSAS017028	Bareilly	0	0	0	0	0	0	0	0	0	0	0
SAL_KA4752AA	PNUSAS017694	Bareilly	0	0	0	0	0	0	0	0	0	0	0
SAL_KA4721AA	PNUSAS018348	Bareilly	0	0	0	0	0	0	0	0	0	0	0
SAL_KA4716AA	PNUSAS017054	Bareilly	0	0	0	0	0	0	0	0	0	0	0
SAL_KA4683AA	PNUSAS018347	Bareilly	0	0	0	0	0	0	0	0	0	0	0
SAL_KA4687AA	PNUSAS017338	Bareilly	0	0	0	0	0	0	0	0	0	0	0
SAL_KA4054AA	RM_235	Bareilly	0	0	0	0	0	0	0	0	0	0	0
SAL_KA4062AA	RM_231	Bareilly	0	0	0	0	0	0	0	0	0	0	0
SAL_KA5047AA	FDA00003586	Bareilly	0	0	0	0	0	0	0	0	0	0	0
SAL_KA5454AA	PNUSAS018507	Bareilly	0	0	0	0	0	0	0	0	0	0	0
SAL_KA5371AA	PNUSAS015463	Bareilly	0	0	0	0	0	0	0	0	0	0	0

SAL_KA5299AA	PNUSAS017874	Bareilly	0	0	0	0	0	0	0	0	0	0	0
SAL_KA5191AA	PNUSAS018032	Bareilly	0	0	0	0	0	0	0	0	0	0	0
SAL_KA5185AA	PNUSAS017788	Bareilly	0	0	0	0	0	0	0	0	0	0	0
SAL_KA5636AA	PNUSAS018761	Bareilly	0	0	0	0	0	0	0	0	0	0	0
SAL_KA5947AA	PNUSAS019140	Bareilly	0	0	0	0	0	0	0	0	0	0	0
SAL_KA5934AA	PNUSAS017009	Bareilly	0	0	0	0	0	0	0	0	0	0	0
SAL_KA5825AA	PNUSAS019069	Bareilly	0	0	0	0	0	0	0	0	0	0	0
SAL_KA5991AA	PNUSAS017600	Bareilly	0	0	0	0	0	0	0	0	0	0	0
SAL_KA6049AA	PNUSAS018595	Bareilly	0	0	0	0	0	0	0	0	0	0	0
SAL_KA5974AA	PNUSAS018153	Bareilly	0	0	0	0	0	0	0	0	0	0	0
SAL_KA6350AA	PNUSAS018958	Bareilly	0	0	0	0	0	0	0	0	0	0	0
SAL_KA6793AA	PNUSAS013052	Bareilly	0	0	0	0	0	0	0	0	0	0	0
SAL_KA6786AA	PNUSAS013057	Bareilly	0	0	0	0	0	0	0	0	0	0	0
SAL_KA6799AA	PNUSAS013044	Bareilly	0	0	0	0	0	0	0	0	0	0	0
SAL_KA6651AA	PNUSAS018815	Bareilly	0	0	0	0	0	0	0	0	0	0	0
SAL_KA6451AA	2015AM-0960	Bareilly	0	0	0	0	0	0	0	0	0	0	0
SAL_KA7565AA	PNUSAS020189	Bareilly	0	0	0	0	0	0	0	0	0	0	0
SAL_KA7550AA	PNUSAS018358	Bareilly	0	0	0	0	0	0	0	0	0	0	0
SAL_KA7459AA	PNUSAS019142	Bareilly	0	0	0	0	0	0	0	0	0	0	0
SAL_KA7462AA	PNUSAS019148	Bareilly	0	0	0	0	0	0	0	0	0	0	0
SAL_KA7411AA	PNUSAS015564	Bareilly	0	0	0	0	0	0	0	0	0	0	0
SAL_KA7420AA	PNUSAS015557	Bareilly	0	0	0	0	0	0	0	0	0	0	0
SAL_KA7361AA	PNUSAS019535	Bareilly	0	0	0	0	0	0	0	0	0	0	0
SAL_KA7367AA	PNUSAS018299	Bareilly	0	0	0	0	0	0	0	0	0	0	0
SAL_KA7264AA	PNUSAS018698	Bareilly	0	0	0	0	0	0	0	0	0	0	0
SAL_KA8032AA	PNUSAS020076	Bareilly	0	0	0	0	0	0	0	0	0	0	0
SAL_KA8053AA	PNUSAS020081	Bareilly	0	0	0	0	0	0	0	0	0	0	0
SAL_KA7943AA	PNUSAS020429	Bareilly	0	0	0	0	0	0	0	0	0	0	0
SAL_KA7956AA	PNUSAS020261	Bareilly	0	0	0	0	0	0	0	0	0	0	0
SAL_KA7841AA	PNUSAS015495	Bareilly	0	0	0	0	0	0	0	0	0	0	0
SAL_KA7866AA	PNUSAS019875	Bareilly	0	0	0	0	0	0	0	0	0	0	0
SAL_KA7875AA	PNUSAS019878	Bareilly	0	0	0	0	0	0	0	0	0	0	0
SAL_KA7910AA	PNUSAS020087	Bareilly	0	0	0	0	0	0	0	0	0	0	0
SAL_KA8275AA	PNUSAS020249	Bareilly	0	0	0	0	0	0	0	0	0	0	0
SAL_KA8598AA	PNUSAS020128	Bareilly	0	0	0	0	0	0	0	0	0	0	0
SAL_KA8576AA	PNUSAS020131	Bareilly	0	0	0	0	0	0	0	0	0	0	0
SAL_KA8525AA	PNUSAS020321	Bareilly	0	0	0	0	0	0	0	0	0	0	0
SAL_KA9012AA	PNUSAS020500	Bareilly	0	0	0	0	0	0	0	0	0	0	0
SAL_KA9802AA	PNUSAS019767	Bareilly	0	0	0	0	0	0	0	0	0	0	0
SAL_KA9044AA	PNUSAS020611	Bareilly	0	0	0	0	0	0	0	0	0	0	0
SAL_KA9602AA	PNUSAS017122	Bareilly	0	0	0	0	0	0	0	0	0	0	0
SAL_KA9616AA	PNUSAS020007	Bareilly	0	0	0	0	0	0	0	0	0	0	0
SAL_KA9584AA	PNUSAS019828	Bareilly	0	0	0	0	0	0	0	0	0	0	0
SAL_KA9515AA	CNSV-T3-MD10-10-RV-B	Bareilly	0	0	0	0	0	0	0	0	0	0	0
SAL_KA9506AA	CNSV-T3-MD10-10-RV-A	Bareilly	0	0	0	0	0	0	0	0	0	0	0
SAL_LA0377AA	CNSV-T3-MD10-10-RV-E	Bareilly	0	0	0	0	0	0	0	0	0	0	0
SAL_LA0455AA	PNUSAS021375	Bareilly	0	0	0	0	0	0	0	0	0	0	0
SAL_LA0459AA	PNUSAS020047	Bareilly	0	0	0	0	0	0	0	0	0	0	0
SAL_LA1367AA	PNUSAS021791	Bareilly	0	0	0	0	0	0	0	0	0	0	0
SAL_LA1345AA	PNUSAS021719	Bareilly	0	0	0	0	0	0	0	0	0	0	0
SAL_LA1221AA	PNUSAS021429	Bareilly	0	0	0	0	0	0	0	0	0	0	0
SAL_LA1058AA	PNUSAS018403	Bareilly	0	0	0	0	0	0	0	0	0	0	0
SAL_LA3235AA	17039862	Bareilly	0	0	0	0	0	0	0	0	0	0	0
SAL_LA2241AA	PNUSAS019427	Bareilly	0	0	0	0	0	0	0	0	0	0	0
SAL_LA3762AA	PNUSAS021264	Bareilly	0	0	0	0	0	0	0	0	0	0	0
SAL_LA3774AA	PNUSAS015498	Bareilly	0	0	0	0	0	0	0	0	0	0	0
SAL_LA3717AA	PNUSAS021156	Bareilly	0	0	0	0	0	0	0	0	0	0	0
SAL_LA4049AA	RM_89	Bareilly	0	0	0	0	0	0	0	0	0	0	0
SAL_LA3627AA	PNUSAS021671	Bareilly	0	0	0	0	0	0	0	0	0	0	0
SAL_LA4205AA	PNUSAS021986	Bareilly	0	0	0	0	0	0	0	0	0	0	0
SAL_LA3948AA	PNUSAS021940	Bareilly	0	0	0	0	0	0	0	0	0	0	0
SAL_LA4110AA	PNUSAS021360	Bareilly	0	0	0	0	0	0	0	0	0	0	0
SAL_LA4392AA	PNUSAS021470	Bareilly	0	0	0	0	0	0	0	0	0	0	0
SAL_LA4394AA	PNUSAS021466	Bareilly	0	0	0	0	0	0	0	0	0	0	0
SAL_LA4487AA	PNUSAS023342	Bareilly	0	0	0	0	0	0	0	0	0	0	0
SAL_LA5028AA	PNUSAS023195	Bareilly	0	0	0	0	0	0	0	0	0	0	0
SAL_KA8433AA	PNUSAS019652	Bareilly	0	0	0	0	0	0	0	0	0	0	0
SAL_LA4883AA	PNUSAS022019	Bareilly	0	0	0	0	0	0	0	0	0	0	0
SAL_LA4888AA	PNUSAS022255	Bareilly	0	0	0	0	0	0	0	0	0	0	0
SAL_LA4890AA	PNUSAS022017	Bareilly	0	0	0	0	0	0	0	0	0	0	0
SAL_LA4849AA	PNUSAS022976	Bareilly	0	0	0	0	0	0	0	0	0	0	0
SAL_LA6867AA	PNUSAS023452	Bareilly	0	0	0	0	0	0	0	0	0	0	0
SAL_LA6839AA	PNUSAS023583	Bareilly	0	0	0	0	0	0	0	0	0	0	0

SAL_LA6538AA	PNUSAS023538	Bareilly	0	0	0	0	0	0	0	0	0	0	0
SAL_LA6552AA	PNUSAS019656	Bareilly	0	0	0	0	0	0	0	0	0	0	0
SAL_LA6485AA	PNUSAS022037	Bareilly	0	0	0	0	0	0	0	0	0	0	0
SAL_LA6515AA	PNUSAS019655	Bareilly	0	0	0	0	0	0	0	0	0	0	0
SAL_LA6428AA	PNUSAS021999	Bareilly	0	0	0	0	0	0	0	0	0	0	0
SAL_LA6439AA	PNUSAS024029	Bareilly	0	0	0	0	0	0	0	0	0	0	0
SAL_LA6482AA	PNUSAS023426	Bareilly	0	0	0	0	0	0	0	0	0	0	0
SAL_LA6503AA	PNUSAS023622	Bareilly	0	0	0	0	0	0	0	0	0	0	0
SAL_LA6304AA	MDP-12-00022	Bareilly	0	0	0	0	0	0	0	0	0	0	0
SAL_LA6258AA	MDP-12-00021	Bareilly	0	0	0	0	0	0	0	0	0	0	0
SAL_LA7361AA	PNUSAS013729	Bareilly	0	0	0	0	0	0	0	0	0	0	0
SAL_LA9776AA	PNUSAS022869	Bareilly	0	0	0	0	0	0	0	0	0	0	0
SAL_LA9817AA	PNUSAS022227	Bareilly	0	0	0	0	0	0	0	0	0	0	0
SAL_LA9746AA	PNUSAS023218	Bareilly	0	0	0	0	0	0	0	0	0	0	0
SAL_LA9265AA	PNUSAS023461	Bareilly	0	0	0	0	0	0	0	0	0	0	0
SAL_LA8997AA	PNUSAS023421	Bareilly	0	0	0	0	0	0	0	0	0	0	0
SAL_MA0197AA	PNUSAS025044	Bareilly	0	0	0	0	0	0	0	0	0	0	0
SAL_MA0161AA	PNUSAS024956	Bareilly	0	0	0	0	0	0	0	0	0	0	0
SAL_MA0177AA	PNUSAS021816	Bareilly	0	0	0	0	0	0	0	0	0	0	0
SAL_MA0029AA	PNUSAS023749	Bareilly	0	0	0	0	0	0	0	0	0	0	0
SAL_MA0513AA	PNUSAS025146	Bareilly	0	0	0	0	0	0	0	0	0	0	0
SAL_MA0415AA	PNUSAS024911	Bareilly	0	0	0	0	0	0	0	0	0	0	0
SAL_MA0424AA	PNUSAS024904	Bareilly	0	0	0	0	0	0	0	0	0	0	0
SAL_MA0425AA	PNUSAS024906	Bareilly	0	0	0	0	0	0	0	0	0	0	0
SAL_MA0376AA	PNUSAS024389	Bareilly	0	0	0	0	0	0	0	0	0	0	0
SAL_MA0984AA	PNUSAS025189	Bareilly	0	0	0	0	0	0	0	0	0	0	0
SAL_MA0898AA	PNUSAS023903	Bareilly	0	0	0	0	0	0	0	0	0	0	0
SAL_MA1129AA	MS170307	Bareilly	0	0	0	0	0	0	0	0	0	0	0
SAL_MA1182AA	PNUSAS023507	Bareilly	0	0	0	0	0	0	0	0	0	0	0
SAL_MA1445AA	PNUSAS024968	Bareilly	0	0	0	0	0	0	0	0	0	0	0
SAL_MA1349AA	PNUSAS024715	Bareilly	0	0	0	0	0	0	0	0	0	0	0
SAL_MA1669AA	PNUSAS024481	Bareilly	0	0	0	0	0	0	0	0	0	0	0
SAL_MA1679AA	PNUSAS025715	Bareilly	0	0	0	0	0	0	0	0	0	0	0
SAL_MA1638AA	PNUSAS025747	Bareilly	0	0	0	0	0	0	0	0	0	0	0
SAL_MA1579AA	PNUSAS025654	Bareilly	0	0	0	0	0	0	0	0	0	0	0
SAL_MA2479AA	25-14-20171031	Bareilly	0	0	0	0	0	0	0	0	0	0	0
SAL_MA2726AA	PNUSAS026112	Bareilly	0	0	0	0	0	0	0	0	0	0	0
SAL_MA2779AA	PNUSAS016312	Bareilly	0	0	0	0	0	0	0	0	0	0	0
SAL_MA2619AA	PNUSAS024387	Bareilly	0	0	0	0	0	0	0	0	0	0	0
SAL_MA3275AA	PNUSAS026446	Bareilly	0	0	0	0	0	0	0	0	0	16	0
SAL_MA3243AA	PNUSAS024713	Bareilly	0	0	0	0	0	0	0	0	0	0	0
SAL_MA3224AA	PNUSAS024299	Bareilly	0	0	0	0	0	0	0	0	0	0	0
SAL_MA4732AA	PNUSAS027047	Bareilly	0	0	0	0	0	0	0	0	0	0	0
SAL_MA4740AA	PNUSAS027054	Bareilly	0	0	0	0	0	0	0	0	0	0	0
SAL_MA4699AA	PNUSAS027064	Bareilly	0	0	0	0	0	0	0	0	0	0	0
SAL_MA4701AA	PNUSAS025988	Bareilly	0	0	0	0	0	0	0	0	0	0	0
SAL_MA4522AA	PNUSAS025758	Bareilly	0	0	0	0	0	0	0	0	0	0	0
SAL_MA4477AA	PNUSAS026579	Bareilly	0	0	0	0	0	0	0	0	0	0	0
SAL_MA4823AA	PNUSAS026485	Bareilly	0	0	0	0	0	0	0	0	0	0	0
SAL_MA5103AA	PNUSAS021519	Bareilly	0	0	0	0	0	0	0	0	0	0	0
SAL_MA5135AA	PNUSAS026299	Bareilly	0	0	0	0	0	0	0	0	0	0	0
SAL_DA1781AA	H130180329	Bareilly	0	0	0	0	0	0	0	0	0	0	0
SAL_EA0294AA	FCC0083	Bareilly	0	0	0	0	0	0	0	0	0	0	0
SAL_BA5844AA	M203 / NCTC20075	Bareilly	0	0	0	0	0	0	0	0	0	0	0
SAL_CA2357AA	31608	Bareilly	0	0	0	0	0	0	0	0	0	0	0
SAL_DA7983AA	FSW0127	Bareilly	0	0	0	0	0	0	0	0	0	0	0
SAL_DA0058AA	POR1-0056	Bareilly	0	0	0	0	0	0	0	0	0	0	0
SAL_BA5858AA	M202 / NCTC20075	Bareilly	0	0	0	0	0	0	0	0	0	0	0
SAL_BA6617AA	S-0497	Bareilly	0	0	0	0	0	0	0	0	0	0	0
SAL_CA1701AA	CFSAN000185	Bareilly	0	0	0	0	0	0	0	0	0	0	0
SAL_CA2903AA	84462	Bareilly	0	0	0	0	0	0	0	0	0	0	0
SAL_CA6321AA	46626	Bareilly	0	0	0	0	0	0	0	0	0	0	0
SAL_EA2783AA	DHMH-A15091834	Bareilly	0	0	0	0	0	0	0	0	0	0	0
SAL_DA9361AA	VA_WGS-00108	Bareilly	0	0	0	0	0	0	0	0	0	0	0
SAL_CA8001AA	FDA00002845	Bareilly	0	0	0	0	0	0	0	0	0	0	0
SAL_BA2136AA	CFSAN000189	Bareilly	0	0	0	0	0	0	0	0	0	0	0
SAL_HA5784AA	PNUSAS005740	Bareilly	0	0	0	0	0	0	0	0	0	0	0
SAL_HA9362AA	PNUSAS006305	Bareilly	0	0	0	0	0	0	0	0	0	0	0
SAL_HA9222AA	PNUSAS006706	Bareilly	0	0	0	0	0	0	0	0	0	0	0
SAL_HA4493AA	PNUSAS005066	Bareilly	0	0	0	0	0	0	0	0	0	0	0
SAL_IA0127AA	PNUSAS006263	Bareilly	0	0	0	0	0	0	0	0	0	0	0
SAL_GA1828AA	BCW_5233	Bareilly	1	1	1	4	0	0	1	1	1	1	1
SAL_GA7932AA	2014AM-1216	Bareilly	0	0	0	0	0	0	0	0	0	0	0

SAL_GA7011AA	CFSAN045033	Bareilly	0	0	0	0	0	0	0	0	0	0	0
SAL_FA4856AA	FDA00010067	Bareilly	0	0	0	0	0	0	0	0	0	0	0
SAL_FA4758AA	FSIS1606078	Bareilly	0	0	0	0	0	0	0	0	0	0	0
SAL_FA4368AA	197070	Bareilly	0	0	0	0	0	0	0	0	0	0	0
SAL_EA8675AA	VA-WGS-00649	Bareilly	0	0	0	0	0	0	0	0	0	0	0
SAL_EA6520AA	FDA00000298	Bareilly	0	0	0	0	0	0	0	0	0	0	0
SAL_EA6289AA	MOD1-Lipp-722	Bareilly	0	0	0	0	0	0	0	0	0	0	0
SAL_IA2541AA	PNUSAS007526	Bareilly	0	0	0	0	0	0	0	0	0	0	0
SAL_IA3740AA	PNUSAS006639	Bareilly	0	0	0	0	0	0	0	0	0	0	0
SAL_IA7485AA	PNUSAS009083	Bareilly	0	0	0	0	0	0	0	0	0	0	0
SAL_IA9580AA	PNUSAS009842	Bareilly	0	0	0	0	0	0	0	0	0	0	0
SAL_JA0253AA	PNUSAS009345	Bareilly	0	0	0	0	0	0	0	0	0	0	0
SAL_JA2018AA	PNUSAS011457	Bareilly	0	0	0	0	0	0	0	0	0	0	0
SAL_JA4462AA	CNSV-T3-MD11-1-RV-B	Bareilly	0	0	0	0	0	0	0	0	0	0	0
SAL_JA2644AA	PNUSAS011568	Bareilly	0	0	0	0	0	0	0	0	0	0	0
SAL_JA2819AA	VA-WGS-17062	Bareilly	0	0	0	0	0	0	0	0	0	0	0
SAL_JA2996AA	PNUSAS012198	Bareilly	0	0	0	0	0	0	0	0	0	0	0
SAL_JA5486AA	335537	Bareilly	0	0	0	0	0	0	0	0	0	0	0
SAL_JA6643AA	PNUSAS011968	Bareilly	0	0	0	0	0	0	0	0	0	0	0
SAL_JA7564AA	PNUSAS013600	Bareilly	0	0	0	0	0	0	0	0	0	0	0
SAL_JA8097AA	356522	Bareilly	0	0	0	0	0	0	0	0	0	0	0
SAL_JA8634AA	PNUSAS014482	Bareilly	0	0	0	0	0	0	0	0	0	0	0
SAL_JA9866AA	PNUSAS014159	Bareilly	0	0	0	0	0	0	0	0	0	0	0
SAL_KA0675AA	2015AM-1823	Bareilly	0	0	0	0	0	0	0	0	0	0	0
SAL_KA1315AA	PNUSAS016407	Bareilly	0	0	0	0	0	0	0	0	0	0	0
SAL_KA5464AA	PNUSAS017862	Bareilly	0	0	0	0	0	0	0	0	0	0	0
SAL_KA7404AA	PNUSAS015957	Bareilly	0	0	0	0	0	0	0	0	0	0	0
SAL_KA6597AA	PNUSAS019209	Bareilly	0	0	0	0	0	0	0	0	0	0	0
SAL_KA6749AA	PNUSAS018099	Bareilly	0	0	0	0	0	0	0	0	0	0	0
SAL_KA7349AA	PNUSAS019540	Bareilly	0	0	0	0	0	0	0	0	0	0	0
SAL_KA9043AA	PNUSAS020624	Bareilly	0	0	0	0	0	0	0	0	0	0	0
SAL_KA9596AA	PNUSAS017120	Bareilly	0	0	0	0	0	0	0	0	0	0	0
SAL_LA1352AA	PNUSAS021805	Bareilly	0	0	0	0	0	0	0	0	0	0	0
SAL_LA4155AA	PNUSAS021614	Bareilly	0	0	0	0	0	0	0	0	0	0	0
SAL_LA4111AA	PNUSAS021368	Bareilly	0	0	0	0	0	0	0	0	0	0	0
SAL_LA4957AA	PNUSAS021542	Bareilly	0	0	0	0	0	0	0	0	0	0	0
SAL_LA6678AA	PNUSAS024193	Bareilly	0	0	0	0	0	0	0	0	0	0	0
SAL_LA9243AA	PNUSAS022695	Bareilly	0	0	0	0	0	0	0	0	0	0	0
SAL_MA2635AA	PNUSAS023415	Bareilly	0	0	0	0	0	0	0	0	0	0	0
SAL_MA3375AA	PNUSAS024546	Bareilly	0	0	0	0	0	0	0	0	0	0	0
SAL_MA5604AA	PNUSAS027102	Bareilly	0	0	0	0	0	0	0	0	0	0	0
SAL_MA6383AA	PNUSAS027602	Bareilly	0	0	0	0	0	0	0	0	0	0	0
SAL_MA6655AA	PNUSAS027137	Bareilly	0	0	0	0	0	0	0	0	0	0	0
SAL_MA6583AA	PNUSAS027699	Bareilly	0	0	0	0	0	0	0	0	0	0	0
SAL_MA6576AA	PNUSAS027706	Bareilly	0	0	0	0	0	0	0	0	0	0	0
SAL_MA6578AA	PNUSAS027708	Bareilly	0	0	0	0	0	0	0	0	0	0	0
SAL_MA6748AA	345403	Bareilly	0	0	0	0	0	0	0	0	0	0	0
SAL_MA7177AA	PNUSAS025169	Bareilly	0	0	0	0	0	0	0	0	0	0	0
SAL_MA7119AA	PNUSAS023748	Bareilly	0	0	0	0	0	0	0	0	0	0	0
SAL_MA7114AA	PNUSAS025537	Bareilly	0	0	0	0	0	0	0	0	0	0	0
SAL_MA7097AA	PNUSAS025166	Bareilly	0	0	0	0	0	0	0	0	0	0	0
SAL_MA7071AA	PNUSAS025445	Bareilly	0	0	0	0	0	0	0	0	0	0	0
SAL_MA7032AA	PNUSAS025173	Bareilly	0	0	0	0	0	0	0	0	0	0	0
SAL_MA7057AA	PNUSAS026010	Bareilly	0	0	0	0	0	0	0	0	0	0	0
SAL_MA7047AA	PNUSAS025163	Bareilly	0	0	0	0	0	0	0	0	0	0	0
SAL_MA7020AA	PNUSAS027304	Bareilly	0	0	0	0	0	0	0	0	0	0	0
SAL_MA7642AA	PNUSAS028554	Bareilly	0	0	0	0	0	0	0	0	0	0	0
SAL_MA7652AA	PNUSAS028558	Bareilly	0	0	0	0	0	0	0	0	0	0	0
SAL_MA7785AA	PNUSAS028467	Bareilly	0	0	0	0	0	0	0	0	0	0	0
SAL_MA7722AA	PNUSAS026907	Bareilly	0	0	0	0	0	0	0	0	0	0	0
SAL_MA7885AA	PNUSAS026135	Bareilly	0	0	0	0	0	0	0	0	0	0	0
SAL_MA7711AA	PNUSAS026738	Bareilly	0	0	0	0	0	0	0	0	0	0	0
SAL_MA7756AA	PNUSAS028190	Bareilly	0	0	0	0	0	0	0	0	0	0	0
SAL_MA8073AA	103624	Bareilly	0	0	0	0	0	0	0	0	0	0	0
SAL_MA8378AA	PNUSAS027154	Bareilly	0	0	0	0	0	0	0	0	0	0	0
SAL_MA8310AA	PNUSAS027358	Bareilly	0	0	0	0	0	0	0	0	0	0	0
SAL_MA8323AA	PNUSAS027352	Bareilly	0	0	0	0	0	0	0	0	0	0	0
SAL_MA8244AA	PNUSAS027650	Bareilly	0	0	0	0	0	0	0	0	0	0	0
SAL_MA8213AA	PNUSAS029088	Bareilly	0	0	0	0	0	0	0	0	0	16	0
SAL_MA8234AA	PNUSAS027658	Bareilly	0	0	0	0	0	0	0	0	0	0	0
SAL_MA8212AA	PNUSAS029087	Bareilly	0	0	0	0	0	0	0	0	0	16	0
SAL_NA0767AA	32-8-8101279	Bareilly	0	0	0	0	0	0	0	0	0	0	0
SAL_NA0001AA	almonella_Bareilly_78-1	Bareilly	0	0	0	0	0	0	0	0	0	0	0

SAL_NA0735AA	PNUSAS029264	Bareilly	0	0	0	0	0	0	0	0	0	0	0
SAL_NA0750AA	PNUSAS029540	Bareilly	0	0	0	0	0	0	0	0	0	0	0
SAL_NA1139AA	PNUSAS026798	Bareilly	0	0	0	0	0	0	0	0	0	0	0
SAL_LA3351AA	MS170242	Bareilly	0	0	0	0	0	0	0	0	0	0	0
SAL_NA1295AA	PNUSAS029630	Bareilly	0	0	0	0	0	0	0	0	0	0	0
SAL_NA1747AA	PNUSAS029054	Bareilly	0	0	0	0	0	0	0	0	0	0	0
SAL_NA1728AA	PNUSAS027880	Bareilly	0	0	0	0	0	0	0	0	0	0	0
SAL_NA1705AA	PNUSAS030055	Bareilly	0	0	0	0	0	0	0	0	0	16	0
SAL_NA2485AA	PNUSAS029347	Bareilly	0	0	0	0	0	0	0	0	0	0	0
SAL_NA2469AA	PNUSAS029926	Bareilly	0	0	0	0	0	0	0	0	0	0	0
SAL_NA2566AA	PNUSAS030212	Bareilly	0	0	0	0	0	0	0	0	0	0	0
SAL_NA2997AA	PNUSAS030169	Bareilly	0	0	0	0	0	0	0	0	0	0	0
SAL_NA2985AA	PNUSAS023396	Bareilly	0	0	0	0	0	0	0	0	0	0	0
SAL_NA2672AA	PNUSAS030074	Bareilly	0	0	0	0	0	0	0	0	0	0	0
SAL_NA3122AA	PNUSAS029985	Bareilly	0	0	0	0	0	0	0	0	0	0	0
SAL_NA3069AA	PNUSAS030319	Bareilly	0	0	0	0	0	0	0	0	0	0	0
SAL_NA3014AA	PNUSAS030472	Bareilly	0	0	0	0	0	0	0	0	0	0	0
SAL_NA3200AA	PNUSAS030328	Bareilly	0	0	0	0	0	0	0	0	0	0	0
SAL_NA3182AA	PNUSAS030642	Bareilly	0	0	0	0	0	0	0	0	0	0	0
SAL_NA3416AA	PNUSAS030664	Bareilly	0	0	0	0	0	0	0	0	0	0	0
SAL_NA3766AA	PNUSAS030731	Bareilly	0	0	0	0	0	0	0	0	0	0	0
SAL_NA3778AA	PNUSAS028368	Bareilly	0	0	0	0	0	0	0	0	0	0	0
SAL_NA3786AA	PNUSAS030879	Bareilly	0	0	0	0	0	0	0	0	0	0	0
SAL_NA3552AA	PNUSAS030996	Bareilly	0	0	0	0	0	0	0	0	0	0	0
SAL_NA3858AA	2015AM-2566	Bareilly	0	0	0	0	0	0	0	0	0	0	0
SAL_NA3878AA	2015AM-2467	Bareilly	0	0	0	0	0	0	0	0	0	0	0
SAL_NA4226AA	PNUSAS031012	Bareilly	0	0	0	0	0	0	0	0	0	0	0
SAL_NA4112AA	PNUSAS031106	Bareilly	0	0	0	0	0	0	0	0	0	0	0
SAL_NA4310AA	PNUSAS031263	Bareilly	0	0	0	0	0	0	0	0	0	0	0
SAL_NA4462AA	PNUSAS031276	Bareilly	0	0	0	0	0	0	0	0	0	0	0
SAL_NA5120AA	PNUSAS031688	Bareilly	0	0	0	0	0	0	0	0	0	0	0
SAL_NA5313AA	2015AM-2241	Bareilly	0	0	0	0	0	0	0	0	0	0	0
SAL_NA5305AA	2015AM-2242	Bareilly	0	0	0	0	0	0	0	0	0	0	0
SAL_NA5284AA	2015AM-1989	Bareilly	0	0	0	0	0	0	0	0	0	0	0
SAL_NA5566AA	PNUSAS031634	Bareilly	0	0	0	0	0	0	0	0	0	0	0
SAL_NA5576AA	PNUSAS032145	Bareilly	0	0	0	0	0	0	0	0	0	0	0
SAL_NA5603AA	2015AM-1963	Bareilly	0	0	0	0	0	0	0	0	0	0	0
SAL_NA5622AA	2015AM-0942	Bareilly	0	0	0	0	0	0	0	0	0	0	0
SAL_NA5595AA	2015AM-1964	Bareilly	0	0	0	0	0	0	0	0	0	0	0
SAL_NA5550AA	PNUSAS031641	Bareilly	0	0	0	0	0	0	0	0	0	0	0
SAL_NA5482AA	PNUSAS031728	Bareilly	0	0	0	0	0	0	0	0	0	0	0
SAL_NA6145AA	2015AM-2530	Bareilly	0	0	0	0	0	0	0	0	0	0	0
SAL_NA6092AA	2015AM-2466	Bareilly	0	0	0	0	0	0	0	0	0	0	0
SAL_NA6070AA	2015AM-2567	Bareilly	0	0	0	0	0	0	0	0	0	0	0
SAL_NA5926AA	PNUSAS028422	Bareilly	0	0	0	0	0	0	0	0	0	0	0
SAL_NA5824AA	2015AM-2748	Bareilly	0	0	0	0	0	0	0	0	0	0	0
SAL_NA6590AA	PNUSAS031996	Bareilly	0	0	0	0	0	0	0	0	0	0	0
SAL_NA6452AA	2015AM-2938	Bareilly	0	0	0	0	0	0	0	0	0	0	0
SAL_NA6937AA	PNUSAS032017	Bareilly	0	0	0	0	0	0	0	0	0	0	0
SAL_NA6947AA	PNUSAS032010	Bareilly	0	0	0	0	0	0	0	0	0	0	0
SAL_NA6925AA	PNUSAS032031	Bareilly	0	0	0	0	0	0	0	0	0	0	0
SAL_NA6891AA	PNUSAS032170	Bareilly	0	0	0	0	0	0	0	0	0	0	0
SAL_NA8056AA	387194	Bareilly	0	0	0	0	0	0	0	0	0	0	0
SAL_NA8073AA	423893	Bareilly	0	0	0	0	0	0	0	0	0	0	0
SAL_NA8034AA	385823	Bareilly	0	0	0	0	0	0	0	0	0	0	0
SAL_NA8049AA	394023	Bareilly	0	0	0	0	0	0	0	0	0	0	0
SAL_NA7596AA	PNUSAS032181	Bareilly	0	0	0	0	0	0	0	0	0	0	0
SAL_NA7603AA	PNUSAS032498	Bareilly	0	0	0	0	0	0	0	0	0	0	0
SAL_NA7453AA	2015AM-3480	Bareilly	0	0	0	0	0	0	0	0	0	0	0
SAL_NA7294AA	PNUSAS028440	Bareilly	0	0	0	0	0	0	0	0	0	0	0
SAL_NA8433AA	PNUSAS032916	Bareilly	0	0	0	0	0	0	0	0	0	0	0
SAL_NA8326AA	PNUSAS032410	Bareilly	0	0	0	0	0	0	0	0	0	0	0
SAL_NA8340AA	PNUSAS029394	Bareilly	0	0	0	0	0	0	0	0	0	0	0
SAL_NA8355AA	PNUSAS029376	Bareilly	0	0	0	0	0	0	0	0	0	0	0
SAL_NA8822AA	PNUSAS033158	Bareilly	0	0	0	0	0	0	0	0	0	16	0
SAL_NA8710AA	PNUSAS032888	Bareilly	0	0	0	0	0	0	0	0	0	0	0
SAL_NA9421AA	PNUSAS028453	Bareilly	0	0	0	0	0	0	0	0	0	0	0
SAL_NA9380AA	PNUSAS033307	Bareilly	0	0	0	0	0	0	0	0	0	0	0
SAL_NA9381AA	PNUSAS033312	Bareilly	0	0	0	0	0	0	0	0	0	0	0
SAL_NA9468AA	PNUSAS033327	Bareilly	0	0	0	0	0	0	0	0	0	0	0
SAL_OA0136AA	PNUSAS027940	Bareilly	0	0	0	0	0	0	0	0	0	0	0
SAL_OA0121AA	PNUSAS028147	Bareilly	0	0	0	0	0	0	0	0	0	0	0
SAL_OA0046AA	PNUSAS030678	Bareilly	0	0	0	0	0	0	0	0	0	0	0

SAL_NA9903AA	PNUSAS033430	Bareilly	0	0	0	0	0	0	0	0	0	0	0
SAL_NA9835AA	PNUSAS033998	Bareilly	0	0	0	0	0	0	0	0	0	0	0
SAL_OA0634AA	PNUSAS034353	Bareilly	0	0	0	0	0	0	0	0	0	0	0
SAL_OA1149AA	PNUSAS033374	Bareilly	0	0	0	0	0	0	0	0	0	0	0
SAL_OA5956AA	100493	Bareilly	0	0	0	0	0	0	0	0	0	0	0
SAL_OA5744AA	PNUSAS033893	Bareilly	0	0	0	0	0	0	0	0	0	0	0
SAL_OA7417AA	PNUSAS036850	Bareilly	0	0	0	0	0	0	0	0	0	0	0
SAL_OA7428AA	PNUSAS036325	Bareilly	0	0	0	0	0	0	0	0	0	0	0
SAL_OA5235AA	PNUSAS035493	Bareilly	0	0	0	0	0	0	0	0	0	0	0
SAL_PA0061AA	PNUSAS037916	Bareilly	0	0	0	0	0	0	0	0	0	0	0
SAL_OA9957AA	PNUSAS037735	Bareilly	0	0	0	0	0	0	0	0	0	0	0
SAL_OA7844AA	PNUSAS035704	Bareilly	0	0	0	0	0	0	0	0	0	0	0
SAL_OA7738AA	PNUSAS036970	Bareilly	0	0	0	0	0	0	0	0	0	0	0
SAL_OA5037AA	PNUSAS036711	Bareilly	0	0	0	0	0	0	0	0	0	0	0
SAL_OA4940AA	PNUSAS035547	Bareilly	0	0	0	0	0	0	0	0	0	0	0
SAL_OA4731AA	PNUSAS036660	Bareilly	0	0	0	0	0	0	0	0	0	0	0
SAL_OA4634AA	PNUSAS035146	Bareilly	0	0	0	0	0	0	0	0	0	0	0
SAL_OA4635AA	PNUSAS035796	Bareilly	0	0	0	0	0	0	0	0	0	0	0
SAL_OA4609AA	PNUSAS033209	Bareilly	0	0	0	0	0	0	0	0	0	0	0
SAL_OA4585AA	PNUSAS036929	Bareilly	0	0	0	0	0	0	0	0	0	0	0
SAL_OA4512AA	PNUSAS035750	Bareilly	0	0	0	0	0	0	0	0	0	0	0
SAL_OA4472AA	PNUSAS032266	Bareilly	0	0	0	0	0	0	0	0	0	0	0
SAL_OA8090AA	PNUSAS037758	Bareilly	0	0	0	0	0	0	0	0	0	0	0
SAL_OA8053AA	PNUSAS037332	Bareilly	0	0	0	0	0	0	0	0	0	0	0
SAL_OA8112AA	CFSAN058606	Bareilly	0	0	0	0	0	0	0	0	0	0	0
SAL_OA8211AA	CFSAN058592	Bareilly	0	0	0	0	0	0	0	0	0	0	0
SAL_OA4950AA	PNUSAS035540	Bareilly	0	0	0	0	0	0	0	0	0	0	0
SAL_PA0601AA	PNUSAS037523	Bareilly	0	0	0	0	0	0	0	0	0	0	0
SAL_PA0696AA	CFSAN071967	Bareilly	0	0	0	0	0	0	0	0	0	0	0
SAL_PA0838AA	PNUSAS037628	Bareilly	0	0	0	0	0	0	0	0	0	0	0
SAL_PA1025AA	PNUSAS038138	Bareilly	0	0	0	0	0	0	0	0	0	0	0
SAL_PA1329AA	PNUSAS038109	Bareilly	0	0	0	0	0	0	0	0	0	0	0
SAL_PA1680AA	PNUSAS038679	Bareilly	0	0	0	0	0	0	0	0	0	0	0
SAL_PA3367AA	PNUSAS039121	Bareilly	0	0	0	0	0	0	0	0	0	0	0
SAL_PA4153AA	CNSV-T11-MD05-10-RV-E	Bareilly	0	0	0	0	0	0	0	0	0	0	0
SAL_PA4128AA	CNSV-T11-MD05-10-TT-C	Bareilly	0	0	0	0	0	0	0	0	0	0	0
SAL_PA4116AA	CNSV-T11-MD05-10-TT-E	Bareilly	0	0	0	0	0	0	0	0	0	0	0
SAL_PA4269AA	424572	Bareilly	0	0	0	0	0	0	0	0	0	0	0
SAL_PA4248AA	113026	Bareilly	0	0	0	0	0	0	0	0	0	0	0
SAL_PA4465AA	PNUSAS039741	Bareilly	0	0	0	0	0	0	0	0	0	0	0
SAL_PA4372AA	PNUSAS039557	Bareilly	0	0	0	0	0	0	0	0	0	0	0
SAL_PA4378AA	PNUSAS039556	Bareilly	0	0	0	0	0	0	0	0	0	0	0
SAL_PA4793AA	227069	Bareilly	0	0	0	0	0	0	0	0	0	0	0
SAL_PA4803AA	391119	Bareilly	0	0	0	0	0	0	0	0	0	0	0
SAL_PA5379AA	CNSV-T13-MD04-10-RV-C	Bareilly	0	0	0	0	0	0	0	0	0	0	0
SAL_PA5277AA	CNSV-T11-MD10-10-RV-A	Bareilly	0	0	0	0	0	0	0	0	0	0	0
SAL_PA5279AA	CNSV-T11-MD10-10-RV-C	Bareilly	0	0	0	0	0	0	0	0	0	0	0
SAL_PA5281AA	CNSV-T11-MD10-10-RV-E	Bareilly	0	0	0	0	0	0	0	0	0	0	0
SAL_PA5228AA	CNSV-T11-MD04-1-RV-A	Bareilly	0	0	0	0	0	0	0	0	0	0	0
SAL_PA5769AA	136764	Bareilly	0	0	0	0	0	0	0	0	0	0	0
SAL_PA5775AA	137135	Bareilly	0	0	0	0	0	0	0	0	0	0	0
SAL_PA5774AA	137134	Bareilly	0	0	0	0	0	0	0	0	0	0	0
SAL_PA5723AA	PNUSAS040033	Bareilly	0	0	0	0	0	0	0	0	0	0	0
SAL_PA5698AA	PNUSAS039539	Bareilly	0	0	0	0	0	0	0	0	0	0	0
SAL_PA5868AA	PNUSAS040348	Bareilly	0	0	0	0	0	0	0	0	0	0	0
SAL_PA5912AA	PNUSAS040395	Bareilly	0	0	0	0	0	0	0	0	0	0	0
SAL_PA6245AA	222381	Bareilly	0	0	0	0	0	0	0	0	0	0	0
SAL_PA7055AA	PNUSAS040608	Bareilly	0	0	0	0	0	0	0	0	0	0	0
SAL_PA7070AA	PNUSAS040609	Bareilly	0	0	0	0	0	0	0	0	0	0	0
SAL_PA6974AA	PNUSAS040509	Bareilly	0	0	0	0	0	0	0	0	0	0	0
SAL_PA6983AA	PNUSAS040788	Bareilly	0	0	0	0	0	0	0	0	0	0	0
SAL_PA6932AA	PNUSAS040650	Bareilly	0	0	0	0	0	0	0	0	0	0	0
SAL_PA6913AA	PNUSAS040648	Bareilly	0	0	0	0	0	0	0	0	0	0	0
SAL_PA6943AA	PNUSAS039537	Bareilly	0	0	0	0	0	0	0	0	0	0	0
SAL_PA6593AA	253401	Bareilly	0	0	0	0	0	0	0	0	0	0	0
SAL_PA7289AA	286809	Bareilly	0	0	0	0	0	0	0	0	0	0	0
SAL_PA7257AA	311421	Bareilly	0	0	0	0	0	0	0	0	0	0	0
SAL_PA7194AA	CNSV-T13-MD04-10-TT-C	Bareilly	0	0	0	0	0	0	0	0	0	0	0
SAL_PA7363AA	PNUSAS040728	Bareilly	0	0	0	0	0	0	0	0	0	0	0
SAL_PA7515AA	268659	Bareilly	0	0	0	0	0	0	0	0	0	0	0
SAL_PA7771AA	319733	Bareilly	0	0	0	0	0	0	0	0	0	0	0
SAL_PA7705AA	CNSV-T14-MD05-10-TT-E	Bareilly	0	0	0	0	0	0	0	0	0	0	0
SAL_PA8066AA	416297	Bareilly	0	0	0	0	0	0	0	0	0	0	0

SAL_PA7920AA	169647	Bareilly	0	0	0	0	0	0	0	0	0	0	0
SAL_PA7865AA	254645	Bareilly	0	0	0	0	0	0	0	0	0	0	0
SAL_PA8330AA	393902	Bareilly	0	0	0	0	0	0	0	0	0	0	0
SAL_PA8353AA	437045	Bareilly	0	0	0	0	0	0	0	0	0	0	0
SAL_PA7854AA	455109	Bareilly	0	0	0	0	0	0	0	0	0	0	0
SAL_PA7824AA	291817	Bareilly	0	0	0	0	0	0	0	0	0	0	0
SAL_PA7826AA	376207	Bareilly	0	0	0	0	0	0	0	0	0	0	0
SAL_PA8770AA	PNUSAS041231	Bareilly	0	0	0	0	0	0	0	0	0	0	0
SAL_PA8681AA	527496	Bareilly	0	0	0	0	0	0	0	0	0	0	0
SAL_PA8497AA	302874	Bareilly	0	0	0	0	0	0	0	0	0	0	0
SAL_PA8613AA	307465	Bareilly	0	0	0	0	0	0	0	0	0	0	0
SAL_PA9026AA	374036	Bareilly	0	0	0	0	0	0	0	0	0	0	0
SAL_PA9030AA	261438	Bareilly	0	0	0	0	0	0	0	0	0	0	0
SAL_PA9031AA	227031	Bareilly	0	0	0	0	0	0	0	0	0	0	0
SAL_PA9520AA	301906	Bareilly	0	0	0	0	0	0	0	0	0	0	0
SAL_PA9464AA	329896	Bareilly	0	0	0	0	0	0	0	0	0	0	0
SAL_PA9802AA	PNUSAS041821	Bareilly	0	0	0	0	0	0	0	0	0	0	0
SAL_PA9962AA	383795	Bareilly	0	0	0	0	0	0	0	0	0	0	0
SAL_QA0053AA	PNUSAS041921	Bareilly	0	0	0	0	0	0	0	0	0	0	0
SAL_QA0016AA	PNUSAS041956	Bareilly	0	0	0	0	0	0	0	0	0	0	0
SAL_QA0252AA	CNSV-T14-MD05-10-TT-C	Bareilly	0	0	0	0	0	0	0	0	0	0	0
SAL_QA0243AA	CNSV-T15-MD11-1-TT-C	Bareilly	0	0	0	0	0	0	0	0	0	0	0
SAL_QA0250AA	CNSV-T14-MD05-10-RV-E	Bareilly	0	0	0	0	0	0	0	0	0	0	0
SAL_QA0514AA	379339	Bareilly	0	0	0	0	0	0	0	0	0	0	0
SAL_QA0506AA	414658	Bareilly	0	0	0	0	0	0	0	0	0	0	0
SAL_QA0465AA	470209	Bareilly	0	0	0	0	0	0	0	0	0	0	0
SAL_QA0684AA	188376	Bareilly	0	0	0	0	0	0	0	0	0	0	0
SAL_QA0689AA	453219	Bareilly	0	0	0	0	0	0	0	0	0	0	0
SAL_QA0816AA	PNUSAS042154	Bareilly	0	0	0	0	0	0	0	0	0	0	0
SAL_QA0807AA	PNUSAS042157	Bareilly	0	0	0	0	0	0	0	0	0	0	0
SAL_QA0785AA	PNUSAS019347	Bareilly	0	0	0	0	0	0	0	0	0	0	0
SAL_QA0786AA	PNUSAS019339	Bareilly	0	0	0	0	0	0	0	0	0	0	0
SAL_QA0783AA	PNUSAS019337	Bareilly	0	0	0	0	0	0	0	0	0	0	0
SAL_QA0794AA	PNUSAS019341	Bareilly	0	0	0	0	0	0	0	0	0	0	0
SAL_QA1342AA	385781	Bareilly	0	0	0	0	0	0	0	0	0	0	0
SAL_QA1314AA	166287	Bareilly	0	0	0	0	0	0	0	0	0	0	0
SAL_QA1108AA	PNUSAS042461	Bareilly	0	0	0	0	0	0	0	0	0	0	0
SAL_QA3777AA	24-4-18-8100506-L	Bareilly	0	0	0	0	0	0	0	0	0	0	0
SAL_QA1020AA	PNUSAS042430	Bareilly	0	0	0	0	0	0	0	0	0	0	0
SAL_QA1023AA	PNUSAS042434	Bareilly	0	0	0	0	0	0	0	0	0	0	0
SAL_QA0864AA	183734	Bareilly	0	0	0	0	0	0	0	0	0	0	0
SAL_QA0838AA	196371	Bareilly	0	0	0	0	0	0	0	0	0	0	0
SAL_QA0840AA	542077	Bareilly	0	0	0	0	0	0	0	0	0	0	0
SAL_QA1521AA	205745	Bareilly	0	0	0	0	0	0	0	0	0	0	0
SAL_QA1889AA	127523	Bareilly	0	0	0	0	0	0	0	0	0	0	0
SAL_QA1814AA	459911	Bareilly	0	0	0	0	0	0	0	0	0	0	0
SAL_QA1748AA	376053	Bareilly	0	0	0	0	0	0	0	0	0	0	0
SAL_QA1734AA	271080	Bareilly	0	0	0	0	0	0	0	0	0	0	0
SAL_QA2512AA	435395	Bareilly	0	0	0	0	0	0	0	0	0	0	0
SAL_QA2517AA	341378	Bareilly	0	0	0	0	0	0	0	0	0	0	0
SAL_QA2481AA	505949	Bareilly	0	0	0	0	0	0	0	0	0	0	0
SAL_QA2213AA	242405	Bareilly	0	0	0	0	0	0	0	0	0	0	0
SAL_QA2154AA	191875	Bareilly	0	0	0	0	0	0	0	0	0	0	0
SAL_QA2059AA	260056	Bareilly	0	0	0	0	0	0	0	0	0	0	0
SAL_QA2004AA	427573	Bareilly	0	0	0	0	0	0	0	0	0	0	0
SAL_QA6300AA	CZ_17_0491	Bareilly	0	0	0	0	0	0	0	0	0	0	0
SAL_QA2897AA	178730	Bareilly	0	0	0	0	0	0	0	0	0	0	0
SAL_QA2854AA	301638	Bareilly	0	0	0	0	0	0	0	0	0	0	0
SAL_QA2712AA	253450	Bareilly	0	0	0	0	0	0	0	0	0	0	0
SAL_QA2720AA	158982	Bareilly	0	0	0	0	0	0	0	0	0	0	0
SAL_QA3491AA	205744	Bareilly	0	0	0	0	0	0	0	0	0	0	0
SAL_QA3475AA	245523	Bareilly	0	0	0	0	0	0	0	0	0	0	0
SAL_QA3449AA	543860	Bareilly	0	0	0	0	0	0	0	0	0	0	0
SAL_QA3383AA	531297	Bareilly	0	0	0	0	0	0	0	0	0	0	0
SAL_QA3350AA	255568	Bareilly	0	0	0	0	0	0	0	0	0	0	0
SAL_QA3304AA	486511	Bareilly	0	0	0	0	0	0	0	0	0	0	0
SAL_QA3285AA	313246	Bareilly	0	0	0	0	0	0	0	0	0	0	0
SAL_QA3217AA	491026	Bareilly	0	0	0	0	0	0	0	0	0	0	0
SAL_QA3706AA	PNUSAS042361	Bareilly	0	0	0	0	0	0	0	0	0	0	0
SAL_QA3652AA	PNUSAS042716	Bareilly	0	0	0	0	0	0	0	0	0	0	0
SAL_QA3599AA	PNUSAS042476	Bareilly	0	0	0	0	0	0	0	0	0	0	0
SAL_QA3607AA	PNUSAS040391	Bareilly	0	0	0	0	0	0	0	0	0	0	0
SAL_QA3600AA	PNUSAS041980	Bareilly	0	0	0	0	0	0	0	0	0	0	0

SAL_QA2477AA	242425	Bareilly	0	0	0	0	0	0	0	0	0	0	0
SAL_QA3933AA	388627	Bareilly	0	0	0	0	0	0	0	0	0	0	0
SAL_QA3905AA	483093	Bareilly	0	0	0	0	0	0	0	0	0	0	0
SAL_QA3937AA	439523	Bareilly	0	0	0	0	0	0	0	0	0	0	0
SAL_QA3907AA	199935	Bareilly	0	0	0	0	0	0	0	0	0	0	0
SAL_QA3855AA	327176	Bareilly	0	0	0	0	0	0	0	0	0	0	0
SAL_QA3857AA	305183	Bareilly	0	0	0	0	0	0	0	0	0	0	0
SAL_QA3815AA	320943	Bareilly	0	0	0	0	0	0	0	0	0	0	0
SAL_QA3803AA	159678	Bareilly	0	0	0	0	0	0	0	0	0	0	0
SAL_QA4115AA	474902	Bareilly	0	0	0	0	0	0	0	0	0	0	0
SAL_QA4137AA	376299	Bareilly	0	0	0	0	0	0	0	0	0	0	0
SAL_QA4098AA	299846	Bareilly	0	0	0	0	0	0	0	0	0	0	0
SAL_QA4110AA	288956	Bareilly	0	0	0	0	0	0	0	0	0	0	0
SAL_QA4545AA	PNUSAS043548	Bareilly	0	0	0	0	0	0	0	0	0	0	0
SAL_QA4541AA	PNUSAS043553	Bareilly	0	0	0	0	0	0	0	0	0	0	0
SAL_QA4542AA	PNUSAS043549	Bareilly	0	0	0	0	0	0	0	0	0	0	0
SAL_QA4501AA	PNUSAS042907	Bareilly	0	0	0	0	0	0	0	0	0	0	0
SAL_QA4511AA	PNUSAS037574	Bareilly	0	0	0	0	0	0	0	0	0	0	0
SAL_QA4481AA	PNUSAS038549	Bareilly	0	0	0	0	0	0	0	0	0	0	0
SAL_QA4924AA	286738	Bareilly	0	0	0	0	0	0	0	0	0	0	0
SAL_QA4833AA	429168	Bareilly	0	0	0	0	0	0	0	0	0	0	0
SAL_QA4707AA	274278	Bareilly	0	0	0	0	0	0	0	0	0	0	0
SAL_QA4668AA	282611	Bareilly	0	0	0	0	0	0	0	0	0	0	0
SAL_QA5511AA	432266	Bareilly	0	0	0	0	0	0	0	0	0	0	0
SAL_QA5453AA	266962	Bareilly	0	0	0	0	0	0	0	0	0	0	0
SAL_QA5267AA	285858	Bareilly	0	0	0	0	0	0	0	0	0	0	0
SAL_QA5259AA	524282	Bareilly	0	0	0	0	0	0	0	0	0	0	0
SAL_QA5212AA	275947	Bareilly	0	0	0	0	0	0	0	0	0	0	0
SAL_QA5164AA	154855	Bareilly	0	0	0	0	0	0	0	0	0	0	0
SAL_QA5175AA	265759	Bareilly	0	0	0	0	0	0	0	0	0	0	0
SAL_QA5131AA	262668	Bareilly	0	0	0	0	0	0	0	0	0	0	0
SAL_QA4057AA	187593	Bareilly	0	0	0	0	0	0	0	0	0	0	0
SAL_QA5848AA	337074	Bareilly	0	0	0	0	0	0	0	0	0	0	0
SAL_QA5780AA	390060	Bareilly	0	0	0	0	0	0	0	0	0	0	0
SAL_QA6084AA	PNUSAS042756	Bareilly	0	0	0	0	0	0	0	0	0	0	0
SAL_QA6266AA	132054	Bareilly	0	0	0	0	0	0	0	0	0	0	0
SAL_QA6194AA	328586	Bareilly	0	0	0	0	0	0	0	0	0	0	0
SAL_QA6601AA	267749	Bareilly	0	0	0	0	0	0	0	0	0	0	0
SAL_QA6573AA	374035	Bareilly	0	0	0	0	0	0	0	0	0	106	0
SAL_QA6533AA	442712	Bareilly	0	0	0	0	0	0	0	0	0	0	0
SAL_QA6424AA	316653	Bareilly	0	0	0	0	0	0	0	0	0	0	0
SAL_QA6777AA	174773	Bareilly	0	0	0	0	0	0	0	0	0	0	0
SAL_QA7110AA	PNUSAS042210	Bareilly	0	0	0	0	0	0	0	0	0	0	0
SAL_QA7306AA	212783	Bareilly	0	0	0	0	0	0	0	0	0	0	0
SAL_QA7237AA	305212	Bareilly	0	0	0	0	0	0	0	0	0	0	0
SAL_QA7235AA	322285	Bareilly	0	0	0	0	0	0	0	0	0	0	0
SAL_QA6182AA	164857	Bareilly	0	0	0	0	0	0	0	0	0	0	0
SAL_QA7696AA	392958	Bareilly	0	0	0	0	0	0	0	0	0	0	0
SAL_QA7633AA	206685	Bareilly	0	0	0	0	0	0	0	0	0	0	0
SAL_QA7655AA	123840	Bareilly	0	0	0	0	0	0	0	0	0	0	0
SAL_QA7656AA	506413	Bareilly	0	0	0	0	0	0	0	0	0	0	0
SAL_QA7571AA	218465	Bareilly	0	0	0	0	0	0	0	0	0	0	0
SAL_QA7582AA	371922	Bareilly	0	0	0	0	0	0	0	0	0	0	0
SAL_QA7541AA	442665	Bareilly	0	0	0	0	0	0	0	0	0	0	0
SAL_QA7544AA	323893	Bareilly	0	0	0	0	0	0	0	0	0	0	0
SAL_QA7458AA	556781	Bareilly	0	0	0	0	0	0	0	0	0	0	0
SAL_QA7460AA	264164	Bareilly	0	0	0	0	0	0	0	0	0	0	0
SAL_QA7463AA	553858	Bareilly	0	0	0	0	0	0	0	0	0	0	0
SAL_QA8431AA	500370	Bareilly	0	0	0	0	0	0	0	0	0	0	0
SAL_QA8382AA	500302	Bareilly	0	0	0	0	0	0	0	0	0	0	0
SAL_QA8353AA	440136	Bareilly	0	0	0	0	0	0	0	0	0	0	0
SAL_QA8304AA	214726	Bareilly	0	0	0	0	0	0	0	0	0	0	0
SAL_QA8261AA	261939	Bareilly	0	0	0	0	0	0	0	0	0	0	0
SAL_QA8508AA	PNUSAS040926	Bareilly	0	0	0	0	0	0	0	0	0	0	0
SAL_QA8516AA	PNUSAS042298	Bareilly	0	0	0	0	0	0	0	0	0	0	0
SAL_QA8859AA	109898	Bareilly	0	0	0	0	0	0	0	0	0	0	0
SAL_QA8854AA	270101	Bareilly	0	0	0	0	0	0	0	0	0	0	0
SAL_QA8844AA	152370	Bareilly	0	0	0	0	0	0	0	0	0	0	0
SAL_QA8855AA	445172	Bareilly	0	0	0	0	0	0	0	0	0	0	0
SAL_QA8803AA	257394	Bareilly	0	0	0	0	0	0	0	0	0	0	0
SAL_QA8778AA	503441	Bareilly	0	0	0	0	0	0	0	0	0	0	0
SAL_QA8721AA	358441	Bareilly	0	0	0	0	0	0	0	0	0	0	0
SAL_QA8708AA	211652	Bareilly	0	0	0	0	0	0	0	0	0	0	0

SAL_QA8643AA	107753	Bareilly	0	0	0	0	0	0	0	0	0	0	0
SAL_QA8657AA	513485	Bareilly	0	0	0	0	0	0	0	0	0	0	0
SAL_RA6858AA	14508	Bareilly	0	0	0	0	0	0	0	0	0	0	0
SAL_QA9082AA	PNUSAS044146	Bareilly	0	0	0	0	0	0	0	0	0	0	0
SAL_QA8941AA	PNUSAS044586	Bareilly	0	0	0	0	0	0	0	0	0	0	0
SAL_QA8930AA	PNUSAS044433	Bareilly	0	0	0	0	0	0	0	0	0	0	0
SAL_QA8934AA	PNUSAS044441	Bareilly	0	0	0	0	0	0	0	0	0	0	0
SAL_QA8923AA	PNUSAS044289	Bareilly	0	0	0	0	0	0	0	0	0	0	0
SAL_QA9464AA	197107	Bareilly	0	0	0	0	0	0	0	0	0	0	0
SAL_QA9443AA	381265	Bareilly	0	0	0	0	0	0	0	0	0	0	0
SAL_QA9605AA	PNUSAS043812	Bareilly	0	0	0	0	0	0	0	0	0	0	0
SAL_QA9592AA	PNUSAS044528	Bareilly	0	0	0	0	0	0	0	0	0	0	0
SAL_QA9674AA	PNUSAS044282	Bareilly	0	0	0	0	0	0	0	0	0	0	0
SAL_QA9350AA	115287	Bareilly	0	0	0	0	0	0	0	0	0	0	0
SAL_QA9316AA	193284	Bareilly	0	0	0	0	0	0	0	0	0	0	0
SAL_QA9269AA	231989	Bareilly	0	0	0	0	0	0	0	0	0	0	0
SAL_QA9272AA	378666	Bareilly	0	0	0	0	0	0	0	0	0	0	0
SAL_QA9795AA	466042	Bareilly	0	0	0	0	0	0	0	0	0	0	0
SAL_RA0103AA	PNUSAS044026	Bareilly	0	0	0	0	0	0	0	0	0	0	0
SAL_RA0107AA	PNUSAS044025	Bareilly	0	0	0	0	0	0	0	0	0	0	0
SAL_RA0442AA	360487	Bareilly	0	0	0	0	0	0	0	0	0	0	0
SAL_RA0403AA	296818	Bareilly	0	0	0	0	0	0	0	0	0	0	0
SAL_RA0350AA	261485	Bareilly	0	0	0	0	0	0	0	0	0	0	0
SAL_RA0296AA	287589	Bareilly	0	0	0	0	0	0	0	0	0	0	0
SAL_RA0298AA	307490	Bareilly	0	0	0	0	0	0	0	0	0	0	0
SAL_RA0308AA	164707	Bareilly	0	0	0	0	0	0	0	0	0	0	0
SAL_RA0261AA	255589	Bareilly	0	0	0	0	0	0	0	0	0	0	0
SAL_RA0187AA	245483	Bareilly	0	0	0	0	0	0	0	0	0	0	0
SAL_RA0216AA	389720	Bareilly	0	0	0	0	0	0	0	0	0	0	0
SAL_RA0231AA	361787	Bareilly	0	0	0	0	0	0	0	0	0	0	0
SAL_RA1115AA	191871	Bareilly	0	0	0	0	0	0	0	0	0	0	0
SAL_RA1051AA	306487	Bareilly	0	0	0	0	0	0	0	0	0	0	0
SAL_RA0913AA	267815	Bareilly	0	0	0	0	0	0	0	0	0	0	0
SAL_RA0870AA	382664	Bareilly	0	0	0	0	0	0	0	0	0	0	0
SAL_RA0690AA	180543	Bareilly	0	0	0	0	0	0	0	0	0	0	0
SAL_RA0621AA	114080	Bareilly	0	0	0	0	0	0	0	0	0	0	0
SAL_RA0538AA	282597	Bareilly	0	0	0	0	0	0	0	0	0	0	0
SAL_RA1160AA	PNUSAS044939	Bareilly	0	0	0	0	0	0	0	0	0	0	0
SAL_RA1140AA	PNUSAS044942	Bareilly	0	0	0	0	0	0	0	0	0	0	0
SAL_RA1359AA	553772	Bareilly	0	0	0	0	0	0	0	0	0	0	0
SAL_RA1364AA	506433	Bareilly	0	0	0	0	0	0	0	0	0	0	0
SAL_RA1329AA	387525	Bareilly	0	0	0	0	0	0	0	0	0	0	0
SAL_RA1341AA	279385	Bareilly	0	0	0	0	0	0	0	0	0	0	0
SAL_RA1462AA	PNUSAS043991	Bareilly	0	0	0	0	0	0	0	0	0	0	0
SAL_RA1438AA	PNUSAS044414	Bareilly	0	0	0	0	0	0	0	0	0	0	0
SAL_RA1447AA	PNUSAS043989	Bareilly	0	0	0	0	0	0	0	0	0	0	0
SAL_RA2483AA	PNUSAS045298	Bareilly	0	0	0	0	0	0	0	0	0	0	0
SAL_RA2495AA	PNUSAS044701	Bareilly	0	0	0	0	0	0	0	0	0	0	0
SAL_RA2750AA	286833	Bareilly	0	0	0	0	0	0	0	0	0	0	0
SAL_RA3140AA	PNUSAS045119	Bareilly	0	0	0	0	0	0	0	0	0	0	0
SAL_RA3075AA	PNUSAS044844	Bareilly	0	0	0	0	0	0	0	0	0	0	0
SAL_RA3002AA	PNUSAS043660	Bareilly	0	0	0	0	0	0	0	0	0	0	0
SAL_RA2993AA	PNUSAS043425	Bareilly	0	0	0	0	0	0	0	0	0	0	0
SAL_RA2964AA	PNUSAS043692	Bareilly	0	0	0	0	0	0	0	0	0	0	0
SAL_RA3348AA	157277	Bareilly	0	0	0	0	0	0	0	0	0	0	0
SAL_RA3330AA	320971	Bareilly	0	0	0	0	0	0	0	0	0	0	0
SAL_RA3295AA	249386	Bareilly	0	0	0	0	0	0	0	0	0	0	0
SAL_RA3235AA	211116	Bareilly	0	0	0	0	0	0	0	0	0	0	0
SAL_RA3777AA	373861	Bareilly	0	0	0	0	0	0	0	0	0	0	0
SAL_RA3754AA	524788	Bareilly	0	0	0	0	0	0	0	0	0	0	0
SAL_RA3713AA	215076	Bareilly	0	0	0	0	0	0	0	0	0	0	0
SAL_RA3635AA	414671	Bareilly	0	0	0	0	0	0	0	0	0	0	0
SAL_RA3563AA	338382	Bareilly	0	0	0	0	0	0	0	0	0	0	0
SAL_RA4063AA	304479	Bareilly	0	0	0	0	0	0	0	0	0	0	0
SAL_RA4073AA	286732	Bareilly	0	0	0	0	0	0	0	0	0	0	0
SAL_RA4051AA	529846	Bareilly	0	0	0	0	0	0	0	0	0	0	0
SAL_RA3962AA	320989	Bareilly	0	0	0	0	0	0	0	0	0	0	0
SAL_RA3933AA	451985	Bareilly	0	0	0	0	0	0	0	0	0	0	0
SAL_RA3912AA	550677	Bareilly	0	0	0	0	0	0	0	0	0	0	0
SAL_RA4250AA	CNSV-T15-MD11-1-RV-B	Bareilly	0	0	0	0	0	0	0	0	0	0	0
SAL_RA4252AA	CNSV-T15-MD11-1-RV-C	Bareilly	0	0	0	0	0	0	0	0	0	0	0
SAL_RA4425AA	136763	Bareilly	0	0	0	0	0	0	0	0	0	0	0
SAL_RA4417AA	240166	Bareilly	0	0	0	0	0	0	0	0	0	0	0

SAL_RA4332AA	140603	Bareilly	0	0	0	0	0	0	0	0	0	0	0
SAL_RA6041AA	PNUSAS045626	Bareilly	0	0	0	0	0	0	0	0	0	0	0
SAL_RA5971AA	PNUSAS044868	Bareilly	0	0	0	0	0	0	0	0	0	0	0
SAL_RA5853AA	PNUSAS045561	Bareilly	0	0	0	0	0	0	0	0	0	0	0
SAL_RA5846AA	PNUSAS044299	Bareilly	0	0	0	0	0	0	0	0	0	0	0
SAL_RA5794AA	267027	Bareilly	0	0	0	0	0	0	0	0	0	0	0
SAL_RA5732AA	418209	Bareilly	0	0	0	0	0	0	0	0	0	0	0
SAL_RA5636AA	204428	Bareilly	0	0	0	0	0	0	0	0	0	0	0
SAL_RA5667AA	381348	Bareilly	0	0	0	0	0	0	0	0	0	0	0
SAL_RA5601AA	353890	Bareilly	0	0	0	0	0	0	0	0	0	0	0
SAL_RA5516AA	218444	Bareilly	0	0	0	0	0	0	0	0	0	0	0
SAL_RA5424AA	191964	Bareilly	0	0	0	0	0	0	0	0	0	0	0
SAL_RA2603AA	134018	Bareilly	0	0	0	0	0	0	0	0	0	0	0
SAL_RA6341AA	PNUSAS044978	Bareilly	0	0	0	0	0	0	0	0	0	0	0
SAL_RA6317AA	PNUSAS045442	Bareilly	0	0	0	0	0	0	0	0	0	0	0
SAL_RA6403AA	PNUSAS045063	Bareilly	0	0	0	0	0	0	0	0	0	0	0
SAL_RA6670AA	454854	Bareilly	0	0	0	0	0	0	0	0	0	0	0
SAL_RA6634AA	356625	Bareilly	0	0	0	0	0	0	0	0	0	0	0
SAL_SA2425AA	CZ_17_0968	Bareilly	0	0	0	0	0	0	0	0	0	0	0
SAL_SA2424AA	CZ_17_1068	Bareilly	0	0	0	0	0	0	0	0	0	0	0
SAL_SA2426AA	CZ_17_1057	Bareilly	0	0	0	0	0	0	0	0	0	0	0
SAL_SA2427AA	CZ_17_0768	Bareilly	0	0	0	0	0	0	0	0	0	0	0
SAL_SA2428AA	CZ_17_0882	Bareilly	0	0	0	0	0	0	0	0	0	0	0
SAL_SA2429AA	CZ_17_0848	Bareilly	0	0	0	0	0	0	0	0	0	0	0
SAL_SA2431AA	CZ_17_0871	Bareilly	0	0	0	0	0	0	0	0	0	0	0
SAL_SA2430AA	CZ_17_0778	Bareilly	0	0	0	0	0	0	0	0	0	0	0
SAL_SA2432AA	CZ_17_1042	Bareilly	0	0	0	0	0	0	0	0	0	0	0
SAL_SA2435AA	CZ_17_0937	Bareilly	0	0	0	0	0	0	0	0	0	0	0
SAL_SA2433AA	CZ_17_1013	Bareilly	0	0	0	0	0	0	0	0	0	0	0
SAL_SA2434AA	CZ_17_1050	Bareilly	0	0	0	0	0	0	0	0	0	0	0
SAL_SA2436AA	CZ_17_0509	Bareilly	0	0	0	0	0	0	0	0	0	0	0
SAL_SA2438AA	CZ_17_0854	Bareilly	0	0	0	0	0	0	0	0	0	0	0
SAL_SA2439AA	CZ_17_0920	Bareilly	0	0	0	0	0	0	0	0	0	0	0
SAL_RA6619AA	370602	Bareilly	0	0	0	0	0	0	0	0	0	0	0
SAL_RA6504AA	227953	Bareilly	0	0	0	0	0	0	0	0	0	0	0
SAL_RA6467AA	542095	Bareilly	0	0	0	0	0	0	0	0	0	0	0
SAL_RA6798AA	PNUSAS046227	Bareilly	0	0	0	0	0	0	0	0	0	0	0
SAL_RA6172AA	PNUSAS042946	Bareilly	0	0	0	0	0	0	0	0	0	0	0
SAL_RA7380AA	488724	Bareilly	0	0	0	0	0	0	0	0	0	0	0
SAL_RA7385AA	373262	Bareilly	0	0	0	0	0	0	0	0	0	0	0
SAL_RA7347AA	568267	Bareilly	0	0	0	0	0	0	0	0	0	0	0
SAL_RA7297AA	335544	Bareilly	0	0	0	0	0	0	0	0	0	0	0
SAL_RA7299AA	327913	Bareilly	0	0	0	0	0	0	0	0	0	0	0
SAL_RA7271AA	509960	Bareilly	0	0	0	0	0	0	0	0	0	0	0
SAL_RA7262AA	399376	Bareilly	0	0	0	0	0	0	0	0	0	0	0
SAL_RA7557AA	PNUSAS044306	Bareilly	0	0	0	0	0	0	0	0	0	0	0
SAL_RA7879AA	539043	Bareilly	0	0	0	0	0	0	0	0	0	0	0
SAL_RA7694AA	123992	Bareilly	0	0	0	0	0	0	0	0	0	0	0
SAL_RA8104AA	328567	Bareilly	0	0	0	0	0	0	0	0	0	0	0
SAL_RA8085AA	143951	Bareilly	0	0	0	0	0	0	0	0	0	0	0
SAL_RA8056AA	529208	Bareilly	0	0	0	0	0	0	0	0	0	0	0
SAL_RA7972AA	455136	Bareilly	0	0	0	0	0	0	0	0	0	0	0
SAL_RA8218AA	393924	Bareilly	0	0	0	0	0	0	0	0	0	0	0
SAL_RA8229AA	527502	Bareilly	0	0	0	0	0	0	0	0	0	0	0
SAL_RA8203AA	308549	Bareilly	0	0	0	0	0	0	0	0	0	0	0
SAL_SA3015AA	18EP001605	Bareilly	0	0	0	0	0	0	0	0	0	0	0
SAL_RA5483AA	131499	Bareilly	0	0	0	0	0	0	0	0	0	0	0
SAL_SA0038AA	CVM N17S224	Bareilly	0	0	0	0	0	0	0	0	0	0	0
SAL_RA6765AA	PNUSAS046012	Bareilly	0	0	0	0	0	0	0	0	0	0	0
SAL_RA8076AA	391740	Bareilly	0	0	0	0	0	0	0	0	0	0	0
SAL_SA1031AA	ı10e-c799-4a91-8976-d7	Bareilly	0	0	0	0	0	0	0	0	0	0	0
SAL_SA1075AA	CNSV-T14-MD05-10-RV-A	Bareilly	0	0	0	0	0	0	0	0	0	0	0
SAL_SA2682AA	FSIS11813219	Bareilly	0	0	0	0	0	0	0	0	0	0	0
SAL_SA1055AA	CNSV-T15-MD05-0.1-RV-A	Bareilly	0	0	0	0	0	0	0	0	0	0	0
SAL_SA1057AA	CNSV-T15-MD05-10-RV-B	Bareilly	0	0	0	0	0	0	0	0	0	0	0
SAL_SA1051AA	CNSV-T15-MD05-0.1-RV-I	Bareilly	0	0	0	0	0	0	0	0	0	0	0
SAL_SA4370AA	FDA00007804	Bareilly	0	0	0	0	0	0	0	0	0	0	0
SAL_SA4373AA	FDA00007808	Bareilly	0	0	0	0	0	0	0	0	0	0	0
SAL_SA4380AA	FDA00007807	Bareilly	0	0	0	0	0	0	0	0	0	0	0
SAL_SA4382AA	FDA00007803	Bareilly	0	0	0	0	0	0	0	0	0	0	0
SAL_SA4290AA	FSIS11813881	Bareilly	0	0	0	0	0	0	0	0	0	0	0
SAL_SA5848AA	601612	Bareilly	0	0	0	0	0	0	0	0	0	0	0
SAL_SA5819AA	584672	Bareilly	0	0	0	0	0	0	0	0	0	0	0

SAL_SA6423AA	595671	Bareilly	0	0	0	0	0	0	0	0	0	0	0
SAL_SA6369AA	603398	Bareilly	0	0	0	0	0	0	0	0	0	0	0
SAL_SA6376AA	207527	Bareilly	0	0	0	0	0	0	0	0	0	0	0
SAL_SA6323AA	592657	Bareilly	0	0	0	0	0	0	0	0	0	0	0
SAL_SA6272AA	575089	Bareilly	0	0	0	0	0	0	0	0	0	0	0
SAL_SA6762AA	542008	Bareilly	0	0	0	0	0	0	0	0	0	0	0
SAL_SA6725AA	601652	Bareilly	0	0	0	0	0	0	0	0	0	0	0
SAL_SA6576AA	261908	Bareilly	0	0	0	0	0	0	0	0	0	0	0
SAL_SA6319AA	571976	Bareilly	0	0	0	0	0	0	0	0	0	0	0
SAL_SA7102AA	312093	Bareilly	0	0	0	0	0	0	0	0	0	0	0
SAL_SA6197AA	591833	Bareilly	0	0	0	0	0	0	0	0	0	0	0
SAL_SA7556AA	256141	Bareilly	0	0	0	0	0	0	0	0	0	0	0
SAL_SA7507AA	401612	Bareilly	0	0	0	0	0	0	0	0	0	0	0
SAL_SA7422AA	203616	Bareilly	0	0	0	0	0	0	0	0	0	0	0
SAL_SA7770AA	590529	Bareilly	0	0	0	0	0	0	0	0	0	0	0
SAL_SA7678AA	143982	Bareilly	0	0	0	0	0	0	0	0	0	0	0
SAL_SA7691AA	195783	Bareilly	0	0	0	0	0	0	0	0	0	0	0
SAL_SA7991AA	267812	Bareilly	0	0	0	0	0	0	0	0	0	0	0
SAL_SA7978AA	516882	Bareilly	0	0	0	0	0	0	0	0	0	0	0
SAL_SA7936AA	277866	Bareilly	0	0	0	0	0	0	0	0	0	0	0
SAL_SA7889AA	560637	Bareilly	0	0	0	0	0	0	0	0	0	0	0
SAL_SA8713AA	607336	Bareilly	0	0	0	0	0	0	0	0	0	0	0
SAL_SA8681AA	287718	Bareilly	0	0	0	0	0	0	0	0	0	0	0
SAL_SA8615AA	598923	Bareilly	0	0	0	0	0	0	0	0	0	0	0
SAL_SA8577AA	279370	Bareilly	0	0	0	0	0	0	0	0	0	0	0
SAL_SA8600AA	294700	Bareilly	0	0	0	0	0	0	0	0	0	0	0
SAL_SA8932AA	601677	Bareilly	0	0	0	0	0	0	0	0	0	0	0
SAL_SA8878AA	203998	Bareilly	0	0	0	0	0	0	0	0	0	0	0
SAL_SA9453AA	529854	Bareilly	0	0	0	0	0	0	0	0	0	0	0
SAL_SA8636AA	271091	Bareilly	0	0	0	0	0	0	0	0	0	0	0
SAL_TA0363AA	611061	Bareilly	0	0	0	0	0	0	0	0	0	0	0
SAL_TA0344AA	613847	Bareilly	0	0	0	0	0	0	0	0	0	0	0
SAL_TA0316AA	612608	Bareilly	0	0	0	0	0	0	0	0	0	0	0
SAL_TA0805AA	617904	Bareilly	0	0	0	0	0	0	0	0	0	0	0
SAL_TA0899AA	616205	Bareilly	0	0	0	0	0	0	0	0	0	0	0
SAL_TA2978AA	617010	Bareilly	0	0	0	0	0	0	0	0	0	0	0
SAL_TA5230AA	612636	Bareilly	0	0	0	0	0	0	0	0	0	0	0
SAL_TA5994AA	611465	Bareilly	0	0	0	0	0	0	0	0	0	0	0
SAL_TA5982AA	610780	Bareilly	0	0	0	0	0	0	0	0	0	0	0
SAL_TA5947AA	611387	Bareilly	0	0	0	0	0	0	0	0	0	0	0
SAL_TA6748AA	632971	Bareilly	0	0	0	0	0	0	0	0	0	0	0
SAL_TA7200AA	SMG-18-2196	Bareilly	0	0	0	0	0	0	0	0	0	0	0
SAL_TA7271AA	VA-WGS-18099	Bareilly	0	0	0	0	0	0	0	0	0	0	0
SAL_TA7256AA	VA-WGS-18096	Bareilly	0	0	0	0	0	0	0	0	0	0	0
SAL_TA7262AA	VA-WGS-18098	Bareilly	0	0	0	0	0	0	0	0	0	0	0
SAL_TA7263AA	VA-WGS-18097	Bareilly	0	0	0	0	0	0	0	0	0	0	0
SAL_TA7483AA	636253	Bareilly	0	0	0	0	0	0	0	0	0	0	0
SAL_SA3338AA	FSIS11813299	Bareilly	0	0	0	0	0	0	0	0	0	0	0
SAL_SA5855AA	593585	Bareilly	0	0	0	0	0	0	0	0	0	0	0
SAL_SA7607AA	105322	Bareilly	0	0	0	0	0	0	0	0	0	0	0
SAL_OA2470AA	MOD1-Lipp-110	Bareilly	0	0	0	0	0	0	0	0	0	0	0
SAL_PA2597AA	PNUSAS036108	Bareilly	0	0	0	0	0	0	0	0	0	0	0
SAL_QA5395AA	455154	Bareilly	0	0	0	0	0	0	0	0	0	0	0
SAL_QA5320AA	251831	Bareilly	0	0	0	0	0	0	0	0	0	0	0
SAL_OA1233AA	PNUSAS034231	Bareilly	0	0	0	0	0	0	0	0	0	0	0
SAL_PA3762AA	FDA00012858	Bareilly	0	0	0	0	0	0	0	0	0	0	0
SAL_OA1367AA	PNUSAS034767	Bareilly	0	0	0	0	0	0	0	0	0	0	0
SAL_HA5544AA	CFSAN035737	Bareilly	0	0	0	0	0	0	0	0	0	0	0
SAL_TA8082AA	AP-2-VA-WGS-18097	Bareilly	0	0	0	0	0	0	0	0	0	0	0
SAL_TA8084AA	AP-3-VA-WGS-18098	Bareilly	0	0	0	0	0	0	0	0	0	0	0
SAL_TA8085AA	AP-4-VA-WGS-18099	Bareilly	0	0	0	0	0	0	0	0	0	0	0
SAL_OA1343AA	PNUSAS031701	Bareilly	0	0	0	0	0	0	0	0	0	0	0
SAL_OA1341AA	PNUSAS031706	Bareilly	0	0	0	0	0	0	0	0	0	0	0
SAL_QA7961AA	PNUSAS043977	Bareilly	0	0	0	0	0	0	0	0	0	0	0
SAL_MA1648AA	PNUSAS024053	Bareilly	0	0	0	0	0	0	0	0	0	0	0
SAL_NA6993AA	PNUSAS028552	Bareilly	0	0	0	0	0	0	0	0	0	0	0
SAL_OA1375AA	PNUSAS034771	Bareilly	0	0	0	0	0	0	0	0	0	0	0
SAL_TA8390AA	638785	Bareilly	0	0	0	0	0	0	0	0	0	0	0
SAL_QA2498AA	367223	Bareilly	0	0	0	0	0	0	0	0	0	0	0
SAL_OA3722AA	MOD1-Jwm-11	Bareilly	0	0	0	0	0	0	0	0	0	0	0
SAL_OA1357AA	PNUSAS031702	Bareilly	0	0	0	0	0	0	0	0	0	0	0
SAL_OA1512AA	PNUSAS031703	Bareilly	0	0	0	0	0	0	0	0	0	0	0
SAL_KA2368AA	2015AM-1501	Bareilly	0	0	0	0	0	0	0	0	0	0	0

SAL_OA3725AA	MOD1-Pcm-37	Bareilly	0	0	0	0	0	0	0	0	0	0	0
SAL_QA1942AA	325719	Bareilly	0	0	0	0	0	0	0	0	0	0	0
SAL_QA2288AA	533439	Bareilly	0	0	0	0	0	0	0	0	0	0	0
SAL_QA2480AA	279557	Bareilly	0	0	0	0	0	0	0	0	0	0	0
SAL_QA7746AA	157266	Bareilly	0	0	0	0	0	0	0	0	0	0	0
SAL_KA7376AA	PNUSAS017551	Bareilly	0	0	0	0	0	0	0	0	0	0	0
SAL_QA9834AA	557656	Bareilly	0	0	0	0	0	0	0	0	0	0	0
SAL_OA3734AA	MOD1-Ppo-85	Bareilly	0	0	0	0	0	0	0	0	0	0	0
SAL_KA5541AA	PNUSAS018494	Bareilly	0	0	0	0	0	0	0	0	0	0	0
SAL_OA1317AA	PNUSAS030611	Bareilly	0	0	0	0	0	0	0	0	0	0	0
SAL_OA0848AA	PNUSAS031837	Bareilly	0	0	0	0	0	0	0	0	0	0	0
SAL_QA3669AA	PNUSAS019326	Bareilly	0	0	0	0	0	0	0	0	0	0	0
SAL_QA5342AA	224050	Bareilly	0	0	0	0	0	0	0	0	0	0	0
SAL_QA5362AA	424686	Bareilly	0	0	0	0	0	0	0	0	0	0	0
SAL_QA5405AA	528555	Bareilly	0	0	0	0	0	0	0	0	0	0	0
SAL_OA1221AA	PNUSAS033699	Bareilly	0	0	0	0	0	0	0	0	0	0	0
SAL_OA1250AA	PNUSAS034228	Bareilly	0	0	0	0	0	0	0	0	0	0	0
SAL_OA1301AA	PNUSAS030545	Bareilly	0	0	0	0	0	0	0	0	0	0	0
SAL_OA1953AA	PNUSAS028451	Bareilly	0	0	0	0	0	0	0	0	0	0	0
SAL_OA2436AA	MOD1-Icp-301	Bareilly	0	0	0	0	0	0	0	0	0	0	0
SAL_OA2221AA	PNUSAS034211	Bareilly	0	0	0	0	0	0	0	0	0	0	0
SAL_OA2030AA	PNUSAS035259	Bareilly	0	0	0	0	0	0	0	0	0	0	0
SAL_OA2463AA	MOD1-Lipp-724	Bareilly	0	0	0	0	0	0	0	0	0	0	0
SAL_OA2234AA	PNUSAS034205	Bareilly	0	0	0	0	0	0	0	0	0	0	0
SAL_OA5172AA	PNUSAS035876	Bareilly	0	0	0	0	0	0	0	0	0	0	0
SAL_OA5209AA	PNUSAS036293	Bareilly	0	0	0	0	0	0	0	0	0	0	0
SAL_OA5245AA	PNUSAS035488	Bareilly	0	0	0	0	0	0	0	0	0	0	0
SAL_OA5277AA	PNUSAS035321	Bareilly	0	0	0	0	0	0	0	0	0	0	0
SAL_OA5274AA	PNUSAS035308	Bareilly	0	0	0	0	0	0	0	0	0	0	0
SAL_OA5418AA	PNUSAS035441	Bareilly	0	0	0	0	0	0	0	0	0	0	0
SAL_OA5474AA	PNUSAS035234	Bareilly	0	0	0	0	0	0	0	0	0	0	0
SAL_PA1352AA	PNUSAS038021	Bareilly	0	0	0	0	0	0	0	0	0	0	0
SAL_PA1521AA	PNUSAS037719	Bareilly	0	0	0	0	0	0	0	0	0	0	0
SAL_PA1502AA	PNUSAS037089	Bareilly	0	0	0	0	0	0	0	0	0	0	0
SAL_PA1855AA	PNUSAS038064	Bareilly	0	0	0	0	0	0	0	0	0	0	0
SAL_PA2303AA	PNUSAS038380	Bareilly	0	0	0	0	0	0	0	0	0	0	0
SAL_PA2385AA	PNUSAS038711	Bareilly	0	0	0	0	0	0	0	0	0	0	0
SAL_PA3439AA	PNUSAS038354	Bareilly	0	0	0	0	0	0	0	0	0	0	0
SAL_PA3564AA	CFSAN000189	Bareilly	0	0	0	0	0	0	0	0	0	0	0
SAL_PA3563AA	CFSAN000191	Bareilly	0	0	0	0	0	0	0	0	0	0	0
SAL_PA3761AA	FDA00012859	Bareilly	0	0	0	0	0	0	0	0	0	0	0
SAL_PA4130AA	CNSV-T11-MD04-1-TT-A	Bareilly	0	0	0	0	0	0	0	0	0	0	0
SAL_PA4115AA	CNSV-T11-MD05-10-RV-C	Bareilly	0	0	0	0	0	0	0	0	0	0	0
SAL_TA9545AA	639805	Bareilly	0	0	0	0	0	0	0	0	0	0	0
SAL_OA1369AA	PNUSAS031694	Bareilly	0	0	0	0	0	0	0	0	0	0	0
SAL_UA1718AA	623194	Bareilly	0	0	0	0	0	0	0	0	0	0	0
SAL_UA2736AA	648606	Bareilly	0	0	0	0	0	0	0	0	0	0	0
SAL_UA2762AA	648620	Bareilly	0	0	0	0	0	0	0	0	0	0	0
SAL_UA2831AA	649899	Bareilly	0	0	0	0	0	0	0	0	0	0	0
SAL_UA2813AA	649013	Bareilly	0	0	0	0	0	0	0	0	0	0	0
SAL_UA6871AA	AG18-0130	Bareilly	0	0	0	0	0	0	0	0	0	0	0
SAL_UA6793AA	661011	Bareilly	0	0	0	0	0	0	0	0	0	0	0
SAL_UA7248AA	662869	Bareilly	0	0	0	0	0	0	0	0	0	0	0
SAL_UA7245AA	662832	Bareilly	0	0	0	0	0	0	0	0	0	0	0
SAL_UA3005AA	647804	Bareilly	0	0	0	0	0	0	0	0	0	0	0
SAL_UA3720AA	598149	Bareilly	0	0	0	0	0	0	0	0	0	0	0
SAL_UA3739AA	597066	Bareilly	0	0	0	0	0	0	0	0	0	0	0
SAL_UA6828AA	CFSAN089919	Bareilly	0	0	0	0	0	0	0	0	0	0	0
SAL_UA4926AA	CFSAN089066	Bareilly	0	0	0	0	0	0	0	0	0	0	0
SAL_UA4922AA	CFSAN089065	Bareilly	0	0	0	0	0	0	0	0	0	0	0
SAL_UA5239AA	FDA00002514	Bareilly	0	0	0	0	0	0	0	0	0	0	0
SAL_UA5255AA	651989	Bareilly	0	0	0	0	0	0	0	0	0	0	0
SAL_UA5958AA	588924	Bareilly	0	0	0	0	0	0	0	0	0	0	0
SAL_UA6058AA	654005	Bareilly	0	0	0	0	0	0	0	0	0	0	0
SAL_UA6172AA	579291	Bareilly	0	0	0	0	0	0	0	0	0	0	0
SAL_UA6381AA	657140	Bareilly	0	0	0	0	0	0	0	0	0	0	0
SAL_UA6451AA	573634	Bareilly	0	0	0	0	0	0	0	0	0	0	0
SAL_DA6791AA	CFSAN000203	Bareilly	0	0	0	0	0	0	0	0	0	0	0
SAL_DA8041AA	CFSAN001112	Bareilly	0	0	0	0	0	0	0	0	0	0	0
SAL_CA1522AA	CFSAN000966	Bareilly	0	0	0	0	0	0	0	0	0	0	0
SAL_DA8039AA	CFSAN001099	Bareilly	0	0	0	0	0	0	0	0	0	0	0
SAL_DA8037AA	CFSAN001118	Bareilly	0	0	0	0	0	0	0	0	0	0	0
SAL_CA1481AA	CFSAN001087	Bareilly	0	0	0	0	0	0	0	0	0	0	0

SAL_CA1669AA	CFSAN000204	Bareilly	0	0	0	0	0	0	0	0	0	0	0
SAL_CA1696AA	CFSAN000190	Bareilly	0	0	0	0	0	0	0	0	0	0	0
SAL_CA1546AA	CFSAN000956	Bareilly	0	0	0	0	0	0	0	0	0	0	0
SAL_CA1673AA	CFSAN000195	Bareilly	0	0	0	0	0	0	0	0	0	0	0
SAL_CA1531AA	CFSAN000962	Bareilly	0	0	0	0	0	0	0	0	0	0	0
SAL_CA1547AA	CFSAN000955	Bareilly	0	0	0	0	0	0	0	0	0	0	0
SAL_CA1520AA	CFSAN000967	Bareilly	0	0	0	0	0	0	0	0	0	0	0
SAL_UA7871AA	579924	Bareilly	0	0	0	0	0	0	0	0	0	0	0
SAL_UA8162AA	CZ_17_0675	Bareilly	0	0	0	0	0	0	0	0	0	0	0
SAL_UA8228AA	CZ_18_0970	Bareilly	0	0	0	0	0	0	0	0	0	0	0
SAL_UA9624AA	588507	Bareilly	0	0	0	0	0	0	0	0	0	0	0
SAL_VA0143AA	669737	Bareilly	0	0	0	0	0	0	0	0	0	0	0
SAL_VA0080AA	568288	Bareilly	0	0	0	0	0	0	0	0	0	0	0
SAL_VA0049AA	625287	Bareilly	0	0	0	0	0	0	0	0	0	0	0
SAL_UA9875AA	559067	Bareilly	0	0	0	0	0	0	0	0	0	0	0
SAL_UA9877AA	553758	Bareilly	0	0	0	0	0	0	0	0	0	0	0
SAL_UA9888AA	674047	Bareilly	0	0	0	0	0	0	0	0	0	0	0
SAL_UA9866AA	553891	Bareilly	0	0	0	0	0	0	0	0	0	0	0
SAL_UA9741AA	568304	Bareilly	0	0	0	0	0	0	0	0	0	0	0
SAL_VA0254AA	612982	Bareilly	0	0	0	0	0	0	0	0	0	0	0
SAL_VA0827AA	529822	Bareilly	0	0	0	0	0	0	0	0	0	0	0
SAL_VA0460AA	539944	Bareilly	0	0	0	0	0	0	0	0	0	0	0
SAL_VA0404AA	542081	Bareilly	0	0	0	0	0	0	0	0	0	0	0
SAL_VA0725AA	524290	Bareilly	0	0	0	0	0	0	0	0	0	0	0
SAL_VA1121AA	526776	Bareilly	0	0	0	0	0	0	0	0	0	0	0
SAL_VA1199AA	507283	Bareilly	0	0	0	0	0	0	0	0	0	0	0
SAL_VA1045AA	511856	Bareilly	0	0	0	0	0	0	0	0	0	0	0
SAL_VA0960AA	516861	Bareilly	0	0	0	0	0	0	0	0	0	0	0
SAL_VA0969AA	674348	Bareilly	0	0	0	0	0	0	0	0	0	0	0
SAL_VA1651AA	PNUSAS051008	Bareilly	0	0	0	0	0	0	0	0	0	0	0
SAL_VA1666AA	PNUSAS053267	Bareilly	0	0	0	0	0	0	0	0	0	0	0
SAL_VA1334AA	676384	Bareilly	0	0	0	0	0	0	0	0	0	0	0
SAL_VA1302AA	480728	Bareilly	0	0	0	0	0	0	0	0	0	0	0
SAL_VA1314AA	488735	Bareilly	0	0	0	0	0	0	0	0	0	0	0
SAL_VA7634AA	201707985	Bareilly	0	0	0	0	0	0	0	0	0	0	0
SAL_VA2100AA	672549	Bareilly	0	0	0	0	0	0	0	0	0	0	0
SAL_VA2070AA	453215	Bareilly	0	0	0	0	0	0	0	0	0	0	0
SAL_VA1972AA	435398	Bareilly	0	0	0	0	0	0	0	0	0	0	0
SAL_VA3468AA	396024	Bareilly	0	0	0	0	0	0	0	0	0	0	0
SAL_VA3421AA	397686	Bareilly	0	0	0	0	0	0	0	0	0	0	0
SAL_VA2371AA	474884	Bareilly	0	0	0	0	0	0	0	0	0	0	0
SAL_VA2269AA	483086	Bareilly	0	0	0	0	0	0	0	0	0	0	0
SAL_VA1930AA	478023	Bareilly	0	0	0	0	0	0	0	0	0	0	0
SAL_VA1858AA	443531	Bareilly	0	0	0	0	0	0	0	0	0	0	0
SAL_VA1740AA	PNUSAS054377	Bareilly	0	0	0	0	0	0	0	0	0	0	0
SAL_VA1732AA	PNUSAS047836	Bareilly	0	0	0	0	0	0	0	0	0	0	0
SAL_VA2475AA	423128	Bareilly	0	0	0	0	0	0	0	0	0	0	0
SAL_VA2435AA	430230	Bareilly	0	0	0	0	0	0	0	0	0	0	0
SAL_VA2397AA	455133	Bareilly	0	0	0	0	0	0	0	0	0	0	0
SAL_VA2782AA	420536	Bareilly	0	0	0	0	0	0	0	0	0	0	0
SAL_VA2779AA	430241	Bareilly	0	0	0	0	0	0	0	0	0	0	0
SAL_VA2712AA	429191	Bareilly	0	0	0	0	0	0	0	0	0	0	0
SAL_VA5735AA	SA20034666	Bareilly	0	0	0	0	0	0	0	0	0	0	0
SAL_VA5290AA	Bareilly ATCC-9115	Bareilly	0	0	0	0	0	0	0	0	0	0	0
SAL_VA5291AA	Bareilly ATCC-9115	Bareilly	0	0	0	0	0	0	0	0	0	0	0
SAL_VA5292AA	Bareilly ATCC-9115	Bareilly	0	0	0	0	0	0	0	0	0	0	0
SAL_VA5293AA	Bareilly ATCC-9115	Bareilly	0	0	0	0	0	0	0	0	0	0	0
SAL_VA5294AA	Bareilly ATCC-9115	Bareilly	0	0	0	0	0	0	0	0	0	0	0
SAL_VA5295AA	Bareilly ATCC-9115	Bareilly	0	0	0	0	0	0	0	0	0	0	0
SAL_VA5296AA	Bareilly ATCC-9115	Bareilly	0	0	0	0	0	0	0	0	0	0	0
SAL_VA5204AA	Bareilly ATCC-9115	Bareilly	0	0	0	0	0	0	0	0	0	0	0
SAL_VA5205AA	Bareilly ATCC-9115	Bareilly	0	0	0	0	0	0	0	0	0	0	0
SAL_VA5206AA	Bareilly ATCC-9115	Bareilly	0	0	0	0	0	0	0	0	0	0	0
SAL_VA5207AA	Bareilly ATCC-9115	Bareilly	0	0	0	0	0	0	0	0	0	0	0
SAL_VA5208AA	Bareilly ATCC-9115	Bareilly	0	0	0	0	0	0	0	0	0	0	0
SAL_VA5209AA	Bareilly ATCC-9115	Bareilly	0	0	0	0	0	0	0	0	0	0	0
SAL_VA5211AA	Bareilly ATCC-9115	Bareilly	0	0	0	0	0	0	0	0	0	0	0
SAL_VA5212AA	Bareilly ATCC-9115	Bareilly	0	0	0	0	0	0	0	0	0	0	0
SAL_VA5167AA	Bareilly ATCC-9115	Bareilly	0	0	0	0	0	0	0	0	0	0	0
SAL_VA5168AA	Bareilly ATCC-9115	Bareilly	0	0	0	0	0	0	0	0	0	0	0
SAL_VA5169AA	Bareilly ATCC-9115	Bareilly	0	0	0	0	0	0	0	0	0	0	0
SAL_VA5170AA	Bareilly ATCC-9115	Bareilly	0	0	0	0	0	0	0	0	0	0	0
SAL_VA5171AA	Bareilly ATCC-9115	Bareilly	0	0	0	0	0	0	0	0	0	0	0

SAL_VA5172AA	Bareilly ATCC-9115	Bareilly	0	0	0	0	0	0	0	0	0	0	0
SAL_VA5173AA	Bareilly ATCC-9115	Bareilly	0	0	0	0	0	0	0	0	0	0	0
SAL_VA5163AA	Bareilly ATCC-9115	Bareilly	0	0	0	0	0	0	0	0	0	0	0
SAL_VA5164AA	Bareilly ATCC-9115	Bareilly	0	0	0	0	0	0	0	0	0	0	0
SAL_VA5166AA	Bareilly ATCC-9115	Bareilly	0	0	0	0	0	0	0	0	0	0	0
SAL_VA5148AA	Bareilly ATCC-9115	Bareilly	0	0	0	0	0	0	0	0	0	0	0
SAL_VA5059AA	Bareilly ATCC-9115	Bareilly	0	0	0	0	0	0	0	0	0	0	0
SAL_VA5047AA	Bareilly ATCC-9115	Bareilly	0	0	0	0	0	0	0	0	0	0	0
SAL_VA5048AA	Bareilly ATCC-9115	Bareilly	0	0	0	0	0	0	0	0	0	0	0
SAL_VA5023AA	Bareilly ATCC-9115	Bareilly	0	0	0	0	0	0	0	0	0	0	0
SAL_VA5026AA	Bareilly ATCC-9115	Bareilly	0	0	0	0	0	0	0	0	0	0	0
SAL_VA5022AA	Bareilly ATCC-9115	Bareilly	0	0	0	0	0	0	0	0	0	0	0
SAL_VA5025AA	Bareilly ATCC-9115	Bareilly	0	0	0	0	0	0	0	0	0	0	0
SAL_VA5028AA	Bareilly ATCC-9115	Bareilly	0	0	0	0	0	0	0	0	0	0	0
SAL_VA5029AA	Bareilly ATCC-9115	Bareilly	0	0	0	0	0	0	0	0	0	0	0
SAL_VA5027AA	Bareilly ATCC-9115	Bareilly	0	0	0	0	0	0	0	0	0	0	0
SAL_VA5213AA	Bareilly ATCC-9115	Bareilly	0	0	0	0	0	0	0	0	0	0	0
SAL_VA5024AA	Bareilly ATCC-9115	Bareilly	0	0	0	0	0	0	0	0	0	0	0
SAL_VA5210AA	Bareilly ATCC-9115	Bareilly	0	0	0	0	0	0	0	0	0	0	0
SAL_VA4893AA	Bareilly ATCC-9115	Bareilly	0	0	0	0	0	0	0	0	0	0	0
SAL_VA4894AA	Bareilly ATCC-9115	Bareilly	0	0	0	0	0	0	0	0	0	0	0
SAL_VA4895AA	Bareilly ATCC-9115	Bareilly	0	0	0	0	0	0	0	0	0	0	0
SAL_VA4896AA	Bareilly ATCC-9115	Bareilly	0	0	0	0	0	0	0	0	0	0	0
SAL_VA5020AA	Bareilly ATCC-9115	Bareilly	0	0	0	0	0	0	0	0	0	0	0
SAL_VA5019AA	Bareilly ATCC-9115	Bareilly	0	0	0	0	0	0	0	0	0	0	0
SAL_VA4508AA	397388	Bareilly	0	0	0	0	0	0	0	0	0	0	0
SAL_VA4356AA	681343	Bareilly	0	0	0	0	0	0	0	0	0	0	0
SAL_VA4374AA	404920	Bareilly	0	0	0	0	0	0	0	0	0	0	0
SAL_VA6904AA	381332	Bareilly	0	0	0	0	0	0	0	0	0	0	0
SAL_VA7005AA	FSIS21923493	Bareilly	0	0	0	0	0	0	0	0	0	0	0
SAL_VA7222AA	685867	Bareilly	0	0	0	0	0	0	0	0	0	0	0
SAL_VA7178AA	682269	Bareilly	0	0	0	0	0	0	0	0	0	0	0
SAL_VA7103AA	678010	Bareilly	0	0	0	0	0	0	0	0	0	0	0
SAL_VA8305AA	363615	Bareilly	0	0	0	0	0	0	0	0	0	0	0
SAL_VA8537AA	347992	Bareilly	0	0	0	0	0	0	0	0	0	0	0
SAL_VA8508AA	690367	Bareilly	0	0	0	0	0	0	0	0	0	0	0
SAL_VA8529AA	340249	Bareilly	0	0	0	0	0	0	0	0	0	0	0
SAL_VA8501AA	360486	Bareilly	0	0	0	0	0	0	0	0	0	0	0
SAL_VA8755AA	318520	Bareilly	0	0	0	0	0	0	0	0	0	0	0
SAL_VA8657AA	345577	Bareilly	0	0	0	0	0	0	0	0	0	0	0
SAL_VA9010AA	293169	Bareilly	0	0	0	0	0	0	0	0	0	0	0
SAL_VA9035AA	298828	Bareilly	0	0	0	0	0	0	0	0	0	0	0
SAL_VA9062AA	FDA00013911	Bareilly	0	0	0	0	0	0	0	0	0	0	0
SAL_VA8966AA	293032	Bareilly	0	0	0	0	0	0	0	0	0	0	0
SAL_VA9450AA	277870	Bareilly	0	0	0	0	0	0	0	0	0	0	0
SAL_VA9441AA	278656	Bareilly	0	0	0	0	0	0	0	0	0	0	0
SAL_VA9319AA	278064	Bareilly	0	0	0	0	0	0	0	0	0	0	0
SAL_VA9315AA	280290	Bareilly	0	0	0	0	0	0	0	0	0	0	0
SAL_VA9612AA	271137	Bareilly	0	0	0	0	0	0	0	0	0	0	0
SAL_VA9552AA	266955	Bareilly	0	0	0	0	0	0	0	0	0	0	0
SAL_VA9569AA	286832	Bareilly	0	0	0	0	0	0	0	0	0	0	0
SAL_VA9570AA	692630	Bareilly	0	0	0	0	0	0	0	0	0	0	0
SAL_VA9542AA	693337	Bareilly	0	0	0	0	0	0	0	0	0	0	0
SAL_VA9555AA	270061	Bareilly	0	0	0	0	0	0	0	0	0	0	0
SAL_VA9816AA	256143	Bareilly	0	0	0	0	0	0	0	0	0	0	0
SAL_VA9761AA	255535	Bareilly	0	0	0	0	0	0	0	0	0	0	0
SAL_VA9704AA	260041	Bareilly	0	0	0	0	0	0	0	0	0	0	0
SAL_VA9714AA	270098	Bareilly	0	0	0	0	0	0	0	0	0	0	0
SAL_VA9667AA	256171	Bareilly	0	0	0	0	0	0	0	0	0	0	0
SAL_WA0020AA	221658	Bareilly	0	0	0	0	0	0	0	0	0	0	0
SAL_VA9910AA	241788	Bareilly	0	0	0	0	0	0	0	0	0	0	0
SAL_VA9911AA	235040	Bareilly	0	0	0	0	0	0	0	0	0	0	0
SAL_VA9927AA	235116	Bareilly	0	0	0	0	0	0	0	0	0	0	0
SAL_VA9885AA	261431	Bareilly	0	0	0	0	0	0	0	0	0	0	0
SAL_WA0269AA	215085	Bareilly	0	0	0	0	0	0	0	0	0	0	0
SAL_WA0203AA	218459	Bareilly	0	0	0	0	0	0	0	0	0	0	0
SAL_WA0208AA	198249	Bareilly	0	0	0	0	0	0	0	0	0	0	0
SAL_WA0223AA	235034	Bareilly	0	0	0	0	0	0	0	0	0	0	0
SAL_WA0182AA	227109	Bareilly	0	0	0	0	0	0	0	0	0	0	0
SAL_WA0148AA	221707	Bareilly	0	0	0	0	0	0	0	0	0	0	0
SAL_WA0484AA	195830	Bareilly	0	0	0	0	0	0	0	0	0	0	0
SAL_WA0504AA	185968	Bareilly	0	0	0	0	0	0	0	0	0	0	0
SAL_WA0465AA	205735	Bareilly	0	0	0	0	0	0	0	0	0	0	0

SAL_WA0289AA	211132	Bareilly	0	0	0	0	0	0	0	0	0	0	0
SAL_WA0776AA	216220	Bareilly	0	0	0	0	0	0	0	0	0	0	0
SAL_WA0726AA	203656	Bareilly	0	0	0	0	0	0	0	0	0	0	0
SAL_WA0959AA	173728	Bareilly	0	0	0	0	0	0	0	0	0	0	0
SAL_WA0865AA	142210	Bareilly	0	0	0	0	0	0	0	0	0	0	0
SAL_WA0887AA	154860	Bareilly	0	0	0	0	0	0	0	0	0	0	0
SAL_VA4086AA	CDPH_CSalm302	Bareilly	0	0	0	0	0	0	0	0	0	0	0
SAL_WA0381AA	178933	Bareilly	0	0	0	0	0	0	0	0	0	0	0
SAL_WA3248AA	RM_350	Bareilly	0	0	0	0	0	0	0	0	0	0	0
SAL_WA2375AA	1090801400	Bareilly	0	0	0	0	0	0	0	0	0	0	0
SAL_WA1188AA	697909	Bareilly	0	0	0	0	0	0	0	0	0	0	0
SAL_WA1729AA	107272	Bareilly	0	0	0	0	0	0	0	0	0	0	0
SAL_WA3332AA	707085	Bareilly	0	0	0	0	0	0	0	0	0	0	0
SAL_WA4316AA	19EP000645	Bareilly	0	0	0	0	0	0	0	0	0	0	0
SAL_WA4274AA	712534	Bareilly	0	0	0	0	0	0	0	0	0	0	0
SAL_HA4373AA	FDA00010762	Barmbek	0	0	0	0	0	0	0	0	0	0	0
SAL_PA8838AA	20-11-18-8100414-W	Barmbek	0	0	0	0	0	0	0	0	0	0	0
SAL_JA5364AA	PNUSAS011861	Bassadji	0	0	0	0	0	0	0	0	0	0	0
SAL_FA8173AA	PNUSAS002229	Bassadji	0	0	0	0	0	0	0	0	0	0	0
SAL_JA1639AA	PNUSAS011317	Batonrouge	0	0	0	0	0	0	0	0	0	0	0
SAL_JA5307AA	PNUSAS013192	Batonrouge	0	0	0	0	0	0	0	0	0	0	0
SAL_BA6424AA	3075	Beaudesert	0	0	0	0	0	0	0	0	0	0	0
SAL_DA3472AA	CFSAN023261	Beaudesert	0	0	0	0	0	0	0	0	0	0	0
SAL_HA4503AA	PNUSAS002744	Beaudesert	0	0	0	0	0	0	0	0	0	0	0
SAL_JA3286AA	PNUSAS011855	Beaudesert	0	0	0	0	0	0	0	0	0	0	0
SAL_JA3712AA	PNUSAS012988	Beaudesert	0	0	0	0	0	0	0	0	0	0	0
SAL_LA1149AA	PNUSAS018565	Beaudesert	0	0	0	0	0	0	0	0	0	0	0
SAL_HA5576AA	L00709-14	Beaudesert	0	0	0	0	0	0	0	0	0	0	0
SAL_PA9292AA	PNUSAS041218	Beaudesert	0	0	0	0	0	0	0	0	0	0	0
SAL_WA0464AA	198248	Beaudesert	0	0	0	0	0	0	0	0	0	0	0
SAL_EA0443AA	FCC0098	Bere	0	0	0	0	0	0	0	0	0	0	0
SAL_EA0285AA	FCC0075	Bere	0	0	0	0	0	0	0	0	0	0	0
SAL_DA8109AA	FAR0089	Bere	0	0	0	0	0	0	0	0	0	0	0
SAL_DA6315AA	FNE0113	Bere	0	0	0	0	0	0	0	0	0	0	0
SAL_DA6080AA	MOD1_Sal_842	Bere	0	0	0	0	0	0	0	0	0	0	0
SAL_DA3538AA	FNE0164	Bere	0	0	0	0	0	0	0	0	0	0	0
SAL_DA1404AA	H124920617	Bere	0	0	0	0	0	0	0	0	0	0	0
SAL_DA1372AA	H124780531	Bere	0	0	0	0	0	0	0	0	0	0	0
SAL_DA0354AA	FDA00003498	Bere	0	0	0	0	0	0	0	0	0	0	0
SAL_DA0330AA	FDA00003499	Bere	0	0	0	0	0	0	0	0	0	0	0
SAL_CA9771AA	FNW19i61	Bere	0	0	0	0	0	0	0	0	0	0	0
SAL_CA7027AA	FDA00001110	Bere	0	0	0	0	0	0	0	0	0	0	0
SAL_CA3819AA	84432	Bere	0	0	0	0	0	0	0	0	0	0	0
SAL_BA1882AA	FDA00001276	Bere	0	0	0	0	0	0	0	0	0	0	0
SAL_BA1365AA	FDA00002110	Bere	0	0	0	0	0	0	0	0	0	0	0
SAL_GA2857AA	CRJJGF_00054	Bere	0	0	0	0	0	0	0	0	0	0	0
SAL_FA8479AA	PNUSAS002188	Bere	0	0	0	0	0	0	0	0	0	0	0
SAL_IA9168AA	PNUSAS009476	Bere	0	0	0	0	0	0	0	0	0	0	0
SAL_JA0428AA	PNUSAS010686	Bere	0	0	0	0	0	0	0	0	0	0	0
SAL_KA2138AA	FDA00000510	Bere	0	0	0	0	0	0	0	0	0	0	0
SAL_LA6248AA	CFSAN069033	Bere	0	0	0	0	0	0	0	0	0	0	0
SAL_MA0330AA	FSIS11704673	Bere	0	0	0	0	0	0	0	0	0	0	0
SAL_DA0313AA	FDA00003500	Bere	0	0	0	0	0	0	0	0	0	0	0
SAL_EA4866AA	CFSAN034776	Bere	0	0	0	0	0	0	0	0	0	0	0
SAL_HA9279AA	FDA00001935	Bere	0	0	0	0	0	0	0	0	0	0	0
SAL_MA8651AA	ERS2013969	Bere	0	0	0	0	0	0	0	0	0	0	0
SAL_MA8608AA	ERS2014012	Bere	0	0	0	0	0	0	0	0	0	0	0
SAL_MA8615AA	ERS2014005	Bere	0	0	0	0	0	0	0	0	0	0	0
SAL_MA8584AA	ERS2014036	Bere	0	0	0	0	0	0	0	0	0	0	0
SAL_NA6373AA	FSIS11706962	Bere	0	0	0	0	0	0	0	0	0	0	0
SAL_QA6476AA	110973	Bere	0	0	0	0	0	0	0	0	0	0	0
SAL_RA1808AA	FDA00009755	Bere	0	0	0	0	0	0	0	0	0	0	0
SAL_PA3011AA	PNUSAS038934	Bere	0	0	0	0	0	0	0	0	0	0	0
SAL_UA7055AA	TTU_420	Bere	0	0	0	0	0	0	0	0	0	0	0
SAL_UA7110AA	TTU_412	Bere	0	0	0	0	0	0	0	0	0	0	0
SAL_UA8400AA	201502185	Bere	0	0	0	0	0	0	0	0	0	0	0
SAL_UA8591AA	201510137	Bere	0	0	0	0	0	0	0	0	0	0	0
SAL_VA8054AA	FSIS31901568	Bere	0	0	0	0	0	0	0	0	0	0	0
SAL_FA6306AA	CFSAN032159	Bergen	0	0	0	0	0	0	0	0	0	0	0
SAL_HA3010AA	PNUSAS004934	Bergen	0	0	0	0	0	0	0	0	0	0	0
SAL_JA7460AA	CFSAN059892	Bergen	0	0	0	0	0	0	0	0	0	0	0
SAL_JA1547AA	PNUSAS008914	Bergen	0	0	0	0	0	0	0	0	0	0	0
SAL_MA6070AA	ST350	Bergen	0	0	0	0	0	0	0	0	0	0	0

SAL_MA9146AA	FDA00003752	Bergen	0	0	0	0	0	0	0	0	0	0	0
SAL_NA6551AA	PNUSAS031495	Bergen	0	0	0	0	0	0	0	0	0	0	0
SAL_OA4558AA	FDA00003754	Bergen	0	0	0	0	0	0	0	0	0	0	0
SAL_TA8825AA	FSIS11815714	Bergen	0	0	0	0	0	0	0	0	0	0	0
SAL_UA0002AA	640976	Bergen	0	0	0	0	0	0	0	0	0	0	0
SAL_RA7222AA	296687	Berlin	0	0	0	0	0	0	0	0	0	0	0
SAL_BA1879AA	ADRDL-14-7632	Berta	5	4	6	64	0	0	0	0	0	0	0
SAL_EA0511AA	VA_WGS-00008	Berta	5	4	6	64	0	0	0	0	0	0	0
SAL_DA3757AA	CFSAN008692	Berta	5	4	6	65	0	0	0	0	0	0	0
SAL_FA5489AA	VA-WGS-16049	Berta	5	4	6	64	0	0	0	0	0	0	0
SAL_FA5484AA	VA-WGS-16054	Berta	5	4	6	64	0	0	0	0	0	0	0
SAL_FA5227AA	CFSAN047688	Berta	5	4	6	64	0	0	0	0	0	0	0
SAL_FA5228AA	IEH-NGS-SAL-04030	Berta	5	4	6	64	0	0	0	0	0	0	0
SAL_FA5225AA	IEH-NGS-SAL-04033	Berta	5	4	6	64	0	0	0	0	0	0	0
SAL_FA5224AA	CFSAN047691	Berta	5	4	6	64	0	0	0	0	0	0	0
SAL_FA5223AA	CFSAN047692	Berta	5	4	6	64	0	0	0	0	0	0	0
SAL_FA5222AA	IEH-NGS-SAL-04036	Berta	5	4	6	64	0	0	0	0	0	0	0
SAL_FA5126AA	VA-WGS-16036	Berta	5	4	6	64	0	0	0	0	0	0	0
SAL_FA5050AA	PNUSAS001758	Berta	5	4	6	64	0	0	0	0	0	0	0
SAL_FA4846AA	CVM-N19821	Berta	5	4	6	64	0	0	0	0	0	0	0
SAL_FA2786AA	CVM N57619F	Berta	5	4	6	64	0	0	0	0	0	0	0
SAL_FA2405AA	S001631_WI_M03453_	Berta	5	4	6	64	0	0	0	0	0	0	0
SAL_FA2407AA	S001629_WI_M03453_	Berta	5	4	6	64	0	0	0	0	0	0	0
SAL_FA2404AA	S001632_WI_M03453_	Berta	5	4	6	64	0	0	0	0	0	0	0
SAL_FA2161AA	FSIS1605832	Berta	5	4	6	64	0	0	0	0	0	0	0
SAL_FA1729AA	CFSAN046142	Berta	5	4	6	64	0	0	0	0	0	0	0
SAL_FA1559AA	IEH-NGS-SAL-03616	Berta	5	4	6	64	0	0	0	0	0	0	0
SAL_FA1560AA	CFSAN046127	Berta	5	4	6	64	0	0	0	0	0	0	0
SAL_FA1558AA	IEH-NGS-SAL-03617	Berta	5	4	6	64	0	0	0	0	0	0	0
SAL_FA1555AA	IEH-NGS-SAL-03620	Berta	5	4	6	64	0	0	0	0	0	0	0
SAL_FA0551AA	CVM-N25392	Berta	5	4	6	64	0	0	0	0	0	0	0
SAL_EA9153AA	WAPHL-SAL-A01102	Berta	5	4	6	64	0	0	0	0	0	0	0
SAL_EA9078AA	NY-N20056	Berta	5	4	6	64	0	0	0	0	0	0	0
SAL_EA8554AA	WAPHL-SAL-A00974	Berta	5	4	6	64	0	0	0	0	0	0	0
SAL_EA6874AA	CVM-N23818	Berta	5	4	6	64	0	0	0	0	0	0	0
SAL_EA5322AA	PNUSAS001169	Berta	5	4	6	64	0	0	0	0	0	0	0
SAL_EA4918AA	VA-WGS-00534	Berta	5	4	6	64	0	0	0	0	0	0	0
SAL_EA4919AA	VA-WGS-00533	Berta	5	4	6	64	0	0	0	0	0	0	0
SAL_EA3793AA	CVM N45924	Berta	5	4	6	64	0	0	0	0	0	0	0
SAL_EA3792AA	CVM N45925	Berta	5	4	6	64	0	0	0	0	0	0	0
SAL_EA3110AA	VA-WGS-00545	Berta	5	4	6	64	0	0	0	0	0	0	0
SAL_EA2979AA	CVM-N16430	Berta	5	4	6	64	0	0	0	0	0	0	0
SAL_EA2651AA	WAPHL_SAL-A00858	Berta	5	4	6	64	0	0	0	0	0	0	0
SAL_EA2131AA	CVM N52027	Berta	5	4	6	64	0	0	0	0	0	0	0
SAL_EA2116AA	CVM N52042	Berta	5	4	6	64	0	0	0	0	0	0	0
SAL_EA2105AA	CVM N52053	Berta	5	4	6	64	0	0	0	0	0	0	0
SAL_EA2118AA	CVM N52040	Berta	5	4	6	64	0	0	0	0	0	0	0
SAL_EA2073AA	CVM N53030	Berta	5	4	6	64	0	0	0	0	0	0	0
SAL_EA2032AA	CVM N53072	Berta	5	4	6	64	0	0	0	0	0	0	0
SAL_EA2003AA	CVM N53844	Berta	5	4	6	64	0	0	0	0	0	0	0
SAL_EA0792AA	AZ_TG68340	Berta	5	4	6	64	0	0	0	0	0	0	0
SAL_EA0028AA	NY_BAC0500002095	Berta	5	4	6	64	0	0	0	0	0	0	0
SAL_DA9918AA	NY_IDR1000016511	Berta	5	4	6	64	0	0	0	0	0	0	0
SAL_DA9868AA	NY_BAC0900001692	Berta	5	4	6	64	0	0	0	0	0	0	0
SAL_DA9705AA	MDH-2013-00154	Berta	5	4	6	64	0	0	0	0	0	0	0
SAL_DA9509AA	WAPHL-SAL-A00238	Berta	5	4	6	64	0	0	0	0	0	0	0
SAL_DA9475AA	WAPHL-SAL-A00240	Berta	5	4	6	64	0	0	0	0	0	0	0
SAL_DA9372AA	WAPHL-SAL-A00241	Berta	5	4	6	64	0	0	0	0	0	0	0
SAL_DA9362AA	WAPHL-SAL-A00239	Berta	5	4	6	64	0	0	0	0	0	0	0
SAL_DA8416AA	FNW18t56	Berta	5	4	6	64	0	0	0	0	0	0	0
SAL_DA8191AA	VA_WGS-00177	Berta	5	4	6	64	0	0	0	0	0	0	0
SAL_DA8185AA	VA_WGS-00176	Berta	5	4	6	64	0	0	0	0	0	0	0
SAL_DA7824AA	MDH-2014-00103	Berta	5	4	6	64	0	0	0	0	0	0	0
SAL_DA7575AA	NY-swgs1149	Berta	5	4	6	64	0	0	0	0	0	0	0
SAL_DA7172AA	CFSAN012662	Berta	5	4	6	64	0	0	0	0	0	0	0
SAL_DA6973AA	FL-NRM048	Berta	5	4	6	64	0	0	0	0	0	0	0
SAL_DA6751AA	FL-NRM049	Berta	5	4	6	64	0	0	0	0	0	0	0
SAL_DA4297AA	MOD1-Sal_901	Berta	5	4	6	64	0	0	0	0	0	0	0
SAL_DA4291AA	MOD1-Sal_900	Berta	5	4	6	64	0	0	0	0	0	0	0
SAL_DA4286AA	MOD1-Sal_899	Berta	5	4	6	64	0	0	0	0	0	0	0
SAL_DA3897AA	WAPHL_SAL-A00525	Berta	5	4	6	64	0	0	0	0	0	0	0
SAL_DA3889AA	WAPHL_SAL-A00530	Berta	5	4	6	64	0	0	0	0	0	0	0
SAL_DA3845AA	WAPHL_SAL-A00526	Berta	5	4	6	64	0	0	0	0	0	0	0

SAL_DA1865AA	NY-swgs1510	Berta	5	4	6	64	0	0	0	0	0	0	0
SAL_DA0231AA	VA-WGS-00346	Berta	5	4	6	64	0	0	0	0	0	0	0
SAL_CA9312AA	NY-32393	Berta	5	4	6	64	0	0	0	0	0	0	0
SAL_CA9206AA	PNUSAS000122	Berta	5	4	6	64	0	0	0	0	0	0	0
SAL_CA9208AA	PNUSAS000120	Berta	5	4	6	64	0	0	0	0	0	0	0
SAL_CA8658AA	FL-NRM150	Berta	5	4	6	64	0	0	0	0	0	0	0
SAL_CA8611AA	FL-NRM151	Berta	5	4	6	64	0	0	0	0	0	0	0
SAL_CA8137AA	FL-NRM239	Berta	5	4	6	64	0	0	0	0	0	0	0
SAL_CA7811AA	FL-NRM260	Berta	5	4	6	64	0	0	0	0	0	0	0
SAL_CA7549AA	OLC-759	Berta	5	4	6	64	0	0	0	0	0	0	0
SAL_CA7548AA	ATCC 8392	Berta	5	4	6	64	0	0	0	0	0	0	0
SAL_CA7532AA	FL-NRM290	Berta	5	4	6	64	0	0	0	0	0	0	0
SAL_CA7515AA	FL-NRM289	Berta	5	4	6	64	0	0	0	0	0	0	0
SAL_BA8839AA	NY_BAC0500008888	Berta	5	4	6	64	0	0	0	0	0	0	0
SAL_BA8799AA	NY_BAC0600010669	Berta	5	4	6	64	0	0	0	0	0	0	0
SAL_BA7831AA	7030-sc-2010-09-29-985	Berta	5	4	6	64	0	0	0	0	0	0	0
SAL_BA7802AA	3634-sc-2010-09-29-985	Berta	5	4	6	64	0	0	0	0	0	0	0
SAL_BA7391AA	SA20103550	Berta	5	4	6	64	0	0	0	0	0	0	0
SAL_BA7285AA	WAPHL_SAL-A00726	Berta	5	4	6	64	0	0	0	0	0	0	0
SAL_BA6955AA	WAPHL_SAL-A00737	Berta	5	4	6	64	0	0	0	0	0	0	0
SAL_BA6524AA	2015K-0191	Berta	5	4	6	64	0	0	0	0	0	0	0
SAL_BA4845AA	NY-N13661	Berta	5	4	6	64	0	0	0	0	0	0	0
SAL_BA1498AA	AZ-TG74920	Berta	5	4	6	64	0	0	0	0	0	0	0
SAL_BA1255AA	CFSAN035083	Berta	5	4	6	64	0	0	0	0	0	0	0
SAL_BA0752AA	ADRDL-14-15151	Berta	5	4	6	64	0	0	0	0	0	0	0
SAL_FA6087AA	VA-WGS-16064	Berta	5	4	6	64	0	0	0	0	0	0	0
SAL_FA6667AA	CVM-N27409	Berta	5	4	6	64	0	0	0	0	0	0	0
SAL_FA6834AA	CVM-N28363	Berta	5	4	6	64	0	0	0	0	0	0	0
SAL_FA6933AA	FSIS1606624	Berta	5	4	6	64	0	0	0	0	0	0	0
SAL_FA7281AA	FDA00002634	Berta	5	4	6	64	0	0	0	0	0	0	0
SAL_FA7280AA	FDA00002635	Berta	5	4	6	64	0	0	0	0	0	0	0
SAL_FA7656AA	CFSAN031237	Berta	5	4	6	64	0	0	0	0	0	0	0
SAL_FA7740AA	PNUSAS002339	Berta	5	4	6	64	0	0	0	0	0	0	0
SAL_FA7292AA	FSIS1606574	Berta	5	4	6	64	0	0	0	0	0	0	0
SAL_GA3692AA	BCW_2190	Berta	5	4	6	64	0	0	0	0	0	0	0
SAL_GA3691AA	BCW_2191	Berta	5	4	6	64	0	0	0	0	0	0	0
SAL_GA3689AA	BCW_2194	Berta	5	4	6	64	0	0	0	0	0	0	0
SAL_GA3456AA	BCW_3980	Berta	5	4	6	64	0	0	0	0	0	0	0
SAL_GA3443AA	BCW_3996	Berta	5	4	6	64	0	0	0	0	0	0	0
SAL_GA3434AA	BCW_4011	Berta	5	4	6	65	0	0	0	0	0	0	0
SAL_GA3425AA	BCW_2066	Berta	5	4	6	64	0	0	0	0	0	0	0
SAL_GA3408AA	BCW_2130	Berta	5	4	6	64	0	0	0	0	0	0	0
SAL_GA4392AA	PNUSAS002587	Berta	5	4	6	64	0	0	0	0	0	0	0
SAL_GA2866AA	CRJJGF_00045	Berta	5	4	6	64	0	0	0	0	0	0	0
SAL_GA2439AA	BCW_1976	Berta	5	4	6	64	0	0	0	0	0	0	0
SAL_FA9616AA	CVM-N23819	Berta	5	4	6	64	0	0	0	0	0	0	0
SAL_GA5131AA	PNUSAS002884	Berta	5	4	6	64	0	0	0	0	0	0	0
SAL_FA9305AA	CVM-N26653	Berta	5	4	6	64	0	0	0	0	0	0	0
SAL_FA9304AA	CVM-N26654	Berta	5	4	6	64	0	0	0	0	0	0	0
SAL_GA5164AA	V-CLASP-D-56	Berta	5	4	6	64	0	0	0	0	0	0	0
SAL_FA9048AA	CVM N40408	Berta	5	4	6	64	0	0	0	0	0	0	0
SAL_FA8911AA	CVM N42470	Berta	5	4	6	64	0	0	0	0	0	0	0
SAL_FA8910AA	CVM N42471	Berta	5	4	6	64	0	0	0	0	0	0	0
SAL_FA8680AA	CVM N31398	Berta	5	4	6	64	0	0	0	0	0	0	0
SAL_FA8679AA	CVM N31399	Berta	5	4	6	64	0	0	0	0	0	0	0
SAL_FA8674AA	CVM N31404	Berta	5	4	6	64	0	0	0	0	0	0	0
SAL_FA8542AA	CVM N37918	Berta	5	4	6	64	0	0	0	0	0	0	0
SAL_GA6389AA	E2016011018	Berta	5	4	6	65	0	0	0	0	0	0	0
SAL_GA6620AA	PNUSAS002982	Berta	5	4	6	64	0	0	0	0	0	0	0
SAL_GA7969AA	2014AM-0422	Berta	5	4	6	64	0	0	0	0	0	0	0
SAL_GA7920AA	2014AM-0327	Berta	5	4	6	64	0	0	0	0	0	0	0
SAL_GA7918AA	2014AM-3013	Berta	5	4	6	64	0	0	0	0	0	0	0
SAL_GA8121AA	PNUSAS003475	Berta	5	4	6	64	0	0	0	0	0	0	0
SAL_GA8195AA	PNUSAS003524	Berta	5	4	6	64	0	0	0	0	0	0	0
SAL_GA8638AA	PNUSAS003519	Berta	5	4	6	64	0	0	0	0	0	0	0
SAL_GA8790AA	PNUSAS003304	Berta	5	4	6	64	0	0	0	0	0	0	0
SAL_GA8791AA	PNUSAS003303	Berta	5	4	6	65	0	0	0	0	0	0	0
SAL_HA0002AA	PNUSAS004093	Berta	5	4	6	64	0	0	0	0	0	0	0
SAL_HA0261AA	PNUSAS004208	Berta	5	4	6	64	0	0	0	0	0	0	0
SAL_HA0445AA	PNUSAS003493	Berta	5	4	6	64	0	0	0	0	0	0	0
SAL_HA0876AA	PNUSAS004297	Berta	5	4	6	64	0	0	0	0	0	0	0
SAL_HA0743AA	PNUSAS004451	Berta	5	4	6	64	0	0	0	0	0	0	0
SAL_HA0634AA	2014AM-2265	Berta	5	4	6	64	0	0	0	0	0	0	0

SAL_HA0502AA	PNUSAS004420	Berta	5	4	6	64	0	0	0	0	0	0	0
SAL_HA0504AA	PNUSAS004418	Berta	5	4	6	64	0	0	0	0	0	0	0
SAL_HA0507AA	PNUSAS004415	Berta	5	4	6	64	0	0	0	0	0	0	0
SAL_HA0510AA	PNUSAS004412	Berta	5	4	6	64	0	0	0	0	0	0	0
SAL_HA0509AA	PNUSAS004413	Berta	5	4	6	64	0	0	0	0	0	0	0
SAL_HA0508AA	PNUSAS004414	Berta	5	4	6	64	0	0	0	0	0	0	0
SAL_HA0501AA	PNUSAS004421	Berta	5	4	6	64	0	0	0	0	0	0	0
SAL_HA0958AA	E2016015834	Berta	44	4	6	64	0	0	0	0	0	0	0
SAL_HA1260AA	PNUSAS004669	Berta	5	4	6	64	0	0	0	0	0	0	0
SAL_HA1266AA	PNUSAS004668	Berta	5	4	6	64	0	0	0	0	0	0	0
SAL_HA1682AA	PNUSAS004708	Berta	5	4	6	64	0	0	0	0	0	0	0
SAL_HA2899AA	FSIS1608070	Berta	5	4	6	64	0	0	0	0	0	0	0
SAL_HA2962AA	PNUSAS004766	Berta	5	4	6	64	0	0	0	0	0	0	0
SAL_HA4097AA	PNUSAS004842	Berta	5	4	6	64	0	0	0	0	0	0	0
SAL_HA4630AA	FSIS1608488	Berta	44	4	6	64	0	0	0	0	0	0	0
SAL_HA4471AA	PNUSAS005367	Berta	5	4	6	65	0	0	0	0	0	0	0
SAL_HA5082AA	S05025-14	Berta	5	4	6	64	0	0	0	0	0	0	0
SAL_HA5308AA	PNUSAS005193	Berta	5	4	6	64	0	0	0	0	0	0	0
SAL_HA5287AA	PNUSAS005203	Berta	5	4	6	64	0	0	0	0	0	0	0
SAL_HA5285AA	PNUSAS005204	Berta	5	4	6	64	0	0	0	0	0	0	0
SAL_HA0000AA	PNUSAS004095	Berta	5	4	6	64	0	0	0	0	0	0	0
SAL_HA6450AA	FSIS1608672	Berta	44	4	6	64	0	0	0	0	0	0	0
SAL_HA6273AA	PNUSAS005585	Berta	5	4	6	64	0	0	0	0	0	0	0
SAL_HA5838AA	PNUSAS005809	Berta	5	4	6	65	0	0	0	0	0	0	0
SAL_HA7685AA	FSIS1609107	Berta	5	4	6	64	0	0	0	0	0	0	0
SAL_HA7798AA	PNUSAS005314	Berta	5	4	6	64	0	0	0	0	0	0	0
SAL_HA7799AA	PNUSAS005313	Berta	5	4	6	64	0	0	0	0	0	0	0
SAL_HA8600AA	PNUSAS006406	Berta	5	4	6	64	0	0	0	0	0	0	0
SAL_HA8585AA	PNUSAS006029	Berta	5	4	6	64	0	0	0	0	0	0	0
SAL_HA8327AA	PNUSAS006070	Berta	5	4	6	64	0	0	0	0	0	0	0
SAL_HA8330AA	PNUSAS006446	Berta	5	4	6	64	0	0	0	0	0	0	0
SAL_HA8269AA	PNUSAS006495	Berta	5	4	6	64	0	0	0	0	0	0	0
SAL_HA9361AA	PNUSAS006181	Berta	5	4	6	64	0	0	0	0	0	0	0
SAL_HA9681AA	PNUSAS006905	Berta	5	4	6	64	0	0	0	0	0	0	0
SAL_IA0191AA	PNUSAS006068	Berta	5	4	6	64	0	0	0	0	0	0	0
SAL_IA0506AA	PNUSAS007407	Berta	5	4	6	64	0	0	0	0	0	0	0
SAL_IA2355AA	FSIS1609601	Berta	5	4	143	64	0	0	0	0	0	0	0
SAL_IA2364AA	FSIS1609399	Berta	5	4	6	64	0	0	0	0	0	0	0
SAL_IA2708AA	CFSAN059260	Berta	5	4	6	64	0	0	0	0	0	0	0
SAL_IA2604AA	PNUSAS007337	Berta	5	4	6	64	0	0	0	0	0	0	0
SAL_IA2533AA	PNUSAS006636	Berta	5	4	6	64	0	0	0	0	0	0	0
SAL_IA3724AA	PNUSAS006849	Berta	5	4	6	64	0	0	0	0	0	0	0
SAL_IA3733AA	PNUSAS006837	Berta	5	4	6	64	0	0	0	0	0	0	0
SAL_IA3713AA	PNUSAS006187	Berta	5	4	6	64	0	0	0	0	0	0	0
SAL_IA4660AA	FSIS1710066	Berta	44	4	6	64	0	0	0	0	0	0	0
SAL_IA4838AA	PNUSAS007796	Berta	5	4	6	64	0	0	0	0	0	0	0
SAL_IA4884AA	FSIS1710223	Berta	44	4	6	64	0	0	0	0	0	0	0
SAL_IA4954AA	CFSAN059261	Berta	5	4	6	64	0	0	0	0	0	0	0
SAL_IA5025AA	PNUSAS006684	Berta	5	4	6	64	0	0	0	0	0	0	0
SAL_IA6021AA	CFSAN059763	Berta	5	4	6	64	0	0	0	0	0	0	0
SAL_IA5554AA	FSIS1710190	Berta	5	4	6	64	0	0	0	0	0	0	0
SAL_IA5687AA	PNUSAS008115	Berta	5	4	6	64	0	0	0	0	0	0	0
SAL_IA7216AA	PNUSAS008754	Berta	5	4	6	64	0	0	0	0	0	0	0
SAL_IA7157AA	ADRDL-932	Berta	5	4	6	64	0	0	0	0	0	0	0
SAL_IA7471AA	PNUSAS009098	Berta	5	4	6	64	0	0	0	0	0	0	0
SAL_IA7555AA	FSIS1710541	Berta	5	4	6	270	0	0	0	0	0	0	0
SAL_IA8277AA	PNUSAS008792	Berta	5	4	6	64	0	0	0	0	0	0	0
SAL_IA8272AA	PNUSAS008796	Berta	5	4	6	64	0	0	0	0	0	0	0
SAL_IA8274AA	PNUSAS008795	Berta	5	4	6	64	0	0	0	0	0	0	0
SAL_IA8275AA	PNUSAS008793	Berta	5	4	6	64	0	0	0	0	0	0	0
SAL_IA8278AA	PNUSAS008794	Berta	5	4	6	65	0	0	0	0	0	0	0
SAL_IA8279AA	PNUSAS008789	Berta	5	4	6	64	0	0	0	0	0	0	0
SAL_IA8383AA	PNUSAS008677	Berta	5	4	143	64	0	0	0	0	0	0	0
SAL_IA8845AA	PNUSAS008382	Berta	5	4	6	65	0	0	0	0	0	0	0
SAL_IA9038AA	FSIS1710682	Berta	5	4	6	64	0	0	0	0	0	0	0
SAL_IA9023AA	FSIS1710575	Berta	5	4	6	64	0	0	0	0	0	0	0
SAL_IA9501AA	PNUSAS009584	Berta	5	4	6	64	0	0	0	0	0	0	0
SAL_IA9425AA	PNUSAS009583	Berta	5	4	6	65	0	0	0	0	0	0	0
SAL_IA9398AA	PNUSAS009302	Berta	5	4	6	64	0	0	0	0	0	0	0
SAL_IA9234AA	PNUSAS006960	Berta	5	4	6	64	0	0	0	0	0	0	0
SAL_IA9138AA	PNUSAS009252	Berta	5	4	6	64	0	0	0	0	0	0	0
SAL_IA9193AA	PNUSAS009847	Berta	5	4	6	64	0	0	0	0	0	0	0
SAL_IA9847AA	PNUSAS009286	Berta	5	4	6	64	0	0	0	0	0	0	0

SAL_IA9669AA	PNUSAS009383	Berta	5	4	6	65	0	0	0	0	0	0	0
SAL_IA9667AA	PNUSAS009385	Berta	5	4	6	64	0	0	0	0	0	0	0
SAL_IA9673AA	PNUSAS009386	Berta	5	4	6	64	0	0	0	0	0	0	0
SAL_IA9671AA	PNUSAS009388	Berta	5	4	6	65	0	0	0	0	0	0	0
SAL_IA9672AA	PNUSAS009387	Berta	5	4	6	64	0	0	0	0	0	0	0
SAL_IA9713AA	FSIS1710948	Berta	44	4	6	64	0	0	0	0	0	0	0
SAL_IA9650AA	PNUSAS009415	Berta	5	4	6	64	0	0	0	0	0	0	0
SAL_IA9648AA	PNUSAS009413	Berta	5	4	6	64	0	0	0	0	0	0	0
SAL_IA9651AA	PNUSAS009410	Berta	5	4	6	64	0	0	0	0	0	0	0
SAL_IA9655AA	PNUSAS009409	Berta	5	4	6	64	0	0	0	0	0	0	0
SAL_IA9653AA	PNUSAS009414	Berta	5	4	6	64	0	0	0	0	0	0	0
SAL_IA9652AA	PNUSAS009412	Berta	5	4	6	64	0	0	0	0	0	0	0
SAL_IA9658AA	PNUSAS009401	Berta	5	4	6	64	0	0	0	0	0	0	0
SAL_IA9657AA	PNUSAS009405	Berta	5	4	6	65	0	0	0	0	0	0	0
SAL_IA9659AA	PNUSAS009403	Berta	5	4	6	64	0	0	0	0	0	0	0
SAL_IA9661AA	PNUSAS009404	Berta	5	4	6	64	0	0	0	0	0	0	0
SAL_IA9663AA	PNUSAS009394	Berta	5	4	6	64	0	0	0	0	0	0	0
SAL_IA9654AA	PNUSAS009408	Berta	5	4	6	64	0	0	0	0	0	0	0
SAL_JA0095AA	PNUSAS009814	Berta	5	4	6	64	0	0	0	0	0	0	0
SAL_JA0239AA	PNUSAS010073	Berta	5	4	6	64	0	0	0	0	0	0	0
SAL_JA0893AA	PNUSAS008967	Berta	5	4	6	64	0	0	0	0	0	0	0
SAL_JA1027AA	PNUSAS010973	Berta	5	4	6	64	0	0	0	0	0	0	0
SAL_JA0906AA	PNUSAS008958	Berta	5	4	6	64	0	0	0	0	0	0	0
SAL_JA1656AA	PNUSAS010576	Berta	5	4	6	64	0	0	0	0	0	0	0
SAL_JA0571AA	PNUSAS009752	Berta	5	4	6	64	0	0	0	0	0	0	0
SAL_JA2156AA	FSIS1700076	Berta	5	4	6	64	0	0	0	0	0	0	0
SAL_JA2099AA	ADRDL-991	Berta	5	4	6	64	0	0	0	0	0	0	0
SAL_JA2079AA	FSIS1700189	Berta	5	4	6	64	0	0	0	0	0	0	0
SAL_JA2471AA	PNUSAS010842	Berta	5	4	6	64	0	0	0	0	0	0	0
SAL_JA2458AA	PNUSAS010772	Berta	5	4	6	64	0	0	0	0	0	0	0
SAL_JA2420AA	PNUSAS011731	Berta	5	4	6	65	0	0	0	0	0	0	0
SAL_JA3381AA	PNUSAS010572	Berta	5	4	6	64	0	0	0	0	0	0	0
SAL_JA3244AA	PNUSAS011101	Berta	5	4	6	64	0	0	0	0	0	0	0
SAL_JA2997AA	PNUSAS012197	Berta	5	4	6	64	0	0	0	0	0	0	0
SAL_JA3673AA	FSIS1700368	Berta	5	4	6	64	0	0	0	0	0	0	0
SAL_JA3476AA	PNUSAS011106	Berta	5	4	6	64	0	0	0	0	0	0	0
SAL_JA4057AA	PNUSAS011111	Berta	5	4	6	64	0	0	0	0	0	0	0
SAL_JA3873AA	PNUSAS012661	Berta	5	4	6	64	0	0	0	0	0	0	0
SAL_JA3862AA	PNUSAS012660	Berta	5	4	6	64	0	0	0	0	0	0	0
SAL_JA3754AA	PNUSAS012408	Berta	5	4	6	64	0	0	0	0	0	0	0
SAL_JA4954AA	PNUSAS012997	Berta	5	4	6	64	0	0	0	0	0	0	0
SAL_JA5012AA	PNUSAS012949	Berta	5	4	6	65	0	0	0	0	0	0	0
SAL_JA5251AA	CFSAN061057	Berta	5	4	6	64	0	0	0	0	0	0	0
SAL_JA5177AA	CFSAN061889	Berta	5	4	6	64	0	0	0	0	0	0	0
SAL_JA5182AA	CFSAN061890	Berta	5	4	6	64	0	0	0	0	0	0	0
SAL_JA5179AA	CFSAN061888	Berta	5	4	6	64	0	0	0	0	0	0	0
SAL_JA6241AA	PNUSAS010661	Berta	5	4	6	64	0	0	0	0	0	0	0
SAL_JA5916AA	PNUSAS004730	Berta	5	4	6	64	0	0	0	0	0	0	0
SAL_JA5855AA	PNUSAS013662	Berta	5	4	6	64	0	0	0	0	0	0	0
SAL_JA5178AA	CFSAN061887	Berta	5	4	6	64	0	0	0	0	0	0	0
SAL_JA5181AA	CFSAN061891	Berta	5	4	6	64	0	0	0	0	0	0	0
SAL_JA5611AA	PNUSAS013586	Berta	5	4	6	64	0	0	0	0	0	0	0
SAL_JA5740AA	PNUSAS013353	Berta	5	4	6	64	0	0	0	0	0	0	0
SAL_JA7490AA	FSIS1701126	Berta	5	4	6	64	0	0	0	0	0	0	0
SAL_JA7708AA	PNUSAS014236	Berta	5	4	6	64	0	0	0	0	0	0	0
SAL_JA7681AA	PNUSAS014260	Berta	5	4	6	64	0	0	0	0	0	0	0
SAL_JA7693AA	PNUSAS014263	Berta	5	4	6	64	0	0	0	0	0	0	0
SAL_JA8539AA	FSIS1701530	Berta	44	4	6	64	0	0	0	0	0	0	0
SAL_JA4676AA	CVM 43764	Berta	5	4	6	64	0	0	0	0	0	0	0
SAL_JA8899AA	FSIS1701546	Berta	5	4	6	64	0	0	0	0	0	0	0
SAL_JA9045AA	PNUSAS014797	Berta	5	4	6	64	0	0	0	0	0	0	0
SAL_JA9841AA	FSIS1701926	Berta	5	4	6	64	0	0	0	0	0	0	0
SAL_KA0001AA	PNUSAS015793	Berta	5	4	6	64	0	0	0	0	0	0	0
SAL_KA0012AA	PNUSAS015078	Berta	5	4	6	64	0	0	0	0	0	0	0
SAL_KA0702AA	2015AM-1239	Berta	5	4	6	64	0	0	0	0	0	0	0
SAL_KA0502AA	PNUSAS016166	Berta	5	4	6	64	0	0	0	0	0	0	0
SAL_KA0390AA	PNUSAS016352	Berta	5	4	6	64	0	0	0	0	0	0	0
SAL_KA0905AA	PNUSAS015193	Berta	5	4	6	65	0	0	0	0	0	0	0
SAL_KA0867AA	PNUSAS016309	Berta	5	4	6	64	0	0	0	0	0	0	0
SAL_KA1358AA	2015AM-1835	Berta	5	4	6	64	0	0	0	0	0	0	0
SAL_KA1065AA	PNUSAS016490	Berta	5	4	6	64	0	0	0	0	0	0	0
SAL_KA1058AA	PNUSAS016492	Berta	5	4	6	64	0	0	0	0	0	0	0
SAL_KA1021AA	FSIS1702026	Berta	5	4	6	64	0	0	0	0	0	0	0

SAL_KA0984AA	2015AM-0523	Berta	5	4	6	65	0	0	0	0	0	0	0
SAL_KA1754AA	PNUSAS015862	Berta	5	4	6	64	0	0	0	0	0	0	0
SAL_KA1944AA	PNUSAS016742	Berta	5	4	6	65	0	0	0	0	0	0	0
SAL_KA2238AA	PNUSAS017256	Berta	5	4	6	65	0	0	0	0	0	0	0
SAL_KA2906AA	PNUSAS014595	Berta	5	4	6	64	0	0	0	0	0	0	0
SAL_KA2909AA	PNUSAS016919	Berta	5	4	6	64	0	0	0	0	0	0	0
SAL_KA2776AA	PNUSAS017307	Berta	5	4	6	64	0	0	0	0	0	0	0
SAL_KA3424AA	PNUSAS017327	Berta	5	4	6	64	0	0	0	0	0	0	0
SAL_KA3270AA	PNUSAS017826	Berta	5	4	6	64	0	0	0	0	0	0	0
SAL_KA4037AA	RM_356	Berta	5	4	6	64	0	0	0	0	0	0	0
SAL_KA4009AA	RM_260	Berta	5	4	6	64	0	0	0	0	0	0	0
SAL_KA3998AA	RM_282	Berta	5	4	6	64	0	0	0	0	0	0	0
SAL_KA5407AA	PNUSAS017650	Berta	5	4	6	64	0	0	0	0	0	0	0
SAL_KA5186AA	PNUSAS018034	Berta	5	4	6	65	0	0	0	0	0	0	0
SAL_KA5658AA	2015AM-1183	Berta	5	4	6	64	0	0	0	0	0	0	0
SAL_KA5780AA	PNUSAS013050	Berta	5	4	6	64	0	0	0	0	0	0	0
SAL_KA5873AA	PNUSAS016549	Berta	5	4	6	64	0	0	0	0	0	0	0
SAL_KA6619AA	PNUSAS019727	Berta	5	4	6	64	0	0	0	0	0	0	0
SAL_KA6642AA	FSIS1703216	Berta	5	4	6	64	0	0	0	0	0	0	0
SAL_KA6589AA	PNUSAS019217	Berta	5	4	6	64	0	0	0	0	0	0	0
SAL_KA6459AA	2015AM-1458	Berta	5	4	6	64	0	0	0	0	0	0	0
SAL_KA7148AA	FSIS1703410	Berta	5	4	6	64	0	0	0	0	0	0	0
SAL_KA7478AA	PNUSAS019317	Berta	5	4	6	64	0	0	0	0	0	0	0
SAL_KA7416AA	PNUSAS015561	Berta	5	4	6	64	0	0	0	0	0	0	0
SAL_KA7864AA	PNUSAS019995	Berta	5	4	6	64	0	0	0	0	0	0	0
SAL_KA7863AA	PNUSAS019877	Berta	5	4	6	64	0	0	0	0	0	0	0
SAL_KA8169AA	2015AM-1550	Berta	5	4	6	64	0	0	0	0	0	0	0
SAL_KA8307AA	PNUSAS018287	Berta	5	4	6	64	0	0	0	0	0	0	0
SAL_KA8216AA	PNUSAS019538	Berta	5	4	6	64	0	0	0	0	0	0	0
SAL_KA8269AA	PNUSAS020026	Berta	5	4	6	65	0	0	0	0	0	0	0
SAL_KA8361AA	2015AM-1055	Berta	5	4	6	64	0	0	0	0	0	0	0
SAL_KA8647AA	PNUSAS020105	Berta	5	4	6	64	0	0	0	0	0	0	0
SAL_KA8640AA	PNUSAS020101	Berta	5	4	6	64	0	0	0	0	0	0	0
SAL_KA8643AA	PNUSAS020099	Berta	5	4	6	64	0	0	0	0	0	0	0
SAL_KA8568AA	PNUSAS019756	Berta	5	4	6	65	0	0	0	0	0	0	0
SAL_KA8652AA	PNUSAS020100	Berta	5	4	6	64	0	0	0	0	0	0	0
SAL_KA8946AA	2015AM-0739	Berta	5	4	6	64	0	0	0	0	0	0	0
SAL_KA9060AA	PNUSAS020744	Berta	5	4	6	64	0	0	0	0	0	0	0
SAL_KA9017AA	PNUSAS020497	Berta	5	4	6	64	0	0	0	0	0	0	0
SAL_KA9688AA	PNUSAS020667	Berta	5	4	6	64	0	0	0	0	0	0	0
SAL_KA9808AA	PNUSAS019768	Berta	5	4	6	64	0	0	0	0	0	0	0
SAL_KA9902AA	FSIS1703469	Berta	5	4	143	64	0	0	0	0	0	0	0
SAL_LA0261AA	PNUSAS021511	Berta	5	4	6	64	0	0	0	0	0	0	0
SAL_LA0229AA	PNUSAS021099	Berta	5	4	6	64	0	0	0	0	0	0	0
SAL_LA0216AA	2015AM-1753	Berta	5	4	6	64	0	0	0	0	0	0	0
SAL_LA0217AA	2015AM-1754	Berta	5	4	6	64	0	0	0	0	0	0	0
SAL_LA0453AA	PNUSAS021613	Berta	5	4	6	64	0	0	0	0	0	0	0
SAL_LA1142AA	CFSAN067276	Berta	5	4	6	64	0	0	0	0	0	0	0
SAL_LA1252AA	PNUSAS021066	Berta	5	4	6	64	0	0	0	0	0	0	0
SAL_LA1208AA	PNUSAS018719	Berta	5	4	6	64	0	0	0	0	0	0	0
SAL_LA1171AA	PNUSAS021599	Berta	5	4	6	64	0	0	0	0	0	0	0
SAL_LA2191AA	PNUSAS022163	Berta	5	4	6	64	0	0	0	0	0	0	0
SAL_LA2177AA	PNUSAS022164	Berta	5	4	6	64	0	0	0	0	0	0	0
SAL_LA3330AA	PNUSAS020103	Berta	5	4	6	64	0	0	0	0	0	0	0
SAL_LA3309AA	PNUSAS019021	Berta	5	4	6	64	0	0	0	0	0	0	0
SAL_LA3323AA	PNUSAS020102	Berta	5	4	6	64	0	0	0	0	0	0	0
SAL_LA3866AA	PNUSAS021955	Berta	5	4	6	64	0	0	0	0	0	0	0
SAL_LA3882AA	PNUSAS021963	Berta	5	4	6	64	0	0	0	0	0	0	0
SAL_LA4047AA	RM_366	Berta	5	4	6	64	0	0	0	0	0	0	0
SAL_LA3665AA	FSIS21720141	Berta	5	4	6	64	0	0	0	0	0	0	0
SAL_LA4967AA	PNUSAS021536	Berta	5	4	6	64	0	0	0	0	0	0	0
SAL_LA4968AA	PNUSAS021530	Berta	5	4	6	64	0	0	0	0	0	0	0
SAL_KA9512AA	PNUSAS020716	Berta	5	4	6	64	0	0	0	0	0	0	0
SAL_LA6793AA	PNUSAS023532	Berta	5	4	6	64	0	0	0	0	0	0	0
SAL_LA6608AA	PNUSAS022481	Berta	5	4	6	64	0	0	0	0	0	0	0
SAL_LA6578AA	PNUSAS022201	Berta	5	4	6	65	0	0	0	0	0	0	0
SAL_LA6465AA	PNUSAS023428	Berta	5	4	6	64	0	0	0	0	0	0	0
SAL_LA6481AA	PNUSAS023335	Berta	5	4	6	64	0	0	0	0	0	0	0
SAL_LA6426AA	PNUSAS024133	Berta	5	4	6	64	0	0	0	0	0	0	0
SAL_LA6307AA	PNUSAS022644	Berta	5	4	6	65	0	0	0	0	0	0	0
SAL_LA6902AA	PNUSAS022314	Berta	5	4	6	64	0	0	0	0	0	0	0
SAL_LA7463AA	PNUSAS022911	Berta	5	4	6	64	0	0	0	0	0	0	0
SAL_LA7452AA	PNUSAS021775	Berta	5	4	6	65	0	0	0	0	0	0	0

SAL_LA7418AA	PNUSAS023729	Berta	5	4	6	64	0	0	0	0	0	0	0
SAL_LA9885AA	2015AM-0116	Berta	5	4	6	64	0	0	0	0	0	0	0
SAL_LA9266AA	PNUSAS024394	Berta	5	4	6	64	0	0	0	0	0	0	0
SAL_LA9179AA	PNUSAS024470	Berta	5	4	6	65	0	0	0	0	0	0	0
SAL_MA0230AA	PNUSAS024322	Berta	5	4	6	64	0	0	0	0	0	0	0
SAL_MA0196AA	PNUSAS025045	Berta	5	4	6	64	0	0	0	0	0	0	0
SAL_MA0182AA	PNUSAS024397	Berta	5	4	6	64	0	0	0	0	0	0	0
SAL_MA0539AA	PNUSAS022134	Berta	5	4	6	64	0	0	0	0	0	0	0
SAL_MA0501AA	PNUSAS022918	Berta	5	4	6	64	0	0	0	0	0	0	0
SAL_MA1012AA	PNUSAS025345	Berta	5	4	6	64	0	0	0	0	0	0	0
SAL_MA1015AA	PNUSAS025087	Berta	5	4	6	64	0	0	0	0	0	0	0
SAL_MA1639AA	PNUSAS025751	Berta	5	4	6	65	0	0	0	0	0	0	0
SAL_MA1576AA	PNUSAS024328	Berta	5	4	6	64	0	0	0	0	0	0	0
SAL_MA1722AA	PNUSAS026005	Berta	5	4	6	64	0	0	0	0	0	0	0
SAL_MA2901AA	PNUSAS014317	Berta	5	4	6	65	0	0	0	0	0	0	0
SAL_MA2877AA	PNUSAS025207	Berta	5	4	6	64	0	0	0	0	0	0	0
SAL_MA2842AA	PNUSAS026343	Berta	5	4	6	64	0	0	0	0	0	0	0
SAL_MA3420AA	PNUSAS026392	Berta	5	4	6	64	0	0	0	0	0	0	0
SAL_MA3928AA	CFSAN070508	Berta	5	4	6	64	0	0	0	0	0	0	0
SAL_MA3915AA	CFSAN070510	Berta	5	4	6	64	0	0	0	0	0	0	0
SAL_MA4702AA	PNUSAS025987	Berta	5	4	6	64	0	0	0	0	0	0	0
SAL_MA4664AA	PNUSAS025992	Berta	5	4	6	64	0	0	0	0	0	0	0
SAL_MA4631AA	PNUSAS023227	Berta	5	4	6	64	0	0	0	0	0	0	0
SAL_MA4498AA	PNUSAS026721	Berta	5	4	6	64	0	0	0	0	0	0	0
SAL_MA4886AA	PNUSAS025800	Berta	5	4	6	64	0	0	0	0	0	0	0
SAL_BA6517AA	2015K-0228	Berta	5	4	6	65	0	0	0	0	0	0	0
SAL_DA6205AA	FSE0107	Berta	5	4	6	64	0	0	0	0	0	0	0
SAL_EA2002AA	CVM N53845	Berta	5	4	6	64	0	0	0	0	0	0	0
SAL_FA2408AA	S001628_WI_M03453_(	Berta	5	4	6	65	0	0	0	0	0	0	0
SAL_IA9521AA	PNUSAS009261	Berta	5	4	6	64	0	0	0	0	0	0	0
SAL_IA9232AA	PNUSAS009686	Berta	5	4	6	65	0	0	0	0	0	0	0
SAL_IA9664AA	PNUSAS009407	Berta	5	4	6	64	0	0	0	0	0	0	0
SAL_JA0112AA	PNUSAS009648	Berta	5	4	6	65	0	0	0	0	0	0	0
SAL_JA8744AA	PNUSAS014555	Berta	5	4	6	64	0	0	0	0	0	0	0
SAL_KA5493AA	PNUSAS017652	Berta	5	4	6	64	0	0	0	0	0	0	0
SAL_KA5818AA	PNUSAS018452	Berta	5	4	6	64	0	0	0	0	0	0	0
SAL_LA6520AA	PNUSAS024056	Berta	5	4	6	64	0	0	0	0	0	0	0
SAL_MA2240AA	FSIS21720340	Berta	44	4	6	64	0	0	0	0	0	0	0
SAL_MA6675AA	PNUSAS027132	Berta	5	4	6	64	0	0	0	0	0	0	0
SAL_MA7112AA	PNUSAS025540	Berta	5	4	6	64	0	0	0	0	0	0	0
SAL_MA7009AA	PNUSAS026012	Berta	5	4	6	64	0	0	0	0	0	0	0
SAL_MA7657AA	PNUSAS028231	Berta	5	4	6	65	0	0	0	0	0	0	0
SAL_MA7594AA	PNUSAS028266	Berta	5	4	6	64	0	0	0	0	0	0	0
SAL_MA7524AA	PNUSAS028235	Berta	5	4	6	64	0	0	0	0	0	0	0
SAL_MA7809AA	PNUSAS026584	Berta	5	4	6	64	0	0	0	0	0	0	0
SAL_MA7769AA	PNUSAS028189	Berta	5	4	6	65	0	0	0	0	0	0	0
SAL_MA8406AA	PNUSAS028517	Berta	5	4	6	64	0	0	0	0	0	0	0
SAL_MA8226AA	PNUSAS029012	Berta	5	4	6	64	0	0	0	0	0	0	0
SAL_MA8169AA	PNUSAS024726	Berta	5	4	6	64	0	0	0	0	0	0	0
SAL_MA8912AA	PNUSAS029114	Berta	5	4	6	64	0	0	0	0	0	0	0
SAL_MA8826AA	FSIS11705649	Berta	44	4	6	64	0	0	0	0	0	0	0
SAL_MA9027AA	FSIS11705336	Berta	5	4	6	64	0	0	0	0	0	0	0
SAL_MA9781AA	CVM N16S242	Berta	5	4	6	64	0	0	0	0	0	0	0
SAL_MA9786AA	CVM N16S343	Berta	5	4	6	64	0	0	0	0	0	0	0
SAL_MA9784AA	CVM N16S349	Berta	5	4	6	64	0	0	0	0	0	0	0
SAL_MA9673AA	CVM N16S301	Berta	5	4	6	64	0	0	0	0	0	0	0
SAL_MA9591AA	CVM N16S267	Berta	5	4	6	64	0	0	0	0	0	0	0
SAL_MA9598AA	CVM N16S327	Berta	5	4	6	64	0	0	0	0	0	0	0
SAL_MA9599AA	CVM N16S328	Berta	5	4	6	64	0	0	0	0	0	0	0
SAL_NA0725AA	PNUSAS029262	Berta	5	4	6	64	0	0	0	0	0	0	0
SAL_NA1774AA	PNUSAS028607	Berta	5	4	6	65	0	0	0	0	0	0	0
SAL_NA1778AA	PNUSAS025470	Berta	5	4	6	64	0	0	0	0	0	0	0
SAL_NA1753AA	PNUSAS025288	Berta	5	4	6	64	0	0	0	0	0	0	0
SAL_NA1699AA	PNUSAS025552	Berta	5	4	6	64	0	0	0	0	0	0	0
SAL_NA1397AA	FSIS11705096	Berta	5	4	6	64	0	0	0	0	0	0	0
SAL_NA2085AA	PNUSAS023825	Berta	5	4	6	64	0	0	0	0	0	0	0
SAL_NA2467AA	PNUSAS029919	Berta	5	4	6	64	0	0	0	0	0	0	0
SAL_NA2472AA	PNUSAS029916	Berta	5	4	6	64	0	0	0	0	0	0	0
SAL_NA2337AA	FSIS21720763	Berta	44	4	6	64	0	0	0	0	0	0	0
SAL_NA2965AA	PNUSAS029772	Berta	5	4	6	65	0	0	0	0	0	0	0
SAL_NA3073AA	PNUSAS030313	Berta	5	4	6	65	0	0	0	0	0	0	0
SAL_NA3036AA	PNUSAS029842	Berta	5	4	6	64	0	0	0	0	0	0	0
SAL_NA3245AA	CFSAN073604	Berta	5	4	6	64	0	0	0	0	0	0	0

SAL_NA3248AA	CFSAN073603	Berta	5	4	6	64	0	0	0	0	0	0	0
SAL_NA3469AA	PNUSAS024291	Berta	5	4	6	64	0	0	0	0	0	0	0
SAL_NA3423AA	PNUSAS027649	Berta	5	4	6	64	0	0	0	0	0	0	0
SAL_NA3730AA	PNUSAS030896	Berta	5	4	6	64	0	0	0	0	0	0	0
SAL_NA3591AA	PNUSAS030170	Berta	5	4	6	64	0	0	0	0	0	0	0
SAL_NA3528AA	PNUSAS031052	Berta	5	4	6	64	0	0	0	0	0	0	0
SAL_NA4247AA	PNUSAS030909	Berta	5	4	6	64	0	0	0	0	0	0	0
SAL_NA4211AA	PNUSAS031034	Berta	5	4	6	65	0	0	0	0	0	0	0
SAL_NA4380AA	FSIS31800030	Berta	44	4	6	64	0	0	0	0	0	0	0
SAL_NA4382AA	FSIS21720892	Berta	5	4	6	64	0	0	0	0	0	0	0
SAL_NA4544AA	FSIS11706944	Berta	5	4	6	64	0	0	0	0	0	0	0
SAL_NA4708AA	PNUSAS031302	Berta	5	4	6	64	0	0	0	0	0	0	0
SAL_NA4871AA	PNUSAS030709	Berta	5	4	6	64	0	0	0	0	0	0	0
SAL_NA5257AA	2015AM-2005	Berta	5	4	6	64	0	0	0	0	0	0	0
SAL_NA5527AA	PNUSAS032101	Berta	5	4	6	64	0	0	0	0	0	0	0
SAL_NA5453AA	PNUSAS031791	Berta	5	4	6	65	0	0	0	0	0	0	0
SAL_NA6192AA	CFSAN073608	Berta	5	4	6	64	0	0	0	0	0	0	0
SAL_NA6028AA	2015AM-2430	Berta	5	4	6	64	0	0	0	0	0	0	0
SAL_NA5960AA	PNUSAS031882	Berta	5	4	6	64	0	0	0	0	0	0	0
SAL_NA5929AA	PNUSAS028417	Berta	5	4	6	64	0	0	0	0	0	0	0
SAL_NA5823AA	2015AM-2749	Berta	5	4	6	64	0	0	0	0	0	0	0
SAL_NA5848AA	CFSAN073613	Berta	5	4	6	64	0	0	0	0	0	0	0
SAL_NA6563AA	PNUSAS032332	Berta	5	4	6	64	0	0	0	0	0	0	0
SAL_NA7005AA	PNUSAS029874	Berta	5	4	6	64	0	0	0	0	0	0	0
SAL_NA6899AA	PNUSAS031763	Berta	5	4	6	64	0	0	0	0	0	0	0
SAL_NA6830AA	PNUSAS032387	Berta	5	4	6	328	0	0	0	0	0	0	0
SAL_NA6749AA	PNUSAS032470	Berta	5	4	6	64	0	0	0	0	0	0	0
SAL_NA7518AA	CFSAN074820	Berta	5	4	6	64	0	0	0	0	0	0	0
SAL_NA8212AA	PNUSAS031795	Berta	44	4	6	64	0	0	0	0	0	0	0
SAL_NA8508AA	PNUSAS032898	Berta	5	4	6	64	0	0	0	0	0	0	0
SAL_NA8372AA	PNUSAS033038	Berta	5	4	6	64	0	0	0	0	0	0	0
SAL_NA8430AA	PNUSAS032567	Berta	5	4	6	64	0	0	0	0	0	0	0
SAL_NA8754AA	FSIS21821044	Berta	5	4	6	64	0	0	0	0	0	0	0
SAL_NA8713AA	PNUSAS032885	Berta	5	4	6	64	0	0	0	0	0	0	0
SAL_NA8697AA	PNUSAS032954	Berta	5	4	6	64	0	0	0	0	0	0	0
SAL_NA8633AA	PNUSAS033399	Berta	5	4	6	64	0	0	0	0	0	0	0
SAL_NA9474AA	PNUSAS032869	Berta	5	4	6	64	0	0	0	0	0	0	0
SAL_NA9337AA	PNUSAS033574	Berta	5	4	6	64	0	0	0	0	0	0	0
SAL_NA9208AA	CFSAN075246	Berta	5	4	6	64	0	0	0	0	0	0	0
SAL_OA0243AA	PNUSAS034099	Berta	5	4	6	64	0	0	0	0	0	0	0
SAL_OA0851AA	PNUSAS031847	Berta	5	4	6	64	0	0	0	0	0	0	0
SAL_OA0974AA	CFSAN075599	Berta	5	4	6	64	0	0	0	0	0	0	0
SAL_OA1160AA	PNUSAS033378	Berta	5	4	6	64	0	0	0	0	0	0	0
SAL_OA1164AA	PNUSAS031614	Berta	5	4	6	64	0	0	0	0	0	0	0
SAL_OA1263AA	PNUSAS033914	Berta	5	4	6	64	0	0	0	0	0	0	0
SAL_OA7388AA	PNUSAS036879	Berta	5	4	6	64	0	0	0	0	0	0	0
SAL_OA7448AA	FSIS11808665	Berta	5	4	6	64	0	0	0	0	0	0	0
SAL_PA0056AA	PNUSAS037201	Berta	5	4	6	64	0	0	0	0	0	0	0
SAL_OA5073AA	PNUSAS034536	Berta	5	4	6	64	0	0	0	0	0	0	0
SAL_OA5077AA	PNUSAS034537	Berta	5	4	6	64	0	0	0	0	0	0	0
SAL_OA4810AA	PNUSAS036064	Berta	5	4	6	64	0	0	0	0	0	0	0
SAL_OA4584AA	PNUSAS036931	Berta	5	4	6	65	0	0	0	0	0	0	0
SAL_OA4265AA	PNUSAS032901	Berta	5	4	6	64	0	0	0	0	0	0	0
SAL_OA4269AA	PNUSAS027959	Berta	5	4	6	64	0	0	0	0	0	0	0
SAL_OA3804AA	CFSAN075636	Berta	5	4	6	64	0	0	0	0	0	0	0
SAL_OA3819AA	FSIS21821274	Berta	5	4	6	64	0	0	0	0	0	0	0
SAL_PA0489AA	CFSAN076792	Berta	5	4	6	64	0	0	0	0	0	0	0
SAL_PA0493AA	CFSAN076783	Berta	5	4	6	64	0	0	0	0	0	0	0
SAL_PA0502AA	CFSAN077976	Berta	5	4	6	64	0	0	0	0	0	0	0
SAL_PA0505AA	CFSAN076791	Berta	5	4	6	64	0	0	0	0	0	0	0
SAL_PA0466AA	CFSAN076785	Berta	5	4	6	64	0	0	0	0	0	0	0
SAL_PA0471AA	CFSAN076786	Berta	5	4	6	64	0	0	0	0	0	0	0
SAL_PA0477AA	CFSAN076787	Berta	5	4	6	64	0	0	0	0	0	0	0
SAL_PA0901AA	CFSAN077993	Berta	5	4	6	64	0	0	0	0	0	0	0
SAL_PA0885AA	CFSAN077996	Berta	5	4	6	64	0	0	0	0	0	0	0
SAL_PA0883AA	CFSAN077987	Berta	5	4	6	64	0	0	0	0	0	0	0
SAL_PA0822AA	CFSAN077994	Berta	5	4	6	64	0	0	0	0	0	0	0
SAL_PA1183AA	CFSAN077998	Berta	5	4	6	64	0	0	0	0	0	0	0
SAL_PA1398AA	PNUSAS038274	Berta	5	4	6	64	0	0	0	0	0	0	0
SAL_PA1384AA	PNUSAS036687	Berta	5	4	6	64	0	0	0	0	0	0	0
SAL_PA1322AA	PNUSAS038102	Berta	5	4	6	64	0	0	0	0	0	0	0
SAL_PA1473AA	PNUSAS037093	Berta	5	4	6	64	0	0	0	0	0	0	0
SAL_PA1847AA	CFSAN078654	Berta	5	4	6	64	0	0	0	0	0	0	0

SAL_PA1950AA	CFSAN078661	Berta	5	4	6	64	0	0	0	0	0	0	0	
SAL_PA1952AA	CFSAN078655	Berta	5	4	6	64	0	0	0	0	0	0	0	
SAL_PA2104AA	CFSAN078656	Berta	5	4	6	64	0	0	0	0	0	0	0	
SAL_PA2472AA	CFSAN078644	Berta	5	4	6	64	0	0	0	0	0	0	0	
SAL_PA2474AA	CFSAN078647	Berta	5	4	6	64	0	0	0	0	0	0	0	
SAL_PA3329AA	CFSAN079998	Berta	5	4	6	64	0	0	0	0	0	0	0	
SAL_PA3321AA	CFSAN079999	Berta	5	4	6	64	0	0	0	0	0	0	0	
SAL_PA3797AA	CFSAN080015	Berta	5	4	6	64	0	0	0	0	0	0	0	
SAL_PA3883AA	PNUSAS039426	Berta	5	4	6	64	0	0	0	0	0	0	0	
SAL_PA3849AA	PNUSAS039386	Berta	5	4	6	65	0	0	0	0	0	0	0	
SAL_PA4324AA	107755	Berta	5	4	6	64	0	0	0	0	0	0	0	
SAL_PA4490AA	PNUSAS034417	Berta	5	4	6	64	0	0	0	0	0	0	0	
SAL_PA4419AA	PNUSAS039850	Berta	5	4	6	64	0	0	0	0	0	0	0	
SAL_PA4399AA	PNUSAS039851	Berta	5	4	6	64	0	0	0	0	0	0	0	
SAL_PA4364AA	PNUSAS039812	Berta	5	4	6	64	0	0	0	0	0	0	0	
SAL_PA4735AA	PNUSAS040002	Berta	5	4	6	64	0	0	0	0	0	0	0	
SAL_PA5047AA	CFSAN080456	Berta	5	4	6	64	0	0	0	0	0	0	0	
SAL_PA4987AA	CFSAN080452	Berta	5	4	6	64	0	0	0	0	0	0	0	
SAL_PA4988AA	CFSAN080414	Berta	5	4	6	64	0	0	0	0	0	0	0	
SAL_PA4989AA	CFSAN080415	Berta	5	4	6	64	0	0	0	0	0	0	0	
SAL_PA5296AA	PNUSAS039663	Berta	5	4	6	64	0	0	0	0	0	0	0	
SAL_PA5265AA	CFSAN080578	Berta	5	4	6	64	0	0	0	0	0	0	0	
SAL_PA5268AA	CFSAN080579	Berta	5	4	6	64	0	0	0	0	0	0	0	
SAL_PA5932AA	PNUSAS038400	Berta	5	4	6	64	0	0	0	0	0	0	0	
SAL_PA5885AA	PNUSAS040339	Berta	5	4	6	64	0	0	0	0	0	0	0	
SAL_PA5984AA	PNUSAS040190	Berta	5	4	6	64	0	0	0	0	0	0	0	
SAL_PA6225AA	113003	Berta	5	4	6	64	0	0	0	0	0	0	0	
SAL_PA6317AA	PNUSAS040199	Berta	5	4	6	64	0	0	0	0	0	0	0	
SAL_PA6520AA	PNUSAS040912	Berta	5	4	6	64	0	0	0	0	0	0	0	
SAL_PA7181AA	CFSAN080859	Berta	5	4	6	64	0	0	0	0	0	0	0	
SAL_PA7322AA	CFSAN080961	Berta	5	4	6	64	0	0	0	0	0	0	0	
SAL_PA7158AA	CFSAN080890	Berta	5	4	6	64	0	0	0	0	0	0	0	
SAL_PA7166AA	CFSAN080908	Berta	5	4	6	64	0	0	0	0	0	0	0	
SAL_PA7355AA	CFSAN080960	Berta	5	4	6	64	0	0	0	0	0	0	0	
SAL_PA7343AA	CFSAN080958	Berta	5	4	6	64	0	0	0	0	0	0	0	
SAL_PA7632AA	PNUSAS040915	Berta	5	4	6	64	0	0	0	0	0	0	0	
SAL_PA7708AA	CFSAN080962	Berta	5	4	6	64	0	0	0	0	0	0	0	
SAL_PA9301AA	PNUSAS041574	Berta	5	4	6	64	0	0	0	0	0	0	0	
SAL_PA9138AA	PNUSAS040954	Berta	5	4	6	64	0	0	0	0	0	0	0	
SAL_PA9443AA	CFSAN081709	Berta	5	4	6	64	0	0	0	0	0	0	0	
SAL_PA9440AA	CFSAN081712	Berta	5	4	6	64	0	0	0	0	0	0	0	
SAL_PA9442AA	CFSAN081696	Berta	5	4	6	64	0	0	0	0	0	0	0	
SAL_PA9405AA	CFSAN081764	Berta	5	4	6	64	0	0	0	0	0	0	0	
SAL_PA9406AA	CFSAN081767	Berta	5	4	6	64	0	0	0	0	0	0	0	
SAL_PA9407AA	CFSAN081768	Berta	5	4	6	64	0	0	0	0	0	0	0	
SAL_PA9399AA	CFSAN081766	Berta	5	4	6	64	0	0	0	0	0	0	0	
SAL_QA0182AA	PNUSAS041756	Berta	5	4	6	64	0	0	0	0	0	0	0	
SAL_QA0189AA	PNUSAS042130	Berta	5	4	6	64	0	0	0	0	0	0	0	
SAL_QA0125AA	PNUSAS041537	Berta	5	4	6	64	0	0	0	0	0	0	0	
SAL_PA9416AA	CFSAN081773	Berta	5	4	6	64	0	0	0	0	0	0	0	
SAL_QA0645AA	FSIS21821746	Berta	5	4	143	64	0	0	0	0	0	0	0	
SAL_QA0055AA	PNUSAS040649	Berta	5	4	6	65	0	0	0	0	0	0	0	
SAL_QA1100AA	PNUSAS042457	Berta	5	4	6	64	0	0	0	0	0	0	0	
SAL_QA0965AA	PNUSAS042253	Berta	5	4	6	64	0	0	0	0	0	0	0	
SAL_QA1014AA	PNUSAS042436	Berta	5	4	6	64	0	0	0	0	0	0	0	
SAL_QA2041AA	155700	Berta	5	4	6	64	0	0	0	0	0	0	0	
SAL_QA3002AA	PNUSAS043089	Berta	5	4	6	64	0	0	0	0	0	0	0	
SAL_QA3648AA	PNUSAS042628	Berta	5	4	6	64	0	0	0	0	0	0	0	
SAL_QA3622AA	PNUSAS043194	Berta	5	4	6	64	0	0	0	0	0	0	0	
SAL_QA4172AA	PNUSAS041999	Berta	5	4	6	64	0	0	0	0	0	0	0	
SAL_QA4409AA	PNUSAS040066	Berta	5	4	6	64	0	0	0	0	0	0	0	
SAL_QA5660AA	PNUSAS043400	Berta	5	4	6	64	0	0	0	0	0	0	0	
SAL_QA5739AA	PNUSAS043662	Berta	5	4	6	64	0	0	0	0	0	0	0	
SAL_QA6047AA	PNUSAS043341	Berta	5	4	6	64	0	0	0	0	0	0	0	
SAL_QA6333AA	CFSAN082338	Berta	5	4	6	64	0	0	0	0	0	0	0	
SAL_QA6834AA	FSIS11810747	Berta	5	4	6	64	0	0	0	0	0	0	0	
SAL_QA7084AA	PNUSAS043240	Berta	5	4	6	64	0	0	0	0	0	0	0	
SAL_QA7846AA	218408	Berta	5	4	6	64	0	0	0	0	0	0	0	
SAL_QA9116AA	PNUSAS044606	Berta	5	4	6	64	0	0	0	0	0	0	0	
SAL_QA8898AA	PNUSAS042763	Berta	5	4	6	64	0	0	0	0	0	0	0	
SAL_QA8920AA	PNUSAS044292	Berta	5	4	6	64	0	0	0	0	0	0	0	
SAL_QA8896AA	PNUSAS042765	Berta	5	4	6	64	0	0	0	0	0	0	0	
SAL_RA3000AA	PNUSAS043875	Berta	5	4	6	64	0	0	0	0	0	0	0	

SAL_RA6104AA	CFSAN082973	Berta	5	4	6	64	0	0	0	0	0	0	0
SAL_RA6105AA	CFSAN082972	Berta	5	4	6	64	0	0	0	0	0	0	0
SAL_RA6029AA	PNUSAS045803	Berta	5	4	6	64	0	0	0	0	0	0	0
SAL_RA5923AA	PNUSAS045091	Berta	5	4	6	64	0	0	0	0	0	0	0
SAL_RA5844AA	PNUSAS044297	Berta	5	4	6	64	0	0	0	0	0	0	0
SAL_RA5781AA	CFSAN082954	Berta	5	4	6	64	0	0	0	0	0	0	0
SAL_RA5751AA	CFSAN082941	Berta	5	4	6	64	0	0	0	0	0	0	0
SAL_RA5738AA	CFSAN082945	Berta	5	4	6	64	0	0	0	0	0	0	0
SAL_RA5740AA	CFSAN082953	Berta	5	4	6	64	0	0	0	0	0	0	0
SAL_RA6629AA	327162	Berta	5	4	6	64	0	0	0	0	0	0	0
SAL_RA7555AA	PNUSAS044476	Berta	5	4	6	64	0	0	0	0	0	0	0
SAL_RA7556AA	PNUSAS045048	Berta	5	4	6	64	0	0	0	0	0	0	0
SAL_RA9323AA	CFSAN084124	Berta	5	4	6	64	0	0	0	0	0	0	0
SAL_RA9320AA	CFSAN084079	Berta	5	4	6	64	0	0	0	0	0	0	0
SAL_RA9288AA	CFSAN084155	Berta	5	4	6	64	0	0	0	0	0	0	0
SAL_RA9290AA	CFSAN084116	Berta	5	4	6	64	0	0	0	0	0	0	0
SAL_RA9273AA	CFSAN084115	Berta	5	4	6	64	0	0	0	0	0	0	0
SAL_RA9252AA	CFSAN084080	Berta	5	4	6	64	0	0	0	0	0	0	0
SAL_RA9201AA	CFSAN084020	Berta	5	4	6	64	0	0	0	0	0	0	0
SAL_RA9399AA	CFSAN084035	Berta	5	4	6	64	0	0	0	0	0	0	0
SAL_RA9362AA	CFSAN083983	Berta	5	4	6	64	0	0	0	0	0	0	0
SAL_RA9360AA	CFSAN083984	Berta	5	4	6	64	0	0	0	0	0	0	0
SAL_RA9762AA	CFSAN084200	Berta	5	4	6	64	0	0	0	0	0	0	0
SAL_RA9933AA	FSIS11812491	Berta	5	4	6	64	0	0	0	0	0	0	0
SAL_SA0249AA	CVM N17S823	Berta	5	4	6	64	0	0	0	0	0	0	0
SAL_SA0236AA	CVM N17S125	Berta	5	4	6	64	0	0	0	0	0	0	0
SAL_SA0151AA	CVM N17S334	Berta	5	4	6	64	0	0	0	0	0	0	0
SAL_SA0113AA	CVM N17S265	Berta	5	4	6	64	0	0	0	0	0	0	0
SAL_SA0112AA	CVM N17S266	Berta	5	4	6	64	0	0	0	0	0	0	0
SAL_SA0078AA	CVM N17S1171	Berta	5	4	6	64	0	0	0	0	0	0	0
SAL_SA0001AA	FSIS11812731	Berta	5	4	6	64	0	0	0	0	0	0	0
SAL_SA0981AA	FSIS11812828	Berta	44	4	6	64	0	0	0	0	0	0	0
SAL_SA0983AA	FSIS11812817	Berta	5	4	6	64	0	0	0	0	0	0	0
SAL_SA0901AA	FSIS11812678	Berta	5	4	6	64	0	0	0	0	0	0	0
SAL_SA1099AA	FSIS21821915	Berta	5	4	6	64	0	0	0	0	0	0	0
SAL_SA3308AA	OH-18-18132-2	Berta	5	4	6	64	0	0	0	0	0	0	0
SAL_SA4319AA	FSIS21822100	Berta	5	4	6	64	0	0	0	0	0	0	0
SAL_SA4925AA	579098	Berta	5	4	6	64	0	0	0	0	0	0	0
SAL_SA7961AA	594714	Berta	5	4	6	64	0	0	0	0	0	0	0
SAL_SA9289AA	CVM N17S267	Berta	5	4	6	64	0	0	0	0	0	0	0
SAL_SA9261AA	CVM N17S268	Berta	5	4	6	64	0	0	0	0	0	0	0
SAL_TA6096AA	AL-18-VL-OH-PA-Ag-001	Berta	5	4	6	64	0	0	0	0	0	0	0
SAL_TA6586AA	FSIS21822580	Berta	5	4	6	64	0	0	0	0	0	0	0
SAL_OA1588AA	CFSAN075594	Berta	5	4	6	64	0	0	0	0	0	0	0
SAL_OA5373AA	PNUSAS035683	Berta	5	4	6	65	0	0	0	0	0	0	0
SAL_TA7803AA	FSIS21822689	Berta	5	4	6	64	0	0	0	0	0	0	0
SAL_TA8529AA	FSIS21822728	Berta	44	4	6	64	0	0	0	0	0	0	0
SAL_NA0993AA	PNUSAS029504	Berta	5	4	6	64	0	0	0	0	0	0	0
SAL_KA2353AA	2015AM-1522	Berta	5	4	6	64	0	0	0	0	0	0	0
SAL_OA0827AA	CFSAN075595	Berta	5	4	6	64	0	0	0	0	0	0	0
SAL_OA0925AA	PNUSAS034538	Berta	5	4	6	64	0	0	0	0	0	0	0
SAL_KA0131AA	PNUSAS013796	Berta	5	4	6	64	0	0	0	0	0	0	0
SAL_QA5348AA	377345	Berta	5	4	6	64	0	0	0	0	0	0	0
SAL_OA1231AA	PNUSAS034219	Berta	5	4	6	64	0	0	0	0	0	0	0
SAL_OA2206AA	PNUSAS034210	Berta	5	4	6	65	0	0	0	0	0	0	0
SAL_OA5171AA	PNUSAS035881	Berta	5	4	6	64	0	0	0	0	0	0	0
SAL_OA5205AA	PNUSAS036285	Berta	5	4	6	64	0	0	0	0	0	0	0
SAL_OA5199AA	PNUSAS036288	Berta	5	4	6	64	0	0	0	0	0	0	0
SAL_OA5599AA	PNUSAS035469	Berta	5	4	6	64	0	0	0	0	0	0	0
SAL_PA1191AA	CFSAN077999	Berta	5	4	6	64	0	0	0	0	0	0	0
SAL_PA1238AA	TXAML1800301	Berta	5	4	6	64	0	0	0	0	0	0	0
SAL_PA1350AA	PNUSAS038225	Berta	5	4	6	64	0	0	0	0	0	0	0
SAL_PA1938AA	PNUSAS038732	Berta	5	4	6	64	0	0	0	0	0	0	0
SAL_PA2483AA	CFSAN078645	Berta	5	4	6	64	0	0	0	0	0	0	0
SAL_PA2502AA	FSIS21821452	Berta	5	4	6	64	0	0	0	0	0	0	0
SAL_PA2714AA	PNUSAS038846	Berta	5	4	6	64	0	0	0	0	0	0	0
SAL_PA2710AA	PNUSAS038989	Berta	5	4	6	64	0	0	0	0	0	0	0
SAL_PA2735AA	PNUSAS038843	Berta	5	4	6	64	0	0	0	0	0	0	0
SAL_PA3124AA	30-fff3-11e6-a0c0-3c4a92	Berta	5	4	6	64	0	0	0	0	0	0	0
SAL_PA3129AA	30-fff3-11e6-a0c0-3c4a92	Berta	5	4	6	64	0	0	0	0	0	0	0
SAL_PA3460AA	PNUSAS039268	Berta	5	4	6	64	0	0	0	0	0	0	0
SAL_PA3526AA	CFSAN080005	Berta	5	4	6	64	0	0	0	0	0	0	0
SAL_PA3707AA	PNUSAS039427	Berta	5	4	6	64	0	0	0	0	0	0	0

SAL_PA3823AA	PNUSAS039524	Berta	5	4	6	64	0	0	0	0	0	0	0
SAL_PA4234AA	PNUSAS039434	Berta	5	4	6	64	0	0	0	0	0	0	0
SAL_RA8498AA	SA20141895	Berta	5	4	6	65	0	0	0	0	0	0	0
SAL_UA6826AA	CFSAN089910	Berta	5	4	6	64	0	0	0	0	0	0	0
SAL_UA5716AA	FSIS21823014	Berta	5	4	6	64	0	0	0	0	0	0	0
SAL_UA5920AA	FSIS11816697	Berta	5	4	6	64	0	0	0	0	0	0	0
SAL_UA7800AA	CFSAN090584	Berta	5	4	6	64	0	0	0	0	0	0	0
SAL_UA7801AA	CFSAN090583	Berta	5	4	6	64	0	0	0	0	0	0	0
SAL_UA8064AA	ADRDL-2294	Berta	5	4	6	64	0	0	0	0	0	0	0
SAL_UA8326AA	AL-18-VL-NY-UP-NBC-001	Berta	5	4	6	64	0	0	0	0	0	0	0
SAL_VA6697AA	BCW_4986	Berta	5	4	6	65	0	0	0	0	0	0	0
SAL_VA5867AA	SA20101561	Berta	5	4	6	64	0	0	0	0	0	0	0
SAL_VA4253AA	CDPH_CSalm116	Berta	5	4	6	64	0	0	0	0	0	0	0
SAL_VA4136AA	CDPH_CSalm043	Berta	5	4	6	64	0	0	0	0	0	0	0
SAL_VA4195AA	CDPH_CSalm015	Berta	5	4	6	64	0	0	0	0	0	0	0
SAL_VA9528AA	272518	Berta	5	4	6	64	0	0	0	0	0	0	0
SAL_WA0647AA	FSIS11918329	Berta	5	4	6	64	0	0	0	0	0	0	0
SAL_WA0943AA	140713	Berta	5	4	6	64	0	0	0	0	0	0	0
SAL_WA0871AA	697696	Berta	5	4	6	64	0	0	0	0	0	0	0
SAL_WA1485AA	FSIS11918934	Berta	5	4	6	64	0	0	0	0	0	0	0
SAL_WA3674AA	CFSAN087442	Berta	5	4	6	64	0	0	0	0	0	0	0
SAL_WA3601AA	CFSAN087527	Berta	5	4	6	64	0	0	0	0	0	0	0
SAL_WA3576AA	CFSAN087507	Berta	5	4	6	64	0	0	0	0	0	0	0
SAL_WA3737AA	CFSAN087583	Berta	5	4	6	64	0	0	0	0	0	0	0
SAL_WA3740AA	CFSAN087582	Berta	5	4	6	64	0	0	0	0	0	0	0
SAL_WA3551AA	CFSAN087618	Berta	5	4	6	64	0	0	0	0	0	0	0
SAL_WA3554AA	CFSAN087609	Berta	5	4	6	64	0	0	0	0	0	0	0
SAL_WA3563AA	CFSAN087610	Berta	5	4	6	64	0	0	0	0	0	0	0
SAL_WA3531AA	CFSAN087506	Berta	5	4	6	64	0	0	0	0	0	0	0
SAL_DA4280AA	MOD1_Sal_883	Bietri	0	0	0	0	0	0	0	0	0	0	0
SAL_IA2678AA	CFSAN059209	Bietri	0	0	0	0	0	0	0	0	0	0	0
SAL_NA2358AA	CFSAN073484	Bietri	0	0	0	0	0	0	0	0	0	0	0
SAL_SA3002AA	CFSAN070319	Bietri	0	0	0	0	0	0	0	0	0	0	0
SAL_WA0524AA	195808	Bietri	0	0	0	0	0	0	0	0	0	0	0
SAL_BA9469AA	5835	Bispebjerg	0	0	0	0	0	0	0	0	0	0	0
SAL_EA2868AA	FDA00001850	Bispebjerg	0	0	0	0	0	0	0	0	0	0	0
SAL_EA0872AA	M276 / NCTC20091	Bispebjerg	0	0	0	0	0	0	0	0	0	0	0
SAL_CA5896AA	58496	Bispebjerg	0	0	0	0	0	0	0	0	0	0	0
SAL_CA1270AA	37076	Bispebjerg	0	0	0	0	0	0	0	0	0	0	0
SAL_BA8787AA	5967	Bispebjerg	0	0	0	0	0	0	0	0	0	0	0
SAL_BA4840AA	CFSAN022625	Bispebjerg	0	0	0	0	0	0	0	0	0	0	0
SAL_BA4836AA	CFSAN022626	Bispebjerg	0	0	0	0	0	0	0	0	0	0	0
SAL_GA2080AA	BCW_3060	Bispebjerg	0	0	0	0	0	0	0	0	0	0	0
SAL_IA7801AA	FDA00002413	Bispebjerg	0	0	0	0	0	0	0	0	0	0	0
SAL_LA3183AA	CK_93/00007711	Bispebjerg	0	0	0	0	0	0	0	0	0	0	0
SAL_LA3198AA	CK_00/00009885	Bispebjerg	0	0	0	0	0	0	0	0	0	0	0
SAL_LA3214AA	CK_95/00002717	Bispebjerg	0	0	0	0	0	0	0	0	0	0	0
SAL_LA3222AA	S/790913	Bispebjerg	0	0	0	0	0	0	0	0	0	0	0
SAL_CA3564AA	89895	Bispebjerg	0	0	0	0	0	0	0	0	0	0	0
SAL_BA5832AA	M264 / NCTC20084	Bispebjerg	1	6	10	18	5	2	7	7	8	9	3
SAL_CA1431AA	51298	Bispebjerg	0	0	0	0	0	0	0	0	0	0	0
SAL_EA0843AA	M443 / NCTC20137	Bispebjerg	0	0	0	0	0	0	0	0	0	0	0
SAL_DA8222AA	0296-sc-2010-09-29-985	Bispebjerg	0	0	0	0	0	0	0	0	0	0	0
SAL_EA0209AA	FSF0012	Bispebjerg	0	0	0	0	0	0	0	0	0	0	0
SAL_CA3847AA	27066	Bispebjerg	0	0	0	0	0	0	0	0	0	0	0
SAL_CA6487AA	48216	Bispebjerg	0	0	0	0	0	0	0	0	0	0	0
SAL_FA1077AA	PNUSAS001534	Bispebjerg	0	0	0	0	0	0	0	0	0	0	0
SAL_QA2261AA	299900	Bispebjerg	0	0	0	0	0	0	0	0	0	0	0
SAL_QA8265AA	271089	Bispebjerg	0	0	0	0	0	0	0	0	0	0	0
SAL_SA1987AA	S/20072006	Bispebjerg	0	0	0	0	0	0	0	0	0	0	0
SAL_SA1985AA	8886/98	Bispebjerg	0	0	0	0	0	0	0	0	0	0	0
SAL_SA1984AA	8356/95	Bispebjerg	1	17	10	18	5	2	188	7	8	241	3
SAL_SA1983AA	8261/94	Bispebjerg	1	17	10	18	5	2	188	7	8	241	3
SAL_SA1982AA	8260/94	Bispebjerg	1	17	10	18	5	2	188	7	8	241	3
SAL_SA1981AA	8259/94	Bispebjerg	1	17	10	18	5	2	188	7	8	241	3
SAL_SA1980AA	26K	Bispebjerg	0	0	0	0	0	0	0	0	0	0	0
SAL_SA1979AA	270K	Bispebjerg	1	17	10	404	5	2	7	7	8	9	3
SAL_SA1786AA	3226/81	Bispebjerg	0	0	0	0	0	0	0	0	0	0	0
SAL_SA1785AA	25K	Bispebjerg	0	0	0	0	0	0	0	0	0	0	0
SAL_RA7213AA	238369	Bispebjerg	0	0	0	0	0	0	0	0	0	0	0
SAL_RA8081AA	296243	Bispebjerg	0	0	0	0	0	0	0	0	0	0	0
SAL_UA5303AA	647799	Bispebjerg	0	0	0	0	0	0	0	0	0	0	0
SAL_VA7447AA	201703351	Blancmesnil	0	0	0	0	0	0	0	0	0	0	0

SAL_EA0465AA	FAR0015	Blijdorp	0	0	0	0	0	0	0	0	0	0	0
SAL_FA2399AA	CFSAN031494	Blijdorp	0	0	0	0	0	0	0	0	0	0	0
SAL_EA5894AA	CFSAN031490	Blijdorp	0	0	0	0	0	0	0	0	0	0	0
SAL_EA5891AA	CFSAN031485	Blijdorp	0	0	0	0	0	0	0	0	0	0	0
SAL_EA5879AA	CFSAN031489	Blijdorp	0	0	0	0	0	0	0	0	0	0	0
SAL_EA5422AA	CFSAN031495	Blijdorp	0	0	0	0	0	0	0	0	0	0	0
SAL_EA3983AA	CFSAN031461	Blijdorp	0	0	0	0	0	0	0	0	0	0	0
SAL_GA8735AA	FDA00003439	Blijdorp	0	0	0	0	0	0	0	0	0	0	0
SAL_FA2401AA	CFSAN031493	Blijdorp	0	0	0	0	0	0	0	0	0	0	0
SAL_NA4997AA	01-20-18.8100017	Blijdorp	0	0	0	0	0	0	0	0	0	0	0
SAL_OA2123AA	201555	Blijdorp	0	0	0	0	0	0	0	0	0	0	0
SAL_WA2569AA	CK_01/00000560	Blijdorp	0	0	0	0	0	0	0	0	0	0	0
SAL_WA2426AA	CK_05/00001820	Blijdorp	0	0	0	0	0	0	0	0	0	0	0
SAL_DA6083AA	MOD1_Sal_843	Blockley	0	0	0	0	0	0	0	0	0	0	0
SAL_FA5544AA	PNUSAS001946	Blockley	0	0	0	0	0	0	0	0	0	0	0
SAL_FA4651AA	169637	Blockley	0	0	0	0	0	0	0	0	0	0	0
SAL_FA4603AA	189223	Blockley	0	0	0	0	0	0	0	0	0	0	0
SAL_FA3081AA	CVM N56250	Blockley	0	0	0	0	0	0	0	0	0	0	0
SAL_FA3007AA	CVM N58022	Blockley	0	0	0	0	0	0	0	0	0	0	0
SAL_EA7872AA	18677	Blockley	0	0	0	0	0	0	0	0	0	0	0
SAL_EA7360AA	20922	Blockley	0	0	0	0	0	0	0	0	0	0	0
SAL_EA6918AA	21855	Blockley	0	0	0	0	0	0	0	0	0	0	0
SAL_EA6582AA	FSIS1505168	Blockley	0	0	0	0	0	0	0	0	0	0	0
SAL_EA6342AA	FSIS1505161	Blockley	0	0	0	0	0	0	0	0	0	0	0
SAL_EA2042AA	CVM N53061	Blockley	0	0	0	0	0	0	0	0	0	0	0
SAL_DA8768AA	WAPHL-SAL-A00275	Blockley	0	0	0	0	0	0	0	0	0	0	0
SAL_DA7213AA	CFSAN012642	Blockley	0	0	0	0	0	0	0	0	0	0	0
SAL_DA7056AA	MDH-2014-0005	Blockley	0	0	0	0	0	0	0	0	0	0	0
SAL_DA6661AA	MDH-2014-00277	Blockley	0	0	0	0	0	0	0	0	0	0	0
SAL_DA6435AA	MDH-2014-00291	Blockley	0	0	0	0	0	0	0	0	0	0	0
SAL_DA5429AA	CFSAN022434	Blockley	0	0	0	0	0	0	0	0	0	0	0
SAL_DA2257AA	2012K-0840	Blockley	0	0	0	0	0	0	0	0	0	0	0
SAL_DA0608AA	H123740558	Blockley	0	0	0	0	0	0	0	0	0	0	0
SAL_DA0462AA	H123780513	Blockley	0	0	0	0	0	0	0	0	0	0	0
SAL_DA0139AA	FDA00000723	Blockley	0	0	0	0	0	0	0	0	0	0	0
SAL_CA7902AA	FDA00002411	Blockley	0	0	0	0	0	0	0	0	0	0	0
SAL_CA7457AA	NY-N11231	Blockley	0	0	0	0	0	0	0	0	0	0	0
SAL_CA5733AA	78657	Blockley	0	0	0	0	0	0	0	0	0	0	0
SAL_CA5340AA	73633	Blockley	0	0	0	0	0	0	0	0	0	0	0
SAL_CA5278AA	73626	Blockley	0	0	0	0	0	0	0	0	0	0	0
SAL_CA4626AA	63017	Blockley	0	0	0	0	0	0	0	0	0	0	0
SAL_CA4367AA	26640	Blockley	0	0	0	0	0	0	0	0	0	0	0
SAL_CA4055AA	67531	Blockley	0	0	0	0	0	0	0	0	0	0	0
SAL_CA3260AA	68962	Blockley	0	0	0	0	0	0	0	0	0	0	0
SAL_CA2584AA	31631	Blockley	0	0	0	0	0	0	0	0	0	0	0
SAL_CA2110AA	27807	Blockley	0	0	0	0	0	0	0	0	0	0	0
SAL_BA9447AA	65032	Blockley	0	0	0	0	0	0	0	0	0	0	0
SAL_BA8983AA	90479	Blockley	0	0	0	0	0	0	0	0	0	0	0
SAL_BA8600AA	CFSAN005641	Blockley	0	0	0	0	0	0	0	0	0	0	0
SAL_BA8211AA	9456	Blockley	0	0	0	0	0	0	0	0	0	0	0
SAL_BA6609AA	S-0936	Blockley	0	0	0	0	0	0	0	0	0	0	0
SAL_BA6418AA	FDA00000841	Blockley	0	0	0	0	0	0	0	0	0	0	0
SAL_BA6336AA	NY-N13144	Blockley	0	0	0	0	0	0	0	0	0	0	0
SAL_BA4991AA	FDA00000877	Blockley	0	0	0	0	0	0	0	0	0	0	0
SAL_BA4993AA	FDA00008980	Blockley	0	0	0	0	0	0	0	0	0	0	0
SAL_BA1257AA	CFSAN035088	Blockley	0	0	0	0	0	0	0	0	0	0	0
SAL_BA1240AA	CFSAN035081	Blockley	0	0	0	0	0	0	0	0	0	0	0
SAL_FA6401AA	FDA00010142	Blockley	0	0	0	0	0	0	0	0	0	0	0
SAL_FA6829AA	CVM-N28597	Blockley	0	0	0	0	0	0	0	0	0	0	0
SAL_FA8162AA	PNUSAS002319	Blockley	0	0	0	0	0	0	0	0	0	0	0
SAL_FA7213AA	PNUSAS001976	Blockley	0	0	0	0	0	0	0	0	0	0	0
SAL_GA2766AA	CRJJGF_00147	Blockley	0	0	0	0	0	0	0	0	0	0	0
SAL_GA2669AA	05-0359	Blockley	0	0	0	0	0	0	0	0	0	0	0
SAL_GA2666AA	79-1229	Blockley	0	0	0	0	0	0	0	0	0	0	0
SAL_GA2533AA	67-3024	Blockley	0	0	0	0	0	0	0	0	0	0	0
SAL_GA2338AA	BCW_2646	Blockley	0	0	0	0	0	0	0	0	0	0	0
SAL_GA2232AA	BCW_5988	Blockley	0	0	0	0	0	0	0	0	0	0	0
SAL_GA2219AA	BCW_6132	Blockley	0	0	0	0	0	0	0	0	0	0	0
SAL_GA1677AA	BCW_2880	Blockley	0	0	0	0	0	0	0	0	0	0	0
SAL_GA4715AA	FSIS1607041	Blockley	0	0	0	0	0	0	0	0	0	0	0
SAL_GA5077AA	PNUSAS002931	Blockley	0	0	0	0	0	0	0	0	0	0	0
SAL_GA5075AA	PNUSAS002933	Blockley	0	0	0	0	0	0	0	0	0	0	0
SAL_GA5076AA	PNUSAS002932	Blockley	0	0	0	0	0	0	0	0	0	0	0

SAL_GA5069AA	PNUSAS002939	Blockley	0	0	0	0	0	0	0	0	0	0	0
SAL_GA6045AA	FSIS1607202	Blockley	0	0	0	0	0	0	0	0	0	0	0
SAL_GA8675AA	PNUSAS003258	Blockley	0	0	0	0	0	0	0	0	0	0	0
SAL_GA9193AA	CFSAN031520	Blockley	0	0	0	0	0	0	0	0	0	0	0
SAL_GA9194AA	CFSAN031519	Blockley	0	0	0	0	0	0	0	0	0	0	0
SAL_GA9195AA	CFSAN031518	Blockley	0	0	0	0	0	0	0	0	0	0	0
SAL_GA9191AA	CFSAN031522	Blockley	0	0	0	0	0	0	0	0	0	0	0
SAL_GA9192AA	CFSAN031521	Blockley	0	0	0	0	0	0	0	0	0	0	0
SAL_HA0161AA	PNUSAS004113	Blockley	0	0	0	0	0	0	0	0	0	0	0
SAL_HA1093AA	14085183	Blockley	0	0	0	0	0	0	0	0	0	0	0
SAL_HA3832AA	PNUSAS004878	Blockley	0	0	0	0	0	0	0	0	0	0	0
SAL_HA4216AA	PNUSAS004964	Blockley	0	0	0	0	0	0	0	0	0	0	0
SAL_HA2662AA	FDA00010841	Blockley	0	0	0	0	0	0	0	0	0	0	0
SAL_HA5697AA	PNUSAS005590	Blockley	0	0	0	0	0	0	0	0	0	0	0
SAL_HA7616AA	CFSAN056830	Blockley	0	0	0	0	0	0	0	0	0	0	0
SAL_IA1532AA	285853	Blockley	0	0	0	0	0	0	0	0	0	0	0
SAL_IA1928AA	PNUSAS007295	Blockley	0	0	0	0	0	0	0	0	0	0	0
SAL_IA3047AA	140242	Blockley	0	0	0	0	0	0	0	0	0	0	0
SAL_IA6213AA	CFSAN059138	Blockley	0	0	0	0	0	0	0	0	0	0	0
SAL_IA8317AA	PNUSAS008932	Blockley	0	0	0	0	0	0	0	0	0	0	0
SAL_IA9520AA	PNUSAS009259	Blockley	0	0	0	0	0	0	0	0	0	0	0
SAL_JA1710AA	PNUSAS011684	Blockley	0	0	0	0	0	0	0	0	0	0	0
SAL_JA3715AA	PNUSAS012990	Blockley	0	0	0	0	0	0	0	0	0	0	0
SAL_JA6090AA	FSIS1700891	Blockley	0	0	0	0	0	0	0	0	0	0	0
SAL_JA8288AA	352148	Blockley	0	0	0	0	0	0	0	0	0	0	0
SAL_KA0810AA	PNUSAS016057	Blockley	0	0	0	0	0	0	0	0	0	0	0
SAL_KA3265AA	PNUSAS017818	Blockley	0	0	0	0	0	0	0	0	0	0	0
SAL_KA4299AA	ZJ-65	Blockley	0	0	0	0	0	0	0	0	0	0	0
SAL_KA4238AA	ZJ-76C	Blockley	0	0	0	0	0	0	0	0	0	0	0
SAL_KA4292AA	ZJ-51C	Blockley	0	0	0	0	0	0	0	0	0	0	0
SAL_KA4980AA	PNUSAS016366	Blockley	0	0	0	0	0	0	0	0	0	0	0
SAL_KA5204AA	PNUSAS018370	Blockley	0	0	0	0	0	0	0	0	0	0	0
SAL_KA5920AA	PNUSAS019081	Blockley	0	0	0	0	0	0	0	0	0	0	0
SAL_KA7041AA	PNUSAS018841	Blockley	0	0	0	0	0	0	0	0	0	0	0
SAL_KA6952AA	PNUSAS019227	Blockley	0	0	0	0	0	0	0	0	0	0	0
SAL_KA6856AA	PNUSAS017511	Blockley	0	0	0	0	0	0	0	0	0	0	0
SAL_KA8049AA	PNUSAS019792	Blockley	0	0	0	0	0	0	0	0	0	0	0
SAL_KA8029AA	PNUSAS019799	Blockley	0	0	0	0	0	0	0	0	0	0	0
SAL_KA7993AA	FSIS1703488	Blockley	0	0	0	0	0	0	0	0	0	0	0
SAL_KA8152AA	2015AM-1551	Blockley	0	0	0	0	0	0	0	0	0	0	0
SAL_KA8422AA	2015AM-1056	Blockley	0	0	0	0	0	0	0	0	0	0	0
SAL_KA8905AA	PNUSAS019911	Blockley	0	0	0	0	0	0	0	0	0	0	0
SAL_KA9071AA	PNUSAS020745	Blockley	0	0	0	0	0	0	0	0	0	0	0
SAL_KA8976AA	2015AM-0316	Blockley	0	0	0	0	0	0	0	0	0	0	0
SAL_LA0039AA	FSIS21720006	Blockley	0	0	0	0	0	0	0	0	0	0	0
SAL_LA4181AA	PNUSAS022632	Blockley	0	0	0	0	0	0	0	0	0	0	0
SAL_LA4415AA	PNUSAS022490	Blockley	0	0	0	0	0	0	0	0	0	0	0
SAL_LA4920AA	PNUSAS022824	Blockley	0	0	0	0	0	0	0	0	0	0	0
SAL_LA6816AA	PNUSAS023172	Blockley	0	0	0	0	0	0	0	0	0	0	0
SAL_LA6609AA	PNUSAS022216	Blockley	0	0	0	0	0	0	0	0	0	0	0
SAL_LA6569AA	PNUSAS022511	Blockley	0	0	0	0	0	0	0	0	0	0	0
SAL_LA7487AA	PNUSAS024279	Blockley	0	0	0	0	0	0	0	0	0	0	0
SAL_LA9798AA	PNUSAS022852	Blockley	0	0	0	0	0	0	0	0	0	0	0
SAL_MA4708AA	PNUSAS026215	Blockley	0	0	0	0	0	0	0	0	0	0	0
SAL_MA4919AA	PNUSAS025273	Blockley	0	0	0	0	0	0	0	0	0	0	0
SAL_MA5164AA	PNUSAS026976	Blockley	0	0	0	0	0	0	0	0	0	0	0
SAL_CA4965AA	73615	Blockley	0	0	0	0	0	0	0	0	0	0	0
SAL_DA1137AA	H124040535	Blockley	0	0	0	0	0	0	0	0	0	0	0
SAL_GA2526AA	86-0206	Blockley	0	0	0	0	0	0	0	0	0	0	0
SAL_IA9680AA	PNUSAS008900	Blockley	0	0	0	0	0	0	0	0	0	0	0
SAL_MA7285AA	PNUSAS024989	Blockley	0	0	0	0	0	0	0	0	0	0	0
SAL_MA8440AA	PNUSAS029060	Blockley	0	0	0	0	0	0	0	0	0	0	0
SAL_MA9215AA	ricia-Salmonella-blockley	Blockley	0	0	0	0	0	0	0	0	0	0	0
SAL_MA9884AA	CVM N16S256	Blockley	0	0	0	0	0	0	0	0	0	0	0
SAL_NA1198AA	PNUSAS029496	Blockley	0	0	0	0	0	0	0	0	0	0	0
SAL_NA1102AA	PNUSAS027936	Blockley	0	0	0	0	0	0	0	0	0	0	0
SAL_NA1947AA	PNUSAS029959	Blockley	0	0	0	0	0	0	0	0	0	0	0
SAL_NA2066AA	PNUSAS029366	Blockley	0	0	0	0	0	0	0	0	0	0	0
SAL_NA3628AA	PNUSAS030430	Blockley	0	0	0	0	0	0	0	0	0	0	0
SAL_NA6002AA	2015AM-2431	Blockley	0	0	0	0	0	0	0	0	0	0	0
SAL_NA5835AA	2015AM-2750	Blockley	0	0	0	0	0	0	0	0	0	0	0
SAL_NA6883AA	PNUSAS031773	Blockley	0	0	0	0	0	0	0	0	0	0	0
SAL_NA8148AA	2015AM-3654	Blockley	0	0	0	0	0	0	0	0	0	0	0

SAL_NA8993AA	PNUSAS033226	Blockley	0	0	0	0	0	0	0	0	0	0	0
SAL_NA9269AA	PNUSAS033125	Blockley	0	0	0	0	0	0	0	0	0	0	0
SAL_OA0577AA	FSIS21821107	Blockley	0	0	0	0	0	0	0	0	0	0	0
SAL_OA1718AA	159838_2	Blockley	0	0	0	0	0	0	0	0	0	0	0
SAL_OA1730AA	163013_2	Blockley	0	0	0	0	0	0	0	0	0	0	0
SAL_OA1567AA	159838	Blockley	0	0	0	0	0	0	0	0	0	0	0
SAL_OA1580AA	163013	Blockley	0	0	0	0	0	0	0	0	0	0	0
SAL_OA5865AA	PNUSAS035076	Blockley	0	0	0	0	0	0	0	0	0	0	0
SAL_OA7817AA	PNUSAS025428	Blockley	0	0	0	0	0	0	0	0	0	0	0
SAL_OA4849AA	PNUSAS036228	Blockley	0	0	0	0	0	0	0	0	0	0	0
SAL_OA4704AA	PNUSAS025290	Blockley	0	0	0	0	0	0	0	0	0	0	0
SAL_OA4677AA	PNUSAS025414	Blockley	0	0	0	0	0	0	0	0	0	0	0
SAL_OA4679AA	PNUSAS025492	Blockley	0	0	0	0	0	0	0	0	0	0	0
SAL_PA4638AA	PNUSAS038963	Blockley	0	0	0	0	0	0	0	0	0	0	0
SAL_PA6014AA	PNUSAS039926	Blockley	0	0	0	0	0	0	0	0	0	0	0
SAL_PA6261AA	FSIS21821620	Blockley	0	0	0	0	0	0	0	0	0	0	0
SAL_PA6980AA	PNUSAS041013	Blockley	0	0	0	0	0	0	0	0	0	0	0
SAL_PA7981AA	178943	Blockley	0	0	0	0	0	0	0	0	0	0	0
SAL_PA8258AA	PNUSAS041046	Blockley	0	0	0	0	0	0	0	0	0	0	0
SAL_PA8260AA	PNUSAS040861	Blockley	0	0	0	0	0	0	0	0	0	0	0
SAL_PA8136AA	PNUSAS041199	Blockley	0	0	0	0	0	0	0	0	0	0	0
SAL_PA8913AA	319661	Blockley	0	0	0	0	0	0	0	0	0	0	0
SAL_PA9676AA	264120	Blockley	0	0	0	0	0	0	0	0	0	0	0
SAL_QA0113AA	PNUSAS041253	Blockley	0	0	0	0	0	0	0	0	0	0	0
SAL_QA0644AA	FSIS21821735	Blockley	0	0	0	0	0	0	0	0	0	0	0
SAL_QA0005AA	FSIS31800484	Blockley	0	0	0	0	0	0	0	0	0	0	0
SAL_QA1110AA	PNUSAS042456	Blockley	0	0	0	0	0	0	0	0	0	0	0
SAL_QA2451AA	123874	Blockley	0	0	0	0	0	0	0	0	0	0	0
SAL_QA4296AA	PNUSAS039516	Blockley	0	0	0	0	0	0	0	0	0	0	0
SAL_QA4801AA	318347	Blockley	0	0	0	0	0	0	0	0	0	0	0
SAL_QA5426AA	428152	Blockley	0	0	0	0	0	0	0	0	0	0	0
SAL_QA5126AA	152458	Blockley	0	0	0	0	0	0	0	0	0	0	0
SAL_QA6192AA	413075	Blockley	0	0	0	0	0	0	0	0	0	0	0
SAL_QA6560AA	306681	Blockley	0	0	0	0	0	0	0	0	0	0	0
SAL_QA6936AA	FSIS11810655	Blockley	0	0	0	0	0	0	0	0	0	0	0
SAL_RA0477AA	SMG-18-834	Blockley	0	0	0	0	0	0	0	0	0	0	0
SAL_QA7034AA	PNUSAS043523	Blockley	0	0	0	0	0	0	0	0	0	0	0
SAL_QA7430AA	275794	Blockley	0	0	0	0	0	0	0	0	0	0	0
SAL_QA7310AA	119471	Blockley	0	0	0	0	0	0	0	0	0	0	0
SAL_QA8767AA	365343	Blockley	0	0	0	0	0	0	0	0	0	0	0
SAL_QA8962AA	PNUSAS044583	Blockley	0	0	0	0	0	0	0	0	0	0	0
SAL_QA8943AA	PNUSAS044767	Blockley	0	0	0	0	0	0	0	0	0	0	0
SAL_QA9307AA	279955	Blockley	0	0	0	0	0	0	0	0	0	0	0
SAL_QA9960AA	316332	Blockley	0	0	0	0	0	0	0	0	0	0	0
SAL_RA0412AA	184410	Blockley	0	0	0	0	0	0	0	0	0	0	0
SAL_RA1101AA	157363	Blockley	0	0	0	0	0	0	0	0	0	0	0
SAL_RA1425AA	PNUSAS044773	Blockley	0	0	0	0	0	0	0	0	0	0	0
SAL_RA2715AA	534821	Blockley	0	0	0	0	0	0	0	0	0	0	0
SAL_RA2890AA	163162	Blockley	0	0	0	0	0	0	0	0	0	0	0
SAL_RA3023AA	PNUSAS045089	Blockley	0	0	0	0	0	0	0	0	0	0	0
SAL_RA3406AA	298812	Blockley	0	0	0	0	0	0	0	0	0	0	0
SAL_RA4116AA	298984	Blockley	0	0	0	0	0	0	0	0	0	0	0
SAL_RA6413AA	PNUSAS045331	Blockley	0	0	0	0	0	0	0	0	0	0	0
SAL_RA6255AA	PNUSAS045611	Blockley	0	0	0	0	0	0	0	0	0	0	0
SAL_RA6791AA	PNUSAS045971	Blockley	0	0	0	0	0	0	0	0	0	0	0
SAL_RA7473AA	568272	Blockley	0	0	0	0	0	0	0	0	0	0	0
SAL_RA7788AA	FSIS11811742	Blockley	0	0	0	0	0	0	0	0	0	0	0
SAL_RA8597AA	NC_WHO_S102	Blockley	0	0	0	0	0	0	0	0	0	0	0
SAL_RA8704AA	FSIS11812200	Blockley	0	0	0	0	0	0	0	0	0	0	0
SAL_RA6749AA	PNUSAS043683	Blockley	0	0	0	0	0	0	0	0	0	0	0
SAL_SA3995AA	70234	Blockley	0	0	0	0	0	0	0	0	0	0	0
SAL_SA5302AA	CK_99/00002859	Blockley	0	0	0	0	0	0	0	0	0	0	0
SAL_SA5300AA	CK_99/00001242	Blockley	0	0	0	0	0	0	0	0	0	0	0
SAL_SA5301AA	CK_99/00001287	Blockley	0	0	0	0	0	0	0	0	0	0	0
SAL_SA5267AA	CK_93/00012507	Blockley	0	0	0	0	0	0	0	0	0	0	0
SAL_SA4687AA	579883	Blockley	0	0	0	0	0	0	0	0	0	0	0
SAL_SA7150AA	266935	Blockley	0	0	0	0	0	0	0	0	0	0	0
SAL_SA7832AA	587821	Blockley	0	0	0	0	0	0	0	0	0	0	0
SAL_TA0839AA	615620	Blockley	0	0	0	0	0	0	0	0	0	0	0
SAL_TA1822AA	112209.12	Blockley	0	0	0	0	0	0	0	0	0	0	0
SAL_TA5233AA	611431	Blockley	0	0	0	0	0	0	0	0	0	0	0
SAL_TA8549AA	FSIS21822714	Blockley	0	0	0	0	0	0	0	0	0	0	0
SAL_QA9935AA	240154	Blockley	0	0	0	0	0	0	0	0	0	0	0

SAL_PA1354AA	PNUSAS038339	Blockley	0	0	0	0	0	0	0	0	0	0	0
SAL_PA2498AA	FSIS21821451	Blockley	0	0	0	0	0	0	0	0	0	0	0
SAL_PA3661AA	FSIS31800356	Blockley	0	0	0	0	0	0	0	0	0	0	0
SAL_UA4750AA	651080	Blockley	0	0	0	0	0	0	0	0	0	0	0
SAL_UA6990AA	659345	Blockley	0	0	0	0	0	0	0	0	0	0	0
SAL_UA7291AA	18EP002554	Blockley	0	0	0	0	0	0	0	0	0	0	0
SAL_UA9494AA	19EP000120	Blockley	0	0	0	0	0	0	0	0	0	0	0
SAL_VA0098AA	628802	Blockley	0	0	0	0	0	0	0	0	0	0	0
SAL_VA4270AA	CDPH_CSalm237	Blockley	0	0	0	0	0	0	0	0	0	0	0
SAL_VA6708AA	FSIS21923500	Blockley	0	0	0	0	0	0	0	0	0	0	0
SAL_VA6024AA	NWH-10	Blockley	0	0	0	0	0	0	0	0	0	0	0
SAL_VA5930AA	SA20030330	Blockley	0	0	0	0	0	0	0	0	0	0	0
SAL_VA5860AA	SA20092116	Blockley	0	0	0	0	0	0	0	0	0	0	0
SAL_VA5684AA	SA20112999	Blockley	0	0	0	0	0	0	0	0	0	0	0
SAL_VA4584AA	CCK-DO-012	Blockley	0	0	0	0	0	0	0	0	0	0	0
SAL_VA4585AA	RCK-D-038	Blockley	0	0	0	0	0	0	0	0	0	0	0
SAL_VA4383AA	393926	Blockley	0	0	0	0	0	0	0	0	0	0	0
SAL_VA8345AA	FSIS31901601	Blockley	0	0	0	0	0	0	0	0	0	0	0
SAL_VA8747AA	314929	Blockley	0	0	0	0	0	0	0	0	0	0	0
SAL_VA9122AA	318345	Blockley	0	0	0	0	0	0	0	0	0	0	0
SAL_VA9525AA	285589	Blockley	0	0	0	0	0	0	0	0	0	0	0
SAL_VA4155AA	CDPH_CSalm035	Blockley	0	0	0	0	0	0	0	0	0	0	0
SAL_WA0486AA	185444	Blockley	0	0	0	0	0	0	0	0	0	0	0
SAL_WA1066AA	FSIS11918568	Blockley	0	0	0	0	0	0	0	0	0	0	0
SAL_SA6140AA	572310	Bokanjac	0	0	0	0	0	0	0	0	0	0	0
SAL_CA8016AA	FDA00000785	Bonariensis	0	0	0	0	0	0	0	0	0	0	0
SAL_DA1516AA	H132840478	Bonariensis	0	0	0	0	0	0	0	0	0	0	0
SAL_CA5481AA	78675	Bonariensis	0	0	0	0	0	0	0	0	0	0	0
SAL_GA5048AA	PNUSAS002905	Bonariensis	0	0	0	0	0	0	0	0	0	0	0
SAL_GA6427AA	PNUSAS002736	Bonariensis	0	0	0	0	0	0	0	0	0	0	0
SAL_GA6573AA	PNUSAS003169	Bonariensis	0	0	0	0	0	0	0	0	0	0	0
SAL_JA1081AA	PNUSAS007052	Bonariensis	0	0	0	0	0	0	0	0	0	0	0
SAL_JA7644AA	PNUSAS011937	Bonariensis	0	0	0	0	0	0	0	0	0	0	0
SAL_KA6834AA	PNUSAS017531	Bonariensis	0	0	0	0	0	0	0	0	0	0	0
SAL_LA7415AA	PNUSAS023254	Bonariensis	0	0	0	0	0	0	0	0	0	0	0
SAL_LA7371AA	PNUSAS013725	Bonariensis	0	0	0	0	0	0	0	0	0	0	0
SAL_CA3531AA	83585	Bonariensis	0	0	0	0	0	0	0	0	0	0	0
SAL_BA6450AA	797205	Bonariensis	0	0	0	0	0	0	0	0	0	0	0
SAL_CA3922AA	67545	Bonariensis	0	0	0	0	0	0	0	0	0	0	0
SAL_DA1503AA	H124640554	Bonariensis	0	0	0	0	0	0	0	0	0	0	0
SAL_EA8022AA	18678	Bonariensis	0	0	0	0	0	0	0	0	0	0	0
SAL_FA6515AA	PNUSAS001990	Bonariensis	0	0	0	0	0	0	0	0	0	0	0
SAL_GA3352AA	BCW_2647	Bonariensis	0	0	0	0	0	0	0	0	0	0	0
SAL_FA7349AA	PNUSAS002149	Bonariensis	0	0	0	0	0	0	0	0	0	0	0
SAL_FA6050AA	PNUSAS001743	Bonariensis	0	0	0	0	0	0	0	0	0	0	0
SAL_KA6342AA	PNUSAS019219	Bonariensis	0	0	0	0	0	0	0	0	0	0	0
SAL_LA4548AA	PNUSAS022543	Bonariensis	0	0	0	0	0	0	0	0	0	0	0
SAL_MA8286AA	PNUSAS011838	Bonariensis	0	0	0	0	0	0	0	0	0	0	0
SAL_OA0532AA	FDA00012544	Bonariensis	0	0	0	0	0	0	0	0	0	0	0
SAL_QA0674AA	107792	Bonariensis	0	0	0	0	0	0	0	0	0	0	0
SAL_QA2329AA	313300	Bonariensis	0	0	0	0	0	0	0	0	0	0	0
SAL_QA7249AA	182852	Bonariensis	0	0	0	0	0	0	0	0	0	0	0
SAL_QA8310AA	526150	Bonariensis	0	0	0	0	0	0	0	0	0	0	0
SAL_RA6493AA	286734	Bonariensis	0	0	0	0	0	0	0	0	0	0	0
SAL_SA5884AA	591950	Bonariensis	0	0	0	0	0	0	0	0	0	0	0
SAL_SA7741AA	285533	Bonariensis	0	0	0	0	0	0	0	0	0	0	0
SAL_SA7956AA	592578	Bonariensis	0	0	0	0	0	0	0	0	0	0	0
SAL_TA6723AA	627387	Bonariensis	0	0	0	0	0	0	0	0	0	0	0
SAL_OA1666AA	PNUSAS034110	Bonariensis	0	0	0	0	0	0	0	0	0	0	0
SAL_OA2315AA	PNUSAS033491	Bonariensis	0	0	0	0	0	0	0	0	0	0	0
SAL_PA3392AA	PNUSAS039168	Bonariensis	0	0	0	0	0	0	0	0	0	0	0
SAL_VA4438AA	387502	Bonariensis	0	0	0	0	0	0	0	0	0	0	0
SAL_VA9682AA	252421	Bonariensis	0	0	0	0	0	0	0	0	0	0	0
SAL_BA5875AA	M130 / NCTC20056	Bovismorbificans	12	15	40	62	15	0	40	4	5	5	1
SAL_BA7477AA	93515	Bovismorbificans	12	0	39	62	15	0	3	4	5	78	1
SAL_BA9932AA	:-2012-07-27T11:52:57Z-	Bovismorbificans	0	0	0	0	0	0	0	0	0	0	0
SAL_DA1975AA	FDA00002020	Bovismorbificans	0	0	0	0	0	0	0	0	0	0	0
SAL_DA7842AA	FAR0129	Bovismorbificans	0	0	0	0	0	0	0	0	0	0	0
SAL_CA4297AA	9130	Bovismorbificans	0	0	0	0	0	0	0	0	0	0	0
SAL_DA9146AA	FNE0078	Bovismorbificans	0	0	0	0	0	0	0	0	0	0	0
SAL_EA5883AA	FSIS1504461	Bovismorbificans	0	0	0	0	0	0	0	0	0	0	0
SAL_FA5989AA	CFSAN045336	Bovismorbificans	0	0	0	0	0	0	0	0	0	0	0
SAL_FA5988AA	CFSAN045337	Bovismorbificans	0	0	0	0	0	0	0	0	0	0	0

SAL_FA5781AA	MS150106	Bovismorbificans	0	0	0	0	0	0	0	0	0	0	0
SAL_FA5665AA	MS150003	Bovismorbificans	0	0	0	0	0	0	0	0	0	0	0
SAL_FA4662AA	157294	Bovismorbificans	12	0	39	62	15	0	35	4	62	5	1
SAL_FA4640AA	203653	Bovismorbificans	12	0	39	62	15	0	3	4	5	78	1
SAL_FA4631AA	152428	Bovismorbificans	12	0	39	62	15	0	35	4	62	5	1
SAL_FA4455AA	193400	Bovismorbificans	0	0	0	0	0	0	0	0	0	0	0
SAL_FA4183AA	136769	Bovismorbificans	12	0	39	62	15	0	35	4	62	5	1
SAL_FA4095AA	171331	Bovismorbificans	12	0	39	62	15	0	35	4	62	5	1
SAL_FA4072AA	164706	Bovismorbificans	0	0	0	0	0	0	0	0	0	0	0
SAL_FA3251AA	FSIS1606031	Bovismorbificans	0	0	0	0	0	0	0	0	0	0	0
SAL_FA2505AA	FDA00000081	Bovismorbificans	0	0	0	0	0	0	0	0	0	0	0
SAL_FA1928AA	PNUSAS001663	Bovismorbificans	0	0	0	0	0	0	0	0	0	0	0
SAL_EA9365AA	FDA00004972	Bovismorbificans	12	0	39	62	15	0	3	4	5	78	1
SAL_EA9021AA	FDA00004974	Bovismorbificans	12	0	39	62	15	0	0	4	0	78	0
SAL_EA7837AA	6469	Bovismorbificans	0	0	0	0	0	0	0	0	0	0	0
SAL_EA7676AA	91963	Bovismorbificans	0	0	0	0	0	0	0	0	0	0	0
SAL_EA7526AA	29954	Bovismorbificans	12	0	39	62	15	0	35	4	62	5	1
SAL_EA7539AA	77325	Bovismorbificans	0	0	0	0	0	0	0	0	0	0	0
SAL_EA7480AA	18511	Bovismorbificans	0	0	0	0	0	0	0	0	0	0	0
SAL_EA2860AA	FDA00001843	Bovismorbificans	0	0	0	0	0	0	0	0	0	0	0
SAL_EA2703AA	FDA00000441	Bovismorbificans	0	0	0	0	0	0	0	0	0	0	0
SAL_EA2709AA	FDA00000400	Bovismorbificans	0	0	0	0	0	0	0	0	0	0	0
SAL_DA8533AA	FSE0001	Bovismorbificans	0	0	0	0	0	0	0	0	0	0	0
SAL_DA7613AA	D23769	Bovismorbificans	12	0	39	62	15	0	35	4	62	5	1
SAL_DA6247AA	FSE0123	Bovismorbificans	0	0	0	0	0	0	0	0	0	0	0
SAL_DA6254AA	FSE0120	Bovismorbificans	0	0	0	0	0	0	0	0	0	0	0
SAL_DA4500AA	276608	Bovismorbificans	0	0	0	0	0	0	0	0	0	0	0
SAL_DA4498AA	A5893	Bovismorbificans	12	0	39	0	15	0	35	4	62	5	1
SAL_DA4499AA	A8737	Bovismorbificans	12	0	39	0	15	0	35	4	62	5	1
SAL_DA4501AA	3114	Bovismorbificans	12	0	39	62	15	0	35	4	62	5	1
SAL_DA4497AA	A31126	Bovismorbificans	12	0	39	62	15	0	35	4	62	5	1
SAL_DA4492AA	653308	Bovismorbificans	0	0	0	0	0	0	0	0	0	0	0
SAL_DA4490AA	499208	Bovismorbificans	0	0	0	0	0	0	0	0	0	0	0
SAL_DA4051AA	VA-WGS-00264	Bovismorbificans	0	0	0	0	0	0	0	0	0	0	0
SAL_DA3084AA	CFSAN024184	Bovismorbificans	0	0	0	0	0	0	0	0	0	0	0
SAL_DA1250AA	H124440681	Bovismorbificans	0	0	0	0	0	0	0	0	0	0	0
SAL_DA1089AA	H120340709	Bovismorbificans	0	0	0	0	0	0	0	0	0	0	0
SAL_DA1008AA	H131320438	Bovismorbificans	12	0	39	62	15	0	35	4	62	5	1
SAL_DA0794AA	H124440648	Bovismorbificans	0	0	0	0	0	0	0	0	0	0	0
SAL_DA0791AA	H124760774	Bovismorbificans	0	0	0	0	0	0	0	0	0	0	0
SAL_DA0364AA	FDA00003264	Bovismorbificans	0	0	0	0	0	0	0	0	0	0	0
SAL_DA0355AA	FDA00003263	Bovismorbificans	0	0	0	0	0	0	0	0	0	0	0
SAL_DA0320AA	FDA00003266	Bovismorbificans	0	0	0	0	0	0	0	0	0	0	0
SAL_DA0321AA	FDA00003265	Bovismorbificans	0	0	0	0	0	0	0	0	0	0	0
SAL_CA9360AA	CFSAN027134	Bovismorbificans	0	0	0	0	0	0	0	0	0	0	0
SAL_CA9359AA	CFSAN027135	Bovismorbificans	0	0	0	0	0	0	0	0	0	0	0
SAL_CA9349AA	IEH-NGS-SAL-01659	Bovismorbificans	0	0	0	0	0	0	0	0	0	0	0
SAL_CA8451AA	FDA00000889	Bovismorbificans	0	0	0	0	0	0	0	0	0	0	0
SAL_CA6226AA	53982	Bovismorbificans	12	0	39	62	15	0	35	4	62	5	1
SAL_CA6139AA	48204	Bovismorbificans	0	0	0	0	0	0	0	0	0	0	0
SAL_CA5911AA	64989	Bovismorbificans	0	0	0	0	0	0	0	0	0	0	0
SAL_CA5729AA	78709	Bovismorbificans	12	0	39	0	15	0	35	4	62	5	1
SAL_CA5127AA	78626	Bovismorbificans	0	0	0	0	0	0	0	0	0	0	0
SAL_CA4583AA	34147	Bovismorbificans	0	0	0	0	0	0	0	0	0	0	0
SAL_CA4515AA	37764	Bovismorbificans	0	0	0	0	0	0	0	0	0	0	0
SAL_CA4219AA	72332	Bovismorbificans	0	0	0	0	0	0	0	0	0	79	0
SAL_CA4147AA	46558	Bovismorbificans	0	0	0	0	0	0	0	0	0	0	0
SAL_CA3980AA	40305	Bovismorbificans	12	0	39	62	15	0	3	4	5	78	1
SAL_CA3698AA	65113	Bovismorbificans	0	0	0	0	0	0	0	0	0	0	0
SAL_CA3406AA	65036	Bovismorbificans	0	0	0	0	0	0	0	0	0	0	0
SAL_CA3139AA	32380	Bovismorbificans	12	0	39	62	15	0	35	4	62	5	1
SAL_CA3122AA	25202	Bovismorbificans	0	0	0	0	0	0	0	0	0	0	0
SAL_CA3096AA	67543	Bovismorbificans	0	0	0	0	0	0	0	0	0	0	0
SAL_CA2883AA	75968	Bovismorbificans	0	0	0	0	0	0	0	0	0	0	0
SAL_CA2541AA	93541	Bovismorbificans	0	0	0	0	0	0	0	0	0	0	0
SAL_CA2330AA	93539	Bovismorbificans	0	0	0	0	0	0	0	0	0	0	0
SAL_CA1936AA	67542	Bovismorbificans	0	0	0	0	0	0	0	0	0	0	0
SAL_CA1850AA	94244	Bovismorbificans	0	0	0	0	0	0	0	0	0	0	0
SAL_CA1695AA	42157	Bovismorbificans	0	0	0	0	0	0	0	0	0	0	0
SAL_CA1087AA	23458	Bovismorbificans	12	0	39	62	15	0	35	4	62	5	1
SAL_CA0001AA	73205	Bovismorbificans	0	0	0	0	0	0	0	0	0	0	0
SAL_BA8926AA	42134	Bovismorbificans	0	0	0	0	0	0	0	0	0	0	0
SAL_BA8748AA	24993	Bovismorbificans	0	0	0	0	0	0	0	0	0	0	0

SAL_BA8708AA	45578	Bovismorbificans	12	0	39	62	15	0	35	4	62	5	1
SAL_BA8135AA	6459	Bovismorbificans	0	0	0	0	0	0	0	0	0	0	0
SAL_BA7545AA	99773	Bovismorbificans	0	0	0	0	0	0	0	0	0	0	0
SAL_BA3585AA	CFSAN029984	Bovismorbificans	0	0	0	0	0	0	0	0	0	0	0
SAL_BA3525AA	CFSAN029985	Bovismorbificans	0	0	0	0	0	0	0	0	0	0	0
SAL_BA3520AA	CFSAN029987	Bovismorbificans	0	0	0	0	0	0	0	0	0	0	0
SAL_BA1424AA	FDA00002010	Bovismorbificans	0	0	0	0	0	0	0	0	0	0	0
SAL_BA0987AA	130496	Bovismorbificans	0	0	0	0	0	0	0	0	0	0	0
SAL_BA0989AA	140700	Bovismorbificans	12	0	39	62	15	0	35	4	62	5	1
SAL_BA0991AA	135052	Bovismorbificans	12	0	39	62	15	0	35	4	62	5	1
SAL_BA0986AA	101306	Bovismorbificans	0	0	0	0	0	0	0	0	0	0	0
SAL_BA0983AA	130392	Bovismorbificans	12	0	39	62	15	0	35	4	62	5	1
SAL_BA0988AA	140683	Bovismorbificans	12	0	39	62	15	0	35	4	62	5	1
SAL_BA0985AA	135059	Bovismorbificans	12	0	39	62	15	0	35	4	62	5	1
SAL_BA0990AA	142150	Bovismorbificans	12	0	39	62	15	0	35	4	62	5	1
SAL_BA0982AA	136555	Bovismorbificans	12	0	39	62	15	0	35	4	62	5	1
SAL_BA0981AA	135060	Bovismorbificans	12	0	39	62	15	0	35	4	62	5	1
SAL_BA0984AA	140197	Bovismorbificans	12	0	39	62	15	0	35	4	62	5	1
SAL_BA0980AA	136569	Bovismorbificans	12	0	39	62	15	0	35	4	62	5	1
SAL_BA0979AA	136556	Bovismorbificans	12	0	39	62	15	0	35	4	62	5	1
SAL_BA0978AA	137160	Bovismorbificans	12	0	39	62	15	0	35	4	62	5	1
SAL_BA0977AA	56968	Bovismorbificans	12	0	39	62	15	0	35	4	62	5	1
SAL_BA0976AA	136554	Bovismorbificans	12	0	39	62	15	0	35	4	62	5	1
SAL_BA0975AA	103617	Bovismorbificans	0	0	0	0	0	0	0	0	0	0	0
SAL_BA0974AA	136549	Bovismorbificans	12	0	39	62	15	0	35	4	62	5	1
SAL_FA7519AA	FDA00001663	Bovismorbificans	0	0	0	0	0	0	0	0	0	0	0
SAL_FA7741AA	PNUSAS002345	Bovismorbificans	0	0	0	0	0	0	0	0	0	0	0
SAL_FA7905AA	FSIS1606764	Bovismorbificans	0	0	0	0	0	0	0	0	0	0	0
SAL_FA8064AA	PNUSAS002156	Bovismorbificans	12	0	39	62	15	0	3	4	5	78	1
SAL_FA8141AA	FSIS1606883	Bovismorbificans	0	0	0	0	0	0	0	0	0	0	0
SAL_FA8223AA	ARIM-SBVM1410-05	Bovismorbificans	12	0	39	62	15	0	3	4	5	78	1
SAL_GA2841AA	CRJJGF_00070	Bovismorbificans	0	0	0	0	0	0	0	0	0	0	0
SAL_GA2703AA	96-0578	Bovismorbificans	0	0	0	0	0	0	0	0	0	0	0
SAL_GA2700AA	02-0459	Bovismorbificans	0	0	0	0	0	0	0	0	0	0	0
SAL_GA2687AA	91-0332	Bovismorbificans	0	0	0	0	0	0	0	0	0	0	0
SAL_GA3889AA	BCW_3304	Bovismorbificans	0	0	0	0	0	0	0	0	0	0	0
SAL_GA3888AA	BCW_3305	Bovismorbificans	0	0	0	0	0	0	0	0	0	0	0
SAL_GA3886AA	BCW_3307	Bovismorbificans	0	0	0	0	0	0	0	0	0	0	0
SAL_GA3885AA	BCW_3308	Bovismorbificans	0	0	0	0	0	0	0	0	0	0	0
SAL_GA3652AA	BCW_3292	Bovismorbificans	12	0	39	62	15	0	3	4	5	78	1
SAL_GA3651AA	BCW_3294	Bovismorbificans	12	0	39	62	15	0	3	4	5	78	1
SAL_GA3650AA	BCW_3295	Bovismorbificans	12	0	39	62	15	0	3	4	5	78	1
SAL_GA3648AA	BCW_3297	Bovismorbificans	12	0	39	62	15	0	3	4	5	78	1
SAL_GA3514AA	BCW_2845	Bovismorbificans	12	15	40	62	39	0	40	4	5	5	1
SAL_GA3351AA	BCW_2648	Bovismorbificans	12	0	39	62	15	0	3	4	5	78	1
SAL_GA2064AA	BCW_3293	Bovismorbificans	12	0	39	62	15	0	3	4	5	78	1
SAL_GA2063AA	BCW_3298	Bovismorbificans	12	0	39	62	15	0	3	4	5	78	1
SAL_GA2062AA	BCW_3299	Bovismorbificans	12	0	39	62	15	0	3	4	5	78	1
SAL_GA2061AA	BCW_3300	Bovismorbificans	12	0	39	62	15	0	3	4	5	78	1
SAL_GA2059AA	BCW_3303	Bovismorbificans	0	0	0	0	0	0	0	0	0	0	0
SAL_GA2060AA	BCW_3302	Bovismorbificans	12	0	39	62	15	0	3	4	5	78	1
SAL_GA2058AA	BCW_3310	Bovismorbificans	0	0	0	0	0	0	0	0	0	0	0
SAL_GA2057AA	BCW_3311	Bovismorbificans	0	0	0	0	0	0	0	0	0	0	0
SAL_GA2035AA	BCW_4232	Bovismorbificans	12	0	39	62	15	0	3	4	5	78	1
SAL_GA1759AA	N30673	Bovismorbificans	0	0	0	0	0	0	0	0	0	0	0
SAL_GA1672AA	BCW_3309	Bovismorbificans	12	0	39	62	15	0	3	4	5	78	1
SAL_GA3887AA	BCW_3306	Bovismorbificans	0	0	0	0	0	0	0	0	0	0	0
SAL_GA3649AA	BCW_3296	Bovismorbificans	12	0	39	62	15	0	3	4	5	78	1
SAL_GA0345AA	NS015PDT_D9-sc-228062	Bovismorbificans	0	0	0	0	0	0	0	0	0	0	0
SAL_GA0291AA	72_G_033-sc-2280728	Bovismorbificans	0	0	0	0	0	0	0	0	0	0	0
SAL_GA4808AA	PNUSAS002782	Bovismorbificans	0	0	0	0	0	0	0	0	0	0	0
SAL_GA5080AA	PNUSAS002647	Bovismorbificans	0	0	0	0	0	0	0	0	0	0	0
SAL_FA8731AA	CVM N30673	Bovismorbificans	0	0	0	0	0	0	0	0	0	0	0
SAL_GA3647AA	BCW_3301	Bovismorbificans	12	0	39	62	15	0	3	4	5	78	1
SAL_GA5934AA	FSIS1607106	Bovismorbificans	0	0	0	0	0	0	0	0	0	0	0
SAL_GA7186AA	CFSAN025353	Bovismorbificans	0	0	0	0	0	0	0	0	0	0	0
SAL_GA7381AA	FDA00001443	Bovismorbificans	0	0	0	0	0	0	0	0	0	0	0
SAL_GA7514AA	PNUSAS003337	Bovismorbificans	0	0	0	0	0	0	0	0	0	0	0
SAL_GA7759AA	FSIS1607385	Bovismorbificans	0	0	0	0	0	0	0	0	0	0	0
SAL_GA8577AA	PNUSAS003488	Bovismorbificans	0	0	0	0	0	0	0	0	0	0	0
SAL_GA8580AA	PNUSAS003360	Bovismorbificans	0	0	0	0	0	0	0	0	0	0	0
SAL_GA8642AA	VA-WGS-16188	Bovismorbificans	0	0	0	0	0	0	0	0	0	0	0
SAL_GA9412AA	PNUSAS003705	Bovismorbificans	0	0	0	0	0	0	0	0	0	0	0

SAL_HA1592AA	PNUSAS004784	Bovismorbificans	12	15	40	62	15	0	40	4	180	5	1
SAL_HA1572AA	PNUSAS004824	Bovismorbificans	0	0	0	0	0	0	0	0	0	0	0
SAL_HA2791AA	ADRDL-16-12667	Bovismorbificans	0	0	0	0	0	0	0	0	0	0	0
SAL_HA2260AA	S04543-15	Bovismorbificans	0	0	0	0	0	0	0	0	0	0	0
SAL_HA2201AA	S05462-15	Bovismorbificans	0	0	0	0	0	0	0	0	0	0	0
SAL_HA5111AA	L01541-14	Bovismorbificans	0	0	0	0	0	0	0	0	0	0	0
SAL_HA3394AA	wd3	Bovismorbificans	0	0	0	0	0	0	0	0	0	0	0
SAL_HA2386AA	L00019-15	Bovismorbificans	0	0	0	0	0	0	0	0	0	0	0
SAL_HA5590AA	S00960-16	Bovismorbificans	12	0	39	62	15	0	3	4	5	78	1
SAL_HA5600AA	S04795-14	Bovismorbificans	0	0	0	0	0	0	0	0	0	0	0
SAL_HA6937AA	FSIS1608903	Bovismorbificans	0	0	0	0	0	0	0	0	0	0	0
SAL_HA6334AA	FSIS1608953	Bovismorbificans	0	0	0	0	0	0	0	0	0	0	0
SAL_HA7767AA	FSIS1608887	Bovismorbificans	0	0	0	0	0	0	0	0	0	0	0
SAL_HA8186AA	S/20161444	Bovismorbificans	0	0	0	0	0	0	0	0	0	0	0
SAL_HA8247AA	PNUSAS006475	Bovismorbificans	0	0	0	0	0	0	0	0	0	0	0
SAL_HA9332AA	PNUSAS006876	Bovismorbificans	0	0	0	0	0	0	0	0	0	0	0
SAL_IA2223AA	MS160085	Bovismorbificans	0	0	0	0	0	0	0	0	0	0	0
SAL_IA1118AA	330435	Bovismorbificans	0	0	0	0	0	0	0	0	0	0	0
SAL_IA2331AA	FSIS1709846	Bovismorbificans	0	0	0	0	0	0	0	0	0	0	0
SAL_IA3592AA	323099	Bovismorbificans	0	0	0	0	0	0	0	0	0	0	0
SAL_IA3773AA	PNUSAS007462	Bovismorbificans	0	0	0	0	0	0	0	0	0	0	0
SAL_IA3764AA	PNUSAS007571	Bovismorbificans	0	0	0	0	0	0	0	0	0	0	0
SAL_IA5893AA	MS160420	Bovismorbificans	0	0	0	0	0	0	0	0	0	0	0
SAL_IA6973AA	MS140073	Bovismorbificans	0	0	0	0	0	0	0	0	0	0	0
SAL_IA6708AA	MS150317	Bovismorbificans	0	0	0	0	0	0	0	0	0	0	0
SAL_IA7267AA	FDA00011280	Bovismorbificans	0	0	0	0	0	0	0	0	0	0	0
SAL_IA7268AA	FDA00011279	Bovismorbificans	0	0	0	0	0	0	0	0	0	0	0
SAL_IA7715AA	S/20161444-20/02	Bovismorbificans	0	0	0	0	0	0	0	0	0	0	0
SAL_JA0023AA	FSIS1710917	Bovismorbificans	0	0	0	0	0	0	0	0	0	0	0
SAL_JA0058AA	Q7-8-20170120	Bovismorbificans	0	0	0	0	0	0	0	0	0	0	0
SAL_JA0066AA	S/20170120	Bovismorbificans	0	0	0	0	0	0	0	0	0	0	0
SAL_JA0092AA	PNUSAS010403	Bovismorbificans	0	0	0	0	0	0	0	0	0	0	0
SAL_JA1381AA	229836	Bovismorbificans	0	0	0	0	0	0	0	0	0	0	0
SAL_JA1380AA	286721	Bovismorbificans	0	0	0	0	0	0	0	0	0	0	0
SAL_JA1384AA	136768	Bovismorbificans	12	0	39	62	15	0	35	4	62	5	1
SAL_JA1382AA	333393	Bovismorbificans	0	0	0	0	0	0	0	0	0	0	0
SAL_JA1306AA	290690	Bovismorbificans	0	0	0	0	0	0	0	0	0	0	0
SAL_JA1307AA	333944	Bovismorbificans	0	0	0	0	0	0	0	0	0	0	0
SAL_JA1304AA	223590	Bovismorbificans	0	0	0	0	0	0	0	0	0	0	0
SAL_JA1305AA	329841	Bovismorbificans	0	0	0	0	0	0	0	0	0	0	0
SAL_JA1303AA	142472	Bovismorbificans	0	0	0	0	0	0	0	0	0	0	0
SAL_JA1406AA	329849	Bovismorbificans	0	0	0	0	0	0	0	0	0	0	0
SAL_JA1410AA	160802	Bovismorbificans	0	0	0	0	0	0	0	0	0	0	0
SAL_JA1343AA	311410	Bovismorbificans	0	0	0	0	0	0	0	0	0	0	0
SAL_JA1345AA	318295	Bovismorbificans	0	0	0	0	0	0	0	0	0	0	0
SAL_JA1344AA	316657	Bovismorbificans	0	0	0	0	0	0	0	0	0	0	0
SAL_JA1349AA	138325	Bovismorbificans	0	0	0	0	0	0	0	0	0	0	0
SAL_JA1359AA	280299	Bovismorbificans	0	0	0	0	0	0	0	0	0	0	0
SAL_JA1353AA	340169	Bovismorbificans	0	0	0	0	0	0	0	0	0	0	0
SAL_JA1357AA	315667	Bovismorbificans	0	0	0	0	0	0	0	0	0	0	0
SAL_JA1356AA	315001	Bovismorbificans	0	0	0	0	0	0	0	0	0	0	0
SAL_JA1355AA	260215	Bovismorbificans	0	0	0	0	0	0	0	0	0	0	0
SAL_JA1352AA	140659	Bovismorbificans	12	0	39	62	15	0	35	4	62	5	1
SAL_JA1354AA	329864	Bovismorbificans	0	0	0	0	0	0	0	0	0	0	0
SAL_JA1361AA	218462	Bovismorbificans	0	0	0	0	0	0	0	0	0	0	0
SAL_JA1373AA	273631	Bovismorbificans	0	0	0	0	0	0	0	0	0	0	0
SAL_JA1371AA	248640	Bovismorbificans	0	0	0	0	0	0	0	0	0	0	0
SAL_JA1372AA	140597	Bovismorbificans	12	0	39	62	15	0	35	4	62	5	1
SAL_JA1376AA	333410	Bovismorbificans	0	0	0	0	0	0	0	0	0	0	0
SAL_JA1375AA	335574	Bovismorbificans	0	0	0	0	0	0	0	0	0	0	0
SAL_JA1387AA	299838	Bovismorbificans	12	0	39	62	15	0	35	4	62	5	1
SAL_JA1377AA	218476	Bovismorbificans	0	0	0	0	0	0	0	0	0	0	0
SAL_JA1378AA	335504	Bovismorbificans	0	0	0	0	0	0	0	0	0	0	0
SAL_JA1379AA	261335	Bovismorbificans	0	0	0	0	0	0	0	0	0	0	0
SAL_JA1392AA	341270	Bovismorbificans	0	0	0	0	0	0	0	0	0	0	0
SAL_JA1394AA	143643	Bovismorbificans	0	0	0	0	0	0	0	0	0	0	0
SAL_JA1396AA	208759	Bovismorbificans	0	0	0	0	0	0	0	0	0	0	0
SAL_JA1398AA	329850	Bovismorbificans	0	0	0	0	0	0	0	0	0	0	0
SAL_JA1397AA	149227	Bovismorbificans	0	0	0	0	0	0	0	0	0	0	0
SAL_JA1400AA	299004	Bovismorbificans	12	0	39	62	15	0	3	4	5	78	1
SAL_JA1346AA	337042	Bovismorbificans	0	0	0	0	0	0	0	0	0	0	0
SAL_JA1320AA	178938	Bovismorbificans	0	0	0	0	0	0	0	0	0	0	0
SAL_JA1314AA	257390	Bovismorbificans	0	0	0	0	0	0	0	0	0	0	0

SAL_JA1317AA	128210	Bovismorbificans	0	0	0	0	0	0	0	0	0	0	0
SAL_JA1338AA	185979	Bovismorbificans	0	0	0	0	0	0	0	0	0	0	0
SAL_JA1340AA	245083	Bovismorbificans	0	0	0	0	0	0	0	0	0	0	0
SAL_JA1341AA	278199	Bovismorbificans	0	0	0	0	0	0	0	0	0	0	0
SAL_JA1339AA	308486	Bovismorbificans	0	0	0	0	0	0	0	0	0	0	0
SAL_JA1278AA	310289	Bovismorbificans	0	0	0	0	0	0	0	0	0	0	0
SAL_JA1280AA	330514	Bovismorbificans	0	0	0	0	0	0	0	0	0	0	0
SAL_JA1282AA	249342	Bovismorbificans	0	0	0	0	0	0	0	0	0	0	0
SAL_JA1281AA	212782	Bovismorbificans	0	0	0	0	0	0	0	0	0	0	0
SAL_JA1283AA	315173	Bovismorbificans	0	0	0	0	0	0	0	0	0	0	0
SAL_JA1285AA	140602	Bovismorbificans	12	0	39	62	15	0	35	4	62	5	1
SAL_JA1292AA	267026	Bovismorbificans	12	0	39	62	15	0	3	4	5	78	1
SAL_JA1290AA	305220	Bovismorbificans	12	0	39	62	15	0	35	4	62	5	1
SAL_JA1297AA	337073	Bovismorbificans	0	0	0	0	0	0	0	0	0	0	0
SAL_JA1284AA	322510	Bovismorbificans	0	0	0	0	0	0	0	0	0	0	0
SAL_JA1294AA	330508	Bovismorbificans	0	0	0	0	0	0	0	0	0	0	0
SAL_JA1289AA	282592	Bovismorbificans	0	0	0	0	0	0	0	0	0	0	0
SAL_JA1291AA	330453	Bovismorbificans	0	0	0	0	0	0	0	0	0	0	0
SAL_JA1296AA	208768	Bovismorbificans	0	0	0	0	0	0	0	0	0	0	0
SAL_JA1293AA	316686	Bovismorbificans	0	0	0	0	0	0	0	0	0	0	0
SAL_JA1286AA	275793	Bovismorbificans	0	0	0	0	0	0	0	0	0	0	0
SAL_JA1295AA	330528	Bovismorbificans	0	0	0	0	0	0	0	0	0	0	0
SAL_JA1301AA	173715	Bovismorbificans	0	0	0	0	0	0	0	0	0	0	0
SAL_JA1298AA	143652	Bovismorbificans	0	0	0	0	0	0	0	0	0	0	0
SAL_JA1300AA	138330	Bovismorbificans	12	0	39	62	15	0	35	4	62	5	1
SAL_JA1313AA	138363	Bovismorbificans	12	0	39	62	15	0	35	4	62	5	1
SAL_JA1299AA	232714	Bovismorbificans	0	0	0	0	0	0	0	0	0	0	0
SAL_JA1315AA	208947	Bovismorbificans	0	0	0	0	0	0	0	0	0	0	0
SAL_JA1316AA	329133	Bovismorbificans	0	0	0	0	0	0	0	0	0	0	0
SAL_JA1321AA	330455	Bovismorbificans	0	0	0	0	0	0	0	0	0	0	0
SAL_JA1318AA	329869	Bovismorbificans	0	0	0	0	0	0	0	0	0	0	0
SAL_JA1310AA	159653	Bovismorbificans	0	0	0	0	0	0	0	0	0	0	0
SAL_JA1302AA	242376	Bovismorbificans	0	0	0	0	0	0	0	0	0	0	0
SAL_JA1288AA	353836	Bovismorbificans	0	0	0	0	0	0	0	0	0	0	0
SAL_JA1309AA	330535	Bovismorbificans	0	0	0	0	0	0	0	0	0	0	0
SAL_JA1287AA	271166	Bovismorbificans	0	0	0	0	0	0	0	0	0	0	0
SAL_JA1844AA	8-18-20170157	Bovismorbificans	0	0	0	0	0	0	0	0	0	0	0
SAL_JA1199AA	PNUSAS011070	Bovismorbificans	0	0	0	0	0	0	0	0	0	0	0
SAL_JA1128AA	PNUSAS010351	Bovismorbificans	0	0	0	0	0	0	0	0	0	0	0
SAL_JA1162AA	ADRDL-909	Bovismorbificans	0	0	0	0	0	0	0	0	0	0	0
SAL_JA1034AA	PNUSAS010989	Bovismorbificans	0	0	0	0	0	0	0	0	0	0	0
SAL_JA0529AA	PNUSAS009192	Bovismorbificans	0	0	0	0	0	0	0	0	0	0	0
SAL_JA2566AA	PNUSAS011658	Bovismorbificans	0	0	0	0	0	0	0	0	0	0	0
SAL_JA2572AA	PNUSAS011660	Bovismorbificans	0	0	0	0	0	0	0	0	0	0	0
SAL_JA2579AA	PNUSAS011757	Bovismorbificans	0	0	0	0	0	0	0	0	0	0	0
SAL_JA3968AA	PNUSAS012799	Bovismorbificans	0	0	0	0	0	0	0	0	0	0	0
SAL_JA3818AA	PNUSAS011130	Bovismorbificans	0	0	0	0	0	0	0	0	0	0	0
SAL_JA4591AA	PNUSAS013064	Bovismorbificans	0	0	0	0	0	0	0	0	0	0	0
SAL_JA4562AA	PNUSAS010259	Bovismorbificans	0	0	0	0	0	0	0	0	0	0	0
SAL_JA4955AA	PNUSAS012998	Bovismorbificans	0	0	0	0	0	0	0	0	0	0	0
SAL_JA6146AA	PNUSAS011156	Bovismorbificans	0	0	0	0	0	0	0	0	0	0	0
SAL_JA6021AA	PNUSAS013102	Bovismorbificans	0	0	0	0	0	0	0	0	0	0	0
SAL_JA6878AA	342948	Bovismorbificans	12	0	39	62	15	0	35	4	62	5	1
SAL_JA7192AA	10-5-20170334	Bovismorbificans	0	0	0	0	0	0	0	0	0	0	0
SAL_JA7768AA	PNUSAS014680	Bovismorbificans	0	0	0	0	0	0	0	0	0	0	0
SAL_KA0855AA	PNUSAS016293	Bovismorbificans	0	0	0	0	0	0	0	0	0	0	0
SAL_KA2285AA	14-16-20170519	Bovismorbificans	0	0	0	0	0	0	0	0	0	0	0
SAL_KA2795AA	PNUSAS017383	Bovismorbificans	12	15	40	62	15	0	40	4	180	5	1
SAL_KA3067AA	FSIS1702349	Bovismorbificans	0	0	0	0	0	0	0	0	0	0	0
SAL_KA3334AA	PNUSAS016649	Bovismorbificans	0	0	0	0	0	0	0	0	0	0	0
SAL_KA3596AA	PNUSAS015735	Bovismorbificans	0	0	0	0	0	0	0	0	0	0	0
SAL_KA4663AA	PNUSAS017592	Bovismorbificans	0	0	0	0	0	0	0	0	0	0	0
SAL_KA4075AA	H103540740	Bovismorbificans	0	0	0	0	0	0	0	0	0	0	0
SAL_KA5083AA	16-30-20170481	Bovismorbificans	12	0	39	62	15	0	3	4	5	78	1
SAL_KA5087AA	16-32-20170483	Bovismorbificans	12	0	39	62	15	0	3	4	5	78	1
SAL_KA5290AA	PNUSAS017353	Bovismorbificans	12	15	40	62	15	0	40	4	180	5	1
SAL_KA5218AA	PNUSAS018170	Bovismorbificans	0	0	0	0	0	0	0	0	0	0	0
SAL_KA4960AA	PNUSAS017843	Bovismorbificans	0	0	0	0	0	0	0	0	0	0	0
SAL_KA6106AA	CFSAN065567	Bovismorbificans	0	0	0	0	0	0	0	0	0	0	0
SAL_KA7180AA	17-27-20170635	Bovismorbificans	0	0	0	0	0	0	0	0	0	0	0
SAL_KA6624AA	PNUSAS019726	Bovismorbificans	0	0	0	0	0	0	0	0	0	0	0
SAL_KA7554AA	PNUSAS019603	Bovismorbificans	0	0	0	0	0	0	0	0	0	0	0
SAL_KA8933AA	SbovisNL	Bovismorbificans	0	0	0	0	0	0	0	0	0	0	0

SAL_KA8612AA	PNUSAS020504	Bovismorbificans	0	0	0	0	0	0	0	0	0	0	0
SAL_KA8578AA	PNUSAS019499	Bovismorbificans	0	0	0	0	0	0	0	0	0	0	0
SAL_KA8963AA	2015AM-1119	Bovismorbificans	0	0	0	0	0	0	0	0	0	0	0
SAL_KA9094AA	PNUSAS019883	Bovismorbificans	0	0	0	0	0	0	0	0	0	0	0
SAL_KA8604AA	PNUSAS020357	Bovismorbificans	0	0	0	0	0	0	0	0	0	0	0
SAL_LA0114AA	19-17-20170695	Bovismorbificans	12	0	39	62	15	0	35	4	62	5	1
SAL_LA0122AA	19-8-20170620	Bovismorbificans	12	0	39	62	15	0	35	4	62	5	1
SAL_LA0131AA	19-6-20170482	Bovismorbificans	12	0	39	62	15	0	3	4	5	78	1
SAL_KA9569AA	PNUSAS020044	Bovismorbificans	0	0	0	0	0	0	0	0	0	0	0
SAL_KA9371AA	PNUSAS018314	Bovismorbificans	0	0	0	0	0	0	0	0	0	0	0
SAL_LA0005AA	FSIS1703547	Bovismorbificans	0	0	0	0	0	0	0	0	0	0	0
SAL_KA9287AA	PNUSAS020570	Bovismorbificans	12	15	40	62	15	0	40	4	5	5	1
SAL_KA9880AA	PNUSAS020548	Bovismorbificans	12	15	40	62	15	0	40	4	5	5	1
SAL_LA3851AA	PNUSAS021254	Bovismorbificans	0	0	0	0	0	0	0	0	0	0	0
SAL_LA4517AA	PNUSAS022962	Bovismorbificans	0	0	0	0	0	0	0	0	0	0	0
SAL_LA5609AA	20-3-20170776	Bovismorbificans	0	0	0	0	0	0	0	0	0	0	0
SAL_LA5610AA	20-2-20170775	Bovismorbificans	0	0	0	0	0	0	0	0	0	0	0
SAL_LA8123AA	23-31-GMI17-0001	Bovismorbificans	0	0	0	0	0	0	0	0	0	0	0
SAL_LA9944AA	24-16-20170991	Bovismorbificans	0	0	0	0	0	0	0	0	0	0	0
SAL_LA9001AA	FSIS11704643	Bovismorbificans	0	0	0	0	0	0	0	0	0	0	0
SAL_MA0279AA	PNUSAS024838	Bovismorbificans	12	0	39	62	15	0	3	4	5	78	1
SAL_MA2245AA	FSIS21720389	Bovismorbificans	0	0	0	0	0	0	0	0	0	0	0
SAL_MA2812AA	S17BD06274	Bovismorbificans	0	0	0	0	0	0	0	0	0	0	0
SAL_MA2814AA	S17BD06276	Bovismorbificans	0	0	0	0	0	0	0	0	0	0	0
SAL_MA3038AA	FDA00000864	Bovismorbificans	0	0	0	0	0	0	0	0	0	0	0
SAL_MA3054AA	FDA00001728	Bovismorbificans	0	0	0	0	0	0	0	0	0	0	0
SAL_MA3045AA	FDA00000963	Bovismorbificans	0	0	0	0	0	0	0	0	0	0	0
SAL_MA3046AA	FDA00001726	Bovismorbificans	0	0	0	0	0	0	0	0	0	0	0
SAL_MA2938AA	PNUSAS024680	Bovismorbificans	0	0	0	0	0	0	0	0	0	0	0
SAL_MA2936AA	PNUSAS024677	Bovismorbificans	0	0	0	0	0	0	0	0	0	0	0
SAL_MA3263AA	PNUSAS026445	Bovismorbificans	0	0	0	0	0	0	0	0	0	0	0
SAL_MA3871AA	FSIS11705121	Bovismorbificans	0	0	0	0	0	0	0	0	0	0	0
SAL_MA4467AA	PNUSAS026682	Bovismorbificans	0	0	0	0	0	0	0	0	0	0	0
SAL_MA4852AA	PNUSAS026474	Bovismorbificans	0	0	0	0	0	0	0	0	0	0	0
SAL_DA6667AA	FMA0176	Bovismorbificans	0	0	0	0	0	0	0	0	0	0	0
SAL_BA5790AA	M123 / NCTC20052	Bovismorbificans	12	0	39	62	15	0	3	4	5	78	1
SAL_DA3865AA	FDA00003911	Bovismorbificans	0	0	0	0	0	0	0	0	0	0	0
SAL_CA4584AA	27007	Bovismorbificans	0	0	0	0	0	0	0	0	0	0	0
SAL_BA1920AA	FDA00001031	Bovismorbificans	0	0	0	0	0	0	0	0	0	0	0
SAL_DA2245AA	2013K-0459	Bovismorbificans	0	0	0	0	0	0	0	0	0	0	0
SAL_DA0383AA	H125040595	Bovismorbificans	0	0	0	0	0	0	0	0	0	0	0
SAL_BA4683AA	CFSAN006232	Bovismorbificans	0	0	0	0	0	0	0	0	0	0	0
SAL_CA8363AA	FDA00003839	Bovismorbificans	0	0	0	0	0	0	0	0	0	0	0
SAL_DA4493AA	A16982	Bovismorbificans	12	0	39	62	15	0	35	4	62	5	1
SAL_CA2529AA	65439	Bovismorbificans	0	0	0	0	0	0	0	0	0	0	0
SAL_GA9190AA	CFSAN031523	Bovismorbificans	0	0	0	0	0	0	0	0	0	0	0
SAL_HA5344AA	FDA00010963	Bovismorbificans	0	0	0	0	0	0	0	0	0	0	0
SAL_GA9413AA	PNUSAS003704	Bovismorbificans	0	0	0	0	0	0	0	0	0	0	0
SAL_HA5112AA	L01540-14	Bovismorbificans	0	0	0	0	0	0	0	0	0	0	0
SAL_HA9688AA	PNUSAS006896	Bovismorbificans	0	0	0	0	0	0	0	0	0	0	0
SAL_HA8955AA	3114	Bovismorbificans	12	0	39	62	15	0	35	4	62	5	1
SAL_GA2714AA	06-0615	Bovismorbificans	0	0	0	0	0	0	0	0	0	0	0
SAL_HA2387AA	L00018-15	Bovismorbificans	0	0	0	0	0	0	0	0	0	0	0
SAL_GA5084AA	PNUSAS002653	Bovismorbificans	0	0	0	0	0	0	0	0	0	0	0
SAL_GA0361AA	VNS20240-sc-2280587	Bovismorbificans	0	0	0	0	0	0	0	0	0	0	0
SAL_GA2056AA	BCW_3312	Bovismorbificans	0	0	0	0	0	0	0	0	0	0	0
SAL_FA8222AA	ARIM-SBVM730-07	Bovismorbificans	12	0	39	62	15	0	0	0	5	78	1
SAL_FA6119AA	PNUSAS002048	Bovismorbificans	0	0	0	0	0	0	0	0	0	0	0
SAL_FA4612AA	172396	Bovismorbificans	12	0	39	62	15	0	35	4	62	5	1
SAL_FA1326AA	FSIS1605762	Bovismorbificans	0	0	0	0	0	0	0	0	0	0	0
SAL_EA7014AA	97269	Bovismorbificans	0	0	0	0	0	0	0	0	0	0	0
SAL_IA8876AA	PNUSAS008672	Bovismorbificans	0	0	0	0	0	0	0	0	0	0	0
SAL_JA1342AA	299804	Bovismorbificans	0	0	0	0	0	0	0	0	0	0	0
SAL_JA1348AA	154841	Bovismorbificans	0	0	0	0	0	0	0	0	0	0	0
SAL_JA0578AA	PNUSAS010620	Bovismorbificans	0	0	0	0	0	0	0	0	0	0	0
SAL_JA2394AA	PNUSAS011777	Bovismorbificans	12	15	40	62	15	0	40	4	5	5	1
SAL_JA5698AA	PNUSAS013377	Bovismorbificans	12	0	39	62	15	0	3	4	5	78	1
SAL_JA7014AA	PNUSAS011925	Bovismorbificans	0	0	0	0	0	0	0	0	0	0	0
SAL_KA1186AA	2015AM-1552	Bovismorbificans	0	0	0	0	0	0	0	0	0	0	0
SAL_KA4685AA	PNUSAS017715	Bovismorbificans	0	0	0	0	0	0	0	0	0	0	0
SAL_LA7174AA	2008748698	Bovismorbificans	0	0	0	0	0	0	0	0	0	0	0
SAL_MA5545AA	PNUSAS026852	Bovismorbificans	12	0	39	62	15	0	3	4	5	78	1
SAL_MA6250AA	PNUSAS027208	Bovismorbificans	0	0	0	0	0	0	0	0	0	0	0

SAL_MA6206AA	PNUSAS027827	Bovismorbificans	0	0	0	0	0	0	0	0	0	0	0
SAL_MA6535AA	FSIS11705701	Bovismorbificans	0	0	0	0	0	0	0	0	0	0	0
SAL_MA6713AA	29-13-17.8101226	Bovismorbificans	12	0	39	62	15	0	35	4	62	5	1
SAL_MA7141AA	PNUSAS025586	Bovismorbificans	12	0	39	62	15	0	3	4	5	78	1
SAL_MA7613AA	PNUSAS027981	Bovismorbificans	0	0	0	0	0	0	0	0	0	0	0
SAL_MA8434AA	PNUSAS026601	Bovismorbificans	0	0	0	0	0	0	0	0	0	0	0
SAL_MA8788AA	FSIS11705043	Bovismorbificans	0	0	0	0	0	0	0	0	0	0	0
SAL_NA0390AA	FSIS21720703	Bovismorbificans	0	0	0	0	0	0	0	0	0	0	0
SAL_NA0301AA	PNUSAS028213	Bovismorbificans	0	0	0	0	0	0	0	0	0	0	0
SAL_NA1009AA	PNUSAS029318	Bovismorbificans	0	0	0	0	0	0	0	0	0	0	0
SAL_LA3353AA	MS170240	Bovismorbificans	0	0	0	0	0	0	0	0	0	0	0
SAL_NA1285AA	PNUSAS028628	Bovismorbificans	0	0	0	0	0	0	0	0	0	0	0
SAL_NA1707AA	PNUSAS030046	Bovismorbificans	0	0	0	0	0	0	0	0	0	0	0
SAL_NA2196AA	0709_12	Bovismorbificans	0	0	0	0	0	0	0	0	0	0	0
SAL_NA3115AA	PNUSAS023137	Bovismorbificans	0	0	0	0	0	0	0	0	0	0	0
SAL_NA3310AA	FSIS21720805	Bovismorbificans	0	0	0	0	0	0	0	0	0	0	0
SAL_NA4224AA	PNUSAS031026	Bovismorbificans	0	0	0	0	0	0	0	0	0	0	0
SAL_NA4119AA	PNUSAS031094	Bovismorbificans	0	0	0	0	0	0	0	0	0	0	0
SAL_NA4131AA	FSIS11706627	Bovismorbificans	0	0	0	0	0	0	0	0	0	0	0
SAL_NA5616AA	2015AM-2269	Bovismorbificans	0	0	0	0	0	0	0	0	0	0	0
SAL_NA6052AA	2015AM-2526	Bovismorbificans	0	0	0	0	0	0	0	0	0	0	0
SAL_NA6071AA	2015AM-2568	Bovismorbificans	12	15	40	62	15	0	40	4	5	5	1
SAL_NA5913AA	PNUSAS030981	Bovismorbificans	0	0	0	0	0	0	0	0	0	0	0
SAL_NA8258AA	S11	Bovismorbificans	12	0	39	62	15	0	3	4	5	78	1
SAL_NA8314AA	4-16-8100088	Bovismorbificans	12	0	39	62	15	0	3	4	5	78	1
SAL_NA8315AA	4-15-8100087	Bovismorbificans	12	0	39	62	15	0	3	4	5	78	1
SAL_NA8405AA	NPHL 170792	Bovismorbificans	0	0	0	0	0	0	0	0	0	0	0
SAL_NA9500AA	FSIS11807371	Bovismorbificans	0	0	0	0	0	0	0	0	0	0	0
SAL_NA9696AA	CFSAN075262	Bovismorbificans	0	0	0	0	0	0	0	0	0	0	0
SAL_NA9729AA	6-8-8100099	Bovismorbificans	12	0	39	62	15	0	3	4	5	78	1
SAL_NA9964AA	FSIS11807571	Bovismorbificans	0	0	0	0	0	0	0	0	0	0	0
SAL_NA9799AA	PNUSAS011180	Bovismorbificans	0	0	0	0	0	0	0	0	0	0	0
SAL_OA0269AA	PNUSAS034046	Bovismorbificans	0	0	0	0	0	0	0	0	0	0	0
SAL_OA1190AA	PNUSAS034490	Bovismorbificans	0	0	0	0	0	0	0	0	0	0	0
SAL_OA4227AA	18EP000407	Bovismorbificans	0	0	0	0	0	0	0	0	0	0	0
SAL_OA7345AA	PNUSAS037262	Bovismorbificans	0	0	0	0	0	0	0	0	0	0	0
SAL_OA7494AA	FSIS11808285	Bovismorbificans	0	0	0	0	0	0	0	0	0	0	0
SAL_PA2852AA	15-11-18-8100283-S	Bovismorbificans	0	0	0	0	0	0	0	0	0	0	0
SAL_PA2774AA	PNUSAS039198	Bovismorbificans	0	0	0	0	0	0	0	0	0	0	0
SAL_PA4504AA	FSIS21821537	Bovismorbificans	0	0	0	0	0	0	0	0	0	0	0
SAL_PA4728AA	PNUSAS040006	Bovismorbificans	0	0	0	0	0	0	0	0	0	0	0
SAL_PA4837AA	401591	Bovismorbificans	0	0	0	0	0	0	0	0	0	0	0
SAL_PA5597AA	308764	Bovismorbificans	12	0	39	62	15	0	35	4	62	5	1
SAL_PA6131AA	115994	Bovismorbificans	0	0	0	0	0	0	0	0	0	0	0
SAL_PA6145AA	470555	Bovismorbificans	0	0	0	0	0	0	0	0	0	0	0
SAL_PA6120AA	438779	Bovismorbificans	0	0	0	0	0	0	0	0	0	0	0
SAL_PA6302AA	PNUSAS040879	Bovismorbificans	0	0	0	0	0	0	0	0	0	0	0
SAL_PA6525AA	18EP000677	Bovismorbificans	12	0	39	62	15	0	3	4	5	78	1
SAL_PA6905AA	PNUSAS040431	Bovismorbificans	0	0	0	0	0	0	0	0	0	0	0
SAL_PA7919AA	451996	Bovismorbificans	12	0	39	62	15	0	35	4	62	5	1
SAL_PA7886AA	520500	Bovismorbificans	0	0	0	0	0	0	0	0	0	0	0
SAL_PA8248AA	PNUSAS041044	Bovismorbificans	0	0	0	0	0	0	0	0	0	0	0
SAL_PA8817AA	FDA00001697	Bovismorbificans	0	0	0	0	0	0	0	0	0	0	0
SAL_PA8570AA	450307	Bovismorbificans	0	0	0	0	0	0	0	0	0	0	0
SAL_QA0301AA	378708	Bovismorbificans	12	0	39	0	15	0	35	4	62	5	1
SAL_QA0303AA	430232	Bovismorbificans	12	0	39	0	15	0	35	4	62	5	1
SAL_QA0507AA	420534	Bovismorbificans	0	0	0	0	0	0	0	0	0	0	0
SAL_QA0712AA	528544	Bovismorbificans	12	0	39	0	4	0	3	4	5	5	1
SAL_QA0473AA	506862	Bovismorbificans	0	0	0	0	0	0	0	0	0	0	0
SAL_QA1391AA	PNUSAS042617	Bovismorbificans	0	0	0	0	0	0	0	0	0	0	0
SAL_QA1353AA	388636	Bovismorbificans	0	0	0	0	0	0	0	0	0	0	0
SAL_QA3762AA	24-21-17-8100832-D	Bovismorbificans	12	0	39	62	15	0	35	4	62	5	1
SAL_QA1500AA	385894	Bovismorbificans	0	0	0	0	0	0	0	0	0	0	0
SAL_QA2468AA	379401	Bovismorbificans	0	0	0	0	0	0	0	0	0	0	0
SAL_QA4977AA	18EP000926	Bovismorbificans	0	0	0	0	0	0	0	0	0	0	0
SAL_QA2309AA	372699	Bovismorbificans	0	0	0	0	0	0	0	0	0	0	0
SAL_QA2277AA	455102	Bovismorbificans	12	0	39	62	15	0	35	4	62	5	1
SAL_QA2163AA	121430	Bovismorbificans	0	0	0	0	0	0	0	0	0	0	0
SAL_QA2058AA	419056	Bovismorbificans	0	0	0	0	0	0	0	0	0	0	0
SAL_QA1481AA	370870	Bovismorbificans	0	0	0	0	0	0	0	0	0	0	0
SAL_QA2915AA	450305	Bovismorbificans	0	0	0	0	0	0	0	0	0	0	0
SAL_QA2884AA	500375	Bovismorbificans	0	0	0	0	0	0	0	0	0	0	0
SAL_QA2670AA	482151	Bovismorbificans	0	0	0	0	0	0	0	0	0	0	0

SAL_QA3342AA	404341	Bovismorbificans	12	0	39	62	15	0	35	4	62	5	1
SAL_QA3345AA	363587	Bovismorbificans	0	0	0	0	0	0	0	0	0	0	0
SAL_QA3913AA	412038	Bovismorbificans	0	0	0	0	0	0	0	0	0	0	0
SAL_QA3873AA	313892	Bovismorbificans	12	0	39	0	15	0	35	4	62	5	1
SAL_QA3954AA	FSIS21821897	Bovismorbificans	0	0	0	0	0	0	0	4	0	0	1
SAL_QA4358AA	PNUSAS042323	Bovismorbificans	0	0	0	0	0	0	0	0	0	0	0
SAL_QA4187AA	PNUSAS040690	Bovismorbificans	0	0	0	0	0	0	0	0	0	0	0
SAL_QA4826AA	470349	Bovismorbificans	0	0	0	0	0	0	0	0	0	0	0
SAL_QA5460AA	122212	Bovismorbificans	0	0	0	0	0	0	0	0	0	0	0
SAL_QA5886AA	411469	Bovismorbificans	0	0	0	0	0	0	0	0	0	0	0
SAL_QA5831AA	392821	Bovismorbificans	12	0	39	0	15	0	35	4	62	5	1
SAL_QA5771AA	513487	Bovismorbificans	0	0	0	0	0	0	0	0	0	0	0
SAL_QA5781AA	462575	Bovismorbificans	0	0	0	0	0	0	0	0	0	0	0
SAL_QA6045AA	PNUSAS043486	Bovismorbificans	0	0	0	0	0	0	0	0	0	0	0
SAL_QA6191AA	282491	Bovismorbificans	12	0	39	0	15	0	3	4	5	78	1
SAL_QA6132AA	FDA00004273	Bovismorbificans	0	0	0	0	0	0	0	0	0	0	0
SAL_QA6610AA	511816	Bovismorbificans	0	0	0	0	0	0	0	0	0	0	0
SAL_QA6481AA	392963	Bovismorbificans	12	0	39	0	15	0	35	4	62	5	1
SAL_QA6431AA	319686	Bovismorbificans	12	0	39	0	15	0	35	4	62	5	1
SAL_QA6446AA	392890	Bovismorbificans	12	0	39	0	15	0	35	4	62	5	1
SAL_QA5499AA	379402	Bovismorbificans	0	0	0	0	0	0	0	0	0	0	0
SAL_QA5501AA	364560	Bovismorbificans	0	0	0	0	0	0	0	0	0	0	0
SAL_QA6746AA	513486	Bovismorbificans	0	0	0	0	0	0	0	0	0	0	0
SAL_QA7028AA	PNUSAS042213	Bovismorbificans	12	0	39	62	15	0	35	4	62	5	1
SAL_QA7352AA	311163	Bovismorbificans	12	0	39	0	15	0	35	4	62	5	1
SAL_QA7339AA	478619	Bovismorbificans	12	0	39	0	15	0	35	4	62	5	1
SAL_QA7265AA	391281	Bovismorbificans	0	0	0	0	0	0	0	0	0	0	0
SAL_RA3210AA	SMG-18-895-R	Bovismorbificans	12	0	39	62	15	0	3	4	5	78	1
SAL_QA7699AA	372702	Bovismorbificans	0	0	0	0	0	0	0	0	0	0	0
SAL_RA3808AA	SMG-18-912	Bovismorbificans	0	0	0	0	0	0	0	0	0	0	0
SAL_QA7639AA	412127	Bovismorbificans	12	0	39	0	15	0	35	4	62	5	1
SAL_QA8428AA	416286	Bovismorbificans	12	0	39	62	15	0	35	4	62	5	1
SAL_QA8396AA	397666	Bovismorbificans	0	0	0	0	0	0	0	0	0	0	0
SAL_QA8363AA	438780	Bovismorbificans	0	0	0	0	0	0	0	0	0	0	0
SAL_QA8171AA	236214	Bovismorbificans	0	0	0	0	0	0	0	0	0	0	0
SAL_QA8783AA	450310	Bovismorbificans	0	0	0	0	0	0	0	0	0	0	0
SAL_QA8751AA	392931	Bovismorbificans	12	0	39	62	15	0	35	4	62	5	1
SAL_QA8710AA	526180	Bovismorbificans	0	0	0	0	0	0	0	0	0	0	0
SAL_QA8912AA	FSIS11811127	Bovismorbificans	0	0	0	0	0	0	0	0	0	0	0
SAL_RA6979AA	SMG-18-954	Bovismorbificans	12	0	39	62	15	0	3	4	5	78	1
SAL_RA6982AA	SMG-18-955	Bovismorbificans	12	0	39	62	15	0	3	4	5	78	1
SAL_RA0327AA	367464	Bovismorbificans	0	0	0	0	0	0	0	0	0	0	0
SAL_RA0273AA	122208	Bovismorbificans	0	0	0	0	0	0	0	0	0	0	0
SAL_RA0998AA	387147	Bovismorbificans	0	0	0	0	0	0	0	0	0	0	0
SAL_RA0953AA	FSIS11811233	Bovismorbificans	12	0	39	62	15	0	181	4	5	78	1
SAL_RA0805AA	399295	Bovismorbificans	0	0	0	0	0	0	0	0	0	0	0
SAL_RA1450AA	PNUSAS043983	Bovismorbificans	0	0	0	0	0	0	0	0	0	0	0
SAL_RA2848AA	513476	Bovismorbificans	0	0	0	0	0	0	0	0	0	0	0
SAL_RA3227AA	555483	Bovismorbificans	0	0	0	0	0	0	0	0	0	0	0
SAL_RA3308AA	453248	Bovismorbificans	0	0	0	0	0	0	0	0	0	0	0
SAL_RA3934AA	457303	Bovismorbificans	0	0	0	0	0	0	0	0	0	0	0
SAL_RA4423AA	449714	Bovismorbificans	0	0	0	0	0	0	0	0	0	0	0
SAL_RA5912AA	PNUSAS045629	Bovismorbificans	0	0	0	0	0	0	0	0	0	0	0
SAL_RA5643AA	449717	Bovismorbificans	0	0	0	0	0	0	0	0	0	0	0
SAL_RA5633AA	449708	Bovismorbificans	0	0	0	0	0	0	0	0	0	0	0
SAL_SA0868AA	SMG-18-1177	Bovismorbificans	0	0	0	0	0	0	0	0	0	0	0
SAL_RA2710AA	467159	Bovismorbificans	0	0	0	0	0	0	0	0	0	0	0
SAL_RA2655AA	378636	Bovismorbificans	12	0	39	0	15	0	35	4	62	5	1
SAL_RA2665AA	495810	Bovismorbificans	0	0	0	0	0	0	0	0	0	0	0
SAL_RA2684AA	402879	Bovismorbificans	12	0	39	0	15	0	35	4	62	5	1
SAL_RA6420AA	PNUSAS040518	Bovismorbificans	0	0	0	0	0	0	0	0	0	0	0
SAL_RA6550AA	492501	Bovismorbificans	0	0	0	0	0	0	0	0	0	0	0
SAL_RA7504AA	550773	Bovismorbificans	0	0	0	0	0	0	0	0	0	0	0
SAL_RA7476AA	423861	Bovismorbificans	0	0	0	0	0	0	0	0	0	0	0
SAL_RA7304AA	568334	Bovismorbificans	12	0	39	0	15	0	35	4	62	5	1
SAL_RA7274AA	569956	Bovismorbificans	0	0	0	0	0	0	0	0	0	0	0
SAL_RA7043AA	417417	Bovismorbificans	12	0	39	62	15	0	35	4	62	5	1
SAL_RA7016AA	376244	Bovismorbificans	0	0	0	0	0	0	0	0	0	0	0
SAL_RA8124AA	538388	Bovismorbificans	0	0	0	0	0	0	0	0	0	0	0
SAL_RA7940AA	449716	Bovismorbificans	0	0	0	0	0	0	0	0	0	0	0
SAL_RA8319AA	394936	Bovismorbificans	12	0	39	0	15	0	35	4	62	5	1
SAL_RA8681AA	FSIS11812173	Bovismorbificans	0	0	0	0	0	0	0	0	0	0	0
SAL_RA7289AA	568326	Bovismorbificans	12	0	39	0	15	0	3	4	5	78	1

SAL_SA1052AA	CNSV-T15-MD05-0.1-RV-	Bovismorbificans	0	0	0	0	0	0	0	0	0	0	0
SAL_SA3299AA	OH-18-18132-11	Bovismorbificans	0	0	0	0	0	0	0	0	0	0	0
SAL_SA3306AA	OH-18-18132-8	Bovismorbificans	0	0	0	0	0	0	0	0	0	0	0
SAL_SA6540AA	18EP001797	Bovismorbificans	12	0	39	62	15	0	3	4	5	78	1
SAL_SA4904AA	582293	Bovismorbificans	12	0	39	62	15	0	35	4	62	5	1
SAL_SA4835AA	445187	Bovismorbificans	12	0	39	0	15	0	35	4	62	5	1
SAL_SA4855AA	585972	Bovismorbificans	0	0	0	0	0	0	0	0	0	0	0
SAL_SA7314AA	L1055	Bovismorbificans	0	0	0	0	0	0	0	0	0	0	0
SAL_SA5882AA	572337	Bovismorbificans	0	0	0	0	0	0	0	0	0	0	0
SAL_SA5833AA	576311	Bovismorbificans	0	0	0	0	0	0	0	0	0	0	0
SAL_SA5782AA	594701	Bovismorbificans	0	0	0	0	0	0	0	0	0	0	0
SAL_SA6334AA	449711	Bovismorbificans	0	0	0	0	0	0	0	0	0	0	0
SAL_SA6571AA	470350	Bovismorbificans	0	0	0	0	0	0	0	0	0	0	0
SAL_SA7148AA	580431	Bovismorbificans	0	0	0	0	0	0	0	0	0	0	0
SAL_SA7554AA	527504	Bovismorbificans	12	0	39	0	15	0	35	4	62	5	1
SAL_SA7494AA	578036	Bovismorbificans	0	0	0	0	0	0	0	0	0	0	0
SAL_SA7393AA	449712	Bovismorbificans	0	0	0	0	0	0	0	0	0	0	0
SAL_SA7357AA	578051	Bovismorbificans	0	0	0	0	0	0	0	0	0	0	0
SAL_SA8601AA	586808	Bovismorbificans	0	0	0	0	0	0	0	0	0	0	0
SAL_SA9572AA	391273	Bovismorbificans	12	0	39	62	15	0	35	4	62	5	1
SAL_SA9578AA	404923	Bovismorbificans	12	0	39	0	15	0	35	4	62	5	1
SAL_TA0204AA	50-30-18-8101003-Y	Bovismorbificans	0	0	0	0	0	0	0	0	0	0	0
SAL_TA0219AA	50-15-18-8100988-N	Bovismorbificans	0	0	0	0	0	0	0	0	0	0	0
SAL_TA0238AA	49-17-18.8100961.F	Bovismorbificans	0	0	0	0	0	0	0	0	0	0	0
SAL_TA0545AA	SMG-18-1952	Bovismorbificans	0	0	0	0	0	0	0	0	0	0	0
SAL_TA1612AA	SMG-18-2049	Bovismorbificans	0	0	0	0	0	0	0	0	0	0	0
SAL_TA2083AA	SMG-18-2095	Bovismorbificans	0	0	0	0	0	0	0	0	0	0	0
SAL_TA2360AA	620409	Bovismorbificans	12	0	39	0	15	0	35	4	62	5	1
SAL_TA2783AA	624467	Bovismorbificans	0	0	0	0	0	0	0	0	0	0	0
SAL_TA4212AA	56-18-18-8101166-M	Bovismorbificans	12	0	39	62	15	0	3	4	5	78	1
SAL_TA5279AA	628057	Bovismorbificans	12	0	39	62	15	0	3	4	5	78	1
SAL_TA6297AA	SMG-18-2161	Bovismorbificans	12	0	39	62	15	0	3	4	5	78	1
SAL_TA6302AA	SMG-18-2135	Bovismorbificans	12	0	39	62	15	0	3	4	5	78	1
SAL_TA6300AA	SMG-18-2165	Bovismorbificans	0	0	0	0	0	0	0	0	0	0	0
SAL_TA6476AA	Salmonella enterica	Bovismorbificans	0	0	0	0	0	0	0	0	0	0	0
SAL_OA5311AA	PNUSAS035942	Bovismorbificans	0	0	0	0	0	0	0	0	0	0	0
SAL_PA1664AA	PNUSAS038713	Bovismorbificans	0	0	0	0	0	0	0	0	0	0	0
SAL_TA7695AA	R17.5433	Bovismorbificans	0	0	0	0	0	0	0	0	0	0	0
SAL_TA7849AA	ples of swine - serotype	Bovismorbificans	0	0	0	0	0	0	0	0	0	0	0
SAL_TA7821AA	FSIS31801249	Bovismorbificans	0	0	0	0	0	0	0	0	0	0	0
SAL_QA7973AA	PNUSAS043391	Bovismorbificans	0	0	0	0	0	0	0	0	0	0	0
SAL_TA8340AA	638756	Bovismorbificans	0	0	0	0	0	0	0	0	0	0	0
SAL_TA8197AA	ADRDL-2280	Bovismorbificans	0	0	0	0	0	0	0	0	0	0	0
SAL_SA6823AA	573515	Bovismorbificans	0	0	0	0	0	0	0	0	0	0	0
SAL_OA3095AA	S05124-09	Bovismorbificans	0	0	0	0	0	0	0	0	0	0	0
SAL_OA1290AA	PNUSAS034567	Bovismorbificans	0	0	0	0	0	0	0	0	0	0	0
SAL_OA1669AA	PNUSAS033272	Bovismorbificans	0	0	0	0	0	0	0	0	0	0	0
SAL_OA2295AA	PNUSAS034018	Bovismorbificans	0	0	0	0	0	0	0	0	0	0	0
SAL_OA3075AA	S05900-09	Bovismorbificans	0	0	0	0	0	0	0	0	0	0	0
SAL_OA3079AA	S05880-09	Bovismorbificans	0	0	0	0	0	0	0	0	0	0	0
SAL_OA3076AA	S05899-09	Bovismorbificans	0	0	0	0	0	0	0	0	0	0	0
SAL_OA3094AA	S05129-09	Bovismorbificans	0	0	0	0	0	0	0	0	0	0	0
SAL_PA1983AA	0-e94c-11e6-874d-3c4a9	Bovismorbificans	0	0	0	0	0	0	0	0	0	0	0
SAL_TA9820AA	SMG-18-2317	Bovismorbificans	12	0	39	62	15	0	3	4	5	78	1
SAL_UA1628AA	CFSAN064033	Bovismorbificans	0	0	0	0	0	0	0	0	0	0	0
SAL_UA3693AA	599475	Bovismorbificans	0	0	0	0	0	0	0	0	0	0	0
SAL_UA3726AA	601715	Bovismorbificans	12	0	39	62	15	0	35	4	62	5	1
SAL_UA4733AA	598023	Bovismorbificans	12	0	39	62	15	0	35	4	62	5	1
SAL_UA4816AA	592660	Bovismorbificans	0	0	0	0	0	0	0	0	0	0	0
SAL_UA6526AA	580486	Bovismorbificans	0	0	0	0	0	0	0	0	0	0	0
SAL_UA7017AA	659327	Bovismorbificans	12	0	39	0	15	0	35	4	62	5	1
SAL_UA7813AA	OH-16-2271	Bovismorbificans	0	0	0	0	0	0	0	0	0	0	0
SAL_UA8066AA	ADRDL-2288	Bovismorbificans	0	0	0	0	0	0	0	0	0	0	0
SAL_UA8304AA	668686	Bovismorbificans	12	0	39	0	15	0	35	4	62	5	1
SAL_UA9564AA	633453	Bovismorbificans	0	0	0	0	0	0	0	0	0	0	0
SAL_VA0213AA	568269	Bovismorbificans	0	0	0	0	0	0	0	0	0	0	0
SAL_UA9986AA	623192	Bovismorbificans	0	0	0	0	0	0	0	0	0	0	0
SAL_UA9934AA	556819	Bovismorbificans	12	0	39	0	15	0	3	4	5	78	1
SAL_VA0756AA	513473	Bovismorbificans	0	0	0	0	0	0	0	0	0	0	0
SAL_VA0421AA	549482	Bovismorbificans	0	0	0	0	0	0	0	0	0	0	0
SAL_VA0446AA	534813	Bovismorbificans	0	0	0	0	0	0	0	0	0	0	0
SAL_VA0402AA	543891	Bovismorbificans	0	0	0	0	0	0	0	0	0	0	0
SAL_VA1150AA	513484	Bovismorbificans	0	0	0	0	0	0	0	0	0	0	0

SAL_VA1152AA	674349	Bovismorbificans	12	0	39	0	15	0	35	4	62	5	1
SAL_VA7061AA	SMG-19-202	Bovismorbificans	0	0	0	0	0	0	0	0	0	0	0
SAL_VA1424AA	492500	Bovismorbificans	0	0	0	0	0	0	0	0	0	0	0
SAL_VA1331AA	492502	Bovismorbificans	0	0	0	0	0	0	0	0	0	0	0
SAL_VA1586AA	H2 - 013	Bovismorbificans	0	0	0	0	0	0	0	0	0	0	0
SAL_VA7446AA	201703159	Bovismorbificans	0	0	0	0	0	0	0	0	0	0	0
SAL_VA7544AA	201705939	Bovismorbificans	0	0	0	0	0	0	0	0	0	0	0
SAL_VA2215AA	474631	Bovismorbificans	0	0	0	0	0	0	0	0	0	0	0
SAL_VA2202AA	470200	Bovismorbificans	0	0	0	0	0	0	0	0	0	0	0
SAL_VA2122AA	449718	Bovismorbificans	0	0	0	0	0	0	0	0	0	0	0
SAL_VA3277AA	416399	Bovismorbificans	0	0	0	0	0	0	0	0	0	0	0
SAL_VA3212AA	680062	Bovismorbificans	12	0	39	0	15	0	35	4	62	5	1
SAL_VA3472AA	397386	Bovismorbificans	0	0	0	0	0	0	0	0	0	0	0
SAL_VA3412AA	394877	Bovismorbificans	0	0	0	0	0	0	0	0	0	0	0
SAL_VA3448AA	404900	Bovismorbificans	0	0	0	0	0	0	0	0	0	0	0
SAL_VA2304AA	486488	Bovismorbificans	0	0	0	0	0	0	0	0	0	0	0
SAL_VA2274AA	475834	Bovismorbificans	0	0	0	0	0	0	0	0	0	0	0
SAL_VA2004AA	438776	Bovismorbificans	0	0	0	0	0	0	0	0	0	0	0
SAL_VA1919AA	440065	Bovismorbificans	0	0	0	0	0	0	0	0	0	0	0
SAL_VA1877AA	452487	Bovismorbificans	0	0	0	0	0	0	0	0	0	0	0
SAL_VA1896AA	436919	Bovismorbificans	0	0	0	0	0	0	0	0	0	0	0
SAL_VA2471AA	431390	Bovismorbificans	12	0	39	62	15	0	35	4	62	5	1
SAL_VA2565AA	425680	Bovismorbificans	0	0	0	0	0	0	0	0	0	0	0
SAL_VA2437AA	423913	Bovismorbificans	0	0	0	0	0	0	0	0	0	0	0
SAL_VA2415AA	445952	Bovismorbificans	0	0	0	0	0	0	0	0	0	0	0
SAL_VA2771AA	423860	Bovismorbificans	0	0	0	0	0	0	0	0	0	0	0
SAL_VA1932AA	449715	Bovismorbificans	0	0	0	0	0	0	0	0	0	0	0
SAL_VA1939AA	438777	Bovismorbificans	0	0	0	0	0	0	0	0	0	0	0
SAL_VA5737AA	SA20092314	Bovismorbificans	0	0	0	0	0	0	0	0	0	0	0
SAL_VA5686AA	SA20112854	Bovismorbificans	0	0	0	0	0	0	0	0	0	0	0
SAL_VA5693AA	SA20090203	Bovismorbificans	0	0	0	0	0	0	0	0	0	0	0
SAL_VA4512AA	392965	Bovismorbificans	12	0	39	62	15	0	35	4	62	5	1
SAL_VA4405AA	391275	Bovismorbificans	12	0	39	0	15	0	35	4	62	5	1
SAL_VA4336AA	392887	Bovismorbificans	12	0	39	0	15	0	35	4	62	5	1
SAL_VA4370AA	397689	Bovismorbificans	12	0	39	0	15	0	35	4	62	5	1
SAL_WA1595AA	SMG-19-375	Bovismorbificans	12	0	39	62	15	0	35	4	0	5	1
SAL_WA1631AA	19EP000543	Bovismorbificans	0	0	0	0	0	0	0	0	0	0	0
SAL_VA6943AA	372681	Bovismorbificans	12	0	39	0	15	0	35	4	62	5	1
SAL_VA6858AA	381315	Bovismorbificans	12	0	39	0	15	0	35	4	62	5	1
SAL_VA7247AA	FSIS31901560	Bovismorbificans	0	0	0	0	0	0	0	0	0	0	0
SAL_VA7226AA	683123	Bovismorbificans	12	0	39	0	15	0	35	4	62	5	1
SAL_VA7207AA	678541	Bovismorbificans	0	0	0	0	0	0	0	0	0	0	0
SAL_VA7152AA	682253	Bovismorbificans	12	0	39	0	15	0	35	4	62	5	1
SAL_VA8244AA	690348	Bovismorbificans	0	0	0	0	0	0	0	0	0	0	0
SAL_VA8623AA	360310	Bovismorbificans	0	0	0	0	0	0	0	0	0	0	0
SAL_VA9168AA	307437	Bovismorbificans	12	0	39	0	15	0	35	4	62	5	1
SAL_VA9628AA	692616	Bovismorbificans	12	0	39	0	15	0	35	4	62	5	1
SAL_VA9666AA	692622	Bovismorbificans	12	0	39	0	15	0	35	4	62	5	1
SAL_VA4055AA	CDPH_CSalm295	Bovismorbificans	0	0	0	0	0	0	0	0	0	0	0
SAL_WA1324AA	699228	Bovismorbificans	0	0	0	0	0	0	0	0	0	0	0
SAL_WA3285AA	QA08/18	Bovismorbificans	0	0	0	0	0	0	0	0	0	0	0
SAL_WA1186AA	699229	Bovismorbificans	12	0	39	0	15	0	35	4	62	5	1
SAL_WA2179AA	CK_00/00000735	Bovismorbificans	0	0	0	0	0	0	0	0	0	0	0
SAL_WA1680AA	109258	Bovismorbificans	0	0	0	0	0	0	0	0	0	0	0
SAL_WA2113AA	SAL-18-VL-OH-IN-0022	Bovismorbificans	0	0	0	0	0	0	0	0	0	0	0
SAL_WA3335AA	707090	Bovismorbificans	12	0	39	0	15	0	35	4	62	5	1
SAL_WA3625AA	CFSAN087495	Bovismorbificans	0	0	0	0	0	0	0	0	0	0	0
SAL_WA3572AA	CFSAN087524	Bovismorbificans	0	0	0	0	0	0	0	0	0	0	0
SAL_WA3944AA	710475	Bovismorbificans	0	0	0	0	0	0	0	0	0	0	0
SAL_OA0472AA	FD01843868	Bradford	0	0	0	0	0	0	0	0	0	0	0
SAL_DA2358AA	POR1-0017	Braenderup	0	0	0	0	0	0	0	0	0	0	0
SAL_BA9908AA	:-2012-07-27T11:53:07Z-	Braenderup	0	0	0	0	0	0	0	0	0	0	0
SAL_CA4747AA	65410	Braenderup	0	0	0	0	0	0	0	0	0	0	0
SAL_DA1780AA	H123760513	Braenderup	0	0	0	0	0	0	0	0	0	0	0
SAL_DA8705AA	FL-MDD-77	Braenderup	0	0	0	0	0	0	0	0	0	0	0
SAL_EA3260AA	CFSAN034994	Braenderup	0	0	0	0	0	0	0	0	0	0	0
SAL_CA2363AA	63909	Braenderup	0	0	0	0	0	0	0	0	0	0	0
SAL_BA1597AA	CFSAN035388	Braenderup	0	0	0	0	0	0	0	0	0	0	0
SAL_CA5232AA	79027	Braenderup	0	0	0	0	0	0	0	0	0	0	0
SAL_BA8080AA	72305	Braenderup	0	0	0	0	0	0	0	0	0	0	0
SAL_FA5855AA	FDA00009775	Braenderup	0	0	0	0	0	0	0	0	0	0	0
SAL_FA5534AA	PNUSAS001889	Braenderup	0	0	0	0	0	0	0	0	0	0	0
SAL_FA5375AA	043V3	Braenderup	0	0	0	0	0	0	0	0	0	0	0

SAL_FA5025AA	2013K-1014	Braenderup	0	0	0	0	0	0	0	0	0	0	0
SAL_FA4909AA	221647	Braenderup	0	0	0	0	0	0	0	0	0	0	0
SAL_FA4908AA	232002	Braenderup	0	0	0	0	0	0	0	0	0	0	0
SAL_FA4903AA	236210	Braenderup	0	0	0	0	0	0	0	0	0	0	0
SAL_FA4901AA	232729	Braenderup	0	0	0	0	0	0	0	0	0	0	0
SAL_FA4907AA	229174	Braenderup	0	0	0	0	0	0	0	0	0	0	0
SAL_FA4905AA	223501	Braenderup	0	0	0	0	0	0	0	0	0	0	0
SAL_FA4906AA	218455	Braenderup	0	0	0	0	0	0	0	0	0	0	0
SAL_FA4904AA	222198	Braenderup	0	0	0	0	0	0	0	0	0	0	0
SAL_FA4897AA	223492	Braenderup	0	0	0	0	0	0	0	0	0	0	0
SAL_FA4902AA	232713	Braenderup	0	0	0	0	0	0	0	0	0	0	0
SAL_FA4899AA	223491	Braenderup	0	0	0	0	0	0	0	0	0	0	0
SAL_FA4892AA	221650	Braenderup	0	0	0	0	0	0	0	0	0	0	0
SAL_FA4898AA	226970	Braenderup	0	0	0	0	0	0	0	0	0	0	0
SAL_FA4900AA	227044	Braenderup	0	0	0	0	0	0	0	0	0	0	0
SAL_FA4894AA	229072	Braenderup	0	0	0	0	0	0	0	0	0	0	0
SAL_FA4890AA	236276	Braenderup	0	0	0	0	0	0	0	0	0	0	0
SAL_FA4893AA	227009	Braenderup	0	0	0	0	0	0	0	0	0	0	0
SAL_FA4896AA	229858	Braenderup	0	0	0	0	0	0	0	0	0	0	0
SAL_FA4883AA	223498	Braenderup	0	0	0	0	0	0	0	0	0	0	0
SAL_FA4895AA	223500	Braenderup	0	0	0	0	0	0	0	0	0	0	0
SAL_FA4885AA	231232	Braenderup	0	0	0	0	0	0	0	0	0	0	0
SAL_FA4884AA	221649	Braenderup	0	0	0	0	0	0	0	0	0	0	0
SAL_FA4888AA	231973	Braenderup	0	0	0	0	0	0	0	0	0	0	0
SAL_FA4880AA	223496	Braenderup	0	0	0	0	0	0	0	0	0	0	0
SAL_FA4887AA	223503	Braenderup	0	0	0	0	0	0	0	0	0	0	0
SAL_FA4886AA	216308	Braenderup	0	0	0	0	0	0	0	0	0	0	0
SAL_FA4889AA	236277	Braenderup	0	0	0	0	0	0	0	0	0	0	0
SAL_FA4879AA	231113	Braenderup	0	0	0	0	0	0	0	0	0	0	0
SAL_FA4881AA	223490	Braenderup	0	0	0	0	0	0	0	0	0	0	0
SAL_FA4878AA	221648	Braenderup	0	0	0	0	0	0	0	0	0	0	0
SAL_FA4882AA	223495	Braenderup	0	0	0	0	0	0	0	0	0	0	0
SAL_FA4891AA	222192	Braenderup	0	0	0	0	0	0	0	0	0	0	0
SAL_FA4649AA	196405	Braenderup	0	0	0	0	0	0	0	0	0	0	0
SAL_FA4591AA	169590	Braenderup	0	0	0	0	0	0	0	0	0	0	0
SAL_FA4528AA	192909	Braenderup	0	0	0	0	0	0	0	0	0	0	0
SAL_FA4520AA	128241	Braenderup	0	0	0	0	0	0	0	0	0	0	0
SAL_FA4468AA	142683	Braenderup	0	0	0	0	0	0	0	0	0	0	0
SAL_FA4445AA	211764	Braenderup	0	0	0	0	0	0	0	0	0	0	0
SAL_FA4432AA	113049	Braenderup	0	0	0	0	0	0	0	0	0	0	0
SAL_FA4419AA	113048	Braenderup	0	0	0	0	0	0	0	0	0	0	0
SAL_FA4378AA	185980	Braenderup	0	0	0	0	0	0	0	0	0	0	0
SAL_FA4276AA	199813	Braenderup	0	0	0	0	0	0	0	0	0	0	0
SAL_FA4186AA	187552	Braenderup	0	0	0	0	0	0	0	0	0	0	0
SAL_FA4175AA	191963	Braenderup	0	0	0	0	0	0	0	0	0	0	0
SAL_FA4149AA	160835	Braenderup	0	0	0	0	0	0	0	0	0	0	0
SAL_FA4025AA	2013K-1016	Braenderup	0	0	0	0	0	0	0	0	0	0	0
SAL_FA4028AA	2013K-1019	Braenderup	0	0	0	0	0	0	0	0	0	0	0
SAL_FA4023AA	2013K-1184	Braenderup	0	0	0	0	0	0	0	0	0	0	0
SAL_FA4027AA	2013K-1018	Braenderup	0	0	0	0	0	0	0	0	0	0	0
SAL_FA4026AA	2013K-1017	Braenderup	0	0	0	0	0	0	0	0	0	0	0
SAL_FA4029AA	2013K-1020	Braenderup	0	0	0	0	0	0	0	0	0	0	0
SAL_FA4024AA	2013K-1013	Braenderup	0	0	0	0	0	0	0	0	0	0	0
SAL_FA4022AA	2013K-1011	Braenderup	0	0	0	0	0	0	0	0	0	0	0
SAL_FA4010AA	FDA00007853	Braenderup	0	0	0	0	0	0	0	0	0	0	0
SAL_FA3063AA	CVM N56565	Braenderup	0	0	0	0	0	0	0	0	0	0	0
SAL_FA2423AA	PNUSAS001735	Braenderup	0	0	0	0	0	0	0	0	0	0	0
SAL_FA2301AA	PNUSAS001450	Braenderup	0	0	0	0	0	0	0	0	0	0	0
SAL_FA2302AA	PNUSAS001449	Braenderup	0	0	0	0	0	0	0	0	0	0	0
SAL_FA2300AA	PNUSAS001451	Braenderup	0	0	0	0	0	0	0	0	0	0	0
SAL_FA2299AA	PNUSAS001452	Braenderup	0	0	0	0	0	0	0	0	0	0	0
SAL_FA1347AA	FDA00009943	Braenderup	0	0	0	0	0	0	0	0	0	0	0
SAL_FA1316AA	WAPHL-SAL-A01046	Braenderup	0	0	0	0	0	0	0	0	0	0	0
SAL_FA0829AA	CFSAN038504	Braenderup	0	0	0	0	0	0	0	0	0	0	0
SAL_FA0651AA	FDA00002929	Braenderup	0	0	0	0	0	0	0	0	0	0	0
SAL_FA0578AA	IEH-NGS-SAL-03387	Braenderup	0	0	0	0	0	0	0	0	0	0	0
SAL_EA9868AA	CFSAN038477	Braenderup	0	0	0	0	0	0	0	0	0	0	0
SAL_EA9870AA	CFSAN038543	Braenderup	0	0	0	0	0	0	0	0	0	0	0
SAL_EA9867AA	CFSAN038480	Braenderup	0	0	0	0	0	0	0	0	0	0	0
SAL_EA9869AA	CFSAN038460	Braenderup	0	0	0	0	0	0	0	0	0	0	0
SAL_EA9603AA	PNUSAS001457	Braenderup	0	0	0	0	0	0	0	0	0	0	0
SAL_EA9554AA	CFSAN038512	Braenderup	0	0	0	0	0	0	0	0	0	0	0
SAL_EA9558AA	CFSAN038508	Braenderup	0	0	0	0	0	0	0	0	0	0	0

SAL_EA9559AA	CFSAN038507	Braenderup	0	0	0	0	0	0	0	0	0	0	0
SAL_EA9553AA	CFSAN038513	Braenderup	0	0	0	0	0	0	0	0	0	0	0
SAL_EA9551AA	CFSAN038515	Braenderup	0	0	0	0	0	0	0	0	0	0	0
SAL_EA9552AA	CFSAN038514	Braenderup	0	0	0	0	0	0	0	0	0	0	0
SAL_EA9555AA	CFSAN038511	Braenderup	0	0	0	0	0	0	0	0	0	0	0
SAL_EA9548AA	CFSAN038518	Braenderup	0	0	0	0	0	0	0	0	0	0	0
SAL_EA9550AA	CFSAN038516	Braenderup	0	0	0	0	0	0	0	0	0	0	0
SAL_EA9549AA	CFSAN038517	Braenderup	0	0	0	0	0	0	0	0	0	0	0
SAL_EA9557AA	CFSAN038509	Braenderup	0	0	0	0	0	0	0	0	0	0	0
SAL_EA9556AA	CFSAN038510	Braenderup	0	0	0	0	0	0	0	0	0	0	0
SAL_EA9131AA	CFSAN031735	Braenderup	0	0	0	0	0	0	0	0	0	0	0
SAL_EA9014AA	NY-N20005	Braenderup	0	0	0	0	0	0	0	0	0	0	0
SAL_EA8868AA	WAPHL-SAL-A01024	Braenderup	0	0	0	0	0	0	0	0	0	0	0
SAL_EA8682AA	FDA00007851	Braenderup	0	0	0	0	0	0	0	0	0	0	0
SAL_EA8606AA	NY-N19896	Braenderup	0	0	0	0	0	0	0	0	0	0	0
SAL_EA8599AA	POR1-0163	Braenderup	0	0	0	0	0	0	0	0	0	0	0
SAL_EA8585AA	POR1-0104	Braenderup	0	0	0	0	0	0	0	0	0	0	0
SAL_EA8581AA	FDA00007841	Braenderup	0	0	0	0	0	0	0	0	0	0	0
SAL_EA8580AA	FDA00007859	Braenderup	0	0	0	0	0	0	0	0	0	0	0
SAL_EA8219AA	CFSAN031815	Braenderup	0	0	0	0	0	0	0	0	0	0	0
SAL_EA8117AA	FDA00009673	Braenderup	0	0	0	0	0	0	0	0	0	0	0
SAL_EA8095AA	45208	Braenderup	0	0	0	0	0	0	0	0	0	0	0
SAL_EA8032AA	21099	Braenderup	0	0	0	0	0	0	0	0	0	0	0
SAL_EA7952AA	53032	Braenderup	0	0	0	0	0	0	0	0	0	0	0
SAL_EA7954AA	53028	Braenderup	0	0	0	0	0	0	0	0	0	0	0
SAL_EA7876AA	53037	Braenderup	0	0	0	0	0	0	0	0	0	0	0
SAL_EA7860AA	40361	Braenderup	0	0	0	0	0	0	0	0	0	0	0
SAL_EA7843AA	21067	Braenderup	0	0	0	0	0	0	0	0	0	0	0
SAL_EA7720AA	56977	Braenderup	0	0	0	0	0	0	0	0	0	0	0
SAL_EA7690AA	56984	Braenderup	0	0	0	0	0	0	0	0	0	0	0
SAL_EA7500AA	21859	Braenderup	0	0	0	0	0	0	0	0	0	0	0
SAL_EA7452AA	45207	Braenderup	0	0	0	0	0	0	0	0	0	0	0
SAL_EA7442AA	58578	Braenderup	0	0	0	0	0	0	0	0	0	0	0
SAL_EA7426AA	97262	Braenderup	0	0	0	0	0	0	0	0	0	0	0
SAL_EA7188AA	15226	Braenderup	0	0	0	0	0	0	0	0	0	0	0
SAL_EA7073AA	1859	Braenderup	0	0	0	0	0	0	0	0	0	0	0
SAL_EA6915AA	95009	Braenderup	0	0	0	0	0	0	0	0	0	0	0
SAL_EA6864AA	FDA00007830	Braenderup	0	0	0	0	0	0	0	0	0	0	0
SAL_EA6862AA	FDA00007832	Braenderup	0	0	0	0	0	0	0	0	0	0	0
SAL_EA6785AA	CFSAN038461	Braenderup	0	0	0	0	0	0	0	0	0	0	0
SAL_EA6783AA	CFSAN038467	Braenderup	0	0	0	0	0	0	0	0	0	0	0
SAL_EA6781AA	CFSAN038469	Braenderup	0	0	0	0	0	0	0	0	0	0	0
SAL_EA6776AA	CFSAN038474	Braenderup	0	0	0	0	0	0	0	0	0	0	0
SAL_EA6777AA	CFSAN038473	Braenderup	0	0	0	0	0	0	0	0	0	0	0
SAL_EA6784AA	CFSAN038466	Braenderup	0	0	0	0	0	0	0	0	0	0	0
SAL_EA6780AA	CFSAN038470	Braenderup	0	0	0	0	0	0	0	0	0	0	0
SAL_EA6782AA	CFSAN038468	Braenderup	0	0	0	0	0	0	0	0	0	0	0
SAL_EA6775AA	CFSAN038475	Braenderup	0	0	0	0	0	0	0	0	0	0	0
SAL_EA6778AA	CFSAN038472	Braenderup	0	0	0	0	0	0	0	0	0	0	0
SAL_EA6779AA	CFSAN038471	Braenderup	0	0	0	0	0	0	0	0	0	0	0
SAL_EA6488AA	CFSAN038462	Braenderup	0	0	0	0	0	0	0	0	0	0	0
SAL_EA6487AA	CFSAN038464	Braenderup	0	0	0	0	0	0	0	0	0	0	0
SAL_EA6478AA	CFSAN038478	Braenderup	0	0	0	0	0	0	0	0	0	0	0
SAL_EA6476AA	CFSAN038481	Braenderup	0	0	0	0	0	0	0	0	0	0	0
SAL_EA6484AA	CFSAN038465	Braenderup	0	0	0	0	0	0	0	0	0	0	0
SAL_EA6479AA	CFSAN038476	Braenderup	0	0	0	0	0	0	0	0	0	0	0
SAL_EA6475AA	CFSAN038482	Braenderup	0	0	0	0	0	0	0	0	0	0	0
SAL_EA6477AA	CFSAN038479	Braenderup	0	0	0	0	0	0	0	0	0	0	0
SAL_EA6356AA	CFSAN038528	Braenderup	0	0	0	0	0	0	0	0	0	0	0
SAL_EA6293AA	MOD1-Lipp-111	Braenderup	0	0	0	0	0	0	0	0	0	0	0
SAL_EA6294AA	MOD1-Lipp-13	Braenderup	0	0	0	0	0	0	0	0	0	0	0
SAL_EA5824AA	CFSAN035014	Braenderup	0	0	0	0	0	0	0	0	0	0	0
SAL_EA5823AA	CFSAN035015	Braenderup	0	0	0	0	0	0	0	0	0	0	0
SAL_EA5794AA	CFSAN038519	Braenderup	0	0	0	0	0	0	0	0	0	0	0
SAL_EA5790AA	CFSAN038524	Braenderup	0	0	0	0	0	0	0	0	0	0	0
SAL_EA5793AA	CFSAN038520	Braenderup	0	0	0	0	0	0	0	0	0	0	0
SAL_EA5791AA	CFSAN038523	Braenderup	0	0	0	0	0	0	0	0	0	0	0
SAL_EA5783AA	CFSAN038532	Braenderup	0	0	0	0	0	0	0	0	0	0	0
SAL_EA5784AA	CFSAN038531	Braenderup	0	0	0	0	0	0	0	0	0	0	0
SAL_EA5789AA	CFSAN038525	Braenderup	0	0	0	0	0	0	0	0	0	0	0
SAL_EA5787AA	CFSAN038527	Braenderup	0	0	0	0	0	0	0	0	0	0	0
SAL_EA5786AA	CFSAN038529	Braenderup	0	0	0	0	0	0	0	0	0	0	0
SAL_EA5785AA	CFSAN038530	Braenderup	0	0	0	0	0	0	0	0	0	0	0

SAL_EA5792AA	CFSAN038521	Braenderup	0	0	0	0	0	0	0	0	0	0	0
SAL_EA5788AA	CFSAN038526	Braenderup	0	0	0	0	0	0	0	0	0	0	0
SAL_EA5781AA	CFSAN038534	Braenderup	0	0	0	0	0	0	0	0	0	0	0
SAL_EA5779AA	CFSAN038536	Braenderup	0	0	0	0	0	0	0	0	0	0	0
SAL_EA5777AA	CFSAN038538	Braenderup	0	0	0	0	0	0	0	0	0	0	0
SAL_EA5778AA	CFSAN038537	Braenderup	0	0	0	0	0	0	0	0	0	0	0
SAL_EA5782AA	CFSAN038533	Braenderup	0	0	0	0	0	0	0	0	0	0	0
SAL_EA5768AA	CFSAN038487	Braenderup	0	0	0	0	0	0	0	0	0	0	0
SAL_EA5780AA	CFSAN038535	Braenderup	0	0	0	0	0	0	0	0	0	0	0
SAL_EA5773AA	CFSAN038542	Braenderup	0	0	0	0	0	0	0	0	0	0	0
SAL_EA5775AA	CFSAN038540	Braenderup	0	0	0	0	0	0	0	0	0	0	0
SAL_EA5772AA	CFSAN038483	Braenderup	0	0	0	0	0	0	0	0	0	0	0
SAL_EA5776AA	CFSAN038539	Braenderup	0	0	0	0	0	0	0	0	0	0	0
SAL_EA5771AA	CFSAN038484	Braenderup	0	0	0	0	0	0	0	0	0	0	0
SAL_EA5770AA	CFSAN038485	Braenderup	0	0	0	0	0	0	0	0	0	0	0
SAL_EA5769AA	CFSAN038486	Braenderup	0	0	0	0	0	0	0	0	0	0	0
SAL_EA5765AA	CFSAN038490	Braenderup	0	0	0	0	0	0	0	0	0	0	0
SAL_EA5761AA	CFSAN038494	Braenderup	0	0	0	0	0	0	0	0	0	0	0
SAL_EA5767AA	CFSAN038488	Braenderup	0	0	0	0	0	0	0	0	0	0	0
SAL_EA5766AA	CFSAN038489	Braenderup	0	0	0	0	0	0	0	0	0	0	0
SAL_EA5763AA	CFSAN038492	Braenderup	0	0	0	0	0	0	0	0	0	0	0
SAL_EA5760AA	CFSAN038547	Braenderup	0	0	0	0	0	0	0	0	0	0	0
SAL_EA5764AA	CFSAN038491	Braenderup	0	0	0	0	0	0	0	0	0	0	0
SAL_EA5762AA	CFSAN038493	Braenderup	0	0	0	0	0	0	0	0	0	0	0
SAL_EA5774AA	CFSAN038541	Braenderup	0	0	0	0	0	0	0	0	0	0	0
SAL_EA5572AA	VA-WGS-00565	Braenderup	0	0	0	0	0	0	0	0	0	0	0
SAL_EA5532AA	MOD1-Lipp-081	Braenderup	0	0	0	0	0	0	0	0	0	0	0
SAL_EA5526AA	MOD1-Pga-1	Braenderup	0	0	0	0	0	0	0	0	0	0	0
SAL_EA5381AA	PNUSAS001238	Braenderup	0	0	0	0	0	0	0	0	0	0	0
SAL_EA5382AA	PNUSAS001237	Braenderup	0	0	0	0	0	0	0	0	0	0	0
SAL_EA5379AA	PNUSAS001239	Braenderup	0	0	0	0	0	0	0	0	0	0	0
SAL_EA5171AA	NY-N157	Braenderup	0	0	0	0	0	0	0	0	0	0	0
SAL_EA4894AA	CFSAN033398	Braenderup	0	0	0	0	0	0	0	0	0	0	0
SAL_EA4893AA	CFSAN033399	Braenderup	0	0	0	0	0	0	0	0	0	0	0
SAL_EA4641AA	CFSAN033240	Braenderup	0	0	0	0	0	0	0	0	0	0	0
SAL_EA4642AA	CFSAN033239	Braenderup	0	0	0	0	0	0	0	0	0	0	0
SAL_EA4597AA	CFSAN033236	Braenderup	0	0	0	0	0	0	0	0	0	0	0
SAL_EA4598AA	CFSAN033235	Braenderup	0	0	0	0	0	0	0	0	0	0	0
SAL_EA4583AA	CFSAN038545	Braenderup	0	0	0	0	0	0	0	0	0	0	0
SAL_EA4584AA	CFSAN038544	Braenderup	0	0	0	0	0	0	0	0	0	0	0
SAL_EA4244AA	VA-WGS-00568	Braenderup	0	0	0	0	0	0	0	0	0	0	0
SAL_EA4145AA	CFSAN036849	Braenderup	0	0	0	0	0	0	0	0	0	0	0
SAL_EA4142AA	CFSAN036852	Braenderup	0	0	0	0	0	0	0	0	0	0	0
SAL_EA4144AA	CFSAN036850	Braenderup	0	0	0	0	0	0	0	0	0	0	0
SAL_EA4140AA	CFSAN036854	Braenderup	0	0	0	0	0	0	0	0	0	0	0
SAL_EA4139AA	CFSAN036855	Braenderup	0	0	0	0	0	0	0	0	0	0	0
SAL_EA4138AA	CFSAN036856	Braenderup	0	0	0	0	0	0	0	0	0	0	0
SAL_EA4143AA	CFSAN036851	Braenderup	0	0	0	0	0	0	0	0	0	0	0
SAL_EA4141AA	CFSAN036853	Braenderup	0	0	0	0	0	0	0	0	0	0	0
SAL_EA4137AA	CFSAN036857	Braenderup	0	0	0	0	0	0	0	0	0	0	0
SAL_EA4136AA	CFSAN036858	Braenderup	0	0	0	0	0	0	0	0	0	0	0
SAL_EA4135AA	CFSAN036859	Braenderup	0	0	0	0	0	0	0	0	0	0	0
SAL_EA4076AA	CFSAN038546	Braenderup	0	0	0	0	0	0	0	0	0	0	0
SAL_EA4006AA	FDA00009437	Braenderup	0	0	0	0	0	0	0	0	0	0	0
SAL_EA3663AA	CVM N48701	Braenderup	0	0	0	0	0	0	0	0	0	0	0
SAL_EA3644AA	CVM N50427	Braenderup	0	0	0	0	0	0	0	0	0	0	0
SAL_EA3518AA	FDA00009436	Braenderup	0	0	0	0	0	0	0	0	0	0	0
SAL_EA3279AA	CFSAN038495	Braenderup	0	0	0	0	0	0	0	0	0	0	0
SAL_EA3274AA	CFSAN038497	Braenderup	0	0	0	0	0	0	0	0	0	0	0
SAL_EA3277AA	CFSAN038496	Braenderup	0	0	0	0	0	0	0	0	0	0	0
SAL_EA3272AA	CFSAN038498	Braenderup	0	0	0	0	0	0	0	0	0	0	0
SAL_EA3270AA	CFSAN038499	Braenderup	0	0	0	0	0	0	0	0	0	0	0
SAL_EA3269AA	CFSAN038500	Braenderup	0	0	0	0	0	0	0	0	0	0	0
SAL_EA3266AA	CFSAN038502	Braenderup	0	0	0	0	0	0	0	0	0	0	0
SAL_EA3268AA	CFSAN038501	Braenderup	0	0	0	0	0	0	0	0	0	0	0
SAL_EA3264AA	CFSAN038503	Braenderup	0	0	0	0	0	0	0	0	0	0	0
SAL_EA3263AA	CFSAN038505	Braenderup	0	0	0	0	0	0	0	0	0	0	0
SAL_EA3256AA	CFSAN035001	Braenderup	0	0	0	0	0	0	0	0	0	0	0
SAL_EA3223AA	FDA00007838	Braenderup	0	0	0	0	0	0	0	0	0	0	0
SAL_EA3222AA	FDA00008913	Braenderup	0	0	0	0	0	0	0	0	0	0	0
SAL_EA3201AA	CFSAN038522	Braenderup	0	0	0	0	0	0	0	0	0	0	0
SAL_EA3202AA	CFSAN036860	Braenderup	0	0	0	0	0	0	0	0	0	0	0
SAL_EA2678AA	CFSAN034205	Braenderup	0	0	0	0	0	0	0	0	0	0	0

SAL_EA2061AA	CVM N53042	Braenderup	0	0	0	0	0	0	0	0	0	0	0
SAL_EA1559AA	PNUSAS000677	Braenderup	0	0	0	0	0	0	0	0	0	0	0
SAL_EA1392AA	FL_UFL-SR9	Braenderup	0	0	0	0	0	0	0	0	0	0	0
SAL_EA1393AA	FL_UFL-SR8	Braenderup	0	0	0	0	0	0	0	0	0	0	0
SAL_EA1387AA	FL_UFL-SR14	Braenderup	0	0	0	0	0	0	0	0	0	0	0
SAL_EA0522AA	AZ_TG68511	Braenderup	0	0	0	0	0	0	0	0	0	0	0
SAL_EA0469AA	FL-UFLWL9	Braenderup	0	0	0	0	0	0	0	0	0	0	0
SAL_EA0379AA	FL-UFL-550	Braenderup	0	0	0	0	0	0	0	0	0	0	0
SAL_EA0358AA	FL-MJ601	Braenderup	0	0	0	0	0	0	0	0	0	0	0
SAL_EA0359AA	FL-MJ583	Braenderup	0	0	0	0	0	0	0	0	0	0	0
SAL_EA0343AA	FL-UFL-539	Braenderup	0	0	0	0	0	0	0	0	0	0	0
SAL_EA0322AA	FL-MJ576	Braenderup	0	0	0	0	0	0	0	0	0	0	0
SAL_EA0257AA	CFSAN005398	Braenderup	0	0	0	0	0	0	0	0	0	0	0
SAL_EA0256AA	CFSAN005399	Braenderup	0	0	0	0	0	0	0	0	0	0	0
SAL_EA0136AA	WAPHL_SAL-A00105	Braenderup	0	0	0	0	0	0	0	0	0	0	0
SAL_DA9648AA	CFSAN005394	Braenderup	0	0	0	0	0	0	0	0	0	0	0
SAL_DA9637AA	CFSAN005397	Braenderup	0	0	0	0	0	0	0	0	0	0	0
SAL_DA9633AA	CFSAN005395	Braenderup	0	0	0	0	0	0	0	0	0	0	0
SAL_DA8867AA	UFL-593	Braenderup	0	0	0	0	0	0	0	0	0	0	0
SAL_DA8863AA	UFL-839	Braenderup	0	0	0	0	0	0	0	0	0	0	0
SAL_DA8815AA	UFL-1029	Braenderup	0	0	0	0	0	0	0	0	0	0	0
SAL_DA8575AA	FSE0042	Braenderup	0	0	0	0	0	0	0	0	0	0	0
SAL_DA8440AA	fnw19J12	Braenderup	0	0	0	0	0	0	0	0	0	0	0
SAL_DA8415AA	fnw19J13	Braenderup	0	0	0	0	0	0	0	0	0	0	0
SAL_DA8405AA	fnw19J11	Braenderup	0	0	0	0	0	0	0	0	0	0	0
SAL_DA8228AA	3795-sc-2010-09-29-985	Braenderup	0	0	0	0	0	0	0	0	0	0	0
SAL_DA7787AA	FSW0152	Braenderup	0	0	0	0	0	0	0	0	0	0	0
SAL_DA7509AA	FLUFL-841	Braenderup	0	0	0	0	0	0	0	0	0	0	0
SAL_DA7127AA	FL-NRM001	Braenderup	0	0	0	0	0	0	0	0	0	0	0
SAL_DA7097AA	FL-NRM004	Braenderup	0	0	0	0	0	0	0	0	0	0	0
SAL_DA7092AA	FL-NRM002	Braenderup	0	0	0	0	0	0	0	0	0	0	0
SAL_DA7100AA	FL-NRM003	Braenderup	0	0	0	0	0	0	0	0	0	0	0
SAL_DA7083AA	FL-NRM007	Braenderup	0	0	0	0	0	0	0	0	0	0	0
SAL_DA7072AA	FL-NRM005	Braenderup	0	0	0	0	0	0	0	0	0	0	0
SAL_DA7071AA	FL-NRM006	Braenderup	0	0	0	0	0	0	0	0	0	0	0
SAL_DA6166AA	FLDOH-28	Braenderup	0	0	0	0	0	0	0	0	0	0	0
SAL_DA5693AA	FLUFL-1031	Braenderup	0	0	0	0	0	0	0	0	0	0	0
SAL_DA5649AA	CFSAN018653	Braenderup	0	0	0	0	0	0	0	0	0	0	0
SAL_DA5583AA	CFSAN018680	Braenderup	0	0	0	0	0	0	0	0	0	0	0
SAL_DA5466AA	FDA00004352	Braenderup	0	0	0	0	0	0	0	0	0	0	0
SAL_DA5111AA	NY_IDR1200029858	Braenderup	0	0	0	0	0	0	0	0	0	0	0
SAL_DA3990AA	CFSAN023152	Braenderup	0	0	0	0	0	0	0	0	0	0	0
SAL_DA3774AA	2012K-1324	Braenderup	0	0	0	0	0	0	0	0	0	0	0
SAL_DA3772AA	2012K-1326	Braenderup	0	0	0	0	0	0	0	0	0	0	0
SAL_DA3773AA	2012K-1325	Braenderup	0	0	0	0	0	0	0	0	0	0	0
SAL_DA3775AA	2012K-1316	Braenderup	0	0	0	0	0	0	0	0	0	0	0
SAL_DA3771AA	2012K-1327	Braenderup	0	0	0	0	0	0	0	0	0	0	0
SAL_DA3770AA	2012K-1328	Braenderup	0	0	0	0	0	0	0	0	0	0	0
SAL_DA3769AA	2014K-0290	Braenderup	0	0	0	0	0	0	0	0	0	0	0
SAL_DA3768AA	2014K-0314	Braenderup	0	0	0	0	0	0	0	0	0	0	0
SAL_DA3767AA	2014K-0603	Braenderup	0	0	0	0	0	0	0	0	0	0	0
SAL_DA3766AA	2014K-0605	Braenderup	0	0	0	0	0	0	0	0	0	0	0
SAL_DA3765AA	2014K-0606	Braenderup	0	0	0	0	0	0	0	0	0	0	0
SAL_DA3267AA	2014K-0675	Braenderup	0	0	0	0	0	0	0	0	0	0	0
SAL_DA3268AA	2014K-0614	Braenderup	0	0	0	0	0	0	0	0	0	0	0
SAL_DA3125AA	CFSAN024176	Braenderup	0	0	0	0	0	0	0	0	0	0	0
SAL_DA3112AA	CFSAN024173	Braenderup	0	0	0	0	0	0	0	0	0	0	0
SAL_DA3090AA	CFSAN024174	Braenderup	0	0	0	0	0	0	0	0	0	0	0
SAL_DA2991AA	CFSAN005396	Braenderup	0	0	0	0	0	0	0	0	0	0	0
SAL_DA1756AA	FDA00002023	Braenderup	0	0	0	0	0	0	0	0	0	0	0
SAL_DA1755AA	FDA00002024	Braenderup	0	0	0	0	0	0	0	0	0	0	0
SAL_DA0976AA	H125140348	Braenderup	0	0	0	0	0	0	0	0	0	0	0
SAL_DA0884AA	H123820533	Braenderup	0	0	0	0	0	0	0	0	0	0	0
SAL_DA0512AA	H123760508	Braenderup	0	0	0	0	0	0	0	0	0	0	0
SAL_DA0387AA	H124860457	Braenderup	0	0	0	0	0	0	0	0	0	0	0
SAL_DA0205AA	FLUFL-595	Braenderup	0	0	0	0	0	0	0	0	0	0	0
SAL_DA0201AA	FLUFL-599	Braenderup	0	0	0	0	0	0	0	0	0	0	0
SAL_DA0188AA	FLUFL-594	Braenderup	0	0	0	0	0	0	0	0	0	0	0
SAL_DA0170AA	PNUSAS000064	Braenderup	0	0	0	0	0	0	0	0	0	0	0
SAL_CA9959AA	FLUFL-704	Braenderup	0	0	0	0	0	0	0	0	0	0	0
SAL_CA9949AA	FLUFL-703	Braenderup	0	0	0	0	0	0	0	0	0	0	0
SAL_CA9733AA	2014K-0952	Braenderup	0	0	0	0	0	0	0	0	0	0	0
SAL_CA9740AA	2014K-0930	Braenderup	0	0	0	0	0	0	0	0	0	0	0

SAL_CA9742AA	2014K-0928	Braenderup	0	0	0	0	0	0	0	0	0	0	0
SAL_CA9732AA	2014K-0953	Braenderup	0	0	0	0	0	0	0	0	0	0	0
SAL_CA9731AA	2014K-0954	Braenderup	0	0	0	0	0	0	0	0	0	0	0
SAL_CA9635AA	VA-WGS-00381	Braenderup	0	0	0	0	0	0	0	0	0	0	0
SAL_CA9615AA	VA-WGS-00380	Braenderup	0	0	0	0	0	0	0	0	0	0	0
SAL_CA9536AA	CFSAN027110	Braenderup	0	0	0	0	0	0	0	0	0	0	0
SAL_CA9400AA	2014K-0955	Braenderup	0	0	0	0	0	0	0	0	0	0	0
SAL_CA9399AA	2014K-0956	Braenderup	0	0	0	0	0	0	0	0	0	0	0
SAL_CA9286AA	CFSAN024818	Braenderup	0	0	0	0	0	0	0	0	0	0	0
SAL_CA9283AA	CFSAN025690	Braenderup	0	0	0	0	0	0	0	0	0	0	0
SAL_CA9280AA	CFSAN025706	Braenderup	0	0	0	0	0	0	0	0	0	0	0
SAL_CA8907AA	CFSAN024811	Braenderup	0	0	0	0	0	0	0	0	0	0	0
SAL_CA8902AA	CFSAN024807	Braenderup	0	0	0	0	0	0	0	0	0	0	0
SAL_CA8894AA	CFSAN024817	Braenderup	0	0	0	0	0	0	0	0	0	0	0
SAL_CA8884AA	CFSAN024828	Braenderup	0	0	0	0	0	0	0	0	0	0	0
SAL_CA8873AA	CFSAN024806	Braenderup	0	0	0	0	0	0	0	0	0	0	0
SAL_CA8858AA	CFSAN024815	Braenderup	0	0	0	0	0	0	0	0	0	0	0
SAL_CA8646AA	IEH-NGS-SAL-01824	Braenderup	0	0	0	0	0	0	0	0	0	0	0
SAL_CA8504AA	CFSAN028517	Braenderup	0	0	0	0	0	0	0	0	0	0	0
SAL_CA8341AA	NY-N6	Braenderup	0	0	0	0	0	0	0	0	0	0	0
SAL_CA8154AA	CFSAN028042	Braenderup	0	0	0	0	0	0	0	0	0	0	0
SAL_CA7935AA	FDA00008820	Braenderup	0	0	0	0	0	0	0	0	0	0	0
SAL_CA7805AA	IEH-NGS-SAL-02007	Braenderup	0	0	0	0	0	0	0	0	0	0	0
SAL_CA7773AA	NY-N158	Braenderup	0	0	0	0	0	0	0	0	0	0	0
SAL_CA7774AA	NY-N159	Braenderup	0	0	0	0	0	0	0	0	0	0	0
SAL_CA7543AA	CFSAN027384	Braenderup	0	0	0	0	0	0	0	0	0	0	0
SAL_CA7506AA	CFSAN027382	Braenderup	0	0	0	0	0	0	0	0	0	0	0
SAL_CA7507AA	CFSAN027381	Braenderup	0	0	0	0	0	0	0	0	0	0	0
SAL_CA7258AA	CFSAN030119	Braenderup	0	0	0	0	0	0	0	0	0	0	0
SAL_CA7106AA	CFSAN030191	Braenderup	0	0	0	0	0	0	0	0	0	0	0
SAL_CA6784AA	44729	Braenderup	0	0	0	0	0	0	0	0	0	0	0
SAL_CA6444AA	50105	Braenderup	0	0	0	0	0	0	0	0	0	0	0
SAL_CA6397AA	62618	Braenderup	0	0	0	0	0	0	0	0	0	0	0
SAL_CA6346AA	49705	Braenderup	0	0	0	0	0	0	0	0	0	0	0
SAL_CA6348AA	62571	Braenderup	0	0	0	0	0	0	0	0	0	0	0
SAL_CA6312AA	48276	Braenderup	0	0	0	0	0	0	0	0	0	0	0
SAL_CA6228AA	48236	Braenderup	0	0	0	0	0	0	0	0	0	0	0
SAL_CA6227AA	57673	Braenderup	0	0	0	0	0	0	0	0	0	0	0
SAL_CA6062AA	48275	Braenderup	0	0	0	0	0	0	0	0	0	0	0
SAL_CA5890AA	59990	Braenderup	0	0	0	0	0	0	0	0	0	0	0
SAL_CA5792AA	80297	Braenderup	0	0	0	0	0	0	0	0	0	0	0
SAL_CA5770AA	78642	Braenderup	0	0	0	0	0	0	0	0	0	0	0
SAL_CA5712AA	69692	Braenderup	0	0	0	0	0	0	0	0	0	0	0
SAL_CA5536AA	69724	Braenderup	0	0	0	0	0	0	0	0	0	0	0
SAL_CA5486AA	73642	Braenderup	0	0	0	0	0	0	0	0	0	0	0
SAL_CA5458AA	78761	Braenderup	0	0	0	0	0	0	0	0	0	0	0
SAL_CA5418AA	PNUSAS000212	Braenderup	0	0	0	0	0	0	0	0	0	0	0
SAL_CA5184AA	75754	Braenderup	0	0	0	0	0	0	0	0	0	0	0
SAL_CA5085AA	79061	Braenderup	0	0	0	0	0	0	0	0	0	0	0
SAL_CA4996AA	73562	Braenderup	0	0	0	0	0	0	0	0	0	0	0
SAL_CA4846AA	14478	Braenderup	0	0	0	0	0	0	0	0	0	0	0
SAL_CA4769AA	32383	Braenderup	0	0	0	0	0	0	0	0	0	0	0
SAL_CA4525AA	13373	Braenderup	0	0	0	0	0	0	0	0	0	0	0
SAL_CA4489AA	51280	Braenderup	0	0	0	0	0	0	0	0	0	0	0
SAL_CA4479AA	13855	Braenderup	0	0	0	0	0	0	0	0	0	0	0
SAL_CA4460AA	14479	Braenderup	0	0	0	0	0	0	0	0	0	0	0
SAL_CA4456AA	45581	Braenderup	0	0	0	0	0	0	0	0	0	0	0
SAL_CA4439AA	52997	Braenderup	0	0	0	0	0	0	0	0	0	0	0
SAL_CA4389AA	51244	Braenderup	0	0	0	0	0	0	0	0	0	0	0
SAL_CA4379AA	53010	Braenderup	0	0	0	0	0	0	0	0	0	0	0
SAL_CA4371AA	14502	Braenderup	0	0	0	0	0	0	0	0	0	0	0
SAL_CA4366AA	5905	Braenderup	0	0	0	0	0	0	0	0	0	0	0
SAL_CA4306AA	91284	Braenderup	0	0	0	0	0	0	0	0	0	0	0
SAL_CA4211AA	24045	Braenderup	0	0	0	0	0	0	0	0	0	0	0
SAL_CA4143AA	50340	Braenderup	0	0	0	0	0	0	0	0	0	0	0
SAL_CA4031AA	63745	Braenderup	0	0	0	0	0	0	0	0	0	0	0
SAL_CA3966AA	26669	Braenderup	0	0	0	0	0	0	0	0	0	0	0
SAL_CA3946AA	38725	Braenderup	0	0	0	0	0	0	0	0	0	0	0
SAL_CA3894AA	57119	Braenderup	0	0	0	0	0	0	0	0	0	0	0
SAL_CA3850AA	57132	Braenderup	0	0	0	0	0	0	0	0	0	0	0
SAL_CA3835AA	55545	Braenderup	0	0	0	0	0	0	0	0	0	0	0
SAL_CA3677AA	37021	Braenderup	0	0	0	0	0	0	0	0	0	0	0
SAL_CA3675AA	3751	Braenderup	0	0	0	0	0	0	0	0	0	0	0

SAL_CA3358AA	13822	Braenderup	0	0	0	0	0	0	0	0	0	0	0
SAL_CA3264AA	5370	Braenderup	0	0	0	0	0	0	0	0	0	0	0
SAL_CA3239AA	5966	Braenderup	0	0	0	0	0	0	0	0	0	0	0
SAL_CA3164AA	13352	Braenderup	0	0	0	0	0	0	0	0	0	0	0
SAL_CA3057AA	9162	Braenderup	0	0	0	0	0	0	0	0	0	0	0
SAL_CA2981AA	5834	Braenderup	0	0	0	0	0	0	0	0	0	0	0
SAL_CA2931AA	9277	Braenderup	0	0	0	0	0	0	0	0	0	0	0
SAL_CA2805AA	67554	Braenderup	0	0	0	0	0	0	0	0	0	0	0
SAL_CA2677AA	84425	Braenderup	0	0	0	0	0	0	0	0	0	0	0
SAL_CA2517AA	36984	Braenderup	0	0	0	0	0	0	0	0	0	0	0
SAL_CA2477AA	72288	Braenderup	0	0	0	0	0	0	0	0	0	0	0
SAL_CA2437AA	9458	Braenderup	0	0	0	0	0	0	0	0	0	0	0
SAL_CA2454AA	84431	Braenderup	0	0	0	0	0	0	0	0	0	0	0
SAL_CA2336AA	57124	Braenderup	0	0	0	0	0	0	0	0	0	0	0
SAL_CA2302AA	91245	Braenderup	0	0	0	0	0	0	0	0	0	0	0
SAL_CA2266AA	99767	Braenderup	0	0	0	0	0	0	0	0	0	0	0
SAL_CA2147AA	93546	Braenderup	0	0	0	0	0	0	0	0	0	0	0
SAL_CA2103AA	90491	Braenderup	0	0	0	0	0	0	0	0	0	0	0
SAL_CA1469AA	91257	Braenderup	0	0	0	0	0	0	0	0	0	0	0
SAL_CA1347AA	39466	Braenderup	0	0	0	0	0	0	0	0	0	0	0
SAL_CA1178AA	65007	Braenderup	0	0	0	0	0	0	0	0	0	0	0
SAL_CA1108AA	96934	Braenderup	0	0	0	0	0	0	0	0	0	0	0
SAL_CA0916AA	63701	Braenderup	0	0	0	0	0	0	0	0	0	0	0
SAL_CA0892AA	24035	Braenderup	0	0	0	0	0	0	0	0	0	0	0
SAL_CA0522AA	61852	Braenderup	0	0	0	0	0	0	0	0	0	0	0
SAL_CA0470AA	7385	Braenderup	0	0	0	0	0	0	0	0	0	0	0
SAL_CA0453AA	63768	Braenderup	0	0	0	0	0	0	0	0	0	0	0
SAL_CA0380AA	12165	Braenderup	0	0	0	0	0	0	0	0	0	0	0
SAL_CA0238AA	14477	Braenderup	0	0	0	0	0	0	0	0	0	0	0
SAL_CA0218AA	83696	Braenderup	0	0	0	0	0	0	0	0	0	0	0
SAL_CA0083AA	26653	Braenderup	0	0	0	0	0	0	0	0	0	0	0
SAL_BA9980AA	38394	Braenderup	0	0	0	0	0	0	0	0	0	0	0
SAL_BA9971AA	:-2012-07-27T11:52:43Z-	Braenderup	0	0	0	0	0	0	0	0	0	0	0
SAL_BA9968AA	13820	Braenderup	0	0	0	0	0	0	0	0	0	0	0
SAL_BA9975AA	:-2012-07-27T11:52:41Z-	Braenderup	0	0	0	0	0	0	0	0	0	0	0
SAL_BA9924AA	:-2012-07-27T11:53:00Z-	Braenderup	0	0	0	0	0	0	0	0	0	0	0
SAL_BA9918AA	:-2012-07-27T11:53:02Z-	Braenderup	0	0	0	0	0	0	0	0	0	0	0
SAL_BA9879AA	:-2012-07-27T11:53:20Z-	Braenderup	0	0	0	0	0	0	0	0	0	0	0
SAL_BA9459AA	9459	Braenderup	0	0	0	0	0	0	0	0	0	0	0
SAL_BA9418AA	65438	Braenderup	0	0	0	0	0	0	0	0	0	0	0
SAL_BA9413AA	56122	Braenderup	0	0	0	0	0	0	0	0	0	0	0
SAL_BA9408AA	51294	Braenderup	0	0	0	0	0	0	0	0	0	0	0
SAL_BA9322AA	40347	Braenderup	0	0	0	0	0	0	0	0	0	0	0
SAL_BA9286AA	38335	Braenderup	0	0	0	0	0	0	0	0	0	0	0
SAL_BA9194AA	21772	Braenderup	0	0	0	0	0	0	0	0	0	0	0
SAL_BA9140AA	27785	Braenderup	0	0	0	0	0	0	0	0	0	0	0
SAL_BA8957AA	55541	Braenderup	0	0	0	0	0	0	0	0	0	0	0
SAL_BA8791AA	63635	Braenderup	0	0	0	0	0	0	0	0	0	0	0
SAL_BA8769AA	83348	Braenderup	0	0	0	0	0	0	0	0	0	0	0
SAL_BA8715AA	NY_FSL S10-939	Braenderup	0	0	0	0	0	0	0	0	0	0	0
SAL_BA8566AA	63615	Braenderup	0	0	0	0	0	0	0	0	0	0	0
SAL_BA8466AA	55570	Braenderup	0	0	0	0	0	0	0	0	0	0	0
SAL_BA8422AA	45575	Braenderup	0	0	0	0	0	0	0	0	0	0	0
SAL_BA8206AA	CFSAN005735	Braenderup	0	0	0	0	0	0	0	0	0	0	0
SAL_BA8120AA	91970	Braenderup	0	0	0	0	0	0	0	0	0	0	0
SAL_BA8004AA	0000-0020-7803	Braenderup	0	0	0	0	0	0	0	0	0	0	0
SAL_BA7889AA	9294	Braenderup	0	0	0	0	0	0	0	0	0	0	0
SAL_BA7851AA	5828	Braenderup	0	0	0	0	0	0	0	0	0	0	0
SAL_BA7566AA	51288	Braenderup	0	0	0	0	0	0	0	0	0	0	0
SAL_BA7375AA	CFSAN006206	Braenderup	0	0	0	0	0	0	0	0	0	0	0
SAL_BA7385AA	CFSAN006223	Braenderup	0	0	0	0	0	0	0	0	0	0	0
SAL_BA7346AA	CFSAN006197	Braenderup	0	0	0	0	0	0	0	0	0	0	0
SAL_BA7214AA	CFSAN030247	Braenderup	0	0	0	0	0	0	0	0	0	0	0
SAL_BA6976AA	CFSAN030280	Braenderup	0	0	0	0	0	0	0	0	0	0	0
SAL_BA6916AA	CFSAN030245	Braenderup	0	0	0	0	0	0	0	0	0	0	0
SAL_BA6855AA	CFSAN006214	Braenderup	0	0	0	0	0	0	0	0	0	0	0
SAL_BA6820AA	CFSAN030249	Braenderup	0	0	0	0	0	0	0	0	0	0	0
SAL_BA6822AA	FDA00002375	Braenderup	0	0	0	0	0	0	0	0	0	0	0
SAL_BA6755AA	CFSAN006217	Braenderup	0	0	0	0	0	0	0	0	0	0	0
SAL_BA6655AA	211903	Braenderup	0	0	0	0	0	0	0	0	0	0	0
SAL_BA6649AA	1055673	Braenderup	0	0	0	0	0	0	0	0	0	0	0
SAL_BA6635AA	CFSAN030728	Braenderup	0	0	0	0	0	0	0	0	0	0	0
SAL_BA6377AA	09:MF0009695R	Braenderup	0	0	0	0	0	0	0	0	0	0	0

SAL_BA6241AA	CFSAN031100	Braenderup	0	0	0	0	0	0	0	0	0	0	0
SAL_BA6237AA	CFSAN031103	Braenderup	0	0	0	0	0	0	0	0	0	0	0
SAL_BA6224AA	CFSAN031098	Braenderup	0	0	0	0	0	0	0	0	0	0	0
SAL_BA6084AA	FLUFL-C4.2	Braenderup	0	0	0	0	0	0	0	0	0	0	0
SAL_BA5853AA	FLUFL-04E01347	Braenderup	0	0	0	0	0	0	0	0	0	0	0
SAL_BA5817AA	FLUFL-C6.3	Braenderup	0	0	0	0	0	0	0	0	0	0	0
SAL_BA5757AA	CFSAN031140	Braenderup	0	0	0	0	0	0	0	0	0	0	0
SAL_BA5509AA	CFSAN029926	Braenderup	0	0	0	0	0	0	0	0	0	0	0
SAL_BA5496AA	CFSAN031294	Braenderup	0	0	0	0	0	0	0	0	0	0	0
SAL_BA5490AA	CFSAN031301	Braenderup	0	0	0	0	0	0	0	0	0	0	0
SAL_BA5491AA	CFSAN031300	Braenderup	0	0	0	0	0	0	0	0	0	0	0
SAL_BA5130AA	CFSAN002757	Braenderup	0	0	0	0	0	0	0	0	0	0	0
SAL_BA5132AA	CFSAN002756	Braenderup	0	0	0	0	0	0	0	0	0	0	0
SAL_BA5104AA	CFSAN005653	Braenderup	0	0	0	0	0	0	0	0	0	0	0
SAL_BA4937AA	CFSAN031165	Braenderup	0	0	0	0	0	0	0	0	0	0	0
SAL_BA4769AA	IEH-NGS-SAL-02655	Braenderup	0	0	0	0	0	0	0	0	0	0	0
SAL_BA4706AA	CFSAN005661	Braenderup	0	0	0	0	0	0	0	0	0	0	0
SAL_BA4656AA	CFSAN006248	Braenderup	0	0	0	0	0	0	0	0	0	0	0
SAL_BA4645AA	CFSAN005392	Braenderup	0	0	0	0	0	0	0	0	0	0	0
SAL_BA4643AA	CFSAN005393	Braenderup	0	0	0	0	0	0	0	0	0	0	0
SAL_BA4588AA	CFSAN029924	Braenderup	0	0	0	0	0	0	0	0	0	0	0
SAL_BA4570AA	CFSAN029925	Braenderup	0	0	0	0	0	0	0	0	0	0	0
SAL_BA4420AA	CFSAN031246	Braenderup	0	0	0	0	0	0	0	0	0	0	0
SAL_BA4330AA	CFSAN032315	Braenderup	0	0	0	0	0	0	0	0	0	0	0
SAL_BA4276AA	CFSAN032320	Braenderup	0	0	0	0	0	0	0	0	0	0	0
SAL_BA4138AA	IEH-NGS-SAL-02791	Braenderup	0	0	0	0	0	0	0	0	0	0	0
SAL_BA4134AA	IEH-NGS-SAL-02796	Braenderup	0	0	0	0	0	0	0	0	0	0	0
SAL_BA4088AA	CFSAN032361	Braenderup	0	0	0	0	0	0	0	0	0	0	0
SAL_BA3969AA	CFSAN032537	Braenderup	0	0	0	0	0	0	0	0	0	0	0
SAL_BA3943AA	CFSAN032551	Braenderup	0	0	0	0	0	0	0	0	0	0	0
SAL_BA3935AA	CFSAN032555	Braenderup	0	0	0	0	0	0	0	0	0	0	0
SAL_BA3915AA	IEH-NGS-SAL-02852	Braenderup	0	0	0	0	0	0	0	0	0	0	0
SAL_BA3897AA	CFSAN032574	Braenderup	0	0	0	0	0	0	0	0	0	0	0
SAL_BA3893AA	IEH-NGS-SAL-02862	Braenderup	0	0	0	0	0	0	0	0	0	0	0
SAL_BA3889AA	IEH-NGS-SAL-02864	Braenderup	0	0	0	0	0	0	0	0	0	0	0
SAL_BA3727AA	2012K-1528	Braenderup	0	0	0	0	0	0	0	0	0	0	0
SAL_BA3725AA	2013K-0742	Braenderup	0	0	0	0	0	0	0	0	0	0	0
SAL_BA3564AA	CFSAN006250	Braenderup	0	0	0	0	0	0	0	0	0	0	0
SAL_BA3535AA	CFSAN029993	Braenderup	0	0	0	0	0	0	0	0	0	0	0
SAL_BA3226AA	PNUSAS000447	Braenderup	0	0	0	0	0	0	0	0	0	0	0
SAL_BA2989AA	AZ_TG68128	Braenderup	0	0	0	0	0	0	0	0	0	0	0
SAL_BA2975AA	AZ_TG68176	Braenderup	0	0	0	0	0	0	0	0	0	0	0
SAL_BA2940AA	CFSAN030846	Braenderup	0	0	0	0	0	0	0	0	0	0	0
SAL_BA2936AA	CFSAN030492	Braenderup	0	0	0	0	0	0	0	0	0	0	0
SAL_BA2930AA	CFSAN030498	Braenderup	0	0	0	0	0	0	0	0	0	0	0
SAL_BA2926AA	CFSAN030824	Braenderup	0	0	0	0	0	0	0	0	0	0	0
SAL_BA2912AA	CFSAN030635	Braenderup	0	0	0	0	0	0	0	0	0	0	0
SAL_BA2901AA	CFSAN030637	Braenderup	0	0	0	0	0	0	0	0	0	0	0
SAL_BA2902AA	CFSAN030502	Braenderup	0	0	0	0	0	0	0	0	0	0	0
SAL_BA2893AA	CFSAN030093	Braenderup	0	0	0	0	0	0	0	0	0	0	0
SAL_BA2892AA	CFSAN030493	Braenderup	0	0	0	0	0	0	0	0	0	0	0
SAL_BA2873AA	CFSAN030834	Braenderup	0	0	0	0	0	0	0	0	0	0	0
SAL_BA2869AA	CFSAN030641	Braenderup	0	0	0	0	0	0	0	0	0	0	0
SAL_BA2880AA	CFSAN030854	Braenderup	0	0	0	0	0	0	0	0	0	0	0
SAL_BA2872AA	CFSAN030840	Braenderup	0	0	0	0	0	0	0	0	0	0	0
SAL_BA2861AA	CFSAN030830	Braenderup	0	0	0	0	0	0	0	0	0	0	0
SAL_BA2857AA	CFSAN030095	Braenderup	0	0	0	0	0	0	0	0	0	0	0
SAL_BA2852AA	CFSAN030853	Braenderup	0	0	0	0	0	0	0	0	0	0	0
SAL_BA2847AA	CFSAN030487	Braenderup	0	0	0	0	0	0	0	0	0	0	0
SAL_BA2837AA	CFSAN032972	Braenderup	0	0	0	0	0	0	0	0	0	0	0
SAL_BA2805AA	CFSAN030640	Braenderup	0	0	0	0	0	0	0	0	0	0	0
SAL_BA2763AA	CFSAN030638	Braenderup	0	0	0	0	0	0	0	0	0	0	0
SAL_BA2752AA	CFSAN030099	Braenderup	0	0	0	0	0	0	0	0	0	0	0
SAL_BA2252AA	FDA00004464	Braenderup	0	0	0	0	0	0	0	0	0	0	0
SAL_BA2184AA	CFSAN034145	Braenderup	0	0	0	0	0	0	0	0	0	0	0
SAL_BA2179AA	CFSAN034143	Braenderup	0	0	0	0	0	0	0	0	0	0	0
SAL_BA2013AA	ADRDL-14-9169	Braenderup	0	0	0	0	0	0	0	0	0	0	0
SAL_BA1737AA	CFSAN034229	Braenderup	0	0	0	0	0	0	0	0	0	0	0
SAL_BA1744AA	CFSAN034216	Braenderup	0	0	0	0	0	0	0	0	0	0	0
SAL_BA1705AA	CFSAN034218	Braenderup	0	0	0	0	0	0	0	0	0	0	0
SAL_BA1696AA	CFSAN034215	Braenderup	0	0	0	0	0	0	0	0	0	0	0
SAL_BA1658AA	CFSAN034213	Braenderup	0	0	0	0	0	0	0	0	0	0	0
SAL_BA1659AA	CFSAN030076	Braenderup	0	0	0	0	0	0	0	0	0	0	0

SAL_BA1618AA	CFSAN034219	Braenderup	0	0	0	0	0	0	0	0	0	0	0
SAL_BA1623AA	CFSAN030073	Braenderup	0	0	0	0	0	0	0	0	0	0	0
SAL_BA1604AA	CFSAN035394	Braenderup	0	0	0	0	0	0	0	0	0	0	0
SAL_BA1601AA	CFSAN035389	Braenderup	0	0	0	0	0	0	0	0	0	0	0
SAL_BA1593AA	CFSAN035393	Braenderup	0	0	0	0	0	0	0	0	0	0	0
SAL_BA1596AA	FDA00003343	Braenderup	0	0	0	0	0	0	0	0	0	0	0
SAL_BA1592AA	CFSAN035399	Braenderup	0	0	0	0	0	0	0	0	0	0	0
SAL_BA1471AA	FSIS1503214	Braenderup	0	0	0	0	0	0	0	0	0	0	0
SAL_BA1244AA	CFSAN035277	Braenderup	0	0	0	0	0	0	0	0	0	0	0
SAL_BA1182AA	CFSAN035397	Braenderup	0	0	0	0	0	0	0	0	0	0	0
SAL_BA1177AA	CFSAN035068	Braenderup	0	0	0	0	0	0	0	0	0	0	0
SAL_BA1174AA	CFSAN035398	Braenderup	0	0	0	0	0	0	0	0	0	0	0
SAL_BA1166AA	CFSAN035396	Braenderup	0	0	0	0	0	0	0	0	0	0	0
SAL_BA1153AA	CFSAN035390	Braenderup	0	0	0	0	0	0	0	0	0	0	0
SAL_BA1150AA	CFSAN035391	Braenderup	0	0	0	0	0	0	0	0	0	0	0
SAL_BA1151AA	CFSAN035395	Braenderup	0	0	0	0	0	0	0	0	0	0	0
SAL_BA1152AA	CFSAN035392	Braenderup	0	0	0	0	0	0	0	0	0	0	0
SAL_BA1059AA	CFSAN035306	Braenderup	0	0	0	0	0	0	0	0	0	0	0
SAL_BA1047AA	CFSAN035310	Braenderup	0	0	0	0	0	0	0	0	0	0	0
SAL_BA0914AA	CFSAN035304	Braenderup	0	0	0	0	0	0	0	0	0	0	0
SAL_BA0880AA	CFSAN035294	Braenderup	0	0	0	0	0	0	0	0	0	0	0
SAL_BA0850AA	CFSAN035303	Braenderup	0	0	0	0	0	0	0	0	0	0	0
SAL_AA7197AA	0000-0021-0884	Braenderup	0	0	0	0	0	0	0	0	0	0	0
SAL_AA7148AA	PNUSAS000624	Braenderup	0	0	0	0	0	0	0	0	0	0	0
SAL_AA7147AA	PNUSAS000625	Braenderup	0	0	0	0	0	0	0	0	0	0	0
SAL_AA7144AA	PNUSAS000626	Braenderup	0	0	0	0	0	0	0	0	0	0	0
SAL_FA6024AA	PNUSAS002029	Braenderup	0	0	0	0	0	0	0	0	0	0	0
SAL_FA6397AA	CVM-N27388	Braenderup	0	0	0	0	0	0	0	0	0	0	0
SAL_FA6356AA	CFSAN045611	Braenderup	0	0	0	0	0	0	0	0	0	0	0
SAL_FA6355AA	CFSAN045612	Braenderup	0	0	0	0	0	0	0	0	0	0	0
SAL_FA6598AA	PNUSAS002137	Braenderup	0	0	0	0	0	0	0	0	0	0	0
SAL_FA6766AA	2013K-1015	Braenderup	0	0	0	0	0	0	0	0	0	0	0
SAL_FA7145AA	PNUSAS002198	Braenderup	0	0	0	0	0	0	0	0	0	0	0
SAL_FA7366AA	PNUSAS002265	Braenderup	0	0	0	0	0	0	0	0	0	0	0
SAL_FA7560AA	235029	Braenderup	0	0	0	0	0	0	0	0	0	0	0
SAL_FA7559AA	243858	Braenderup	0	0	0	0	0	0	0	0	0	0	0
SAL_FA7557AA	240171	Braenderup	0	0	0	0	0	0	0	0	0	0	0
SAL_FA7558AA	246188	Braenderup	0	0	0	0	0	0	0	0	0	0	0
SAL_FA7556AA	256119	Braenderup	0	0	0	0	0	0	0	0	0	0	0
SAL_FA7555AA	235042	Braenderup	0	0	0	0	0	0	0	0	0	0	0
SAL_FA7554AA	235025	Braenderup	0	0	0	0	0	0	0	0	0	0	0
SAL_FA7553AA	240209	Braenderup	0	0	0	0	0	0	0	0	0	0	0
SAL_FA7552AA	235030	Braenderup	0	0	0	0	0	0	0	0	0	0	0
SAL_FA7551AA	238711	Braenderup	0	0	0	0	0	0	0	0	0	0	0
SAL_FA7550AA	236243	Braenderup	0	0	0	0	0	0	0	0	0	0	0
SAL_FA7549AA	238463	Braenderup	0	0	0	0	0	0	0	0	0	0	0
SAL_FA7548AA	241834	Braenderup	0	0	0	0	0	0	0	0	0	0	0
SAL_FA7547AA	255584	Braenderup	0	0	0	0	0	0	0	0	0	0	0
SAL_FA7546AA	229868	Braenderup	0	0	0	0	0	0	0	0	0	0	0
SAL_FA7545AA	242401	Braenderup	0	0	0	0	0	0	0	0	0	0	0
SAL_FA7544AA	232738	Braenderup	0	0	0	0	0	0	0	0	0	0	0
SAL_FA7542AA	240148	Braenderup	0	0	0	0	0	0	0	0	0	0	0
SAL_FA7541AA	232085	Braenderup	0	0	0	0	0	0	0	0	0	0	0
SAL_FA7540AA	240152	Braenderup	0	0	0	0	0	0	0	0	0	0	0
SAL_FA7543AA	240149	Braenderup	0	0	0	0	0	0	0	0	0	0	0
SAL_FA7539AA	227959	Braenderup	0	0	0	0	0	0	0	0	0	0	0
SAL_FA7530AA	240158	Braenderup	0	0	0	0	0	0	0	0	0	0	0
SAL_FA7538AA	241824	Braenderup	0	0	0	0	0	0	0	0	0	0	0
SAL_FA7536AA	231112	Braenderup	0	0	0	0	0	0	0	0	0	0	0
SAL_FA7537AA	229845	Braenderup	0	0	0	0	0	0	0	0	0	0	0
SAL_FA7529AA	236212	Braenderup	0	0	0	0	0	0	0	0	0	0	0
SAL_FA7528AA	236268	Braenderup	0	0	0	0	0	0	0	0	0	0	0
SAL_FA7526AA	235028	Braenderup	0	0	0	0	0	0	0	0	0	0	0
SAL_FA7535AA	235045	Braenderup	0	0	0	0	0	0	0	0	0	0	0
SAL_FA7527AA	229073	Braenderup	0	0	0	0	0	0	0	0	0	0	0
SAL_FA7525AA	231100	Braenderup	0	0	0	0	0	0	0	0	0	0	0
SAL_FA7534AA	240178	Braenderup	0	0	0	0	0	0	0	0	0	0	0
SAL_FA7531AA	238375	Braenderup	0	0	0	0	0	0	0	0	0	0	0
SAL_FA7533AA	245606	Braenderup	0	0	0	0	0	0	0	0	0	0	0
SAL_FA7507AA	CFSAN032616	Braenderup	0	0	0	0	0	0	0	0	0	0	0
SAL_FA7464AA	CFSAN032647	Braenderup	0	0	0	0	0	0	0	0	0	0	0
SAL_FA7761AA	PNUSAS002349	Braenderup	0	0	0	0	0	0	0	0	0	0	0
SAL_FA7750AA	PNUSAS002290	Braenderup	0	0	0	0	0	0	0	0	0	0	0

SAL_FA7855AA	CFSAN006205	Braenderup	0	0	0	0	0	0	0	0	0	0	0
SAL_FA8002AA	2016K-0446	Braenderup	0	0	0	0	0	0	0	0	0	0	0
SAL_FA7998AA	2016K-0448	Braenderup	0	0	0	0	0	0	0	0	0	0	0
SAL_FA7997AA	2016K-0449	Braenderup	0	0	0	0	0	0	0	0	0	0	0
SAL_FA7996AA	2016K-0450	Braenderup	0	0	0	0	0	0	0	0	0	0	0
SAL_FA7995AA	2016K-0451	Braenderup	0	0	0	0	0	0	0	0	0	0	0
SAL_FA7994AA	2016K-0452	Braenderup	0	0	0	0	0	0	0	0	0	0	0
SAL_FA7993AA	2016K-0453	Braenderup	0	0	0	0	0	0	0	0	0	0	0
SAL_FA7968AA	PNUSAS002360	Braenderup	0	0	0	0	0	0	0	0	0	0	0
SAL_FA7965AA	PNUSAS002371	Braenderup	0	0	0	0	0	0	0	0	0	0	0
SAL_FA7964AA	PNUSAS002361	Braenderup	0	0	0	0	0	0	0	0	0	0	0
SAL_FA7962AA	PNUSAS002362	Braenderup	0	0	0	0	0	0	0	0	0	0	0
SAL_FA7961AA	PNUSAS002363	Braenderup	0	0	0	0	0	0	0	0	0	0	0
SAL_FA7960AA	PNUSAS002364	Braenderup	0	0	0	0	0	0	0	0	0	0	0
SAL_FA7959AA	PNUSAS002365	Braenderup	0	0	0	0	0	0	0	0	0	0	0
SAL_FA7957AA	PNUSAS002367	Braenderup	0	0	0	0	0	0	0	0	0	0	0
SAL_FA7958AA	PNUSAS002366	Braenderup	0	0	0	0	0	0	0	0	0	0	0
SAL_FA7956AA	PNUSAS002372	Braenderup	0	0	0	0	0	0	0	0	0	0	0
SAL_FA8151AA	PNUSAS002593	Braenderup	0	0	0	0	0	0	0	0	0	0	0
SAL_FA8150AA	PNUSAS002628	Braenderup	0	0	0	0	0	0	0	0	0	0	0
SAL_BA6673AA	08:MF0011083	Braenderup	0	0	0	0	0	0	0	0	0	0	0
SAL_FA8252AA	FSIS1606931	Braenderup	0	0	0	0	0	0	0	0	0	0	0
SAL_FA7226AA	PNUSAS002200	Braenderup	0	0	0	0	0	0	0	0	0	0	0
SAL_FA7224AA	PNUSAS002110	Braenderup	0	0	0	0	0	0	0	0	0	0	0
SAL_FA7164AA	PNUSAS002124	Braenderup	0	0	0	0	0	0	0	0	0	0	0
SAL_GA2864AA	CRJJGF_00047	Braenderup	0	0	0	0	0	0	0	0	0	0	0
SAL_GA4343AA	PNUSAS002663	Braenderup	0	0	0	0	0	0	0	0	0	0	0
SAL_GA3442AA	BCW_3997	Braenderup	0	0	0	0	0	0	0	0	0	0	0
SAL_GA3350AA	BCW_2651	Braenderup	0	0	0	0	0	0	0	0	0	0	0
SAL_GA3145AA	NCTR-SF57	Braenderup	0	0	0	0	0	0	0	0	0	0	0
SAL_GA4421AA	2016K-0457	Braenderup	0	0	0	0	0	0	0	0	0	0	0
SAL_GA4400AA	2016K-0471	Braenderup	0	0	0	0	0	0	0	0	0	0	0
SAL_GA2865AA	CRJJGF_00046	Braenderup	0	0	0	0	0	0	0	0	0	0	0
SAL_GA2408AA	BCW_2011	Braenderup	0	0	0	0	0	0	0	0	0	0	0
SAL_GA2337AA	BCW_2649	Braenderup	0	0	0	0	0	0	0	0	0	0	0
SAL_GA2336AA	BCW_2650	Braenderup	0	0	0	0	0	0	0	0	0	0	0
SAL_GA2180AA	BCW_6257	Braenderup	0	0	0	0	0	0	0	0	0	0	0
SAL_GA2179AA	BCW_6258	Braenderup	0	0	0	0	0	0	0	0	0	0	0
SAL_GA2178AA	BCW_6259	Braenderup	0	0	0	0	0	0	0	0	0	0	0
SAL_GA2133AA	BCW_6304	Braenderup	0	0	0	0	0	0	0	0	0	0	0
SAL_GA2082AA	BCW_3058	Braenderup	0	0	0	0	0	0	0	0	0	0	0
SAL_GA2041AA	BCW_3993	Braenderup	0	0	0	0	0	0	0	0	0	0	0
SAL_GA2042AA	BCW_3975	Braenderup	0	0	0	0	0	0	0	0	0	0	0
SAL_GA4497AA	FDA00007849	Braenderup	0	0	0	0	0	0	0	0	0	0	0
SAL_GA4496AA	FDA00007848	Braenderup	0	0	0	0	0	0	0	0	0	0	0
SAL_GA1938AA	BCW_4901	Braenderup	0	0	0	0	0	0	0	0	0	0	0
SAL_GA1940AA	BCW_4899	Braenderup	0	0	0	0	0	0	0	0	0	0	0
SAL_GA1932AA	BCW_4907	Braenderup	0	0	0	0	0	0	0	0	0	0	0
SAL_GA1930AA	BCW_4909	Braenderup	0	0	0	0	0	0	0	0	0	0	0
SAL_GA1924AA	BCW_4915	Braenderup	0	0	0	0	0	0	0	0	0	0	0
SAL_GA1917AA	BCW_4922	Braenderup	0	0	0	0	0	0	0	0	0	0	0
SAL_GA1906AA	BCW_4933	Braenderup	0	0	0	0	0	0	0	0	0	0	0
SAL_GA1905AA	BCW_4934	Braenderup	0	0	0	0	0	0	0	0	0	0	0
SAL_GA1904AA	BCW_4935	Braenderup	0	0	0	0	0	0	0	0	0	0	0
SAL_GA4635AA	LH_M227	Braenderup	0	0	0	0	0	0	0	0	0	0	0
SAL_GA4643AA	LH_M30	Braenderup	0	0	0	0	0	0	0	0	0	0	0
SAL_GA1751AA	N32758	Braenderup	0	0	0	0	0	0	0	0	0	0	0
SAL_GA1683AA	BCW_2854	Braenderup	0	0	0	0	0	0	0	0	0	0	0
SAL_GA4639AA	LH_M258	Braenderup	0	0	0	0	0	0	0	0	0	0	0
SAL_GA1657AA	BCW_3972	Braenderup	0	0	0	0	0	0	0	0	0	0	0
SAL_GA0355AA	VNSC2114-sc-2280603	Braenderup	0	0	0	0	0	0	0	0	0	0	0
SAL_GA0245AA	CG23C_2_1-sc-2280776	Braenderup	0	0	0	0	0	0	0	0	0	0	0
SAL_GA0246AA	CG23C_1_1-sc-2280775	Braenderup	0	0	0	0	0	0	0	0	0	0	0
SAL_GA0244AA	CG23C_3_1-sc-2280777	Braenderup	0	0	0	0	0	0	0	0	0	0	0
SAL_GA0218AA	MT48F-sc-2280829	Braenderup	0	0	0	0	0	0	0	0	0	0	0
SAL_FA9510AA	CFSAN038506	Braenderup	0	0	0	0	0	0	0	0	0	0	0
SAL_GA4842AA	CFSAN048015	Braenderup	0	0	0	0	0	0	0	0	0	0	0
SAL_GA4841AA	CFSAN048016	Braenderup	0	0	0	0	0	0	0	0	0	0	0
SAL_GA4796AA	2016K-0447	Braenderup	0	0	0	0	0	0	0	0	0	0	0
SAL_GA4777AA	PNUSAS002828	Braenderup	0	0	0	0	0	0	0	0	0	0	0
SAL_GA4997AA	H103540775	Braenderup	0	0	0	0	0	0	0	0	0	0	0
SAL_GA4758AA	PNUSAS002760	Braenderup	0	0	0	0	0	0	0	0	0	0	0
SAL_GA4704AA	FSIS1606997	Braenderup	0	0	0	0	0	0	0	0	0	0	0

SAL_GA5142AA	PNUSAS002921	Braenderup	0	0	0	0	0	0	0	0	0	0	0
SAL_GA5072AA	PNUSAS002844	Braenderup	0	0	0	0	0	0	0	0	0	0	0
SAL_GA5067AA	PNUSAS002755	Braenderup	0	0	0	0	0	0	0	0	0	0	0
SAL_GA5024AA	PNUSAS002845	Braenderup	0	0	0	0	0	0	0	0	0	0	0
SAL_FA9283AA	CFSAN046993	Braenderup	0	0	0	0	0	0	0	0	0	0	0
SAL_FA9282AA	CFSAN046994	Braenderup	0	0	0	0	0	0	0	0	0	0	0
SAL_GA5169AA	V-CLASP-D-51	Braenderup	0	0	0	0	0	0	0	0	0	0	0
SAL_GA5642AA	CVM N62455	Braenderup	0	0	0	0	0	0	0	0	0	0	0
SAL_GA5636AA	CVM N62461	Braenderup	0	0	0	0	0	0	0	0	0	0	0
SAL_GA5486AA	CFSAN039259	Braenderup	0	0	0	0	0	0	0	0	0	0	0
SAL_GA5439AA	PNUSAS002756	Braenderup	0	0	0	0	0	0	0	0	0	0	0
SAL_FA8997AA	CVM N41747	Braenderup	0	0	0	0	0	0	0	0	0	0	0
SAL_FA8951AA	CVM N42235	Braenderup	0	0	0	0	0	0	0	0	0	0	0
SAL_GA5923AA	PNUSAS002764	Braenderup	0	0	0	0	0	0	0	0	0	0	0
SAL_FA8601AA	CVM N32758	Braenderup	0	0	0	0	0	0	0	0	0	0	0
SAL_FA8487AA	PNUSAS002370	Braenderup	0	0	0	0	0	0	0	0	0	0	0
SAL_GA5590AA	CVM N62515	Braenderup	0	0	0	0	0	0	0	0	0	0	0
SAL_GA5959AA	FSIS1606996	Braenderup	0	0	0	0	0	0	0	0	0	0	0
SAL_GA6118AA	PNUSAS002763	Braenderup	0	0	0	0	0	0	0	0	0	0	0
SAL_GA6102AA	PNUSAS002940	Braenderup	0	0	0	0	0	0	0	0	0	0	0
SAL_GA6097AA	PNUSAS002941	Braenderup	0	0	0	0	0	0	0	0	0	0	0
SAL_GA6096AA	PNUSAS002942	Braenderup	0	0	0	0	0	0	0	0	0	0	0
SAL_GA6095AA	PNUSAS002943	Braenderup	0	0	0	0	0	0	0	0	0	0	0
SAL_GA6070AA	PNUSAS002762	Braenderup	0	0	0	0	0	0	0	0	0	0	0
SAL_GA6526AA	PNUSAS002359	Braenderup	0	0	0	0	0	0	0	0	0	0	0
SAL_GA6540AA	PNUSAS003230	Braenderup	0	0	0	0	0	0	0	0	0	0	0
SAL_GA6374AA	2016K-0562	Braenderup	0	0	0	0	0	0	0	0	0	0	0
SAL_GA6366AA	2016K-0555	Braenderup	0	0	0	0	0	0	0	0	0	0	0
SAL_GA6486AA	PNUSAS003211	Braenderup	0	0	0	0	0	0	0	0	0	0	0
SAL_GA6326AA	CFSAN048052	Braenderup	0	0	0	0	0	0	0	0	0	0	0
SAL_GA6633AA	PNUSAS002754	Braenderup	0	0	0	0	0	0	0	0	0	0	0
SAL_GA6606AA	PNUSAS002761	Braenderup	0	0	0	0	0	0	0	0	0	0	0
SAL_GA6558AA	PNUSAS003178	Braenderup	0	0	0	0	0	0	0	0	0	0	0
SAL_GA7153AA	CFSAN044886	Braenderup	0	0	0	0	0	0	0	0	0	0	0
SAL_GA7152AA	CFSAN044887	Braenderup	0	0	0	0	0	0	0	0	0	0	0
SAL_GA7149AA	CFSAN044890	Braenderup	0	0	0	0	0	0	0	0	0	0	0
SAL_GA7069AA	CFSAN044975	Braenderup	0	0	0	0	0	0	0	0	0	0	0
SAL_GA7068AA	CFSAN044976	Braenderup	0	0	0	0	0	0	0	0	0	0	0
SAL_GA7036AA	CFSAN045008	Braenderup	0	0	0	0	0	0	0	0	0	0	0
SAL_GA6971AA	FDA00010504	Braenderup	0	0	0	0	0	0	0	0	0	0	0
SAL_GA6970AA	FDA00010505	Braenderup	0	0	0	0	0	0	0	0	0	0	0
SAL_GA6969AA	FDA00010506	Braenderup	0	0	0	0	0	0	0	0	0	0	0
SAL_GA6968AA	FDA00010507	Braenderup	0	0	0	0	0	0	0	0	0	0	0
SAL_GA7438AA	FSIS1607334	Braenderup	0	0	0	0	0	0	0	0	0	0	0
SAL_GA7383AA	FDA00007850	Braenderup	0	0	0	0	0	0	0	0	0	0	0
SAL_GA7374AA	FDA00004741	Braenderup	0	0	0	0	0	0	0	0	0	0	0
SAL_GA7520AA	PNUSAS003213	Braenderup	0	0	0	0	0	0	0	0	0	0	0
SAL_GA7519AA	PNUSAS003214	Braenderup	0	0	0	0	0	0	0	0	0	0	0
SAL_GA7564AA	PNUSAS003216	Braenderup	0	0	0	0	0	0	0	0	0	0	0
SAL_GA7502AA	PNUSAS003215	Braenderup	0	0	0	0	0	0	0	0	0	0	0
SAL_GA7522AA	PNUSAS003210	Braenderup	0	0	0	0	0	0	0	0	0	0	0
SAL_GA7521AA	PNUSAS003212	Braenderup	0	0	0	0	0	0	0	0	0	0	0
SAL_GA7665AA	CFSAN037730	Braenderup	0	0	0	0	0	0	0	0	0	0	0
SAL_GA7993AA	PNUSAS003104	Braenderup	0	0	0	0	0	0	0	0	0	0	0
SAL_GA7840AA	2014AM-1282	Braenderup	0	0	0	0	0	0	0	0	0	0	0
SAL_GA8078AA	PNUSAS002368	Braenderup	0	0	0	0	0	0	0	0	0	0	0
SAL_GA8077AA	PNUSAS002369	Braenderup	0	0	0	0	0	0	0	0	0	0	0
SAL_GA8066AA	PNUSAS003218	Braenderup	0	0	0	0	0	0	0	0	0	0	0
SAL_GA8067AA	PNUSAS003217	Braenderup	0	0	0	0	0	0	0	0	0	0	0
SAL_GA8436AA	FSIS1607460	Braenderup	0	0	0	0	0	0	0	0	0	0	0
SAL_GA8558AA	PNUSAS003371	Braenderup	0	0	0	0	0	0	0	0	0	0	0
SAL_GA8659AA	PNUSAS002592	Braenderup	0	0	0	0	0	0	0	0	0	0	0
SAL_GA8713AA	FSIS1607497	Braenderup	0	0	0	0	0	0	0	0	0	0	0
SAL_GA8888AA	PNUSAS003670	Braenderup	0	0	0	0	0	0	0	0	0	0	0
SAL_GA9006AA	CFSAN047927	Braenderup	0	0	0	0	0	0	0	0	0	0	0
SAL_GA9038AA	CFSAN047907	Braenderup	0	0	0	0	0	0	0	0	0	0	0
SAL_GA9039AA	CFSAN047906	Braenderup	0	0	0	0	0	0	0	0	0	0	0
SAL_GA9205AA	CFSAN031508	Braenderup	0	0	0	0	0	0	0	0	0	0	0
SAL_GA9184AA	CFSAN031529	Braenderup	0	0	0	0	0	0	0	0	0	0	0
SAL_GA9185AA	CFSAN031528	Braenderup	0	0	0	0	0	0	0	0	0	0	0
SAL_GA9188AA	CFSAN031525	Braenderup	0	0	0	0	0	0	0	0	0	0	0
SAL_GA9186AA	CFSAN031527	Braenderup	0	0	0	0	0	0	0	0	0	0	0
SAL_GA9187AA	CFSAN031526	Braenderup	0	0	0	0	0	0	0	0	0	0	0

SAL_GA9189AA	CFSAN031524	Braenderup	0	0	0	0	0	0	0	0	0	0
SAL_GA9275AA	PNUSAS003674	Braenderup	0	0	0	0	0	0	0	0	0	0
SAL_GA9254AA	PNUSAS003881	Braenderup	0	0	0	0	0	0	0	0	0	0
SAL_GA9822AA	PNUSAS003765	Braenderup	0	0	0	0	0	0	0	0	0	0
SAL_GA9781AA	PNUSAS004058	Braenderup	0	0	0	0	0	0	0	0	0	0
SAL_GA9981AA	PNUSAS003971	Braenderup	0	0	0	0	0	0	0	0	0	0
SAL_HA0315AA	PNUSAS003461	Braenderup	0	0	0	0	0	0	0	0	0	0
SAL_HA0756AA	PNUSAS004437	Braenderup	0	0	0	0	0	0	0	0	0	0
SAL_HA0747AA	PNUSAS004447	Braenderup	0	0	0	0	0	0	0	0	0	0
SAL_HA0723AA	PNUSAS004494	Braenderup	0	0	0	0	0	0	0	0	0	0
SAL_HA1064AA	ARIM-SBRP270-13	Braenderup	0	0	0	0	0	0	0	0	0	0
SAL_HA1287AA	FSIS1607863	Braenderup	0	0	0	0	0	0	0	0	0	0
SAL_HA1369AA	PNUSAS004430	Braenderup	0	0	0	0	0	0	0	0	0	0
SAL_HA1447AA	CFSAN035945	Braenderup	0	0	0	0	0	0	0	0	0	0
SAL_HA1446AA	CFSAN035947	Braenderup	0	0	0	0	0	0	0	0	0	0
SAL_HA1448AA	CFSAN035944	Braenderup	0	0	0	0	0	0	0	0	0	0
SAL_HA1441AA	CFSAN035980	Braenderup	0	0	0	0	0	0	0	0	0	0
SAL_GA9807AA	PNUSAS003669	Braenderup	0	0	0	0	0	0	0	0	0	0
SAL_HA1764AA	PNUSAS002757	Braenderup	0	0	0	0	0	0	0	0	0	0
SAL_HA1759AA	PNUSAS002759	Braenderup	0	0	0	0	0	0	0	0	0	0
SAL_HA1765AA	PNUSAS002758	Braenderup	0	0	0	0	0	0	0	0	0	0
SAL_HA2941AA	FSIS1608104	Braenderup	0	0	0	0	0	0	0	0	0	0
SAL_HA2950AA	FSIS1608099	Braenderup	0	0	0	0	0	0	0	0	0	0
SAL_HA4057AA	PNUSAS005171	Braenderup	0	0	0	0	0	0	0	0	0	0
SAL_HA4338AA	OH-1401527	Braenderup	0	0	0	0	0	0	0	0	0	0
SAL_HA4547AA	CFSAN035943	Braenderup	0	0	0	0	0	0	0	0	0	0
SAL_HA4548AA	CFSAN035948	Braenderup	0	0	0	0	0	0	0	0	0	0
SAL_HA4553AA	CFSAN035937	Braenderup	0	0	0	0	0	0	0	0	0	0
SAL_HA4555AA	CFSAN035938	Braenderup	0	0	0	0	0	0	0	0	0	0
SAL_HA4534AA	CFSAN035950	Braenderup	0	0	0	0	0	0	0	0	0	0
SAL_HA4481AA	CFSAN035982	Braenderup	0	0	0	0	0	0	0	0	0	0
SAL_HA4701AA	FSIS1608648	Braenderup	0	0	0	0	0	0	0	0	0	0
SAL_HA4477AA	CFSAN035981	Braenderup	0	0	0	0	0	0	0	0	0	0
SAL_HA4792AA	10-0026	Braenderup	0	0	0	0	0	0	0	0	0	0
SAL_HA4960AA	278955	Braenderup	0	0	0	0	0	0	0	0	0	0
SAL_HA4949AA	315114	Braenderup	0	0	0	0	0	0	0	0	0	0
SAL_HA4890AA	152977	Braenderup	0	0	0	0	0	0	0	0	0	0
SAL_HA5169AA	12-0031	Braenderup	0	0	0	0	0	0	0	0	0	0
SAL_HA5170AA	1133653	Braenderup	0	0	0	0	0	0	0	0	0	0
SAL_HA5171AA	168713	Braenderup	0	0	0	0	0	0	0	0	0	0
SAL_HA5038AA	1133318	Braenderup	0	0	0	0	0	0	0	0	0	0
SAL_HA4977AA	1772127	Braenderup	0	0	0	0	0	0	0	0	0	0
SAL_HA5023AA	1779798	Braenderup	0	0	0	0	0	0	0	0	0	0
SAL_HA5006AA	1800982	Braenderup	0	0	0	0	0	0	0	0	0	0
SAL_HA5017AA	1958402	Braenderup	0	0	0	0	0	0	0	0	0	0
SAL_HA5063AA	2089524	Braenderup	0	0	0	0	0	0	0	0	0	0
SAL_HA5055AA	2065417	Braenderup	0	0	0	0	0	0	0	0	0	0
SAL_HA5409AA	13-2733	Braenderup	0	0	0	0	0	0	0	0	0	0
SAL_HA5048AA	2067609	Braenderup	0	0	0	0	0	0	0	0	0	0
SAL_HA5403AA	13-7069	Braenderup	0	0	0	0	0	0	0	0	0	0
SAL_HA5418AA	13-2740	Braenderup	0	0	0	0	0	0	0	0	0	0
SAL_HA5439AA	14-0315	Braenderup	0	0	0	0	0	0	0	0	0	0
SAL_HA5328AA	PNUSAS005641	Braenderup	0	0	0	0	0	0	0	0	0	0
SAL_HA4987AA	1865264	Braenderup	0	0	0	0	0	0	0	0	0	0
SAL_HA5572AA	L01045-14	Braenderup	0	0	0	0	0	0	0	0	0	0
SAL_HA5058AA	13-0112	Braenderup	0	0	0	0	0	0	0	0	0	0
SAL_HA2509AA	CFSAN035792	Braenderup	0	0	0	0	0	0	0	0	0	0
SAL_HA2497AA	CFSAN035793	Braenderup	0	0	0	0	0	0	0	0	0	0
SAL_HA2535AA	CFSAN035762	Braenderup	0	0	0	0	0	0	0	0	0	0
SAL_HA4729AA	FSIS1608448	Braenderup	0	0	0	0	0	0	0	0	0	0
SAL_HA3849AA	PNUSAS005283	Braenderup	0	0	0	0	0	0	0	0	0	0
SAL_HA6506AA	FSIS1609174	Braenderup	0	0	0	0	0	0	0	0	0	0
SAL_HA6505AA	FSIS1609173	Braenderup	0	0	0	0	0	0	0	0	0	0
SAL_HA5835AA	PNUSAS005810	Braenderup	0	0	0	0	0	0	0	0	0	0
SAL_HA6657AA	CFSAN056744	Braenderup	0	0	0	0	0	0	0	0	0	0
SAL_HA6747AA	OH-1404949	Braenderup	0	0	0	0	0	0	0	0	0	0
SAL_HA5975AA	CFSAN056588	Braenderup	0	0	0	0	0	0	0	0	0	0
SAL_HA6103AA	FSIS1608804	Braenderup	0	0	0	0	0	0	0	0	0	0
SAL_HA6903AA	PNUSAS006044	Braenderup	0	0	0	0	0	0	0	0	0	0
SAL_HA6900AA	PNUSAS005501	Braenderup	0	0	0	0	0	0	0	0	0	0
SAL_HA6856AA	PNUSAS006018	Braenderup	0	0	0	0	0	0	0	0	0	0
SAL_HA6809AA	PNUSAS005616	Braenderup	0	0	0	0	0	0	0	0	0	0
SAL_HA6787AA	PNUSAS005626	Braenderup	0	0	0	0	0	0	0	0	0	0

SAL_HA7338AA	PNUSAS006064	Braenderup	0	0	0	0	0	0	0	0	0	0	0
SAL_HA7348AA	PNUSAS005774	Braenderup	0	0	0	0	0	0	0	0	0	0	0
SAL_HA7346AA	PNUSAS005776	Braenderup	0	0	0	0	0	0	0	0	0	0	0
SAL_HA7360AA	PNUSAS006280	Braenderup	0	0	0	0	0	0	0	0	0	0	0
SAL_HA8634AA	PNUSAS005182	Braenderup	0	0	0	0	0	0	0	0	0	0	0
SAL_HA8617AA	PNUSAS005747	Braenderup	0	0	0	0	0	0	0	0	0	0	0
SAL_HA8594AA	PNUSAS005986	Braenderup	0	0	0	0	0	0	0	0	0	0	0
SAL_HA8212AA	CFSAN035928	Braenderup	0	0	0	0	0	0	0	0	0	0	0
SAL_HA8694AA	FSIS1609451	Braenderup	0	0	0	0	0	0	0	0	0	0	0
SAL_HA8724AA	CFSAN058067	Braenderup	0	0	0	0	0	0	0	0	0	0	0
SAL_HA8726AA	CFSAN058070	Braenderup	0	0	0	0	0	0	0	0	0	0	0
SAL_HA8720AA	CFSAN058065	Braenderup	0	0	0	0	0	0	0	0	0	0	0
SAL_HA8721AA	CFSAN058068	Braenderup	0	0	0	0	0	0	0	0	0	0	0
SAL_HA8723AA	CFSAN058069	Braenderup	0	0	0	0	0	0	0	0	0	0	0
SAL_HA8725AA	CFSAN058066	Braenderup	0	0	0	0	0	0	0	0	0	0	0
SAL_HA8727AA	CFSAN058071	Braenderup	0	0	0	0	0	0	0	0	0	0	0
SAL_HA8728AA	CFSAN058072	Braenderup	0	0	0	0	0	0	0	0	0	0	0
SAL_HA8729AA	CFSAN058076	Braenderup	0	0	0	0	0	0	0	0	0	0	0
SAL_HA8732AA	CFSAN058074	Braenderup	0	0	0	0	0	0	0	0	0	0	0
SAL_HA8730AA	CFSAN058073	Braenderup	0	0	0	0	0	0	0	0	0	0	0
SAL_HA8908AA	NCTC5750	Braenderup	0	0	0	0	0	0	0	0	0	0	0
SAL_HA8731AA	CFSAN058075	Braenderup	0	0	0	0	0	0	0	0	0	0	0
SAL_HA8795AA	NSUOC-0161-W5X	Braenderup	0	0	0	0	0	0	0	0	0	0	0
SAL_HA9237AA	PNUSAS004903	Braenderup	0	0	0	0	0	0	0	0	0	0	0
SAL_HA9128AA	CFSAN058078	Braenderup	0	0	0	0	0	0	0	0	0	0	0
SAL_HA9226AA	PNUSAS004904	Braenderup	0	0	0	0	0	0	0	0	0	0	0
SAL_HA9583AA	PNUSAS006502	Braenderup	0	0	0	0	0	0	0	0	0	0	0
SAL_HA9582AA	PNUSAS006501	Braenderup	0	0	0	0	0	0	0	0	0	0	0
SAL_HA9585AA	PNUSAS006505	Braenderup	0	0	0	0	0	0	0	0	0	0	0
SAL_HA9591AA	PNUSAS006500	Braenderup	0	0	0	0	0	0	0	0	0	0	0
SAL_HA9563AA	PNUSAS006503	Braenderup	0	0	0	0	0	0	0	0	0	0	0
SAL_HA9580AA	PNUSAS006504	Braenderup	0	0	0	0	0	0	0	0	0	0	0
SAL_HA9499AA	PNUSAS005521	Braenderup	0	0	0	0	0	0	0	0	0	0	0
SAL_HA9716AA	CFSAN058815	Braenderup	0	0	0	0	0	0	0	0	0	0	0
SAL_IA0568AA	PNUSAS007494	Braenderup	0	0	0	0	0	0	0	0	0	0	0
SAL_IA0553AA	PNUSAS005505	Braenderup	0	0	0	0	0	0	0	0	0	0	0
SAL_IA0552AA	PNUSAS005520	Braenderup	0	0	0	0	0	0	0	0	0	0	0
SAL_IA0409AA	PNUSAS007354	Braenderup	0	0	0	0	0	0	0	0	0	0	0
SAL_IA0407AA	PNUSAS007355	Braenderup	0	0	0	0	0	0	0	0	0	0	0
SAL_IA0388AA	PNUSAS007185	Braenderup	0	0	0	0	0	0	0	0	0	0	0
SAL_IA0383AA	PNUSAS007194	Braenderup	0	0	0	0	0	0	0	0	0	0	0
SAL_IA1862AA	285971	Braenderup	0	0	0	0	0	0	0	0	0	0	0
SAL_IA1842AA	255526	Braenderup	0	0	0	0	0	0	0	0	0	0	0
SAL_IA1224AA	189190	Braenderup	0	0	0	0	0	0	0	0	0	0	0
SAL_IA0754AA	171282	Braenderup	0	0	0	0	0	0	0	0	0	0	0
SAL_IA2737AA	MS160099	Braenderup	0	0	0	0	0	0	0	0	0	0	0
SAL_IA2683AA	FDA00007915	Braenderup	0	0	0	0	0	0	0	0	0	0	0
SAL_IA2670AA	CFSAN059212	Braenderup	0	0	0	0	0	0	0	0	0	0	0
SAL_IA2531AA	PNUSAS006633	Braenderup	0	0	0	0	0	0	0	0	0	0	0
SAL_IA2560AA	PNUSAS007449	Braenderup	0	0	0	0	0	0	0	0	0	0	0
SAL_IA3791AA	PNUSAS007310	Braenderup	0	0	0	0	0	0	0	0	0	0	0
SAL_IA3712AA	PNUSAS007538	Braenderup	0	0	0	0	0	0	0	0	0	0	0
SAL_IA3725AA	PNUSAS006844	Braenderup	0	0	0	0	0	0	0	0	0	0	0
SAL_IA4646AA	PNUSAS008059	Braenderup	0	0	0	0	0	0	0	0	0	0	0
SAL_IA3707AA	FSIS1710136	Braenderup	0	0	0	0	0	0	0	0	0	0	0
SAL_IA5030AA	MS160346	Braenderup	0	0	0	0	0	0	0	0	0	0	0
SAL_IA5016AA	PNUSAS006886	Braenderup	0	0	0	0	0	0	0	0	0	0	0
SAL_IA5216AA	NSUOC-0073-W3B2	Braenderup	0	0	0	0	0	0	0	0	0	0	0
SAL_IA5267AA	CFSAN058002	Braenderup	0	0	0	0	0	0	0	0	0	0	0
SAL_IA5944AA	MS160358	Braenderup	0	0	0	0	0	0	0	0	0	0	0
SAL_IA5837AA	PNUSAS007016	Braenderup	0	0	0	0	0	0	0	0	0	0	0
SAL_IA5946AA	MS160356	Braenderup	0	0	0	0	0	0	0	0	0	0	0
SAL_IA6338AA	FSIS1709974	Braenderup	0	0	0	0	0	0	0	0	0	0	0
SAL_IA6297AA	PNUSAS008240	Braenderup	0	0	0	0	0	0	0	0	0	0	0
SAL_IA6304AA	PNUSAS008229	Braenderup	0	0	0	0	0	0	0	0	0	0	0
SAL_IA6259AA	PNUSAS007786	Braenderup	0	0	0	0	0	0	0	0	0	0	0
SAL_IA6159AA	PNUSAS008169	Braenderup	0	0	0	0	0	0	0	0	0	0	0
SAL_IA6154AA	PNUSAS006840	Braenderup	0	0	0	0	0	0	0	0	0	0	0
SAL_IA5668AA	PNUSAS008035	Braenderup	0	0	0	0	0	0	0	0	0	0	0
SAL_IA5741AA	NSUOC-0226-W1X	Braenderup	0	0	0	0	0	0	0	0	0	0	0
SAL_IA5692AA	PNUSAS008137	Braenderup	0	0	0	0	0	0	0	0	0	0	0
SAL_IA5671AA	PNUSAS008034	Braenderup	0	0	0	0	0	0	0	0	0	0	0
SAL_IA5398AA	PNUSAS008026	Braenderup	0	0	0	0	0	0	0	0	0	0	0

SAL_IA5745AA	NSUOC-0227-W1H	Braenderup	0	0	0	0	0	0	0	0	0	0	0
SAL_IA6482AA	PNUSAS008321	Braenderup	0	0	0	0	0	0	0	0	0	0	0
SAL_IA6527AA	PNUSAS008742	Braenderup	0	0	0	0	0	0	0	0	0	0	0
SAL_IA6581AA	CFSAN058783	Braenderup	0	0	0	0	0	0	0	0	0	0	0
SAL_IA7276AA	S/20170043	Braenderup	0	0	0	0	0	0	0	0	0	0	0
SAL_IA7191AA	PNUSAS008371	Braenderup	0	0	0	0	0	0	0	0	0	0	0
SAL_IA7120AA	OH-16-1758-2T	Braenderup	0	0	0	0	0	0	0	0	0	0	0
SAL_IA7122AA	OH-16-1758-2B	Braenderup	0	0	0	0	0	0	0	0	0	0	0
SAL_IA7088AA	OH-16-1757-8T	Braenderup	0	0	0	0	0	0	0	0	0	0	0
SAL_IA7091AA	OH-16-1757-7T	Braenderup	0	0	0	0	0	0	0	0	0	0	0
SAL_IA7094AA	OH-16-1757-4B	Braenderup	0	0	0	0	0	0	0	0	0	0	0
SAL_IA7092AA	OH-16-1757-5T	Braenderup	0	0	0	0	0	0	0	0	0	0	0
SAL_IA7096AA	OH-16-1757-6T	Braenderup	0	0	0	0	0	0	0	0	0	0	0
SAL_IA7099AA	OH-16-1757-8B	Braenderup	0	0	0	0	0	0	0	0	0	0	0
SAL_IA7344AA	S/20170056	Braenderup	0	0	0	0	0	0	0	0	0	0	0
SAL_IA7366AA	FDA00007847	Braenderup	0	0	0	0	0	0	0	0	0	0	0
SAL_IA7488AA	PNUSAS009082	Braenderup	0	0	0	0	0	0	0	0	0	0	0
SAL_IA7415AA	CFSAN060226	Braenderup	0	0	0	0	0	0	0	0	0	0	0
SAL_IA7402AA	CFSAN060225	Braenderup	0	0	0	0	0	0	0	0	0	0	0
SAL_IA7581AA	PNUSAS009130	Braenderup	0	0	0	0	0	0	0	0	0	0	0
SAL_IA7583AA	PNUSAS009110	Braenderup	0	0	0	0	0	0	0	0	0	0	0
SAL_IA7603AA	PNUSAS009111	Braenderup	0	0	0	0	0	0	0	0	0	0	0
SAL_IA7587AA	PNUSAS009101	Braenderup	0	0	0	0	0	0	0	0	0	0	0
SAL_IA7797AA	FDA00007837	Braenderup	0	0	0	0	0	0	0	0	0	0	0
SAL_IA8152AA	ADRDL-963	Braenderup	0	0	0	0	0	0	0	0	0	0	0
SAL_IA8250AA	PNUSAS008834	Braenderup	0	0	0	0	0	0	0	0	0	0	0
SAL_IA8418AA	PNUSAS009243	Braenderup	0	0	0	0	0	0	0	0	0	0	0
SAL_IA8366AA	PNUSAS009060	Braenderup	0	0	0	0	0	0	0	0	0	0	0
SAL_IA8367AA	PNUSAS009062	Braenderup	0	0	0	0	0	0	0	0	0	0	0
SAL_IA8373AA	PNUSAS008686	Braenderup	0	0	0	0	0	0	0	0	0	0	0
SAL_IA8413AA	PNUSAS008763	Braenderup	0	0	0	0	0	0	0	0	0	0	0
SAL_IA8372AA	PNUSAS009054	Braenderup	0	0	0	0	0	0	0	0	0	0	0
SAL_IA8827AA	PNUSAS008096	Braenderup	0	0	0	0	0	0	0	0	0	0	0
SAL_IA8829AA	PNUSAS008094	Braenderup	0	0	0	0	0	0	0	0	0	0	0
SAL_IA8936AA	PNUSAS009372	Braenderup	0	0	0	0	0	0	0	0	0	0	0
SAL_IA8922AA	PNUSAS009377	Braenderup	0	0	0	0	0	0	0	0	0	0	0
SAL_IA9085AA	S/20160256-1	Braenderup	0	0	0	0	0	0	0	0	0	0	0
SAL_IA9452AA	PNUSAS009536	Braenderup	0	0	0	0	0	0	0	0	0	0	0
SAL_IA9465AA	PNUSAS006966	Braenderup	0	0	0	0	0	0	0	0	0	0	0
SAL_IA9326AA	PNUSAS006940	Braenderup	0	0	0	0	0	0	0	0	0	0	0
SAL_IA9352AA	PNUSAS009426	Braenderup	0	0	0	0	0	0	0	0	0	0	0
SAL_IA9281AA	PNUSAS009555	Braenderup	0	0	0	0	0	0	0	0	0	0	0
SAL_IA9294AA	PNUSAS009423	Braenderup	0	0	0	0	0	0	0	0	0	0	0
SAL_IA9259AA	PNUSAS009493	Braenderup	0	0	0	0	0	0	0	0	0	0	0
SAL_IA9147AA	PNUSAS010166	Braenderup	0	0	0	0	0	0	0	0	0	0	0
SAL_IA9354AA	PNUSAS009655	Braenderup	0	0	0	0	0	0	0	0	0	0	0
SAL_IA9564AA	PNUSAS009459	Braenderup	0	0	0	0	0	0	0	0	0	0	0
SAL_IA9569AA	PNUSAS008281	Braenderup	0	0	0	0	0	0	0	0	0	0	0
SAL_IA9822AA	PNUSAS009605	Braenderup	0	0	0	0	0	0	0	0	0	0	0
SAL_IA9798AA	PNUSAS009949	Braenderup	0	0	0	0	0	0	0	0	0	0	0
SAL_IA9804AA	PNUSAS009828	Braenderup	0	0	0	0	0	0	0	0	0	0	0
SAL_IA9852AA	PNUSAS009282	Braenderup	0	0	0	0	0	0	0	0	0	0	0
SAL_IA9872AA	PNUSAS010294	Braenderup	0	0	0	0	0	0	0	0	0	0	0
SAL_IA9879AA	PNUSAS010292	Braenderup	0	0	0	0	0	0	0	0	0	0	0
SAL_IA9876AA	PNUSAS010289	Braenderup	0	0	0	0	0	0	0	0	0	0	0
SAL_JA0150AA	PNUSAS010106	Braenderup	0	0	0	0	0	0	0	0	0	0	0
SAL_JA0329AA	NSUOC-0276-S4-2X	Braenderup	0	0	0	0	0	0	0	0	0	0	0
SAL_JA0301AA	PNUSAS009570	Braenderup	0	0	0	0	0	0	0	0	0	0	0
SAL_JA0434AA	PNUSAS010681	Braenderup	0	0	0	0	0	0	0	0	0	0	0
SAL_JA0325AA	NSUOC-0277-W5H	Braenderup	0	0	0	0	0	0	0	0	0	0	0
SAL_JA0865AA	TX-ACF1700589	Braenderup	0	0	0	0	0	0	0	0	0	0	0
SAL_JA1486AA	PNUSAS010762	Braenderup	0	0	0	0	0	0	0	0	0	0	0
SAL_JA1532AA	PNUSAS010753	Braenderup	0	0	0	0	0	0	0	0	0	0	0
SAL_JA1543AA	PNUSAS008909	Braenderup	0	0	0	0	0	0	0	0	0	0	0
SAL_JA1417AA	NSUOC-0181-S1-2H	Braenderup	0	0	0	0	0	0	0	0	0	0	0
SAL_JA1418AA	NSUOC-0179-W5H	Braenderup	0	0	0	0	0	0	0	0	0	0	0
SAL_JA1221AA	PNUSAS010992	Braenderup	0	0	0	0	0	0	0	0	0	0	0
SAL_JA1151AA	ADRDL-1072	Braenderup	0	0	0	0	0	0	0	0	0	0	0
SAL_JA1091AA	PNUSAS010803	Braenderup	0	0	0	0	0	0	0	0	0	0	0
SAL_JA1108AA	PNUSAS011250	Braenderup	0	0	0	0	0	0	0	0	0	0	0
SAL_JA1025AA	PNUSAS011225	Braenderup	0	0	0	0	0	0	0	0	0	0	0
SAL_JA1058AA	PNUSAS011054	Braenderup	0	0	0	0	0	0	0	0	0	0	0
SAL_JA0931AA	PNUSAS009351	Braenderup	0	0	0	0	0	0	0	0	0	0	0

SAL_JA0570AA	PNUSAS009748	Braenderup	0	0	0	0	0	0	0	0	0	0	0
SAL_JA0449AA	PNUSAS010291	Braenderup	0	0	0	0	0	0	0	0	0	0	0
SAL_JA0372AA	PNUSAS010286	Braenderup	0	0	0	0	0	0	0	0	0	0	0
SAL_JA1646AA	PNUSAS011287	Braenderup	0	0	0	0	0	0	0	0	0	0	0
SAL_JA1641AA	PNUSAS011010	Braenderup	0	0	0	0	0	0	0	0	0	0	0
SAL_JA1648AA	PNUSAS011009	Braenderup	0	0	0	0	0	0	0	0	0	0	0
SAL_JA1643AA	PNUSAS009487	Braenderup	0	0	0	0	0	0	0	0	0	0	0
SAL_JA1618AA	PNUSAS011336	Braenderup	0	0	0	0	0	0	0	0	0	0	0
SAL_JA1614AA	PNUSAS011474	Braenderup	0	0	0	0	0	0	0	0	0	0	0
SAL_JA0563AA	PNUSAS009751	Braenderup	0	0	0	0	0	0	0	0	0	0	0
SAL_JA1789AA	PNUSAS010921	Braenderup	0	0	0	0	0	0	0	0	0	0	0
SAL_JA1714AA	PNUSAS011683	Braenderup	0	0	0	0	0	0	0	0	0	0	0
SAL_JA1999AA	PNUSAS011540	Braenderup	0	0	0	0	0	0	0	0	0	0	0
SAL_JA1978AA	PNUSAS011451	Braenderup	0	0	0	0	0	0	0	0	0	0	0
SAL_JA2016AA	PNUSAS011458	Braenderup	0	0	0	0	0	0	0	0	0	0	0
SAL_JA1968AA	PNUSAS011496	Braenderup	0	0	0	0	0	0	0	0	0	0	0
SAL_JA2008AA	PNUSAS011537	Braenderup	0	0	0	0	0	0	0	0	0	0	0
SAL_JA2119AA	FSIS1710674	Braenderup	0	0	0	0	0	0	0	0	0	0	0
SAL_JA2101AA	ADRDL-996	Braenderup	0	0	0	0	0	0	0	0	0	0	0
SAL_JA2040AA	PNUSAS011604	Braenderup	0	0	0	0	0	0	0	0	0	0	0
SAL_JA2002AA	PNUSAS011536	Braenderup	0	0	0	0	0	0	0	0	0	0	0
SAL_JA2017AA	PNUSAS011527	Braenderup	0	0	0	0	0	0	0	0	0	0	0
SAL_JA2342AA	OH-16-11203-3T	Braenderup	0	0	0	0	0	0	0	0	0	0	0
SAL_JA2675AA	PNUSAS011964	Braenderup	0	0	0	0	0	0	0	0	0	0	0
SAL_JA2600AA	PNUSAS012024	Braenderup	0	0	0	0	0	0	0	0	0	0	0
SAL_JA2502AA	PNUSAS010138	Braenderup	0	0	0	0	0	0	0	0	0	0	0
SAL_JA2507AA	PNUSAS010140	Braenderup	0	0	0	0	0	0	0	0	0	0	0
SAL_JA2475AA	PNUSAS010836	Braenderup	0	0	0	0	0	0	0	0	0	0	0
SAL_JA2462AA	PNUSAS010770	Braenderup	0	0	0	0	0	0	0	0	0	0	0
SAL_JA2459AA	PNUSAS010789	Braenderup	0	0	0	0	0	0	0	0	0	0	0
SAL_JA2414AA	PNUSAS011280	Braenderup	0	0	0	0	0	0	0	0	0	0	0
SAL_JA2898AA	PNUSAS009881	Braenderup	0	0	0	0	0	0	0	0	0	0	0
SAL_JA2878AA	PNUSAS011358	Braenderup	0	0	0	0	0	0	0	0	0	0	0
SAL_JA2917AA	PNUSAS011716	Braenderup	0	0	0	0	0	0	0	0	0	0	0
SAL_JA3296AA	PNUSAS012240	Braenderup	0	0	0	0	0	0	0	0	0	0	0
SAL_JA3237AA	PNUSAS010508	Braenderup	0	0	0	0	0	0	0	0	0	0	0
SAL_JA3217AA	PNUSAS011420	Braenderup	0	0	0	0	0	0	0	0	0	0	0
SAL_JA3159AA	PNUSAS011485	Braenderup	0	0	0	0	0	0	0	0	0	0	0
SAL_JA3591AA	FSIS1700628	Braenderup	0	0	0	0	0	0	0	0	0	0	0
SAL_JA3477AA	PNUSAS010702	Braenderup	0	0	0	0	0	0	0	0	0	0	0
SAL_JA4009AA	PNUSAS012432	Braenderup	0	0	0	0	0	0	0	0	0	0	0
SAL_JA4010AA	PNUSAS012548	Braenderup	0	0	0	0	0	0	0	0	0	0	0
SAL_JA3807AA	PNUSAS011120	Braenderup	0	0	0	0	0	0	0	0	0	0	0
SAL_JA3768AA	PNUSAS012356	Braenderup	0	0	0	0	0	0	0	0	0	0	0
SAL_JA4601AA	CVM 43839	Braenderup	0	0	0	0	0	0	0	0	0	0	0
SAL_JA4595AA	CVM 43845	Braenderup	0	0	0	0	0	0	0	0	0	0	0
SAL_JA4543AA	PNUSAS013298	Braenderup	0	0	0	0	0	0	0	0	0	0	0
SAL_JA4508AA	PNUSAS012917	Braenderup	0	0	0	0	0	0	0	0	0	0	0
SAL_JA4968AA	PNUSAS012833	Braenderup	0	0	0	0	0	0	0	0	0	0	0
SAL_JA4978AA	PNUSAS012923	Braenderup	0	0	0	0	0	0	0	0	0	0	0
SAL_JA4979AA	PNUSAS012927	Braenderup	0	0	0	0	0	0	0	0	0	0	0
SAL_JA4945AA	PNUSAS013206	Braenderup	0	0	0	0	0	0	0	0	0	0	0
SAL_JA4904AA	PNUSAS013536	Braenderup	0	0	0	0	0	0	0	0	0	0	0
SAL_JA4989AA	PNUSAS013525	Braenderup	0	0	0	0	0	0	0	0	0	0	0
SAL_JA4628AA	CVM 43812	Braenderup	0	0	0	0	0	0	0	0	0	0	0
SAL_JA5077AA	PNUSAS013605	Braenderup	0	0	0	0	0	0	0	0	0	0	0
SAL_JA6532AA	349301	Braenderup	0	0	0	0	0	0	0	0	0	0	0
SAL_JA6420AA	329846	Braenderup	0	0	0	0	0	0	0	0	0	0	0
SAL_JA6347AA	337029	Braenderup	0	0	0	0	0	0	0	0	0	0	0
SAL_JA6354AA	330524	Braenderup	0	0	0	0	0	0	0	0	0	0	0
SAL_JA6321AA	PNUSAS012134	Braenderup	0	0	0	0	0	0	0	0	0	0	0
SAL_JA6209AA	PNUSAS010314	Braenderup	0	0	0	0	0	0	0	0	0	0	0
SAL_JA6178AA	PNUSAS011408	Braenderup	0	0	0	0	0	0	0	0	0	0	0
SAL_JA6415AA	353875	Braenderup	0	0	0	0	0	0	0	0	0	0	0
SAL_JA6024AA	PNUSAS012611	Braenderup	0	0	0	0	0	0	0	0	0	0	0
SAL_JA5777AA	353927	Braenderup	0	0	0	0	0	0	0	0	0	0	0
SAL_JA6455AA	368252	Braenderup	0	0	0	0	0	0	0	0	0	0	0
SAL_JA5756AA	PNUSAS013342	Braenderup	0	0	0	0	0	0	0	0	0	0	0
SAL_JA5773AA	PNUSAS013338	Braenderup	0	0	0	0	0	0	0	0	0	0	0
SAL_JA5557AA	PNUSAS005232	Braenderup	0	0	0	0	0	0	0	0	0	0	0
SAL_JA5755AA	PNUSAS013341	Braenderup	0	0	0	0	0	0	0	0	0	0	0
SAL_JA5493AA	349285	Braenderup	0	0	0	0	0	0	0	0	0	0	0
SAL_JA5439AA	362101	Braenderup	0	0	0	0	0	0	0	0	0	0	0

SAL_JA5454AA	PNUSAS013003	Braenderup	0	0	0	0	0	0	0	0	0	0	0
SAL_JA5361AA	PNUSAS012779	Braenderup	0	0	0	0	0	0	0	0	0	0	0
SAL_JA5340AA	PNUSAS013073	Braenderup	0	0	0	0	0	0	0	0	0	0	0
SAL_JA6906AA	348698	Braenderup	0	0	0	0	0	0	0	0	0	0	0
SAL_JA6922AA	337006	Braenderup	0	0	0	0	0	0	0	0	0	0	0
SAL_JA6804AA	PNUSAS013210	Braenderup	0	0	0	0	0	0	0	0	0	0	0
SAL_JA6745AA	FSIS1701209	Braenderup	0	0	0	0	0	0	0	0	0	0	0
SAL_JA6675AA	PNUSAS013271	Braenderup	0	0	0	0	0	0	0	0	0	0	0
SAL_JA6640AA	PNUSAS013561	Braenderup	0	0	0	0	0	0	0	0	0	0	0
SAL_JA7186AA	PNUSAS014459	Braenderup	0	0	0	0	0	0	0	0	0	0	0
SAL_JA7136AA	PNUSAS013965	Braenderup	0	0	0	0	0	0	0	0	0	0	0
SAL_JA7167AA	PNUSAS013889	Braenderup	0	0	0	0	0	0	0	0	0	0	0
SAL_JA7156AA	PNUSAS013895	Braenderup	0	0	0	0	0	0	0	0	0	0	0
SAL_JA7068AA	PNUSAS013801	Braenderup	0	0	0	0	0	0	0	0	0	0	0
SAL_JA7299AA	PNUSAS014638	Braenderup	0	0	0	0	0	0	0	0	0	0	0
SAL_JA7283AA	PNUSAS014407	Braenderup	0	0	0	0	0	0	0	0	0	0	0
SAL_JA7282AA	PNUSAS014637	Braenderup	0	0	0	0	0	0	0	0	0	0	0
SAL_JA7279AA	PNUSAS014415	Braenderup	0	0	0	0	0	0	0	0	0	0	0
SAL_JA8305AA	337034	Braenderup	0	0	0	0	0	0	0	0	0	0	0
SAL_JA8283AA	366340	Braenderup	0	0	0	0	0	0	0	0	0	0	0
SAL_JA8217AA	343728	Braenderup	0	0	0	0	0	0	0	0	0	0	0
SAL_JA8212AA	363507	Braenderup	0	0	0	0	0	0	0	0	0	0	0
SAL_JA8115AA	350672	Braenderup	0	0	0	0	0	0	0	0	0	0	0
SAL_JA8086AA	329803	Braenderup	0	0	0	0	0	0	0	0	0	0	0
SAL_JA8089AA	333934	Braenderup	0	0	0	0	0	0	0	0	0	0	0
SAL_JA7984AA	360318	Braenderup	0	0	0	0	0	0	0	0	0	0	0
SAL_JA7857AA	360257	Braenderup	0	0	0	0	0	0	0	0	0	0	0
SAL_JA7742AA	PNUSAS014047	Braenderup	0	0	0	0	0	0	0	0	0	0	0
SAL_JA7761AA	PNUSAS014943	Braenderup	0	0	0	0	0	0	0	0	0	0	0
SAL_JA7664AA	PNUSAS014585	Braenderup	0	0	0	0	0	0	0	0	0	0	0
SAL_JA7836AA	340256	Braenderup	0	0	0	0	0	0	0	0	0	0	0
SAL_JA7752AA	PNUSAS013454	Braenderup	0	0	0	0	0	0	0	0	0	0	0
SAL_JA8765AA	PNUSAS015049	Braenderup	0	0	0	0	0	0	0	0	0	0	0
SAL_JA8623AA	PNUSAS014282	Braenderup	0	0	0	0	0	0	0	0	0	0	0
SAL_JA8627AA	PNUSAS014281	Braenderup	0	0	0	0	0	0	0	0	0	0	0
SAL_JA8652AA	PNUSAS014471	Braenderup	0	0	0	0	0	0	0	0	0	0	0
SAL_JA8701AA	PNUSAS013489	Braenderup	0	0	0	0	0	0	0	0	0	0	0
SAL_JA9544AA	NSUOC-0359-S5-2B	Braenderup	0	0	0	0	0	0	0	0	0	0	0
SAL_JA9547AA	NSUOC-0369-W1B	Braenderup	0	0	0	0	0	0	0	0	0	0	0
SAL_JA9460AA	PNUSAS014376	Braenderup	0	0	0	0	0	0	0	0	0	0	0
SAL_JA9473AA	PNUSAS013860	Braenderup	0	0	0	0	0	0	0	0	0	0	0
SAL_JA9486AA	PNUSAS014375	Braenderup	0	0	0	0	0	0	0	0	0	0	0
SAL_JA9400AA	PNUSAS014930	Braenderup	0	0	0	0	0	0	0	0	0	0	0
SAL_JA9377AA	PNUSAS014802	Braenderup	0	0	0	0	0	0	0	0	0	0	0
SAL_JA9372AA	PNUSAS014803	Braenderup	0	0	0	0	0	0	0	0	0	0	0
SAL_JA9419AA	PNUSAS014898	Braenderup	0	0	0	0	0	0	0	0	0	0	0
SAL_JA9359AA	PNUSAS014529	Braenderup	0	0	0	0	0	0	0	0	0	0	0
SAL_JA9773AA	FSIS1701677	Braenderup	0	0	0	0	0	0	0	0	0	0	0
SAL_JA8980AA	PNUSAS015262	Braenderup	0	0	0	0	0	0	0	0	0	0	0
SAL_JA9686AA	PNUSAS015467	Braenderup	0	0	0	0	0	0	0	0	0	0	0
SAL_JA9315AA	PNUSAS014530	Braenderup	0	0	0	0	0	0	0	0	0	0	0
SAL_JA9299AA	PNUSAS014528	Braenderup	0	0	0	0	0	0	0	0	0	0	0
SAL_JA9338AA	PNUSAS015199	Braenderup	0	0	0	0	0	0	0	0	0	0	0
SAL_JA9227AA	PNUSAS015241	Braenderup	0	0	0	0	0	0	0	0	0	0	0
SAL_JA9236AA	PNUSAS015174	Braenderup	0	0	0	0	0	0	0	0	0	0	0
SAL_JA9167AA	PNUSAS014061	Braenderup	0	0	0	0	0	0	0	0	0	0	0
SAL_JA9168AA	PNUSAS014751	Braenderup	0	0	0	0	0	0	0	0	0	0	0
SAL_JA9112AA	PNUSAS014609	Braenderup	0	0	0	0	0	0	0	0	0	0	0
SAL_JA9124AA	PNUSAS014603	Braenderup	0	0	0	0	0	0	0	0	0	0	0
SAL_KA5764AA	PNUSAS016425	Braenderup	0	0	0	0	0	0	0	0	0	0	0
SAL_JA9086AA	PNUSAS014605	Braenderup	0	0	0	0	0	0	0	0	0	0	0
SAL_JA9088AA	PNUSAS014757	Braenderup	0	0	0	0	0	0	0	0	0	0	0
SAL_JA9090AA	PNUSAS015110	Braenderup	0	0	0	0	0	0	0	0	0	0	0
SAL_JA9096AA	PNUSAS014602	Braenderup	0	0	0	0	0	0	0	0	0	0	0
SAL_JA9141AA	PNUSAS014611	Braenderup	0	0	0	0	0	0	0	0	0	0	0
SAL_JA9122AA	PNUSAS014608	Braenderup	0	0	0	0	0	0	0	0	0	0	0
SAL_JA9097AA	PNUSAS014606	Braenderup	0	0	0	0	0	0	0	0	0	0	0
SAL_JA9038AA	PNUSAS014432	Braenderup	0	0	0	0	0	0	0	0	0	0	0
SAL_JA9262AA	PNUSAS015153	Braenderup	0	0	0	0	0	0	0	0	0	0	0
SAL_JA9138AA	PNUSAS014610	Braenderup	0	0	0	0	0	0	0	0	0	0	0
SAL_KA0175AA	OH-16-23658-1	Braenderup	0	0	0	0	0	0	0	0	0	0	0
SAL_KA0133AA	PNUSAS013791	Braenderup	0	0	0	0	0	0	0	0	0	0	0
SAL_JA9968AA	PNUSAS015143	Braenderup	0	0	0	0	0	0	0	0	0	0	0

SAL_KA0015AA	PNUSAS015915	Braenderup	0	0	0	0	0	0	0	0	0	0	0
SAL_JA9995AA	PNUSAS015788	Braenderup	0	0	0	0	0	0	0	0	0	0	0
SAL_JA9919AA	2015AM-1311	Braenderup	0	0	0	0	0	0	0	0	0	0	0
SAL_KA0068AA	PNUSAS014692	Braenderup	0	0	0	0	0	0	0	0	0	0	0
SAL_KA0125AA	PNUSAS015361	Braenderup	0	0	0	0	0	0	0	0	0	0	0
SAL_KA0095AA	PNUSAS015303	Braenderup	0	0	0	0	0	0	0	0	0	0	0
SAL_KA0781AA	PNUSAS015614	Braenderup	0	0	0	0	0	0	0	0	0	0	0
SAL_KA0787AA	PNUSAS015613	Braenderup	0	0	0	0	0	0	0	0	0	0	0
SAL_KA0796AA	PNUSAS015591	Braenderup	0	0	0	0	0	0	0	0	0	0	0
SAL_KA0514AA	PNUSAS016388	Braenderup	0	0	0	0	0	0	0	0	0	0	0
SAL_KA0535AA	PNUSAS016394	Braenderup	0	0	0	0	0	0	0	0	0	0	0
SAL_KA0452AA	PNUSAS014016	Braenderup	0	0	0	0	0	0	0	0	0	0	0
SAL_KA0441AA	PNUSAS014027	Braenderup	0	0	0	0	0	0	0	0	0	0	0
SAL_KA0361AA	PNUSAS016274	Braenderup	0	0	0	0	0	0	0	0	0	0	0
SAL_KA0362AA	PNUSAS016273	Braenderup	0	0	0	0	0	0	0	0	0	0	0
SAL_KA0303AA	PNUSAS015965	Braenderup	0	0	0	0	0	0	0	0	0	0	0
SAL_KA0283AA	FSIS1702157	Braenderup	0	0	0	0	0	0	0	0	0	0	0
SAL_KA0882AA	PNUSAS016258	Braenderup	0	0	0	0	0	0	0	0	0	0	0
SAL_KA0672AA	2015AM-0084	Braenderup	0	0	0	0	0	0	0	0	0	0	0
SAL_KA0863AA	PNUSAS014501	Braenderup	0	0	0	0	0	0	0	0	0	0	0
SAL_KA0264AA	2015AM-0650	Braenderup	0	0	0	0	0	0	0	0	0	0	0
SAL_KA1302AA	PNUSAS014408	Braenderup	0	0	0	0	0	0	0	0	0	0	0
SAL_KA1342AA	PNUSAS014805	Braenderup	0	0	0	0	0	0	0	0	0	0	0
SAL_KA1147AA	2015AM-0831	Braenderup	0	0	0	0	0	0	0	0	0	0	0
SAL_KA1189AA	2015AM-1553	Braenderup	0	0	0	0	0	0	0	0	0	0	0
SAL_KA0973AA	FSIS1701740	Braenderup	0	0	0	0	0	0	0	0	0	0	0
SAL_KA0952AA	PNUSAS016200	Braenderup	0	0	0	0	0	0	0	0	0	0	0
SAL_KA0921AA	PNUSAS016064	Braenderup	0	0	0	0	0	0	0	0	0	0	0
SAL_KA0922AA	PNUSAS016065	Braenderup	0	0	0	0	0	0	0	0	0	0	0
SAL_KA1092AA	OH-16-28037-2	Braenderup	0	0	0	0	0	0	0	0	0	0	0
SAL_KA0913AA	PNUSAS016066	Braenderup	0	0	0	0	0	0	0	0	0	0	0
SAL_KA1083AA	OH-17-06017-6	Braenderup	0	0	0	0	0	0	0	0	0	0	0
SAL_KA1110AA	2015AM-2097	Braenderup	0	0	0	0	0	0	0	0	0	0	0
SAL_KA1783AA	PNUSAS015541	Braenderup	0	0	0	0	0	0	0	0	0	0	0
SAL_KA1742AA	PNUSAS016813	Braenderup	0	0	0	0	0	0	0	0	0	0	0
SAL_KA1798AA	PNUSAS015823	Braenderup	0	0	0	0	0	0	0	0	0	0	0
SAL_KA1815AA	PNUSAS016199	Braenderup	0	0	0	0	0	0	0	0	0	0	0
SAL_KA1690AA	PNUSAS016067	Braenderup	0	0	0	0	0	0	0	0	0	0	0
SAL_KA1734AA	PNUSAS016505	Braenderup	0	0	0	0	0	0	0	0	0	0	0
SAL_KA1693AA	PNUSAS015722	Braenderup	0	0	0	0	0	0	0	0	0	0	0
SAL_KA1676AA	FSIS1702285	Braenderup	0	0	0	0	0	0	0	0	0	0	0
SAL_KA2011AA	PNUSAS015633	Braenderup	0	0	0	0	0	0	0	0	0	0	0
SAL_KA1976AA	PNUSAS016726	Braenderup	0	0	0	0	0	0	0	0	0	0	0
SAL_KA2020AA	PNUSAS014801	Braenderup	0	0	0	0	0	0	0	0	0	0	0
SAL_KA1978AA	PNUSAS016955	Braenderup	0	0	0	0	0	0	0	0	0	0	0
SAL_KA1942AA	PNUSAS015822	Braenderup	0	0	0	0	0	0	0	0	0	0	0
SAL_KA1982AA	PNUSAS016270	Braenderup	0	0	0	0	0	0	0	0	0	0	0
SAL_KA2162AA	FDA00007840	Braenderup	0	0	0	0	0	0	0	0	0	0	0
SAL_KA2790AA	PNUSAS016608	Braenderup	0	0	0	0	0	0	0	0	0	0	0
SAL_KA2787AA	PNUSAS016607	Braenderup	0	0	0	0	0	0	0	0	0	0	0
SAL_KA2724AA	PNUSAS014441	Braenderup	0	0	0	0	0	0	0	0	0	0	0
SAL_KA2675AA	PNUSAS016123	Braenderup	0	0	0	0	0	0	0	0	0	0	0
SAL_KA2621AA	PNUSAS017213	Braenderup	0	0	0	0	0	0	0	0	0	0	0
SAL_KA2608AA	PNUSAS016073	Braenderup	0	0	0	0	0	0	0	0	0	0	0
SAL_KA2610AA	PNUSAS017218	Braenderup	0	0	0	0	0	0	0	0	0	0	0
SAL_KA2415AA	FSIS1702456	Braenderup	0	0	0	0	0	0	0	0	0	0	0
SAL_KA3071AA	FSIS1702345	Braenderup	0	0	0	0	0	0	0	0	0	0	0
SAL_KA3479AA	PNUSAS017289	Braenderup	0	0	0	0	0	0	0	0	0	0	0
SAL_KA3442AA	PNUSAS017995	Braenderup	0	0	0	0	0	0	0	0	0	0	0
SAL_KA3447AA	PNUSAS017494	Braenderup	0	0	0	0	0	0	0	0	0	0	0
SAL_KA3387AA	PNUSAS015276	Braenderup	0	0	0	0	0	0	0	0	0	0	0
SAL_KA3311AA	PNUSAS017598	Braenderup	0	0	0	0	0	0	0	0	0	0	0
SAL_KA3296AA	PNUSAS014800	Braenderup	0	0	0	0	0	0	0	0	0	0	0
SAL_KA3288AA	PNUSAS017298	Braenderup	0	0	0	0	0	0	0	0	0	0	0
SAL_KA3220AA	PNUSAS015369	Braenderup	0	0	0	0	0	0	0	0	0	0	0
SAL_KA3384AA	PNUSAS015277	Braenderup	0	0	0	0	0	0	0	0	0	0	0
SAL_KA3192AA	PNUSAS017343	Braenderup	0	0	0	0	0	0	0	0	0	0	0
SAL_KA3432AA	PNUSAS017500	Braenderup	0	0	0	0	0	0	0	0	0	0	0
SAL_KA3219AA	PNUSAS015370	Braenderup	0	0	0	0	0	0	0	0	0	0	0
SAL_KA3214AA	PNUSAS015573	Braenderup	0	0	0	0	0	0	0	0	0	0	0
SAL_KA3359AA	PNUSAS016017	Braenderup	0	0	0	0	0	0	0	0	0	0	0
SAL_KA3232AA	PNUSAS017552	Braenderup	0	0	0	0	0	0	0	0	0	0	0
SAL_KA3171AA	PNUSAS016930	Braenderup	0	0	0	0	0	0	0	0	0	0	0

SAL_KA3209AA	PNUSAS015371	Braenderup	0	0	0	0	0	0	0	0	0	0	0
SAL_KA4793AA	2015AM-1126	Braenderup	0	0	0	0	0	0	0	0	0	0	0
SAL_KA4749AA	PNUSAS017695	Braenderup	0	0	0	0	0	0	0	0	0	0	0
SAL_KA4757AA	PNUSAS016779	Braenderup	0	0	0	0	0	0	0	0	0	0	0
SAL_KA4777AA	PNUSAS017426	Braenderup	0	0	0	0	0	0	0	0	0	0	0
SAL_KA4665AA	PNUSAS018008	Braenderup	0	0	0	0	0	0	0	0	0	0	0
SAL_KA4668AA	PNUSAS018015	Braenderup	0	0	0	0	0	0	0	0	0	0	0
SAL_KA4669AA	PNUSAS018011	Braenderup	0	0	0	0	0	0	0	0	0	0	0
SAL_KA4670AA	PNUSAS018010	Braenderup	0	0	0	0	0	0	0	0	0	0	0
SAL_KA4656AA	PNUSAS017074	Braenderup	0	0	0	0	0	0	0	0	0	0	0
SAL_KA4658AA	PNUSAS018009	Braenderup	0	0	0	0	0	0	0	0	0	0	0
SAL_KA4640AA	PNUSAS018021	Braenderup	0	0	0	0	0	0	0	0	0	0	0
SAL_KA4648AA	PNUSAS018018	Braenderup	0	0	0	0	0	0	0	0	0	0	0
SAL_KA4587AA	FSIS1702433	Braenderup	0	0	0	0	0	0	0	0	0	0	0
SAL_KA4673AA	PNUSAS018351	Braenderup	0	0	0	0	0	0	0	0	0	0	0
SAL_KA4702AA	PNUSAS017567	Braenderup	0	0	0	0	0	0	0	0	0	0	0
SAL_KA4693AA	PNUSAS017568	Braenderup	0	0	0	0	0	0	0	0	0	0	0
SAL_KA4128AA	H103540792	Braenderup	0	0	0	0	0	0	0	0	0	0	0
SAL_KA4965AA	PNUSAS017769	Braenderup	0	0	0	0	0	0	0	0	0	0	0
SAL_KA4949AA	PNUSAS016611	Braenderup	0	0	0	0	0	0	0	0	0	0	0
SAL_KA4956AA	PNUSAS017913	Braenderup	0	0	0	0	0	0	0	0	0	0	0
SAL_KA4964AA	PNUSAS017898	Braenderup	0	0	0	0	0	0	0	0	0	0	0
SAL_KA4928AA	PNUSAS017921	Braenderup	0	0	0	0	0	0	0	0	0	0	0
SAL_KA4910AA	2015AM-1252	Braenderup	0	0	0	0	0	0	0	0	0	0	0
SAL_KA5044AA	FDA00007842	Braenderup	0	0	0	0	0	0	0	0	0	0	0
SAL_KA5040AA	CFSAN065047	Braenderup	0	0	0	0	0	0	0	0	0	0	0
SAL_KA4993AA	2015AM-1207	Braenderup	0	0	0	0	0	0	0	0	0	0	0
SAL_KA5093AA	16-9-20170570	Braenderup	0	0	0	0	0	0	0	0	0	0	0
SAL_KA5579AA	FSIS1702947	Braenderup	0	0	0	0	0	0	0	0	0	0	0
SAL_KA5542AA	PNUSAS018263	Braenderup	0	0	0	0	0	0	0	0	0	0	0
SAL_KA5532AA	PNUSAS018267	Braenderup	0	0	0	0	0	0	0	0	0	0	0
SAL_KA5525AA	PNUSAS018497	Braenderup	0	0	0	0	0	0	0	0	0	0	0
SAL_KA5527AA	PNUSAS018880	Braenderup	0	0	0	0	0	0	0	0	0	0	0
SAL_KA5510AA	PNUSAS018855	Braenderup	0	0	0	0	0	0	0	0	0	0	0
SAL_KA5431AA	PNUSAS017750	Braenderup	0	0	0	0	0	0	0	0	0	0	0
SAL_KA5395AA	PNUSAS018026	Braenderup	0	0	0	0	0	0	0	0	0	0	0
SAL_KA5379AA	PNUSAS017648	Braenderup	0	0	0	0	0	0	0	0	0	0	0
SAL_KA5385AA	PNUSAS014818	Braenderup	0	0	0	0	0	0	0	0	0	0	0
SAL_KA5337AA	PNUSAS014815	Braenderup	0	0	0	0	0	0	0	0	0	0	0
SAL_KA5283AA	PNUSAS017749	Braenderup	0	0	0	0	0	0	0	0	0	0	0
SAL_KA5274AA	PNUSAS015064	Braenderup	0	0	0	0	0	0	0	0	0	0	0
SAL_KA5358AA	PNUSAS018554	Braenderup	0	0	0	0	0	0	0	0	0	0	0
SAL_KA5435AA	PNUSAS018553	Braenderup	0	0	0	0	0	0	0	0	0	0	0
SAL_KA5223AA	PNUSAS018161	Braenderup	0	0	0	0	0	0	0	0	0	0	0
SAL_KA5180AA	PNUSAS018043	Braenderup	0	0	0	0	0	0	0	0	0	0	0
SAL_KA5189AA	PNUSAS018749	Braenderup	0	0	0	0	0	0	0	0	0	0	0
SAL_KA5143AA	FSIS1702988	Braenderup	0	0	0	0	0	0	0	0	0	0	0
SAL_KA5152AA	PNUSAS018733	Braenderup	0	0	0	0	0	0	0	0	0	0	0
SAL_KA5158AA	PNUSAS018304	Braenderup	0	0	0	0	0	0	0	0	0	0	0
SAL_KA5125AA	PNUSAS018738	Braenderup	0	0	0	0	0	0	0	0	0	0	0
SAL_KA5742AA	FSIS1702992	Braenderup	0	0	0	0	0	0	0	0	0	0	0
SAL_KA5620AA	PNUSAS018041	Braenderup	0	0	0	0	0	0	0	0	0	0	0
SAL_KA5629AA	PNUSAS017373	Braenderup	0	0	0	0	0	0	0	0	0	0	0
SAL_KA5637AA	PNUSAS017371	Braenderup	0	0	0	0	0	0	0	0	0	0	0
SAL_KA5650AA	2015AM-0495	Braenderup	0	0	0	0	0	0	0	0	0	0	0
SAL_KA5614AA	PNUSAS018042	Braenderup	0	0	0	0	0	0	0	0	0	0	0
SAL_KA5616AA	PNUSAS019101	Braenderup	0	0	0	0	0	0	0	0	0	0	0
SAL_KA5709AA	2015AM-1208	Braenderup	0	0	0	0	0	0	0	0	0	0	0
SAL_KA5621AA	PNUSAS018039	Braenderup	0	0	0	0	0	0	0	0	0	0	0
SAL_KA6139AA	2015AM-0198	Braenderup	0	0	0	0	0	0	0	0	0	0	0
SAL_KA6082AA	CFSAN065548	Braenderup	0	0	0	0	0	0	0	0	0	0	0
SAL_KA5998AA	PNUSAS017629	Braenderup	0	0	0	0	0	0	0	0	0	0	0
SAL_KA6291AA	FSIS1703103	Braenderup	0	0	0	0	0	0	0	0	0	0	0
SAL_KA6400AA	PNUSAS018044	Braenderup	0	0	0	0	0	0	0	0	0	0	0
SAL_KA6404AA	PNUSAS018054	Braenderup	0	0	0	0	0	0	0	0	0	0	0
SAL_KA6403AA	PNUSAS018045	Braenderup	0	0	0	0	0	0	0	0	0	0	0
SAL_KA6407AA	PNUSAS019637	Braenderup	0	0	0	0	0	0	0	0	0	0	0
SAL_KA6356AA	PNUSAS019122	Braenderup	0	0	0	0	0	0	0	0	0	0	0
SAL_KA6346AA	PNUSAS019135	Braenderup	0	0	0	0	0	0	0	0	0	0	0
SAL_KA7053AA	PNUSAS017908	Braenderup	0	0	0	0	0	0	0	0	0	0	0
SAL_KA7006AA	PNUSAS018320	Braenderup	0	0	0	0	0	0	0	0	0	0	0
SAL_KA7004AA	PNUSAS018324	Braenderup	0	0	0	0	0	0	0	0	0	0	0
SAL_KA6977AA	PNUSAS019075	Braenderup	0	0	0	0	0	0	0	0	0	0	0

SAL_KA7017AA	PNUSAS018900	Braenderup	0	0	0	0	0	0	0	0	0	0	0
SAL_KA6902AA	PNUSAS017372	Braenderup	0	0	0	0	0	0	0	0	0	0	0
SAL_KA6896AA	PNUSAS016603	Braenderup	0	0	0	0	0	0	0	0	0	0	0
SAL_KA6901AA	PNUSAS017125	Braenderup	0	0	0	0	0	0	0	0	0	0	0
SAL_KA6907AA	PNUSAS016597	Braenderup	0	0	0	0	0	0	0	0	0	0	0
SAL_KA6831AA	PNUSAS018040	Braenderup	0	0	0	0	0	0	0	0	0	0	0
SAL_KA6819AA	PNUSAS015065	Braenderup	0	0	0	0	0	0	0	0	0	0	0
SAL_KA6821AA	PNUSAS015066	Braenderup	0	0	0	0	0	0	0	0	0	0	0
SAL_KA6905AA	PNUSAS017126	Braenderup	0	0	0	0	0	0	0	0	0	0	0
SAL_KA6852AA	PNUSAS017513	Braenderup	0	0	0	0	0	0	0	0	0	0	0
SAL_KA6714AA	PNUSAS019178	Braenderup	0	0	0	0	0	0	0	0	0	0	0
SAL_KA6785AA	PNUSAS013056	Braenderup	0	0	0	0	0	0	0	0	0	0	0
SAL_KA6979AA	PNUSAS018832	Braenderup	0	0	0	0	0	0	0	0	0	0	0
SAL_KA6630AA	PNUSAS018825	Braenderup	0	0	0	0	0	0	0	0	0	0	0
SAL_KA6575AA	PNUSAS019155	Braenderup	0	0	0	0	0	0	0	0	0	0	0
SAL_KA6631AA	PNUSAS018824	Braenderup	0	0	0	0	0	0	0	0	0	0	0
SAL_KA6583AA	PNUSAS019689	Braenderup	0	0	0	0	0	0	0	0	0	0	0
SAL_KA6462AA	2015AM-1459	Braenderup	0	0	0	0	0	0	0	0	0	0	0
SAL_KA7125AA	PNUSAS014981	Braenderup	0	0	0	0	0	0	0	0	0	0	0
SAL_KA7617AA	PNUSAS020051	Braenderup	0	0	0	0	0	0	0	0	0	0	0
SAL_KA7629AA	PNUSAS019525	Braenderup	0	0	0	0	0	0	0	0	0	0	0
SAL_KA7593AA	PNUSAS020176	Braenderup	0	0	0	0	0	0	0	0	0	0	0
SAL_KA7581AA	PNUSAS019043	Braenderup	0	0	0	0	0	0	0	0	0	0	0
SAL_KA7434AA	PNUSAS018060	Braenderup	0	0	0	0	0	0	0	0	0	0	0
SAL_KA7439AA	PNUSAS018693	Braenderup	0	0	0	0	0	0	0	0	0	0	0
SAL_KA7444AA	PNUSAS018061	Braenderup	0	0	0	0	0	0	0	0	0	0	0
SAL_KA7449AA	PNUSAS018063	Braenderup	0	0	0	0	0	0	0	0	0	0	0
SAL_KA7429AA	PNUSAS018474	Braenderup	0	0	0	0	0	0	0	0	0	0	0
SAL_KA7433AA	PNUSAS018692	Braenderup	0	0	0	0	0	0	0	0	0	0	0
SAL_KA7437AA	PNUSAS018062	Braenderup	0	0	0	0	0	0	0	0	0	0	0
SAL_KA7354AA	PNUSAS019225	Braenderup	0	0	0	0	0	0	0	0	0	0	0
SAL_KA7391AA	PNUSAS018478	Braenderup	0	0	0	0	0	0	0	0	0	0	0
SAL_KA7274AA	PNUSAS018087	Braenderup	0	0	0	0	0	0	0	0	0	0	0
SAL_KA7676AA	FSIS1703450	Braenderup	0	0	0	0	0	0	0	0	0	0	0
SAL_KA8045AA	PNUSAS020078	Braenderup	0	0	0	0	0	0	0	0	0	0	0
SAL_KA8005AA	PNUSAS016782	Braenderup	0	0	0	0	0	0	0	0	0	0	0
SAL_KA7968AA	PNUSAS020259	Braenderup	0	0	0	0	0	0	0	0	0	0	0
SAL_KA7948AA	PNUSAS019411	Braenderup	0	0	0	0	0	0	0	0	0	0	0
SAL_KA7950AA	PNUSAS019410	Braenderup	0	0	0	0	0	0	0	0	0	0	0
SAL_KA7816AA	2017K-0603	Braenderup	0	0	0	0	0	0	0	0	0	0	0
SAL_KA7828AA	PNUSAS020160	Braenderup	0	0	0	0	0	0	0	0	0	0	0
SAL_KA7831AA	PNUSAS015490	Braenderup	0	0	0	0	0	0	0	0	0	0	0
SAL_KA7763AA	PNUSAS020211	Braenderup	0	0	0	0	0	0	0	0	0	0	0
SAL_KA8137AA	2015AM-0621	Braenderup	0	0	0	0	0	0	0	0	0	0	0
SAL_KA8193AA	2015AM-0997	Braenderup	0	0	0	0	0	0	0	0	0	0	0
SAL_KA8272AA	PNUSAS020025	Braenderup	0	0	0	0	0	0	0	0	0	0	0
SAL_KA8224AA	PNUSAS019543	Braenderup	0	0	0	0	0	0	0	0	0	0	0
SAL_KA8505AA	PNUSAS020757	Braenderup	0	0	0	0	0	0	0	0	0	0	0
SAL_KA8504AA	PNUSAS020762	Braenderup	0	0	0	0	0	0	0	0	0	0	0
SAL_KA8480AA	PNUSAS018641	Braenderup	0	0	0	0	0	0	0	0	0	0	0
SAL_KA8462AA	PNUSAS018646	Braenderup	0	0	0	0	0	0	0	0	0	0	0
SAL_KA8444AA	PNUSAS018675	Braenderup	0	0	0	0	0	0	0	0	0	0	0
SAL_KA8645AA	PNUSAS020364	Braenderup	0	0	0	0	0	0	0	0	0	0	0
SAL_KA8379AA	2015AM-1867	Braenderup	0	0	0	0	0	0	0	0	0	0	0
SAL_KA8391AA	2015AM-2128	Braenderup	0	0	0	0	0	0	0	0	0	0	0
SAL_KA8377AA	2015AM-0474	Braenderup	0	0	0	0	0	0	0	0	0	0	0
SAL_KA8583AA	PNUSAS019660	Braenderup	0	0	0	0	0	0	0	0	0	0	0
SAL_KA8609AA	PNUSAS020688	Braenderup	0	0	0	0	0	0	0	0	0	0	0
SAL_KA8521AA	PNUSAS020057	Braenderup	0	0	0	0	0	0	0	0	0	0	0
SAL_KA8546AA	PNUSAA018552	Braenderup	0	0	0	0	0	0	0	0	0	0	0
SAL_KA9104AA	PNUSAS019986	Braenderup	0	0	0	0	0	0	0	0	0	0	0
SAL_KA9105AA	PNUSAS019987	Braenderup	0	0	0	0	0	0	0	0	0	0	0
SAL_KA9092AA	PNUSAS019984	Braenderup	0	0	0	0	0	0	0	0	0	0	0
SAL_KA8904AA	PNUSAS019931	Braenderup	0	0	0	0	0	0	0	0	0	0	0
SAL_KA9024AA	PNUSAS019979	Braenderup	0	0	0	0	0	0	0	0	0	0	0
SAL_KA8596AA	PNUSAS019482	Braenderup	0	0	0	0	0	0	0	0	0	0	0
SAL_KA9222AA	2015AM-0683	Braenderup	0	0	0	0	0	0	0	0	0	0	0
SAL_KA8900AA	PNUSAS019384	Braenderup	0	0	0	0	0	0	0	0	0	0	0
SAL_KA9706AA	PNUSAS018131	Braenderup	0	0	0	0	0	0	0	0	0	0	0
SAL_KA9695AA	PNUSAS019640	Braenderup	0	0	0	0	0	0	0	0	0	0	0
SAL_KA9697AA	PNUSAS019638	Braenderup	0	0	0	0	0	0	0	0	0	0	0
SAL_KA9699AA	PNUSAS019004	Braenderup	0	0	0	0	0	0	0	0	0	0	0
SAL_KA9692AA	PNUSAS019639	Braenderup	0	0	0	0	0	0	0	0	0	0	0

SAL_KA9660AA	TX-ACF1701983S	Braenderup	0	0	0	0	0	0	0	0	0	0	0
SAL_KA9634AA	PNUSAS020804	Braenderup	0	0	0	0	0	0	0	0	0	0	0
SAL_KA9637AA	PNUSAS020807	Braenderup	0	0	0	0	0	0	0	0	0	0	0
SAL_KA9594AA	PNUSAS019120	Braenderup	0	0	0	0	0	0	0	0	0	0	0
SAL_KA9532AA	PNUSAS020772	Braenderup	0	0	0	0	0	0	0	0	0	0	0
SAL_KA9540AA	CFSAN067282	Braenderup	0	0	0	0	0	0	0	0	0	0	0
SAL_KA9525AA	PNUSAS019641	Braenderup	0	0	0	0	0	0	0	0	0	0	0
SAL_KA9523AA	PNUSAS019642	Braenderup	0	0	0	0	0	0	0	0	0	0	0
SAL_KA9518AA	PNUSAS019644	Braenderup	0	0	0	0	0	0	0	0	0	0	0
SAL_KA9519AA	PNUSAS019643	Braenderup	0	0	0	0	0	0	0	0	0	0	0
SAL_KA9503AA	CFSAN067283	Braenderup	0	0	0	0	0	0	0	0	0	0	0
SAL_KA9381AA	CFSAN067288	Braenderup	0	0	0	0	0	0	0	0	0	0	0
SAL_KA9384AA	CFSAN067294	Braenderup	0	0	0	0	0	0	0	0	0	0	0
SAL_KA9393AA	CFSAN067289	Braenderup	0	0	0	0	0	0	0	0	0	0	0
SAL_KA9291AA	CFSAN067297	Braenderup	0	0	0	0	0	0	0	0	0	0	0
SAL_KA9352AA	2017K-0678	Braenderup	0	0	0	0	0	0	0	0	0	0	0
SAL_KA7628AA	PNUSAS019524	Braenderup	0	0	0	0	0	0	0	0	0	0	0
SAL_KA9985AA	PNUSAS021015	Braenderup	0	0	0	0	0	0	0	0	0	0	0
SAL_KA9977AA	PNUSAS020041	Braenderup	0	0	0	0	0	0	0	0	0	0	0
SAL_KA9897AA	FSIS1703506	Braenderup	0	0	0	0	0	0	0	0	0	0	0
SAL_KA9883AA	PNUSAS020549	Braenderup	0	0	0	0	0	0	0	0	0	0	0
SAL_KA9959AA	PNUSAS021002	Braenderup	0	0	0	0	0	0	0	0	0	0	0
SAL_LA0295AA	PNUSAS021552	Braenderup	0	0	0	0	0	0	0	0	0	0	0
SAL_LA0271AA	PNUSAS021510	Braenderup	0	0	0	0	0	0	0	0	0	0	0
SAL_LA0304AA	PNUSAS021102	Braenderup	0	0	0	0	0	0	0	0	0	0	0
SAL_LA0235AA	PNUSAS020200	Braenderup	0	0	0	0	0	0	0	0	0	0	0
SAL_LA0256AA	PNUSAS020583	Braenderup	0	0	0	0	0	0	0	0	0	0	0
SAL_LA0215AA	2015AM-1755	Braenderup	0	0	0	0	0	0	0	0	0	0	0
SAL_LA0183AA	2015AM-0515	Braenderup	0	0	0	0	0	0	0	0	0	0	0
SAL_LA0263AA	PNUSAS019051	Braenderup	0	0	0	0	0	0	0	0	0	0	0
SAL_LA0360AA	FDA00011972	Braenderup	0	0	0	0	0	0	0	0	0	0	0
SAL_LA0493AA	PNUSAS019580	Braenderup	0	0	0	0	0	0	0	0	0	0	0
SAL_LA0437AA	PNUSAS019970	Braenderup	0	0	0	0	0	0	0	0	0	0	0
SAL_LA0622AA	FSIS1703689	Braenderup	0	0	0	0	0	0	0	0	0	0	0
SAL_LA0609AA	FSIS11703972	Braenderup	0	0	0	0	0	0	0	0	0	0	0
SAL_LA0613AA	FSIS11703971	Braenderup	0	0	0	0	0	0	0	0	0	0	0
SAL_LA0721AA	PNUSAS020917	Braenderup	0	0	0	0	0	0	0	0	0	0	0
SAL_LA1077AA	PNUSAS021678	Braenderup	0	0	0	0	0	0	0	0	0	0	0
SAL_LA1337AA	NSUOC-0187-S6-2H	Braenderup	0	0	0	0	0	0	0	0	0	0	0
SAL_LA1236AA	PNUSAS021732	Braenderup	0	0	0	0	0	0	0	0	0	0	0
SAL_LA1474AA	FSIS21720092	Braenderup	0	0	0	0	0	0	0	0	0	0	0
SAL_LA1477AA	PNUSAS021611	Braenderup	0	0	0	0	0	0	0	0	0	0	0
SAL_LA2290AA	PNUSAS021447	Braenderup	0	0	0	0	0	0	0	0	0	0	0
SAL_LA2230AA	PNUSAS020992	Braenderup	0	0	0	0	0	0	0	0	0	0	0
SAL_LA2294AA	PNUSAS021887	Braenderup	0	0	0	0	0	0	0	0	0	0	0
SAL_LA2295AA	PNUSAS021432	Braenderup	0	0	0	0	0	0	0	0	0	0	0
SAL_LA2184AA	PNUSAS021591	Braenderup	0	0	0	0	0	0	0	0	0	0	0
SAL_LA3321AA	PNUSAS021874	Braenderup	0	0	0	0	0	0	0	0	0	0	0
SAL_LA3291AA	PNUSAS021981	Braenderup	0	0	0	0	0	0	0	0	0	0	0
SAL_LA3848AA	PNUSAS022180	Braenderup	0	0	0	0	0	0	0	0	0	0	0
SAL_LA3792AA	PNUSAS021910	Braenderup	0	0	0	0	0	0	0	0	0	0	0
SAL_LA3775AA	PNUSAS022145	Braenderup	0	0	0	0	0	0	0	0	0	0	0
SAL_LA3777AA	PNUSAS022384	Braenderup	0	0	0	0	0	0	0	0	0	0	0
SAL_LA3871AA	PNUSAS021971	Braenderup	0	0	0	0	0	0	0	0	0	0	0
SAL_LA3807AA	PNUSAS021933	Braenderup	0	0	0	0	0	0	0	0	0	0	0
SAL_LA3729AA	PNUSAS021155	Braenderup	0	0	0	0	0	0	0	0	0	0	0
SAL_LA3715AA	PNUSAS021153	Braenderup	0	0	0	0	0	0	0	0	0	0	0
SAL_LA3719AA	PNUSAS021151	Braenderup	0	0	0	0	0	0	0	0	0	0	0
SAL_LA3742AA	PNUSAS020278	Braenderup	0	0	0	0	0	0	0	0	0	0	0
SAL_LA3951AA	PNUSAS021900	Braenderup	0	0	0	0	0	0	0	0	0	0	0
SAL_LA3902AA	PNUSAS021902	Braenderup	0	0	0	0	0	0	0	0	0	0	0
SAL_LA3916AA	PNUSAS021901	Braenderup	0	0	0	0	0	0	0	0	0	0	0
SAL_LA4279AA	PNUSAS021677	Braenderup	0	0	0	0	0	0	0	0	0	0	0
SAL_LA4312AA	PNUSAS021888	Braenderup	0	0	0	0	0	0	0	0	0	0	0
SAL_LA4317AA	PNUSAS021440	Braenderup	0	0	0	0	0	0	0	0	0	0	0
SAL_LA4174AA	PNUSAS020478	Braenderup	0	0	0	0	0	0	0	0	0	0	0
SAL_LA4207AA	PNUSAS022382	Braenderup	0	0	0	0	0	0	0	0	0	0	0
SAL_LA4294AA	PNUSAS022831	Braenderup	0	0	0	0	0	0	0	0	0	0	0
SAL_LA4104AA	PNUSAS022415	Braenderup	0	0	0	0	0	0	0	0	0	0	0
SAL_LA5026AA	PNUSAS023186	Braenderup	0	0	0	0	0	0	0	0	0	0	0
SAL_LA5027AA	PNUSAS022275	Braenderup	0	0	0	0	0	0	0	0	0	0	0
SAL_LA5009AA	PNUSAS019507	Braenderup	0	0	0	0	0	0	0	0	0	0	0
SAL_LA4961AA	PNUSAS023083	Braenderup	0	0	0	0	0	0	0	0	0	0	0

SAL_LA5030AA	PNUSAS022285	Braenderup	0	0	0	0	0	0	0	0	0	0	0
SAL_LA4901AA	PNUSAS021897	Braenderup	0	0	0	0	0	0	0	0	0	0	0
SAL_LA4909AA	PNUSAS020476	Braenderup	0	0	0	0	0	0	0	0	0	0	0
SAL_LA4891AA	PNUSAS019453	Braenderup	0	0	0	0	0	0	0	0	0	0	0
SAL_LA4881AA	PNUSAS022020	Braenderup	0	0	0	0	0	0	0	0	0	0	0
SAL_LA4879AA	PNUSAS022021	Braenderup	0	0	0	0	0	0	0	0	0	0	0
SAL_LA4863AA	PNUSAS022964	Braenderup	0	0	0	0	0	0	0	0	0	0	0
SAL_LA4791AA	FSIS11704362	Braenderup	0	0	0	0	0	0	0	0	0	0	0
SAL_LA5014AA	PNUSAS019508	Braenderup	0	0	0	0	0	0	0	0	0	0	0
SAL_LA4976AA	PNUSAS022844	Braenderup	0	0	0	0	0	0	0	0	0	0	0
SAL_LA5084AA	2017K-0712	Braenderup	0	0	0	0	0	0	0	0	0	0	0
SAL_LA5088AA	2017K-0710	Braenderup	0	0	0	0	0	0	0	0	0	0	0
SAL_LA5632AA	20-23-20170799	Braenderup	0	0	0	0	0	0	0	0	0	0	0
SAL_LA6962AA	22-9-20170880	Braenderup	0	0	0	0	0	0	0	0	0	0	0
SAL_LA6845AA	PNUSAS022978	Braenderup	0	0	0	0	0	0	0	0	0	0	0
SAL_LA6615AA	PNUSAS022515	Braenderup	0	0	0	0	0	0	0	0	0	0	0
SAL_LA6570AA	PNUSAS023394	Braenderup	0	0	0	0	0	0	0	0	0	0	0
SAL_LA6511AA	PNUSAS022904	Braenderup	0	0	0	0	0	0	0	0	0	0	0
SAL_LA6505AA	PNUSAS023626	Braenderup	0	0	0	0	0	0	0	0	0	0	0
SAL_LA6517AA	PNUSAS023620	Braenderup	0	0	0	0	0	0	0	0	0	0	0
SAL_LA6385AA	PNUSAS023292	Braenderup	0	0	0	0	0	0	0	0	0	0	0
SAL_LA6373AA	PNUSAS022045	Braenderup	0	0	0	0	0	0	0	0	0	0	0
SAL_LA6345AA	PNUSAS024124	Braenderup	0	0	0	0	0	0	0	0	0	0	0
SAL_LA7453AA	PNUSAS023674	Braenderup	0	0	0	0	0	0	0	0	0	0	0
SAL_LA7468AA	PNUSAS021249	Braenderup	0	0	0	0	0	0	0	0	0	0	0
SAL_LA7382AA	PNUSAS013121	Braenderup	0	0	0	0	0	0	0	0	0	0	0
SAL_LA9941AA	24-23-20171005	Braenderup	0	0	0	0	0	0	0	0	0	0	0
SAL_LA8192AA	FSIS21720258	Braenderup	0	0	0	0	0	0	0	0	0	0	0
SAL_LA8196AA	FSIS21720254	Braenderup	0	0	0	0	0	0	0	0	0	0	0
SAL_LA8202AA	FSIS21720248	Braenderup	0	0	0	0	0	0	0	0	0	0	0
SAL_LA8172AA	FDA00007844	Braenderup	0	0	0	0	0	0	0	0	0	0	0
SAL_LA9790AA	PNUSAS022858	Braenderup	0	0	0	0	0	0	0	0	0	0	0
SAL_LA9807AA	PNUSAS022235	Braenderup	0	0	0	0	0	0	0	0	0	0	0
SAL_LA9774AA	PNUSAS022873	Braenderup	0	0	0	0	0	0	0	0	0	0	0
SAL_LA9723AA	PNUSAS022033	Braenderup	0	0	0	0	0	0	0	0	0	0	0
SAL_LA9720AA	PNUSAS022035	Braenderup	0	0	0	0	0	0	0	0	0	0	0
SAL_LA9726AA	PNUSAS022032	Braenderup	0	0	0	0	0	0	0	0	0	0	0
SAL_LA9309AA	PNUSAS024340	Braenderup	0	0	0	0	0	0	0	0	0	0	0
SAL_LA9310AA	PNUSAS022010	Braenderup	0	0	0	0	0	0	0	0	0	0	0
SAL_LA9152AA	PNUSAS023633	Braenderup	0	0	0	0	0	0	0	0	0	0	0
SAL_LA9073AA	PNUSAS024425	Braenderup	0	0	0	0	0	0	0	0	0	0	0
SAL_LA9038AA	PNUSAS024437	Braenderup	0	0	0	0	0	0	0	0	0	0	0
SAL_LA9042AA	PNUSAS024438	Braenderup	0	0	0	0	0	0	0	0	0	0	0
SAL_LA9057AA	PNUSAS024434	Braenderup	0	0	0	0	0	0	0	0	0	0	0
SAL_MA0200AA	PNUSAS022692	Braenderup	0	0	0	0	0	0	0	0	0	0	0
SAL_MA0181AA	PNUSAS024393	Braenderup	0	0	0	0	0	0	0	0	0	0	0
SAL_MA0090AA	PNUSAS023476	Braenderup	0	0	0	0	0	0	0	0	0	0	0
SAL_MA0088AA	PNUSAS023478	Braenderup	0	0	0	0	0	0	0	0	0	0	0
SAL_MA0003AA	PNUSAS024625	Braenderup	0	0	0	0	0	0	0	0	0	0	0
SAL_MA0486AA	PNUSAS024504	Braenderup	0	0	0	0	0	0	0	0	0	0	0
SAL_MA0392AA	PNUSAS024170	Braenderup	0	0	0	0	0	0	0	0	0	0	0
SAL_MA0369AA	PNUSAS018076	Braenderup	0	0	0	0	0	0	0	0	0	0	0
SAL_MA0395AA	PNUSAS024174	Braenderup	0	0	0	0	0	0	0	0	0	0	0
SAL_MA0340AA	FSIS11704674	Braenderup	0	0	0	0	0	0	0	0	0	0	0
SAL_MA0947AA	PNUSAS025536	Braenderup	0	0	0	0	0	0	0	0	0	0	0
SAL_MA0901AA	PNUSAS025314	Braenderup	0	0	0	0	0	0	0	0	0	0	0
SAL_MA1380AA	PNUSAS025702	Braenderup	0	0	0	0	0	0	0	0	0	0	0
SAL_MA1361AA	PNUSAS025701	Braenderup	0	0	0	0	0	0	0	0	0	0	0
SAL_MA1319AA	FSIS11704698	Braenderup	0	0	0	0	0	0	0	0	0	0	0
SAL_MA1296AA	CFSAN070272	Braenderup	0	0	0	0	0	0	0	0	0	0	0
SAL_MA1282AA	B1715404	Braenderup	0	0	0	0	0	0	0	0	0	0	0
SAL_MA1602AA	PNUSAS025882	Braenderup	0	0	0	0	0	0	0	0	0	0	0
SAL_MA1657AA	PNUSAS025318	Braenderup	0	0	0	0	0	0	0	0	0	0	0
SAL_MA1600AA	PNUSAS025941	Braenderup	0	0	0	0	0	0	0	0	0	0	0
SAL_MA1705AA	PNUSAS026536	Braenderup	0	0	0	0	0	0	0	0	0	0	0
SAL_MA2241AA	FSIS21720416	Braenderup	0	0	0	0	0	0	0	0	0	0	0
SAL_MA2566AA	SSI-AC392	Braenderup	0	0	0	0	0	0	0	0	0	0	0
SAL_MA2738AA	PNUSAS026565	Braenderup	0	0	0	0	0	0	0	0	0	0	0
SAL_MA2783AA	PNUSAS026545	Braenderup	0	0	0	0	0	0	0	0	0	0	0
SAL_MA2627AA	PNUSAS023414	Braenderup	0	0	0	0	0	0	0	0	0	0	0
SAL_MA2629AA	PNUSAS023411	Braenderup	0	0	0	0	0	0	0	0	0	0	0
SAL_MA2906AA	PNUSAS025710	Braenderup	0	0	0	0	0	0	0	0	0	0	0
SAL_MA2890AA	PNUSAS025022	Braenderup	0	0	0	0	0	0	0	0	0	0	0

SAL_MA2825AA	PNUSAS025556	Braenderup	0	0	0	0	0	0	0	0	0	0	0
SAL_MA3378AA	PNUSAS024543	Braenderup	0	0	0	0	0	0	0	0	0	0	0
SAL_MA3325AA	PNUSAS026419	Braenderup	0	0	0	0	0	0	0	0	0	0	0
SAL_MA3346AA	PNUSAS024818	Braenderup	0	0	0	0	0	0	0	0	0	0	0
SAL_MA3374AA	PNUSAS024293	Braenderup	0	0	0	0	0	0	0	0	0	0	0
SAL_MA3329AA	PNUSAS026426	Braenderup	0	0	0	0	0	0	0	0	0	0	0
SAL_MA3305AA	PNUSAS026634	Braenderup	0	0	0	0	0	0	0	0	0	0	0
SAL_MA3298AA	PNUSAS025239	Braenderup	0	0	0	0	0	0	0	0	0	0	0
SAL_MA3136AA	34936	Braenderup	0	0	0	0	0	0	0	0	0	0	0
SAL_MA3455AA	PNUSAS026382	Braenderup	0	0	0	0	0	0	0	0	0	0	0
SAL_MA3516AA	PNUSAS026488	Braenderup	0	0	0	0	0	0	0	0	0	0	0
SAL_MA3561AA	26-4-20171074	Braenderup	0	0	0	0	0	0	0	0	0	0	0
SAL_MA3615AA	PNUSAS024783	Braenderup	0	0	0	0	0	0	0	0	0	0	0
SAL_MA3853AA	PNUSAS026143	Braenderup	0	0	0	0	0	0	0	0	0	0	0
SAL_MA4752AA	PNUSAS027097	Braenderup	0	0	0	0	0	0	0	0	0	0	0
SAL_MA4639AA	PNUSAS026367	Braenderup	0	0	0	0	0	0	0	0	0	0	0
SAL_MA4605AA	PNUSAS025210	Braenderup	0	0	0	0	0	0	0	0	0	0	0
SAL_MA4642AA	PNUSAS027084	Braenderup	0	0	0	0	0	0	0	0	0	0	0
SAL_MA4584AA	PNUSAS025256	Braenderup	0	0	0	0	0	0	0	0	0	0	0
SAL_MA4602AA	PNUSAS024365	Braenderup	0	0	0	0	0	0	0	0	0	0	0
SAL_MA4546AA	PNUSAS026843	Braenderup	0	0	0	0	0	0	0	0	0	0	0
SAL_MA4472AA	PNUSAS026697	Braenderup	0	0	0	0	0	0	0	0	0	0	0
SAL_MA4466AA	PNUSAS026685	Braenderup	0	0	0	0	0	0	0	0	0	0	0
SAL_MA4992AA	PNUSAS027264	Braenderup	0	0	0	0	0	0	0	0	0	0	0
SAL_MA4958AA	PNUSAS027121	Braenderup	0	0	0	0	0	0	0	0	0	0	0
SAL_MA4754AA	FSIS21720484	Braenderup	0	0	0	0	0	0	0	0	0	0	0
SAL_MA5169AA	PNUSAS026967	Braenderup	0	0	0	0	0	0	0	0	0	0	0
SAL_MA5122AA	PNUSAS027619	Braenderup	0	0	0	0	0	0	0	0	0	0	0
SAL_BA1982AA	FDA00004500	Braenderup	0	0	0	0	0	0	0	0	0	0	0
SAL_CA0518AA	68670	Braenderup	0	0	0	0	0	0	0	0	0	0	0
SAL_DA3426AA	IEH-NGS-SAL-01260	Braenderup	0	0	0	0	0	0	0	0	0	0	0
SAL_BA1212AA	CFSAN030817	Braenderup	0	0	0	0	0	0	0	0	0	0	0
SAL_CA3539AA	90172	Braenderup	0	0	0	0	0	0	0	0	0	0	0
SAL_CA4696AA	69276	Braenderup	0	0	0	0	0	0	0	0	0	0	0
SAL_CA5468AA	78752	Braenderup	0	0	0	0	0	0	0	0	0	0	0
SAL_CA9739AA	2014K-0940	Braenderup	0	0	0	0	0	0	0	0	0	0	0
SAL_DA2008AA	VA-WGS-00330	Braenderup	0	0	0	0	0	0	0	0	0	0	0
SAL_BA6379AA	09:MF0010690R	Braenderup	0	0	0	0	0	0	0	0	0	0	0
SAL_CA1813AA	38404	Braenderup	0	0	0	0	0	0	0	0	0	0	0
SAL_CA5364AA	80354	Braenderup	0	0	0	0	0	0	0	0	0	0	0
SAL_EA8587AA	POR1-0098	Braenderup	0	0	0	0	0	0	0	0	0	0	0
SAL_BA8059AA	ATCC 10708	Braenderup	0	0	0	0	0	0	0	0	0	0	0
SAL_BA9915AA	:-2012-07-27T11:53:04Z-	Braenderup	0	0	0	0	0	0	0	0	0	0	0
SAL_HA5959AA	CFSAN056577	Braenderup	0	0	0	0	0	0	0	0	0	0	0
SAL_GA4418AA	PNUSAS002627	Braenderup	0	0	0	0	0	0	0	0	0	0	0
SAL_HA8733AA	CFSAN058077	Braenderup	0	0	0	0	0	0	0	0	0	0	0
SAL_FA7532AA	240208	Braenderup	0	0	0	0	0	0	0	0	0	0	0
SAL_EA6863AA	FDA00007831	Braenderup	0	0	0	0	0	0	0	0	0	0	0
SAL_IA2313AA	CFSAN059175	Braenderup	0	0	0	0	0	0	0	0	0	0	0
SAL_IA5899AA	MS160414	Braenderup	0	0	0	0	0	0	0	0	0	0	0
SAL_IA6543AA	PNUSAS008731	Braenderup	0	0	0	0	0	0	0	0	0	0	0
SAL_IA8927AA	PNUSAS009368	Braenderup	0	0	0	0	0	0	0	0	0	0	0
SAL_IA9229AA	PNUSAS009561	Braenderup	0	0	0	0	0	0	0	0	0	0	0
SAL_IA9581AA	PNUSAS009839	Braenderup	0	0	0	0	0	0	0	0	0	0	0
SAL_JA0506AA	PNUSAS010553	Braenderup	0	0	0	0	0	0	0	0	0	0	0
SAL_JA2001AA	PNUSAS011539	Braenderup	0	0	0	0	0	0	0	0	0	0	0
SAL_JA3232AA	PNUSAS011079	Braenderup	0	0	0	0	0	0	0	0	0	0	0
SAL_JA3008AA	PNUSAS011988	Braenderup	0	0	0	0	0	0	0	0	0	0	0
SAL_JA3984AA	PNUSAS012306	Braenderup	0	0	0	0	0	0	0	0	0	0	0
SAL_JA3914AA	PNUSAS011961	Braenderup	0	0	0	0	0	0	0	0	0	0	0
SAL_JA3799AA	PNUSAS012406	Braenderup	0	0	0	0	0	0	0	0	0	0	0
SAL_JA5859AA	358409	Braenderup	0	0	0	0	0	0	0	0	0	0	0
SAL_JA5981AA	PNUSAS011295	Braenderup	0	0	0	0	0	0	0	0	0	0	0
SAL_JA9386AA	PNUSAS014925	Braenderup	0	0	0	0	0	0	0	0	0	0	0
SAL_KA0684AA	2015AM-0982	Braenderup	0	0	0	0	0	0	0	0	0	0	0
SAL_KA1859AA	PNUSAS014367	Braenderup	0	0	0	0	0	0	0	0	0	0	0
SAL_KA2656AA	PNUSAS016983	Braenderup	0	0	0	0	0	0	0	0	0	0	0
SAL_KA3478AA	PNUSAS017288	Braenderup	0	0	0	0	0	0	0	0	0	0	0
SAL_KA3640AA	PNUSAS015575	Braenderup	0	0	0	0	0	0	0	0	0	0	0
SAL_KA4631AA	PNUSAS018278	Braenderup	0	0	0	0	0	0	0	0	0	0	0
SAL_KA5041AA	CFSAN065052	Braenderup	0	0	0	0	0	0	0	0	0	0	0
SAL_KA6814AA	PNUSAS018946	Braenderup	0	0	0	0	0	0	0	0	0	0	0
SAL_KA7958AA	PNUSAS020260	Braenderup	0	0	0	0	0	0	0	0	0	0	0

SAL_KA7310AA	PNUSAS019787	Braenderup	0	0	0	0	0	0	0	0	0	0	0
SAL_KA6916AA	PNUSAS017573	Braenderup	0	0	0	0	0	0	0	0	0	0	0
SAL_KA6734AA	PNUSAS018980	Braenderup	0	0	0	0	0	0	0	0	0	0	0
SAL_KA8416AA	2015AM-2167	Braenderup	0	0	0	0	0	0	0	0	0	0	0
SAL_KA7888AA	PNUSAS019862	Braenderup	0	0	0	0	0	0	0	0	0	0	0
SAL_KA8938AA	2015AM-1756	Braenderup	0	0	0	0	0	0	0	0	0	0	0
SAL_LA0499AA	PNUSAS019584	Braenderup	0	0	0	0	0	0	0	0	0	0	0
SAL_LA4320AA	PNUSAS022595	Braenderup	0	0	0	0	0	0	0	0	0	0	0
SAL_LA4819AA	PNUSAS023058	Braenderup	0	0	0	0	0	0	0	0	0	0	0
SAL_LA7411AA	PNUSAS023253	Braenderup	0	0	0	0	0	0	0	0	0	0	0
SAL_LA9899AA	2015AM-0841	Braenderup	0	0	0	0	0	0	0	0	0	0	0
SAL_LA9076AA	PNUSAS024426	Braenderup	0	0	0	0	0	0	0	0	0	0	0
SAL_MA0497AA	PNUSAS024296	Braenderup	0	0	0	0	0	0	0	0	0	0	0
SAL_MA6032AA	SA20026289	Braenderup	0	0	0	0	0	0	0	0	0	0	0
SAL_MA6387AA	PNUSAS026928	Braenderup	0	0	0	0	0	0	0	0	0	0	0
SAL_MA6390AA	PNUSAS026929	Braenderup	0	0	0	0	0	0	0	0	0	0	0
SAL_MA6394AA	PNUSAS026926	Braenderup	0	0	0	0	0	0	0	0	0	0	0
SAL_MA6395AA	PNUSAS026927	Braenderup	0	0	0	0	0	0	0	0	0	0	0
SAL_MA6375AA	PNUSAS016763	Braenderup	0	0	0	0	0	0	0	0	0	0	0
SAL_MA6299AA	PNUSAS027858	Braenderup	0	0	0	0	0	0	0	0	0	0	0
SAL_MA6119AA	PNUSAS027898	Braenderup	0	0	0	0	0	0	0	0	0	0	0
SAL_MA6662AA	PNUSAS026592	Braenderup	0	0	0	0	0	0	0	0	0	0	0
SAL_MA6672AA	PNUSAS026591	Braenderup	0	0	0	0	0	0	0	0	0	0	0
SAL_MA6667AA	PNUSAS026590	Braenderup	0	0	0	0	0	0	0	0	0	0	0
SAL_MA6591AA	PNUSAS025840	Braenderup	0	0	0	0	0	0	0	0	0	0	0
SAL_MA6565AA	PNUSAS025851	Braenderup	0	0	0	0	0	0	0	0	0	0	0
SAL_MA6584AA	PNUSAS025847	Braenderup	0	0	0	0	0	0	0	0	0	0	0
SAL_MA7157AA	PNUSAS027172	Braenderup	0	0	0	0	0	0	0	0	0	0	0
SAL_MA7069AA	PNUSAS026015	Braenderup	0	0	0	0	0	0	0	0	0	0	0
SAL_MA7051AA	PNUSAS027774	Braenderup	0	0	0	0	0	0	0	0	0	0	0
SAL_MA6980AA	CFSAN071435	Braenderup	0	0	0	0	0	0	0	0	0	0	0
SAL_MA6856AA	CFSAN071461	Braenderup	0	0	0	0	0	0	0	0	0	0	0
SAL_MA6832AA	ADRDL-1277	Braenderup	0	0	0	0	0	0	0	0	0	0	0
SAL_MA6813AA	SAL-17-VL-SD-OK-0006	Braenderup	0	0	0	0	0	0	0	0	0	0	0
SAL_MA7258AA	PNUSAS028061	Braenderup	0	0	0	0	0	0	0	0	0	0	0
SAL_MA7629AA	PNUSAS027028	Braenderup	0	0	0	0	0	0	0	0	0	0	0
SAL_MA7635AA	PNUSAS028562	Braenderup	0	0	0	0	0	0	0	0	0	0	0
SAL_MA7656AA	CFSAN071194	Braenderup	0	0	0	0	0	0	0	0	0	0	0
SAL_MA7572AA	PNUSAS028267	Braenderup	0	0	0	0	0	0	0	0	0	0	0
SAL_MA7593AA	PNUSAS028268	Braenderup	0	0	0	0	0	0	0	0	0	0	0
SAL_MA7537AA	2017K-1015	Braenderup	0	0	0	0	0	0	0	0	0	0	0
SAL_MA7445AA	FSIS11705897	Braenderup	0	0	0	0	0	0	0	0	0	0	0
SAL_MA7926AA	PNUSAS028310	Braenderup	0	0	0	0	0	0	0	0	0	0	0
SAL_MA7848AA	PNUSAS028574	Braenderup	0	0	0	0	0	0	0	0	0	0	0
SAL_MA7966AA	30-27-8101169	Braenderup	0	0	0	0	0	0	0	0	0	0	0
SAL_MA7941AA	PNUSAS028308	Braenderup	0	0	0	0	0	0	0	0	0	0	0
SAL_MA7773AA	PNUSAS021629	Braenderup	0	0	0	0	0	0	0	0	0	0	0
SAL_MA7949AA	CFSAN071826	Braenderup	0	0	0	0	0	0	0	0	0	0	0
SAL_MA7993AA	FSIS21720646	Braenderup	0	0	0	0	0	0	0	0	0	0	0
SAL_MA8066AA	FDA00012370	Braenderup	0	0	0	0	0	0	0	0	0	0	0
SAL_MA8043AA	PNUSAS028817	Braenderup	0	0	0	0	0	0	0	0	0	0	0
SAL_MA8758AA	ERS2012172	Braenderup	0	0	0	0	0	0	0	0	0	0	0
SAL_MA8433AA	PNUSAS026599	Braenderup	0	0	0	0	0	0	0	0	0	0	0
SAL_MA8425AA	PNUSAS026596	Braenderup	0	0	0	0	0	0	0	0	0	0	0
SAL_MA8341AA	PNUSAS027990	Braenderup	0	0	0	0	0	0	0	0	0	0	0
SAL_MA8315AA	PNUSAS027815	Braenderup	0	0	0	0	0	0	0	0	0	0	0
SAL_MA8223AA	PNUSAS029011	Braenderup	0	0	0	0	0	0	0	0	0	0	0
SAL_MA8965AA	PNUSAS029099	Braenderup	0	0	0	0	0	0	0	0	0	0	0
SAL_MA8173AA	PNUSAS028907	Braenderup	0	0	0	0	0	0	0	0	0	0	0
SAL_MA8994AA	PNUSAS026991	Braenderup	0	0	0	0	0	0	0	0	0	0	0
SAL_MA8920AA	PNUSAS029000	Braenderup	0	0	0	0	0	0	0	0	0	0	0
SAL_MA8936AA	PNUSAS028984	Braenderup	0	0	0	0	0	0	0	0	0	0	0
SAL_MA8903AA	FSIS11706031	Braenderup	0	0	0	0	0	0	0	0	0	0	0
SAL_NA0370AA	PNUSAS024289	Braenderup	0	0	0	0	0	0	0	0	0	0	0
SAL_NA0310AA	PNUSAS026354	Braenderup	0	0	0	0	0	0	0	0	0	0	0
SAL_NA0309AA	PNUSAS027756	Braenderup	0	0	0	0	0	0	0	0	0	0	0
SAL_NA0237AA	PNUSAS029201	Braenderup	0	0	0	0	0	0	0	0	0	0	0
SAL_NA0210AA	PNUSAS029215	Braenderup	0	0	0	0	0	0	0	0	0	0	0
SAL_NA0213AA	PNUSAS029209	Braenderup	0	0	0	0	0	0	0	0	0	0	0
SAL_NA0216AA	PNUSAS029214	Braenderup	0	0	0	0	0	0	0	0	0	0	0
SAL_NA0097AA	nterica_subs_enterica_6,	Braenderup	0	0	0	0	0	0	0	0	0	0	0
SAL_MA9925AA	CVM N16S381	Braenderup	0	0	0	0	0	0	0	0	0	0	0
SAL_MA9873AA	CVM N16S404	Braenderup	0	0	0	0	0	0	0	0	0	0	0

SAL_MA9823AA	CVM N16S017	Braenderup	0	0	0	0	0	0	0	0	0	0	0
SAL_MA9624AA	CVM N16S058	Braenderup	0	0	0	0	0	0	0	0	0	0	0
SAL_MA9731AA	CVM N16S299	Braenderup	0	0	0	0	0	0	0	0	0	0	0
SAL_NA0733AA	PNUSAS029547	Braenderup	0	0	0	0	0	0	0	0	0	0	0
SAL_NA0818AA	PNUSAS028504	Braenderup	0	0	0	0	0	0	0	0	0	0	0
SAL_NA0858AA	PNUSAS029677	Braenderup	0	0	0	0	0	0	0	0	0	0	0
SAL_NA0588AA	PNUSAS029514	Braenderup	0	0	0	0	0	0	0	0	0	0	0
SAL_NA0602AA	PNUSAS029326	Braenderup	0	0	0	0	0	0	0	0	0	0	0
SAL_NA0627AA	PNUSAS028901	Braenderup	0	0	0	0	0	0	0	0	0	0	0
SAL_NA0637AA	PNUSAS028895	Braenderup	0	0	0	0	0	0	0	0	0	0	0
SAL_NA0920AA	VA-WGS-17110	Braenderup	0	0	0	0	0	0	0	0	0	0	0
SAL_OA0190AA	HJ30-VA-WGS-17108	Braenderup	0	0	0	0	0	0	0	0	0	0	0
SAL_NA0921AA	VA-WGS-17108	Braenderup	0	0	0	0	0	0	0	0	0	0	0
SAL_NA0963AA	OH-17-14144	Braenderup	0	0	0	0	0	0	0	0	0	0	0
SAL_OA0191AA	HJ31-VA-WGS-17109	Braenderup	0	0	0	0	0	0	0	0	0	0	0
SAL_NA0924AA	VA-WGS-17109	Braenderup	0	0	0	0	0	0	0	0	0	0	0
SAL_NA0965AA	OH-17-14778	Braenderup	0	0	0	0	0	0	0	0	0	0	0
SAL_NA1216AA	PNUSAS029362	Braenderup	0	0	0	0	0	0	0	0	0	0	0
SAL_NA1163AA	PNUSAS026791	Braenderup	0	0	0	0	0	0	0	0	0	0	0
SAL_NA1175AA	PNUSAS026250	Braenderup	0	0	0	0	0	0	0	0	0	0	0
SAL_NA1186AA	NPHL 170820	Braenderup	0	0	0	0	0	0	0	0	0	0	0
SAL_NA1031AA	PNUSAS029132	Braenderup	0	0	0	0	0	0	0	0	0	0	0
SAL_NA1274AA	PNUSAS026030	Braenderup	0	0	0	0	0	0	0	0	0	0	0
SAL_NA1275AA	PNUSAS026032	Braenderup	0	0	0	0	0	0	0	0	0	0	0
SAL_NA1794AA	PNUSAS026877	Braenderup	0	0	0	0	0	0	0	0	0	0	0
SAL_NA1677AA	PNUSAS029447	Braenderup	0	0	0	0	0	0	0	0	0	0	0
SAL_NA1433AA	CFSAN071444	Braenderup	0	0	0	0	0	0	0	0	0	0	0
SAL_NA1889AA	PNUSAS029392	Braenderup	0	0	0	0	0	0	0	0	0	0	0
SAL_NA2033AA	33-5-8101297	Braenderup	0	0	0	0	0	0	0	0	0	0	0
SAL_NA2419AA	PNUSAS030284	Braenderup	0	0	0	0	0	0	0	0	0	0	0
SAL_NA2974AA	PNUSAS029780	Braenderup	0	0	0	0	0	0	0	0	0	0	0
SAL_NA2827AA	PNUSAS030123	Braenderup	0	0	0	0	0	0	0	0	0	0	0
SAL_NA2715AA	CFSAN031860	Braenderup	0	0	0	0	0	0	0	0	0	0	0
SAL_NA2664AA	PNUSAS030369	Braenderup	0	0	0	0	0	0	0	0	0	0	0
SAL_NA3137AA	PNUSAS023348	Braenderup	0	0	0	0	0	0	0	0	0	0	0
SAL_NA3076AA	PNUSAS030338	Braenderup	0	0	0	0	0	0	0	0	0	0	0
SAL_NA3025AA	PNUSAS029847	Braenderup	0	0	0	0	0	0	0	0	0	0	0
SAL_NA3282AA	FSIS11706344	Braenderup	0	0	0	0	0	0	0	0	0	0	0
SAL_NA3191AA	PNUSAS030352	Braenderup	0	0	0	0	0	0	0	0	0	0	0
SAL_NA3405AA	FSIS11705672	Braenderup	0	0	0	0	0	0	0	0	0	0	0
SAL_NA3797AA	PNUSAS029600	Braenderup	0	0	0	0	0	0	0	0	0	0	0
SAL_NA3802AA	PNUSAS024781	Braenderup	0	0	0	0	0	0	0	0	0	0	0
SAL_NA3731AA	PNUSAS030894	Braenderup	0	0	0	0	0	0	0	0	0	0	0
SAL_NA3794AA	PNUSAS030635	Braenderup	0	0	0	0	0	0	0	0	0	0	0
SAL_NA3539AA	PNUSAS031000	Braenderup	0	0	0	0	0	0	0	0	0	0	0
SAL_NA3565AA	PNUSAS030490	Braenderup	0	0	0	0	0	0	0	0	0	0	0
SAL_NA3497AA	PNUSAS030867	Braenderup	0	0	0	0	0	0	0	0	0	0	0
SAL_NA3831AA	2015AM-3364	Braenderup	0	0	0	0	0	0	0	0	0	0	0
SAL_NA3885AA	2015AM-2392	Braenderup	0	0	0	0	0	0	0	0	0	0	0
SAL_NA4251AA	PNUSAS029603	Braenderup	0	0	0	0	0	0	0	0	0	0	0
SAL_NA4253AA	PNUSAS031185	Braenderup	0	0	0	0	0	0	0	0	0	0	0
SAL_NA4248AA	PNUSAS027916	Braenderup	0	0	0	0	0	0	0	0	0	0	0
SAL_NA4114AA	PNUSAS031113	Braenderup	0	0	0	0	0	0	0	0	0	0	0
SAL_NA4104AA	PNUSAS031100	Braenderup	0	0	0	0	0	0	0	0	0	0	0
SAL_NA4108AA	PNUSAS031102	Braenderup	0	0	0	0	0	0	0	0	0	0	0
SAL_NA4113AA	PNUSAS031114	Braenderup	0	0	0	0	0	0	0	0	0	0	0
SAL_NA4058AA	PNUSAS030346	Braenderup	0	0	0	0	0	0	0	0	0	0	0
SAL_NA4312AA	PNUSAS031268	Braenderup	0	0	0	0	0	0	0	0	0	0	0
SAL_NA4317AA	PNUSAS031346	Braenderup	0	0	0	0	0	0	0	0	0	0	0
SAL_NA4492AA	PNUSAS031227	Braenderup	0	0	0	0	0	0	0	0	0	0	0
SAL_NA4526AA	FSIS11706947	Braenderup	0	0	0	0	0	0	0	0	0	0	0
SAL_NA4511AA	PNUSAS031522	Braenderup	0	0	0	0	0	0	0	0	0	0	0
SAL_NA4539AA	FSIS11706506	Braenderup	0	0	0	0	0	0	0	0	0	0	0
SAL_NA4597AA	FSIS21720872	Braenderup	0	0	0	0	0	0	0	0	0	0	0
SAL_NA4600AA	FSIS31700007	Braenderup	0	0	0	0	0	0	0	0	0	0	0
SAL_NA4567AA	2014AM-2699	Braenderup	0	0	0	0	0	0	0	0	0	0	0
SAL_NA4735AA	PNUSAS028388	Braenderup	0	0	0	0	0	0	0	0	0	0	0
SAL_NA4744AA	PNUSAS028392	Braenderup	0	0	0	0	0	0	0	0	0	0	0
SAL_NA4747AA	PNUSAS028372	Braenderup	0	0	0	0	0	0	0	0	0	0	0
SAL_NA4760AA	PNUSAS031235	Braenderup	0	0	0	0	0	0	0	0	0	0	0
SAL_NA4751AA	PNUSAS028379	Braenderup	0	0	0	0	0	0	0	0	0	0	0
SAL_NA4676AA	PNUSAS031131	Braenderup	0	0	0	0	0	0	0	0	0	0	0
SAL_NA4639AA	FSIS11706996	Braenderup	0	0	0	0	0	0	0	0	0	0	0

SAL_NA4903AA	PNUSAS031347	Braenderup	0	0	0	0	0	0	0	0	0	0	0
SAL_NA4848AA	PNUSAS031601	Braenderup	0	0	0	0	0	0	0	0	0	0	0
SAL_NA4809AA	PNUSAS031282	Braenderup	0	0	0	0	0	0	0	0	0	0	0
SAL_NA4973AA	PNUSAS029616	Braenderup	0	0	0	0	0	0	0	0	0	0	0
SAL_NA5426AA	2015AM-0923	Braenderup	0	0	0	0	0	0	0	0	0	0	0
SAL_NA5407AA	PNUSAS030162	Braenderup	0	0	0	0	0	0	0	0	0	0	0
SAL_NA5378AA	PNUSAS030944	Braenderup	0	0	0	0	0	0	0	0	0	0	0
SAL_NA5320AA	2015AM-0117	Braenderup	0	0	0	0	0	0	0	0	0	0	0
SAL_NA5285AA	2015AM-1990	Braenderup	0	0	0	0	0	0	0	0	0	0	0
SAL_NA5268AA	2015AM-2323	Braenderup	0	0	0	0	0	0	0	0	0	0	0
SAL_NA5265AA	2015AM-2006	Braenderup	0	0	0	0	0	0	0	0	0	0	0
SAL_NA5621AA	2015AM-2270	Braenderup	0	0	0	0	0	0	0	0	0	0	0
SAL_NA5574AA	PNUSAS032152	Braenderup	0	0	0	0	0	0	0	0	0	0	0
SAL_NA5481AA	PNUSAS031929	Braenderup	0	0	0	0	0	0	0	0	0	0	0
SAL_NA5442AA	PNUSAS031939	Braenderup	0	0	0	0	0	0	0	0	0	0	0
SAL_NA6153AA	2015AM-2533	Braenderup	0	0	0	0	0	0	0	0	0	0	0
SAL_NA6149AA	2015AM-2667	Braenderup	0	0	0	0	0	0	0	0	0	0	0
SAL_NA6150AA	2015AM-2532	Braenderup	0	0	0	0	0	0	0	0	0	0	0
SAL_NA6085AA	2015AM-2468	Braenderup	0	0	0	0	0	0	0	0	0	0	0
SAL_NA6159AA	2015AM-2531	Braenderup	0	0	0	0	0	0	0	0	0	0	0
SAL_NA6020AA	2015AM-2607	Braenderup	0	0	0	0	0	0	0	0	0	0	0
SAL_NA6026AA	2015AM-2432	Braenderup	0	0	0	0	0	0	0	0	0	0	0
SAL_NA6040AA	2015AM-2433	Braenderup	0	0	0	0	0	0	0	0	0	0	0
SAL_NA5952AA	PNUSAS029412	Braenderup	0	0	0	0	0	0	0	0	0	0	0
SAL_NA5869AA	PNUSAS032066	Braenderup	0	0	0	0	0	0	0	0	0	0	0
SAL_NA5873AA	PNUSAS032063	Braenderup	0	0	0	0	0	0	0	0	0	0	0
SAL_NA5884AA	PNUSAS032071	Braenderup	0	0	0	0	0	0	0	0	0	0	0
SAL_NA5905AA	PNUSAS032043	Braenderup	0	0	0	0	0	0	0	0	0	0	0
SAL_NA5909AA	PNUSAS032045	Braenderup	0	0	0	0	0	0	0	0	0	0	0
SAL_NA5882AA	PNUSAS032069	Braenderup	0	0	0	0	0	0	0	0	0	0	0
SAL_NA5860AA	PNUSAS028660	Braenderup	0	0	0	0	0	0	0	0	0	0	0
SAL_NA5696AA	PNUSAS031923	Braenderup	0	0	0	0	0	0	0	0	0	0	0
SAL_NA5722AA	PNUSAS031616	Braenderup	0	0	0	0	0	0	0	0	0	0	0
SAL_NA6603AA	PNUSAS031887	Braenderup	0	0	0	0	0	0	0	0	0	0	0
SAL_NA6500AA	2015AM-2970	Braenderup	0	0	0	0	0	0	0	0	0	0	0
SAL_NA6357AA	FSIS21820915	Braenderup	0	0	0	0	0	0	0	0	0	0	0
SAL_NA7031AA	PNUSAS028959	Braenderup	0	0	0	0	0	0	0	0	0	0	0
SAL_NA6933AA	PNUSAS032021	Braenderup	0	0	0	0	0	0	0	0	0	0	0
SAL_NA6961AA	PNUSAS032435	Braenderup	0	0	0	0	0	0	0	0	0	0	0
SAL_NA6908AA	PNUSAS031762	Braenderup	0	0	0	0	0	0	0	0	0	0	0
SAL_NA6829AA	PNUSAS028424	Braenderup	0	0	0	0	0	0	0	0	0	0	0
SAL_NA6818AA	PNUSAS028429	Braenderup	0	0	0	0	0	0	0	0	0	0	0
SAL_NA4249AA	PNUSAS029604	Braenderup	0	0	0	0	0	0	0	0	0	0	0
SAL_NA7210AA	FSIS11807129	Braenderup	0	0	0	0	0	0	0	0	0	0	0
SAL_NA7458AA	2015AM-3483	Braenderup	0	0	0	0	0	0	0	0	0	0	0
SAL_NA7482AA	2015AM-3484	Braenderup	0	0	0	0	0	0	0	0	0	0	0
SAL_NA7403AA	2015AM-3345	Braenderup	0	0	0	0	0	0	0	0	0	0	0
SAL_NA7405AA	2015AM-3346	Braenderup	0	0	0	0	0	0	0	0	0	0	0
SAL_NA7441AA	2015AM-3482	Braenderup	0	0	0	0	0	0	0	0	0	0	0
SAL_NA7289AA	PNUSAS032801	Braenderup	0	0	0	0	0	0	0	0	0	0	0
SAL_NA7295AA	PNUSAS028437	Braenderup	0	0	0	0	0	0	0	0	0	0	0
SAL_NA8219AA	PNUSAS032640	Braenderup	0	0	0	0	0	0	0	0	0	0	0
SAL_NA8133AA	2015AM-3655	Braenderup	0	0	0	0	0	0	0	0	0	0	0
SAL_NA8088AA	2015AM-3608	Braenderup	0	0	0	0	0	0	0	0	0	0	0
SAL_NA8534AA	PNUSAS031584	Braenderup	0	0	0	0	0	0	0	0	0	0	0
SAL_NA8538AA	PNUSAS031572	Braenderup	0	0	0	0	0	0	0	0	0	0	0
SAL_NA8413AA	PNUSAS019002	Braenderup	0	0	0	0	0	0	0	0	0	0	0
SAL_NA8364AA	PNUSAS033137	Braenderup	0	0	0	0	0	0	0	0	0	0	0
SAL_NA8370AA	PNUSAS033039	Braenderup	0	0	0	0	0	0	0	0	0	0	0
SAL_NA9016AA	18FMFA000068-1	Braenderup	0	0	0	0	0	0	0	0	0	0	0
SAL_NA9018AA	18FMFA000068-10	Braenderup	0	0	0	0	0	0	0	0	0	0	0
SAL_NA8728AA	FSIS21821041	Braenderup	0	0	0	0	0	0	0	0	0	0	0
SAL_NA8719AA	PNUSAS031975	Braenderup	0	0	0	0	0	0	0	0	0	0	0
SAL_NA8698AA	PNUSAS032889	Braenderup	0	0	0	0	0	0	0	0	0	0	0
SAL_NA9453AA	PNUSAS033341	Braenderup	0	0	0	0	0	0	0	0	0	0	0
SAL_NA9390AA	PNUSAS033470	Braenderup	0	0	0	0	0	0	0	0	0	0	0
SAL_OA0192AA	HJ32-VA-WGS-17109	Braenderup	0	0	0	0	0	0	0	0	0	0	0
SAL_OA0031AA	PNUSAS031171	Braenderup	0	0	0	0	0	0	0	0	0	0	0
SAL_OA0021AA	PNUSAS031427	Braenderup	0	0	0	0	0	0	0	0	0	0	0
SAL_NA9981AA	PNUSAS030540	Braenderup	0	0	0	0	0	0	0	0	0	0	0
SAL_NA9986AA	PNUSAS026920	Braenderup	0	0	0	0	0	0	0	0	0	0	0
SAL_NA9910AA	PNUSAS032779	Braenderup	0	0	0	0	0	0	0	0	0	0	0
SAL_NA9839AA	PNUSAS033994	Braenderup	0	0	0	0	0	0	0	0	0	0	0

SAL_OA0284AA	PNUSAS034047	Braenderup	0	0	0	0	0	0	0	0	0	0	0
SAL_OA0268AA	PNUSAS034051	Braenderup	0	0	0	0	0	0	0	0	0	0	0
SAL_OA0245AA	PNUSAS034096	Braenderup	0	0	0	0	0	0	0	0	0	0	0
SAL_OA0595AA	PNUSAS033855	Braenderup	0	0	0	0	0	0	0	0	0	0	0
SAL_OA0579AA	FSIS21821078	Braenderup	0	0	0	0	0	0	0	0	0	0	0
SAL_OA1193AA	PNUSAS034488	Braenderup	0	0	0	0	0	0	0	0	0	0	0
SAL_OA1207AA	PNUSAS034251	Braenderup	0	0	0	0	0	0	0	0	0	0	0
SAL_OA1185AA	PNUSAS033728	Braenderup	0	0	0	0	0	0	0	0	0	0	0
SAL_OA1178AA	PNUSAS029429	Braenderup	0	0	0	0	0	0	0	0	0	0	0
SAL_OA1137AA	PNUSAS033595	Braenderup	0	0	0	0	0	0	0	0	0	0	0
SAL_OA1107AA	PNUSAS033605	Braenderup	0	0	0	0	0	0	0	0	0	0	0
SAL_OA1033AA	PNUSAS034297	Braenderup	0	0	0	0	0	0	0	0	0	0	0
SAL_PA0787AA	HJ34-VA-WGS-18001	Braenderup	0	0	0	0	0	0	0	0	0	0	0
SAL_OA4224AA	11-12-18-8100184-N	Braenderup	0	0	0	0	0	0	0	0	0	0	0
SAL_OA4223AA	11-10-18-8100182-L	Braenderup	0	0	0	0	0	0	0	0	0	0	0
SAL_OA5967AA	100740	Braenderup	0	0	0	0	0	0	0	0	0	0	0
SAL_OA5868AA	PNUSAS034918	Braenderup	0	0	0	0	0	0	0	0	0	0	0
SAL_OA5857AA	PNUSAS035084	Braenderup	0	0	0	0	0	0	0	0	0	0	0
SAL_OA5817AA	PNUSAS032274	Braenderup	0	0	0	0	0	0	0	0	0	0	0
SAL_OA5787AA	PNUSAS035266	Braenderup	0	0	0	0	0	0	0	0	0	0	0
SAL_OA5789AA	PNUSAS035265	Braenderup	0	0	0	0	0	0	0	0	0	0	0
SAL_OA5790AA	PNUSAS035264	Braenderup	0	0	0	0	0	0	0	0	0	0	0
SAL_OA5800AA	PNUSAS033181	Braenderup	0	0	0	0	0	0	0	0	0	0	0
SAL_OA7303AA	FSIS31800270	Braenderup	0	0	0	0	0	0	0	0	0	0	0
SAL_OA7392AA	PNUSAS035949	Braenderup	0	0	0	0	0	0	0	0	0	0	0
SAL_OA7360AA	PNUSAS037252	Braenderup	0	0	0	0	0	0	0	0	0	0	0
SAL_OA7334AA	PNUSAS037267	Braenderup	0	0	0	0	0	0	0	0	0	0	0
SAL_OA7408AA	PNUSAS037519	Braenderup	0	0	0	0	0	0	0	0	0	0	0
SAL_PA0789AA	HJ35-VA-WGS-18001	Braenderup	0	0	0	0	0	0	0	0	0	0	0
SAL_OA9165AA	HJ35-WGS-VA18005	Braenderup	0	0	0	0	0	0	0	0	0	0	0
SAL_OA9953AA	PNUSAS037724	Braenderup	0	0	0	0	0	0	0	0	0	0	0
SAL_OA9900AA	PNUSAS037103	Braenderup	0	0	0	0	0	0	0	0	0	0	0
SAL_OA5106AA	PNUSAS035662	Braenderup	0	0	0	0	0	0	0	0	0	0	0
SAL_OA7896AA	PNUSAS037226	Braenderup	0	0	0	0	0	0	0	0	0	0	0
SAL_OA7898AA	PNUSAS037227	Braenderup	0	0	0	0	0	0	0	0	0	0	0
SAL_OA7859AA	PNUSAS035722	Braenderup	0	0	0	0	0	0	0	0	0	0	0
SAL_OA7814AA	PNUSAS036517	Braenderup	0	0	0	0	0	0	0	0	0	0	0
SAL_OA7826AA	PNUSAS033107	Braenderup	0	0	0	0	0	0	0	0	0	0	0
SAL_OA7830AA	PNUSAS025491	Braenderup	0	0	0	0	0	0	0	0	0	0	0
SAL_OA7792AA	PNUSAS037363	Braenderup	0	0	0	0	0	0	0	0	0	0	0
SAL_OA7803AA	PNUSAS036513	Braenderup	0	0	0	0	0	0	0	0	0	0	0
SAL_OA7752AA	PNUSAS037553	Braenderup	0	0	0	0	0	0	0	0	0	0	0
SAL_OA7748AA	PNUSAS037075	Braenderup	0	0	0	0	0	0	0	0	0	0	0
SAL_OA5039AA	PNUSAS036396	Braenderup	0	0	0	0	0	0	0	0	0	0	0
SAL_OA5018AA	PNUSAS035758	Braenderup	0	0	0	0	0	0	0	0	0	0	0
SAL_OA4986AA	PNUSAS036801	Braenderup	0	0	0	0	0	0	0	0	0	0	0
SAL_OA5004AA	PNUSAS035759	Braenderup	0	0	0	0	0	0	0	0	0	0	0
SAL_OA5010AA	PNUSAS035760	Braenderup	0	0	0	0	0	0	0	0	0	0	0
SAL_OA4967AA	PNUSAS033671	Braenderup	0	0	0	0	0	0	0	0	0	0	0
SAL_OA4729AA	PNUSAS036515	Braenderup	0	0	0	0	0	0	0	0	0	0	0
SAL_OA4743AA	PNUSAS036514	Braenderup	0	0	0	0	0	0	0	0	0	0	0
SAL_OA4745AA	PNUSAS036516	Braenderup	0	0	0	0	0	0	0	0	0	0	0
SAL_OA4716AA	PNUSAS036510	Braenderup	0	0	0	0	0	0	0	0	0	0	0
SAL_OA4656AA	PNUSAS036245	Braenderup	0	0	0	0	0	0	0	0	0	0	0
SAL_OA4657AA	PNUSAS036244	Braenderup	0	0	0	0	0	0	0	0	0	0	0
SAL_OA4688AA	PNUSAS025297	Braenderup	0	0	0	0	0	0	0	0	0	0	0
SAL_OA4640AA	PNUSAS036511	Braenderup	0	0	0	0	0	0	0	0	0	0	0
SAL_OA4608AA	PNUSAS036613	Braenderup	0	0	0	0	0	0	0	0	0	0	0
SAL_OA4404AA	PNUSAS036030	Braenderup	0	0	0	0	0	0	0	0	0	0	0
SAL_OA4437AA	PNUSAS035871	Braenderup	0	0	0	0	0	0	0	0	0	0	0
SAL_OA4449AA	PNUSAS035798	Braenderup	0	0	0	0	0	0	0	0	0	0	0
SAL_OA8073AA	PNUSAS037766	Braenderup	0	0	0	0	0	0	0	0	0	0	0
SAL_OA8139AA	PNUSAS035058	Braenderup	0	0	0	0	0	0	0	0	0	0	0
SAL_OA8293AA	PNUSAS036862	Braenderup	0	0	0	0	0	0	0	0	0	0	0
SAL_OA8254AA	CFSAN058529	Braenderup	0	0	0	0	0	0	0	0	0	0	0
SAL_OA4960AA	PNUSAS033685	Braenderup	0	0	0	0	0	0	0	0	0	0	0
SAL_PA0697AA	CFSAN071968	Braenderup	0	0	0	0	0	0	0	0	0	0	0
SAL_PA0808AA	FDA00012905	Braenderup	0	0	0	0	0	0	0	0	0	0	0
SAL_PA0799AA	PNUSAS036509	Braenderup	0	0	0	0	0	0	0	0	0	0	0
SAL_PA0929AA	PNUSAS036308	Braenderup	0	0	0	0	0	0	0	0	0	0	0
SAL_PA0919AA	PNUSAS035842	Braenderup	0	0	0	0	0	0	0	0	0	0	0
SAL_PA1063AA	FSIS11808859	Braenderup	0	0	0	0	0	0	0	0	0	0	0
SAL_PA1022AA	PNUSAS038140	Braenderup	0	0	0	0	0	0	0	0	0	0	0

SAL_PA0989AA	PNUSAS038190	Braenderup	0	0	0	0	0	0	0	0	0	0	0
SAL_PA1439AA	PNUSAS038558	Braenderup	0	0	0	0	0	0	0	0	0	0	0
SAL_PA1452AA	PNUSAS038425	Braenderup	0	0	0	0	0	0	0	0	0	0	0
SAL_PA1263AA	PNUSAS038443	Braenderup	0	0	0	0	0	0	0	0	0	0	0
SAL_PA1956AA	0-e94d-11e6-874d-3c4a9	Braenderup	0	0	0	0	0	0	0	0	0	0	0
SAL_PA2357AA	PNUSAS033613	Braenderup	0	0	0	0	0	0	0	0	0	0	0
SAL_PA2339AA	PNUSAS037730	Braenderup	0	0	0	0	0	0	0	0	0	0	0
SAL_PA2352AA	PNUSAS033510	Braenderup	0	0	0	0	0	0	0	0	0	0	0
SAL_PA2181AA	FDA00012961	Braenderup	0	0	0	0	0	0	0	0	0	0	0
SAL_PA1691AA	PNUSAS038686	Braenderup	0	0	0	0	0	0	0	0	0	0	0
SAL_PA0358AA	PNUSAS037542	Braenderup	0	0	0	0	0	0	0	0	0	0	0
SAL_PA2525AA	FSIS11809209	Braenderup	0	0	0	0	0	0	0	0	0	0	0
SAL_PA3068AA	0-e94c-11e6-874d-3c4a92	Braenderup	0	0	0	0	0	0	0	0	0	0	0
SAL_PA3069AA	0-e94c-11e6-874d-3c4a9	Braenderup	0	0	0	0	0	0	0	0	0	0	0
SAL_PA3799AA	CFSAN075577	Braenderup	0	0	0	0	0	0	0	0	0	0	0
SAL_PA3935AA	PNUSAS038577	Braenderup	0	0	0	0	0	0	0	0	0	0	0
SAL_PA3930AA	PNUSAS039777	Braenderup	0	0	0	0	0	0	0	0	0	0	0
SAL_PA3831AA	PNUSAS038996	Braenderup	0	0	0	0	0	0	0	0	0	0	0
SAL_PA3835AA	PNUSAS039385	Braenderup	0	0	0	0	0	0	0	0	0	0	0
SAL_PA4229AA	PNUSAS038472	Braenderup	0	0	0	0	0	0	0	0	0	0	0
SAL_PA4202AA	PNUSAS039263	Braenderup	0	0	0	0	0	0	0	0	0	0	0
SAL_PA4336AA	CFSAN078618	Braenderup	0	0	0	0	0	0	0	0	0	0	0
SAL_PA4337AA	CFSAN078619	Braenderup	0	0	0	0	0	0	0	0	0	0	0
SAL_PA4333AA	CFSAN078620	Braenderup	0	0	0	0	0	0	0	0	0	0	0
SAL_PA4335AA	CFSAN078617	Braenderup	0	0	0	0	0	0	0	0	0	0	0
SAL_PA4458AA	PNUSAS034422	Braenderup	0	0	0	0	0	0	0	0	0	0	0
SAL_PA4403AA	PNUSAS039852	Braenderup	0	0	0	0	0	0	0	0	0	0	0
SAL_PA4390AA	PNUSAS039599	Braenderup	0	0	0	0	0	0	0	0	0	0	0
SAL_PA4350AA	PNUSAS039810	Braenderup	0	0	0	0	0	0	0	0	0	0	0
SAL_PA4684AA	PNUSAS039875	Braenderup	0	0	0	0	0	0	0	0	0	0	0
SAL_PA4642AA	PNUSAS038958	Braenderup	0	0	0	0	0	0	0	0	0	0	0
SAL_PA4643AA	PNUSAS038966	Braenderup	0	0	0	0	0	0	0	0	0	0	0
SAL_PA4653AA	PNUSAS039720	Braenderup	0	0	0	0	0	0	0	0	0	0	0
SAL_PA4832AA	262656	Braenderup	0	0	0	0	0	0	0	0	0	0	0
SAL_PA4952AA	299930	Braenderup	0	0	0	0	0	0	0	0	0	0	0
SAL_PA4927AA	192866	Braenderup	0	0	0	0	0	0	0	0	0	0	0
SAL_PA4913AA	FSIS21821554	Braenderup	0	0	0	0	0	0	0	0	0	0	0
SAL_PA5329AA	PNUSAS039662	Braenderup	0	0	0	0	0	0	0	0	0	0	0
SAL_PA5332AA	PNUSAS039674	Braenderup	0	0	0	0	0	0	0	0	0	0	0
SAL_PA5307AA	PNUSAS038635	Braenderup	0	0	0	0	0	0	0	0	0	0	0
SAL_PA5466AA	PNUSAS038917	Braenderup	0	0	0	0	0	0	0	0	0	0	0
SAL_PA5442AA	PNUSAS038863	Braenderup	0	0	0	0	0	0	0	0	0	0	0
SAL_PA5935AA	PNUSAS038395	Braenderup	0	0	0	0	0	0	0	0	0	0	0
SAL_PA5910AA	PNUSAS039461	Braenderup	0	0	0	0	0	0	0	0	0	0	0
SAL_PA5980AA	PNUSAS040178	Braenderup	0	0	0	0	0	0	0	0	0	0	0
SAL_PA5863AA	PNUSAS040445	Braenderup	0	0	0	0	0	0	0	0	0	0	0
SAL_PA5913AA	PNUSAS040012	Braenderup	0	0	0	0	0	0	0	0	0	0	0
SAL_PA6239AA	260055	Braenderup	0	0	0	0	0	0	0	0	0	0	0
SAL_PA6210AA	454888	Braenderup	0	0	0	0	0	0	0	0	0	0	0
SAL_PA6214AA	214728	Braenderup	0	0	0	0	0	0	0	0	0	0	0
SAL_PA6162AA	471167	Braenderup	0	0	0	0	0	0	0	0	0	0	0
SAL_PA6468AA	PNUSAS040704	Braenderup	0	0	0	0	0	0	0	0	0	0	0
SAL_PA7113AA	PNUSAS040544	Braenderup	0	0	0	0	0	0	0	0	0	0	0
SAL_PA7013AA	PNUSAS040421	Braenderup	0	0	0	0	0	0	0	0	0	0	0
SAL_PA7000AA	PNUSAS040785	Braenderup	0	0	0	0	0	0	0	0	0	0	0
SAL_PA6979AA	PNUSAS040787	Braenderup	0	0	0	0	0	0	0	0	0	0	0
SAL_PA6908AA	PNUSAS040146	Braenderup	0	0	0	0	0	0	0	0	0	0	0
SAL_PA7224AA	FSIS11809875	Braenderup	0	0	0	0	0	0	0	0	0	0	0
SAL_PA7395AA	PNUSAS040804	Braenderup	0	0	0	0	0	0	0	0	0	0	0
SAL_PA7457AA	PNUSAS040780	Braenderup	0	0	0	0	0	0	0	0	0	0	0
SAL_PA7446AA	PNUSAS040781	Braenderup	0	0	0	0	0	0	0	0	0	0	0
SAL_PA7529AA	290806	Braenderup	0	0	0	0	0	0	0	0	0	0	0
SAL_PA7649AA	PNUSAS039649	Braenderup	0	0	0	0	0	0	0	0	0	0	0
SAL_PA7642AA	PNUSAS039642	Braenderup	0	0	0	0	0	0	0	0	0	0	0
SAL_PA7572AA	PNUSAS040134	Braenderup	0	0	0	0	0	0	0	0	0	0	0
SAL_PA7634AA	PNUSAS039644	Braenderup	0	0	0	0	0	0	0	0	0	0	0
SAL_PA7742AA	45259	Braenderup	0	0	0	0	0	0	0	0	0	0	0
SAL_PA7730AA	166284	Braenderup	0	0	0	0	0	0	0	0	0	0	0
SAL_PA7711AA	FDA00013003	Braenderup	0	0	0	0	0	0	0	0	0	0	0
SAL_PA7683AA	FDA00013002	Braenderup	0	0	0	0	0	0	0	0	0	0	0
SAL_PA8072AA	443566	Braenderup	0	0	0	0	0	0	0	0	0	0	0
SAL_PA8049AA	440012	Braenderup	0	0	0	0	0	0	0	0	0	0	0
SAL_PA7992AA	298784	Braenderup	0	0	0	0	0	0	0	0	0	0	0

SAL_PA8431AA	370637	Braenderup	0	0	0	0	0	0	0	0	0	0	0
SAL_PA8252AA	PNUSAS040864	Braenderup	0	0	0	0	0	0	0	0	0	0	0
SAL_PA8392AA	516952	Braenderup	0	0	0	0	0	0	0	0	0	0	0
SAL_PA7832AA	312854	Braenderup	0	0	0	0	0	0	0	0	0	0	0
SAL_PA8148AA	PNUSAS040757	Braenderup	0	0	0	0	0	0	0	0	0	0	0
SAL_PA8118AA	PNUSAS040161	Braenderup	0	0	0	0	0	0	0	0	0	0	0
SAL_PA8200AA	PNUSAS041068	Braenderup	0	0	0	0	0	0	0	0	0	0	0
SAL_PA8184AA	PNUSAS041188	Braenderup	0	0	0	0	0	0	0	0	0	0	0
SAL_PA8198AA	PNUSAS041071	Braenderup	0	0	0	0	0	0	0	0	0	0	0
SAL_PA8823AA	HJ34-VA-WGS-18004	Braenderup	0	0	0	0	0	0	0	0	0	0	0
SAL_PA8441AA	PNUSAS038249	Braenderup	0	0	0	0	0	0	0	0	0	0	0
SAL_PA8796AA	PNUSAS041448	Braenderup	0	0	0	0	0	0	0	0	0	0	0
SAL_PA8795AA	PNUSAS041457	Braenderup	0	0	0	0	0	0	0	0	0	0	0
SAL_PA8775AA	PNUSAS041263	Braenderup	0	0	0	0	0	0	0	0	0	0	0
SAL_PA8776AA	PNUSAS041262	Braenderup	0	0	0	0	0	0	0	0	0	0	0
SAL_PA8510AA	127430	Braenderup	0	0	0	0	0	0	0	0	0	0	0
SAL_PA8604AA	126196	Braenderup	0	0	0	0	0	0	0	0	0	0	0
SAL_PA8524AA	370630	Braenderup	0	0	0	0	0	0	0	0	0	0	0
SAL_PA9324AA	PNUSAS040833	Braenderup	0	0	0	0	0	0	0	0	0	0	0
SAL_PA9299AA	PNUSAS040946	Braenderup	0	0	0	0	0	0	0	0	0	0	0
SAL_PA9300AA	PNUSAS040945	Braenderup	0	0	0	0	0	0	0	0	0	0	0
SAL_PA9255AA	PNUSAS041136	Braenderup	0	0	0	0	0	0	0	0	0	0	0
SAL_PA9246AA	PNUSAS041149	Braenderup	0	0	0	0	0	0	0	0	0	0	0
SAL_PA9215AA	PNUSAS041578	Braenderup	0	0	0	0	0	0	0	0	0	0	0
SAL_PA9156AA	PNUSAS041366	Braenderup	0	0	0	0	0	0	0	0	0	0	0
SAL_PA9136AA	PNUSAS041030	Braenderup	0	0	0	0	0	0	0	0	0	0	0
SAL_PA9013AA	388725	Braenderup	0	0	0	0	0	0	0	0	0	0	0
SAL_PA9010AA	282499	Braenderup	0	0	0	0	0	0	0	0	0	0	0
SAL_PA8927AA	279405	Braenderup	0	0	0	0	0	0	0	0	0	0	0
SAL_PA8930AA	272486	Braenderup	0	0	0	0	0	0	0	0	0	0	0
SAL_PA8889AA	508254	Braenderup	0	0	0	0	0	0	0	0	0	0	0
SAL_PA9458AA	265277	Braenderup	0	0	0	0	0	0	0	0	0	0	0
SAL_PA9584AA	PNUSAS041273	Braenderup	0	0	0	0	0	0	0	0	0	0	0
SAL_PA9859AA	2018K-0087	Braenderup	0	0	0	0	0	0	0	0	0	0	0
SAL_PA9806AA	PNUSAS041477	Braenderup	0	0	0	0	0	0	0	0	0	0	0
SAL_PA9709AA	289053	Braenderup	0	0	0	0	0	0	0	0	0	0	0
SAL_PA9603AA	SAL-17-VL-LA-TX-0030	Braenderup	0	0	0	0	0	0	0	0	0	0	0
SAL_QA0201AA	PNUSAS042186	Braenderup	0	0	0	0	0	0	0	0	0	0	0
SAL_QA0073AA	PNUSAS041981	Braenderup	0	0	0	0	0	0	0	0	0	0	0
SAL_QA0064AA	PNUSAS041814	Braenderup	0	0	0	0	0	0	0	0	0	0	0
SAL_QA0068AA	PNUSAS040240	Braenderup	0	0	0	0	0	0	0	0	0	0	0
SAL_QA0034AA	PNUSAS041930	Braenderup	0	0	0	0	0	0	0	0	0	0	0
SAL_QA0020AA	PNUSAS041961	Braenderup	0	0	0	0	0	0	0	0	0	0	0
SAL_QA0322AA	432241	Braenderup	0	0	0	0	0	0	0	0	0	0	0
SAL_QA0279AA	427576	Braenderup	0	0	0	0	0	0	0	0	0	0	0
SAL_QA0420AA	275953	Braenderup	0	0	0	0	0	0	0	0	0	0	0
SAL_QA0402AA	349289	Braenderup	0	0	0	0	0	0	0	0	0	0	0
SAL_QA0547AA	PNUSAS040947	Braenderup	0	0	0	0	0	0	0	0	0	0	0
SAL_QA0541AA	PNUSAS041794	Braenderup	0	0	0	0	0	0	0	0	0	0	0
SAL_QA1975AA	23-6-18-8100481-V	Braenderup	0	0	0	0	0	0	0	0	0	0	0
SAL_QA0464AA	312827	Braenderup	0	0	0	0	0	0	0	0	0	0	0
SAL_QA0819AA	PNUSAS042152	Braenderup	0	0	0	0	0	0	0	0	0	0	0
SAL_QA1336AA	421178	Braenderup	0	0	0	0	0	0	0	0	0	0	0
SAL_QA1301AA	305256	Braenderup	0	0	0	0	0	0	0	0	0	0	0
SAL_QA1270AA	PNUSAS042193	Braenderup	0	0	0	0	0	0	0	0	0	0	0
SAL_QA1116AA	PNUSAS041785	Braenderup	0	0	0	0	0	0	0	0	0	0	0
SAL_QA1082AA	PNUSAS041788	Braenderup	0	0	0	0	0	0	0	0	0	0	0
SAL_QA1090AA	PNUSAS041787	Braenderup	0	0	0	0	0	0	0	0	0	0	0
SAL_QA1092AA	PNUSAS041789	Braenderup	0	0	0	0	0	0	0	0	0	0	0
SAL_QA1094AA	PNUSAS041792	Braenderup	0	0	0	0	0	0	0	0	0	0	0
SAL_QA1095AA	PNUSAS041786	Braenderup	0	0	0	0	0	0	0	0	0	0	0
SAL_QA1084AA	PNUSAS041790	Braenderup	0	0	0	0	0	0	0	0	0	0	0
SAL_QA1083AA	PNUSAS041791	Braenderup	0	0	0	0	0	0	0	0	0	0	0
SAL_QA3756AA	24-16-18-8100518-M	Braenderup	0	0	0	0	0	0	0	0	0	0	0
SAL_QA1063AA	PNUSAS041758	Braenderup	0	0	0	0	0	0	0	0	0	0	0
SAL_QA0984AA	PNUSAS042510	Braenderup	0	0	0	0	0	0	0	0	0	0	0
SAL_QA1496AA	420645	Braenderup	0	0	0	0	0	0	0	0	0	0	0
SAL_QA1566AA	PNUSAS042693	Braenderup	0	0	0	0	0	0	0	0	0	0	0
SAL_QA1927AA	315006	Braenderup	0	0	0	0	0	0	0	0	0	0	0
SAL_QA1898AA	291845	Braenderup	0	0	0	0	0	0	0	0	0	0	0
SAL_QA1908AA	304455	Braenderup	0	0	0	0	0	0	0	0	0	0	0
SAL_QA1915AA	279361	Braenderup	0	0	0	0	0	0	0	0	0	0	0
SAL_QA1854AA	423160	Braenderup	0	0	0	0	0	0	0	0	0	0	0

SAL_QA1856AA	138373	Braenderup	0	0	0	0	0	0	0	0	0	0	0
SAL_QA1808AA	173530	Braenderup	0	0	0	0	0	0	0	0	0	0	0
SAL_QA1810AA	353893	Braenderup	0	0	0	0	0	0	0	0	0	0	0
SAL_QA1751AA	249340	Braenderup	0	0	0	0	0	0	0	0	0	0	0
SAL_QA2470AA	503422	Braenderup	0	0	0	0	0	0	0	0	0	0	0
SAL_QA2462AA	300693	Braenderup	0	0	0	0	0	0	0	0	0	0	0
SAL_QA2461AA	296707	Braenderup	0	0	0	0	0	0	0	0	0	0	0
SAL_QA4966AA	25-9-18-8100527-Z	Braenderup	0	0	0	0	0	0	0	0	0	0	0
SAL_QA2353AA	392895	Braenderup	0	0	0	0	0	0	0	0	0	0	0
SAL_QA2303AA	482162	Braenderup	0	0	0	0	0	0	0	0	0	0	0
SAL_QA2242AA	113016	Braenderup	0	0	0	0	0	0	0	0	0	0	0
SAL_QA2274AA	421162	Braenderup	0	0	0	0	0	0	0	0	0	0	0
SAL_QA2227AA	306659	Braenderup	0	0	0	0	0	0	0	0	0	0	0
SAL_QA2074AA	306602	Braenderup	0	0	0	0	0	0	0	0	0	0	0
SAL_QA2044AA	473216	Braenderup	0	0	0	0	0	0	0	0	0	0	0
SAL_QA2779AA	323068	Braenderup	0	0	0	0	0	0	0	0	0	0	0
SAL_QA2767AA	414744	Braenderup	0	0	0	0	0	0	0	0	0	0	0
SAL_QA2728AA	191945	Braenderup	0	0	0	0	0	0	0	0	0	0	0
SAL_QA2718AA	119494	Braenderup	0	0	0	0	0	0	0	0	0	0	0
SAL_QA2698AA	441432	Braenderup	0	0	0	0	0	0	0	0	0	0	0
SAL_QA3103AA	PNUSAS043208	Braenderup	0	0	0	0	0	0	0	0	0	0	0
SAL_QA3083AA	PNUSAS043104	Braenderup	0	0	0	0	0	0	0	0	0	0	0
SAL_QA3061AA	PNUSAS041034	Braenderup	0	0	0	0	0	0	0	0	0	0	0
SAL_QA1939AA	215110	Braenderup	0	0	0	0	0	0	0	0	0	0	0
SAL_QA3473AA	182192	Braenderup	0	0	0	0	0	0	0	0	0	0	0
SAL_QA3422AA	274274	Braenderup	0	0	0	0	0	0	0	0	0	0	0
SAL_QA3437AA	173729	Braenderup	0	0	0	0	0	0	0	0	0	0	0
SAL_QA3364AA	147851	Braenderup	0	0	0	0	0	0	0	0	0	0	0
SAL_QA3271AA	105336	Braenderup	0	0	0	0	0	0	0	0	0	0	0
SAL_QA3227AA	427564	Braenderup	0	0	0	0	0	0	0	0	0	0	0
SAL_QA3226AA	138369	Braenderup	0	0	0	0	0	0	0	0	0	0	0
SAL_QA3533AA	PNUSAS039059	Braenderup	0	0	0	0	0	0	0	0	0	0	0
SAL_QA3586AA	PNUSAS042884	Braenderup	0	0	0	0	0	0	0	0	0	0	0
SAL_QA3585AA	PNUSAS042118	Braenderup	0	0	0	0	0	0	0	0	0	0	0
SAL_QA3921AA	342931	Braenderup	0	0	0	0	0	0	0	0	0	0	0
SAL_QA3889AA	416033	Braenderup	0	0	0	0	0	0	0	0	0	0	0
SAL_QA3869AA	129508	Braenderup	0	0	0	0	0	0	0	0	0	0	0
SAL_QA3850AA	394906	Braenderup	0	0	0	0	0	0	0	0	0	0	0
SAL_QA3790AA	362104	Braenderup	0	0	0	0	0	0	0	0	0	0	0
SAL_QA2464AA	296843	Braenderup	0	0	0	0	0	0	0	0	0	0	0
SAL_QA3962AA	FSIS21821887	Braenderup	0	0	0	0	0	0	0	0	0	0	0
SAL_QA3951AA	FSIS21821894	Braenderup	0	0	0	0	0	0	0	0	0	0	0
SAL_QA4148AA	123875	Braenderup	0	0	0	0	0	0	0	0	0	0	0
SAL_QA4023AA	548560	Braenderup	0	0	0	0	0	0	0	0	0	0	0
SAL_QA3998AA	435427	Braenderup	0	0	0	0	0	0	0	0	0	0	0
SAL_QA3972AA	NY88800902	Braenderup	0	0	0	0	0	0	0	0	0	0	0
SAL_QA3973AA	NY88691779	Braenderup	0	0	0	0	0	0	0	0	0	0	0
SAL_QA3977AA	FDA00007829	Braenderup	0	0	0	0	0	0	0	0	0	0	0
SAL_QA3978AA	NY88691739	Braenderup	0	0	0	0	0	0	0	0	0	0	0
SAL_QA4013AA	392799	Braenderup	0	0	0	0	0	0	0	0	0	0	0
SAL_QA4266AA	PNUSAS041796	Braenderup	0	0	0	0	0	0	0	0	0	0	0
SAL_QA4270AA	PNUSAS041797	Braenderup	0	0	0	0	0	0	0	0	0	0	0
SAL_QA4238AA	PNUSAS042521	Braenderup	0	0	0	0	0	0	0	0	0	0	0
SAL_QA4242AA	PNUSAS042520	Braenderup	0	0	0	0	0	0	0	0	0	0	0
SAL_QA4188AA	PNUSAS040688	Braenderup	0	0	0	0	0	0	0	0	0	0	0
SAL_QA4170AA	PNUSAS042005	Braenderup	0	0	0	0	0	0	0	0	0	0	0
SAL_QA4538AA	PNUSAS043506	Braenderup	0	0	0	0	0	0	0	0	0	0	0
SAL_QA4473AA	PNUSAS039506	Braenderup	0	0	0	0	0	0	0	0	0	0	0
SAL_QA4466AA	PNUSAS039990	Braenderup	0	0	0	0	0	0	0	0	0	0	0
SAL_QA4626AA	PNUSAS042762	Braenderup	0	0	0	0	0	0	0	0	0	0	0
SAL_QA4631AA	PNUSAS041746	Braenderup	0	0	0	0	0	0	0	0	0	0	0
SAL_QA4622AA	PNUSAS042003	Braenderup	0	0	0	0	0	0	0	0	0	0	0
SAL_QA4388AA	PNUSAS040691	Braenderup	0	0	0	0	0	0	0	0	0	0	0
SAL_QA4858AA	290805	Braenderup	0	0	0	0	0	0	0	0	0	0	0
SAL_QA4746AA	261379	Braenderup	0	0	0	0	0	0	0	0	0	0	0
SAL_QA4742AA	211769	Braenderup	0	0	0	0	0	0	0	0	0	0	0
SAL_QA4758AA	306718	Braenderup	0	0	0	0	0	0	0	0	0	0	0
SAL_QA4727AA	360327	Braenderup	0	0	0	0	0	0	0	0	0	0	0
SAL_QA5559AA	442673	Braenderup	0	0	0	0	0	0	0	0	0	0	0
SAL_QA5445AA	278606	Braenderup	0	0	0	0	0	0	0	0	0	0	0
SAL_QA5459AA	131464	Braenderup	0	0	0	0	0	0	0	0	0	0	0
SAL_QA3593AA	PNUSAS042252	Braenderup	0	0	0	0	0	0	0	0	0	0	0
SAL_QA5231AA	418225	Braenderup	0	0	0	0	0	0	0	0	0	0	0

SAL_QA5668AA	PNUSAS041462	Braenderup	0	0	0	0	0	0	0	0	0	0	0
SAL_QA5644AA	PNUSAS043643	Braenderup	0	0	0	0	0	0	0	0	0	0	0
SAL_QA5157AA	267737	Braenderup	0	0	0	0	0	0	0	0	0	0	0
SAL_QA5696AA	PNUSAS042989	Braenderup	0	0	0	0	0	0	0	0	0	0	0
SAL_QA5984AA	TTU_010	Braenderup	0	0	0	0	0	0	0	0	0	0	0
SAL_QA5985AA	TTU_007	Braenderup	0	0	0	0	0	0	0	0	0	0	0
SAL_QA5982AA	TTU_008	Braenderup	0	0	0	0	0	0	0	0	0	0	0
SAL_QA5915AA	FSIS11810737	Braenderup	0	0	0	0	0	0	0	0	0	0	0
SAL_QA5906AA	369991	Braenderup	0	0	0	0	0	0	0	0	0	0	0
SAL_QA5844AA	300664	Braenderup	0	0	0	0	0	0	0	0	0	0	0
SAL_QA5740AA	TTU_009	Braenderup	0	0	0	0	0	0	0	0	0	0	0
SAL_QA6017AA	PNUSAS043231	Braenderup	0	0	0	0	0	0	0	0	0	0	0
SAL_QA6043AA	PNUSAS043790	Braenderup	0	0	0	0	0	0	0	0	0	0	0
SAL_QA6008AA	PNUSAS043579	Braenderup	0	0	0	0	0	0	0	0	0	0	0
SAL_QA5989AA	PNUSAS041873	Braenderup	0	0	0	0	0	0	0	0	0	0	0
SAL_QA6239AA	369002	Braenderup	0	0	0	0	0	0	0	0	0	0	0
SAL_QA6245AA	294963	Braenderup	0	0	0	0	0	0	0	0	0	0	0
SAL_QA6160AA	336960	Braenderup	0	0	0	0	0	0	0	0	0	0	0
SAL_QA6175AA	107117	Braenderup	0	0	0	0	0	0	0	0	0	0	0
SAL_QA6349AA	PNUSAS044187	Braenderup	0	0	0	0	0	0	0	0	0	0	0
SAL_QA6366AA	PNUSAS043612	Braenderup	0	0	0	0	0	0	0	0	0	0	0
SAL_QA6616AA	205626	Braenderup	0	0	0	0	0	0	0	0	0	0	0
SAL_QA6588AA	302530	Braenderup	0	0	0	0	0	0	0	0	0	0	0
SAL_QA6570AA	265765	Braenderup	0	0	0	0	0	0	0	0	0	0	0
SAL_QA6543AA	267834	Braenderup	0	0	0	0	0	0	0	0	0	0	0
SAL_QA6384AA	TTU_021	Braenderup	0	0	0	0	0	0	0	0	0	0	0
SAL_QA6394AA	TTU_030	Braenderup	0	0	0	0	0	0	0	0	0	0	0
SAL_QA6444AA	300675	Braenderup	0	0	0	0	0	0	0	0	0	0	0
SAL_QA6805AA	370595	Braenderup	0	0	0	0	0	0	0	0	0	0	0
SAL_QA6755AA	298788	Braenderup	0	0	0	0	0	0	0	0	0	0	0
SAL_QA6686AA	TTU_016	Braenderup	0	0	0	0	0	0	0	0	0	0	0
SAL_QA3831AA	128184	Braenderup	0	0	0	0	0	0	0	0	0	0	0
SAL_QA7186AA	PNUSAS042286	Braenderup	0	0	0	0	0	0	0	0	0	0	0
SAL_QA7190AA	PNUSAS042273	Braenderup	0	0	0	0	0	0	0	0	0	0	0
SAL_QA7194AA	PNUSAS043492	Braenderup	0	0	0	0	0	0	0	0	0	0	0
SAL_QA7196AA	PNUSAS043625	Braenderup	0	0	0	0	0	0	0	0	0	0	0
SAL_QA7158AA	PNUSAS041652	Braenderup	0	0	0	0	0	0	0	0	0	0	0
SAL_QA7154AA	PNUSAS043483	Braenderup	0	0	0	0	0	0	0	0	0	0	0
SAL_QA7127AA	PNUSAS041102	Braenderup	0	0	0	0	0	0	0	0	0	0	0
SAL_QA7087AA	PNUSAS041114	Braenderup	0	0	0	0	0	0	0	0	0	0	0
SAL_QA7125AA	PNUSAS043482	Braenderup	0	0	0	0	0	0	0	0	0	0	0
SAL_QA7131AA	PNUSAS041121	Braenderup	0	0	0	0	0	0	0	0	0	0	0
SAL_QA6954AA	PNUSAS043430	Braenderup	0	0	0	0	0	0	0	0	0	0	0
SAL_QA7454AA	423158	Braenderup	0	0	0	0	0	0	0	0	0	0	0
SAL_QA7388AA	534011	Braenderup	0	0	0	0	0	0	0	0	0	0	0
SAL_QA7295AA	271067	Braenderup	0	0	0	0	0	0	0	0	0	0	0
SAL_QA7308AA	279386	Braenderup	0	0	0	0	0	0	0	0	0	0	0
SAL_QA7233AA	306594	Braenderup	0	0	0	0	0	0	0	0	0	0	0
SAL_QA7901AA	313849	Braenderup	0	0	0	0	0	0	0	0	0	0	0
SAL_QA8139AA	FSIS11810939	Braenderup	0	0	0	0	0	0	0	0	0	0	0
SAL_QA7620AA	193290	Braenderup	0	0	0	0	0	0	0	0	0	0	0
SAL_QA8057AA	FSIS11810940	Braenderup	0	0	0	0	0	0	0	0	0	0	0
SAL_QA7588AA	253359	Braenderup	0	0	0	0	0	0	0	0	0	0	0
SAL_QA7538AA	388638	Braenderup	0	0	0	0	0	0	0	0	0	0	0
SAL_QA7502AA	299416	Braenderup	0	0	0	0	0	0	0	0	0	0	0
SAL_QA7464AA	127429	Braenderup	0	0	0	0	0	0	0	0	0	0	0
SAL_QA8376AA	313199	Braenderup	0	0	0	0	0	0	0	0	0	0	0
SAL_QA8335AA	300818	Braenderup	0	0	0	0	0	0	0	0	0	0	0
SAL_QA8236AA	103764	Braenderup	0	0	0	0	0	0	0	0	0	0	0
SAL_QA8237AA	180531	Braenderup	0	0	0	0	0	0	0	0	0	0	0
SAL_QA8164AA	425584	Braenderup	0	0	0	0	0	0	0	0	0	0	0
SAL_QA8144AA	FDA00009760	Braenderup	0	0	0	0	0	0	0	0	0	0	0
SAL_RA4607AA	456168	Braenderup	0	0	0	0	0	0	0	0	0	0	0
SAL_RA4631AA	424712	Braenderup	0	0	0	0	0	0	0	0	0	0	0
SAL_QA8520AA	TTU_033	Braenderup	0	0	0	0	0	0	0	0	0	0	0
SAL_QA8861AA	557295	Braenderup	0	0	0	0	0	0	0	0	0	0	0
SAL_QA8712AA	280670	Braenderup	0	0	0	0	0	0	0	0	0	0	0
SAL_QA8691AA	304594	Braenderup	0	0	0	0	0	0	0	0	0	0	0
SAL_QA8677AA	294970	Braenderup	0	0	0	0	0	0	0	0	0	0	0
SAL_QA8641AA	274312	Braenderup	0	0	0	0	0	0	0	0	0	0	0
SAL_QA8623AA	FSIS11810966	Braenderup	0	0	0	0	0	0	0	0	0	0	0
SAL_QA9020AA	PNUSAS042029	Braenderup	0	0	0	0	0	0	0	0	0	0	0
SAL_QA9003AA	PNUSAS044240	Braenderup	0	0	0	0	0	0	0	0	0	0	0

SAL_QA9136AA	PNUSAS044608	Braenderup	0	0	0	0	0	0	0	0	0	0	0
SAL_QA9150AA	PNUSAS044510	Braenderup	0	0	0	0	0	0	0	0	0	0	0
SAL_QA9127AA	PNUSAS044655	Braenderup	0	0	0	0	0	0	0	0	0	0	0
SAL_QA9096AA	PNUSAS044609	Braenderup	0	0	0	0	0	0	0	0	0	0	0
SAL_QA9050AA	PNUSAS044245	Braenderup	0	0	0	0	0	0	0	0	0	0	0
SAL_QA8928AA	PNUSAS044435	Braenderup	0	0	0	0	0	0	0	0	0	0	0
SAL_QA8892AA	PNUSAS043684	Braenderup	0	0	0	0	0	0	0	0	0	0	0
SAL_QA9530AA	205625	Braenderup	0	0	0	0	0	0	0	0	0	0	0
SAL_QA9408AA	429805	Braenderup	0	0	0	0	0	0	0	0	0	0	0
SAL_QA9636AA	PNUSAS043867	Braenderup	0	0	0	0	0	0	0	0	0	0	0
SAL_QA9270AA	200625	Braenderup	0	0	0	0	0	0	0	0	0	0	0
SAL_QA9965AA	155604	Braenderup	0	0	0	0	0	0	0	0	0	0	0
SAL_QA9981AA	157372	Braenderup	0	0	0	0	0	0	0	0	0	0	0
SAL_RA0164AA	PNUSAS025986	Braenderup	0	0	0	0	0	0	0	0	0	0	0
SAL_RA0458AA	PNUSAS043413	Braenderup	0	0	0	0	0	0	0	0	0	0	0
SAL_RA0441AA	325715	Braenderup	0	0	0	0	0	0	0	0	0	0	0
SAL_RA0402AA	164688	Braenderup	0	0	0	0	0	0	0	0	0	0	0
SAL_RA0400AA	190667	Braenderup	0	0	0	0	0	0	0	0	0	0	0
SAL_RA0411AA	451990	Braenderup	0	0	0	0	0	0	0	0	0	0	0
SAL_RA0340AA	294996	Braenderup	0	0	0	0	0	0	0	0	0	0	0
SAL_RA0304AA	383832	Braenderup	0	0	0	0	0	0	0	0	0	0	0
SAL_RA0278AA	560709	Braenderup	0	0	0	0	0	0	0	0	0	0	0
SAL_RA1075AA	164698	Braenderup	0	0	0	0	0	0	0	0	0	0	0
SAL_RA1058AA	290809	Braenderup	0	0	0	0	0	0	0	0	0	0	0
SAL_RA1001AA	232780	Braenderup	0	0	0	0	0	0	0	0	0	0	0
SAL_RA0999AA	196425	Braenderup	0	0	0	0	0	0	0	0	0	0	0
SAL_RA0959AA	140253	Braenderup	0	0	0	0	0	0	0	0	0	0	0
SAL_RA0938AA	360333	Braenderup	0	0	0	0	0	0	0	0	0	0	0
SAL_RA0882AA	178729	Braenderup	0	0	0	0	0	0	0	0	0	0	0
SAL_RA0818AA	FSIS11810639	Braenderup	0	0	0	0	0	0	0	0	0	0	0
SAL_RA0796AA	432246	Braenderup	0	0	0	0	0	0	0	0	0	0	0
SAL_RA0760AA	298791	Braenderup	0	0	0	0	0	0	0	0	0	0	0
SAL_RA0717AA	306720	Braenderup	0	0	0	0	0	0	0	0	0	0	0
SAL_RA0671AA	294933	Braenderup	0	0	0	0	0	0	0	0	0	0	0
SAL_RA0610AA	385832	Braenderup	0	0	0	0	0	0	0	0	0	0	0
SAL_RA0597AA	294965	Braenderup	0	0	0	0	0	0	0	0	0	0	0
SAL_RA0600AA	288749	Braenderup	0	0	0	0	0	0	0	0	0	0	0
SAL_RA0573AA	273545	Braenderup	0	0	0	0	0	0	0	0	0	0	0
SAL_RA1156AA	PNUSAS044891	Braenderup	0	0	0	0	0	0	0	0	0	0	0
SAL_RA1313AA	313309	Braenderup	0	0	0	0	0	0	0	0	0	0	0
SAL_RA1262AA	FSIS11811189	Braenderup	0	0	0	0	0	0	0	0	0	0	0
SAL_RA1233AA	PNUSAS044947	Braenderup	0	0	0	0	0	0	0	0	0	0	0
SAL_RA1250AA	FSIS11811256	Braenderup	0	0	0	0	0	0	0	0	0	0	0
SAL_RA1227AA	PNUSAS044833	Braenderup	0	0	0	0	0	0	0	0	0	0	0
SAL_RA1420AA	PNUSAS045288	Braenderup	0	0	0	0	0	0	0	0	0	0	0
SAL_RA1403AA	PNUSAS045012	Braenderup	0	0	0	0	0	0	0	0	0	0	0
SAL_RA1402AA	PNUSAS044397	Braenderup	0	0	0	0	0	0	0	0	0	0	0
SAL_RA2551AA	PNUSAS044094	Braenderup	0	0	0	0	0	0	0	0	0	0	0
SAL_RA2513AA	PNUSAS045031	Braenderup	0	0	0	0	0	0	0	0	0	0	0
SAL_RA2490AA	PNUSAS043950	Braenderup	0	0	0	0	0	0	0	0	0	0	0
SAL_RA2744AA	374598	Braenderup	0	0	0	0	0	0	0	0	0	0	0
SAL_RA2879AA	267811	Braenderup	0	0	0	0	0	0	0	0	0	0	0
SAL_RA3072AA	PNUSAS044841	Braenderup	0	0	0	0	0	0	0	0	0	0	0
SAL_RA3079AA	PNUSAS044857	Braenderup	0	0	0	0	0	0	0	0	0	0	0
SAL_RA3056AA	PNUSAS045410	Braenderup	0	0	0	0	0	0	0	0	0	0	0
SAL_RA3001AA	PNUSAS043878	Braenderup	0	0	0	0	0	0	0	0	0	0	0
SAL_RA3489AA	160790	Braenderup	0	0	0	0	0	0	0	0	0	0	0
SAL_RA3477AA	241823	Braenderup	0	0	0	0	0	0	0	0	0	0	0
SAL_RA3480AA	563466	Braenderup	0	0	0	0	0	0	0	0	0	0	0
SAL_RA3384AA	563418	Braenderup	0	0	0	0	0	0	0	0	0	0	0
SAL_RA3346AA	170321	Braenderup	0	0	0	0	0	0	0	0	0	0	0
SAL_RA3353AA	266736	Braenderup	0	0	0	0	0	0	0	0	0	0	0
SAL_RA3504AA	PNUSAS044826	Braenderup	0	0	0	0	0	0	0	0	0	0	0
SAL_RA3493AA	PNUSAS044810	Braenderup	0	0	0	0	0	0	0	0	0	0	0
SAL_RA3507AA	PNUSAS043289	Braenderup	0	0	0	0	0	0	0	0	0	0	0
SAL_RA3687AA	562941	Braenderup	0	0	0	0	0	0	0	0	0	0	0
SAL_RA3710AA	204001	Braenderup	0	0	0	0	0	0	0	0	0	0	0
SAL_RA3662AA	560697	Braenderup	0	0	0	0	0	0	0	0	0	0	0
SAL_RA3624AA	337110	Braenderup	0	0	0	0	0	0	0	0	0	0	0
SAL_RA3588AA	548865	Braenderup	0	0	0	0	0	0	0	0	0	0	0
SAL_RA3594AA	293740	Braenderup	0	0	0	0	0	0	0	0	0	0	0
SAL_RA3582AA	529255	Braenderup	0	0	0	0	0	0	0	0	0	0	0
SAL_RA4151AA	339518	Braenderup	0	0	0	0	0	0	0	0	0	0	0

SAL_RA4053AA	444079	Braenderup	0	0	0	0	0	0	0	0	0	0	0
SAL_RA3985AA	261331	Braenderup	0	0	0	0	0	0	0	0	0	0	0
SAL_RA4197AA	TTU_223	Braenderup	0	0	0	0	0	0	0	0	0	0	0
SAL_RA4202AA	TTU_226	Braenderup	0	0	0	0	0	0	0	0	0	0	0
SAL_RA3881AA	TTU_032	Braenderup	0	0	0	0	0	0	0	0	0	0	0
SAL_RA3887AA	TTU_034	Braenderup	0	0	0	0	0	0	0	0	0	0	0
SAL_RA3867AA	TTU_017	Braenderup	0	0	0	0	0	0	0	0	0	0	0
SAL_RA3873AA	TTU_029	Braenderup	0	0	0	0	0	0	0	0	0	0	0
SAL_RA3883AA	TTU_011	Braenderup	0	0	0	0	0	0	0	0	0	0	0
SAL_RA3886AA	TTU_012	Braenderup	0	0	0	0	0	0	0	0	0	0	0
SAL_RA4261AA	TTU_015	Braenderup	0	0	0	0	0	0	0	0	0	0	0
SAL_RA4262AA	TTU_026	Braenderup	0	0	0	0	0	0	0	0	0	0	0
SAL_QA9957AA	282498	Braenderup	0	0	0	0	0	0	0	0	0	0	0
SAL_RA4257AA	TTU_031	Braenderup	0	0	0	0	0	0	0	0	0	0	0
SAL_RA4259AA	TTU_018	Braenderup	0	0	0	0	0	0	0	0	0	0	0
SAL_RA4267AA	NY89351971	Braenderup	0	0	0	0	0	0	0	0	0	0	0
SAL_RA4563AA	215107	Braenderup	0	0	0	0	0	0	0	0	0	0	0
SAL_RA4521AA	296854	Braenderup	0	0	0	0	0	0	0	0	0	0	0
SAL_RA4549AA	214731	Braenderup	0	0	0	0	0	0	0	0	0	0	0
SAL_RA4537AA	492503	Braenderup	0	0	0	0	0	0	0	0	0	0	0
SAL_RA4468AA	215101	Braenderup	0	0	0	0	0	0	0	0	0	0	0
SAL_RA4471AA	302942	Braenderup	0	0	0	0	0	0	0	0	0	0	0
SAL_RA4313AA	216309	Braenderup	0	0	0	0	0	0	0	0	0	0	0
SAL_RA2508AA	PNUSAS045025	Braenderup	0	0	0	0	0	0	0	0	0	0	0
SAL_RA6162AA	Avian	Braenderup	0	0	0	0	0	0	0	0	0	0	0
SAL_RA6139AA	NC_WHO_S008	Braenderup	0	0	0	0	0	0	0	0	0	0	0
SAL_RA6073AA	PNUSAS043177	Braenderup	0	0	0	0	0	0	0	0	0	0	0
SAL_RA6076AA	PNUSAS045352	Braenderup	0	0	0	0	0	0	0	0	0	0	0
SAL_RA6007AA	PNUSAS045385	Braenderup	0	0	0	0	0	0	0	0	0	0	0
SAL_RA6008AA	PNUSAS045386	Braenderup	0	0	0	0	0	0	0	0	0	0	0
SAL_RA5995AA	PNUSAS045369	Braenderup	0	0	0	0	0	0	0	0	0	0	0
SAL_RA5969AA	PNUSAS044870	Braenderup	0	0	0	0	0	0	0	0	0	0	0
SAL_RA5977AA	PNUSAS045368	Braenderup	0	0	0	0	0	0	0	0	0	0	0
SAL_RA5983AA	PNUSAS045361	Braenderup	0	0	0	0	0	0	0	0	0	0	0
SAL_RA5916AA	PNUSAS044046	Braenderup	0	0	0	0	0	0	0	0	0	0	0
SAL_RA5924AA	PNUSAS043503	Braenderup	0	0	0	0	0	0	0	0	0	0	0
SAL_RA5885AA	PNUSAS045634	Braenderup	0	0	0	0	0	0	0	0	0	0	0
SAL_RA5663AA	215103	Braenderup	0	0	0	0	0	0	0	0	0	0	0
SAL_RA5558AA	456169	Braenderup	0	0	0	0	0	0	0	0	0	0	0
SAL_RA5689AA	218451	Braenderup	0	0	0	0	0	0	0	0	0	0	0
SAL_RA5440AA	215102	Braenderup	0	0	0	0	0	0	0	0	0	0	0
SAL_RA5441AA	557668	Braenderup	0	0	0	0	0	0	0	0	0	0	0
SAL_RA2677AA	310044	Braenderup	0	0	0	0	0	0	0	0	0	0	0
SAL_RA2670AA	158917	Braenderup	0	0	0	0	0	0	0	0	0	0	0
SAL_RA2632AA	548526	Braenderup	0	0	0	0	0	0	0	0	0	0	0
SAL_RA6378AA	PNUSAS046059	Braenderup	0	0	0	0	0	0	0	0	0	0	0
SAL_RA6681AA	FSIS11811672	Braenderup	0	0	0	0	0	0	0	0	0	0	0
SAL_RA6674AA	215104	Braenderup	0	0	0	0	0	0	0	0	0	0	0
SAL_RA6662AA	215106	Braenderup	0	0	0	0	0	0	0	0	0	0	0
SAL_RA6632AA	311976	Braenderup	0	0	0	0	0	0	0	0	0	0	0
SAL_SA1531AA	M35	Braenderup	0	0	0	0	0	0	0	0	0	0	0
SAL_SA1532AA	M19	Braenderup	0	0	0	0	0	0	0	0	0	0	0
SAL_SA1533AA	M258	Braenderup	0	0	0	0	0	0	0	0	0	0	0
SAL_RA6576AA	238467	Braenderup	0	0	0	0	0	0	0	0	0	0	0
SAL_RA6491AA	311977	Braenderup	0	0	0	0	0	0	0	0	0	0	0
SAL_RA6820AA	PNUSAS046208	Braenderup	0	0	0	0	0	0	0	0	0	0	0
SAL_RA6209AA	PNUSAS045223	Braenderup	0	0	0	0	0	0	0	0	0	0	0
SAL_RA6866AA	FSIS11811758	Braenderup	0	0	0	0	0	0	0	0	0	0	0
SAL_RA6878AA	FSIS11811762	Braenderup	0	0	0	0	0	0	0	0	0	0	0
SAL_RA7497AA	431401	Braenderup	0	0	0	0	0	0	0	0	0	0	0
SAL_RA7514AA	412230	Braenderup	0	0	0	0	0	0	0	0	0	0	0
SAL_RA7387AA	218453	Braenderup	0	0	0	0	0	0	0	0	0	0	0
SAL_RA7332AA	290698	Braenderup	0	0	0	0	0	0	0	0	0	0	0
SAL_RA7246AA	242379	Braenderup	0	0	0	0	0	0	0	0	0	0	0
SAL_RA7598AA	PNUSAS042871	Braenderup	0	0	0	0	0	0	0	0	0	0	0
SAL_RA7601AA	PNUSAS042870	Braenderup	0	0	0	0	0	0	0	0	0	0	0
SAL_RA7581AA	PNUSAS042873	Braenderup	0	0	0	0	0	0	0	0	0	0	0
SAL_RA7568AA	PNUSAS040515	Braenderup	0	0	0	0	0	0	0	0	0	0	0
SAL_RA7549AA	PNUSAS046031	Braenderup	0	0	0	0	0	0	0	0	0	0	0
SAL_RA7181AA	413199	Braenderup	0	0	0	0	0	0	0	0	0	0	0
SAL_RA7798AA	159888	Braenderup	0	0	0	0	0	0	0	0	0	0	0
SAL_RA7905AA	FSIS11811721	Braenderup	0	0	0	0	0	0	0	0	0	0	0
SAL_RA8090AA	569986	Braenderup	0	0	0	0	0	0	0	0	0	0	0

SAL_RA8058AA	271146	Braenderup	0	0	0	0	0	0	0	0	0	0	0
SAL_RA8061AA	175578	Braenderup	0	0	0	0	0	0	0	0	0	0	0
SAL_RA8009AA	456167	Braenderup	0	0	0	0	0	0	0	0	0	0	0
SAL_RA8013AA	280658	Braenderup	0	0	0	0	0	0	0	0	0	0	0
SAL_RA7962AA	287572	Braenderup	0	0	0	0	0	0	0	0	0	0	0
SAL_RA7936AA	313277	Braenderup	0	0	0	0	0	0	0	0	0	0	0
SAL_RA8304AA	296842	Braenderup	0	0	0	0	0	0	0	0	0	0	0
SAL_RA8234AA	157327	Braenderup	0	0	0	0	0	0	0	0	0	0	0
SAL_RA8285AA	532488	Braenderup	0	0	0	0	0	0	0	0	0	0	0
SAL_RA8369AA	FSIS11811976	Braenderup	0	0	0	0	0	0	0	0	0	0	0
SAL_RA8483AA	CFSAN082662	Braenderup	0	0	0	0	0	0	0	0	0	0	0
SAL_RA8482AA	CFSAN082659	Braenderup	0	0	0	0	0	0	0	0	0	0	0
SAL_RA8486AA	CFSAN082660	Braenderup	0	0	0	0	0	0	0	0	0	0	0
SAL_RA8484AA	CFSAN082663	Braenderup	0	0	0	0	0	0	0	0	0	0	0
SAL_RA5685AA	183610	Braenderup	0	0	0	0	0	0	0	0	0	0	0
SAL_RA5451AA	223499	Braenderup	0	0	0	0	0	0	0	0	0	0	0
SAL_RA9212AA	FSIS11812279	Braenderup	0	0	0	0	0	0	0	0	0	0	0
SAL_RA9779AA	TTU_020	Braenderup	0	0	0	0	0	0	0	0	0	0	0
SAL_RA9775AA	TTU_019	Braenderup	0	0	0	0	0	0	0	0	0	0	0
SAL_RA9772AA	TTU_227	Braenderup	0	0	0	0	0	0	0	0	0	0	0
SAL_SA0989AA	FSIS11812815	Braenderup	0	0	0	0	0	0	0	0	0	0	0
SAL_SA1129AA	FSIS11813007	Braenderup	0	0	0	0	0	0	0	0	0	0	0
SAL_SA2686AA	FSIS11813224	Braenderup	0	0	0	0	0	0	0	0	0	0	0
SAL_SA3355AA	FSIS11813284	Braenderup	0	0	0	0	0	0	0	0	0	0	0
SAL_SA3441AA	CFSAN064218	Braenderup	0	0	0	0	0	0	0	0	0	0	0
SAL_SA3429AA	FSIS21822005	Braenderup	0	0	0	0	0	0	0	0	0	0	0
SAL_SA3611AA	FSIS31800954	Braenderup	0	0	0	0	0	0	0	0	0	0	0
SAL_SA3612AA	FSIS21822025	Braenderup	0	0	0	0	0	0	0	0	0	0	0
SAL_SA3658AA	FSIS11813672	Braenderup	0	0	0	0	0	0	0	0	0	0	0
SAL_SA3789AA	SAL-18-VL-NY-FL-0001	Braenderup	0	0	0	0	0	0	0	0	0	0	0
SAL_SA4345AA	FSIS21822086	Braenderup	0	0	0	0	0	0	0	0	0	0	0
SAL_SA4311AA	FSIS31801013	Braenderup	0	0	0	0	0	0	0	0	0	0	0
SAL_SA4884AA	579269	Braenderup	0	0	0	0	0	0	0	0	0	0	0
SAL_SA4895AA	579244	Braenderup	0	0	0	0	0	0	0	0	0	0	0
SAL_SA4875AA	572019	Braenderup	0	0	0	0	0	0	0	0	0	0	0
SAL_SA4813AA	585961	Braenderup	0	0	0	0	0	0	0	0	0	0	0
SAL_SA4736AA	584633	Braenderup	0	0	0	0	0	0	0	0	0	0	0
SAL_SA4744AA	588492	Braenderup	0	0	0	0	0	0	0	0	0	0	0
SAL_SA5985AA	599579	Braenderup	0	0	0	0	0	0	0	0	0	0	0
SAL_SA5976AA	587823	Braenderup	0	0	0	0	0	0	0	0	0	0	0
SAL_SA5942AA	579896	Braenderup	0	0	0	0	0	0	0	0	0	0	0
SAL_SA5809AA	585943	Braenderup	0	0	0	0	0	0	0	0	0	0	0
SAL_SA6469AA	573554	Braenderup	0	0	0	0	0	0	0	0	0	0	0
SAL_SA6193AA	556821	Braenderup	0	0	0	0	0	0	0	0	0	0	0
SAL_SA6758AA	590462	Braenderup	0	0	0	0	0	0	0	0	0	0	0
SAL_SA6680AA	586080	Braenderup	0	0	0	0	0	0	0	0	0	0	0
SAL_SA7123AA	590853	Braenderup	0	0	0	0	0	0	0	0	0	0	0
SAL_SA7510AA	588917	Braenderup	0	0	0	0	0	0	0	0	0	0	0
SAL_SA7696AA	278112	Braenderup	0	0	0	0	0	0	0	0	0	0	0
SAL_SA7682AA	329132	Braenderup	0	0	0	0	0	0	0	0	0	0	0
SAL_SA7256AA	602754	Braenderup	0	0	0	0	0	0	0	0	0	0	0
SAL_SA7962AA	296692	Braenderup	0	0	0	0	0	0	0	0	0	245	0
SAL_SA7890AA	300737	Braenderup	0	0	0	0	0	0	0	0	0	0	0
SAL_SA8735AA	414738	Braenderup	0	0	0	0	0	0	0	0	0	0	0
SAL_SA8975AA	608529	Braenderup	0	0	0	0	0	0	0	0	0	0	0
SAL_SA7493AA	582942	Braenderup	0	0	0	0	0	0	0	0	0	0	0
SAL_SA8893AA	321066	Braenderup	0	0	0	0	0	0	0	0	0	0	0
SAL_SA8896AA	421165	Braenderup	0	0	0	0	0	0	0	0	0	0	0
SAL_SA9006AA	FDA00007833	Braenderup	0	0	0	0	0	0	0	0	0	0	0
SAL_SA8837AA	FSIS11813719	Braenderup	0	0	0	0	0	0	0	0	0	0	0
SAL_SA9561AA	538390	Braenderup	0	0	0	0	0	0	0	0	0	0	0
SAL_SA9553AA	608534	Braenderup	0	0	0	0	0	0	0	0	0	0	0
SAL_SA9527AA	609431	Braenderup	0	0	0	0	0	0	0	0	0	0	0
SAL_SA9472AA	608541	Braenderup	0	0	0	0	0	0	0	0	0	0	0
SAL_SA9475AA	369994	Braenderup	0	0	0	0	0	0	0	0	0	0	0
SAL_SA9430AA	134035	Braenderup	0	0	0	0	0	0	0	0	0	0	0
SAL_SA8945AA	607377	Braenderup	0	0	0	0	0	0	0	0	0	0	0
SAL_SA9721AA	FSIS21822172	Braenderup	0	0	0	0	0	0	0	0	0	0	0
SAL_TA0314AA	610784	Braenderup	0	0	0	0	0	0	0	0	0	0	0
SAL_TA0907AA	613805	Braenderup	0	0	0	0	0	0	0	0	0	0	0
SAL_TA0816AA	615608	Braenderup	0	0	0	0	0	0	0	0	0	0	0
SAL_TA0743AA	579109	Braenderup	0	0	0	0	0	0	0	0	0	0	0
SAL_TA0744AA	275765	Braenderup	0	0	0	0	0	0	0	0	0	0	0

SAL_TA0751AA	617013	Braenderup	0	0	0	0	0	0	0	0	0	0	0
SAL_TA0754AA	616287	Braenderup	0	0	0	0	0	0	0	0	0	0	0
SAL_TA1267AA	OH-18-20073-2T	Braenderup	0	0	0	0	0	0	0	0	0	0	0
SAL_TA1252AA	FDA00007834	Braenderup	0	0	0	0	0	0	0	0	0	0	0
SAL_TA1253AA	FDA00007835	Braenderup	0	0	0	0	0	0	0	0	0	0	0
SAL_TA2423AA	20151013A18	Braenderup	0	0	0	0	0	0	0	0	0	0	0
SAL_TA2433AA	20151013B22	Braenderup	0	0	0	0	0	0	0	0	0	0	0
SAL_TA2435AA	20151013C4	Braenderup	0	0	0	0	0	0	0	0	0	0	0
SAL_TA2381AA	623509	Braenderup	0	0	0	0	0	0	0	0	0	0	0
SAL_TA2394AA	624476	Braenderup	0	0	0	0	0	0	0	0	0	0	0
SAL_TA2297AA	624459	Braenderup	0	0	0	0	0	0	0	0	0	0	0
SAL_TA2809AA	20151007C21	Braenderup	0	0	0	0	0	0	0	0	0	0	0
SAL_TA2811AA	20151006A5	Braenderup	0	0	0	0	0	0	0	0	0	0	0
SAL_TA2822AA	20151006B10	Braenderup	0	0	0	0	0	0	0	0	0	0	0
SAL_TA2744AA	626645	Braenderup	0	0	0	0	0	0	0	0	0	0	0
SAL_TA2636AA	SAL-18-VL-LA-TX-0007	Braenderup	0	0	0	0	0	0	0	0	0	0	0
SAL_TA2932AA	625716	Braenderup	0	0	0	0	0	0	0	0	0	0	0
SAL_TA5352AA	627311	Braenderup	0	0	0	0	0	0	0	0	0	0	0
SAL_TA5356AA	627312	Braenderup	0	0	0	0	0	0	0	0	0	0	0
SAL_TA5318AA	621516	Braenderup	0	0	0	0	0	0	0	0	0	0	0
SAL_TA5308AA	625281	Braenderup	0	0	0	0	0	0	0	0	0	0	0
SAL_TA5251AA	615528	Braenderup	0	0	0	0	0	0	0	0	0	0	0
SAL_TA6194AA	628049	Braenderup	0	0	0	0	0	0	0	0	0	0	0
SAL_TA6179AA	612650	Braenderup	0	0	0	0	0	0	0	0	0	0	0
SAL_TA6186AA	617833	Braenderup	0	0	0	0	0	0	0	0	0	0	0
SAL_TA6193AA	623460	Braenderup	0	0	0	0	0	0	0	0	0	0	0
SAL_TA6618AA	SAL-18-VL-OH-WA-0017	Braenderup	0	0	0	0	0	0	0	0	0	0	0
SAL_TA6953AA	621459	Braenderup	0	0	0	0	0	0	0	0	0	0	0
SAL_TA6899AA	633421	Braenderup	0	0	0	0	0	0	0	0	0	0	0
SAL_TA7060AA	CFSAN087714	Braenderup	0	0	0	0	0	0	0	0	0	0	0
SAL_TA7278AA	VA-WGS-18095	Braenderup	0	0	0	0	0	0	0	0	0	0	0
SAL_TA7258AA	VA-WGS-18094	Braenderup	0	0	0	0	0	0	0	0	0	0	0
SAL_TA7269AA	VA-WGS-18090	Braenderup	0	0	0	0	0	0	0	0	0	0	0
SAL_TA7382AA	634670	Braenderup	0	0	0	0	0	0	0	0	0	0	0
SAL_TA7423AA	634752	Braenderup	0	0	0	0	0	0	0	0	0	0	0
SAL_TA7422AA	633490	Braenderup	0	0	0	0	0	0	0	0	0	0	0
SAL_TA2373AA	624481	Braenderup	0	0	0	0	0	0	0	0	0	0	0
SAL_OA2286AA	PNUSAS034021	Braenderup	0	0	0	0	0	0	0	0	0	0	0
SAL_OA5281AA	PNUSAS035311	Braenderup	0	0	0	0	0	0	0	0	0	0	0
SAL_OA5159AA	PNUSAS035885	Braenderup	0	0	0	0	0	0	0	0	0	0	0
SAL_PA3685AA	PNUSAS039217	Braenderup	0	0	0	0	0	0	0	0	0	0	0
SAL_PA3658AA	FSIS31800326	Braenderup	0	0	0	0	0	0	0	0	0	0	0
SAL_PA3718AA	PNUSAS039356	Braenderup	0	0	0	0	0	0	0	0	0	0	0
SAL_OA8304AA	HJ-34	Braenderup	0	0	0	0	0	0	0	0	0	0	0
SAL_OA5243AA	PNUSAS035484	Braenderup	0	0	0	0	0	0	0	0	0	0	0
SAL_TA8075AA	WEK-3-VA-WGS-18090	Braenderup	0	0	0	0	0	0	0	0	0	0	0
SAL_IA4627AA	PNUSAS008072	Braenderup	0	0	0	0	0	0	0	0	0	0	0
SAL_TA8081AA	PPL-2-VA-WGS-18095	Braenderup	0	0	0	0	0	0	0	0	0	0	0
SAL_TA8080AA	PPL-1-VA-WGS-18094	Braenderup	0	0	0	0	0	0	0	0	0	0	0
SAL_OA1352AA	PNUSAS031714	Braenderup	0	0	0	0	0	0	0	0	0	0	0
SAL_QA7964AA	PNUSAS043653	Braenderup	0	0	0	0	0	0	0	0	0	0	0
SAL_KA0140AA	PNUSAS013814	Braenderup	0	0	0	0	0	0	0	0	0	0	0
SAL_KA0138AA	PNUSAS013806	Braenderup	0	0	0	0	0	0	0	0	0	0	0
SAL_KA2983AA	FSIS1702314	Braenderup	0	0	0	0	0	0	0	0	0	0	0
SAL_MA1573AA	PNUSAS025628	Braenderup	0	0	0	0	0	0	0	0	0	0	0
SAL_TA7435AA	616273	Braenderup	0	0	0	0	0	0	0	0	0	0	0
SAL_TA8353AA	617846	Braenderup	0	0	0	0	0	0	0	0	0	0	0
SAL_QA6240AA	488681	Braenderup	0	0	0	0	0	0	0	0	0	0	0
SAL_OA0895AA	PNUSAS033586	Braenderup	0	0	0	0	0	0	0	0	0	0	0
SAL_OA0924AA	PNUSAS034346	Braenderup	0	0	0	0	0	0	0	0	0	0	0
SAL_QA7766AA	327920	Braenderup	0	0	0	0	0	0	0	0	0	0	0
SAL_QA8043AA	PNUSAS042648	Braenderup	0	0	0	0	0	0	0	0	0	0	0
SAL_OA0943AA	FSIS21821088	Braenderup	0	0	0	0	0	0	0	0	0	0	0
SAL_OA1430AA	FSIS11706687	Braenderup	0	0	0	0	0	0	0	0	0	0	0
SAL_PA5121AA	PNUSAS039039	Braenderup	0	0	0	0	0	0	0	0	0	0	0
SAL_QA9950AA	212753	Braenderup	0	0	0	0	0	0	0	0	0	0	0
SAL_QA8019AA	PNUSAS042519	Braenderup	0	0	0	0	0	0	0	0	0	0	0
SAL_QA8023AA	PNUSAS041784	Braenderup	0	0	0	0	0	0	0	0	0	0	0
SAL_OA0847AA	PNUSAS031838	Braenderup	0	0	0	0	0	0	0	0	0	0	0
SAL_TA8953AA	640970	Braenderup	0	0	0	0	0	0	0	0	0	0	0
SAL_QA5327AA	427417	Braenderup	0	0	0	0	0	0	0	0	0	0	0
SAL_QA5381AA	286801	Braenderup	0	0	0	0	0	0	0	0	0	0	0
SAL_OA1228AA	PNUSAS033700	Braenderup	0	0	0	0	0	0	0	0	0	0	0

SAL_OA1235AA	PNUSAS034218	Braenderup	0	0	0	0	0	0	0	0	0	0	0
SAL_OA1709AA	PNUSAS033379	Braenderup	0	0	0	0	0	0	0	0	0	0	0
SAL_OA1923AA	PNUSAS033844	Braenderup	0	0	0	0	0	0	0	0	0	0	0
SAL_OA2322AA	PNUSAS033489	Braenderup	0	0	0	0	0	0	0	0	0	0	0
SAL_OA2192AA	FDA00004196	Braenderup	0	0	0	0	0	0	0	0	0	0	0
SAL_OA2438AA	MOD1-Tw-229c	Braenderup	0	0	0	0	0	0	0	0	0	0	0
SAL_OA2005AA	PNUSAS034975	Braenderup	0	0	0	0	0	0	0	0	0	0	0
SAL_OA2014AA	PNUSAS035450	Braenderup	0	0	0	0	0	0	0	0	0	0	0
SAL_OA2040AA	PNUSAS035258	Braenderup	0	0	0	0	0	0	0	0	0	0	0
SAL_OA4392AA	PNUSAS035907	Braenderup	0	0	0	0	0	0	0	0	0	0	0
SAL_OA5167AA	PNUSAS035882	Braenderup	0	0	0	0	0	0	0	0	0	0	0
SAL_OA5194AA	PNUSAS035866	Braenderup	0	0	0	0	0	0	0	0	0	0	0
SAL_OA5228AA	PNUSAS035804	Braenderup	0	0	0	0	0	0	0	0	0	0	0
SAL_OA5254AA	PNUSAS035317	Braenderup	0	0	0	0	0	0	0	0	0	0	0
SAL_OA5271AA	PNUSAS035298	Braenderup	0	0	0	0	0	0	0	0	0	0	0
SAL_OA5339AA	PNUSAS035261	Braenderup	0	0	0	0	0	0	0	0	0	0	0
SAL_OA5352AA	PNUSAS033172	Braenderup	0	0	0	0	0	0	0	0	0	0	0
SAL_OA5604AA	PNUSAS036414	Braenderup	0	0	0	0	0	0	0	0	0	0	0
SAL_OA5545AA	PNUSAS034449	Braenderup	0	0	0	0	0	0	0	0	0	0	0
SAL_OA7324AA	PNUSAS037379	Braenderup	0	0	0	0	0	0	0	0	0	0	0
SAL_OA8308AA	HJ-35	Braenderup	0	0	0	0	0	0	0	0	0	0	0
SAL_PA1353AA	PNUSAS038336	Braenderup	0	0	0	0	0	0	0	0	0	0	0
SAL_PA1513AA	PNUSAS038153	Braenderup	0	0	0	0	0	0	0	0	0	0	0
SAL_PA1656AA	PNUSAS038696	Braenderup	0	0	0	0	0	0	0	0	0	0	0
SAL_PA1669AA	PNUSAS038607	Braenderup	0	0	0	0	0	0	0	0	0	0	0
SAL_PA1856AA	PNUSAS036259	Braenderup	0	0	0	0	0	0	0	0	0	0	0
SAL_PA1919AA	PNUSAS038479	Braenderup	0	0	0	0	0	0	0	0	0	0	0
SAL_PA1935AA	PNUSAS037897	Braenderup	0	0	0	0	0	0	0	0	0	0	0
SAL_PA2019AA	PNUSAS037950	Braenderup	0	0	0	0	0	0	0	0	0	0	0
SAL_PA2175AA	FDA00012973	Braenderup	0	0	0	0	0	0	0	0	0	0	0
SAL_PA2140AA	FDA00012959	Braenderup	0	0	0	0	0	0	0	0	0	0	0
SAL_PA2141AA	FDA00012962	Braenderup	0	0	0	0	0	0	0	0	0	0	0
SAL_PA2197AA	FDA00012974	Braenderup	0	0	0	0	0	0	0	0	0	0	0
SAL_PA2252AA	PNUSAS038634	Braenderup	0	0	0	0	0	0	0	0	0	0	0
SAL_PA2415AA	PNUSAS038432	Braenderup	0	0	0	0	0	0	0	0	0	0	0
SAL_PA2403AA	PNUSAS038690	Braenderup	0	0	0	0	0	0	0	0	0	0	0
SAL_PA2138AA	FDA00012960	Braenderup	0	0	0	0	0	0	0	0	0	0	0
SAL_PA2575AA	PNUSAS038765	Braenderup	0	0	0	0	0	0	0	0	0	0	0
SAL_PA2583AA	PNUSAS036489	Braenderup	0	0	0	0	0	0	0	0	0	0	0
SAL_PA2604AA	PNUSAS036103	Braenderup	0	0	0	0	0	0	0	0	0	0	0
SAL_PA2621AA	PNUSAS035384	Braenderup	0	0	0	0	0	0	0	0	0	0	0
SAL_PA2630AA	PNUSAS035377	Braenderup	0	0	0	0	0	0	0	0	0	0	0
SAL_PA2716AA	PNUSAS036750	Braenderup	0	0	0	0	0	0	0	0	0	0	0
SAL_PA2768AA	PNUSAS039197	Braenderup	0	0	0	0	0	0	0	0	0	0	0
SAL_PA2905AA	PNUSAS036889	Braenderup	0	0	0	0	0	0	0	0	0	0	0
SAL_PA3063AA	PNUSAS035691	Braenderup	0	0	0	0	0	0	0	0	0	0	0
SAL_PA3452AA	PNUSAS038364	Braenderup	0	0	0	0	0	0	0	0	0	0	0
SAL_PA3448AA	PNUSAS038369	Braenderup	0	0	0	0	0	0	0	0	0	0	0
SAL_PA3913AA	PNUSAS039372	Braenderup	0	0	0	0	0	0	0	0	0	0	0
SAL_PA3928AA	PNUSAS038578	Braenderup	0	0	0	0	0	0	0	0	0	0	0
SAL_PA4081AA	FSIS11808999	Braenderup	0	0	0	0	0	0	0	0	0	0	0
SAL_TA9662AA	FSIS31801258	Braenderup	0	0	0	0	0	0	0	0	0	0	0
SAL_TA9732AA	643209	Braenderup	0	0	0	0	0	0	0	0	0	0	0
SAL_TA9799AA	FSIS11816121	Braenderup	0	0	0	0	0	0	0	0	0	0	0
SAL_UA2217AA	OH-18-1300-1T	Braenderup	0	0	0	0	0	0	0	0	0	0	0
SAL_UA2200AA	FDA00013738	Braenderup	0	0	0	0	0	0	0	0	0	0	0
SAL_UA2205AA	OH-17-24605-2M	Braenderup	0	0	0	0	0	0	0	0	0	0	0
SAL_UA2210AA	OH-17-24604-1B	Braenderup	0	0	0	0	0	0	0	0	0	0	0
SAL_UA1700AA	619032	Braenderup	0	0	0	0	0	0	0	0	0	0	0
SAL_UA1636AA	FSIS31801300	Braenderup	0	0	0	0	0	0	0	0	0	0	0
SAL_UA2240AA	FDA00013737	Braenderup	0	0	0	0	0	0	0	0	0	0	0
SAL_UA2776AA	648638	Braenderup	0	0	0	0	0	0	0	0	0	0	0
SAL_UA2730AA	645994	Braenderup	0	0	0	0	0	0	0	0	0	0	0
SAL_UA2735AA	647895	Braenderup	0	0	0	0	0	0	0	0	0	0	0
SAL_UA2748AA	647419	Braenderup	0	0	0	0	0	0	0	0	0	0	0
SAL_UA2919AA	627405	Braenderup	0	0	0	0	0	0	0	0	0	0	0
SAL_UA3106AA	19_Braenderup2_cp	Braenderup	0	0	0	0	0	0	0	0	0	0	0
SAL_UA3000AA	637885	Braenderup	0	0	0	0	0	0	0	0	0	0	0
SAL_UA3014AA	605822	Braenderup	0	0	0	0	0	0	0	0	0	0	0
SAL_UA3030AA	640964	Braenderup	0	0	0	0	0	0	0	0	0	0	0
SAL_UA3102AA	24_Braenderup1_cp	Braenderup	0	0	0	0	0	0	0	0	0	0	0
SAL_UA3689AA	625776	Braenderup	0	0	0	0	0	0	0	0	0	0	0
SAL_UA3731AA	597041	Braenderup	0	0	0	0	0	0	0	0	0	0	0

SAL_UA4720AA	596218	Braenderup	0	0	0	0	0	0	0	0	0	0	0
SAL_UA4730AA	595662	Braenderup	0	0	0	0	0	0	0	0	0	0	0
SAL_UA5133AA	FSIS31801333	Braenderup	0	0	0	0	0	0	0	0	0	0	0
SAL_UA5195AA	FSIS21822956	Braenderup	0	0	0	0	0	0	0	0	0	0	0
SAL_UA5209AA	FSIS21822958	Braenderup	0	0	0	0	0	0	0	0	0	0	0
SAL_UA5241AA	593482	Braenderup	0	0	0	0	0	0	0	0	0	0	0
SAL_UA5311AA	652957	Braenderup	0	0	0	0	0	0	0	0	0	0	0
SAL_UA5457AA	591919	Braenderup	0	0	0	0	0	0	0	0	0	0	0
SAL_UA5607AA	587812	Braenderup	0	0	0	0	0	0	0	0	0	0	0
SAL_UA6000AA	584690	Braenderup	0	0	0	0	0	0	0	0	0	0	0
SAL_UA6097AA	582792	Braenderup	0	0	0	0	0	0	0	0	0	0	0
SAL_UA6161AA	579110	Braenderup	0	0	0	0	0	0	0	0	0	0	0
SAL_UA6207AA	656754	Braenderup	0	0	0	0	0	0	0	0	0	0	0
SAL_UA6216AA	580498	Braenderup	0	0	0	0	0	0	0	0	0	0	0
SAL_UA6106AA	582287	Braenderup	0	0	0	0	0	0	0	0	0	0	0
SAL_UA6491AA	FSIS21823049	Braenderup	0	0	0	0	0	0	0	0	0	0	0
SAL_UA6934AA	573629	Braenderup	0	0	0	0	0	0	0	0	0	0	0
SAL_UA7046AA	572521	Braenderup	0	0	0	0	0	0	0	0	0	0	0
SAL_UA7293AA	ATCC BAA-664	Braenderup	0	0	0	0	0	0	0	0	0	0	0
SAL_UA7791AA	662833	Braenderup	0	0	0	0	0	0	0	0	0	0	0
SAL_UA7496AA	572352	Braenderup	0	0	0	0	0	0	0	0	0	0	0
SAL_UA8147AA	668790	Braenderup	0	0	0	0	0	0	0	0	0	0	0
SAL_UA8319AA	668800	Braenderup	0	0	0	0	0	0	0	0	0	0	0
SAL_UA8467AA	201500113	Braenderup	0	0	0	0	0	0	0	0	0	0	0
SAL_UA8845AA	FSIS31901471	Braenderup	0	0	0	0	0	0	0	0	0	0	0
SAL_UA9585AA	626692	Braenderup	0	0	0	0	0	0	0	0	0	0	0
SAL_UA9563AA	600760	Braenderup	0	0	0	0	0	0	0	0	0	0	0
SAL_VA0140AA	563471	Braenderup	0	0	0	0	0	0	0	0	0	0	0
SAL_VA0074AA	620456	Braenderup	0	0	0	0	0	0	0	0	0	0	0
SAL_VA0069AA	636260	Braenderup	0	0	0	0	0	0	0	0	0	0	0
SAL_VA0025AA	569960	Braenderup	0	0	0	0	0	0	0	0	0	0	0
SAL_UA9930AA	556798	Braenderup	0	0	0	0	0	0	0	0	0	0	0
SAL_UA9902AA	568244	Braenderup	0	0	0	0	0	0	0	0	0	0	0
SAL_UA9906AA	555488	Braenderup	0	0	0	0	0	0	0	0	0	0	0
SAL_VA0732AA	621766	Braenderup	0	0	0	0	0	0	0	0	0	0	0
SAL_VA0573AA	FSIS11917362	Braenderup	0	0	0	0	0	0	0	0	0	0	0
SAL_VA0449AA	549506	Braenderup	0	0	0	0	0	0	0	0	0	0	0
SAL_VA2578AA	HJ-35-VA-WGS-18005	Braenderup	0	0	0	0	0	0	0	0	0	0	0
SAL_VA1131AA	506432	Braenderup	0	0	0	0	0	0	0	0	0	0	0
SAL_VA1176AA	548942	Braenderup	0	0	0	0	0	0	0	0	0	0	0
SAL_VA1193AA	506859	Braenderup	0	0	0	0	0	0	0	0	0	0	0
SAL_VA0978AA	513468	Braenderup	0	0	0	0	0	0	0	0	0	0	0
SAL_VA1675AA	PNUSAS051517	Braenderup	0	0	0	0	0	0	0	0	0	0	0
SAL_VA1673AA	PNUSAS051520	Braenderup	0	0	0	0	0	0	0	0	0	0	0
SAL_VA1674AA	PNUSAS051518	Braenderup	0	0	0	0	0	0	0	0	0	0	0
SAL_VA1672AA	PNUSAS051521	Braenderup	0	0	0	0	0	0	0	0	0	0	0
SAL_VA7079AA	5255	Braenderup	0	0	0	0	0	0	0	0	0	0	0
SAL_VA7253AA	5262	Braenderup	0	0	0	0	0	0	0	0	0	0	0
SAL_VA7255AA	5263	Braenderup	0	0	0	0	0	0	0	0	0	0	0
SAL_VA7257AA	5264	Braenderup	0	0	0	0	0	0	0	0	0	0	0
SAL_VA0957AA	673605	Braenderup	0	0	0	0	0	0	0	0	0	0	0
SAL_VA1263AA	493370	Braenderup	0	0	0	0	0	0	0	0	0	0	0
SAL_VA1536AA	RS-SF02560/13	Braenderup	0	0	0	0	0	0	0	0	0	0	0
SAL_VA7692AA	201703454	Braenderup	0	0	0	0	0	0	0	0	0	0	0
SAL_VA2277AA	484699	Braenderup	0	0	0	0	0	0	0	0	0	0	0
SAL_VA2133AA	458060	Braenderup	0	0	0	0	0	0	0	0	0	0	0
SAL_VA1830AA	PNUSAS047152	Braenderup	0	0	0	0	0	0	0	0	0	0	0
SAL_VA1843AA	PNUSAS028303	Braenderup	0	0	0	0	0	0	0	0	0	0	0
SAL_VA1842AA	PNUSAS028306	Braenderup	0	0	0	0	0	0	0	0	0	0	0
SAL_VA3242AA	443538	Braenderup	0	0	0	0	0	0	0	0	0	0	0
SAL_VA1822AA	PNUSAS047002	Braenderup	0	0	0	0	0	0	0	0	0	0	0
SAL_VA1836AA	PNUSAS047001	Braenderup	0	0	0	0	0	0	0	0	0	0	0
SAL_VA3133AA	417356	Braenderup	0	0	0	0	0	0	0	0	0	0	0
SAL_VA3090AA	419044	Braenderup	0	0	0	0	0	0	0	0	0	0	0
SAL_VA1811AA	PNUSAS047003	Braenderup	0	0	0	0	0	0	0	0	0	0	0
SAL_VA1818AA	PNUSAS048844	Braenderup	0	0	0	0	0	0	0	0	0	0	0
SAL_VA1805AA	PNUSAS055168	Braenderup	0	0	0	0	0	0	0	0	0	0	0
SAL_VA3457AA	399345	Braenderup	0	0	0	0	0	0	0	0	0	0	0
SAL_VA1770AA	PNUSAS048847	Braenderup	0	0	0	0	0	0	0	0	0	0	0
SAL_VA1774AA	PNUSAS047004	Braenderup	0	0	0	0	0	0	0	0	0	0	0
SAL_VA2243AA	486540	Braenderup	0	0	0	0	0	0	0	0	0	0	0
SAL_VA2150AA	449703	Braenderup	0	0	0	0	0	0	0	0	0	0	0
SAL_VA2067AA	467362	Braenderup	0	0	0	0	0	0	0	0	0	0	0

SAL_VA6728AA	FSIS21923480	Braenderup	0	0	0	0	0	0	0	0	0	0	0
SAL_VA6727AA	FSIS21923483	Braenderup	0	0	0	0	0	0	0	0	0	0	0
SAL_VA1745AA	PNUSAS052503	Braenderup	0	0	0	0	0	0	0	0	0	0	0
SAL_VA1768AA	PNUSAS048851	Braenderup	0	0	0	0	0	0	0	0	0	0	0
SAL_VA2519AA	424712	Braenderup	0	0	0	0	0	0	0	0	0	0	0
SAL_VA2566AA	422464	Braenderup	0	0	0	0	0	0	0	0	0	0	0
SAL_VA2448AA	422462	Braenderup	0	0	0	0	0	0	0	0	0	0	0
SAL_VA2430AA	429783	Braenderup	0	0	0	0	0	0	0	0	0	0	0
SAL_VA2637AA	AG19-0033	Braenderup	0	0	0	0	0	0	0	0	0	0	0
SAL_VA5896AA	SA20080071	Braenderup	0	0	0	0	0	0	0	0	0	0	0
SAL_VA5694AA	SA20080149	Braenderup	0	0	0	0	0	0	0	0	0	0	0
SAL_VA4394AA	388783	Braenderup	0	0	0	0	0	0	0	0	0	0	0
SAL_VA6928AA	378656	Braenderup	0	0	0	0	0	0	0	0	0	0	0
SAL_VA6878AA	378587	Braenderup	0	0	0	0	0	0	0	0	0	0	0
SAL_VA7116AA	389627	Braenderup	0	0	0	0	0	0	0	0	0	0	0
SAL_VA7137AA	381701	Braenderup	0	0	0	0	0	0	0	0	0	0	0
SAL_VA7188AA	370593	Braenderup	0	0	0	0	0	0	0	0	0	0	0
SAL_VA7763AA	FSIS11917609	Braenderup	0	0	0	0	0	0	0	0	0	0	0
SAL_VA7838AA	FSIS11917774	Braenderup	0	0	0	0	0	0	0	0	0	0	0
SAL_VA7923AA	VA-WGS-19005	Braenderup	0	0	0	0	0	0	0	0	0	0	0
SAL_VA7925AA	VA-WGS-19004	Braenderup	0	0	0	0	0	0	0	0	0	0	0
SAL_VA7921AA	VA-WGS-19007	Braenderup	0	0	0	0	0	0	0	0	0	0	0
SAL_VA7926AA	VA-WGS-19003	Braenderup	0	0	0	0	0	0	0	0	0	0	0
SAL_VA7928AA	VA-WGS-19006	Braenderup	0	0	0	0	0	0	0	0	0	0	0
SAL_VA7920AA	VA-WGS-19001	Braenderup	0	0	0	0	0	0	0	0	0	0	0
SAL_VA7919AA	VA-WGS-19002	Braenderup	0	0	0	0	0	0	0	0	0	0	0
SAL_VA8004AA	FSIS11917573	Braenderup	0	0	0	0	0	0	0	0	0	0	0
SAL_VA8086AA	FDA00013866	Braenderup	0	0	0	0	0	0	0	0	0	0	0
SAL_VA8278AA	363599	Braenderup	0	0	0	0	0	0	0	0	0	0	0
SAL_VA8228AA	378705	Braenderup	0	0	0	0	0	0	0	0	0	0	0
SAL_VA8581AA	690326	Braenderup	0	0	0	0	0	0	0	0	0	0	0
SAL_VA8391AA	690302	Braenderup	0	0	0	0	0	0	0	0	0	0	0
SAL_VA8455AA	690330	Braenderup	0	0	0	0	0	0	0	0	0	0	0
SAL_VA8590AA	FSIS11918015	Braenderup	0	0	0	0	0	0	0	0	0	0	0
SAL_VA8793AA	319664	Braenderup	0	0	0	0	0	0	0	0	0	0	0
SAL_VA8728AA	690381	Braenderup	0	0	0	0	0	0	0	0	0	0	0
SAL_VA8685AA	691179	Braenderup	0	0	0	0	0	0	0	0	0	0	0
SAL_VA9214AA	307216	Braenderup	0	0	0	0	0	0	0	0	0	0	0
SAL_VA9221AA	306678	Braenderup	0	0	0	0	0	0	0	0	0	0	0
SAL_VA9185AA	320211	Braenderup	0	0	0	0	0	0	0	0	0	0	0
SAL_VA9111AA	314953	Braenderup	0	0	0	0	0	0	0	0	0	0	0
SAL_VA9046AA	294990	Braenderup	0	0	0	0	0	0	0	0	0	0	0
SAL_VA9005AA	305206	Braenderup	0	0	0	0	0	0	0	0	0	0	0
SAL_VA9009AA	296855	Braenderup	0	0	0	0	0	0	0	0	0	0	0
SAL_VA9024AA	300676	Braenderup	0	0	0	0	0	0	0	0	0	0	0
SAL_VA9037AA	299881	Braenderup	0	0	0	0	0	0	0	0	0	0	0
SAL_VA8939AA	306525	Braenderup	0	0	0	0	0	0	0	0	0	0	0
SAL_VA8943AA	293143	Braenderup	0	0	0	0	0	0	0	0	0	0	0
SAL_VA8944AA	296178	Braenderup	0	0	0	0	0	0	0	0	0	0	0
SAL_VA8937AA	306474	Braenderup	0	0	0	0	0	0	0	0	0	0	0
SAL_VA9416AA	280667	Braenderup	0	0	0	0	0	0	0	0	0	0	0
SAL_VA9423AA	280312	Braenderup	0	0	0	0	0	0	0	0	0	0	0
SAL_VA9433AA	293049	Braenderup	0	0	0	0	0	0	0	0	0	0	0
SAL_VA9298AA	310288	Braenderup	0	0	0	0	0	0	0	0	0	0	0
SAL_VA9326AA	279932	Braenderup	0	0	0	0	0	0	0	0	0	0	0
SAL_VA9284AA	291846	Braenderup	0	0	0	0	0	0	0	0	0	0	0
SAL_VA4179AA	CDPH_CSalm141	Braenderup	0	0	0	0	0	0	0	0	0	0	0
SAL_VA9600AA	279943	Braenderup	0	0	0	0	0	0	0	0	0	0	0
SAL_VA9794AA	262680	Braenderup	0	0	0	0	0	0	0	0	0	0	0
SAL_VA9817AA	252422	Braenderup	0	0	0	0	0	0	0	0	0	0	0
SAL_VA9736AA	267769	Braenderup	0	0	0	0	0	0	0	0	0	0	0
SAL_VA9698AA	261938	Braenderup	0	0	0	0	0	0	0	0	0	0	0
SAL_VA9705AA	261439	Braenderup	0	0	0	0	0	0	0	0	0	0	0
SAL_VA9712AA	264111	Braenderup	0	0	0	0	0	0	0	0	0	0	0
SAL_VA9833AA	FSIS21923651	Braenderup	0	0	0	0	0	0	0	0	0	0	0
SAL_VA9982AA	223493	Braenderup	0	0	0	0	0	0	0	0	0	0	0
SAL_VA9991AA	223502	Braenderup	0	0	0	0	0	0	0	0	0	0	0
SAL_WA0003AA	241830	Braenderup	0	0	0	0	0	0	0	0	0	0	0
SAL_VA9963AA	223497	Braenderup	0	0	0	0	0	0	0	0	0	0	0
SAL_VA9961AA	253456	Braenderup	0	0	0	0	0	0	0	0	0	0	0
SAL_VA9873AA	259256	Braenderup	0	0	0	0	0	0	0	0	0	0	0
SAL_VA9874AA	224028	Braenderup	0	0	0	0	0	0	0	0	0	0	0
SAL_WA0274AA	215105	Braenderup	0	0	0	0	0	0	0	0	0	0	0

SAL_WA0234AA	197098	Braenderup	0	0	0	0	0	0	0	0	0	0	0
SAL_WA0247AA	212798	Braenderup	0	0	0	0	0	0	0	0	0	0	0
SAL_WA0254AA	211176	Braenderup	0	0	0	0	0	0	0	0	0	0	0
SAL_WA0258AA	212739	Braenderup	0	0	0	0	0	0	0	0	0	0	0
SAL_WA0259AA	215108	Braenderup	0	0	0	0	0	0	0	0	0	0	0
SAL_WA0270AA	199915	Braenderup	0	0	0	0	0	0	0	0	0	0	0
SAL_WA0124AA	212769	Braenderup	0	0	0	0	0	0	0	0	0	0	0
SAL_WA0545AA	211141	Braenderup	0	0	0	0	0	0	0	0	0	0	0
SAL_WA0493AA	221645	Braenderup	0	0	0	0	0	0	0	0	0	0	0
SAL_WA0528AA	240167	Braenderup	0	0	0	0	0	0	0	0	0	0	0
SAL_WA0455AA	190570	Braenderup	0	0	0	0	0	0	0	0	0	0	0
SAL_WA0636AA	FSIS11918326	Braenderup	0	0	0	0	0	0	0	0	0	0	0
SAL_WA0372AA	179797	Braenderup	0	0	0	0	0	0	0	0	0	0	0
SAL_WA0366AA	178771	Braenderup	0	0	0	0	0	0	0	0	0	0	0
SAL_WA0300AA	178661	Braenderup	0	0	0	0	0	0	0	0	0	0	0
SAL_WA0995AA	160832	Braenderup	0	0	0	0	0	0	0	0	0	0	0
SAL_WA0919AA	166295	Braenderup	0	0	0	0	0	0	0	0	0	0	0
SAL_WA0765AA	174770	Braenderup	0	0	0	0	0	0	0	0	0	0	0
SAL_WA0935AA	146197	Braenderup	0	0	0	0	0	0	0	0	0	0	0
SAL_WA0805AA	160831	Braenderup	0	0	0	0	0	0	0	0	0	0	0
SAL_WA3171AA	RM_303	Braenderup	0	0	0	0	0	0	0	0	0	0	0
SAL_WA2755AA	RM_428	Braenderup	0	0	0	0	0	0	0	0	0	0	0
SAL_WA2707AA	RM_47	Braenderup	0	0	0	0	0	0	0	0	0	0	0
SAL_WA2571AA	RM_151	Braenderup	0	0	0	0	0	0	0	0	0	0	0
SAL_WA2398AA	RM_272	Braenderup	0	0	0	0	0	0	0	0	0	0	0
SAL_WA1243AA	130397	Braenderup	0	0	0	0	0	0	0	0	0	0	0
SAL_WA1246AA	130416	Braenderup	0	0	0	0	0	0	0	0	0	0	0
SAL_WA2213AA	CK_97/00013527	Braenderup	0	0	0	0	0	0	0	0	0	0	0
SAL_WA1407AA	107210	Braenderup	0	0	0	0	0	0	0	0	0	0	0
SAL_WA1591AA	129485	Braenderup	0	0	0	0	0	0	0	0	0	0	0
SAL_WA1531AA	115268	Braenderup	0	0	0	0	0	0	0	0	0	0	0
SAL_WA1507AA	122203	Braenderup	0	0	0	0	0	0	0	0	0	0	0
SAL_WA1394AA	SAL-18-VL-SD-NC-0020	Braenderup	0	0	0	0	0	0	0	0	0	0	0
SAL_WA1687AA	126202	Braenderup	0	0	0	0	0	0	0	0	0	0	0
SAL_WA1690AA	103661	Braenderup	0	0	0	0	0	0	0	0	0	0	0
SAL_WA1970AA	705225	Braenderup	0	0	0	0	0	0	0	0	0	0	0
SAL_WA2053AA	704768	Braenderup	0	0	0	0	0	0	0	0	0	0	0
SAL_WA2143AA	FSIS11919165	Braenderup	0	0	0	0	0	0	0	0	0	0	0
SAL_VA8070AA	NYVetLIRN-120	Braenderup	0	0	0	0	0	0	0	0	0	0	0
SAL_WA3640AA	CFSAN087557	Braenderup	0	0	0	0	0	0	0	0	0	0	0
SAL_WA3730AA	CFSAN087597	Braenderup	0	0	0	0	0	0	0	0	0	0	0
SAL_WA3963AA	VA-WGS-19020	Braenderup	0	0	0	0	0	0	0	0	0	0	0
SAL_WA4010AA	FSIS11919205	Braenderup	0	0	0	0	0	0	0	0	0	0	0
SAL_GA1856AA	BCW_5094	Brancaster	0	0	0	0	0	0	0	0	0	0	0
SAL_HA3291AA	m22	Brancaster	0	0	0	0	0	0	0	0	0	0	0
SAL_HA3292AA	m24	Brancaster	0	0	0	0	0	0	0	0	0	0	0
SAL_HA3293AA	m29	Brancaster	0	0	0	0	0	0	0	0	0	0	0
SAL_HA3298AA	t24	Brancaster	0	0	0	0	0	0	0	0	0	0	0
SAL_HA3696AA	ww22	Brancaster	0	0	0	0	0	0	0	0	0	0	0
SAL_HA3419AA	ww82	Brancaster	0	0	0	0	0	0	0	0	0	0	0
SAL_HA3416AA	ww281	Brancaster	0	0	0	0	0	0	0	0	0	0	0
SAL_HA3418AA	ww81	Brancaster	0	0	0	0	0	0	0	0	0	0	0
SAL_JA7914AA	348754	Brancaster	0	0	0	0	0	0	0	0	0	0	0
SAL_CA2420AA	25201	Brancaster	0	0	0	0	0	0	0	0	0	0	0
SAL_GA0686AA	pn1918_18-sc-2235577	Brancaster	0	0	0	0	0	0	0	0	0	0	0
SAL_DA4157AA	FCC0170	Brancaster	0	0	0	0	0	0	0	0	0	0	0
SAL_OA9704AA	sg_t24	Brancaster	0	0	0	0	0	0	0	0	0	0	0
SAL_OA9725AA	sg_m29	Brancaster	0	0	0	0	0	0	0	0	0	0	0
SAL_OA9718AA	sg_m22	Brancaster	0	0	0	0	0	0	0	0	0	0	0
SAL_OA9724AA	sg_m24	Brancaster	0	0	0	0	0	0	0	0	0	0	0
SAL_PA0335AA	CFSAN064137	Brancaster	0	0	0	0	0	0	0	0	0	0	0
SAL_PA0338AA	CFSAN064140	Brancaster	0	0	0	0	0	0	0	0	0	0	0
SAL_PA0333AA	CFSAN064139	Brancaster	0	0	0	0	0	0	0	0	0	0	0
SAL_PA0526AA	CFSAN064141	Brancaster	0	0	0	0	0	0	0	0	0	0	0
SAL_PA0533AA	CFSAN064169	Brancaster	0	0	0	0	0	0	0	0	0	0	0
SAL_PA0527AA	CFSAN064142	Brancaster	0	0	0	0	0	0	0	0	0	0	0
SAL_PA8658AA	314942	Brancaster	0	0	0	0	0	0	0	0	0	0	0
SAL_PA9766AA	PNUSAS041541	Brancaster	0	0	0	0	0	0	0	0	0	0	0
SAL_QA0116AA	PNUSAS041769	Brancaster	0	0	0	0	0	0	0	0	0	0	0
SAL_QA0406AA	158955	Brancaster	0	0	0	0	0	0	0	0	0	0	0
SAL_QA2053AA	453973	Brancaster	0	0	0	0	0	0	0	0	0	0	0
SAL_TA7735AA	R17.3098	Brancaster	0	0	0	0	0	0	0	0	0	0	0
SAL_TA7751AA	R17.3139	Brancaster	0	0	0	0	0	0	0	0	0	0	0

SAL_TA7754AA	R17.3183	Brancaster	0	0	0	0	0	0	0	0	0	0	0
SAL_TA7748AA	R17.3111	Brancaster	0	0	0	0	0	0	0	0	0	0	0
SAL_TA7715AA	R16.4421	Brancaster	0	0	0	0	0	0	0	0	0	0	0
SAL_TA7675AA	R17.0770	Brancaster	0	0	0	0	0	0	0	0	0	0	0
SAL_TA7678AA	R17.1480	Brancaster	0	0	0	0	0	0	0	0	0	0	0
SAL_TA7677AA	R17.1437	Brancaster	0	0	0	0	0	0	0	0	0	0	0
SAL_TA7679AA	R17.1311	Brancaster	0	0	0	0	0	0	0	0	0	0	0
SAL_TA7685AA	R17.3299	Brancaster	0	0	0	0	0	0	0	0	0	0	0
SAL_TA7688AA	R17.3109	Brancaster	0	0	0	0	0	0	0	0	0	0	0
SAL_TA7684AA	R17.3193	Brancaster	0	0	0	0	0	0	0	0	0	0	0
SAL_TA7645AA	R17.4770	Brancaster	0	0	0	0	0	0	0	0	0	0	0
SAL_TA7642AA	R17.0268	Brancaster	0	0	0	0	0	0	0	0	0	0	0
SAL_TA7624AA	R16.1761	Brancaster	0	0	0	0	0	0	0	0	0	0	0
SAL_TA7623AA	R16.1591	Brancaster	0	0	0	0	0	0	0	0	0	0	0
SAL_TA7628AA	R16.2584	Brancaster	0	0	0	0	0	0	0	0	0	0	0
SAL_TA7543AA	R17.5197	Brancaster	0	0	0	0	0	0	0	0	0	0	0
SAL_PA0336AA	CFSAN064138	Brancaster	0	0	0	0	0	0	0	0	0	0	0
SAL_VA9668AA	250705	Brancaster	0	0	0	0	0	0	0	0	0	0	0
SAL_WA0456AA	187607	Brancaster	0	0	0	0	0	0	0	0	0	0	0
SAL_WA0891AA	173545	Brancaster	0	0	0	0	0	0	0	0	0	0	0
SAL_DA4170AA	CFSAN022571	Brandenburg	0	0	0	0	0	0	0	0	0	0	0
SAL_DA0374AA	H130161242	Brandenburg	21	14	36	56	16	0	0	0	0	0	0
SAL_EA0437AA	AZ_TG68495	Brandenburg	0	0	0	0	0	0	0	0	0	0	0
SAL_CA3291AA	46002	Brandenburg	0	0	0	0	0	0	0	0	0	0	0
SAL_FA5542AA	PNUSAS001950	Brandenburg	0	0	0	0	0	0	0	0	0	0	0
SAL_FA4759AA	FSIS1606076	Brandenburg	0	0	0	0	0	0	0	0	0	0	0
SAL_FA4161AA	116563	Brandenburg	0	0	0	0	0	0	0	0	0	0	0
SAL_FA2951AA	CVM N57060F	Brandenburg	0	0	0	0	0	0	0	0	0	0	0
SAL_FA2918AA	CVM N57181F	Brandenburg	0	0	0	0	0	0	0	0	0	0	0
SAL_FA2795AA	CVM N57596F	Brandenburg	0	0	0	0	0	0	0	0	0	0	0
SAL_FA2732AA	CVM N57774F	Brandenburg	0	0	0	0	0	0	0	0	0	0	0
SAL_FA2672AA	CVM N57957F	Brandenburg	0	0	0	0	0	0	0	0	0	0	0
SAL_FA2602AA	IEH-NGS-SAL-03884	Brandenburg	0	0	0	0	0	0	0	0	0	0	0
SAL_FA2531AA	IEH-NGS-SAL-03770	Brandenburg	0	0	0	0	0	0	0	0	0	0	0
SAL_FA2530AA	IEH-NGS-SAL-03771	Brandenburg	0	0	0	0	0	0	0	0	0	0	0
SAL_FA2483AA	IEH-NGS-SAL-03793	Brandenburg	0	0	0	0	0	0	0	0	0	0	0
SAL_FA2486AA	IEH-NGS-SAL-03790	Brandenburg	0	0	0	0	0	0	0	0	0	0	0
SAL_FA2482AA	IEH-NGS-SAL-03794	Brandenburg	0	0	0	0	0	0	0	0	0	0	0
SAL_FA2484AA	IEH-NGS-SAL-03792	Brandenburg	0	0	0	0	0	0	0	0	0	0	0
SAL_FA2357AA	IEH-NGS-SAL-03830	Brandenburg	0	0	0	0	0	0	0	0	0	0	0
SAL_FA2162AA	FSIS1605834	Brandenburg	0	0	0	0	0	0	0	0	0	0	0
SAL_FA1262AA	CVM-N18572	Brandenburg	0	0	0	0	0	0	0	0	0	0	0
SAL_FA1236AA	FDA00003269	Brandenburg	0	0	0	0	0	0	0	0	0	0	0
SAL_FA1235AA	FDA00003270	Brandenburg	0	0	0	0	0	0	0	0	0	0	0
SAL_FA1219AA	IEH-NGS-SAL-03532	Brandenburg	0	0	0	0	0	0	0	0	0	0	0
SAL_FA0901AA	FSIS1605574	Brandenburg	0	0	0	0	0	0	0	0	0	0	0
SAL_FA0490AA	FSIS1605455	Brandenburg	0	0	0	0	0	0	0	0	0	0	0
SAL_EA9570AA	FSIS1605653	Brandenburg	0	0	0	0	0	0	0	0	0	0	0
SAL_EA8174AA	CVM-N23753	Brandenburg	0	0	0	0	0	0	0	0	0	0	0
SAL_EA6330AA	2015K-1386	Brandenburg	0	0	0	0	0	0	0	0	0	0	0
SAL_EA6329AA	2015K-1387	Brandenburg	0	0	0	0	0	0	0	0	0	0	0
SAL_EA6304AA	2015K-1342	Brandenburg	0	0	0	0	0	0	0	0	0	0	0
SAL_EA6301AA	2015K-1398	Brandenburg	0	0	0	0	0	0	0	0	0	0	0
SAL_EA5651AA	2015K-1177	Brandenburg	0	0	0	0	0	0	0	0	0	0	0
SAL_EA5649AA	2015K-1181	Brandenburg	0	0	0	0	0	0	0	0	0	0	0
SAL_EA5650AA	2015K-1178	Brandenburg	0	0	0	0	0	0	0	0	0	0	0
SAL_EA5619AA	2015K-1255	Brandenburg	0	0	0	0	0	0	0	0	0	0	0
SAL_EA5199AA	VA-WGS-00522	Brandenburg	0	0	0	0	0	0	0	0	0	0	0
SAL_EA5101AA	2015K-1254	Brandenburg	0	0	0	0	0	0	0	0	0	0	0
SAL_EA3771AA	CVM N45949	Brandenburg	0	0	0	0	0	0	0	0	0	0	0
SAL_EA3705AA	CVM N47716	Brandenburg	0	0	0	0	0	0	0	0	0	0	0
SAL_EA3607AA	CVM N51253	Brandenburg	0	0	0	0	0	0	0	0	0	0	0
SAL_EA2729AA	FDA00001983	Brandenburg	0	0	0	0	0	0	0	0	0	0	0
SAL_EA2691AA	NY-N14745	Brandenburg	0	0	0	0	0	0	0	0	0	0	0
SAL_EA2468AA	FDA00000508	Brandenburg	0	0	0	0	0	0	0	0	0	0	0
SAL_EA2025AA	CVM N53079	Brandenburg	0	0	0	0	0	0	0	0	0	0	0
SAL_EA1954AA	CVM N54709	Brandenburg	0	0	0	0	0	0	0	0	0	0	0
SAL_EA1894AA	CVM N55385	Brandenburg	0	0	0	0	0	0	0	0	0	0	0
SAL_EA1305AA	AZ_TG68344	Brandenburg	0	0	0	0	0	0	0	0	0	0	0
SAL_EA0842AA	M451 / NCTC20138	Brandenburg	0	0	0	0	0	0	0	0	0	0	0
SAL_DA9364AA	WAPHL-SAL-A00227	Brandenburg	0	0	0	0	0	0	0	0	0	0	0
SAL_DA8468AA	WAPHL-SAL-A00322	Brandenburg	0	0	0	0	0	0	0	0	0	0	0
SAL_DA8254AA	l676-sc-2010-09-29-9858	Brandenburg	0	0	0	0	0	0	0	0	0	0	0

SAL_DA8089AA	WAPHL-SAL-A00356	Brandenburg	0	0	0	0	0	0	0	0	0	0	0
SAL_DA7411AA	WAPHL-SAL-A00388	Brandenburg	0	0	0	0	0	0	0	0	0	0	0
SAL_DA7302AA	MDH-2014-00060	Brandenburg	0	0	0	0	0	0	0	0	0	0	0
SAL_DA7122AA	MDH-2014-00017	Brandenburg	0	0	0	0	0	0	0	0	0	0	0
SAL_DA7111AA	WAPHL-SAL-A00389	Brandenburg	0	0	0	0	0	0	0	0	0	0	0
SAL_DA7069AA	MDH-2014-00034	Brandenburg	0	0	0	0	0	0	0	0	0	0	0
SAL_DA7064AA	MDH-2014-00010	Brandenburg	0	0	0	0	0	0	0	0	0	0	0
SAL_DA7068AA	MDH-2014-00038	Brandenburg	0	0	0	0	0	0	0	0	0	0	0
SAL_DA6969AA	MDH-2014-00011	Brandenburg	0	0	0	0	0	0	0	0	0	0	0
SAL_DA6835AA	CFSAN012694	Brandenburg	0	0	0	0	0	0	0	0	0	0	0
SAL_DA3921AA	CFSAN023197	Brandenburg	0	0	0	0	0	0	0	0	0	0	0
SAL_DA3884AA	MDH-2014-00475	Brandenburg	0	0	0	0	0	0	0	0	0	0	0
SAL_DA2717AA	MDH-2014-00524	Brandenburg	0	0	0	0	0	0	0	0	0	0	0
SAL_DA2694AA	MDH-2014-00526	Brandenburg	0	0	0	0	0	0	0	0	0	0	0
SAL_DA2559AA	MDH-2014-00545	Brandenburg	0	0	0	0	0	0	0	0	0	0	0
SAL_DA2446AA	MDH-2014-00551	Brandenburg	0	0	0	0	0	0	0	0	0	0	0
SAL_DA2382AA	AZ-TG74812	Brandenburg	0	0	0	0	0	0	0	0	0	0	0
SAL_DA2271AA	06-0753	Brandenburg	0	0	0	0	0	0	0	0	0	0	0
SAL_DA1973AA	WAPHL_SAL-A00603	Brandenburg	0	0	0	0	0	0	0	0	0	0	0
SAL_DA1972AA	WAPHL_SAL-A00604	Brandenburg	0	0	0	0	0	0	0	0	0	0	0
SAL_DA0036AA	2013K-1661	Brandenburg	0	0	0	0	0	0	0	0	0	0	0
SAL_CA8831AA	AZ-TG74700	Brandenburg	0	0	0	0	0	0	0	0	0	0	0
SAL_CA8771AA	AZ-TG74728	Brandenburg	0	0	0	0	0	0	0	0	0	0	0
SAL_CA8296AA	CFSAN028004	Brandenburg	0	0	0	0	0	0	0	0	0	0	0
SAL_CA7547AA	CFSAN027396	Brandenburg	0	0	0	0	0	0	0	0	0	0	0
SAL_CA6567AA	49699	Brandenburg	0	0	0	0	0	0	0	0	0	0	0
SAL_CA2977AA	86869	Brandenburg	0	0	0	0	0	0	0	0	0	0	0
SAL_CA2939AA	37787	Brandenburg	0	0	0	0	0	0	0	0	0	0	0
SAL_CA2924AA	3900	Brandenburg	0	0	0	0	0	0	0	0	0	0	0
SAL_CA2620AA	21775	Brandenburg	0	0	0	0	0	0	0	0	0	0	0
SAL_CA2514AA	65456	Brandenburg	0	0	0	0	0	0	0	0	0	0	0
SAL_BA9685AA	37030	Brandenburg	0	0	0	0	0	0	0	0	0	0	0
SAL_BA9464AA	14918	Brandenburg	0	0	0	0	0	0	0	0	0	0	0
SAL_BA9367AA	27049	Brandenburg	0	0	0	0	0	0	0	0	0	0	0
SAL_BA5782AA	M76 / NCTC20043	Brandenburg	0	0	0	0	0	0	0	0	0	0	0
SAL_BA5771AA	M77 / NCTC20043	Brandenburg	0	0	0	0	0	0	0	0	0	0	0
SAL_BA5507AA	FL-NRM166	Brandenburg	0	0	0	0	0	0	0	0	0	0	0
SAL_BA4290AA	FDA00004606	Brandenburg	0	0	0	0	0	0	0	0	0	0	0
SAL_BA3208AA	WAPHL_SAL-A00789	Brandenburg	0	0	0	0	0	0	0	0	0	0	0
SAL_BA3197AA	WAPHL_SAL-A00788	Brandenburg	0	0	0	0	0	0	0	0	0	0	0
SAL_BA2409AA	WAPHL_SAL-A00825	Brandenburg	0	0	0	0	0	0	0	0	0	0	0
SAL_BA2378AA	WAPHL_SAL-A00824	Brandenburg	0	0	0	0	0	0	0	0	0	0	0
SAL_FA6676AA	CVM-N27392	Brandenburg	0	0	0	0	0	0	0	0	0	0	0
SAL_FA6792AA	FSIS1503001	Brandenburg	0	0	0	0	0	0	0	0	0	0	0
SAL_FA6955AA	FSIS1606635	Brandenburg	0	0	0	0	0	0	0	0	0	0	0
SAL_GA2848AA	CRJJGF_00063	Brandenburg	0	0	0	0	0	0	0	0	0	0	0
SAL_GA2847AA	CRJJGF_00064	Brandenburg	0	0	0	0	0	0	0	0	0	0	0
SAL_GA3879AA	BCW_1516	Brandenburg	0	0	0	0	0	0	0	0	0	0	0
SAL_GA3682AA	BCW_2203	Brandenburg	0	0	0	0	0	0	0	0	0	0	0
SAL_GA3349AA	BCW_2652	Brandenburg	0	0	0	0	0	0	0	0	0	0	0
SAL_GA1982AA	BCW_4837	Brandenburg	0	0	0	0	0	0	0	0	0	0	0
SAL_GA1981AA	BCW_4838	Brandenburg	0	0	0	0	0	0	0	0	0	0	0
SAL_GA1978AA	BCW_4842	Brandenburg	0	0	0	0	0	0	0	0	0	0	0
SAL_GA1958AA	BCW_4862	Brandenburg	0	0	0	0	0	0	0	0	0	0	0
SAL_GA1955AA	BCW_4865	Brandenburg	0	0	0	0	0	0	0	0	0	0	0
SAL_GA1954AA	BCW_4866	Brandenburg	0	0	0	0	0	0	0	0	0	0	0
SAL_GA1956AA	BCW_4864	Brandenburg	0	0	0	0	0	0	0	0	0	0	0
SAL_GA1953AA	BCW_4867	Brandenburg	0	0	0	0	0	0	0	0	0	0	0
SAL_GA1874AA	BCW_5053	Brandenburg	0	0	0	0	0	0	0	0	0	0	0
SAL_GA1852AA	BCW_5098	Brandenburg	0	0	0	0	0	0	0	0	0	0	0
SAL_GA1847AA	BCW_5103	Brandenburg	0	0	0	0	0	0	0	0	0	0	0
SAL_GA3489AA	BCW_2883	Brandenburg	0	0	0	0	0	0	0	0	0	0	0
SAL_GA4886AA	25	Brandenburg	0	0	0	0	0	0	0	0	0	0	0
SAL_GA4887AA	24	Brandenburg	0	0	0	0	0	0	0	0	0	0	0
SAL_GA4888AA	23	Brandenburg	0	0	0	0	0	0	0	0	0	0	0
SAL_GA4891AA	26	Brandenburg	0	0	0	0	0	0	0	0	0	0	0
SAL_FA9601AA	VA-WGS-00476	Brandenburg	0	0	0	0	0	0	0	0	0	0	0
SAL_GA4780AA	PNUSAS002800	Brandenburg	0	0	0	0	0	0	0	0	0	0	0
SAL_GA4751AA	PNUSAS002323	Brandenburg	0	0	0	0	0	0	0	0	0	0	0
SAL_FA9430AA	CVM-N26652	Brandenburg	0	0	0	0	0	0	0	0	0	0	0
SAL_GA5082AA	PNUSAS002650	Brandenburg	0	0	0	0	0	0	0	0	0	0	0
SAL_FA9311AA	AZ-TG74360	Brandenburg	0	0	0	0	0	0	0	0	0	0	0
SAL_FA9271AA	CFSAN047005	Brandenburg	0	0	0	0	0	0	0	0	0	0	0

SAL_FA9269AA	CFSAN047007	Brandenburg	0	0	0	0	0	0	0	0	0	0	0
SAL_FA9087AA	CVM N40367	Brandenburg	0	0	0	0	0	0	0	0	0	0	0
SAL_FA9088AA	CVM N40366	Brandenburg	0	0	0	0	0	0	0	0	0	0	0
SAL_GA5518AA	PNUSAS002864	Brandenburg	0	0	0	0	0	0	0	0	0	0	0
SAL_FA8657AA	CVM N31596	Brandenburg	0	0	0	0	0	0	0	0	0	0	0
SAL_FA8424AA	PNUSAS002424	Brandenburg	0	0	0	0	0	0	0	0	0	0	0
SAL_GA7581AA	PNUSAS003033	Brandenburg	0	0	0	0	0	0	0	0	0	0	0
SAL_GA7799AA	2014AM-1651	Brandenburg	0	0	0	0	0	0	0	0	0	0	0
SAL_GA7801AA	2014AM-0994	Brandenburg	0	0	0	0	0	0	0	0	0	0	0
SAL_GA8734AA	FSIS1607501	Brandenburg	0	0	0	0	0	0	0	0	0	0	0
SAL_GA9399AA	PNUSAS003709	Brandenburg	0	0	0	0	0	0	0	0	0	0	0
SAL_HA0160AA	PNUSAS004114	Brandenburg	0	0	0	0	0	0	0	0	0	0	0
SAL_HA1063AA	ARIM-SBRA1024-13	Brandenburg	0	0	0	0	0	0	0	0	0	0	0
SAL_HA1477AA	Luisa Moreno	Brandenburg	0	0	0	0	0	0	0	0	0	0	0
SAL_GA9638AA	CFSAN001008	Brandenburg	0	0	0	0	0	0	0	0	0	0	0
SAL_HA2998AA	PNUSAS004618	Brandenburg	0	0	0	0	0	0	0	0	0	0	0
SAL_HA4459AA	PNUSAS005159	Brandenburg	0	0	0	0	0	0	0	0	0	0	0
SAL_HA5115AA	L01033-14	Brandenburg	0	0	0	0	0	0	0	0	0	0	0
SAL_HA4737AA	FSIS1608556	Brandenburg	0	0	0	0	0	0	0	0	0	0	0
SAL_HA6147AA	PNUSAS005917	Brandenburg	0	0	0	0	0	0	0	0	0	0	0
SAL_HA8810AA	FSIS1609541	Brandenburg	0	0	0	0	0	0	0	0	0	0	0
SAL_HA9507AA	PNUSAS006745	Brandenburg	0	0	0	0	0	0	0	0	0	0	0
SAL_HA9474AA	PNUSAS006754	Brandenburg	0	0	0	0	0	0	0	0	0	0	0
SAL_HA9487AA	PNUSAS006750	Brandenburg	0	0	0	0	0	0	0	0	0	0	0
SAL_HA9657AA	ADRDL-794	Brandenburg	0	0	0	0	0	0	0	0	0	0	0
SAL_HA9699AA	PNUSAS006900	Brandenburg	0	0	0	0	0	0	0	0	0	0	0
SAL_IA0144AA	ADRDL-813	Brandenburg	0	0	0	0	0	0	0	0	0	0	0
SAL_IA0364AA	PNUSAS006627	Brandenburg	0	0	0	0	0	0	0	0	0	0	0
SAL_IA1768AA	287554	Brandenburg	0	0	0	0	0	0	0	0	0	0	0
SAL_IA1950AA	PNUSAS007391	Brandenburg	0	0	0	0	0	0	0	0	0	0	0
SAL_IA2305AA	CFSAN059163	Brandenburg	0	0	0	0	0	0	0	0	0	0	0
SAL_IA2293AA	CFSAN059160	Brandenburg	0	0	0	0	0	0	0	0	0	0	0
SAL_IA2310AA	CFSAN059164	Brandenburg	0	0	0	0	0	0	0	0	0	0	0
SAL_IA2308AA	CFSAN059165	Brandenburg	0	0	0	0	0	0	0	0	0	0	0
SAL_IA2658AA	CFSAN059227	Brandenburg	0	0	0	0	0	0	0	0	0	0	0
SAL_IA2665AA	CFSAN059223	Brandenburg	0	0	0	0	0	0	0	0	0	0	0
SAL_IA4651AA	FSIS1710078	Brandenburg	0	0	0	0	0	0	0	0	0	0	0
SAL_IA6148AA	CFSAN059147	Brandenburg	0	0	0	0	0	0	0	0	0	0	0
SAL_IA5547AA	FSIS1710192	Brandenburg	0	0	0	0	0	0	0	0	0	0	0
SAL_IA5448AA	PNUSAS007939	Brandenburg	0	0	0	0	0	0	0	0	0	0	0
SAL_IA5473AA	FSIS1710385	Brandenburg	0	0	0	0	0	0	0	0	0	0	0
SAL_IA5403AA	PNUSAS008512	Brandenburg	0	0	0	0	0	0	0	0	0	0	0
SAL_IA6383AA	FSIS1710393	Brandenburg	0	0	0	0	0	0	0	0	0	0	0
SAL_IA6587AA	FSIS1710370	Brandenburg	0	0	0	0	0	0	0	0	0	0	0
SAL_IA7595AA	PNUSAS009124	Brandenburg	0	0	0	0	0	0	0	0	0	0	0
SAL_IA7600AA	PNUSAS009123	Brandenburg	0	0	0	0	0	0	0	0	0	0	0
SAL_IA7599AA	PNUSAS009127	Brandenburg	0	0	0	0	0	0	0	0	0	0	0
SAL_IA7678AA	FSIS1710345	Brandenburg	0	0	0	0	0	0	0	0	0	0	0
SAL_IA8310AA	PNUSAS009145	Brandenburg	0	0	0	0	0	0	0	0	0	0	0
SAL_IA8915AA	FSIS1710739	Brandenburg	0	0	0	0	0	0	0	0	0	0	0
SAL_IA9251AA	PNUSAS009321	Brandenburg	0	0	0	0	0	0	0	0	0	0	0
SAL_IA9801AA	PNUSAS010056	Brandenburg	0	0	0	0	0	0	0	0	0	0	0
SAL_JA1945AA	PNUSAS011508	Brandenburg	0	0	0	0	0	0	0	0	0	0	0
SAL_JA2840AA	FSIS1700224	Brandenburg	0	0	0	0	0	0	0	0	0	0	0
SAL_JA2887AA	PNUSAS012040	Brandenburg	0	0	0	0	0	0	0	0	0	0	0
SAL_JA3267AA	PNUSAS011866	Brandenburg	0	0	0	0	0	0	0	0	0	0	0
SAL_JA3014AA	PNUSAS012286	Brandenburg	0	0	0	0	0	0	0	0	0	0	0
SAL_JA3605AA	FSIS1700441	Brandenburg	0	0	0	0	0	0	0	0	0	0	0
SAL_JA4418AA	955/71	Brandenburg	0	0	0	0	0	0	0	0	0	0	0
SAL_JA4609AA	CVM 43831	Brandenburg	0	0	0	0	0	0	0	0	0	0	0
SAL_JA4669AA	CVM 43771	Brandenburg	0	0	0	0	0	0	0	0	0	0	0
SAL_JA6299AA	PNUSAS013152	Brandenburg	0	0	0	0	0	0	0	0	0	0	0
SAL_JA6045AA	FSIS1700936	Brandenburg	0	0	0	0	0	0	0	0	0	0	0
SAL_JA5951AA	348066	Brandenburg	0	0	0	0	0	0	0	0	0	0	0
SAL_JA5818AA	345719	Brandenburg	0	0	0	0	0	0	0	0	0	0	0
SAL_JA5410AA	353899	Brandenburg	0	0	0	0	0	0	0	0	0	0	0
SAL_JA6766AA	FSIS1700869	Brandenburg	0	0	0	0	0	0	0	0	0	0	0
SAL_JA6663AA	PNUSAS013621	Brandenburg	0	0	0	0	0	0	0	0	0	0	0
SAL_JA8411AA	343807	Brandenburg	21	14	36	56	16	0	0	0	0	0	0
SAL_JA8338AA	346895	Brandenburg	21	14	36	56	16	0	0	0	0	0	0
SAL_JA7858AA	356630	Brandenburg	0	0	0	0	0	0	0	0	0	0	0
SAL_JA7620AA	PNUSAS012963	Brandenburg	0	0	0	0	0	0	0	0	0	0	0
SAL_JA8606AA	FSIS1700871	Brandenburg	0	0	0	0	0	0	0	0	0	0	0

SAL_JA8637AA	PNUSAS014840	Brandenburg	0	0	0	0	0	0	0	0	0	0	0
SAL_JA8847AA	FSIS1701281	Brandenburg	0	0	0	0	0	0	0	0	0	0	0
SAL_JA9019AA	MS170117	Brandenburg	0	0	0	0	0	0	0	0	0	0	0
SAL_JA9020AA	MS170116	Brandenburg	0	0	0	0	0	0	0	0	0	0	0
SAL_JA9021AA	MS170115	Brandenburg	0	0	0	0	0	0	0	0	0	0	0
SAL_JA9016AA	MS170120	Brandenburg	0	0	0	0	0	0	0	0	0	0	0
SAL_KA1128AA	2015AM-0092	Brandenburg	0	0	0	0	0	0	0	0	0	0	0
SAL_KA1621AA	MS170143	Brandenburg	0	0	0	0	0	0	0	0	0	0	0
SAL_KA1622AA	MS170142	Brandenburg	0	0	0	0	0	0	0	0	0	0	0
SAL_KA1623AA	MS170141	Brandenburg	0	0	0	0	0	0	0	0	0	0	0
SAL_KA1626AA	MS170138	Brandenburg	0	0	0	0	0	0	0	0	0	0	0
SAL_KA1627AA	MS170137	Brandenburg	0	0	0	0	0	0	0	0	0	0	0
SAL_KA1635AA	MS170129	Brandenburg	0	0	0	0	0	0	0	0	0	0	0
SAL_KA1639AA	MS170125	Brandenburg	0	0	0	0	0	0	0	0	0	0	0
SAL_KA1640AA	MS170124	Brandenburg	0	0	0	0	0	0	0	0	0	0	0
SAL_KA1641AA	MS170123	Brandenburg	0	0	0	0	0	0	0	0	0	0	0
SAL_KA1620AA	MS170144	Brandenburg	0	0	0	0	0	0	0	0	0	0	0
SAL_KA1624AA	MS170140	Brandenburg	0	0	0	0	0	0	0	0	0	0	0
SAL_KA1625AA	MS170139	Brandenburg	0	0	0	0	0	0	0	0	0	0	0
SAL_KA1628AA	MS170136	Brandenburg	0	0	0	0	0	0	0	0	0	0	0
SAL_KA1632AA	MS170132	Brandenburg	0	0	0	0	0	0	0	0	0	0	0
SAL_KA1633AA	MS170131	Brandenburg	0	0	0	0	0	0	0	0	0	0	0
SAL_KA1634AA	MS170130	Brandenburg	0	0	0	0	0	0	0	0	0	0	0
SAL_KA1912AA	PNUSAS014705	Brandenburg	0	0	0	0	0	0	0	0	0	0	0
SAL_KA2202AA	MS170145	Brandenburg	0	0	0	0	0	0	0	0	0	0	0
SAL_KA2160AA	FDA00004599	Brandenburg	0	0	0	0	0	0	0	0	0	0	0
SAL_KA2179AA	MS170171	Brandenburg	0	0	0	0	0	0	0	0	0	0	0
SAL_KA2181AA	MS170169	Brandenburg	0	0	0	0	0	0	0	0	0	0	0
SAL_KA2180AA	MS170170	Brandenburg	0	0	0	0	0	0	0	0	0	0	0
SAL_KA2176AA	MS170175	Brandenburg	0	0	0	0	0	0	0	0	0	0	0
SAL_KA2184AA	MS170164	Brandenburg	0	0	0	0	0	0	0	0	0	0	0
SAL_KA2183AA	MS170165	Brandenburg	0	0	0	0	0	0	0	0	0	0	0
SAL_KA2186AA	MS170162	Brandenburg	0	0	0	0	0	0	0	0	0	0	0
SAL_KA2193AA	MS170155	Brandenburg	0	0	0	0	0	0	0	0	0	0	0
SAL_KA2194AA	MS170154	Brandenburg	0	0	0	0	0	0	0	0	0	0	0
SAL_KA2192AA	MS170156	Brandenburg	0	0	0	0	0	0	0	0	0	0	0
SAL_KA2196AA	MS170152	Brandenburg	0	0	0	0	0	0	0	0	0	0	0
SAL_KA2187AA	MS170161	Brandenburg	0	0	0	0	0	0	0	0	0	0	0
SAL_KA2185AA	MS170163	Brandenburg	0	0	0	0	0	0	0	0	0	0	0
SAL_KA2199AA	MS170149	Brandenburg	0	0	0	0	0	0	0	0	0	0	0
SAL_KA2198AA	MS170150	Brandenburg	0	0	0	0	0	0	0	0	0	0	0
SAL_KA2391AA	FSIS1702336	Brandenburg	0	0	0	0	0	0	0	0	0	0	0
SAL_KA3308AA	PNUSAS014859	Brandenburg	0	0	0	0	0	0	0	0	0	0	0
SAL_KA4620AA	PNUSAS015986	Brandenburg	0	0	0	0	0	0	0	0	0	0	0
SAL_KA8729AA	SARB3 (Fidelma Boyd)	Brandenburg	0	0	0	0	0	0	0	0	0	0	0
SAL_KA6150AA	2015AM-0496	Brandenburg	0	0	0	0	0	0	0	0	0	0	0
SAL_KA6283AA	FSIS1703131	Brandenburg	0	0	0	0	0	0	0	0	0	0	0
SAL_KA7171AA	17-4-20170606	Brandenburg	0	0	0	0	0	0	0	0	0	0	0
SAL_KA6921AA	PNUSAS012215	Brandenburg	0	0	0	0	0	0	0	0	0	0	0
SAL_KA7523AA	PNUSAS019710	Brandenburg	0	0	0	0	0	0	0	0	0	0	0
SAL_KA7846AA	PNUSAS020111	Brandenburg	0	0	0	0	0	0	0	0	0	0	0
SAL_KA9191AA	17036600	Brandenburg	0	0	0	0	0	0	0	0	0	0	0
SAL_KA8980AA	2015AM-0314	Brandenburg	0	0	0	0	0	0	0	0	0	0	0
SAL_KA9773AA	PNUSAS020113	Brandenburg	0	0	0	0	0	0	0	0	0	0	0
SAL_KA9395AA	CFSAN067291	Brandenburg	0	0	0	0	0	0	0	0	0	0	0
SAL_LA0587AA	FSIS11703979	Brandenburg	0	0	0	0	0	0	0	0	0	0	0
SAL_LA1130AA	CFSAN067863	Brandenburg	0	0	0	0	0	0	0	0	0	0	0
SAL_LA1386AA	CFSAN067874	Brandenburg	0	0	0	0	0	0	0	0	0	0	0
SAL_LA3189AA	3205	Brandenburg	0	0	0	0	0	0	0	0	0	0	0
SAL_LA3153AA	01-01676	Brandenburg	0	0	0	0	0	0	0	0	0	0	0
SAL_LA2273AA	PNUSAS021829	Brandenburg	0	0	0	0	0	0	0	0	0	0	0
SAL_LA3859AA	PNUSAS022176	Brandenburg	0	0	0	0	0	0	0	0	0	0	0
SAL_LA3925AA	PNUSAS022610	Brandenburg	0	0	0	0	0	0	0	0	0	0	0
SAL_LA4475AA	PNUSAS023007	Brandenburg	0	0	0	0	0	0	0	0	0	0	0
SAL_LA4468AA	PNUSAS022829	Brandenburg	0	0	0	0	0	0	0	0	0	0	0
SAL_LA6471AA	PNUSAS024033	Brandenburg	0	0	0	0	0	0	0	0	0	0	0
SAL_LA6764AA	PNUSAS022981	Brandenburg	0	0	0	0	0	0	0	0	0	0	0
SAL_LA6094AA	CFSAN069176	Brandenburg	0	0	0	0	0	0	0	0	0	0	0
SAL_LA8213AA	FSIS21720237	Brandenburg	0	0	0	0	0	0	0	0	0	0	0
SAL_MA0011AA	PNUSAS025071	Brandenburg	0	0	0	0	0	0	0	0	0	0	0
SAL_MA0365AA	FSIS11704670	Brandenburg	0	0	0	0	0	0	0	0	0	0	0
SAL_MA0379AA	PNUSAS024175	Brandenburg	0	0	0	0	0	0	0	0	0	0	0
SAL_MA1244AA	CFSAN070288	Brandenburg	0	0	0	0	0	0	0	0	0	0	0

SAL_MA1428AA	PNUSAS025513	Brandenburg	0	0	0	0	0	0	0	0	0	0	0
SAL_MA1258AA	34944	Brandenburg	0	0	0	0	0	0	0	0	0	0	0
SAL_MA2283AA	FSIS21720387	Brandenburg	0	0	0	0	0	0	0	0	0	0	0
SAL_MA2590AA	FSIS21720434	Brandenburg	0	0	0	0	0	0	0	0	0	0	0
SAL_MA2868AA	PNUSAS026326	Brandenburg	0	0	0	0	0	0	0	0	0	0	0
SAL_MA3509AA	PNUSAS024235	Brandenburg	0	0	0	0	0	0	0	0	0	0	0
SAL_MA3944AA	34930	Brandenburg	0	0	0	0	0	0	0	0	0	0	0
SAL_MA3924AA	CFSAN070520	Brandenburg	0	0	0	0	0	0	0	0	0	0	0
SAL_MA4787AA	FSIS11704570	Brandenburg	0	0	0	0	0	0	0	0	0	0	0
SAL_MA5451AA	28-23-8101210	Brandenburg	0	0	0	0	0	0	0	0	0	0	0
SAL_DA8243AA	2443-sc-2010-09-29-9858	Brandenburg	0	0	0	0	0	0	0	0	0	0	0
SAL_BA2232AA	FDA00001198	Brandenburg	0	0	0	0	0	0	0	0	0	0	0
SAL_DA0439AA	H123600587	Brandenburg	0	0	0	0	0	0	0	0	0	0	0
SAL_CA7304AA	FDA00001094	Brandenburg	0	0	0	0	0	0	0	0	0	0	0
SAL_DA5456AA	NY-29459	Brandenburg	0	0	0	0	0	0	0	0	0	0	0
SAL_DA5314AA	AZ-TG74720	Brandenburg	0	0	0	0	0	0	0	0	0	0	0
SAL_CA2616AA	85751	Brandenburg	0	0	0	0	0	0	0	0	0	0	0
SAL_BA6528AA	NY-N13192	Brandenburg	0	0	0	0	0	0	0	0	0	0	0
SAL_BA8642AA	69929	Brandenburg	0	0	0	0	0	0	0	0	0	0	0
SAL_GA9287AA	FSIS1607592	Brandenburg	0	0	0	0	0	0	0	0	0	0	0
SAL_HA7777AA	PNUSAS006152	Brandenburg	0	0	0	0	0	0	0	0	0	0	0
SAL_GA1967AA	BCW_4853	Brandenburg	0	0	0	0	0	0	0	0	0	0	0
SAL_GA1861AA	BCW_5089	Brandenburg	0	0	0	0	0	0	0	0	0	0	0
SAL_EA7938AA	21861	Brandenburg	0	0	0	0	0	0	0	0	0	0	0
SAL_IA4828AA	MS160225	Brandenburg	0	0	0	0	0	0	0	0	0	0	0
SAL_IA4761AA	MS160180	Brandenburg	0	0	0	0	0	0	0	0	0	0	0
SAL_JA5989AA	357756	Brandenburg	0	0	0	0	0	0	0	0	0	0	0
SAL_KA3552AA	FSIS1701908	Brandenburg	0	0	0	0	0	0	0	0	0	0	0
SAL_KA5394AA	PNUSAS017443	Brandenburg	0	0	0	0	0	0	0	0	0	0	0
SAL_LA2373AA	FSIS11704094	Brandenburg	0	0	0	0	0	0	0	0	0	0	0
SAL_MA5607AA	PNUSAS025617	Brandenburg	0	0	0	0	0	0	0	0	0	0	0
SAL_MA7234AA	PNUSAS028105	Brandenburg	0	0	0	0	0	0	0	0	0	0	0
SAL_MA6951AA	CFSAN071427	Brandenburg	0	0	0	0	0	0	0	0	0	0	0
SAL_MA6967AA	CFSAN071429	Brandenburg	0	0	0	0	0	0	0	0	0	0	0
SAL_MA6922AA	CFSAN071428	Brandenburg	0	0	0	0	0	0	0	0	0	0	0
SAL_MA7661AA	TX-ACF1702407	Brandenburg	0	0	0	0	0	0	0	0	0	0	0
SAL_MA7662AA	CFSAN071180	Brandenburg	0	0	0	0	0	0	0	0	0	0	0
SAL_MA7504AA	FSIS11705462	Brandenburg	0	0	0	0	0	0	0	0	0	0	0
SAL_MA7736AA	PNUSAS028727	Brandenburg	0	0	0	0	0	0	0	0	0	0	0
SAL_MA8480AA	ERS2017954	Brandenburg	0	0	0	0	0	0	0	0	0	0	0
SAL_MA8846AA	FSIS11705528	Brandenburg	0	0	0	0	0	0	0	0	0	0	0
SAL_MA9081AA	CFSAN071832	Brandenburg	0	0	0	0	0	0	0	0	0	0	0
SAL_MA9015AA	CFSAN071842	Brandenburg	0	0	0	0	0	0	0	0	0	0	0
SAL_MA9018AA	CFSAN071841	Brandenburg	0	0	0	0	0	0	0	0	0	0	0
SAL_MA9028AA	FSIS11705335	Brandenburg	0	0	0	0	0	0	0	0	0	0	0
SAL_NA0179AA	CFSAN072132	Brandenburg	0	0	0	0	0	0	0	0	0	0	0
SAL_NA0171AA	CFSAN071843	Brandenburg	0	0	0	0	0	0	0	0	0	0	0
SAL_MA9676AA	CVM N16S020	Brandenburg	0	0	0	0	0	0	0	0	0	0	0
SAL_MA9631AA	CVM N16S367	Brandenburg	0	0	0	0	0	0	0	0	0	0	0
SAL_NA0723AA	PNUSAS029257	Brandenburg	0	0	0	0	0	0	0	0	0	0	0
SAL_NA0975AA	CFSAN072170	Brandenburg	0	0	0	0	0	0	0	0	0	0	0
SAL_NA1357AA	FSIS21720675	Brandenburg	0	0	0	0	0	0	0	0	0	0	0
SAL_NA1651AA	FSIS11706319	Brandenburg	0	0	0	0	0	0	0	0	0	0	0
SAL_NA1624AA	FSIS11706329	Brandenburg	0	0	0	0	0	0	0	0	0	0	0
SAL_NA2027AA	CFSAN072623	Brandenburg	0	0	0	0	0	0	0	0	0	0	0
SAL_NA3171AA	CFSAN073564	Brandenburg	0	0	0	0	0	0	0	0	0	0	0
SAL_NA3437AA	PNUSAS030663	Brandenburg	0	0	0	0	0	0	0	0	0	0	0
SAL_NA3600AA	PNUSAS030239	Brandenburg	0	0	0	0	0	0	0	0	0	0	0
SAL_NA3912AA	2015AM-3319	Brandenburg	0	0	0	0	0	0	0	0	0	0	0
SAL_NA4346AA	PNUSAS030691	Brandenburg	0	0	0	0	0	0	0	0	0	0	0
SAL_NA5136AA	PNUSAS030498	Brandenburg	0	0	0	0	0	0	0	0	0	0	0
SAL_NA5262AA	2015AM-2007	Brandenburg	0	0	0	0	0	0	0	0	0	0	0
SAL_NA5270AA	2015AM-1942	Brandenburg	0	0	0	0	0	0	0	0	0	0	0
SAL_NA6063AA	2015AM-2569	Brandenburg	0	0	0	0	0	0	0	0	0	0	0
SAL_NA6132AA	2015AM-2393	Brandenburg	0	0	0	0	0	0	0	0	0	0	0
SAL_NA5856AA	CFSAN074239	Brandenburg	0	0	0	0	0	0	0	0	0	0	0
SAL_NA6571AA	PNUSAS032330	Brandenburg	0	0	0	0	0	0	0	0	0	0	0
SAL_NA7507AA	FSIS31800062	Brandenburg	0	0	0	0	0	0	0	0	0	0	0
SAL_NA9005AA	PNUSAS031575	Brandenburg	0	0	0	0	0	0	0	0	0	0	0
SAL_NA8923AA	FSIS11807256	Brandenburg	0	0	0	0	0	0	0	0	0	0	0
SAL_NA8739AA	FSIS21821035	Brandenburg	0	0	0	0	0	0	0	0	0	0	0
SAL_NA9665AA	FSIS31800116	Brandenburg	0	0	0	0	0	0	0	0	0	0	0
SAL_OA7467AA	FSIS21821344	Brandenburg	0	0	0	0	0	0	0	0	0	0	0

SAL_OA7471AA	FSIS11807905	Brandenburg	0	0	0	0	0	0	0	0	0	0	0
SAL_OA9962AA	PNUSAS037419	Brandenburg	0	0	0	0	0	0	0	0	0	0	0
SAL_OA4624AA	PNUSAS035956	Brandenburg	0	0	0	0	0	0	0	0	0	0	0
SAL_OA3818AA	FSIS21821267	Brandenburg	0	0	0	0	0	0	0	0	0	0	0
SAL_OA3835AA	FSIS21821199	Brandenburg	0	0	0	0	0	0	0	0	0	0	0
SAL_PA1359AA	PNUSAS038046	Brandenburg	0	0	0	0	0	0	0	0	0	0	0
SAL_PA3803AA	CFSAN078680	Brandenburg	0	0	0	0	0	0	0	0	0	0	0
SAL_PA4146AA	CFSAN078633	Brandenburg	0	0	0	0	0	0	0	0	0	0	0
SAL_PA4154AA	CFSAN078632	Brandenburg	0	0	0	0	0	0	0	0	0	0	0
SAL_PA4222AA	PNUSAS039629	Brandenburg	0	0	0	0	0	0	0	0	0	0	0
SAL_PA4332AA	CFSAN078621	Brandenburg	0	0	0	0	0	0	0	0	0	0	0
SAL_PA4266AA	379326	Brandenburg	0	0	0	0	0	0	0	0	0	0	0
SAL_PA4334AA	CFSAN078622	Brandenburg	0	0	0	0	0	0	0	0	0	0	0
SAL_PA4665AA	PNUSAS039711	Brandenburg	0	0	0	0	0	0	0	0	0	0	0
SAL_PA5472AA	PNUSAS038913	Brandenburg	0	0	0	0	0	0	0	0	0	0	0
SAL_PA5685AA	PNUSAS040046	Brandenburg	0	0	0	0	0	0	0	0	0	0	0
SAL_PA6299AA	PNUSAS040208	Brandenburg	0	0	0	0	0	0	0	0	0	0	0
SAL_PA6296AA	PNUSAS040200	Brandenburg	0	0	0	0	0	0	0	0	0	0	0
SAL_PA7107AA	PNUSAS040541	Brandenburg	0	0	0	0	0	0	0	0	0	0	0
SAL_PA7250AA	CFSAN080878	Brandenburg	0	0	0	0	0	0	0	0	0	0	0
SAL_PA7229AA	CFSAN080880	Brandenburg	0	0	0	0	0	0	0	0	0	0	0
SAL_PA7200AA	CFSAN080876	Brandenburg	0	0	0	0	0	0	0	0	0	0	0
SAL_PA7208AA	CFSAN080883	Brandenburg	0	0	0	0	0	0	0	0	0	0	0
SAL_PA7186AA	CFSAN080875	Brandenburg	0	0	0	0	0	0	0	0	0	0	0
SAL_PA7188AA	CFSAN080872	Brandenburg	0	0	0	0	0	0	0	0	0	0	0
SAL_PA7202AA	CFSAN080877	Brandenburg	0	0	0	0	0	0	0	0	0	0	0
SAL_PA7396AA	CFSAN080879	Brandenburg	0	0	0	0	0	0	0	0	0	0	0
SAL_PA7460AA	CFSAN080953	Brandenburg	0	0	0	0	0	0	0	0	0	0	0
SAL_PA7335AA	FSIS11809390	Brandenburg	0	0	0	0	0	0	0	0	0	0	0
SAL_PA7685AA	CFSAN080881	Brandenburg	0	0	0	0	0	0	0	0	0	0	0
SAL_PA8310AA	NC_SH291	Brandenburg	0	0	0	0	0	0	0	0	0	0	0
SAL_PA8318AA	FSIS11809998	Brandenburg	0	0	0	0	0	0	0	0	0	0	0
SAL_PA9113AA	FSIS31800436	Brandenburg	0	0	0	0	0	0	0	0	0	0	0
SAL_PA9533AA	NC_SH686	Brandenburg	0	0	0	0	0	0	0	0	0	0	0
SAL_QA1194AA	FSIS11810248	Brandenburg	0	0	0	0	0	0	0	0	0	0	0
SAL_QA1306AA	164935	Brandenburg	0	0	0	0	0	0	0	0	0	0	0
SAL_QA1287AA	PNUSAS042189	Brandenburg	0	0	0	0	0	0	0	0	0	0	0
SAL_QA0896AA	128164	Brandenburg	0	0	0	0	0	0	0	0	0	0	0
SAL_QA1099AA	PNUSAS042226	Brandenburg	0	0	0	0	0	0	0	0	0	0	0
SAL_QA2618AA	FSIS21821721	Brandenburg	0	0	0	0	0	0	0	0	0	0	0
SAL_QA3466AA	248633	Brandenburg	0	0	0	0	0	0	0	0	0	0	0
SAL_QA3453AA	251869	Brandenburg	0	0	0	0	0	0	0	0	0	0	0
SAL_QA3936AA	313263	Brandenburg	0	0	0	0	0	0	0	0	0	0	0
SAL_QA2179AA	399505	Brandenburg	0	0	0	0	0	0	0	0	0	0	0
SAL_QA4132AA	FDA00004878	Brandenburg	0	0	0	0	0	0	0	0	0	0	0
SAL_QA5261AA	227039	Brandenburg	0	0	0	0	0	0	0	0	0	0	0
SAL_QA5640AA	PNUSAS043645	Brandenburg	0	0	0	0	0	0	0	0	0	0	0
SAL_QA6603AA	374022	Brandenburg	0	0	0	0	0	0	0	0	0	0	0
SAL_QA6526AA	342572	Brandenburg	21	14	36	56	16	0	0	0	0	0	0
SAL_QA6430AA	320938	Brandenburg	0	0	0	0	0	0	0	0	0	0	0
SAL_QA6646AA	FSIS31800435	Brandenburg	0	0	0	0	0	0	0	0	0	0	0
SAL_QA6702AA	304423	Brandenburg	0	0	0	0	0	0	0	0	0	0	0
SAL_QA6998AA	PNUSAS044131	Brandenburg	0	0	0	0	0	0	0	0	0	0	0
SAL_QA6959AA	PNUSAS044133	Brandenburg	0	0	0	0	0	0	0	0	0	0	0
SAL_QA7332AA	316240	Brandenburg	0	0	0	0	0	0	0	0	0	0	0
SAL_QA7567AA	329825	Brandenburg	21	14	36	56	16	0	0	0	0	0	0
SAL_QA7476AA	341359	Brandenburg	0	0	0	0	0	0	0	0	0	0	0
SAL_QA8432AA	285668	Brandenburg	0	0	0	0	0	0	0	0	0	0	0
SAL_QA8235AA	484126	Brandenburg	0	0	0	0	0	0	0	0	0	0	0
SAL_QA8556AA	FSIS11810931	Brandenburg	0	0	0	0	0	0	0	0	0	0	0
SAL_QA8645AA	190568	Brandenburg	0	0	0	0	0	0	0	0	0	0	0
SAL_QA9235AA	FSIS11811115	Brandenburg	0	0	0	0	0	0	0	0	0	0	0
SAL_RA0108AA	PNUSAS044024	Brandenburg	0	0	0	0	0	0	0	0	0	0	0
SAL_RA0652AA	245120	Brandenburg	0	0	0	0	0	0	0	0	0	0	0
SAL_RA1503AA	0b1-ec00-4ec4-b35e-9d5	Brandenburg	0	0	0	0	0	0	0	0	0	0	0
SAL_RA3539AA	FSIS11811382	Brandenburg	0	0	0	0	0	0	0	0	0	0	0
SAL_RA4055AA	257354	Brandenburg	0	0	0	0	0	0	0	0	0	0	0
SAL_RA3847AA	CFSAN082320	Brandenburg	0	0	0	0	0	0	0	0	0	0	0
SAL_RA4422AA	224062	Brandenburg	0	0	0	0	0	0	0	0	0	0	0
SAL_RA6101AA	PNUSAS045356	Brandenburg	0	0	0	0	0	0	0	0	0	0	0
SAL_RA6030AA	PNUSAS045810	Brandenburg	0	0	0	0	0	0	0	0	0	0	0
SAL_RA5704AA	212788	Brandenburg	0	0	0	0	0	0	0	0	0	0	0
SAL_RA5573AA	267022	Brandenburg	0	0	0	0	0	0	0	0	0	0	0

SAL_RA5427AA	212787	Brandenburg	0	0	0	0	0	0	0	0	0	0	0
SAL_RA6281AA	PNUSAS045610	Brandenburg	0	0	0	0	0	0	0	0	0	0	0
SAL_RA6551AA	411989	Brandenburg	0	0	0	0	0	0	0	0	0	0	0
SAL_RA1803AA	395-c0eb-484c-8395-e66	Brandenburg	0	0	0	0	0	0	0	0	0	0	0
SAL_RA6833AA	PNUSAS043599	Brandenburg	0	0	0	0	0	0	0	0	0	0	0
SAL_RA7784AA	FSIS11811641	Brandenburg	0	0	0	0	0	0	0	0	0	0	0
SAL_RA7896AA	568354	Brandenburg	0	0	0	0	0	0	0	0	0	0	0
SAL_RA8651AA	FSIS11812222	Brandenburg	0	0	0	0	0	0	0	0	0	0	0
SAL_RA9234AA	CFSAN084107	Brandenburg	0	0	0	0	0	0	0	0	0	0	0
SAL_SA0174AA	CVM N17S1521	Brandenburg	0	0	0	0	0	0	0	0	0	0	0
SAL_SA5374AA	H2559	Brandenburg	0	0	0	0	0	0	0	0	0	0	0
SAL_SA3201AA		Brandenburg	0	0	0	0	0	0	0	0	0	0	0
SAL_SA3330AA	SAL-18-VL-LA-KS-0004	Brandenburg	0	0	0	0	0	0	0	0	0	0	0
SAL_SA3362AA	FSIS31800937	Brandenburg	0	0	0	0	0	0	0	0	0	0	0
SAL_SA3621AA	FSIS31800946	Brandenburg	0	0	0	0	0	0	0	0	0	0	0
SAL_SA3687AA	FSIS11813708	Brandenburg	0	0	0	0	0	0	0	0	0	0	0
SAL_SA5620AA	SE06/8	Brandenburg	0	0	0	0	0	0	0	0	0	0	0
SAL_SA4776AA	581307	Brandenburg	0	0	0	0	0	0	0	0	0	0	0
SAL_SA4678AA	601691	Brandenburg	0	0	0	0	0	0	0	0	0	0	0
SAL_SA7544AA	318346	Brandenburg	0	0	0	0	0	0	0	0	0	0	0
SAL_SA8885AA	420523	Brandenburg	0	0	0	0	0	0	0	0	0	0	0
SAL_SA9478AA	350640	Brandenburg	0	0	0	0	0	0	0	0	0	0	0
SAL_SA9705AA	FSIS31801044	Brandenburg	0	0	0	0	0	0	0	0	0	0	0
SAL_TA0375AA	613091	Brandenburg	0	0	0	0	0	0	0	0	0	0	0
SAL_TA1389AA	FSIS11814652	Brandenburg	0	0	0	0	0	0	0	0	0	0	0
SAL_TA1840AA	101309.15	Brandenburg	0	0	0	0	0	0	0	0	0	0	0
SAL_TA2460AA	SAL-18-VL-LA-KS-0015	Brandenburg	0	0	0	0	0	0	0	0	0	0	0
SAL_TA3032AA	FSIS11815065	Brandenburg	0	0	0	0	0	0	0	0	0	0	0
SAL_TA7015AA	CFSAN087654	Brandenburg	0	0	0	0	0	0	0	0	0	0	0
SAL_TA7217AA	SMG-18-2186	Brandenburg	0	0	0	0	0	0	0	0	0	0	0
SAL_SA6696AA	593588	Brandenburg	0	0	0	0	0	0	0	0	0	0	0
SAL_TA2053AA	CFSAN043223	Brandenburg	0	0	0	0	0	0	0	0	0	0	0
SAL_TA7809AA	mples of swine - serotype	Brandenburg	0	0	0	0	0	0	0	0	0	0	0
SAL_TA7836AA	mples of swine - serotype	Brandenburg	0	0	0	0	0	0	0	0	0	0	0
SAL_TA7840AA	amples of swine - serotyp	Brandenburg	0	0	0	0	0	0	0	0	0	0	0
SAL_TA7846AA	amples of swine - serotyp	Brandenburg	0	0	0	0	0	0	0	0	0	0	0
SAL_TA8514AA	636371	Brandenburg	0	0	0	0	0	0	0	0	0	0	0
SAL_TA8433AA	638074	Brandenburg	0	0	0	0	0	0	0	0	0	0	0
SAL_KA2177AA	MS170174	Brandenburg	0	0	0	0	0	0	0	0	0	0	0
SAL_PA8708AA	308783	Brandenburg	0	0	0	0	0	0	0	0	0	0	0
SAL_OA2356AA	FSIS21821133	Brandenburg	0	0	0	0	0	0	0	0	0	0	0
SAL_OA5270AA	PNUSAS035306	Brandenburg	0	0	0	0	0	0	0	0	0	0	0
SAL_OA7472AA	FSIS11808583	Brandenburg	0	0	0	0	0	0	0	0	0	0	0
SAL_PA2159AA	FSIS11808916	Brandenburg	0	0	0	0	0	0	0	0	0	0	0
SAL_PA2790AA	PNUSAS033535	Brandenburg	0	0	0	0	0	0	0	0	0	0	0
SAL_PA8686AA	164684	Brandenburg	0	0	0	0	0	0	0	0	0	0	0
SAL_QA0377AA	164687	Brandenburg	0	0	0	0	0	0	0	0	0	0	0
SAL_RA1009AA	421180	Brandenburg	0	0	0	0	0	0	0	0	0	0	0
SAL_TA9660AA	FSIS31801280	Brandenburg	0	0	0	0	0	0	0	0	0	0	0
SAL_TA9802AA	FSIS11816130	Brandenburg	0	0	0	0	0	0	0	0	0	0	0
SAL_TA9976AA	642305	Brandenburg	0	0	0	0	0	0	0	0	0	0	0
SAL_TA9967AA	644154	Brandenburg	0	0	0	0	0	0	0	0	0	0	0
SAL_RA8497AA	SA20113174	Brandenburg	0	0	0	0	0	0	0	0	0	0	0
SAL_RA8523AA	SA20064858	Brandenburg	0	0	0	0	0	0	0	0	0	0	0
SAL_UA1649AA	FSIS31801254	Brandenburg	0	0	0	0	0	0	0	0	0	0	0
SAL_UA2848AA	649898	Brandenburg	21	14	36	56	16	0	0	0	0	0	0
SAL_UA3121AA	01_Brandenburg_h	Brandenburg	0	0	0	0	0	0	0	0	0	0	0
SAL_UA6899AA	CFSAN089893	Brandenburg	0	0	0	0	0	0	0	0	0	0	0
SAL_UA3081AA	45_Brandenburg	Brandenburg	0	0	0	0	0	0	0	0	0	0	0
SAL_UA3091AA	35_Brandenburg_cp	Brandenburg	0	0	0	0	0	0	0	0	0	0	0
SAL_UA7684AA	AHD232	Brandenburg	0	0	0	0	0	0	0	0	0	0	0
SAL_UA9894AA	559799	Brandenburg	0	0	0	0	0	0	0	0	0	0	0
SAL_VA1459AA	UFRGS-SA004	Brandenburg	0	0	0	0	0	0	0	0	0	0	0
SAL_VA8040AA	CZ_18_0367	Brandenburg	0	0	0	0	0	0	0	0	0	0	0
SAL_VA8065AA	CZ_18_0621	Brandenburg	0	0	0	0	0	0	0	0	0	0	0
SAL_VA3305AA	408613	Brandenburg	0	0	0	0	0	0	0	0	0	0	0
SAL_VA8095AA	CZ_18_0680	Brandenburg	0	0	0	0	0	0	0	0	0	0	0
SAL_VA8096AA	CZ_18_0703	Brandenburg	0	0	0	0	0	0	0	0	0	0	0
SAL_VA8100AA	CZ_18_0731	Brandenburg	0	0	0	0	0	0	0	0	0	0	0
SAL_VA8107AA	CZ_18_1058	Brandenburg	0	0	0	0	0	0	0	0	0	0	0
SAL_VA8106AA	CZ_18_0971	Brandenburg	0	0	0	0	0	0	0	0	0	0	0
SAL_VA2794AA	414577	Brandenburg	0	0	0	0	0	0	0	0	0	0	0
SAL_VA5920AA	ST122	Brandenburg	0	0	0	0	0	0	0	0	0	0	0

SAL_VA5743AA	S-0474	Brandenburg	0	0	0	0	0	0	0	0	0	0	0
SAL_VA4510AA	400309	Brandenburg	0	0	0	0	0	0	0	0	0	0	0
SAL_VA4419AA	408816	Brandenburg	0	0	0	0	0	0	0	0	0	0	0
SAL_VA4424AA	404315	Brandenburg	0	0	0	0	0	0	0	0	0	0	0
SAL_VA6888AA	408198	Brandenburg	0	0	0	0	0	0	0	0	0	0	0
SAL_VA7172AA	392862	Brandenburg	0	0	0	0	0	0	0	0	0	0	0
SAL_VA7844AA	FSIS11917856	Brandenburg	0	0	0	0	0	0	0	0	0	0	0
SAL_VA7834AA	FSIS11917857	Brandenburg	0	0	0	0	0	0	0	0	0	0	0
SAL_VA7946AA	FSIS11917861	Brandenburg	0	0	0	0	0	0	0	0	0	0	0
SAL_VA8265AA	385500	Brandenburg	0	0	0	0	0	0	0	0	0	0	0
SAL_VA4222AA	CDPH_CSalm353	Brandenburg	0	0	0	0	0	0	0	0	0	0	0
SAL_VA9031AA	296210	Brandenburg	0	0	0	0	0	0	0	0	0	0	0
SAL_VA9244AA	E1S1356	Brandenburg	0	0	0	0	0	0	0	0	0	0	0
SAL_VA9547AA	693309	Brandenburg	0	0	0	0	0	0	0	0	0	0	0
SAL_VA9718AA	695974	Brandenburg	0	0	0	0	0	0	0	0	0	0	0
SAL_VA9999AA	238677	Brandenburg	0	0	0	0	0	0	0	0	0	0	0
SAL_VA9926AA	245087	Brandenburg	0	0	0	0	0	0	0	0	0	0	0
SAL_WA0170AA	232721	Brandenburg	0	0	0	0	0	0	0	0	0	0	0
SAL_WA0181AA	212785	Brandenburg	0	0	0	0	0	0	0	0	0	0	0
SAL_WA0547AA	205612	Brandenburg	0	0	0	0	0	0	0	0	0	0	0
SAL_WA0534AA	212786	Brandenburg	0	0	0	0	0	0	0	0	0	0	0
SAL_WA0449AA	212789	Brandenburg	0	0	0	0	0	0	0	0	0	0	0
SAL_WA0421AA	176239	Brandenburg	21	14	36	56	16	0	0	0	0	0	0
SAL_WA0698AA	169586	Brandenburg	0	0	0	0	0	0	0	0	0	0	0
SAL_WA0745AA	166383	Brandenburg	0	0	0	0	0	0	0	0	0	0	0
SAL_WA1328AA	123921	Brandenburg	0	0	0	0	0	0	0	0	0	0	0
SAL_WA3053AA	GW_S01178-08	Brandenburg	0	0	0	0	0	0	0	0	0	0	0
SAL_WA2878AA	H103540735	Brandenburg	0	0	0	0	0	0	0	0	0	0	0
SAL_WA2407AA	CIT_ST95	Brandenburg	0	0	0	0	0	0	0	0	0	0	0
SAL_WA2252AA	SM-DU-2008-04-001368	Brandenburg	0	0	0	0	0	0	0	0	0	0	0
SAL_VA7998AA	FSIS11917567	Brandenburg	0	0	0	0	0	0	0	0	0	0	0
SAL_WA3331AA	707197	Brandenburg	0	0	0	0	0	0	0	0	0	0	0
SAL_WA3684AA	CFSAN087462	Brandenburg	0	0	0	0	0	0	0	0	0	0	0
SAL_WA4191AA	SMG-19-434	Brandenburg	21	14	36	56	16	0	0	0	0	0	0
SAL_EA1386AA	FL_UFL-SR15	Brazil	0	0	0	0	0	0	0	0	0	0	0
SAL_LA7477AA	PNUSAS019990	Brazil	0	0	0	0	0	0	0	0	0	0	0
SAL_IA9461AA	PNUSAS009260	Brazil	0	0	0	0	0	0	0	0	0	0	0
SAL_JA9349AA	PNUSAS008123	Brazil	0	0	0	0	0	0	0	0	0	0	0
SAL_OA2482AA	MOD1-Stmin-300	Brazil	0	0	0	0	0	0	0	0	0	0	0
SAL_KA7633AA	PNUSAS019528	Brazos	0	0	0	0	0	0	0	0	0	0	0
SAL_FA3262AA	FSIS1606022	Breda	0	0	0	0	0	0	0	0	0	0	0
SAL_FA2869AA	CVM N57332F	Breda	0	0	0	0	0	0	0	0	0	0	0
SAL_FA2859AA	CVM N57358F	Breda	0	0	0	0	0	0	0	0	0	0	0
SAL_EA9264AA	FSIS1504825	Breda	0	0	0	0	0	0	0	0	0	0	0
SAL_EA3021AA	FSIS1502793	Breda	0	0	0	0	0	0	0	0	0	0	0
SAL_EA1845AA	FSIS1502276	Breda	0	0	0	0	0	0	0	0	0	0	0
SAL_EA1665AA	FSIS1500755	Breda	0	0	0	0	0	0	0	0	0	0	0
SAL_EA0415AA	AZ_TG68463	Breda	0	0	0	0	0	0	0	0	0	0	0
SAL_DA0352AA	FDA00003524	Breda	0	0	0	0	0	0	0	0	0	0	0
SAL_DA0312AA	FDA00003521	Breda	0	0	0	0	0	0	0	0	0	0	0
SAL_DA0303AA	FDA00003522	Breda	0	0	0	0	0	0	0	0	0	0	0
SAL_DA0297AA	FDA00003523	Breda	0	0	0	0	0	0	0	0	0	0	0
SAL_BA9948AA	:-2012-07-27T11:52:51Z-	Breda	0	0	0	0	0	0	0	0	0	0	0
SAL_BA8970AA	53115	Breda	0	0	0	0	0	0	0	0	0	0	0
SAL_BA3103AA	FSIS1501016	Breda	0	0	0	0	0	0	0	0	0	0	0
SAL_FA6197AA	FSIS1606330	Breda	0	0	0	0	0	0	0	0	0	0	0
SAL_FA6327AA	FSIS1606512	Breda	0	0	0	0	0	0	0	0	0	0	0
SAL_FA7745AA	PNUSAS002341	Breda	0	0	0	0	0	0	0	0	0	0	0
SAL_FA7588AA	FSIS1606688	Breda	0	0	0	0	0	0	0	0	0	0	0
SAL_FA9776AA	FSIS1504589	Breda	0	0	0	0	0	0	0	0	0	0	0
SAL_GA4750AA	PNUSAS002322	Breda	0	0	0	0	0	0	0	0	0	0	0
SAL_FA8478AA	PNUSAS002189	Breda	0	0	0	0	0	0	0	0	0	0	0
SAL_HA6961AA	CFSAN056792	Breda	0	0	0	0	0	0	0	0	0	0	0
SAL_HA7641AA	FSIS1609215	Breda	0	0	0	0	0	0	0	0	0	0	0
SAL_HA7738AA	PNUSAS006290	Breda	0	0	0	0	0	0	0	0	0	0	0
SAL_HA8805AA	FSIS1609411	Breda	0	0	0	0	0	0	0	0	0	0	0
SAL_IA1073AA	171349	Breda	0	0	0	0	0	0	0	0	0	0	0
SAL_IA5368AA	FSIS1709979	Breda	0	0	0	0	0	0	0	0	0	0	0
SAL_IA9297AA	PNUSAS007048	Breda	0	0	0	0	0	0	0	0	0	0	0
SAL_IA9768AA	FSIS1700059	Breda	0	0	0	0	0	0	0	0	0	0	0
SAL_JA1075AA	PNUSAS007062	Breda	0	0	0	0	0	0	0	0	0	0	0
SAL_JA1900AA	PNUSAS011595	Breda	0	0	0	0	0	0	0	0	0	0	0
SAL_JA2306AA	FSIS1700270	Breda	0	0	0	0	0	0	0	0	0	0	0

SAL_JA2384AA	PNUSAS010947	Breda	0	0	0	0	0	0	0	0	0	0	0
SAL_JA3563AA	PNUSAS012619	Breda	0	0	0	0	0	0	0	0	0	0	0
SAL_JA7036AA	PNUSAS012531	Breda	0	0	0	0	0	0	0	0	0	0	0
SAL_JA7007AA	PNUSAS011922	Breda	0	0	0	0	0	0	0	0	0	0	0
SAL_JA7668AA	PNUSAS014544	Breda	0	0	0	0	0	0	0	0	0	0	0
SAL_JA7674AA	PNUSAS014208	Breda	0	0	0	0	0	0	0	0	0	0	0
SAL_JA7550AA	PNUSAS014207	Breda	0	0	0	0	0	0	0	0	0	0	0
SAL_JA9437AA	PNUSAS014907	Breda	0	0	0	0	0	0	0	0	0	0	0
SAL_JA9043AA	PNUSAS014209	Breda	0	0	0	0	0	0	0	0	0	0	0
SAL_JA9059AA	PNUSAS014204	Breda	0	0	0	0	0	0	0	0	0	0	0
SAL_KA0202AA	E2016008485	Breda	0	0	0	0	0	0	0	0	0	0	0
SAL_KA0126AA	PNUSAS015360	Breda	0	0	0	0	0	0	0	0	0	0	0
SAL_KA0607AA	2015AM-1407	Breda	0	0	0	0	0	0	0	0	0	0	0
SAL_KA1326AA	PNUSAS016172	Breda	0	0	0	0	0	0	0	0	0	0	0
SAL_KA1332AA	2017K-0449	Breda	0	0	0	0	0	0	0	0	0	0	0
SAL_KA0966AA	FSIS1701749	Breda	0	0	0	0	0	0	0	0	0	0	0
SAL_KA3610AA	PNUSAS016534	Breda	0	0	0	0	0	0	0	0	0	0	0
SAL_KA4725AA	PNUSAS016576	Breda	0	0	0	0	0	0	0	0	0	0	0
SAL_KA5582AA	FSIS1702945	Breda	0	0	0	0	0	0	0	0	0	0	0
SAL_KA6009AA	2017K-0545	Breda	0	0	0	0	0	0	0	0	0	0	0
SAL_KA6976AA	PNUSAS018132	Breda	0	0	0	0	0	0	0	0	0	0	0
SAL_KA8990AA	2017K-0621	Breda	0	0	0	0	0	0	0	0	0	0	0
SAL_KA9855AA	FSIS1703558	Breda	0	0	0	0	0	0	0	0	0	0	0
SAL_LA1398AA	CFSAN067884	Breda	0	0	0	0	0	0	0	0	0	0	0
SAL_LA4425AA	PNUSAS023167	Breda	0	0	0	0	0	0	0	0	0	0	0
SAL_LA4717AA	PNUSAS016575	Breda	0	0	0	0	0	0	0	0	0	0	0
SAL_LA5623AA	20-17-20170793	Breda	0	0	0	0	0	0	0	0	0	0	0
SAL_LA7091AA	2012122081	Breda	0	0	0	0	0	0	0	0	0	0	0
SAL_LA7048AA	2007750373	Breda	0	0	0	0	0	0	0	0	0	0	0
SAL_MA0331AA	FSIS11704668	Breda	0	0	0	0	0	0	0	0	0	0	0
SAL_FA2762AA	CVM N57680F	Breda	0	0	0	0	0	0	0	0	0	0	0
SAL_MA8831AA	FSIS11705341	Breda	0	0	0	0	0	0	0	0	0	0	0
SAL_NA0551AA	FSIS11706155	Breda	0	0	0	0	0	0	0	0	0	0	0
SAL_OA4260AA	PNUSAS030633	Breda	0	0	0	0	0	0	0	0	0	0	0
SAL_OA3769AA	FSIS31800220	Breda	0	0	0	0	0	0	0	0	0	0	0
SAL_PA7243AA	FSIS11809387	Breda	0	0	0	0	0	0	0	0	0	0	0
SAL_QA1877AA	432173	Breda	0	0	0	0	0	0	0	0	0	0	0
SAL_QA2370AA	408510	Breda	0	0	0	0	0	0	0	0	0	0	0
SAL_QA2560AA	PNUSAS042730	Breda	0	0	0	0	0	0	0	0	0	0	0
SAL_QA9847AA	176328	Breda	0	0	0	0	0	0	0	0	0	0	0
SAL_RA6967AA	FSIS11811414	Breda	0	0	0	0	0	0	0	0	0	0	0
SAL_SA3768AA	FSIS11813410	Breda	0	0	0	0	0	0	0	0	0	0	0
SAL_SA4356AA	FSIS31801029	Breda	0	0	0	0	0	0	0	0	0	0	0
SAL_TA1907AA	FSIS11814657	Breda	0	0	0	0	0	0	0	0	0	0	0
SAL_TA5294AA	627286	Breda	0	0	0	0	0	0	0	0	0	0	0
SAL_OA1441AA	FSIS11807264	Breda	0	0	0	0	0	0	0	0	0	0	0
SAL_OA5633AA	FSIS11808312	Breda	0	0	0	0	0	0	0	0	0	0	0
SAL_UA9650AA	OH-18-19710	Breda	0	0	0	0	0	0	0	0	0	0	0
SAL_VA0926AA	FSIS31901506	Breda	0	0	0	0	0	0	0	0	0	0	0
SAL_VA9836AA	FSIS21923657	Breda	0	0	0	0	0	0	0	0	0	0	0
SAL_VA4096AA	CDPH_CSalm076	Breda	0	0	0	0	0	0	0	0	0	0	0
SAL_WA3348AA	707080	Breda	0	0	0	0	0	0	0	0	0	0	0
SAL_WA3677AA	CFSAN087448	Breda	0	0	0	0	0	0	0	0	0	0	0
SAL_EA0536AA	FDN0072	Bredeney	0	0	0	0	0	0	0	0	0	0	0
SAL_EA0597AA	FDN0076	Bredeney	0	0	0	0	0	0	0	0	0	0	0
SAL_DA5102AA	FMA0140	Bredeney	0	0	0	0	0	0	0	0	0	0	0
SAL_BA5460AA	FDA00002609	Bredeney	0	0	0	0	0	0	0	0	0	0	0
SAL_CA6508AA	49723	Bredeney	0	0	0	0	0	0	0	0	0	0	0
SAL_DA9599AA	FMA0047	Bredeney	0	0	0	0	0	0	0	0	0	0	0
SAL_EA1933AA	CVM N54730	Bredeney	0	0	0	0	0	0	0	0	0	0	0
SAL_BA6602AA	S-1807	Bredeney	0	0	0	0	0	0	0	0	0	0	0
SAL_BA7924AA	90462	Bredeney	0	0	0	0	0	0	0	0	0	0	0
SAL_FA5176AA	FDA00003953	Bredeney	0	0	0	0	0	0	0	0	0	0	0
SAL_FA4664AA	201561	Bredeney	0	0	0	0	0	0	0	0	0	0	0
SAL_FA4644AA	188355	Bredeney	0	0	0	0	0	0	0	0	0	0	0
SAL_FA4567AA	158968	Bredeney	0	0	0	0	0	0	0	0	0	0	0
SAL_FA4492AA	159694	Bredeney	0	0	0	0	0	0	0	0	0	0	0
SAL_FA4067AA	188165	Bredeney	0	0	0	0	0	0	0	0	0	0	0
SAL_FA3318AA	IEH-NGS-SAL-03934	Bredeney	0	0	0	0	0	0	0	0	0	0	0
SAL_FA3319AA	CFSAN047590	Bredeney	0	0	0	0	0	0	0	0	0	0	0
SAL_FA2840AA	CVM N57407F	Bredeney	0	0	0	0	0	0	0	0	0	0	0
SAL_FA2744AA	CVM N57723F	Bredeney	0	0	0	0	0	0	0	0	0	0	0
SAL_FA0910AA	FDA00002458	Bredeney	0	0	0	0	0	0	0	0	0	0	0

SAL_EA7636AA	43920	Bredeney	0	0	0	0	0	0	0	0	0	0	0
SAL_EA7549AA	43954	Bredeney	0	0	0	0	0	0	0	0	0	0	0
SAL_EA7181AA	40335	Bredeney	0	0	0	0	0	0	0	0	0	0	0
SAL_EA6923AA	21110	Bredeney	0	0	0	0	0	0	0	0	0	0	0
SAL_EA6846AA	FDA00004095	Bredeney	0	0	0	0	0	0	0	0	0	0	0
SAL_EA6378AA	FSIS1503440	Bredeney	0	0	0	0	0	0	0	0	0	0	0
SAL_EA3871AA	CVM N43475	Bredeney	0	0	0	0	0	0	0	0	0	0	0
SAL_EA2014AA	CVM N53833	Bredeney	0	0	0	0	0	0	0	0	0	0	0
SAL_EA1483AA	FSIS1500648	Bredeney	0	0	0	0	0	0	0	0	0	0	0
SAL_EA1122AA	M309 / NCTC20222	Bredeney	0	0	0	0	0	0	0	0	0	0	0
SAL_EA0575AA	FDN0086	Bredeney	0	0	0	0	0	0	0	0	0	0	0
SAL_EA0529AA	FDN0061	Bredeney	0	0	0	0	0	0	0	0	0	0	0
SAL_EA0533AA	FDN0064	Bredeney	0	0	0	0	0	0	0	0	0	0	0
SAL_EA0489AA	FAR0018	Bredeney	0	0	0	0	0	0	0	0	0	0	0
SAL_EA0132AA	WAPHL_SAL-A00111	Bredeney	0	0	0	0	0	0	0	0	0	0	0
SAL_DA9992AA	FMA0005	Bredeney	0	0	0	0	0	0	0	0	0	0	0
SAL_DA9921AA	FMA0025	Bredeney	0	0	0	0	0	0	0	0	0	0	0
SAL_DA9904AA	FMA0034	Bredeney	0	0	0	0	0	0	0	0	0	0	0
SAL_DA9903AA	FMA0037	Bredeney	0	0	0	0	0	0	0	0	0	0	0
SAL_DA9843AA	FMA0024	Bredeney	0	0	0	0	0	0	0	0	0	0	0
SAL_DA9836AA	FMA0029	Bredeney	0	0	0	0	0	0	0	0	0	0	0
SAL_DA9686AA	VA_WGS-00049	Bredeney	0	0	0	0	0	0	0	0	0	0	0
SAL_DA9610AA	FMA0019	Bredeney	0	0	0	0	0	0	0	0	0	0	0
SAL_DA9604AA	FMA0041	Bredeney	0	0	0	0	0	0	0	0	0	0	0
SAL_DA9579AA	FMA0012	Bredeney	0	0	0	0	0	0	0	0	0	0	0
SAL_DA9511AA	VA_WGS-00105	Bredeney	0	0	0	0	0	0	0	0	0	0	0
SAL_DA9371AA	WAPHL-SAL-A00244	Bredeney	0	0	0	0	0	0	0	0	0	0	0
SAL_DA9369AA	WAPHL-SAL-A00242	Bredeney	0	0	0	0	0	0	0	0	0	0	0
SAL_DA8419AA	MDH-2014-00175	Bredeney	0	0	0	0	0	0	0	0	0	0	0
SAL_DA7415AA	MDH-2014-00067	Bredeney	0	0	0	0	0	0	0	0	0	0	0
SAL_DA7153AA	CFSAN012636	Bredeney	0	0	0	0	0	0	0	0	0	0	0
SAL_DA7104AA	FL-NRM028	Bredeney	0	0	0	0	0	0	0	0	0	0	0
SAL_DA7058AA	VA_WGS-00199	Bredeney	0	0	0	0	0	0	0	0	0	0	0
SAL_DA6077AA	MOD1_Sal_844	Bredeney	0	0	0	0	0	0	0	0	0	0	0
SAL_DA4234AA	MDH-2014-00429	Bredeney	0	0	0	0	0	0	0	0	0	0	0
SAL_DA4053AA	MDH-2014-00434	Bredeney	0	0	0	0	0	0	0	0	0	0	0
SAL_DA4045AA	AZ-TG74772	Bredeney	0	0	0	0	0	0	0	0	0	0	0
SAL_DA4008AA	AZ-TG74756	Bredeney	0	0	0	0	0	0	0	0	0	0	0
SAL_DA2955AA	CFSAN023683	Bredeney	0	0	0	0	0	0	0	0	0	0	0
SAL_DA2356AA	FDN0080	Bredeney	0	0	0	0	0	0	0	0	0	0	0
SAL_DA2253AA	2012K-1361	Bredeney	0	0	0	0	0	0	0	0	0	0	0
SAL_DA2152AA	FDN0042	Bredeney	0	0	0	0	0	0	0	0	0	0	0
SAL_DA2153AA	FDN0070	Bredeney	0	0	0	0	0	0	0	0	0	0	0
SAL_DA1287AA	H124440665	Bredeney	0	0	0	0	0	0	0	0	0	0	0
SAL_DA1224AA	H123640752	Bredeney	0	0	0	0	0	0	0	0	0	0	0
SAL_DA1068AA	H120800519	Bredeney	0	0	0	0	0	0	0	0	0	0	0
SAL_DA0539AA	H124440666	Bredeney	0	0	0	0	0	0	0	0	0	0	0
SAL_DA0381AA	H124520451	Bredeney	0	0	0	0	0	0	0	0	0	0	0
SAL_CA8797AA	NY-32502	Bredeney	0	0	0	0	0	0	0	0	0	0	0
SAL_CA8796AA	AZ-TG74768	Bredeney	0	0	0	0	0	0	0	0	0	0	0
SAL_CA8774AA	NY-32503	Bredeney	0	0	0	0	0	0	0	0	0	0	0
SAL_CA8770AA	AZ-TG74760	Bredeney	0	0	0	0	0	0	0	0	0	0	0
SAL_CA7031AA	NY-N11246	Bredeney	0	0	0	0	0	0	0	0	0	0	0
SAL_CA6706AA	64992	Bredeney	0	0	0	0	0	0	0	0	0	0	0
SAL_CA6700AA	60385	Bredeney	0	0	0	0	0	0	0	0	0	0	0
SAL_CA6674AA	63500	Bredeney	0	0	0	0	0	0	0	0	0	0	0
SAL_CA6631AA	63561	Bredeney	0	0	0	0	0	0	0	0	0	0	0
SAL_CA6275AA	64940	Bredeney	0	0	0	0	0	0	0	0	0	0	0
SAL_CA5828AA	70640	Bredeney	0	0	0	0	0	0	0	0	0	0	0
SAL_CA5499AA	75755	Bredeney	0	0	0	0	0	0	0	0	0	0	0
SAL_CA5447AA	75738	Bredeney	0	0	0	0	0	0	0	0	0	0	0
SAL_CA4436AA	5923	Bredeney	0	0	0	0	0	0	0	0	0	0	0
SAL_CA4230AA	25332	Bredeney	0	0	0	0	0	0	0	0	0	0	0
SAL_CA3430AA	83317	Bredeney	0	0	0	0	0	0	0	0	0	0	0
SAL_CA3396AA	5870	Bredeney	0	0	0	0	0	0	0	0	0	0	0
SAL_CA3338AA	83316	Bredeney	0	0	0	0	0	0	0	0	0	0	0
SAL_CA3300AA	65425	Bredeney	0	0	0	0	0	0	0	0	0	0	0
SAL_CA3283AA	65422	Bredeney	0	0	0	0	0	0	0	0	0	0	0
SAL_CA2893AA	40255	Bredeney	0	0	0	0	0	0	0	0	0	0	0
SAL_CA2573AA	60168	Bredeney	0	0	0	0	0	0	0	0	0	0	0
SAL_CA2237AA	50315	Bredeney	0	0	0	0	0	0	0	0	0	0	0
SAL_CA0850AA	53130	Bredeney	0	0	0	0	0	0	0	0	0	0	0
SAL_CA0662AA	83731	Bredeney	0	0	0	0	0	0	0	0	0	0	0

SAL_BA9659AA	61888	Bredeney	0	0	0	0	0	0	0	0	0	0	0
SAL_BA9398AA	50880	Bredeney	0	0	0	0	0	0	0	0	0	0	0
SAL_BA8740AA	36489	Bredeney	0	0	0	0	0	0	0	0	0	0	0
SAL_BA6632AA	401129	Bredeney	0	0	0	0	0	0	0	0	0	0	0
SAL_BA6313AA	NY-N13194	Bredeney	0	0	0	0	0	0	0	0	0	0	0
SAL_BA5472AA	FDA00002610	Bredeney	0	0	0	0	0	0	0	0	0	0	0
SAL_GA2836AA	CRJJGF_00075	Bredeney	0	0	0	0	0	0	0	0	0	0	0
SAL_GA2835AA	CRJJGF_00076	Bredeney	0	0	0	0	0	0	0	0	0	0	0
SAL_GA2630AA	08-1537	Bredeney	0	0	0	0	0	0	0	0	0	0	0
SAL_GA2627AA	2012K-1362	Bredeney	0	0	0	0	0	0	0	0	0	0	0
SAL_GA2622AA	00-0315	Bredeney	0	0	0	0	0	0	0	0	0	0	0
SAL_GA2618AA	04-0041	Bredeney	0	0	0	0	0	0	0	0	0	0	0
SAL_GA2615AA	51-2633	Bredeney	0	0	0	0	0	0	0	0	0	0	0
SAL_GA2614AA	58-5391	Bredeney	0	0	0	0	0	0	0	0	0	0	0
SAL_GA3545AA	BCW_2794	Bredeney	0	0	0	0	0	0	0	0	0	0	0
SAL_GA3531AA	BCW_2826	Bredeney	0	0	0	0	0	0	0	0	0	0	0
SAL_GA3488AA	BCW_2885	Bredeney	0	0	0	0	0	0	0	0	0	0	0
SAL_GA3215AA	N29351	Bredeney	0	0	0	0	0	0	0	0	0	0	0
SAL_GA2335AA	BCW_2653	Bredeney	0	0	0	0	0	0	0	0	0	0	0
SAL_GA2032AA	BCW_4350	Bredeney	0	0	0	0	0	0	0	0	0	0	0
SAL_GA4894AA	CCC_L5	Bredeney	0	0	0	0	0	0	0	0	0	0	0
SAL_FA9726AA	FDA00001799	Bredeney	0	0	0	0	0	0	0	0	0	0	0
SAL_FA9707AA	ADRDL-NS 021A	Bredeney	0	0	0	0	0	0	0	0	0	0	0
SAL_FA9706AA	ADRDL-NS 021B	Bredeney	0	0	0	0	0	0	0	0	0	0	0
SAL_FA9677AA	CFSAN042063	Bredeney	0	0	0	0	0	0	0	0	0	0	0
SAL_GA4623AA	CCC_K2	Bredeney	0	0	0	0	0	0	0	0	0	0	0
SAL_GA4991AA	CCC_94-3042	Bredeney	0	0	0	0	0	0	0	0	0	0	0
SAL_GA4993AA	CCC_95-0269	Bredeney	0	0	0	0	0	0	0	0	0	0	0
SAL_GA4996AA	CCC_L3	Bredeney	0	0	0	0	0	0	0	0	0	0	0
SAL_GA4624AA	CCC_R3	Bredeney	0	0	0	0	0	0	0	0	0	0	0
SAL_GA5837AA	CCC_In-Sin2	Bredeney	0	0	0	0	0	0	0	0	0	0	0
SAL_GA5852AA	CK_93/00007708-1	Bredeney	0	0	0	0	0	0	0	0	0	0	0
SAL_GA5849AA	CCC_M1	Bredeney	0	0	0	0	0	0	0	0	0	0	0
SAL_FA8801AA	CVM N29351	Bredeney	0	0	0	0	0	0	0	0	0	0	0
SAL_GA6380AA	FDA00010471	Bredeney	0	0	0	0	0	0	0	0	0	0	0
SAL_GA6426AA	PNUSAS002737	Bredeney	0	0	0	0	0	0	0	0	0	0	0
SAL_GA7189AA	CFSAN025350	Bredeney	0	0	0	0	0	0	0	0	0	0	0
SAL_GA7187AA	CFSAN025352	Bredeney	0	0	0	0	0	0	0	0	0	0	0
SAL_GA7545AA	CFSAN025370	Bredeney	0	0	0	0	0	0	0	0	0	0	0
SAL_GA7550AA	CFSAN025364	Bredeney	0	0	0	0	0	0	0	0	0	0	0
SAL_GA7544AA	CFSAN025371	Bredeney	0	0	0	0	0	0	0	0	0	0	0
SAL_GA9571AA	FDA00001729	Bredeney	0	0	0	0	0	0	0	0	0	0	0
SAL_GA9477AA	CVM N62964	Bredeney	0	0	0	0	0	0	0	0	0	0	0
SAL_GA9570AA	FDA00001730	Bredeney	0	0	0	0	0	0	0	0	0	0	0
SAL_HA0446AA	PNUSAS004043	Bredeney	0	0	0	0	0	0	0	0	0	0	0
SAL_HA0658AA	2014AM-2647	Bredeney	0	0	0	0	0	0	0	0	0	0	0
SAL_HA2822AA	FSIS1608121	Bredeney	0	0	0	0	0	0	0	0	0	0	0
SAL_HA3992AA	FSIS1608361	Bredeney	0	0	0	0	0	0	0	0	0	0	0
SAL_HA5971AA	CFSAN056585	Bredeney	0	0	0	0	0	0	0	0	0	0	0
SAL_HA7265AA	_Salmonella_newport_N	Bredeney	0	0	0	0	0	0	0	0	0	0	0
SAL_HA8596AA	PNUSAS006407	Bredeney	0	0	0	0	0	0	0	0	0	0	0
SAL_HA8265AA	PNUSAS006496	Bredeney	0	0	0	0	0	0	0	0	0	0	0
SAL_IA1806AA	287743	Bredeney	0	0	0	0	0	0	0	0	0	0	0
SAL_IA2761AA	MS160144	Bredeney	0	0	0	0	0	0	0	0	0	0	0
SAL_IA5908AA	MS160401	Bredeney	0	0	0	0	0	0	0	0	0	0	0
SAL_IA6088AA	PNUSAS008172	Bredeney	0	0	0	0	0	0	0	0	0	0	0
SAL_IA5770AA	MS150001	Bredeney	0	0	0	0	0	0	0	0	0	0	0
SAL_IA6401AA	FSIS1710508	Bredeney	0	0	0	0	0	0	0	0	0	0	0
SAL_IA7223AA	PNUSAS008755	Bredeney	0	0	0	0	0	0	0	0	0	0	0
SAL_IA8165AA	FDA00002894	Bredeney	0	0	0	0	0	0	0	0	0	0	0
SAL_IA8166AA	FDA00002895	Bredeney	0	0	0	0	0	0	0	0	0	0	0
SAL_IA9348AA	PNUSAS009293	Bredeney	0	0	0	0	0	0	0	0	0	0	0
SAL_JA0849AA	ADRDL-1029	Bredeney	0	0	0	0	0	0	0	0	0	0	0
SAL_JA0845AA	ADRDL-1026	Bredeney	0	0	0	0	0	0	0	0	0	0	0
SAL_JA1484AA	PNUSAS010765	Bredeney	0	0	0	0	0	0	0	0	0	0	0
SAL_JA1178AA	FDAARGOS_314	Bredeney	0	0	0	0	0	0	0	0	0	0	0
SAL_JA0491AA	ADRDL-1000	Bredeney	0	0	0	0	0	0	0	0	0	0	0
SAL_JA2097AA	ADRDL-989	Bredeney	0	0	0	0	0	0	0	0	0	0	0
SAL_JA3273AA	PNUSAS011864	Bredeney	0	0	0	0	0	0	0	0	0	0	0
SAL_JA4187AA	FSIS1700591	Bredeney	0	0	0	0	0	0	0	0	0	0	0
SAL_JA5436AA	340163	Bredeney	0	0	0	0	0	0	0	0	0	0	0
SAL_JA5393AA	360339	Bredeney	0	0	0	0	0	0	0	0	0	0	0
SAL_JA6944AA	FSIS1701225	Bredeney	0	0	0	0	0	0	0	0	0	0	0

SAL_JA7989AA	358406	Bredeney	0	0	0	0	0	0	0	0	0	0	0
SAL_JA7905AA	330555	Bredeney	0	0	0	0	0	0	0	0	0	0	0
SAL_JA8901AA	FSIS1701849	Bredeney	0	0	0	0	0	0	0	0	0	0	0
SAL_KA0165AA	OH-16-22611-5	Bredeney	0	0	0	0	0	0	0	0	0	0	0
SAL_JA8986AA	PNUSAS014389	Bredeney	0	0	0	0	0	0	0	0	0	0	0
SAL_KA1040AA	FSIS1702037	Bredeney	0	0	0	0	0	0	0	0	0	0	0
SAL_MA2503AA	RJ1067_Salmonella_ssp_	Bredeney	0	0	0	0	0	0	0	0	0	0	0
SAL_KA1576AA	PAT-15-16238SA	Bredeney	0	0	0	0	0	0	0	0	0	0	0
SAL_KA1573AA	PR-IISPV-25087	Bredeney	0	0	0	0	0	0	0	0	0	0	0
SAL_KA4212AA	102 VAN-22	Bredeney	0	0	0	0	0	0	0	0	0	0	0
SAL_KA4922AA	PNUSAS017773	Bredeney	0	0	0	0	0	0	0	0	0	0	0
SAL_KA7194AA	17-23-20170631	Bredeney	0	0	0	0	0	0	0	0	0	0	0
SAL_KA8867AA	17016851	Bredeney	0	0	0	0	0	0	0	0	0	0	0
SAL_LA0441AA	PNUSAS019301	Bredeney	0	0	0	0	0	0	0	0	0	0	0
SAL_LA1391AA	PNUSAS021837	Bredeney	0	0	0	0	0	0	0	0	0	0	0
SAL_LA3176AA	M92	Bredeney	0	0	0	0	0	0	0	0	0	0	0
SAL_LA3221AA	3206	Bredeney	0	0	0	0	0	0	0	0	0	0	0
SAL_LA4509AA	PNUSAS023022	Bredeney	0	0	0	0	0	0	0	0	0	0	0
SAL_LA6391AA	PNUSAS023670	Bredeney	0	0	0	0	0	0	0	0	0	0	0
SAL_MA0291AA	PNUSAS024844	Bredeney	0	0	0	0	0	0	0	0	0	0	0
SAL_MA2554AA	SSI-AC406	Bredeney	0	0	0	0	0	0	0	0	0	0	0
SAL_MA2667AA	PNUSAS026165	Bredeney	0	0	0	0	0	0	0	0	0	0	0
SAL_MA3024AA	FDA00001059	Bredeney	0	0	0	0	0	0	0	0	0	0	0
SAL_MA3150AA	FSIS11704402	Bredeney	0	0	0	0	0	0	0	0	0	0	0
SAL_MA4617AA	PNUSAS024358	Bredeney	0	0	0	0	0	0	0	0	0	0	0
SAL_DA4059AA	MDH-2014-00444	Bredeney	0	0	0	0	0	0	0	0	0	0	0
SAL_EA0537AA	FDN0060	Bredeney	0	0	0	0	0	0	0	0	0	0	0
SAL_CA6400AA	49716	Bredeney	0	0	0	0	0	0	0	0	0	0	0
SAL_BA1703AA	FDA00009237	Bredeney	0	0	0	0	0	0	0	0	0	0	0
SAL_DA0889AA	H120920340	Bredeney	0	0	0	0	0	0	0	0	0	0	0
SAL_DA8421AA	VA_WGS-00162	Bredeney	0	0	0	0	0	0	0	0	0	0	0
SAL_HA8650AA	PNUSAS006046	Bredeney	0	0	0	0	0	0	0	0	0	0	0
SAL_BA7954AA	CFSAN001080	Bredeney	0	0	0	0	0	0	0	0	0	0	0
SAL_HA4378AA	FDA00010922	Bredeney	0	0	0	0	0	0	0	0	0	0	0
SAL_GA7549AA	CFSAN025367	Bredeney	0	0	0	0	0	0	0	0	0	0	0
SAL_GA6379AA	FDA00010472	Bredeney	0	0	0	0	0	0	0	0	0	0	0
SAL_FA7203AA	PNUSAS002078	Bredeney	0	0	0	0	0	0	0	0	0	0	0
SAL_FA1890AA	FSIS1605894	Bredeney	0	0	0	0	0	0	0	0	0	0	0
SAL_FA1349AA	FDA00004071	Bredeney	0	0	0	0	0	0	0	0	0	0	0
SAL_FA0040AA	8317_1993-sc-2416924	Bredeney	0	0	0	0	0	0	0	0	0	0	0
SAL_IA9697AA	PNUSAS010349	Bredeney	0	0	0	0	0	0	0	0	0	0	0
SAL_JA1660AA	PNUSAS011305	Bredeney	0	0	0	0	0	0	0	0	0	0	0
SAL_IA7655AA	MS170010	Bredeney	0	0	0	0	0	0	0	0	0	0	0
SAL_MA5694AA	ERS2000938	Bredeney	0	0	0	0	0	0	0	0	0	0	0
SAL_MA6528AA	FSIS21720591	Bredeney	0	0	0	0	0	0	0	0	0	0	0
SAL_MA7330AA	PNUSAS028037	Bredeney	0	0	0	0	0	0	0	0	0	0	0
SAL_MA7571AA	PNUSAS027833	Bredeney	0	0	0	0	0	0	0	0	0	0	0
SAL_MA7590AA	PNUSAS028264	Bredeney	0	0	0	0	0	0	0	0	0	0	0
SAL_MA8490AA	ERS2017944	Bredeney	0	0	0	0	0	0	0	0	0	0	0
SAL_MA8482AA	ERS2017952	Bredeney	0	0	0	0	0	0	0	0	0	0	0
SAL_NA0340AA	PNUSAS027915	Bredeney	0	0	0	0	0	0	0	0	0	0	0
SAL_NA0109AA	lmonella_Bredeney_203	Bredeney	0	0	0	0	0	0	0	0	0	0	0
SAL_MA9508AA	lmonella_Bredeney_118	Bredeney	0	0	0	0	0	0	0	0	0	0	0
SAL_NA4053AA	FSIS11706531	Bredeney	0	0	0	0	0	0	0	0	0	0	0
SAL_NA5135AA	ADRDL-1651	Bredeney	0	0	0	0	0	0	0	0	0	0	0
SAL_NA5528AA	PNUSAS030746	Bredeney	0	0	0	0	0	0	0	0	0	0	0
SAL_NA5874AA	PNUSAS032062	Bredeney	0	0	0	0	0	0	0	0	0	0	0
SAL_NA5831AA	2015AM-2751	Bredeney	0	0	0	0	0	0	0	0	0	0	0
SAL_NA7642AA	12-121747_S13	Bredeney	0	0	0	0	0	0	0	0	0	0	0
SAL_NA7643AA	12-121745_S12	Bredeney	0	0	0	0	0	0	0	0	0	0	0
SAL_NA9430AA	PNUSAS032784	Bredeney	0	0	0	0	0	0	0	0	0	0	0
SAL_OA8142AA	PNUSAS035002	Bredeney	0	0	0	0	0	0	0	0	0	0	0
SAL_PA0918AA	PNUSAS035843	Bredeney	0	0	0	0	0	0	0	0	0	0	0
SAL_PA4455AA	PNUSAS035969	Bredeney	0	0	0	0	0	0	0	0	0	0	0
SAL_PA5633AA	302913	Bredeney	0	0	0	0	0	0	0	0	0	0	0
SAL_PA5593AA	289650	Bredeney	0	0	0	0	0	0	0	0	0	0	0
SAL_PA6111AA	287736	Bredeney	0	0	0	0	0	0	0	0	0	0	0
SAL_PA6363AA	464K	Bredeney	0	0	0	0	0	0	0	0	0	0	0
SAL_PA7341AA	FSIS11809922	Bredeney	0	0	0	0	0	0	0	0	0	0	0
SAL_PA7903AA	480753	Bredeney	0	0	0	0	0	0	0	0	0	0	0
SAL_PA8335AA	247209	Bredeney	0	0	0	0	0	0	0	0	0	0	0
SAL_PA8384AA	346907	Bredeney	0	0	0	0	0	0	0	0	0	0	0
SAL_PA8537AA	435423	Bredeney	0	0	0	0	0	0	0	0	0	0	0

SAL_PA8841AA	20-4-18-8100407-Z	Bredeney	0	0	0	0	0	0	0	0	0	0	0
SAL_PA9705AA	465521	Bredeney	0	0	0	0	0	0	0	0	0	0	0
SAL_QA0417AA	307239	Bredeney	0	0	0	0	0	0	0	0	0	0	0
SAL_QA1358AA	173709	Bredeney	0	0	0	0	0	0	0	0	0	0	0
SAL_QA1934AA	440880	Bredeney	0	0	0	0	0	0	0	0	0	0	0
SAL_QA2403AA	527520	Bredeney	0	0	0	0	0	0	0	0	0	0	0
SAL_QA2336AA	322514	Bredeney	0	0	0	0	0	0	0	0	0	0	0
SAL_QA2057AA	528563	Bredeney	0	0	0	0	0	0	0	0	0	0	0
SAL_QA3090AA	PNUSAS043102	Bredeney	0	0	0	0	0	0	0	0	0	0	0
SAL_QA6667AA	26-19-18-8100552-E	Bredeney	0	0	0	0	0	0	0	0	0	0	0
SAL_QA3980AA	FDA00004902	Bredeney	0	0	0	0	0	0	0	0	0	0	0
SAL_QA4748AA	157250	Bredeney	0	0	0	0	0	0	0	0	0	0	0
SAL_QA4736AA	435359	Bredeney	0	0	0	0	0	0	0	0	0	0	0
SAL_QA5122AA	286731	Bredeney	0	0	0	0	0	0	0	0	0	0	0
SAL_QA5974AA	416375	Bredeney	0	0	0	0	0	0	0	0	0	0	0
SAL_QA6539AA	529841	Bredeney	0	0	0	0	0	0	0	0	0	0	0
SAL_QA6951AA	PNUSAS043433	Bredeney	0	0	0	0	0	0	0	0	0	0	0
SAL_QA7364AA	416338	Bredeney	0	0	0	0	0	0	0	0	0	0	0
SAL_QA7688AA	327910	Bredeney	0	0	0	0	0	0	0	0	0	0	0
SAL_QA8808AA	289673	Bredeney	0	0	0	0	0	0	0	0	0	0	0
SAL_QA9467AA	421683	Bredeney	0	0	0	0	0	0	0	0	0	0	0
SAL_QA9975AA	163119	Bredeney	0	0	0	0	0	0	0	0	0	0	0
SAL_QA9979AA	127417	Bredeney	0	0	0	0	0	0	0	0	0	0	0
SAL_QA9917AA	437049	Bredeney	0	0	0	0	0	0	0	0	0	0	0
SAL_RA0420AA	322265	Bredeney	0	0	0	0	0	0	0	0	0	0	0
SAL_RA0348AA	193382	Bredeney	0	0	0	0	0	0	0	0	0	0	0
SAL_RA1107AA	456770	Bredeney	0	0	0	0	0	0	0	0	0	0	0
SAL_RA1042AA	298929	Bredeney	0	0	0	0	0	0	0	0	0	0	0
SAL_RA1055AA	123877	Bredeney	0	0	0	0	0	0	0	0	0	0	0
SAL_RA0691AA	470217	Bredeney	0	0	0	0	0	0	0	0	0	0	0
SAL_RA8618AA	SMG-18-1057	Bredeney	0	0	0	0	0	0	0	0	0	0	0
SAL_RA8617AA	SMG-18-1058	Bredeney	0	0	0	0	0	0	0	0	0	0	0
SAL_RA2907AA	137126	Bredeney	0	0	0	0	0	0	0	0	0	0	0
SAL_RA3299AA	312814	Bredeney	0	0	0	0	0	0	0	0	0	0	0
SAL_RA3990AA	397660	Bredeney	0	0	0	0	0	0	0	0	0	0	0
SAL_QA9994AA	142493	Bredeney	0	0	0	0	0	0	0	0	0	0	0
SAL_RA7525AA	568275	Bredeney	0	0	0	0	0	0	0	0	0	0	0
SAL_RA7194AA	310307	Bredeney	0	0	0	0	0	0	0	0	0	0	0
SAL_RA7065AA	321062	Bredeney	0	0	0	0	0	0	0	0	0	0	0
SAL_RA7752AA	203659	Bredeney	0	0	0	0	0	0	0	0	0	0	0
SAL_SA3580AA	SMG-18-1627	Bredeney	0	0	0	0	0	0	0	0	0	0	0
SAL_RA7976AA	197083	Bredeney	0	0	0	0	0	0	0	0	0	0	0
SAL_SA2944AA	FSIS31800922	Bredeney	0	0	0	0	0	0	0	0	0	0	0
SAL_SA4963AA	F1485/07	Bredeney	0	0	0	0	0	0	0	0	0	0	0
SAL_SA4858AA	593515	Bredeney	0	0	0	0	0	0	0	0	0	0	0
SAL_SA7842AA	516922	Bredeney	0	0	0	0	0	0	0	0	0	0	0
SAL_SA8668AA	288971	Bredeney	0	0	0	0	0	0	0	0	0	0	0
SAL_SA9491AA	431379	Bredeney	0	0	0	0	0	0	0	0	0	0	0
SAL_SA9464AA	335582	Bredeney	0	0	0	0	0	0	0	0	0	0	0
SAL_TA0482AA	267029	Bredeney	0	0	0	0	0	0	0	0	0	0	0
SAL_TA0485AA	607790	Bredeney	0	0	0	0	0	0	0	0	0	0	0
SAL_TA0445AA	610757	Bredeney	0	0	0	0	0	0	0	0	0	0	0
SAL_TA0371AA	548566	Bredeney	0	0	0	0	0	0	0	0	0	0	0
SAL_TA0797AA	615652	Bredeney	0	0	0	0	0	0	0	0	0	0	0
SAL_TA1398AA	FSIS11814370	Bredeney	0	0	0	0	0	0	0	0	0	0	0
SAL_TA1868AA	120308.28	Bredeney	0	0	0	0	0	0	0	0	0	0	0
SAL_TA2991AA	625762	Bredeney	0	0	0	0	0	0	0	0	0	0	0
SAL_TA5937AA	FSIS21822548	Bredeney	0	0	0	0	0	0	0	0	0	0	0
SAL_TA6210AA	611353	Bredeney	0	0	0	0	0	0	0	0	0	0	0
SAL_TA6728AA	628059	Bredeney	0	0	0	0	0	0	0	0	0	0	0
SAL_TA8294AA	638753	Bredeney	0	0	0	0	0	0	0	0	0	0	0
SAL_TA8404AA	638767	Bredeney	0	0	0	0	0	0	0	0	0	0	0
SAL_TA8596AA	OH-17-16340-1	Bredeney	0	0	0	0	0	0	0	0	0	0	0
SAL_QA7789AA	383813	Bredeney	0	0	0	0	0	0	0	0	0	0	0
SAL_OA5388AA	PNUSAS035091	Bredeney	0	0	0	0	0	0	0	0	0	0	0
SAL_HA0136AA	NCTC6026	Bredeney	0	0	0	0	0	0	0	0	0	0	0
SAL_UA2683AA	SMG-18-2332	Bredeney	0	0	0	0	0	0	0	0	0	0	0
SAL_UA2812AA	649904	Bredeney	0	0	0	0	0	0	0	0	0	0	0
SAL_UA7176AA	FSIS21923151	Bredeney	0	0	0	0	0	0	0	0	0	0	0
SAL_UA7258AA	662846	Bredeney	0	0	0	0	0	0	0	0	0	0	0
SAL_UA3099AA	27_Bredeney_cp	Bredeney	0	0	0	0	0	0	0	0	0	0	0
SAL_UA3734AA	603415	Bredeney	0	0	0	0	0	0	0	0	0	0	0
SAL_UA5290AA	650051	Bredeney	0	0	0	0	0	0	0	0	0	0	0

SAL_UA5285AA	650042	Bredeney	0	0	0	0	0	0	0	0	0	0	0
SAL_UA5318AA	652938	Bredeney	0	0	0	0	0	0	0	0	0	0	0
SAL_UA6331AA	598021	Bredeney	0	0	0	0	0	0	0	0	0	0	0
SAL_UA6733AA	NL_L16	Bredeney	0	0	0	0	0	0	0	0	0	0	0
SAL_UA6748AA	F1480/07	Bredeney	0	0	0	0	0	0	0	0	0	0	0
SAL_UA7852AA	CFSAN090603	Bredeney	0	0	0	0	0	0	0	0	0	0	0
SAL_UA8691AA	669754	Bredeney	0	0	0	0	0	0	0	0	0	0	0
SAL_VA0146AA	568372	Bredeney	0	0	0	0	0	0	0	0	0	0	0
SAL_VA0206AA	568302	Bredeney	0	0	0	0	0	0	0	0	0	0	0
SAL_UA9810AA	572006	Bredeney	0	0	0	0	0	0	0	0	0	0	0
SAL_VA0601AA	201804612	Bredeney	0	0	0	0	0	0	0	0	0	0	0
SAL_VA0641AA	201806259	Bredeney	0	0	0	0	0	0	0	0	0	0	0
SAL_VA7462AA	201703898	Bredeney	0	0	0	0	0	0	0	0	0	0	0
SAL_VA7657AA	201709804	Bredeney	0	0	0	0	0	0	0	0	0	226	0
SAL_VA3456AA	401617	Bredeney	0	0	0	0	0	0	0	0	0	0	0
SAL_VA1987AA	439500	Bredeney	0	0	0	0	0	0	0	0	0	0	0
SAL_VA6056AA	F7	Bredeney	0	0	0	0	0	0	0	0	0	0	0
SAL_VA5897AA	SA20082654	Bredeney	0	0	0	0	0	0	0	0	0	0	0
SAL_VA5842AA	ST156	Bredeney	0	0	0	0	0	0	0	0	0	0	0
SAL_VA5844AA	SA20081835	Bredeney	0	0	0	0	0	0	0	0	0	0	0
SAL_VA5802AA	SA20030746	Bredeney	0	0	0	0	0	0	0	0	0	0	0
SAL_VA4415AA	404894	Bredeney	0	0	0	0	0	0	0	0	0	0	0
SAL_VA7037AA	AMC 301	Bredeney	0	0	0	0	0	0	0	0	0	0	0
SAL_VA7748AA	FSIS11917605	Bredeney	0	0	0	0	0	0	0	0	0	0	0
SAL_VA8264AA	690356	Bredeney	0	0	0	0	0	0	0	0	0	0	0
SAL_VA8184AA	688992	Bredeney	0	0	0	0	0	0	0	0	0	0	0
SAL_VA8202AA	682277	Bredeney	0	0	0	0	0	0	0	0	0	0	0
SAL_VA9077AA	308752	Bredeney	0	0	0	0	0	0	0	0	0	0	0
SAL_VA8986AA	301681	Bredeney	0	0	0	0	0	0	0	0	0	0	0
SAL_VA8919AA	299417	Bredeney	0	0	0	0	0	0	0	0	0	0	0
SAL_VA9438AA	288972	Bredeney	0	0	0	0	0	0	0	0	0	0	0
SAL_VA4188AA	CDPH_CSalm362	Bredeney	0	0	0	0	0	0	0	0	0	0	0
SAL_VA9820AA	256085	Bredeney	0	0	0	0	0	0	0	0	0	0	0
SAL_WA0548AA	264100	Bredeney	0	0	0	0	0	0	0	0	0	0	0
SAL_WA0642AA	FSIS11918333	Bredeney	0	0	0	0	0	0	0	0	0	0	0
SAL_WA0789AA	168347	Bredeney	0	0	0	0	0	0	0	0	0	0	0
SAL_WA0989AA	146076	Bredeney	0	0	0	0	0	0	0	0	0	0	0
SAL_WA3294AA	CCC_In-Sin1	Bredeney	0	0	0	0	0	0	0	0	0	0	0
SAL_WA3289AA	CCC_P4	Bredeney	0	0	0	0	0	0	0	0	0	0	0
SAL_WA3208AA	JE_S09-001173	Bredeney	0	0	0	0	0	0	0	0	0	0	0
SAL_WA3051AA	JE_S09-001174	Bredeney	0	0	0	0	0	0	0	0	0	0	0
SAL_WA2918AA	NL_L22	Bredeney	0	0	0	0	0	0	0	0	0	0	0
SAL_WA2785AA	NL_L10	Bredeney	0	0	0	0	0	0	0	0	0	0	0
SAL_WA2698AA	CIT_D78	Bredeney	0	0	0	0	0	0	0	0	0	0	0
SAL_WA2621AA	103 VAN-22	Bredeney	0	0	0	0	0	0	0	0	0	0	0
SAL_WA2475AA	CCC_H4-1	Bredeney	0	0	0	0	0	0	0	0	0	0	0
SAL_WA2406AA	CIT_ST96	Bredeney	0	0	0	0	0	0	0	0	0	0	0
SAL_WA2405AA	JE_S09-001707	Bredeney	0	0	0	0	0	0	0	0	0	0	0
SAL_WA2220AA	NL_L6	Bredeney	0	0	0	0	0	0	0	0	0	0	0
SAL_WA2225AA	JE_S09-001432	Bredeney	0	0	0	0	0	0	0	0	0	0	0
SAL_WA1267AA	699245	Bredeney	0	0	0	0	0	0	0	0	0	0	0
SAL_WA1244AA	130398	Bredeney	0	0	0	0	0	0	0	0	0	0	0
SAL_WA2188AA	BD-DU-2006-11-005330	Bredeney	0	0	0	0	0	0	0	0	0	0	0
SAL_WA2215AA	GW_S05182-08	Bredeney	0	0	0	0	0	0	0	0	0	0	0
SAL_WA1973AA	704760	Bredeney	0	0	0	0	0	0	0	0	0	0	0
SAL_VA4465AA	387077	Breukelen	0	0	0	0	0	0	0	0	0	0	0
SAL_PA8396AA	265720	Brevik	0	0	0	0	0	0	0	0	0	0	0
SAL_QA5862AA	265841	Brevik	0	0	0	0	0	0	0	0	0	0	0
SAL_GA3338AA	BCW_1597	Brisbane	0	0	0	0	0	0	0	0	0	0	0
SAL_CA7096AA	FDA00000241	Brunei	0	0	0	0	0	0	0	0	0	0	0
SAL_EA9164AA	53128	Brunei	0	0	0	0	0	0	0	0	0	0	0
SAL_DA5279AA	FDA00000379	Brunei	0	0	0	0	0	0	0	0	0	0	0
SAL_FA1919AA	FDA00002520	Brunei	0	0	0	0	0	0	0	0	0	0	0
SAL_FA1580AA	CFSAN045758	Brunei	0	0	0	0	0	0	0	0	0	0	0
SAL_FA1584AA	IEH-NGS-SAL-03591	Brunei	0	0	0	0	0	0	0	0	0	0	0
SAL_FA1578AA	CFSAN046109	Brunei	0	0	0	0	0	0	0	0	0	0	0
SAL_FA1583AA	CFSAN045755	Brunei	0	0	0	0	0	0	0	0	0	0	0
SAL_FA1579AA	CFSAN045759	Brunei	0	0	0	0	0	0	0	0	0	0	0
SAL_FA1254AA	FDA00001291	Brunei	0	0	0	0	0	0	0	0	0	0	0
SAL_EA7865AA	20911	Brunei	0	0	0	0	0	0	0	0	0	0	0
SAL_EA6524AA	FDA00000299	Brunei	0	0	0	0	0	0	0	0	0	0	0
SAL_EA6455AA	FDA00009672	Brunei	0	0	0	0	0	0	0	0	0	0	0
SAL_EA3215AA	FDA00002382	Brunei	0	0	0	0	0	0	0	0	0	0	0

SAL_EA2940AA	FDA00000344	Brunei	0	0	0	0	0	0	0	0	0	0	0
SAL_EA2722AA	FDA00001913	Brunei	0	0	0	0	0	0	0	0	0	0	0
SAL_EA0639AA	FSW0048	Brunei	0	0	0	0	0	0	0	0	0	0	0
SAL_EA0451AA	FSF0031	Brunei	0	0	0	0	0	0	0	0	0	0	0
SAL_EA0388AA	FSF0040	Brunei	0	0	0	0	0	0	0	0	0	0	0
SAL_EA0220AA	FNE0016	Brunei	0	0	0	0	0	0	0	0	0	0	0
SAL_DA9782AA	FNE0067	Brunei	0	0	0	0	0	0	0	0	0	0	0
SAL_DA9115AA	FNE0079	Brunei	0	0	0	0	0	0	0	0	0	0	0
SAL_DA6890AA	FMA0159	Brunei	0	0	0	0	0	0	0	0	0	0	0
SAL_DA6341AA	FMA0196	Brunei	0	0	0	0	0	0	0	0	0	0	0
SAL_DA5825AA	FNW19a27	Brunei	0	0	0	0	0	0	0	0	0	0	0
SAL_DA5261AA	FSF0109	Brunei	0	0	0	0	0	0	0	0	0	0	0
SAL_DA3959AA	FNE0156	Brunei	0	0	0	0	0	0	0	0	0	0	0
SAL_DA2216AA	FNW19e68	Brunei	0	0	0	0	0	0	0	0	0	0	0
SAL_DA2147AA	FDA00000429	Brunei	0	0	0	0	0	0	0	0	0	0	0
SAL_DA1657AA	FDA00003493	Brunei	0	0	0	0	0	0	0	0	0	0	0
SAL_DA1635AA	FDA00003489	Brunei	0	0	0	0	0	0	0	0	0	0	0
SAL_DA1632AA	FDA00003520	Brunei	0	0	0	0	0	0	0	0	0	0	0
SAL_DA0091AA	FDA00000793	Brunei	0	0	0	0	0	0	0	0	0	0	0
SAL_DA0084AA	FDA00000806	Brunei	0	0	0	0	0	0	0	0	0	0	0
SAL_DA0086AA	FDA00000794	Brunei	0	0	0	0	0	0	0	0	0	0	0
SAL_CA9972AA	FDA00003679	Brunei	0	0	0	0	0	0	0	0	0	0	0
SAL_CA8888AA	FDA00008598	Brunei	0	0	0	0	0	0	0	0	0	0	0
SAL_CA8426AA	FDA00000979	Brunei	0	0	0	0	0	0	0	0	0	0	0
SAL_CA8436AA	FDA00000915	Brunei	0	0	0	0	0	0	0	0	0	0	0
SAL_CA8326AA	FDA00003449	Brunei	0	0	0	0	0	0	0	0	0	0	0
SAL_CA8256AA	FDA00003797	Brunei	0	0	0	0	0	0	0	0	0	0	0
SAL_CA8253AA	FDA00003798	Brunei	0	0	0	0	0	0	0	0	0	0	0
SAL_CA8252AA	FDA00003796	Brunei	0	0	0	0	0	0	0	0	0	0	0
SAL_CA8216AA	FDA00008776	Brunei	0	0	0	0	0	0	0	0	0	0	0
SAL_CA8215AA	FDA00008777	Brunei	0	0	0	0	0	0	0	0	0	0	0
SAL_CA7035AA	FDA00002404	Brunei	0	0	0	0	0	0	0	0	0	0	0
SAL_BA7288AA	FDA00002719	Brunei	0	0	0	0	0	0	0	0	0	0	0
SAL_BA2646AA	FDA00001220	Brunei	0	0	0	0	0	0	0	0	0	0	0
SAL_BA2211AA	FDA00001206	Brunei	0	0	0	0	0	0	0	0	0	0	0
SAL_BA1750AA	CFSAN034893	Brunei	0	0	0	0	0	0	0	0	0	0	0
SAL_BA1663AA	FDA00001422	Brunei	0	0	0	0	0	0	0	0	0	0	0
SAL_BA1632AA	FDA00001421	Brunei	0	0	0	0	0	0	0	0	0	0	0
SAL_BA1631AA	FDA00001423	Brunei	0	0	0	0	0	0	0	0	0	0	0
SAL_BA1439AA	FDA00000630	Brunei	0	0	0	0	0	0	0	0	0	0	0
SAL_BA1427AA	FDA00000593	Brunei	0	0	0	0	0	0	0	0	0	0	0
SAL_BA0779AA	FDA00001881	Brunei	0	0	0	0	0	0	0	0	0	0	0
SAL_FA6194AA	PNUSAS001505	Brunei	0	0	0	0	0	0	0	0	0	0	0
SAL_FA8046AA	FDA00010264	Brunei	0	0	0	0	0	0	0	0	0	0	0
SAL_GA4538AA	FDA00001710	Brunei	0	0	0	0	0	0	0	0	0	0	0
SAL_GA4818AA	FDA00000169	Brunei	0	0	0	0	0	0	0	0	0	0	0
SAL_FA8441AA	FDA00000151	Brunei	0	0	0	0	0	0	0	0	0	0	0
SAL_GA6880AA	Salm-QAP2-2016	Brunei	0	0	0	0	0	0	0	0	0	0	0
SAL_GA8499AA	FDA00010516	Brunei	0	0	0	0	0	0	0	0	0	0	0
SAL_GA8508AA	FDA00010527	Brunei	0	0	0	0	0	0	0	0	0	0	0
SAL_GA8756AA	FDA00001450	Brunei	0	0	0	0	0	0	0	0	0	0	0
SAL_GA9237AA	FDA00010594	Brunei	0	0	0	0	0	0	0	0	0	0	0
SAL_HA2666AA	FDA00010836	Brunei	0	0	0	0	0	0	0	0	0	0	0
SAL_MA3059AA	FDA00003336	Brunei	0	0	0	0	0	0	0	0	0	0	0
SAL_DA7028AA	FMA0155	Brunei	0	0	0	0	0	0	0	0	0	0	0
SAL_EA2865AA	FDA00001846	Brunei	0	0	0	0	0	0	0	0	0	0	0
SAL_DA3954AA	FNE0160	Brunei	0	0	0	0	0	0	0	0	0	0	0
SAL_DA1637AA	FDA00003457	Brunei	0	0	0	0	0	0	0	0	0	0	0
SAL_BA2663AA	FDA00001392	Brunei	0	0	0	0	0	0	0	0	0	0	0
SAL_CA7290AA	FDA00001067	Brunei	0	0	0	0	0	0	0	0	0	0	0
SAL_CA7890AA	FDA00003016	Brunei	0	0	0	0	0	0	0	0	0	0	0
SAL_DA1634AA	FDA00003490	Brunei	0	0	0	0	0	0	0	0	0	0	0
SAL_DA1638AA	FDA00000718	Brunei	0	0	0	0	0	0	0	0	0	0	0
SAL_DA1873AA	FDA00000140	Brunei	0	0	0	0	0	0	0	0	0	0	0
SAL_DA7349AA	FSW0185	Brunei	0	0	0	0	0	0	0	0	0	0	0
SAL_DA6120AA	FNE0124	Brunei	0	0	0	0	0	0	0	0	0	0	0
SAL_DA6117AA	FCC0147	Brunei	0	0	0	0	0	0	0	0	0	0	0
SAL_DA9779AA	FNE0055	Brunei	0	0	0	0	0	0	0	0	0	0	0
SAL_BA1952AA	FDA00000994	Brunei	0	0	0	0	0	0	0	0	0	0	0
SAL_BA5839AA	FDA00001186	Brunei	0	0	0	0	0	0	0	0	0	0	0
SAL_CA8008AA	FDA00002818	Brunei	0	0	0	0	0	0	0	0	0	0	0
SAL_DA5998AA	FDA00000028	Brunei	0	0	0	0	0	0	0	0	0	0	0
SAL_DA7361AA	FSW0190	Brunei	0	0	0	0	0	0	0	0	0	0	0

SAL_DA5280AA	FDA00000307	Brunei	0	0	0	0	0	0	0	0	0	0	0
SAL_BA1687AA	FDA00001408	Brunei	0	0	0	0	0	0	0	0	0	0	0
SAL_EA2732AA	FDA00000548	Brunei	0	0	0	0	0	0	0	0	0	0	0
SAL_HA2664AA	FDA00010838	Brunei	0	0	0	0	0	0	0	0	0	0	0
SAL_HA5343AA	FDA00010962	Brunei	0	0	0	0	0	0	0	0	0	0	0
SAL_GA0259AA	SR090-sc-2280762	Brunei	0	0	0	0	0	0	0	0	0	0	0
SAL_FA8047AA	FDA00010263	Brunei	0	0	0	0	0	0	0	0	0	0	0
SAL_EA6530AA	FDA00000301	Brunei	0	0	0	0	0	0	0	0	0	0	0
SAL_IA2456AA	FDA00011140	Brunei	0	0	0	0	0	0	0	0	0	0	0
SAL_IA2682AA	FDA00011223	Brunei	0	0	0	0	0	0	0	0	0	0	0
SAL_PA8259AA	PNUSAS041378	Brunei	0	0	0	0	0	0	0	0	0	0	0
SAL_QA0392AA	225078	Brunei	0	0	0	0	0	0	0	0	0	0	0
SAL_QA3140AA	18EP000870	Brunei	0	0	0	0	0	0	0	0	0	0	0
SAL_QA2220AA	393983	Brunei	0	0	0	0	0	0	0	0	0	0	0
SAL_QA4710AA	244404	Brunei	0	0	0	0	0	0	0	0	0	0	0
SAL_QA5287AA	306608	Brunei	0	0	0	0	0	0	0	0	0	0	0
SAL_QA6902AA	PNUSAS044058	Brunei	0	0	0	0	0	0	0	0	0	0	0
SAL_RA1319AA	459906	Brunei	0	0	0	0	0	0	0	0	0	0	0
SAL_RA3780AA	1509	Brunei	0	0	0	0	0	0	0	0	0	0	0
SAL_RA4244AA	FDA00009768	Brunei	0	0	0	0	0	0	0	0	0	0	0
SAL_RA4524AA	217081	Brunei	0	0	0	0	0	0	0	0	0	0	0
SAL_RA4531AA	182028	Brunei	0	0	0	0	0	0	0	0	0	0	0
SAL_RA4540AA	461187	Brunei	0	0	0	0	0	0	0	0	0	0	0
SAL_RA4341AA	172448	Brunei	0	0	0	0	0	0	0	0	0	0	0
SAL_RA6164AA	2303	Brunei	0	0	0	0	0	0	0	0	0	0	0
SAL_RA5459AA	153183	Brunei	0	0	0	0	0	0	0	0	0	0	0
SAL_RA5450AA	182024	Brunei	0	0	0	0	0	0	0	0	0	0	0
SAL_RA5434AA	223603	Brunei	0	0	0	0	0	0	0	0	0	0	0
SAL_RA6637AA	182025	Brunei	0	0	0	0	0	0	0	0	0	0	0
SAL_RA6498AA	201520	Brunei	0	0	0	0	0	0	0	0	0	0	0
SAL_RA1787AA	FDA00002385	Brunei	0	0	0	0	0	0	0	0	0	0	0
SAL_RA7184AA	182026	Brunei	0	0	0	0	0	0	0	0	0	0	0
SAL_RA7122AA	508263	Brunei	0	0	0	0	0	0	0	0	0	0	0
SAL_RA8287AA	182027	Brunei	0	0	0	0	0	0	0	0	0	0	0
SAL_SA7379AA	224064	Brunei	0	0	0	0	0	0	0	0	0	0	0
SAL_SA8989AA	208783	Brunei	0	0	0	0	0	0	0	0	0	0	0
SAL_SA9518AA	609992	Brunei	0	0	0	0	0	0	0	0	0	0	0
SAL_VA2252AA	461177	Brunei	0	0	0	0	0	0	0	0	0	0	0
SAL_VA2507AA	427413	Brunei	0	0	0	0	0	0	0	0	0	0	0
SAL_WA0264AA	208784	Brunei	0	0	0	0	0	0	0	0	0	0	0
SAL_WA0232AA	692598	Brunei	0	0	0	0	0	0	0	0	0	0	0
SAL_WA0146AA	224066	Brunei	0	0	0	0	0	0	0	0	0	0	0
SAL_VA0681AA	201809630	Businga	5	4	6	8	0	0	0	0	0	0	0
SAL_DA4413AA	FDN0103	Butantan	0	0	0	0	0	0	0	0	0	0	0
SAL_DA2487AA	FDN0160	Butantan	0	0	0	0	0	0	0	0	0	0	0
SAL_CA7977AA	FDA00002865	Butantan	0	0	0	0	0	0	0	0	0	0	0
SAL_DA9334AA	FNW19H27	Butantan	0	0	0	0	0	0	0	0	0	0	0
SAL_FA5854AA	FDA00009776	Buzu	0	0	0	0	0	0	0	0	0	0	0
SAL_FA1073AA	PNUSAS001627	Buzu	0	0	0	0	0	0	0	0	0	0	0
SAL_DA6838AA	CFSAN008301	Buzu	0	0	0	0	0	0	0	0	0	0	0
SAL_BA6669AA	08:MF0016603	Buzu	0	0	0	0	0	0	0	0	0	0	0
SAL_DA6861AA	CFSAN008300	Buzu	0	0	0	0	0	0	0	0	0	0	0
SAL_SA1458AA	RK_08:MF0016603R	Buzu	0	0	0	0	0	0	0	0	0	0	0
SAL_VA8786AA	320313	Cairina	0	0	0	0	0	0	3	4	0	0	1
SAL_LA6076AA	FDA00003157	Canada	0	0	0	0	0	0	0	0	0	0	0
SAL_LA7446AA	FDA00003154	Canada	0	0	0	0	0	0	0	0	0	0	0
SAL_CA4323AA	14860	Canada	0	0	0	0	0	0	0	0	0	0	0
SAL_GA4397AA	PNUSAS002315	Canada	0	0	0	0	0	0	0	0	0	0	0
SAL_QA7373AA	117613	Canada	0	0	0	0	0	0	0	0	0	0	0
SAL_VA7376AA	201606215	Canada	0	0	0	0	0	0	0	0	0	0	0
SAL_VA7378AA	201606144	Canada	0	0	0	0	0	0	0	0	0	0	0
SAL_VA7452AA	201703421	Canada	0	0	0	0	0	0	0	0	0	0	0
SAL_VA4083AA	CDPH_CSalm309	Canada	0	0	0	0	0	0	0	0	0	0	0
SAL_EA4073AA	FDA00009399	Caracas	0	0	0	0	0	0	0	0	0	0	0
SAL_EA4074AA	FDA00009398	Caracas	0	0	0	0	0	0	0	0	0	0	0
SAL_DA9587AA	FAR0067	Caracas	0	0	0	0	0	0	0	0	0	0	0
SAL_DA9581AA	FAR0068	Caracas	0	0	0	0	0	0	0	0	0	0	0
SAL_DA5894AA	FSE0193	Caracas	0	0	0	0	0	0	0	0	0	0	0
SAL_DA1178AA	H123200436	Caracas	0	0	0	0	0	0	0	0	0	0	0
SAL_DA1037AA	H124540691	Caracas	0	0	0	0	0	0	0	0	0	0	0
SAL_CA9431AA	FDA00000549	Caracas	0	0	0	0	0	0	0	0	0	0	0
SAL_CA9281AA	FDA00003828	Caracas	0	0	0	0	0	0	0	0	0	0	0
SAL_CA9250AA	FDA00003824	Caracas	0	0	0	0	0	0	0	0	0	0	0

SAL_CA8427AA	FDA00000906	Caracas	0	0	0	0	0	0	0	0	0	0	0
SAL_CA7987AA	FDA00001007	Caracas	0	0	0	0	0	0	0	0	0	0	0
SAL_CA7236AA	FDA00001026	Caracas	0	0	0	0	0	0	0	0	0	0	0
SAL_BA2064AA	FL_FLDACS-35514	Caracas	0	0	0	0	0	0	0	0	0	0	0
SAL_BA1958AA	FDA00002197	Caracas	0	0	0	0	0	0	0	0	0	0	0
SAL_BA1902AA	FDA00002196	Caracas	0	0	0	0	0	0	0	0	0	0	0
SAL_BA1372AA	FDA00000704	Caracas	0	0	0	0	0	0	0	0	0	0	0
SAL_HA4370AA	FDA00002051	Caracas	0	0	0	0	0	0	0	0	0	0	0
SAL_LA2548AA	JM_03/06	Caracas	0	0	0	0	0	0	0	0	0	0	0
SAL_BA1354AA	FDA00000687	Caracas	0	0	0	0	0	0	0	0	0	0	0
SAL_BA6432AA	06-0402	Caracas	0	0	0	0	0	0	0	0	0	0	0
SAL_CA7963AA	FDA00001008	Caracas	0	0	0	0	0	0	0	0	0	0	0
SAL_DA1332AA	H130240134	Caracas	0	0	0	0	0	0	0	0	0	0	0
SAL_NA1220AA	PNUSAS029067	Caracas	0	0	0	0	0	0	0	0	0	0	0
SAL_NA2800AA	CFSAN031832	Caracas	0	0	0	0	0	0	0	0	0	0	0
SAL_NA5436AA	FDA00012489	Caracas	0	0	0	0	0	0	0	0	0	0	0
SAL_QA3795AA	376206	Caracas	0	0	0	0	0	0	0	0	0	0	0
SAL_RA7079AA	267044	Caracas	0	0	0	0	0	0	0	0	0	0	0
SAL_SA3285AA		Caracas	0	0	0	0	0	0	0	0	0	0	0
SAL_WA0157AA	206722	Caracas	0	0	0	0	0	0	0	0	0	0	0
SAL_QA7804AA	376301	Cardoner	0	0	0	0	0	0	0	0	0	0	0
SAL_DA6136AA	MOD1_Sal_845	Carmel	0	0	0	0	0	0	0	0	0	0	0
SAL_DA1361AA	H123200288	Carmel	0	0	0	0	0	0	0	0	0	0	0
SAL_CA4430AA	38730	Carmel	0	0	0	0	0	0	0	0	0	0	0
SAL_BA8575AA	23466	Carmel	0	0	0	0	0	0	0	0	0	0	0
SAL_BA6393AA	334442	Carmel	0	0	0	0	0	0	0	0	0	0	0
SAL_FA8208AA	FDA00003799	Carmel	0	0	0	0	0	0	0	0	0	0	0
SAL_EA0626AA	FSW0043	Carmel	21	16	36	68	16	0	0	0	0	50	0
SAL_FA8207AA	FDA00003800	Carmel	0	0	0	0	0	0	0	0	0	0	0
SAL_IA9434AA	PNUSAS006968	Carmel	0	0	0	0	0	0	0	0	0	0	0
SAL_MA8104AA	103630	Carmel	0	0	0	0	0	0	0	0	0	0	0
SAL_PA9623AA	279944	Carmel	0	0	0	0	0	0	0	0	0	0	0
SAL_QA0583AA	PNUSAS042224	Carmel	0	0	0	0	0	0	0	0	0	0	0
SAL_QA4799AA	465599	Carmel	0	0	0	0	0	0	0	0	0	75	0
SAL_QA5828AA	381333	Carmel	0	0	0	0	0	0	0	0	0	0	0
SAL_RA4491AA	267002	Carmel	0	0	0	0	0	0	0	0	0	0	0
SAL_HA0154AA	NCTC9872	Carmel	0	0	0	0	0	0	0	0	0	0	0
SAL_VA9364AA	278085	Carmel	0	0	0	0	0	0	0	0	0	0	0
SAL_VA4178AA	CDPH_CSalm142	Carmel	0	0	0	0	0	0	0	0	0	0	0
SAL_VA4100AA	CDPH_CSalm072	Carmel	0	0	0	0	0	0	0	0	0	0	0
SAL_WA1592AA	113028	Carmel	0	0	0	0	0	0	0	0	0	0	0
SAL_WA3305AA	707198	Carmel	0	0	0	0	0	0	0	0	0	0	0
SAL_WA3440AA	707174	Carmel	0	0	0	0	0	0	0	0	0	0	0
SAL_DA2363AA	FNW19a77	Carrau	0	0	0	0	0	0	0	0	0	0	0
SAL_IA6282AA	PNUSAS008308	Carrau	0	0	0	0	0	0	0	0	0	0	0
SAL_LA7328AA	PNUSAS022462	Carrau	0	0	0	0	0	0	0	0	0	0	0
SAL_EA0153AA	NY_IDR1000036085	Carrau	0	0	0	0	0	0	0	0	0	0	0
SAL_DA2387AA	FDA00003305	Carrau	0	0	0	0	0	0	0	0	0	0	0
SAL_BA1538AA	FDA00003393	Carrau	0	0	0	0	0	0	0	0	0	0	0
SAL_DA7796AA	FSW0160	Carrau	0	0	0	0	0	0	0	0	0	0	0
SAL_DA8657AA	FNW19H96	Carrau	0	0	0	0	0	0	0	0	0	0	0
SAL_HA3079AA	PNUSAS004857	Carrau	0	0	0	0	0	0	0	0	0	0	0
SAL_HA0300AA	PNUSAS003723	Carrau	0	0	0	0	0	0	0	0	0	0	0
SAL_JA8641AA	PNUSAS014480	Carrau	0	0	0	0	0	0	0	0	0	0	0
SAL_KA3305AA	PNUSAS015770	Carrau	0	0	0	0	0	0	0	0	0	0	0
SAL_CA9023AA	IEH-NGS-SAL-01753	Cerro	0	0	0	0	0	0	0	0	0	0	0
SAL_CA7052AA	VA-WGS-00412	Cerro	0	0	0	0	0	0	0	0	0	0	0
SAL_CA7056AA	VA-WGS-00416	Cerro	0	0	0	0	0	0	0	0	0	0	0
SAL_EA9482AA	FDA00009753	Cerro	0	0	0	0	0	0	0	0	0	0	0
SAL_EA3032AA	FSIS1502801	Cerro	0	0	0	0	0	0	0	0	0	0	0
SAL_EA3015AA	FSIS1503023	Cerro	0	0	0	0	0	0	0	0	0	0	0
SAL_FA5852AA	FDA00002817	Cerro	0	0	0	0	0	0	0	0	0	0	0
SAL_FA5448AA	FSIS1606282	Cerro	0	0	0	0	0	0	0	0	0	0	0
SAL_FA5425AA	FSIS1606287	Cerro	0	0	0	0	0	0	0	0	0	0	0
SAL_FA5418AA	FSIS1606198	Cerro	0	0	0	0	0	0	0	0	0	0	0
SAL_FA5412AA	FSIS1606189	Cerro	0	0	0	0	0	0	0	0	0	0	0
SAL_FA5062AA	PNUSA001871	Cerro	0	0	0	0	0	0	0	0	0	0	0
SAL_FA4792AA	FSIS1606123	Cerro	0	0	0	0	0	0	0	0	0	0	0
SAL_FA4055AA	FSIS1606127	Cerro	0	0	0	0	0	0	0	0	0	0	0
SAL_FA4049AA	FSIS1606175	Cerro	0	0	0	0	0	0	0	0	0	0	0
SAL_FA3259AA	FSIS1606029	Cerro	0	0	0	0	0	0	0	0	0	0	0
SAL_FA2778AA	CVM N57633F	Cerro	0	0	0	0	0	0	0	0	0	0	0
SAL_FA2461AA	FSIS1605975	Cerro	0	0	0	0	0	0	0	0	0	0	0

SAL_FA2207AA	FSIS1605825	Cerro	0	0	0	0	0	0	0	0	0	0	0
SAL_FA2203AA	FSIS1605826	Cerro	0	0	0	0	0	0	0	0	0	0	0
SAL_FA2197AA	FSIS1605914	Cerro	0	0	0	0	0	0	0	0	0	0	0
SAL_FA2159AA	FSIS1605836	Cerro	0	0	0	0	0	0	0	0	0	0	0
SAL_FA1709AA	CFSAN046165	Cerro	0	0	0	0	0	0	0	0	0	0	0
SAL_FA1324AA	FSIS1605758	Cerro	0	0	0	0	0	0	0	0	0	0	0
SAL_FA1169AA	ADRDL-12069-12-1-1A	Cerro	0	0	0	0	0	0	0	0	0	0	0
SAL_FA1171AA	ADRDL-5442-12-1-1A	Cerro	0	0	0	0	0	0	0	0	0	0	0
SAL_FA1170AA	ADRDL-39020-08-1-6A	Cerro	0	0	0	0	0	0	0	0	0	0	0
SAL_EA9876AA	FSIS1605429	Cerro	0	0	0	0	0	0	0	0	0	0	0
SAL_EA9571AA	FSIS1605654	Cerro	0	0	0	0	0	0	0	0	0	0	0
SAL_EA9237AA	FDA00009754	Cerro	0	0	0	0	0	0	0	0	0	0	0
SAL_EA8795AA	FSIS1504957	Cerro	0	0	0	0	0	0	0	0	0	0	0
SAL_EA8665AA	NY-N19959	Cerro	0	0	0	0	0	0	0	0	0	0	0
SAL_EA8377AA	FSIS1504601	Cerro	0	0	0	0	0	0	0	0	0	0	0
SAL_EA6902AA	77329	Cerro	0	0	0	0	0	0	0	0	0	0	0
SAL_EA6339AA	FSIS1505155	Cerro	0	0	0	0	0	0	0	0	0	0	0
SAL_EA6334AA	FSIS1505158	Cerro	0	0	0	0	0	0	0	0	0	0	0
SAL_EA6221AA	FSIS1504874	Cerro	0	0	0	0	0	0	0	0	0	0	0
SAL_EA6177AA	ADRDL-15-4131	Cerro	0	0	0	0	0	0	0	0	0	0	0
SAL_EA6175AA	ADRDL-15-11026	Cerro	0	0	0	0	0	0	0	0	0	0	0
SAL_EA6172AA	ADRDL-15-7702	Cerro	0	0	0	0	0	0	0	0	0	0	0
SAL_EA6134AA	FSIS1501292	Cerro	0	0	0	0	0	0	0	0	0	0	0
SAL_EA6129AA	FSIS1501934	Cerro	0	0	0	0	0	0	0	0	0	0	0
SAL_EA5956AA	ADRDL-15-1225	Cerro	0	0	0	0	0	0	0	0	0	0	0
SAL_EA5501AA	FSIS1504928	Cerro	0	0	0	0	0	0	0	0	0	0	0
SAL_EA5351AA	FSIS1503810	Cerro	0	0	0	0	0	0	0	0	0	0	0
SAL_EA4964AA	ADRDL-14-20151	Cerro	0	0	0	0	0	0	0	0	0	0	0
SAL_EA4037AA	FDA00009407	Cerro	0	0	0	0	0	0	0	0	0	0	0
SAL_EA3949AA	FSIS1504697	Cerro	0	0	0	0	0	0	0	0	0	0	0
SAL_EA3046AA	FSIS1502799	Cerro	0	0	0	0	0	0	0	0	0	0	0
SAL_EA2856AA	FDA00000347	Cerro	0	0	0	0	0	0	0	0	0	0	0
SAL_EA2726AA	FDA00001917	Cerro	0	0	0	0	0	0	0	0	0	0	0
SAL_EA2725AA	FDA00001918	Cerro	0	0	0	0	0	0	0	0	0	0	0
SAL_EA2513AA	ADRDL-LA-72-2014	Cerro	0	0	0	0	0	0	0	0	0	0	0
SAL_EA2491AA	ADRDL-LA-95-2014	Cerro	0	0	0	0	0	0	0	0	0	0	0
SAL_EA2496AA	ADRDL-LA-89-2014	Cerro	0	0	0	0	0	0	0	0	0	0	0
SAL_EA2484AA	ADRDL-LA-34-2014	Cerro	0	0	0	0	0	0	0	0	0	0	0
SAL_EA2009AA	CVM N53838	Cerro	0	0	0	0	0	0	0	0	0	0	0
SAL_EA1853AA	FSIS1503793	Cerro	0	0	0	0	0	0	0	0	0	0	0
SAL_EA1482AA	FSIS1500646	Cerro	0	0	0	0	0	0	0	0	0	0	0
SAL_EA1459AA	FSIS1500730	Cerro	0	0	0	0	0	0	0	0	0	0	0
SAL_EA1468AA	FSIS1500718	Cerro	0	0	0	0	0	0	0	0	0	0	0
SAL_EA1461AA	FSIS1500717	Cerro	0	0	0	0	0	0	0	0	0	0	0
SAL_EA1426AA	FSIS1500994	Cerro	0	0	0	0	0	0	0	0	0	0	0
SAL_EA1413AA	NY_FSL S10-1809	Cerro	0	0	0	0	0	0	0	0	0	0	0
SAL_EA1360AA	NY_FSL S10-1823	Cerro	0	0	0	0	0	0	0	0	0	0	0
SAL_EA0532AA	FDN0067	Cerro	0	0	0	0	0	0	0	0	0	0	0
SAL_EA0528AA	FDN0063	Cerro	0	0	0	0	0	0	0	0	0	0	0
SAL_EA0070AA	NY_IDR1200021873-01	Cerro	0	0	0	0	0	0	0	0	0	0	0
SAL_DA9905AA	FMA0023	Cerro	0	0	0	0	0	0	0	0	0	0	0
SAL_DA9652AA	FSF0073	Cerro	0	0	0	0	0	0	0	0	0	0	0
SAL_DA8574AA	VA_WGS-00139	Cerro	0	0	0	0	0	0	0	0	0	0	0
SAL_DA8227AA	3205-sc-2010-09-29-985	Cerro	0	0	0	0	0	0	0	0	0	0	0
SAL_DA6833AA	VA-WGS-00254	Cerro	0	0	0	0	0	0	0	0	0	0	0
SAL_DA6510AA	VA-WGS-00262	Cerro	0	0	0	0	0	0	0	0	0	0	0
SAL_DA6512AA	VA-WGS-00266	Cerro	0	0	0	0	0	0	0	0	0	0	0
SAL_DA6409AA	NY-FSL-R8-1631	Cerro	0	0	0	0	0	0	0	0	0	0	0
SAL_DA6195AA	NY-FSL-R8-1632	Cerro	0	0	0	0	0	0	0	0	0	0	0
SAL_DA6189AA	NY-FSL-R8-2026	Cerro	0	0	0	0	0	0	0	0	0	0	0
SAL_DA6172AA	NY-FSL-R8-2818	Cerro	0	0	0	0	0	0	0	0	0	0	0
SAL_DA6090AA	MOD1_Sal_846	Cerro	0	0	0	0	0	0	0	0	0	0	0
SAL_DA5987AA	CFSAN018460	Cerro	0	0	0	0	0	0	0	0	0	0	0
SAL_DA5961AA	CFSAN018445	Cerro	0	0	0	0	0	0	0	0	0	0	0
SAL_DA5691AA	NY-FSL-R8-2852	Cerro	0	0	0	0	0	0	0	0	0	0	0
SAL_DA5684AA	NY-FSL-R8-3412	Cerro	0	0	0	0	0	0	0	0	0	0	0
SAL_DA5660AA	NY-FSL-R8-4030	Cerro	0	0	0	0	0	0	0	0	0	0	0
SAL_DA5665AA	NY-FSL-R8-3138	Cerro	0	0	0	0	0	0	0	0	0	0	0
SAL_DA5642AA	NY-FSL-R8-3445	Cerro	0	0	0	0	0	0	0	0	0	0	0
SAL_DA5641AA	NY-FSL-R8-3664	Cerro	0	0	0	0	0	0	0	0	0	0	0
SAL_DA5639AA	NY-FSL-R8-4025	Cerro	0	0	0	0	0	0	0	0	0	0	0
SAL_DA5581AA	CFSAN018682	Cerro	0	0	0	0	0	0	0	0	0	0	0
SAL_DA5535AA	CFSAN018718	Cerro	0	0	0	0	0	0	0	0	0	0	0

SAL_DA5520AA	IEH-NGS-SAL-00882	Cerro	0	0	0	0	0	0	0	0	0	0	0
SAL_DA5272AA	FDA00000223	Cerro	0	0	0	0	0	0	0	0	0	0	0
SAL_DA4104AA	VA-WGS-00263	Cerro	0	0	0	0	0	0	0	0	0	0	0
SAL_DA3972AA	IEH-NGS-SAL-01158	Cerro	0	0	0	0	0	0	0	0	0	0	0
SAL_DA2151AA	FDN0065	Cerro	0	0	0	0	0	0	0	0	0	0	0
SAL_DA1390AA	H131140776	Cerro	0	0	0	0	0	0	0	0	0	0	0
SAL_DA0671AA	H133140592	Cerro	0	0	0	0	0	0	0	0	0	0	0
SAL_DA0468AA	H132820455	Cerro	0	0	0	0	0	0	0	0	0	0	0
SAL_DA0123AA	FDA00003642	Cerro	0	0	0	0	0	0	0	0	0	0	0
SAL_DA0064AA	FDA00003641	Cerro	0	0	0	0	0	0	0	0	0	0	0
SAL_CA9792AA	FDA00003686	Cerro	0	0	0	0	0	0	0	0	0	0	0
SAL_CA9574AA	IEH-NGS-SAL-01641	Cerro	0	0	0	0	0	0	0	0	0	0	0
SAL_CA9575AA	IEH-NGS-SAL-01637	Cerro	0	0	0	0	0	0	0	0	0	0	0
SAL_CA9567AA	IEH-NGS-SAL-01639	Cerro	0	0	0	0	0	0	0	0	0	0	0
SAL_CA9563AA	CFSAN027114	Cerro	0	0	0	0	0	0	0	0	0	0	0
SAL_CA9364AA	VA-WGS-00363	Cerro	0	0	0	0	0	0	0	0	0	0	0
SAL_CA9267AA	CFSAN025701	Cerro	0	0	0	0	0	0	0	0	0	0	0
SAL_CA9128AA	IEH-NGS-SAL-01782	Cerro	0	0	0	0	0	0	0	0	0	0	0
SAL_CA9115AA	CFSAN027470	Cerro	0	0	0	0	0	0	0	0	0	0	0
SAL_CA9120AA	CFSAN027549	Cerro	0	0	0	0	0	0	0	0	0	0	0
SAL_CA9111AA	CFSAN027471	Cerro	0	0	0	0	0	0	0	0	0	0	0
SAL_CA9085AA	IEH-NGS-SAL-01769	Cerro	0	0	0	0	0	0	0	0	0	0	0
SAL_CA9024AA	IEH-NGS-SAL-01752	Cerro	0	0	0	0	0	0	0	0	0	0	0
SAL_CA8875AA	CFSAN025709	Cerro	0	0	0	0	0	0	0	0	0	0	0
SAL_CA8874AA	CFSAN024804	Cerro	0	0	0	0	0	0	0	0	0	0	0
SAL_CA8626AA	IEH-NGS-SAL-01825	Cerro	0	0	0	0	0	0	0	0	0	0	0
SAL_CA8292AA	FDA00008278	Cerro	0	0	0	0	0	0	0	0	0	0	0
SAL_CA8173AA	FDA00008788	Cerro	0	0	0	0	0	0	0	0	0	0	0
SAL_CA8160AA	FDA00008787	Cerro	0	0	0	0	0	0	0	0	0	0	0
SAL_CA7130AA	VA-WGS-00410	Cerro	0	0	0	0	0	0	0	0	0	0	0
SAL_CA7049AA	VA-WGS-00413	Cerro	0	0	0	0	0	0	0	0	0	0	0
SAL_CA4612AA	42159	Cerro	0	0	0	0	0	0	0	0	0	0	0
SAL_CA4552AA	23463	Cerro	0	0	0	0	0	0	0	0	0	0	0
SAL_CA2764AA	14100	Cerro	0	0	0	0	0	0	0	0	0	0	0
SAL_CA2566AA	42158	Cerro	0	0	0	0	0	0	0	0	0	0	0
SAL_CA0906AA	23517	Cerro	0	0	0	0	0	0	0	0	0	0	0
SAL_BA9890AA	63619	Cerro	0	0	0	0	0	0	0	0	0	0	0
SAL_BA8902AA	NY_FSL P3-592	Cerro	0	0	0	0	0	0	0	0	0	0	0
SAL_BA8906AA	NY_FSL S10-228	Cerro	0	0	0	0	0	0	0	0	0	0	0
SAL_BA8900AA	NY_FSL S10-545	Cerro	0	0	0	0	0	0	0	0	0	0	0
SAL_BA8886AA	NY_FSL S10-732	Cerro	0	0	0	0	0	0	0	0	0	0	0
SAL_BA8703AA	NY_FSL S10-861	Cerro	0	0	0	0	0	0	0	0	0	0	0
SAL_BA8699AA	NY_FSL P3-1548	Cerro	0	0	0	0	0	0	0	0	0	0	0
SAL_BA8705AA	NY_FSL P3-1140	Cerro	0	0	0	0	0	0	0	0	0	0	0
SAL_BA8697AA	NY_FSL P3-1550	Cerro	0	0	0	0	0	0	0	0	0	0	0
SAL_BA8616AA	IEH_NGS_SAL_00233	Cerro	0	0	0	0	0	0	0	0	0	0	0
SAL_BA7849AA	27057	Cerro	0	0	0	0	0	0	0	0	0	0	0
SAL_BA5098AA	CFSAN005658	Cerro	0	0	0	0	0	0	0	0	0	0	0
SAL_BA4508AA	FDA00004490	Cerro	0	0	0	0	0	0	0	0	0	0	0
SAL_BA3033AA	NY_FSL S10-1411	Cerro	0	0	0	0	0	0	0	0	0	0	0
SAL_BA2996AA	NY_FSL S10-1741	Cerro	0	0	0	0	0	0	0	0	0	0	0
SAL_BA2760AA	CFSAN033528	Cerro	0	0	0	0	0	0	0	0	0	0	0
SAL_BA2614AA	ADRDL-14-15211	Cerro	0	0	0	0	0	0	0	0	0	0	0
SAL_BA2597AA	ADRDL-14-15156	Cerro	0	0	0	0	0	0	0	0	0	0	0
SAL_BA2161AA	CFSAN034147	Cerro	0	0	0	0	0	0	0	0	0	0	0
SAL_BA2144AA	NY_FSL S10-1742	Cerro	0	0	0	0	0	0	0	0	0	0	0
SAL_BA1961AA	ADRDL-14-20516	Cerro	0	0	0	0	0	0	0	0	0	0	0
SAL_BA1684AA	CFSAN034224	Cerro	0	0	0	0	0	0	0	0	0	0	0
SAL_BA1667AA	FDA00003337	Cerro	0	0	0	0	0	0	0	0	0	0	0
SAL_BA1654AA	CFSAN034222	Cerro	0	0	0	0	0	0	0	0	0	0	0
SAL_BA1458AA	ADRDL-LA-4-2014	Cerro	0	0	0	0	0	0	0	0	0	0	0
SAL_BA1414AA	ADRDL-LA-2-2014	Cerro	0	0	0	0	0	0	0	0	0	0	0
SAL_BA1401AA	ADRDL-LA-14-2014	Cerro	0	0	0	0	0	0	0	0	0	0	0
SAL_BA1380AA	ADRDL-LA-15-2014	Cerro	0	0	0	0	0	0	0	0	0	0	0
SAL_BA1310AA	CVM-N14793	Cerro	0	0	0	0	0	0	0	0	0	0	0
SAL_BA1267AA	ADRDL-LA-54-2014	Cerro	0	0	0	0	0	0	0	0	0	0	0
SAL_BA1265AA	ADRDL-LA-53-2014	Cerro	0	0	0	0	0	0	0	0	0	0	0
SAL_BA0786AA	FDA00009318	Cerro	0	0	0	0	0	0	0	0	0	0	0
SAL_FA6157AA	IEH-NGS-SAL-04357	Cerro	0	0	0	0	0	0	0	0	0	0	0
SAL_FA6173AA	FSIS1606401	Cerro	0	0	0	0	0	0	0	0	0	0	0
SAL_FA6334AA	FSIS1606505	Cerro	0	0	0	0	0	0	0	0	0	0	0
SAL_FA6303AA	FSIS1606514	Cerro	0	0	0	0	0	0	0	0	0	0	0
SAL_FA6298AA	FSIS1606517	Cerro	0	0	0	0	0	0	0	0	0	0	0

SAL_FA6931AA	FSIS1606632	Cerro	0	0	0	0	0	0	0	0	0	0	0
SAL_FA7417AA	ADRDL15-22922	Cerro	0	0	0	0	0	0	0	0	0	0	0
SAL_FA7578AA	FSIS1606700	Cerro	0	0	0	0	0	0	0	0	0	0	0
SAL_FA7577AA	FSIS1606702	Cerro	0	0	0	0	0	0	0	0	0	0	0
SAL_FA7898AA	FSIS1606771	Cerro	0	0	0	0	0	0	0	0	0	0	0
SAL_FA7895AA	FSIS1606774	Cerro	0	0	0	0	0	0	0	0	0	0	0
SAL_FA7894AA	FSIS1606777	Cerro	0	0	0	0	0	0	0	0	0	0	0
SAL_FA8142AA	FSIS1606880	Cerro	0	0	0	0	0	0	0	0	0	0	0
SAL_FA8138AA	FSIS1606890	Cerro	0	0	0	0	0	0	0	0	0	0	0
SAL_FA8248AA	FSIS1606927	Cerro	0	0	0	0	0	0	0	0	0	0	0
SAL_FA8234AA	FSIS1606955	Cerro	0	0	0	0	0	0	0	0	0	0	0
SAL_FA7208AA	PNUSAS002073	Cerro	0	0	0	0	0	0	0	0	0	0	0
SAL_GA2776AA	CRJJGF_00136	Cerro	0	0	0	0	0	0	0	0	0	0	0
SAL_GA2629AA	2010K-1259	Cerro	0	0	0	0	0	0	0	0	0	0	0
SAL_GA2623AA	99-0449	Cerro	0	0	0	0	0	0	0	0	0	0	0
SAL_GA2610AA	77-3451	Cerro	0	0	0	0	0	0	0	0	0	0	0
SAL_GA4336AA	PNUSAS002642	Cerro	0	0	0	0	0	0	0	0	0	0	0
SAL_GA3836AA	BCW_1607	Cerro	0	0	0	0	0	0	0	0	0	0	0
SAL_GA3348AA	BCW_2654	Cerro	0	0	0	0	0	0	0	0	0	0	0
SAL_GA3347AA	BCW_2655	Cerro	0	0	0	0	0	0	0	0	0	0	0
SAL_GA2870AA	CRJJGF_00041	Cerro	0	0	0	0	0	0	0	0	0	0	0
SAL_GA2437AA	BCW_1978	Cerro	0	0	0	0	0	0	0	0	0	0	0
SAL_GA2426AA	BCW_1989	Cerro	0	0	0	0	0	0	0	0	0	0	0
SAL_GA2418AA	BCW_1997	Cerro	0	0	0	0	0	0	0	0	0	0	0
SAL_GA2224AA	BCW_6062	Cerro	0	0	0	0	0	0	0	0	0	0	0
SAL_GA2126AA	BCW_6505	Cerro	0	0	0	0	0	0	0	0	0	0	0
SAL_GA3897AA	BCW_2849	Cerro	0	0	0	0	0	0	0	0	0	0	0
SAL_GA0347AA	VNS004_D10-sc-2280618	Cerro	0	0	0	0	0	0	0	0	0	0	0
SAL_FA9727AA	FDA00001800	Cerro	0	0	0	0	0	0	0	0	0	0	0
SAL_GA4723AA	FSIS1606970	Cerro	0	0	0	0	0	0	0	0	0	0	0
SAL_GA4719AA	FSIS1606973	Cerro	0	0	0	0	0	0	0	0	0	0	0
SAL_GA4695AA	FSIS1606976	Cerro	0	0	0	0	0	0	0	0	0	0	0
SAL_GA5177AA	V-CLASP-D-43	Cerro	0	0	0	0	0	0	0	0	0	0	0
SAL_GA5462AA	CFSAN042338	Cerro	0	0	0	0	0	0	0	0	0	0	0
SAL_GA5443AA	CFSAN042340	Cerro	0	0	0	0	0	0	0	0	0	0	0
SAL_GA5423AA	CFSAN042347	Cerro	0	0	0	0	0	0	0	0	0	0	0
SAL_GA5424AA	CFSAN042346	Cerro	0	0	0	0	0	0	0	0	0	0	0
SAL_GA5420AA	CFSAN042350	Cerro	0	0	0	0	0	0	0	0	0	0	0
SAL_GA5418AA	CFSAN042352	Cerro	0	0	0	0	0	0	0	0	0	0	0
SAL_GA5419AA	CFSAN042351	Cerro	0	0	0	0	0	0	0	0	0	0	0
SAL_GA5408AA	CFSAN042355	Cerro	0	0	0	0	0	0	0	0	0	0	0
SAL_GA5352AA	CFSAN042857	Cerro	0	0	0	0	0	0	0	0	0	0	0
SAL_GA5337AA	CFSAN042872	Cerro	0	0	0	0	0	0	0	0	0	0	0
SAL_GA5338AA	CFSAN042871	Cerro	0	0	0	0	0	0	0	0	0	0	0
SAL_GA5335AA	CFSAN042874	Cerro	0	0	0	0	0	0	0	0	0	0	0
SAL_GA5272AA	CFSAN042937	Cerro	0	0	0	0	0	0	0	0	0	0	0
SAL_GA5271AA	CFSAN042938	Cerro	0	0	0	0	0	0	0	0	0	0	0
SAL_GA5269AA	CFSAN042940	Cerro	0	0	0	0	0	0	0	0	0	0	0
SAL_GA5265AA	CFSAN042941	Cerro	0	0	0	0	0	0	0	0	0	0	0
SAL_FA9029AA	CVM N40926	Cerro	0	0	0	0	0	0	0	0	0	0	0
SAL_FA8875AA	CVM N42506	Cerro	0	0	0	0	0	0	0	0	0	0	0
SAL_GA5421AA	CFSAN042349	Cerro	0	0	0	0	0	0	0	0	0	0	0
SAL_GA5422AA	CFSAN042348	Cerro	0	0	0	0	0	0	0	0	0	0	0
SAL_GA6029AA	FDA00001681	Cerro	0	0	0	0	0	0	0	0	0	0	0
SAL_GA5945AA	FSIS1607158	Cerro	0	0	0	0	0	0	0	0	0	0	0
SAL_GA5941AA	FSIS1607156	Cerro	0	0	0	0	0	0	0	0	0	0	0
SAL_GA6061AA	FSIS1607189	Cerro	0	0	0	0	0	0	0	0	0	0	0
SAL_GA6319AA	FSIS1607207	Cerro	0	0	0	0	0	0	0	0	0	0	0
SAL_GA7772AA	FSIS1607335	Cerro	0	0	0	0	0	0	0	0	0	0	0
SAL_GA8034AA	FSIS1607403	Cerro	0	0	0	0	0	0	0	0	0	0	0
SAL_GA8449AA	FSIS1607449	Cerro	0	0	0	0	0	0	0	0	0	0	0
SAL_GA8440AA	FSIS1607443	Cerro	0	0	0	0	0	0	0	0	0	0	0
SAL_GA8491AA	TX-ACF1601730	Cerro	0	0	0	0	0	0	0	0	0	0	0
SAL_GA8591AA	FDA00010559	Cerro	0	0	0	0	0	0	0	0	0	0	0
SAL_GA8720AA	FSIS1607496	Cerro	0	0	0	0	0	0	0	0	0	0	0
SAL_GA8719AA	FSIS1607498	Cerro	0	0	0	0	0	0	0	0	0	0	0
SAL_GA8733AA	FSIS1607502	Cerro	0	0	0	0	0	0	0	0	0	0	0
SAL_GA8851AA	OH-1011898	Cerro	0	0	0	0	0	0	0	0	0	0	0
SAL_GA9314AA	FSIS1607549	Cerro	0	0	0	0	0	0	0	0	0	0	0
SAL_GA9596AA	FSIS1607712	Cerro	0	0	0	0	0	0	0	0	0	0	0
SAL_GA9464AA	FSIS1607645	Cerro	0	0	0	0	0	0	0	0	0	0	0
SAL_GA9773AA	NYVetLIRN-5	Cerro	0	0	0	0	0	0	0	0	0	0	0
SAL_GA9767AA	NYVetLIRN-11	Cerro	0	0	0	0	0	0	0	0	0	0	0

SAL_GA9703AA	CFSAN042948	Cerro	0	0	0	0	0	0	0	0	0	0	0
SAL_GA9704AA	CFSAN042947	Cerro	0	0	0	0	0	0	0	0	0	0	0
SAL_GA9701AA	CFSAN042950	Cerro	0	0	0	0	0	0	0	0	0	0	0
SAL_GA9712AA	CFSAN039246	Cerro	0	0	0	0	0	0	0	0	0	0	0
SAL_GA9705AA	CFSAN042946	Cerro	0	0	0	0	0	0	0	0	0	0	0
SAL_HA0106AA	FDA00004953	Cerro	0	0	0	0	0	0	0	0	0	0	0
SAL_HA0415AA	CFSAN025426	Cerro	0	0	0	0	0	0	0	0	0	0	0
SAL_HA0412AA	CFSAN025431	Cerro	0	0	0	0	0	0	0	0	0	0	0
SAL_HA0413AA	CFSAN025430	Cerro	0	0	0	0	0	0	0	0	0	0	0
SAL_HA0894AA	PNUSAS004251	Cerro	0	0	0	0	0	0	0	0	0	0	0
SAL_HA0808AA	CFSAN048188	Cerro	0	0	0	0	0	0	0	0	0	0	0
SAL_HA0804AA	FSIS1607793	Cerro	0	0	0	0	0	0	0	0	0	0	0
SAL_HA0827AA	FSIS1607799	Cerro	0	0	0	0	0	0	0	0	0	0	0
SAL_HA0802AA	FSIS1607783	Cerro	0	0	0	0	0	0	0	0	0	0	0
SAL_HA0803AA	FSIS1607792	Cerro	0	0	0	0	0	0	0	0	0	0	0
SAL_HA0823AA	FSIS1607800	Cerro	0	0	0	0	0	0	0	0	0	0	0
SAL_HA0829AA	FSIS1607820	Cerro	0	0	0	0	0	0	0	0	0	0	0
SAL_HA0799AA	FSIS1607785	Cerro	0	0	0	0	0	0	0	0	0	0	0
SAL_HA0721AA	FSIS1607811	Cerro	0	0	0	0	0	0	0	0	0	0	0
SAL_HA1272AA	FSIS1607867	Cerro	0	0	0	0	0	0	0	0	0	0	0
SAL_HA1314AA	FSIS1607962	Cerro	0	0	0	0	0	0	0	0	0	0	0
SAL_GA9711AA	CFSAN039247	Cerro	0	0	0	0	0	0	0	0	0	0	0
SAL_GA9702AA	CFSAN042949	Cerro	0	0	0	0	0	0	0	0	0	0	0
SAL_HA1312AA	FSIS1607956	Cerro	0	0	0	0	0	0	0	0	0	0	0
SAL_HA2922AA	FSIS1608113	Cerro	0	0	0	0	0	0	0	0	0	0	0
SAL_HA2933AA	FSIS1608146	Cerro	0	0	0	0	0	0	0	0	0	0	0
SAL_HA2823AA	FSIS1608117	Cerro	0	0	0	0	0	0	0	0	0	0	0
SAL_HA2760AA	CFSAN048259	Cerro	0	0	0	0	0	0	0	0	0	0	0
SAL_HA4068AA	PNUSAS005213	Cerro	0	0	0	0	0	0	0	0	0	0	0
SAL_HA4751AA	FSIS1608388	Cerro	0	0	0	0	0	0	0	0	0	0	0
SAL_HA4732AA	FSIS1608551	Cerro	0	0	0	0	0	0	0	0	0	0	0
SAL_HA4926AA	11-0573	Cerro	0	0	0	0	0	0	0	0	0	0	0
SAL_HA5195AA	12-0352	Cerro	0	0	0	0	0	0	0	0	0	0	0
SAL_HA5159AA	2123096	Cerro	0	0	0	0	0	0	0	0	0	0	0
SAL_HA5110AA	L01666-14	Cerro	0	0	0	0	0	0	0	0	0	0	0
SAL_HA5122AA	L00796-14	Cerro	0	0	0	0	0	0	0	0	0	0	0
SAL_HA5078AA	S05892-14	Cerro	0	0	0	0	0	0	0	0	0	0	0
SAL_HA5079AA	S05891-14	Cerro	0	0	0	0	0	0	0	0	0	0	0
SAL_HA5084AA	S04603-14	Cerro	0	0	0	0	0	0	0	0	0	0	0
SAL_HA4975AA	13-0670	Cerro	0	0	0	0	0	0	0	0	0	0	0
SAL_HA5487AA	CFSAN056379	Cerro	0	0	0	0	0	0	0	0	0	0	0
SAL_HA5500AA	CFSAN056562	Cerro	0	0	0	0	0	0	0	0	0	0	0
SAL_HA5622AA	L01052-16	Cerro	0	0	0	0	0	0	0	0	0	0	0
SAL_HA5638AA	S02664-15	Cerro	0	0	0	0	0	0	0	0	0	0	0
SAL_HA6468AA	FSIS1609105	Cerro	0	0	0	0	0	0	0	0	0	0	0
SAL_HA3994AA	FSIS1608357	Cerro	0	0	0	0	0	0	0	0	0	0	0
SAL_HA5668AA	L01181-15	Cerro	0	0	0	0	0	0	0	0	0	0	0
SAL_HA5666AA	L01641-15	Cerro	0	0	0	0	0	0	0	0	0	0	0
SAL_HA5660AA	L02584-15	Cerro	0	0	0	0	0	0	0	0	0	0	0
SAL_HA5980AA	CFSAN056590	Cerro	0	0	0	0	0	0	0	0	0	0	0
SAL_HA6031AA	CFSAN056629	Cerro	0	0	0	0	0	0	0	0	0	0	0
SAL_HA6092AA	CFSAN056612	Cerro	0	0	0	0	0	0	0	0	0	0	0
SAL_HA6091AA	CFSAN056613	Cerro	0	0	0	0	0	0	0	0	0	0	0
SAL_HA6088AA	CFSAN056616	Cerro	0	0	0	0	0	0	0	0	0	0	0
SAL_HA6938AA	FSIS1608898	Cerro	0	0	0	0	0	0	0	0	0	0	0
SAL_HA6111AA	FSIS1608679	Cerro	0	0	0	0	0	0	0	0	0	0	0
SAL_HA6966AA	CFSAN056787	Cerro	0	0	0	0	0	0	0	0	0	0	0
SAL_HA6967AA	CFSAN056788	Cerro	0	0	0	0	0	0	0	0	0	0	0
SAL_HA7181AA	CFSAN056779	Cerro	0	0	0	0	0	0	0	0	0	0	0
SAL_HA7190AA	CFSAN056783	Cerro	0	0	0	0	0	0	0	0	0	0	0
SAL_HA7193AA	CFSAN056785	Cerro	0	0	0	0	0	0	0	0	0	0	0
SAL_HA7194AA	CFSAN056780	Cerro	0	0	0	0	0	0	0	0	0	0	0
SAL_HA7240AA	5_PRJ1050_Salmonella_c	Cerro	0	0	0	0	0	0	0	0	0	0	0
SAL_HA6276AA	PNUSAS004339	Cerro	0	0	0	0	0	0	0	0	0	0	0
SAL_HA7703AA	FSIS1609250	Cerro	0	0	0	0	0	0	0	0	0	0	0
SAL_HA7683AA	FSIS1609114	Cerro	0	0	0	0	0	0	0	0	0	0	0
SAL_HA7765AA	FSIS1608890	Cerro	0	0	0	0	0	0	0	0	0	0	0
SAL_HA7768AA	FSIS1608885	Cerro	0	0	0	0	0	0	0	0	0	0	0
SAL_HA8808AA	FSIS1609409	Cerro	0	0	0	0	0	0	0	0	0	0	0
SAL_HA8819AA	FSIS1609551	Cerro	0	0	0	0	0	0	0	0	0	0	0
SAL_HA8809AA	FSIS1609554	Cerro	0	0	0	0	0	0	0	0	0	0	0
SAL_HA8804AA	FSIS1609407	Cerro	0	0	0	0	0	0	0	0	0	0	0
SAL_HA8823AA	FSIS1609408	Cerro	0	0	0	0	0	0	0	0	0	0	0

SAL_HA8815AA	FSIS1609410	Cerro	0	0	0	0	0	0	0	0	0	0	0
SAL_HA9219AA	PNUSAS006709	Cerro	0	0	0	0	0	0	0	0	0	0	0
SAL_HA9100AA	FSIS1609223	Cerro	0	0	0	0	0	0	0	0	0	0	0
SAL_HA9556AA	PNUSAS006356	Cerro	0	0	0	0	0	0	0	0	0	0	0
SAL_HA9540AA	PNUSAS006805	Cerro	0	0	0	0	0	0	0	0	0	0	0
SAL_HA9674AA	PNUSAS006907	Cerro	0	0	0	0	0	0	0	0	0	0	0
SAL_HA9646AA	ADRDL-797	Cerro	0	0	0	0	0	0	0	0	0	0	0
SAL_HA9871AA	FSIS1609688	Cerro	0	0	0	0	0	0	0	0	0	0	0
SAL_IA0303AA	FDA00004405	Cerro	0	0	0	0	0	0	0	0	0	0	0
SAL_IA2009AA	FSIS1609727	Cerro	0	0	0	0	0	0	0	0	0	0	0
SAL_IA2344AA	FSIS1709854	Cerro	0	0	0	0	0	0	0	0	0	0	0
SAL_IA2373AA	FSIS1609392	Cerro	0	0	0	0	0	0	0	0	0	0	0
SAL_IA2375AA	FSIS1609393	Cerro	0	0	0	0	0	0	0	0	0	0	0
SAL_IA2371AA	FSIS1609396	Cerro	0	0	0	0	0	0	0	0	0	0	0
SAL_IA2378AA	FSIS1609389	Cerro	0	0	0	0	0	0	0	0	0	0	0
SAL_IA2705AA	CFSAN059258	Cerro	0	0	0	0	0	0	0	0	0	0	0
SAL_IA3700AA	FSIS1710102	Cerro	0	0	0	0	0	0	0	0	0	0	0
SAL_IA4944AA	OH-16-23862	Cerro	0	0	0	0	0	0	0	0	0	0	0
SAL_IA5154AA	OH-16-27627	Cerro	0	0	0	0	0	0	0	0	0	0	0
SAL_IA5143AA	OH-16-27138	Cerro	0	0	0	0	0	0	0	0	0	0	0
SAL_IA4950AA	OH-16-24779	Cerro	0	0	0	0	0	0	0	0	0	0	0
SAL_IA5352AA	FSIS1710083	Cerro	0	0	0	0	0	0	0	0	0	0	0
SAL_IA5332AA	OH-17-509-3	Cerro	0	0	0	0	0	0	0	0	0	0	0
SAL_IA5333AA	FSIS1710093	Cerro	0	0	0	0	0	0	0	0	0	0	0
SAL_IA5949AA	MS160353	Cerro	0	0	0	0	0	0	0	0	0	0	0
SAL_IA6336AA	FSIS1709966	Cerro	0	0	0	0	0	0	0	0	0	0	0
SAL_IA5502AA	CFSAN059805	Cerro	0	0	0	0	0	0	0	0	0	0	0
SAL_IA5436AA	PNUSAS008453	Cerro	0	0	0	0	0	0	0	0	0	0	0
SAL_IA5423AA	PNUSAS008458	Cerro	0	0	0	0	0	0	0	0	0	0	0
SAL_IA7466AA	PNUSAS009096	Cerro	0	0	0	0	0	0	0	0	0	0	0
SAL_IA7443AA	OH-16-2178	Cerro	0	0	0	0	0	0	0	0	0	0	0
SAL_IA7442AA	OH-16-2176	Cerro	0	0	0	0	0	0	0	0	0	0	0
SAL_IA7417AA	OH-17-500	Cerro	0	0	0	0	0	0	0	0	0	0	0
SAL_IA7400AA	OH-17-867A	Cerro	0	0	0	0	0	0	0	0	0	0	0
SAL_IA7406AA	OH-17-877	Cerro	0	0	0	0	0	0	0	0	0	0	0
SAL_IA7398AA	OH-17-628	Cerro	0	0	0	0	0	0	0	0	0	0	0
SAL_IA7544AA	FSIS1710545	Cerro	0	0	0	0	0	0	0	0	0	0	0
SAL_IA7580AA	PNUSAS009137	Cerro	0	0	0	0	0	0	0	0	0	0	0
SAL_IA7780AA	OH-16-3963-1	Cerro	0	0	0	0	0	0	0	0	0	0	0
SAL_IA8231AA	PNUSAS008872	Cerro	0	0	0	0	0	0	0	0	0	0	0
SAL_IA8992AA	FSIS1710843	Cerro	0	0	0	0	0	0	0	0	0	0	0
SAL_IA8991AA	FSIS1710842	Cerro	0	0	0	0	0	0	0	0	0	0	0
SAL_IA9008AA	FSIS1710683	Cerro	0	0	0	0	0	0	0	0	0	0	0
SAL_IA8987AA	FSIS1710859	Cerro	0	0	0	0	0	0	0	0	0	0	0
SAL_IA8968AA	ADRDL-906	Cerro	0	0	0	0	0	0	0	0	0	0	0
SAL_IA9007AA	FSIS1710855	Cerro	0	0	0	0	0	0	0	0	0	0	0
SAL_IA9844AA	PNUSAS008845	Cerro	0	0	0	0	0	0	0	0	0	0	0
SAL_IA9771AA	FSIS1700053	Cerro	0	0	0	0	0	0	0	0	0	0	0
SAL_IA9775AA	FSIS1700048	Cerro	0	0	0	0	0	0	0	0	0	0	0
SAL_IA9738AA	FSIS1710713	Cerro	0	0	0	0	0	0	0	0	0	0	0
SAL_IA9732AA	FSIS1710560	Cerro	0	0	0	0	0	0	0	0	0	0	0
SAL_JA0039AA	FSIS1710720	Cerro	0	0	0	0	0	0	0	0	0	0	0
SAL_JA0332AA	ADRDL-16050019	Cerro	0	0	0	0	0	0	0	0	0	0	0
SAL_JA1515AA	PNUSAS010823	Cerro	0	0	0	0	0	0	0	0	0	0	0
SAL_JA1596AA	PNUSAS010787	Cerro	0	0	0	0	0	0	0	0	0	0	0
SAL_JA1138AA	12B06394-7	Cerro	0	0	0	0	0	0	0	0	0	0	0
SAL_JA1137AA	15B12129-1	Cerro	0	0	0	0	0	0	0	0	0	0	0
SAL_JA0602AA	CFSAN025432	Cerro	0	0	0	0	0	0	0	0	0	0	0
SAL_JA0600AA	CFSAN025429	Cerro	0	0	0	0	0	0	0	0	0	0	0
SAL_JA1850AA	FSIS1710211	Cerro	0	0	0	0	0	0	0	0	0	0	0
SAL_JA1851AA	FSIS1710209	Cerro	0	0	0	0	0	0	0	0	0	0	0
SAL_JA1854AA	FSIS1710207	Cerro	0	0	0	0	0	0	0	0	0	0	0
SAL_JA2274AA	FSIS1700240	Cerro	0	0	0	0	0	0	0	0	0	0	0
SAL_JA2801AA	FSIS1710876	Cerro	0	0	0	0	0	0	0	0	0	0	0
SAL_JA2850AA	FSIS1710984	Cerro	0	0	0	0	0	0	0	0	0	0	0
SAL_JA2962AA	FSIS1700429	Cerro	0	0	0	0	0	0	0	0	0	0	0
SAL_JA2955AA	FSIS1700432	Cerro	0	0	0	0	0	0	0	0	0	0	0
SAL_JA3459AA	FSIS1700343	Cerro	0	0	0	0	0	0	0	0	0	0	0
SAL_JA3131AA	FSIS1700285	Cerro	0	0	0	0	0	0	0	0	0	0	0
SAL_JA3603AA	FSIS1700439	Cerro	0	0	0	0	0	0	0	0	0	0	0
SAL_JA4213AA	FSIS1700572	Cerro	0	0	0	0	0	0	0	0	0	0	0
SAL_JA4217AA	FSIS1700562	Cerro	0	0	0	0	0	0	0	0	0	0	0
SAL_JA4218AA	FSIS1700577	Cerro	0	0	0	0	0	0	0	0	0	0	0

SAL_JA4276AA	OH-16-14199	Cerro	0	0	0	0	0	0	0	0	0	0	0
SAL_JA4478AA	OH-16-18000-2	Cerro	0	0	0	0	0	0	0	0	0	0	0
SAL_JA4586AA	PNUSAS013060	Cerro	0	0	0	0	0	0	0	0	0	0	0
SAL_JA6092AA	FSIS1700613	Cerro	0	0	0	0	0	0	0	0	0	0	0
SAL_JA6046AA	FSIS1700942	Cerro	0	0	0	0	0	0	0	0	0	0	0
SAL_JA5386AA	342552	Cerro	0	0	0	0	0	0	0	0	0	0	0
SAL_JA6765AA	PNUSAS013289	Cerro	0	0	0	0	0	0	0	0	0	0	0
SAL_JA7025AA	PNUSAS011920	Cerro	0	0	0	0	0	0	0	0	0	0	0
SAL_JA7487AA	FSIS1700593	Cerro	0	0	0	0	0	0	0	0	0	0	0
SAL_JA8249AA	346586	Cerro	0	0	0	0	0	0	0	0	0	0	0
SAL_JA7967AA	337036	Cerro	0	0	0	0	0	0	0	0	0	0	0
SAL_JA7593AA	PNUSAS014481	Cerro	0	0	0	0	0	0	0	0	0	0	0
SAL_JA7498AA	FSIS1700578	Cerro	0	0	0	0	0	0	0	0	0	0	0
SAL_JA8784AA	FSIS1701089	Cerro	0	0	0	0	0	0	0	0	0	0	0
SAL_JA8526AA	FSIS1701072	Cerro	0	0	0	0	0	0	0	0	0	0	0
SAL_JA8869AA	FSIS1701476	Cerro	0	0	0	0	0	0	0	0	0	0	0
SAL_JA9253AA	PNUSAS015159	Cerro	0	0	0	0	0	0	0	0	0	0	0
SAL_JA8845AA	FSIS1701283	Cerro	0	0	0	0	0	0	0	0	0	0	0
SAL_JA8863AA	FSIS1701471	Cerro	0	0	0	0	0	0	0	0	0	0	0
SAL_KA0189AA	E2016008473	Cerro	0	0	0	0	0	0	0	0	0	0	0
SAL_KA0201AA	E2016008487	Cerro	0	0	0	0	0	0	0	0	0	0	0
SAL_JA9937AA	PNUSAS015343	Cerro	0	0	0	0	0	0	0	0	0	0	0
SAL_JA8878AA	FSIS1701811	Cerro	0	0	0	0	0	0	0	0	0	0	0
SAL_KA0990AA	FSIS1702233	Cerro	0	0	0	0	0	0	0	0	0	0	0
SAL_KA2033AA	FSIS1701904	Cerro	0	0	0	0	0	0	0	0	0	0	0
SAL_KA2125AA	FDA00004715	Cerro	0	0	0	0	0	0	0	0	0	0	0
SAL_KA2093AA	OH-17-1314	Cerro	0	0	0	0	0	0	0	0	0	0	0
SAL_KA2097AA	OH-17-2028-3	Cerro	0	0	0	0	0	0	0	0	0	0	0
SAL_KA2090AA	ADRDL-15-3528	Cerro	0	0	0	0	0	0	0	0	0	0	0
SAL_KA2094AA	ADRDL-15-3591	Cerro	0	0	0	0	0	0	0	0	0	0	0
SAL_KA2101AA	OH-17-2547	Cerro	0	0	0	0	0	0	0	0	0	0	0
SAL_KA2959AA	FSIS1701895	Cerro	0	0	0	0	0	0	0	0	0	0	0
SAL_KA3127AA	FDA00000440	Cerro	0	0	0	0	0	0	0	0	0	0	0
SAL_KA3128AA	FDA00001903	Cerro	0	0	0	0	0	0	0	0	0	0	0
SAL_KA2405AA	FSIS1702211	Cerro	0	0	0	0	0	0	0	0	0	0	0
SAL_KA3070AA	FSIS1702346	Cerro	0	0	0	0	0	0	0	0	0	0	0
SAL_KA3549AA	FSIS1702435	Cerro	0	0	0	0	0	0	0	0	0	0	0
SAL_KA3584AA	FSIS1702511	Cerro	0	0	0	0	0	0	0	0	0	0	0
SAL_KA3704AA	FSIS1702810	Cerro	0	0	0	0	0	0	0	0	0	0	0
SAL_KA3718AA	FSIS1702800	Cerro	0	0	0	0	0	0	0	0	0	0	0
SAL_KA3693AA	FSIS1701409	Cerro	0	0	0	0	0	0	0	0	0	0	0
SAL_KA4825AA	FSIS1702772	Cerro	0	0	0	0	0	0	0	0	0	0	0
SAL_KA4026AA	RM_80	Cerro	0	0	0	0	0	0	0	0	0	0	0
SAL_KA5552AA	FSIS1703072	Cerro	0	0	0	0	0	0	0	0	0	0	0
SAL_KA5731AA	FSIS1703089	Cerro	0	0	0	0	0	0	0	0	0	0	0
SAL_KA7142AA	FSIS1703188	Cerro	0	0	0	0	0	0	0	0	0	0	0
SAL_KA7162AA	FSIS1703408	Cerro	0	0	0	0	0	0	0	0	0	0	0
SAL_KA7682AA	FSIS1703471	Cerro	0	0	0	0	0	0	0	0	0	0	0
SAL_KA7707AA	2015AM-0145	Cerro	0	0	0	0	0	0	0	0	0	0	0
SAL_KA9145AA	AL-17-VL-OH-IN-PU-000	Cerro	0	0	0	0	0	0	0	0	0	0	0
SAL_LA0013AA	FSIS1703571	Cerro	0	0	0	0	0	0	0	0	0	0	0
SAL_KA9919AA	FSIS1703636	Cerro	0	0	0	0	0	0	0	0	0	0	0
SAL_KA9910AA	FSIS1703639	Cerro	0	0	0	0	0	0	0	0	0	0	0
SAL_KA9840AA	FSIS1703869	Cerro	0	0	0	0	0	0	0	0	0	0	0
SAL_KA9846AA	FSIS1703554	Cerro	0	0	0	0	0	0	0	0	0	0	0
SAL_KA9871AA	PNUSAS021092	Cerro	0	0	0	0	0	0	0	0	0	0	0
SAL_LA0640AA	FSIS11703929	Cerro	0	0	0	0	0	0	0	0	0	0	0
SAL_LA0630AA	FSIS1703738	Cerro	0	0	0	0	0	0	0	0	0	0	0
SAL_LA0610AA	FSIS11703978	Cerro	0	0	0	0	0	0	0	0	0	0	0
SAL_LA0615AA	FSIS1703690	Cerro	0	0	0	0	0	0	0	0	0	0	0
SAL_LA1163AA	FSIS21720082	Cerro	0	0	0	0	0	0	0	0	0	0	0
SAL_LA1480AA	FSIS21720084	Cerro	0	0	0	0	0	0	0	0	0	0	0
SAL_LA1809AA	FSIS11704062	Cerro	0	0	0	0	0	0	0	0	0	0	0
SAL_LA1808AA	FSIS11704097	Cerro	0	0	0	0	0	0	0	0	0	0	0
SAL_LA2281AA	PNUSAS020977	Cerro	0	0	0	0	0	0	0	0	0	0	0
SAL_LA3907AA	PNUSAS022618	Cerro	0	0	0	0	0	0	0	0	0	0	0
SAL_LA4188AA	PNUSAS020459	Cerro	0	0	0	0	0	0	0	0	0	0	0
SAL_LA4495AA	PNUSAS022262	Cerro	0	0	0	0	0	0	0	0	0	0	0
SAL_LA5051AA	FSIS11704391	Cerro	0	0	0	0	0	0	0	0	0	0	0
SAL_LA5052AA	FSIS11704395	Cerro	0	0	0	0	0	0	0	0	0	0	0
SAL_LA4782AA	FSIS11704048	Cerro	0	0	0	0	0	0	0	0	0	0	0
SAL_LA4753AA	PNUSAS021884	Cerro	0	0	0	0	0	0	0	0	0	0	0
SAL_LA3105AA	CH08.030	Cerro	0	0	0	0	0	0	0	0	0	0	0

SAL_LA6079AA	FDA00009420	Cerro	0	0	0	0	0	0	0	0	0	0	0
SAL_LA9557AA	FSIS11704380	Cerro	0	0	0	0	0	0	0	0	0	0	0
SAL_MA0338AA	FSIS11704762	Cerro	0	0	0	0	0	0	0	0	0	0	0
SAL_MA1286AA	B1713414	Cerro	0	0	0	0	0	0	0	0	0	0	0
SAL_MA1272AA	B1716611	Cerro	0	0	0	0	0	0	0	0	0	0	0
SAL_MA2791AA	PNUSAS022390	Cerro	0	0	0	0	0	0	0	0	0	0	0
SAL_MA2762AA	PNUSAS024923	Cerro	0	0	0	0	0	0	0	0	0	0	0
SAL_MA2991AA	SAL-17-VL-LA-ND-0005	Cerro	0	0	0	0	0	0	0	0	0	0	0
SAL_MA3402AA	FSIS21720466	Cerro	0	0	0	0	0	0	0	0	0	0	0
SAL_MA3145AA	FSIS11704232	Cerro	0	0	0	0	0	0	0	0	0	0	0
SAL_MA3950AA	TX-ACF1702190	Cerro	0	0	0	0	0	0	0	0	0	0	0
SAL_MA5030AA	FSIS1702651	Cerro	0	0	0	0	0	0	0	0	0	0	0
SAL_MA5033AA	FSIS1702654	Cerro	0	0	0	0	0	0	0	0	0	0	0
SAL_MA5056AA	FSIS1702784	Cerro	0	0	0	0	0	0	0	0	0	0	0
SAL_MA5065AA	FSIS1702779	Cerro	0	0	0	0	0	0	0	0	0	0	0
SAL_MA5062AA	FSIS1702782	Cerro	0	0	0	0	0	0	0	0	0	0	0
SAL_MA5271AA	OH-17-21081	Cerro	0	0	0	0	0	0	0	0	0	0	0
SAL_MA5267AA	OH-17-20164	Cerro	0	0	0	0	0	0	0	0	0	0	0
SAL_MA5265AA	OH-17-21295	Cerro	0	0	0	0	0	0	0	0	0	0	0
SAL_MA5269AA	OH-17-20991-1	Cerro	0	0	0	0	0	0	0	0	0	0	0
SAL_MA5266AA	OH-17-20029	Cerro	0	0	0	0	0	0	0	0	0	0	0
SAL_MA5317AA	FSIS11705171	Cerro	0	0	0	0	0	0	0	0	0	0	0
SAL_MA5435AA	OH-17-18709	Cerro	0	0	0	0	0	0	0	0	0	0	0
SAL_MA5436AA	OH-17-19808	Cerro	0	0	0	0	0	0	0	0	0	0	0
SAL_EA1484AA	FSIS1500644	Cerro	0	0	0	0	0	0	0	0	0	0	0
SAL_GA9589AA	FSIS1607713	Cerro	0	0	0	0	0	0	0	0	0	0	0
SAL_BA2885AA	CFSAN030836	Cerro	0	0	0	0	0	0	0	0	0	0	0
SAL_EA0381AA	NY_208191001	Cerro	0	0	0	0	0	0	0	0	0	0	0
SAL_EA0298AA	FDN0028	Cerro	0	0	0	0	0	0	0	0	0	0	0
SAL_CA8607AA	CFSAN027952	Cerro	0	0	0	0	0	0	0	0	0	0	0
SAL_DA6538AA	FNW19a10	Cerro	0	0	0	0	0	0	0	0	0	0	0
SAL_CA8792AA	AUCVM14-31	Cerro	0	0	0	0	0	0	0	0	0	0	0
SAL_DA6509AA	VA-WGS-00267	Cerro	0	0	0	0	0	0	0	0	0	0	0
SAL_DA8007AA	FSW0113	Cerro	0	0	0	0	0	0	0	0	0	0	0
SAL_CA8179AA	CFSAN001588	Cerro	0	0	0	0	0	0	0	0	0	0	0
SAL_EA0336AA	NY_02022199	Cerro	0	0	0	0	0	0	0	0	0	0	0
SAL_EA4270AA	FDA00001839	Cerro	0	0	0	0	0	0	0	0	0	0	0
SAL_EA6153AA	FSIS1501182	Cerro	0	0	0	0	0	0	0	0	0	0	0
SAL_EA6127AA	FSIS1501929	Cerro	0	0	0	0	0	0	0	0	0	0	0
SAL_EA6587AA	FSIS1501887	Cerro	0	0	0	0	0	0	0	0	0	0	0
SAL_HA3039AA	FSIS1608041	Cerro	0	0	0	0	0	0	0	0	0	0	0
SAL_HA0467AA	PNUSAS004316	Cerro	0	0	0	0	0	0	0	0	0	0	0
SAL_HA2929AA	FSIS1608109	Cerro	0	0	0	0	0	0	0	0	0	0	0
SAL_HA7225AA	FDA00010814	Cerro	0	0	0	0	0	0	0	0	0	0	0
SAL_GA9763AA	NYVetLIRN-15	Cerro	0	0	0	0	0	0	0	0	0	0	0
SAL_HA9650AA	ADRDL-787	Cerro	0	0	0	0	0	0	0	0	0	0	0
SAL_HA8876AA	NCTC8271	Cerro	0	0	0	0	0	0	0	0	0	0	0
SAL_GA6138AA	FDA00010463	Cerro	0	0	0	0	0	0	0	0	0	0	0
SAL_FA8244AA	FSIS1606930	Cerro	0	0	0	0	0	0	0	0	0	0	0
SAL_GA7762AA	FSIS1607350	Cerro	0	0	0	0	0	0	0	0	0	0	0
SAL_GA8686AA	ADRDL 16-5772	Cerro	0	0	0	0	0	0	0	0	0	0	0
SAL_GA8697AA	PNUSAS002427	Cerro	0	0	0	0	0	0	0	0	0	0	0
SAL_GA7753AA	FSIS1607395	Cerro	0	0	0	0	0	0	0	0	0	0	0
SAL_GA6046AA	FSIS1607199	Cerro	0	0	0	0	0	0	0	0	0	0	0
SAL_GA5969AA	FSIS1607173	Cerro	0	0	0	0	0	0	0	0	0	0	0
SAL_GA5270AA	CFSAN042939	Cerro	0	0	0	0	0	0	0	0	0	0	0
SAL_GA5445AA	CFSAN042339	Cerro	0	0	0	0	0	0	0	0	0	0	0
SAL_FA5435AA	FSIS1606185	Cerro	0	0	0	0	0	0	0	0	0	0	0
SAL_IA1990AA	FSIS1609564	Cerro	0	0	0	0	0	0	0	0	0	0	0
SAL_IA5335AA	FSIS1710082	Cerro	0	0	0	0	0	0	0	0	0	0	0
SAL_IA6594AA	FSIS1710240	Cerro	0	0	0	0	0	0	0	0	0	0	0
SAL_IA7408AA	OH-16-28001	Cerro	0	0	0	0	0	0	0	0	0	0	0
SAL_IA7401AA	OH-17-639	Cerro	0	0	0	0	0	0	0	0	0	0	0
SAL_JA0035AA	FSIS1710867	Cerro	0	0	0	0	0	0	0	0	0	0	0
SAL_JA0598AA	CFSAN025428	Cerro	0	0	0	0	0	0	0	0	0	0	0
SAL_JA4266AA	FDA00011689	Cerro	0	0	0	0	0	0	0	0	0	0	0
SAL_JA8592AA	FSIS1700927	Cerro	0	0	0	0	0	0	0	0	0	0	0
SAL_JA8780AA	FSIS1701095	Cerro	0	0	0	0	0	0	0	0	0	0	0
SAL_JA8851AA	FSIS1701358	Cerro	0	0	0	0	0	0	0	0	0	0	0
SAL_KA2103AA	OH-17-2272	Cerro	0	0	0	0	0	0	0	0	0	0	0
SAL_KA2385AA	FSIS1702226	Cerro	0	0	0	0	0	0	0	0	0	0	0
SAL_KA3701AA	FSIS1702574	Cerro	0	0	0	0	0	0	0	0	0	0	0
SAL_KA6215AA	CFSAN064282	Cerro	0	0	0	0	0	0	0	0	0	0	0

SAL_KA8320AA	FSIS1703365	Cerro	0	0	0	0	0	0	0	0	0	0	0
SAL_LA0016AA	FSIS1703568	Cerro	0	0	0	0	0	0	0	0	0	0	0
SAL_LA4353AA	FSIS11704267	Cerro	0	0	0	0	0	0	0	0	0	0	0
SAL_GA2977AA	serovar Cerro 87	Cerro	0	0	0	0	0	0	0	0	0	0	0
SAL_MA6207AA	PNUSAS027828	Cerro	0	0	0	0	0	0	0	0	0	0	0
SAL_MA7366AA	OH-17-6632	Cerro	0	0	0	0	0	0	0	0	0	0	0
SAL_MA7381AA	FSIS21720633	Cerro	0	0	0	0	0	0	0	0	0	0	0
SAL_MA7390AA	ADRDL-1555	Cerro	0	0	0	0	0	0	0	0	0	0	0
SAL_MA7405AA	ADRDL-1559	Cerro	0	0	0	0	0	0	0	0	0	0	0
SAL_MA7349AA	OH-17-7329	Cerro	0	0	0	0	0	0	0	0	0	0	0
SAL_MA7184AA	PNUSAS028106	Cerro	0	0	0	0	0	0	0	0	0	0	0
SAL_MA7095AA	PNUSAS026016	Cerro	0	0	0	0	0	0	0	0	0	0	0
SAL_MA6880AA	ADRDL-1292	Cerro	0	0	0	0	0	0	0	0	0	0	0
SAL_MA7474AA	FSIS11705700	Cerro	0	0	0	0	0	0	0	0	0	0	0
SAL_MA7840AA	OH-17-5830	Cerro	0	0	0	0	0	0	0	0	0	0	0
SAL_MA8407AA	PNUSAS028516	Cerro	0	0	0	0	0	0	0	0	0	0	0
SAL_MA8869AA	FSIS11705523	Cerro	0	0	0	0	0	0	0	0	0	0	0
SAL_MA8824AA	FSIS11705650	Cerro	0	0	0	0	0	0	0	0	0	0	0
SAL_MA8787AA	FSIS11705051	Cerro	0	0	0	0	0	0	0	0	0	0	0
SAL_MA8789AA	FSIS11705984	Cerro	0	0	0	0	0	0	0	0	0	0	0
SAL_MA8815AA	FSIS11705660	Cerro	0	0	0	0	0	0	0	0	0	0	0
SAL_MA9077AA	OH-17-11156-1	Cerro	0	0	0	0	0	0	0	0	0	0	0
SAL_MA9082AA	OH-17-12649	Cerro	0	0	0	0	0	0	0	0	0	0	0
SAL_MA9083AA	OH-17-9766	Cerro	0	0	0	0	0	0	0	0	0	0	0
SAL_MA9042AA	FSIS11705344	Cerro	0	0	0	0	0	0	0	0	0	0	0
SAL_MA9044AA	FSIS11705862	Cerro	0	0	0	0	0	0	0	0	0	0	0
SAL_MA9016AA	OH-17-10164	Cerro	0	0	0	0	0	0	0	0	0	0	0
SAL_MA9023AA	FSIS11705076	Cerro	0	0	0	0	0	0	0	0	0	0	0
SAL_NA0581AA	PNUSAS029522	Cerro	0	0	0	0	0	0	0	0	0	0	0
SAL_NA0935AA	OH-17-22328-1	Cerro	0	0	0	0	0	0	0	0	0	0	0
SAL_NA0943AA	OH-17-14027	Cerro	0	0	0	0	0	0	0	0	0	0	0
SAL_NA0946AA	OH-17-14028	Cerro	0	0	0	0	0	0	0	0	0	0	0
SAL_NA9736AA	HJ27-VA-WGS-17105	Cerro	0	0	0	0	0	0	0	0	0	0	0
SAL_NA0964AA	VA-WGS-17105	Cerro	0	0	0	0	0	0	0	0	0	0	0
SAL_NA0903AA	CFSAN072640	Cerro	0	0	0	0	0	0	0	0	0	0	0
SAL_NA0902AA	FDA00000613	Cerro	0	0	0	0	0	0	0	0	0	0	0
SAL_OA0188AA	HJ28-VA-WGS-17106	Cerro	0	0	0	0	0	0	0	0	0	0	0
SAL_NA0925AA	VA-WGS-17106	Cerro	0	0	0	0	0	0	0	0	0	0	0
SAL_NA0951AA	OH-17-13414-1	Cerro	0	0	0	0	0	0	0	0	0	0	0
SAL_NA1339AA	PNUSAS029484	Cerro	0	0	0	0	0	0	0	0	0	0	0
SAL_NA1254AA	FDA00000589	Cerro	0	0	0	0	0	0	0	0	0	0	0
SAL_NA1668AA	FSIS11705539	Cerro	0	0	0	0	0	0	0	0	0	0	0
SAL_NA1641AA	SAL-17-VL-LA-KY-0010	Cerro	0	0	0	0	0	0	0	0	0	0	0
SAL_NA1638AA	FSIS11705346	Cerro	0	0	0	0	0	0	0	0	0	0	0
SAL_NA2004AA	FSIS11706152	Cerro	0	0	0	0	0	0	0	0	0	0	0
SAL_NA1987AA	FSIS11706274	Cerro	0	0	0	0	0	0	0	0	0	0	0
SAL_NA2005AA	FSIS11706130	Cerro	0	0	0	0	0	0	0	0	0	0	0
SAL_NA1990AA	FSIS11706135	Cerro	0	0	0	0	0	0	0	0	0	0	0
SAL_NA2536AA	PNUSAS025486	Cerro	0	0	0	0	0	0	0	0	0	0	0
SAL_NA3279AA	CFSAN073600	Cerro	0	0	0	0	0	0	0	0	0	0	0
SAL_NA3227AA	CFSAN073596	Cerro	0	0	0	0	0	0	0	0	0	0	0
SAL_NA3244AA	CFSAN073580	Cerro	0	0	0	0	0	0	0	0	0	0	0
SAL_NA3570AA	PNUSAS029955	Cerro	0	0	0	0	0	0	0	0	0	0	0
SAL_NA4071AA	FSIS11706620	Cerro	0	0	0	0	0	0	0	0	0	0	0
SAL_NA4079AA	FSIS11706704	Cerro	0	0	0	0	0	0	0	0	0	0	0
SAL_NA4038AA	FSIS11706676	Cerro	0	0	0	0	0	0	0	0	0	0	0
SAL_NA3934AA	OH-17-23924	Cerro	0	0	0	0	0	0	0	0	0	0	0
SAL_NA4502AA	PNUSAS031524	Cerro	0	0	0	0	0	0	0	0	0	0	0
SAL_NA4538AA	FSIS11706942	Cerro	0	0	0	0	0	0	0	0	0	0	0
SAL_NA4520AA	FSIS11706488	Cerro	0	0	0	0	0	0	0	0	0	0	0
SAL_NA4628AA	FSIS11706977	Cerro	0	0	0	0	0	0	0	0	0	0	0
SAL_NA4945AA	PNUSAS031656	Cerro	0	0	0	0	0	0	0	0	0	0	0
SAL_NA5780AA	2015AM-2820	Cerro	0	0	0	0	0	0	0	0	0	0	0
SAL_NA5760AA	2015AM-2778	Cerro	0	0	0	0	0	0	0	0	0	0	0
SAL_NA5676AA	OH-17-23738	Cerro	0	0	0	0	0	0	0	0	0	0	0
SAL_NA6421AA	FSIS11706296	Cerro	0	0	0	0	0	0	0	0	0	0	0
SAL_NA6424AA	FSIS11706294	Cerro	0	0	0	0	0	0	0	0	0	0	0
SAL_NA6312AA	FSIS11706986	Cerro	0	0	0	0	0	0	0	0	0	0	0
SAL_NA6227AA	FSIS11807112	Cerro	0	0	0	0	0	0	0	0	0	0	0
SAL_NA6936AA	PNUSAS032025	Cerro	0	0	0	0	0	0	0	0	0	0	0
SAL_NA8033AA	449466	Cerro	0	0	0	0	0	0	0	0	0	0	0
SAL_NA8259AA	S12	Cerro	0	0	0	0	0	0	0	0	0	0	0
SAL_NA8089AA	2015AM-3620	Cerro	0	0	0	0	0	0	0	0	0	0	0

SAL_NA8277AA	FSIS11706480	Cerro	0	0	0	0	0	0	0	0	0	0	0
SAL_NA8573AA	PNUSAS032989	Cerro	0	0	0	0	0	0	0	0	0	0	0
SAL_NA8941AA	FSIS11807266	Cerro	0	0	0	0	0	0	0	0	0	0	0
SAL_NA8943AA	FSIS11807272	Cerro	0	0	0	0	0	0	0	0	0	0	0
SAL_NA8765AA	CFSAN075238	Cerro	0	0	0	0	0	0	0	0	0	0	0
SAL_NA8772AA	FSIS11807277	Cerro	0	0	0	0	0	0	0	0	0	0	0
SAL_NA9516AA	FSIS11807374	Cerro	0	0	0	0	0	0	0	0	0	0	0
SAL_NA9436AA	PNUSAS032516	Cerro	0	0	0	0	0	0	0	0	0	0	0
SAL_NA9268AA	PNUSAS033360	Cerro	0	0	0	0	0	0	0	0	0	0	0
SAL_OA0169AA	FSIS11807111	Cerro	0	0	0	0	0	0	0	0	0	0	0
SAL_OA0172AA	FSIS11807557	Cerro	0	0	0	0	0	0	0	0	0	0	0
SAL_OA0158AA	FSIS11807359	Cerro	0	0	0	0	0	0	0	0	0	0	0
SAL_OA0159AA	FSIS11807358	Cerro	0	0	0	0	0	0	0	0	0	0	0
SAL_OA0164AA	FSIS11807553	Cerro	0	0	0	0	0	0	0	0	0	0	0
SAL_NA9902AA	PNUSAS033432	Cerro	0	0	0	0	0	0	0	0	0	0	0
SAL_OA0209AA	PNUSAS010061	Cerro	0	0	0	0	0	0	0	0	0	0	0
SAL_OA0622AA	PNUSAS033765	Cerro	0	0	0	0	0	0	0	0	0	0	0
SAL_OA0761AA	FSIS11807714	Cerro	0	0	0	0	0	0	0	0	0	0	0
SAL_OA7225AA	OH-18-3763-3	Cerro	0	0	0	0	0	0	0	0	0	0	0
SAL_OA7240AA	OH-18-3763-2	Cerro	0	0	0	0	0	0	0	0	0	0	0
SAL_OA9996AA	FSIS11808298	Cerro	0	0	0	0	0	0	0	0	0	0	0
SAL_OA9976AA	FSIS11808311	Cerro	0	0	0	0	0	0	0	0	0	0	0
SAL_OA9977AA	FSIS11808325	Cerro	0	0	0	0	0	0	0	0	0	0	0
SAL_OA7492AA	FSIS11807903	Cerro	0	0	0	0	0	0	0	0	0	0	0
SAL_OA5000AA	PNUSAS035480	Cerro	0	0	0	0	0	0	0	0	0	0	0
SAL_OA8126AA	OH-17-28106	Cerro	0	0	0	0	0	0	0	0	0	0	0
SAL_OA8129AA	OH-17-27660	Cerro	0	0	0	0	0	0	0	0	0	0	0
SAL_OA8130AA	OH-18-2031	Cerro	0	0	0	0	0	0	0	0	0	0	0
SAL_OA8103AA	FSIS11808631	Cerro	0	0	0	0	0	0	0	0	0	0	0
SAL_PA0551AA	FSIS11808805	Cerro	0	0	0	0	0	0	0	0	0	0	0
SAL_PA0645AA	FSIS11808934	Cerro	0	0	0	0	0	0	0	0	0	0	0
SAL_PA1714AA	FSIS11809060	Cerro	0	0	0	0	0	0	0	0	0	0	0
SAL_PA1779AA	FSIS11809072	Cerro	0	0	0	0	0	0	0	0	0	0	0
SAL_PA2975AA	FSIS11808929	Cerro	0	0	0	0	0	0	0	0	0	0	0
SAL_PA2963AA	FSIS11809212	Cerro	0	0	0	0	0	0	0	0	0	0	0
SAL_PA2964AA	FSIS11809219	Cerro	0	0	0	0	0	0	0	0	0	0	0
SAL_PA4094AA	FSIS11809475	Cerro	0	0	0	0	0	0	0	0	0	0	0
SAL_PA5194AA	OH-18-7797	Cerro	0	0	0	0	0	0	0	0	0	0	0
SAL_PA5195AA	OH-18-8369-1	Cerro	0	0	0	0	0	0	0	0	0	0	0
SAL_PA5190AA	OH-18-8377-1	Cerro	0	0	0	0	0	0	0	0	0	0	0
SAL_PA5539AA	FSIS11809567	Cerro	0	0	0	0	0	0	0	0	0	0	0
SAL_PA5572AA	FSIS11809717	Cerro	0	0	0	0	0	0	0	0	0	0	0
SAL_PA5540AA	FSIS11809565	Cerro	0	0	0	0	0	0	0	0	0	0	0
SAL_PA5536AA	FSIS11809573	Cerro	0	0	0	0	0	0	0	0	0	0	0
SAL_PA5720AA	PNUSAS040036	Cerro	0	0	0	0	0	0	0	0	0	0	0
SAL_PA6241AA	205729	Cerro	0	0	0	0	0	0	0	0	0	0	0
SAL_PA6977AA	PNUSAS041023	Cerro	0	0	0	0	0	0	0	0	0	0	0
SAL_PA7234AA	FSIS11809871	Cerro	0	0	0	0	0	0	0	0	0	0	0
SAL_PA7236AA	FSIS11809379	Cerro	0	0	0	0	0	0	0	0	0	0	0
SAL_PA7226AA	FSIS11809873	Cerro	0	0	0	0	0	0	0	0	0	0	0
SAL_PA7223AA	FSIS11809876	Cerro	0	0	0	0	0	0	0	0	0	0	0
SAL_PA7193AA	CFSAN080903	Cerro	0	0	0	0	0	0	0	0	0	0	0
SAL_PA8812AA	OH-12-578-1	Cerro	0	0	0	0	0	0	0	0	0	0	0
SAL_PA9364AA	FSIS11809857	Cerro	0	0	0	0	0	0	0	0	0	0	0
SAL_PA9371AA	FSIS11809394	Cerro	0	0	0	0	0	0	0	0	0	0	0
SAL_PA9363AA	FSIS11809858	Cerro	0	0	0	0	0	0	0	0	0	0	0
SAL_PA8984AA	FSIS11810006	Cerro	0	0	0	0	0	0	0	0	0	0	0
SAL_PA8956AA	271332	Cerro	0	0	0	0	0	0	0	0	0	0	0
SAL_QA0002AA	FSIS31800488	Cerro	0	0	0	0	0	0	0	0	0	0	0
SAL_QA0775AA	PNUSAS042160	Cerro	0	0	0	0	0	0	0	0	0	0	0
SAL_QA1300AA	536068	Cerro	0	0	0	0	0	0	0	0	0	0	0
SAL_QA1258AA	ADRDL-1926	Cerro	0	0	0	0	0	0	0	0	0	0	0
SAL_QA1160AA	FSIS11810213	Cerro	0	0	0	0	0	0	0	0	0	0	0
SAL_QA1401AA	ADRDL-1921	Cerro	0	0	0	0	0	0	0	0	0	0	0
SAL_QA1826AA	FSIS11810201	Cerro	0	0	0	0	0	0	0	0	0	0	0
SAL_QA1706AA	FSIS11808547	Cerro	0	0	0	0	0	0	0	0	0	0	0
SAL_QA2859AA	450321	Cerro	0	0	0	0	0	0	0	0	0	0	0
SAL_QA2654AA	428203	Cerro	0	0	0	0	0	0	0	0	0	0	0
SAL_QA3223AA	203970	Cerro	0	0	0	0	0	0	0	0	0	0	0
SAL_QA4165AA	FDA00004876	Cerro	0	0	0	0	0	0	0	0	0	0	0
SAL_QA4354AA	PNUSAS042325	Cerro	0	0	0	0	0	0	0	0	0	0	0
SAL_QA4597AA	FSIS11810419	Cerro	0	0	0	0	0	0	0	0	0	0	0
SAL_QA5159AA	143564	Cerro	0	0	0	0	0	0	0	0	0	0	0

SAL_QA6580AA	187573	Cerro	0	0	0	0	0	0	0	0	0	0	0
SAL_QA6939AA	FSIS11810207	Cerro	0	0	0	0	0	0	0	0	0	0	0
SAL_QA6767AA	493374	Cerro	0	0	0	0	0	0	0	0	0	0	0
SAL_QA7112AA	PNUSAS042801	Cerro	0	0	0	0	0	0	0	0	0	0	0
SAL_QA8479AA	PNUSAS044141	Cerro	0	0	0	0	0	0	0	0	0	0	0
SAL_QA8544AA	FSIS11810664	Cerro	0	0	0	0	0	0	0	0	0	0	0
SAL_QA8552AA	FSIS11807607	Cerro	0	0	0	0	0	0	0	0	0	0	0
SAL_QA8542AA	FSIS11808809	Cerro	0	0	0	0	0	0	0	0	0	0	0
SAL_QA8541AA	FSIS11810203	Cerro	0	0	0	0	0	0	0	0	0	0	0
SAL_QA8939AA	PNUSAS044576	Cerro	0	0	0	0	0	0	0	0	0	0	0
SAL_QA9405AA	532482	Cerro	0	0	0	0	0	0	0	0	0	0	0
SAL_QA9360AA	559076	Cerro	0	0	0	0	0	0	0	0	0	0	0
SAL_RA1277AA	FSIS11811094	Cerro	0	0	0	0	0	0	0	0	0	0	0
SAL_RA3544AA	FSIS11811383	Cerro	0	0	0	0	0	0	0	0	0	0	0
SAL_RA4185AA	FSIS11811393	Cerro	0	0	0	0	0	0	0	0	0	0	0
SAL_RA4191AA	FSIS11811392	Cerro	0	0	0	0	0	0	0	0	0	0	0
SAL_RA4227AA	VA-WGS-18081	Cerro	0	0	0	0	0	0	0	0	0	0	0
SAL_RA3872AA	FDA00001569	Cerro	0	0	0	0	0	0	0	0	0	0	0
SAL_RA4478AA	266991	Cerro	0	0	0	0	0	0	0	0	0	0	0
SAL_RA6091AA	CFSAN082970	Cerro	0	0	0	0	0	0	0	0	0	0	0
SAL_RA5767AA	CFSAN082932	Cerro	0	0	0	0	0	0	0	0	0	0	0
SAL_RA5768AA	CFSAN082925	Cerro	0	0	0	0	0	0	0	0	0	0	0
SAL_RA6328AA	PNUSAS045406	Cerro	0	0	0	0	0	0	0	0	0	0	0
SAL_RA6678AA	FSIS11811666	Cerro	0	0	0	0	0	0	0	0	0	0	0
SAL_RA6602AA	266985	Cerro	0	0	0	0	0	0	0	0	0	0	0
SAL_RA6884AA	FSIS11811748	Cerro	0	0	0	0	0	0	0	0	0	0	0
SAL_RA6955AA	FSIS11811371	Cerro	0	0	0	0	0	0	0	0	0	0	0
SAL_RA7774AA	FSIS11811626	Cerro	0	0	0	0	0	0	0	0	0	0	0
SAL_RA7681AA	536060	Cerro	0	0	0	0	0	0	0	0	0	0	0
SAL_RA8240AA	365825	Cerro	0	0	0	0	0	0	0	0	0	0	0
SAL_RA8411AA	FSIS11811729	Cerro	0	0	0	0	0	0	0	0	0	0	0
SAL_RA8364AA	FSIS11811973	Cerro	0	0	0	0	0	0	0	0	0	0	0
SAL_RA8148AA	458051	Cerro	0	0	0	0	0	0	0	0	0	0	0
SAL_RA8678AA	FSIS11811944	Cerro	0	0	0	0	0	0	0	0	0	0	0
SAL_RA8709AA	FSIS11811619	Cerro	0	0	0	0	0	0	0	0	0	0	0
SAL_RA9338AA	CFSAN084091	Cerro	0	0	0	0	0	0	0	0	0	0	0
SAL_RA9304AA	CFSAN084120	Cerro	0	0	0	0	0	0	0	0	0	0	0
SAL_RA9263AA	CFSAN083986	Cerro	0	0	0	0	0	0	0	0	0	0	0
SAL_RA9417AA	CFSAN084061	Cerro	0	0	0	0	0	0	0	0	0	0	0
SAL_RA9897AA	SAL-18-VL-LA-KY-0006	Cerro	0	0	0	0	0	0	0	0	0	0	0
SAL_SA0559AA	OH-18-11242	Cerro	0	0	0	0	0	0	0	0	0	0	0
SAL_SA0383AA		Cerro	0	0	0	0	0	0	0	0	0	0	0
SAL_RA9980AA	FSIS11812445	Cerro	0	0	0	0	0	0	0	0	0	0	0
SAL_RA9954AA	FSIS11812442	Cerro	0	0	0	0	0	0	0	0	0	0	0
SAL_SA0895AA	FSIS11812674	Cerro	0	0	0	0	0	0	0	0	0	0	0
SAL_SA0896AA	FSIS11812684	Cerro	0	0	0	0	0	0	0	0	0	0	0
SAL_SA0897AA	FSIS11812683	Cerro	0	0	0	0	0	0	0	0	0	0	0
SAL_SA1155AA	FSIS11812842	Cerro	0	0	0	0	0	0	0	0	0	0	0
SAL_SA1144AA	FSIS11813012	Cerro	0	0	0	0	0	0	0	0	0	0	0
SAL_SA2523AA	OH-18-10058	Cerro	0	0	0	0	0	0	0	0	0	0	0
SAL_SA2524AA	OH-18-647	Cerro	0	0	0	0	0	0	0	0	0	0	0
SAL_SA2498AA	AG18-0022	Cerro	0	0	0	0	0	0	0	0	0	0	0
SAL_SA2583AA	FSIS11812981	Cerro	0	0	0	0	0	0	0	0	0	0	0
SAL_SA2582AA	FSIS11812393	Cerro	0	0	0	0	0	0	0	0	0	0	0
SAL_SA2618AA	FSIS11812971	Cerro	0	0	0	0	0	0	0	0	0	0	0
SAL_SA2634AA	FSIS11812855	Cerro	0	0	0	0	0	0	0	0	0	0	0
SAL_SA2637AA	FSIS11812848	Cerro	0	0	0	0	0	0	0	0	0	0	0
SAL_SA2617AA	FSIS11812847	Cerro	0	0	0	0	0	0	0	0	0	0	0
SAL_SA2701AA	FSIS11813075	Cerro	0	0	0	0	0	0	0	0	0	0	0
SAL_SA2858AA	FSIS11813213	Cerro	0	0	0	0	0	0	0	0	0	0	0
SAL_SA2852AA	FSIS11813206	Cerro	0	0	0	0	0	0	0	0	0	0	0
SAL_SA2848AA	FSIS11813212	Cerro	0	0	0	0	0	0	0	0	0	0	0
SAL_SA3668AA	FSIS11813396	Cerro	0	0	0	0	0	0	0	0	0	0	0
SAL_SA3651AA	FSIS11813667	Cerro	0	0	0	0	0	0	0	0	0	0	0
SAL_SA3644AA	FSIS11813661	Cerro	0	0	0	0	0	0	0	0	0	0	0
SAL_SA3734AA	FSIS11813397	Cerro	0	0	0	0	0	0	0	0	0	0	0
SAL_SA4051AA	FSIS31800991	Cerro	0	0	0	0	0	0	0	0	0	0	0
SAL_SA4332AA	FSIS31801018	Cerro	0	0	0	0	0	0	0	0	0	0	0
SAL_SA4295AA	FSIS11813886	Cerro	0	0	0	0	0	0	0	0	0	0	0
SAL_SA5776AA	FSIS11813909	Cerro	0	0	0	0	0	0	0	0	0	0	0
SAL_SA4799AA	579255	Cerro	0	0	0	0	0	0	0	0	0	0	0
SAL_SA6044AA	FSIS11813907	Cerro	0	0	0	0	0	0	0	0	0	0	0
SAL_SA6105AA	595627	Cerro	0	0	0	0	0	0	0	0	0	0	0

SAL_SA6513AA	FSIS11813991	Cerro	0	0	0	0	0	0	0	0	0	0	0
SAL_SA7501AA	572026	Cerro	0	0	0	0	0	0	0	0	0	0	0
SAL_SA7358AA	FSIS11813693	Cerro	0	0	0	0	0	0	0	0	0	0	0
SAL_SA8939AA	607843	Cerro	0	0	0	0	0	0	0	0	0	0	0
SAL_SA9540AA	442151	Cerro	0	0	0	0	0	0	0	0	0	0	0
SAL_SA9642AA	FSIS11814293	Cerro	0	0	0	0	0	0	0	0	0	0	0
SAL_TA0172AA	FDA00001091	Cerro	0	0	0	0	0	0	0	0	0	0	0
SAL_TA0121AA	FSIS31801111	Cerro	0	0	0	0	0	0	0	0	0	0	0
SAL_TA0949AA	615601	Cerro	0	0	0	0	0	0	0	0	0	0	0
SAL_TA0777AA	610778	Cerro	0	0	0	0	0	0	0	0	0	0	0
SAL_TA1375AA	FSIS11814640	Cerro	0	0	0	0	0	0	0	0	0	0	0
SAL_TA1371AA	FSIS11814650	Cerro	0	0	0	0	0	0	0	0	0	0	0
SAL_TA1293AA	OH-18-7669	Cerro	0	0	0	0	0	0	0	0	0	0	0
SAL_TA1271AA	OH-18-7266	Cerro	0	0	0	0	0	0	0	0	0	0	0
SAL_TA1275AA	OH-18-6069	Cerro	0	0	0	0	0	0	0	0	0	0	0
SAL_TA1273AA	OH-18-7116	Cerro	0	0	0	0	0	0	0	0	0	0	0
SAL_TA1274AA	OH-18-20386	Cerro	0	0	0	0	0	0	0	0	0	0	0
SAL_TA2017AA	FSIS11814850	Cerro	0	0	0	0	0	0	0	0	0	0	0
SAL_TA2012AA	FSIS11814856	Cerro	0	0	0	0	0	0	0	0	0	0	0
SAL_TA2376AA	624390	Cerro	0	0	0	0	0	0	0	0	0	0	0
SAL_TA2682AA	FSIS11814811	Cerro	0	0	0	0	0	0	0	0	0	0	0
SAL_TA2686AA	FSIS11814812	Cerro	0	0	0	0	0	0	0	0	0	0	0
SAL_TA2642AA	FSIS11814945	Cerro	0	0	0	0	0	0	0	0	0	0	0
SAL_TA2637AA	SAL-18-VL-LA-ND-0013	Cerro	0	0	0	0	0	0	0	0	0	0	0
SAL_TA3037AA	FSIS11815082	Cerro	0	0	0	0	0	0	0	0	0	0	0
SAL_TA4198AA	56-31-18-8101179-A	Cerro	0	0	0	0	0	0	0	0	0	0	0
SAL_TA5343AA	627366	Cerro	0	0	0	0	0	0	0	0	0	0	0
SAL_TA6601AA	SAL-18-VL-OH-IN-0017	Cerro	0	0	0	0	0	0	0	0	0	0	0
SAL_TA6615AA	OH-18-22479	Cerro	0	0	0	0	0	0	0	0	0	0	0
SAL_TA6605AA	OH-18-21765	Cerro	0	0	0	0	0	0	0	0	0	0	0
SAL_TA6581AA	FSIS31801191	Cerro	0	0	0	0	0	0	0	0	0	0	0
SAL_TA6591AA	AL-18-VL-NY-UP-NBC-00	Cerro	0	0	0	0	0	0	0	0	0	0	0
SAL_TA6597AA	AL-18-VL-NY-UP-NBC-00	Cerro	0	0	0	0	0	0	0	0	0	0	0
SAL_TA6430AA	FSIS11815364	Cerro	0	0	0	0	0	0	0	0	0	0	0
SAL_TA6794AA	FSIS11815509	Cerro	0	0	0	0	0	0	0	0	0	0	0
SAL_TA6782AA	FSIS11815519	Cerro	0	0	0	0	0	0	0	0	0	0	0
SAL_TA6984AA	CFSAN087679	Cerro	0	0	0	0	0	0	0	0	0	0	0
SAL_TA7134AA	CFSAN087629	Cerro	0	0	0	0	0	0	0	0	0	0	0
SAL_TA7155AA	CFSAN087648	Cerro	0	0	0	0	0	0	0	0	0	0	0
SAL_TA7174AA	CFSAN087705	Cerro	0	0	0	0	0	0	0	0	0	0	0
SAL_TA7491AA	634639	Cerro	0	0	0	0	0	0	0	0	0	0	0
SAL_TA6599AA	OH-18-22168	Cerro	0	0	0	0	0	0	0	0	0	0	0
SAL_PA2549AA	FSIS11807547	Cerro	0	0	0	0	0	0	0	0	0	0	0
SAL_GA7755AA	FSIS1607392	Cerro	0	0	0	0	0	0	0	0	0	0	0
SAL_OA1403AA	FSIS11807707	Cerro	0	0	0	0	0	0	0	0	0	0	0
SAL_TA8478AA	637889	Cerro	0	0	0	0	0	0	0	0	0	0	0
SAL_TA8460AA	638653	Cerro	0	0	0	0	0	0	0	0	0	0	0
SAL_TA8613AA	ADRDL-240	Cerro	0	0	0	0	0	0	0	0	0	0	0
SAL_TA8604AA	ADRDL-241	Cerro	0	0	0	0	0	0	0	0	0	0	0
SAL_TA8600AA	ADRDL-234	Cerro	0	0	0	0	0	0	0	0	0	0	0
SAL_TA8610AA	ADRDL-243	Cerro	0	0	0	0	0	0	0	0	0	0	0
SAL_TA8611AA	ADRDL-232	Cerro	0	0	0	0	0	0	0	0	0	0	0
SAL_TA8616AA	ADRDL-238	Cerro	0	0	0	0	0	0	0	0	0	0	0
SAL_TA8615AA	ADRDL-233	Cerro	0	0	0	0	0	0	0	0	0	0	0
SAL_TA8617AA	ADRDL-237	Cerro	0	0	0	0	0	0	0	0	0	0	0
SAL_TA8618AA	ADRDL-236	Cerro	0	0	0	0	0	0	0	0	0	0	0
SAL_TA8598AA	ADRDL-231	Cerro	0	0	0	0	0	0	0	0	0	0	0
SAL_TA8609AA	ADRDL-267	Cerro	0	0	0	0	0	0	0	0	0	0	0
SAL_TA8590AA	ADRDL-242	Cerro	0	0	0	0	0	0	0	0	0	0	0
SAL_TA8595AA	ADRDL-268	Cerro	0	0	0	0	0	0	0	0	0	0	0
SAL_TA8760AA	FSIS31801240	Cerro	0	0	0	0	0	0	0	0	0	0	0
SAL_TA8584AA	ADRDL-230	Cerro	0	0	0	0	0	0	0	0	0	0	0
SAL_TA8798AA	FSIS11815725	Cerro	0	0	0	0	0	0	0	0	0	0	0
SAL_TA8832AA	FSIS11815704	Cerro	0	0	0	0	0	0	0	0	0	0	0
SAL_OA1411AA	FSIS11807709	Cerro	0	0	0	0	0	0	0	0	0	0	0
SAL_OA1414AA	FSIS11807689	Cerro	0	0	0	0	0	0	0	0	0	0	0
SAL_OA1440AA	FSIS11807275	Cerro	0	0	0	0	0	0	0	0	0	0	0
SAL_OA2101AA	FSIS31800175	Cerro	0	0	0	0	0	0	0	0	0	0	0
SAL_PA1246AA	FSIS11808870	Cerro	0	0	0	0	0	0	0	0	0	0	0
SAL_PA1607AA	FSIS21821433	Cerro	0	0	0	0	0	0	0	0	0	0	0
SAL_PA1783AA	FSIS11809069	Cerro	0	0	0	0	0	0	0	0	0	0	0
SAL_PA2529AA	FSIS11808645	Cerro	0	0	0	0	0	0	0	0	0	0	0
SAL_PA2741AA	PNUSAS038802	Cerro	0	0	0	0	0	0	0	0	0	0	0

SAL_PA2550AA	FSIS11808779	Cerro	0	0	0	0	0	0	0	0	0	0	0
SAL_PA3038AA	FSIS11809373	Cerro	0	0	0	0	0	0	0	0	0	0	0
SAL_PA3498AA	SAL-17-VL-NY-FL-0027	Cerro	0	0	0	0	0	0	0	0	0	0	0
SAL_PA3653AA	FSIS11809228	Cerro	0	0	0	0	0	0	0	0	0	0	0
SAL_PA6060AA	FSIS11809720	Cerro	0	0	0	0	0	0	0	0	0	0	0
SAL_TA9637AA	FSIS11815940	Cerro	0	0	0	0	0	0	0	0	0	0	0
SAL_TA9795AA	FSIS11816110	Cerro	0	0	0	0	0	0	0	0	0	0	0
SAL_UA2211AA	OH-18-18060	Cerro	0	0	0	0	0	0	0	0	0	0	0
SAL_UA2204AA	OH-18-17930	Cerro	0	0	0	0	0	0	0	0	0	0	0
SAL_UA2694AA	649005	Cerro	0	0	0	0	0	0	0	0	0	0	0
SAL_UA2982AA	643150	Cerro	0	0	0	0	0	0	0	0	0	0	0
SAL_UA3105AA	20_Cerro_b	Cerro	0	0	0	0	0	0	0	0	0	0	0
SAL_UA6902AA	AG18-0104	Cerro	0	0	0	0	0	0	0	0	0	0	0
SAL_UA6921AA	AG18-0095	Cerro	0	0	0	0	0	0	0	0	0	0	0
SAL_UA6874AA	AG18-0120	Cerro	0	0	0	0	0	0	0	0	0	0	0
SAL_UA6892AA	AG18-0079	Cerro	0	0	0	0	0	0	0	0	0	0	0
SAL_UA6896AA	AG18-0115	Cerro	0	0	0	0	0	0	0	0	0	0	0
SAL_UA6841AA	AG18-0129	Cerro	0	0	0	0	0	0	0	0	0	0	0
SAL_UA6854AA	AG18-0133	Cerro	0	0	0	0	0	0	0	0	0	0	0
SAL_UA6846AA	AG18-0097	Cerro	0	0	0	0	0	0	0	0	0	0	0
SAL_UA6857AA	AG18-0076	Cerro	0	0	0	0	0	0	0	0	0	0	0
SAL_UA6863AA	AG18-0075	Cerro	0	0	0	0	0	0	0	0	0	0	0
SAL_UA6865AA	OH-18-23227	Cerro	0	0	0	0	0	0	0	0	0	0	0
SAL_UA6809AA	FSIS11816932	Cerro	0	0	0	0	0	0	0	0	0	0	0
SAL_UA6813AA	FSIS11816931	Cerro	0	0	0	0	0	0	0	0	0	0	0
SAL_UA7240AA	FSIS11816687	Cerro	0	0	0	0	0	0	0	0	0	0	0
SAL_UA2969AA	650021	Cerro	0	0	0	0	0	0	0	0	0	0	0
SAL_UA4479AA	OH-15-17370	Cerro	0	0	0	0	0	0	0	0	0	0	0
SAL_UA4698AA	OH-15-24377	Cerro	0	0	0	0	0	0	0	0	0	0	0
SAL_UA4690AA	OH-15-24743	Cerro	0	0	0	0	0	0	0	0	0	0	0
SAL_UA4689AA	OH-15-19685	Cerro	0	0	0	0	0	0	0	0	0	0	0
SAL_UA4687AA	OH-15-19898	Cerro	0	0	0	0	0	0	0	0	0	0	0
SAL_UA4712AA	OH-15-19695	Cerro	0	0	0	0	0	0	0	0	0	0	0
SAL_UA4683AA	OH-15-23889-E	Cerro	0	0	0	0	0	0	0	0	0	0	0
SAL_UA4711AA	OH-15-19898-B	Cerro	0	0	0	0	0	0	0	0	0	0	0
SAL_UA4682AA	OH-15-20229	Cerro	0	0	0	0	0	0	0	0	0	0	0
SAL_UA4708AA	OH-15-26628	Cerro	0	0	0	0	0	0	0	0	0	0	0
SAL_UA4705AA	OH-15-24866	Cerro	0	0	0	0	0	0	0	0	0	0	0
SAL_UA4700AA	OH-15-24542	Cerro	0	0	0	0	0	0	0	0	0	0	0
SAL_UA5182AA	FSIS11816370	Cerro	0	0	0	0	0	0	0	0	0	0	0
SAL_UA5709AA	587361	Cerro	0	0	0	0	0	0	0	0	0	0	0
SAL_UA5911AA	FSIS11816567	Cerro	0	0	0	0	0	0	0	0	0	0	0
SAL_UA5942AA	FSIS11816501	Cerro	0	0	0	0	0	0	0	0	0	0	0
SAL_UA6671AA	AG18-0053	Cerro	0	0	0	0	0	0	0	0	0	0	0
SAL_UA6686AA	AG18-0067	Cerro	0	0	0	0	0	0	0	0	0	0	0
SAL_UA6685AA	AG18-0048	Cerro	0	0	0	0	0	0	0	0	0	0	0
SAL_UA7107AA	AG18-0039	Cerro	0	0	0	0	0	0	0	0	0	0	0
SAL_UA7065AA	AG18-0038	Cerro	0	0	0	0	0	0	0	0	0	0	0
SAL_UA7080AA	AG18-0047	Cerro	0	0	0	0	0	0	0	0	0	0	0
SAL_UA7093AA	AG18-0056	Cerro	0	0	0	0	0	0	0	0	0	0	0
SAL_UA7900AA	ADRDL-2327	Cerro	0	0	0	0	0	0	0	0	0	0	0
SAL_UA7906AA	ADRDL-2323	Cerro	0	0	0	0	0	0	0	0	0	0	0
SAL_UA7908AA	ADRDL-2358	Cerro	0	0	0	0	0	0	0	0	0	0	0
SAL_UA7891AA	ADRDL-2313	Cerro	0	0	0	0	0	0	0	0	0	0	0
SAL_UA7894AA	ADRDL-2354	Cerro	0	0	0	0	0	0	0	0	0	0	0
SAL_UA7899AA	ADRDL-2310	Cerro	0	0	0	0	0	0	0	0	0	0	0
SAL_UA7901AA	ADRDL-2318	Cerro	0	0	0	0	0	0	0	0	0	0	0
SAL_UA7904AA	ADRDL-2322	Cerro	0	0	0	0	0	0	0	0	0	0	0
SAL_UA7923AA	ADRDL-2329	Cerro	0	0	0	0	0	0	0	0	0	0	0
SAL_UA7897AA	ADRDL-2367	Cerro	0	0	0	0	0	0	0	0	0	0	0
SAL_UA7892AA	ADRDL-2303	Cerro	0	0	0	0	0	0	0	0	0	0	0
SAL_UA7896AA	ADRDL-2347	Cerro	0	0	0	0	0	0	0	0	0	0	0
SAL_UA7911AA	ADRDL-2353	Cerro	0	0	0	0	0	0	0	0	0	0	0
SAL_UA7898AA	ADRDL-2307	Cerro	0	0	0	0	0	0	0	0	0	0	0
SAL_UA7928AA	ADRDL-2359	Cerro	0	0	0	0	0	0	0	0	0	0	0
SAL_UA7915AA	ADRDL-2331	Cerro	0	0	0	0	0	0	0	0	0	0	0
SAL_UA7912AA	ADRDL-2364	Cerro	0	0	0	0	0	0	0	0	0	0	0
SAL_UA7876AA	ADRDL-2326	Cerro	0	0	0	0	0	0	0	0	0	0	0
SAL_UA7877AA	ADRDL-2356	Cerro	0	0	0	0	0	0	0	0	0	0	0
SAL_UA7881AA	ADRDL-2336	Cerro	0	0	0	0	0	0	0	0	0	0	0
SAL_UA7886AA	ADRDL-2335	Cerro	0	0	0	0	0	0	0	0	0	0	0
SAL_UA7842AA	OH-16-4703	Cerro	0	0	0	0	0	0	0	0	0	0	0
SAL_UA7859AA	ADRDL-2311	Cerro	0	0	0	0	0	0	0	0	0	0	0

SAL_UA7863AA	ADRDL-2357	Cerro	0	0	0	0	0	0	0	0	0	0	0
SAL_UA7868AA	ADRDL-2361	Cerro	0	0	0	0	0	0	0	0	0	0	0
SAL_UA7921AA	ADRDL-2328	Cerro	0	0	0	0	0	0	0	0	0	0	0
SAL_UA7932AA	ADRDL-2346	Cerro	0	0	0	0	0	0	0	0	0	0	0
SAL_UA7933AA	ADRDL-2345	Cerro	0	0	0	0	0	0	0	0	0	0	0
SAL_UA7836AA	ADRDL-2352	Cerro	0	0	0	0	0	0	0	0	0	0	0
SAL_UA7838AA	ADRDL-2341	Cerro	0	0	0	0	0	0	0	0	0	0	0
SAL_UA7840AA	ADRDL-2355	Cerro	0	0	0	0	0	0	0	0	0	0	0
SAL_UA7844AA	ADRDL-2362	Cerro	0	0	0	0	0	0	0	0	0	0	0
SAL_UA7846AA	ADRDL-2319	Cerro	0	0	0	0	0	0	0	0	0	0	0
SAL_UA7865AA	ADRDL-2368	Cerro	0	0	0	0	0	0	0	0	0	0	0
SAL_UA7940AA	ADRDL-2342	Cerro	0	0	0	0	0	0	0	0	0	0	0
SAL_UA7848AA	ADRDL-2306	Cerro	0	0	0	0	0	0	0	0	0	0	0
SAL_UA7873AA	ADRDL-2330	Cerro	0	0	0	0	0	0	0	0	0	0	0
SAL_UA7942AA	ADRDL-2348	Cerro	0	0	0	0	0	0	0	0	0	0	0
SAL_UA7895AA	ADRDL-2304	Cerro	0	0	0	0	0	0	0	0	0	0	0
SAL_UA7910AA	ADRDL-2309	Cerro	0	0	0	0	0	0	0	0	0	0	0
SAL_UA7917AA	ADRDL-2360	Cerro	0	0	0	0	0	0	0	0	0	0	0
SAL_UA7924AA	ADRDL-2325	Cerro	0	0	0	0	0	0	0	0	0	0	0
SAL_UA7926AA	ADRDL-2321	Cerro	0	0	0	0	0	0	0	0	0	0	0
SAL_UA7931AA	ADRDL-2332	Cerro	0	0	0	0	0	0	0	0	0	0	0
SAL_UA7936AA	ADRDL-2363	Cerro	0	0	0	0	0	0	0	0	0	0	0
SAL_UA7845AA	ADRDL-2339	Cerro	0	0	0	0	0	0	0	0	0	0	0
SAL_UA7856AA	ADRDL-2333	Cerro	0	0	0	0	0	0	0	0	0	0	0
SAL_UA7858AA	ADRDL-2349	Cerro	0	0	0	0	0	0	0	0	0	0	0
SAL_UA7860AA	ADRDL-2314	Cerro	0	0	0	0	0	0	0	0	0	0	0
SAL_UA7861AA	ADRDL-2351	Cerro	0	0	0	0	0	0	0	0	0	0	0
SAL_UA7823AA	ADRDL-2312	Cerro	0	0	0	0	0	0	0	0	0	0	0
SAL_UA7825AA	ADRDL-2365	Cerro	0	0	0	0	0	0	0	0	0	0	0
SAL_UA7826AA	ADRDL-2320	Cerro	0	0	0	0	0	0	0	0	0	0	0
SAL_UA7828AA	OH-16-721-1	Cerro	0	0	0	0	0	0	0	0	0	0	0
SAL_UA7831AA	ADRDL-2350	Cerro	0	0	0	0	0	0	0	0	0	0	0
SAL_UA7832AA	ADRDL-2337	Cerro	0	0	0	0	0	0	0	0	0	0	0
SAL_UA7850AA	ADRDL-2366	Cerro	0	0	0	0	0	0	0	0	0	0	0
SAL_UA7851AA	ADRDL-2308	Cerro	0	0	0	0	0	0	0	0	0	0	0
SAL_UA7883AA	ADRDL-2316	Cerro	0	0	0	0	0	0	0	0	0	0	0
SAL_UA7816AA	OH-16-1348	Cerro	0	0	0	0	0	0	0	0	0	0	0
SAL_UA7798AA	OH-16-7003	Cerro	0	0	0	0	0	0	0	0	0	0	0
SAL_UA7756AA	FSIS11816759	Cerro	0	0	0	0	0	0	0	0	0	0	0
SAL_UA7745AA	FSIS11816984	Cerro	0	0	0	0	0	0	0	0	0	0	0
SAL_UA7487AA	FSIS11917056	Cerro	0	0	0	0	0	0	0	0	0	0	0
SAL_UA8062AA	ADRDL-2378	Cerro	0	0	0	0	0	0	0	0	0	0	0
SAL_UA8068AA	ADRDL-2372	Cerro	0	0	0	0	0	0	0	0	0	0	0
SAL_UA7857AA	ADRDL-2324	Cerro	0	0	0	0	0	0	0	0	0	0	0
SAL_UA7855AA	ADRDL-2305	Cerro	0	0	0	0	0	0	0	0	0	0	0
SAL_UA8067AA	ADRDL-2374	Cerro	0	0	0	0	0	0	0	0	0	0	0
SAL_UA7807AA	OH-16-4242	Cerro	0	0	0	0	0	0	0	0	0	0	0
SAL_UA8053AA	ADRDL-2381	Cerro	0	0	0	0	0	0	0	0	0	0	0
SAL_UA8049AA	ADRDL-2375	Cerro	0	0	0	0	0	0	0	0	0	0	0
SAL_UA8047AA	ADRDL-2377	Cerro	0	0	0	0	0	0	0	0	0	0	0
SAL_UA8048AA	ADRDL-2376	Cerro	0	0	0	0	0	0	0	0	0	0	0
SAL_UA8059AA	ADRDL-2379	Cerro	0	0	0	0	0	0	0	0	0	0	0
SAL_UA8058AA	ADRDL-2380	Cerro	0	0	0	0	0	0	0	0	0	0	0
SAL_UA8043AA	ADRDL-2371	Cerro	0	0	0	0	0	0	0	0	0	0	0
SAL_UA8055AA	ADRDL-2370	Cerro	0	0	0	0	0	0	0	0	0	0	0
SAL_UA8050AA	ADRDL-2369	Cerro	0	0	0	0	0	0	0	0	0	0	0
SAL_UA7804AA	OH-16-2044	Cerro	0	0	0	0	0	0	0	0	0	0	0
SAL_VA6960AA	VA-WGS-18110	Cerro	0	0	0	0	0	0	0	0	0	0	0
SAL_UA8240AA	WMD-10	Cerro	0	0	0	0	0	0	0	0	0	0	0
SAL_UA8243AA	WMD-13	Cerro	0	0	0	0	0	0	0	0	0	0	0
SAL_UA8912AA	AG18-0088	Cerro	0	0	0	0	0	0	0	0	0	0	0
SAL_UA8909AA	AG19-0015	Cerro	0	0	0	0	0	0	0	0	0	0	0
SAL_UA8911AA	AG18-0084	Cerro	0	0	0	0	0	0	0	0	0	0	0
SAL_UA8903AA	AG18-0098	Cerro	0	0	0	0	0	0	0	0	0	0	0
SAL_UA8902AA	AG19-0014	Cerro	0	0	0	0	0	0	0	0	0	0	0
SAL_UA8877AA	AG19-0007	Cerro	0	0	0	0	0	0	0	0	0	0	0
SAL_UA8881AA	AG19-0020	Cerro	0	0	0	0	0	0	0	0	0	0	0
SAL_UA8886AA	AG19-0010	Cerro	0	0	0	0	0	0	0	0	0	0	0
SAL_UA8880AA	AG19-0021	Cerro	0	0	0	0	0	0	0	0	0	0	0
SAL_UA8889AA	AG18-0090	Cerro	0	0	0	0	0	0	0	0	0	0	0
SAL_UA8896AA	AG19-0019	Cerro	0	0	0	0	0	0	0	0	0	0	0
SAL_VA0227AA	FSIS21923294	Cerro	0	0	0	0	0	0	0	0	0	0	0
SAL_UA9759AA	646001	Cerro	0	0	0	0	0	0	0	0	0	0	0

SAL_VA0699AA	201810865	Cerro	0	0	0	0	0	0	0	0	0	0	0
SAL_VA0530AA	ADRDL-2221	Cerro	0	0	0	0	0	0	0	0	0	0	0
SAL_VA7539AA	201705804	Cerro	0	0	0	0	0	0	0	0	0	0	0
SAL_VA1837AA	PNUSAS040586	Cerro	0	0	0	0	0	0	0	0	0	0	0
SAL_VA1522AA	FSIS11917445	Cerro	0	0	0	0	0	0	0	0	0	0	0
SAL_VA4298AA	FSIS11705328	Cerro	0	0	0	0	0	0	0	0	0	0	0
SAL_VA6519AA	FSL R8-3192	Cerro	0	0	0	0	0	0	0	0	0	0	0
SAL_VA6521AA	BOV2-0012	Cerro	0	0	0	0	0	0	0	0	0	0	0
SAL_VA6528AA	BOV1-0128	Cerro	0	0	0	0	0	0	0	0	0	0	0
SAL_VA6525AA	BOV2-0001	Cerro	0	0	0	0	0	0	0	0	0	0	0
SAL_VA6530AA	BOV1-0121	Cerro	0	0	0	0	0	0	0	0	0	0	0
SAL_VA6532AA	BOV1-0119	Cerro	0	0	0	0	0	0	0	0	0	0	0
SAL_VA6538AA	BOV1-0095	Cerro	0	0	0	0	0	0	0	0	0	0	0
SAL_VA6524AA	BOV2-0002	Cerro	0	0	0	0	0	0	0	0	0	0	0
SAL_VA6536AA	BOV1-0108	Cerro	0	0	0	0	0	0	0	0	0	0	0
SAL_VA6522AA	BOV2-0011	Cerro	0	0	0	0	0	0	0	0	0	0	0
SAL_VA6529AA	BOV1-0124	Cerro	0	0	0	0	0	0	0	0	0	0	0
SAL_VA6520AA	FSL R8-3803	Cerro	0	0	0	0	0	0	0	0	0	0	0
SAL_VA6534AA	BOV1-0116	Cerro	0	0	0	0	0	0	0	0	0	0	0
SAL_VA6542AA	BOV1-0062	Cerro	0	0	0	0	0	0	0	0	0	0	0
SAL_VA6523AA	BOV2-0003	Cerro	0	0	0	0	0	0	0	0	0	0	0
SAL_VA6543AA	BOV1-0059	Cerro	0	0	0	0	0	0	0	0	0	0	0
SAL_VA6531AA	BOV1-0120	Cerro	0	0	0	0	0	0	0	0	0	0	0
SAL_VA6527AA	BOV1-0217	Cerro	0	0	0	0	0	0	0	0	0	0	0
SAL_VA6540AA	BOV1-0077	Cerro	0	0	0	0	0	0	0	0	0	0	0
SAL_VA6537AA	BOV1-0003	Cerro	0	0	0	0	0	0	0	0	0	0	0
SAL_VA6544AA	BOV1-0042	Cerro	0	0	0	0	0	0	0	0	0	0	0
SAL_VA6539AA	BOV1-0093	Cerro	0	0	0	0	0	0	0	0	0	0	0
SAL_VA6549AA	BOV1-0001	Cerro	0	0	0	0	0	0	0	0	0	0	0
SAL_VA6548AA	BOV1-0002	Cerro	0	0	0	0	0	0	0	0	0	0	0
SAL_VA6546AA	BOV1-0030	Cerro	0	0	0	0	0	0	0	0	0	0	0
SAL_VA6547AA	BOV1-0254	Cerro	0	0	0	0	0	0	0	0	0	0	0
SAL_VA6545AA	BOV1-0037	Cerro	0	0	0	0	0	0	0	0	0	0	0
SAL_VA6541AA	BOV1-0071	Cerro	0	0	0	0	0	0	0	0	0	0	0
SAL_VA6533AA	BOV1-0118	Cerro	0	0	0	0	0	0	0	0	0	0	0
SAL_VA6526AA	BOV1-0004	Cerro	0	0	0	0	0	0	0	0	0	0	0
SAL_VA6535AA	BOV1-0115	Cerro	0	0	0	0	0	0	0	0	0	0	0
SAL_VA6474AA	FSL R9-1362	Cerro	0	0	0	0	0	0	0	0	0	0	0
SAL_VA6472AA	FSL R9-1899	Cerro	0	0	0	0	0	0	0	0	0	0	0
SAL_VA6480AA	FSL R9-3505	Cerro	0	0	0	0	0	0	0	0	0	0	0
SAL_VA6478AA	FSL A5-0218	Cerro	0	0	0	0	0	0	0	0	0	0	0
SAL_VA6481AA	FSL R9-3612	Cerro	0	0	0	0	0	0	0	0	0	0	0
SAL_VA6484AA	FSL R9-3074	Cerro	0	0	0	0	0	0	0	0	0	0	0
SAL_VA6483AA	FSL R9-3026	Cerro	0	0	0	0	0	0	0	0	0	0	0
SAL_VA6486AA	FSL R9-1131	Cerro	0	0	0	0	0	0	0	0	0	0	0
SAL_VA6476AA	FSL R8-8941	Cerro	0	0	0	0	0	0	0	0	0	0	0
SAL_VA6495AA	FSL S10-1244	Cerro	0	0	0	0	0	0	0	0	0	0	0
SAL_VA6477AA	FSL R8-8603	Cerro	0	0	0	0	0	0	0	0	0	0	0
SAL_VA6475AA	FSL R9-1361	Cerro	0	0	0	0	0	0	0	0	0	0	0
SAL_VA6479AA	FSL R9-2601	Cerro	0	0	0	0	0	0	0	0	0	0	0
SAL_VA6473AA	FSL R9-1838	Cerro	0	0	0	0	0	0	0	0	0	0	0
SAL_VA6488AA	FSL S10-1762	Cerro	0	0	0	0	0	0	0	0	0	0	0
SAL_VA6487AA	FSL R9-1068	Cerro	0	0	0	0	0	0	0	0	0	0	0
SAL_VA6489AA	FSL S10-1742	Cerro	0	0	0	0	0	0	0	0	0	0	0
SAL_VA6485AA	FSL R9-2513	Cerro	0	0	0	0	0	0	0	0	0	0	0
SAL_VA6490AA	FSL R8-9775	Cerro	0	0	0	0	0	0	0	0	0	0	0
SAL_VA6499AA	FSL R8-9087	Cerro	0	0	0	0	0	0	0	0	0	0	0
SAL_VA6492AA	FSL R9-0520	Cerro	0	0	0	0	0	0	0	0	0	0	0
SAL_VA6498AA	FSL R8-8605	Cerro	0	0	0	0	0	0	0	0	0	0	0
SAL_VA6501AA	FSL S10-0229	Cerro	0	0	0	0	0	0	0	0	0	0	0
SAL_VA6497AA	FSL R8-8203	Cerro	0	0	0	0	0	0	0	0	0	0	0
SAL_VA6500AA	FSL S10-0550	Cerro	0	0	0	0	0	0	0	0	0	0	0
SAL_VA6496AA	FSL R8-7941	Cerro	0	0	0	0	0	0	0	0	0	0	0
SAL_VA6504AA	BOV1-0014	Cerro	0	0	0	0	0	0	0	0	0	0	0
SAL_VA6505AA	FSL R8-4819	Cerro	0	0	0	0	0	0	0	0	0	0	0
SAL_VA6503AA	FSL R8-5047	Cerro	0	0	0	0	0	0	0	0	0	0	0
SAL_VA6502AA	FSL R8-5925	Cerro	0	0	0	0	0	0	0	0	0	0	0
SAL_VA6507AA	FSL R8-4516	Cerro	0	0	0	0	0	0	0	0	0	0	0
SAL_VA6506AA	FSL R8-7279	Cerro	0	0	0	0	0	0	0	0	0	0	0
SAL_VA6516AA	FSL R8-3446	Cerro	0	0	0	0	0	0	0	0	0	0	0
SAL_VA6508AA	FSL R8-3416	Cerro	0	0	0	0	0	0	0	0	0	0	0
SAL_VA6512AA	FSL R8-3655	Cerro	0	0	0	0	0	0	0	0	0	0	0
SAL_VA6510AA	FSL R8-3438	Cerro	0	0	0	0	0	0	0	0	0	0	0

SAL_VA6517AA	FSL R8-3460	Cerro	0	0	0	0	0	0	0	0	0	0	0
SAL_VA6511AA	FSL R8-4025	Cerro	0	0	0	0	0	0	0	0	0	0	0
SAL_VA6509AA	FSL R8-3146	Cerro	0	0	0	0	0	0	0	0	0	0	0
SAL_VA6513AA	FSL R8-3434	Cerro	0	0	0	0	0	0	0	0	0	0	0
SAL_VA6515AA	BOV1-0008	Cerro	0	0	0	0	0	0	0	0	0	0	0
SAL_VA6518AA	FSL R8-3195	Cerro	0	0	0	0	0	0	0	0	0	0	0
SAL_VA6514AA	FSL R8-3449	Cerro	0	0	0	0	0	0	0	0	0	0	0
SAL_VA1845AA	441464	Cerro	0	0	0	0	0	0	0	0	0	0	0
SAL_VA2638AA	AG19-0057	Cerro	0	0	0	0	0	0	0	0	0	0	0
SAL_VA2829AA	AG19-0092	Cerro	0	0	0	0	0	0	0	0	0	0	0
SAL_VA2818AA	AG19-0069	Cerro	0	0	0	0	0	0	0	0	0	0	0
SAL_VA2821AA	AG19-0093	Cerro	0	0	0	0	0	0	0	0	0	0	0
SAL_VA3360AA	AG19-0045	Cerro	0	0	0	0	0	0	0	0	0	0	0
SAL_VA3371AA	AG19-0052	Cerro	0	0	0	0	0	0	0	0	0	0	0
SAL_VA3367AA	AG19-0065	Cerro	0	0	0	0	0	0	0	0	0	0	0
SAL_VA3382AA	AG19-0077	Cerro	0	0	0	0	0	0	0	0	0	0	0
SAL_VA3385AA	AG19-0070	Cerro	0	0	0	0	0	0	0	0	0	0	0
SAL_VA3386AA	AG19-0049	Cerro	0	0	0	0	0	0	0	0	0	0	0
SAL_VA3389AA	AG19-0040	Cerro	0	0	0	0	0	0	0	0	0	0	0
SAL_VA3336AA	AG19-0034	Cerro	0	0	0	0	0	0	0	0	0	0	0
SAL_VA3341AA	AG19-0071	Cerro	0	0	0	0	0	0	0	0	0	0	0
SAL_VA3337AA	AG19-0074	Cerro	0	0	0	0	0	0	0	0	0	0	0
SAL_VA3332AA	AG19-0091	Cerro	0	0	0	0	0	0	0	0	0	0	0
SAL_VA3344AA	AG19-0032	Cerro	0	0	0	0	0	0	0	0	0	0	0
SAL_VA3355AA	AG19-0090	Cerro	0	0	0	0	0	0	0	0	0	0	0
SAL_VA3334AA	AG19-0054	Cerro	0	0	0	0	0	0	0	0	0	0	0
SAL_VA3333AA	AG19-0046	Cerro	0	0	0	0	0	0	0	0	0	0	0
SAL_VA6469AA	FSL R9-1901	Cerro	0	0	0	0	0	0	0	0	0	0	0
SAL_VA6471AA	BOV1-0017	Cerro	0	0	0	0	0	0	0	0	0	0	0
SAL_VA6491AA	FSL R9-0539	Cerro	0	0	0	0	0	0	0	0	0	0	0
SAL_VA6468AA	FSL R9-1902	Cerro	0	0	0	0	0	0	0	0	0	0	0
SAL_VA6470AA	FSL R9-1900	Cerro	0	0	0	0	0	0	0	0	0	0	0
SAL_VA6482AA	BOV1-0016	Cerro	0	0	0	0	0	0	0	0	0	0	0
SAL_VA6493AA	BOV1-0015	Cerro	0	0	0	0	0	0	0	0	0	0	0
SAL_VA1951AA	439512	Cerro	0	0	0	0	0	0	0	0	0	0	0
SAL_VA6464AA	BOV1-0253	Cerro	0	0	0	0	0	0	0	0	0	0	0
SAL_VA6466AA	FSL R9-1904	Cerro	0	0	0	0	0	0	0	0	0	0	0
SAL_VA6467AA	FSL R9-1903	Cerro	0	0	0	0	0	0	0	0	0	0	0
SAL_VA6465AA	FSL R9-1905	Cerro	0	0	0	0	0	0	0	0	0	0	0
SAL_VA5877AA	SA20103397	Cerro	0	0	0	0	0	0	0	0	0	0	0
SAL_VA5832AA	SA20094721	Cerro	0	0	0	0	0	0	0	0	0	0	0
SAL_VA5810AA	SA20031084	Cerro	0	0	0	0	0	0	0	0	0	0	0
SAL_VA6951AA	VA-WGS-18113	Cerro	0	0	0	0	0	0	0	0	0	0	0
SAL_VA4315AA	OH-17-6134	Cerro	0	0	0	0	0	0	0	0	0	0	0
SAL_VA7699AA	FSIS11917572	Cerro	0	0	0	0	0	0	0	0	0	0	0
SAL_VA7721AA	FSIS11917546	Cerro	0	0	0	0	0	0	0	0	0	0	0
SAL_VA7970AA	FSIS11917760	Cerro	0	0	0	0	0	0	0	0	0	0	0
SAL_VA8048AA	FSIS31901588	Cerro	0	0	0	0	0	0	0	0	0	0	0
SAL_VA8604AA	FSIS11918032	Cerro	0	0	0	0	0	0	0	0	0	0	0
SAL_VA4158AA	CDPH_CSalm030	Cerro	0	0	0	0	0	0	0	0	0	0	0
SAL_VA9489AA	692628	Cerro	0	0	0	0	0	0	0	0	0	0	0
SAL_VA4131AA	CDPH_CSalm323	Cerro	0	0	0	0	0	0	0	0	0	0	0
SAL_WA1085AA	FSIS11918020	Cerro	0	0	0	0	0	0	0	0	0	0	0
SAL_WA1092AA	FSIS11918323	Cerro	0	0	0	0	0	0	0	0	0	0	0
SAL_WA1084AA	FSIS11918018	Cerro	0	0	0	0	0	0	0	0	0	0	0
SAL_WA3190AA	GW_S04760-06	Cerro	0	0	0	0	0	0	0	0	0	0	0
SAL_WA3039AA	GW_S02983-08	Cerro	0	0	0	0	0	0	0	0	0	0	0
SAL_WA2410AA	GW_S02945-07	Cerro	0	0	0	0	0	0	0	0	0	0	0
SAL_WA1222AA	130410	Cerro	0	0	0	0	0	0	0	0	0	0	0
SAL_WA1574AA	FSIS11918964	Cerro	0	0	0	0	0	0	0	0	0	0	0
SAL_WA1581AA	FSIS11918945	Cerro	0	0	0	0	0	0	0	0	0	0	0
SAL_WA2099AA	AG19-0123	Cerro	0	0	0	0	0	0	0	0	0	0	0
SAL_WA2120AA	FSIS11919181	Cerro	0	0	0	0	0	0	0	0	0	0	0
SAL_WA2135AA	FSIS11919169	Cerro	0	0	0	0	0	0	0	0	0	0	0
SAL_WA2144AA	AL-18-VL-OH-PA-Ag-002	Cerro	0	0	0	0	0	0	0	0	0	0	0
SAL_WA2126AA	AG19-0112	Cerro	0	0	0	0	0	0	0	0	0	0	0
SAL_WA2131AA	AG19-0124	Cerro	0	0	0	0	0	0	0	0	0	0	0
SAL_WA2145AA	AL-18-VL-OH-PA-Ag-001	Cerro	0	0	0	0	0	0	0	0	0	0	0
SAL_WA1074AA	FSIS11918322	Cerro	0	0	0	0	0	0	0	0	0	0	0
SAL_WA1858AA	FSIS11918527	Cerro	0	0	0	0	0	0	0	0	0	0	0
SAL_VA6494AA	FSL S10-1411	Cerro	0	0	0	0	0	0	0	0	0	0	0
SAL_WA3488AA	CFSAN092331	Cerro	0	0	0	0	0	0	0	0	0	0	0
SAL_WA3577AA	CFSAN087562	Cerro	0	0	0	0	0	0	0	0	0	0	0

SAL_WA3579AA	AG19-0064	Cerro	0	0	0	0	0	0	0	0	0	0	0
SAL_WA3581AA	AG19-0044	Cerro	0	0	0	0	0	0	0	0	0	0	0
SAL_WA3585AA	AG19-0075	Cerro	0	0	0	0	0	0	0	0	0	0	0
SAL_WA3561AA	AG19-0106	Cerro	0	0	0	0	0	0	0	0	0	0	0
SAL_WA4021AA	FSIS11919189	Cerro	0	0	0	0	0	0	0	0	0	0	0
SAL_WA4025AA	FSIS11918686	Cerro	0	0	0	0	0	0	0	0	0	0	0
SAL_WA4030AA	FSIS11918533	Cerro	0	0	0	0	0	0	0	0	0	0	0
SAL_GA2482AA	BCW_1522	Champaign	0	0	0	0	0	0	0	0	0	0	0
SAL_EA3240AA	FDA00009478	Champaign	0	0	0	0	0	0	0	0	0	0	0
SAL_CA3483AA	6442	Chandans	0	0	0	0	0	0	0	0	0	0	0
SAL_SA3017AA	CFSAN070328	Chandans	0	0	0	0	0	0	0	0	0	0	0
SAL_DA8268AA	769324-sc-2010-09-29-9	Chester	0	0	0	0	0	0	0	0	0	0	0
SAL_DA6075AA	MOD1_Sal_849	Chester	0	0	0	0	0	0	0	0	0	0	0
SAL_CA8262AA	FDA00003766	Chester	0	0	0	0	0	0	0	0	0	0	0
SAL_CA2785AA	31624	Chester	0	0	0	0	0	0	0	0	0	0	0
SAL_CA4984AA	78627	Chester	0	0	0	0	0	0	0	0	0	0	0
SAL_EA7727AA	2277	Chester	0	0	0	0	0	0	0	0	0	0	0
SAL_CA9273AA	FDA00003818	Chester	0	0	0	0	0	0	0	0	0	0	0
SAL_CA3670AA	13823	Chester	0	0	0	0	0	0	0	0	0	0	0
SAL_FA4685AA	205631	Chester	0	0	0	0	0	0	0	0	0	0	0
SAL_FA4611AA	129521	Chester	0	0	0	0	0	0	0	0	0	0	0
SAL_FA4579AA	178660	Chester	0	0	0	0	0	0	0	0	0	0	0
SAL_FA4478AA	157330	Chester	0	0	0	0	0	0	0	0	0	0	0
SAL_FA4385AA	140190	Chester	0	0	0	0	0	0	0	0	0	0	0
SAL_FA4335AA	171151	Chester	0	0	0	0	0	0	0	0	0	0	0
SAL_FA4199AA	157275	Chester	0	0	0	0	0	0	0	0	0	0	0
SAL_FA4119AA	142188	Chester	0	0	0	0	0	0	0	0	0	0	0
SAL_FA1931AA	PNUSAS001660	Chester	0	0	0	0	0	0	0	0	0	0	0
SAL_FA1490AA	PNUSAS001212	Chester	0	0	0	0	0	0	0	0	0	0	0
SAL_FA1076AA	PNUSAS001535	Chester	0	0	0	0	0	0	0	0	0	0	0
SAL_FA0720AA	HIY0053	Chester	0	0	0	0	0	0	0	0	0	0	0
SAL_EA9165AA	53080	Chester	0	0	0	0	0	0	0	0	0	0	0
SAL_EA7932AA	37095	Chester	0	0	0	0	0	0	0	0	0	0	0
SAL_EA7738AA	39470	Chester	0	0	0	0	0	0	0	0	0	0	0
SAL_EA7704AA	2279	Chester	0	0	0	0	0	0	0	0	0	0	0
SAL_EA7584AA	29303	Chester	0	0	0	0	0	0	0	0	0	0	0
SAL_EA7307AA	58572	Chester	0	0	0	0	0	0	0	0	0	0	0
SAL_EA7196AA	58573	Chester	0	0	0	0	0	0	0	0	0	0	0
SAL_EA7095AA	58540	Chester	0	0	0	0	0	0	0	0	0	0	0
SAL_EA5275AA	FDA00001826	Chester	0	0	0	0	0	0	0	0	0	0	0
SAL_EA2700AA	FDA00000438	Chester	0	0	0	0	0	0	0	0	0	0	0
SAL_EA0986AA	'0-sc-2013-02-28T11:35:4	Chester	0	0	0	0	0	0	0	0	0	0	0
SAL_EA0306AA	FSW0066	Chester	0	0	0	0	0	0	0	0	0	0	0
SAL_EA0100AA	WAPHL_SAL-A00072	Chester	0	0	0	0	0	0	0	0	0	0	0
SAL_DA8245AA	/1261-sc-2010-09-29-985	Chester	0	0	0	0	0	0	0	0	0	0	0
SAL_DA6626AA	FDN0120	Chester	0	0	0	0	0	0	0	0	0	0	0
SAL_DA1630AA	FDA00000714	Chester	0	0	0	0	0	0	0	0	0	0	0
SAL_DA1594AA	NY-30356	Chester	0	0	0	0	0	0	0	0	0	0	0
SAL_DA1592AA	WAPHL_SAL-A00612	Chester	0	0	0	0	0	0	0	0	0	0	0
SAL_DA0760AA	H132720100	Chester	0	0	0	0	0	0	0	0	0	0	0
SAL_DA0723AA	H123960556	Chester	0	0	0	0	0	0	0	0	0	0	0
SAL_DA0704AA	H123300323	Chester	0	0	0	0	0	0	0	0	0	0	0
SAL_DA0644AA	H124940571	Chester	0	0	0	0	0	0	0	0	0	0	0
SAL_CA7794AA	FL-NRM251	Chester	0	0	0	0	0	0	0	0	0	0	0
SAL_CA7599AA	FDA00003069	Chester	0	0	0	0	0	0	0	0	0	0	0
SAL_CA6785AA	44694	Chester	0	0	0	0	0	0	0	0	0	0	0
SAL_CA6266AA	57659	Chester	0	0	0	0	0	0	0	0	0	0	0
SAL_CA6235AA	59991	Chester	0	0	0	0	0	0	0	0	0	0	0
SAL_CA6136AA	60056	Chester	0	0	0	0	0	0	0	0	0	0	0
SAL_CA5677AA	78634	Chester	0	0	0	0	0	0	0	0	0	0	0
SAL_CA5532AA	73645	Chester	0	0	0	0	0	0	0	0	0	0	0
SAL_CA5538AA	69737	Chester	0	0	0	0	0	0	0	0	0	0	0
SAL_CA5339AA	80335	Chester	0	0	0	0	0	0	0	0	0	0	0
SAL_CA5270AA	73648	Chester	0	0	0	0	0	0	0	0	0	0	0
SAL_CA5130AA	78669	Chester	0	0	0	0	0	0	0	0	0	0	0
SAL_CA5112AA	69188	Chester	0	0	0	0	0	0	0	0	0	0	0
SAL_CA3953AA	14127	Chester	0	0	0	0	0	0	0	0	0	0	0
SAL_CA3609AA	37774	Chester	0	0	0	0	0	0	0	0	0	0	0
SAL_CA2820AA	21773	Chester	0	0	0	0	0	0	0	0	0	0	0
SAL_CA2450AA	39457	Chester	0	0	0	0	0	0	0	0	0	0	0
SAL_CA2069AA	69946	Chester	0	0	0	0	0	0	0	0	0	0	0
SAL_CA0895AA	27046	Chester	0	0	0	0	0	0	0	0	0	0	0
SAL_CA0718AA	75921	Chester	0	0	0	0	0	0	0	0	0	0	0

SAL_CA0016AA	65401	Chester	0	0	0	0	0	0	0	0	0	0	0
SAL_BA9537AA	25336	Chester	0	0	0	0	0	0	0	0	0	0	0
SAL_BA8903AA	9158	Chester	0	0	0	0	0	0	0	0	0	0	0
SAL_BA8897AA	51276	Chester	0	0	0	0	0	0	0	0	0	0	0
SAL_BA8850AA	32542	Chester	0	0	0	0	0	0	0	0	0	0	0
SAL_BA8603AA	38755	Chester	0	0	0	0	0	0	0	0	0	0	0
SAL_BA7661AA	53008	Chester	0	0	0	0	0	0	0	0	0	0	0
SAL_BA7485AA	40284	Chester	0	0	0	0	0	0	0	0	0	0	0
SAL_BA7454AA	9152	Chester	0	0	0	0	0	0	0	0	0	0	0
SAL_BA7460AA	3771	Chester	0	0	0	0	0	0	0	0	0	0	0
SAL_BA7433AA	38752	Chester	0	0	0	0	0	0	0	0	0	0	0
SAL_BA6368AA	09:MF0001707R	Chester	0	0	0	0	0	0	0	0	0	0	0
SAL_BA2574AA	WAPHL_SAL-A00811	Chester	0	0	0	0	0	0	0	0	0	0	0
SAL_BA1724AA	FDA00001438	Chester	0	0	0	0	0	0	0	0	0	0	0
SAL_BA1673AA	FDA00009220	Chester	0	0	0	0	0	0	0	0	0	0	0
SAL_FA7156AA	PNUSAS002177	Chester	0	0	0	0	0	0	0	0	0	0	0
SAL_GA2601AA	07-0282	Chester	0	0	0	0	0	0	0	0	0	0	0
SAL_GA2596AA	2010K-1260	Chester	0	0	0	0	0	0	0	0	0	0	0
SAL_GA2588AA	98-0380	Chester	0	0	0	0	0	0	0	0	0	0	0
SAL_GA2583AA	05-0274	Chester	0	0	0	0	0	0	0	0	0	0	0
SAL_GA2581AA	84-1315	Chester	0	0	0	0	0	0	0	0	0	0	0
SAL_GA2578AA	58-1506	Chester	0	0	0	0	0	0	0	0	0	0	0
SAL_GA3508AA	BCW_2857	Chester	0	0	0	0	0	0	0	0	0	0	0
SAL_GA2140AA	BCW_6297	Chester	0	0	0	0	0	0	0	0	0	0	0
SAL_GA0708AA	pe4278_5-sc-2235520	Chester	0	0	0	0	0	0	0	0	0	0	0
SAL_GA0635AA	dj19646_8-sc-2235663	Chester	0	0	0	0	0	0	0	0	0	0	0
SAL_GA4748AA	PNUSAS002325	Chester	0	0	0	0	0	0	0	0	0	0	0
SAL_GA4738AA	HIY0276	Chester	0	0	0	0	0	0	0	0	0	0	0
SAL_GA4733AA	HIY0262	Chester	0	0	0	0	0	0	0	0	0	0	0
SAL_GA4732AA	HIY0269	Chester	0	0	0	0	0	0	0	0	0	0	0
SAL_GA4735AA	HIY0260	Chester	0	0	0	0	0	0	0	0	0	0	0
SAL_GA4731AA	HIY0274	Chester	0	0	0	0	0	0	0	0	0	0	0
SAL_GA4729AA	HIY0275	Chester	0	0	0	0	0	0	0	0	0	0	0
SAL_GA4730AA	HIY0035	Chester	0	0	0	0	0	0	0	0	0	0	0
SAL_FA9473AA	FDA00009983	Chester	0	0	0	0	0	0	0	0	0	0	0
SAL_FA9472AA	FDA00009984	Chester	0	0	0	0	0	0	0	0	0	0	0
SAL_FA9401AA	AZ-TG74356	Chester	0	0	0	0	0	0	0	0	0	0	0
SAL_GA5519AA	PNUSAS002863	Chester	0	0	0	0	0	0	0	0	0	0	0
SAL_GA7363AA	SS154	Chester	0	0	0	0	0	0	0	0	0	0	0
SAL_GA9149AA	14064489	Chester	0	0	0	0	0	0	0	0	0	0	0
SAL_GA9150AA	14064554	Chester	0	0	0	0	0	0	0	0	0	0	0
SAL_GA9148AA	C131197	Chester	0	0	0	0	0	0	0	0	0	0	0
SAL_GA9151AA	15050368	Chester	0	0	0	0	0	0	0	0	0	0	0
SAL_HA0411AA	HIY0256	Chester	0	0	0	0	0	0	0	0	0	0	0
SAL_HA2851AA	FSIS1608086	Chester	0	0	0	0	0	0	0	0	0	0	0
SAL_HA3037AA	FSIS1503981	Chester	0	0	0	0	0	0	0	0	0	0	0
SAL_HA2852AA	FSIS1608084	Chester	0	0	0	0	0	0	0	0	0	0	0
SAL_HA2857AA	FSIS1608087	Chester	0	0	0	0	0	0	0	0	0	0	0
SAL_HA2878AA	PNUSAS005133	Chester	0	0	0	0	0	0	0	0	0	0	0
SAL_HA2876AA	PNUSAS005132	Chester	0	0	0	0	0	0	0	0	0	0	0
SAL_HA2875AA	PNUSAS005134	Chester	0	0	0	0	0	0	0	0	0	0	0
SAL_HA2880AA	PNUSAS005129	Chester	0	0	0	0	0	0	0	0	0	0	0
SAL_HA4087AA	PNUSAS005215	Chester	0	0	0	0	0	0	0	0	0	0	0
SAL_HA5800AA	PNUSAS005414	Chester	0	0	0	0	0	0	0	0	0	0	0
SAL_HA4073AA	PNUSAS005217	Chester	0	0	0	0	0	0	0	0	0	0	0
SAL_HA6309AA	FSIS1608817	Chester	0	0	0	0	0	0	0	0	0	0	0
SAL_HA8267AA	PNUSAS006497	Chester	0	0	0	0	0	0	0	0	0	0	0
SAL_IA1766AA	140622	Chester	0	0	0	0	0	0	0	0	0	0	0
SAL_IA1327AA	285676	Chester	0	0	0	0	0	0	0	0	0	0	0
SAL_IA2797AA	222568	Chester	0	0	0	0	0	0	0	0	0	0	0
SAL_IA4986AA	S/20170020	Chester	0	0	0	0	0	0	0	0	0	0	0
SAL_IA5897AA	MS160416	Chester	0	0	0	0	0	0	0	0	0	0	0
SAL_IA6442AA	PNUSAS008546	Chester	0	0	0	0	0	0	0	0	0	0	0
SAL_IA7182AA	PNUSAS008374	Chester	0	0	0	0	0	0	0	0	0	0	0
SAL_IA8196AA	PNUSAS008788	Chester	0	0	0	0	0	0	0	0	0	0	0
SAL_IA8276AA	PNUSAS008375	Chester	0	0	0	0	0	0	0	0	0	0	0
SAL_IA8282AA	PNUSAS009066	Chester	0	0	0	0	0	0	0	0	0	0	0
SAL_IA8445AA	17011761	Chester	0	0	0	0	0	0	0	0	0	0	0
SAL_IA9073AA	S/20170140	Chester	0	0	0	0	0	0	0	0	0	0	0
SAL_IA9075AA	S/20170143	Chester	0	0	0	0	0	0	0	0	0	0	0
SAL_IA9074AA	S/20170135	Chester	0	0	0	0	0	0	0	0	0	0	0
SAL_IA9076AA	S/20170158	Chester	0	0	0	0	0	0	0	0	0	0	0
SAL_IA9328AA	PNUSAS009659	Chester	0	0	0	0	0	0	0	0	0	0	0

SAL_IA9121AA	PNUSAS009244	Chester	0	0	0	0	0	0	0	0	0	0	0
SAL_IA9904AA	PNUSAS010468	Chester	0	0	0	0	0	0	0	0	0	0	0
SAL_JA0517AA	PNUSAS009729	Chester	0	0	0	0	0	0	0	0	0	0	0
SAL_JA1508AA	PNUSAS010915	Chester	0	0	0	0	0	0	0	0	0	0	0
SAL_JA1696AA	S/20170140V	Chester	0	0	0	0	0	0	0	0	0	0	0
SAL_JA0514AA	PNUSAS009725	Chester	0	0	0	0	0	0	0	0	0	0	0
SAL_JA4404AA	201605878	Chester	0	0	0	0	0	0	0	0	0	0	0
SAL_JA6452AA	343432	Chester	0	0	0	0	0	0	0	0	0	0	0
SAL_JA6467AA	343438	Chester	0	0	0	0	0	0	0	0	0	0	0
SAL_JA6017AA	335219	Chester	0	0	0	0	0	0	0	0	0	0	0
SAL_JA5959AA	338424	Chester	0	0	0	0	0	0	0	0	0	0	0
SAL_JA5908AA	337095	Chester	0	0	0	0	0	0	0	0	0	0	0
SAL_JA5883AA	352049	Chester	0	0	0	0	0	0	0	0	0	0	0
SAL_JA5853AA	345743	Chester	0	0	0	0	0	0	0	0	0	0	0
SAL_JA5592AA	343405	Chester	0	0	0	0	0	0	0	0	0	0	0
SAL_JA5623AA	343419	Chester	0	0	0	0	0	0	0	0	0	0	0
SAL_JA6014AA	335549	Chester	0	0	0	0	0	0	0	0	0	0	0
SAL_JA5387AA	337014	Chester	0	0	0	0	0	0	0	0	0	0	0
SAL_JA5382AA	368258	Chester	0	0	0	0	0	0	0	0	0	0	0
SAL_JA6924AA	335027	Chester	0	0	0	0	0	0	0	0	0	0	0
SAL_JA6877AA	345745	Chester	0	0	0	0	0	0	0	0	0	0	0
SAL_JA6845AA	346573	Chester	0	0	0	0	0	0	0	0	0	0	0
SAL_JA6876AA	368335	Chester	0	0	0	0	0	0	0	0	0	0	0
SAL_JA5678AA	359271	Chester	0	0	0	0	0	0	0	0	0	0	0
SAL_JA7197AA	10-13-20170343	Chester	0	0	0	0	0	0	0	0	0	0	0
SAL_JA8442AA	361774	Chester	0	0	0	0	0	0	0	0	0	0	0
SAL_JA8383AA	336981	Chester	0	0	0	0	0	0	0	0	0	0	0
SAL_JA8381AA	364564	Chester	0	0	0	0	0	0	0	0	0	0	0
SAL_JA7444AA	PNUSAS013499	Chester	0	0	0	0	0	0	0	0	0	0	0
SAL_JA8351AA	343436	Chester	0	0	0	0	0	0	0	0	0	0	0
SAL_JA8289AA	343425	Chester	0	0	0	0	0	0	0	0	0	0	0
SAL_JA8266AA	344029	Chester	0	0	0	0	0	0	0	0	0	0	0
SAL_JA8207AA	356095	Chester	0	0	0	0	0	0	0	0	0	0	0
SAL_JA8211AA	333940	Chester	0	0	0	0	0	0	0	0	0	0	0
SAL_JA8176AA	339513	Chester	0	0	0	0	0	0	0	0	0	0	0
SAL_JA8117AA	339492	Chester	0	0	0	0	0	0	0	0	0	0	0
SAL_JA8034AA	348067	Chester	0	0	0	0	0	0	0	0	0	0	0
SAL_JA7924AA	351328	Chester	0	0	0	0	0	0	0	0	0	0	0
SAL_JA7863AA	364144	Chester	0	0	0	0	0	0	0	0	0	0	0
SAL_JA8406AA	341259	Chester	0	0	0	0	0	0	0	0	0	0	0
SAL_JA7771AA	346614	Chester	0	0	0	0	0	0	0	0	0	0	0
SAL_JA8690AA	PNUSAS011914	Chester	0	0	0	0	0	0	0	0	0	0	0
SAL_JA9161AA	PNUSAS015378	Chester	0	0	0	0	0	0	0	0	0	0	0
SAL_JA9029AA	MS170097	Chester	0	0	0	0	0	0	0	0	0	0	0
SAL_KA0417AA	PNUSAS014511	Chester	0	0	0	0	0	0	0	0	0	0	0
SAL_KA1340AA	PNUSAS014715	Chester	0	0	0	0	0	0	0	0	0	0	0
SAL_KA2059AA	12-7-20170425	Chester	0	0	0	0	0	0	0	0	0	0	0
SAL_KA1636AA	MS170128	Chester	0	0	0	0	0	0	0	0	0	0	0
SAL_KA1895AA	PNUSAS015719	Chester	0	0	0	0	0	0	0	0	0	0	0
SAL_KA2228AA	PNUSAS016866	Chester	0	0	0	0	0	0	0	0	0	0	0
SAL_KA2629AA	PNUSAS017589	Chester	0	0	0	0	0	0	0	0	0	0	0
SAL_KA4171AA	14 PAR-27	Chester	0	0	0	0	0	0	0	0	0	0	0
SAL_KA8003AA	PNUSAS016784	Chester	0	0	0	0	0	0	0	0	0	0	0
SAL_LA0089AA	18-27-20170680	Chester	0	0	0	0	0	0	0	0	0	0	0
SAL_LA0308AA	PNUSAS021104	Chester	0	0	0	0	0	0	0	0	0	0	0
SAL_LA0757AA	PNUSAS019966	Chester	0	0	0	0	0	0	0	0	0	0	0
SAL_LA3154AA	M105	Chester	0	0	0	0	0	0	0	0	0	0	0
SAL_LA6384AA	PNUSAS022418	Chester	0	0	0	0	0	0	0	0	0	0	0
SAL_LA4448AA	MS170176	Chester	0	0	0	0	0	0	0	0	0	0	0
SAL_MA1551AA	PNUSAS025752	Chester	0	0	0	0	0	0	0	0	0	0	0
SAL_MA2958AA	PNUSAS025005	Chester	0	0	0	0	0	0	0	0	0	0	0
SAL_MA3560AA	26-31-20171104	Chester	0	0	0	0	0	0	0	0	0	0	0
SAL_CA3868AA	41963	Chester	0	0	0	0	0	0	0	0	0	0	0
SAL_BA6437AA	1494329	Chester	0	0	0	0	0	0	0	0	0	0	0
SAL_CA3917AA	93558	Chester	0	0	0	0	0	0	0	0	0	0	0
SAL_DA8246AA	M62-sc-2010-09-29-9858	Chester	0	0	0	0	0	0	0	0	0	0	0
SAL_BA2471AA	FDA00009151	Chester	0	0	0	0	0	0	0	0	0	0	0
SAL_BA6364AA	08:MF0018567	Chester	0	0	0	0	0	0	0	0	0	0	0
SAL_DA9109AA	FNE0083	Chester	0	0	0	0	0	0	0	0	0	0	0
SAL_FA4261AA	185927	Chester	0	0	0	0	0	0	0	0	0	0	0
SAL_HA4036AA	PNUSAS005216	Chester	0	0	0	0	0	0	0	0	0	0	0
SAL_HA9368AA	PNUSAS006300	Chester	0	0	0	0	0	0	0	0	0	0	0
SAL_GA8576AA	PNUSAS003489	Chester	0	0	0	0	0	0	0	0	0	0	0

SAL_FA7737AA	FDA00001655	Chester	0	0	0	0	0	0	0	0	0	0	0
SAL_FA4091AA	182092	Chester	0	0	0	0	0	0	0	0	0	0	0
SAL_IA3807AA	PNUSAS007639	Chester	0	0	0	0	0	0	0	0	0	0	0
SAL_JA2490AA	PNUSAS010411	Chester	0	0	0	0	0	0	0	0	0	0	0
SAL_JA3437AA	FSIS1700412	Chester	0	0	0	0	0	0	0	0	0	0	0
SAL_JA5795AA	PNUSAS012342	Chester	0	0	0	0	0	0	0	0	0	0	0
SAL_JA6880AA	365385	Chester	0	0	0	0	0	0	0	0	0	0	0
SAL_JA8390AA	335082	Chester	0	0	0	0	0	0	0	0	0	0	0
SAL_JA9171AA	PNUSAS014785	Chester	0	0	0	0	0	0	0	0	0	0	0
SAL_KA3647AA	PNUSAS017565	Chester	0	0	0	0	0	0	0	0	0	0	0
SAL_LA0307AA	PNUSAS021106	Chester	0	0	0	0	0	0	0	0	0	0	0
SAL_LA1153AA	FDA00012132	Chester	0	0	0	0	0	0	0	0	0	0	0
SAL_LA4396AA	PNUSAS021467	Chester	0	0	0	0	0	0	0	0	0	0	0
SAL_BA8061AA	ATCC 11997	Chester	0	0	0	0	0	0	0	0	0	0	0
SAL_MA7062AA	PNUSAS027777	Chester	0	0	0	0	0	0	0	0	0	0	0
SAL_MA7958AA	30-16-8101248	Chester	0	0	0	0	0	0	0	0	0	0	0
SAL_MA7968AA	30-28-8101170	Chester	0	0	0	0	0	0	0	0	0	0	0
SAL_MA7971AA	30-20-8101253	Chester	0	0	0	0	0	0	0	0	0	0	0
SAL_MA8063AA	ARIMSH258-16	Chester	0	0	0	0	0	0	0	0	0	0	0
SAL_MA8004AA	ARIMSTM706-17	Chester	0	0	0	0	0	0	0	0	0	0	0
SAL_MA8011AA	PNUSAS028116	Chester	0	0	0	0	0	0	0	0	0	0	0
SAL_MA7995AA	ARIMSANA704-17	Chester	0	0	0	0	0	0	0	0	0	0	0
SAL_MA8000AA	ARIMSANA703-17	Chester	0	0	0	0	0	0	0	0	0	0	0
SAL_MA8792AA	FSIS11705074	Chester	0	0	0	0	0	0	0	0	0	0	0
SAL_NA0764AA	32-6-8101277	Chester	0	0	0	0	0	0	0	0	0	0	0
SAL_NA0769AA	32-24-8101295	Chester	0	0	0	0	0	0	0	0	0	0	0
SAL_JA1249AA	MS170054	Chester	0	0	0	0	0	0	0	0	0	0	0
SAL_NA3157AA	34-29-8101353	Chester	0	0	0	0	0	0	0	0	0	0	0
SAL_NA3164AA	34-18-8101343	Chester	0	0	0	0	0	0	0	0	0	0	0
SAL_NA4414AA	35-19-8101358	Chester	0	0	0	0	0	0	0	0	0	0	0
SAL_NA5467AA	PNUSAS031733	Chester	0	0	0	0	0	0	0	0	0	0	0
SAL_NA6005AA	2015AM-2434	Chester	0	0	0	0	0	0	0	0	0	0	0
SAL_NA7252AA	3-20-8100866	Chester	0	0	0	0	0	0	0	0	0	0	0
SAL_NA9855AA	PNUSAS033834	Chester	0	0	0	0	0	0	0	0	0	0	0
SAL_OA0436AA	FD01845442	Chester	0	0	0	0	0	0	0	0	0	0	0
SAL_OA9915AA	PNUSAS025418	Chester	0	0	0	0	0	0	0	0	0	0	0
SAL_OA4711AA	PNUSAS025295	Chester	0	0	0	0	0	0	0	0	0	0	0
SAL_PA1427AA	172493	Chester	0	0	0	0	0	0	0	0	0	0	0
SAL_PA1435AA	105317	Chester	0	0	0	0	0	0	0	0	0	0	0
SAL_PA1686AA	PNUSAS038653	Chester	0	0	0	0	0	0	0	0	0	0	0
SAL_PA4284AA	338423	Chester	0	0	0	0	0	0	0	0	0	0	0
SAL_PA4743AA	PNUSAS039996	Chester	0	0	0	0	0	0	0	0	0	0	0
SAL_PA6252AA	511841	Chester	0	0	0	0	0	0	0	0	0	0	0
SAL_PA6448AA	451997	Chester	0	0	0	0	0	0	0	0	0	0	0
SAL_PA6425AA	169640	Chester	0	0	0	0	0	0	0	0	0	0	0
SAL_PA6427AA	305278	Chester	0	0	0	0	0	0	0	0	0	0	0
SAL_PA7302AA	205633	Chester	0	0	0	0	0	0	0	0	0	0	0
SAL_PA7579AA	PNUSAS040139	Chester	0	0	0	0	0	0	0	0	0	0	0
SAL_PA7585AA	PNUSAS040136	Chester	0	0	0	0	0	0	0	0	0	0	0
SAL_PA7959AA	323890	Chester	0	0	0	0	0	0	0	0	0	0	0
SAL_PA8386AA	307457	Chester	0	0	0	0	0	0	0	0	0	0	0
SAL_PA8227AA	PNUSAS041128	Chester	0	0	0	0	0	0	0	0	0	0	0
SAL_PA8483AA	290610	Chester	0	0	0	0	0	0	0	0	0	0	0
SAL_PA9247AA	PNUSAS041252	Chester	0	0	0	0	0	0	0	0	0	0	0
SAL_PA9167AA	PNUSAS041447	Chester	0	0	0	0	0	0	0	0	0	0	0
SAL_PA9131AA	PNUSAS041364	Chester	0	0	0	0	0	0	0	0	0	0	0
SAL_PA8995AA	271128	Chester	0	0	0	0	0	0	0	0	0	0	0
SAL_PA8999AA	323083	Chester	0	0	0	0	0	0	0	0	0	0	0
SAL_PA8882AA	172424	Chester	0	0	0	0	0	0	0	0	0	0	0
SAL_PA9488AA	314957	Chester	0	0	0	0	0	0	0	0	0	0	0
SAL_PA9586AA	PNUSAS041715	Chester	0	0	0	0	0	0	0	0	0	0	0
SAL_PA9985AA	216227	Chester	0	0	0	0	0	0	0	0	0	0	0
SAL_QA0351AA	370624	Chester	0	0	0	0	0	0	0	0	0	0	0
SAL_QA0355AA	397420	Chester	0	0	0	0	0	0	0	0	0	0	0
SAL_QA0328AA	261386	Chester	0	0	0	0	0	0	0	0	0	0	0
SAL_QA0485AA	256135	Chester	0	0	0	0	0	0	0	0	0	0	0
SAL_QA0382AA	526179	Chester	0	0	0	0	0	0	0	0	0	0	0
SAL_QA0672AA	531221	Chester	0	0	0	0	0	0	0	0	0	0	0
SAL_QA1362AA	185889	Chester	0	0	0	0	0	0	0	0	0	0	0
SAL_QA1351AA	238372	Chester	0	0	0	0	0	0	0	0	0	0	0
SAL_QA1335AA	414572	Chester	0	0	0	0	0	0	0	0	0	0	0
SAL_QA1319AA	377343	Chester	0	0	0	0	0	0	0	0	0	0	0
SAL_QA2516AA	342956	Chester	0	0	0	0	0	0	0	0	0	0	0

SAL_QA2467AA	475800	Chester	0	0	0	0	0	0	0	0	0	0	0
SAL_QA2391AA	267720	Chester	0	0	0	0	0	0	0	0	0	0	0
SAL_QA2200AA	408835	Chester	0	0	0	0	0	0	0	0	0	0	0
SAL_QA2123AA	155621	Chester	0	0	0	0	0	0	0	0	0	0	0
SAL_QA1982AA	392853	Chester	0	0	0	0	0	0	0	0	0	0	0
SAL_QA2924AA	370915	Chester	0	0	0	0	0	0	0	0	0	0	0
SAL_QA2795AA	458082	Chester	0	0	0	0	0	0	0	0	0	0	0
SAL_QA2710AA	174650	Chester	0	0	0	0	0	0	0	0	0	0	0
SAL_QA1421AA	279388	Chester	0	0	0	0	0	0	0	0	0	0	0
SAL_QA3444AA	440162	Chester	0	0	0	0	0	0	0	0	0	0	0
SAL_QA3283AA	421681	Chester	0	0	0	0	0	0	0	0	0	0	0
SAL_QA3194AA	231988	Chester	0	0	0	0	0	0	0	0	0	0	0
SAL_QA3930AA	431296	Chester	0	0	0	0	0	0	0	0	0	0	0
SAL_QA3893AA	503413	Chester	0	0	0	0	0	0	0	0	0	0	0
SAL_QA3818AA	365370	Chester	0	0	0	0	0	0	0	0	0	0	0
SAL_QA2419AA	429159	Chester	0	0	0	0	0	0	0	0	0	0	0
SAL_QA4077AA	273535	Chester	0	0	0	0	0	0	0	0	0	0	0
SAL_QA4362AA	PNUSAS040402	Chester	0	0	0	0	0	0	0	0	0	0	0
SAL_QA4827AA	476614	Chester	0	0	0	0	0	0	0	0	0	0	0
SAL_QA4825AA	126187	Chester	0	0	0	0	0	0	0	0	0	0	0
SAL_QA4790AA	265295	Chester	0	0	0	0	0	0	0	0	0	0	0
SAL_QA4765AA	404702	Chester	0	0	0	0	0	0	0	0	0	0	0
SAL_QA4774AA	264157	Chester	0	0	0	0	0	0	0	0	0	0	0
SAL_QA5214AA	180555	Chester	0	0	0	0	0	0	0	0	0	0	0
SAL_QA5220AA	335020	Chester	0	0	0	0	0	0	0	0	0	0	0
SAL_QA5108AA	278611	Chester	0	0	0	0	0	0	0	0	0	0	0
SAL_QA5951AA	127409	Chester	0	0	0	0	0	0	0	0	0	0	0
SAL_QA4055AA	550830	Chester	0	0	0	0	0	0	0	0	0	0	0
SAL_QA5843AA	294693	Chester	0	0	0	0	0	0	0	0	0	0	0
SAL_QA6278AA	408443	Chester	0	0	0	0	0	0	0	0	0	0	0
SAL_QA6247AA	533375	Chester	0	0	0	0	0	0	0	0	0	0	0
SAL_QA6590AA	146100	Chester	0	0	0	0	0	0	0	0	0	0	0
SAL_QA4754AA	335538	Chester	0	0	0	0	0	0	0	0	0	0	0
SAL_QA6439AA	443661	Chester	0	0	0	0	0	0	0	0	0	0	0
SAL_QA5473AA	397389	Chester	0	0	0	0	0	0	0	0	0	0	0
SAL_QA6732AA	310209	Chester	0	0	0	0	0	0	0	0	0	0	0
SAL_QA7387AA	288773	Chester	0	0	0	0	0	0	0	0	0	0	0
SAL_QA7335AA	173532	Chester	0	0	0	0	0	0	0	0	0	0	0
SAL_QA7245AA	302632	Chester	0	0	0	0	0	0	0	0	0	0	0
SAL_QA6171AA	414587	Chester	0	0	0	0	0	0	0	0	0	0	0
SAL_QA7735AA	221710	Chester	0	0	0	0	0	0	0	0	0	0	0
SAL_RA3806AA	SMG-18-905	Chester	0	0	0	0	0	0	0	0	0	0	0
SAL_QA7662AA	317679	Chester	0	0	0	0	0	0	0	0	0	0	0
SAL_QA7560AA	302937	Chester	0	0	0	0	0	0	0	0	0	0	0
SAL_QA8402AA	355058	Chester	0	0	0	0	0	0	0	0	0	0	0
SAL_QA8362AA	486706	Chester	0	0	0	0	0	0	0	0	0	0	0
SAL_QA8314AA	294943	Chester	0	0	0	0	0	0	0	0	0	0	0
SAL_QA8871AA	253906	Chester	0	0	0	0	0	0	0	0	0	0	0
SAL_QA8775AA	296625	Chester	0	0	0	0	0	0	0	0	0	0	0
SAL_QA8744AA	456154	Chester	0	0	0	0	0	0	0	0	0	0	0
SAL_QA8759AA	408635	Chester	0	0	0	0	0	0	0	0	0	0	0
SAL_QA8656AA	278086	Chester	0	0	0	0	0	0	0	0	0	0	0
SAL_QA9503AA	382849	Chester	0	0	0	0	0	0	0	0	0	0	0
SAL_QA9425AA	159878	Chester	0	0	0	0	0	0	0	0	0	0	0
SAL_QA9328AA	559024	Chester	0	0	0	0	0	0	0	0	0	0	0
SAL_QA9686AA	PNUSAS044281	Chester	0	0	0	0	0	0	0	0	0	0	0
SAL_RA0006AA	248557	Chester	0	0	0	0	0	0	0	0	0	0	0
SAL_QA9937AA	288898	Chester	0	0	0	0	0	0	0	0	0	0	0
SAL_QA9879AA	291840	Chester	0	0	0	0	0	0	0	0	0	0	0
SAL_QA9843AA	345749	Chester	0	0	0	0	0	0	0	0	0	0	0
SAL_RA0450AA	442169	Chester	0	0	0	0	0	0	0	0	0	0	0
SAL_RA0345AA	188180	Chester	0	0	0	0	0	0	0	0	0	0	0
SAL_RA0850AA	308739	Chester	0	0	0	0	0	0	0	0	0	0	0
SAL_RA0753AA	211175	Chester	0	0	0	0	0	0	0	0	0	0	0
SAL_RA0644AA	461141	Chester	0	0	0	0	0	0	0	0	0	0	0
SAL_RA0623AA	186532	Chester	0	0	0	0	0	0	0	0	0	0	0
SAL_RA1360AA	159902	Chester	0	0	0	0	0	0	0	0	0	0	0
SAL_RA2886AA	410822	Chester	0	0	0	0	0	0	0	0	0	0	0
SAL_RA2829AA	456198	Chester	0	0	0	0	0	0	0	0	0	0	0
SAL_RA3422AA	238699	Chester	0	0	0	0	0	0	0	0	0	0	0
SAL_RA3310AA	511818	Chester	0	0	0	0	0	0	0	0	0	0	0
SAL_RA3265AA	418246	Chester	0	0	0	0	0	0	0	0	0	0	0
SAL_RA3699AA	280332	Chester	0	0	0	0	0	0	0	0	0	0	0

SAL_RA4082AA	296838	Chester	0	0	0	0	0	0	0	0	0	0	0
SAL_RA4084AA	563015	Chester	0	0	0	0	0	0	0	0	0	0	0
SAL_RA4041AA	440894	Chester	0	0	0	0	0	0	0	0	0	0	0
SAL_RA3988AA	356527	Chester	0	0	0	0	0	0	0	0	0	0	0
SAL_QA9932AA	342537	Chester	0	0	0	0	0	0	0	0	0	0	0
SAL_RA5882AA	PNUSAS045219	Chester	0	0	0	0	0	0	0	0	0	0	0
SAL_RA5416AA	565956	Chester	0	0	0	0	0	0	0	0	0	0	0
SAL_RA2698AA	212772	Chester	0	0	0	0	0	0	0	0	0	0	0
SAL_RA2582AA	272492	Chester	0	0	0	0	0	0	0	0	0	0	0
SAL_SA1415AA	AU169 KAK-SS	Chester	0	0	0	0	0	0	0	0	0	0	0
SAL_RA6588AA	301611	Chester	0	0	0	0	0	0	0	0	0	0	0
SAL_RA6584AA	470355	Chester	0	0	0	0	0	0	0	0	0	0	0
SAL_RA7412AA	568441	Chester	0	0	0	0	0	0	0	0	0	0	0
SAL_RA7429AA	185861	Chester	0	0	0	0	0	0	0	0	0	0	0
SAL_RA7295AA	417412	Chester	0	0	0	0	0	0	0	0	0	0	0
SAL_RA7290AA	568461	Chester	0	0	0	0	0	0	0	0	0	0	0
SAL_RA7277AA	568426	Chester	0	0	0	0	0	0	0	0	0	0	0
SAL_RA7268AA	534023	Chester	0	0	0	0	0	0	0	0	0	0	0
SAL_RA7160AA	451969	Chester	0	0	0	0	0	0	0	0	0	0	0
SAL_RA7837AA	313151	Chester	0	0	0	0	0	0	0	0	0	0	0
SAL_RA7813AA	408197	Chester	0	0	0	0	0	0	0	0	0	0	0
SAL_RA7707AA	296695	Chester	0	0	0	0	0	0	0	0	0	0	0
SAL_RA8066AA	570854	Chester	0	0	0	0	0	0	0	0	0	0	0
SAL_RA7978AA	570003	Chester	0	0	0	0	0	0	0	0	0	0	0
SAL_RA7971AA	293053	Chester	0	0	0	0	0	0	0	0	0	0	0
SAL_RA7986AA	313196	Chester	0	0	0	0	0	0	0	0	0	0	0
SAL_RA3898AA	311980	Chester	0	0	0	0	0	0	0	0	0	0	0
SAL_SA3001AA	CFSAN070323	Chester	0	0	0	0	0	0	0	0	0	0	0
SAL_SA3022AA	CFSAN070332	Chester	0	0	0	0	0	0	0	0	0	0	0
SAL_SA5466AA	MC_04-0441	Chester	0	0	0	0	0	0	0	0	0	0	0
SAL_SA5458AA	MC_04-0105	Chester	0	0	0	0	0	0	0	0	0	0	0
SAL_SA5269AA	CK_94/00000542	Chester	0	0	0	0	0	0	0	0	0	244	0
SAL_SA4914AA	591902	Chester	0	0	0	0	0	0	0	0	0	0	0
SAL_SA4854AA	576386	Chester	0	0	0	0	0	0	0	0	0	0	0
SAL_SA4891AA	598897	Chester	0	0	0	0	0	0	0	0	0	0	0
SAL_SA6003AA	445931	Chester	0	0	0	0	0	0	0	0	0	0	0
SAL_SA5936AA	576395	Chester	0	0	0	0	0	0	0	0	0	0	0
SAL_SA5786AA	572535	Chester	0	0	0	0	0	0	0	0	0	0	0
SAL_SA6414AA	573643	Chester	0	0	0	0	0	0	0	0	0	0	0
SAL_SA6364AA	408437	Chester	0	0	0	0	0	0	0	0	0	0	0
SAL_SA6339AA	595645	Chester	0	0	0	0	0	0	0	0	0	0	0
SAL_SA6656AA	601703	Chester	0	0	0	0	0	0	0	0	0	0	0
SAL_SA6615AA	598065	Chester	0	0	0	0	0	0	0	0	0	0	0
SAL_SA6553AA	591826	Chester	0	0	0	0	0	0	0	0	0	0	0
SAL_SA6569AA	598936	Chester	0	0	0	0	0	0	0	0	0	0	0
SAL_SA7421AA	298892	Chester	0	0	0	0	0	0	0	0	0	0	0
SAL_SA7095AA	594689	Chester	0	0	0	0	0	0	0	0	0	0	0
SAL_SA7436AA	572329	Chester	0	0	0	0	0	0	0	0	0	0	0
SAL_SA7417AA	604846	Chester	0	0	0	0	0	0	0	0	0	0	0
SAL_SA7699AA	315195	Chester	0	0	0	0	0	0	0	0	0	0	0
SAL_SA7709AA	211648	Chester	0	0	0	0	0	0	0	0	0	0	0
SAL_SA7668AA	348748	Chester	0	0	0	0	0	0	0	0	0	0	0
SAL_SA7995AA	416288	Chester	0	0	0	0	0	0	0	0	0	0	0
SAL_SA8751AA	346912	Chester	0	0	0	0	0	0	0	0	0	0	0
SAL_SA8596AA	320326	Chester	0	0	0	0	0	0	0	0	0	0	0
SAL_TA0380AA	612646	Chester	0	0	0	0	0	0	0	0	0	0	0
SAL_TA0343AA	188370	Chester	0	0	0	0	0	0	0	0	0	0	0
SAL_TA0347AA	613083	Chester	0	0	0	0	0	0	0	0	0	0	0
SAL_TA0372AA	612670	Chester	0	0	0	0	0	0	0	0	0	0	0
SAL_TA0615AA	1806W26156	Chester	0	0	0	0	0	0	0	0	0	0	0
SAL_TA0841AA	616232	Chester	0	0	0	0	0	0	0	0	0	0	0
SAL_TA0886AA	611407	Chester	0	0	0	0	0	0	0	0	0	0	0
SAL_TA0749AA	616292	Chester	0	0	0	0	0	0	0	0	0	0	0
SAL_TA0977AA	52-25-18-8101057-H	Chester	0	0	0	0	0	0	0	0	0	0	0
SAL_TA1543AA	53-16-18-8101079-F	Chester	0	0	0	0	0	0	0	0	0	0	0
SAL_TA2935AA	616269	Chester	0	0	0	0	0	0	0	0	0	0	0
SAL_TA2917AA	625690	Chester	0	0	0	0	0	0	0	0	0	0	0
SAL_TA5222AA	615622	Chester	0	0	0	0	0	0	0	0	0	0	0
SAL_TA5942AA	607871	Chester	0	0	0	0	0	0	0	0	0	0	0
SAL_TA6673AA	628807	Chester	0	0	0	0	0	0	0	0	0	0	0
SAL_TA6675AA	624461	Chester	0	0	0	0	0	0	0	0	0	0	0
SAL_TA6755AA	628850	Chester	0	0	0	0	0	0	0	0	0	0	0
SAL_TA6901AA	633423	Chester	0	0	0	0	0	0	0	0	0	0	0

SAL_QA5416AA	195561	Chester	0	0	0	0	0	0	0	0	0	0	0
SAL_TA8299AA	636356	Chester	0	0	0	0	0	0	0	0	0	0	0
SAL_OA1385AA	PNUSAS034769	Chester	0	0	0	0	0	0	0	0	0	0	0
SAL_QA7745AA	367491	Chester	0	0	0	0	0	0	0	0	0	0	0
SAL_QA7792AA	182810	Chester	0	0	0	0	0	0	0	0	0	0	0
SAL_TA8928AA	639827	Chester	0	0	0	0	0	0	0	0	0	0	0
SAL_TA8933AA	639825	Chester	0	0	0	0	0	0	0	0	0	0	0
SAL_PA0379AA	PNUSAS035446	Chester	0	0	0	0	0	0	0	0	0	0	0
SAL_PA1429AA	126208	Chester	0	0	0	0	0	0	0	0	0	0	0
SAL_PA1428AA	170469	Chester	0	0	0	0	0	0	0	0	0	0	0
SAL_PA1434AA	174724	Chester	0	0	0	0	0	0	0	0	0	0	0
SAL_PA1433AA	137124	Chester	0	0	0	0	0	0	0	0	0	0	0
SAL_PA1432AA	143554	Chester	0	0	0	0	0	0	0	0	0	0	0
SAL_PA1431AA	161831	Chester	0	0	0	0	0	0	0	0	0	0	0
SAL_PA1430AA	131496	Chester	0	0	0	0	0	0	0	0	0	0	0
SAL_PA2293AA	PNUSAS038377	Chester	0	0	0	0	0	0	0	0	0	0	0
SAL_PA3920AA	PNUSAS038937	Chester	0	0	0	0	0	0	0	0	0	0	0
SAL_TA9544AA	640312	Chester	0	0	0	0	0	0	0	0	0	0	0
SAL_TA9555AA	640313	Chester	0	0	0	0	0	0	0	0	0	0	0
SAL_TA9749AA	643198	Chester	0	0	0	0	0	0	0	0	0	0	0
SAL_UA2743AA	646014	Chester	0	0	0	0	0	0	0	0	0	0	0
SAL_UA6603AA	15EP000527	Chester	0	0	0	0	0	0	0	0	0	0	0
SAL_UA7212AA	661025	Chester	0	0	0	0	0	0	0	0	0	0	0
SAL_UA2939AA	627399	Chester	0	0	0	0	0	0	0	0	0	0	0
SAL_UA3701AA	599582	Chester	0	0	0	0	0	0	0	0	0	0	0
SAL_UA5973AA	654697	Chester	0	0	0	0	0	0	0	0	0	0	0
SAL_UA5960AA	654698	Chester	0	0	0	0	0	0	0	0	0	0	0
SAL_UA6215AA	646007	Chester	0	0	0	0	0	0	0	0	0	0	0
SAL_UA6599AA	15EP001414	Chester	0	0	0	0	0	0	0	0	0	0	0
SAL_UA6618AA	1112-0999	Chester	0	0	0	0	0	0	0	0	0	0	0
SAL_UA6593AA	16EP001141	Chester	0	0	0	0	0	0	0	0	0	0	0
SAL_UA6619AA	1112-0672	Chester	0	0	0	0	0	0	0	0	0	0	0
SAL_UA6591AA	16EP001252	Chester	0	0	0	0	0	0	0	0	0	0	0
SAL_UA6594AA	16EP001015	Chester	0	0	0	0	0	0	0	0	0	0	0
SAL_UA6592AA	16EP001189	Chester	0	0	0	0	0	0	0	0	0	0	0
SAL_UA6617AA	12EP000069	Chester	0	0	0	0	0	0	0	0	0	0	0
SAL_UA6590AA	16EP001293	Chester	0	0	0	0	0	0	0	0	0	0	0
SAL_UA6589AA	16EP001347	Chester	0	0	0	0	0	0	0	0	0	0	0
SAL_UA6616AA	12EP000178	Chester	0	0	0	0	0	0	0	0	0	0	0
SAL_UA6615AA	12EP000528	Chester	0	0	0	0	0	0	0	0	0	0	0
SAL_UA6588AA	16EP001403	Chester	0	0	0	0	0	0	0	0	0	0	0
SAL_UA6614AA	12EP001601	Chester	0	0	0	0	0	0	0	0	0	0	0
SAL_UA6613AA	13EP002315	Chester	0	0	0	0	0	0	0	0	0	0	0
SAL_UA6587AA	16EP001575	Chester	0	0	0	0	0	0	0	0	0	0	0
SAL_UA6586AA	16EP001596	Chester	0	0	0	0	0	0	0	0	0	0	0
SAL_UA6612AA	13EP002352	Chester	0	0	0	0	0	0	0	0	0	0	0
SAL_UA6585AA	16EP001724	Chester	0	0	0	0	0	0	0	0	0	0	0
SAL_UA6583AA	16EP002084	Chester	0	0	0	0	0	0	0	0	0	0	0
SAL_UA6630AA	1000-9039	Chester	0	0	0	0	0	0	0	0	0	0	0
SAL_UA6610AA	13EP002625	Chester	0	0	0	0	0	0	0	0	0	0	0
SAL_UA6611AA	13EP002492	Chester	0	0	0	0	0	0	0	0	0	0	0
SAL_UA6580AA	16EP002170	Chester	0	0	0	0	0	0	0	0	0	0	0
SAL_UA6609AA	14EP001690	Chester	0	0	0	0	0	0	0	0	0	0	0
SAL_UA6582AA	16EP002135	Chester	0	0	0	0	0	0	0	0	0	0	0
SAL_UA6581AA	16EP002146	Chester	0	0	0	0	0	0	0	0	0	0	0
SAL_UA6608AA	14EP001873	Chester	0	0	0	0	0	0	0	0	0	0	0
SAL_UA6629AA	1030-4055	Chester	0	0	0	0	0	0	0	0	0	0	0
SAL_UA6627AA	1108-1979	Chester	0	0	0	0	0	0	0	0	0	0	0
SAL_UA6626AA	1108-3031	Chester	0	0	0	0	0	0	0	0	0	0	0
SAL_UA6628AA	1106-3639	Chester	0	0	0	0	0	0	0	0	0	0	0
SAL_UA6604AA	15EP000454	Chester	0	0	0	0	0	0	0	0	0	0	0
SAL_UA6606AA	14EP002099	Chester	0	0	0	0	0	0	0	0	0	0	0
SAL_UA6605AA	14EP002610	Chester	0	0	0	0	0	0	0	0	0	0	0
SAL_UA6601AA	15EP001087	Chester	0	0	0	0	0	0	0	0	0	0	0
SAL_UA6607AA	14EP002011	Chester	0	0	0	0	0	0	0	0	0	0	0
SAL_UA6602AA	15EP001067	Chester	0	0	0	0	0	0	0	0	0	0	0
SAL_UA6600AA	15EP001273	Chester	0	0	0	0	0	0	0	0	0	0	0
SAL_UA6625AA	1109-0327	Chester	0	0	0	0	0	0	0	0	0	0	0
SAL_UA6624AA	1110-1067	Chester	0	0	0	0	0	0	0	0	0	0	0
SAL_UA6623AA	1110-1610	Chester	0	0	0	0	0	0	0	0	0	0	0
SAL_UA6596AA	16EP000506	Chester	0	0	0	0	0	0	0	0	0	0	0
SAL_UA6595AA	16EP000606	Chester	0	0	0	0	0	0	0	0	0	0	0
SAL_UA6622AA	1110-2630	Chester	0	0	0	0	0	0	0	0	0	0	0

SAL_UA6598AA	15EP001474	Chester	0	0	0	0	0	0	0	0	0	0	0
SAL_UA6621AA	1110-2702	Chester	0	0	0	0	0	0	0	0	0	0	0
SAL_UA6597AA	16EP000476	Chester	0	0	0	0	0	0	0	0	0	0	0
SAL_UA6620AA	1111-1890	Chester	0	0	0	0	0	0	0	0	0	0	0
SAL_UA7011AA	655383	Chester	0	0	0	0	0	0	0	0	0	0	0
SAL_UA9630AA	671920	Chester	0	0	0	0	0	0	0	0	0	0	0
SAL_UA9571AA	642287	Chester	0	0	0	0	0	0	0	0	0	0	0
SAL_UA9702AA	672548	Chester	0	0	0	0	0	0	0	0	0	0	0
SAL_VA0192AA	674052	Chester	0	0	0	0	0	0	0	0	0	0	0
SAL_VA0212AA	568298	Chester	0	0	0	0	0	0	0	0	0	0	0
SAL_UA9924AA	557302	Chester	0	0	0	0	0	0	0	0	0	0	0
SAL_UA9903AA	555486	Chester	0	0	0	0	0	0	0	0	0	0	0
SAL_UA9891AA	561114	Chester	0	0	0	0	0	0	0	0	0	0	0
SAL_VA0436AA	539049	Chester	0	0	0	0	0	0	0	0	0	0	0
SAL_VA1328AA	478533	Chester	0	0	0	0	0	0	0	0	0	0	0
SAL_VA7406AA	201701196	Chester	0	0	0	0	0	0	0	0	0	0	0
SAL_VA2137AA	459887	Chester	0	0	0	0	0	0	0	0	0	0	0
SAL_VA2112AA	462646	Chester	0	0	0	0	0	0	0	0	0	0	0
SAL_VA2075AA	454869	Chester	0	0	0	0	0	0	0	0	0	0	0
SAL_VA2029AA	678009	Chester	0	0	0	0	0	0	0	0	0	0	0
SAL_VA1950AA	448182	Chester	0	0	0	0	0	0	0	0	0	0	0
SAL_VA3241AA	416293	Chester	0	0	0	0	0	0	0	0	0	0	0
SAL_VA3188AA	413188	Chester	0	0	0	0	0	0	0	0	0	0	0
SAL_VA3089AA	680520	Chester	0	0	0	0	0	0	0	0	0	0	0
SAL_VA3442AA	412015	Chester	0	0	0	0	0	0	0	0	0	0	0
SAL_VA2328AA	478628	Chester	0	0	0	0	0	0	0	0	0	0	0
SAL_VA2322AA	474908	Chester	0	0	0	0	0	0	0	0	0	0	0
SAL_VA2285AA	481507	Chester	0	0	0	0	0	0	0	0	0	0	0
SAL_VA3393AA	417393	Chester	0	0	0	0	0	0	0	0	0	0	0
SAL_VA1886AA	442660	Chester	0	0	0	0	0	0	0	0	0	0	0
SAL_VA2445AA	431380	Chester	0	0	0	0	0	0	0	0	0	0	0
SAL_VA2466AA	435445	Chester	0	0	0	0	0	0	0	0	0	0	0
SAL_VA2759AA	419125	Chester	0	0	0	0	0	0	0	0	0	0	0
SAL_VA2770AA	416201	Chester	0	0	0	0	0	0	0	0	0	0	0
SAL_VA2739AA	680508	Chester	0	0	0	0	0	0	0	0	0	0	0
SAL_VA5899AA	SA20032106	Chester	0	0	0	0	0	0	0	0	0	0	0
SAL_VA5913AA	SA20032178	Chester	0	0	0	0	0	0	0	0	0	0	0
SAL_VA5700AA	SA20032088	Chester	0	0	0	0	0	0	0	0	0	0	0
SAL_VA5342AA	343422	Chester	0	0	0	0	0	0	0	0	0	0	0
SAL_VA4488AA	401589	Chester	0	0	0	0	0	0	0	0	0	0	0
SAL_VA6862AA	376095	Chester	0	0	0	0	0	0	0	0	0	0	0
SAL_VA8290AA	371823	Chester	0	0	0	0	0	0	0	0	0	0	0
SAL_VA8175AA	362083	Chester	0	0	0	0	0	0	0	0	0	0	0
SAL_VA8584AA	366346	Chester	0	0	0	0	0	0	0	0	0	0	0
SAL_VA8510AA	345583	Chester	0	0	0	0	0	0	0	0	0	0	0
SAL_VA8482AA	328561	Chester	0	0	0	0	0	0	0	0	0	0	0
SAL_VA8621AA	343389	Chester	0	0	0	0	0	0	0	0	0	0	0
SAL_VA8773AA	314906	Chester	0	0	0	0	0	0	0	0	0	0	0
SAL_VA8723AA	691143	Chester	0	0	0	0	0	0	0	0	0	0	0
SAL_VA8738AA	316692	Chester	0	0	0	0	0	0	0	0	0	0	0
SAL_VA8656AA	315010	Chester	0	0	0	0	0	0	0	0	0	0	0
SAL_VA8639AA	323845	Chester	0	0	0	0	0	0	0	0	0	0	0
SAL_VA9207AA	311153	Chester	0	0	0	0	0	0	0	0	0	0	0
SAL_VA9153AA	323841	Chester	0	0	0	0	0	0	0	0	0	0	0
SAL_VA9112AA	302896	Chester	0	0	0	0	0	0	0	0	0	0	0
SAL_VA9074AA	345746	Chester	0	0	0	0	0	0	0	0	0	0	0
SAL_VA9030AA	311972	Chester	0	0	0	0	0	0	0	0	0	0	0
SAL_VA9084AA	302912	Chester	0	0	0	0	0	0	0	0	0	0	0
SAL_VA8926AA	298820	Chester	0	0	0	0	0	0	0	0	0	0	0
SAL_VA9622AA	282605	Chester	0	0	0	0	0	0	0	0	0	0	0
SAL_VA9613AA	288811	Chester	0	0	0	0	0	0	0	0	0	0	0
SAL_VA9942AA	242368	Chester	0	0	0	0	0	0	0	0	0	0	0
SAL_VA9950AA	305252	Chester	0	0	0	0	0	0	0	0	0	0	0
SAL_VA9967AA	243882	Chester	0	0	0	0	0	0	0	0	0	0	0
SAL_VA9891AA	224104	Chester	0	0	0	0	0	0	0	0	0	0	0
SAL_VA9956AA	245501	Chester	0	0	0	0	0	0	0	0	0	0	0
SAL_WA0190AA	218441	Chester	0	0	0	0	0	0	0	0	0	0	0
SAL_WA0235AA	207565	Chester	0	0	0	0	0	0	0	0	0	0	0
SAL_WA0205AA	199940	Chester	0	0	0	0	0	0	0	0	0	0	0
SAL_WA0608AA	190797	Chester	0	0	0	0	0	0	0	0	0	0	0
SAL_WA0520AA	194211	Chester	0	0	0	0	0	0	0	0	0	0	0
SAL_WA0423AA	182185	Chester	0	0	0	0	0	0	0	0	0	0	0
SAL_WA0350AA	170457	Chester	0	0	0	0	0	0	0	0	0	0	0

SAL_WA0341AA	171298	Chester	0	0	0	0	0	0	0	0	0	0	0
SAL_WA0973AA	152416	Chester	0	0	0	0	0	0	0	0	0	0	0
SAL_WA0877AA	697911	Chester	0	0	0	0	0	0	0	0	0	0	0
SAL_VA4092AA	CDPH_CSalm304	Chester	0	0	0	0	0	0	0	0	0	0	0
SAL_WA1316AA	131500	Chester	0	0	0	0	0	0	0	0	0	0	0
SAL_WA3271AA	MC_07-0740	Chester	0	0	0	0	0	0	0	0	0	0	0
SAL_WA2887AA	H103540752	Chester	0	0	0	0	0	0	0	0	0	0	0
SAL_WA2681AA	MC_07-0594	Chester	0	0	0	0	0	0	0	0	0	0	0
SAL_WA2510AA	MC_07-0859	Chester	0	0	0	0	0	0	0	0	0	0	0
SAL_WA2606AA	MC_08-0719	Chester	0	0	0	0	0	0	0	0	0	0	0
SAL_WA2584AA	MC_06-0610	Chester	0	0	0	0	0	0	0	0	0	0	0
SAL_WA2387AA	H103540745	Chester	0	0	0	0	0	0	0	0	0	0	0
SAL_WA1192AA	136756	Chester	0	0	0	0	0	0	0	0	0	0	0
SAL_WA1542AA	109855	Chester	0	0	0	0	0	0	0	0	0	0	0
SAL_WA1650AA	133985	Chester	0	0	0	0	0	0	0	0	0	0	0
SAL_WA3317AA	707099	Chester	0	0	0	0	0	0	0	0	0	0	0
SAL_DA1893AA	FDA00000108	Chicago	0	0	0	0	0	0	0	0	0	0	0
SAL_BA5500AA	CFSAN031291	Chicago	0	0	0	0	0	0	0	0	0	0	0
SAL_BA5493AA	CFSAN031298	Chicago	0	0	0	0	0	0	0	0	0	0	0
SAL_FA7272AA	FDA00001637	Chicago	0	0	0	0	0	0	0	0	0	0	0
SAL_CA7676AA	FSF0187	Chicago	0	0	0	0	0	0	0	0	0	0	0
SAL_DA6322AA	FNE0099	Chicago	0	0	0	0	0	0	0	0	0	0	0
SAL_BA5495AA	CFSAN031297	Chicago	0	0	0	0	0	0	0	0	0	0	0
SAL_QA3114AA	PNUSAS041774	Chicago	0	0	0	0	0	0	0	0	0	0	0
SAL_SA7600AA	503425	Chicago	0	0	0	0	0	0	0	0	0	0	0
SAL_SA6156AA	588463	Chicago	0	0	0	0	0	0	0	0	0	0	0
SAL_VA7359AA	201702548	Chicago	0	0	0	0	0	0	0	0	0	0	0
SAL_VA7405AA	201701195	Chicago	0	0	0	0	0	0	0	0	0	0	0
SAL_BA9503AA	A50	Choleraesuis	0	0	0	0	0	0	37	3	67	73	1
SAL_BA9149AA	M227 / NCTC20343	Choleraesuis	0	0	0	0	0	0	0	0	0	0	0
SAL_DA4453AA	RKS1245	Choleraesuis	0	0	0	0	0	0	38	3	68	74	1
SAL_DA5919AA	MDH-2014-00350	Choleraesuis	0	0	0	0	0	0	37	3	69	72	1
SAL_DA4548AA	RKS1235	Choleraesuis	0	0	0	0	0	0	37	3	67	73	1
SAL_FA1057AA	74_8	Choleraesuis	0	0	0	0	0	0	38	3	68	74	1
SAL_FA1054AA	67_1	Choleraesuis	0	0	0	0	0	0	38	3	68	74	1
SAL_FA1051AA	63_2	Choleraesuis	0	0	0	0	0	0	0	0	0	0	0
SAL_FA1049AA	63_3	Choleraesuis	0	0	0	0	0	0	0	3	68	74	1
SAL_FA1056AA	73_1	Choleraesuis	0	0	0	0	0	0	37	3	67	73	1
SAL_FA1028AA	FX_201207049	Choleraesuis	0	0	0	0	0	0	0	0	0	0	0
SAL_FA1043AA	60_2	Choleraesuis	0	0	0	0	0	0	38	3	68	74	1
SAL_FA1048AA	62_6	Choleraesuis	0	0	0	0	0	0	38	3	68	74	1
SAL_FA1046AA	62_2	Choleraesuis	0	0	0	0	0	0	37	3	67	72	1
SAL_FA1047AA	60_3	Choleraesuis	0	0	0	0	0	0	37	56	67	73	1
SAL_FA1029AA	201209171	Choleraesuis	0	0	0	0	0	0	37	3	67	73	1
SAL_FA1037AA	55_9	Choleraesuis	0	0	0	0	0	0	0	0	0	0	0
SAL_FA1045AA	62_4	Choleraesuis	0	0	0	0	0	0	37	3	67	73	1
SAL_FA1025AA	201008276	Choleraesuis	0	0	0	0	0	0	37	3	67	72	21
SAL_FA1016AA	0200_2011	Choleraesuis	0	0	0	0	0	0	37	3	67	73	1
SAL_EA9214AA	FX_88_4	Choleraesuis	0	0	0	0	0	0	0	0	0	0	0
SAL_EA9206AA	68_3	Choleraesuis	0	0	0	0	0	0	0	0	0	0	0
SAL_EA9194AA	04_3622	Choleraesuis	0	0	0	0	0	0	37	3	67	73	1
SAL_EA9167AA	FX_1629_2011	Choleraesuis	0	0	0	0	0	0	0	0	0	0	0
SAL_EA9203AA	67_11	Choleraesuis	0	0	0	0	0	0	38	3	68	74	1
SAL_EA9193AA	04_1262	Choleraesuis	0	0	0	0	0	0	37	3	104	72	1
SAL_EA9170AA	08_0694	Choleraesuis	0	0	0	0	0	0	37	3	67	72	21
SAL_EA9200AA	65_15	Choleraesuis	0	0	0	0	0	0	38	3	68	74	1
SAL_EA9178AA	09_0197	Choleraesuis	0	0	0	0	0	0	37	3	67	72	21
SAL_EA9173AA	55_5	Choleraesuis	0	0	0	0	0	0	37	3	67	73	1
SAL_EA9169AA	05_1616	Choleraesuis	0	0	0	0	0	0	37	58	104	72	1
SAL_EA4970AA	ADRDL-14-18151	Choleraesuis	0	0	0	0	0	0	37	3	67	72	1
SAL_EA2966AA	MDH-2014-00048	Choleraesuis	0	0	0	0	0	0	37	3	67	72	1
SAL_DA7814AA	MDH-2014-00099	Choleraesuis	0	0	0	0	0	0	37	3	67	72	1
SAL_DA7359AA	MDH-2014-00072	Choleraesuis	0	0	0	0	0	0	37	3	67	72	1
SAL_DA7341AA	MDH-2014-00081	Choleraesuis	0	0	0	0	0	0	37	3	120	72	1
SAL_DA7116AA	MDH-2014-00026	Choleraesuis	0	0	0	0	0	0	37	3	67	72	1
SAL_DA7038AA	MDH-2014-00035	Choleraesuis	0	0	0	0	0	0	37	3	69	72	1
SAL_DA6207AA	MDH-2014-00330	Choleraesuis	0	0	0	0	0	0	37	3	69	72	1
SAL_DA6153AA	MDH-2014-00338	Choleraesuis	0	0	0	0	0	0	37	3	69	72	1
SAL_DA6144AA	MDH-2014-00347	Choleraesuis	0	0	0	0	0	0	37	3	69	72	1
SAL_DA6140AA	MDH-2014-00342	Choleraesuis	0	0	0	0	0	0	37	3	69	72	1
SAL_DA6034AA	MDH-2014-00359	Choleraesuis	0	0	0	0	0	0	37	3	67	72	1
SAL_DA6035AA	MDH-2014-00353	Choleraesuis	0	0	0	0	0	0	37	3	67	72	1
SAL_DA5947AA	MDH-2014-00354	Choleraesuis	0	0	0	0	0	0	37	3	69	72	1

SAL_DA5727AA	MDH-2014-00373	Choleraesuis	0	0	0	0	0	0	37	3	67	72	1
SAL_DA5161AA	MDH-2014-00387	Choleraesuis	0	0	0	0	0	0	37	3	67	72	1
SAL_DA5119AA	MDH-2014-00394	Choleraesuis	0	0	0	0	0	0	37	3	67	72	1
SAL_DA4553AA	RKS1241	Choleraesuis	0	0	0	0	0	0	37	3	128	72	1
SAL_DA4552AA	RKS1229	Choleraesuis	0	0	0	0	0	0	37	3	67	72	1
SAL_DA4549AA	05-03241	Choleraesuis	0	0	0	0	0	0	37	3	67	72	1
SAL_DA4551AA	RKS1256	Choleraesuis	0	0	0	0	0	0	38	3	70	74	1
SAL_DA4531AA	CY1	Choleraesuis	0	0	0	0	0	0	37	3	67	72	1
SAL_DA4519AA	RKS1264	Choleraesuis	0	0	0	0	0	0	37	3	67	73	1
SAL_DA4522AA	CN182	Choleraesuis	0	0	0	0	0	0	37	3	67	72	1
SAL_DA4525AA	B27	Choleraesuis	0	0	0	0	0	0	37	3	67	72	1
SAL_DA4526AA	CN101	Choleraesuis	0	0	0	0	0	0	37	3	67	72	1
SAL_DA4459AA	RKS1233	Choleraesuis	0	0	0	0	0	0	38	3	68	74	1
SAL_DA4450AA	RKS1252	Choleraesuis	0	0	0	0	0	0	37	3	67	72	1
SAL_DA4430AA	MDH-2014-00400	Choleraesuis	0	0	0	0	0	0	37	3	67	72	1
SAL_DA4196AA	CFSAN023106	Choleraesuis	0	0	0	0	0	0	37	3	67	72	1
SAL_DA4149AA	CFSAN012639	Choleraesuis	0	0	0	0	0	0	37	3	67	72	1
SAL_DA4094AA	MDH-2014-00443	Choleraesuis	0	0	0	0	0	0	37	3	69	72	1
SAL_DA4000AA	CFSAN023160	Choleraesuis	0	0	0	0	0	0	37	3	67	72	1
SAL_DA3019AA	MDH-2014-00415	Choleraesuis	0	0	0	0	0	0	37	3	69	72	1
SAL_DA2987AA	CFSAN023669	Choleraesuis	0	0	0	0	0	0	38	3	70	74	1
SAL_DA2718AA	MDH-2014-00519	Choleraesuis	0	0	0	0	0	0	37	3	69	72	1
SAL_DA2688AA	MDH-2014-00531	Choleraesuis	0	0	0	0	0	0	37	3	67	72	1
SAL_DA2567AA	MDH-2014-00537	Choleraesuis	0	0	0	0	0	0	37	3	69	72	1
SAL_DA2445AA	MDH-2014-00555	Choleraesuis	0	0	0	0	0	0	37	3	69	72	1
SAL_BA9511AA	SARB4	Choleraesuis	0	0	0	0	0	0	37	3	67	72	1
SAL_BA9158AA	M263 / NCTC20356	Choleraesuis	0	0	0	0	0	0	38	3	85	74	1
SAL_BA9147AA	M179 / NCTC20332	Choleraesuis	0	0	0	0	0	0	37	3	67	73	1
SAL_BA7903AA	93506	Choleraesuis	0	0	0	0	0	0	38	3	85	74	1
SAL_BA5794AA	M55 / NCTC20033	Choleraesuis	0	0	0	0	0	0	37	3	67	73	1
SAL_BA4849AA	CFSAN022628	Choleraesuis	0	0	0	0	0	0	37	3	67	72	1
SAL_BA4843AA	CFSAN022623	Choleraesuis	0	0	0	0	0	0	37	3	67	72	1
SAL_BA4830AA	CFSAN022631	Choleraesuis	0	0	0	0	0	0	0	0	0	0	0
SAL_BA1962AA	ADRDL-14-7194	Choleraesuis	0	0	0	0	0	0	37	3	67	72	1
SAL_BA1953AA	ADRDL-14-13296	Choleraesuis	0	0	0	0	0	0	37	3	67	72	1
SAL_FA6896AA	Sal-598	Choleraesuis	0	0	0	0	0	0	37	3	67	72	1
SAL_GA2859AA	CRJJGF_00052	Choleraesuis	0	0	0	0	0	0	37	3	67	72	1
SAL_GA2765AA	CRJJGF_00148	Choleraesuis	0	0	0	0	0	0	37	3	0	72	1
SAL_GA3992AA	SOP_13	Choleraesuis	0	0	0	0	0	0	37	3	67	72	1
SAL_GA3363AA	BCW_2627	Choleraesuis	0	0	0	0	0	0	37	3	120	72	1
SAL_GA3314AA	CDC 3327/54	Choleraesuis	0	0	0	0	0	0	38	3	68	74	1
SAL_GA3090AA	NCTR-SF22	Choleraesuis	0	0	0	0	0	0	37	3	67	72	1
SAL_GA2511AA	2011K-0282	Choleraesuis	0	0	0	0	0	0	37	3	67	72	1
SAL_GA2492AA	05-0703	Choleraesuis	0	0	0	0	0	0	37	3	67	72	1
SAL_GA2493AA	04-0075	Choleraesuis	0	0	0	0	0	0	38	3	68	74	1
SAL_GA2334AA	BCW_2657	Choleraesuis	0	0	0	0	0	0	37	3	67	72	1
SAL_GA2333AA	BCW_2658	Choleraesuis	0	0	0	0	0	0	38	3	85	74	1
SAL_GA2091AA	BCW_3049	Choleraesuis	0	0	0	0	0	0	38	3	85	74	1
SAL_GA2075AA	BCW_3065	Choleraesuis	0	0	0	0	0	0	38	3	85	74	1
SAL_GA2054AA	BCW_3373	Choleraesuis	0	0	0	0	0	0	0	0	0	0	0
SAL_GA2034AA	BCW_4233	Choleraesuis	0	0	0	0	0	0	37	3	120	72	1
SAL_GA1491AA	2013-60-102-2	Choleraesuis	0	0	0	0	0	0	37	3	67	72	1
SAL_GA1490AA	2013-60-1557	Choleraesuis	0	0	0	0	0	0	37	3	67	72	1
SAL_GA1489AA	12-03619	Choleraesuis	0	0	0	0	0	0	37	3	67	72	1
SAL_GA1488AA	947_2007	Choleraesuis	0	0	0	0	0	0	37	3	67	72	1
SAL_GA1487AA	2007-60-2369-3	Choleraesuis	0	0	0	0	0	0	37	3	67	73	1
SAL_GA1486AA	1221_2012	Choleraesuis	0	0	0	0	0	0	37	3	67	73	1
SAL_GA1484AA	9924281-1_isolate	Choleraesuis	0	0	0	0	0	0	37	3	67	73	1
SAL_GA1485AA	9924031-1	Choleraesuis	0	0	0	0	0	0	37	3	67	73	1
SAL_GA0933AA	gnb00526	Choleraesuis	0	0	0	0	0	0	37	85	67	73	1
SAL_GA0578AA	gnb00217	Choleraesuis	0	0	0	0	0	0	37	85	67	73	1
SAL_GA0383AA	VNB1490	Choleraesuis	0	0	0	0	0	0	38	3	68	74	1
SAL_GA0379AA	VNB1532	Choleraesuis	0	0	0	0	0	0	38	3	68	74	1
SAL_GA0376AA	VNB1581	Choleraesuis	0	0	0	0	0	0	38	3	68	74	1
SAL_GA0333AA	Hue_95	Choleraesuis	0	0	0	0	0	0	38	3	68	74	1
SAL_GA0192AA	1819	Choleraesuis	0	0	0	0	0	0	38	3	68	74	1
SAL_GA0174AA	2399	Choleraesuis	0	0	0	0	0	0	38	3	85	74	1
SAL_GA0145AA	VNB805	Choleraesuis	0	0	0	0	0	0	38	3	68	74	1
SAL_GA0135AA	VNB1081	Choleraesuis	0	0	0	0	0	0	38	3	68	74	1
SAL_GA0129AA	VNB1182	Choleraesuis	0	0	0	0	0	0	38	3	68	74	1
SAL_GA0128AA	VNB1197	Choleraesuis	0	0	0	0	0	0	38	3	68	74	1
SAL_GA0126AA	VNB1223	Choleraesuis	0	0	0	0	0	0	38	3	68	74	1
SAL_GA0125AA	VNB1246	Choleraesuis	0	0	0	0	0	0	38	3	68	74	1

SAL_GA0121AA	VNB1298	Choleraesuis	0	0	0	0	0	0	38	3	68	74	1
SAL_GA0118AA	VNB1347	Choleraesuis	0	0	0	0	0	0	38	3	68	74	1
SAL_FA1026AA	FX_201204175	Choleraesuis	0	0	0	0	0	0	0	0	0	0	0
SAL_GA7664AA	CFSAN037731	Choleraesuis	0	0	0	0	0	0	38	3	68	74	1
SAL_GA8883AA	PNUSAS003726	Choleraesuis	0	0	0	0	0	0	37	3	173	136	1
SAL_HA7555AA	RKI_16-04961	Choleraesuis	0	0	0	0	0	0	38	3	85	74	1
SAL_HA8954AA	A50	Choleraesuis	0	0	0	0	0	0	37	3	67	73	1
SAL_IA2082AA	FSIS1609578	Choleraesuis	0	0	0	0	0	0	37	3	67	72	1
SAL_JA0433AA	PNUSAS010674	Choleraesuis	0	0	0	0	0	0	37	3	104	136	1
SAL_JA0705AA	0-9c4c-11e6-8f55-3c4a9	Choleraesuis	0	0	0	0	0	0	37	85	67	73	1
SAL_JA0704AA	l0-9c4c-11e6-8f55-3c4a9	Choleraesuis	0	0	0	0	0	0	37	85	67	73	1
SAL_JA0706AA	0-9c4c-11e6-8f55-3c4a9	Choleraesuis	0	0	0	0	0	0	37	85	67	73	1
SAL_JA0700AA	0-9c4c-11e6-8f55-3c4a9	Choleraesuis	0	0	0	0	0	0	37	85	67	73	1
SAL_JA3279AA	PNUSAS011847	Choleraesuis	0	0	0	0	0	0	37	3	67	72	1
SAL_KA4068AA	UMxr_1797	Choleraesuis	0	0	0	0	0	0	37	3	67	72	1
SAL_KA4069AA	UMxr_890	Choleraesuis	0	0	0	0	0	0	37	3	67	72	1
SAL_KA4198AA	UMxr_995	Choleraesuis	0	0	0	0	0	0	37	3	67	72	1
SAL_KA4199AA	UMxr_1000	Choleraesuis	0	0	0	0	0	0	37	3	67	72	1
SAL_KA4205AA	UMxr_1382	Choleraesuis	0	0	0	0	0	0	37	3	67	72	1
SAL_KA4206AA	UMxr_973	Choleraesuis	0	0	0	0	0	0	37	3	67	72	1
SAL_KA4213AA	UMxr_1785	Choleraesuis	0	0	0	0	0	0	37	3	67	72	1
SAL_KA4214AA	UMxr_971	Choleraesuis	0	0	0	0	0	0	37	3	67	72	1
SAL_KA4220AA	UMxr_1794	Choleraesuis	0	0	0	0	0	0	37	3	67	73	1
SAL_KA4170AA	UMxr_994	Choleraesuis	0	0	0	0	0	0	37	3	67	72	1
SAL_KA4176AA	UMxr_861	Choleraesuis	0	0	0	0	0	0	37	3	67	72	1
SAL_KA4177AA	UMxr_932	Choleraesuis	0	0	0	0	0	0	37	3	67	72	1
SAL_KA4184AA	UMxr_986	Choleraesuis	0	0	0	0	0	0	37	3	67	72	1
SAL_KA4185AA	UMxr_928	Choleraesuis	0	0	0	0	0	0	37	3	67	72	1
SAL_KA4147AA	UMxr_826	Choleraesuis	0	0	0	0	0	0	38	3	68	74	1
SAL_KA4148AA	UMxr_1793	Choleraesuis	0	0	0	0	0	0	37	3	67	73	1
SAL_KA4155AA	UMxr_829	Choleraesuis	0	0	0	0	0	0	38	3	68	74	1
SAL_KA4156AA	UMxr_1789	Choleraesuis	0	0	0	0	0	0	37	3	67	72	1
SAL_KA4157AA	UMxr_967	Choleraesuis	0	0	0	0	0	0	37	3	67	72	1
SAL_KA4163AA	UMxr_984	Choleraesuis	0	0	0	0	0	0	37	3	67	72	1
SAL_KA4168AA	UMxr_934	Choleraesuis	0	0	0	0	0	0	38	3	68	74	1
SAL_KA4169AA	UMxr_981	Choleraesuis	0	0	0	0	0	0	37	3	67	72	1
SAL_KA4149AA	UMxr_966	Choleraesuis	0	0	0	0	0	0	37	3	67	72	1
SAL_LA9327AA	PNUSAS023652	Choleraesuis	0	0	0	0	0	0	37	3	69	72	1
SAL_BA8292AA	LNT182	Choleraesuis	0	0	0	0	0	0	37	3	67	72	1
SAL_DA5984AA	MDH-2014-00352	Choleraesuis	0	0	0	0	0	0	37	3	69	72	1
SAL_BA9141AA	M244 / NCTC20350	Choleraesuis	0	0	0	0	0	0	38	3	70	74	1
SAL_DA4544AA	RKS1228	Choleraesuis	0	0	0	0	0	0	37	3	67	72	1
SAL_DA4545AA	48-0391	Choleraesuis	0	0	0	0	0	0	0	0	0	0	0
SAL_DA4554AA	RKS1253	Choleraesuis	0	0	0	0	0	0	37	3	67	72	1
SAL_EA9197AA	57_1_CS	Choleraesuis	0	0	0	0	0	0	54	3	103	89	1
SAL_EA9761AA	C500	Choleraesuis	0	0	0	0	0	0	0	0	0	0	0
SAL_GA2520AA	07-0094	Choleraesuis	0	0	0	0	0	0	38	3	85	74	1
SAL_HA6274AA	PNUSAS005586	Choleraesuis	0	0	0	0	0	0	0	0	0	0	0
SAL_GA0579AA	gnb00435	Choleraesuis	0	0	0	0	0	0	37	85	67	73	1
SAL_GA0124AA	VNB1247	Choleraesuis	0	0	0	0	0	0	38	3	85	74	1
SAL_EA9784AA	SC-B67	Choleraesuis	0	0	0	0	0	0	37	3	67	72	1
SAL_GA1483AA	9924286-1_63	Choleraesuis	0	0	0	0	0	0	37	3	67	73	1
SAL_GA1492AA	2012-60-3235-1	Choleraesuis	0	0	0	0	0	0	37	3	67	72	1
SAL_FA8219AA	ARIM-SCHS1083-05	Choleraesuis	0	0	0	0	0	0	37	3	67	73	1
SAL_EA9183AA	61_1_CS	Choleraesuis	0	0	0	0	0	0	37	3	67	73	1
SAL_KA8725AA	SARB5	Choleraesuis	0	0	0	0	0	0	0	0	0	0	0
SAL_MA6907AA	ADRDL-1604	Choleraesuis	0	0	0	0	0	0	37	3	69	72	1
SAL_MA7668AA	ADRDL-1591	Choleraesuis	0	0	0	0	0	0	37	3	67	72	1
SAL_NA0076AA	258-15	Choleraesuis	0	0	0	0	0	0	37	3	67	72	1
SAL_NA0146AA	138-15	Choleraesuis	0	0	0	0	0	0	37	3	67	72	1
SAL_NA0158AA	109-16	Choleraesuis	0	0	0	0	0	0	37	3	67	72	1
SAL_NA2233AA	1629_11	Choleraesuis	0	0	0	0	0	0	0	0	0	0	0
SAL_NA2185AA	0201_11	Choleraesuis	0	0	0	0	0	0	37	3	67	73	1
SAL_NA2215AA	1342_12	Choleraesuis	0	0	0	0	0	0	37	3	67	73	1
SAL_NA2132AA	1834_13	Choleraesuis	0	0	0	0	0	0	0	0	0	0	0
SAL_NA2173AA	0200_11	Choleraesuis	0	0	0	0	0	0	37	3	67	73	1
SAL_NA2176AA	0002_10	Choleraesuis	0	0	0	0	0	0	37	3	67	72	1
SAL_NA5151AA	PNUSAS030481	Choleraesuis	0	0	0	0	0	0	37	3	104	136	1
SAL_OA0133AA	PNUSAS026780	Choleraesuis	0	0	0	0	0	0	0	0	0	0	0
SAL_OA2046AA	140593	Choleraesuis	0	0	0	0	0	0	37	3	67	73	1
SAL_OA2047AA	142490	Choleraesuis	0	0	0	0	0	0	37	3	67	72	1
SAL_OA2122AA	195537	Choleraesuis	0	0	0	0	0	0	38	3	85	74	1
SAL_OA2129AA	424599	Choleraesuis	0	0	0	0	0	0	37	3	67	73	1

SAL_OA2132AA	488618	Choleraesuis	0	0	0	0	0	0	38	3	85	74	1
SAL_OA2131AA	446499	Choleraesuis	0	0	0	0	0	0	37	3	67	72	1
SAL_OA2133AA	488620	Choleraesuis	0	0	0	0	0	0	0	0	0	0	0
SAL_PA0758AA	488621	Choleraesuis	0	0	0	0	0	0	0	0	0	0	0
SAL_PA0763AA	241773	Choleraesuis	0	0	0	0	0	0	37	3	104	72	1
SAL_OA2121AA	159903	Choleraesuis	0	0	0	0	0	0	38	3	85	74	1
SAL_OA4203AA	RKS1222	Choleraesuis	0	0	0	0	0	0	37	3	67	72	1
SAL_OA4204AA	CN231	Choleraesuis	0	0	0	0	0	0	37	3	67	72	1
SAL_OA7431AA	PNUSAS036848	Choleraesuis	0	0	0	0	0	0	37	3	104	136	1
SAL_PA0131AA	6447/88	Choleraesuis	0	0	0	0	0	0	37	3	67	215	1
SAL_PA0130AA	9519/04	Choleraesuis	0	0	0	0	0	0	37	3	67	72	1
SAL_PA0136AA	H2538	Choleraesuis	0	0	0	0	0	0	37	3	67	72	1
SAL_PA0132AA	6631/88	Choleraesuis	0	0	0	0	0	0	38	3	68	74	1
SAL_PA0139AA	H2524	Choleraesuis	0	0	0	0	0	0	37	3	67	72	1
SAL_PA0140AA	H2490	Choleraesuis	0	0	0	0	0	0	37	3	67	0	1
SAL_PA0141AA	H2493	Choleraesuis	0	0	0	0	0	0	37	3	67	72	1
SAL_PA0147AA	1358856	Choleraesuis	0	0	0	0	0	0	37	3	67	216	1
SAL_PA0148AA	8538/96	Choleraesuis	0	0	0	0	0	0	38	3	68	74	1
SAL_PA0152AA	9520/04	Choleraesuis	0	0	0	0	0	0	37	3	67	72	1
SAL_PA0150AA	5176/85	Choleraesuis	0	0	0	0	0	0	37	3	67	73	1
SAL_PA0151AA	34K	Choleraesuis	0	0	0	0	0	0	38	3	85	74	1
SAL_PA0155AA	UMxr_927	Choleraesuis	0	0	0	0	0	0	37	3	67	72	1
SAL_PA0138AA	H2531	Choleraesuis	0	0	0	0	0	0	37	3	67	72	1
SAL_PA0137AA	H2487	Choleraesuis	0	0	0	0	0	0	37	3	67	72	1
SAL_PA0153AA	2058964	Choleraesuis	0	0	0	0	0	0	37	3	104	72	1
SAL_PA0162AA	RKS1257	Choleraesuis	0	0	0	0	0	0	37	3	67	72	1
SAL_PA0167AA	NVSL6321	Choleraesuis	0	0	0	0	0	0	37	3	67	72	1
SAL_PA0159AA	M273	Choleraesuis	0	0	0	0	0	0	37	3	67	72	1
SAL_PA0158AA	M193	Choleraesuis	0	0	0	0	0	0	37	3	67	72	1
SAL_PA0169AA	UMxr_987	Choleraesuis	0	0	0	0	0	0	37	3	67	72	1
SAL_PA0177AA	UMxr_929	Choleraesuis	0	0	0	0	0	0	37	3	67	72	1
SAL_PA0111AA	RKS1248	Choleraesuis	0	0	0	0	0	0	38	3	68	74	1
SAL_PA0109AA	RKS1249	Choleraesuis	0	0	0	0	0	0	38	3	85	74	1
SAL_PA0110AA	RKS1263	Choleraesuis	0	0	0	0	0	0	37	3	67	72	1
SAL_PA0124AA	96-01171	Choleraesuis	0	0	0	0	0	0	37	3	67	72	21
SAL_OA9972AA	PNUSAS037704	Choleraesuis	0	0	0	0	0	0	37	3	104	136	1
SAL_PA0641AA	ST-21402	Choleraesuis	0	0	0	0	0	0	37	3	67	72	1
SAL_PA0765AA	265721	Choleraesuis	0	0	0	0	0	0	38	3	85	74	1
SAL_PA0143AA	8629/96	Choleraesuis	0	0	0	0	0	0	37	3	67	72	1
SAL_PA1636AA	PNUSAS038071	Choleraesuis	0	0	0	0	0	0	37	3	104	136	1
SAL_PA4274AA	135017	Choleraesuis	0	0	0	0	0	0	37	3	67	73	1
SAL_PA4535AA	24043	Choleraesuis	0	0	0	0	0	0	0	0	0	0	0
SAL_PA6418AA	477986	Choleraesuis	0	0	0	0	0	0	0	0	0	0	0
SAL_PA8568AA	520482	Choleraesuis	0	0	0	0	0	0	38	3	85	74	1
SAL_QA1507AA	454852	Choleraesuis	0	0	0	0	0	0	38	3	85	74	1
SAL_QA1454AA	488622	Choleraesuis	0	0	0	0	0	0	37	3	67	212	1
SAL_QA5515AA	345356	Choleraesuis	0	0	0	0	0	0	0	0	0	0	0
SAL_QA9030AA	PNUSAS044337	Choleraesuis	0	0	0	0	0	0	37	3	67	72	1
SAL_RA0047AA	MOD1_Per61	Choleraesuis	0	0	0	0	0	0	38	3	68	74	1
SAL_RA8554AA	543921	Choleraesuis	0	0	0	0	0	0	38	3	85	74	1
SAL_RA8555AA	543923	Choleraesuis	0	0	0	0	0	0	37	3	67	73	1
SAL_RA8557AA	543925	Choleraesuis	0	0	0	0	0	0	37	3	67	73	1
SAL_RA8562AA	543935	Choleraesuis	0	0	0	0	0	0	37	3	67	72	1
SAL_RA4160AA	484110	Choleraesuis	0	0	0	0	0	0	0	0	0	0	0
SAL_RA4473AA	543915	Choleraesuis	0	0	0	0	0	0	38	3	68	74	1
SAL_RA5654AA	545237	Choleraesuis	0	0	0	0	0	0	37	3	67	73	1
SAL_RA6217AA	PNUSAS046397	Choleraesuis	0	0	0	0	0	0	37	3	67	72	1
SAL_RA7373AA	488619	Choleraesuis	0	0	0	0	0	0	38	3	68	74	1
SAL_RA7292AA	423189	Choleraesuis	0	0	0	0	0	0	38	3	85	74	1
SAL_RA7749AA	520481	Choleraesuis	0	0	0	0	0	0	38	3	85	74	1
SAL_RA7669AA	543937	Choleraesuis	0	0	0	0	0	0	38	3	85	74	1
SAL_RA8357AA	103757	Choleraesuis	0	0	0	0	0	0	37	3	67	72	1
SAL_KA0254AA	63951	Choleraesuis	0	0	0	0	0	0	37	3	67	72	1
SAL_SA5191AA	IVB651/79	Choleraesuis	0	0	0	0	0	0	0	0	0	0	0
SAL_SA5027AA	S01-0409	Choleraesuis	0	0	0	0	0	0	37	3	67	72	1
SAL_SA3858AA	SAL-18-VL-SD-MN-0002	Choleraesuis	0	0	0	0	0	0	37	3	67	72	1
SAL_SA7045AA		Choleraesuis	0	0	0	0	0	0	37	3	67	72	1
SAL_TA0513AA	2967-89	Choleraesuis	0	0	0	0	0	0	37	3	67	72	1
SAL_TA0577AA	Sal17	Choleraesuis	0	0	0	0	0	0	37	3	67	73	1
SAL_TA0576AA	Sal21	Choleraesuis	0	0	0	0	0	0	37	3	67	73	1
SAL_TA0578AA	Sal9	Choleraesuis	0	0	0	0	0	0	37	3	67	73	1
SAL_TA0580AA	Sal13	Choleraesuis	0	0	0	0	0	0	37	3	67	73	1
SAL_TA0573AA	Sal32	Choleraesuis	0	0	0	0	0	0	37	3	67	73	1

SAL_TA0582AA	1	Choleraesuis	0	0	0	0	0	0	37	3	67	73	1
SAL_TA0581AA	Sal8	Choleraesuis	0	0	0	0	0	0	37	3	67	73	1
SAL_TA3966AA		Choleraesuis	0	0	0	0	0	0	38	3	68	74	1
SAL_TA3967AA		Choleraesuis	0	0	0	0	0	0	38	3	68	74	1
SAL_TA3968AA		Choleraesuis	0	0	0	0	0	0	37	3	67	72	1
SAL_OA2478AA	MOD1-9240-Rabbit	Choleraesuis	0	0	0	0	0	0	37	3	120	72	1
SAL_TA7652AA	R16.0365	Choleraesuis	0	0	0	0	0	0	37	3	67	72	1
SAL_TA7576AA	R16.0268	Choleraesuis	0	0	0	0	0	0	37	3	67	72	1
SAL_TA7577AA	R16.0267	Choleraesuis	0	0	0	0	0	0	37	0	67	72	1
SAL_PA3810AA	DSM 14846	Choleraesuis	0	0	0	0	0	0	38	3	85	74	1
SAL_PA0123AA	8892/98	Choleraesuis	0	0	0	0	0	0	37	3	67	72	1
SAL_PA0156AA	UMxr_843	Choleraesuis	0	0	0	0	0	0	37	3	67	72	1
SAL_PA0157AA	UMxr_970	Choleraesuis	0	0	0	0	0	0	37	3	67	72	1
SAL_UA4797AA	SAL-18-VL-SD-MN-0003	Choleraesuis	0	0	0	0	0	0	37	3	120	72	1
SAL_UA8057AA	ADRDL-2289	Choleraesuis	0	0	0	0	0	0	37	3	67	72	1
SAL_VA0697AA	201810583	Choleraesuis	0	0	0	0	0	0	0	0	0	0	0
SAL_VA0703AA	201811043	Choleraesuis	0	0	0	0	0	0	0	0	0	0	0
SAL_VA0743AA	543923	Choleraesuis	0	0	0	0	0	0	37	3	67	73	1
SAL_VA0488AA	SAL-18-VL-SD-IA-0019	Choleraesuis	0	0	0	0	0	0	37	3	67	72	1
SAL_VA0489AA	SAL-18-VL-SD-MN-0014	Choleraesuis	0	0	0	0	0	0	37	3	120	72	1
SAL_VA0896AA	ADRDL-2396	Choleraesuis	0	0	0	0	0	0	37	3	120	72	1
SAL_VA0333AA	543935	Choleraesuis	0	0	0	0	0	0	37	3	67	72	1
SAL_VA0879AA	ADRDL-2398	Choleraesuis	0	0	0	0	0	0	37	3	67	72	1
SAL_VA6995AA	ETR001	Choleraesuis	3	0	0	0	0	0	37	3	362	4	1
SAL_VA1387AA	488621	Choleraesuis	0	0	0	0	0	0	0	0	0	0	0
SAL_VA1405AA	488620	Choleraesuis	0	0	0	0	0	0	0	0	0	0	0
SAL_VA1359AA	488618	Choleraesuis	0	0	0	0	0	0	38	3	85	74	1
SAL_VA7364AA	201701366	Choleraesuis	0	0	0	0	0	0	0	0	0	0	0
SAL_VA7386AA	201700038	Choleraesuis	0	0	0	0	0	0	0	0	0	0	0
SAL_VA7390AA	201700973	Choleraesuis	0	0	0	0	0	0	0	0	0	0	0
SAL_VA7393AA	201701143	Choleraesuis	0	0	0	0	0	0	0	0	0	0	0
SAL_VA7409AA	201701227	Choleraesuis	0	0	0	0	0	0	0	0	0	0	0
SAL_VA7423AA	201702196	Choleraesuis	0	0	0	0	0	0	0	0	0	0	0
SAL_VA7429AA	201702512	Choleraesuis	0	0	0	0	0	0	0	0	0	0	0
SAL_VA7431AA	201702513	Choleraesuis	0	0	0	0	0	0	0	0	0	0	0
SAL_VA7476AA	201704501	Choleraesuis	0	0	0	0	0	0	0	0	0	0	0
SAL_VA7477AA	201704376	Choleraesuis	0	0	0	0	0	0	0	0	0	0	0
SAL_VA7535AA	201705735	Choleraesuis	0	0	0	0	0	0	0	0	0	0	0
SAL_VA7603AA	201707173	Choleraesuis	0	0	0	0	0	0	0	0	0	0	0
SAL_VA7624AA	201707505	Choleraesuis	0	0	0	0	0	0	0	0	0	0	0
SAL_VA7673AA	201710769	Choleraesuis	0	0	0	0	0	0	0	0	0	0	0
SAL_VA2925AA	04-03358	Choleraesuis	0	0	0	0	0	0	38	3	68	74	1
SAL_VA2926AA	0217_2011	Choleraesuis	0	0	0	0	0	0	37	3	67	72	1
SAL_VA2927AA	01-03680	Choleraesuis	0	0	0	0	0	0	38	3	68	74	1
SAL_VA2928AA	0001_2010	Choleraesuis	0	0	0	0	0	0	37	3	67	72	1
SAL_VA2902AA	22522	Choleraesuis	0	0	0	0	0	0	37	3	67	72	1
SAL_VA2904AA	21690	Choleraesuis	0	0	0	0	0	0	37	3	67	72	1
SAL_VA2907AA	2013-60-132-1	Choleraesuis	0	0	0	0	0	0	37	3	67	72	1
SAL_VA2909AA	2013-60-102-7	Choleraesuis	0	0	0	0	0	0	37	3	67	72	1
SAL_VA2915AA	1204_2010	Choleraesuis	0	0	0	0	0	0	37	3	67	72	1
SAL_VA2912AA	2007-60-2369-2	Choleraesuis	0	0	0	0	0	0	37	3	67	73	1
SAL_VA2903AA	22501	Choleraesuis	0	0	0	0	0	0	37	3	67	72	1
SAL_VA2917AA	12-P-BA380	Choleraesuis	0	0	0	0	0	0	37	3	67	72	1
SAL_VA2921AA	09-00524	Choleraesuis	0	0	0	0	0	0	37	3	67	73	1
SAL_VA2919AA	12-03467	Choleraesuis	0	0	0	0	0	0	37	3	67	72	1
SAL_VA2911AA	2007-60-2695-3	Choleraesuis	0	0	0	0	0	0	37	3	67	73	1
SAL_VA2914AA	2002_57_Organs	Choleraesuis	0	0	0	0	0	0	37	3	67	72	1
SAL_VA2918AA	12-P-BA1518	Choleraesuis	0	0	0	0	0	0	37	3	67	72	1
SAL_VA2906AA	20765	Choleraesuis	0	0	0	0	0	0	37	3	67	72	1
SAL_VA2916AA	12-P-BA592	Choleraesuis	0	0	0	0	0	0	37	3	67	72	1
SAL_VA2922AA	06-00244	Choleraesuis	0	0	0	0	0	0	38	3	68	74	1
SAL_VA2908AA	2013-60-103-1	Choleraesuis	0	0	0	0	0	0	37	3	67	72	1
SAL_VA2924AA	04-03359	Choleraesuis	0	0	0	0	0	0	38	3	68	74	1
SAL_VA2920AA	10-P-PA2216	Choleraesuis	0	0	0	0	0	0	37	3	67	72	1
SAL_VA2923AA	04CEB50SAL-1	Choleraesuis	0	0	0	0	0	0	37	3	67	72	1
SAL_VA2877AA	ERS1496901	Choleraesuis	0	0	0	0	0	0	37	3	67	73	1
SAL_VA2874AA	9924284-1_Lever	Choleraesuis	0	0	0	0	0	0	37	3	67	73	1
SAL_VA2880AA	9924281-1_Tonsil	Choleraesuis	0	0	0	0	0	0	37	3	67	73	1
SAL_VA2886AA	9924230-1	Choleraesuis	0	0	0	0	0	0	37	3	67	73	1
SAL_VA2890AA	9924195-20	Choleraesuis	0	0	0	0	0	0	37	3	67	73	1
SAL_VA2888AA	9924196-12	Choleraesuis	0	0	0	0	0	0	37	3	67	73	1
SAL_VA2885AA	9924260-13	Choleraesuis	0	0	0	0	0	0	37	3	67	73	1
SAL_VA2872AA	9924286-1_611	Choleraesuis	0	0	0	0	0	0	37	3	67	73	1

SAL_VA2891AA	9924195-18	Choleraesuis	0	0	0	0	0	0	37	3	67	73	1
SAL_VA2892AA	9915794-2	Choleraesuis	0	0	0	0	0	0	37	3	67	73	1
SAL_VA2894AA	772050196	Choleraesuis	0	0	0	0	0	0	37	3	67	73	1
SAL_VA2893AA	95CEB06SAL-2	Choleraesuis	0	0	0	0	0	0	37	3	370	72	1
SAL_VA2879AA	9924283-1_Lung	Choleraesuis	0	0	0	0	0	0	37	3	67	73	1
SAL_VA2895AA	7224252-1	Choleraesuis	0	0	0	0	0	0	37	3	67	73	1
SAL_VA2883AA	9924281-1_Ileoceacale	Choleraesuis	0	0	0	0	0	0	37	3	67	73	1
SAL_VA2897AA	35155	Choleraesuis	0	0	0	0	0	0	37	3	67	72	1
SAL_VA2896AA	7223955-17	Choleraesuis	0	0	0	0	0	0	37	3	67	73	1
SAL_VA2900AA	30163	Choleraesuis	0	0	0	0	0	0	37	3	67	72	1
SAL_VA2905AA	208_P_2001_Organs	Choleraesuis	0	0	0	0	0	0	37	3	67	72	1
SAL_VA2901AA	25042_2007	Choleraesuis	0	0	0	0	0	0	37	3	67	72	1
SAL_VA2842AA	TA12-17181	Choleraesuis	0	0	0	0	0	0	37	3	67	72	1
SAL_VA2844AA	TA12-14010	Choleraesuis	0	0	0	0	0	0	37	3	67	72	1
SAL_VA2846AA	TA11-04807	Choleraesuis	0	0	0	0	0	0	37	3	67	72	1
SAL_VA2848AA	TA10-18838	Choleraesuis	0	0	0	0	0	0	37	3	67	72	1
SAL_VA2843AA	TA12-14668	Choleraesuis	0	0	0	0	0	0	37	3	67	72	1
SAL_VA2850AA	SCS-IT-34	Choleraesuis	0	0	0	0	0	0	37	3	67	72	1
SAL_VA2852AA	SCS-IT-25	Choleraesuis	0	0	0	0	0	0	37	3	67	72	1
SAL_VA2854AA	SCS-IT-1	Choleraesuis	0	0	0	0	0	0	37	3	67	72	1
SAL_VA2847AA	TA10-23756	Choleraesuis	0	0	0	0	0	0	37	3	67	72	1
SAL_VA2856AA	MRS-13-00384	Choleraesuis	0	0	0	0	0	0	37	3	67	72	1
SAL_VA2851AA	SCS-IT-29	Choleraesuis	0	0	0	0	0	0	38	3	68	74	1
SAL_VA2845AA	TA11-7639	Choleraesuis	0	0	0	0	0	0	37	3	67	72	1
SAL_VA2859AA	MRS-13-00231	Choleraesuis	0	0	0	0	0	0	37	3	67	72	1
SAL_VA2853AA	SCS-IT-21	Choleraesuis	0	0	0	0	0	0	38	3	68	74	1
SAL_VA2849AA	SCS-IT-38	Choleraesuis	0	0	0	0	0	0	38	3	68	74	1
SAL_VA2861AA	9924364-10	Choleraesuis	0	0	0	0	0	0	37	3	67	73	1
SAL_VA2857AA	MRS-13-00383	Choleraesuis	0	0	0	0	0	0	38	3	68	74	1
SAL_VA2860AA	9924636-1	Choleraesuis	0	0	0	0	0	0	37	3	67	73	1
SAL_VA2858AA	MRS-13-00335	Choleraesuis	0	0	0	0	0	0	37	3	67	72	1
SAL_VA2862AA	9924325-4	Choleraesuis	0	0	0	0	0	0	37	3	67	73	1
SAL_VA2866AA	9924288-1_667	Choleraesuis	0	0	0	0	0	0	37	3	67	73	1
SAL_VA2855AA	MRS-13-00407	Choleraesuis	0	0	0	0	0	0	37	3	67	72	1
SAL_VA2838AA	TA13-2369	Choleraesuis	0	0	0	0	0	0	37	3	67	72	86
SAL_VA2839AA	TA12-9851	Choleraesuis	0	0	0	0	0	0	37	3	67	72	1
SAL_VA2837AA	TA13-677	Choleraesuis	0	0	0	0	0	0	37	3	67	72	1
SAL_VA2840AA	TA12-7256	Choleraesuis	0	0	0	0	0	0	37	3	67	72	1
SAL_VA3527AA	ERS2045594	Choleraesuis	0	0	0	0	0	0	37	3	67	72	1
SAL_VA2841AA	TA12-5803	Choleraesuis	0	0	0	0	0	0	37	3	67	72	1
SAL_VA1925AA	446499	Choleraesuis	0	0	0	0	0	0	37	3	67	72	1
SAL_VA2419AA	424599	Choleraesuis	0	0	0	0	0	0	37	3	67	73	1
SAL_VA5937AA	SA20052655	Choleraesuis	0	0	0	0	0	0	37	3	67	72	1
SAL_VA5891AA	SA20041838	Choleraesuis	0	0	0	0	0	0	37	3	67	136	1
SAL_VA5901AA	SA20055321	Choleraesuis	18	3	5	0	3	0	37	3	67	72	1
SAL_VA5857AA	SA20033187	Choleraesuis	0	0	0	0	0	0	37	3	67	136	1
SAL_VA5789AA	SA20080939	Choleraesuis	0	0	0	0	0	0	37	3	67	72	1
SAL_VA5679AA	SA20052656	Choleraesuis	0	0	0	0	0	0	37	3	67	72	1
SAL_VA5552AA	CFS0016	Choleraesuis	0	0	0	0	0	0	0	0	0	0	0
SAL_VA5553AA	CFS0015	Choleraesuis	0	0	0	0	0	0	38	3	85	74	1
SAL_VA2910AA	2007-60-2695-5	Choleraesuis	0	0	0	0	0	0	37	3	67	73	1
SAL_VA2913AA	2007-60-2369-1	Choleraesuis	0	0	0	0	0	0	37	3	67	73	1
SAL_VA9254AA	CFSAN091477	Choleraesuis	0	0	0	0	0	0	0	0	0	0	0
SAL_VA9941AA	241773	Choleraesuis	0	0	0	0	0	0	37	3	104	72	1
SAL_WA3197AA	96-02404	Choleraesuis	0	0	0	0	0	0	38	3	68	74	1
SAL_WA2919AA	06-01706	Choleraesuis	0	0	0	0	0	0	37	3	67	73	1
SAL_WA2910AA	96-08742	Choleraesuis	0	0	0	0	0	0	38	3	68	74	1
SAL_WA2248AA	00-01049	Choleraesuis	0	0	0	0	0	0	38	3	68	74	1
SAL_WA2249AA	00-01048	Choleraesuis	0	0	0	0	0	0	38	3	68	74	1
SAL_WA1385AA	SAL-18-VL-SD-IA-0032	Choleraesuis	0	0	0	0	0	0	37	3	67	72	1
SAL_VA2878AA	24284-1_Blindtarmsindh	Choleraesuis	0	0	0	0	0	0	37	3	67	73	1
SAL_WA2086AA		Choleraesuis	0	0	0	0	0	0	0	0	0	0	0
SAL_WA2091AA		Choleraesuis	0	0	0	0	0	0	0	0	0	0	0
SAL_VA2899AA	33820	Choleraesuis	0	0	0	0	0	0	37	3	67	72	1
SAL_VA3566AA	NRRL B-4212	Choleraesuis	0	0	0	0	0	0	38	3	70	74	1
SAL_MA3205AA	PNUSAS024072	Clackamas	0	0	0	0	0	0	0	0	0	0	0
SAL_KA1354AA	2015AM-0199	Clackamas	0	0	0	0	0	0	0	0	0	0	0
SAL_MA6245AA	PNUSAS026124	Clackamas	0	0	0	0	0	0	0	0	0	0	0
SAL_MA7083AA	PNUSAS027116	Clackamas	0	0	0	0	0	0	0	0	0	0	0
SAL_NA0870AA	PNUSAS028142	Clackamas	0	0	0	0	0	0	0	0	0	0	0
SAL_RA6758AA	PNUSAS045764	Clackamas	0	0	0	0	0	0	0	0	0	0	0
SAL_FA8197AA	FDA00010291	Cleveland	0	0	0	0	0	0	0	0	0	0	0
SAL_KA0803AA	PNUSAS015590	Cleveland	0	0	0	0	0	0	0	0	0	0	0

SAL_LA5012AA	PNUSAS020138	Cleveland	0	0	0	0	0	0	0	0	0	0	0
SAL_NA2424AA	PNUSAS030273	Cochise	0	0	0	0	0	0	0	0	0	0	0
SAL_EA7345AA	53058	Coeln	0	0	0	0	0	0	0	0	0	0	0
SAL_EA1011AA	5-sc-2013-02-28T11:35:1	Coeln	0	0	0	0	0	0	0	0	0	0	0
SAL_EA0947AA	3-sc-2013-02-28T11:34:5	Coeln	0	0	0	0	0	0	0	0	0	0	0
SAL_EA0036AA	FSF0016	Coeln	0	0	0	0	0	0	0	0	0	0	0
SAL_DA1433AA	H123960555	Coeln	0	0	0	0	0	0	0	0	0	0	0
SAL_DA1423AA	H121660242	Coeln	0	0	0	0	0	0	0	0	0	0	0
SAL_DA1395AA	H124080645	Coeln	0	0	0	0	0	0	0	0	0	0	0
SAL_DA1381AA	H124080646	Coeln	0	0	0	0	0	0	0	0	0	0	0
SAL_CA6491AA	60080	Coeln	0	0	0	0	0	0	0	0	0	0	0
SAL_CA6332AA	48274	Coeln	0	0	0	0	0	0	0	0	0	0	0
SAL_CA6316AA	60041	Coeln	0	0	0	0	0	0	0	0	0	0	0
SAL_CA6001AA	48261	Coeln	0	0	0	0	0	0	0	0	0	0	0
SAL_CA4728AA	63037	Coeln	0	0	0	0	0	0	0	0	0	0	0
SAL_CA4428AA	61886	Coeln	0	0	0	0	0	0	0	0	0	0	0
SAL_CA4280AA	34161	Coeln	0	0	0	0	0	0	0	0	0	0	0
SAL_CA4116AA	45992	Coeln	0	0	0	0	0	0	0	0	0	0	0
SAL_CA3861AA	63924	Coeln	0	0	0	0	0	0	0	0	0	0	0
SAL_CA2812AA	61883	Coeln	0	0	0	0	0	0	0	0	0	0	0
SAL_CA2693AA	37100	Coeln	0	0	0	0	0	0	0	0	0	0	0
SAL_CA2126AA	36490	Coeln	0	0	0	0	0	0	0	0	0	0	0
SAL_CA1880AA	41967	Coeln	0	0	0	0	0	0	0	0	0	0	0
SAL_CA0654AA	63592	Coeln	0	0	0	0	0	0	0	0	0	0	0
SAL_BA9585AA	65411	Coeln	0	0	0	0	0	0	0	0	0	0	0
SAL_BA9552AA	55557	Coeln	0	0	0	0	0	0	0	0	0	0	0
SAL_BA8505AA	63032	Coeln	0	0	0	0	0	0	0	0	0	0	0
SAL_BA8336AA	37797	Coeln	0	0	0	0	0	0	0	0	0	0	0
SAL_BA7697AA	37067	Coeln	0	0	0	0	0	0	0	0	0	0	0
SAL_HA1958AA	S02923-14	Coeln	0	0	0	0	0	0	0	0	0	0	0
SAL_HA8890AA	NCTC6754	Coeln	0	0	0	0	0	0	0	0	0	0	0
SAL_IA0727AA	128190	Coeln	0	0	0	0	0	0	0	0	0	0	0
SAL_IA3003AA	285708	Coeln	0	0	0	0	0	0	0	0	0	0	0
SAL_IA4726AA	MS160224	Coeln	0	0	0	0	0	0	0	0	0	0	0
SAL_IA6701AA	MS150327	Coeln	0	0	0	0	0	0	0	0	0	0	0
SAL_IA8370AA	PNUSAS009059	Coeln	0	0	0	0	0	0	0	0	0	0	0
SAL_JA2910AA	PNUSAS011980	Coeln	0	0	0	0	0	0	0	0	0	0	0
SAL_KA0505AA	PNUSAS016165	Coeln	0	0	0	0	0	0	0	0	0	0	0
SAL_KA7359AA	PNUSAS018379	Coeln	0	0	0	0	0	0	0	0	0	0	0
SAL_LA4607AA	PNUSAS021601	Coeln	0	0	0	0	0	0	0	0	0	0	0
SAL_LA6558AA	PNUSAS022521	Coeln	0	0	0	0	0	0	0	0	0	0	0
SAL_LA3377AA	MS170212	Coeln	0	0	0	0	0	0	0	0	0	0	0
SAL_MA5456AA	28-18-8101205	Coeln	0	0	0	0	0	0	0	0	0	0	0
SAL_DA6124AA	MOD1_Sal_850	Coeln	0	0	0	0	0	0	0	0	0	0	0
SAL_BA6607AA	S-0435	Coeln	0	0	0	0	0	0	0	0	0	0	0
SAL_CA6466AA	60119	Coeln	0	0	0	0	0	0	0	0	0	0	0
SAL_FA4706AA	170372	Coeln	0	0	0	0	0	0	0	0	0	0	0
SAL_NA2032AA	33-6-8101298	Coeln	0	0	0	0	0	0	0	0	0	0	0
SAL_NA3554AA	PNUSAS027637	Coeln	0	0	0	0	0	0	0	0	0	0	0
SAL_NA9933AA	PNUSAS034199	Coeln	0	0	0	0	0	0	0	0	0	0	0
SAL_OA1727AA	161403_2	Coeln	0	0	0	0	0	0	0	0	0	0	0
SAL_OA1725AA	161378_2	Coeln	0	0	0	0	0	0	0	0	0	0	0
SAL_OA1573AA	161378	Coeln	0	0	0	0	0	0	0	0	0	0	0
SAL_OA1577AA	161403	Coeln	0	0	0	0	0	0	0	0	0	0	0
SAL_PA1757AA	12-21-18-8100211-Q	Coeln	0	0	0	0	0	0	0	0	0	0	0
SAL_PA4778AA	413183	Coeln	0	0	0	0	0	0	0	0	0	0	0
SAL_PA4816AA	385817	Coeln	0	0	0	0	0	0	0	0	0	0	0
SAL_PA6223AA	311448	Coeln	0	0	0	0	0	0	0	0	0	0	0
SAL_PA7510AA	315000	Coeln	0	0	0	0	0	0	0	0	0	0	0
SAL_PA7756AA	302940	Coeln	0	0	0	0	0	0	0	0	0	0	0
SAL_PA7808AA	175567	Coeln	0	0	0	0	0	0	0	0	0	0	0
SAL_PA8355AA	427531	Coeln	0	0	0	0	0	0	0	0	0	0	0
SAL_PA9020AA	138395	Coeln	0	0	0	0	0	0	0	0	0	0	0
SAL_PA8874AA	423130	Coeln	0	0	0	0	0	0	0	0	0	0	0
SAL_PA9511AA	343421	Coeln	0	0	0	0	0	0	0	0	0	0	0
SAL_PA9515AA	176276	Coeln	0	0	0	0	0	0	0	0	0	0	0
SAL_QA0386AA	322504	Coeln	0	0	0	0	0	0	0	0	0	0	0
SAL_QA1891AA	420518	Coeln	0	0	0	0	0	0	0	0	0	0	0
SAL_QA2715AA	408512	Coeln	0	0	0	0	0	0	0	0	0	0	0
SAL_QA4792AA	170413	Coeln	0	0	0	0	0	0	0	0	0	0	0
SAL_QA5876AA	155640	Coeln	0	0	0	0	0	0	0	0	0	0	0
SAL_QA6250AA	419059	Coeln	0	0	0	0	0	0	0	0	0	0	0
SAL_QA6638AA	391111	Coeln	0	0	0	0	0	0	0	0	0	0	0

SAL_QA7292AA	389714	Coeln	0	0	0	0	0	0	0	0	0	0	0
SAL_QA7271AA	271149	Coeln	0	0	0	0	0	0	0	0	0	0	0
SAL_QA7934AA	391307	Coeln	0	0	0	0	0	0	0	0	0	0	0
SAL_QA7887AA	166288	Coeln	0	0	0	0	0	0	0	0	0	0	0
SAL_QA7838AA	387528	Coeln	0	0	0	0	0	0	0	0	0	0	0
SAL_QA7646AA	431292	Coeln	0	0	0	0	0	0	0	0	0	0	0
SAL_QA7511AA	167057	Coeln	0	0	0	0	0	0	0	0	0	0	0
SAL_QA8386AA	307235	Coeln	0	0	0	0	0	0	0	0	0	0	0
SAL_QA8451AA	157246	Coeln	0	0	0	0	0	0	0	0	0	0	0
SAL_QA8282AA	159674	Coeln	0	0	0	0	0	0	0	0	0	0	0
SAL_QA9327AA	161840	Coeln	0	0	0	0	0	0	0	0	0	0	0
SAL_RA2458AA	:278-da88-47bf-bcea-6d9	Coeln	0	0	0	0	0	0	0	0	0	0	0
SAL_RA3473AA	546620	Coeln	0	0	0	0	0	0	0	0	0	0	0
SAL_RA3374AA	421177	Coeln	0	0	0	0	0	0	0	0	0	0	0
SAL_RA3236AA	408525	Coeln	0	0	0	0	0	0	0	0	0	0	0
SAL_RA3636AA	565048	Coeln	0	0	0	0	0	0	0	0	0	0	0
SAL_RA3910AA	310201	Coeln	0	0	0	0	0	0	0	0	0	0	0
SAL_RA9853AA	SMG-18-1135	Coeln	0	0	0	0	0	0	0	0	0	0	0
SAL_RA4385AA	561012	Coeln	0	0	0	0	0	0	0	0	0	0	0
SAL_RA5665AA	399485	Coeln	0	0	0	0	0	0	0	0	0	0	0
SAL_RA2608AA	320232	Coeln	0	0	0	0	0	0	0	0	0	0	0
SAL_RA1804AA	'551-3cab-45c5-aafd-87b	Coeln	0	0	0	0	0	0	0	0	0	0	0
SAL_RA7308AA	413182	Coeln	0	0	0	0	0	0	0	0	0	0	0
SAL_RA7112AA	275796	Coeln	0	0	0	0	0	0	0	0	0	0	0
SAL_RA8098AA	570848	Coeln	0	0	0	0	0	0	0	0	0	0	0
SAL_RA7950AA	304609	Coeln	0	0	0	0	0	0	0	0	0	0	0
SAL_RA8262AA	314994	Coeln	0	0	0	0	0	0	0	0	0	0	0
SAL_RA7323AA	430235	Coeln	0	0	0	0	0	0	0	0	0	0	0
SAL_SA6085AA	572332	Coeln	0	0	0	0	0	0	0	0	0	0	0
SAL_SA7550AA	438618	Coeln	0	0	0	0	0	0	0	0	0	0	0
SAL_SA7385AA	590878	Coeln	0	0	0	0	0	0	0	0	0	0	0
SAL_SA8874AA	607338	Coeln	0	0	0	0	0	0	0	0	0	0	0
SAL_SA9469AA	129427	Coeln	0	0	0	0	0	0	0	0	0	0	0
SAL_SA9407AA	152418	Coeln	0	0	0	0	0	0	0	0	0	0	0
SAL_TA1600AA	SMG-18-2039	Coeln	0	0	0	0	0	0	0	0	0	0	0
SAL_TA2353AA	624383	Coeln	0	0	0	0	0	0	0	0	0	0	0
SAL_TA2319AA	619907	Coeln	0	0	0	0	0	0	0	0	0	0	0
SAL_TA5341AA	624464	Coeln	0	0	0	0	0	0	0	0	0	0	0
SAL_TA5362AA	621470	Coeln	0	0	0	0	0	0	0	0	0	0	0
SAL_TA5968AA	621492	Coeln	0	0	0	0	0	0	0	0	0	0	0
SAL_TA6519AA	619935	Coeln	0	0	0	0	0	0	0	0	0	0	0
SAL_QA5310AA	288793	Coeln	0	0	0	0	0	0	0	0	0	0	0
SAL_PA1867AA	PNUSAS038523	Coeln	0	0	0	0	0	0	0	0	0	0	0
SAL_TA8375AA	638137	Coeln	0	0	0	0	0	0	0	0	0	0	0
SAL_QA9923AA	430245	Coeln	0	0	0	0	0	0	0	0	0	0	0
SAL_QA4674AA	413184	Coeln	0	0	0	0	0	0	0	0	0	0	0
SAL_UA2725AA	647787	Coeln	0	0	0	0	0	0	0	0	0	0	0
SAL_UA5975AA	CZ_18_0620	Coeln	0	0	0	0	0	0	0	0	0	0	0
SAL_UA3035AA	605785	Coeln	0	0	0	0	0	0	0	0	0	0	0
SAL_UA5353AA	636263	Coeln	0	0	0	0	0	0	0	0	0	0	0
SAL_UA6093AA	586784	Coeln	0	0	0	0	0	0	0	0	0	0	0
SAL_UA6446AA	657276	Coeln	0	0	0	0	0	0	0	0	0	0	0
SAL_UA7398AA	568465	Coeln	0	0	0	0	0	0	0	0	0	0	0
SAL_UA8161AA	CZ_18_1015	Coeln	0	0	0	0	0	0	0	0	0	0	0
SAL_UA8180AA	CZ_18_0987	Coeln	0	0	0	0	0	0	0	0	0	0	0
SAL_UA8182AA	CZ_18_1009	Coeln	0	0	0	0	0	0	0	0	0	0	0
SAL_UA8184AA	CZ_18_1057	Coeln	0	0	0	0	0	0	0	0	0	0	0
SAL_UA8224AA	CZ_18_0667	Coeln	0	0	0	0	0	0	0	0	0	0	0
SAL_UA8183AA	CZ_18_1030	Coeln	0	0	0	0	0	0	0	0	0	0	0
SAL_UA8225AA	CZ_18_0897	Coeln	0	0	0	0	0	0	0	0	0	0	0
SAL_UA8229AA	CZ_18_1043	Coeln	0	0	0	0	0	0	0	0	0	0	0
SAL_UA8227AA	CZ_18_0932	Coeln	0	0	0	0	0	0	0	0	0	0	0
SAL_UA8232AA	CZ_18_0923	Coeln	0	0	0	0	0	0	0	0	0	0	0
SAL_UA8230AA	CZ_18_1047	Coeln	0	0	0	0	0	0	0	0	0	0	0
SAL_UA8231AA	CZ_18_1072	Coeln	0	0	0	0	0	0	0	0	0	0	0
SAL_UA8226AA	CZ_18_0914	Coeln	0	0	0	0	0	0	0	0	0	0	0
SAL_UA9618AA	669722	Coeln	0	0	0	0	0	0	0	0	0	0	0
SAL_VA0207AA	674055	Coeln	0	0	0	0	0	0	0	0	0	0	0
SAL_VA7860AA	CZ_16_0409	Coeln	0	0	0	0	0	0	0	0	0	0	0
SAL_VA7940AA	CZ_19_0060	Coeln	0	0	0	0	0	0	0	0	0	0	0
SAL_VA8017AA	CZ_19_0059	Coeln	0	0	0	0	0	0	0	0	0	0	0
SAL_VA2241AA	470341	Coeln	0	0	0	0	0	0	0	0	0	0	0
SAL_VA5760AA	SA20053118	Coeln	0	0	0	0	0	0	0	0	0	0	0

SAL_VA4493AA	390033	Coeln	0	0	0	0	0	0	0	0	0	0	0
SAL_VA7154AA	385495	Coeln	0	0	0	0	0	0	0	0	0	0	0
SAL_VA8238AA	364192	Coeln	0	0	0	0	0	0	0	0	0	0	0
SAL_VA9176AA	302560	Coeln	0	0	0	0	0	0	0	0	0	0	0
SAL_VA9072AA	312145	Coeln	0	0	0	0	0	0	0	0	0	0	0
SAL_VA9419AA	283134	Coeln	0	0	0	0	0	0	0	0	0	0	0
SAL_VA4049AA	CDPH_CSalm299	Coeln	0	0	0	0	0	0	0	0	0	0	0
SAL_WA1293AA	130358	Coeln	0	0	0	0	0	0	0	0	0	0	0
SAL_CA3999AA	55552	Coleypark	0	0	0	0	0	0	0	0	0	0	0
SAL_DA6889AA	FMA0164	Coleypark	0	0	0	0	0	0	0	0	0	0	0
SAL_PA8042AA	387066	Coleypark	0	0	0	0	0	0	0	0	0	0	0
SAL_PA0529AA	CFSAN064143	Coleypark	0	0	0	0	0	0	0	0	0	0	0
SAL_CA2830AA	61901	Colindale	0	0	0	0	0	0	0	0	0	0	0
SAL_DA6135AA	MOD1_Sal_851	Colindale	0	0	0	0	0	0	0	0	0	0	0
SAL_DA1141AA	H120680267	Colindale	0	0	0	0	0	0	0	0	0	0	0
SAL_DA0744AA	H122220307	Colindale	0	0	0	0	0	0	0	0	0	0	0
SAL_DA0690AA	H125180325	Colindale	0	0	0	0	0	0	0	0	0	0	0
SAL_DA0646AA	H122520644	Colindale	0	0	0	0	0	0	0	0	0	0	0
SAL_DA0586AA	H132560437	Colindale	0	0	0	0	0	0	0	0	0	0	0
SAL_CA6092AA	57655	Colindale	0	0	0	0	0	0	0	0	0	0	0
SAL_CA4346AA	61865	Colindale	0	0	0	0	0	0	0	0	0	0	0
SAL_CA2957AA	41945	Colindale	0	0	0	0	0	0	0	0	0	0	0
SAL_CA1664AA	3891	Colindale	0	0	0	0	0	0	0	0	0	0	0
SAL_BA7721AA	26646	Colindale	0	0	0	0	0	0	0	0	0	0	0
SAL_BA6411AA	713837	Colindale	0	0	0	0	0	0	0	0	0	0	0
SAL_GA3890AA	BCW_2908	Colindale	0	0	0	0	0	0	0	0	0	0	0
SAL_GA3345AA	BCW_2661	Colindale	0	0	0	0	0	0	0	0	0	0	0
SAL_GA1313AA	PO2751-sc-1993746	Colindale	0	0	0	0	0	0	0	0	0	0	0
SAL_GA6423AA	PNUSAS002740	Colindale	0	0	0	0	0	0	0	0	0	0	0
SAL_GA9386AA	PNUSAS001698	Colindale	0	0	0	0	0	0	0	0	0	0	0
SAL_HA2685AA	ICOPHAI16770	Colindale	0	0	0	0	0	0	0	0	0	0	0
SAL_JA3274AA	PNUSAS011860	Colindale	0	0	0	0	0	0	0	0	0	0	0
SAL_KA1663AA	colindale_DE_34	Colindale	0	0	0	0	0	0	0	0	0	0	0
SAL_KA1665AA	colindale_HP_21I	Colindale	0	0	0	0	0	0	0	0	0	0	0
SAL_KA1667AA	colindale_HD_34E	Colindale	0	0	0	0	0	0	0	0	0	0	0
SAL_KA1662AA	colindale_DE_138	Colindale	0	0	0	0	0	0	0	0	0	0	0
SAL_KA1666AA	colindale_HD_32D	Colindale	0	0	0	0	0	0	0	0	0	0	0
SAL_KA1668AA	colindale_HD_3A	Colindale	0	0	0	0	0	0	0	0	0	0	0
SAL_LA1920AA	MC_05-0256	Colindale	0	0	0	0	0	0	0	0	0	0	0
SAL_DA1623AA	FNE0197	Colindale	0	0	0	0	0	0	0	0	0	0	0
SAL_GA9207AA	CFSAN031406	Colindale	0	0	0	0	0	0	0	0	0	0	0
SAL_CA0159AA	91976	Colindale	0	0	0	0	0	0	0	0	0	0	0
SAL_PA0520AA	CFSAN064147	Colindale	0	0	0	0	0	0	0	0	0	0	0
SAL_PA0522AA	CFSAN064146	Colindale	0	0	0	0	0	0	0	0	0	0	0
SAL_PA0525AA	CFSAN064148	Colindale	0	0	0	0	0	0	0	0	0	0	0
SAL_PA0524AA	CFSAN064149	Colindale	0	0	0	0	0	0	0	0	0	0	0
SAL_PA0496AA	CFSAN064144	Colindale	0	0	0	0	0	0	0	0	0	0	0
SAL_PA9703AA	149343	Colindale	0	0	0	0	0	0	0	0	0	0	0
SAL_RA0344AA	413061	Colindale	0	0	0	0	0	0	0	0	0	0	0
SAL_RA0739AA	193392	Colindale	0	0	0	0	0	0	0	0	0	0	0
SAL_RA3558AA	169617	Colindale	0	0	0	0	0	0	0	0	0	0	0
SAL_RA2638AA	109871	Colindale	0	0	0	0	0	0	0	0	0	0	0
SAL_RA7465AA	568390	Colindale	0	0	0	0	0	0	0	0	0	0	0
SAL_RA7376AA	565965	Colindale	0	0	0	0	0	0	0	0	0	0	0
SAL_RA8207AA	320990	Colindale	0	0	0	0	0	0	0	0	0	0	0
SAL_SA2985AA	CFSAN070311	Colindale	0	0	0	0	0	0	0	0	0	0	0
SAL_SA2969AA	CFSAN070300	Colindale	0	0	0	0	0	0	0	0	0	0	0
SAL_RA9675AA		Colindale	0	0	0	0	0	0	0	0	0	0	0
SAL_PA0411AA	CFSAN064145	Colindale	0	0	0	0	0	0	0	0	0	0	0
SAL_TA9760AA	643184	Colindale	0	0	0	0	0	0	0	0	0	0	0
SAL_TA9958AA	CFSAN083285	Colindale	92	0	211	425	83	0	0	0	0	0	0
SAL_UA2810AA	643097	Colindale	0	0	0	0	0	0	0	0	0	0	0
SAL_UA3024AA	650057	Colindale	0	0	0	0	0	0	0	0	0	0	0
SAL_VA4187AA	CDPH_CSalm369	Colindale	0	0	0	0	0	0	0	0	0	0	0
SAL_QA2231AA	170337	Coquilhatville	0	0	0	0	0	0	0	0	0	0	0
SAL_QA4066AA	529859	Coquilhatville	0	0	0	0	0	0	0	0	0	0	0
SAL_DA6078AA	FNE0125	Coromandel	0	0	0	0	0	0	0	0	0	0	0
SAL_CA5714AA	75663	Corvallis	0	0	0	0	0	0	0	0	0	0	0
SAL_DA2261AA	2011K-1348	Corvallis	0	0	0	0	0	0	0	0	0	0	0
SAL_FA4628AA	129372	Corvallis	0	0	0	0	0	0	0	0	0	0	0
SAL_FA4607AA	205594	Corvallis	0	0	0	0	0	0	0	0	0	0	0
SAL_FA4599AA	201556	Corvallis	0	0	0	0	0	0	0	0	0	0	0
SAL_FA4609AA	163194	Corvallis	0	0	0	0	0	0	0	0	0	0	0

SAL_FA4576AA	182066	Corvallis	0	0	0	0	0	0	0	0	0	0	0
SAL_FA4536AA	175582	Corvallis	0	0	0	0	0	0	0	0	0	0	0
SAL_FA4300AA	185919	Corvallis	0	0	0	0	0	0	0	0	0	0	0
SAL_FA4287AA	168337	Corvallis	0	0	0	0	0	0	0	0	0	0	0
SAL_FA4254AA	175569	Corvallis	0	0	0	0	0	0	0	0	0	0	0
SAL_FA4230AA	205632	Corvallis	0	0	0	0	0	0	0	0	0	0	0
SAL_FA4141AA	172487	Corvallis	0	0	0	0	0	0	0	0	0	0	0
SAL_FA4118AA	186525	Corvallis	0	0	0	0	0	0	0	0	0	0	0
SAL_FA1742AA	N08-281	Corvallis	0	0	0	0	0	0	0	0	0	0	0
SAL_FA1744AA	N08-280	Corvallis	0	0	0	0	0	0	0	0	0	0	0
SAL_FA1740AA	N08-284	Corvallis	0	0	0	0	0	0	0	0	0	0	0
SAL_FA1629AA	FDA00009959	Corvallis	0	0	0	0	0	0	0	0	0	0	0
SAL_FA1627AA	FDA00009961	Corvallis	0	0	0	0	0	0	0	0	0	0	0
SAL_EA9161AA	53081	Corvallis	0	0	0	0	0	0	0	0	0	0	0
SAL_EA9160AA	53086	Corvallis	0	0	0	0	0	0	0	0	0	0	0
SAL_EA7977AA	29942	Corvallis	0	0	0	0	0	0	0	0	0	0	0
SAL_EA7554AA	29298	Corvallis	0	0	0	0	0	0	0	0	0	0	0
SAL_EA7077AA	18576	Corvallis	0	0	0	0	0	0	0	0	0	0	0
SAL_EA7002AA	18635	Corvallis	0	0	0	0	0	0	0	0	0	0	0
SAL_EA6978AA	103196	Corvallis	0	0	0	0	0	0	0	0	0	0	0
SAL_EA2877AA	FDA00001853	Corvallis	0	0	0	0	0	0	0	0	0	0	0
SAL_DA9768AA	FNE0062	Corvallis	0	0	0	0	0	0	0	0	0	0	0
SAL_DA8224AA	5583-sc-2010-09-29-985	Corvallis	0	0	0	0	0	0	0	0	0	0	0
SAL_DA6258AA	FSE0131	Corvallis	0	0	0	0	0	0	0	0	0	0	0
SAL_DA6130AA	MOD1_Sal_847	Corvallis	0	0	0	0	0	0	0	0	0	0	0
SAL_DA2670AA	FDN0146	Corvallis	0	0	0	0	0	0	0	0	0	0	0
SAL_DA2267AA	07-0704	Corvallis	0	0	0	0	0	0	0	0	0	0	0
SAL_DA2165AA	FDN0145	Corvallis	0	0	0	0	0	0	0	0	0	0	0
SAL_DA1111AA	H124820314	Corvallis	0	0	0	0	0	0	0	0	0	0	0
SAL_DA1074AA	H124160539	Corvallis	0	0	0	0	0	0	0	0	0	0	0
SAL_DA0880AA	H132540712	Corvallis	0	0	0	0	0	0	0	0	0	0	0
SAL_DA0488AA	H123800500	Corvallis	0	0	0	0	0	0	0	0	0	0	0
SAL_CA8955AA	FDA00003836	Corvallis	0	0	0	0	0	0	0	0	0	0	0
SAL_CA7370AA	CFSAN004278	Corvallis	0	0	0	0	0	0	0	0	0	0	0
SAL_CA7231AA	CFSAN004279	Corvallis	0	0	0	0	0	0	0	0	0	0	0
SAL_CA6549AA	66461	Corvallis	0	0	0	0	0	0	0	0	0	0	0
SAL_CA6484AA	54010	Corvallis	0	0	0	0	0	0	0	0	0	0	0
SAL_CA5120AA	78652	Corvallis	0	0	0	0	0	0	0	0	0	0	0
SAL_CA5109AA	70673	Corvallis	0	0	0	0	0	0	0	0	0	0	0
SAL_CA5113AA	43400	Corvallis	0	0	0	0	0	0	0	0	0	0	0
SAL_CA5053AA	75607	Corvallis	0	0	0	0	0	0	0	0	0	0	0
SAL_CA4791AA	38323	Corvallis	0	0	0	0	0	0	0	0	0	0	0
SAL_CA4746AA	83345	Corvallis	0	0	0	0	0	0	0	0	0	0	0
SAL_CA4419AA	41939	Corvallis	0	0	0	0	0	0	0	0	0	0	0
SAL_CA4261AA	14120	Corvallis	0	0	0	0	0	0	0	0	0	0	0
SAL_CA3439AA	55559	Corvallis	0	0	0	0	0	0	0	0	0	0	0
SAL_CA3019AA	12142	Corvallis	0	0	0	0	0	0	0	0	0	0	0
SAL_CA2595AA	34162	Corvallis	0	0	0	0	0	0	0	0	0	0	0
SAL_CA2590AA	13377	Corvallis	0	0	0	0	0	0	0	0	0	0	0
SAL_CA2120AA	57120	Corvallis	0	0	0	0	0	0	0	0	0	0	0
SAL_CA2079AA	99051	Corvallis	0	0	0	0	0	0	0	0	0	0	0
SAL_CA2006AA	45990	Corvallis	0	0	0	0	0	0	0	0	0	0	0
SAL_CA1841AA	40343	Corvallis	0	0	0	0	0	0	0	0	0	0	0
SAL_CA1638AA	37805	Corvallis	0	0	0	0	0	0	0	0	0	0	0
SAL_CA1250AA	38745	Corvallis	0	0	0	0	0	0	0	0	0	0	0
SAL_CA0868AA	75927	Corvallis	0	0	0	0	0	0	0	0	0	0	0
SAL_CA0703AA	40239	Corvallis	0	0	0	0	0	0	0	0	0	0	0
SAL_CA0665AA	69921	Corvallis	0	0	0	0	0	0	0	0	0	0	0
SAL_BA9480AA	75925	Corvallis	0	0	0	0	0	0	0	0	0	0	0
SAL_BA9339AA	6453	Corvallis	0	0	0	0	0	0	0	0	0	0	0
SAL_BA8884AA	98255	Corvallis	0	0	0	0	0	0	0	0	0	0	0
SAL_BA8342AA	39468	Corvallis	0	0	0	0	0	0	0	0	0	0	0
SAL_BA8285AA	6455	Corvallis	0	0	0	0	0	0	0	0	0	0	0
SAL_BA6680AA	09:MF0014001R	Corvallis	0	0	0	0	0	0	0	0	0	0	0
SAL_BA6375AA	09:MF0009245R	Corvallis	0	0	0	0	0	0	0	0	0	0	0
SAL_BA1772AA	FDA00000782	Corvallis	0	0	0	0	0	0	0	0	0	0	0
SAL_BA1540AA	FDA00003387	Corvallis	0	0	0	0	0	0	0	0	0	0	0
SAL_FA6143AA	PNUSAS001962	Corvallis	0	0	0	0	0	0	0	0	0	0	0
SAL_FA6103AA	PNUSAS001850	Corvallis	0	0	0	0	0	0	0	0	0	0	0
SAL_FA6434AA	PNUSAS001699	Corvallis	0	0	0	0	0	0	0	0	0	0	0
SAL_FA7225AA	PNUSAS002202	Corvallis	0	0	0	0	0	0	0	0	0	0	0
SAL_GA3641AA	BCW_2667	Corvallis	0	0	0	0	0	0	0	0	0	0	0
SAL_GA3507AA	BCW_2858	Corvallis	0	0	0	0	0	0	0	0	0	0	0

SAL_GA2510AA	2011K-1068	Corvallis	0	0	0	0	0	0	0	0	0	0	0
SAL_GA1682AA	BCW_2855	Corvallis	0	0	0	0	0	0	0	0	0	0	0
SAL_GA3640AA	BCW_2669	Corvallis	0	0	0	0	0	0	0	0	0	0	0
SAL_GA0279AA	72_V_040-sc-2280741	Corvallis	0	0	0	0	0	0	0	0	0	0	0
SAL_GA0276AA	72_V_183-sc-2280744	Corvallis	0	0	0	0	0	0	0	0	0	0	0
SAL_GA0156AA	VNB334-sc-2280611	Corvallis	0	0	0	0	0	0	0	0	0	0	0
SAL_GA0162AA	VNB78-sc-2280597	Corvallis	0	0	0	0	0	0	0	0	0	0	0
SAL_GA4869AA	FDA00010401	Corvallis	0	0	0	0	0	0	0	0	0	0	0
SAL_GA4734AA	HIY0041	Corvallis	0	0	0	0	0	0	0	0	0	0	0
SAL_GA4737AA	HIY0169	Corvallis	0	0	0	0	0	0	0	0	0	0	0
SAL_GA4736AA	HIY0180	Corvallis	0	0	0	0	0	0	0	0	0	0	0
SAL_FA8420AA	PNUSAS002435	Corvallis	0	0	0	0	0	0	0	0	0	0	0
SAL_HA4096AA	PNUSAS004841	Corvallis	0	0	0	0	0	0	0	0	0	0	0
SAL_HA4597AA	09-1853	Corvallis	0	0	0	0	0	0	0	0	0	0	0
SAL_HA5214AA	2092781	Corvallis	0	0	0	0	0	0	0	0	0	0	0
SAL_HA5201AA	737730	Corvallis	0	0	0	0	0	0	0	0	0	0	0
SAL_HA5164AA	1975903	Corvallis	0	0	0	0	0	0	0	0	0	0	0
SAL_HA5383AA	ARIM-SCOR775-05	Corvallis	0	0	0	0	0	0	0	0	0	0	0
SAL_HA5057AA	1373649	Corvallis	0	0	0	0	0	0	0	0	0	0	0
SAL_HA5377AA	ARIM-SCOR1078-05	Corvallis	0	0	0	0	0	0	0	0	0	0	0
SAL_HA5694AA	PNUSAS005592	Corvallis	0	0	0	0	0	0	0	0	0	0	0
SAL_IA1188AA	285947	Corvallis	0	0	0	0	0	0	0	0	0	0	0
SAL_IA2250AA	MS160043	Corvallis	0	0	0	0	0	0	0	0	0	0	0
SAL_IA7236AA	PNUSAS008747	Corvallis	0	0	0	0	0	0	0	0	0	0	0
SAL_IA7713AA	S/20170032	Corvallis	0	0	0	0	0	0	0	0	0	0	0
SAL_IA7722AA	S/20170061	Corvallis	0	0	0	0	0	0	0	0	0	0	0
SAL_JA1083AA	PNUSAS007057	Corvallis	0	0	0	0	0	0	0	0	0	0	0
SAL_JA2094AA	PNUSAS011231	Corvallis	0	0	0	0	0	0	0	0	0	0	0
SAL_JA5253AA	PNUSAS006234	Corvallis	0	0	0	0	0	0	0	0	0	0	0
SAL_JA6349AA	346901	Corvallis	0	0	0	0	0	0	0	0	0	0	0
SAL_JA6310AA	352076	Corvallis	0	0	0	0	0	0	0	0	0	0	0
SAL_JA6332AA	358428	Corvallis	0	0	0	0	0	0	0	0	0	0	0
SAL_JA6152AA	PNUSAS012129	Corvallis	0	0	0	0	0	0	0	0	0	0	0
SAL_JA5899AA	348034	Corvallis	0	0	0	0	0	0	0	0	0	0	0
SAL_JA7304AA	PNUSAS014067	Corvallis	0	0	0	0	0	0	0	0	0	0	0
SAL_JA8356AA	330560	Corvallis	0	0	0	0	0	0	0	0	0	0	0
SAL_JA8328AA	341405	Corvallis	0	0	0	0	0	0	0	0	0	0	0
SAL_JA8331AA	333396	Corvallis	0	0	0	0	0	0	0	0	0	0	0
SAL_JA8243AA	335285	Corvallis	0	0	0	0	0	0	0	0	0	0	0
SAL_JA8184AA	356079	Corvallis	0	0	0	0	0	0	0	0	0	0	0
SAL_JA8045AA	341406	Corvallis	0	0	0	0	0	0	0	0	0	0	0
SAL_JA8028AA	335078	Corvallis	0	0	0	0	0	0	0	0	0	0	0
SAL_JA8459AA	11-10-20170287	Corvallis	0	0	0	0	0	0	0	0	0	0	0
SAL_JA8073AA	356624	Corvallis	0	0	0	0	0	0	0	0	0	0	0
SAL_JA7795AA	355045	Corvallis	0	0	0	0	0	0	0	0	0	0	0
SAL_JA8617AA	PNUSAS013654	Corvallis	0	0	0	0	0	0	0	0	0	0	0
SAL_KA0831AA	PNUSAS016180	Corvallis	0	0	0	0	0	0	0	0	0	0	0
SAL_KA2080AA	12-21-20170455	Corvallis	0	0	0	0	0	0	0	0	0	0	0
SAL_KA3731AA	15-22-20170287	Corvallis	0	0	0	0	0	0	0	0	0	0	0
SAL_KA3760AA	PNUSAS017059	Corvallis	0	0	0	0	0	0	0	0	0	0	0
SAL_KA6994AA	PNUSAS016778	Corvallis	0	0	0	0	0	0	0	0	0	0	0
SAL_LA0112AA	19-3-20170585	Corvallis	0	0	0	0	0	0	0	0	0	0	0
SAL_LA3933AA	PNUSAS022579	Corvallis	0	0	0	0	0	0	0	0	0	0	0
SAL_LA7509AA	PNUSAS024273	Corvallis	0	0	0	0	0	0	0	0	0	0	0
SAL_CA3268AA	96927	Corvallis	0	0	0	0	0	0	0	0	0	0	0
SAL_CA4711AA	87413	Corvallis	0	0	0	0	0	0	0	0	0	0	0
SAL_CA6125AA	53984	Corvallis	0	0	0	0	0	0	0	0	0	0	0
SAL_BA0749AA	ADRDL-NGUA-38	Corvallis	0	0	0	0	0	0	0	0	0	0	0
SAL_EA7761AA	81990	Corvallis	0	0	0	0	0	0	0	0	0	0	0
SAL_FA4332AA	191669	Corvallis	0	0	0	0	0	0	0	0	0	0	0
SAL_JA6026AA	PNUSAS011882	Corvallis	0	0	0	0	0	0	0	0	0	0	0
SAL_JA7468AA	CFSAN059903	Corvallis	0	0	0	0	0	0	0	0	0	0	0
SAL_BA9900AA	45972	Corvallis	0	0	0	0	0	0	0	0	0	0	0
SAL_MA6743AA	345401	Corvallis	0	0	0	0	0	0	0	0	0	0	0
SAL_MA8092AA	103662	Corvallis	0	0	0	0	0	0	0	0	0	0	0
SAL_MA8467AA	ERS2017967	Corvallis	0	0	0	0	0	0	0	0	0	0	0
SAL_MA8471AA	ERS2017963	Corvallis	0	0	0	0	0	0	0	0	0	0	0
SAL_MA8367AA	PNUSAS028202	Corvallis	0	0	0	0	0	0	0	0	0	0	0
SAL_NA4985AA	01-10-18.8100007	Corvallis	0	0	0	0	0	0	0	0	0	0	0
SAL_NA7184AA	PNUSAS032472	Corvallis	0	0	0	0	0	0	0	0	0	0	0
SAL_NA9725AA	6-10-8100101	Corvallis	0	0	0	0	0	0	0	0	0	0	0
SAL_OA5936AA	100479	Corvallis	0	0	0	0	0	0	0	0	0	0	0
SAL_OA5940AA	100730	Corvallis	0	0	0	0	0	0	0	0	0	0	0

SAL_OA7230AA	PNUSAS036081	Corvallis	0	0	0	0	0	0	0	0	0	0	0
SAL_OA6646AA	CFSAN078454	Corvallis	0	0	0	0	0	0	0	0	0	0	0
SAL_PA0072AA	18EP000443	Corvallis	0	0	0	0	0	0	0	0	0	0	0
SAL_PA0519AA	CFSAN064150	Corvallis	0	0	0	0	0	0	0	0	0	0	0
SAL_PA0521AA	CFSAN064151	Corvallis	0	0	0	0	0	0	0	0	0	0	0
SAL_PA4316AA	123986	Corvallis	0	0	0	0	0	0	0	0	0	0	0
SAL_PA5181AA	18EP000620	Corvallis	0	0	0	0	0	0	0	0	0	0	0
SAL_PA4975AA	412019	Corvallis	0	0	0	0	0	0	0	0	0	0	0
SAL_PA4963AA	327992	Corvallis	0	0	0	0	0	0	0	0	0	0	0
SAL_PA5739AA	PNUSAS040226	Corvallis	0	0	0	0	0	0	0	0	0	0	0
SAL_PA5644AA	231106	Corvallis	0	0	0	0	0	0	0	0	0	0	0
SAL_PA5628AA	221702	Corvallis	0	0	0	0	0	0	0	0	0	0	0
SAL_PA6236AA	266929	Corvallis	0	0	0	0	0	0	0	0	0	0	0
SAL_PA6159AA	509836	Corvallis	0	0	0	0	0	0	0	0	0	0	0
SAL_PA7276AA	402756	Corvallis	0	0	0	0	0	0	0	0	0	0	0
SAL_PA7535AA	393998	Corvallis	0	0	0	0	0	0	0	0	0	0	0
SAL_PA7507AA	173539	Corvallis	0	0	0	0	0	0	0	0	0	0	0
SAL_PA7874AA	146077	Corvallis	0	0	0	0	0	0	0	0	0	0	0
SAL_PA8369AA	157321	Corvallis	0	0	0	0	0	0	0	0	0	0	0
SAL_PA8379AA	516905	Corvallis	0	0	0	0	0	0	0	0	0	0	0
SAL_PA7846AA	323884	Corvallis	0	0	0	0	0	0	0	0	0	0	0
SAL_PA7829AA	127534	Corvallis	0	0	0	0	0	0	0	0	0	0	0
SAL_PA7868AA	531223	Corvallis	0	0	0	0	0	0	0	0	0	0	0
SAL_PA8531AA	280331	Corvallis	0	0	0	0	0	0	0	0	0	0	0
SAL_PA9139AA	PNUSAS041362	Corvallis	0	0	0	0	0	0	0	0	0	0	0
SAL_PA8860AA	227042	Corvallis	0	0	0	0	0	0	0	0	0	0	0
SAL_QA0280AA	323875	Corvallis	0	0	0	0	0	0	0	0	0	0	0
SAL_QA0519AA	313232	Corvallis	0	0	0	0	0	0	0	0	0	0	0
SAL_QA0413AA	158963	Corvallis	0	0	0	0	0	0	0	0	0	0	0
SAL_QA1849AA	243833	Corvallis	0	0	0	0	0	0	0	0	0	0	0
SAL_QA1724AA	316192	Corvallis	0	0	0	0	0	0	0	0	0	0	0
SAL_QA2519AA	373905	Corvallis	0	0	0	0	0	0	0	0	0	0	0
SAL_QA2087AA	171159	Corvallis	0	0	0	0	0	0	0	0	0	0	0
SAL_QA2025AA	114064	Corvallis	0	0	0	0	0	0	0	0	0	0	0
SAL_QA1998AA	216222	Corvallis	0	0	0	0	0	0	0	0	0	0	0
SAL_QA2782AA	399348	Corvallis	0	0	0	0	0	0	0	0	0	0	0
SAL_QA2732AA	311417	Corvallis	0	0	0	0	0	0	0	0	0	0	0
SAL_QA1837AA	229092	Corvallis	0	0	0	0	0	0	0	0	0	0	0
SAL_QA3832AA	351318	Corvallis	0	0	0	0	0	0	0	0	0	0	0
SAL_QA4946AA	184415	Corvallis	0	0	0	0	0	0	0	0	0	0	0
SAL_QA4922AA	453242	Corvallis	0	0	0	0	0	0	0	0	0	0	0
SAL_QA4808AA	304623	Corvallis	0	0	0	0	0	0	0	0	0	0	0
SAL_QA5975AA	140189	Corvallis	0	0	0	0	0	0	0	0	0	0	0
SAL_QA5834AA	194257	Corvallis	0	0	0	0	0	0	0	0	0	0	0
SAL_QA6151AA	449458	Corvallis	0	0	0	0	0	0	0	0	0	0	0
SAL_QA6602AA	217077	Corvallis	0	0	0	0	0	0	0	0	0	0	0
SAL_QA6527AA	318320	Corvallis	0	0	0	0	0	0	0	0	0	0	0
SAL_QA6544AA	216347	Corvallis	0	0	0	0	0	0	0	0	0	0	0
SAL_QA6434AA	134263	Corvallis	0	0	0	0	0	0	0	0	0	0	0
SAL_QA6697AA	421624	Corvallis	0	0	0	0	0	0	0	0	0	0	0
SAL_QA7268AA	249345	Corvallis	0	0	0	0	0	0	0	0	0	0	0
SAL_QA7700AA	420517	Corvallis	0	0	0	0	0	0	0	0	0	0	0
SAL_QA7703AA	282484	Corvallis	0	0	0	0	0	0	0	0	0	0	0
SAL_QA7572AA	411987	Corvallis	0	0	0	0	0	0	0	0	0	0	0
SAL_QA8391AA	179632	Corvallis	0	0	0	0	0	0	0	0	0	0	0
SAL_QA8274AA	524779	Corvallis	0	0	0	0	0	0	0	0	0	0	0
SAL_QA8275AA	193396	Corvallis	0	0	0	0	0	0	0	0	0	0	0
SAL_QA8771AA	373182	Corvallis	0	0	0	0	0	0	0	0	0	0	0
SAL_QA9973AA	286737	Corvallis	0	0	0	0	0	0	0	0	0	0	0
SAL_QA9830AA	178936	Corvallis	0	0	0	0	0	0	0	0	0	0	0
SAL_QA9839AA	549484	Corvallis	0	0	0	0	0	0	0	0	0	0	0
SAL_RA0335AA	439999	Corvallis	0	0	0	0	0	0	0	0	0	0	0
SAL_RA0352AA	116588	Corvallis	0	0	0	0	0	0	0	0	0	0	0
SAL_RA0355AA	315675	Corvallis	0	0	0	0	0	0	0	0	0	0	0
SAL_RA0980AA	163179	Corvallis	0	0	0	0	0	0	0	0	0	0	0
SAL_RA0846AA	296155	Corvallis	0	0	0	0	0	0	0	0	0	0	0
SAL_RA0855AA	170464	Corvallis	0	0	0	0	0	0	0	0	0	0	0
SAL_RA0881AA	278662	Corvallis	0	0	0	0	0	0	0	0	0	0	0
SAL_RA0667AA	209695	Corvallis	0	0	0	0	0	0	0	0	0	0	0
SAL_RA0589AA	254640	Corvallis	0	0	0	0	0	0	0	0	0	0	0
SAL_RA0540AA	454880	Corvallis	0	0	0	0	0	0	0	0	0	0	0
SAL_RA1317AA	212792	Corvallis	0	0	0	0	0	0	0	0	0	0	0
SAL_RA2627AA	114036	Corvallis	0	0	0	0	0	0	0	0	0	0	0

SAL_RA2837AA	202232	Corvallis	0	0	0	0	0	0	0	0	0	0	0
SAL_RA3437AA	218470	Corvallis	0	0	0	0	0	0	0	0	0	0	0
SAL_RA3387AA	516926	Corvallis	0	0	0	0	0	0	0	0	0	0	0
SAL_RA3324AA	248559	Corvallis	0	0	0	0	0	0	0	0	0	0	0
SAL_RA8799AA	18EP001287	Corvallis	0	0	0	0	0	0	0	0	0	0	0
SAL_RA8800AA	18EP001286	Corvallis	0	0	0	0	0	0	0	0	0	0	0
SAL_RA3720AA	131468	Corvallis	0	0	0	0	0	0	0	0	0	0	0
SAL_QA9359AA	557659	Corvallis	0	0	0	0	0	0	0	0	0	0	0
SAL_RA3932AA	361775	Corvallis	0	0	0	0	0	0	0	0	0	0	0
SAL_RA0007AA	416342	Corvallis	0	0	0	0	0	0	0	0	0	0	0
SAL_SA2483AA	SMG-18-1280	Corvallis	0	0	0	0	0	0	0	0	0	0	0
SAL_SA2485AA	SMG-18-1278	Corvallis	0	0	0	0	0	0	0	0	0	0	0
SAL_RA7508AA	568431	Corvallis	0	0	0	0	0	0	0	0	0	0	0
SAL_RA7407AA	203999	Corvallis	0	0	0	0	0	0	0	0	0	0	0
SAL_RA7384AA	298763	Corvallis	0	0	0	0	0	0	0	0	0	0	0
SAL_RA7223AA	408451	Corvallis	0	0	0	0	0	0	0	0	0	0	0
SAL_RA7688AA	442675	Corvallis	0	0	0	0	0	0	0	0	0	0	0
SAL_RA8310AA	570827	Corvallis	0	0	0	0	0	0	0	0	0	0	0
SAL_SA3870AA	SMG-18-1646	Corvallis	0	0	0	0	0	0	0	0	0	0	0
SAL_SA2792AA	a98b-ce4f-4c9f-92e4-1fc	Corvallis	0	0	0	0	0	0	0	0	0	0	0
SAL_SA4820AA	596247	Corvallis	0	0	0	0	0	0	0	0	0	0	0
SAL_SA4720AA	576334	Corvallis	0	0	0	0	0	0	0	0	0	0	0
SAL_SA5990AA	591818	Corvallis	0	0	0	0	0	0	0	0	0	0	0
SAL_SA5866AA	599567	Corvallis	0	0	0	0	0	0	0	0	0	0	0
SAL_SA6216AA	569975	Corvallis	0	0	0	0	0	0	0	0	0	0	0
SAL_SA6221AA	582263	Corvallis	0	0	0	0	0	0	0	0	0	0	0
SAL_SA6120AA	582261	Corvallis	0	0	0	0	0	0	0	0	0	0	0
SAL_SA7528AA	573596	Corvallis	0	0	0	0	0	0	0	0	0	0	0
SAL_SA7676AA	588496	Corvallis	0	0	0	0	0	0	0	0	0	0	0
SAL_SA8027AA	166274	Corvallis	0	0	0	0	0	0	0	0	0	0	0
SAL_SA8012AA	313418	Corvallis	0	0	0	0	0	0	0	0	0	0	0
SAL_SA8674AA	123935	Corvallis	0	0	0	0	0	0	0	0	0	0	0
SAL_SA8907AA	305254	Corvallis	0	0	0	0	0	0	0	0	0	0	0
SAL_SA8752AA	569999	Corvallis	0	0	0	0	0	0	0	0	0	0	0
SAL_TA0822AA	168336	Corvallis	0	0	0	0	0	0	0	0	0	0	0
SAL_TA0759AA	617847	Corvallis	0	0	0	0	0	0	0	0	0	0	0
SAL_TA2056AA	CFSAN043224	Corvallis	0	0	0	0	0	0	0	0	0	0	0
SAL_TA2029AA	CFSAN043220	Corvallis	0	0	0	0	0	0	0	0	0	0	0
SAL_TA2385AA	624380	Corvallis	0	0	0	0	0	0	0	0	0	0	0
SAL_TA2262AA	623511	Corvallis	0	0	0	0	0	0	0	0	0	0	0
SAL_TA2754AA	619021	Corvallis	0	0	0	0	0	0	0	0	0	0	0
SAL_TA2722AA	620403	Corvallis	0	0	0	0	0	0	0	0	0	0	0
SAL_TA2941AA	623238	Corvallis	0	0	0	0	0	0	0	0	0	0	0
SAL_TA5333AA	627384	Corvallis	0	0	0	0	0	0	0	0	0	0	0
SAL_TA6009AA	619023	Corvallis	0	0	0	0	0	0	0	0	0	0	0
SAL_TA5955AA	625730	Corvallis	0	0	0	0	0	0	0	0	0	0	0
SAL_TA6550AA	628840	Corvallis	0	0	0	0	0	0	0	0	0	0	0
SAL_TA6497AA	620425	Corvallis	0	0	0	0	0	0	0	0	0	0	0
SAL_TA7726AA	R17.2745	Corvallis	0	0	0	0	0	0	0	0	0	0	0
SAL_TA7686AA	R17.3352	Corvallis	0	0	0	0	0	0	0	0	0	0	0
SAL_TA7640AA	R16.4577	Corvallis	0	0	0	0	0	0	0	0	0	0	0
SAL_QA9955AA	430234	Corvallis	0	0	0	0	0	0	0	0	0	0	0
SAL_TA8905AA	18EP002311	Corvallis	0	0	0	0	0	0	0	0	0	0	0
SAL_QA9908AA	157217	Corvallis	0	0	0	0	0	0	0	0	0	0	0
SAL_OA5362AA	PNUSAS030666	Corvallis	0	0	0	0	0	0	0	0	0	0	0
SAL_OA5614AA	PNUSAS036845	Corvallis	0	0	0	0	0	0	0	0	0	0	0
SAL_PA1355AA	PNUSAS038228	Corvallis	0	0	0	0	0	0	0	0	0	0	0
SAL_PA2666AA	PNUSAS038313	Corvallis	0	0	0	0	0	0	0	0	0	0	0
SAL_UA1617AA	12-01738	Corvallis	0	0	0	0	0	0	0	0	0	0	0
SAL_UA6312AA	SMG-18-2467	Corvallis	0	0	0	0	0	0	0	0	0	0	0
SAL_UA7215AA	573538	Corvallis	0	0	0	0	0	0	0	0	0	0	0
SAL_UA2986AA	650020	Corvallis	0	0	0	0	0	0	0	0	0	0	0
SAL_UA5327AA	653649	Corvallis	0	0	0	0	0	0	0	0	0	0	0
SAL_UA6519AA	574293	Corvallis	0	0	0	0	0	0	0	0	0	0	0
SAL_UA6938AA	659374	Corvallis	0	0	0	0	0	0	0	0	0	0	0
SAL_UA7835AA	661017	Corvallis	0	0	0	0	0	0	0	0	0	0	0
SAL_UA7395AA	568467	Corvallis	0	0	0	0	0	0	0	0	0	0	0
SAL_UA9574AA	579913	Corvallis	0	0	0	0	0	0	0	0	0	0	0
SAL_UA9534AA	648983	Corvallis	0	0	0	0	0	0	0	0	0	0	0
SAL_VA0077AA	654004	Corvallis	0	0	0	0	0	0	0	0	0	0	0
SAL_VA1153AA	526152	Corvallis	0	0	0	0	0	0	0	0	0	0	0
SAL_VA1183AA	508289	Corvallis	0	0	0	0	0	0	0	0	0	0	0
SAL_VA1067AA	505459	Corvallis	0	0	0	0	0	0	0	0	0	0	0

SAL_VA0984AA	503432	Corvallis	0	0	0	0	0	0	0	0	0	0	0
SAL_VA0938AA	507284	Corvallis	0	0	0	0	0	0	0	0	0	0	0
SAL_VA1390AA	493404	Corvallis	0	0	0	0	0	0	0	0	0	0	0
SAL_VA7438AA	201702681	Corvallis	0	0	0	0	0	0	0	0	0	0	0
SAL_VA1575AA	SSSE-17	Corvallis	0	0	0	0	0	0	0	0	0	0	0
SAL_VA7541AA	201705889	Corvallis	0	0	0	0	0	0	0	0	0	0	0
SAL_VA2051AA	453257	Corvallis	0	0	0	0	0	0	0	0	0	0	0
SAL_VA3248AA	414756	Corvallis	0	0	0	0	0	0	0	0	0	0	0
SAL_VA3511AA	395279	Corvallis	0	0	0	0	0	0	0	0	0	0	0
SAL_VA1990AA	436928	Corvallis	0	0	0	0	0	0	0	0	0	0	0
SAL_VA2480AA	424700	Corvallis	0	0	0	0	0	0	0	0	0	0	0
SAL_VA2691AA	430231	Corvallis	0	0	0	0	0	0	0	0	0	0	0
SAL_VA5882AA	SA20102400	Corvallis	0	0	0	0	0	0	0	0	0	0	0
SAL_VA5852AA	SA20094636	Corvallis	0	0	0	0	0	0	0	0	0	0	0
SAL_VA5715AA	SA20094619	Corvallis	0	0	0	0	0	0	0	0	0	0	0
SAL_VA6907AA	684728	Corvallis	0	0	0	0	0	0	0	0	0	0	0
SAL_VA8309AA	686624	Corvallis	0	0	0	0	0	0	0	0	0	0	0
SAL_VA8217AA	687632	Corvallis	0	0	0	0	0	0	0	0	0	0	0
SAL_VA8576AA	369965	Corvallis	0	0	0	0	0	0	0	0	0	0	0
SAL_VA8461AA	685853	Corvallis	0	0	0	0	0	0	0	0	0	0	0
SAL_VA8807AA	318304	Corvallis	0	0	0	0	0	0	0	0	0	0	0
SAL_VA8708AA	317690	Corvallis	0	0	0	0	0	0	0	0	0	0	0
SAL_VA8665AA	323028	Corvallis	0	0	0	0	0	0	0	0	0	0	0
SAL_VA9219AA	306708	Corvallis	0	0	0	0	0	0	0	0	0	0	0
SAL_VA9147AA	307467	Corvallis	0	0	0	0	0	0	0	0	0	0	0
SAL_VA4200AA	CDPH_CSalm023	Corvallis	0	0	0	0	0	0	0	0	0	0	0
SAL_VA4223AA	CDPH_CSalm352	Corvallis	0	0	0	0	0	0	0	0	0	0	0
SAL_VA9880AA	238667	Corvallis	0	0	0	0	0	0	0	0	0	0	0
SAL_WA0173AA	209981	Corvallis	0	0	0	0	0	0	0	0	0	0	0
SAL_WA0434AA	175572	Corvallis	0	0	0	0	0	0	0	0	0	0	0
SAL_WA0299AA	193328	Corvallis	0	0	0	0	0	0	0	0	0	0	0
SAL_WA2683AA	H103540748	Corvallis	0	0	0	0	0	0	0	0	0	0	0
SAL_WA2650AA	GW_S01753-07	Corvallis	0	0	0	0	0	0	0	0	0	0	0
SAL_WA2616AA	H102900584	Corvallis	0	0	0	0	0	0	0	0	0	0	0
SAL_WA1427AA	126108	Corvallis	0	0	0	0	0	0	0	0	0	0	0
SAL_WA1425AA	122706	Corvallis	0	0	0	0	0	0	0	0	0	0	0
SAL_DA2273AA	06-0611	Cotham	0	0	0	0	0	0	0	0	0	0	0
SAL_DA3274AA	2014K-0192	Cotham	0	0	0	0	0	0	0	0	0	0	0
SAL_DA2247AA	2013K-0303	Cotham	0	0	0	0	0	0	0	0	0	0	0
SAL_DA2144AA	08-0258	Cotham	0	0	0	0	0	0	0	0	0	0	0
SAL_DA1218AA	H121020654	Cotham	0	0	0	0	0	0	0	0	0	0	0
SAL_CA3183AA	32514	Cotham	0	0	0	0	0	0	0	0	0	0	0
SAL_CA2414AA	98237	Cotham	0	0	0	0	0	0	0	0	0	0	0
SAL_CA2382AA	13364	Cotham	0	0	0	0	0	0	0	0	0	0	0
SAL_BA6444AA	1599642	Cotham	0	0	0	0	0	0	0	0	0	0	0
SAL_FA6149AA	PNUSAS001955	Cotham	0	0	0	0	0	0	0	0	0	0	0
SAL_FA6441AA	PNUSAS001705	Cotham	0	0	0	0	0	0	0	0	0	0	0
SAL_FA6435AA	PNUSAS001706	Cotham	0	0	0	0	0	0	0	0	0	0	0
SAL_FA6432AA	PNUSAS001707	Cotham	0	0	0	0	0	0	0	0	0	0	0
SAL_FA7345AA	PNUSAS002153	Cotham	0	0	0	0	0	0	0	0	0	0	0
SAL_FA8058AA	PNUSAS002440	Cotham	0	0	0	0	0	0	0	0	0	0	0
SAL_FA8056AA	PNUSAS002442	Cotham	0	0	0	0	0	0	0	0	0	0	0
SAL_EA3473AA	BS_05577-sc-2322340	Cotham	0	0	0	0	0	0	0	0	0	0	0
SAL_FA7223AA	PNUSAS002204	Cotham	0	0	0	0	0	0	0	0	0	0	0
SAL_GA6631AA	PNUSAS002862	Cotham	0	0	0	0	0	0	0	0	0	0	0
SAL_GA6561AA	PNUSAS003186	Cotham	0	0	0	0	0	0	0	0	0	0	0
SAL_GA9865AA	PNUSAS003943	Cotham	0	0	0	0	0	0	0	0	0	0	0
SAL_HA0066AA	PNUSAS004197	Cotham	0	0	0	0	0	0	0	0	0	0	0
SAL_HA5755AA	PNUSAS005401	Cotham	0	0	0	0	0	0	0	0	0	0	0
SAL_HA8583AA	PNUSAS006034	Cotham	0	0	0	0	0	0	0	0	0	0	0
SAL_IA1740AA	319711	Cotham	0	0	0	0	0	0	0	0	0	0	0
SAL_IA6245AA	PNUSAS008170	Cotham	0	0	0	0	0	0	0	0	0	0	0
SAL_IA8826AA	PNUSAS008097	Cotham	0	0	0	0	0	0	0	0	0	0	0
SAL_IA9435AA	PNUSAS006928	Cotham	0	0	0	0	0	0	0	0	0	0	0
SAL_JA0067AA	S/20170121	Cotham	0	0	0	0	0	0	0	0	0	0	0
SAL_JA1746AA	PNUSAS011628	Cotham	0	0	0	0	0	0	0	0	0	0	0
SAL_JA1923AA	PNUSAS009943	Cotham	0	0	0	0	0	0	0	0	0	0	0
SAL_JA3242AA	PNUSAS010503	Cotham	0	0	0	0	0	0	0	0	0	0	0
SAL_JA7414AA	PNUSAS011921	Cotham	0	0	0	0	0	0	0	0	0	0	0
SAL_JA8625AA	PNUSAS013400	Cotham	0	0	0	0	0	0	0	0	0	0	0
SAL_KA0819AA	PNUSAS016716	Cotham	0	0	0	0	0	0	0	0	0	0	0
SAL_KA1066AA	PNUSAS015190	Cotham	0	0	0	0	0	0	0	0	0	0	0
SAL_KA1736AA	PNUSAS015866	Cotham	0	0	0	0	0	0	0	0	0	0	0

SAL_KA4804AA	2015AM-0391	Cotham	0	0	0	0	0	0	0	0	0	0	0
SAL_KA8685AA	18 GREG	Cotham	0	0	0	0	0	0	0	0	0	0	0
SAL_KA5657AA	2015AM-1184	Cotham	0	0	0	0	0	0	0	0	0	0	0
SAL_LA0203AA	2015AM-0684	Cotham	0	0	0	0	0	0	0	0	0	0	0
SAL_LA3940AA	PNUSAS022568	Cotham	0	0	0	0	0	0	0	0	0	0	0
SAL_LA3986AA	PNUSAS022071	Cotham	0	0	0	0	0	0	0	0	0	0	0
SAL_LA5005AA	PNUSAS023001	Cotham	0	0	0	0	0	0	0	0	0	0	0
SAL_LA5605AA	20-29-20170805	Cotham	0	0	0	0	0	0	0	0	0	0	0
SAL_LA7430AA	PNUSAS022705	Cotham	0	0	0	0	0	0	0	0	0	0	0
SAL_LA9886AA	2015AM-0723	Cotham	0	0	0	0	0	0	0	0	0	0	0
SAL_MA4924AA	PNUSAS026733	Cotham	0	0	0	0	0	0	0	0	0	0	0
SAL_CA0584AA	14106	Cotham	0	0	0	0	0	0	0	0	0	0	0
SAL_DA0624AA	H120820428	Cotham	0	0	0	0	0	0	0	0	0	0	0
SAL_HA8378AA	PNUSAS006055	Cotham	0	0	0	0	0	0	0	0	0	0	0
SAL_HA9202AA	PNUSAS002752	Cotham	0	0	0	0	0	0	0	0	0	0	0
SAL_KA0582AA	2015AM-0411	Cotham	0	0	0	0	0	0	0	0	0	0	0
SAL_MA8094AA	104259	Cotham	0	0	0	0	0	0	0	0	0	0	0
SAL_NA0721AA	PNUSAS029555	Cotham	0	0	0	0	0	0	0	0	0	0	0
SAL_NA3855AA	2015AM-3586	Cotham	0	0	0	0	0	0	0	0	0	0	0
SAL_NA5101AA	PNUSAS031627	Cotham	0	0	0	0	0	0	0	0	0	0	0
SAL_NA6871AA	PNUSAS032398	Cotham	0	0	0	0	0	0	0	0	0	0	0
SAL_NA7357AA	2015AM-3414	Cotham	0	0	0	0	0	0	0	0	0	0	0
SAL_NA8103AA	2015AM-3609	Cotham	0	0	0	0	0	0	0	0	0	0	0
SAL_NA8830AA	PNUSAS033154	Cotham	0	0	0	0	0	0	0	0	0	0	0
SAL_OA0122AA	PNUSAS028161	Cotham	0	0	0	0	0	0	0	0	0	0	0
SAL_OA0651AA	PNUSAS034284	Cotham	0	0	0	0	0	0	0	0	0	0	0
SAL_OA5743AA	PNUSAS034957	Cotham	0	0	0	0	0	0	0	0	0	0	0
SAL_OA5733AA	PNUSAS032704	Cotham	0	0	0	0	0	0	0	0	0	0	0
SAL_OA7300AA	PNUSAS010055	Cotham	0	0	0	0	0	0	0	0	0	0	0
SAL_OA4251AA	PNUSAS027855	Cotham	0	0	0	0	0	0	0	0	0	0	0
SAL_PA3440AA	PNUSAS038357	Cotham	0	0	0	0	0	0	0	0	0	0	0
SAL_PA4491AA	PNUSAS034441	Cotham	0	0	0	0	0	0	0	0	0	0	0
SAL_PA7799AA	117302	Cotham	0	0	0	0	0	0	0	0	0	0	0
SAL_PA8552AA	378599	Cotham	0	0	0	0	0	0	0	0	0	0	0
SAL_QA1103AA	PNUSAS042228	Cotham	0	0	0	0	0	0	0	0	0	0	0
SAL_QA1031AA	PNUSAS041877	Cotham	0	0	0	0	0	0	0	0	0	0	0
SAL_QA3251AA	387080	Cotham	0	0	0	0	0	0	0	0	0	0	0
SAL_QA3529AA	PNUSAS039056	Cotham	0	0	0	0	0	0	0	0	0	0	0
SAL_QA5685AA	PNUSAS042901	Cotham	0	0	0	0	0	0	0	0	0	0	0
SAL_QA7580AA	437945	Cotham	0	0	0	0	0	0	0	0	0	0	0
SAL_QA9462AA	113486	Cotham	0	0	0	0	0	0	0	0	0	0	0
SAL_RA6066AA	PNUSAS045617	Cotham	0	0	0	0	0	0	0	0	0	0	0
SAL_RA5957AA	PNUSAS044866	Cotham	0	0	0	0	0	0	0	0	0	0	0
SAL_SA5028AA	S07-0815	Cotham	0	0	0	0	0	0	0	0	0	0	0
SAL_SA5981AA	585940	Cotham	0	0	0	0	0	0	0	0	0	0	0
SAL_SA5961AA	582277	Cotham	0	0	0	0	0	0	0	0	0	0	0
SAL_SA7591AA	582816	Cotham	0	0	0	0	0	0	0	0	0	0	0
SAL_KA2337AA	PNUSAS017385	Cotham	0	0	0	0	0	0	0	0	0	0	0
SAL_KA5410AA	PNUSAS017079	Cotham	0	0	0	0	0	0	0	0	0	0	0
SAL_QA7763AA	160853	Cotham	0	0	0	0	0	0	0	0	0	0	0
SAL_QA3631AA	PNUSAS043249	Cotham	0	0	0	0	0	0	0	0	0	0	0
SAL_OA1681AA	PNUSAS034580	Cotham	0	0	0	0	0	0	0	0	0	0	0
SAL_UA6850AA	AG18-0117	Cotham	0	0	0	0	0	0	0	0	0	0	0
SAL_UA4828AA	647900	Cotham	0	0	0	0	0	0	0	0	0	0	0
SAL_VA0741AA	541358	Cotham	0	0	0	0	0	0	0	0	0	0	0
SAL_VA9241AA	302629	Cotham	0	0	0	0	0	0	0	0	0	0	0
SAL_VA4213AA	CDPH_CSalm126	Cotham	0	0	0	0	0	0	0	0	0	0	0
SAL_WA0026AA	222566	Cotham	0	0	0	0	0	0	0	0	0	0	0
SAL_VA9971AA	242407	Cotham	0	0	0	0	0	0	0	0	0	0	0
SAL_WA0997AA	142202	Cotham	0	0	0	0	0	0	0	0	0	0	0
SAL_WA1518AA	142499	Cotham	0	0	0	0	0	0	0	0	0	0	0
SAL_WA3445AA	708201	Cotham	0	0	0	0	0	0	0	0	0	0	0
SAL_BA1941AA	FDA00004409	Cubana	0	0	0	0	0	0	0	0	0	0	0
SAL_CA8455AA	FDA00002601	Cubana	0	0	0	0	0	0	0	0	0	0	0
SAL_DA9632AA	FAR0059	Cubana	0	0	0	0	0	0	0	0	0	0	0
SAL_FA5692AA	FDA00002590	Cubana	0	0	0	0	0	0	0	0	0	0	0
SAL_FA4987AA	FDA00001505	Cubana	0	0	0	0	0	0	0	0	0	0	0
SAL_FA4654AA	200633	Cubana	0	0	0	0	0	0	0	0	0	0	0
SAL_FA4005AA	FDA00010043	Cubana	0	0	0	0	0	0	0	0	0	0	0
SAL_FA2240AA	FDA00009972	Cubana	0	0	0	0	0	0	0	0	0	0	0
SAL_FA1245AA	FDA00002804	Cubana	0	0	0	0	0	0	0	0	0	0	0
SAL_FA0797AA	FDA00009907	Cubana	0	0	0	0	0	0	0	0	0	0	0
SAL_FA0796AA	FDA00009908	Cubana	0	0	0	0	0	0	0	0	0	0	0

SAL_FA0799AA	FDA00009905	Cubana	0	0	0	0	0	0	0	0	0	0	0
SAL_FA0798AA	FDA00009906	Cubana	0	0	0	0	0	0	0	0	0	0	0
SAL_EA6521AA	FDA00001673	Cubana	0	0	0	0	0	0	0	0	0	0	0
SAL_EA6508AA	FDA00002261	Cubana	0	0	0	0	0	0	0	0	0	0	0
SAL_EA6481AA	FSIS1504269	Cubana	0	0	0	0	0	0	0	0	0	0	0
SAL_EA6453AA	FDA00009670	Cubana	0	0	0	0	0	0	0	0	0	0	0
SAL_EA6336AA	FSIS1505164	Cubana	0	0	0	0	0	0	0	0	0	0	0
SAL_EA2825AA	FDA00000360	Cubana	0	0	0	0	0	0	0	0	0	0	0
SAL_EA1322AA	FL_FLDACS-99442	Cubana	0	0	0	0	0	0	0	0	0	0	0
SAL_EA0646AA	FSW0037	Cubana	0	0	0	0	0	0	0	0	0	0	0
SAL_EA0560AA	FCC0041	Cubana	0	0	0	0	0	0	0	0	0	0	0
SAL_EA0174AA	NY_IDR1000037972	Cubana	0	0	0	0	0	0	0	0	0	0	0
SAL_DA9820AA	FMA0069	Cubana	0	0	0	0	0	0	0	0	0	0	0
SAL_DA6047AA	IEH-NGS-SAL-00640	Cubana	0	0	0	0	0	0	0	0	0	0	0
SAL_DA6039AA	IEH-NGS-SAL-00628	Cubana	0	0	0	0	0	0	0	0	0	0	0
SAL_DA5625AA	FDA00004006	Cubana	0	0	0	0	0	0	0	0	0	0	0
SAL_DA5614AA	FDA00004079	Cubana	0	0	0	0	0	0	0	0	0	0	0
SAL_DA5613AA	FDA00004080	Cubana	0	0	0	0	0	0	0	0	0	0	0
SAL_DA5615AA	FDA00004078	Cubana	0	0	0	0	0	0	0	0	0	0	0
SAL_DA2196AA	FNW19a94	Cubana	0	0	0	0	0	0	0	0	0	0	0
SAL_DA2038AA	AM04707	Cubana	0	0	0	0	0	0	0	0	0	0	0
SAL_DA1537AA	FDA00003988	Cubana	0	0	0	0	0	0	0	0	0	0	0
SAL_DA1023AA	H123960570	Cubana	0	0	0	0	0	0	0	0	0	0	0
SAL_CA9381AA	FDA00004795	Cubana	0	0	0	0	0	0	0	0	0	0	0
SAL_CA8852AA	IEH-NGS-SAL-01797	Cubana	0	0	0	0	0	0	0	0	0	0	0
SAL_CA8681AA	CFSAN027939	Cubana	0	0	0	0	0	0	0	0	0	0	0
SAL_CA8673AA	CFSAN027938	Cubana	0	0	0	0	0	0	0	0	0	0	0
SAL_CA8667AA	CFSAN027930	Cubana	0	0	0	0	0	0	0	0	0	0	0
SAL_CA8665AA	CFSAN027936	Cubana	0	0	0	0	0	0	0	0	0	0	0
SAL_CA8645AA	IEH-NGS-SAL-01829	Cubana	0	0	0	0	0	0	0	0	0	0	0
SAL_CA8620AA	IEH-NGS-SAL-01833	Cubana	0	0	0	0	0	0	0	0	0	0	0
SAL_CA8477AA	IEH-NGS-SAL-01856	Cubana	0	0	0	0	0	0	0	0	0	0	0
SAL_CA8489AA	FDA00002604	Cubana	0	0	0	0	0	0	0	0	0	0	0
SAL_CA8456AA	FDA00002600	Cubana	0	0	0	0	0	0	0	0	0	0	0
SAL_CA8447AA	CFSAN027977	Cubana	0	0	0	0	0	0	0	0	0	0	0
SAL_CA8391AA	FDA00002606	Cubana	0	0	0	0	0	0	0	0	0	0	0
SAL_CA8385AA	FDA00002605	Cubana	0	0	0	0	0	0	0	0	0	0	0
SAL_CA8367AA	CFSAN027959	Cubana	0	0	0	0	0	0	0	0	0	0	0
SAL_CA8352AA	CFSAN027979	Cubana	0	0	0	0	0	0	0	0	0	0	0
SAL_CA8307AA	FDA00002602	Cubana	0	0	0	0	0	0	0	0	0	0	0
SAL_CA8304AA	FDA00002599	Cubana	0	0	0	0	0	0	0	0	0	0	0
SAL_CA7431AA	FSF0201	Cubana	0	0	0	0	0	0	0	0	0	0	0
SAL_BA7952AA	CFSAN001083	Cubana	0	0	0	0	0	0	0	0	0	0	0
SAL_BA6859AA	FDA00002664	Cubana	0	0	0	0	0	0	0	0	0	0	0
SAL_BA5120AA	IEH_NGS_SAL_00176	Cubana	0	0	0	0	0	0	0	0	0	0	0
SAL_BA4314AA	FDA00004667	Cubana	0	0	0	0	0	0	0	0	0	0	0
SAL_BA1771AA	FDA00002223	Cubana	0	0	0	0	0	0	0	0	0	0	0
SAL_BA1765AA	FDA00002270	Cubana	0	0	0	0	0	0	0	0	0	0	0
SAL_BA0907AA	FDA00001886	Cubana	0	0	0	0	0	0	0	0	0	0	0
SAL_BA0870AA	FDA00001883	Cubana	0	0	0	0	0	0	0	0	0	0	0
SAL_FA6183AA	FSIS1606395	Cubana	0	0	0	0	0	0	0	0	0	0	0
SAL_FA6662AA	2016K-0248	Cubana	0	0	0	0	0	0	0	0	0	0	0
SAL_FA7462AA	CFSAN032645	Cubana	0	0	0	0	0	0	0	0	0	0	0
SAL_FA7665AA	FDA00003712	Cubana	0	0	0	0	0	0	0	0	0	0	0
SAL_FA7902AA	FSIS1606767	Cubana	0	0	0	0	0	0	0	0	0	0	0
SAL_FA8182AA	FDA00003352	Cubana	0	0	0	0	0	0	0	0	0	0	0
SAL_GA2823AA	CRJJGF_00088	Cubana	0	0	0	0	0	0	0	0	0	0	0
SAL_GA3837AA	BCW_1606	Cubana	0	0	0	0	0	0	0	0	0	0	0
SAL_GA3082AA	BCW_4351	Cubana	0	0	0	0	0	0	0	0	0	0	0
SAL_GA2549AA	98-0352	Cubana	0	0	0	0	0	0	0	0	0	0	0
SAL_GA2527AA	89-0199	Cubana	0	0	0	0	0	0	0	0	0	0	0
SAL_GA2531AA	49-1017	Cubana	0	0	0	0	0	0	0	0	0	0	0
SAL_GA4922AA	FDA00010319	Cubana	0	0	0	0	0	0	0	0	0	0	0
SAL_FA9465AA	FDA00002812	Cubana	0	0	0	0	0	0	0	0	0	0	0
SAL_FA9464AA	FDA00002813	Cubana	0	0	0	0	0	0	0	0	0	0	0
SAL_FA8435AA	FDA00010245	Cubana	0	0	0	0	0	0	0	0	0	0	0
SAL_FA8434AA	FDA00010246	Cubana	0	0	0	0	0	0	0	0	0	0	0
SAL_FA8433AA	FDA00010247	Cubana	0	0	0	0	0	0	0	0	0	0	0
SAL_FA8432AA	FDA00010248	Cubana	0	0	0	0	0	0	0	0	0	0	0
SAL_FA8431AA	FDA00010249	Cubana	0	0	0	0	0	0	0	0	0	0	0
SAL_FA8430AA	FDA00010250	Cubana	0	0	0	0	0	0	0	0	0	0	0
SAL_FA8429AA	FDA00010251	Cubana	0	0	0	0	0	0	0	0	0	0	0
SAL_GA6051AA	FSIS1607170	Cubana	0	0	0	0	0	0	0	0	0	0	0

SAL_GA8459AA	OH-10-00034	Cubana	0	0	0	0	0	0	0	0	0	0	0
SAL_GA8776AA	PNUSAS003473	Cubana	0	0	0	0	0	0	0	0	0	0	0
SAL_GA8820AA	OH-11-00011	Cubana	0	0	0	0	0	0	0	0	0	0	0
SAL_GA9073AA	OH-11-00012	Cubana	0	0	0	0	0	0	0	0	0	0	0
SAL_GA9224AA	FDA00010635	Cubana	0	0	0	0	0	0	0	0	0	0	0
SAL_GA9311AA	FSIS1607554	Cubana	0	0	0	0	0	0	0	0	0	0	0
SAL_GA9340AA	FDA00010642	Cubana	0	0	0	0	0	0	0	0	0	0	0
SAL_HA0109AA	FDA00004949	Cubana	0	0	0	0	0	0	0	0	0	0	0
SAL_HA0108AA	FDA00004950	Cubana	0	0	0	0	0	0	0	0	0	0	0
SAL_HA0107AA	FDA00004951	Cubana	0	0	0	0	0	0	0	0	0	0	0
SAL_HA5973AA	CFSAN056583	Cubana	0	0	0	0	0	0	0	0	0	0	0
SAL_HA6090AA	CFSAN056614	Cubana	0	0	0	0	0	0	0	0	0	0	0
SAL_HA7228AA	FDA00010806	Cubana	0	0	0	0	0	0	0	0	0	0	0
SAL_HA7663AA	FDA00011096	Cubana	0	0	0	0	0	0	0	0	0	0	0
SAL_HA7662AA	FDA00011097	Cubana	0	0	0	0	0	0	0	0	0	0	0
SAL_HA7665AA	FDA00011094	Cubana	0	0	0	0	0	0	0	0	0	0	0
SAL_HA7664AA	FDA00011095	Cubana	0	0	0	0	0	0	0	0	0	0	0
SAL_HA7660AA	FDA00011099	Cubana	0	0	0	0	0	0	0	0	0	0	0
SAL_HA7666AA	FDA00011093	Cubana	0	0	0	0	0	0	0	0	0	0	0
SAL_HA7661AA	FDA00011098	Cubana	0	0	0	0	0	0	0	0	0	0	0
SAL_IA5109AA	FDA00002309	Cubana	0	0	0	0	0	0	0	0	0	0	0
SAL_IA7372AA	FDA00011318	Cubana	0	0	0	0	0	0	0	0	0	0	0
SAL_JA1056AA	PNUSAS011049	Cubana	0	0	0	0	0	0	0	0	0	0	0
SAL_JA4410AA	194K	Cubana	0	0	0	0	0	0	0	0	0	0	0
SAL_JA6695AA	PNUSAS013276	Cubana	0	0	0	0	0	0	0	0	0	0	0
SAL_KA0212AA	FDA00011926	Cubana	0	0	0	0	0	0	0	0	0	0	0
SAL_KA1897AA	PNUSAS014713	Cubana	0	0	0	0	0	0	0	0	0	0	0
SAL_KA4660AA	PNUSAS017073	Cubana	0	0	0	0	0	0	0	0	0	0	0
SAL_KA4050AA	RM_486	Cubana	0	0	0	0	0	0	0	0	0	0	0
SAL_KA3979AA	RM_92	Cubana	0	0	0	0	0	0	0	0	0	0	0
SAL_KA5798AA	PNUSAS018146	Cubana	0	0	0	0	0	0	0	0	0	0	0
SAL_KA8600AA	PNUSAS019647	Cubana	0	0	0	0	0	0	0	0	0	0	0
SAL_LA0310AA	FDA00012097	Cubana	0	0	0	0	0	0	0	0	0	0	0
SAL_LA0920AA	FDA00002591	Cubana	0	0	0	0	0	0	0	0	0	0	0
SAL_LA2346AA	FDA00002289	Cubana	0	0	0	0	0	0	0	0	0	0	0
SAL_LA5592AA	FDA00000809	Cubana	0	0	0	0	0	0	0	0	0	0	0
SAL_LA5594AA	FDA00000730	Cubana	0	0	0	0	0	0	0	0	0	0	0
SAL_LA6267AA	MDP-11-00060	Cubana	0	0	0	0	0	0	0	0	0	0	0
SAL_LA6050AA	FSIS11704414	Cubana	0	0	0	0	0	0	0	0	0	0	0
SAL_LA9572AA	FDA00002339	Cubana	0	0	0	0	0	0	0	0	0	0	0
SAL_MA1269AA	B1714040	Cubana	0	0	0	0	0	0	0	0	0	0	0
SAL_MA4454AA	FDA00000182	Cubana	0	0	0	0	0	0	0	0	0	0	0
SAL_DA6267AA	FNE0144	Cubana	0	0	0	0	0	0	0	0	0	0	0
SAL_BA1780AA	FDA00000749	Cubana	0	0	0	0	0	0	0	0	0	0	0
SAL_CA8470AA	FDA00002603	Cubana	0	0	0	0	0	0	0	0	0	0	0
SAL_DA5626AA	FDA00004005	Cubana	0	0	0	0	0	0	0	0	0	0	0
SAL_BA9450AA	CFSAN002050	Cubana	0	0	0	0	0	0	0	0	0	0	0
SAL_GA9339AA	FDA00010643	Cubana	0	0	0	0	0	0	0	0	0	0	0
SAL_HA5825AA	OH-1404746	Cubana	0	0	0	0	0	0	0	0	0	0	0
SAL_HA7220AA	FDA00010815	Cubana	0	0	0	0	0	0	0	0	0	0	0
SAL_HA6084AA	FDA00011014	Cubana	0	0	0	0	0	0	0	0	0	0	0
SAL_FA8448AA	FDA00000114	Cubana	0	0	0	0	0	0	0	0	0	0	0
SAL_EA6461AA	FDA00002260	Cubana	0	0	0	0	0	0	0	0	0	0	0
SAL_JA0240AA	PNUSAS010078	Cubana	0	0	0	0	0	0	0	0	0	0	0
SAL_JA2461AA	PNUSAS010790	Cubana	0	0	0	0	0	0	0	0	0	0	0
SAL_JA3269AA	PNUSAS011865	Cubana	0	0	0	0	0	0	0	0	0	0	0
SAL_LA1059AA	PNUSAS018401	Cubana	0	0	0	0	0	0	0	0	0	0	0
SAL_MA7364AA	OH-17-8676-5	Cubana	0	0	0	0	0	0	0	0	0	0	0
SAL_MA6812AA	FDA00004944	Cubana	0	0	0	0	0	0	0	0	0	0	0
SAL_NA0369AA	PNUSAS015872	Cubana	0	0	0	0	0	0	0	0	0	0	0
SAL_NA1618AA	FSIS11706331	Cubana	0	0	0	0	0	0	0	0	0	0	0
SAL_NA4365AA	DFPST0087	Cubana	0	0	0	0	0	0	0	0	0	0	0
SAL_NA4369AA	DFPST0084	Cubana	0	0	0	0	0	0	0	0	0	0	0
SAL_NA5887AA	PNUSAS030974	Cubana	0	0	0	0	0	0	0	0	0	0	0
SAL_NA9954AA	FSIS31800128	Cubana	0	0	0	0	0	0	0	0	0	0	0
SAL_OA5697AA	FDA00012752	Cubana	0	0	0	0	0	0	0	0	0	0	0
SAL_OA5700AA	FDA00012750	Cubana	0	0	0	0	0	0	0	0	0	0	0
SAL_OA5701AA	FDA00012749	Cubana	0	0	0	0	0	0	0	0	0	0	0
SAL_OA7351AA	PNUSAS037177	Cubana	0	0	0	0	0	0	0	0	0	0	0
SAL_OA5435AA	PNUSAS035639	Cubana	0	0	0	0	0	0	0	0	0	0	0
SAL_OA9624AA	PNUSAS037232	Cubana	0	0	0	0	0	0	0	0	0	0	0
SAL_PA0371AA	PNUSAS037890	Cubana	0	0	0	0	0	0	0	0	0	0	0
SAL_PA0685AA	CFSAN071944	Cubana	0	0	0	0	0	0	0	0	0	0	0

SAL_PA0821AA	CFSAN077981	Cubana	0	0	0	0	0	0	0	0	0	0	0
SAL_PA1590AA	CFSAN078006	Cubana	0	0	0	0	0	0	0	0	0	0	0
SAL_PA1558AA	CFSAN078014	Cubana	0	0	0	0	0	0	0	0	0	0	0
SAL_PA1585AA	CFSAN078010	Cubana	0	0	0	0	0	0	0	0	0	0	0
SAL_PA4285AA	278205	Cubana	0	0	0	0	0	0	0	0	0	0	0
SAL_PA4436AA	PNUSAS039190	Cubana	0	0	0	0	0	0	0	0	0	0	0
SAL_PA4359AA	PNUSAS039818	Cubana	0	0	0	0	0	0	0	0	0	0	0
SAL_PA5706AA	PNUSAS040051	Cubana	0	0	0	0	0	0	0	0	0	0	0
SAL_PA6948AA	PNUSAS040685	Cubana	0	0	0	0	0	0	0	0	0	0	0
SAL_PA7568AA	PNUSAS039191	Cubana	0	0	0	0	0	0	0	0	0	0	0
SAL_PA8141AA	PNUSAS040389	Cubana	0	0	0	0	0	0	0	0	0	0	0
SAL_PA8208AA	PNUSAS041070	Cubana	0	0	0	0	0	0	0	0	0	0	0
SAL_PA8437AA	FDA00001699	Cubana	0	0	0	0	0	0	0	0	0	0	0
SAL_PA8579AA	278194	Cubana	0	0	0	0	0	0	0	0	0	0	0
SAL_PA9178AA	PNUSAS041484	Cubana	0	0	0	0	0	0	0	0	0	0	0
SAL_PA9566AA	PNUSAS041316	Cubana	0	0	0	0	0	0	0	0	0	0	0
SAL_QA0940AA	PNUSAS041332	Cubana	0	0	0	0	0	0	0	0	0	0	0
SAL_QA3673AA	PNUSAS040683	Cubana	0	0	0	0	0	0	0	0	0	0	0
SAL_QA4035AA	238469	Cubana	0	0	0	0	0	0	0	0	0	0	0
SAL_QA8490AA	PNUSAS042976	Cubana	0	0	0	0	0	0	0	0	0	0	0
SAL_RA0170AA	PNUSAS043916	Cubana	0	0	0	0	0	0	0	0	0	0	0
SAL_RA0242AA	399751	Cubana	0	0	0	0	0	0	0	0	0	0	0
SAL_RA1119AA	190590	Cubana	0	0	0	0	0	0	0	0	0	0	0
SAL_RA3701AA	279516	Cubana	0	0	0	0	0	0	0	0	0	0	0
SAL_RA5674AA	250717	Cubana	0	0	0	0	0	0	0	0	0	0	0
SAL_SA2800AA	18038291	Cubana	0	0	0	0	0	0	0	0	0	0	0
SAL_RA6807AA	PNUSAS045115	Cubana	0	0	0	0	0	0	0	0	0	0	0
SAL_SA4369AA	FDA00004678	Cubana	0	0	0	0	0	0	0	0	0	0	0
SAL_SA9663AA	FSIS31801090	Cubana	0	0	0	0	0	0	0	0	0	0	0
SAL_TA6980AA	CFSAN087643	Cubana	0	0	0	0	0	0	0	0	0	0	0
SAL_PA1520AA	PNUSAS038019	Cubana	0	0	0	0	0	0	0	0	0	0	0
SAL_PA1559AA	CFSAN078005	Cubana	0	0	0	0	0	0	0	0	0	0	0
SAL_FA7797AA	CFSAN032650	Cubana	0	0	0	0	0	0	0	0	0	0	0
SAL_TA8829AA	FSIS11815713	Cubana	0	0	0	0	0	0	0	0	0	0	0
SAL_NA1255AA	FDA00001472	Cubana	0	0	0	0	0	0	0	0	0	0	0
SAL_OA6049AA	CFSAN078051	Cubana	0	0	0	0	0	0	0	0	0	0	0
SAL_OA6057AA	CFSAN078049	Cubana	0	0	0	0	0	0	0	0	0	0	0
SAL_OA6673AA	CFSAN078050	Cubana	0	0	0	0	0	0	0	0	0	0	0
SAL_OA6682AA	CFSAN078053	Cubana	0	0	0	0	0	0	0	0	0	0	0
SAL_PA1568AA	CFSAN078013	Cubana	0	0	0	0	0	0	0	0	0	0	0
SAL_PA1587AA	CFSAN078007	Cubana	0	0	0	0	0	0	0	0	0	0	0
SAL_PA1555AA	CFSAN078008	Cubana	0	0	0	0	0	0	0	0	0	0	0
SAL_PA1589AA	CFSAN078009	Cubana	0	0	0	0	0	0	0	0	0	0	0
SAL_PA1582AA	CFSAN078012	Cubana	0	0	0	0	0	0	0	0	0	0	0
SAL_PA1586AA	CFSAN078011	Cubana	0	0	0	0	0	0	0	0	0	0	0
SAL_PA1576AA	CFSAN078015	Cubana	0	0	0	0	0	0	0	0	0	0	0
SAL_PA1573AA	CFSAN078004	Cubana	0	0	0	0	0	0	0	0	0	0	0
SAL_PA2017AA	PNUSAS038739	Cubana	0	0	0	0	0	0	0	0	0	0	0
SAL_PA4129AA	CFSAN078636	Cubana	0	0	0	0	0	0	0	0	0	0	0
SAL_PA6039AA	PNUSAS037168	Cubana	0	0	0	0	0	0	0	0	0	0	0
SAL_TA9644AA	FDA00000773	Cubana	0	0	0	0	0	0	0	0	0	0	0
SAL_UA1767AA	CFSAN088526	Cubana	0	0	0	0	0	0	0	0	0	0	0
SAL_UA1711AA	CFSAN088674	Cubana	0	0	0	0	0	0	0	0	0	0	0
SAL_UA4924AA	CFSAN089107	Cubana	0	0	0	0	0	0	0	0	0	0	0
SAL_UA4919AA	CFSAN089129	Cubana	0	0	0	0	0	0	0	0	0	0	0
SAL_UA6042AA	651047	Cubana	0	0	0	0	0	0	0	0	0	0	0
SAL_UA8716AA	FSIS11917064	Cubana	0	0	0	0	0	0	0	0	0	0	0
SAL_VA0893AA	ADRDL-2386	Cubana	0	0	0	0	0	0	0	0	0	0	0
SAL_VA1258AA	480750	Cubana	0	0	0	0	0	0	0	0	0	0	0
SAL_VA7403AA	201701193	Cubana	0	0	0	0	0	0	0	0	0	0	0
SAL_VA7413AA	201701396	Cubana	0	0	0	0	0	0	0	0	0	0	0
SAL_VA7468AA	201704058	Cubana	0	0	0	0	0	0	0	0	0	0	0
SAL_VA7484AA	201704723	Cubana	0	0	0	0	0	0	0	0	0	0	0
SAL_VA7599AA	201707151	Cubana	0	0	0	0	0	0	0	0	0	0	0
SAL_VA7607AA	201709375	Cubana	0	0	0	0	0	0	0	0	0	0	0
SAL_VA7626AA	201707678	Cubana	0	0	0	0	0	0	0	0	0	0	0
SAL_VA7693AA	201703490	Cubana	0	0	0	0	0	0	0	0	0	0	0
SAL_VA3033AA	DAC35	Cubana	0	0	0	0	0	0	0	0	0	0	0
SAL_VA3037AA	DAC28	Cubana	0	0	0	0	0	0	0	0	0	0	0
SAL_VA4217AA	CDPH_CSalm132	Cubana	0	0	0	0	0	0	0	0	0	0	0
SAL_VA9517AA	267014	Cubana	0	0	0	0	0	0	0	0	0	0	0
SAL_WA3034AA	GW_S01409-06	Cubana	0	0	0	0	0	0	0	0	0	0	0
SAL_WA3023AA	GW_S04270-08	Cubana	0	0	0	0	0	0	0	0	0	0	0

SAL_WA1560AA	122238	Cubana	0	0	0	0	0	0	0	0	0	0	0
SAL_WA3678AA	CFSAN087456	Cubana	0	0	0	0	0	0	0	0	0	0	0
SAL_WA3739AA	CFSAN087607	Cubana	0	0	0	0	0	0	0	0	0	0	0
SAL_WA3546AA	CFSAN087608	Cubana	0	0	0	0	0	0	0	0	0	0	0
SAL_NA5234AA	ADRDL-1614	Cumberland	0	0	0	0	0	0	0	0	0	0	0
SAL_EA7918AA	21063	Curacao	0	0	0	0	0	0	0	0	0	0	0
SAL_DA0402AA	H120220759	Curacao	0	0	0	0	0	0	0	0	0	0	0
SAL_CA2569AA	86849	Curacao	0	0	0	0	0	0	0	0	0	0	0
SAL_CA0152AA	40338	Curacao	0	0	0	0	0	0	0	0	0	0	0
SAL_DA0431AA	H124320370	Curacao	0	0	0	0	0	0	0	0	0	0	0
SAL_EA0946AA	4-sc-2013-02-28T11:35:	Curacao	0	0	0	0	0	0	0	0	0	0	0
SAL_PA5670AA	PNUSAS034851	Curacao	0	0	0	0	0	0	0	0	0	0	0
SAL_QA4209AA	PNUSAS039727	Curacao	0	0	0	0	0	0	0	0	0	0	0
SAL_SA6253AA	582286	Curacao	0	0	0	0	0	0	0	0	0	0	0
SAL_WA0202AA	229069	Curacao	0	0	0	0	0	0	0	0	0	0	0
SAL_FA6504AA	FDA00010135	Dahra	0	0	0	0	0	0	0	0	0	0	0
SAL_EA8093AA	18636	Derby	0	0	0	0	0	0	0	0	0	0	0
SAL_BA8190AA	CFSAN005743	Derby	0	0	0	0	0	0	0	0	0	0	0
SAL_EA3175AA	CVM-N16414	Derby	0	0	0	0	0	0	0	0	0	0	0
SAL_BA9342AA	69894	Derby	0	0	0	0	0	0	0	0	0	0	0
SAL_DA0020AA	FDA00003562	Derby	0	0	0	0	0	0	0	0	0	0	0
SAL_EA6145AA	FSIS1500963	Derby	0	0	0	0	0	0	0	0	0	0	0
SAL_DA0258AA	FDA00000199	Derby	0	0	0	0	0	0	0	0	0	0	0
SAL_BA5116AA	IEH_NGS_SAL_00186	Derby	0	0	0	0	0	0	0	0	0	0	0
SAL_EA3038AA	FSIS1502795	Derby	0	0	0	0	0	0	0	0	0	0	0
SAL_FA5836AA	HIY0224	Derby	0	0	0	0	0	0	0	0	0	0	0
SAL_FA5714AA	CFSAN032044	Derby	0	0	0	0	0	0	0	0	0	0	0
SAL_FA5711AA	CFSAN032050	Derby	0	0	0	0	0	0	0	0	0	0	0
SAL_FA5713AA	CFSAN032049	Derby	0	0	0	0	0	0	0	0	0	0	0
SAL_FA5618AA	CFSAN032094	Derby	0	0	0	0	0	0	0	0	0	0	0
SAL_FA5611AA	CFSAN032092	Derby	0	0	0	0	0	0	0	0	0	0	0
SAL_FA5612AA	CFSAN032097	Derby	0	0	0	0	0	0	0	0	0	0	0
SAL_FA5614AA	CFSAN032084	Derby	0	0	0	0	0	0	0	0	0	0	0
SAL_FA5608AA	CFSAN032090	Derby	0	0	0	0	0	0	0	0	0	0	0
SAL_FA5601AA	CFSAN032083	Derby	0	0	0	0	0	0	0	0	0	0	0
SAL_FA5607AA	CFSAN032085	Derby	0	0	0	0	0	0	0	0	0	0	0
SAL_FA5599AA	CFSAN032086	Derby	0	0	0	0	0	0	0	0	0	0	0
SAL_FA5598AA	CFSAN032081	Derby	0	0	0	0	0	0	0	0	0	0	0
SAL_FA5470AA	CFSAN032064	Derby	0	0	0	0	0	0	0	0	0	0	0
SAL_FA5468AA	CFSAN032072	Derby	0	0	0	0	0	0	0	0	0	0	0
SAL_FA5458AA	CFSAN032063	Derby	0	0	0	0	0	0	0	0	0	0	0
SAL_FA5456AA	CFSAN032071	Derby	0	0	0	0	0	0	0	0	0	0	0
SAL_FA5462AA	CFSAN032065	Derby	0	0	0	0	0	0	0	0	0	0	0
SAL_FA5447AA	FSIS1606285	Derby	0	0	0	0	0	0	0	0	0	0	0
SAL_FA5437AA	FSIS1606176	Derby	0	0	0	0	0	0	0	0	0	0	0
SAL_FA5145AA	CFSAN032015	Derby	0	0	0	0	0	0	0	0	0	0	0
SAL_FA5102AA	CFSAN032024	Derby	0	0	0	0	0	0	0	0	0	0	0
SAL_FA5107AA	CFSAN031996	Derby	0	0	0	0	0	0	0	0	0	0	0
SAL_FA5103AA	CFSAN031986	Derby	0	0	0	0	0	0	0	0	0	0	0
SAL_FA5100AA	CFSAN032019	Derby	0	0	0	0	0	0	0	0	0	0	0
SAL_FA5110AA	CFSAN032006	Derby	0	0	0	0	0	0	0	0	0	0	0
SAL_FA5082AA	CFSAN032025	Derby	0	0	0	0	0	0	0	0	0	0	0
SAL_FA5075AA	CFSAN032008	Derby	0	0	0	0	0	0	0	0	0	0	0
SAL_FA4967AA	CFSAN047617	Derby	0	0	0	0	0	0	0	0	0	0	0
SAL_FA4925AA	CFSAN045274	Derby	0	0	0	0	0	0	0	0	0	0	0
SAL_FA4803AA	CVM-N27363	Derby	0	0	0	0	0	0	0	0	0	0	0
SAL_FA4793AA	FSIS1606118	Derby	0	0	0	0	0	0	0	0	0	0	0
SAL_FA4789AA	CFSAN032039	Derby	0	0	0	0	0	0	0	0	0	0	0
SAL_FA4786AA	CFSAN032042	Derby	0	0	0	0	0	0	0	0	0	0	0
SAL_FA4779AA	CFSAN032038	Derby	0	0	0	0	0	0	0	0	0	0	0
SAL_FA4623AA	181077	Derby	0	0	0	0	0	0	0	0	0	0	0
SAL_FA4494AA	117270	Derby	0	0	0	0	0	0	0	0	0	0	0
SAL_FA4449AA	122646	Derby	0	0	0	0	0	0	0	0	0	0	0
SAL_FA3298AA	CVM-N23666	Derby	0	0	0	0	0	0	0	0	0	0	0
SAL_FA2991AA	CVM N56916F	Derby	0	0	0	0	0	0	0	0	0	0	0
SAL_FA2995AA	CVM N58036	Derby	0	0	0	0	0	0	0	0	0	0	0
SAL_FA2987AA	CVM N56925F	Derby	0	0	0	0	0	0	0	0	0	0	0
SAL_FA2979AA	CVM N56956F	Derby	0	0	0	0	0	0	0	0	0	0	0
SAL_FA2984AA	CVM N56945F	Derby	0	0	0	0	0	0	0	0	0	0	0
SAL_FA2980AA	CVM N56950F	Derby	0	0	0	0	0	0	0	0	0	0	0
SAL_FA2957AA	CVM N57046F	Derby	0	0	0	0	0	0	0	0	0	0	0
SAL_FA2960AA	CVM N57036F	Derby	0	0	0	0	0	0	0	0	0	0	0
SAL_FA2947AA	CVM N57069F	Derby	0	0	0	0	0	0	0	0	0	0	0

SAL_FA2935AA	CVM N57117F	Derby	0	0	0	0	0	0	0	0	0	0
SAL_FA2933AA	CVM N57125F	Derby	0	0	0	0	0	0	0	0	0	0
SAL_FA2926AA	CVM N57149F	Derby	0	0	0	0	0	0	0	0	0	0
SAL_FA2917AA	CVM N57183F	Derby	0	0	0	0	0	0	0	0	0	0
SAL_FA2911AA	CVM N57195F	Derby	0	0	0	0	0	0	0	0	0	0
SAL_FA2892AA	CVM N57252F	Derby	0	0	0	0	0	0	0	0	0	0
SAL_FA2900AA	CVM N57226F	Derby	0	0	0	0	0	0	0	0	0	0
SAL_FA2888AA	CVM N57264F	Derby	0	0	0	0	0	0	0	0	0	0
SAL_FA2883AA	CVM N57285F	Derby	0	0	0	0	0	0	0	0	0	0
SAL_FA2878AA	CVM N57298F	Derby	0	0	0	0	0	0	0	0	0	0
SAL_FA2851AA	CVM N57388F	Derby	0	0	0	0	0	0	0	0	0	0
SAL_FA2839AA	CVM N57408F	Derby	0	0	0	0	0	0	0	0	0	0
SAL_FA2828AA	CVM N57468F	Derby	0	0	0	0	0	0	0	0	0	0
SAL_FA2803AA	CVM N57571F	Derby	0	0	0	0	0	0	0	0	0	0
SAL_FA2796AA	CVM N57595F	Derby	0	0	0	0	0	0	0	0	0	0
SAL_FA2792AA	CVM N57603F	Derby	0	0	0	0	0	0	0	0	0	0
SAL_FA2785AA	CVM N57621F	Derby	0	0	0	0	0	0	0	0	0	0
SAL_FA2784AA	CVM N57622F	Derby	0	0	0	0	0	0	0	0	0	0
SAL_FA2781AA	CVM N57630F	Derby	0	0	0	0	0	0	0	0	0	0
SAL_FA2779AA	CVM N57632F	Derby	0	0	0	0	0	0	0	0	0	0
SAL_FA2774AA	CVM N57648F	Derby	0	0	0	0	0	0	0	0	0	0
SAL_FA2769AA	CVM N57659F	Derby	0	0	0	0	0	0	0	0	0	0
SAL_FA2765AA	CVM N57667F	Derby	0	0	0	0	0	0	0	0	0	0
SAL_FA2761AA	CVM N57683F	Derby	0	0	0	0	0	0	0	0	0	0
SAL_FA2745AA	CVM N57722F	Derby	0	0	0	0	0	0	0	0	0	0
SAL_FA2759AA	CVM N57689F	Derby	0	0	0	0	0	0	0	0	0	0
SAL_FA2742AA	CVM N57731F	Derby	0	0	0	0	0	0	0	0	0	0
SAL_FA2731AA	CVM N57775F	Derby	0	0	0	0	0	0	0	0	0	0
SAL_FA2723AA	CVM N57792F	Derby	0	0	0	0	0	0	0	0	0	0
SAL_FA2722AA	CVM N57800F	Derby	0	0	0	0	0	0	0	0	0	0
SAL_FA2718AA	CVM N57811F	Derby	0	0	0	0	0	0	0	0	0	0
SAL_FA2720AA	CVM N57804F	Derby	0	0	0	0	0	0	0	0	0	0
SAL_FA2712AA	CVM N57830F	Derby	0	0	0	0	0	0	0	0	0	0
SAL_FA2694AA	CVM N57886F	Derby	0	0	0	0	0	0	0	0	0	0
SAL_FA2664AA	CVM N58616F	Derby	0	0	0	0	0	0	0	0	0	0
SAL_FA2669AA	CVM N57961F	Derby	0	0	0	0	0	0	0	0	0	0
SAL_FA2655AA	HIY0057	Derby	0	0	0	0	0	0	0	0	0	0
SAL_FA2656AA	HIY0019	Derby	0	0	0	0	0	0	0	0	0	0
SAL_FA2653AA	HIY0372	Derby	0	0	0	0	0	0	0	0	0	0
SAL_FA2527AA	CFSAN047033	Derby	0	0	0	0	0	0	0	0	0	0
SAL_FA2375AA	IEH-NGS-SAL-03810	Derby	0	0	0	0	0	0	0	0	0	0
SAL_FA2345AA	CFSAN047101	Derby	0	0	0	0	0	0	0	0	0	0
SAL_FA2214AA	CVM-N19775	Derby	0	0	0	0	0	0	0	0	0	0
SAL_FA2165AA	FSIS1605837	Derby	0	0	0	0	0	0	0	0	0	0
SAL_FA2164AA	FSIS1605835	Derby	0	0	0	0	0	0	0	0	0	0
SAL_FA1933AA	PNUSAS001657	Derby	0	0	0	0	0	0	0	0	0	0
SAL_FA1884AA	FSIS1605823	Derby	0	0	0	0	0	0	0	0	0	0
SAL_FA1866AA	FDA00001608	Derby	0	0	0	0	0	0	0	0	0	0
SAL_FA1749AA	HIY0236	Derby	0	0	0	0	0	0	0	0	0	0
SAL_FA1735AA	HIY0317	Derby	0	0	0	0	0	0	0	0	0	0
SAL_FA1738AA	HIY0316	Derby	0	0	0	0	0	0	0	0	0	0
SAL_FA1566AA	CFSAN046121	Derby	0	0	0	0	0	0	0	0	0	0
SAL_FA1565AA	IEH-NGS-SAL-03610	Derby	0	0	0	0	0	0	0	0	0	0
SAL_FA1328AA	FSIS1605756	Derby	0	0	0	0	0	0	0	0	0	0
SAL_FA1276AA	CVM-N18557	Derby	0	0	0	0	0	0	0	0	0	0
SAL_FA1247AA	FDA00002975	Derby	0	0	0	0	0	0	0	0	0	0
SAL_FA1220AA	IEH-NGS-SAL-03531	Derby	0	0	0	0	0	0	0	0	0	0
SAL_FA1218AA	IEH-NGS-SAL-03533	Derby	0	0	0	0	0	0	0	0	0	0
SAL_FA1203AA	IEH-NGS-SAL-03548	Derby	0	0	0	0	0	0	0	0	0	0
SAL_FA1204AA	IEH-NGS-SAL-03547	Derby	0	0	0	0	0	0	0	0	0	0
SAL_FA1193AA	IEH-NGS-SAL-03558	Derby	0	0	0	0	0	0	0	0	0	0
SAL_FA1189AA	CFSAN045725	Derby	0	0	0	0	0	0	0	0	0	0
SAL_FA1192AA	CFSAN045722	Derby	0	0	0	0	0	0	0	0	0	0
SAL_FA1181AA	CFSAN045733	Derby	0	0	0	0	0	0	0	0	0	0
SAL_FA1177AA	CFSAN045737	Derby	0	0	0	0	0	0	0	0	0	0
SAL_FA1156AA	NY-N20116	Derby	0	0	0	0	0	0	0	0	0	0
SAL_FA0896AA	FSIS1605572	Derby	0	0	0	0	0	0	0	0	0	0
SAL_FA0837AA	FSIS1605658	Derby	0	0	0	0	0	0	0	0	0	0
SAL_FA0790AA	HIY0132	Derby	0	0	0	0	0	0	0	0	0	0
SAL_FA0788AA	HIY0354	Derby	0	0	0	0	0	0	0	0	0	0
SAL_FA0787AA	HIY0367	Derby	0	0	0	0	0	0	0	0	0	0
SAL_FA0789AA	HIY0074	Derby	0	0	0	0	0	0	0	0	0	0
SAL_FA0784AA	HIY0114	Derby	0	0	0	0	0	0	0	0	0	0

SAL_FA0783AA	HIY0127	Derby	0	0	0	0	0	0	0	0	0	0	0
SAL_FA0786AA	HIY0374	Derby	0	0	0	0	0	0	0	0	0	0	0
SAL_FA0782AA	HIY0352	Derby	0	0	0	0	0	0	0	0	0	0	0
SAL_FA0785AA	HIY0381	Derby	0	0	0	0	0	0	0	0	0	0	0
SAL_FA0781AA	HIY0373	Derby	0	0	0	0	0	0	0	0	0	0	0
SAL_FA0779AA	HIY0378	Derby	0	0	0	0	0	0	0	0	0	0	0
SAL_FA0777AA	HIY0389	Derby	0	0	0	0	0	0	0	0	0	0	0
SAL_FA0780AA	HIY0375	Derby	0	0	0	0	0	0	0	0	0	0	0
SAL_FA0778AA	HIY0384	Derby	0	0	0	0	0	0	0	0	0	0	0
SAL_FA0759AA	CFSAN045665	Derby	0	0	0	0	0	0	0	0	0	0	0
SAL_FA0758AA	CFSAN045666	Derby	0	0	0	0	0	0	0	0	0	0	0
SAL_FA0757AA	CFSAN045667	Derby	0	0	0	0	0	0	0	0	0	0	0
SAL_FA0756AA	IEH-NGS-SAL-03505	Derby	0	0	0	0	0	0	0	0	0	0	0
SAL_FA0743AA	CFSAN045681	Derby	0	0	0	0	0	0	0	0	0	0	0
SAL_FA0725AA	HIY0120	Derby	0	0	0	0	0	0	0	0	0	0	0
SAL_FA0727AA	HIY0056	Derby	0	0	0	0	0	0	0	0	0	0	0
SAL_FA0728AA	HIY0047	Derby	0	0	0	0	0	0	0	0	0	0	0
SAL_FA0721AA	HIY0186	Derby	0	0	0	0	0	0	0	0	0	0	0
SAL_FA0591AA	CFSAN045537	Derby	0	0	0	0	0	0	0	0	0	0	0
SAL_FA0583AA	IEH-NGS-SAL-03382	Derby	0	0	0	0	0	0	0	0	0	0	0
SAL_FA0568AA	CFSAN045584	Derby	0	0	0	0	0	0	0	0	0	0	0
SAL_FA0567AA	CFSAN045585	Derby	0	0	0	0	0	0	0	0	0	0	0
SAL_FA0560AA	CFSAN045594	Derby	0	0	0	0	0	0	0	0	0	0	0
SAL_FA0559AA	CFSAN045595	Derby	0	0	0	0	0	0	0	0	0	0	0
SAL_FA0555AA	CFSAN045601	Derby	0	0	0	0	0	0	0	0	0	0	0
SAL_FA0537AA	NY-N20103	Derby	0	0	0	0	0	0	0	0	0	0	0
SAL_FA0557AA	CFSAN045597	Derby	0	0	0	0	0	0	0	0	0	0	0
SAL_FA0512AA	FDA00000973	Derby	0	0	0	0	0	0	0	0	0	0	0
SAL_FA0432AA	CFSAN042473	Derby	0	0	0	0	0	0	0	0	0	0	0
SAL_FA0397AA	FSIS1605546	Derby	0	0	0	0	0	0	0	0	0	0	0
SAL_EA9895AA	FSIS1605557	Derby	0	0	0	0	0	0	0	0	0	0	0
SAL_EA9878AA	FSIS1605443	Derby	0	0	0	0	0	0	0	0	0	0	0
SAL_EA9806AA	CVM-N23747	Derby	0	0	0	0	0	0	0	0	0	0	0
SAL_EA9577AA	NY-N20088	Derby	0	0	0	0	0	0	0	0	0	0	0
SAL_EA9576AA	NY-N20089	Derby	0	0	0	0	0	0	0	0	0	0	0
SAL_EA9567AA	FSIS1605650	Derby	0	0	0	0	0	0	0	0	0	0	0
SAL_EA9502AA	CFSAN045572	Derby	0	0	0	0	0	0	0	0	0	0	0
SAL_EA9507AA	IEH-NGS-SAL-03404	Derby	0	0	0	0	0	0	0	0	0	0	0
SAL_EA9504AA	CFSAN045570	Derby	0	0	0	0	0	0	0	0	0	0	0
SAL_EA9493AA	CFSAN045581	Derby	0	0	0	0	0	0	0	0	0	0	0
SAL_EA9456AA	CVM-N23848	Derby	0	0	0	0	0	0	0	0	0	0	0
SAL_EA9363AA	FDA00000288	Derby	0	0	0	0	0	0	0	0	0	0	0
SAL_EA9266AA	FSIS1504812	Derby	0	0	0	0	0	0	0	0	0	0	0
SAL_EA8839AA	CVM-N23822	Derby	0	0	0	0	0	0	0	0	0	0	0
SAL_EA8782AA	FSIS1504961	Derby	0	0	0	0	0	0	0	0	0	0	0
SAL_EA8792AA	FSIS1504827	Derby	0	0	0	0	0	0	0	0	0	0	0
SAL_EA8781AA	FSIS1504759	Derby	0	0	0	0	0	0	0	0	0	0	0
SAL_EA8778AA	FSIS1504753	Derby	0	0	0	0	0	0	0	0	0	0	0
SAL_EA8464AA	FSIS1505033	Derby	0	0	0	0	0	0	0	0	0	0	0
SAL_EA8465AA	FSIS1505028	Derby	0	0	0	0	0	0	0	0	0	0	0
SAL_EA8405AA	ADRDL-15-16081	Derby	0	0	0	0	0	0	0	0	0	0	0
SAL_EA8397AA	ADRDL-15-14678	Derby	0	0	0	0	0	0	0	0	0	0	0
SAL_EA8391AA	FSIS1503600	Derby	0	0	0	0	0	0	0	0	0	0	0
SAL_EA7393AA	43911	Derby	0	0	0	0	0	0	0	0	0	0	0
SAL_EA7369AA	1511	Derby	0	0	0	0	0	0	0	0	0	0	0
SAL_EA6604AA	FSIS1504187	Derby	0	0	0	0	0	0	0	0	0	0	0
SAL_EA6527AA	FDA00001804	Derby	0	0	0	0	0	0	0	0	0	0	0
SAL_EA6485AA	FSIS1504205	Derby	0	0	0	0	0	0	0	0	0	0	0
SAL_EA6482AA	FSIS1504216	Derby	0	0	0	0	0	0	0	0	0	0	0
SAL_EA6364AA	FSIS1503924	Derby	0	0	0	0	0	0	0	0	0	0	0
SAL_EA6361AA	FSIS1503948	Derby	0	0	0	0	0	0	0	0	0	0	0
SAL_EA6369AA	FSIS1503922	Derby	0	0	0	0	0	0	0	0	0	0	0
SAL_EA6338AA	FSIS1505165	Derby	0	0	0	0	0	0	0	0	0	0	0
SAL_EA6223AA	FSIS1504875	Derby	0	0	0	0	0	0	0	0	0	0	0
SAL_EA6213AA	FSIS1504872	Derby	0	0	0	0	0	0	0	0	0	0	0
SAL_EA6131AA	FSIS1501865	Derby	0	0	0	0	0	0	0	0	0	0	0
SAL_EA6103AA	FSIS1501941	Derby	0	0	0	0	0	0	0	0	0	0	0
SAL_EA5973AA	VA-WGS-00614	Derby	0	0	0	0	0	0	0	0	0	0	0
SAL_EA5741AA	FSIS1503040	Derby	0	0	0	0	0	0	0	0	0	0	0
SAL_EA5737AA	FSIS1502875	Derby	0	0	0	0	0	0	0	0	0	0	0
SAL_EA5717AA	FSIS1502599	Derby	0	0	0	0	0	0	0	0	0	0	0
SAL_EA5710AA	FSIS1502443	Derby	0	0	0	0	0	0	0	0	0	0	0
SAL_EA5712AA	FSIS1502593	Derby	0	0	0	0	0	0	0	0	0	0	0

SAL_EA5349AA	FSIS1503817	Derby	0	0	0	0	0	0	0	0	0	0	0
SAL_EA4269AA	FDA00001840	Derby	0	0	0	0	0	0	0	0	0	0	0
SAL_EA3716AA	CVM N46856	Derby	0	0	0	0	0	0	0	0	0	0	0
SAL_EA3678AA	CVM N48686	Derby	0	0	0	0	0	0	0	0	0	0	0
SAL_EA3173AA	CVM-N16415	Derby	0	0	0	0	0	0	0	0	0	0	0
SAL_EA3154AA	CVM-N15832	Derby	0	0	0	0	0	0	0	0	0	0	0
SAL_EA3033AA	FSIS1502804	Derby	0	0	0	0	0	0	0	0	0	0	0
SAL_EA2984AA	CVM-N16435	Derby	0	0	0	0	0	0	0	0	0	0	0
SAL_EA2737AA	CVM-N15253	Derby	0	0	0	0	0	0	0	0	0	0	0
SAL_EA2542AA	CVM-N15252	Derby	0	0	0	0	0	0	0	0	0	0	0
SAL_EA2344AA	VA-WGS-00615	Derby	0	0	0	0	0	0	0	0	0	0	0
SAL_EA2083AA	CVM N53019	Derby	0	0	0	0	0	0	0	0	0	0	0
SAL_EA1979AA	CVM N54274	Derby	0	0	0	0	0	0	0	0	0	0	0
SAL_EA1991AA	CVM N54262	Derby	0	0	0	0	0	0	0	0	0	0	0
SAL_EA1883AA	CVM N55396	Derby	0	0	0	0	0	0	0	0	0	0	0
SAL_EA1479AA	FSIS1500647	Derby	0	0	0	0	0	0	0	0	0	0	0
SAL_EA1432AA	FSIS1500762	Derby	0	0	0	0	0	0	0	0	0	0	0
SAL_EA0258AA	FSW0082	Derby	0	0	0	0	0	0	0	0	0	0	0
SAL_EA0044AA	FSF0014	Derby	0	0	0	0	0	0	0	0	0	0	0
SAL_DA9982AA	WAPHL_SAL-A00143	Derby	0	0	0	0	0	0	0	0	0	0	0
SAL_DA9711AA	MDH-2013-00032	Derby	0	0	0	0	0	0	0	0	0	0	0
SAL_DA9515AA	VA_WGS-00121	Derby	0	0	0	0	0	0	0	0	0	0	0
SAL_DA7255AA	MDH-2014-00054	Derby	0	0	0	0	0	0	0	0	0	0	0
SAL_DA7191AA	CFSAN012657	Derby	0	0	0	0	0	0	0	0	0	0	0
SAL_DA7190AA	CFSAN012661	Derby	0	0	0	0	0	0	0	0	0	0	0
SAL_DA7032AA	MDH-2014-00031	Derby	0	0	0	0	0	0	0	0	0	0	0
SAL_DA6434AA	MDH-2014-00078	Derby	0	0	0	0	0	0	0	0	0	0	0
SAL_DA6407AA	MDH-2014-00289	Derby	0	0	0	0	0	0	0	0	0	0	0
SAL_DA5674AA	MDH-2014-00377	Derby	0	0	0	0	0	0	0	0	0	0	0
SAL_DA4434AA	MDH-2014-00407	Derby	0	0	0	0	0	0	0	0	0	0	0
SAL_DA4426AA	MDH-2014-00397	Derby	0	0	0	0	0	0	0	0	0	0	0
SAL_DA4318AA	MOD1-Sal_902	Derby	0	0	0	0	0	0	0	0	0	0	0
SAL_DA4296AA	MOD1-Sal_903	Derby	0	0	0	0	0	0	0	0	0	0	0
SAL_DA4246AA	NY-swgs1182	Derby	0	0	0	0	0	0	0	0	0	0	0
SAL_DA4223AA	MDH-2014-00431	Derby	0	0	0	0	0	0	0	0	0	0	0
SAL_DA4217AA	MDH-2014-00432	Derby	0	0	0	0	0	0	0	0	0	0	0
SAL_DA4174AA	WAPHL_SAL-A00501	Derby	0	0	0	0	0	0	0	0	0	0	0
SAL_DA4073AA	MDH-2014-00448	Derby	0	0	0	0	0	0	0	0	0	0	0
SAL_DA4062AA	MDH-2014-00449	Derby	0	0	0	0	0	0	0	0	0	0	0
SAL_DA3875AA	WAPHL_SAL-A00533	Derby	0	0	0	0	0	0	0	0	0	0	0
SAL_DA3870AA	WAPHL_SAL-A00532	Derby	0	0	0	0	0	0	0	0	0	0	0
SAL_DA3859AA	MDH-2014-00481	Derby	0	0	0	0	0	0	0	0	0	0	0
SAL_DA3232AA	MDH-2014-00503	Derby	0	0	0	0	0	0	0	0	0	0	0
SAL_DA3226AA	MDH-2014-00506	Derby	0	0	0	0	0	0	0	0	0	0	0
SAL_DA3011AA	MDH-2014-00418	Derby	0	0	0	0	0	0	0	0	0	0	0
SAL_DA3007AA	CFSAN022548	Derby	0	0	0	0	0	0	0	0	0	0	0
SAL_DA2936AA	MDH-2014-00515	Derby	0	0	0	0	0	0	0	0	0	0	0
SAL_DA2929AA	MDH-2014-00514	Derby	0	0	0	0	0	0	0	0	0	0	0
SAL_DA2719AA	MDH-2014-00518	Derby	0	0	0	0	0	0	0	0	0	0	0
SAL_DA2565AA	MDH-2014-00543	Derby	0	0	0	0	0	0	0	0	0	0	0
SAL_DA2521AA	FDA00003933	Derby	0	0	0	0	0	0	0	0	0	0	0
SAL_DA2491AA	FDA00003934	Derby	0	0	0	0	0	0	0	0	0	0	0
SAL_DA2448AA	MDH-2014-00557	Derby	0	0	0	0	0	0	0	0	0	0	0
SAL_DA2279AA	FDA00007941	Derby	0	0	0	0	0	0	0	0	0	0	0
SAL_DA1480AA	FDA00000150	Derby	0	0	0	0	0	0	0	0	0	0	0
SAL_DA1079AA	H133220765	Derby	0	0	0	0	0	0	0	0	0	0	0
SAL_DA0983AA	H121420373	Derby	0	0	0	0	0	0	0	0	0	0	0
SAL_DA0932AA	H122800357	Derby	0	0	0	0	0	0	0	0	0	0	0
SAL_DA0695AA	H124760756	Derby	0	0	0	0	0	0	0	0	0	0	0
SAL_DA0596AA	H123540655	Derby	0	0	0	0	0	0	0	0	0	0	0
SAL_DA0111AA	FDA00000769	Derby	0	0	0	0	0	0	0	0	0	0	0
SAL_CA8913AA	FDA00004830	Derby	0	0	0	0	0	0	0	0	0	0	0
SAL_CA8859AA	FDA00004828	Derby	0	0	0	0	0	0	0	0	0	0	0
SAL_CA8450AA	FL-NRM174	Derby	0	0	0	0	0	0	0	0	0	0	0
SAL_CA8272AA	CFSAN028001	Derby	0	0	0	0	0	0	0	0	0	0	0
SAL_CA8211AA	CFSAN028011	Derby	0	0	0	0	0	0	0	0	0	0	0
SAL_CA7639AA	FDA00004466	Derby	0	0	0	0	0	0	0	0	0	0	0
SAL_CA7228AA	HIY0305	Derby	0	0	0	0	0	0	0	0	0	0	0
SAL_CA7227AA	HIY0408	Derby	0	0	0	0	0	0	0	0	0	0	0
SAL_CA7226AA	HIY0006	Derby	0	0	0	0	0	0	0	0	0	0	0
SAL_CA7225AA	HIY0189	Derby	0	0	0	0	0	0	0	0	0	0	0
SAL_CA7224AA	HIY0359	Derby	0	0	0	0	0	0	0	0	0	0	0
SAL_CA7223AA	HIY0350	Derby	0	0	0	0	0	0	0	0	0	0	0

SAL_CA7222AA	HIY0046	Derby	0	0	0	0	0	0	0	0	0	0	0
SAL_CA7221AA	HIY0357	Derby	0	0	0	0	0	0	0	0	0	0	0
SAL_CA7216AA	HIY0023	Derby	0	0	0	0	0	0	0	0	0	0	0
SAL_CA7215AA	HIY0392	Derby	0	0	0	0	0	0	0	0	0	0	0
SAL_CA7214AA	HIY0394	Derby	0	0	0	0	0	0	0	0	0	0	0
SAL_CA7213AA	HIY0144	Derby	0	0	0	0	0	0	0	0	0	0	0
SAL_CA7211AA	HIY0089	Derby	0	0	0	0	0	0	0	0	0	0	0
SAL_CA7209AA	HIY0095	Derby	0	0	0	0	0	0	0	0	0	0	0
SAL_CA7212AA	HIY0252	Derby	0	0	0	0	0	0	0	0	0	0	0
SAL_CA7201AA	HIY0182	Derby	0	0	0	0	0	0	0	0	0	0	0
SAL_CA7204AA	HIY0016	Derby	0	0	0	0	0	0	0	0	0	0	0
SAL_CA7210AA	HIY0402	Derby	0	0	0	0	0	0	0	0	0	0	0
SAL_CA7197AA	HIY0360	Derby	0	0	0	0	0	0	0	0	0	0	0
SAL_CA7202AA	HIY0126	Derby	0	0	0	0	0	0	0	0	0	0	0
SAL_CA7203AA	HIY0031	Derby	0	0	0	0	0	0	0	0	0	0	0
SAL_CA7199AA	HIY0261	Derby	0	0	0	0	0	0	0	0	0	0	0
SAL_CA7200AA	HIY0036	Derby	0	0	0	0	0	0	0	0	0	0	0
SAL_CA7066AA	NY-N11232	Derby	0	0	0	0	0	0	0	0	0	0	0
SAL_CA7051AA	FDA00001106	Derby	0	0	0	0	0	0	0	0	0	0	0
SAL_CA7028AA	NY-N11233	Derby	0	0	0	0	0	0	0	0	0	0	0
SAL_CA6950AA	CFSAN006186	Derby	0	0	0	0	0	0	0	0	0	0	0
SAL_CA6850AA	44675	Derby	0	0	0	0	0	0	0	0	0	0	0
SAL_CA6676AA	60396	Derby	0	0	0	0	0	0	0	0	0	0	0
SAL_CA6665AA	50140	Derby	0	0	0	0	0	0	0	0	0	0	0
SAL_CA6637AA	54035	Derby	0	0	0	0	0	0	0	0	0	0	0
SAL_CA6285AA	46624	Derby	0	0	0	0	0	0	0	0	0	0	0
SAL_CA5451AA	71536	Derby	0	0	0	0	0	0	0	0	0	0	0
SAL_CA5042AA	73649	Derby	0	0	0	0	0	0	0	0	0	0	0
SAL_CA4562AA	97854	Derby	0	0	0	0	0	0	0	0	0	0	0
SAL_CA4287AA	7398	Derby	0	0	0	0	0	0	0	0	0	0	0
SAL_CA4246AA	25182	Derby	0	0	0	0	0	0	0	0	0	0	0
SAL_CA4126AA	69281	Derby	0	0	0	0	0	0	0	0	0	0	0
SAL_CA3447AA	87401	Derby	0	0	0	0	0	0	0	0	0	0	0
SAL_CA3038AA	98271	Derby	0	0	0	0	0	0	0	0	0	0	0
SAL_CA2765AA	67571	Derby	0	0	0	0	0	0	0	0	0	0	0
SAL_CA2689AA	65111	Derby	0	0	0	0	0	0	0	0	0	0	0
SAL_BA9972AA	75994	Derby	0	0	0	0	0	0	0	0	0	0	0
SAL_BA9543AA	9250	Derby	0	0	0	0	0	0	0	0	0	0	0
SAL_BA9287AA	CFSAN002746	Derby	0	0	0	0	0	0	0	0	0	0	0
SAL_BA9289AA	CFSAN002745	Derby	0	0	0	0	0	0	0	0	0	0	0
SAL_BA8924AA	80598	Derby	0	0	0	0	0	0	0	0	0	0	0
SAL_BA8594AA	CFSAN005657	Derby	0	0	0	0	0	0	0	0	0	0	0
SAL_BA8225AA	CFSAN005721	Derby	0	0	0	0	0	0	0	0	0	0	0
SAL_BA7360AA	NY-N13143	Derby	0	0	0	0	0	0	0	0	0	0	0
SAL_BA7335AA	CFSAN006202	Derby	0	0	0	0	0	0	0	0	0	0	0
SAL_BA6768AA	CFSAN006137	Derby	0	0	0	0	0	0	0	0	0	0	0
SAL_BA5834AA	M208 / NCTC20076	Derby	0	0	0	0	0	0	0	0	0	0	0
SAL_BA5092AA	CFSAN005669	Derby	0	0	0	0	0	0	0	0	0	0	0
SAL_BA5094AA	CFSAN005668	Derby	0	0	0	0	0	0	0	0	0	0	0
SAL_BA4685AA	CFSAN006231	Derby	0	0	0	0	0	0	0	0	0	0	0
SAL_BA4595AA	CFSAN029957	Derby	0	0	0	0	0	0	0	0	0	0	0
SAL_BA3454AA	NY-N14212	Derby	0	0	0	0	0	0	0	0	0	0	0
SAL_BA2826AA	dn171	Derby	0	0	0	0	0	0	0	0	0	0	0
SAL_BA2683AA	FDA00001237	Derby	0	0	0	0	0	0	0	0	0	0	0
SAL_BA2609AA	ADRDL-14-13894	Derby	0	0	0	0	0	0	0	0	0	0	0
SAL_BA2063AA	FDA00000902	Derby	0	0	0	0	0	0	0	0	0	0	0
SAL_BA2056AA	ADRDL-14-20140	Derby	0	0	0	0	0	0	0	0	0	0	0
SAL_BA2015AA	ADRDL-14-15465	Derby	0	0	0	0	0	0	0	0	0	0	0
SAL_BA1296AA	FDA00000938	Derby	0	0	0	0	0	0	0	0	0	0	0
SAL_BA0703AA	ADRDL-14-13593	Derby	0	0	0	0	0	0	0	0	0	0	0
SAL_AA9842AA	c-2013-10-08T14:25:43Z	Derby	0	0	0	0	0	0	0	0	0	0	0
SAL_AA9844AA	c-2013-10-08T14:25:40Z	Derby	0	0	0	0	0	0	0	0	0	0	0
SAL_AA9840AA	c-2013-10-08T14:25:45Z	Derby	0	0	0	0	0	0	0	0	0	0	0
SAL_AA9839AA	c-2013-10-08T14:25:46Z	Derby	0	0	0	0	0	0	0	0	0	0	0
SAL_AA9841AA	c-2013-10-08T14:25:44Z	Derby	0	0	0	0	0	0	0	0	0	0	0
SAL_AA9838AA	c-2013-10-08T14:25:47Z	Derby	0	0	0	0	0	0	0	0	0	0	0
SAL_AA9836AA	c-2013-10-08T14:25:49Z	Derby	0	0	0	0	0	0	0	0	0	0	0
SAL_AA9821AA	c-2013-10-08T14:25:54Z	Derby	0	0	0	0	0	0	0	0	0	0	0
SAL_AA9820AA	c-2013-10-08T14:25:55Z	Derby	0	0	0	0	0	0	0	0	0	0	0
SAL_AA9823AA	c-2013-10-08T14:25:52Z	Derby	0	0	0	0	0	0	0	0	0	0	0
SAL_AA9822AA	c-2013-10-08T14:25:53Z	Derby	0	0	0	0	0	0	0	0	0	0	0
SAL_AA9819AA	c-2013-10-08T14:25:59Z	Derby	0	0	0	0	0	0	0	0	0	0	0
SAL_AA9818AA	c-2013-10-08T14:26:00Z	Derby	0	0	0	0	0	0	0	0	0	0	0

SAL_AA9817AA	c-2013-10-08T14:26:01Z	Derby	0	0	0	0	0	0	0	0	0	0	0
SAL_AA9815AA	c-2013-10-08T14:26:04Z	Derby	0	0	0	0	0	0	0	0	0	0	0
SAL_AA9814AA	c-2013-10-08T14:26:05Z	Derby	0	0	0	0	0	0	0	0	0	0	0
SAL_AA9802AA	c-2013-10-08T14:26:06Z	Derby	0	0	0	0	0	0	0	0	0	0	0
SAL_AA9801AA	c-2013-10-08T14:26:07Z	Derby	0	0	0	0	0	0	0	0	0	0	0
SAL_AA9799AA	c-2013-10-08T14:26:09Z	Derby	0	0	0	0	0	0	0	0	0	0	0
SAL_AA9798AA	c-2013-10-08T14:26:10Z	Derby	0	0	0	0	0	0	0	0	0	0	0
SAL_AA9797AA	c-2013-10-08T14:26:11Z	Derby	0	0	0	0	0	0	0	0	0	0	0
SAL_AA9796AA	c-2013-10-08T14:26:12Z	Derby	0	0	0	0	0	0	0	0	0	0	0
SAL_AA9793AA	c-2013-10-08T14:26:16Z	Derby	0	0	0	0	0	0	0	0	0	0	0
SAL_AA9800AA	c-2013-10-08T14:26:08Z	Derby	0	0	0	0	0	0	0	0	0	0	0
SAL_FA6212AA	FSIS1606325	Derby	0	0	0	0	0	0	0	0	0	0	0
SAL_FA6184AA	FSIS1606394	Derby	0	0	0	0	0	0	0	0	0	0	0
SAL_FA6196AA	FSIS1606396	Derby	0	0	0	0	0	0	0	0	0	0	0
SAL_FA6251AA	CFSAN032108	Derby	0	0	0	0	0	0	0	0	0	0	0
SAL_FA6409AA	FDA00010162	Derby	0	0	0	0	0	0	0	0	0	0	0
SAL_FA6357AA	CFSAN045610	Derby	0	0	0	0	0	0	0	0	0	0	0
SAL_FA6437AA	PNUSAS001697	Derby	0	0	0	0	0	0	0	0	0	0	0
SAL_FA6336AA	FSIS1606503	Derby	0	0	0	0	0	0	0	0	0	0	0
SAL_FA6297AA	FSIS1606519	Derby	0	0	0	0	0	0	0	0	0	0	0
SAL_FA6795AA	FDA00010200	Derby	0	0	0	0	0	0	0	0	0	0	0
SAL_FA6794AA	FDA00010201	Derby	0	0	0	0	0	0	0	0	0	0	0
SAL_FA6796AA	FDA00010199	Derby	0	0	0	0	0	0	0	0	0	0	0
SAL_FA7166AA	Derby	Derby	0	0	0	0	0	0	0	0	0	0	0
SAL_FA7336AA	RKI_14-00470	Derby	0	0	0	0	0	0	0	0	0	0	0
SAL_FA7332AA	RKI_14-00117	Derby	0	0	0	0	0	0	0	0	0	0	0
SAL_FA7337AA	RKI_04-05621	Derby	0	0	0	0	0	0	0	0	0	0	0
SAL_FA7374AA	RKI_14-00651	Derby	0	0	0	0	0	0	0	0	0	0	0
SAL_FA7376AA	RKI_07-05911	Derby	0	0	0	0	0	0	0	0	0	0	0
SAL_FA7380AA	RKI_12-01254	Derby	0	0	0	0	0	0	0	0	0	0	0
SAL_FA7382AA	RKI_07-01167	Derby	0	0	0	0	0	0	0	0	0	0	0
SAL_FA7391AA	BfR_13-SA02767	Derby	0	0	0	0	0	0	0	0	0	0	0
SAL_FA7396AA	RKI_14-00426	Derby	0	0	0	0	0	0	0	0	0	0	0
SAL_FA7426AA	ADRDL15-26411	Derby	0	0	0	0	0	0	0	0	0	0	0
SAL_FA7567AA	RKI_07-02601	Derby	0	0	0	0	0	0	0	0	0	0	0
SAL_FA7570AA	RKI_07-04162	Derby	0	0	0	0	0	0	0	0	0	0	0
SAL_FA7579AA	FSIS1606699	Derby	0	0	0	0	0	0	0	0	0	0	0
SAL_FA7747AA	PNUSAS002235	Derby	0	0	0	0	0	0	0	0	0	0	0
SAL_FA7897AA	FSIS1606772	Derby	0	0	0	0	0	0	0	0	0	0	0
SAL_FA7903AA	FSIS1606766	Derby	0	0	0	0	0	0	0	0	0	0	0
SAL_FA8100AA	ADRDL15-27201	Derby	0	0	0	0	0	0	0	0	0	0	0
SAL_FA8226AA	CFSAN045632	Derby	0	0	0	0	0	0	0	0	0	0	0
SAL_FA8225AA	CFSAN045631	Derby	0	0	0	0	0	0	0	0	0	0	0
SAL_FA8224AA	CFSAN045630	Derby	0	0	0	0	0	0	0	0	0	0	0
SAL_FA8266AA	CFSAN045645	Derby	0	0	0	0	0	0	0	0	0	0	0
SAL_FA8228AA	FSIS1606957	Derby	0	0	0	0	0	0	0	0	0	0	0
SAL_FA7585AA	FSIS1606689	Derby	0	0	0	0	0	0	0	0	0	0	0
SAL_FA7297AA	FSIS1606571	Derby	0	0	0	0	0	0	0	0	0	0	0
SAL_FA7291AA	FSIS1606569	Derby	0	0	0	0	0	0	0	0	0	0	0
SAL_FA6932AA	FSIS1606625	Derby	0	0	0	0	0	0	0	0	0	0	0
SAL_FA9772AA	FSIS1502423	Derby	0	0	0	0	0	0	0	0	0	0	0
SAL_FA9753AA	FSIS1502420	Derby	0	0	0	0	0	0	0	0	0	0	0
SAL_GA4200AA	Sderby-0807T13477	Derby	0	0	0	0	0	0	0	0	0	0	0
SAL_GA4378AA	CVM-N23665	Derby	0	0	0	0	0	0	0	0	0	0	0
SAL_GA3719AA	BCW_2038	Derby	0	0	0	0	0	0	0	0	0	0	0
SAL_GA3639AA	BCW_2671	Derby	0	0	0	0	0	0	0	0	0	0	0
SAL_GA3638AA	BCW_2672	Derby	0	0	0	0	0	0	0	0	0	0	0
SAL_GA3637AA	BCW_2674	Derby	0	0	0	0	0	0	0	0	0	0	0
SAL_GA3520AA	BCW_2839	Derby	0	0	0	0	0	0	0	0	0	0	0
SAL_GA3479AA	BCW_2899	Derby	0	0	0	0	0	0	0	0	0	0	0
SAL_GA3417AA	BCW_2099	Derby	0	0	0	0	0	0	0	0	0	0	0
SAL_GA3394AA	CFSAN005825	Derby	0	0	0	0	0	0	0	0	0	0	0
SAL_GA3393AA	CFSAN005826	Derby	0	0	0	0	0	0	0	0	0	0	0
SAL_GA3391AA	CFSAN005828	Derby	0	0	0	0	0	0	0	0	0	0	0
SAL_GA3390AA	CFSAN005829	Derby	0	0	0	0	0	0	0	0	0	0	0
SAL_GA3392AA	CFSAN005827	Derby	0	0	0	0	0	0	0	0	0	0	0
SAL_GA3244AA	N26607	Derby	0	0	0	0	0	0	0	0	0	0	0
SAL_GA3204AA	BCW_2199	Derby	0	0	0	0	0	0	0	0	0	0	0
SAL_GA2971AA	Sente087	Derby	0	0	0	0	0	0	0	0	0	0	0
SAL_GA2889AA	CRJJGF_00022	Derby	0	0	0	0	0	0	0	0	0	0	0
SAL_GA2414AA	BCW_2001	Derby	0	0	0	0	0	0	0	0	0	0	0
SAL_GA2214AA	BCW_6222	Derby	0	0	0	0	0	0	0	0	0	0	0
SAL_GA2213AA	BCW_6223	Derby	0	0	0	0	0	0	0	0	0	0	0

SAL_GA2209AA	BCW_6227	Derby	0	0	0	0	0	0	0	0	0	0	0
SAL_GA2196AA	BCW_6240	Derby	0	0	0	0	0	0	0	0	0	0	0
SAL_GA2195AA	BCW_6241	Derby	0	0	0	0	0	0	0	0	0	0	0
SAL_GA2194AA	BCW_6242	Derby	0	0	0	0	0	0	0	0	0	0	0
SAL_GA2193AA	BCW_6243	Derby	0	0	0	0	0	0	0	0	0	0	0
SAL_GA2154AA	BCW_6283	Derby	0	0	0	0	0	0	0	0	0	0	0
SAL_GA2146AA	BCW_6291	Derby	0	0	0	0	0	0	0	0	0	0	0
SAL_GA2143AA	BCW_6294	Derby	0	0	0	0	0	0	0	0	0	0	0
SAL_GA2139AA	BCW_6298	Derby	0	0	0	0	0	0	0	0	0	0	0
SAL_GA2134AA	BCW_6303	Derby	0	0	0	0	0	0	0	0	0	0	0
SAL_GA1880AA	BCW_5047	Derby	0	0	0	0	0	0	0	0	0	0	0
SAL_GA1876AA	BCW_5051	Derby	0	0	0	0	0	0	0	0	0	0	0
SAL_GA1779AA	BCW_2053	Derby	0	0	0	0	0	0	0	0	0	0	0
SAL_GA1748AA	N37937	Derby	0	0	0	0	0	0	0	0	0	0	0
SAL_GA1716AA	BCW_2673	Derby	0	0	0	0	0	0	0	0	0	0	0
SAL_GA1617AA	07CR553	Derby	0	0	0	0	0	0	0	0	0	0	0
SAL_GA1299AA	3232_07-sc-2140689	Derby	0	0	0	0	0	0	0	0	0	0	0
SAL_GA0370AA	VNS10211-sc-2280567	Derby	0	0	0	0	0	0	0	0	0	0	0
SAL_GA0357AA	VNS30150-sc-2280600	Derby	0	0	0	0	0	0	0	0	0	0	0
SAL_GA0349AA	VNSC2510-sc-2280614	Derby	0	0	0	0	0	0	0	0	0	0	0
SAL_GA0341AA	NS153THL_GB-sc-228063	Derby	0	0	0	0	0	0	0	0	0	0	0
SAL_GA0340AA	NS153THL_SPP-sc-22806	Derby	0	0	0	0	0	0	0	0	0	0	0
SAL_GA0328AA	71_G_199-sc-2280657	Derby	0	0	0	0	0	0	0	0	0	0	0
SAL_GA0318AA	71_H_072-sc-2280678	Derby	0	0	0	0	0	0	0	0	0	0	0
SAL_GA0317AA	71_H_103-sc-2280680	Derby	0	0	0	0	0	0	0	0	0	0	0
SAL_GA0311AA	71_H_239-sc-2280694	Derby	0	0	0	0	0	0	0	0	0	0	0
SAL_GA0310AA	71_H_287-sc-2280696	Derby	0	0	0	0	0	0	0	0	0	0	0
SAL_GA0307AA	71_H_363-sc-2280701	Derby	0	0	0	0	0	0	0	0	0	0	0
SAL_GA0306AA	71_H_379-sc-2280703	Derby	0	0	0	0	0	0	0	0	0	0	0
SAL_GA0299AA	71_V_222-sc-2280716	Derby	0	0	0	0	0	0	0	0	0	0	0
SAL_GA0284AA	72_H_297-sc-2280736	Derby	0	0	0	0	0	0	0	0	0	0	0
SAL_GA0256AA	CG01C_1_1-sc-2280765	Derby	0	0	0	0	0	0	0	0	0	0	0
SAL_GA0243AA	CG61R-sc-2280778	Derby	0	0	0	0	0	0	0	0	0	0	0
SAL_GA0240AA	CT09C_1_1-sc-2280781	Derby	0	0	0	0	0	0	0	0	0	0	0
SAL_GA0180AA	2125-sc-2280559	Derby	0	0	0	0	0	0	0	0	0	0	0
SAL_FA9716AA	FDA00000289	Derby	0	0	0	0	0	0	0	0	0	0	0
SAL_GA4908AA	FSIS1607133	Derby	0	0	0	0	0	0	0	0	0	0	0
SAL_GA4814AA	PNUSAS001708	Derby	0	0	0	0	0	0	0	0	0	0	0
SAL_GA4691AA	FSIS1606974	Derby	0	0	0	0	0	0	0	0	0	0	0
SAL_FA9270AA	CFSAN047006	Derby	0	0	0	0	0	0	0	0	0	0	0
SAL_FA9261AA	CFSAN047015	Derby	0	0	0	0	0	0	0	0	0	0	0
SAL_GA5204AA	V-CLASP-D-18	Derby	0	0	0	0	0	0	0	0	0	0	0
SAL_GA5715AA	CVM N58633	Derby	0	0	0	0	0	0	0	0	0	0	0
SAL_GA5702AA	CVM N58646	Derby	0	0	0	0	0	0	0	0	0	0	0
SAL_GA5662AA	CVM N59324	Derby	0	0	0	0	0	0	0	0	0	0	0
SAL_GA5609AA	CVM N62493	Derby	0	0	0	0	0	0	0	0	0	0	0
SAL_GA5259AA	FDA00004475	Derby	0	0	0	0	0	0	0	0	0	0	0
SAL_FA9070AA	CVM N40386	Derby	0	0	0	0	0	0	0	0	0	0	0
SAL_FA8874AA	CVM N42507	Derby	0	0	0	0	0	0	0	0	0	0	0
SAL_FA8725AA	CVM N30679	Derby	0	0	0	0	0	0	0	0	0	0	0
SAL_FA8699AA	CVM N30705	Derby	0	0	0	0	0	0	0	0	0	0	0
SAL_GA5951AA	FSIS1607163	Derby	0	0	0	0	0	0	0	0	0	0	0
SAL_GA5948AA	FSIS1607157	Derby	0	0	0	0	0	0	0	0	0	0	0
SAL_GA6310AA	FDA00001741	Derby	0	0	0	0	0	0	0	0	0	0	0
SAL_GA6303AA	FDA00001803	Derby	0	0	0	0	0	0	0	0	0	0	0
SAL_GA6260AA	FSIS1607284	Derby	0	0	0	0	0	0	0	0	0	0	0
SAL_GA6875AA	FSIS1607300	Derby	0	0	0	0	0	0	0	0	0	0	0
SAL_GA7447AA	FSIS1607326	Derby	0	0	0	0	0	0	0	0	0	0	0
SAL_GA7372AA	FDA00004749	Derby	0	0	0	0	0	0	0	0	0	0	0
SAL_GA7449AA	14S003	Derby	0	0	0	0	0	0	0	0	0	0	0
SAL_GA7483AA	PNUSAS003279	Derby	0	0	0	0	0	0	0	0	0	0	0
SAL_GA7947AA	2014AM-1155	Derby	0	0	0	0	0	0	0	0	0	0	0
SAL_GA7955AA	2014AM-2972	Derby	0	0	0	0	0	0	0	0	0	0	0
SAL_GA7963AA	2014AM-0431	Derby	0	0	0	0	0	0	0	0	0	0	0
SAL_GA7750AA	FSIS1607396	Derby	0	0	0	0	0	0	0	0	0	0	0
SAL_GA7776AA	FSIS1607347	Derby	0	0	0	0	0	0	0	0	0	0	0
SAL_GA8156AA	ADRDL15-24545	Derby	0	0	0	0	0	0	0	0	0	0	0
SAL_GA8040AA	FDA00009800	Derby	0	0	0	0	0	0	0	0	0	0	0
SAL_GA8690AA	ADRDL 16-4242	Derby	0	0	0	0	0	0	0	0	0	0	0
SAL_GA9055AA	ADRDL_R120	Derby	0	0	0	0	0	0	0	0	0	0	0
SAL_GA9313AA	FSIS1607545	Derby	0	0	0	0	0	0	0	0	0	0	0
SAL_GA9330AA	SH36	Derby	0	0	0	0	0	0	0	0	0	0	0
SAL_GA9429AA	PNUSAS003780	Derby	0	0	0	0	0	0	0	0	0	0	0

SAL_GA9538AA	CVM N58682	Derby	0	0	0	0	0	0	0	0	0	0	0
SAL_GA9456AA	FSIS1607659	Derby	0	0	0	0	0	0	0	0	0	0	0
SAL_GA9490AA	CVM N62951	Derby	0	0	0	0	0	0	0	0	0	0	0
SAL_GA9997AA	PNUSAS003899	Derby	0	0	0	0	0	0	0	0	0	0	0
SAL_HA0093AA	PNUSAS003900	Derby	0	0	0	0	0	0	0	0	0	0	0
SAL_HA0251AA	PNUSAS003896	Derby	0	0	0	0	0	0	0	0	0	0	0
SAL_HA0656AA	2014AM-2506	Derby	0	0	0	0	0	0	0	0	0	0	0
SAL_HA0657AA	2014AM-2649	Derby	0	0	0	0	0	0	0	0	0	0	0
SAL_HA0621AA	2014AM-1683	Derby	0	0	0	0	0	0	0	0	0	0	0
SAL_HA1147AA	ADRDL-R238	Derby	0	0	0	0	0	0	0	0	0	0	0
SAL_HA1268AA	PNUSAS004667	Derby	0	0	0	0	0	0	0	0	0	0	0
SAL_HA1306AA	FSIS1607970	Derby	0	0	0	0	0	0	0	0	0	0	0
SAL_HA1523AA	ADRDL-261	Derby	0	0	0	0	0	0	0	0	0	0	0
SAL_HA1754AA	FSIS1607787	Derby	0	0	0	0	0	0	0	0	0	0	0
SAL_HA1751AA	FSIS1608053	Derby	0	0	0	0	0	0	0	0	0	0	0
SAL_HA2949AA	FSIS1608102	Derby	0	0	0	0	0	0	0	0	0	0	0
SAL_HA3108AA	FSIS1608273	Derby	0	0	0	0	0	0	0	0	0	0	0
SAL_HA2854AA	FSIS1608122	Derby	0	0	0	0	0	0	0	0	0	0	0
SAL_HA2798AA	ADRDL-16-6332	Derby	0	0	0	0	0	0	0	0	0	0	0
SAL_HA2768AA	ADRDL-16-10508	Derby	0	0	0	0	0	0	0	0	0	0	0
SAL_HA2143AA	S00665-03	Derby	0	0	0	0	0	0	0	0	0	0	0
SAL_HA2119AA	S00369-05	Derby	0	0	0	0	0	0	0	0	0	0	0
SAL_HA2127AA	L02054-05	Derby	0	0	0	0	0	0	0	0	0	0	0
SAL_HA2131AA	S00027-04	Derby	0	0	0	0	0	0	0	0	0	0	0
SAL_HA2138AA	S06791-03	Derby	0	0	0	0	0	0	0	0	0	0	0
SAL_HA2144AA	S00601-03	Derby	0	0	0	0	0	0	0	0	0	0	0
SAL_HA2094AA	S05879-07	Derby	0	0	0	0	0	0	0	0	0	0	0
SAL_HA2100AA	S06372-06	Derby	0	0	0	0	0	0	0	0	0	0	0
SAL_HA2106AA	S01058-06	Derby	0	0	0	0	0	0	0	0	0	0	0
SAL_HA2105AA	S02455-06	Derby	0	0	0	0	0	0	0	0	0	0	0
SAL_HA2107AA	S00094-06	Derby	0	0	0	0	0	0	0	0	0	0	0
SAL_HA2046AA	S04599-10	Derby	0	0	0	0	0	0	0	0	0	0	0
SAL_HA2048AA	S01732-10	Derby	0	0	0	0	0	0	0	0	0	0	0
SAL_HA2049AA	S01346-10	Derby	0	0	0	0	0	0	0	0	0	0	0
SAL_HA2029AA	S03692-11	Derby	0	0	0	0	0	0	0	0	0	0	0
SAL_HA2032AA	S01956-11	Derby	0	0	0	0	0	0	0	0	0	0	0
SAL_HA2034AA	S01642-11	Derby	0	0	0	0	0	0	0	0	0	0	0
SAL_HA1994AA	S06467-12	Derby	0	0	0	0	0	0	0	0	0	0	0
SAL_HA4334AA	CFSAN048490	Derby	0	0	0	0	0	0	0	0	0	0	0
SAL_HA4318AA	CFSAN048479	Derby	0	0	0	0	0	0	0	0	0	0	0
SAL_HA4634AA	FSIS1608483	Derby	0	0	0	0	0	0	0	0	0	0	0
SAL_HA5119AA	L00933-14	Derby	0	0	0	0	0	0	0	0	0	0	0
SAL_HA5124AA	L00670-14	Derby	0	0	0	0	0	0	0	0	0	0	0
SAL_HA5376AA	CFSAN048496	Derby	0	0	0	0	0	0	0	0	0	0	0
SAL_HA5379AA	ARIM-SDER124-08	Derby	0	0	0	0	0	0	0	0	0	0	0
SAL_HA2704AA	FSIS1608254	Derby	0	0	0	0	0	0	0	0	0	0	0
SAL_HA2706AA	FSIS1608253	Derby	0	0	0	0	0	0	0	0	0	0	0
SAL_HA2663AA	FDA00000312	Derby	0	0	0	0	0	0	0	0	0	0	0
SAL_HA6581AA	FSIS1608420	Derby	0	0	0	0	0	0	0	0	0	0	0
SAL_HA5968AA	CFSAN056584	Derby	0	0	0	0	0	0	0	0	0	0	0
SAL_HA6075AA	FDA00001926	Derby	0	0	0	0	0	0	0	0	0	0	0
SAL_HA6067AA	FDA00001100	Derby	0	0	0	0	0	0	0	0	0	0	0
SAL_HA6804AA	PNUSAS005618	Derby	0	0	0	0	0	0	0	0	0	0	0
SAL_HA6305AA	FSIS1608827	Derby	0	0	0	0	0	0	0	0	0	0	0
SAL_HA6301AA	FSIS1608829	Derby	0	0	0	0	0	0	0	0	0	0	0
SAL_HA7611AA	CFSAN056823	Derby	0	0	0	0	0	0	0	0	0	0	0
SAL_HA7643AA	FSIS1609219	Derby	0	0	0	0	0	0	0	0	0	0	0
SAL_HA7645AA	FSIS1609213	Derby	0	0	0	0	0	0	0	0	0	0	0
SAL_HA8268AA	PNUSAS006494	Derby	0	0	0	0	0	0	0	0	0	0	0
SAL_HA9115AA	CFSAN058108	Derby	0	0	0	0	0	0	0	0	0	0	0
SAL_HA9589AA	PNUSAS006794	Derby	0	0	0	0	0	0	0	0	0	0	0
SAL_HA9539AA	PNUSAS006528	Derby	0	0	0	0	0	0	0	0	0	0	0
SAL_HA9601AA	FSIS1609345	Derby	0	0	0	0	0	0	0	0	0	0	0
SAL_IA0099AA	0-3918-11e6-bcc0-3c4a9	Derby	0	0	0	0	0	0	0	0	0	0	0
SAL_IA0255AA	FSIS1609721	Derby	0	0	0	0	0	0	0	0	0	0	0
SAL_IA0571AA	PNUSAS007092	Derby	0	0	0	0	0	0	0	0	0	0	0
SAL_IA1394AA	330501	Derby	0	0	0	0	0	0	0	0	0	0	0
SAL_IA2019AA	FSIS1609723	Derby	0	0	0	0	0	0	0	0	0	0	0
SAL_IA1996AA	FSIS1709804	Derby	0	0	0	0	0	0	0	0	0	0	0
SAL_IA1980AA	FSIS1609732	Derby	0	0	0	0	0	0	0	0	0	0	0
SAL_IA2285AA	FSIS1609744	Derby	0	0	0	0	0	0	0	0	0	0	0
SAL_IA2205AA	FSIS1609716	Derby	0	0	0	0	0	0	0	0	0	0	0
SAL_IA2208AA	FSIS1609719	Derby	0	0	0	0	0	0	0	0	0	0	0

SAL_IA2095AA	FSIS1609666	Derby	0	0	0	0	0	0	0	0	0	0	0
SAL_IA2365AA	FSIS1609398	Derby	0	0	0	0	0	0	0	0	0	0	0
SAL_IA2321AA	FSIS1709855	Derby	0	0	0	0	0	0	0	0	0	0	0
SAL_IA2325AA	FSIS1709856	Derby	0	0	0	0	0	0	0	0	0	0	0
SAL_IA2473AA	CFSAN029956	Derby	0	0	0	0	0	0	0	0	0	0	0
SAL_IA3355AA	329908	Derby	0	0	0	0	0	0	0	0	0	0	0
SAL_IA2695AA	CFSAN059205	Derby	0	0	0	0	0	0	0	0	0	0	0
SAL_IA2635AA	CFSAN059246	Derby	0	0	0	0	0	0	0	0	0	0	0
SAL_IA4874AA	FSIS1710218	Derby	0	0	0	0	0	0	0	0	0	0	0
SAL_IA4866AA	FSIS1710169	Derby	0	0	0	0	0	0	0	0	0	0	0
SAL_IA4922AA	CFSAN059298	Derby	0	0	0	0	0	0	0	0	0	0	0
SAL_IA4934AA	CFSAN059304	Derby	0	0	0	0	0	0	0	0	0	0	0
SAL_IA5103AA	FDA00011215	Derby	0	0	0	0	0	0	0	0	0	0	0
SAL_IA4936AA	CFSAN059305	Derby	0	0	0	0	0	0	0	0	0	0	0
SAL_IA5338AA	FSIS1710224	Derby	0	0	0	0	0	0	0	0	0	0	0
SAL_IA5543AA	FSIS1710244	Derby	0	0	0	0	0	0	0	0	0	0	0
SAL_IA5532AA	CFSAN059821	Derby	0	0	0	0	0	0	0	0	0	0	0
SAL_IA5476AA	FSIS1710196	Derby	0	0	0	0	0	0	0	0	0	0	0
SAL_IA5485AA	FSIS1709973	Derby	0	0	0	0	0	0	0	0	0	0	0
SAL_IA5472AA	FSIS1710178	Derby	0	0	0	0	0	0	0	0	0	0	0
SAL_IA6491AA	PNUSAS008100	Derby	0	0	0	0	0	0	0	0	0	0	0
SAL_IA6484AA	PNUSAS008325	Derby	0	0	0	0	0	0	0	0	0	0	0
SAL_IA6620AA	FSIS1710371	Derby	0	0	0	0	0	0	0	0	0	0	0
SAL_IA6618AA	FSIS1710346	Derby	0	0	0	0	0	0	0	0	0	0	0
SAL_IA6962AA	MS150111	Derby	0	0	0	0	0	0	0	0	0	0	0
SAL_IA6683AA	FSIS1710257	Derby	0	0	0	0	0	0	0	0	0	0	0
SAL_IA7239AA	PNUSAS008389	Derby	0	0	0	0	0	0	0	0	0	0	0
SAL_IA7097AA	CFSAN059822	Derby	0	0	0	0	0	0	0	0	0	0	0
SAL_IA7564AA	FSIS1710550	Derby	0	0	0	0	0	0	0	0	0	0	0
SAL_IA7543AA	FSIS1710547	Derby	0	0	0	0	0	0	0	0	0	0	0
SAL_IA7667AA	FSIS1710592	Derby	0	0	0	0	0	0	0	0	0	0	0
SAL_IA7664AA	FSIS1710583	Derby	0	0	0	0	0	0	0	0	0	0	0
SAL_IA7724AA	S/20170065	Derby	0	0	0	0	0	0	0	0	0	0	0
SAL_IA7787AA	HIY0012	Derby	0	0	0	0	0	0	0	0	0	0	0
SAL_IA7792AA	HIY0018	Derby	0	0	0	0	0	0	0	0	0	0	0
SAL_IA7788AA	HIY0017	Derby	0	0	0	0	0	0	0	0	0	0	0
SAL_IA8868AA	PNUSAS008350	Derby	0	0	0	0	0	0	0	0	0	0	0
SAL_IA8853AA	PNUSAS008378	Derby	0	0	0	0	0	0	0	0	0	0	0
SAL_IA8847AA	PNUSAS008379	Derby	0	0	0	0	0	0	0	0	0	0	0
SAL_IA8993AA	FSIS1710849	Derby	0	0	0	0	0	0	0	0	0	0	0
SAL_IA9004AA	FSIS1710853	Derby	0	0	0	0	0	0	0	0	0	0	0
SAL_IA8995AA	FSIS1710861	Derby	0	0	0	0	0	0	0	0	0	0	0
SAL_IA9140AA	PNUSAS009249	Derby	0	0	0	0	0	0	0	0	0	0	0
SAL_IA9780AA	FSIS1711036	Derby	0	0	0	0	0	0	0	0	0	0	0
SAL_IA9773AA	FSIS1711021	Derby	0	0	0	0	0	0	0	0	0	0	0
SAL_IA9781AA	FSIS1711026	Derby	0	0	0	0	0	0	0	0	0	0	0
SAL_IA9794AA	FSIS1700047	Derby	0	0	0	0	0	0	0	0	0	0	0
SAL_IA9734AA	FSIS1710556	Derby	0	0	0	0	0	0	0	0	0	0	0
SAL_IA9763AA	FSIS1710939	Derby	0	0	0	0	0	0	0	0	0	0	0
SAL_JA0005AA	HIY0020	Derby	0	0	0	0	0	0	0	0	0	0	0
SAL_JA0047AA	FSIS1710872	Derby	0	0	0	0	0	0	0	0	0	0	0
SAL_JA0036AA	FSIS1710870	Derby	0	0	0	0	0	0	0	0	0	0	0
SAL_JA0123AA	PNUSAS010079	Derby	0	0	0	0	0	0	0	0	0	0	0
SAL_JA0596AA	NC_NCF5D0-L4	Derby	0	0	0	0	0	0	0	0	0	0	0
SAL_JA0612AA	NC_NCF5D0-A4	Derby	0	0	0	0	0	0	0	0	0	0	0
SAL_JA1386AA	283182	Derby	0	0	0	0	0	0	0	0	0	0	0
SAL_JA1350AA	267755	Derby	0	0	0	0	0	0	0	0	0	0	0
SAL_JA1374AA	212745	Derby	0	0	0	0	0	0	0	0	0	0	0
SAL_JA1545AA	PNUSAS008911	Derby	0	0	0	0	0	0	0	0	0	0	0
SAL_JA1611AA	17011617	Derby	0	0	0	0	0	0	0	0	0	0	0
SAL_JA1358AA	163120	Derby	0	0	0	0	0	0	0	0	0	0	0
SAL_JA1364AA	271333	Derby	0	0	0	0	0	0	0	0	0	0	0
SAL_JA1367AA	205615	Derby	0	0	0	0	0	0	0	0	0	0	0
SAL_JA1366AA	109911	Derby	0	0	0	0	0	0	0	0	0	0	0
SAL_JA1370AA	273570	Derby	0	0	0	0	0	0	0	0	0	0	0
SAL_JA1385AA	232778	Derby	0	0	0	0	0	0	0	0	0	0	0
SAL_JA1389AA	301659	Derby	0	0	0	0	0	0	0	0	0	0	0
SAL_JA1388AA	190645	Derby	0	0	0	0	0	0	0	0	0	0	0
SAL_JA1391AA	260043	Derby	0	0	0	0	0	0	0	0	0	0	0
SAL_JA1390AA	152469	Derby	0	0	0	0	0	0	0	0	0	0	0
SAL_JA1393AA	169633	Derby	0	0	0	0	0	0	0	0	0	0	0
SAL_JA1395AA	260046	Derby	0	0	0	0	0	0	0	0	0	0	0
SAL_JA1404AA	120576	Derby	0	0	0	0	0	0	0	0	0	0	0

SAL_JA1402AA	243841	Derby	0	0	0	0	0	0	0	0	0	0	0
SAL_JA1401AA	173552	Derby	0	0	0	0	0	0	0	0	0	0	0
SAL_JA1408AA	341373	Derby	0	0	0	0	0	0	0	0	0	0	0
SAL_JA1405AA	198252	Derby	0	0	0	0	0	0	0	0	0	0	0
SAL_JA1403AA	322283	Derby	0	0	0	0	0	0	0	0	0	0	0
SAL_JA1407AA	261432	Derby	0	0	0	0	0	0	0	0	0	0	0
SAL_JA1412AA	227035	Derby	0	0	0	0	0	0	0	0	0	0	0
SAL_JA1362AA	302890	Derby	0	0	0	0	0	0	0	0	0	0	0
SAL_JA1363AA	262734	Derby	0	0	0	0	0	0	0	0	0	0	0
SAL_JA1347AA	115262	Derby	0	0	0	0	0	0	0	0	0	0	0
SAL_JA1327AA	330531	Derby	0	0	0	0	0	0	0	0	0	0	0
SAL_JA1326AA	265324	Derby	0	0	0	0	0	0	0	0	0	0	0
SAL_JA1325AA	289056	Derby	0	0	0	0	0	0	0	0	0	0	0
SAL_JA1328AA	173721	Derby	0	0	0	0	0	0	0	0	0	0	0
SAL_JA1331AA	170294	Derby	0	0	0	0	0	0	0	0	0	0	0
SAL_JA1329AA	140702	Derby	0	0	0	0	0	0	0	0	0	0	0
SAL_JA1330AA	265285	Derby	0	0	0	0	0	0	0	0	0	0	0
SAL_JA1333AA	178848	Derby	0	0	0	0	0	0	0	0	0	0	0
SAL_JA1319AA	221652	Derby	0	0	0	0	0	0	0	0	0	0	0
SAL_JA1335AA	171284	Derby	0	0	0	0	0	0	0	0	0	0	0
SAL_JA1334AA	223521	Derby	0	0	0	0	0	0	0	0	0	0	0
SAL_JA1337AA	168342	Derby	0	0	0	0	0	0	0	0	0	0	0
SAL_JA1279AA	103725	Derby	0	0	0	0	0	0	0	0	0	0	0
SAL_JA1312AA	164936	Derby	0	0	0	0	0	0	0	0	0	0	0
SAL_JA1323AA	129502	Derby	0	0	0	0	0	0	0	0	0	0	0
SAL_JA1322AA	282430	Derby	0	0	0	0	0	0	0	0	0	0	0
SAL_JA0458AA	FSIS1710981	Derby	0	0	0	0	0	0	0	0	0	0	0
SAL_JA0355AA	PNUSAS009775	Derby	0	0	0	0	0	0	0	0	0	0	0
SAL_JA0344AA	PNUSAS010201	Derby	0	0	0	0	0	0	0	0	0	0	0
SAL_JA1673AA	CFSAN045629	Derby	0	0	0	0	0	0	0	0	0	0	0
SAL_JA1670AA	CFSAN045588	Derby	0	0	0	0	0	0	0	0	0	0	0
SAL_JA1671AA	CFSAN045593	Derby	0	0	0	0	0	0	0	0	0	0	0
SAL_JA1921AA	PNUSAS011410	Derby	0	0	0	0	0	0	0	0	0	0	0
SAL_JA1855AA	FSIS1710212	Derby	0	0	0	0	0	0	0	0	0	0	0
SAL_JA2158AA	FSIS1700018	Derby	0	0	0	0	0	0	0	0	0	0	0
SAL_JA2028AA	PNUSAS011368	Derby	0	0	0	0	0	0	0	0	0	0	0
SAL_JA2337AA	HIY0058	Derby	0	0	0	0	0	0	0	0	0	0	0
SAL_JA2273AA	FSIS1700245	Derby	0	0	0	0	0	0	0	0	0	0	0
SAL_JA2367AA	FSIS1710660	Derby	0	0	0	0	0	0	0	0	0	0	0
SAL_JA2363AA	FSIS1710659	Derby	0	0	0	0	0	0	0	0	0	0	0
SAL_JA2564AA	PNUSAS011759	Derby	0	0	0	0	0	0	0	0	0	0	0
SAL_JA2796AA	FSIS1710875	Derby	0	0	0	0	0	0	0	0	0	0	0
SAL_JA2786AA	HIY0032	Derby	0	0	0	0	0	0	0	0	0	0	0
SAL_JA2788AA	HIY0039	Derby	0	0	0	0	0	0	0	0	0	0	0
SAL_JA2833AA	FSIS1700229	Derby	0	0	0	0	0	0	0	0	0	0	0
SAL_JA3411AA	FSIS1700527	Derby	0	0	0	0	0	0	0	0	0	0	0
SAL_JA3674AA	FSIS1700364	Derby	0	0	0	0	0	0	0	0	0	0	0
SAL_JA3629AA	SARB11	Derby	0	0	0	0	0	0	0	0	0	0	0
SAL_JA3662AA	FSIS1700353	Derby	0	0	0	0	0	0	0	0	0	0	0
SAL_JA3690AA	FSIS1700611	Derby	0	0	0	0	0	0	0	0	0	0	0
SAL_JA3627AA	SARB10	Derby	0	0	0	0	0	0	0	0	0	0	0
SAL_JA4190AA	FSIS1700945	Derby	0	0	0	0	0	0	0	0	0	0	0
SAL_JA4201AA	FSIS1700948	Derby	0	0	0	0	0	0	0	0	0	0	0
SAL_JA5030AA	FSIS1701129	Derby	0	0	0	0	0	0	0	0	0	0	0
SAL_JA4972AA	PNUSAS013535	Derby	0	0	0	0	0	0	0	0	0	0	0
SAL_JA4891AA	PNUSAS006004	Derby	0	0	0	0	0	0	0	0	0	0	0
SAL_JA5017AA	FSIS1701138	Derby	0	0	0	0	0	0	0	0	0	0	0
SAL_JA6375AA	FSIS1701020	Derby	0	0	0	0	0	0	0	0	0	0	0
SAL_JA6181AA	PNUSAS012686	Derby	0	0	0	0	0	0	0	0	0	0	0
SAL_JA6038AA	FSIS1700782	Derby	0	0	0	0	0	0	0	0	0	0	0
SAL_JA5621AA	PNUSAS013581	Derby	0	0	0	0	0	0	0	0	0	0	0
SAL_JA6661AA	PNUSAS014108	Derby	0	0	0	0	0	0	0	0	0	0	0
SAL_JA6617AA	FSIS1701212	Derby	0	0	0	0	0	0	0	0	0	0	0
SAL_JA6938AA	FSIS1701299	Derby	0	0	0	0	0	0	0	0	0	0	0
SAL_JA7185AA	PNUSAS014465	Derby	0	0	0	0	0	0	0	0	0	0	0
SAL_JA7052AA	PNUSAS013750	Derby	0	0	0	0	0	0	0	0	0	0	0
SAL_JA7505AA	bench5	Derby	0	0	0	0	0	0	0	0	0	0	0
SAL_JA7482AA	FSIS1700793	Derby	0	0	0	0	0	0	0	0	0	0	0
SAL_JA8582AA	FSIS1701087	Derby	0	0	0	0	0	0	0	0	0	0	0
SAL_JA8523AA	FSIS1700777	Derby	0	0	0	0	0	0	0	0	0	0	0
SAL_JA4661AA	CVM 43779	Derby	0	0	0	0	0	0	0	0	0	0	0
SAL_JA9512AA	PNUSAS011782	Derby	0	0	0	0	0	0	0	0	0	0	0
SAL_JA9201AA	FSIS1701558	Derby	0	0	0	0	0	0	0	0	0	0	0

SAL_JA9818AA	FSIS1701590	Derby	0	0	0	0	0	0	0	0	0	0	0
SAL_KA0199AA	E2016008491	Derby	0	0	0	0	0	0	0	0	0	0	0
SAL_KA0203AA	E2016008489	Derby	0	0	0	0	0	0	0	0	0	0	0
SAL_JA9810AA	FSIS1701603	Derby	0	0	0	0	0	0	0	0	0	0	0
SAL_KA0493AA	PNUSAS016386	Derby	0	0	0	0	0	0	0	0	0	0	0
SAL_KA0879AA	PNUSAS016260	Derby	0	0	0	0	0	0	0	0	0	0	0
SAL_KA0285AA	FSIS1701812	Derby	0	0	0	0	0	0	0	0	0	0	0
SAL_KA0870AA	PNUSAS016257	Derby	0	0	0	0	0	0	0	0	0	0	0
SAL_KA0873AA	PNUSAS016259	Derby	0	0	0	0	0	0	0	0	0	0	0
SAL_KA1030AA	FSIS1702044	Derby	0	0	0	0	0	0	0	0	0	0	0
SAL_KA1033AA	FSIS1702046	Derby	0	0	0	0	0	0	0	0	0	0	0
SAL_KA0930AA	PNUSAS015860	Derby	0	0	0	0	0	0	0	0	0	0	0
SAL_KA1616AA	FSIS1702131	Derby	0	0	0	0	0	0	0	0	0	0	0
SAL_KA1610AA	FSIS1702130	Derby	0	0	0	0	0	0	0	0	0	0	0
SAL_KA1790AA	PNUSAS015538	Derby	0	0	0	0	0	0	0	0	0	0	0
SAL_KA2046AA	FSIS1701900	Derby	0	0	0	0	0	0	0	0	0	0	0
SAL_KA2048AA	FSIS1701901	Derby	0	0	0	0	0	0	0	0	0	0	0
SAL_KA2129AA	FSIS1701998	Derby	0	0	0	0	0	0	0	0	0	0	0
SAL_KA2960AA	FSIS1701886	Derby	0	0	0	0	0	0	0	0	0	0	0
SAL_KA2970AA	FSIS1701898	Derby	0	0	0	0	0	0	0	0	0	0	0
SAL_KA3482AA	FSIS1702601	Derby	0	0	0	0	0	0	0	0	0	0	0
SAL_KA3436AA	PNUSAS017502	Derby	0	0	0	0	0	0	0	0	0	0	0
SAL_KA3582AA	FSIS1702518	Derby	0	0	0	0	0	0	0	0	0	0	0
SAL_KA3715AA	FSIS1702805	Derby	0	0	0	0	0	0	0	0	0	0	0
SAL_KA3700AA	FSIS1702521	Derby	0	0	0	0	0	0	0	0	0	0	0
SAL_KA3711AA	FSIS1702799	Derby	0	0	0	0	0	0	0	0	0	0	0
SAL_KA3691AA	FSIS1701800	Derby	0	0	0	0	0	0	0	0	0	0	0
SAL_KA4828AA	FSIS1702775	Derby	0	0	0	0	0	0	0	0	0	0	0
SAL_KA4830AA	FSIS1702822	Derby	0	0	0	0	0	0	0	0	0	0	0
SAL_KA4832AA	FSIS1702823	Derby	0	0	0	0	0	0	0	0	0	0	0
SAL_KA4615AA	PNUSAS015991	Derby	0	0	0	0	0	0	0	0	0	0	0
SAL_KA4296AA	SF_R0374/09	Derby	0	0	0	0	0	0	0	0	0	0	0
SAL_KA8657AA	SARB9 (Fidelma Boyd)	Derby	0	0	0	0	0	0	0	0	0	0	0
SAL_KA5072AA	FSIS1702816	Derby	0	0	0	0	0	0	0	0	0	0	0
SAL_KA5548AA	FSIS1703076	Derby	0	0	0	0	0	0	0	0	0	0	0
SAL_KA5591AA	FSIS1702854	Derby	0	0	0	0	0	0	0	0	0	0	0
SAL_KA5738AA	FSIS1703085	Derby	0	0	0	0	0	0	0	0	0	0	0
SAL_KA5719AA	FSIS1703081	Derby	0	0	0	0	0	0	0	0	0	0	0
SAL_KA6327AA	PNUSAS019242	Derby	0	0	0	0	0	0	0	0	0	0	0
SAL_KA6975AA	PNUSAS019074	Derby	0	0	0	0	0	0	0	0	0	0	0
SAL_KA7095AA	FSIS1703335	Derby	0	0	0	0	0	0	0	0	0	0	0
SAL_KA7139AA	FSIS1703361	Derby	0	0	0	0	0	0	0	0	0	0	0
SAL_KA7161AA	FSIS1703405	Derby	0	0	0	0	0	0	0	0	0	0	0
SAL_KA7255AA	ADRDL-760	Derby	0	0	0	0	0	0	0	0	0	0	0
SAL_KA7873AA	PNUSAS019631	Derby	0	0	0	0	0	0	0	0	0	0	0
SAL_KA8324AA	FSIS1703372	Derby	0	0	0	0	0	0	0	0	0	0	0
SAL_KA8328AA	FSIS1703371	Derby	0	0	0	0	0	0	0	0	0	0	0
SAL_KA9096AA	PNUSAS020842	Derby	0	0	0	0	0	0	0	0	0	0	0
SAL_KA9170AA	ADRDL-914	Derby	0	0	0	0	0	0	0	0	0	0	0
SAL_KA9147AA	SAL-17-VL-OH-ON-0005	Derby	0	0	0	0	0	0	0	0	0	0	0
SAL_LA0115AA	19-20-20170706	Derby	0	0	0	0	0	0	0	0	0	0	0
SAL_KA9509AA	PNUSAS020718	Derby	0	0	0	0	0	0	0	0	0	0	0
SAL_LA0002AA	FSIS1703656	Derby	0	0	0	0	0	0	0	0	0	0	0
SAL_LA0022AA	FSIS1703561	Derby	0	0	0	0	0	0	0	0	0	0	0
SAL_LA0000AA	FSIS1703549	Derby	0	0	0	0	0	0	0	0	0	0	0
SAL_LA0209AA	2015AM-0276	Derby	0	0	0	0	0	0	0	0	0	0	0
SAL_LA0192AA	2015AM-0542	Derby	0	0	0	0	0	0	0	0	0	0	0
SAL_LA0395AA	FSIS11703945	Derby	0	0	0	0	0	0	0	0	0	0	0
SAL_LA0396AA	FSIS11703952	Derby	0	0	0	0	0	0	0	0	0	0	0
SAL_LA0844AA	CFSAN067835	Derby	0	0	0	0	0	0	0	0	0	0	0
SAL_LA0939AA	FDA00002764	Derby	0	0	0	0	0	0	0	0	0	0	0
SAL_LA0834AA	CFSAN067836	Derby	0	0	0	0	0	0	0	0	0	0	0
SAL_LA0773AA	CFSAN067831	Derby	0	0	0	0	0	0	0	0	0	0	0
SAL_LA0743AA	PNUSAS018173	Derby	0	0	0	0	0	0	0	0	0	0	0
SAL_LA1137AA	CFSAN067854	Derby	0	0	0	0	0	0	0	0	0	0	0
SAL_LA1323AA	CFSAN067888	Derby	0	0	0	0	0	0	0	0	0	0	0
SAL_LA1315AA	CFSAN067860	Derby	0	0	0	0	0	0	0	0	0	0	0
SAL_LA1168AA	PNUSAS021600	Derby	0	0	0	0	0	0	0	0	0	0	0
SAL_LA2357AA	FSIS11704070	Derby	0	0	0	0	0	0	0	0	0	0	0
SAL_LA2347AA	FSIS1703877	Derby	0	0	0	0	0	0	0	0	0	0	0
SAL_LA2874AA	0901F60541	Derby	0	0	0	0	0	0	0	0	0	0	0
SAL_LA2592AA	SA08.021	Derby	0	0	0	0	0	0	0	0	0	0	0
SAL_LA3700AA	FSIS21720130	Derby	0	0	0	0	0	0	0	0	0	0	0

SAL_LA4076AA	DB_21	Derby	0	0	0	0	0	0	0	0	0	0	0
SAL_LA4010AA	FSIS21720146	Derby	0	0	0	0	0	0	0	0	0	0	0
SAL_LA4362AA	FSIS11704261	Derby	0	0	0	0	0	0	0	0	0	0	0
SAL_LA4374AA	FSIS11704242	Derby	0	0	0	0	0	0	0	0	0	0	0
SAL_LA4528AA	PNUSAS022435	Derby	0	0	0	0	0	0	0	0	0	0	0
SAL_LA5008AA	PNUSAS019510	Derby	0	0	0	0	0	0	0	0	0	0	0
SAL_LA4837AA	PNUSAS022933	Derby	0	0	0	0	0	0	0	0	0	0	0
SAL_LA5078AA	255574	Derby	0	0	0	0	0	0	0	0	0	0	0
SAL_LA5079AA	373952	Derby	0	0	0	0	0	0	0	0	0	0	0
SAL_LA5080AA	376310	Derby	0	0	0	0	0	0	0	0	0	0	0
SAL_LA5082AA	252404	Derby	0	0	0	0	0	0	0	0	0	0	0
SAL_LA5357AA	1105-2012-0567	Derby	0	0	0	0	0	0	0	0	0	0	0
SAL_LA6630AA	PNUSAS022222	Derby	0	0	0	0	0	0	0	0	0	0	0
SAL_LA6632AA	PNUSAS022510	Derby	0	0	0	0	0	0	0	0	0	0	0
SAL_LA6451AA	PNUSAS024131	Derby	0	0	0	0	0	0	0	0	0	0	0
SAL_LA6892AA	PNUSAS022322	Derby	0	0	0	0	0	0	0	0	0	0	0
SAL_LA6228AA	CFSAN068060	Derby	0	0	0	0	0	0	0	0	0	0	0
SAL_LA7333AA	PNUSAS023557	Derby	0	0	0	0	0	0	0	0	0	0	0
SAL_LA7902AA	FSIS11704405	Derby	0	0	0	0	0	0	0	0	0	0	0
SAL_LA9917AA	24-11-20170985	Derby	0	0	0	0	0	0	0	0	0	0	0
SAL_LA9578AA	FSIS11704695	Derby	0	0	0	0	0	0	0	0	0	0	0
SAL_LA9168AA	PNUSAS024937	Derby	0	0	0	0	0	0	0	0	0	0	0
SAL_LA9149AA	PNUSAS023636	Derby	0	0	0	0	0	0	0	0	0	0	0
SAL_LA9154AA	PNUSAS023637	Derby	0	0	0	0	0	0	0	0	0	0	0
SAL_LA9008AA	FSIS11704640	Derby	0	0	0	0	0	0	0	0	0	0	0
SAL_MA1502AA	CFSAN070479	Derby	0	0	0	0	0	0	0	0	0	0	0
SAL_MA1336AA	FSIS11704829	Derby	0	0	0	0	0	0	0	0	0	0	0
SAL_MA2230AA	FSIS21720199	Derby	0	0	0	0	0	0	0	0	0	0	0
SAL_MA2304AA	FSIS21720339	Derby	0	0	0	0	0	0	0	0	0	0	0
SAL_MA2546AA	)84_Salmonella_Livingst	Derby	0	0	0	0	0	0	0	0	0	0	0
SAL_MA2728AA	PNUSAS022746	Derby	0	0	0	0	0	0	0	0	0	0	0
SAL_MA2772AA	PNUSAS021954	Derby	0	0	0	0	0	0	0	0	0	0	0
SAL_MA3010AA	34935	Derby	0	0	0	0	0	0	0	0	0	0	0
SAL_MA2985AA	D15-008988	Derby	0	0	0	0	0	0	0	0	0	0	0
SAL_MA2981AA	D14-056105	Derby	0	0	0	0	0	0	0	0	0	0	0
SAL_MA3572AA	26-12-20171082	Derby	0	0	0	0	0	0	0	0	0	0	0
SAL_MA3934AA	CFSAN070513	Derby	0	0	0	0	0	0	0	0	0	0	0
SAL_MA5041AA	FSIS1702644	Derby	0	0	0	0	0	0	0	0	0	0	0
SAL_MA5329AA	HIY0123	Derby	0	0	0	0	0	0	0	0	0	0	0
SAL_MA4779AA	FSIS11704652	Derby	0	0	0	0	0	0	0	0	0	0	0
SAL_CA4105AA	40244	Derby	0	0	0	0	0	0	0	0	0	0	0
SAL_DA5946AA	FDA00000023	Derby	0	0	0	0	0	0	0	0	0	0	0
SAL_CA3390AA	91968	Derby	0	0	0	0	0	0	0	0	0	0	0
SAL_CA4282AA	9454	Derby	0	0	0	0	0	0	0	0	0	0	0
SAL_EA1458AA	FSIS1500742	Derby	0	0	0	0	0	0	0	0	0	0	0
SAL_EA3580AA	CVM N51280	Derby	0	0	0	0	0	0	0	0	0	0	0
SAL_EA8025AA	56931	Derby	0	0	0	0	0	0	0	0	0	0	0
SAL_FA2978AA	CVM N56962F	Derby	0	0	0	0	0	0	0	0	0	0	0
SAL_FA2809AA	CVM N57528F	Derby	0	0	0	0	0	0	0	0	0	0	0
SAL_FA0561AA	CFSAN045592	Derby	0	0	0	0	0	0	0	0	0	0	0
SAL_FA2682AA	CVM N57930F	Derby	0	0	0	0	0	0	0	0	0	0	0
SAL_FA1743AA	HIY0243	Derby	0	0	0	0	0	0	0	0	0	0	0
SAL_HA9866AA	FSIS1609585	Derby	0	0	0	0	0	0	0	0	0	0	0
SAL_HA2827AA	FSIS1608219	Derby	0	0	0	0	0	0	0	0	0	0	0
SAL_GA2136AA	BCW_6301	Derby	0	0	0	0	0	0	0	0	0	0	0
SAL_HA8840AA	NCTC5722	Derby	0	0	0	0	0	0	0	0	0	0	0
SAL_GA0346AA	VNS013_D2-sc-2280620	Derby	0	0	0	0	0	0	0	0	0	0	0
SAL_GA5624AA	CVM N62475	Derby	0	0	0	0	0	0	0	0	0	0	0
SAL_GA0339AA	VNS159_LVT-sc-2280634	Derby	0	0	0	0	0	0	0	0	0	0	0
SAL_GA1878AA	BCW_5049	Derby	0	0	0	0	0	0	0	0	0	0	0
SAL_GA7949AA	2014AM-1149	Derby	0	0	0	0	0	0	0	0	0	0	0
SAL_GA5971AA	FSIS1607184	Derby	0	0	0	0	0	0	0	0	0	0	0
SAL_FA8662AA	CVM N31416	Derby	0	0	0	0	0	0	0	0	0	0	0
SAL_FA7739AA	PNUSAS002340	Derby	0	0	0	0	0	0	0	0	0	0	0
SAL_FA5410AA	FSIS1606193	Derby	0	0	0	0	0	0	0	0	0	0	0
SAL_FA5423AA	FSIS1606290	Derby	0	0	0	0	0	0	0	0	0	0	0
SAL_FA4928AA	CFSAN045271	Derby	0	0	0	0	0	0	0	0	0	0	0
SAL_FA2814AA	CVM N57510F	Derby	0	0	0	0	0	0	0	0	0	0	0
SAL_FA1488AA	Derby	Derby	0	0	0	0	0	0	0	0	0	0	0
SAL_FA0776AA	HIY0050	Derby	0	0	0	0	0	0	0	0	0	0	0
SAL_FA0646AA	NY-N20075	Derby	0	0	0	0	0	0	0	0	0	0	0
SAL_IA2367AA	FSIS1609405	Derby	0	0	0	0	0	0	0	0	0	0	0
SAL_IA2751AA	CFSAN059247	Derby	0	0	0	0	0	0	0	0	0	0	0

SAL_IA4955AA	CFSAN059278	Derby	0	0	0	0	0	0	0	0	0	0	0
SAL_IA5077AA	MS160287	Derby	0	0	0	0	0	0	0	0	0	0	0
SAL_IA7565AA	FSIS1710551	Derby	0	0	0	0	0	0	0	0	0	0	0
SAL_JA1336AA	248576	Derby	0	0	0	0	0	0	0	0	0	0	0
SAL_IA7699AA	MS160164	Derby	0	0	0	0	0	0	0	0	0	0	0
SAL_JA1411AA	241785	Derby	0	0	0	0	0	0	0	0	0	0	0
SAL_JA1311AA	113041	Derby	0	0	0	0	0	0	0	0	0	0	0
SAL_JA1368AA	205616	Derby	0	0	0	0	0	0	0	0	0	0	0
SAL_JA1752AA	PNUSAS011616	Derby	0	0	0	0	0	0	0	0	0	0	0
SAL_JA4219AA	FSIS1700576	Derby	0	0	0	0	0	0	0	0	0	0	0
SAL_JA6055AA	FSIS1701113	Derby	0	0	0	0	0	0	0	0	0	0	0
SAL_JA7231AA	FSIS1701526	Derby	0	0	0	0	0	0	0	0	0	0	0
SAL_KA5566AA	FSIS1702817	Derby	0	0	0	0	0	0	0	0	0	0	0
SAL_LA0766AA	CFSAN067859	Derby	0	0	0	0	0	0	0	0	0	0	0
SAL_LA5059AA	FSIS11704397	Derby	0	0	0	0	0	0	0	0	0	0	0
SAL_LA5077AA	385795	Derby	0	0	0	0	0	0	0	0	0	0	0
SAL_MA6108AA	FSIS11704880	Derby	0	0	0	0	0	0	0	0	0	0	0
SAL_MA6157AA	PNUSAS027417	Derby	0	0	0	0	0	0	0	0	0	0	0
SAL_MA6457AA	FSIS11705044	Derby	0	0	0	0	0	0	0	0	0	0	0
SAL_MA6547AA	FSIS11705702	Derby	0	0	0	0	0	0	0	0	0	0	0
SAL_MA6702AA	FSIS1701754	Derby	0	0	0	0	0	0	0	0	0	0	0
SAL_MA7400AA	ADRDL-1552	Derby	0	0	0	0	0	0	0	0	0	0	0
SAL_MA7371AA	FSIS21720640	Derby	0	0	0	0	0	0	0	0	0	0	0
SAL_MA7357AA	CFSAN071784	Derby	0	0	0	0	0	0	0	0	0	0	0
SAL_MA6909AA	FDA00012294	Derby	0	0	0	0	0	0	0	0	0	0	0
SAL_MA7615AA	PNUSAS028362	Derby	0	0	0	0	0	0	0	0	0	0	0
SAL_MA7477AA	FSIS11705573	Derby	0	0	0	0	0	0	0	0	0	0	0
SAL_MA7478AA	FSIS11705471	Derby	0	0	0	0	0	0	0	0	0	0	0
SAL_MA8735AA	ERS2012195	Derby	0	0	0	0	0	0	0	0	0	0	0
SAL_MA8736AA	ERS2012194	Derby	0	0	0	0	0	0	0	0	0	0	0
SAL_MA8739AA	ERS2012191	Derby	0	0	0	0	0	0	0	0	0	0	0
SAL_MA8741AA	ERS2012189	Derby	0	0	0	0	0	0	0	0	0	0	0
SAL_MA8744AA	ERS2012186	Derby	0	0	0	0	0	0	0	0	0	0	0
SAL_MA8745AA	ERS2012185	Derby	0	0	0	0	0	0	0	0	0	0	0
SAL_MA8746AA	ERS2012184	Derby	0	0	0	0	0	0	0	0	0	0	0
SAL_MA8751AA	ERS2012179	Derby	0	0	0	0	0	0	0	0	0	0	0
SAL_MA8752AA	ERS2012178	Derby	0	0	0	0	0	0	0	0	0	0	0
SAL_MA8753AA	ERS2012177	Derby	0	0	0	0	0	0	0	0	0	0	0
SAL_MA8750AA	ERS2012180	Derby	0	0	0	0	0	0	0	0	0	0	0
SAL_MA8748AA	ERS2012182	Derby	0	0	0	0	0	0	0	0	0	0	0
SAL_MA8756AA	ERS2012174	Derby	0	0	0	0	0	0	0	0	0	0	0
SAL_MA8754AA	ERS2012176	Derby	0	0	0	0	0	0	0	0	0	0	0
SAL_MA8755AA	ERS2012175	Derby	0	0	0	0	0	0	0	0	0	0	0
SAL_MA8764AA	ERS2012166	Derby	0	0	0	0	0	0	0	0	0	0	0
SAL_MA8757AA	ERS2012173	Derby	0	0	0	0	0	0	0	0	0	0	0
SAL_MA8765AA	ERS2012165	Derby	0	0	0	0	0	0	0	0	0	0	0
SAL_MA8731AA	ERS2012199	Derby	0	0	0	0	0	0	0	0	0	0	0
SAL_MA8732AA	ERS2012198	Derby	0	0	0	0	0	0	0	0	0	0	0
SAL_MA8733AA	ERS2012197	Derby	0	0	0	0	0	0	0	0	0	0	0
SAL_MA8734AA	ERS2012196	Derby	0	0	0	0	0	0	0	0	0	0	0
SAL_MA8737AA	ERS2012193	Derby	0	0	0	0	0	0	0	0	0	0	0
SAL_MA8738AA	ERS2012192	Derby	0	0	0	0	0	0	0	0	0	0	0
SAL_MA8740AA	ERS2012190	Derby	0	0	0	0	0	0	0	0	0	0	0
SAL_MA8742AA	ERS2012188	Derby	0	0	0	0	0	0	0	0	0	0	0
SAL_MA8743AA	ERS2012187	Derby	0	0	0	0	0	0	0	0	0	0	0
SAL_MA8749AA	ERS2012181	Derby	0	0	0	0	0	0	0	0	0	0	0
SAL_MA8747AA	ERS2012183	Derby	0	0	0	0	0	0	0	0	0	0	0
SAL_MA8760AA	ERS2012170	Derby	0	0	0	0	0	0	0	0	0	0	0
SAL_MA8762AA	ERS2012168	Derby	0	0	0	0	0	0	0	0	0	0	0
SAL_MA8761AA	ERS2012169	Derby	0	0	0	0	0	0	0	0	0	0	0
SAL_MA8763AA	ERS2012167	Derby	0	0	0	0	0	0	0	0	0	0	0
SAL_MA8902AA	FSIS11706008	Derby	0	0	0	0	0	0	0	0	0	0	0
SAL_MA8851AA	FSIS11705531	Derby	0	0	0	0	0	0	0	0	0	0	0
SAL_MA8836AA	FSIS11704582	Derby	0	0	0	0	0	0	0	0	0	0	0
SAL_MA8862AA	FSIS11705519	Derby	0	0	0	0	0	0	0	0	0	0	0
SAL_MA8877AA	FSIS11705864	Derby	0	0	0	0	0	0	0	0	0	0	0
SAL_MA8800AA	FSIS11705908	Derby	0	0	0	0	0	0	0	0	0	0	0
SAL_MA9097AA	CFSAN070550	Derby	0	0	0	0	0	0	0	0	0	0	0
SAL_MA8812AA	FSIS11705655	Derby	0	0	0	0	0	0	0	0	0	0	0
SAL_MA9047AA	FSIS11705334	Derby	0	0	0	0	0	0	0	0	0	0	0
SAL_MA9060AA	FSIS11705332	Derby	0	0	0	0	0	0	0	0	0	0	0
SAL_MA9033AA	FSIS11704655	Derby	0	0	0	0	0	0	0	0	0	0	0
SAL_MA9038AA	FSIS11705081	Derby	0	0	0	0	0	0	0	0	0	0	0

SAL_MA9014AA	CFSAN070532	Derby	0	0	0	0	0	0	0	0	0	0	0
SAL_MA9020AA	CFSAN070535	Derby	0	0	0	0	0	0	0	0	0	0	0
SAL_MA9103AA	CFSAN070530	Derby	0	0	0	0	0	0	0	0	0	0	0
SAL_MA9106AA	CFSAN070533	Derby	0	0	0	0	0	0	0	0	0	0	0
SAL_NA0356AA	FSIS21720704	Derby	0	0	0	0	0	0	0	0	0	0	0
SAL_NA0072AA	almonella_Derby_269-1	Derby	0	0	0	0	0	0	0	0	0	0	0
SAL_NA0446AA	FSIS11706164	Derby	0	0	0	0	0	0	0	0	0	0	0
SAL_NA0559AA	FSIS11706157	Derby	0	0	0	0	0	0	0	0	0	0	0
SAL_NA0624AA	PNUSAS024727	Derby	0	0	0	0	0	0	0	0	0	0	0
SAL_MA9522AA	almonella_Derby_154-1	Derby	0	0	0	0	0	0	0	0	0	0	0
SAL_NA1047AA	PNUSAS025563	Derby	0	0	0	0	0	0	0	0	0	0	0
SAL_NA1340AA	PNUSAS029487	Derby	0	0	0	0	0	0	0	0	0	0	0
SAL_NA1657AA	FSIS11706309	Derby	0	0	0	0	0	0	0	0	0	0	0
SAL_NA1659AA	FSIS11705675	Derby	0	0	0	0	0	0	0	0	0	0	0
SAL_NA1566AA	9-Salmonella-Derby-2009	Derby	0	0	0	0	0	0	0	0	0	0	0
SAL_NA1569AA	9-Salmonella-Derby-2008	Derby	0	0	0	0	0	0	0	0	0	0	0
SAL_NA1570AA	9-Salmonella-Derby-2008	Derby	0	0	0	0	0	0	0	0	0	0	0
SAL_NA1571AA	9-Salmonella-Derby-201	Derby	0	0	0	0	0	0	0	0	0	0	0
SAL_NA1577AA	109-Salmonella-Derby-F1	Derby	0	0	0	0	0	0	0	0	0	0	0
SAL_NA1572AA	9-Salmonella-Derby-2009	Derby	0	0	0	0	0	0	0	0	0	0	0
SAL_NA1579AA	9-Salmonella-Derby-201	Derby	0	0	0	0	0	0	0	0	0	0	0
SAL_NA1580AA	9-Salmonella-Derby-2007	Derby	0	0	0	0	0	0	0	0	0	0	0
SAL_NA1582AA	9-Salmonella-Derby-200	Derby	0	0	0	0	0	0	0	0	0	0	0
SAL_NA1583AA	109-Salmonella-Derby-F1	Derby	0	0	0	0	0	0	0	0	0	0	0
SAL_NA1585AA	9-Salmonella-Derby-200	Derby	0	0	0	0	0	0	0	0	0	0	0
SAL_NA1594AA	9-Salmonella-Derby-2012	Derby	0	0	0	0	0	0	0	0	0	0	0
SAL_NA1589AA	9-Salmonella-Derby-2013	Derby	0	0	0	0	0	0	0	0	0	0	0
SAL_NA1590AA	9-Salmonella-Derby-2010	Derby	0	0	0	0	0	0	0	0	0	0	0
SAL_NA1586AA	9-Salmonella-Derby-201	Derby	0	0	0	0	0	0	0	0	0	0	0
SAL_NA1591AA	109-Salmonella-Derby-F1	Derby	0	0	0	0	0	0	0	0	0	0	0
SAL_NA1599AA	109-Salmonella-Derby-F1	Derby	0	0	0	0	0	0	0	0	0	0	0
SAL_NA1542AA	PRJ1084_Salmonella_De	Derby	0	0	0	0	0	0	0	0	0	0	0
SAL_NA1543AA	PRJ1084_Salmonella_De	Derby	0	0	0	0	0	0	0	0	0	0	0
SAL_NA1544AA	J1084_Salmonella_Derby	Derby	0	0	0	0	0	0	0	0	0	0	0
SAL_NA1545AA	J1084_Salmonella_Derby	Derby	0	0	0	0	0	0	0	0	0	0	0
SAL_NA1549AA	RJ1084_Salmonella_Derb	Derby	0	0	0	0	0	0	0	0	0	0	0
SAL_NA1557AA	9-Salmonella-Derby-200	Derby	0	0	0	0	0	0	0	0	0	0	0
SAL_NA1555AA	9-Salmonella-Derby-2008	Derby	0	0	0	0	0	0	0	0	0	0	0
SAL_NA1559AA	9-Salmonella-Derby-2011	Derby	0	0	0	0	0	0	0	0	0	0	0
SAL_NA1562AA	9-Salmonella-Derby-2007	Derby	0	0	0	0	0	0	0	0	0	0	0
SAL_NA1563AA	9-Salmonella-Derby-200	Derby	0	0	0	0	0	0	0	0	0	0	0
SAL_NA1568AA	109-Salmonella-Derby-F1	Derby	0	0	0	0	0	0	0	0	0	0	0
SAL_NA1597AA	9-Salmonella-Derby-200	Derby	0	0	0	0	0	0	0	0	0	0	0
SAL_NA1598AA	109-Salmonella-Derby-F1	Derby	0	0	0	0	0	0	0	0	0	0	0
SAL_NA1574AA	9-Salmonella-Derby-201	Derby	0	0	0	0	0	0	0	0	0	0	0
SAL_NA1547AA	J1084_Salmonella_Derby	Derby	0	0	0	0	0	0	0	0	0	0	0
SAL_NA1546AA	PRJ1084_Salmonella_De	Derby	0	0	0	0	0	0	0	0	0	0	0
SAL_NA1603AA	109-Salmonella-Derby-F1	Derby	0	0	0	0	0	0	0	0	0	0	0
SAL_NA1604AA	9-Salmonella-Derby-201	Derby	0	0	0	0	0	0	0	0	0	0	0
SAL_NA1364AA	CFSAN072632	Derby	0	0	0	0	0	0	0	0	0	0	0
SAL_NA1986AA	FSIS11706139	Derby	0	0	0	0	0	0	0	0	0	0	0
SAL_NA1988AA	FSIS11706148	Derby	0	0	0	0	0	0	0	0	0	0	0
SAL_NA2106AA	CFSAN073526	Derby	0	0	0	0	0	0	0	0	0	0	0
SAL_NA2112AA	CFSAN073527	Derby	0	0	0	0	0	0	0	0	0	0	0
SAL_NA2113AA	CFSAN073478	Derby	0	0	0	0	0	0	0	0	0	0	0
SAL_NA2091AA	CFSAN073474	Derby	0	0	0	0	0	0	0	0	0	0	0
SAL_NA2078AA	CFSAN073481	Derby	0	0	0	0	0	0	0	0	0	0	0
SAL_NA2076AA	CFSAN073477	Derby	0	0	0	0	0	0	0	0	0	0	0
SAL_NA2089AA	CFSAN072638	Derby	0	0	0	0	0	0	0	0	0	0	0
SAL_NA2312AA	FDA00003772	Derby	0	0	0	0	0	0	0	0	0	0	0
SAL_NA2305AA	S15_0944	Derby	0	0	0	0	0	0	0	0	0	0	0
SAL_NA2310AA	1677_13	Derby	0	0	0	0	0	0	0	0	0	0	0
SAL_NA2303AA	S15_1303	Derby	0	0	0	0	0	0	0	0	0	0	0
SAL_NA2307AA	S14_0861	Derby	0	0	0	0	0	0	0	0	0	0	0
SAL_NA2275AA	S14_0453	Derby	0	0	0	0	0	0	0	0	0	0	0
SAL_NA2276AA	0298_12	Derby	0	0	0	0	0	0	0	0	0	0	0
SAL_NA2277AA	0923_13	Derby	0	0	0	0	0	0	0	0	0	0	0
SAL_NA2280AA	S14_0640	Derby	0	0	0	0	0	0	0	0	0	0	0
SAL_NA2283AA	S14_0451	Derby	0	0	0	0	0	0	0	0	0	0	0
SAL_NA2293AA	S14_1405	Derby	0	0	0	0	0	0	0	0	0	0	0
SAL_NA2292AA	S14_1553	Derby	0	0	0	0	0	0	0	0	0	0	0
SAL_NA2295AA	S14_1173	Derby	0	0	0	0	0	0	0	0	0	0	0
SAL_NA2296AA	S15_0166	Derby	0	0	0	0	0	0	0	0	0	0	0

SAL_NA2297AA	S14_1008	Derby	0	0	0	0	0	0	0	0	0	0	0
SAL_NA2298AA	S15_1236	Derby	0	0	0	0	0	0	0	0	0	0	0
SAL_NA2294AA	S15_1149	Derby	0	0	0	0	0	0	0	0	0	0	0
SAL_NA2299AA	S15_1752	Derby	0	0	0	0	0	0	0	0	0	0	0
SAL_NA2309AA	S14_1554	Derby	0	0	0	0	0	0	0	0	0	0	0
SAL_NA2256AA	1207_12	Derby	0	0	0	0	0	0	0	0	0	0	0
SAL_NA2260AA	0283_12	Derby	0	0	0	0	0	0	0	0	0	0	0
SAL_NA2267AA	0964_11	Derby	0	0	0	0	0	0	0	0	0	0	0
SAL_NA2271AA	1998_11	Derby	0	0	0	0	0	0	0	0	0	0	0
SAL_NA2281AA	1683_12	Derby	0	0	0	0	0	0	0	0	0	0	0
SAL_NA2286AA	0726_12	Derby	0	0	0	0	0	0	0	0	0	0	0
SAL_NA2287AA	1114_12	Derby	0	0	0	0	0	0	0	0	0	0	0
SAL_NA2289AA	2165_11	Derby	0	0	0	0	0	0	0	0	0	0	0
SAL_NA2261AA	1996_10	Derby	0	0	0	0	0	0	0	0	0	0	0
SAL_NA2270AA	1100_12	Derby	0	0	0	0	0	0	0	0	0	0	0
SAL_NA2273AA	1535_10	Derby	0	0	0	0	0	0	0	0	0	0	0
SAL_NA2278AA	S14_0301	Derby	0	0	0	0	0	0	0	0	0	0	0
SAL_NA2279AA	0345_12	Derby	0	0	0	0	0	0	0	0	0	0	0
SAL_NA2263AA	1784_10	Derby	0	0	0	0	0	0	0	0	0	0	0
SAL_NA2262AA	1230_12	Derby	0	0	0	0	0	0	0	0	0	0	0
SAL_NA2266AA	0849_12	Derby	0	0	0	0	0	0	0	0	0	0	0
SAL_NA2269AA	1015_11	Derby	0	0	0	0	0	0	0	0	0	0	0
SAL_NA2268AA	0890_12	Derby	0	0	0	0	0	0	0	0	0	0	0
SAL_NA2265AA	2079_11	Derby	0	0	0	0	0	0	0	0	0	0	0
SAL_NA2284AA	S14_0449	Derby	0	0	0	0	0	0	0	0	0	0	0
SAL_NA2285AA	S14_0637	Derby	0	0	0	0	0	0	0	0	0	0	0
SAL_NA2257AA	1857_11	Derby	0	0	0	0	0	0	0	0	0	0	0
SAL_NA2258AA	0600_10	Derby	0	0	0	0	0	0	0	0	0	0	0
SAL_NA2379AA	PNUSAS026714	Derby	0	0	0	0	0	0	0	0	0	0	0
SAL_NA2818AA	CFSAN031934	Derby	0	0	0	0	0	0	0	0	0	0	0
SAL_NA2795AA	CFSAN031936	Derby	0	0	0	0	0	0	0	0	0	0	0
SAL_NA2773AA	CFSAN031928	Derby	0	0	0	0	0	0	0	0	0	0	0
SAL_NA2678AA	FSIS11706283	Derby	0	0	0	0	0	0	0	0	0	0	0
SAL_NA2687AA	FSIS11706281	Derby	0	0	0	0	0	0	0	0	0	0	0
SAL_NA3026AA	PNUSAS030082	Derby	0	0	0	0	0	0	0	0	0	0	0
SAL_NA3224AA	CFSAN073570	Derby	0	0	0	0	0	0	0	0	0	0	0
SAL_NA4132AA	FSIS11706618	Derby	0	0	0	0	0	0	0	0	0	0	0
SAL_NA4130AA	FSIS11706615	Derby	0	0	0	0	0	0	0	0	0	0	0
SAL_NA4144AA	FSIS11706803	Derby	0	0	0	0	0	0	0	0	0	0	0
SAL_NA4145AA	FSIS11706613	Derby	0	0	0	0	0	0	0	0	0	0	0
SAL_NA4040AA	FSIS11706802	Derby	0	0	0	0	0	0	0	0	0	0	0
SAL_NA4395AA	FSIS31800029	Derby	0	0	0	0	0	0	0	0	0	0	0
SAL_NA4409AA	35-9-8101278	Derby	0	0	0	0	0	0	0	0	0	0	0
SAL_NA4549AA	FSIS31800039	Derby	0	0	0	0	0	0	0	0	0	0	0
SAL_NA5180AA	CFSAN074319	Derby	0	0	0	0	0	0	0	0	0	0	0
SAL_NA5092AA	CFSAN074318	Derby	0	0	0	0	0	0	0	0	0	0	0
SAL_NA5217AA	CFSAN074327	Derby	0	0	0	0	0	0	0	0	0	0	0
SAL_NA5429AA	2015AM-0046	Derby	0	0	0	0	0	0	0	0	0	0	0
SAL_NA6127AA	CFSAN073476	Derby	0	0	0	0	0	0	0	0	0	0	0
SAL_NA6190AA	CFSAN074233	Derby	0	0	0	0	0	0	0	0	0	0	0
SAL_NA5853AA	CFSAN074234	Derby	0	0	0	0	0	0	0	0	0	0	0
SAL_NA5854AA	CFSAN074237	Derby	0	0	0	0	0	0	0	0	0	0	0
SAL_NA5668AA	CFSAN074362	Derby	0	0	0	0	0	0	0	0	0	0	0
SAL_NA5677AA	CFSAN074344	Derby	0	0	0	0	0	0	0	0	0	0	0
SAL_NA6538AA	2015AM-2926	Derby	0	0	0	0	0	0	0	0	0	0	0
SAL_NA6415AA	FSIS11706612	Derby	0	0	0	0	0	0	0	0	0	0	0
SAL_NA6429AA	FSIS11706286	Derby	0	0	0	0	0	0	0	0	0	0	0
SAL_NA6374AA	FSIS11706952	Derby	0	0	0	0	0	0	0	0	0	0	0
SAL_NA7846AA	WINNING21-Salmonella	Derby	0	0	0	0	0	0	0	0	0	0	0
SAL_NA7847AA	WINNING21-Salmonella	Derby	0	0	0	0	0	0	0	0	0	0	0
SAL_NA7834AA	1571_S9	Derby	0	0	0	0	0	0	0	0	0	0	0
SAL_NA7848AA	WINNING21-Salmonella	Derby	0	0	0	0	0	0	0	0	0	0	0
SAL_NA7849AA	WINNING21-Salmonella	Derby	0	0	0	0	0	0	0	0	0	0	0
SAL_NA7850AA	WINNING21-Salmonella	Derby	0	0	0	0	0	0	0	0	0	0	0
SAL_NA7808AA	17-43359_S21	Derby	0	0	0	0	0	0	0	0	0	0	0
SAL_NA7809AA	17-43357_S20	Derby	0	0	0	0	0	0	0	0	0	0	0
SAL_NA7814AA	17-43352_S15	Derby	0	0	0	0	0	0	0	0	0	0	0
SAL_NA7815AA	17-43351_S14	Derby	0	0	0	0	0	0	0	0	0	0	0
SAL_NA7816AA	17-43350_S13	Derby	0	0	0	0	0	0	0	0	0	0	0
SAL_NA7824AA	1586_S19	Derby	0	0	0	0	0	0	0	0	0	0	0
SAL_NA7827AA	1583_S16	Derby	0	0	0	0	0	0	0	0	0	0	0
SAL_NA7830AA	1580_S13	Derby	0	0	0	0	0	0	0	0	0	0	0
SAL_NA7831AA	1579_S12	Derby	0	0	0	0	0	0	0	0	0	0	0

SAL_NA7832AA	1578_S11	Derby	0	0	0	0	0	0	0	0	0	0	0
SAL_NA7835AA	1568_S8	Derby	0	0	0	0	0	0	0	0	0	0	0
SAL_NA7843AA	WINNING21-Salmonella-	Derby	0	0	0	0	0	0	0	0	0	0	0
SAL_NA7842AA	1560_S1	Derby	0	0	0	0	0	0	0	0	0	0	0
SAL_NA7794AA	17-43376_S35	Derby	0	0	0	0	0	0	0	0	0	0	0
SAL_NA7800AA	17-43369_S29	Derby	0	0	0	0	0	0	0	0	0	0	0
SAL_NA7802AA	17-43367_S27	Derby	0	0	0	0	0	0	0	0	0	0	0
SAL_NA7805AA	17-43362_S24	Derby	0	0	0	0	0	0	0	0	0	0	0
SAL_NA7806AA	17-43361_S23	Derby	0	0	0	0	0	0	0	0	0	0	0
SAL_NA7807AA	17-43360_S22	Derby	0	0	0	0	0	0	0	0	0	0	0
SAL_NA7828AA	1582_S15	Derby	0	0	0	0	0	0	0	0	0	0	0
SAL_NA7829AA	1581_S14	Derby	0	0	0	0	0	0	0	0	0	0	0
SAL_NA7838AA	1565_S5	Derby	0	0	0	0	0	0	0	0	0	0	0
SAL_NA7839AA	1564_S4	Derby	0	0	0	0	0	0	0	0	0	0	0
SAL_NA7761AA	17-43429_S32	Derby	0	0	0	0	0	0	0	0	0	0	0
SAL_NA7767AA	17-43423_S26	Derby	0	0	0	0	0	0	0	0	0	0	0
SAL_NA7769AA	17-43421_S24	Derby	0	0	0	0	0	0	0	0	0	0	0
SAL_NA7773AA	17-43416_S20	Derby	0	0	0	0	0	0	0	0	0	0	0
SAL_NA7770AA	17-43420_S23	Derby	0	0	0	0	0	0	0	0	0	0	0
SAL_NA7772AA	17-43417_S21	Derby	0	0	0	0	0	0	0	0	0	0	0
SAL_NA7774AA	17-43415_S19	Derby	0	0	0	0	0	0	0	0	0	0	0
SAL_NA7779AA	17-43409_S14	Derby	0	0	0	0	0	0	0	0	0	0	0
SAL_NA7780AA	17-43408_S13	Derby	0	0	0	0	0	0	0	0	0	0	0
SAL_NA7781AA	17-43405_S12	Derby	0	0	0	0	0	0	0	0	0	0	0
SAL_NA7782AA	17-43404_S11	Derby	0	0	0	0	0	0	0	0	0	0	0
SAL_NA7785AA	17-43400_S8	Derby	0	0	0	0	0	0	0	0	0	0	0
SAL_NA7786AA	17-43399_S7	Derby	0	0	0	0	0	0	0	0	0	0	0
SAL_NA7788AA	17-43387_S5	Derby	0	0	0	0	0	0	0	0	0	0	0
SAL_NA7787AA	17-43389_S6	Derby	0	0	0	0	0	0	0	0	0	0	0
SAL_NA7789AA	17-43386_S4	Derby	0	0	0	0	0	0	0	0	0	0	0
SAL_NA7792AA	17-43382_S1	Derby	0	0	0	0	0	0	0	0	0	0	0
SAL_NA7793AA	17-43377_S36	Derby	0	0	0	0	0	0	0	0	0	0	0
SAL_NA7795AA	17-43375_S34	Derby	0	0	0	0	0	0	0	0	0	0	0
SAL_NA7796AA	17-43374_S33	Derby	0	0	0	0	0	0	0	0	0	0	0
SAL_NA7797AA	17-43373_S32	Derby	0	0	0	0	0	0	0	0	0	0	0
SAL_NA7798AA	17-43372_S31	Derby	0	0	0	0	0	0	0	0	0	0	0
SAL_NA7799AA	17-43371_S30	Derby	0	0	0	0	0	0	0	0	0	0	0
SAL_NA7803AA	17-43366_S26	Derby	0	0	0	0	0	0	0	0	0	0	0
SAL_NA7804AA	17-43364_S25	Derby	0	0	0	0	0	0	0	0	0	0	0
SAL_NA7820AA	1591_S23	Derby	0	0	0	0	0	0	0	0	0	0	0
SAL_NA7821AA	1590_S22	Derby	0	0	0	0	0	0	0	0	0	0	0
SAL_NA7822AA	1588_S21	Derby	0	0	0	0	0	0	0	0	0	0	0
SAL_NA7823AA	1587_S20	Derby	0	0	0	0	0	0	0	0	0	0	0
SAL_NA7836AA	1567_S7	Derby	0	0	0	0	0	0	0	0	0	0	0
SAL_NA7837AA	1566_S6	Derby	0	0	0	0	0	0	0	0	0	0	0
SAL_NA7717AA	17-106711_S12	Derby	0	0	0	0	0	0	0	0	0	0	0
SAL_NA7712AA	17-106722_S17	Derby	0	0	0	0	0	0	0	0	0	0	0
SAL_NA7742AA	17-94866_S23	Derby	0	0	0	0	0	0	0	0	0	0	0
SAL_NA7743AA	17-94865_S22	Derby	0	0	0	0	0	0	0	0	0	0	0
SAL_NA7746AA	17-94859_S19	Derby	0	0	0	0	0	0	0	0	0	0	0
SAL_NA7747AA	17-94858_S18	Derby	0	0	0	0	0	0	0	0	0	0	0
SAL_NA7744AA	17-94864_S21	Derby	0	0	0	0	0	0	0	0	0	0	0
SAL_NA7745AA	17-94861_S20	Derby	0	0	0	0	0	0	0	0	0	0	0
SAL_NA7748AA	17-94857_S17	Derby	0	0	0	0	0	0	0	0	0	0	0
SAL_NA7749AA	17-94856_S16	Derby	0	0	0	0	0	0	0	0	0	0	0
SAL_NA7751AA	17-94854_S14	Derby	0	0	0	0	0	0	0	0	0	0	0
SAL_NA7750AA	17-94855_S15	Derby	0	0	0	0	0	0	0	0	0	0	0
SAL_NA7753AA	17-94850_S12	Derby	0	0	0	0	0	0	0	0	0	0	0
SAL_NA7754AA	17-94848_S11	Derby	0	0	0	0	0	0	0	0	0	0	0
SAL_NA7755AA	17-94847_S10	Derby	0	0	0	0	0	0	0	0	0	0	0
SAL_NA7757AA	17-43418_S1	Derby	0	0	0	0	0	0	0	0	0	0	0
SAL_NA7756AA	17-94846_S9	Derby	0	0	0	0	0	0	0	0	0	0	0
SAL_NA7762AA	17-43428_S31	Derby	0	0	0	0	0	0	0	0	0	0	0
SAL_NA7758AA	17-43432_S35	Derby	0	0	0	0	0	0	0	0	0	0	0
SAL_NA7768AA	17-43422_S25	Derby	0	0	0	0	0	0	0	0	0	0	0
SAL_NA7764AA	17-43426_S29	Derby	0	0	0	0	0	0	0	0	0	0	0
SAL_NA7776AA	17-43413_S17	Derby	0	0	0	0	0	0	0	0	0	0	0
SAL_NA7771AA	17-43419_S22	Derby	0	0	0	0	0	0	0	0	0	0	0
SAL_NA7825AA	1585_S18	Derby	0	0	0	0	0	0	0	0	0	0	0
SAL_NA7826AA	1584_S17	Derby	0	0	0	0	0	0	0	0	0	0	0
SAL_NA7840AA	1563_S3	Derby	0	0	0	0	0	0	0	0	0	0	0
SAL_NA7841AA	1562_S2	Derby	0	0	0	0	0	0	0	0	0	0	0
SAL_NA7765AA	17-43425_S28	Derby	0	0	0	0	0	0	0	0	0	0	0

SAL_NA7775AA	17-43414_S18	Derby	0	0	0	0	0	0	0	0	0	0	0
SAL_NA7703AA	17-106744_S27	Derby	0	0	0	0	0	0	0	0	0	0	0
SAL_NA7704AA	17-106743_S26	Derby	0	0	0	0	0	0	0	0	0	0	0
SAL_NA7705AA	17-106742_S25	Derby	0	0	0	0	0	0	0	0	0	0	0
SAL_NA7711AA	17-106723_S18	Derby	0	0	0	0	0	0	0	0	0	0	0
SAL_NA7726AA	17-94889_S3	Derby	0	0	0	0	0	0	0	0	0	0	0
SAL_NA7727AA	17-94887_S2	Derby	0	0	0	0	0	0	0	0	0	0	0
SAL_NA7728AA	17-94886_S1	Derby	0	0	0	0	0	0	0	0	0	0	0
SAL_NA7729AA	17-94885_S36	Derby	0	0	0	0	0	0	0	0	0	0	0
SAL_NA7730AA	17-94884_S35	Derby	0	0	0	0	0	0	0	0	0	0	0
SAL_NA7732AA	17-94881_S33	Derby	0	0	0	0	0	0	0	0	0	0	0
SAL_NA7738AA	17-94871_S27	Derby	0	0	0	0	0	0	0	0	0	0	0
SAL_NA7733AA	17-94879_S32	Derby	0	0	0	0	0	0	0	0	0	0	0
SAL_NA7734AA	17-94877_S31	Derby	0	0	0	0	0	0	0	0	0	0	0
SAL_NA7739AA	17-94870_S26	Derby	0	0	0	0	0	0	0	0	0	0	0
SAL_NA7740AA	17-94868_S25	Derby	0	0	0	0	0	0	0	0	0	0	0
SAL_NA7735AA	17-94875_S30	Derby	0	0	0	0	0	0	0	0	0	0	0
SAL_NA7741AA	17-94867_S24	Derby	0	0	0	0	0	0	0	0	0	0	0
SAL_NA7709AA	17-106728_S20	Derby	0	0	0	0	0	0	0	0	0	0	0
SAL_NA7701AA	17-106746_S29	Derby	0	0	0	0	0	0	0	0	0	0	0
SAL_NA7716AA	17-106714_S13	Derby	0	0	0	0	0	0	0	0	0	0	0
SAL_NA7715AA	17-106716_S14	Derby	0	0	0	0	0	0	0	0	0	0	0
SAL_NA7713AA	17-106721_S16	Derby	0	0	0	0	0	0	0	0	0	0	0
SAL_NA7718AA	17-106710_S11	Derby	0	0	0	0	0	0	0	0	0	0	0
SAL_NA7723AA	17-94892_S6	Derby	0	0	0	0	0	0	0	0	0	0	0
SAL_NA7724AA	17-94891_S5	Derby	0	0	0	0	0	0	0	0	0	0	0
SAL_NA7719AA	17-106708_S10	Derby	0	0	0	0	0	0	0	0	0	0	0
SAL_NA7720AA	17-106705_S9	Derby	0	0	0	0	0	0	0	0	0	0	0
SAL_NA7714AA	17-106719_S15	Derby	0	0	0	0	0	0	0	0	0	0	0
SAL_NA7721AA	17-94894_S8	Derby	0	0	0	0	0	0	0	0	0	0	0
SAL_NA7722AA	17-94893_S7	Derby	0	0	0	0	0	0	0	0	0	0	0
SAL_NA7725AA	17-94890_S4	Derby	0	0	0	0	0	0	0	0	0	0	0
SAL_NA7708AA	17-106731_S21	Derby	0	0	0	0	0	0	0	0	0	0	0
SAL_NA7737AA	17-94872_S28	Derby	0	0	0	0	0	0	0	0	0	0	0
SAL_NA7525AA	NC_17MP0290	Derby	0	0	0	0	0	0	0	0	0	0	0
SAL_NA7529AA	NC_SI011	Derby	0	0	0	0	0	0	0	0	0	0	0
SAL_NA7371AA	2015AM-3415	Derby	0	0	0	0	0	0	0	0	0	0	0
SAL_NA7759AA	17-43431_S34	Derby	0	0	0	0	0	0	0	0	0	0	0
SAL_NA7844AA	'WINNING21-Salmonella-	Derby	0	0	0	0	0	0	0	0	0	0	0
SAL_NA7845AA	'WINNING21-Salmonella-	Derby	0	0	0	0	0	0	0	0	0	0	0
SAL_NA8305AA	4-9-8100072	Derby	0	0	0	0	0	0	0	0	0	0	0
SAL_NA8436AA	PNUSAS032910	Derby	0	0	0	0	0	0	0	0	0	0	0
SAL_NA9147AA	2014LSAL03524	Derby	0	0	0	0	0	0	0	0	0	0	0
SAL_NA9148AA	2014LSAL03616	Derby	0	0	0	0	0	0	0	0	0	0	0
SAL_NA9149AA	2014LSAL03694	Derby	0	0	0	0	0	0	0	0	0	0	0
SAL_NA9151AA	2014LSAL03470	Derby	0	0	0	0	0	0	0	0	0	0	0
SAL_NA9152AA	2014LSAL03478	Derby	0	0	0	0	0	0	0	0	0	0	0
SAL_NA9154AA	2014LSAL03521	Derby	0	0	0	0	0	0	0	0	0	0	0
SAL_NA9117AA	2014LSAL01306	Derby	0	0	0	0	0	0	0	0	0	0	0
SAL_NA9118AA	2015LSAL01591	Derby	0	0	0	0	0	0	0	0	0	0	0
SAL_NA9123AA	2015LSAL01029	Derby	0	0	0	0	0	0	0	0	0	0	0
SAL_NA9109AA	2014LSAL01779	Derby	0	0	0	0	0	0	0	0	0	0	0
SAL_NA9110AA	2014LSAL01467	Derby	0	0	0	0	0	0	0	0	0	0	0
SAL_NA9113AA	2014LSAL01499	Derby	0	0	0	0	0	0	0	0	0	0	0
SAL_NA9114AA	2014LSAL01256	Derby	0	0	0	0	0	0	0	0	0	0	0
SAL_NA9115AA	2014LSAL01220	Derby	0	0	0	0	0	0	0	0	0	0	0
SAL_NA9116AA	2014LSAL01311	Derby	0	0	0	0	0	0	0	0	0	0	0
SAL_NA9127AA	2014LSAL05133	Derby	0	0	0	0	0	0	0	0	0	0	0
SAL_NA9146AA	2015LSAL01817	Derby	0	0	0	0	0	0	0	0	0	0	0
SAL_NA9153AA	2014LSAL03510	Derby	0	0	0	0	0	0	0	0	0	0	0
SAL_NA9092AA	2014LSAL02073	Derby	0	0	0	0	0	0	0	0	0	0	0
SAL_NA9103AA	2014LSAL05735	Derby	0	0	0	0	0	0	0	0	0	0	0
SAL_NA9104AA	2014LSAL00814	Derby	0	0	0	0	0	0	0	0	0	0	0
SAL_NA9105AA	2014LSAL05495	Derby	0	0	0	0	0	0	0	0	0	0	0
SAL_NA9106AA	2015LSAL00314	Derby	0	0	0	0	0	0	0	0	0	0	0
SAL_NA9107AA	2015LSAL00428	Derby	0	0	0	0	0	0	0	0	0	0	0
SAL_NA9108AA	2014LSAL01780	Derby	0	0	0	0	0	0	0	0	0	0	0
SAL_NA9111AA	2014LSAL01383	Derby	0	0	0	0	0	0	0	0	0	0	0
SAL_NA9119AA	2015LSAL01314	Derby	0	0	0	0	0	0	0	0	0	0	0
SAL_NA9124AA	2015LSAL01058	Derby	0	0	0	0	0	0	0	0	0	0	0
SAL_NA9135AA	2014LSAL04065	Derby	0	0	0	0	0	0	0	0	0	0	0
SAL_NA9136AA	2014LSAL03985	Derby	0	0	0	0	0	0	0	0	0	0	0
SAL_NA9137AA	2015LSAL02462	Derby	0	0	0	0	0	0	0	0	0	0	0

SAL_NA9139AA	2015LSAL01748	Derby	0	0	0	0	0	0	0	0	0	0	0
SAL_NA9140AA	2015LSAL01716	Derby	0	0	0	0	0	0	0	0	0	0	0
SAL_NA9141AA	2015LSAL01713	Derby	0	0	0	0	0	0	0	0	0	0	0
SAL_NA9142AA	2015LSAL01697	Derby	0	0	0	0	0	0	0	0	0	0	0
SAL_NA9144AA	2015LSAL01829	Derby	0	0	0	0	0	0	0	0	0	0	0
SAL_NA9091AA	2014LSAL02051	Derby	0	0	0	0	0	0	0	0	0	0	0
SAL_NA9097AA	2014LSAL02325	Derby	0	0	0	0	0	0	0	0	0	0	0
SAL_NA9098AA	2014LSAL02431	Derby	0	0	0	0	0	0	0	0	0	0	0
SAL_NA9099AA	2015LSAL00190	Derby	0	0	0	0	0	0	0	0	0	0	0
SAL_NA9100AA	2015LSAL00219	Derby	0	0	0	0	0	0	0	0	0	0	0
SAL_NA9102AA	2015LSAL00137	Derby	0	0	0	0	0	0	0	0	0	0	0
SAL_NA9112AA	2014LSAL01651	Derby	0	0	0	0	0	0	0	0	0	0	0
SAL_NA9125AA	2015LSAL01075	Derby	0	0	0	0	0	0	0	0	0	0	0
SAL_NA9126AA	2015LSAL01179	Derby	0	0	0	0	0	0	0	0	0	0	0
SAL_NA9129AA	2014LSAL03976	Derby	0	0	0	0	0	0	0	0	0	0	0
SAL_NA9130AA	2014LSAL03975	Derby	0	0	0	0	0	0	0	0	0	0	0
SAL_NA9131AA	2014LSAL03974	Derby	0	0	0	0	0	0	0	0	0	0	0
SAL_NA9133AA	2014LSAL04673	Derby	0	0	0	0	0	0	0	0	0	0	0
SAL_NA9145AA	2015LSAL01821	Derby	0	0	0	0	0	0	0	0	0	0	0
SAL_NA9087AA	2015LSAL00989	Derby	0	0	0	0	0	0	0	0	0	0	0
SAL_NA9088AA	2014LSAL00873	Derby	0	0	0	0	0	0	0	0	0	0	0
SAL_NA9121AA	2015LSAL01341	Derby	0	0	0	0	0	0	0	0	0	0	0
SAL_NA9122AA	2015LSAL01474	Derby	0	0	0	0	0	0	0	0	0	0	0
SAL_NA9134AA	2014LSAL04466	Derby	0	0	0	0	0	0	0	0	0	0	0
SAL_NA9128AA	2014LSAL04833	Derby	0	0	0	0	0	0	0	0	0	0	0
SAL_NA9138AA	2015LSAL02005	Derby	0	0	0	0	0	0	0	0	0	0	0
SAL_NA9156AA	2014LSAL03926	Derby	0	0	0	0	0	0	0	0	0	0	0
SAL_NA9095AA	2014LSAL02278	Derby	0	0	0	0	0	0	0	0	0	0	0
SAL_NA9096AA	2014LSAL02285	Derby	0	0	0	0	0	0	0	0	0	0	0
SAL_NA9093AA	2014LSAL02163	Derby	0	0	0	0	0	0	0	0	0	0	0
SAL_NA9094AA	2014LSAL02205	Derby	0	0	0	0	0	0	0	0	0	0	0
SAL_NA9089AA	2014LSAL01781	Derby	0	0	0	0	0	0	0	0	0	0	0
SAL_NA9090AA	2014LSAL01894	Derby	0	0	0	0	0	0	0	0	0	0	0
SAL_NA9086AA	2015LSAL00990	Derby	0	0	0	0	0	0	0	0	0	0	0
SAL_NA9072AA	2014LSAL02547	Derby	0	0	0	0	0	0	0	0	0	0	0
SAL_NA9070AA	2014LSAL03150	Derby	0	0	0	0	0	0	0	0	0	0	0
SAL_NA9069AA	2014LSAL03218	Derby	0	0	0	0	0	0	0	0	0	0	0
SAL_NA9071AA	2014LSAL02838	Derby	0	0	0	0	0	0	0	0	0	0	0
SAL_NA9073AA	2014LSAL02463	Derby	0	0	0	0	0	0	0	0	0	0	0
SAL_NA9074AA	2014LSAL02441	Derby	0	0	0	0	0	0	0	0	0	0	0
SAL_NA9076AA	2014LSAL03344	Derby	0	0	0	0	0	0	0	0	0	0	0
SAL_NA9081AA	2015LSAL00728	Derby	0	0	0	0	0	0	0	0	0	0	0
SAL_NA9082AA	2015LSAL00452	Derby	0	0	0	0	0	0	0	0	0	0	0
SAL_NA9083AA	2015LSAL00429	Derby	0	0	0	0	0	0	0	0	0	0	0
SAL_NA9084AA	2015LSAL00477	Derby	0	0	0	0	0	0	0	0	0	0	0
SAL_NA9085AA	2015LSAL00465	Derby	0	0	0	0	0	0	0	0	0	0	0
SAL_NA9077AA	2014LSAL03342	Derby	0	0	0	0	0	0	0	0	0	0	0
SAL_NA9065AA	2015LSAL02717	Derby	0	0	0	0	0	0	0	0	0	0	0
SAL_NA9066AA	2014LSAL00980	Derby	0	0	0	0	0	0	0	0	0	0	0
SAL_NA9068AA	2014LSAL03309	Derby	0	0	0	0	0	0	0	0	0	0	0
SAL_NA9067AA	2014LSAL03340	Derby	0	0	0	0	0	0	0	0	0	0	0
SAL_NA9079AA	2015LSAL00641	Derby	0	0	0	0	0	0	0	0	0	0	0
SAL_NA9080AA	2015LSAL00981	Derby	0	0	0	0	0	0	0	0	0	0	0
SAL_NA9032AA	2014LSAL03409	Derby	0	0	0	0	0	0	0	0	0	0	0
SAL_NA9062AA	2014LSAL00455	Derby	0	0	0	0	0	0	0	0	0	0	0
SAL_NA9027AA	2014LSAL05538	Derby	0	0	0	0	0	0	0	0	0	0	0
SAL_NA9028AA	2015LSAL03563	Derby	0	0	0	0	0	0	0	0	0	0	0
SAL_NA9029AA	2015LSAL03564	Derby	0	0	0	0	0	0	0	0	0	0	0
SAL_NA9031AA	2014LSAL03401	Derby	0	0	0	0	0	0	0	0	0	0	0
SAL_NA9030AA	2014LSAL03404	Derby	0	0	0	0	0	0	0	0	0	0	0
SAL_NA9063AA	2015LSAL03260	Derby	0	0	0	0	0	0	0	0	0	0	0
SAL_NA9064AA	2015LSAL02588	Derby	0	0	0	0	0	0	0	0	0	0	0
SAL_NA9022AA	2014LSAL00717	Derby	0	0	0	0	0	0	0	0	0	0	0
SAL_NA9023AA	2015LSAL03121	Derby	0	0	0	0	0	0	0	0	0	0	0
SAL_NA9024AA	2014LSAL00337	Derby	0	0	0	0	0	0	0	0	0	0	0
SAL_NA9025AA	2014LSAL00274	Derby	0	0	0	0	0	0	0	0	0	0	0
SAL_NA9059AA	2014LSAL03398	Derby	0	0	0	0	0	0	0	0	0	0	0
SAL_NA9061AA	2015LSAL03285	Derby	0	0	0	0	0	0	0	0	0	0	0
SAL_NA9057AA	2014LSAL03388	Derby	0	0	0	0	0	0	0	0	0	0	0
SAL_NA9058AA	2014LSAL03396	Derby	0	0	0	0	0	0	0	0	0	0	0
SAL_NA9019AA	2015LSAL03548	Derby	0	0	0	0	0	0	0	0	0	0	0
SAL_NA9060AA	2015LSAL03284	Derby	0	0	0	0	0	0	0	0	0	0	0
SAL_NA9043AA	2015LSAL04332	Derby	0	0	0	0	0	0	0	0	0	0	0

SAL_NA9040AA	2015LSAL04227	Derby	0	0	0	0	0	0	0	0	0	0	0
SAL_NA9039AA	2014LSAL03463	Derby	0	0	0	0	0	0	0	0	0	0	0
SAL_NA9045AA	2015LSAL03879	Derby	0	0	0	0	0	0	0	0	0	0	0
SAL_NA9044AA	2015LSAL03891	Derby	0	0	0	0	0	0	0	0	0	0	0
SAL_NA9046AA	2015LSAL04158	Derby	0	0	0	0	0	0	0	0	0	0	0
SAL_NA9049AA	2016LSAL00028	Derby	0	0	0	0	0	0	0	0	0	0	0
SAL_NA9050AA	2014LSAL03355	Derby	0	0	0	0	0	0	0	0	0	0	0
SAL_NA9051AA	2014LSAL03356	Derby	0	0	0	0	0	0	0	0	0	0	0
SAL_NA9052AA	2014LSAL03348	Derby	0	0	0	0	0	0	0	0	0	0	0
SAL_NA9056AA	2014LSAL03383	Derby	0	0	0	0	0	0	0	0	0	0	0
SAL_NA9055AA	2014LSAL03393	Derby	0	0	0	0	0	0	0	0	0	0	0
SAL_NA9033AA	2014LSAL03407	Derby	0	0	0	0	0	0	0	0	0	0	0
SAL_NA9021AA	2014LSAL00762	Derby	0	0	0	0	0	0	0	0	0	0	0
SAL_NA9035AA	2014LSAL03410	Derby	0	0	0	0	0	0	0	0	0	0	0
SAL_NA9034AA	2014LSAL03414	Derby	0	0	0	0	0	0	0	0	0	0	0
SAL_NA9036AA	2014LSAL03420	Derby	0	0	0	0	0	0	0	0	0	0	0
SAL_NA9054AA	2014LSAL03390	Derby	0	0	0	0	0	0	0	0	0	0	0
SAL_NA9037AA	2014LSAL03416	Derby	0	0	0	0	0	0	0	0	0	0	0
SAL_NA9048AA	2016LSAL00056	Derby	0	0	0	0	0	0	0	0	0	0	0
SAL_NA9038AA	2014LSAL03465	Derby	0	0	0	0	0	0	0	0	0	0	0
SAL_NA9053AA	2014LSAL03350	Derby	0	0	0	0	0	0	0	0	0	0	0
SAL_NA9042AA	2015LSAL04562	Derby	0	0	0	0	0	0	0	0	0	0	0
SAL_NA9047AA	2015LSAL04018	Derby	0	0	0	0	0	0	0	0	0	0	0
SAL_NA9041AA	2015LSAL04225	Derby	0	0	0	0	0	0	0	0	0	0	0
SAL_NA9020AA	2015LSAL03550	Derby	0	0	0	0	0	0	0	0	0	0	0
SAL_NA8932AA	CFSAN075285	Derby	0	0	0	0	0	0	0	0	0	0	0
SAL_NA8931AA	CFSAN075284	Derby	0	0	0	0	0	0	0	0	0	0	0
SAL_NA8776AA	FSIS11807263	Derby	0	0	0	0	0	0	0	0	0	0	0
SAL_NA8811AA	FSIS11807222	Derby	0	0	0	0	0	0	0	0	0	0	0
SAL_NA8777AA	FSIS11807261	Derby	0	0	0	0	0	0	0	0	0	0	0
SAL_NA8593AA	ADRDL-1847	Derby	0	0	0	0	0	0	0	0	0	0	0
SAL_NA9522AA	FSIS11807373	Derby	0	0	0	0	0	0	0	0	0	0	0
SAL_NA9132AA	2014LSAL03973	Derby	0	0	0	0	0	0	0	0	0	0	0
SAL_NA9334AA	ADRDL-1844	Derby	0	0	0	0	0	0	0	0	0	0	0
SAL_NA9646AA	FSIS11807400	Derby	0	0	0	0	0	0	0	0	0	0	0
SAL_OA0157AA	FSIS11807361	Derby	0	0	0	0	0	0	0	0	0	0	0
SAL_OA0047AA	PNUSAS030676	Derby	0	0	0	0	0	0	0	0	0	0	0
SAL_NA9963AA	FSIS11807594	Derby	0	0	0	0	0	0	0	0	0	0	0
SAL_NA9815AA	PNUSAS031987	Derby	0	0	0	0	0	0	0	0	0	0	0
SAL_OA0194AA	FSIS31800137	Derby	0	0	0	0	0	0	0	0	0	0	0
SAL_OA0237AA	PNUSAS034106	Derby	0	0	0	0	0	0	0	0	0	0	0
SAL_OA0568AA	FSIS11807543	Derby	0	0	0	0	0	0	0	0	0	0	0
SAL_OA0574AA	FSIS11807542	Derby	0	0	0	0	0	0	0	0	0	0	0
SAL_OA0785AA	FSIS11807716	Derby	0	0	0	0	0	0	0	0	0	0	0
SAL_OA0779AA	FSIS11807713	Derby	0	0	0	0	0	0	0	0	0	0	0
SAL_OA0966AA	FSIS21821116	Derby	0	0	0	0	0	0	0	0	0	0	0
SAL_OA1169AA	PNUSAS034562	Derby	0	0	0	0	0	0	0	0	0	0	0
SAL_OA0986AA	CFSAN075480	Derby	0	0	0	0	0	0	0	0	0	0	0
SAL_OA1549AA	FDA00012549	Derby	0	0	0	0	0	0	0	0	0	0	0
SAL_OA1741AA	8-11-18-8100125-Z	Derby	0	0	0	0	0	0	0	0	0	0	0
SAL_OA4215AA	18EP000399	Derby	0	0	0	0	0	0	0	0	0	0	0
SAL_OA5862AA	PNUSAS035072	Derby	0	0	0	0	0	0	0	0	0	0	0
SAL_OA7399AA	PNUSAS036201	Derby	0	0	0	0	0	0	0	0	0	0	0
SAL_OA7454AA	FSIS11808589	Derby	0	0	0	0	0	0	0	0	0	0	0
SAL_OA4360AA	FDA00012753	Derby	0	0	0	0	0	0	0	0	0	0	0
SAL_OA3755AA	FSIS11808091	Derby	0	0	0	0	0	0	0	0	0	0	0
SAL_OA8128AA	OH-18-1327	Derby	0	0	0	0	0	0	0	0	0	0	0
SAL_PA0458AA	FSIS11808789	Derby	0	0	0	0	0	0	0	0	0	0	0
SAL_PA0523AA	CFSAN064152	Derby	0	0	0	0	0	0	0	0	0	0	0
SAL_PA0498AA	CFSAN064153	Derby	0	0	0	0	0	0	0	0	0	0	0
SAL_PA0511AA	CFSAN064154	Derby	0	0	0	0	0	0	0	0	0	0	0
SAL_PA0473AA	CFSAN075264	Derby	0	0	0	0	0	0	0	0	0	0	0
SAL_PA0474AA	CFSAN075265	Derby	0	0	0	0	0	0	0	0	0	0	0
SAL_PA0855AA	PNUSAS037623	Derby	0	0	0	0	0	0	0	0	0	0	0
SAL_PA1336AA	PNUSAS038100	Derby	0	0	0	0	0	0	0	0	0	0	0
SAL_PA1843AA	CFSAN078681	Derby	0	0	0	0	0	0	0	0	0	0	0
SAL_PA2088AA	CFSAN078671	Derby	0	0	0	0	0	0	0	0	0	0	0
SAL_PA1557AA	OH-17-26788	Derby	0	0	0	0	0	0	0	0	0	0	0
SAL_PA2967AA	FSIS11809213	Derby	0	0	0	0	0	0	0	0	0	0	0
SAL_PA2957AA	FSIS11809211	Derby	0	0	0	0	0	0	0	0	0	0	0
SAL_PA3421AA	PNUSAS038889	Derby	0	0	0	0	0	0	0	0	0	0	0
SAL_PA4480AA	PNUSAS034418	Derby	0	0	0	0	0	0	0	0	0	0	0
SAL_PA4556AA	FSIS11809553	Derby	0	0	0	0	0	0	0	0	0	0	0

SAL_PA4557AA	FSIS11809549	Derby	0	0	0	0	0	0	0	0	0	0	0
SAL_PA4554AA	FSIS11809557	Derby	0	0	0	0	0	0	0	0	0	0	0
SAL_PA4633AA	;almonella_Derby_252-1	Derby	0	0	0	0	0	0	0	0	0	0	0
SAL_PA4750AA	FSIS21821505	Derby	0	0	0	0	0	0	0	0	0	0	0
SAL_PA5021AA	CFSAN080426	Derby	0	0	0	0	0	0	0	0	0	0	0
SAL_PA5005AA	CFSAN080425	Derby	0	0	0	0	0	0	0	0	0	0	0
SAL_PA5430AA	PNUSAS039691	Derby	0	0	0	0	0	0	0	0	0	0	0
SAL_PA5486AA	FSIS11809590	Derby	0	0	0	0	0	0	0	0	0	0	0
SAL_PA5558AA	FSIS11809576	Derby	0	0	0	0	0	0	0	0	0	0	0
SAL_PA5664AA	PNUSAS040379	Derby	0	0	0	0	0	0	0	0	0	0	0
SAL_PA5983AA	PNUSAS040189	Derby	0	0	0	0	0	0	0	0	0	0	0
SAL_PA5858AA	PNUSAS040350	Derby	0	0	0	0	0	0	0	0	0	0	0
SAL_PA6451AA	FSIS21821623	Derby	0	0	0	0	0	0	0	0	0	0	0
SAL_PA7227AA	FSIS11809872	Derby	0	0	0	0	0	0	0	0	0	0	0
SAL_PA7228AA	FSIS11809869	Derby	0	0	0	0	0	0	0	0	0	0	0
SAL_PA7546AA	168592	Derby	0	0	0	0	0	0	0	0	0	0	0
SAL_PA7547AA	420640	Derby	0	0	0	0	0	0	0	0	0	0	0
SAL_PA7548AA	508304	Derby	0	0	0	0	0	0	0	0	0	0	0
SAL_PA7549AA	252403	Derby	0	0	0	0	0	0	0	0	0	0	0
SAL_PA7551AA	419075	Derby	0	0	0	0	0	0	0	0	0	0	0
SAL_PA7552AA	252405	Derby	0	0	0	0	0	0	0	0	0	0	0
SAL_PA7550AA	261932	Derby	0	0	0	0	0	0	0	0	0	0	0
SAL_PA7555AA	136566	Derby	0	0	0	0	0	0	0	0	0	0	0
SAL_PA7556AA	524382	Derby	0	0	0	0	0	0	0	0	0	0	0
SAL_PA7557AA	371811	Derby	0	0	0	0	0	0	0	0	0	0	0
SAL_PA7558AA	399359	Derby	0	0	0	0	0	0	0	0	0	0	0
SAL_PA7559AA	401716	Derby	0	0	0	0	0	0	0	0	0	0	0
SAL_PA7561AA	414730	Derby	0	0	0	0	0	0	0	0	0	0	0
SAL_PA7562AA	471112	Derby	0	0	0	0	0	0	0	0	0	0	0
SAL_PA7541AA	367238	Derby	0	0	0	0	0	0	0	0	0	0	0
SAL_PA7542AA	459975	Derby	0	0	0	0	0	0	0	0	0	0	0
SAL_PA7544AA	424695	Derby	0	0	0	0	0	0	0	0	0	0	0
SAL_PA7543AA	150987	Derby	0	0	0	0	0	0	0	0	0	0	0
SAL_PA7545AA	377335	Derby	0	0	0	0	0	0	0	0	0	0	0
SAL_PA7655AA	FSIS11809749	Derby	0	0	0	0	0	0	0	0	0	0	0
SAL_PA7813AA	500411	Derby	0	0	0	0	0	0	0	0	0	0	0
SAL_PA8448AA	1884-2	Derby	0	0	0	0	0	0	0	0	0	0	0
SAL_PA8450AA	1887-3	Derby	0	0	0	0	0	0	0	0	0	0	0
SAL_PA8458AA	1888-2	Derby	0	0	0	0	0	0	0	0	0	0	0
SAL_PA8315AA	FSIS11808546	Derby	0	0	0	0	0	0	0	0	0	0	0
SAL_PA8297AA	FSIS11809964	Derby	0	0	0	0	0	0	0	0	0	0	0
SAL_PA8821AA	FDA00000431	Derby	0	0	0	0	0	0	0	0	0	0	0
SAL_PA8164AA	PNUSAS041340	Derby	0	0	0	0	0	0	0	0	0	0	0
SAL_PA9315AA	PNUSAS040919	Derby	0	0	0	0	0	0	0	0	0	0	0
SAL_PA9220AA	PNUSAS034516	Derby	0	0	0	0	0	0	0	0	0	0	0
SAL_PA9116AA	FSIS31800466	Derby	0	0	0	0	0	0	0	0	0	0	0
SAL_PA9376AA	FSIS11808795	Derby	0	0	0	0	0	0	0	0	0	0	0
SAL_PA9357AA	FSIS11809861	Derby	0	0	0	0	0	0	0	0	0	0	0
SAL_PA9108AA	FSIS31800441	Derby	0	0	0	0	0	0	0	0	0	0	0
SAL_PA9570AA	FSIS21821733	Derby	0	0	0	0	0	0	0	0	0	0	0
SAL_PA9661AA	FSIS11810052	Derby	0	0	0	0	0	0	0	0	0	0	0
SAL_QA0194AA	PNUSAS042134	Derby	0	0	0	0	0	0	0	0	0	0	0
SAL_QA0587AA	PNUSAS042219	Derby	0	0	0	0	0	0	0	0	0	0	0
SAL_QA0719AA	FSIS11810089	Derby	0	0	0	0	0	0	0	0	0	0	0
SAL_QA0642AA	FSIS21821744	Derby	0	0	0	0	0	0	0	0	0	0	0
SAL_QA1248AA	195644	Derby	0	0	0	0	0	0	0	0	0	0	0
SAL_QA1249AA	492548	Derby	0	0	0	0	0	0	0	0	0	0	0
SAL_QA0758AA	PNUSAS042238	Derby	0	0	0	0	0	0	0	0	0	0	0
SAL_QA1245AA	435456	Derby	0	0	0	0	0	0	0	0	0	0	0
SAL_QA1246AA	372703	Derby	0	0	0	0	0	0	0	0	0	0	0
SAL_QA1247AA	253799	Derby	0	0	0	0	0	0	0	0	0	0	0
SAL_QA1250AA	358034	Derby	0	0	0	0	0	0	0	0	0	0	0
SAL_QA1223AA	FSIS31800503	Derby	0	0	0	0	0	0	0	0	0	0	0
SAL_QA1142AA	FSIS11810194	Derby	0	0	0	0	0	0	0	0	0	0	0
SAL_QA1143AA	FSIS11810188	Derby	0	0	0	0	0	0	0	0	0	0	0
SAL_QA1149AA	FSIS11810200	Derby	0	0	0	0	0	0	0	0	0	0	0
SAL_QA1109AA	PNUSAS042395	Derby	0	0	0	0	0	0	0	0	0	0	0
SAL_QA1076AA	PNUSAS042364	Derby	0	0	0	0	0	0	0	0	0	0	0
SAL_QA4982AA	25-5-18-8100523-V	Derby	0	0	0	0	0	0	0	0	0	0	0
SAL_QA4980AA	25-4-18-8100522-M	Derby	0	0	0	0	0	0	0	0	0	0	0
SAL_QA2934AA	FSIS11810459	Derby	0	0	0	0	0	0	0	0	0	0	0
SAL_QA3640AA	PNUSAS043182	Derby	0	0	0	0	0	0	0	0	0	0	0
SAL_QA6664AA	26-17-18-8100550-G	Derby	0	0	0	0	0	0	0	0	0	0	0

SAL_QA4582AA	FDA00001225	Derby	0	0	0	0	0	0	0	0	0	0	0
SAL_QA4606AA	FSIS11810423	Derby	0	0	0	0	0	0	0	0	0	0	0
SAL_QA5928AA	FSIS11810651	Derby	0	0	0	0	0	0	0	0	0	0	0
SAL_QA5932AA	FSIS11810652	Derby	0	0	0	0	0	0	0	0	0	0	0
SAL_QA6080AA	PNUSAS041295	Derby	0	0	0	0	0	0	0	0	0	0	0
SAL_QA6380AA	TTU_072	Derby	0	0	0	0	0	0	0	0	0	0	0
SAL_QA7076AA	PNUSAS043239	Derby	0	0	0	0	0	0	0	0	0	0	0
SAL_QA7003AA	PNUSAS042046	Derby	0	0	0	0	0	0	0	0	0	0	0
SAL_QA8150AA	CFSAN082351	Derby	0	0	0	0	0	0	0	0	0	0	0
SAL_QA8147AA	CFSAN082352	Derby	0	0	0	0	0	0	0	0	0	0	0
SAL_QA8522AA	FSIS11810836	Derby	0	0	0	0	0	0	0	0	0	0	0
SAL_QA8961AA	PNUSAS044581	Derby	0	0	0	0	0	0	0	0	0	0	0
SAL_RA0177AA	559796	Derby	0	0	0	0	0	0	0	0	0	0	0
SAL_RA3871AA	CFSAN082347	Derby	0	0	0	0	0	0	0	0	0	0	0
SAL_RA4352AA	550818	Derby	0	0	0	0	0	0	0	0	0	0	0
SAL_RA4328AA	559061	Derby	0	0	0	0	0	0	0	0	0	0	0
SAL_RA6106AA	CFSAN082966	Derby	0	0	0	0	0	0	0	0	0	0	0
SAL_RA5810AA	562957	Derby	0	0	0	0	0	0	0	0	0	0	0
SAL_RA5780AA	CFSAN082958	Derby	0	0	0	0	0	0	0	0	0	0	0
SAL_RA5754AA	CFSAN082935	Derby	0	0	0	0	0	0	0	0	0	0	0
SAL_RA5682AA	561011	Derby	0	0	0	0	0	0	0	0	0	0	0
SAL_RA5593AA	554407	Derby	0	0	0	0	0	0	0	0	0	0	0
SAL_RA5603AA	559060	Derby	0	0	0	0	0	0	0	0	0	0	0
SAL_RA5543AA	561104	Derby	0	0	0	0	0	0	0	0	0	0	0
SAL_RA2671AA	373206	Derby	0	0	0	0	0	0	0	0	0	0	0
SAL_RA6653AA	548545	Derby	0	0	0	0	0	0	0	0	0	0	0
SAL_SA1387AA	DP_P7	Derby	0	0	0	0	0	0	0	0	0	0	0
SAL_SA1388AA	DP_P11	Derby	0	0	0	0	0	0	0	0	0	0	0
SAL_SA1386AA	DP_P5	Derby	0	0	0	0	0	0	0	0	0	0	0
SAL_SA1396AA	DP_T85	Derby	0	0	0	0	0	0	0	0	0	0	0
SAL_SA1393AA	DP_P38	Derby	0	0	0	0	0	0	0	0	0	0	0
SAL_SA1421AA	DB_15	Derby	0	0	0	0	0	0	0	0	0	0	0
SAL_SA2016AA	0501M40957	Derby	0	0	0	0	0	0	0	0	0	0	0
SAL_SA1865AA	SF_R0376/09	Derby	0	0	0	0	0	0	0	0	0	0	0
SAL_SA1864AA	SF_R0375/09	Derby	0	0	0	0	0	0	0	0	0	0	0
SAL_SA1863AA	SF_R0373/09	Derby	0	0	0	0	0	0	0	0	0	0	0
SAL_RA6844AA	FSIS11811745	Derby	0	0	0	0	0	0	0	0	0	0	0
SAL_RA7483AA	568382	Derby	0	0	0	0	0	0	0	0	0	0	0
SAL_RA7585AA	PNUSAS045160	Derby	0	0	0	0	0	0	0	0	0	0	0
SAL_RA7636AA	FSIS11811945	Derby	0	0	0	0	0	0	0	0	0	0	0
SAL_RA7651AA	FSIS11811959	Derby	0	0	0	0	0	0	0	0	0	0	0
SAL_RA7634AA	FSIS11811941	Derby	0	0	0	0	0	0	0	0	0	0	0
SAL_RA8133AA	549516	Derby	0	0	0	0	0	0	0	0	0	0	0
SAL_RA7921AA	FSIS11811993	Derby	0	0	0	0	0	0	0	0	0	0	0
SAL_RA8166AA	FSIS11810867	Derby	0	0	0	0	0	0	0	0	0	0	0
SAL_RA8246AA	114059	Derby	0	0	0	0	0	0	0	0	0	0	0
SAL_RA8422AA	FSIS11811970	Derby	0	0	0	0	0	0	0	0	0	0	0
SAL_RA8677AA	FSIS11812176	Derby	0	0	0	0	0	0	0	0	0	0	0
SAL_RA8663AA	FSIS11812219	Derby	0	0	0	0	0	0	0	0	0	0	0
SAL_RA8682AA	FSIS11812171	Derby	0	0	0	0	0	0	0	0	0	0	0
SAL_RA8705AA	FSIS11812197	Derby	0	0	0	0	0	0	0	0	0	0	0
SAL_RA8744AA	FSIS11812167	Derby	0	0	0	0	0	0	0	0	0	0	0
SAL_RA8713AA	FSIS11811635	Derby	0	0	0	0	0	0	0	0	0	0	0
SAL_RA8715AA	FSIS11811625	Derby	0	0	0	0	0	0	0	0	0	0	0
SAL_RA9278AA	CFSAN084125	Derby	0	0	0	0	0	0	0	0	0	0	0
SAL_RA9407AA	CFSAN084059	Derby	0	0	0	0	0	0	0	0	0	0	0
SAL_RA9749AA	CFSAN084187	Derby	0	0	0	0	0	0	0	0	0	0	0
SAL_RA9875AA	FDA00004833	Derby	0	0	0	0	0	0	0	0	0	0	0
SAL_RA9866AA	CFSAN084230	Derby	0	0	0	0	0	0	0	0	0	0	0
SAL_RA9870AA	CFSAN084225	Derby	0	0	0	0	0	0	0	0	0	0	0
SAL_RA9916AA	FSIS11812472	Derby	0	0	0	0	0	0	0	0	0	0	0
SAL_SA0197AA	CVM N17S429	Derby	0	0	0	0	0	0	0	0	0	0	0
SAL_SA0317AA		Derby	0	0	0	0	0	0	0	0	0	0	0
SAL_SA0024AA	CFSAN082346	Derby	0	0	0	0	0	0	0	0	0	0	0
SAL_RA9983AA	FSIS11812706	Derby	0	0	0	0	0	0	0	0	0	0	0
SAL_RA9982AA	FSIS11812704	Derby	0	0	0	0	0	0	0	0	0	0	0
SAL_RA9977AA	FSIS11812460	Derby	0	0	0	0	0	0	0	0	0	0	0
SAL_SA0573AA	FSIS11812547	Derby	0	0	0	0	0	0	0	0	0	0	0
SAL_SA0582AA	FSIS11812690	Derby	0	0	0	0	0	0	0	0	0	0	0
SAL_SA0846AA	FSIS11812713	Derby	0	0	0	0	0	0	0	0	0	0	0
SAL_SA0857AA	FSIS11812733	Derby	0	0	0	0	0	0	0	0	0	0	0
SAL_SA0971AA	FSIS11812819	Derby	0	0	0	0	0	0	0	0	0	0	0
SAL_SA0900AA	FSIS11812676	Derby	0	0	0	0	0	0	0	0	0	0	0

SAL_SA0996AA	FSIS31800794	Derby	0	0	0	0	0	0	0	0	0	0	0
SAL_SA0899AA	FSIS11812679	Derby	0	0	0	0	0	0	0	0	0	0	0
SAL_SA2615AA	FSIS11812975	Derby	0	0	0	0	0	0	0	0	0	0	0
SAL_SA2620AA	FSIS11812967	Derby	0	0	0	0	0	0	0	0	0	0	0
SAL_SA2789AA	9e7-e3ce-4bed-8134-3e8	Derby	0	0	0	0	0	0	0	0	0	0	0
SAL_SA2857AA	FSIS11813188	Derby	0	0	0	0	0	0	0	0	0	0	0
SAL_SA2870AA	FSIS11813207	Derby	0	0	0	0	0	0	0	0	0	0	0
SAL_SA3241AA		Derby	0	0	0	0	0	0	0	0	0	0	0
SAL_SA3316AA	OH-18-18132-9	Derby	0	0	0	0	0	0	0	0	0	0	0
SAL_SA3390AA	FDA00004911	Derby	0	0	0	0	0	0	0	0	0	0	0
SAL_SA3626AA	FSIS21822032	Derby	0	0	0	0	0	0	0	0	0	0	0
SAL_SA3609AA	FSIS31800951	Derby	0	0	0	0	0	0	0	0	0	0	0
SAL_SA3632AA	ADRDL-1947	Derby	0	0	0	0	0	0	0	0	0	0	0
SAL_SA3642AA	FSIS11813675	Derby	0	0	0	0	0	0	0	0	0	0	0
SAL_SA3752AA	FSIS11813402	Derby	0	0	0	0	0	0	0	0	0	0	0
SAL_SA3743AA	FSIS11813431	Derby	0	0	0	0	0	0	0	0	0	0	0
SAL_SA3716AA	FSIS11813424	Derby	0	0	0	0	0	0	0	0	0	0	0
SAL_SA5457AA	MC_04-0048	Derby	0	0	0	0	0	0	0	0	0	0	0
SAL_SA4958AA	CIT_T56	Derby	0	0	0	0	0	0	0	0	0	0	0
SAL_SA4367AA	FSIS31801020	Derby	0	0	0	0	0	0	0	0	0	0	0
SAL_SA4325AA	FSIS31801015	Derby	0	0	0	0	0	0	0	0	0	0	0
SAL_SA6533AA	962SIS2018-1T	Derby	0	0	0	0	0	0	0	0	0	0	0
SAL_SA4919AA	575119	Derby	0	0	0	0	0	0	0	0	0	0	0
SAL_SA6016AA	598136	Derby	0	0	0	0	0	0	0	0	0	0	0
SAL_SA5962AA	592634	Derby	0	0	0	0	0	0	0	0	0	0	0
SAL_SA5909AA	592663	Derby	0	0	0	0	0	0	0	0	0	0	0
SAL_SA5813AA	598898	Derby	0	0	0	0	0	0	0	0	0	0	0
SAL_SA6753AA	575117	Derby	0	0	0	0	0	0	0	0	0	0	0
SAL_SA7201AA	588914	Derby	0	0	0	0	0	0	0	0	0	0	0
SAL_SA7158AA	576370	Derby	0	0	0	0	0	0	0	0	0	0	0
SAL_SA6886AA		Derby	0	0	0	0	0	0	0	0	0	0	0
SAL_SA7361AA	FSIS11813696	Derby	0	0	0	0	0	0	0	0	0	0	0
SAL_SA9354AA	963SIS2018-1T	Derby	0	0	0	0	0	0	0	0	0	0	0
SAL_SA9160AA	CVM N17S1441	Derby	0	0	0	0	0	0	0	0	0	0	0
SAL_TA0139AA	FSIS11814468	Derby	0	0	0	0	0	0	0	0	0	0	0
SAL_TA0094AA	FSIS11814466	Derby	0	0	0	0	0	0	0	0	0	0	0
SAL_TA0357AA	608498	Derby	0	0	0	0	0	0	0	0	0	0	0
SAL_TA1352AA	FSIS31801136	Derby	0	0	0	0	0	0	0	0	0	0	0
SAL_TA1282AA	OH-18-19201	Derby	0	0	0	0	0	0	0	0	0	0	0
SAL_TA1395AA	FSIS11814629	Derby	0	0	0	0	0	0	0	0	0	0	0
SAL_TA1802AA	71409.04	Derby	0	0	0	0	0	0	0	0	0	0	0
SAL_TA2073AA	SMG-18-2078	Derby	0	0	0	0	0	0	0	0	0	0	0
SAL_TA2264AA	620417	Derby	0	0	0	0	0	0	0	0	0	0	0
SAL_TA2713AA	623493	Derby	0	0	0	0	0	0	0	0	0	0	0
SAL_TA2656AA	FSIS11814835	Derby	0	0	0	0	0	0	0	0	0	0	0
SAL_TA2661AA	FSIS11814819	Derby	0	0	0	0	0	0	0	0	0	0	0
SAL_TA2931AA	621748	Derby	0	0	0	0	0	0	0	0	0	0	0
SAL_TA3018AA	FSIS21822482	Derby	0	0	0	0	0	0	0	0	0	0	0
SAL_TA3022AA	FSIS21822481	Derby	0	0	0	0	0	0	0	0	0	0	0
SAL_TA5369AA	FSIS31801181	Derby	0	0	0	0	0	0	0	0	0	0	0
SAL_TA5371AA	FSIS31801169	Derby	0	0	0	0	0	0	0	0	0	0	0
SAL_TA6020AA	FSIS21822532	Derby	0	0	0	0	0	0	0	0	0	0	0
SAL_TA6045AA	FSIS11815146	Derby	0	0	0	0	0	0	0	0	0	0	0
SAL_TA6058AA	FSIS11815283	Derby	0	0	0	0	0	0	0	0	0	0	0
SAL_TA6062AA	FSIS11815279	Derby	0	0	0	0	0	0	0	0	0	0	0
SAL_TA6323AA	6	Derby	0	0	0	0	0	0	0	0	0	0	0
SAL_TA6319AA	3	Derby	0	0	0	0	0	0	0	0	0	0	0
SAL_TA6321AA	7	Derby	0	0	0	0	0	0	0	0	0	0	0
SAL_TA6331AA	2	Derby	0	0	0	0	0	0	0	0	0	0	0
SAL_TA6329AA	1	Derby	0	0	0	0	0	0	0	0	0	0	0
SAL_TA6328AA	4	Derby	0	0	0	0	0	0	0	0	0	0	0
SAL_TA6335AA	15	Derby	0	0	0	0	0	0	0	0	0	0	0
SAL_TA6336AA	20	Derby	0	0	0	0	0	0	0	0	0	0	0
SAL_TA6355AA	28	Derby	0	0	0	0	0	0	0	0	0	0	0
SAL_TA6367AA	50	Derby	0	0	0	0	0	0	0	0	0	0	0
SAL_TA6376AA	65	Derby	0	0	0	0	0	0	0	0	0	0	0
SAL_TA6378AA	66	Derby	0	0	0	0	0	0	0	0	0	0	0
SAL_TA6385AA	72	Derby	0	0	0	0	0	0	0	0	0	0	0
SAL_TA6392AA	73	Derby	0	0	0	0	0	0	0	0	0	0	0
SAL_TA6404AA	90	Derby	0	0	0	0	0	0	0	0	0	0	0
SAL_TA6467AA	Salmonella enterica	Derby	0	0	0	0	0	0	0	0	0	0	0
SAL_TA6703AA	FSIS11815314	Derby	0	0	0	0	0	0	0	0	0	0	0
SAL_TA6805AA	FSIS11815518	Derby	0	0	0	0	0	0	0	0	0	0	0

SAL_TA6806AA	FSIS21822628	Derby	0	0	0	0	0	0	0	0	0	0	0
SAL_TA6780AA	FSIS11815498	Derby	0	0	0	0	0	0	0	0	0	0	0
SAL_TA6784AA	FSIS11815508	Derby	0	0	0	0	0	0	0	0	0	0	0
SAL_TA7030AA	CFSAN087763	Derby	0	0	0	0	0	0	0	0	0	0	0
SAL_TA7003AA	CFSAN087752	Derby	0	0	0	0	0	0	0	0	0	0	0
SAL_TA7249AA	CFSAN087797	Derby	0	0	0	0	0	0	0	0	0	0	0
SAL_TA7332AA	FSIS11815623	Derby	0	0	0	0	0	0	0	0	0	0	0
SAL_TA7462AA	47178	Derby	0	0	0	0	0	0	0	0	0	0	0
SAL_TA7466AA	SENASICA_Sa_U11	Derby	0	0	0	0	0	0	0	0	0	0	0
SAL_PA7554AA	406663	Derby	0	0	0	0	0	0	0	0	0	0	0
SAL_PA1569AA	OH-17-26790	Derby	0	0	0	0	0	0	0	0	0	0	0
SAL_PA2527AA	FSIS11808464	Derby	0	0	0	0	0	0	0	0	0	0	0
SAL_TA7752AA	R16.4830	Derby	0	0	0	0	0	0	0	0	0	0	0
SAL_TA7727AA	R17.2509	Derby	0	0	0	0	0	0	0	0	0	0	0
SAL_TA7693AA	R17.3585	Derby	0	0	0	0	0	0	0	0	0	0	0
SAL_TA7674AA	R17.1236	Derby	0	0	0	0	0	0	0	0	0	0	0
SAL_TA7625AA	R16.1871	Derby	0	0	0	0	0	0	0	0	0	0	0
SAL_TA7626AA	R16.2078	Derby	0	0	0	0	0	0	0	0	0	0	0
SAL_TA7584AA	R17.4007	Derby	0	0	0	0	0	0	0	0	0	0	0
SAL_TA7522AA	R17.6030	Derby	0	0	0	0	0	0	0	0	0	0	0
SAL_TA7530AA	R15.2004	Derby	0	0	0	0	0	0	0	0	0	0	0
SAL_TA7533AA	R15.1399	Derby	0	0	0	0	0	0	0	0	0	0	0
SAL_OA1402AA	FSIS11807704	Derby	0	0	0	0	0	0	0	0	0	0	0
SAL_OA1533AA	SAL-17-VL-OH-WA-0020	Derby	0	0	0	0	0	0	0	0	0	0	0
SAL_TA7857AA	l samples of swine - sero	Derby	0	0	0	0	0	0	0	0	0	0	0
SAL_NA5189AA	CFSAN074280	Derby	0	0	0	0	0	0	0	0	0	0	0
SAL_NA5198AA	CFSAN074322	Derby	0	0	0	0	0	0	0	0	0	0	0
SAL_NA5210AA	CFSAN074328	Derby	0	0	0	0	0	0	0	0	0	0	0
SAL_NA5212AA	CFSAN074324	Derby	0	0	0	0	0	0	0	0	0	0	0
SAL_NA5214AA	CFSAN074325	Derby	0	0	0	0	0	0	0	0	0	0	0
SAL_OA1443AA	FSIS11807270	Derby	0	0	0	0	0	0	0	0	0	0	0
SAL_TA8102AA	SMG-18-2231	Derby	0	0	0	0	0	0	0	0	0	0	0
SAL_TA8482AA	636365	Derby	0	0	0	0	0	0	0	0	0	0	0
SAL_TA8424AA	FSIS11815719	Derby	0	0	0	0	0	0	0	0	0	0	0
SAL_TA8259AA	FSIS21822749	Derby	0	0	0	0	0	0	0	0	0	0	0
SAL_TA8219AA	ADRDL-2268	Derby	0	0	0	0	0	0	0	0	0	0	0
SAL_TA8194AA	ADRDL-2254	Derby	0	0	0	0	0	0	0	0	0	0	0
SAL_TA8801AA	FSIS11815726	Derby	0	0	0	0	0	0	0	0	0	0	0
SAL_TA8857AA	JB10A07	Derby	0	0	0	0	0	0	0	0	0	0	0
SAL_TA8859AA	NA10H35	Derby	0	0	0	0	0	0	0	0	0	0	0
SAL_OA3628AA	L03123-16	Derby	0	0	0	0	0	0	0	0	0	0	0
SAL_OA2118AA	FSIS11807878	Derby	0	0	0	0	0	0	0	0	0	0	0
SAL_OA3763AA	FSIS31800238	Derby	0	0	0	0	0	0	0	0	0	0	0
SAL_OA1415AA	FSIS11807699	Derby	0	0	0	0	0	0	0	0	0	0	0
SAL_NA5219AA	CFSAN074332	Derby	0	0	0	0	0	0	0	0	0	0	0
SAL_NA5218AA	CFSAN074326	Derby	0	0	0	0	0	0	0	0	0	0	0
SAL_OA1423AA	FSIS11807691	Derby	0	0	0	0	0	0	0	0	0	0	0
SAL_TA8940AA	639821	Derby	0	0	0	0	0	0	0	0	0	0	0
SAL_OA1828AA	ADRDL-1822	Derby	0	0	0	0	0	0	0	0	0	0	0
SAL_OA2166AA	ADRDL-1668	Derby	0	0	0	0	0	0	0	0	0	0	0
SAL_OA2380AA	CFSAN075546	Derby	0	0	0	0	0	0	0	0	0	0	0
SAL_OA2361AA	FSIS21821134	Derby	0	0	0	0	0	0	0	0	0	0	0
SAL_OA3066AA	S03927-11	Derby	0	0	0	0	0	0	0	0	0	0	0
SAL_OA5640AA	FSIS11808301	Derby	0	0	0	0	0	0	0	0	0	0	0
SAL_OA5656AA	FSIS21821281	Derby	0	0	0	0	0	0	0	0	0	0	0
SAL_OA5203AA	PNUSAS036292	Derby	0	0	0	0	0	0	0	0	0	0	0
SAL_PA1560AA	OH-17-26789	Derby	0	0	0	0	0	0	0	0	0	0	0
SAL_PA1788AA	FSIS11809079	Derby	0	0	0	0	0	0	0	0	0	0	0
SAL_PA1948AA	CFSAN078651	Derby	0	0	0	0	0	0	0	0	0	0	0
SAL_PA2166AA	FDA00012955	Derby	0	0	0	0	0	0	0	0	0	0	0
SAL_PA2551AA	FSIS11808503	Derby	0	0	0	0	0	0	0	0	0	0	0
SAL_PA2863AA	FSIS21821479	Derby	0	0	0	0	0	0	0	0	0	0	0
SAL_PA2992AA	PNUSAS035526	Derby	0	0	0	0	0	0	0	0	0	0	0
SAL_PA3123AA	40-fff3-11e6-a0c0-3c4a92	Derby	0	0	0	0	0	0	0	0	0	0	0
SAL_PA3120AA	10-fff3-11e6-a0c0-3c4a92	Derby	0	0	0	0	0	0	0	0	0	0	0
SAL_PA3121AA	40-fff3-11e6-a0c0-3c4a92	Derby	0	0	0	0	0	0	0	0	0	0	0
SAL_PA3139AA	70-fff3-11e6-a0c0-3c4a92	Derby	0	0	0	0	0	0	0	0	0	0	0
SAL_PA3521AA	CFSAN078669	Derby	0	0	0	0	0	0	0	0	0	0	0
SAL_PA3583AA	CFSAN075266	Derby	0	0	0	0	0	0	0	0	0	0	0
SAL_PA7553AA	424588	Derby	0	0	0	0	0	0	0	0	0	0	0
SAL_TA9635AA	FSIS11815936	Derby	0	0	0	0	0	0	0	0	0	0	0
SAL_TA9623AA	FSIS11815939	Derby	0	0	0	0	0	0	0	0	0	0	0
SAL_TA9541AA	FSIS11815932	Derby	0	0	0	0	0	0	0	0	0	0	0

SAL_TA9866AA	FSIS11816153	Derby	0	0	0	0	0	0	0	0	0	0	0
SAL_TA9888AA	FSIS11816119	Derby	0	0	0	0	0	0	0	0	0	0	0
SAL_TA9928AA	FSIS21824000	Derby	0	0	0	0	0	0	0	0	0	0	0
SAL_UA1635AA	2014LSAL01779	Derby	0	0	0	0	0	0	0	0	0	0	0
SAL_MA6033AA	SA20035215	Derby	0	0	0	0	0	0	0	0	0	0	0
SAL_UA1719AA	CFSAN088676	Derby	0	0	0	0	0	0	0	0	0	0	0
SAL_UA2642AA	647794	Derby	0	0	0	0	0	0	0	0	0	0	0
SAL_UA2704AA	648995	Derby	0	0	0	0	0	0	0	0	0	0	0
SAL_UA2754AA	647795	Derby	0	0	0	0	0	0	0	0	0	0	0
SAL_UA5143AA	FSIS11815924	Derby	0	0	0	0	0	0	0	0	0	0	0
SAL_UA6925AA	659358	Derby	0	0	0	0	0	0	0	0	0	0	0
SAL_UA6927AA	659340	Derby	0	0	0	0	0	0	0	0	0	0	0
SAL_UA6931AA	659368	Derby	0	0	0	0	0	0	0	0	0	0	0
SAL_UA6875AA	AG18-0123	Derby	0	0	0	0	0	0	0	0	0	0	0
SAL_UA6786AA	659350	Derby	0	0	0	0	0	0	0	0	0	0	0
SAL_UA7230AA	FSIS11816709	Derby	0	0	0	0	0	0	0	0	0	0	0
SAL_UA2997AA	648987	Derby	0	0	0	0	0	0	0	0	0	0	0
SAL_UA3594AA	Sa64	Derby	0	0	0	0	0	0	0	0	0	0	0
SAL_UA3706AA	649897	Derby	0	0	0	0	0	0	0	0	0	0	0
SAL_UA6829AA	CFSAN089912	Derby	0	0	0	0	0	0	0	0	0	0	0
SAL_UA4904AA	CFSAN089060	Derby	0	0	0	0	0	0	0	0	0	0	0
SAL_UA5139AA	FSIS11815926	Derby	0	0	0	0	0	0	0	0	0	0	0
SAL_UA5149AA	FSIS11816337	Derby	0	0	0	0	0	0	0	0	0	0	0
SAL_UA5326AA	651995	Derby	0	0	0	0	0	0	0	0	0	0	0
SAL_UA5415AA	FSIS21823011	Derby	0	0	0	0	0	0	0	0	0	0	0
SAL_UA5604AA	590877	Derby	0	0	0	0	0	0	0	0	0	0	0
SAL_UA5903AA	FSIS11816703	Derby	0	0	0	0	0	0	0	0	0	0	0
SAL_UA5933AA	FSIS11816500	Derby	0	0	0	0	0	0	0	0	0	0	0
SAL_UA5925AA	FSIS11816497	Derby	0	0	0	0	0	0	0	0	0	0	0
SAL_UA5968AA	654710	Derby	0	0	0	0	0	0	0	0	0	0	0
SAL_UA6006AA	654709	Derby	0	0	0	0	0	0	0	0	0	0	0
SAL_UA6179AA	FSIS21823025	Derby	0	0	0	0	0	0	0	0	0	0	0
SAL_UA6134AA	FSIS11816763	Derby	0	0	0	0	0	0	0	0	0	0	0
SAL_UA6501AA	FSIS21823042	Derby	0	0	0	0	0	0	0	0	0	0	0
SAL_UA7004AA	579283	Derby	0	0	0	0	0	0	0	0	0	0	0
SAL_UA6974AA	FSIS21823119	Derby	0	0	0	0	0	0	0	0	0	0	0
SAL_UA7839AA	CFSAN090579	Derby	0	0	0	0	0	0	0	0	0	0	0
SAL_UA7785AA	661023	Derby	0	0	0	0	0	0	0	0	0	0	0
SAL_UA7776AA	FSIS11816920	Derby	0	0	0	0	0	0	0	0	0	0	0
SAL_UA7746AA	FSIS11816982	Derby	0	0	0	0	0	0	0	0	0	0	0
SAL_UA7747AA	FSIS11816981	Derby	0	0	0	0	0	0	0	0	0	0	0
SAL_UA7714AA	AHD173	Derby	0	0	0	0	0	0	0	0	0	0	0
SAL_UA7729AA	AHD194	Derby	0	0	0	0	0	0	0	0	0	0	0
SAL_UA7665AA	AHD228	Derby	0	0	0	0	0	0	0	0	0	0	0
SAL_UA7680AA	AHD209	Derby	0	0	0	0	0	0	0	0	0	0	0
SAL_UA7484AA	FSIS11917059	Derby	0	0	0	0	0	0	0	0	0	0	0
SAL_UA8108AA	666301	Derby	0	0	0	0	0	0	0	0	0	0	0
SAL_UA8153AA	666303	Derby	0	0	0	0	0	0	0	0	0	0	0
SAL_UA8320AA	666306	Derby	0	0	0	0	0	0	0	0	0	0	0
SAL_UA8667AA	201400722	Derby	0	0	0	0	0	0	0	0	0	0	0
SAL_UA8669AA	201409231	Derby	0	0	0	0	0	0	0	0	0	0	0
SAL_UA8672AA	201409218	Derby	0	0	0	0	0	0	0	0	0	0	0
SAL_UA8673AA	201408798	Derby	0	0	0	0	0	0	0	0	0	0	0
SAL_UA8674AA	201409015	Derby	0	0	0	0	0	0	0	0	0	0	0
SAL_UA8675AA	201408715	Derby	0	0	0	0	0	0	0	0	0	0	0
SAL_UA8676AA	201408728	Derby	0	0	0	0	0	0	0	0	0	0	0
SAL_UA8647AA	201400414	Derby	0	0	0	0	0	0	0	0	0	0	0
SAL_UA8648AA	201500621	Derby	0	0	0	0	0	0	0	0	0	0	0
SAL_UA8649AA	201500629	Derby	0	0	0	0	0	0	0	0	0	0	0
SAL_UA8650AA	201500639	Derby	0	0	0	0	0	0	0	0	0	0	0
SAL_UA8651AA	201500733	Derby	0	0	0	0	0	0	0	0	0	0	0
SAL_UA8652AA	201500827	Derby	0	0	0	0	0	0	0	0	0	0	0
SAL_UA8653AA	201500915	Derby	0	0	0	0	0	0	0	0	0	0	0
SAL_UA8654AA	201500958	Derby	0	0	0	0	0	0	0	0	0	0	0
SAL_UA8655AA	201501250	Derby	0	0	0	0	0	0	0	0	0	0	0
SAL_UA8656AA	201507546	Derby	0	0	0	0	0	0	0	0	0	0	0
SAL_UA8657AA	201508312	Derby	0	0	0	0	0	0	0	0	0	0	0
SAL_UA8658AA	201508416	Derby	0	0	0	0	0	0	0	0	0	0	0
SAL_UA8659AA	201508420	Derby	0	0	0	0	0	0	0	0	0	0	0
SAL_UA8660AA	201508491	Derby	0	0	0	0	0	0	0	0	0	0	0
SAL_UA8662AA	201508609	Derby	0	0	0	0	0	0	0	0	0	0	0
SAL_UA8663AA	201508643	Derby	0	0	0	0	0	0	0	0	0	0	0
SAL_UA8665AA	201508783	Derby	0	0	0	0	0	0	0	0	0	0	0

SAL_UA8620AA	201502655	Derby	0	0	0	0	0	0	0	0	0	0	0
SAL_UA8621AA	201502624	Derby	0	0	0	0	0	0	0	0	0	0	0
SAL_UA8626AA	201508967	Derby	0	0	0	0	0	0	0	0	0	0	0
SAL_UA8627AA	201508799	Derby	0	0	0	0	0	0	0	0	0	0	0
SAL_UA8628AA	201507137	Derby	0	0	0	0	0	0	0	0	0	0	0
SAL_UA8629AA	201509065	Derby	0	0	0	0	0	0	0	0	0	0	0
SAL_UA8630AA	201400731	Derby	0	0	0	0	0	0	0	0	0	0	0
SAL_UA8631AA	201509601	Derby	0	0	0	0	0	0	0	0	0	0	0
SAL_UA8634AA	201409920	Derby	0	0	0	0	0	0	0	0	0	0	0
SAL_UA8635AA	201410145	Derby	0	0	0	0	0	0	0	0	0	0	0
SAL_UA8638AA	201409585	Derby	0	0	0	0	0	0	0	0	0	0	0
SAL_UA8639AA	201409907	Derby	0	0	0	0	0	0	0	0	0	0	0
SAL_UA8640AA	201409880	Derby	0	0	0	0	0	0	0	0	0	0	0
SAL_UA8641AA	201506534	Derby	0	0	0	0	0	0	0	0	0	0	0
SAL_UA8642AA	201410475	Derby	0	0	0	0	0	0	0	0	0	0	0
SAL_UA8643AA	201410308	Derby	0	0	0	0	0	0	0	0	0	0	0
SAL_UA8644AA	201501275	Derby	0	0	0	0	0	0	0	0	0	0	0
SAL_UA8645AA	201501287	Derby	0	0	0	0	0	0	0	0	0	0	0
SAL_UA8601AA	201406995	Derby	0	0	0	0	0	0	0	0	0	0	0
SAL_UA8602AA	201407050	Derby	0	0	0	0	0	0	0	0	0	0	0
SAL_UA8604AA	201405538	Derby	0	0	0	0	0	0	0	0	0	0	0
SAL_UA8603AA	201407264	Derby	0	0	0	0	0	0	0	0	0	0	0
SAL_UA8605AA	201405595	Derby	0	0	0	0	0	0	0	0	0	0	0
SAL_UA8607AA	201405871	Derby	0	0	0	0	0	0	0	0	0	0	0
SAL_UA8609AA	201406406	Derby	0	0	0	0	0	0	0	0	0	0	0
SAL_UA8606AA	201405843	Derby	0	0	0	0	0	0	0	0	0	0	0
SAL_UA8608AA	201405900	Derby	0	0	0	0	0	0	0	0	0	0	0
SAL_UA8610AA	201406583	Derby	0	0	0	0	0	0	0	0	0	0	0
SAL_UA8611AA	201406688	Derby	0	0	0	0	0	0	0	0	0	0	0
SAL_UA8612AA	201502943	Derby	0	0	0	0	0	0	0	0	0	0	0
SAL_UA8613AA	201502838	Derby	0	0	0	0	0	0	0	0	0	0	0
SAL_UA8614AA	201502733	Derby	0	0	0	0	0	0	0	0	0	0	0
SAL_UA8615AA	201502666	Derby	0	0	0	0	0	0	0	0	0	0	0
SAL_UA8616AA	201502827	Derby	0	0	0	0	0	0	0	0	0	0	0
SAL_UA8617AA	201502817	Derby	0	0	0	0	0	0	0	0	0	0	0
SAL_UA8618AA	201502623	Derby	0	0	0	0	0	0	0	0	0	0	0
SAL_UA8619AA	201502610	Derby	0	0	0	0	0	0	0	0	0	0	0
SAL_UA8622AA	201506750	Derby	0	0	0	0	0	0	0	0	0	0	0
SAL_UA8623AA	201509256	Derby	0	0	0	0	0	0	0	0	0	0	0
SAL_UA8624AA	201509427	Derby	0	0	0	0	0	0	0	0	0	0	0
SAL_UA8625AA	201509386	Derby	0	0	0	0	0	0	0	0	0	0	0
SAL_UA8632AA	201509541	Derby	0	0	0	0	0	0	0	0	0	0	0
SAL_UA8633AA	201410130	Derby	0	0	0	0	0	0	0	0	0	0	0
SAL_UA8636AA	201410132	Derby	0	0	0	0	0	0	0	0	0	0	0
SAL_UA8637AA	201506447	Derby	0	0	0	0	0	0	0	0	0	0	0
SAL_UA8463AA	201511707	Derby	0	0	0	0	0	0	0	0	0	0	0
SAL_UA8465AA	201500035	Derby	0	0	0	0	0	0	0	0	0	0	0
SAL_UA8466AA	201500189	Derby	0	0	0	0	0	0	0	0	0	0	0
SAL_UA8468AA	201500264	Derby	0	0	0	0	0	0	0	0	0	0	0
SAL_UA8470AA	201500377	Derby	0	0	0	0	0	0	0	0	0	0	0
SAL_UA8471AA	201500314	Derby	0	0	0	0	0	0	0	0	0	0	0
SAL_UA8472AA	201500614	Derby	0	0	0	0	0	0	0	0	0	0	0
SAL_UA8473AA	201500546	Derby	0	0	0	0	0	0	0	0	0	0	0
SAL_UA8474AA	201505858	Derby	0	0	0	0	0	0	0	0	0	0	0
SAL_UA8477AA	201505866	Derby	0	0	0	0	0	0	0	0	0	0	0
SAL_UA8478AA	201506082	Derby	0	0	0	0	0	0	0	0	0	0	0
SAL_UA8444AA	201506695	Derby	0	0	0	0	0	0	0	0	0	0	0
SAL_UA8448AA	201504535	Derby	0	0	0	0	0	0	0	0	0	0	0
SAL_UA8453AA	201504075	Derby	0	0	0	0	0	0	0	0	0	0	0
SAL_UA8454AA	201504172	Derby	0	0	0	0	0	0	0	0	0	0	0
SAL_UA8456AA	201503879	Derby	0	0	0	0	0	0	0	0	0	0	0
SAL_UA8457AA	201504487	Derby	0	0	0	0	0	0	0	0	0	0	0
SAL_UA8458AA	201504515	Derby	0	0	0	0	0	0	0	0	0	0	0
SAL_UA8459AA	201504287	Derby	0	0	0	0	0	0	0	0	0	0	0
SAL_UA8460AA	201504437	Derby	0	0	0	0	0	0	0	0	0	0	0
SAL_UA8461AA	201400726	Derby	0	0	0	0	0	0	0	0	0	0	0
SAL_UA8462AA	201511813	Derby	0	0	0	0	0	0	0	0	0	0	0
SAL_UA8435AA	201412323	Derby	0	0	0	0	0	0	0	0	0	0	0
SAL_UA8436AA	201412326	Derby	0	0	0	0	0	0	0	0	0	0	0
SAL_UA8438AA	201400280	Derby	0	0	0	0	0	0	0	0	0	0	0
SAL_UA8437AA	201400370	Derby	0	0	0	0	0	0	0	0	0	0	0
SAL_UA8439AA	201412831	Derby	0	0	0	0	0	0	0	0	0	0	0
SAL_UA8441AA	201506701	Derby	0	0	0	0	0	0	0	0	0	0	0

SAL_UA8440AA	201412853	Derby	0	0	0	0	0	0	0	0	0	0	0
SAL_UA8446AA	201410957	Derby	0	0	0	0	0	0	0	0	0	0	0
SAL_UA8442AA	201506707	Derby	0	0	0	0	0	0	0	0	0	0	0
SAL_UA8451AA	201410756	Derby	0	0	0	0	0	0	0	0	0	0	0
SAL_UA8449AA	201410586	Derby	0	0	0	0	0	0	0	0	0	0	0
SAL_UA8422AA	201401756	Derby	0	0	0	0	0	0	0	0	0	0	0
SAL_UA8427AA	201401857	Derby	0	0	0	0	0	0	0	0	0	0	0
SAL_UA8421AA	201401198	Derby	0	0	0	0	0	0	0	0	0	0	0
SAL_UA8426AA	201401891	Derby	0	0	0	0	0	0	0	0	0	0	0
SAL_UA8430AA	201412300	Derby	0	0	0	0	0	0	0	0	0	0	0
SAL_UA8432AA	201412149	Derby	0	0	0	0	0	0	0	0	0	0	0
SAL_UA8429AA	201412178	Derby	0	0	0	0	0	0	0	0	0	0	0
SAL_UA8428AA	201409774	Derby	0	0	0	0	0	0	0	0	0	0	0
SAL_UA8431AA	201412140	Derby	0	0	0	0	0	0	0	0	0	0	0
SAL_UA8434AA	201412615	Derby	0	0	0	0	0	0	0	0	0	0	0
SAL_UA8433AA	201412502	Derby	0	0	0	0	0	0	0	0	0	0	0
SAL_UA8416AA	201411406	Derby	0	0	0	0	0	0	0	0	0	0	0
SAL_UA8417AA	201508052	Derby	0	0	0	0	0	0	0	0	0	0	0
SAL_UA8418AA	201401549	Derby	0	0	0	0	0	0	0	0	0	0	0
SAL_UA8413AA	201411591	Derby	0	0	0	0	0	0	0	0	0	0	0
SAL_UA8419AA	201401437	Derby	0	0	0	0	0	0	0	0	0	0	0
SAL_UA8414AA	201508203	Derby	0	0	0	0	0	0	0	0	0	0	0
SAL_UA8423AA	201401716	Derby	0	0	0	0	0	0	0	0	0	0	0
SAL_UA8420AA	201401230	Derby	0	0	0	0	0	0	0	0	0	0	0
SAL_UA8407AA	201411672	Derby	0	0	0	0	0	0	0	0	0	0	0
SAL_UA8410AA	201412046	Derby	0	0	0	0	0	0	0	0	0	0	0
SAL_UA8408AA	201411667	Derby	0	0	0	0	0	0	0	0	0	0	0
SAL_UA8409AA	201412048	Derby	0	0	0	0	0	0	0	0	0	0	0
SAL_UA8397AA	201507709	Derby	0	0	0	0	0	0	0	0	0	0	0
SAL_UA8404AA	201412102	Derby	0	0	0	0	0	0	0	0	0	0	0
SAL_UA8411AA	201412029	Derby	0	0	0	0	0	0	0	0	0	0	0
SAL_UA8412AA	201411996	Derby	0	0	0	0	0	0	0	0	0	0	0
SAL_UA8388AA	201501600	Derby	0	0	0	0	0	0	0	0	0	0	0
SAL_UA8391AA	201501456	Derby	0	0	0	0	0	0	0	0	0	0	0
SAL_UA8395AA	201507861	Derby	0	0	0	0	0	0	0	0	0	0	0
SAL_UA8396AA	201507829	Derby	0	0	0	0	0	0	0	0	0	0	0
SAL_UA8392AA	201501354	Derby	0	0	0	0	0	0	0	0	0	0	0
SAL_UA8405AA	201411878	Derby	0	0	0	0	0	0	0	0	0	0	0
SAL_UA8406AA	201411751	Derby	0	0	0	0	0	0	0	0	0	0	0
SAL_UA8402AA	201400492	Derby	0	0	0	0	0	0	0	0	0	0	0
SAL_UA8389AA	201501530	Derby	0	0	0	0	0	0	0	0	0	0	0
SAL_UA8393AA	201501340	Derby	0	0	0	0	0	0	0	0	0	0	0
SAL_UA8386AA	201501915	Derby	0	0	0	0	0	0	0	0	0	0	0
SAL_UA8390AA	201501496	Derby	0	0	0	0	0	0	0	0	0	0	0
SAL_UA8394AA	201507910	Derby	0	0	0	0	0	0	0	0	0	0	0
SAL_UA8380AA	NC_704140057	Derby	0	0	0	0	0	0	0	0	0	0	0
SAL_UA8734AA	669720	Derby	0	0	0	0	0	0	0	0	0	0	0
SAL_UA8583AA	201502495	Derby	0	0	0	0	0	0	0	0	0	0	0
SAL_UA8576AA	201407237	Derby	0	0	0	0	0	0	0	0	0	0	0
SAL_UA8585AA	201502336	Derby	0	0	0	0	0	0	0	0	0	0	0
SAL_UA8584AA	201502500	Derby	0	0	0	0	0	0	0	0	0	0	0
SAL_UA8586AA	201502433	Derby	0	0	0	0	0	0	0	0	0	0	0
SAL_UA8587AA	201502215	Derby	0	0	0	0	0	0	0	0	0	0	0
SAL_UA8588AA	201502248	Derby	0	0	0	0	0	0	0	0	0	0	0
SAL_UA8589AA	201510223	Derby	0	0	0	0	0	0	0	0	0	0	0
SAL_UA8595AA	201509794	Derby	0	0	0	0	0	0	0	0	0	0	0
SAL_UA8598AA	201510824	Derby	0	0	0	0	0	0	0	0	0	0	0
SAL_UA8600AA	201509385	Derby	0	0	0	0	0	0	0	0	0	0	0
SAL_UA8590AA	201510274	Derby	0	0	0	0	0	0	0	0	0	0	0
SAL_UA8594AA	201510029	Derby	0	0	0	0	0	0	0	0	0	0	0
SAL_UA8597AA	201510727	Derby	0	0	0	0	0	0	0	0	0	0	0
SAL_UA8599AA	201407546	Derby	0	0	0	0	0	0	0	0	0	0	0
SAL_UA8592AA	201510168	Derby	0	0	0	0	0	0	0	0	0	0	0
SAL_UA8567AA	201505837	Derby	0	0	0	0	0	0	0	0	0	0	0
SAL_UA8568AA	201408509	Derby	0	0	0	0	0	0	0	0	0	0	0
SAL_UA8569AA	201407908	Derby	0	0	0	0	0	0	0	0	0	0	0
SAL_UA8570AA	201407779	Derby	0	0	0	0	0	0	0	0	0	0	0
SAL_UA8571AA	201407672	Derby	0	0	0	0	0	0	0	0	0	0	0
SAL_UA8572AA	201505685	Derby	0	0	0	0	0	0	0	0	0	0	0
SAL_UA8574AA	201407354	Derby	0	0	0	0	0	0	0	0	0	0	0
SAL_UA8573AA	201407381	Derby	0	0	0	0	0	0	0	0	0	0	0
SAL_UA8575AA	201400670	Derby	0	0	0	0	0	0	0	0	0	0	0
SAL_UA8579AA	201502549	Derby	0	0	0	0	0	0	0	0	0	0	0

SAL_UA8580AA	201502572	Derby	0	0	0	0	0	0	0	0	0	0	0
SAL_UA8581AA	201502528	Derby	0	0	0	0	0	0	0	0	0	0	0
SAL_UA8578AA	201506375	Derby	0	0	0	0	0	0	0	0	0	0	0
SAL_UA8553AA	201503756	Derby	0	0	0	0	0	0	0	0	0	0	0
SAL_UA8552AA	201503838	Derby	0	0	0	0	0	0	0	0	0	0	0
SAL_UA8554AA	201503593	Derby	0	0	0	0	0	0	0	0	0	0	0
SAL_UA8551AA	201503841	Derby	0	0	0	0	0	0	0	0	0	0	0
SAL_UA8558AA	201503842	Derby	0	0	0	0	0	0	0	0	0	0	0
SAL_UA8562AA	201511433	Derby	0	0	0	0	0	0	0	0	0	0	0
SAL_UA8557AA	201503845	Derby	0	0	0	0	0	0	0	0	0	0	0
SAL_UA8559AA	201511678	Derby	0	0	0	0	0	0	0	0	0	0	0
SAL_UA8565AA	201511948	Derby	0	0	0	0	0	0	0	0	0	0	0
SAL_UA8561AA	201511573	Derby	0	0	0	0	0	0	0	0	0	0	0
SAL_UA8560AA	201511640	Derby	0	0	0	0	0	0	0	0	0	0	0
SAL_UA8564AA	201511935	Derby	0	0	0	0	0	0	0	0	0	0	0
SAL_UA8563AA	201506254	Derby	0	0	0	0	0	0	0	0	0	0	0
SAL_UA8566AA	201400869	Derby	0	0	0	0	0	0	0	0	0	0	0
SAL_UA8542AA	201512581	Derby	0	0	0	0	0	0	0	0	0	0	0
SAL_UA8541AA	201512926	Derby	0	0	0	0	0	0	0	0	0	0	0
SAL_UA8543AA	201400598	Derby	0	0	0	0	0	0	0	0	0	0	0
SAL_UA8539AA	201512881	Derby	0	0	0	0	0	0	0	0	0	0	0
SAL_UA8540AA	201513031	Derby	0	0	0	0	0	0	0	0	0	0	0
SAL_UA8549AA	201503447	Derby	0	0	0	0	0	0	0	0	0	0	0
SAL_UA8547AA	201503587	Derby	0	0	0	0	0	0	0	0	0	0	0
SAL_UA8548AA	201503568	Derby	0	0	0	0	0	0	0	0	0	0	0
SAL_UA8550AA	201503426	Derby	0	0	0	0	0	0	0	0	0	0	0
SAL_UA8525AA	201402462	Derby	0	0	0	0	0	0	0	0	0	0	0
SAL_UA8528AA	201402568	Derby	0	0	0	0	0	0	0	0	0	0	0
SAL_UA8535AA	201512780	Derby	0	0	0	0	0	0	0	0	0	0	0
SAL_UA8537AA	201512811	Derby	0	0	0	0	0	0	0	0	0	0	0
SAL_UA8538AA	201512893	Derby	0	0	0	0	0	0	0	0	0	0	0
SAL_UA8536AA	201512840	Derby	0	0	0	0	0	0	0	0	0	0	0
SAL_UA8527AA	201401922	Derby	0	0	0	0	0	0	0	0	0	0	0
SAL_UA8546AA	201506135	Derby	0	0	0	0	0	0	0	0	0	0	0
SAL_UA8518AA	201512015	Derby	0	0	0	0	0	0	0	0	0	0	0
SAL_UA8517AA	201503418	Derby	0	0	0	0	0	0	0	0	0	0	0
SAL_UA8520AA	201411400	Derby	0	0	0	0	0	0	0	0	0	0	0
SAL_UA8521AA	201403341	Derby	0	0	0	0	0	0	0	0	0	0	0
SAL_UA8526AA	201401917	Derby	0	0	0	0	0	0	0	0	0	0	0
SAL_UA8522AA	201403705	Derby	0	0	0	0	0	0	0	0	0	0	0
SAL_UA8529AA	201402773	Derby	0	0	0	0	0	0	0	0	0	0	0
SAL_UA8530AA	201402484	Derby	0	0	0	0	0	0	0	0	0	0	0
SAL_UA8524AA	201402407	Derby	0	0	0	0	0	0	0	0	0	0	0
SAL_UA8523AA	201508243	Derby	0	0	0	0	0	0	0	0	0	0	0
SAL_UA8531AA	201402511	Derby	0	0	0	0	0	0	0	0	0	0	0
SAL_UA8533AA	201512711	Derby	0	0	0	0	0	0	0	0	0	0	0
SAL_UA8532AA	201512756	Derby	0	0	0	0	0	0	0	0	0	0	0
SAL_UA8534AA	201512808	Derby	0	0	0	0	0	0	0	0	0	0	0
SAL_UA8666AA	201509142	Derby	0	0	0	0	0	0	0	0	0	0	0
SAL_UA8671AA	201409182	Derby	0	0	0	0	0	0	0	0	0	0	0
SAL_UA8509AA	201502967	Derby	0	0	0	0	0	0	0	0	0	0	0
SAL_UA8511AA	201503101	Derby	0	0	0	0	0	0	0	0	0	0	0
SAL_UA8513AA	201512455	Derby	0	0	0	0	0	0	0	0	0	0	0
SAL_UA8515AA	201512534	Derby	0	0	0	0	0	0	0	0	0	0	0
SAL_UA8516AA	201503321	Derby	0	0	0	0	0	0	0	0	0	0	0
SAL_UA8507AA	201503310	Derby	0	0	0	0	0	0	0	0	0	0	0
SAL_UA8510AA	201503047	Derby	0	0	0	0	0	0	0	0	0	0	0
SAL_UA8508AA	201502965	Derby	0	0	0	0	0	0	0	0	0	0	0
SAL_UA8494AA	201403921	Derby	0	0	0	0	0	0	0	0	0	0	0
SAL_UA8493AA	201404154	Derby	0	0	0	0	0	0	0	0	0	0	0
SAL_UA8495AA	201404339	Derby	0	0	0	0	0	0	0	0	0	0	0
SAL_UA8497AA	201405022	Derby	0	0	0	0	0	0	0	0	0	0	0
SAL_UA8496AA	201404239	Derby	0	0	0	0	0	0	0	0	0	0	0
SAL_UA8498AA	201404572	Derby	0	0	0	0	0	0	0	0	0	0	0
SAL_UA8505AA	201503204	Derby	0	0	0	0	0	0	0	0	0	0	0
SAL_UA8506AA	201503251	Derby	0	0	0	0	0	0	0	0	0	0	0
SAL_UA8499AA	201405226	Derby	0	0	0	0	0	0	0	0	0	0	0
SAL_UA8500AA	201405217	Derby	0	0	0	0	0	0	0	0	0	0	0
SAL_UA8668AA	201411020	Derby	0	0	0	0	0	0	0	0	0	0	0
SAL_UA8677AA	201409279	Derby	0	0	0	0	0	0	0	0	0	0	0
SAL_UA8678AA	201409280	Derby	0	0	0	0	0	0	0	0	0	0	0
SAL_UA8481AA	201505354	Derby	0	0	0	0	0	0	0	0	0	0	0
SAL_UA8487AA	201504777	Derby	0	0	0	0	0	0	0	0	0	0	0

SAL_UA8483AA	201504617	Derby	0	0	0	0	0	0	0	0	0	0	0
SAL_UA8482AA	201504624	Derby	0	0	0	0	0	0	0	0	0	0	0
SAL_UA8480AA	201505671	Derby	0	0	0	0	0	0	0	0	0	0	0
SAL_UA8484AA	201504722	Derby	0	0	0	0	0	0	0	0	0	0	0
SAL_UA8486AA	201504798	Derby	0	0	0	0	0	0	0	0	0	0	0
SAL_UA8492AA	201510872	Derby	0	0	0	0	0	0	0	0	0	0	0
SAL_UA8490AA	201510905	Derby	0	0	0	0	0	0	0	0	0	0	0
SAL_UA8488AA	201505111	Derby	0	0	0	0	0	0	0	0	0	0	0
SAL_UA8489AA	201504928	Derby	0	0	0	0	0	0	0	0	0	0	0
SAL_VA0243AA	FSIS21923310	Derby	0	0	0	0	0	0	0	0	0	0	0
SAL_UA9754AA	659348	Derby	0	0	0	0	0	0	0	0	0	0	0
SAL_UA9743AA	648991	Derby	0	0	0	0	0	0	0	0	0	0	0
SAL_VA0706AA	201811098	Derby	0	0	0	0	0	0	0	0	0	0	0
SAL_VA0493AA	SAL-18-VL-SD-IA-0022	Derby	0	0	0	0	0	0	0	0	0	0	0
SAL_VA0495AA	SAL-18-VL-SD-IA-0014	Derby	0	0	0	0	0	0	0	0	0	0	0
SAL_VA0481AA	ADRDL-2235	Derby	0	0	0	0	0	0	0	0	0	0	0
SAL_VA0307AA	548547	Derby	0	0	0	0	0	0	0	0	0	0	0
SAL_UA9270AA		Derby	0	0	0	0	0	0	0	0	0	0	0
SAL_VA2383AA	CFSA231	Derby	0	0	0	0	0	0	0	0	0	0	0
SAL_VA1505AA	UFRGS-SA006	Derby	0	0	0	0	0	0	0	0	0	0	0
SAL_VA7383AA	201607567	Derby	0	0	0	0	0	0	0	0	0	0	0
SAL_VA7411AA	201701301	Derby	0	0	0	0	0	0	0	0	0	0	0
SAL_VA7422AA	201702195	Derby	0	0	0	0	0	0	0	0	0	0	0
SAL_VA7616AA	201707398	Derby	0	0	0	0	0	0	0	0	0	0	0
SAL_VA7637AA	201708120	Derby	0	0	0	0	0	0	0	0	0	0	0
SAL_VA3035AA	DAC40	Derby	0	0	0	0	0	0	0	0	0	0	0
SAL_VA5861AA	SA20030592	Derby	0	0	0	0	0	0	0	0	0	0	0
SAL_VA5831AA	SA20094702	Derby	0	0	0	0	0	0	0	0	0	0	0
SAL_VA6779AA	FSIS11917521	Derby	0	0	0	0	0	0	0	0	0	0	0
SAL_VA6849AA	HIY0184	Derby	0	0	0	0	0	0	0	0	0	0	0
SAL_VA6851AA	HIY0185	Derby	0	0	0	0	0	0	0	0	0	0	0
SAL_WA1607AA	SMG-19-355	Derby	0	0	0	0	0	0	0	0	0	0	0
SAL_VA7109AA	682275	Derby	0	0	0	0	0	0	0	0	0	0	0
SAL_VA7775AA	FSIS11917584	Derby	0	0	0	0	0	0	0	0	0	0	0
SAL_VA7720AA	FSIS11917551	Derby	0	0	0	0	0	0	0	0	0	0	0
SAL_VA7960AA	FSIS11917747	Derby	0	0	0	0	0	0	0	0	0	0	0
SAL_VA8007AA	FSIS11917780	Derby	0	0	0	0	0	0	0	0	0	0	0
SAL_VA8117AA	FSIS11918086	Derby	0	0	0	0	0	0	0	0	0	0	0
SAL_VA8041AA	NYVetLIRN-132	Derby	0	0	0	0	0	0	0	0	0	0	0
SAL_VA8617AA	FSIS11918000	Derby	0	0	0	0	0	0	0	0	0	0	0
SAL_VA8589AA	FSIS11918025	Derby	0	0	0	0	0	0	0	0	0	0	0
SAL_VA9808AA	FSIS11918516	Derby	0	0	0	0	0	0	0	0	0	0	0
SAL_VA9864AA	FSIS31901649	Derby	0	0	0	0	0	0	0	0	0	0	0
SAL_WA0683AA	FSIS11918519	Derby	0	0	0	0	0	0	0	0	0	0	0
SAL_WA3288AA	NL_D35	Derby	0	0	0	0	0	0	0	0	0	0	0
SAL_WA3274AA	SE10/52	Derby	0	0	0	0	0	0	0	0	0	0	0
SAL_WA3261AA	NL_D45	Derby	0	0	0	0	0	0	0	0	0	0	0
SAL_WA3249AA	MC_07-0477	Derby	0	0	0	0	0	0	0	0	0	0	0
SAL_WA3242AA	NL_H17	Derby	0	0	0	0	0	0	0	0	0	0	0
SAL_WA3237AA	US08/97	Derby	0	0	0	0	0	0	0	0	0	0	0
SAL_WA3236AA	DP_T93	Derby	0	0	0	0	0	0	0	0	0	0	0
SAL_WA3239AA	GW_S00056-06	Derby	0	0	0	0	0	0	0	0	0	0	0
SAL_WA3234AA	JE_S09-000753	Derby	0	0	0	0	0	0	0	0	0	0	0
SAL_WA3225AA	NL_D37	Derby	0	0	0	0	0	0	0	0	0	0	0
SAL_WA3221AA	NL_L7	Derby	0	0	0	0	0	0	0	0	0	0	0
SAL_WA3205AA	GW_S02100-06	Derby	0	0	0	0	0	0	0	0	0	0	0
SAL_WA3193AA	GW_S04648-06	Derby	0	0	0	0	0	0	0	0	0	0	0
SAL_WA3178AA	NL_D48	Derby	0	0	0	0	0	0	0	0	0	0	0
SAL_WA3175AA	NL_D1	Derby	0	0	0	0	0	0	0	0	0	0	0
SAL_WA3167AA	NL_C3	Derby	0	0	0	0	0	0	0	0	0	0	0
SAL_WA3168AA	NL_C4	Derby	0	0	0	0	0	0	0	0	0	0	0
SAL_WA3163AA	JE_S09-001868	Derby	0	0	0	0	0	0	0	0	0	0	0
SAL_WA3162AA	NL_D46	Derby	0	0	0	0	0	0	0	0	0	0	0
SAL_WA3166AA	NL_C2	Derby	0	0	0	0	0	0	0	0	0	0	0
SAL_WA3159AA	NL_D49	Derby	0	0	0	0	0	0	0	0	0	0	0
SAL_WA3165AA	NL_D38	Derby	0	0	0	0	0	0	0	0	0	0	0
SAL_WA3131AA	NL_A25	Derby	0	0	0	0	0	0	0	0	0	0	0
SAL_WA3112AA	JE_S09-002580	Derby	0	0	0	0	0	0	0	0	0	0	0
SAL_WA3093AA	JE_S09-001303	Derby	0	0	0	0	0	0	0	0	0	0	0
SAL_WA3100AA	GA1113TM	Derby	0	0	0	0	0	0	0	0	0	0	0
SAL_WA3080AA	NL_A22	Derby	0	0	0	0	0	0	0	0	0	0	0
SAL_WA3079AA	JE_S09-000875	Derby	0	0	0	0	0	0	0	0	0	0	0
SAL_WA3054AA	NL_C1	Derby	0	0	0	0	0	0	0	0	0	0	0

SAL_WA3042AA	JE_S09-002349	Derby	0	0	0	0	0	0	0	0	0	0	0
SAL_WA3043AA	DP_T82	Derby	0	0	0	0	0	0	0	0	0	0	0
SAL_WA3037AA	US07/16	Derby	0	0	0	0	0	0	0	0	0	0	0
SAL_WA3033AA	GW_S00535-06	Derby	0	0	0	0	0	0	0	0	0	0	0
SAL_WA3008AA	BD-OM-2008-01-003285	Derby	0	0	0	0	0	0	0	0	0	0	0
SAL_WA2952AA	JE_S09-002348	Derby	0	0	0	0	0	0	0	0	0	0	0
SAL_WA2940AA	JE_S09-002200	Derby	0	0	0	0	0	0	0	0	0	0	0
SAL_WA2917AA	DP_T52	Derby	0	0	0	0	0	0	0	0	0	0	0
SAL_WA2912AA	NL_D61	Derby	0	0	0	0	0	0	0	0	0	0	0
SAL_WA2900AA	US09/116	Derby	0	0	0	0	0	0	0	0	0	0	0
SAL_WA2886AA	DP_T111	Derby	0	0	0	0	0	0	0	0	0	0	0
SAL_WA2853AA	MC_07-0673	Derby	0	0	0	0	0	0	0	0	0	0	0
SAL_WA2826AA	SE10/60	Derby	0	0	0	0	0	0	0	0	0	0	0
SAL_WA2813AA	JE_S09-002581	Derby	0	0	0	0	0	0	0	0	0	0	0
SAL_WA2811AA	JE_S09-001711	Derby	0	0	0	0	0	0	0	0	0	0	0
SAL_WA2796AA	DP_T84	Derby	0	0	0	0	0	0	0	0	0	0	0
SAL_WA2784AA	JE_S09-001708	Derby	0	0	0	0	0	0	0	0	0	0	0
SAL_WA2787AA	US05/113	Derby	0	0	0	0	0	0	0	0	0	0	0
SAL_WA2774AA	NL_D5	Derby	0	0	0	0	0	0	0	0	0	0	0
SAL_WA2792AA	GA5579RB	Derby	0	0	0	0	0	0	0	0	0	0	0
SAL_WA2770AA	JE_S09-001712	Derby	0	0	0	0	0	0	0	0	0	0	0
SAL_WA2749AA	NL_O6	Derby	0	0	0	0	0	0	0	0	0	0	0
SAL_WA2752AA	BD-DU-2007-12-002194	Derby	0	0	0	0	0	0	0	0	0	0	0
SAL_WA2729AA	MP10/73	Derby	0	0	0	0	0	0	0	0	0	0	0
SAL_WA2708AA	GW_S00046-06	Derby	0	0	0	0	0	0	0	0	0	0	0
SAL_WA2712AA	SE10/20	Derby	0	0	0	0	0	0	0	0	0	0	0
SAL_WA2664AA	US06/94	Derby	0	0	0	0	0	0	0	0	0	0	0
SAL_WA2666AA	NL_P17	Derby	0	0	0	0	0	0	0	0	0	0	0
SAL_WA2659AA	SE10/36	Derby	0	0	0	0	0	0	0	0	0	0	0
SAL_WA2512AA	NL_A4	Derby	0	0	0	0	0	0	0	0	0	0	0
SAL_WA2586AA	NL_O12	Derby	0	0	0	0	0	0	0	0	0	0	0
SAL_WA2595AA	DP_T115	Derby	0	0	0	0	0	0	0	0	0	0	0
SAL_WA2548AA	NL_D36	Derby	0	0	0	0	0	0	0	0	0	0	0
SAL_WA2585AA	NL_A37	Derby	0	0	0	0	0	0	0	0	0	0	0
SAL_WA2493AA	NL_A29	Derby	0	0	0	0	0	0	0	0	0	0	0
SAL_WA2553AA	NL_D32	Derby	0	0	0	0	0	0	0	0	0	0	0
SAL_WA2519AA	NL_A24	Derby	0	0	0	0	0	0	0	0	0	0	0
SAL_WA2488AA	DP_P1	Derby	0	0	0	0	0	0	0	0	0	0	0
SAL_WA2484AA	NL_A23	Derby	0	0	0	0	0	0	0	0	0	0	0
SAL_WA2492AA	JE_S09-002576	Derby	0	0	0	0	0	0	0	0	0	0	0
SAL_WA2489AA	NL_A30	Derby	0	0	0	0	0	0	0	0	0	0	0
SAL_WA2477AA	DP_T88	Derby	0	0	0	0	0	0	0	0	0	0	0
SAL_WA2487AA	DP_P3	Derby	0	0	0	0	0	0	0	0	0	0	0
SAL_WA2471AA	OT09/33	Derby	0	0	0	0	0	0	0	0	0	0	0
SAL_WA2464AA	JE_S09-002425	Derby	0	0	0	0	0	0	0	0	0	0	0
SAL_WA2478AA	NL_A31	Derby	0	0	0	0	0	0	0	0	0	0	0
SAL_WA2451AA	SE08/24	Derby	0	0	0	0	0	0	0	0	0	0	0
SAL_WA2433AA	NL_D16	Derby	0	0	0	0	0	0	0	0	0	0	0
SAL_WA2413AA	SE09/59	Derby	0	0	0	0	0	0	0	0	0	0	0
SAL_WA2391AA	NL_D9	Derby	0	0	0	0	0	0	0	0	0	0	0
SAL_WA2376AA	BD-DU-2007-01-003580	Derby	0	0	0	0	0	0	0	0	0	0	0
SAL_WA2345AA	NL_D24	Derby	0	0	0	0	0	0	0	0	0	0	0
SAL_WA2347AA	NL_D26	Derby	0	0	0	0	0	0	0	0	0	0	0
SAL_WA2308AA	MC_06-0395	Derby	0	0	0	0	0	0	0	0	0	0	0
SAL_WA2310AA	NL_D33	Derby	0	0	0	0	0	0	0	0	0	0	0
SAL_WA2254AA	NL_D53	Derby	0	0	0	0	0	0	0	0	0	0	0
SAL_WA2245AA	NL_L3	Derby	0	0	0	0	0	0	0	0	0	0	0
SAL_WA2242AA	SE05/29	Derby	0	0	0	0	0	0	0	0	0	0	0
SAL_WA2238AA	NL_D14	Derby	0	0	0	0	0	0	0	0	0	0	0
SAL_WA1469AA	FSIS11918942	Derby	0	0	0	0	0	0	0	0	0	0	0
SAL_WA2195AA	SM-DU-2006-12-004559	Derby	0	0	0	0	0	0	0	0	0	0	0
SAL_WA2191AA	BD-PM-2007-07-002432	Derby	0	0	0	0	0	0	0	0	0	0	0
SAL_WA2192AA	BD-PM-2007-03-010764	Derby	0	0	0	0	0	0	0	0	0	0	0
SAL_WA2193AA	SM-DU-2007-03-004435	Derby	0	0	0	0	0	0	0	0	0	0	0
SAL_WA2232AA	NL_D15	Derby	0	0	0	0	0	0	0	0	0	0	0
SAL_WA2255AA	GA6700TM	Derby	0	0	0	0	0	0	0	0	0	0	0
SAL_WA2224AA	NL_D6	Derby	0	0	0	0	0	0	0	0	0	0	0
SAL_WA2260AA	DP_P31	Derby	0	0	0	0	0	0	0	0	0	0	0
SAL_WA2226AA	NL_D54	Derby	0	0	0	0	0	0	0	0	0	0	0
SAL_WA2264AA	NL_A26	Derby	0	0	0	0	0	0	0	0	0	0	0
SAL_WA2285AA	NL_A27	Derby	0	0	0	0	0	0	0	0	0	0	0
SAL_WA2202AA	JE_S09-000642	Derby	0	0	0	0	0	0	0	0	0	0	0
SAL_WA2328AA	NL_H20	Derby	0	0	0	0	0	0	0	0	0	0	0

SAL_WA2352AA	NL_D28	Derby	0	0	0	0	0	0	0	0	0	0	0
SAL_WA2340AA	DP_R19	Derby	0	0	0	0	0	0	0	0	0	0	0
SAL_WA2371AA	NL_L4	Derby	0	0	0	0	0	0	0	0	0	0	0
SAL_WA2414AA	NL_A28	Derby	0	0	0	0	0	0	0	0	0	0	0
SAL_WA2415AA	NL_D42	Derby	0	0	0	0	0	0	0	0	0	0	0
SAL_WA2418AA	JE_S09-001863	Derby	0	0	0	0	0	0	0	0	0	0	0
SAL_WA2468AA	NL_D39	Derby	0	0	0	0	0	0	0	0	0	0	0
SAL_WA2474AA	NL_A32	Derby	0	0	0	0	0	0	0	0	0	0	0
SAL_WA2470AA	NL_D52	Derby	0	0	0	0	0	0	0	0	0	0	0
SAL_WA2479AA	NL_D40	Derby	0	0	0	0	0	0	0	0	0	0	0
SAL_WA2482AA	NL_D47	Derby	0	0	0	0	0	0	0	0	0	0	0
SAL_WA2545AA	NL_F5	Derby	0	0	0	0	0	0	0	0	0	0	0
SAL_WA2534AA	NL_D18	Derby	0	0	0	0	0	0	0	0	0	0	0
SAL_WA2507AA	JE_S09-002317	Derby	0	0	0	0	0	0	0	0	0	0	0
SAL_WA2649AA	JE_S09-000767	Derby	0	0	0	0	0	0	0	0	0	0	0
SAL_WA2665AA	NL_D31	Derby	0	0	0	0	0	0	0	0	0	0	0
SAL_WA2505AA	DP_T83	Derby	0	0	0	0	0	0	0	0	0	0	0
SAL_WA2551AA	NL_D34	Derby	0	0	0	0	0	0	0	0	0	0	0
SAL_WA2636AA	DP_T91	Derby	0	0	0	0	0	0	0	0	0	0	0
SAL_WA2751AA	NL_D44	Derby	0	0	0	0	0	0	0	0	0	0	0
SAL_WA2764AA	NL_D2	Derby	0	0	0	0	0	0	0	0	0	0	0
SAL_WA2587AA	NL_A40	Derby	0	0	0	0	0	0	0	0	0	0	0
SAL_WA2672AA	GA5703TB	Derby	0	0	0	0	0	0	0	0	0	0	0
SAL_WA2688AA	NL_D58	Derby	0	0	0	0	0	0	0	0	0	0	0
SAL_WA2766AA	JE_S09-002582	Derby	0	0	0	0	0	0	0	0	0	0	0
SAL_WA2791AA	NL_A35	Derby	0	0	0	0	0	0	0	0	0	0	0
SAL_WA2793AA	NL_A33	Derby	0	0	0	0	0	0	0	0	0	0	0
SAL_WA2846AA	NL_D30	Derby	0	0	0	0	0	0	0	0	0	0	0
SAL_WA2817AA	NL_D43	Derby	0	0	0	0	0	0	0	0	0	0	0
SAL_WA2857AA	NL_D60	Derby	0	0	0	0	0	0	0	0	0	0	0
SAL_WA2893AA	NL_C6	Derby	0	0	0	0	0	0	0	0	0	0	0
SAL_WA2896AA	NL_D3	Derby	0	0	0	0	0	0	0	0	0	0	0
SAL_WA2953AA	JE_S09-002197	Derby	0	0	0	0	0	0	0	0	0	0	0
SAL_WA3077AA	NL_D27	Derby	0	0	0	0	0	0	0	0	0	0	0
SAL_WA2967AA	NL_D4	Derby	0	0	0	0	0	0	0	0	0	0	0
SAL_WA3099AA	JE_S09-002579	Derby	0	0	0	0	0	0	0	0	0	0	0
SAL_WA2911AA	NL_F3	Derby	0	0	0	0	0	0	0	0	0	0	0
SAL_WA1960AA	704342	Derby	0	0	0	0	0	0	0	0	0	0	0
SAL_WA1935AA	FSIS11918343	Derby	0	0	0	0	0	0	0	0	0	0	0
SAL_WA3752AA	P3S1022	Derby	0	0	0	0	0	0	0	0	0	0	0
SAL_WA3762AA	S1011	Derby	0	0	0	0	0	0	0	0	0	0	0
SAL_VA4273AA	2015LSAL01578	Derby	0	0	0	0	0	0	0	0	0	0	0
SAL_WA3641AA	CFSAN087482	Derby	0	0	0	0	0	0	0	0	0	0	0
SAL_WA3609AA	CFSAN087553	Derby	0	0	0	0	0	0	0	0	0	0	0
SAL_WA3574AA	CFSAN087559	Derby	0	0	0	0	0	0	0	0	0	0	0
SAL_WA3528AA	CFSAN087481	Derby	0	0	0	0	0	0	0	0	0	0	0
SAL_WA3542AA	CFSAN087558	Derby	0	0	0	0	0	0	0	0	0	0	0
SAL_WA4062AA	FSIS11919425	Derby	0	0	0	0	0	0	0	0	0	0	0
SAL_WA4041AA	FSIS11918955	Derby	0	0	0	0	0	0	0	0	0	0	0
SAL_WA4040AA	FSIS11919186	Derby	0	0	0	0	0	0	0	0	0	0	0
SAL_WA4134AA	FSIS31901711	Derby	0	0	0	0	0	0	0	0	0	0	0
SAL_WA4164AA	P3S1022	Derby	0	0	0	0	0	0	0	0	0	0	0
SAL_VA4272AA	2014LSAL05213	Derby	0	0	0	0	0	0	0	0	0	0	0
SAL_TA8918AA	636288	Detmold	0	0	0	0	0	0	0	0	0	0	0
SAL_CA6176AA	59996	Deversoir	0	0	0	0	0	0	0	0	0	0	0
SAL_TA9821AA	SMG-18-2312	Deversoir	0	0	0	0	0	0	0	0	0	0	0
SAL_EA3349AA	BS_00261-sc-2322488	Doel	0	0	0	0	0	0	0	0	0	0	0
SAL_KA0420AA	PNUSAS014507	Doel	0	0	0	0	0	0	0	0	0	0	0
SAL_EA3359AA	BN_02067-sc-2322478	Doel	0	0	0	0	0	0	0	0	0	0	0
SAL_WA1029AA		Doel	0	0	0	0	0	0	0	0	0	0	0
SAL_EA0032AA	FNE0030	Drac	0	0	0	0	0	0	0	0	0	0	0
SAL_BA6407AA	804417	Drac	0	0	0	0	0	0	0	0	0	0	0
SAL_AA9655AA	CN_178_72	Dublin	5	4	6	8	0	0	0	0	0	0	0
SAL_BA7852AA	A207-sc-2010-05-07-278	Dublin	5	4	6	27	0	0	27	5	43	0	1
SAL_CA5570AA	71530	Dublin	0	0	0	0	0	0	0	0	0	0	0
SAL_AA8702AA	PO709	Dublin	5	4	6	7	0	0	5	5	6	5	11
SAL_AA8479AA	19-sc-1925581	Dublin	5	4	6	7	0	0	5	5	6	5	2
SAL_EA9118AA	CFSAN031744	Dublin	5	4	29	7	0	0	5	5	6	5	12
SAL_AA7540AA	PO2066-sc-1993619	Dublin	5	4	6	7	0	0	5	5	6	5	11
SAL_DA6757AA	CFSAN013279	Dublin	5	4	6	7	0	0	5	5	6	5	10
SAL_EA7588AA	53071	Dublin	5	4	6	63	0	0	5	5	6	5	13
SAL_AA9881AA	c-2013-10-08T14:27:14Z	Dublin	5	4	6	7	0	0	5	5	6	5	10
SAL_DA2455AA	MDH-2014-00554	Dublin	5	4	6	7	0	0	5	5	6	5	2

SAL_AA8693AA	PO288-sc-1963258	Dublin	5	4	6	7	0	0	5	5	6	5	10
SAL_FA5717AA	CFSAN048789	Dublin	5	4	6	7	0	0	5	5	6	5	2
SAL_FA5718AA	CFSAN048788	Dublin	5	4	6	7	0	0	5	5	6	5	2
SAL_FA5716AA	CFSAN048790	Dublin	5	4	6	7	0	0	5	5	6	5	2
SAL_FA5715AA	CFSAN048791	Dublin	5	4	6	7	0	0	5	5	6	5	2
SAL_FA5668AA	MS150073	Dublin	5	4	6	7	0	0	5	5	83	5	10
SAL_FA5404AA	PNUSAS001636	Dublin	5	4	6	7	0	0	5	5	6	5	2
SAL_FA5251AA	CFSAN047664	Dublin	5	4	6	7	0	0	5	5	6	5	2
SAL_FA5253AA	CFSAN047662	Dublin	5	4	6	7	0	0	5	5	6	5	2
SAL_FA5250AA	CFSAN047665	Dublin	5	4	6	7	0	0	5	5	6	5	2
SAL_FA5249AA	CFSAN047666	Dublin	5	4	6	7	0	0	5	5	6	5	2
SAL_FA5248AA	IEH-NGS-SAL-04010	Dublin	5	4	6	7	0	0	5	5	6	5	2
SAL_FA5252AA	CFSAN047663	Dublin	5	4	6	7	0	0	5	5	6	5	2
SAL_FA5210AA	CFSAN047697	Dublin	5	4	6	7	0	0	5	5	6	5	2
SAL_FA5068AA	PNUSA001864	Dublin	5	4	6	7	0	0	5	5	6	5	0
SAL_FA4981AA	2023	Dublin	5	4	6	7	0	0	5	5	6	5	10
SAL_FA4978AA	8298	Dublin	5	4	6	7	0	0	5	5	6	5	11
SAL_FA4877AA	1557	Dublin	5	4	6	7	0	0	0	0	0	0	0
SAL_FA4875AA	3170	Dublin	5	4	6	7	0	0	0	0	0	0	0
SAL_FA4873AA	9894	Dublin	5	4	6	7	0	0	5	5	6	5	10
SAL_FA4876AA	5462	Dublin	5	4	6	7	0	0	5	5	0	0	17
SAL_FA4870AA	9836	Dublin	5	4	6	7	0	0	5	5	6	5	17
SAL_FA4874AA	1086	Dublin	5	4	6	7	0	0	0	0	0	0	0
SAL_FA4872AA	1078	Dublin	5	4	6	7	0	0	5	5	6	5	17
SAL_FA4827AA	201200014	Dublin	5	4	6	7	0	0	5	5	6	5	17
SAL_FA4826AA	201001882	Dublin	5	4	6	7	0	0	5	5	6	5	17
SAL_FA4828AA	201200083	Dublin	5	4	6	7	0	0	5	5	6	5	11
SAL_FA4749AA	2892	Dublin	5	4	6	7	0	0	5	5	84	5	17
SAL_FA4750AA	2054	Dublin	5	4	6	7	0	0	5	5	6	5	17
SAL_FA4748AA	9808	Dublin	5	4	6	7	0	0	5	5	6	5	11
SAL_FA4747AA	9588	Dublin	5	4	6	7	0	0	5	5	6	5	17
SAL_FA4746AA	SD	Dublin	5	4	6	8	0	0	5	53	51	5	1
SAL_FA4608AA	200643	Dublin	5	4	6	7	0	0	5	5	6	5	10
SAL_FA4456AA	188170	Dublin	5	4	6	7	0	0	5	5	6	5	18
SAL_FA4411AA	197026	Dublin	5	4	6	7	0	0	5	5	6	5	18
SAL_FA4353AA	199844	Dublin	5	4	6	7	0	0	5	5	6	5	18
SAL_FA4281AA	201516	Dublin	5	4	6	7	0	0	5	5	6	5	10
SAL_FA4240AA	132022	Dublin	5	4	6	7	0	0	5	5	6	5	18
SAL_FA3327AA	CFSAN047780	Dublin	5	4	6	7	0	0	5	5	6	5	2
SAL_FA3323AA	CFSAN047784	Dublin	5	4	6	7	0	0	5	5	6	5	2
SAL_FA3325AA	IEH-NGS-SAL-03927	Dublin	5	4	6	7	0	0	5	5	6	5	19
SAL_FA2470AA	CFSAN047865	Dublin	5	4	6	7	0	0	5	5	6	5	2
SAL_FA2475AA	IEH-NGS-SAL-04066	Dublin	5	4	6	7	0	0	5	5	6	5	2
SAL_FA2297AA	PNUSAS001668	Dublin	5	4	6	7	0	0	5	5	6	5	2
SAL_FA2296AA	PNUSAS001669	Dublin	5	4	6	7	0	0	5	5	6	5	2
SAL_FA2106AA	201512961	Dublin	0	0	0	0	0	0	5	5	6	5	10
SAL_FA2093AA	201600576	Dublin	5	4	6	7	0	0	5	5	0	5	11
SAL_FA2105AA	201512397	Dublin	0	0	0	0	0	0	5	5	6	5	17
SAL_FA2092AA	201512678	Dublin	0	0	0	0	0	0	5	5	6	5	17
SAL_FA2090AA	201600655	Dublin	5	4	6	7	0	0	5	5	6	5	17
SAL_FA2096AA	201512160	Dublin	0	0	0	0	0	0	5	5	6	5	11
SAL_FA2100AA	201600397	Dublin	5	4	6	7	0	0	5	5	6	5	14
SAL_FA2102AA	201600621	Dublin	5	4	6	7	0	0	5	5	6	5	17
SAL_FA2094AA	201511734	Dublin	5	4	6	7	0	0	5	5	6	5	17
SAL_FA2103AA	201509572	Dublin	5	4	6	7	0	0	5	5	6	5	12
SAL_FA2095AA	201513183	Dublin	5	4	6	7	0	0	5	5	6	5	11
SAL_FA2091AA	201512914	Dublin	5	4	6	7	0	0	5	5	6	5	10
SAL_FA2086AA	201512704	Dublin	5	4	6	7	0	0	5	5	6	5	17
SAL_FA2083AA	201600758	Dublin	0	0	0	0	0	0	5	5	6	5	11
SAL_FA2085AA	201513089	Dublin	0	0	0	0	0	0	5	5	6	5	11
SAL_FA2084AA	201512476	Dublin	0	0	0	0	0	0	5	5	6	5	11
SAL_FA2081AA	201509526	Dublin	5	4	6	7	0	0	0	5	6	5	18
SAL_FA2082AA	201512475	Dublin	0	0	0	0	0	0	5	5	6	5	17
SAL_FA2077AA	201512002	Dublin	5	4	6	7	0	0	5	5	6	5	17
SAL_FA2076AA	201512887	Dublin	0	0	0	0	0	0	5	5	6	5	10
SAL_FA2080AA	201512134	Dublin	5	4	6	7	0	0	5	5	6	5	17
SAL_FA2075AA	201511232	Dublin	5	4	6	7	0	0	5	5	6	5	11
SAL_FA2079AA	201512619	Dublin	5	4	6	7	0	0	5	5	6	5	11
SAL_FA2074AA	201600396	Dublin	0	0	0	0	0	0	5	5	6	5	11
SAL_FA2073AA	201513005	Dublin	5	4	6	7	0	0	5	5	6	5	17
SAL_FA2072AA	201600601	Dublin	5	4	6	7	0	0	5	5	6	5	11
SAL_FA2067AA	201512007	Dublin	0	0	0	0	0	0	0	5	6	5	17
SAL_FA2071AA	201512702	Dublin	0	0	0	0	0	0	5	5	6	5	14

SAL_FA2065AA	201600681	Dublin	5	4	6	7	0	0	0	5	6	5	17
SAL_FA2066AA	201509941	Dublin	5	4	6	7	0	0	5	5	6	5	17
SAL_FA2069AA	201512557	Dublin	5	4	6	7	0	0	5	5	6	5	11
SAL_FA2062AA	201508626	Dublin	5	4	6	7	0	0	5	5	6	5	12
SAL_FA2064AA	201513007	Dublin	0	0	0	0	0	0	5	5	6	5	11
SAL_FA2060AA	201509058	Dublin	5	4	6	7	0	0	5	5	6	5	10
SAL_FA2058AA	93_1557	Dublin	5	4	6	7	0	0	0	0	0	0	0
SAL_FA2061AA	201508489	Dublin	5	4	6	7	0	0	5	5	6	5	14
SAL_FA2053AA	02_9836	Dublin	5	4	6	7	0	0	5	5	6	5	17
SAL_FA2044AA	92_9894	Dublin	5	4	6	7	0	0	5	5	6	5	10
SAL_FA2056AA	93_1086	Dublin	5	4	6	7	0	0	0	0	0	0	0
SAL_FA2049AA	08_6645	Dublin	5	4	6	7	0	0	5	5	6	5	10
SAL_FA2052AA	05_1078	Dublin	5	4	6	7	0	0	5	5	6	5	17
SAL_FA2041AA	02_1212	Dublin	5	4	6	7	0	0	5	5	6	5	11
SAL_FA2048AA	02_5213	Dublin	5	4	6	7	0	0	5	5	6	5	2
SAL_FA2045AA	93_5462	Dublin	5	4	6	7	0	0	5	5	0	0	17
SAL_FA2040AA	95_8062	Dublin	0	0	0	0	0	0	0	0	0	0	0
SAL_FA2042AA	00_7892	Dublin	5	4	6	7	0	0	5	5	6	5	10
SAL_FA2046AA	201208251	Dublin	5	4	6	8	0	0	5	53	51	5	1
SAL_FA2038AA	94_2023	Dublin	5	4	6	7	0	0	5	5	6	5	10
SAL_FA2025AA	00_8531	Dublin	5	4	6	7	0	0	0	5	6	5	11
SAL_FA2037AA	99_5828	Dublin	5	4	6	7	0	0	0	5	6	5	12
SAL_FA2022AA	03_2892	Dublin	5	4	6	7	0	0	5	5	84	5	17
SAL_FA2031AA	09_2054	Dublin	5	4	6	7	0	0	5	5	6	5	17
SAL_FA2032AA	93_3170	Dublin	5	4	6	7	0	0	0	0	0	0	0
SAL_FA2026AA	94_8298	Dublin	5	4	6	7	0	0	5	5	6	5	11
SAL_FA2027AA	56136	Dublin	5	4	6	7	0	0	5	5	6	5	17
SAL_FA2023AA	04_4663	Dublin	5	4	6	8	0	0	5	53	51	5	1
SAL_FA2007AA	20152408	Dublin	5	4	6	7	0	0	5	5	6	5	17
SAL_FA2006AA	201504890	Dublin	5	4	6	7	0	0	5	5	6	5	14
SAL_FA2008AA	20151141	Dublin	5	4	6	7	0	0	5	5	6	5	18
SAL_FA2005AA	201412089	Dublin	5	4	6	7	0	0	5	5	6	5	11
SAL_FA2004AA	201506168	Dublin	5	4	6	7	0	0	5	5	6	5	11
SAL_FA2003AA	201103545	Dublin	5	4	6	7	0	0	5	5	6	5	12
SAL_FA2001AA	201402488	Dublin	5	4	6	7	0	0	5	5	0	5	12
SAL_FA2000AA	201310897	Dublin	5	4	6	7	0	0	5	5	6	5	11
SAL_FA2002AA	20130275	Dublin	5	4	6	7	0	0	5	5	6	5	17
SAL_FA1716AA	CFSAN046151	Dublin	5	4	6	7	0	0	5	5	98	5	0
SAL_FA1713AA	CFSAN046162	Dublin	5	4	6	7	0	0	5	5	6	5	2
SAL_FA1711AA	CFSAN046161	Dublin	5	4	6	7	0	0	5	5	6	5	2
SAL_FA0768AA	CFSAN045655	Dublin	5	4	6	7	0	0	5	5	6	5	2
SAL_FA0769AA	CFSAN045654	Dublin	5	4	6	7	0	0	5	5	6	5	2
SAL_FA0641AA	NY-N20074	Dublin	5	4	6	7	0	0	5	5	6	5	2
SAL_FA0642AA	NY-N20073	Dublin	5	4	6	7	0	0	5	5	6	5	2
SAL_EA9976AA	1378_99-sc-2416992	Dublin	5	4	6	7	0	0	5	5	6	5	18
SAL_EA9973AA	7997_98-sc-2416995	Dublin	5	4	6	7	0	0	5	5	6	5	18
SAL_EA9882AA	FSIS1605442	Dublin	5	4	6	7	0	0	0	0	0	0	0
SAL_EA9328AA	1412T10279	Dublin	5	4	6	7	0	0	5	5	6	5	13
SAL_EA9132AA	CFSAN031745	Dublin	5	4	29	7	0	0	5	5	6	5	12
SAL_EA9123AA	CFSAN031746	Dublin	5	4	29	7	0	0	5	5	6	5	12
SAL_EA9035AA	WAPHL-SAL-A01075	Dublin	5	4	6	7	0	0	5	5	6	5	2
SAL_EA7794AA	58590	Dublin	5	4	6	7	0	0	5	5	6	5	17
SAL_EA7001AA	1503	Dublin	5	4	6	7	0	0	0	0	0	0	0
SAL_EA6738AA	ADRDL-119713-12-1-1	Dublin	5	4	6	7	0	0	5	5	6	5	2
SAL_EA6741AA	ADRDL-128975-11-1-1	Dublin	5	4	6	7	0	0	5	5	6	5	2
SAL_EA6743AA	ADRDL-104423-11-1-3	Dublin	5	4	6	7	0	0	5	5	6	5	2
SAL_EA6739AA	ADRDL-124410-12-2-1	Dublin	5	4	6	7	0	0	5	5	6	5	2
SAL_EA6742AA	ADRDL-120331-12-1-4	Dublin	5	4	6	7	0	0	5	5	6	5	2
SAL_EA6746AA	ADRDL-96471-09-2-3	Dublin	5	4	6	7	0	0	5	5	6	5	2
SAL_EA6744AA	ADRDL-53122-12-1-4	Dublin	5	4	6	7	0	0	5	5	6	5	2
SAL_EA6740AA	ADRDL-124307-11-1-1	Dublin	5	4	6	7	0	0	5	5	6	5	2
SAL_EA6745AA	ADRDL-122028-11-3-1	Dublin	5	4	6	7	0	0	5	5	6	5	2
SAL_EA6737AA	ADRDL-52965-11-1-3	Dublin	5	4	6	7	0	0	5	5	6	5	2
SAL_EA6574AA	ADRDL-92283-12-1-4	Dublin	5	4	6	7	0	0	5	0	6	5	2
SAL_EA6575AA	ADRDL-147375-11-2-1	Dublin	5	4	6	7	0	0	5	5	6	5	2
SAL_EA6572AA	ADRDL-119713-12-2-1	Dublin	5	4	6	7	0	0	5	5	6	5	2
SAL_EA6573AA	ADRDL-119715-12-1-1	Dublin	5	4	6	7	0	0	5	5	6	5	2
SAL_EA6570AA	ADRDL-66298-11-1-3	Dublin	5	4	6	7	0	0	5	5	6	5	2
SAL_EA6568AA	ADRDL-128976-11-1-1	Dublin	5	4	6	7	0	0	5	5	6	5	2
SAL_EA6571AA	ADRDL-92283-12-1-5	Dublin	5	4	6	7	0	0	5	5	6	5	2
SAL_EA6565AA	ADRDL-103463-12-1-2	Dublin	5	4	6	7	0	0	5	5	6	5	2
SAL_EA6567AA	ADRDL-130462-12-1-1	Dublin	5	4	6	7	0	0	5	5	6	5	2
SAL_EA6563AA	ADRDL-134051-11-2-3	Dublin	5	4	6	7	0	0	5	5	6	5	2

SAL_EA6566AA	ADRDL-9028-12-1-1	Dublin	5	4	6	7	0	0	5	5	6	5	2
SAL_EA6538AA	ADRDL-108158-10-2-1	Dublin	5	4	6	7	0	0	5	5	6	5	2
SAL_EA6537AA	ADRDL-122028-11-2-1	Dublin	5	4	6	7	0	0	5	5	6	5	2
SAL_EA6539AA	ADRDL-122028-11-1-1	Dublin	5	4	6	7	0	0	5	5	6	5	2
SAL_EA6354AA	ADRDL-125037-11-1-1	Dublin	5	4	6	7	0	0	5	5	6	5	2
SAL_EA6351AA	ADRDL-68048-11-1-7	Dublin	5	4	6	7	0	0	5	5	6	5	10
SAL_EA6344AA	ADRDL-9678-12-5-2	Dublin	5	4	6	7	0	0	5	5	6	5	2
SAL_EA6353AA	ADRDL-43556-11-1-1	Dublin	5	4	6	7	0	0	5	5	6	5	2
SAL_EA6350AA	ADRDL-85038-11-1-2	Dublin	5	4	6	7	0	0	5	5	6	5	2
SAL_EA6355AA	ADRDL-3534-12-1-2	Dublin	5	4	6	7	0	0	5	5	6	5	2
SAL_EA6349AA	ADRDL-132274-11-1-5	Dublin	5	4	6	7	0	0	5	5	6	5	2
SAL_EA6345AA	ADRDL-100227-09-5-1	Dublin	5	4	6	7	0	0	5	5	6	5	2
SAL_EA6347AA	ADRDL-133512-12-1-1	Dublin	5	4	6	7	0	0	5	5	6	5	2
SAL_EA6343AA	ADRDL-124410-12-1-1	Dublin	5	4	6	7	0	0	5	5	6	5	2
SAL_EA6346AA	ADRDL-85038-11-1-1	Dublin	5	4	6	7	0	0	5	5	6	5	2
SAL_EA6352AA	ADRDL-128141-12-1-4	Dublin	5	4	6	7	0	0	5	5	6	5	2
SAL_EA5745AA	TX-ACF1502685	Dublin	5	4	6	126	0	0	5	5	0	5	2
SAL_EA5699AA	PNUSAS001242	Dublin	5	4	6	7	0	0	5	5	6	5	2
SAL_EA5697AA	PNUSAS001244	Dublin	5	4	6	126	0	0	5	5	6	5	2
SAL_EA5696AA	PNUSAS001241	Dublin	5	4	6	126	0	0	5	5	6	5	2
SAL_EA5698AA	PNUSAS001243	Dublin	5	4	6	7	0	0	5	5	6	5	2
SAL_EA5614AA	FSIS1503487	Dublin	5	4	6	7	0	0	5	5	6	5	2
SAL_EA5498AA	ADRDL-14-18017	Dublin	5	4	6	7	0	0	5	5	6	5	2
SAL_EA5492AA	ADRDL-15-3768	Dublin	5	4	6	7	0	0	5	5	6	5	2
SAL_EA5496AA	ADRDL-15-4199	Dublin	5	4	6	7	0	0	5	5	6	5	2
SAL_EA5250AA	FSIS1503374	Dublin	5	4	6	7	0	0	5	5	6	5	2
SAL_EA5251AA	FSIS1503369	Dublin	5	4	6	7	0	0	5	5	6	5	2
SAL_EA5247AA	FSIS1503367	Dublin	5	4	6	7	0	0	5	5	6	5	2
SAL_EA5246AA	FSIS1503371	Dublin	5	4	6	7	0	0	5	5	6	5	2
SAL_EA5245AA	FSIS1503366	Dublin	5	0	6	7	0	0	5	5	6	5	0
SAL_EA5243AA	FSIS1503373	Dublin	5	4	6	7	0	0	0	0	0	5	0
SAL_EA5244AA	FSIS1503370	Dublin	5	4	6	7	0	0	5	5	6	5	2
SAL_EA5242AA	FSIS1503372	Dublin	5	4	6	7	0	0	0	0	0	0	0
SAL_EA5233AA	FSIS1503376	Dublin	5	4	6	7	0	0	5	5	6	5	2
SAL_EA4998AA	CFSAN034797	Dublin	5	4	6	7	0	0	5	5	6	5	2
SAL_EA4995AA	CFSAN034800	Dublin	5	4	6	7	0	0	5	5	0	5	2
SAL_EA4990AA	CFSAN034805	Dublin	5	4	6	7	0	0	5	5	6	5	2
SAL_EA4994AA	IEH-NGS-SAL-03263	Dublin	5	4	6	7	0	0	5	5	6	5	2
SAL_EA4999AA	IEH-NGS-SAL-03258	Dublin	5	4	6	7	0	0	5	5	6	5	2
SAL_EA4989AA	CFSAN034807	Dublin	5	4	6	7	0	0	5	5	6	5	2
SAL_EA4967AA	ADRDL-14-16643	Dublin	5	4	6	7	0	0	5	5	6	5	2
SAL_EA4972AA	ADRDL-14-17669	Dublin	5	4	6	7	0	0	5	5	6	5	2
SAL_EA4968AA	ADRDL-14-18441	Dublin	5	4	6	7	0	0	5	5	6	5	2
SAL_EA4963AA	ADRDL-14-17148	Dublin	5	4	6	7	0	0	5	5	6	5	2
SAL_EA4962AA	ADRDL-14-17229	Dublin	5	4	6	7	0	0	5	5	6	5	10
SAL_EA4675AA	CFSAN033300	Dublin	5	4	6	7	0	0	5	5	6	5	2
SAL_EA4679AA	CFSAN033296	Dublin	5	4	6	7	0	0	5	5	6	5	2
SAL_EA4682AA	IEH-NGS-SAL-03104	Dublin	5	4	6	7	0	0	5	5	6	5	2
SAL_EA4681AA	CFSAN033294	Dublin	5	4	6	7	0	0	5	5	6	5	2
SAL_EA4676AA	CFSAN033299	Dublin	5	4	6	7	0	0	5	5	6	5	2
SAL_EA4678AA	IEH-NGS-SAL-03108	Dublin	5	4	6	7	0	0	5	5	6	5	2
SAL_EA4677AA	IEH-NGS-SAL-03109	Dublin	5	4	6	7	0	0	5	5	6	5	2
SAL_EA3625AA	CVM N50446	Dublin	5	4	6	7	0	0	5	5	6	5	2
SAL_EA3609AA	CVM N51251	Dublin	5	4	6	7	0	0	5	5	6	5	2
SAL_EA3562AA	CVM N51298	Dublin	5	4	6	7	0	0	5	5	6	5	2
SAL_EA3469AA	BS_01603-sc-2322345	Dublin	5	4	6	7	0	0	5	5	6	5	10
SAL_EA3412AA	BS_17148-sc-2322425	Dublin	5	4	6	7	0	0	5	5	0	5	10
SAL_EA3411AA	BS_17488-sc-2322426	Dublin	5	4	6	7	0	0	5	5	6	5	10
SAL_EA3408AA	BS_08588-sc-2322429	Dublin	5	4	6	7	0	0	5	5	6	5	2
SAL_EA3397AA	BN_08157-sc-2322440	Dublin	5	4	6	7	0	0	5	5	6	5	2
SAL_EA3387AA	BN_02564-sc-2322450	Dublin	0	0	0	7	0	0	0	0	0	0	0
SAL_EA3389AA	BN_02289-sc-2322448	Dublin	5	4	6	7	0	0	5	5	6	5	10
SAL_EA3394AA	BN_02384-sc-2322443	Dublin	5	4	6	7	0	0	5	5	6	5	2
SAL_EA3393AA	BN_02524-sc-2322444	Dublin	5	4	6	7	0	0	5	5	6	5	10
SAL_EA3390AA	BN_04642-sc-2322447	Dublin	5	4	6	7	0	0	5	5	6	5	2
SAL_EA2837AA	FDA00000374	Dublin	0	0	0	0	0	0	0	0	0	0	0
SAL_EA2021AA	CVM N53083	Dublin	5	4	6	7	0	0	5	5	6	5	0
SAL_EA1943AA	CVM N54720	Dublin	5	4	6	7	0	0	5	5	6	5	2
SAL_EA0871AA	M277 / NCTC20092	Dublin	5	4	6	7	0	0	5	5	6	5	10
SAL_EA0850AA	M386 / NCTC20120	Dublin	5	4	6	7	0	0	5	5	6	5	0
SAL_EA0783AA	AZ_TG68352	Dublin	5	4	6	7	0	0	5	5	6	5	2
SAL_EA0777AA	AZ_TG68411	Dublin	5	4	6	7	0	0	5	5	6	5	2
SAL_EA0663AA	FSW0034	Dublin	0	0	0	0	0	0	0	0	0	0	0

SAL_EA0471AA	MDH-2013-00180	Dublin	5	4	6	7	0	0	5	5	6	5	2
SAL_EA0459AA	MDH-2013-00181	Dublin	5	4	6	7	0	0	5	5	6	5	2
SAL_EA0407AA	FCC0057	Dublin	5	4	6	7	0	0	5	5	6	5	2
SAL_EA0173AA	WAPHL_SAL-A00059	Dublin	5	4	6	7	0	0	5	5	6	5	0
SAL_EA0099AA	WAPHL_SAL-A00068	Dublin	5	4	6	7	0	0	5	5	6	5	0
SAL_EA0064AA	NY_IDR1200021877-01	Dublin	5	4	6	7	0	0	5	5	6	5	2
SAL_DA9636AA	WAPHL_SAL-A00066	Dublin	5	4	6	7	0	0	5	5	6	5	2
SAL_DA8064AA	WAPHL-SAL-A00348	Dublin	5	4	6	7	0	0	5	5	6	5	2
SAL_DA8058AA	WAPHL-SAL-A00349	Dublin	5	4	6	7	0	0	5	5	6	5	2
SAL_DA7782AA	MDH-2014-00098	Dublin	5	4	6	7	0	0	5	5	6	5	2
SAL_DA7529AA	MDH-2014-00095	Dublin	5	4	6	7	0	0	5	5	6	5	2
SAL_DA7501AA	MDH-2014-00092	Dublin	5	4	6	7	0	0	5	5	6	5	0
SAL_DA7320AA	MDH-2014-00071	Dublin	5	4	6	7	0	0	5	5	6	5	2
SAL_DA7252AA	MDH-2014-00053	Dublin	5	4	6	7	0	0	5	5	6	5	2
SAL_DA7121AA	MDH-2014-00036	Dublin	5	4	6	7	0	0	5	5	6	5	2
SAL_DA7117AA	MDH-2014-00004	Dublin	5	4	6	7	0	0	5	5	6	5	2
SAL_DA7101AA	MDH-2014-00013	Dublin	5	4	6	7	0	0	63	5	6	5	10
SAL_DA7073AA	MDH-2014-00027	Dublin	5	4	6	7	0	0	5	5	6	5	2
SAL_DA7022AA	IEH-NGS-SAL-00454	Dublin	5	4	6	7	0	0	5	5	6	5	2
SAL_DA7011AA	CFSAN013245	Dublin	5	4	6	7	0	0	5	5	6	5	2
SAL_DA7007AA	MDH-2014-00007	Dublin	5	4	6	7	0	0	5	5	6	5	2
SAL_DA7004AA	CFSAN012680	Dublin	5	4	6	7	0	0	63	5	6	5	10
SAL_DA6873AA	CFSAN013247	Dublin	5	4	6	7	0	0	5	5	6	5	1
SAL_DA6879AA	IEH-NGS-SAL-00441	Dublin	5	4	6	7	0	0	5	5	6	5	2
SAL_DA6814AA	IEH-NGS-SAL-00435	Dublin	5	4	6	7	0	0	5	5	6	5	2
SAL_DA6777AA	IEH-NGS-SAL-00457	Dublin	5	4	6	7	0	0	5	5	6	5	2
SAL_DA6783AA	IEH-NGS-SAL-00473	Dublin	5	4	6	7	0	0	63	5	6	5	11
SAL_DA6736AA	IEH-NGS-SAL-00458	Dublin	5	4	6	7	0	0	5	5	6	5	2
SAL_DA6680AA	IEH-NGS-SAL-00466	Dublin	5	4	6	7	0	0	5	5	6	5	2
SAL_DA6575AA	IEH-NGS-SAL-00523	Dublin	5	4	6	7	0	0	5	5	6	5	2
SAL_DA6568AA	WAPHL-SAL-A00399	Dublin	5	4	6	7	0	0	5	5	6	5	2
SAL_DA6523AA	CFSAN013323	Dublin	5	4	6	7	0	0	5	5	6	5	2
SAL_DA6508AA	IEH-NGS-SAL-00530	Dublin	5	4	74	7	0	0	5	5	6	5	2
SAL_DA6323AA	MDH-2014-00304	Dublin	5	4	6	7	0	0	5	5	6	5	2
SAL_DA6277AA	MDH-2014-00312	Dublin	5	4	6	7	0	0	5	5	6	5	2
SAL_DA6150AA	MDH-2014-00348	Dublin	5	4	6	7	0	0	5	5	6	5	2
SAL_DA6046AA	IEH-NGS-SAL-00680	Dublin	5	4	6	7	0	0	5	5	6	5	2
SAL_DA6009AA	CFSAN018490	Dublin	5	4	6	7	0	0	63	5	6	5	10
SAL_DA6011AA	CFSAN018531	Dublin	5	4	6	7	0	0	5	5	123	5	2
SAL_DA6001AA	CFSAN018491	Dublin	5	4	6	7	0	0	63	5	6	5	10
SAL_DA5989AA	IEH-NGS-SAL-00648	Dublin	5	4	6	7	0	0	63	5	6	5	10
SAL_DA5959AA	CFSAN018557	Dublin	5	4	6	7	0	0	5	5	6	5	0
SAL_DA5956AA	CFSAN018496	Dublin	5	4	6	7	0	0	5	5	6	5	0
SAL_DA5952AA	IEH-NGS-SAL-00642	Dublin	5	4	6	7	0	0	5	5	6	5	2
SAL_DA5942AA	CFSAN018523	Dublin	5	4	6	7	0	0	5	5	6	5	2
SAL_DA5937AA	CFSAN018503	Dublin	5	4	6	7	0	0	5	5	6	5	0
SAL_DA5771AA	IEH-NGS-SAL-00780	Dublin	5	4	6	7	0	0	5	5	6	5	0
SAL_DA5756AA	CFSAN018646	Dublin	5	4	6	7	0	0	5	5	6	5	2
SAL_DA5750AA	CFSAN018608	Dublin	5	4	6	7	0	0	5	5	6	5	2
SAL_DA5749AA	CFSAN018615	Dublin	5	4	6	7	0	0	5	5	6	5	0
SAL_DA5730AA	CFSAN018599	Dublin	5	4	6	7	0	0	5	5	123	5	2
SAL_DA5717AA	CFSAN018619	Dublin	5	4	6	7	0	0	5	5	6	5	2
SAL_DA5631AA	CFSAN018648	Dublin	5	4	6	7	0	0	5	5	6	5	0
SAL_DA5588AA	CFSAN018675	Dublin	5	4	6	7	0	0	5	5	6	5	2
SAL_DA5591AA	CFSAN018672	Dublin	5	4	6	7	0	0	5	5	6	5	2
SAL_DA5573AA	IEH-NGS-SAL-00855	Dublin	5	4	6	7	0	0	5	5	6	5	0
SAL_DA5564AA	IEH-NGS-SAL-00841	Dublin	5	4	6	7	0	0	5	5	6	5	0
SAL_DA5565AA	IEH-NGS-SAL-00840	Dublin	5	4	6	7	0	0	5	5	6	5	2
SAL_DA5551AA	CFSAN018716	Dublin	5	4	6	7	0	0	5	5	6	5	2
SAL_DA5507AA	CFSAN018730	Dublin	5	4	6	7	0	0	5	5	6	5	2
SAL_DA5498AA	IEH-NGS-SAL-00876	Dublin	5	4	6	7	0	0	5	5	6	5	0
SAL_DA5474AA	CFSAN018733	Dublin	5	4	6	7	0	0	5	5	6	5	2
SAL_DA5430AA	CFSAN022426	Dublin	5	4	6	7	0	0	63	5	6	5	11
SAL_DA5427AA	IEH-NGS-SAL-00946	Dublin	5	4	6	7	0	0	5	5	6	5	2
SAL_DA5422AA	IEH-NGS-SAL-00922	Dublin	5	4	6	7	0	0	5	5	6	5	2
SAL_DA5405AA	CFSAN022431	Dublin	5	4	6	7	0	0	5	5	6	5	2
SAL_DA5416AA	CFSAN022423	Dublin	5	4	6	7	0	0	63	5	6	5	11
SAL_DA5406AA	CFSAN022419	Dublin	5	4	6	7	0	0	5	5	6	5	2
SAL_DA5402AA	IEH-NGS-SAL-00939	Dublin	5	4	6	7	0	0	5	5	6	5	2
SAL_DA5387AA	CFSAN022442	Dublin	5	4	6	7	0	0	5	5	6	5	2
SAL_DA5391AA	CFSAN022424	Dublin	5	4	6	7	0	0	63	5	6	5	11
SAL_DA5378AA	CFSAN022428	Dublin	5	4	6	7	0	0	5	5	6	5	2
SAL_DA5386AA	CFSAN022446	Dublin	5	4	6	7	0	0	5	5	6	5	2

SAL_DA5377AA	CFSAN022429	Dublin	5	4	6	7	0	0	5	5	6	5	2
SAL_DA5368AA	IEH-NGS-SAL-00914	Dublin	5	4	6	7	0	0	63	5	6	5	10
SAL_DA5367AA	CFSAN022453	Dublin	5	4	6	7	0	0	63	5	6	5	10
SAL_DA5346AA	DRS014085	Dublin	5	4	6	8	0	0	5	53	51	5	1
SAL_DA5339AA	CFSAN022412	Dublin	5	4	6	7	0	0	5	5	6	5	2
SAL_DA5334AA	CFSAN022700	Dublin	5	4	6	7	0	0	5	5	6	5	0
SAL_DA5164AA	IEH-NGS-SAL-00995	Dublin	5	4	6	7	0	0	5	5	6	5	2
SAL_DA5176AA	IEH-NGS-SAL-00996	Dublin	5	4	6	7	0	0	5	5	6	5	2
SAL_DA4722AA	P6	Dublin	5	4	6	7	0	0	5	5	126	5	11
SAL_DA4721AA	J45	Dublin	5	4	6	7	0	0	5	5	6	5	11
SAL_DA4720AA	I82	Dublin	5	4	6	7	0	0	5	5	6	5	10
SAL_DA4719AA	I76	Dublin	5	4	6	7	0	0	5	5	6	5	10
SAL_DA4718AA	I4	Dublin	5	4	6	7	0	0	5	5	6	5	10
SAL_DA4714AA	D62	Dublin	5	4	6	7	0	0	5	5	6	5	10
SAL_DA4419AA	MDH-2014-00077	Dublin	5	4	6	7	0	0	5	5	6	104	2
SAL_DA4295AA	MOD1-Sal_904	Dublin	5	4	6	7	0	0	5	5	6	5	2
SAL_DA4229AA	CFSAN022573	Dublin	5	4	6	7	0	0	5	5	123	5	2
SAL_DA4199AA	MDH-2014-00425	Dublin	5	4	6	7	0	0	5	5	6	5	2
SAL_DA4042AA	MDH-2014-00450	Dublin	5	4	6	7	0	0	5	5	6	5	2
SAL_DA4023AA	MDH-2014-00455	Dublin	5	4	6	7	0	0	5	5	6	5	2
SAL_DA4030AA	MDH-2014-00451	Dublin	5	4	6	7	0	0	5	5	6	5	2
SAL_DA4003AA	CFSAN023151	Dublin	5	4	6	7	0	0	5	5	6	5	2
SAL_DA3429AA	CFSAN023590	Dublin	5	4	6	7	0	0	5	5	6	5	0
SAL_DA3334AA	IEH-NGS-SAL-01272	Dublin	5	4	6	7	0	0	5	5	6	5	2
SAL_DA3023AA	MDH-2014-00420	Dublin	5	4	6	7	0	0	5	5	6	5	2
SAL_DA3004AA	IEH-NGS-SAL-01037	Dublin	5	4	6	7	0	0	63	5	6	5	10
SAL_DA2930AA	MDH-2014-00513	Dublin	5	4	6	7	0	0	5	5	6	5	2
SAL_DA2577AA	MDH-2014-00538	Dublin	5	4	6	7	0	0	5	5	6	5	2
SAL_DA2575AA	MDH-2014-00544	Dublin	5	4	6	7	0	0	63	5	6	5	17
SAL_DA2465AA	WAPHL_SAL-A00575	Dublin	5	4	6	7	0	0	5	5	6	5	2
SAL_DA2454AA	MDH-2014-00546	Dublin	5	4	6	7	0	0	5	5	6	5	2
SAL_DA2450AA	MDH-2014-00552	Dublin	5	4	6	7	0	0	5	5	6	5	2
SAL_DA2355AA	MDH-2014-00565	Dublin	5	4	6	7	0	0	5	5	6	5	2
SAL_DA1676AA	FSIS1400280	Dublin	5	4	6	7	0	0	0	0	0	0	2
SAL_DA1259AA	H124440644	Dublin	5	4	6	7	0	0	5	5	6	5	18
SAL_DA1031AA	H123940588	Dublin	0	0	0	0	0	0	0	0	0	0	0
SAL_DA0931AA	H121940493	Dublin	5	4	6	7	0	0	5	5	6	5	18
SAL_DA0494AA	H121060572	Dublin	5	4	6	7	0	0	5	5	6	5	18
SAL_CA9246AA	FDA00003823	Dublin	0	0	0	0	0	0	0	0	0	0	0
SAL_CA8585AA	FDA00008717	Dublin	5	4	6	7	0	0	5	5	0	5	2
SAL_CA8245AA	NY-N28	Dublin	5	4	6	7	0	0	5	5	6	5	2
SAL_CA6869AA	2015K-0183	Dublin	5	4	6	7	0	0	5	5	6	5	0
SAL_CA6458AA	60142	Dublin	5	4	6	7	0	0	5	5	6	5	18
SAL_CA6407AA	54013	Dublin	0	0	0	0	0	0	0	0	0	0	0
SAL_CA6377AA	63551	Dublin	5	4	6	7	0	0	5	5	83	5	10
SAL_CA6278AA	57615	Dublin	0	0	0	0	0	0	0	0	0	0	0
SAL_CA5979AA	66490	Dublin	5	4	6	7	0	0	5	5	6	5	18
SAL_CA5512AA	75678	Dublin	5	4	6	7	0	0	5	5	6	5	18
SAL_CA5289AA	79091	Dublin	5	4	6	7	0	0	5	5	6	5	11
SAL_CA5095AA	78763	Dublin	5	4	6	7	0	0	5	5	6	5	2
SAL_CA5041AA	78738	Dublin	5	4	6	7	0	0	5	5	6	5	17
SAL_CA4959AA	70665	Dublin	5	4	6	7	0	0	5	5	6	5	18
SAL_CA4892AA	69756	Dublin	5	4	6	7	0	0	5	5	6	5	18
SAL_CA4855AA	72280	Dublin	5	4	6	7	0	0	5	5	6	5	18
SAL_CA4610AA	13813	Dublin	5	4	6	7	0	0	5	5	6	5	18
SAL_CA4551AA	61891	Dublin	5	4	6	7	0	0	5	5	6	5	18
SAL_CA4443AA	73113	Dublin	5	4	6	7	0	0	5	5	6	5	18
SAL_CA4334AA	93566	Dublin	5	4	6	7	0	0	5	5	6	5	18
SAL_CA4021AA	83707	Dublin	5	4	89	7	0	0	5	5	6	5	11
SAL_CA3949AA	5374	Dublin	0	0	0	0	0	0	0	0	0	0	0
SAL_CA3717AA	5372	Dublin	5	4	6	7	0	0	5	5	6	5	18
SAL_CA3621AA	89884	Dublin	5	4	6	7	0	0	5	5	6	5	18
SAL_CA3099AA	69901	Dublin	5	4	6	7	0	0	5	5	6	5	18
SAL_CA0811AA	84430	Dublin	5	4	6	7	0	0	5	5	6	5	18
SAL_CA0294AA	13858	Dublin	5	4	6	7	0	0	5	5	6	0	18
SAL_CA0073AA	6456	Dublin	5	4	6	7	0	0	5	5	6	5	11
SAL_BA9910AA	75976	Dublin	5	4	6	7	0	0	5	5	6	5	18
SAL_BA9878AA	94099	Dublin	5	4	6	7	0	0	5	5	6	5	18
SAL_BA9722AA	:-2012-07-27T11:52:12Z-	Dublin	5	4	6	7	0	0	5	5	6	5	2
SAL_BA9648AA	85738	Dublin	5	4	89	7	0	0	5	5	6	5	11
SAL_BA9502AA	blin_2229-sc-2012-09-04	Dublin	5	4	6	7	0	0	5	5	6	5	10
SAL_BA9360AA	83708	Dublin	5	4	89	7	0	0	5	5	6	5	11
SAL_BA8551AA	WAPHL_SAL-A00036	Dublin	5	4	6	7	0	0	5	5	6	5	0

SAL_BA8546AA	WAPHL_SAL-A00039	Dublin	5	4	6	7	0	0	5	5	6	5	0
SAL_BA8064AA	73112	Dublin	5	4	6	7	0	0	5	5	6	5	18
SAL_BA7951AA	42170	Dublin	5	4	6	7	0	0	5	5	6	5	18
SAL_BA7444AA	61912	Dublin	5	4	6	7	0	0	5	5	6	5	10
SAL_BA7408AA	2015K-0219	Dublin	5	4	6	7	0	0	5	5	6	0	2
SAL_BA7406AA	2015K-0222	Dublin	5	4	6	7	0	0	5	5	98	5	2
SAL_BA6641AA	2443687	Dublin	5	4	6	7	0	0	5	5	6	5	29
SAL_BA6543AA	15MN00093	Dublin	5	4	6	7	0	0	5	5	98	5	2
SAL_BA4972AA	FDA00008979	Dublin	5	4	6	7	0	0	5	5	6	5	17
SAL_BA4821AA	CFSAN022635	Dublin	5	4	6	7	0	0	0	0	0	0	0
SAL_BA4817AA	CFSAN022636	Dublin	5	4	6	7	0	0	0	0	0	0	0
SAL_BA4700AA	CFSAN005664	Dublin	5	4	6	7	0	0	5	5	6	5	0
SAL_BA4698AA	CFSAN005665	Dublin	5	4	6	7	0	0	5	5	6	5	2
SAL_BA4060AA	PNUSAS000200	Dublin	5	4	6	7	0	0	5	5	98	5	2
SAL_BA4062AA	PNUSAS000199	Dublin	5	4	6	7	0	0	5	5	98	5	2
SAL_BA4058AA	PNUSAS000201	Dublin	5	4	6	7	0	0	5	5	0	5	0
SAL_BA3605AA	WAPHL_SAL-A00048	Dublin	5	4	6	7	0	0	5	5	6	5	0
SAL_BA3561AA	TX-ACF1400064	Dublin	5	4	6	7	0	0	5	5	6	5	2
SAL_BA3156AA	WAPHL_SAL-A00801	Dublin	5	4	6	7	0	0	5	5	6	0	2
SAL_BA2959AA	AZ_TG68224	Dublin	5	4	6	7	0	0	5	5	6	5	2
SAL_BA2618AA	ADRDL-14-8408	Dublin	5	4	6	7	0	0	5	5	6	5	2
SAL_BA2611AA	ADRDL-14-15777	Dublin	5	4	6	7	0	0	5	5	6	5	2
SAL_BA2603AA	ADRDL-14-13879	Dublin	5	4	6	7	0	0	5	5	6	5	2
SAL_BA2605AA	ADRDL-14-14468	Dublin	5	4	6	7	0	0	5	5	6	5	0
SAL_BA2065AA	ADRDL-14-19429	Dublin	5	4	6	7	0	0	5	5	6	5	2
SAL_BA2049AA	ADRDL-14-15921	Dublin	5	4	6	7	0	0	5	5	6	5	2
SAL_BA2016AA	ADRDL-14-12435	Dublin	5	4	6	7	0	0	5	5	6	5	0
SAL_BA2009AA	ADRDL-14-18584	Dublin	5	4	6	7	0	0	5	5	6	5	2
SAL_BA2005AA	ADRDL-14-19293	Dublin	5	4	6	7	0	0	5	5	6	5	2
SAL_BA2004AA	ADRDL-14-19507	Dublin	5	4	6	7	0	0	5	5	6	5	2
SAL_BA1998AA	ADRDL-14-7489	Dublin	5	4	6	7	0	0	5	5	0	5	2
SAL_BA1929AA	ADRDL-14-10989	Dublin	5	4	6	7	0	0	5	5	6	5	2
SAL_BA1916AA	ADRDL-14-19199	Dublin	5	4	6	7	0	0	5	5	6	5	2
SAL_BA1889AA	ADRDL-14-20267	Dublin	5	4	6	7	0	0	5	5	6	5	2
SAL_BA1469AA	FSIS1503217	Dublin	5	4	6	7	0	0	5	5	6	5	0
SAL_BA0727AA	ADRDL-14-8649	Dublin	5	4	6	7	0	0	5	5	6	5	2
SAL_BA0719AA	ADRDL-14-11772	Dublin	5	4	6	7	0	0	5	5	6	5	0
SAL_BA0704AA	ADRDL-14-14363	Dublin	5	4	6	7	0	0	5	5	6	5	2
SAL_AA9882AA	c-2013-10-08T14:27:12Z	Dublin	5	4	6	7	0	0	5	5	6	5	10
SAL_AA9880AA	c-2013-10-08T14:27:15Z	Dublin	5	4	6	7	0	0	5	5	6	5	11
SAL_AA9865AA	c-2013-10-08T14:27:19Z	Dublin	5	4	6	7	0	0	0	5	6	5	2
SAL_AA9866AA	c-2013-10-08T14:27:18Z	Dublin	5	4	6	7	0	0	5	5	6	5	10
SAL_AA9673AA	852K	Dublin	5	4	6	7	0	0	0	0	0	0	0
SAL_AA9675AA	99-02017-2	Dublin	0	0	0	0	0	0	0	0	0	0	0
SAL_AA9661AA	SARB13_FB	Dublin	5	4	6	8	0	0	5	53	51	5	1
SAL_AA8712AA	PO400-sc-1963262	Dublin	5	4	6	7	0	0	5	5	6	5	10
SAL_AA8709AA	PO527-sc-1963265	Dublin	5	4	6	7	0	0	5	5	6	5	14
SAL_AA8686AA	PO902-sc-1965389	Dublin	5	4	6	7	0	0	5	5	6	5	29
SAL_AA8477AA	21-sc-1925583	Dublin	5	4	6	7	0	0	5	5	6	5	11
SAL_AA8476AA	22-sc-1925584	Dublin	5	4	6	7	0	0	5	5	6	5	10
SAL_AA8480AA	18-sc-1925580	Dublin	5	4	6	7	0	0	5	5	6	5	18
SAL_AA8475AA	23-sc-1925585	Dublin	5	4	6	7	0	0	5	5	6	5	11
SAL_AA8473AA	25-sc-1925587	Dublin	5	4	6	7	0	0	5	5	6	5	17
SAL_AA8472AA	1-sc-1925563	Dublin	5	4	6	7	0	0	5	5	6	5	17
SAL_AA8478AA	20-sc-1925582	Dublin	5	4	6	7	0	0	5	5	83	5	10
SAL_AA8474AA	24-sc-1925586	Dublin	5	4	6	7	0	0	5	5	6	5	11
SAL_AA8468AA	5-sc-1925567	Dublin	5	4	6	7	0	0	5	5	6	5	18
SAL_AA8469AA	4-sc-1925566	Dublin	5	4	6	7	0	0	5	5	6	5	18
SAL_AA8470AA	3-sc-1925565	Dublin	5	4	6	7	0	0	5	5	6	5	18
SAL_AA8467AA	6-sc-1925568	Dublin	5	4	6	7	0	0	5	5	6	5	18
SAL_AA8466AA	7-sc-1925569	Dublin	5	4	6	7	0	0	5	5	6	5	18
SAL_AA8465AA	8-sc-1925570	Dublin	5	4	6	7	0	0	5	5	6	5	18
SAL_AA8464AA	9-sc-1925571	Dublin	5	4	6	7	0	0	5	5	6	5	18
SAL_AA8463AA	10-sc-1925572	Dublin	5	4	6	7	0	0	5	5	6	5	18
SAL_AA8462AA	11-sc-1925573	Dublin	5	4	6	7	0	0	5	5	6	5	18
SAL_AA8461AA	12-sc-1925574	Dublin	5	4	6	7	0	0	5	5	6	5	29
SAL_AA8460AA	13-sc-1925575	Dublin	5	4	6	7	0	0	5	5	6	5	11
SAL_AA8459AA	14-sc-1925576	Dublin	5	4	6	7	0	0	41	5	6	5	2
SAL_AA8456AA	17-sc-1925579	Dublin	5	4	6	7	0	0	5	5	6	5	12
SAL_AA8457AA	16-sc-1925578	Dublin	5	4	6	7	0	0	5	5	6	5	11
SAL_AA8301AA	BH1226-sc-1993752	Dublin	5	4	89	7	0	0	5	5	6	5	10
SAL_AA7538AA	G192-sc-1993624	Dublin	5	4	6	7	0	0	5	5	6	5	10
SAL_AA7520AA	G1577-sc-1993769	Dublin	5	4	6	7	0	0	5	5	6	5	0

SAL_FA6047AA	PNUSAS001777	Dublin	5	4	6	7	0	0	5	5	98	5	2
SAL_FA6189AA	FSIS1503838	Dublin	5	4	6	7	0	0	5	5	6	5	2
SAL_FA6160AA	FSIS1503968	Dublin	5	4	6	7	0	0	5	5	6	5	2
SAL_FA6161AA	FSIS1503976	Dublin	5	4	6	7	0	0	5	5	6	5	2
SAL_FA6349AA	CFSAN045618	Dublin	5	4	6	7	0	0	5	5	6	5	2
SAL_FA6348AA	CFSAN045619	Dublin	5	4	6	7	0	0	5	5	6	5	2
SAL_FA6822AA	E2016006808	Dublin	5	4	6	7	0	0	5	5	6	5	11
SAL_FA7117AA	ADRDL15-18346	Dublin	5	4	6	7	0	0	5	5	6	5	2
SAL_FA7123AA	ADRDL15-17530	Dublin	5	4	6	7	0	0	5	5	6	5	10
SAL_FA7423AA	ADRDL15-26075	Dublin	5	4	6	7	0	0	5	5	6	5	2
SAL_FA7419AA	ADRDL15-26265	Dublin	5	4	6	7	0	0	5	5	6	5	2
SAL_FA7418AA	ADRDL15-26541	Dublin	5	4	6	7	0	0	5	5	6	5	2
SAL_FA7422AA	ADRDL15-26664	Dublin	5	4	6	7	0	0	5	5	6	5	2
SAL_FA7676AA	8062	Dublin	0	0	0	0	0	0	0	0	0	0	0
SAL_FA7679AA	5828	Dublin	5	4	6	7	0	0	5	5	6	5	12
SAL_FA7680AA	7892	Dublin	5	4	6	7	0	0	5	5	6	5	10
SAL_FA7681AA	8531	Dublin	5	4	6	7	0	0	5	5	6	5	11
SAL_FA7683AA	1212	Dublin	5	4	6	7	0	0	5	5	0	5	11
SAL_FA7684AA	5213	Dublin	5	4	6	7	0	0	5	5	6	5	2
SAL_FA7688AA	6136	Dublin	5	4	6	7	0	0	5	5	6	5	17
SAL_FA7690AA	6645	Dublin	5	4	6	7	0	0	5	5	6	5	10
SAL_FA7692AA	PNUSAS002291	Dublin	5	4	6	7	0	0	5	5	6	5	2
SAL_FA7700AA	201005507	Dublin	5	4	6	7	0	0	0	0	0	0	0
SAL_FA7702AA	201200586	Dublin	5	4	6	7	0	0	5	5	6	5	10
SAL_FA7704AA	201409292	Dublin	0	0	0	0	0	0	0	0	0	0	0
SAL_FA7847AA	SC50	Dublin	5	4	6	7	0	0	5	5	6	5	10
SAL_FA7849AA	SDU1	Dublin	5	4	6	7	0	0	5	5	6	5	10
SAL_FA7850AA	SDU2	Dublin	5	4	6	7	0	0	5	5	6	5	10
SAL_FA8108AA	ADRDL15-20929	Dublin	5	4	6	7	0	0	5	5	6	5	2
SAL_FA8107AA	ADRDL15-19200	Dublin	5	4	6	7	0	0	5	5	6	5	1
SAL_FA8106AA	ADRDL15-21554	Dublin	5	4	6	7	0	0	5	5	6	5	2
SAL_FA8104AA	ADRDL15-22176	Dublin	5	4	6	7	0	0	5	5	6	5	2
SAL_EA3352AA	BS_3212-sc-2322485	Dublin	5	4	6	7	0	0	5	5	6	5	10
SAL_EA3381AA	BN_03241-sc-2322456	Dublin	5	4	6	7	0	0	5	5	6	5	10
SAL_EA3399AA	BN_02376-sc-2322438	Dublin	5	4	6	7	0	0	5	5	6	5	10
SAL_EA3445AA	BS_15905-sc-2322370	Dublin	5	4	6	7	0	0	5	5	6	5	2
SAL_FA2097AA	201600765	Dublin	5	4	6	7	0	0	5	5	6	5	17
SAL_FA2070AA	201600501	Dublin	5	4	6	7	0	0	5	5	6	5	17
SAL_FA7127AA	ADRDL15-17480	Dublin	5	4	6	7	0	0	5	5	6	5	2
SAL_FA7126AA	ADRDL15-18038	Dublin	5	4	6	7	0	0	5	5	6	5	0
SAL_FA7125AA	ADRDL15-16824	Dublin	5	4	6	7	0	0	5	5	6	5	2
SAL_GA2773AA	CRJJGF_00140	Dublin	5	4	6	7	0	0	5	5	6	5	2
SAL_GA2764AA	CRJJGF_00149	Dublin	5	4	6	7	0	0	63	5	6	5	11
SAL_GA2763AA	CRJJGF_00150	Dublin	5	4	6	7	0	0	5	5	6	0	2
SAL_GA4001AA	230A/02	Dublin	5	4	6	7	0	0	5	5	6	5	11
SAL_GA3999AA	2356/02	Dublin	5	4	6	7	0	0	5	5	6	5	1
SAL_GA3996AA	2272/02	Dublin	5	4	6	7	0	0	5	5	6	5	18
SAL_GA3636AA	BCW_2676	Dublin	5	4	6	7	0	0	5	5	6	5	10
SAL_GA3526AA	BCW_2833	Dublin	5	4	6	7	0	0	5	5	6	5	0
SAL_GA3367AA	100065	Dublin	5	4	6	7	0	0	5	5	6	5	1
SAL_GA3309AA	IVB 3540/24	Dublin	0	0	0	0	0	0	0	0	0	0	0
SAL_GA3226AA	N27414	Dublin	5	4	6	7	0	0	5	5	6	5	2
SAL_GA3121AA	BCW_3413	Dublin	5	4	6	7	0	0	5	5	6	5	2
SAL_GA3079AA	BCW_4359	Dublin	5	4	6	7	0	0	5	5	6	5	2
SAL_GA2884AA	CRJJGF_00027	Dublin	5	4	6	7	0	0	5	5	6	5	0
SAL_GA2883AA	CRJJGF_00028	Dublin	5	4	6	7	0	0	5	5	0	5	2
SAL_GA2438AA	BCW_1977	Dublin	5	4	6	7	0	0	5	5	6	5	2
SAL_GA4540AA	FDA00001707	Dublin	0	0	0	0	0	0	0	0	0	0	0
SAL_GA2228AA	BCW_6007	Dublin	5	4	6	7	0	0	5	5	6	5	2
SAL_GA2119AA	BCW_6569	Dublin	5	4	6	7	0	0	5	5	6	5	2
SAL_GA2118AA	BCW_6571	Dublin	5	4	6	7	0	0	5	5	6	5	2
SAL_GA2115AA	BCW_6599	Dublin	5	4	6	7	0	0	5	5	6	5	2
SAL_GA2112AA	BCW_6620	Dublin	5	4	6	7	0	0	5	5	6	5	2
SAL_GA2111AA	BCW_6621	Dublin	5	4	6	7	0	0	5	5	0	5	2
SAL_GA2110AA	BCW_6623	Dublin	5	4	6	7	0	0	5	5	6	5	2
SAL_GA2105AA	BCW_6659	Dublin	5	4	6	7	0	0	5	5	6	5	2
SAL_GA2102AA	BCW_6662	Dublin	5	4	6	7	0	0	5	5	6	5	2
SAL_GA2055AA	BCW_3370	Dublin	5	4	6	7	0	0	63	5	6	5	10
SAL_GA2031AA	BCW_4354	Dublin	5	4	6	7	0	0	5	5	6	5	2
SAL_GA2029AA	BCW_4366	Dublin	5	4	6	7	0	0	5	5	6	5	2
SAL_GA2026AA	BCW_4385	Dublin	5	4	6	7	0	0	5	5	6	5	2
SAL_GA2024AA	BCW_4387	Dublin	5	4	6	7	0	0	5	5	6	5	2
SAL_GA1647AA	BCW_4356	Dublin	5	4	6	7	0	0	5	5	6	5	2

SAL_GA1624AA	SWUN 3710	Dublin	5	4	6	7	0	0	5	5	6	5	1
SAL_GA3994AA	2221/02	Dublin	5	4	6	7	0	0	5	5	6	5	18
SAL_GA3635AA	BCW_2677	Dublin	5	4	6	7	0	0	5	5	6	5	2
SAL_GA0998AA	gha114376-sc-2235223	Dublin	5	4	6	7	0	0	5	5	6	5	10
SAL_GA0990AA	gha73157-sc-2235715	Dublin	5	4	6	7	0	0	5	5	83	5	17
SAL_GA0987AA	gha77958-sc-2235750	Dublin	5	4	6	7	0	0	5	5	6	5	12
SAL_GA0986AA	gha97393-sc-2235765	Dublin	5	4	6	7	0	0	5	5	6	5	10
SAL_GA0962AA	bkf01027-sc-2235123	Dublin	5	4	6	7	0	0	5	5	6	5	17
SAL_GA0911AA	gha115071-sc-2235249	Dublin	5	4	6	7	0	0	5	5	6	5	10
SAL_GA0909AA	gha114424-sc-2235253	Dublin	5	4	6	7	0	0	5	5	6	5	10
SAL_GA0901AA	gha114361-sc-2235268	Dublin	5	4	6	7	0	0	5	5	6	5	10
SAL_GA0871AA	gha114080-sc-2235303	Dublin	5	4	6	7	0	0	5	5	6	5	10
SAL_GA0837AA	gha98835-sc-2235416	Dublin	5	4	6	7	0	0	5	5	6	5	10
SAL_GA0781AA	gha915118-sc-2235494	Dublin	5	4	6	7	0	0	5	5	83	5	11
SAL_GA0747AA	gha601123-sc-2235550	Dublin	1	1	0	7	1	1	5	5	6	5	10
SAL_GA0620AA	gha56474-sc-2235696	Dublin	5	4	6	7	0	0	5	5	6	5	11
SAL_GA0586AA	bkf00635-sc-2235211	Dublin	5	4	6	7	0	0	5	5	6	5	14
SAL_GA0558AA	gha115613-sc-2235094	Dublin	5	4	6	7	0	0	5	5	6	5	10
SAL_GA0559AA	gha114720-sc-2235092	Dublin	5	4	6	7	0	0	5	5	6	5	0
SAL_GA0551AA	gha114586-sc-2235108	Dublin	5	4	6	7	0	0	5	5	98	5	12
SAL_GA0527AA	gha114964-sc-2235157	Dublin	5	4	6	7	0	0	5	5	6	5	0
SAL_GA0526AA	gha115206-sc-2235159	Dublin	5	4	6	7	0	0	5	5	83	5	17
SAL_GA0510AA	gha114424-sc-2235193	Dublin	5	4	6	7	0	0	5	5	6	5	17
SAL_GA0505AA	gha115601-sc-2235202	Dublin	5	4	6	7	0	0	5	5	83	5	10
SAL_GA0503AA	gha400791-sc-2235207	Dublin	5	4	6	7	0	0	5	5	83	5	12
SAL_GA0479AA	gha114645-sc-2235411	Dublin	5	4	6	7	0	0	5	5	6	5	12
SAL_GA0459AA	gha910147-sc-2235317	Dublin	5	4	6	7	0	0	5	5	6	5	10
SAL_GA0421AA	gha911037-sc-2235398	Dublin	5	4	6	7	0	0	5	5	6	5	10
SAL_GA0263AA	SR041-sc-2280758	Dublin	0	0	0	0	0	0	0	0	0	0	0
SAL_GA4947AA	CDPHFDLB-F1605029-002	Dublin	5	4	6	7	0	0	5	5	6	5	2
SAL_GA4946AA	CDPHFDLB-F1605029-003	Dublin	5	4	6	7	0	0	5	5	6	5	2
SAL_GA4948AA	CDPHFDLB-F1605029-001	Dublin	5	4	6	7	0	0	5	5	0	5	2
SAL_GA4945AA	CDPHFDLB-F1606007-001	Dublin	5	4	6	7	0	0	5	5	6	5	2
SAL_GA4944AA	CDPHFDLB-F1606007-002	Dublin	5	4	6	7	0	0	5	5	6	5	2
SAL_GA4902AA	FSIS1607122	Dublin	5	4	6	7	0	0	0	0	0	0	0
SAL_GA4687AA	FSIS1502720	Dublin	5	4	6	7	0	0	5	5	6	5	2
SAL_GA5792AA	CK_03/00000995	Dublin	5	4	6	7	0	0	5	5	6	5	11
SAL_FA9344AA	CFSAN046191	Dublin	5	4	6	7	0	0	5	5	6	5	2
SAL_FA9343AA	CFSAN046192	Dublin	5	4	6	7	0	0	5	5	6	5	2
SAL_FA9338AA	CFSAN046197	Dublin	5	4	6	7	0	0	5	5	6	5	2
SAL_FA9337AA	CFSAN046198	Dublin	5	4	6	7	0	0	5	5	6	5	2
SAL_FA9335AA	CFSAN046200	Dublin	5	4	6	7	0	0	5	5	6	5	2
SAL_FA9336AA	CFSAN046199	Dublin	5	4	6	7	0	0	5	5	6	5	2
SAL_FA9345AA	CFSAN046190	Dublin	5	4	6	7	0	0	5	5	6	5	2
SAL_FA9334AA	CFSAN046201	Dublin	5	4	6	7	0	0	5	5	6	5	2
SAL_FA9331AA	CFSAN046204	Dublin	5	4	6	7	0	0	5	5	6	5	2
SAL_GA5113AA	PNUSAS002704	Dublin	5	4	6	7	0	0	5	5	6	5	2
SAL_FA9317AA	CFSAN046218	Dublin	5	4	6	7	0	0	5	5	6	5	2
SAL_FA9318AA	CFSAN046217	Dublin	5	4	6	7	0	0	5	5	6	5	2
SAL_FA9314AA	CFSAN046975	Dublin	5	4	6	7	0	0	5	5	6	5	2
SAL_FA9316AA	CFSAN046219	Dublin	5	4	6	7	0	0	5	5	6	5	2
SAL_FA9297AA	CFSAN046978	Dublin	5	4	6	7	0	0	5	5	6	5	2
SAL_FA9296AA	CFSAN046979	Dublin	5	4	6	7	0	0	5	5	6	5	2
SAL_GA5473AA	PNUSAS002619	Dublin	5	4	6	7	0	0	5	5	6	5	10
SAL_GA5460AA	PNUSAS002623	Dublin	5	4	6	7	0	0	5	5	6	5	2
SAL_FA9011AA	CVM N41733	Dublin	5	4	6	7	0	0	5	5	6	5	2
SAL_FA8879AA	CVM N42502	Dublin	5	4	6	7	0	0	5	5	6	5	2
SAL_FA8869AA	CVM N42512	Dublin	5	4	6	7	0	0	5	5	6	5	2
SAL_GA6301AA	08BKT035446	Dublin	5	4	6	7	0	0	5	5	6	5	12
SAL_GA6298AA	08BKT048203	Dublin	5	4	6	7	0	0	5	5	6	5	10
SAL_GA6297AA	08BKT076808	Dublin	5	4	6	7	0	0	5	5	6	5	10
SAL_GA6300AA	08BKT036322	Dublin	5	4	6	7	0	0	5	5	6	5	10
SAL_GA6287AA	11BKT008593	Dublin	5	4	6	7	0	0	5	5	6	5	11
SAL_GA6294AA	09BKT036077	Dublin	5	4	6	7	0	0	5	5	6	5	0
SAL_GA6285AA	12BKT065575	Dublin	5	4	6	7	0	0	5	5	6	5	34
SAL_GA6284AA	12BKT069449	Dublin	5	4	6	7	0	0	5	5	6	5	35
SAL_GA6296AA	09BKT035000	Dublin	5	4	6	7	0	0	5	5	6	5	0
SAL_GA6295AA	09BKT035544	Dublin	5	4	6	7	0	0	5	5	6	5	17
SAL_GA6293AA	09BKT040994	Dublin	5	4	6	7	0	0	5	5	6	5	17
SAL_GA6291AA	09BKT053882	Dublin	5	4	6	7	0	0	5	5	6	5	10
SAL_GA6289AA	09BKT077890	Dublin	5	4	6	7	0	0	5	5	6	5	0
SAL_GA6290AA	09BKT066343	Dublin	5	4	6	7	0	0	5	5	6	5	17
SAL_GA6288AA	11BKT008125	Dublin	5	4	6	7	0	0	5	5	6	5	36

SAL_GA6286AA	11BKT065504	Dublin	5	4	6	7	0	0	5	5	6	5	10
SAL_GA6283AA	13BKT078060	Dublin	5	4	6	7	0	0	5	5	6	5	37
SAL_GA6282AA	13BKT083727	Dublin	5	4	6	7	0	0	5	5	6	5	11
SAL_GA6280AA	13BKT092458	Dublin	5	4	6	7	0	0	0	0	0	0	0
SAL_GA6281AA	13BKT092147	Dublin	5	4	6	7	0	0	5	5	6	5	0
SAL_GA6279AA	14BKT005257	Dublin	5	4	6	7	0	0	5	5	6	5	38
SAL_GA6277AA	14BKT011756	Dublin	5	4	6	7	0	0	5	5	6	5	0
SAL_GA6278AA	14BKT008734	Dublin	5	4	6	7	0	0	5	5	6	5	0
SAL_GA6276AA	14BKT023034	Dublin	5	4	6	7	0	0	5	5	6	5	14
SAL_GA6274AA	14BKT027598	Dublin	5	4	6	7	0	0	5	5	6	5	17
SAL_GA6275AA	14BKT023454	Dublin	5	4	6	7	0	0	5	5	6	5	0
SAL_GA6801AA	)-bac1-11e5-aed5-3c4a9	Dublin	5	4	6	7	0	0	5	5	6	5	10
SAL_GA6755AA	)-bac1-11e5-aed5-3c4a9	Dublin	5	4	6	7	0	0	5	5	83	5	10
SAL_GA6742AA	0-bac1-11e5-aed5-3c4a9	Dublin	5	4	6	7	0	0	5	5	6	5	17
SAL_FA2057AA	05_2324	Dublin	5	4	6	7	0	0	5	5	6	5	10
SAL_FA2055AA	99_6207	Dublin	5	4	6	7	0	0	5	5	6	5	10
SAL_FA2051AA	65K_WS247	Dublin	5	4	6	7	0	0	5	5	6	5	10
SAL_FA2033AA	99_1373	Dublin	5	4	6	7	0	0	5	5	6	5	17
SAL_FA2034AA	05_5914	Dublin	5	4	6	7	0	0	5	5	6	5	2
SAL_FA2024AA	98_5329	Dublin	5	4	6	7	0	0	5	5	6	5	10
SAL_FA2043AA	02_1209	Dublin	5	4	6	7	0	0	5	5	6	5	10
SAL_FA2036AA	03_9224	Dublin	5	4	6	7	0	0	5	5	6	5	2
SAL_FA2063AA	201510290	Dublin	5	4	20	7	0	0	5	5	6	5	17
SAL_GA7467AA	PNUSAS003410	Dublin	5	4	6	7	0	0	5	5	6	5	2
SAL_FA2054AA	201208243	Dublin	5	4	6	7	0	0	5	5	6	5	11
SAL_GA7850AA	2014AM-0073	Dublin	5	4	6	7	0	0	5	5	6	5	2
SAL_GA7828AA	2014AM-0756	Dublin	5	4	6	7	0	0	5	5	6	5	2
SAL_GA7781AA	2014AM-0944	Dublin	5	4	6	7	0	0	5	5	6	5	2
SAL_GA8109AA	PNUSAS003346	Dublin	5	4	6	7	0	0	5	5	6	5	10
SAL_GA8161AA	ADRDL15-23573	Dublin	5	4	6	7	0	0	5	5	6	5	2
SAL_GA8155AA	ADRDL15-24097	Dublin	5	4	6	7	0	0	5	5	6	5	2
SAL_GA8162AA	ADRDL15-23024	Dublin	5	4	6	7	0	0	5	5	6	5	2
SAL_GA8213AA	PNUSAS003526	Dublin	5	4	6	7	0	0	5	5	6	5	2
SAL_GA8471AA	ADRDL16-2577	Dublin	5	4	6	7	0	0	5	5	6	5	0
SAL_GA8476AA	ADRDL16-1626	Dublin	5	4	6	7	0	0	5	5	6	5	2
SAL_GA8468AA	ADRDL16-1726	Dublin	5	4	6	7	0	0	0	5	6	5	2
SAL_GA8479AA	ADRDL16-2282	Dublin	5	4	6	7	0	0	5	5	6	5	2
SAL_GA8472AA	ADRDL16-3104	Dublin	5	4	6	7	0	0	5	5	6	5	2
SAL_GA8477AA	ADRDL16-955	Dublin	5	4	6	7	0	0	5	5	6	5	2
SAL_GA9182AA	CFSAN031531	Dublin	5	4	29	7	0	0	5	5	6	5	0
SAL_GA9181AA	CFSAN031532	Dublin	5	4	29	7	0	0	5	5	6	5	17
SAL_GA9277AA	PNUSAS003871	Dublin	5	4	6	7	0	0	5	5	6	5	2
SAL_GA9770AA	NYVetLIRN-8	Dublin	5	4	6	7	0	0	5	5	6	5	10
SAL_GA9775AA	NYVetLIRN-3	Dublin	5	4	6	7	0	0	5	5	6	5	0
SAL_GA9766AA	NYVetLIRN-12	Dublin	5	4	6	7	0	0	5	5	6	5	10
SAL_GA9485AA	CVM N62956	Dublin	5	4	6	7	0	0	0	0	0	0	0
SAL_HA0227AA	ADRDL-16-7090	Dublin	5	4	6	7	0	0	5	5	6	5	2
SAL_HA0810AA	CFSAN048190	Dublin	5	4	6	7	0	0	5	5	6	5	2
SAL_HA0815AA	CFSAN048195	Dublin	5	4	6	7	0	0	5	5	6	5	2
SAL_HA0820AA	CFSAN048200	Dublin	5	4	6	7	0	0	5	5	6	5	2
SAL_HA0816AA	CFSAN048196	Dublin	5	4	6	7	0	0	5	5	6	5	2
SAL_HA0809AA	CFSAN048189	Dublin	5	4	6	7	0	0	5	5	6	5	2
SAL_HA0811AA	CFSAN048191	Dublin	5	4	6	7	0	0	5	5	6	5	2
SAL_HA0818AA	CFSAN048198	Dublin	5	4	6	7	0	0	5	5	6	5	2
SAL_HA0812AA	CFSAN048192	Dublin	5	4	6	7	0	0	5	5	6	5	2
SAL_HA0813AA	CFSAN048193	Dublin	5	4	6	7	0	0	5	5	6	5	2
SAL_HA0819AA	CFSAN048199	Dublin	5	4	6	7	0	0	5	5	6	5	2
SAL_HA0600AA	2014AM-1442	Dublin	5	4	6	7	0	0	5	5	6	5	2
SAL_HA0649AA	2014AM-2142	Dublin	5	4	6	7	0	0	5	5	6	5	2
SAL_HA1045AA	CFSAN048201	Dublin	5	4	6	7	0	0	5	5	6	5	2
SAL_HA1046AA	CFSAN048202	Dublin	5	4	6	7	0	0	5	5	6	5	2
SAL_HA3080AA	PNUSAS004850	Dublin	5	4	6	7	0	0	5	5	6	5	2
SAL_HA3122AA	FSIS1608233	Dublin	5	4	6	7	0	0	5	5	6	5	2
SAL_HA3124AA	FSIS1608232	Dublin	5	4	6	7	0	0	5	5	6	5	2
SAL_HA3125AA	FSIS1608236	Dublin	5	4	6	7	0	0	0	0	0	0	0
SAL_HA3120AA	FSIS1608234	Dublin	5	4	6	7	0	0	5	5	6	5	2
SAL_HA2780AA	ADRDL-16-9870	Dublin	5	4	6	7	0	0	5	5	6	5	2
SAL_HA2782AA	ADRDL-16-9036	Dublin	5	4	6	7	0	0	5	5	6	5	2
SAL_HA2792AA	ADRDL-16-12511	Dublin	5	4	6	7	0	0	5	5	6	5	2
SAL_HA2799AA	ADRDL-16-12377	Dublin	5	4	6	7	0	0	5	5	6	5	2
SAL_HA2776AA	ADRDL-16-11965	Dublin	5	4	6	7	0	0	5	5	6	5	2
SAL_HA3299AA	S3_2007_P	Dublin	5	4	6	7	0	0	5	5	6	5	12
SAL_HA3302AA	CC3_2009_NP	Dublin	5	4	6	7	0	0	5	5	6	5	10

SAL_HA3301AA	Q2_2008_P	Dublin	5	4	6	7	0	0	5	5	6	5	10
SAL_HA3303AA	S4_2010_P	Dublin	5	4	6	7	0	0	5	5	6	5	17
SAL_HA3305AA	M2_2005_P	Dublin	5	4	6	7	0	0	5	5	6	5	2
SAL_HA3306AA	QQ1_2011_P	Dublin	5	4	6	7	0	0	5	5	6	5	10
SAL_HA3311AA	A6_2006_P	Dublin	5	48	6	7	0	0	5	5	6	5	14
SAL_HA3314AA	A5_2006_P	Dublin	5	4	6	7	0	0	5	5	6	5	14
SAL_HA3312AA	M4_2006_NP	Dublin	5	4	6	7	0	0	5	5	6	5	2
SAL_HA3313AA	WW5_2006_NP	Dublin	5	4	6	7	0	0	5	5	6	5	2
SAL_HA3310AA	WW3_2006_NP	Dublin	5	4	6	7	0	0	5	5	6	5	2
SAL_HA3309AA	Q1_2005_P	Dublin	5	4	6	7	0	0	5	5	6	5	10
SAL_HA3321AA	UU4_2012_NP	Dublin	5	4	6	7	0	0	5	5	6	5	10
SAL_HA3320AA	Z1_2012_P	Dublin	5	4	6	7	0	0	5	5	6	5	10
SAL_HA3307AA	VV4_2011_P	Dublin	5	4	6	7	0	0	5	5	6	5	10
SAL_HA3328AA	C2_2014_P	Dublin	5	4	6	7	0	0	5	5	6	5	39
SAL_HA3319AA	S1_2007_P	Dublin	5	4	6	7	0	0	5	5	6	5	12
SAL_HA3316AA	A4_2006_P	Dublin	5	4	6	7	0	0	5	5	6	5	14
SAL_HA3317AA	VV3_2007_P	Dublin	5	4	6	7	0	0	5	5	6	5	10
SAL_HA3329AA	Y3_2014_P	Dublin	5	4	6	7	0	0	5	5	6	5	10
SAL_HA3322AA	I1_2013_P	Dublin	5	4	6	7	0	0	5	5	0	5	10
SAL_HA3323AA	T1_2013_P	Dublin	5	4	6	7	0	0	5	5	6	5	2
SAL_HA3327AA	AA2_2014_P	Dublin	5	4	6	7	0	0	5	5	6	5	17
SAL_HA3326AA	UW1_2014_P	Dublin	5	4	6	7	0	0	5	5	6	5	40
SAL_HA3355AA	TW2_2014_P	Dublin	5	4	6	7	0	0	5	5	6	5	41
SAL_HA3350AA	QQ2_2014_P	Dublin	5	4	6	7	0	0	5	5	6	5	10
SAL_HA3359AA	ZW2_2015_P	Dublin	5	4	6	7	0	0	5	5	186	5	10
SAL_HA3358AA	G2_2015_P	Dublin	5	4	6	7	0	0	5	5	0	5	12
SAL_HA3351AA	VV5_2014_P	Dublin	5	4	6	7	0	0	5	5	6	5	10
SAL_HA3353AA	E1_2014_P	Dublin	5	4	6	7	0	0	5	5	6	5	42
SAL_HA3365AA	EE2_2015_P	Dublin	5	4	6	7	0	0	5	5	0	5	11
SAL_HA3364AA	F2_2015_P	Dublin	5	4	6	7	0	0	5	5	6	5	12
SAL_HA3352AA	UV2_2014_P	Dublin	5	4	6	7	0	0	5	5	6	5	17
SAL_HA3354AA	I2_2014_P	Dublin	5	4	6	7	0	0	5	5	6	5	10
SAL_HA3360AA	U2_2015_P	Dublin	5	4	6	7	0	0	5	5	6	5	2
SAL_HA3361AA	QQ3_2015_P	Dublin	5	4	6	7	0	0	5	5	6	5	10
SAL_HA3372AA	OO5_2001_P	Dublin	5	4	6	7	0	0	5	5	6	5	11
SAL_HA3373AA	OO4_2001_P	Dublin	5	4	6	7	0	0	5	5	6	5	11
SAL_HA3652AA	TT5_2004_NP	Dublin	5	4	6	7	0	0	5	5	6	5	10
SAL_HA3653AA	M1_1999_P	Dublin	5	4	6	7	0	0	5	5	6	5	2
SAL_HA3655AA	UV1_2005_P	Dublin	5	4	6	7	0	0	5	5	6	5	10
SAL_HA3654AA	WW2_2005_NP	Dublin	5	4	6	7	0	0	5	5	6	5	2
SAL_HA3657AA	X1_2004_P	Dublin	5	4	6	7	0	0	5	5	6	5	10
SAL_HA3656AA	TT6_2005_NP	Dublin	5	4	6	7	0	0	5	5	6	5	10
SAL_HA3658AA	X3_2004_P	Dublin	5	4	6	7	0	0	5	5	6	5	10
SAL_HA3660AA	X4_2004_P	Dublin	5	4	6	7	0	0	5	5	0	5	10
SAL_HA3661AA	X2_2004_P	Dublin	5	4	6	7	0	0	5	5	6	5	10
SAL_HA3662AA	RR2_2004_P	Dublin	5	4	6	7	0	0	5	5	6	5	10
SAL_HA3659AA	A2_2005_P	Dublin	5	4	6	7	0	0	5	5	6	5	14
SAL_HA3665AA	RR1_2004_P	Dublin	5	4	6	7	0	0	5	5	0	5	10
SAL_HA3666AA	X5_2004_P	Dublin	5	4	6	7	0	0	5	5	0	5	10
SAL_HA3668AA	X6_2004_P	Dublin	5	4	6	7	0	0	5	5	6	5	10
SAL_HA3667AA	X8_2005_P	Dublin	5	4	6	7	0	0	5	5	6	5	10
SAL_HA3669AA	RR3_2005_P	Dublin	5	4	6	7	0	0	5	5	0	5	14
SAL_HA3670AA	FF1_2001_P	Dublin	5	4	6	7	0	0	5	5	6	5	10
SAL_HA3671AA	X7_2005_P	Dublin	5	4	6	7	0	0	5	5	6	5	10
SAL_HA3672AA	JJ1_2001_P	Dublin	5	4	6	7	0	0	5	5	6	5	10
SAL_HA3674AA	FF2_2001_P	Dublin	5	4	6	7	0	0	5	5	6	5	10
SAL_HA3677AA	L1_2001_P	Dublin	5	4	6	7	0	0	5	5	0	5	17
SAL_HA3673AA	TU3_2010_P	Dublin	5	4	6	7	0	0	5	5	6	5	10
SAL_HA3678AA	TU2_2001_P	Dublin	5	4	6	7	0	0	5	5	83	5	10
SAL_HA3675AA	H2_2001_P	Dublin	5	4	6	7	0	0	5	5	6	5	13
SAL_HA3680AA	JJ3_2001_P	Dublin	5	4	6	7	0	0	5	5	6	5	10
SAL_HA3676AA	V1_2001_P	Dublin	5	4	6	7	0	0	5	5	6	5	10
SAL_HA3679AA	JJ2_2001_P	Dublin	5	4	6	7	0	0	5	5	6	5	10
SAL_HA3681AA	UZ2_2002_P	Dublin	5	4	6	7	0	0	5	5	6	5	11
SAL_HA3683AA	JJ4_2001_P	Dublin	5	4	6	7	0	0	5	5	0	5	10
SAL_HA3685AA	JJ6_2002_P	Dublin	5	4	6	7	0	0	5	5	6	5	10
SAL_HA3682AA	UZ1_2002_P	Dublin	5	4	6	7	0	0	5	5	6	5	17
SAL_HA3684AA	JJ5_2002_P	Dublin	5	4	6	7	0	0	5	5	6	5	10
SAL_HA3688AA	V2_2005_NP	Dublin	5	4	6	7	0	0	5	5	6	5	10
SAL_HA3686AA	FF3_2002_P	Dublin	5	4	6	7	0	0	5	5	6	5	10
SAL_HA3689AA	GG3_2001_P	Dublin	5	4	6	7	0	0	5	5	6	5	10
SAL_HA3690AA	7320161_6	Dublin	5	4	6	7	0	0	5	5	6	5	11
SAL_HA3687AA	N1_2003_NP	Dublin	5	4	6	7	0	0	5	5	6	5	43

SAL_HA3643AA	Y1_2005_P	Dublin	5	4	6	7	0	0	5	5	6	5	10
SAL_HA3692AA	NN2_2001_P	Dublin	5	4	6	7	0	0	5	5	6	5	11
SAL_HA3695AA	U1_1997_P	Dublin	5	4	6	7	0	0	5	5	6	5	11
SAL_HA3691AA	7320169_1	Dublin	5	4	6	7	0	0	5	5	6	5	11
SAL_HA3693AA	NN3_2001_P	Dublin	5	4	6	7	0	0	5	5	6	5	11
SAL_HA3694AA	W2_1998_P	Dublin	5	4	6	7	0	0	5	5	6	5	10
SAL_HA3698AA	GG1_1998_P	Dublin	5	4	6	7	0	0	5	5	6	5	10
SAL_HA3697AA	PP2_2001_P	Dublin	5	4	6	7	0	0	5	5	6	5	2
SAL_HA3699AA	W1_1998_P	Dublin	5	4	6	7	0	0	5	5	6	5	10
SAL_HA3700AA	UU2_2001_NP	Dublin	5	4	6	7	0	0	5	5	6	5	10
SAL_HA3701AA	PP4_2001_P	Dublin	5	4	6	7	0	0	5	5	6	5	2
SAL_HA3702AA	GG5_2001_P	Dublin	5	4	6	7	0	0	5	5	6	5	10
SAL_HA3703AA	R2_2001_P	Dublin	5	4	6	7	0	0	5	5	6	5	10
SAL_HA3705AA	SS4_2000_NP	Dublin	5	4	6	7	0	0	5	5	6	5	10
SAL_HA3704AA	MM4_2000_P	Dublin	5	4	6	7	0	0	5	5	6	5	10
SAL_HA3709AA	PP3_2001_P	Dublin	5	4	6	7	0	0	5	5	6	5	2
SAL_HA3706AA	SS2_2000_NP	Dublin	5	4	6	7	0	0	5	5	6	5	10
SAL_HA3708AA	SS1_2000_NP	Dublin	5	4	6	7	0	0	5	5	6	5	10
SAL_HA3710AA	R1_1999_P	Dublin	5	4	6	7	0	0	5	5	6	5	10
SAL_HA3707AA	SS3_2000_NP	Dublin	5	4	6	7	0	0	5	5	6	5	10
SAL_HA3712AA	MM3_2000_P	Dublin	5	4	6	7	0	0	5	5	6	5	10
SAL_HA3711AA	VV1_2001_P	Dublin	5	4	6	7	0	0	5	5	0	5	10
SAL_HA3715AA	TU1_2001_P	Dublin	5	4	6	7	0	0	5	5	83	5	10
SAL_HA3714AA	II1_1996_P	Dublin	5	4	6	7	0	0	5	5	6	5	11
SAL_HA3713AA	MM2_2000_P	Dublin	5	4	6	7	0	0	5	5	6	5	10
SAL_HA3717AA	S2_2007_P	Dublin	5	4	6	7	0	0	5	5	6	5	12
SAL_HA3716AA	A3_2005_P	Dublin	5	4	6	7	0	0	5	5	6	5	14
SAL_HA3720AA	HH1_1999_P	Dublin	5	4	6	7	0	0	5	5	6	5	10
SAL_HA3719AA	K2_1999_NP	Dublin	5	4	6	7	0	0	5	5	6	5	10
SAL_HA3721AA	VZ1_1999_P	Dublin	5	4	6	7	0	0	5	5	6	5	10
SAL_HA3718AA	DD2_1999_P	Dublin	5	4	6	7	0	0	5	5	6	5	10
SAL_HA3723AA	H1_2000_P	Dublin	5	4	6	7	0	0	5	5	6	5	40
SAL_HA3722AA	9712575_1	Dublin	5	4	6	7	0	0	5	5	6	5	10
SAL_HA3724AA	ZZ1_2000_P	Dublin	5	4	6	7	0	0	5	5	6	5	10
SAL_HA3728AA	KK6_2001_NP	Dublin	5	4	6	7	0	0	5	5	6	5	10
SAL_HA3726AA	LL1_2000_P	Dublin	5	4	6	7	0	0	5	5	0	5	11
SAL_HA3731AA	KK7_2001_NP	Dublin	5	4	6	7	0	0	5	5	6	5	10
SAL_HA3727AA	O1_2000_P	Dublin	5	4	6	7	0	0	5	5	6	5	10
SAL_HA3730AA	ZZ2_2001_NP	Dublin	5	4	6	7	0	0	5	5	6	5	10
SAL_HA3729AA	LL2_2000_P	Dublin	5	4	6	7	0	0	5	5	6	5	11
SAL_HA3733AA	LL5_2001_P	Dublin	5	4	6	7	0	0	5	5	6	5	11
SAL_HA3732AA	KK3_2000_NP	Dublin	5	4	6	7	0	0	5	5	6	5	10
SAL_HA3734AA	KK4_2001_NP	Dublin	5	4	6	7	0	0	5	5	0	5	10
SAL_HA3735AA	KK2_2000_NP	Dublin	5	4	6	7	0	0	5	5	6	5	10
SAL_HA3739AA	PP5_2001_P	Dublin	5	4	6	7	0	0	5	5	6	5	2
SAL_HA3737AA	LL3_2000_P	Dublin	5	4	6	7	0	0	5	5	6	5	11
SAL_HA3736AA	KK5_2001_NP	Dublin	5	4	6	7	0	0	5	5	6	5	10
SAL_HA3738AA	LL4_2001_P	Dublin	5	4	6	7	0	0	5	5	6	5	11
SAL_HA3740AA	UU3_2001_NP	Dublin	5	4	6	7	0	0	5	5	6	5	10
SAL_HA3744AA	PP6_2001_P	Dublin	5	4	6	7	0	0	5	5	6	5	2
SAL_HA3746AA	OO6_2001_P	Dublin	5	4	6	7	0	0	5	5	6	5	11
SAL_HA3741AA	CC1_2001_P	Dublin	5	4	6	7	0	0	5	5	6	137	10
SAL_HA3743AA	NN6_2001_P	Dublin	5	4	6	7	0	0	5	5	6	5	11
SAL_HA3745AA	SS7_2001_NP	Dublin	5	4	6	7	0	0	5	5	0	5	10
SAL_HA3747AA	NN7_2001_P	Dublin	5	4	6	7	0	0	5	5	6	5	11
SAL_HA3748AA	PP7_2001_P	Dublin	5	4	6	7	0	0	5	5	6	5	2
SAL_HA3749AA	OO7_2001_P	Dublin	5	4	6	7	0	0	5	5	6	5	11
SAL_HA3751AA	SS8_2001_NP	Dublin	5	4	6	7	0	0	5	5	0	5	10
SAL_HA3753AA	NN8_2002_P	Dublin	5	4	6	7	0	0	5	5	6	5	11
SAL_HA3754AA	OO8_2002_P	Dublin	5	4	6	7	0	0	5	5	6	5	10
SAL_HA3752AA	SS9_2002_NP	Dublin	5	4	6	7	0	0	5	5	0	5	10
SAL_HA3758AA	VV2_2002_P	Dublin	5	4	6	7	0	0	5	5	6	5	10
SAL_HA3757AA	NN9_2002_P	Dublin	5	4	6	7	0	0	5	5	6	5	11
SAL_HA3755AA	PP8_2002_P	Dublin	5	4	6	7	0	0	5	5	6	5	2
SAL_HA3756AA	PP9_2002_P	Dublin	5	4	6	7	0	0	5	5	6	5	2
SAL_HA3761AA	TT2_2002_P	Dublin	5	4	6	7	0	0	5	5	6	5	10
SAL_HA3760AA	TT1_2002_P	Dublin	5	4	6	7	0	0	5	5	6	5	10
SAL_HA3759AA	F1_2004_P	Dublin	5	4	6	7	0	0	5	5	6	5	29
SAL_HA3762AA	BB4_2003_NP	Dublin	5	4	6	7	0	0	5	5	6	5	10
SAL_HA3763AA	WW1_2004_P	Dublin	5	4	6	7	0	0	5	5	6	5	2
SAL_HA3766AA	P1_1999_NP	Dublin	5	4	6	7	0	0	5	5	6	5	10
SAL_HA3765AA	BB2_2000_P	Dublin	5	4	6	7	0	0	5	5	6	5	10
SAL_HA3764AA	TT3_2002_P	Dublin	5	4	6	7	0	0	5	5	6	5	10

SAL_HA3767AA	BB1_1999_P	Dublin	5	4	6	7	0	0	5	5	6	5	10
SAL_HA3768AA	TW1_1999_P	Dublin	5	4	6	7	0	0	5	5	6	5	12
SAL_HA3772AA	K3	Dublin	5	4	6	7	0	0	5	5	6	5	10
SAL_HA3774AA	K10	Dublin	5	4	6	7	0	0	5	5	6	5	10
SAL_HA3773AA	K4	Dublin	5	4	6	8	0	0	0	0	0	0	0
SAL_HA3771AA	K2	Dublin	5	4	6	7	0	0	5	5	6	5	10
SAL_HA3769AA	MM1_1999_P	Dublin	5	4	6	7	0	0	5	5	6	5	10
SAL_HA3770AA	K1	Dublin	5	4	6	7	0	0	5	5	6	5	10
SAL_HA3775AA	K7	Dublin	5	4	6	7	0	0	5	5	6	5	17
SAL_HA3776AA	K6	Dublin	5	4	6	7	0	0	5	5	6	5	10
SAL_HA3778AA	K8	Dublin	5	4	6	7	0	0	5	5	6	5	10
SAL_HA3777AA	K5	Dublin	5	4	6	7	0	0	5	5	0	5	10
SAL_HA3780AA	NN5_2001_P	Dublin	5	4	6	7	0	0	5	5	6	5	11
SAL_HA3779AA	K9	Dublin	5	4	6	7	0	0	5	5	6	5	10
SAL_HA2142AA	S01833-03	Dublin	5	4	6	7	0	0	5	5	6	5	18
SAL_HA3645AA	OO1_1996_P	Dublin	5	4	6	7	0	0	5	5	6	5	11
SAL_HA3644AA	B1_2003_P	Dublin	5	4	6	7	0	0	5	5	6	5	39
SAL_HA3369AA	TW3_2015_P	Dublin	5	4	6	7	0	0	5	5	6	5	39
SAL_HA3648AA	W3_2003_P	Dublin	5	4	6	7	0	0	5	5	6	5	10
SAL_HA3371AA	GG2_2001_P	Dublin	5	4	6	7	0	0	5	5	6	5	10
SAL_HA3370AA	Z2_2016_P	Dublin	5	4	6	7	0	0	5	5	6	5	10
SAL_HA3375AA	GG4_2001_P	Dublin	5	4	6	7	0	0	5	5	6	5	10
SAL_HA3374AA	SS6_2001_NP	Dublin	5	4	6	7	0	0	5	5	6	5	10
SAL_HA3376AA	A1_2001_P	Dublin	5	4	6	7	0	0	5	5	6	5	29
SAL_HA3377AA	NN1_2001_P	Dublin	5	4	6	7	0	0	5	5	6	5	11
SAL_HA3378AA	AA1_1998_P	Dublin	5	4	6	7	0	0	5	5	6	5	10
SAL_HA3379AA	SS5_2001_NP	Dublin	5	4	6	7	0	0	5	5	6	5	10
SAL_HA3403AA	PP1_1996_P	Dublin	5	4	6	7	0	0	5	5	6	5	11
SAL_HA3380AA	OO3_2000_P	Dublin	5	4	6	7	0	0	5	5	6	5	11
SAL_HA3405AA	C1_1997_P	Dublin	5	4	6	7	0	0	5	5	6	5	17
SAL_HA3404AA	OO2_2000_P	Dublin	5	4	6	7	0	0	5	5	6	5	11
SAL_HA3408AA	BB3_2000_P	Dublin	5	4	6	7	0	0	5	5	6	5	10
SAL_HA3406AA	NN4_2001_P	Dublin	5	4	6	7	0	0	5	5	6	5	11
SAL_HA3410AA	M3_2005_P	Dublin	5	4	6	7	0	0	5	5	6	5	2
SAL_HA3409AA	UU1_2001_NP	Dublin	5	4	6	7	0	0	5	5	6	5	10
SAL_HA3412AA	VZ2_2006_NP	Dublin	5	4	6	7	0	0	5	5	6	5	10
SAL_HA3411AA	O2_2006_P	Dublin	5	4	6	7	0	0	5	5	6	5	10
SAL_HA3413AA	HH2_2005_NP	Dublin	5	4	6	7	0	0	5	5	6	5	10
SAL_HA3422AA	B2_2014_P	Dublin	5	4	6	7	0	0	5	5	6	5	12
SAL_HA3421AA	O3_2006_P	Dublin	5	4	6	7	0	0	5	5	6	5	10
SAL_HA3420AA	J1_2005_P	Dublin	5	4	6	7	0	0	5	5	6	5	13
SAL_HA3424AA	L2_2007_P	Dublin	5	4	6	7	0	0	5	5	6	5	17
SAL_HA3427AA	K1_1997_NP	Dublin	5	4	6	7	0	0	5	5	6	5	10
SAL_HA3426AA	H3_2007_NP	Dublin	5	4	6	7	0	0	5	5	6	5	29
SAL_HA3425AA	II2_1997_P	Dublin	5	4	6	7	0	0	5	5	6	5	10
SAL_HA3428AA	DD1_1997_P	Dublin	5	4	6	7	0	0	5	5	6	5	10
SAL_HA3646AA	KK1_2000_NP	Dublin	5	4	6	7	0	0	5	5	6	5	10
SAL_HA3640AA	D1_2003_P	Dublin	5	4	6	7	0	0	5	5	0	5	10
SAL_HA3641AA	WW4_2003_NP	Dublin	5	4	6	7	0	0	5	5	6	5	2
SAL_HA3649AA	TT4_2004_NP	Dublin	5	4	6	7	0	0	5	5	6	5	10
SAL_HA3651AA	CC2_2005_P	Dublin	5	4	6	7	0	0	5	5	6	137	10
SAL_HA3650AA	D2_2005_P	Dublin	5	4	6	7	0	0	5	5	6	5	29
SAL_HA3642AA	G1_2004_P	Dublin	5	4	6	7	0	0	5	5	83	5	29
SAL_HA3356AA	W4_2015_P	Dublin	5	4	6	7	0	0	5	5	6	5	14
SAL_HA3357AA	T2_2015_P	Dublin	5	4	6	7	0	0	5	5	6	5	2
SAL_HA3366AA	EE1_2015_P	Dublin	5	4	6	7	0	0	5	5	0	5	17
SAL_HA3368AA	R3_2015_P	Dublin	5	4	6	7	0	0	5	5	6	5	29
SAL_HA3367AA	I3_2015_P	Dublin	5	4	6	7	0	0	5	5	6	5	10
SAL_HA1933AA	S01421-97	Dublin	5	4	6	7	0	0	5	5	6	5	10
SAL_HA1948AA	S05576-96	Dublin	5	4	6	7	0	0	5	5	6	5	18
SAL_HA1946AA	S07426-96	Dublin	5	4	6	7	0	0	5	5	6	5	1
SAL_HA1951AA	S10569-94	Dublin	5	4	6	7	0	0	5	5	6	5	1
SAL_HA1952AA	S10260-94	Dublin	5	4	6	7	0	0	5	5	6	5	12
SAL_HA1917AA	S03198-12	Dublin	5	4	6	7	0	0	5	5	6	5	10
SAL_HA1918AA	S00492-10	Dublin	5	4	6	7	0	0	5	5	6	5	18
SAL_HA1879AA	L01197-10	Dublin	5	4	6	7	0	0	5	5	6	5	18
SAL_HA4276AA	CFSAN048327	Dublin	5	4	6	7	0	0	5	5	6	5	0
SAL_HA4728AA	FSIS1608443	Dublin	5	4	6	7	0	0	5	5	6	5	2
SAL_HA3325AA	ZW1_2014_P	Dublin	5	4	6	7	0	0	5	5	186	5	10
SAL_HA3362AA	D3_2015_P	Dublin	5	4	6	7	0	0	5	5	6	5	12
SAL_HA3363AA	P2_2015_P	Dublin	5	4	6	7	0	0	5	5	83	5	14
SAL_HA4756AA	10-0294	Dublin	0	0	0	0	0	0	0	0	0	0	0
SAL_HA4758AA	10-1049	Dublin	0	0	0	0	0	0	0	0	0	0	0

SAL_HA4920AA	2379465	Dublin	0	0	0	0	0	0	0	0	0	0	0
SAL_HA5120AA	L00899-14	Dublin	5	4	6	7	0	0	5	5	6	5	18
SAL_HA5071AA	L00542-15	Dublin	5	4	6	7	0	0	5	5	6	5	11
SAL_HA5081AA	S05420-14	Dublin	5	4	6	7	0	0	5	5	6	5	18
SAL_HA5370AA	CFSAN048502	Dublin	5	4	6	7	0	0	5	5	6	5	2
SAL_HA5348AA	CFSAN048508	Dublin	5	4	6	7	0	0	5	5	6	5	2
SAL_HA5351AA	CFSAN048510	Dublin	5	4	6	7	0	0	5	5	6	5	2
SAL_HA5350AA	CFSAN048507	Dublin	5	4	6	7	0	0	5	5	6	5	2
SAL_HA5355AA	CFSAN048512	Dublin	5	4	6	7	0	0	5	5	6	5	2
SAL_HA5358AA	CFSAN056359	Dublin	5	4	6	7	0	0	5	5	6	5	2
SAL_HA5363AA	CFSAN056366	Dublin	5	4	6	7	0	0	5	5	6	5	2
SAL_HA5346AA	CFSAN048509	Dublin	5	4	6	7	0	0	5	5	6	5	2
SAL_HA5352AA	CFSAN048511	Dublin	5	4	6	7	0	0	5	5	6	5	2
SAL_HA5347AA	CFSAN048505	Dublin	5	4	6	7	0	0	5	5	6	5	2
SAL_HA5249AA	PNUSAS005012	Dublin	5	4	6	7	0	0	5	5	6	5	2
SAL_HA5345AA	CFSAN048504	Dublin	5	4	6	7	0	0	5	5	6	5	2
SAL_HA5349AA	CFSAN048506	Dublin	5	4	6	7	0	0	5	5	6	5	2
SAL_HA5481AA	CFSAN056376	Dublin	5	4	6	7	0	0	5	5	83	5	2
SAL_HA5482AA	CFSAN056377	Dublin	5	4	6	7	0	0	5	5	83	5	2
SAL_HA5489AA	CFSAN056380	Dublin	5	4	6	7	0	0	5	5	6	5	2
SAL_HA5476AA	CFSAN056374	Dublin	5	4	6	7	0	0	5	5	6	5	2
SAL_HA5474AA	CFSAN056367	Dublin	5	4	6	7	0	0	5	5	6	5	2
SAL_HA5477AA	CFSAN056372	Dublin	5	4	6	7	0	0	5	5	6	5	2
SAL_HA5479AA	CFSAN056370	Dublin	5	4	6	7	0	0	5	5	6	5	2
SAL_HA5478AA	CFSAN056371	Dublin	5	4	6	7	0	0	5	5	6	5	2
SAL_HA5480AA	CFSAN056373	Dublin	5	4	6	7	0	0	5	5	6	5	2
SAL_HA5483AA	CFSAN056381	Dublin	5	4	6	7	0	0	5	5	6	5	2
SAL_HA5471AA	CFSAN048503	Dublin	5	4	6	7	0	0	5	5	6	5	2
SAL_HA5475AA	CFSAN056375	Dublin	5	4	6	7	0	0	5	5	6	5	2
SAL_HA5473AA	CFSAN056369	Dublin	5	4	6	7	0	0	5	5	6	5	2
SAL_HA5515AA	CFSAN056857	Dublin	5	4	6	7	0	0	5	5	6	5	2
SAL_HA2712AA	FSIS1608292	Dublin	5	4	6	7	0	0	5	5	6	5	2
SAL_HA0024AA	ADRDL 16-5099	Dublin	5	4	6	7	0	0	5	5	6	5	2
SAL_HA0693AA	E2016015302	Dublin	5	4	6	7	0	0	5	5	6	5	2
SAL_HA5640AA	S02278-15	Dublin	5	4	6	7	0	0	5	5	6	5	11
SAL_HA5643AA	S02004-15	Dublin	5	4	6	7	0	0	5	5	83	5	18
SAL_HA5657AA	L03354-15	Dublin	5	4	6	7	0	0	5	5	6	5	11
SAL_HA5637AA	S02835-15	Dublin	5	4	6	7	0	0	5	5	6	5	18
SAL_HA5635AA	S02981-15	Dublin	5	4	6	7	0	0	5	5	6	5	18
SAL_HA6445AA	FSIS1608938	Dublin	5	4	6	7	0	0	5	5	6	5	2
SAL_HA5672AA	L00481-14	Dublin	5	4	6	7	0	0	5	5	6	5	18
SAL_HA5663AA	L02520-15	Dublin	5	4	6	7	0	0	5	5	6	5	18
SAL_HA4191AA	CFSAN048326	Dublin	5	4	6	7	0	0	5	5	6	5	0
SAL_HA4273AA	CFSAN048328	Dublin	5	4	6	7	0	0	5	5	6	5	0
SAL_HA5362AA	CFSAN056363	Dublin	5	4	6	7	0	0	5	5	6	5	2
SAL_HA6107AA	FSIS1608681	Dublin	5	4	6	7	0	0	5	5	6	5	2
SAL_HA6096AA	CFSAN056851	Dublin	5	4	6	7	0	0	5	5	6	5	2
SAL_HA6882AA	PNUSAS006050	Dublin	5	4	6	7	0	0	5	5	6	5	2
SAL_HA7717AA	FSIS1609229	Dublin	5	4	6	7	0	0	5	5	6	5	2
SAL_HA7705AA	FSIS1609228	Dublin	5	4	6	7	0	0	5	5	6	5	2
SAL_HA8627AA	PNUSAS005187	Dublin	5	4	6	7	0	0	5	5	6	5	2
SAL_HA8424AA	FSIS1609227	Dublin	5	4	6	7	0	0	5	5	6	5	2
SAL_HA8300AA	FSIS1609248	Dublin	5	4	6	7	0	0	5	5	6	5	2
SAL_HA8288AA	TX-ACF1602215	Dublin	5	4	6	7	0	0	5	5	6	5	2
SAL_HA8430AA	FSIS1609225	Dublin	5	4	6	7	0	0	5	5	6	5	2
SAL_HA8429AA	FSIS1609226	Dublin	5	4	6	7	0	0	5	5	6	5	2
SAL_HA8705AA	OH-16-16860	Dublin	5	4	6	7	0	0	5	5	6	5	2
SAL_HA8909AA	NCTC12709	Dublin	5	4	6	7	0	0	5	5	0	5	10
SAL_HA8953AA	3246	Dublin	5	4	6	7	0	0	5	5	6	5	10
SAL_HA9112AA	CFSAN058111	Dublin	5	4	6	7	0	0	5	5	6	5	2
SAL_HA9336AA	PNUSAS006870	Dublin	5	4	6	7	0	0	5	5	6	5	2
SAL_HA9705AA	CFSAN058851	Dublin	5	4	6	7	0	0	5	5	6	5	2
SAL_HA9704AA	CFSAN058853	Dublin	5	4	6	7	0	0	5	5	6	5	2
SAL_HA9692AA	CFSAN058852	Dublin	5	4	6	7	0	0	5	5	6	5	2
SAL_HA9695AA	CFSAN058850	Dublin	5	4	6	7	0	0	5	5	6	5	2
SAL_IA0074AA	0-3918-11e6-bcc0-3c4a9	Dublin	0	0	0	0	0	0	0	0	0	0	0
SAL_IA0150AA	CFSAN058886	Dublin	5	4	6	7	0	0	5	5	6	5	2
SAL_IA0147AA	CFSAN058887	Dublin	5	4	6	7	0	0	5	5	6	5	2
SAL_IA0148AA	CFSAN058889	Dublin	5	4	6	7	0	0	5	5	6	5	2
SAL_IA0149AA	CFSAN058888	Dublin	5	4	6	7	0	0	5	5	6	5	2
SAL_IA0353AA	CFSAN058883	Dublin	5	4	6	7	0	0	5	5	6	5	2
SAL_IA0354AA	CFSAN058881	Dublin	5	4	6	7	0	0	5	5	6	5	2
SAL_IA0357AA	CFSAN058884	Dublin	5	4	6	7	0	0	5	5	6	5	2

SAL_IA0358AA	CFSAN058880	Dublin	5	4	6	7	0	0	5	5	6	5	2
SAL_IA0359AA	CFSAN058885	Dublin	5	4	6	7	0	0	5	5	6	5	2
SAL_IA0351AA	CFSAN058882	Dublin	5	4	6	7	0	0	5	5	6	5	2
SAL_IA0342AA	CFSAN058868	Dublin	5	4	6	7	0	0	5	5	6	5	2
SAL_IA0343AA	CFSAN058866	Dublin	5	4	6	7	0	0	5	5	6	5	2
SAL_IA0339AA	CFSAN058867	Dublin	5	4	6	7	0	0	5	5	6	5	2
SAL_IA0340AA	CFSAN058869	Dublin	5	4	6	7	0	0	5	5	6	5	2
SAL_IA0335AA	CFSAN058863	Dublin	5	4	6	7	0	0	5	5	6	5	2
SAL_IA0314AA	CFSAN058890	Dublin	5	4	6	7	0	0	5	5	6	5	2
SAL_IA0313AA	CFSAN058894	Dublin	5	4	6	7	0	0	5	5	6	5	2
SAL_IA0315AA	CFSAN058893	Dublin	5	4	6	7	0	0	5	5	6	5	2
SAL_IA0317AA	CFSAN058891	Dublin	5	4	6	7	0	0	5	5	6	5	2
SAL_IA0321AA	CFSAN058892	Dublin	5	4	6	7	0	0	5	5	6	5	2
SAL_IA0324AA	CFSAN058896	Dublin	5	4	6	7	0	0	5	5	6	5	2
SAL_IA0323AA	CFSAN058895	Dublin	5	4	6	7	0	0	5	5	6	5	2
SAL_IA0194AA	PNUSAS007087	Dublin	5	4	6	7	0	0	5	5	6	5	2
SAL_IA0189AA	PNUSAS007086	Dublin	5	4	6	7	0	0	5	5	6	5	2
SAL_IA0460AA	PNUSAS007082	Dublin	5	4	6	7	0	0	5	5	6	5	2
SAL_IA0446AA	PNUSAS007089	Dublin	5	4	6	7	0	0	5	5	6	5	2
SAL_IA1889AA	S/20170007	Dublin	5	4	6	7	0	0	5	5	6	5	18
SAL_IA1912AA	PNUSAS006787	Dublin	5	4	6	7	0	0	5	5	6	5	2
SAL_IA2007AA	FSIS1709816	Dublin	5	4	6	7	0	0	5	5	6	5	2
SAL_IA2307AA	CFSAN059178	Dublin	5	4	6	7	0	0	5	5	6	5	2
SAL_IA2428AA	2014AM-2597	Dublin	5	4	6	7	0	0	5	5	6	5	2
SAL_IA2420AA	2014AM-1538	Dublin	5	4	6	7	0	0	5	5	6	5	2
SAL_IA3144AA	188360	Dublin	5	4	6	7	0	0	5	5	6	5	18
SAL_IA2664AA	CFSAN059210	Dublin	5	4	6	7	0	0	5	5	6	5	2
SAL_IA2673AA	CFSAN059219	Dublin	5	4	6	7	0	0	5	5	6	5	2
SAL_IA4675AA	FSIS1710011	Dublin	5	4	6	7	0	0	0	0	0	0	0
SAL_IA4620AA	PNUSAS007479	Dublin	5	4	6	7	0	0	5	5	6	5	2
SAL_IA4966AA	CFSAN059287	Dublin	5	4	6	7	0	0	5	5	6	5	2
SAL_IA4965AA	CFSAN059288	Dublin	5	4	6	7	0	0	5	5	6	5	2
SAL_IA4926AA	CFSAN059739	Dublin	5	4	6	7	0	0	5	5	6	5	2
SAL_IA4938AA	CFSAN059740	Dublin	5	4	6	7	0	0	5	5	6	5	2
SAL_IA4935AA	CFSAN059738	Dublin	5	4	6	7	0	0	5	5	6	5	2
SAL_IA4987AA	S/20170022	Dublin	5	4	6	7	0	0	5	5	6	5	18
SAL_IA4989AA	S/20170023	Dublin	5	4	6	7	0	0	5	5	6	5	18
SAL_IA5179AA	CFSAN058123	Dublin	5	4	6	7	0	0	5	5	6	5	2
SAL_IA5122AA	CFSAN058128	Dublin	5	4	6	7	0	0	5	5	6	5	2
SAL_IA4930AA	CFSAN059737	Dublin	5	4	6	7	0	0	5	5	6	5	2
SAL_IA4928AA	CFSAN059741	Dublin	5	4	6	7	0	0	5	5	6	5	2
SAL_IA4939AA	CFSAN059742	Dublin	5	4	6	7	0	0	5	5	6	5	2
SAL_IA5367AA	FSIS1710010	Dublin	5	4	6	7	0	0	0	0	0	0	0
SAL_IA6251AA	PNUSAS007815	Dublin	5	4	6	7	0	0	5	5	6	5	10
SAL_IA6220AA	PNUSAS007574	Dublin	5	4	6	7	0	0	5	5	6	5	2
SAL_IA6115AA	PNUSAS007907	Dublin	5	4	6	7	0	0	5	5	6	5	2
SAL_IA6224AA	PNUSAS008284	Dublin	5	4	6	7	0	0	5	5	6	5	2
SAL_IA6036AA	PNUSAS007306	Dublin	5	4	6	7	0	0	5	5	0	5	2
SAL_IA6042AA	PNUSAS007749	Dublin	5	4	6	7	0	0	5	5	6	5	2
SAL_IA6038AA	PNUSAS007463	Dublin	5	4	6	7	0	0	5	5	6	5	2
SAL_IA5771AA	PNUSAS007489	Dublin	5	4	6	7	0	0	5	5	0	5	2
SAL_IA6443AA	PNUSAS008543	Dublin	5	4	6	7	0	0	5	5	6	5	2
SAL_IA5424AA	PNUSAS008462	Dublin	5	4	6	7	0	0	5	5	6	5	2
SAL_IA6542AA	PNUSAS008733	Dublin	5	4	6	7	0	0	5	5	6	5	2
SAL_IA6509AA	PNUSAS008538	Dublin	5	4	6	7	0	0	5	5	6	5	2
SAL_IA6588AA	FSIS1710686	Dublin	5	4	6	7	0	0	5	5	6	5	2
SAL_IA6597AA	FSIS1710688	Dublin	5	4	6	7	0	0	5	5	6	5	0
SAL_IA6614AA	FSIS1710687	Dublin	5	4	6	7	0	0	5	5	6	5	2
SAL_IA6657AA	PNUSAS007820	Dublin	5	4	6	7	0	0	5	5	6	5	2
SAL_IA6710AA	MS150315	Dublin	5	4	6	7	0	0	5	5	6	5	17
SAL_IA7241AA	PNUSAS008387	Dublin	5	4	6	7	0	0	5	5	6	5	2
SAL_IA7269AA	S/20170039	Dublin	5	4	6	7	0	0	5	5	6	0	18
SAL_IA7278AA	S/20170042	Dublin	5	4	6	7	0	0	5	5	6	5	18
SAL_IA7251AA	PNUSAS008279	Dublin	5	4	6	7	0	0	5	5	6	5	2
SAL_IA7323AA	PNUSAS008916	Dublin	5	4	6	7	0	0	5	5	6	5	2
SAL_IA7354AA	S/20170052	Dublin	5	4	6	7	0	0	5	5	6	5	18
SAL_IA7396AA	PNUSAS008670	Dublin	5	4	6	7	0	0	5	5	6	5	2
SAL_IA7423AA	CFSAN060232	Dublin	5	4	6	7	0	0	5	5	6	5	2
SAL_IA7604AA	PNUSAS009112	Dublin	5	4	6	7	0	0	5	5	6	5	2
SAL_IA7666AA	FSIS1710596	Dublin	5	4	6	7	0	0	5	5	6	5	2
SAL_IA8300AA	PNUSAS008768	Dublin	5	4	6	7	0	0	5	5	6	5	0
SAL_IA8389AA	PNUSAS008632	Dublin	5	4	6	7	0	0	5	5	6	5	2
SAL_IA8429AA	PNUSAS009160	Dublin	5	4	6	7	0	0	5	5	6	5	2

SAL_IA8444AA	17007603	Dublin	5	4	6	7	0	0	5	5	6	5	10
SAL_IA8848AA	PNUSAS008384	Dublin	5	4	6	7	0	0	5	5	6	5	2
SAL_IA8860AA	PNUSAS009163	Dublin	5	4	6	7	0	0	5	5	6	5	2
SAL_IA8833AA	PNUSAS008406	Dublin	5	4	6	7	0	0	5	5	6	5	2
SAL_IA8962AA	ADRDL-900	Dublin	5	4	6	7	0	0	5	5	6	5	2
SAL_IA8916AA	FSIS1710681	Dublin	5	4	6	7	0	0	0	0	0	0	0
SAL_IA9090AA	S/20170082	Dublin	5	4	6	7	0	0	5	5	6	5	18
SAL_IA9098AA	S/20170091	Dublin	5	4	6	7	0	0	5	5	6	5	18
SAL_IA9555AA	PNUSAS009162	Dublin	5	4	6	7	0	0	5	5	6	5	2
SAL_IA9861AA	PNUSAS009539	Dublin	5	4	6	7	0	0	5	5	0	5	2
SAL_IA9827AA	PNUSAS009604	Dublin	5	4	6	7	0	0	5	5	83	5	2
SAL_IA9721AA	FSIS1710957	Dublin	5	4	6	7	0	0	5	5	6	5	2
SAL_JA0073AA	S/20170133	Dublin	5	4	6	7	0	0	5	5	6	5	18
SAL_JA0086AA	PNUSAS009632	Dublin	5	4	6	7	0	0	5	5	6	5	2
SAL_JA0368AA	PNUSAS009755	Dublin	5	4	6	7	0	0	5	5	6	5	2
SAL_JA0787AA	onella enterica subsp. en	Dublin	5	4	6	7	0	0	5	5	6	5	10
SAL_JA0789AA	onella enterica subsp. en	Dublin	5	4	6	7	0	0	5	5	83	5	10
SAL_JA0803AA	onella enterica subsp. en	Dublin	5	4	6	7	0	0	5	5	6	5	10
SAL_JA0804AA	onella enterica subsp. en	Dublin	5	4	6	7	0	0	5	5	6	5	10
SAL_JA0736AA	onella enterica subsp. en	Dublin	5	4	6	7	0	0	5	5	83	5	10
SAL_JA0743AA	onella enterica subsp. en	Dublin	5	4	6	7	0	0	5	5	6	5	17
SAL_JA0767AA	onella enterica subsp. en	Dublin	5	4	6	7	0	0	5	5	6	5	10
SAL_JA0793AA	onella enterica subsp. en	Dublin	5	4	6	7	0	0	5	5	6	5	10
SAL_JA0796AA	onella enterica subsp. en	Dublin	5	4	6	7	0	0	5	5	6	5	0
SAL_JA0797AA	onella enterica subsp. en	Dublin	5	4	6	7	0	0	5	5	0	5	12
SAL_JA0799AA	onella enterica subsp. en	Dublin	5	4	6	7	0	0	5	5	98	5	12
SAL_JA0805AA	onella enterica subsp. en	Dublin	5	4	6	7	0	0	5	5	6	5	10
SAL_JA0809AA	onella enterica subsp. en	Dublin	5	4	6	7	0	0	5	5	6	5	10
SAL_JA0733AA	onella enterica subsp. en	Dublin	5	4	6	7	0	0	5	5	83	5	12
SAL_JA0739AA	onella enterica subsp. en	Dublin	5	4	6	7	0	0	5	5	83	5	17
SAL_JA0794AA	onella enterica subsp. en	Dublin	5	4	6	7	0	0	5	5	6	5	0
SAL_JA1837AA	8-31-20170184	Dublin	5	4	6	7	0	0	5	5	6	5	18
SAL_JA1839AA	8-13-20170151	Dublin	5	4	6	7	0	0	5	5	6	5	18
SAL_JA0606AA	CFSAN025433	Dublin	5	4	6	7	0	0	5	5	6	5	18
SAL_JA0238AA	PNUSAS010074	Dublin	5	4	6	7	0	0	5	5	6	5	2
SAL_JA1781AA	PNUSAS010804	Dublin	5	4	6	7	0	0	5	5	6	5	2
SAL_JA2069AA	FSIS1700034	Dublin	5	4	6	7	0	0	0	0	0	0	0
SAL_JA2328AA	CFSAN062969	Dublin	5	4	6	7	0	0	5	5	6	5	2
SAL_JA2481AA	PNUSAS010834	Dublin	5	4	6	7	0	0	5	5	6	5	2
SAL_JA2577AA	PNUSAS009176	Dublin	5	4	6	7	0	0	5	5	6	5	2
SAL_JA2539AA	PNUSAS009438	Dublin	5	4	6	7	0	0	5	5	6	5	2
SAL_JA3258AA	PNUSAS011770	Dublin	5	4	6	7	0	0	5	5	6	5	2
SAL_JA3630AA	FSIS1700704	Dublin	5	4	6	126	0	0	5	5	6	5	2
SAL_JA3640AA	FSIS1700542	Dublin	5	4	6	7	0	0	5	5	6	5	2
SAL_JA3531AA	PNUSAS012401	Dublin	5	4	6	7	0	0	5	5	6	5	2
SAL_JA5311AA	PNUSAS012140	Dublin	5	4	6	7	0	0	5	5	6	5	2
SAL_JA3998AA	PNUSAS012141	Dublin	5	4	6	7	0	0	5	5	6	5	2
SAL_JA3803AA	PNUSAS011118	Dublin	5	4	6	7	0	0	5	5	6	5	2
SAL_JA5282AA	9-25-20170290	Dublin	5	4	6	7	0	0	5	5	6	5	18
SAL_JA5295AA	9-24-20170289	Dublin	5	4	6	7	0	0	5	5	6	5	18
SAL_JA5291AA	9-26-20170291	Dublin	5	4	6	7	0	0	5	5	6	5	18
SAL_JA5294AA	9-9-20170210	Dublin	5	4	6	7	0	0	5	5	6	5	18
SAL_JA4649AA	CVM 43791	Dublin	5	4	6	7	0	0	5	5	6	5	2
SAL_JA4600AA	CVM 43840	Dublin	5	4	6	7	0	0	5	5	6	5	2
SAL_JA5022AA	FSIS1701153	Dublin	5	4	6	7	0	0	5	5	6	5	10
SAL_JA6550AA	361820	Dublin	5	4	6	7	0	0	5	5	6	5	18
SAL_JA6396AA	FSIS1701026	Dublin	5	4	6	7	0	0	5	5	6	5	0
SAL_JA6377AA	FSIS1701019	Dublin	5	4	6	7	0	0	5	5	6	5	12
SAL_JA6040AA	FSIS1701152	Dublin	5	4	6	7	0	0	5	5	6	5	2
SAL_JA5684AA	PNUSAS012471	Dublin	5	4	6	7	0	0	5	5	6	5	2
SAL_JA6759AA	FSIS1700870	Dublin	5	4	6	7	0	0	5	5	6	5	2
SAL_JA7110AA	PNUSAS012000	Dublin	5	4	6	7	0	0	5	5	6	5	2
SAL_JA7359AA	PNUSAS013762	Dublin	5	4	6	7	0	0	5	5	6	5	2
SAL_JA7546AA	bench26	Dublin	5	4	6	7	0	0	5	5	6	5	18
SAL_JA7463AA	CFSAN059898	Dublin	5	4	6	7	0	0	5	5	6	5	17
SAL_JA8250AA	345721	Dublin	5	4	6	7	0	0	5	5	6	5	10
SAL_JA8044AA	330451	Dublin	5	4	6	7	0	0	5	5	6	5	11
SAL_JA8461AA	11-13-20170295	Dublin	5	4	6	7	0	0	5	5	6	5	18
SAL_JA8803AA	SD1019	Dublin	5	4	6	7	0	0	5	5	6	5	10
SAL_JA8819AA	SD4891	Dublin	5	4	6	7	0	0	5	5	6	5	10
SAL_JA8815AA	SD3099	Dublin	5	4	6	7	0	0	5	5	6	5	10
SAL_JA8816AA	SD3100	Dublin	5	4	6	7	0	0	5	5	6	5	10
SAL_JA8822AA	SD5564	Dublin	5	4	6	7	0	0	0	0	0	0	0

SAL_JA8825AA	SD595_98	Dublin	5	4	6	7	0	0	5	5	6	5	10
SAL_JA4674AA	CVM 43766	Dublin	5	4	6	7	0	0	0	5	6	5	2
SAL_JA9553AA	CFSAN046164	Dublin	5	4	6	7	0	0	5	5	6	5	2
SAL_JA9368AA	PNUSAS015200	Dublin	5	4	6	7	0	0	5	5	6	5	0
SAL_JA9292AA	PNUSAS015017	Dublin	5	4	6	7	0	0	5	5	6	5	2
SAL_JA9204AA	FSIS1701563	Dublin	5	4	6	7	0	0	0	0	0	0	0
SAL_JA9079AA	SAL-17-VL-NY-FL-0009	Dublin	5	4	6	7	0	0	5	5	6	5	2
SAL_KA0045AA	PNUSAS015762	Dublin	5	4	6	7	0	0	5	5	6	5	0
SAL_KA0765AA	PNUSAS015920	Dublin	5	4	6	7	0	0	5	5	6	5	2
SAL_KA0526AA	PNUSAS016163	Dublin	5	4	6	7	0	0	5	5	6	5	2
SAL_KA0292AA	FSIS1702160	Dublin	5	4	6	7	0	0	5	5	6	5	2
SAL_KA1612AA	FSIS1702108	Dublin	5	4	6	7	0	0	5	5	6	5	10
SAL_MA2505AA	RJ1067_Salmonella_ssp	Dublin	5	4	6	7	0	0	5	5	6	5	10
SAL_KA1584AA	F15-1096-1-380	Dublin	5	4	6	7	0	0	5	5	6	5	10
SAL_KA1047AA	FSIS1702036	Dublin	5	4	6	7	0	0	5	5	6	5	1
SAL_KA2087AA	12-26-20170461	Dublin	5	4	6	7	0	0	5	5	6	5	18
SAL_KA1780AA	PNUSAS016772	Dublin	5	4	6	7	0	0	5	5	83	5	2
SAL_KA1747AA	PNUSAS016502	Dublin	5	4	6	7	0	0	5	5	6	5	2
SAL_KA2899AA	PNUSAS014992	Dublin	5	4	6	7	0	0	5	5	6	5	2
SAL_KA2640AA	PNUSAS015881	Dublin	5	4	6	7	0	0	5	5	6	5	2
SAL_KA3553AA	FSIS1702467	Dublin	5	4	6	7	0	0	5	5	6	5	2
SAL_KA3684AA	FSIS1701412	Dublin	5	4	6	7	0	0	5	5	6	5	2
SAL_KA3736AA	15-1-20170500	Dublin	5	4	6	7	0	0	5	5	6	5	18
SAL_KA4519AA	SGSC4916	Dublin	5	4	6	7	0	0	5	5	6	5	10
SAL_KA4298AA	GW_S00535-09	Dublin	5	4	6	7	0	0	5	5	6	5	18
SAL_KA4307AA	GW_S00402-09	Dublin	5	4	6	7	0	0	5	5	6	5	10
SAL_KA8730AA	SARB12 (Fidelma Boyd)	Dublin	5	4	6	7	0	0	5	5	6	5	2
SAL_KA4314AA	GW_S00637-09	Dublin	5	4	6	7	0	0	5	5	6	5	18
SAL_KA4275AA	GW_S00451-09	Dublin	5	4	6	7	0	0	5	5	6	5	10
SAL_KA4276AA	CE06.005	Dublin	5	4	29	7	0	0	5	5	6	5	2
SAL_KA4282AA	GW_S01238-09	Dublin	5	4	6	7	0	0	5	5	6	5	10
SAL_KA4283AA	SQ08.086	Dublin	5	4	6	7	0	0	5	5	6	5	2
SAL_KA4284AA	GW_S00169-09	Dublin	5	4	6	7	0	0	5	5	6	5	10
SAL_KA4245AA	1090600102	Dublin	5	4	6	7	0	0	5	5	6	5	17
SAL_KA4251AA	GW_S01066-09	Dublin	5	4	6	7	0	0	5	5	6	5	18
SAL_KA4253AA	GW_S00314-09	Dublin	5	4	6	7	0	0	5	5	6	5	18
SAL_KA4260AA	GW_S00510-09	Dublin	5	4	6	7	0	0	5	5	6	5	18
SAL_KA4267AA	GW_S02043-09	Dublin	5	4	6	7	0	0	5	5	6	5	18
SAL_KA4269AA	GW_S00166-09	Dublin	5	4	6	7	0	0	5	5	6	5	18
SAL_KA4228AA	GW_S00708-09	Dublin	5	4	6	7	0	0	5	5	6	5	18
SAL_KA4229AA	1090604773	Dublin	5	4	6	7	0	0	5	5	6	5	14
SAL_KA4235AA	GW_S01207-09	Dublin	5	4	6	7	0	0	5	5	6	5	10
SAL_KA4291AA	GW_S00365-09	Dublin	5	4	6	7	0	0	5	5	6	5	18
SAL_KA4237AA	GW_S00220-09	Dublin	5	4	6	7	0	0	5	5	6	5	10
SAL_KA4244AA	GW_S00460-09	Dublin	5	4	6	7	0	0	5	5	6	5	18
SAL_KA5001AA	2015AM-1434	Dublin	5	4	6	7	0	0	5	5	6	5	2
SAL_KA5105AA	16-22-20170389	Dublin	5	4	6	7	0	0	0	5	6	5	18
SAL_KA5108AA	16-25-20170409	Dublin	5	4	6	7	0	0	5	5	6	5	12
SAL_KA5110AA	16-24-20170408	Dublin	5	4	6	7	0	0	5	5	6	5	18
SAL_KA5111AA	16-27-20170468	Dublin	5	4	6	7	0	0	5	5	6	5	18
SAL_KA5113AA	16-28-20170469	Dublin	5	4	6	7	0	0	5	5	6	5	18
SAL_KA5459AA	PNUSAS017493	Dublin	5	4	6	7	0	0	5	5	6	5	2
SAL_KA5323AA	PNUSAS017959	Dublin	5	4	6	7	0	0	5	5	6	5	2
SAL_KA5937AA	PNUSAS018755	Dublin	0	0	0	0	0	0	0	0	0	0	0
SAL_KA5981AA	FSIS1702757	Dublin	5	4	6	7	0	0	0	0	0	0	0
SAL_KA5753AA	ISUVDL-15-09692	Dublin	5	4	6	7	0	0	5	5	6	5	2
SAL_KA5752AA	ISUVDL-15-09298	Dublin	5	4	6	7	0	0	5	5	6	5	2
SAL_KA6070AA	PNUSAS018851	Dublin	5	4	6	7	0	0	5	5	6	5	2
SAL_KA6242AA	ISUVDL-16-25417	Dublin	5	4	6	7	0	0	5	5	6	5	2
SAL_KA6238AA	ISUVDL-15-31644	Dublin	5	4	6	7	0	0	5	5	6	5	2
SAL_KA6235AA	ISUVDL-15-05897	Dublin	5	4	6	7	0	0	5	5	0	5	2
SAL_KA6241AA	ISUVDL-15-18758	Dublin	5	4	6	7	0	0	5	5	6	5	2
SAL_KA6237AA	ISUVDL-15-08069	Dublin	5	4	6	7	0	0	5	5	0	5	2
SAL_KA6234AA	ISUVDL-15-03678	Dublin	5	4	6	7	0	0	5	5	6	5	2
SAL_KA7175AA	17-9-20170612	Dublin	5	4	6	7	0	0	5	5	6	5	18
SAL_KA7176AA	17-12-20170615	Dublin	5	4	6	7	0	0	5	5	6	5	18
SAL_KA6240AA	ISUVDL-15-33038	Dublin	5	4	6	7	0	0	5	5	6	5	2
SAL_KA6236AA	ISUVDL-15-03572	Dublin	5	4	6	7	0	0	5	5	6	5	2
SAL_KA6239AA	ISUVDL-15-19130	Dublin	5	4	6	7	0	0	5	5	6	5	2
SAL_JA8806AA	SD12612	Dublin	5	4	6	7	0	0	5	5	83	5	10
SAL_JA8817AA	SD3639	Dublin	5	4	6	7	0	0	5	5	6	5	2
SAL_JA8831AA	SD99	Dublin	5	4	6	7	0	0	5	5	6	5	17
SAL_JA8824AA	SD559	Dublin	5	4	6	7	0	0	5	5	6	5	14

SAL_KA7665AA	SD12612-CFSAN060516	Dublin	5	4	6	7	0	0	5	5	83	5	10
SAL_KA7667AA	SD3639-CFSAN060512	Dublin	5	4	6	7	0	0	5	5	6	5	2
SAL_KA7668AA	SD559-CFSAN060481	Dublin	5	4	6	7	0	0	5	5	6	5	17
SAL_KA7670AA	SD99-CFSAN060445	Dublin	5	4	6	7	0	0	5	5	6	5	17
SAL_KA6774AA	PNUSAS019470	Dublin	5	4	6	7	0	0	5	5	6	5	2
SAL_KA6755AA	PNUSAS014425	Dublin	5	4	6	7	0	0	5	5	6	5	2
SAL_KA6753AA	PNUSAS017539	Dublin	5	4	6	7	0	0	5	5	6	5	2
SAL_KA7225AA	ISUVDL-15-37793	Dublin	5	4	6	7	0	0	5	5	6	5	2
SAL_KA7217AA	ISUVDL-15-65359	Dublin	5	4	6	7	0	0	0	5	6	5	2
SAL_KA7222AA	ISUVDL-15-79094	Dublin	5	4	6	7	0	0	5	5	6	5	2
SAL_KA7120AA	FSIS1703337	Dublin	5	4	6	7	0	0	5	5	6	5	2
SAL_KA7197AA	ISUVDL-15-03810	Dublin	5	4	6	7	0	0	5	5	6	5	2
SAL_KA7198AA	ISUVDL-16-23242	Dublin	5	4	6	7	0	0	5	5	6	5	2
SAL_KA7205AA	ISUVDL-15-49913	Dublin	5	4	6	7	0	0	5	5	6	5	2
SAL_KA7206AA	ISUVDL-15-47507	Dublin	5	4	6	7	0	0	5	5	6	5	2
SAL_KA7211AA	ISUVDL-15-70166	Dublin	5	4	6	7	0	0	5	5	6	5	2
SAL_KA7220AA	ISUVDL-15-68599	Dublin	5	4	6	7	0	0	5	5	6	5	2
SAL_KA7223AA	ISUVDL-15-36246	Dublin	5	4	6	7	0	0	5	5	6	5	2
SAL_KA7230AA	ISUVDL-15-79629	Dublin	5	4	6	7	0	0	5	5	6	5	2
SAL_KA7218AA	ISUVDL-15-75208	Dublin	5	4	6	7	0	0	5	5	6	5	2
SAL_KA7219AA	ISUVDL-15-75521	Dublin	5	4	6	7	0	0	5	5	83	5	2
SAL_KA7221AA	ISUVDL-15-36005	Dublin	5	4	6	7	0	0	5	5	6	5	2
SAL_KA7226AA	ISUVDL-15-65998	Dublin	5	4	6	7	0	0	5	5	6	5	2
SAL_KA7227AA	ISUVDL-16-32277	Dublin	5	4	6	7	0	0	5	5	6	5	2
SAL_KA7228AA	ISUVDL-15-83748	Dublin	5	4	6	7	0	0	5	5	6	5	2
SAL_KA7200AA	ISUVDL-15-67014	Dublin	5	4	6	7	0	0	5	5	6	5	2
SAL_KA7201AA	ISUVDL-15-49907	Dublin	5	4	6	7	0	0	5	5	6	5	2
SAL_KA7202AA	ISUVDL-15-67698	Dublin	5	4	6	7	0	0	5	5	6	5	2
SAL_KA7203AA	ISUVDL-15-48168	Dublin	5	4	6	7	0	0	5	5	6	5	2
SAL_KA7204AA	ISUVDL-15-40786	Dublin	5	4	6	7	0	0	5	5	6	5	2
SAL_KA7210AA	ISUVDL-16-11447	Dublin	5	4	6	7	0	0	5	5	6	5	2
SAL_KA7215AA	ISUVDL-15-06494	Dublin	5	4	6	7	0	0	5	5	6	5	2
SAL_KA7199AA	ISUVDL-15-41204	Dublin	5	4	6	7	0	0	5	5	6	5	2
SAL_KA7207AA	ISUVDL-16-32713	Dublin	5	4	6	7	0	0	5	5	6	5	2
SAL_KA7208AA	ISUVDL-16-23727	Dublin	5	4	6	7	0	0	5	5	6	5	2
SAL_KA7431AA	PNUSAS018472	Dublin	5	4	6	7	0	0	5	5	6	5	2
SAL_KA7675AA	FSIS1703461	Dublin	5	4	6	7	0	0	5	5	6	5	2
SAL_KA8039AA	PNUSAS019793	Dublin	0	0	0	0	0	0	0	0	0	0	0
SAL_KA8276AA	PNUSAS020161	Dublin	5	4	6	7	0	0	5	5	6	5	2
SAL_KA8263AA	PNUSAS020027	Dublin	5	4	6	7	0	0	5	5	6	5	2
SAL_KA9171AA	ISUVDL-15-75507	Dublin	5	4	6	7	0	0	5	5	6	5	2
SAL_KA9156AA	ISUVDL-15-43516	Dublin	5	4	6	7	0	0	5	5	6	5	2
SAL_KA9162AA	ISUVDL-17-23667	Dublin	5	4	6	7	0	0	5	5	6	5	0
SAL_KA9166AA	ISUVDL-16-03870	Dublin	5	4	6	7	0	0	5	5	6	5	2
SAL_KA9169AA	ADRDL-913	Dublin	5	4	6	7	0	0	5	5	6	5	2
SAL_KA9167AA	ISUVDL-16-11355	Dublin	5	4	6	7	0	0	5	5	6	5	2
SAL_KA9165AA	ISUVDL-16-36052	Dublin	5	4	6	7	0	0	5	5	6	5	0
SAL_KA9152AA	ISUVDL-15-43254	Dublin	5	4	6	7	0	0	5	5	6	5	2
SAL_KA9164AA	ISUVDL-16-08515	Dublin	5	4	6	7	0	0	5	5	6	5	2
SAL_KA9158AA	ISUVDL-15-81714	Dublin	5	4	6	7	0	0	5	5	6	5	2
SAL_KA9159AA	ISUVDL-15-70006	Dublin	5	4	6	7	0	0	5	5	6	5	2
SAL_KA9157AA	ISUVDL-16-02193	Dublin	5	4	6	7	0	0	5	5	6	5	2
SAL_KA9160AA	ADRDL-915	Dublin	5	4	6	7	0	0	5	5	6	5	2
SAL_KA9149AA	ISUVDL-16-00924	Dublin	5	4	6	7	0	0	5	5	6	5	2
SAL_KA9154AA	ISUVDL-16-04592	Dublin	5	4	6	7	0	0	5	5	6	5	2
SAL_KA9146AA	ISUVDL-15-67387	Dublin	5	4	6	7	0	0	5	5	6	5	2
SAL_KA9148AA	ISUVDL-16-02187	Dublin	5	4	6	7	0	0	5	5	6	5	2
SAL_KA9144AA	ISUVDL-16-33217	Dublin	5	4	6	7	0	0	5	5	6	5	2
SAL_KA9401AA	FDA00011754	Dublin	0	0	0	0	0	0	0	0	0	0	0
SAL_KA7224AA	ISUVDL-15-69454	Dublin	5	4	6	7	0	0	5	5	6	5	2
SAL_KA9332AA	PNUSAS020616	Dublin	5	4	6	7	0	0	5	5	6	5	2
SAL_LA0189AA	2015AM-1357	Dublin	5	4	6	7	0	0	5	5	6	5	2
SAL_LA0821AA	D15-051537	Dublin	5	4	6	7	0	0	5	5	6	5	2
SAL_LA1394AA	CFSAN067876	Dublin	5	4	6	7	0	0	0	0	0	0	0
SAL_LA1390AA	CFSAN067877	Dublin	5	4	6	7	0	0	0	0	0	0	0
SAL_LA1385AA	CFSAN067875	Dublin	5	4	6	7	0	0	0	0	0	0	0
SAL_LA1316AA	CFSAN067264	Dublin	5	4	6	7	0	0	5	5	6	0	2
SAL_LA1465AA	ISUVDL-15-47318	Dublin	5	4	6	7	0	0	5	5	0	5	2
SAL_LA2642AA	MC_08-0443	Dublin	5	4	6	7	0	0	5	5	0	5	11
SAL_LA2651AA	BD-OM-2008-09-002605	Dublin	5	4	6	27	0	0	27	5	43	5	1
SAL_LA2690AA	JE_S09-002316	Dublin	5	4	6	7	0	0	5	5	6	5	2
SAL_LA2699AA	BD-PM-2007-02-008652	Dublin	5	4	6	7	0	0	5	5	6	5	11
SAL_LA2705AA	BD-PM-2008-09-003273	Dublin	5	4	6	27	0	0	27	5	43	5	1

SAL_LA2599AA	RK_P171	Dublin	5	4	6	7	0	0	5	5	6	5	11
SAL_LA2554AA	MC_06-0262	Dublin	5	4	6	7	0	0	5	5	6	5	2
SAL_LA2715AA	BD-OM-2007-03-003314	Dublin	5	4	6	7	0	0	5	5	6	5	11
SAL_LA2716AA	JM_04/12	Dublin	5	4	6	7	0	0	5	5	6	5	11
SAL_LA2634AA	BD-DU-2007-11-004076	Dublin	5	4	6	7	0	0	5	5	6	5	11
SAL_LA2650AA	BD-PM-2007-11-004094	Dublin	5	4	6	7	0	0	5	5	6	5	11
SAL_LA2681AA	BD-PM-2007-10-001037	Dublin	5	4	6	7	0	0	5	5	6	5	18
SAL_LA2683AA	BD-DU-2007-01-006960	Dublin	5	4	6	7	0	0	5	5	6	5	18
SAL_LA2689AA	BD-DU-2008-12-002582	Dublin	5	4	6	7	0	0	5	5	6	179	11
SAL_LA2713AA	BD-DU-2007-09-002816	Dublin	5	4	6	7	0	0	5	5	6	5	18
SAL_LA2653AA	MC_05-0009	Dublin	5	4	6	7	0	0	5	5	6	5	11
SAL_LA2675AA	DB_2	Dublin	5	4	6	7	0	0	5	5	6	5	2
SAL_LA2707AA	RK_P72	Dublin	5	4	6	7	0	0	5	5	126	5	11
SAL_LA2709AA	OT04/222	Dublin	5	4	6	7	0	0	5	5	6	5	13
SAL_LA2659AA	MC_04-0021	Dublin	5	4	6	7	0	0	5	5	6	5	11
SAL_LA2668AA	BD-PM-2007-01-002684	Dublin	5	4	6	7	0	0	5	5	6	5	18
SAL_LA2568AA	SARB14	Dublin	0	0	0	0	0	0	0	0	0	0	0
SAL_LA2698AA	JE_S09-000757	Dublin	5	4	6	7	0	0	5	5	6	5	11
SAL_LA2628AA	JE_S09-000702	Dublin	5	4	6	7	0	0	5	5	6	5	11
SAL_LA2660AA	DB_7	Dublin	5	4	6	7	0	0	5	5	6	5	2
SAL_LA2645AA	BD-DU-2007-04-001249	Dublin	5	4	6	7	0	0	41	5	6	5	2
SAL_LA2673AA	RK_P167	Dublin	5	4	6	7	0	0	5	5	6	5	11
SAL_LA2697AA	BD-DU-2007-10-008218	Dublin	5	4	6	7	0	0	5	5	6	5	0
SAL_LA2691AA	RK_P24	Dublin	5	4	6	7	0	0	5	5	6	5	11
SAL_LA2669AA	M78	Dublin	5	4	6	7	0	0	5	5	6	180	17
SAL_LA2658AA	CK_03/00001160	Dublin	5	4	6	7	0	0	5	5	6	5	17
SAL_LA2674AA	BD-OM-2009-01-000189	Dublin	5	4	6	7	0	0	5	5	6	5	18
SAL_LA2627AA	BD-OM-2007-09-000001	Dublin	5	4	6	7	0	0	5	5	6	5	17
SAL_LA2643AA	BD-OM-2008-02-001666	Dublin	5	4	6	7	0	0	5	5	6	5	18
SAL_LA2661AA	BD-DU-2006-12-006830	Dublin	5	4	6	7	0	0	5	5	6	5	10
SAL_LA2682AA	RK_D62	Dublin	5	4	6	7	0	0	5	5	6	5	10
SAL_LA2714AA	RK_P117	Dublin	5	4	6	7	0	0	5	5	126	5	11
SAL_LA2666AA	BD-OM-2007-11-006155	Dublin	5	4	6	27	0	0	27	0	239	5	1
SAL_LA2635AA	BD-DU-2008-07-002474	Dublin	5	4	6	27	0	0	27	5	43	5	1
SAL_LA2667AA	CIT_VC31	Dublin	5	4	6	7	0	0	5	5	6	5	11
SAL_LA2637AA	MC_06-0025	Dublin	5	4	6	7	0	0	5	5	6	5	11
SAL_LA2629AA	BD-DU-2007-03-003760	Dublin	5	4	6	7	0	0	5	5	6	5	11
SAL_LA2652AA	BD-OM-2008-04-002177	Dublin	5	4	6	7	0	0	5	5	6	5	18
SAL_LA2636AA	BD-DU-2008-03-000912	Dublin	5	4	6	7	0	0	5	5	6	5	10
SAL_LA2644AA	RK_P92	Dublin	5	4	6	7	0	0	5	5	0	5	11
SAL_LA2684AA	JM_01/113	Dublin	5	4	6	7	0	0	5	5	83	5	10
SAL_LA3841AA	PNUSAS022306	Dublin	5	4	6	7	0	0	5	5	6	5	2
SAL_LA2139AA	BD-DU-2006-11-008285	Dublin	5	4	6	7	0	0	5	5	244	5	11
SAL_KA7666AA	SD155-CFSAN060432	Dublin	5	4	0	7	0	0	5	5	6	5	17
SAL_LA4747AA	PNUSAS023259	Dublin	5	4	6	7	0	0	5	5	6	5	2
SAL_LA5011AA	PNUSAS023200	Dublin	5	4	6	7	0	0	5	5	6	5	2
SAL_LA2706AA	JE_S09-002417	Dublin	5	4	6	7	0	0	5	5	6	5	10
SAL_LA2700AA	JM_01/124	Dublin	5	4	6	7	0	0	5	5	6	5	12
SAL_LA5628AA	20-15-20170791	Dublin	5	4	6	7	0	0	5	5	6	5	18
SAL_LA6920AA	21-15-20170810	Dublin	5	4	6	7	0	0	5	5	6	5	18
SAL_LA6959AA	22-24-20170897	Dublin	5	4	6	7	0	0	5	5	6	5	18
SAL_LA8121AA	23-27-20170947	Dublin	5	4	6	7	0	0	5	5	6	5	60
SAL_LA8125AA	23-1-20170901	Dublin	5	4	6	7	0	0	5	5	6	5	18
SAL_LA8135AA	23-9-20170929	Dublin	5	4	6	7	0	0	5	5	6	5	17
SAL_LA8145AA	23-16-20170936	Dublin	5	4	6	7	0	0	5	5	6	5	18
SAL_LA6603AA	PNUSAS022200	Dublin	5	4	6	7	0	0	5	5	6	5	2
SAL_LA6312AA	PNUSAS023297	Dublin	5	4	6	7	0	0	5	5	6	5	2
SAL_LA6202AA	CFSAN069197	Dublin	5	4	6	7	0	0	0	0	0	0	2
SAL_LA7451AA	PNUSAS022663	Dublin	5	4	6	7	0	0	5	5	6	5	2
SAL_LA9928AA	24-6-20170978	Dublin	5	4	6	7	0	0	5	5	83	5	18
SAL_LA9930AA	24-5-20170977	Dublin	5	4	6	7	0	0	134	5	6	5	18
SAL_LA8957AA	PNUSAS022817	Dublin	5	4	6	7	0	0	5	5	6	5	2
SAL_MA0222AA	PNUSAS024752	Dublin	5	4	6	7	0	0	5	5	6	5	2
SAL_MA0153AA	PNUSAS024217	Dublin	5	4	6	7	0	0	5	5	6	5	2
SAL_MA0409AA	PNUSAS024096	Dublin	5	4	6	7	0	0	5	5	6	5	2
SAL_MA0317AA	SAL-17-VL-OH-WA-0008	Dublin	5	4	6	7	0	0	5	5	6	5	2
SAL_MA1160AA	MS170214	Dublin	5	4	6	7	0	0	5	5	6	5	11
SAL_MA1476AA	CFSAN070575	Dublin	5	4	6	7	0	0	5	5	98	5	2
SAL_MA1284AA	CFSAN070290	Dublin	5	4	6	7	0	0	5	5	6	5	2
SAL_MA1256AA	CFSAN069284	Dublin	5	4	6	7	0	0	5	5	6	5	2
SAL_MA2216AA	411465	Dublin	5	4	6	7	0	0	5	5	6	5	18
SAL_MA2202AA	248619	Dublin	5	4	6	7	0	0	5	5	0	5	18
SAL_MA2093AA	404295	Dublin	5	4	6	7	0	0	5	5	6	5	10

SAL_MA2099AA	238452	Dublin	5	4	6	7	0	0	5	5	6	5	18
SAL_MA2139AA	394898	Dublin	5	4	6	7	0	0	5	5	6	5	18
SAL_MA2189AA	238450	Dublin	5	4	6	7	0	0	5	5	6	5	18
SAL_MA2059AA	170404	Dublin	5	4	6	7	0	0	5	5	6	5	11
SAL_MA2065AA	133975	Dublin	5	4	6	7	0	0	5	5	6	5	18
SAL_MA2066AA	229176	Dublin	5	4	6	7	0	0	5	5	6	5	18
SAL_MA2084AA	302613	Dublin	5	4	6	7	0	0	5	5	6	5	18
SAL_MA2179AA	412237	Dublin	5	4	6	7	0	0	5	5	6	5	10
SAL_MA2162AA	414625	Dublin	5	4	6	7	0	0	5	5	6	5	17
SAL_MA1869AA	318526	Dublin	5	4	6	7	0	0	5	5	98	5	18
SAL_MA1914AA	406631	Dublin	5	4	6	7	0	0	5	5	6	5	18
SAL_MA1930AA	423858	Dublin	5	4	6	7	0	0	5	5	6	5	18
SAL_MA2046AA	243834	Dublin	5	4	6	7	0	0	5	5	6	5	10
SAL_MA1736AA	197028	Dublin	5	4	6	7	0	0	5	5	6	5	18
SAL_MA1742AA	289675	Dublin	5	4	6	7	0	0	5	5	6	5	18
SAL_MA1757AA	181063	Dublin	5	4	6	7	0	0	5	5	6	5	10
SAL_MA1786AA	422460	Dublin	5	4	6	7	0	0	5	5	0	5	18
SAL_MA1794AA	394903	Dublin	5	4	6	7	0	0	5	5	6	5	18
SAL_MA1814AA	246168	Dublin	5	4	6	7	0	0	5	5	6	5	18
SAL_MA1851AA	288973	Dublin	5	4	6	7	0	0	5	5	6	5	18
SAL_MA1911AA	427566	Dublin	5	4	6	7	0	0	5	5	6	5	2
SAL_MA1756AA	420584	Dublin	0	0	0	7	0	0	41	5	6	5	2
SAL_MA1809AA	394904	Dublin	5	4	6	7	0	0	5	5	6	5	18
SAL_MA1763AA	420670	Dublin	5	4	6	7	0	0	5	5	6	5	18
SAL_MA1978AA	318534	Dublin	5	4	6	7	0	0	5	5	6	5	18
SAL_MA1755AA	197027	Dublin	5	4	6	7	0	0	5	5	6	5	18
SAL_MA1920AA	189236	Dublin	5	4	6	7	0	0	5	5	6	5	18
SAL_MA1925AA	238444	Dublin	5	4	6	7	0	0	5	5	83	5	18
SAL_MA1951AA	394897	Dublin	5	4	6	7	0	0	5	5	6	5	18
SAL_MA2437AA	364182	Dublin	5	4	6	7	0	0	5	5	6	5	18
SAL_MA1998AA	388697	Dublin	5	4	6	7	0	0	5	5	6	5	10
SAL_MA1995AA	243892	Dublin	5	4	6	7	0	0	0	5	6	5	17
SAL_MA2006AA	169611	Dublin	5	4	6	7	0	0	5	5	6	5	18
SAL_MA2007AA	339515	Dublin	5	4	6	7	0	0	5	5	6	5	12
SAL_MA2022AA	394905	Dublin	5	4	6	7	0	0	5	5	6	5	18
SAL_MA2012AA	197029	Dublin	5	4	6	7	0	0	5	5	6	5	18
SAL_MA2406AA	223588	Dublin	5	4	6	7	0	0	5	5	6	5	18
SAL_MA2086AA	123958	Dublin	5	4	6	7	0	0	5	5	6	5	18
SAL_MA2163AA	391741	Dublin	5	4	6	7	0	0	5	5	6	5	18
SAL_MA2443AA	382857	Dublin	5	4	6	7	0	0	5	5	6	5	18
SAL_MA2455AA	391742	Dublin	5	4	6	7	0	0	5	5	6	5	18
SAL_MA2445AA	394901	Dublin	5	4	6	7	0	0	5	5	6	5	18
SAL_MA2324AA	235039	Dublin	5	4	6	7	0	0	5	0	6	5	18
SAL_MA2325AA	208760	Dublin	5	4	6	7	0	0	5	5	6	5	10
SAL_MA2338AA	204440	Dublin	5	4	6	7	0	0	5	5	6	5	11
SAL_MA2358AA	195812	Dublin	5	4	6	7	0	0	5	5	6	5	11
SAL_MA2373AA	261936	Dublin	5	4	6	7	0	0	5	5	6	5	18
SAL_MA2376AA	394902	Dublin	5	4	6	7	0	0	5	5	6	5	18
SAL_MA2514AA	J1084_Salmonella_Dubli	Dublin	5	4	6	7	0	0	5	5	6	5	10
SAL_MA2509AA	J1084_Salmonella_Dubli	Dublin	5	4	6	7	0	0	5	5	0	5	13
SAL_MA2510AA	J1084_Salmonella_Dubli	Dublin	5	4	6	7	0	0	5	5	6	5	10
SAL_MA2511AA	J1084_Salmonella_Dubli	Dublin	5	4	6	7	0	0	5	5	6	5	10
SAL_MA2512AA	RJ1084_Salmonella_Dubl	Dublin	5	4	6	7	0	0	5	5	6	5	29
SAL_MA2515AA	_PRJ1084_Salmonella_Du	Dublin	5	4	6	7	0	0	5	5	6	5	14
SAL_MA2570AA	SSI-AC386	Dublin	5	4	6	7	0	0	5	5	6	5	17
SAL_MA2513AA	J1084_Salmonella_Dubli	Dublin	5	4	6	7	0	0	5	5	6	5	17
SAL_MA2516AA	_PRJ1084_Salmonella_Du	Dublin	5	4	6	7	0	0	5	5	6	5	10
SAL_MA2721AA	PNUSAS024970	Dublin	5	4	6	7	0	0	5	5	6	5	2
SAL_MA2744AA	PNUSAS019236	Dublin	5	4	6	7	0	0	5	5	6	5	2
SAL_MA2873AA	PNUSAS026324	Dublin	5	4	6	7	0	0	5	5	6	5	2
SAL_MA2821AA	PNUSAS026224	Dublin	5	4	6	7	0	0	0	5	6	5	2
SAL_MA3370AA	PNUSAS024553	Dublin	5	4	6	7	0	0	5	5	6	5	2
SAL_MA3556AA	26-28-20171101	Dublin	5	4	6	7	0	0	5	5	6	5	18
SAL_MA4028AA	373957	Dublin	5	4	6	7	0	0	0	5	6	5	18
SAL_MA4026AA	402735	Dublin	5	4	6	7	0	0	5	5	6	5	10
SAL_MA3993AA	420487	Dublin	5	4	6	7	0	0	5	5	6	5	12
SAL_MA3967AA	265842	Dublin	5	4	6	7	0	0	5	5	6	5	18
SAL_MA3971AA	185895	Dublin	5	4	6	7	0	0	5	5	6	5	17
SAL_MA3974AA	241779	Dublin	5	4	6	7	0	0	5	5	271	5	10
SAL_MA3976AA	373310	Dublin	5	4	6	7	0	0	5	5	6	5	18
SAL_MA3985AA	388693	Dublin	5	4	6	7	0	0	5	5	6	5	18
SAL_MA3990AA	197031	Dublin	5	4	6	7	0	0	5	5	6	5	18
SAL_MA4008AA	107085	Dublin	5	4	6	7	0	0	5	5	6	5	18

SAL_MA3953AA	423184	Dublin	5	4	6	7	0	0	5	5	6	5	1
SAL_MA3943AA	34948	Dublin	5	4	6	7	0	0	5	5	6	5	2
SAL_MA4424AA	197025	Dublin	5	4	6	7	0	0	5	5	6	5	18
SAL_MA5221AA	FSIS1702419	Dublin	5	4	6	7	0	0	0	0	0	0	0
SAL_MA5257AA	27-29-8101182	Dublin	5	4	6	27	0	0	27	5	43	5	1
SAL_MA5255AA	27-28-8101181	Dublin	5	4	6	7	0	0	5	5	6	5	11
SAL_MA5290AA	6665	Dublin	5	4	6	7	0	0	5	5	6	5	17
SAL_MA5357AA	S17BD06094	Dublin	5	4	6	7	0	0	5	5	6	5	17
SAL_DA3573AA	CFSAN023227	Dublin	5	4	6	7	0	0	5	5	6	5	2
SAL_CA0117AA	57161	Dublin	5	4	6	63	0	0	5	5	6	5	29
SAL_AA8458AA	15-sc-1925577	Dublin	5	4	6	7	0	0	5	5	6	5	14
SAL_CA5213AA	79108	Dublin	5	4	6	7	0	0	5	5	6	5	11
SAL_CA5939AA	53997	Dublin	5	4	6	7	0	0	5	5	6	5	10
SAL_AA8471AA	2-sc-1925564	Dublin	5	4	6	7	0	0	41	5	6	5	2
SAL_AA9657AA	88_500	Dublin	5	4	6	8	0	0	5	53	51	5	1
SAL_BA5884AA	M248 / NCTC20082	Dublin	5	4	6	7	0	0	5	5	6	5	10
SAL_DA6974AA	IEH-NGS-SAL00408	Dublin	5	4	6	7	0	0	5	5	6	5	2
SAL_DA4403AA	CFSAN022411	Dublin	5	4	6	7	0	0	5	5	6	5	10
SAL_BA7392AA	SA20093032	Dublin	5	4	6	7	0	0	5	5	6	5	2
SAL_DA6371AA	CFSAN013378	Dublin	5	4	6	7	0	0	5	5	6	5	2
SAL_EA3628AA	CVM N50443	Dublin	5	4	6	7	0	0	5	5	6	5	0
SAL_EA4745AA	IEH-NGS-SAL-03190	Dublin	5	4	6	7	0	0	5	5	6	5	2
SAL_GA9183AA	CFSAN031530	Dublin	5	4	29	7	0	0	5	5	6	5	17
SAL_GA9341AA	FDA00010641	Dublin	0	0	0	0	0	0	0	0	0	0	0
SAL_EA7892AA	18536	Dublin	5	4	6	7	0	0	5	5	6	5	2
SAL_HA5452AA	13-1494	Dublin	5	4	6	231	0	0	5	5	6	5	10
SAL_EA9773AA	CT_02021853	Dublin	5	4	6	7	0	0	5	5	6	5	10
SAL_HA5792AA	PNUSAS005577	Dublin	5	4	6	7	0	0	5	5	0	5	44
SAL_GA4002AA	SD3246	Dublin	5	4	6	7	0	0	5	5	6	5	10
SAL_HA9442AA	CFSAN057824	Dublin	5	4	6	7	0	0	5	5	83	5	52
SAL_GA0532AA	gha300101-sc-2235147	Dublin	5	4	6	7	0	0	5	5	6	5	10
SAL_GA0905AA	gha112574-sc-2235260	Dublin	5	4	6	7	0	0	5	5	6	5	2
SAL_GA0985AA	gha112535-sc-2235779	Dublin	5	4	6	7	0	0	5	5	83	5	10
SAL_GA6299AA	08BKT038205	Dublin	5	4	6	7	0	0	5	5	6	5	10
SAL_GA6292AA	09BKT051452	Dublin	5	4	6	7	0	0	5	5	6	5	17
SAL_GA2422AA	BCW_1993	Dublin	5	4	6	7	0	0	5	5	6	5	2
SAL_FA9069AA	CVM N40387	Dublin	5	4	6	7	0	0	5	5	6	5	1
SAL_AA9656AA	CN_346_64-sc-1769927	Dublin	5	4	6	27	0	0	27	5	43	5	1
SAL_JA1757AA	PNUSAS009161	Dublin	5	4	6	7	0	0	5	5	6	5	2
SAL_JA4689AA	CVM 43751	Dublin	5	4	6	7	0	0	5	5	6	5	2
SAL_JA7010AA	PNUSAS014178	Dublin	0	0	0	0	0	0	0	0	0	0	0
SAL_JA8940AA	FSIS1701805	Dublin	5	4	6	7	0	0	5	5	6	5	2
SAL_KA6233AA	ISUVDL-15-06924	Dublin	5	4	6	7	0	0	5	5	98	5	2
SAL_KA6855AA	PNUSAS017506	Dublin	5	4	6	7	0	0	5	5	6	5	2
SAL_KA7229AA	ISUVDL-16-01250	Dublin	5	4	6	7	0	0	5	5	6	5	2
SAL_KA7209AA	ISUVDL-15-03353	Dublin	5	4	6	7	0	0	5	5	0	5	2
SAL_LA2188AA	PNUSAS020947	Dublin	0	0	0	0	0	0	0	0	0	0	0
SAL_MA2517AA	PRJ1084_Salmonella_Du	Dublin	5	4	6	7	0	0	5	5	98	5	12
SAL_MA5592AA	PNUSAS026268	Dublin	5	4	6	7	0	0	5	5	6	5	2
SAL_MA6079AA	ATCC 39184	Dublin	5	4	6	7	0	0	41	5	6	5	2
SAL_MA5548AA	PNUSAS026856	Dublin	5	4	6	7	0	0	5	5	6	5	2
SAL_MA5561AA	PNUSAS025724	Dublin	5	4	6	7	0	0	5	5	6	5	2
SAL_MA6253AA	PNUSAS027199	Dublin	5	4	6	7	0	0	5	5	6	5	2
SAL_MA6138AA	WAPHLSALA01155	Dublin	5	4	6	7	0	0	5	5	6	5	2
SAL_MA6139AA	WAPHLSALA01156	Dublin	5	4	6	7	0	0	5	5	6	5	2
SAL_MA6156AA	WAPHLSALA01158	Dublin	5	4	6	7	0	0	5	5	6	5	2
SAL_MA6150AA	WAPHLSALA01157	Dublin	5	4	6	7	0	0	5	5	6	5	2
SAL_MA6144AA	WAPHLSALA01154	Dublin	5	4	6	7	0	0	5	5	6	5	2
SAL_MA6090AA	FSIS11705460	Dublin	5	4	6	7	0	0	0	0	0	0	0
SAL_MA6430AA	CFSAN071242	Dublin	5	4	6	7	0	0	5	0	6	5	2
SAL_MA6485AA	FSIS11705552	Dublin	5	4	6	7	0	0	5	5	6	5	2
SAL_MA6479AA	FSIS11705541	Dublin	5	4	6	7	0	0	5	5	6	5	2
SAL_MA6480AA	FSIS11705540	Dublin	5	4	6	7	0	0	5	5	6	5	2
SAL_MA6694AA	PNUSAS027846	Dublin	5	4	6	7	0	0	5	5	6	5	2
SAL_MA6790AA	CQ201707768	Dublin	5	4	6	7	0	0	5	5	6	5	17
SAL_MA6769AA	ADRDL-1573	Dublin	5	4	6	7	0	0	5	5	6	5	2
SAL_MA7407AA	ADRDL-1615	Dublin	5	4	6	7	0	0	5	5	6	5	2
SAL_MA7365AA	OH-17-6484	Dublin	5	4	6	7	0	0	5	5	6	5	2
SAL_MA7254AA	PNUSAS024372	Dublin	5	4	6	7	0	0	5	5	6	5	2
SAL_MA7228AA	PNUSAS026594	Dublin	5	4	6	7	0	0	5	5	6	5	2
SAL_MA6930AA	SAL-17-VL-SD-MN-0012	Dublin	5	4	6	7	0	0	5	5	6	5	2
SAL_MA6867AA	SAL-17-VL-SD-MI-0003	Dublin	5	4	6	7	0	0	5	5	6	5	2
SAL_MA6839AA	ADRDL-1585	Dublin	5	4	6	7	0	0	5	5	6	5	2

SAL_MA7529AA	PNUSAS028236	Dublin	5	4	6	7	0	0	5	5	6	5	2
SAL_MA7515AA	PNUSAS028234	Dublin	5	4	6	7	0	0	5	5	6	5	2
SAL_MA7430AA	FSIS11705753	Dublin	5	4	6	7	0	0	0	0	0	0	0
SAL_MA7435AA	FSIS11705757	Dublin	5	4	6	7	0	0	0	0	0	0	0
SAL_MA7452AA	FSIS11705755	Dublin	5	4	6	7	0	0	5	5	6	5	2
SAL_MA7451AA	FSIS11705756	Dublin	5	4	6	7	0	0	0	0	0	0	0
SAL_MA7839AA	OH-17-4693	Dublin	5	4	6	7	0	0	5	5	6	5	2
SAL_MA7956AA	30-6-8101237	Dublin	5	4	6	7	0	0	5	5	6	5	18
SAL_MA8048AA	PNUSAS028802	Dublin	5	4	6	7	0	0	5	5	6	5	2
SAL_MA8277AA	FSIS21720666	Dublin	5	4	6	7	0	0	5	5	6	5	2
SAL_MA8968AA	PNUSAS029107	Dublin	5	4	6	7	0	0	5	5	6	5	2
SAL_MA8801AA	FSIS11705982	Dublin	5	4	6	7	0	0	0	0	0	0	0
SAL_MA8807AA	FSIS11705899	Dublin	5	4	6	7	0	0	0	0	6	5	0
SAL_MA8790AA	FSIS11705983	Dublin	5	4	6	7	0	0	5	5	6	5	2
SAL_MA8791AA	FSIS11705981	Dublin	5	4	6	7	0	0	0	0	0	0	0
SAL_MA9040AA	FSIS11705856	Dublin	5	4	6	7	0	0	5	5	6	0	2
SAL_MA9011AA	CFSAN071487	Dublin	5	4	6	7	0	0	5	5	6	5	2
SAL_MA9025AA	CFSAN071485	Dublin	5	4	6	7	0	0	5	5	6	5	2
SAL_MA9105AA	CFSAN071486	Dublin	5	4	6	7	0	0	5	5	6	5	2
SAL_MA9117AA	31-17-17.8101271	Dublin	5	4	6	7	0	0	5	5	6	5	11
SAL_MA9125AA	31-8-17.8101260	Dublin	5	4	6	7	0	0	5	5	6	5	18
SAL_MA9132AA	31-23-17.8101140	Dublin	5	4	6	7	0	0	5	5	6	5	18
SAL_MA9135AA	31-9-17.8101261	Dublin	5	4	6	7	0	0	5	5	6	5	18
SAL_NA0762AA	32-19-8101290	Dublin	5	4	6	7	0	0	5	5	6	5	18
SAL_NA0777AA	32-13-8101284	Dublin	5	4	6	7	0	0	5	5	98	5	18
SAL_NA0173AA	CFSAN072184	Dublin	5	4	6	7	0	0	5	5	6	5	2
SAL_MA9705AA	CVM N16S275	Dublin	5	4	6	7	0	0	5	5	6	5	2
SAL_MA9587AA	CVM N16S402	Dublin	5	4	6	7	0	0	5	5	6	5	2
SAL_NA0679AA	PNUSAS021924	Dublin	5	4	6	7	0	0	5	5	6	5	2
SAL_NA0432AA	FSIS11706189	Dublin	5	4	6	7	0	0	5	5	6	5	2
SAL_NA0437AA	FSIS11706188	Dublin	5	4	6	7	0	0	5	5	6	5	2
SAL_NA0547AA	FSIS11706054	Dublin	5	4	6	7	0	0	0	0	0	0	0
SAL_NA0553AA	FSIS11706055	Dublin	5	4	6	7	0	0	0	0	0	0	0
SAL_NA0563AA	FSIS11706052	Dublin	5	4	6	7	0	0	5	5	6	5	2
SAL_NA0569AA	FSIS11706056	Dublin	5	4	6	7	0	0	5	5	98	5	2
SAL_NA0635AA	PNUSAS029172	Dublin	5	4	6	7	0	0	5	5	6	5	11
SAL_NA0971AA	CFSAN072171	Dublin	5	4	6	7	0	0	5	5	6	5	2
SAL_NA1080AA	PNUSAS029228	Dublin	5	4	6	7	0	0	5	5	6	5	2
SAL_NA1115AA	PNUSAS027410	Dublin	5	4	6	7	0	0	5	5	6	5	1
SAL_NA1178AA	PNUSAS026245	Dublin	5	4	6	7	0	0	5	5	6	5	10
SAL_NA1013AA	PNUSAS029862	Dublin	5	4	6	7	0	0	5	5	6	5	2
SAL_NA1241AA	PNUSAS028934	Dublin	5	4	6	7	0	0	0	0	0	5	2
SAL_NA1859AA	FSIS11705754	Dublin	5	4	6	7	0	0	5	5	6	5	2
SAL_NA1854AA	FSIS11706193	Dublin	5	4	6	7	0	0	5	5	6	5	2
SAL_NA1855AA	FSIS11706192	Dublin	5	4	6	7	0	0	5	5	6	5	2
SAL_NA1863AA	FSIS11706191	Dublin	5	4	6	7	0	0	0	0	0	0	0
SAL_NA1754AA	PNUSAS029041	Dublin	5	4	6	7	0	0	5	5	6	5	2
SAL_NA1382AA	FSIS11706377	Dublin	5	4	6	7	0	0	5	5	6	5	0
SAL_NA1386AA	FSIS11706376	Dublin	5	4	6	7	0	0	0	0	0	0	0
SAL_NA1387AA	FSIS11706375	Dublin	5	4	6	7	0	0	5	5	6	5	2
SAL_NA2034AA	33-31-8101333	Dublin	5	4	6	7	0	0	5	5	83	5	18
SAL_NA2098AA	CFSAN073514	Dublin	5	4	6	7	0	0	5	5	6	5	2
SAL_NA3147AA	34-27-8101351	Dublin	5	4	6	7	0	0	5	5	6	5	18
SAL_NA3153AA	34-32-8101356	Dublin	5	4	6	7	0	0	5	5	6	5	11
SAL_NA3155AA	34-28-8101352	Dublin	5	4	6	7	0	0	5	5	6	5	18
SAL_NA3160AA	34-14-8101339	Dublin	5	4	6	7	0	0	5	5	6	5	18
SAL_NA3161AA	34-20-8101264	Dublin	5	4	6	7	0	0	5	5	6	5	18
SAL_NA3302AA	FSIS11706648	Dublin	5	4	6	7	0	0	5	5	6	5	2
SAL_NA3212AA	PNUSAS029898	Dublin	5	4	6	7	0	0	5	5	6	5	2
SAL_NA3488AA	FSIS31700006	Dublin	5	4	6	7	0	0	0	0	0	0	0
SAL_NA3740AA	PNUSAS029852	Dublin	5	4	6	7	0	0	5	5	6	5	2
SAL_NA3733AA	PNUSAS029854	Dublin	5	4	6	7	0	0	5	5	6	5	2
SAL_NA3821AA	2015AM-2708	Dublin	5	4	6	7	0	0	5	5	6	5	2
SAL_NA3892AA	2015AM-0799	Dublin	5	4	6	7	0	0	5	5	6	5	2
SAL_NA4159AA	PNUSAS031147	Dublin	5	4	6	126	0	0	5	5	6	5	2
SAL_NA4142AA	FSIS11706673	Dublin	5	4	6	7	0	0	5	5	6	5	2
SAL_NA4124AA	PNUSAS031191	Dublin	5	4	6	7	0	0	5	5	6	5	2
SAL_NA4368AA	OH-17-26044	Dublin	5	4	6	7	0	0	5	5	6	5	2
SAL_NA4412AA	35-21-8101360	Dublin	5	4	6	7	0	0	5	5	6	5	11
SAL_NA4410AA	35-18-8101357	Dublin	5	4	6	7	0	0	5	5	6	5	2
SAL_NA4429AA	36-8-8101377	Dublin	5	4	6	7	0	0	5	5	6	5	18
SAL_NA4435AA	36-7-8101376	Dublin	5	4	6	7	0	0	5	5	6	5	18
SAL_NA4447AA	36-27-8101398	Dublin	5	4	6	7	0	0	5	5	6	5	18

SAL_NA4448AA	36-25-8101396	Dublin	5	4	6	7	0	0	5	5	6	5	18
SAL_NA4446AA	36-24-8101395	Dublin	5	4	6	7	0	0	5	5	6	5	18
SAL_NA4450AA	36-26-8101397	Dublin	5	4	6	7	0	0	5	5	6	5	18
SAL_NA4572AA	2014AM-0088	Dublin	5	4	6	7	0	0	5	5	6	5	2
SAL_NA4645AA	FSIS11706702	Dublin	5	4	6	7	0	0	0	0	0	0	0
SAL_NA4862AA	PNUSAS031243	Dublin	5	4	6	7	0	0	5	5	6	5	2
SAL_NA4863AA	PNUSAS031549	Dublin	5	4	6	7	0	0	5	5	6	5	2
SAL_NA4865AA	PNUSAS031251	Dublin	5	4	6	7	0	0	5	5	6	5	2
SAL_NA4870AA	PNUSAS030716	Dublin	5	4	6	7	0	0	5	5	0	5	2
SAL_NA4813AA	PNUSAS031513	Dublin	5	4	6	7	0	0	5	5	6	5	2
SAL_NA4828AA	PNUSAS031552	Dublin	0	0	0	0	0	0	0	0	0	0	0
SAL_NA4946AA	PNUSAS031488	Dublin	5	4	6	7	0	0	5	5	6	5	2
SAL_NA4986AA	01-5-18.8100002	Dublin	5	4	6	7	0	0	5	5	6	5	18
SAL_NA5118AA	PNUSAS031808	Dublin	5	4	6	7	0	0	5	5	83	5	2
SAL_NA5093AA	ADRDL-1840	Dublin	5	4	6	7	0	0	5	5	6	5	2
SAL_NA5233AA	ADRDL-1650	Dublin	5	4	6	7	0	0	5	5	6	5	2
SAL_NA5236AA	1608T18957_10	Dublin	5	4	6	7	0	0	5	5	6	5	17
SAL_NA5326AA	2015AM-0200	Dublin	5	4	6	7	0	0	0	0	0	0	0
SAL_NA6191AA	CFSAN074230	Dublin	5	4	6	7	0	0	5	5	6	5	2
SAL_NA6048AA	2015AM-2688	Dublin	5	4	6	7	0	0	5	5	6	5	2
SAL_NA6018AA	2015AM-2608	Dublin	5	4	6	7	0	0	5	5	6	5	2
SAL_NA6635AA	02-1-18.8100020	Dublin	5	4	6	7	0	0	5	5	6	156	18
SAL_NA5911AA	PNUSAS032048	Dublin	5	4	6	7	0	0	5	5	83	5	2
SAL_NA5803AA	2015AM-2790	Dublin	5	4	6	7	0	0	5	5	6	5	2
SAL_NA6275AA	OH-17-23364	Dublin	5	4	6	7	0	0	5	5	6	5	2
SAL_NA6849AA	PNUSAS031993	Dublin	5	4	6	7	0	0	5	5	6	5	2
SAL_NA7245AA	3-8-8100050	Dublin	5	4	6	7	0	0	5	5	6	5	18
SAL_NA7499AA	FSIS31800064	Dublin	5	4	6	7	0	0	5	5	6	5	2
SAL_NA7316AA	PNUSAS029406	Dublin	5	4	6	7	0	0	5	5	6	5	2
SAL_NA7286AA	PNUSAS032767	Dublin	5	4	6	7	0	0	5	5	6	5	10
SAL_NA8310AA	4-18-8100090	Dublin	5	4	6	7	0	0	5	5	6	5	18
SAL_NA8443AA	PNUSAS024676	Dublin	5	4	6	7	0	0	5	5	6	5	2
SAL_NA8446AA	PNUSAS027994	Dublin	5	4	6	7	0	0	5	5	6	5	2
SAL_NA8339AA	PNUSAS023929	Dublin	5	4	6	7	0	0	5	5	6	5	2
SAL_NA8966AA	PNUSAS032973	Dublin	5	4	6	7	0	0	5	5	6	5	2
SAL_NA8938AA	FSIS11807303	Dublin	5	4	6	7	0	0	5	5	6	5	2
SAL_NA8827AA	PNUSAS033152	Dublin	5	4	6	7	0	0	5	5	6	5	2
SAL_NA8720AA	FSIS31800086	Dublin	5	4	6	7	0	0	5	5	6	5	2
SAL_NA8640AA	PNUSAS033396	Dublin	5	4	6	7	0	0	5	5	6	5	2
SAL_NA8631AA	PNUSAS033397	Dublin	5	4	6	7	0	0	5	5	6	5	2
SAL_NA8636AA	PNUSAS033401	Dublin	5	4	6	7	0	0	5	5	6	5	2
SAL_NA9332AA	ADRDL-1807	Dublin	5	4	6	7	0	0	5	5	6	5	2
SAL_NA9333AA	ADRDL-1808	Dublin	5	4	6	7	0	0	5	5	6	5	2
SAL_NA9633AA	CFSAN060419	Dublin	5	4	6	7	0	0	5	5	6	5	10
SAL_NA9638AA	FSIS11807402	Dublin	5	4	6	7	0	0	5	5	6	5	2
SAL_NA9635AA	CFSAN060422	Dublin	5	4	6	7	0	0	0	0	0	0	0
SAL_NA9637AA	FSIS11807403	Dublin	5	4	6	7	0	0	5	5	6	5	2
SAL_NA9645AA	FSIS11807404	Dublin	5	4	6	7	0	0	5	5	6	5	2
SAL_NA9624AA	CFSAN060420	Dublin	5	4	6	7	0	0	5	5	6	5	11
SAL_NA9626AA	CFSAN060437	Dublin	5	4	6	7	0	0	5	5	6	5	10
SAL_NA9627AA	CFSAN060500	Dublin	5	4	6	7	0	0	5	5	6	5	11
SAL_NA9629AA	CFSAN060454	Dublin	5	4	6	7	0	0	5	5	6	5	14
SAL_NA9630AA	CFSAN060455	Dublin	5	4	6	7	0	0	5	5	6	5	14
SAL_NA9631AA	CFSAN060472	Dublin	5	4	6	7	0	0	5	5	6	5	40
SAL_NA9634AA	CFSAN060474	Dublin	5	4	6	7	0	0	5	5	6	5	13
SAL_NA9632AA	CFSAN060473	Dublin	5	4	6	7	0	0	5	5	6	5	14
SAL_NA9623AA	CFSAN060503	Dublin	5	4	6	7	0	0	0	0	0	0	0
SAL_NA9621AA	CFSAN060519	Dublin	5	4	6	7	0	0	5	5	6	5	11
SAL_NA9602AA	CFSAN060449	Dublin	5	4	6	7	0	0	5	5	6	5	12
SAL_NA9605AA	CFSAN060421	Dublin	5	4	6	7	0	0	5	5	6	5	11
SAL_NA9609AA	CFSAN060469	Dublin	5	4	6	7	0	0	5	5	6	5	10
SAL_NA9610AA	CFSAN060425	Dublin	5	4	6	7	0	0	5	5	6	5	11
SAL_NA9611AA	CFSAN060480	Dublin	5	4	6	7	0	0	5	5	6	5	17
SAL_NA9612AA	CFSAN060520	Dublin	5	4	6	7	0	0	5	5	6	5	11
SAL_NA9613AA	CFSAN060521	Dublin	5	4	6	7	0	0	0	0	0	0	0
SAL_NA9614AA	CFSAN060470	Dublin	5	4	6	7	0	0	5	5	6	5	10
SAL_NA9615AA	CFSAN060471	Dublin	5	4	6	7	0	0	5	5	6	5	0
SAL_NA9616AA	CFSAN060504	Dublin	5	4	6	7	0	0	5	5	6	5	12
SAL_NA9620AA	CFSAN060518	Dublin	5	4	6	7	0	0	5	5	6	5	11
SAL_NA9622AA	CFSAN060505	Dublin	5	4	6	7	0	0	5	5	6	5	11
SAL_NA9619AA	CFSAN060502	Dublin	5	4	6	7	0	0	5	5	6	5	10
SAL_NA9618AA	CFSAN060517	Dublin	5	4	6	7	0	0	5	5	6	5	12
SAL_NA9594AA	CFSAN060431	Dublin	5	4	6	7	0	0	5	5	6	5	2

SAL_NA9595AA	CFSAN060468	Dublin	5	4	6	7	0	0	5	5	6	5	10
SAL_NA9596AA	CFSAN060456	Dublin	5	4	6	7	0	0	5	5	83	5	12
SAL_NA9597AA	CFSAN060423	Dublin	5	4	6	334	0	0	5	5	6	5	11
SAL_NA9599AA	CFSAN060424	Dublin	5	4	6	7	0	0	5	5	6	5	14
SAL_NA9600AA	CFSAN060475	Dublin	5	4	6	335	0	0	5	5	6	5	10
SAL_NA9601AA	CFSAN060459	Dublin	5	4	6	7	0	0	5	5	6	5	11
SAL_NA9603AA	CFSAN060452	Dublin	5	4	6	7	0	0	5	5	6	5	10
SAL_NA9604AA	CFSAN060453	Dublin	5	4	6	7	0	0	5	5	6	5	10
SAL_NA9606AA	CFSAN060465	Dublin	5	4	6	7	0	0	5	5	6	5	10
SAL_NA9608AA	CFSAN060460	Dublin	5	4	6	334	0	0	5	5	6	5	10
SAL_NA9580AA	CFSAN060432	Dublin	5	4	6	7	0	0	5	5	6	5	0
SAL_NA9584AA	CFSAN060493	Dublin	5	4	6	7	0	0	5	5	6	5	10
SAL_NA9588AA	CFSAN060445	Dublin	5	4	6	7	0	0	5	5	6	5	0
SAL_NA9589AA	CFSAN060491	Dublin	5	4	6	7	0	0	0	0	0	0	0
SAL_NA9586AA	CFSAN060494	Dublin	5	4	6	7	0	0	5	5	6	5	10
SAL_NA9585AA	CFSAN060426	Dublin	5	4	6	7	0	0	5	5	83	5	14
SAL_NA9590AA	CFSAN060487	Dublin	5	4	6	7	0	0	5	5	6	5	11
SAL_NA9593AA	CFSAN060485	Dublin	5	4	6	7	0	0	5	5	6	5	11
SAL_NA9598AA	CFSAN060484	Dublin	5	4	6	7	0	0	5	5	0	5	12
SAL_NA9592AA	CFSAN060488	Dublin	5	4	6	7	0	0	5	5	6	5	11
SAL_NA9563AA	CFSAN060434	Dublin	5	4	6	7	0	0	5	5	6	5	11
SAL_NA9564AA	CFSAN060450	Dublin	5	4	6	7	0	0	5	5	6	5	10
SAL_NA9566AA	CFSAN060451	Dublin	5	4	6	7	0	0	5	5	6	5	17
SAL_NA9565AA	CFSAN060513	Dublin	5	4	6	7	0	0	5	5	6	5	2
SAL_NA9569AA	CFSAN060447	Dublin	5	4	6	7	0	0	5	5	6	5	17
SAL_NA9567AA	CFSAN060429	Dublin	5	4	6	7	0	0	5	5	6	5	11
SAL_NA9568AA	CFSAN060428	Dublin	5	4	6	335	0	0	5	5	6	5	11
SAL_NA9573AA	CFSAN060443	Dublin	5	4	6	7	0	0	5	5	6	5	11
SAL_NA9571AA	CFSAN060430	Dublin	5	4	6	7	0	0	5	5	6	5	11
SAL_NA9574AA	CFSAN060441	Dublin	5	4	6	7	0	0	5	5	6	5	12
SAL_NA9576AA	CFSAN060439	Dublin	5	4	6	7	0	0	5	5	6	5	10
SAL_NA9577AA	CFSAN060501	Dublin	5	4	6	7	0	0	5	5	6	156	11
SAL_NA9579AA	CFSAN060433	Dublin	5	4	6	7	0	0	5	5	6	5	11
SAL_NA9581AA	CFSAN060486	Dublin	5	4	6	7	0	0	5	5	6	5	11
SAL_NA9583AA	CFSAN060492	Dublin	5	4	6	7	0	0	5	5	6	5	10
SAL_NA9582AA	CFSAN060435	Dublin	5	4	6	7	0	0	5	5	83	5	11
SAL_NA9551AA	CFSAN060530	Dublin	5	4	6	7	0	0	5	5	6	5	11
SAL_NA9557AA	CFSAN060514	Dublin	5	4	6	7	0	0	0	0	0	0	0
SAL_NA9559AA	CFSAN060497	Dublin	5	4	6	7	0	0	5	5	6	5	0
SAL_NA9560AA	CFSAN060529	Dublin	5	4	6	7	0	0	5	5	6	5	2
SAL_NA9558AA	CFSAN060515	Dublin	5	4	6	7	0	0	5	5	0	5	17
SAL_NA9561AA	CFSAN060496	Dublin	5	4	6	7	0	0	5	5	6	5	10
SAL_NA9572AA	CFSAN060489	Dublin	5	4	6	7	0	0	5	5	6	5	11
SAL_NA9562AA	CFSAN060495	Dublin	5	4	6	7	0	0	5	5	6	5	2
SAL_NA9548AA	CFSAN060522	Dublin	5	4	6	7	0	0	5	5	6	5	11
SAL_NA9549AA	CFSAN060482	Dublin	5	4	6	7	0	0	5	5	6	5	17
SAL_NA9550AA	CFSAN060526	Dublin	5	4	6	7	0	0	5	5	6	5	12
SAL_NA9553AA	CFSAN060466	Dublin	5	4	6	7	0	0	5	5	6	5	10
SAL_NA9552AA	CFSAN060532	Dublin	5	4	6	7	0	0	5	5	6	5	17
SAL_NA9556AA	CFSAN060478	Dublin	5	4	6	7	0	0	5	5	6	5	10
SAL_NA9554AA	CFSAN060479	Dublin	5	4	6	7	0	0	5	5	6	5	17
[truncated: 696,685 more chars]
